# Quantitative proteomic comparison of myofibroblasts derived from bone marrow and cornea

**DOI:** 10.1038/s41598-020-73686-w

**Published:** 2020-10-07

**Authors:** Paramananda Saikia, Jack S. Crabb, Luciana L. Dibbin, Madison J. Juszczak, Belinda Willard, Geeng-Fu Jang, Thomas Michael Shiju, John W. Crabb, Steven E. Wilson

**Affiliations:** 1 Cole Eye Institute, I-32, Cleveland Clinic, 9500 Euclid Ave, Cleveland, OH 44195 USA; 2 Lerner Research Institute, Cleveland, OH 44195 USA; 3 Cleveland Clinic, Cleveland Clinic Lerner College of Medicine of Case Western Reserve University, Cleveland, OH 44195 USA

**Keywords:** Proteomics, Corneal diseases

## Abstract

Myofibroblasts are fibroblastic cells that function in wound healing, tissue repair and fibrosis, and arise from bone marrow (BM)-derived fibrocytes and a variety of local progenitor cells. In the cornea, myofibroblasts are derived primarily from stromal keratocytes and from BM-derived fibrocytes after epithelial-stromal and endothelial-stromal injuries. Quantitative proteomic comparison of mature alpha-smooth muscle actin (α-SMA)+ myofibroblasts (verified by immunocytochemistry for vimentin, α-SMA, desmin, and vinculin) generated from rabbit corneal fibroblasts treated with transforming growth factor (TGF) beta-1 or generated directly from cultured BM treated with TGF beta-1 was pursued for insights into possible functional differences. Paired cornea-derived and BM-derived α-SMA+ myofibroblast primary cultures were generated from four New Zealand white rabbits and confirmed to be myofibroblasts by immunocytochemistry. Paired cornea- and BM-derived myofibroblast specimens from each rabbit were analyzed by LC MS/MS iTRAQ technology using an Orbitrap Fusion Lumos Tribrid mass spectrometer, the Mascot search engine, the weighted average quantification method and the UniProt rabbit and human databases. From 2329 proteins quantified with ≥ 2 unique peptides from ≥ 3 rabbits, a total of 673 differentially expressed (DE) proteins were identified. Bioinformatic analysis of DE proteins with Ingenuity Pathway Analysis implicate progenitor-dependent functional differences in myofibroblasts that could impact tissue development. Our results suggest BM-derived myofibroblasts may be more prone to the formation of excessive cellular and extracellular material that are characteristic of fibrosis.

## Introduction

Myofibroblasts are cells that have indispensable roles in normal wound healing and tissue repair in all organs^1^ and the development and persistence of these cells is central to the pathophysiology of fibrosis^2,3^. Myofibroblasts may develop from fibroblasts^4^, fibrocytes^5,6^, epithelial cells or endothelial cells through mesenchymal transition^7,8^ or Schwann cells^9^, and possibly from other cells, depending on the location and type of injury, the genetic makeup of the individual animal and other unknown factors. In the cornea, for example, depending on the injury, the species of animal, and even the individual animal, myofibroblasts develop from keratocytes (via corneal fibroblasts) or fibrocytes in response to epithelial-stromal or endothelial-stromal injury, and resulting ongoing penetration of TGFβ into the corneal stroma at sufficient levels to drive myofibroblast development^5,10^. Keratocytes transition to corneal fibroblasts—which then differentiate into α-smooth muscle actin (α-SMA)-expressing myofibroblasts^11,12^. However, it has also been shown that infiltrating fibrocytes differentiate into myofibroblasts in mice after scar-producing epithelial-stromal injuries to the cornea using chimeric mice expressing green fluorescent protein and immunohistochemistry for fibrocyte and myofibroblast markers^6,13^. Mature myofibroblasts secrete different collagens and other extracellular matrix materials that make up the scars that are characteristic of fibrosis^3^.

Many studies have been performed to identify the progenitor cells for myofibroblasts in lung, liver and other organs^9,14,15^, but little is known about differences in function, if any, between myofibroblasts that arise from local progenitors compared to BM-derived progenitors. This study pursued quantitative proteomic comparison of rabbit myofibroblasts using LC MS/MS Isobaric Tag for Relative and Absolute Quantification (iTRAQ) technology for further insights into whether myofibroblasts that differentiate from corneal keratocytes are functionally different from myofibroblasts that differentiate from BM-derived cells.

## Materials and methods

### Animals

All animals were treated in accordance with the tenets of the ARVO Statement for the Use of Animals in Ophthalmic and Vision Research and the Institutional Animal Control and Use Committee at the Cleveland Clinic approved these studies. Four 12 to 15 week old female New Zealand white rabbits that weighed 2.5 to 3.0 kg were obtained from Charles River Laboratories, MA, USA.

### Myofibroblast primary cell cultures

Four separate primary keratocyte-derived corneal fibroblast cultures were generated from both eyes of the four New Zealand white rabbit corneas, as previously described^16^ with Dulbecco’s Modified Eagle Medium (DMEM, Gibco, Grand Island, NY). Briefly^16^,primary keratocytes were isolated from each cornea by first removing the epithelial and endothelial layers using 0.12 mm forceps and a #64 scalpel blade (BD Beaver, Franklin Lakes, NJ) under a dissecting microscope using the sterile technique. Keratocytes were isolated from the corneal stroma by digestion in sterile Dulbecco’s Modified Eagle Medium (DMEM; Gibco, Grand Island, NY) containing 2.0 mg/ml collagenase (Gibco, Grand Island, NY) and 0.5 mg/ml hyaluronidase (Worthington, Lakewood, NJ) overnight at 37 °C. Cells were spun down and cultured in DMEM (Gibco) with 1% FBS and 20 ng/ml TGF-b1 (R&D, Minneapolis, MN). The medium was changed every 48 h in all cultures.

BM-derived myofibroblast primary cultures were generated from the same four rabbits as described previously to generate cornea-derived myofibroblasts. Briefly, [13 = the tibias and femurs of rabbits were removed and BM cells were harvested by flushing medium and scratching the bone marrow cavity with the end of an 18-gauge needle. BM cells were collected in a petri dish and clumps were teased out and gently dissociated with a one ml pipette to form a single-cell suspension. The suspension of cells was centrifuged at 1500 rpm for 10 min at 4 °C to obtain a cell pellet. Red blood cells were lysed by adding sterile Milli-Q water at 4 °C, followed by dilution with 10X PBS at 4 °C at a ratio of one part PBS to nine parts cell solution, with immediate mixing. Cell suspensions were centrifuged again at 1500 rpm for 10 min at 4 °C and re-suspended in 1 × PBS at 4 °C. Cell viability in the range of 90% to 95% was verified by staining with 0.4% trypan blue. Cells were suspended in PBS at 4 °C at a final concentration of 2 × 10^6^ cells/ml. BM-derived cells, were cultured in DMEM (Gibco) with 1% FBS and 20 ng/ml TGF-β1 (R&D Systems, Minneapolis, MN). The medium was changed every 48 h in all cultures.

Cornea- and BM-derived myofibroblast cultures were each harvested from 6 T-75 flasks after 14 days of culture with 20 ng/ml TGF-β1, the cells washed with PBS, and cell pellets frozen at − 80 °C until analysis.

Cornea- and BM-derived myofibroblast cultures were grown in parallel in Nunc Lab-tek 8-well chamber slides (#154534, Thermo Fisher Sci, Waltham, MA), washed twice in PBS, and after fixation with IC Fixation Buffer (#00-8222-49 Thermo Fisher Scientific) for 10 min, washing twice with PBS and incubated for 1 h with 5% donkey serum. Immunocytochemistry was performed for myofibroblast-related markers by immersing cell layers in antibodies to α-SMA (M0851, DAKO, Glostrup, Denmark, 1:100), vimentin (Mab2105, R&D Systems, Minneapolis, MN, 1:50), desmin (D1033, Millipore Sigma, St Louis, MO, 1:40), or vinculin (MAB3574, Millipore Sigma, 1:100) in PBS for 1 h. Control immunocytochemistry in both cornea- and BM-derived myofibroblasts was performed by substituting isotypic control antibodies as the primary antibody (mouse IgG2a kappa cat#01-675-858, mouse IgG1 cat#02-610-0, or rat IgG2a cat#02-968-8 from Invitrogen, Carlsbad, CA). Slides were washed twice with PBS and incubated for 1 h with the corresponding Alexa Fluor (Thermo Fisher Scientific) secondary antibodies at 1:200 in PBS. Slides were washed 3 times in PBS before application of DAPI and a coverslip sealed with nail polish. Slides were analyzed and imaged with a Leica DM5000 microscope (Leica, Buffalo Grove, IL, USA) equipped with Q-imaging Retiga 4000RV (Surrey, BC, Canada) camera and Image-Pro software (Media Cybernetics Inc., Bethesda, MD, USA).

### Sample preparation

Individual pellets from each of the cornea- and BM-derived myofibroblast preparations were homogenized in 100 mM triethylammonium bicarbonate containing 2% SDS, the protein extracted three times from the cell debris and quantified by AccQ-Tag amino acid analysis^17^. Approximately 290 µg protein per rabbit was recovered from the corneal myofibroblast cultures and 850 µg protein per rabbit from the BM myofibroblast cultures. Soluble myofibroblast protein (100 µg) from each of the specimens was reduced with tris-(2-carboxyethyl) phosphine, cysteines alkylated with methyl methanethiosulfonate, then the protein was precipitated with acetone overnight. Protein pellets were washed two times with ice cold 67% acetone, gently blown-dry with argon and re-suspended in 50 mM mM triethylammonium bicarbonate containing 0.5 mM CaCl_2_ and digested overnight at 37 °C with trypsin (initially with 2% trypsin (w/w), followed in 2 h with another 2% (w/w), and the next day with another 1% (w/w) for 2 h additional incubation). Following proteolysis, soluble peptides were quantified by AccQ-Tag amino acid analysis.

### ITRAQ labeling and peptide fractionation

iTRAQ labeling with an 8-plex iTRAQ kit were performed as previously described^17–21^. In this study, tryptic digests of each myofibroblast preparation (100 µg/specimen) were labeled individually with a different iTRAQ tag and the labeled specimens mixed together in equal amounts and fractionated by reverse phase high performance liquid chromatography (RPHPLC) at pH 10 on a Waters xBridge BEH300 C18 column (3.5µ particle size, 2.1 × 100 mm). Chromatography was performed at a flow rate of 200 µL/min using aqueous acetonitrile/0.1% NH_4_OH solvents, a 0.5%/min acetonitrile gradient over 50 min; absorbance was monitored at 214 nm and fractions were collected at 1 min intervals. Chromatography fractions encompassing the entire elution were selectively combined, dried, and a total of 17 fractions were analyzed by LC MS/MS.

### Protein identification

RPHPLC pH10 chromatography fractions were analyzed by LC MS/MS with an Orbitrap Fusion Lumos Tribrid mass spectrometer^17–20^. Protein identification utilized the Mascot 2.6.2 search engine, and the UniProt rabbit reference proteome database version 20190730, (21,264 sequences, 982 reviewed and 20,282 unreviewed) and the UniProt human reference proteome database version 20190730 (96,464 sequences, 42,412 reviewed and 54,052 unreviewed). The UniProt rabbit database is currently incomplete therefore protein identification utilized both the rabbit and human databases as rabbits are closely related phylogenetically to primates^22^. Proteins were identified in four categories including: (1) proteins characterized only in the rabbit database; (2) proteins characterized in both the rabbit and human databases; (3) proteins uncharacterized in the rabbit database but characterized in the human database; and (4) proteins characterized only in the human database. Sequence Identity between identified rabbit and human proteins was determined using Blast 2.9.0^23^. Protein identification required detection of a minimum of two unique peptides per protein and a database gene symbol. Database search parameters were restricted to three missed tryptic cleavage sites, a precursor ion mass tolerance of 10 ppm, a fragment ion mass tolerance of 20 mmu and a false discovery rate of ≤ 1%. Fixed protein modifications included N-terminal and ε-Lys iTRAQ modifications and S-methyl-Cys. Variable protein modifications included Met oxidation, Asn and Gln deamidation and iTRAQ Tyr. A minimum Mascot ion score of 25 was used for accepting peptide MS/MS spectra.

### Protein quantitation

iTRAQ tags were quantified by the weighted average method^24^ using the Mascot 2.6.2 Summed Intensities program. Protein quantitation required a minimum of two unique peptides per protein, Mascot peptide ion scores ≥ 25, and utilized reporter ion tolerance of 10 ppm. Protein ratios were determined in log space and transformed for reporting. Proteins exhibiting average protein ratios above or below the mean by at least 1 standard deviation and *p* values ≤ 0.05 (pairwise moderated t-test adjusted for multiple testing) were considered significantly elevated or decreased.

### Statistics and bioinformatics

Programs available in R^25^, including the Limma package^26^, were used for normalizing the protein ratios to quantiles, determining standard error of the mean (SEM) and *p* values (moderated t-test) from paired samples with adjustment for multiple testing and for identifying differentially expressed (DE) proteins^27^. For average results, calculation of SEM and adjusted *p* values required ≥ 3 samples. Bioinformatic analyses were performed with Ingenuity Pathways Analysis (Qiagen).

### Western blot analysis

Western blot analysis of cornea-derived and BM-derived myofibroblasts was performed using a previously detailed method^16^ with a 7.5% acrylamide precast gels (Bio-Rad, Hercules, California) and enhanced chemiluminescence for signal detection (GE, Life Sciences, Marlborough, MA). Cellular protein (10 µg) was resolved on the 4–15% SDS-PAGE gels then transferred to PVDF membranes for immunoblotting. The membranes were blocked with 5% non-fat milk and probed with primary antibodies at 4 °C overnight. Primary antibodies were anti-collagen III (cat. no. ARG20786, Arigo Bio, Cedar lane NC, USA, 1:500 dilution), anti-collagen XI (cat no. LS-C151380, LS Bio, USA, 1:1000 dilution), anti-collagen VII (cat no. ab223639, Abcam, USA, 1:5000 dilution) and anti-β actin (cat. no. A5441, SIGMA, USA, 1:5000 dilution). Secondary antibodies (donkey anti-mouse –HRP, cat no. sc2314; donkey anti-goat –HRP, cat no. sc202) were obtained from Santa Cruz Biotechnology (Dallas, TX) and used at 1:10,000 dilution. Western blot signal intensities were quantified by densitometry using Image J software (NIH, Bethesda, MD).

## Results

### Overview

**Figure 1:**
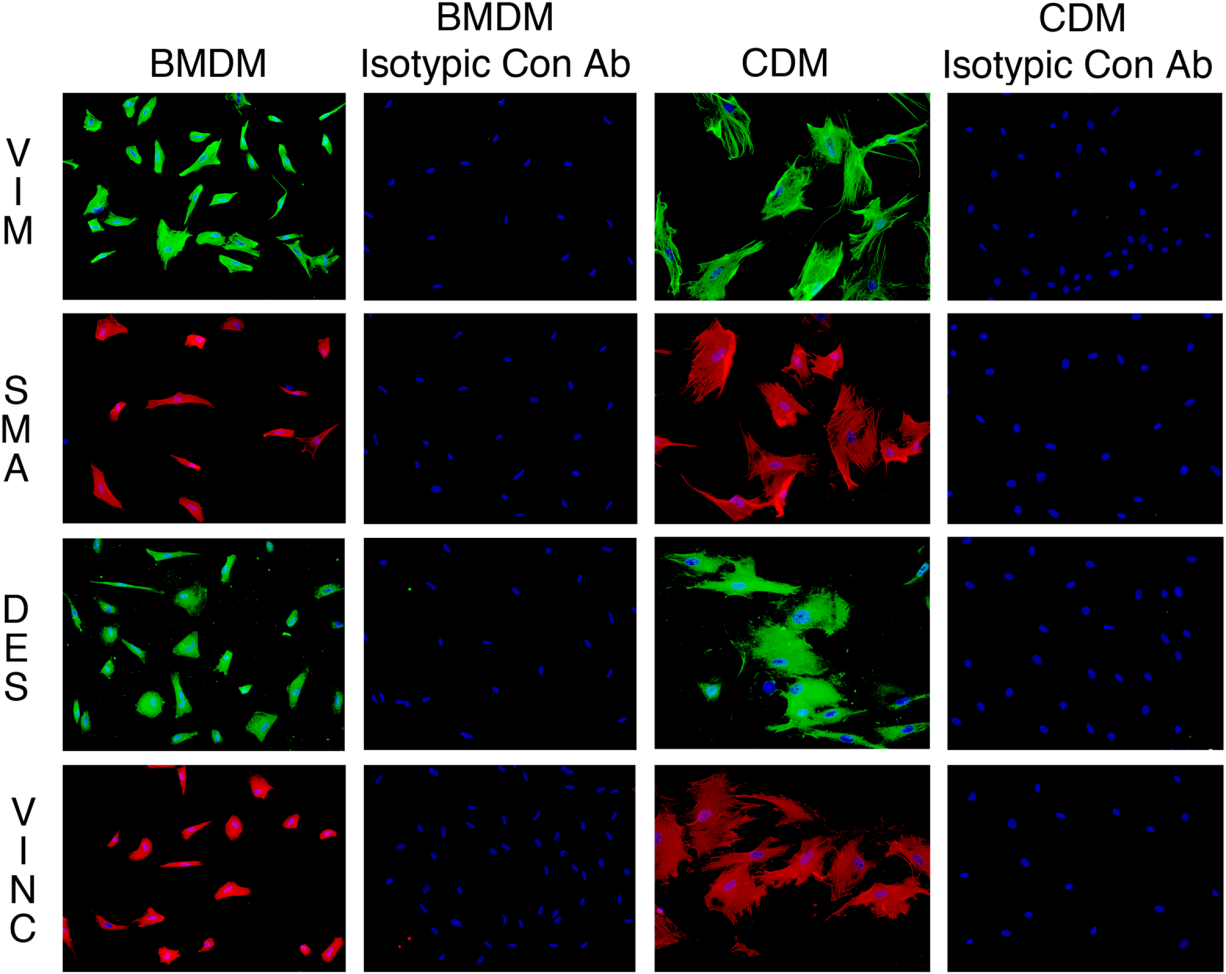
Myofibroblast marker immunocytochemistry in bone marrow-derived myofibroblasts (BMDM) or cornea-derived myofibroblasts (CDM). All cells had immunocytochemistry at 14 days in culture with 20 ng/ml TGF beta-1 for vimentin (VIM), alpha-smooth muscle actin (SMA), desmin (DES) and vinculin (VINC). Mag. ×200. Both BMDM and CDM expressed vimentin, alpha-smooth muscle actin, and desmin indicating they are both VAD-positive myofibroblasts. In addition, both myofibroblasts produced vinculin, another marker commonly produced by myofibroblasts. The corresponding isotypic antibody control immunocytochemistry was also performed for each marker in each myofibroblast cell type. Blue is DAPI staining of nuclei. Mag. 200×.

**Figure 2:**
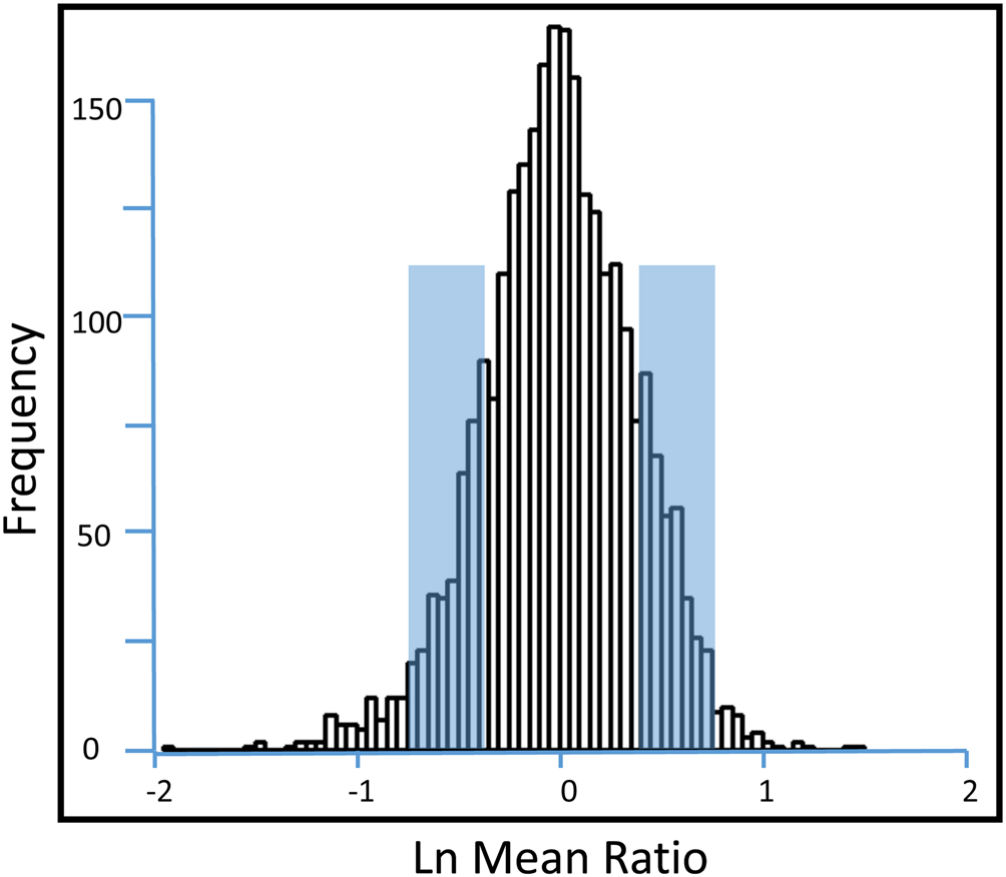
Distribution of myofibroblast protein ratios. The LN mean distribution of myofibroblast protein ratios (cornea/BM) are shown for proteins quantified in paired samples from four different rabbits. The histogram represents a total of 2420 proteins quantified with ≥ 2 unique peptides. LN median = 0, LN Mean = 0, and SD = 0.38. The distribution of protein ratios is near-to-normal and statistically appropriate for comparing the proteomes of myofibroblasts derived from cornea and BM.

**Figure 3:**
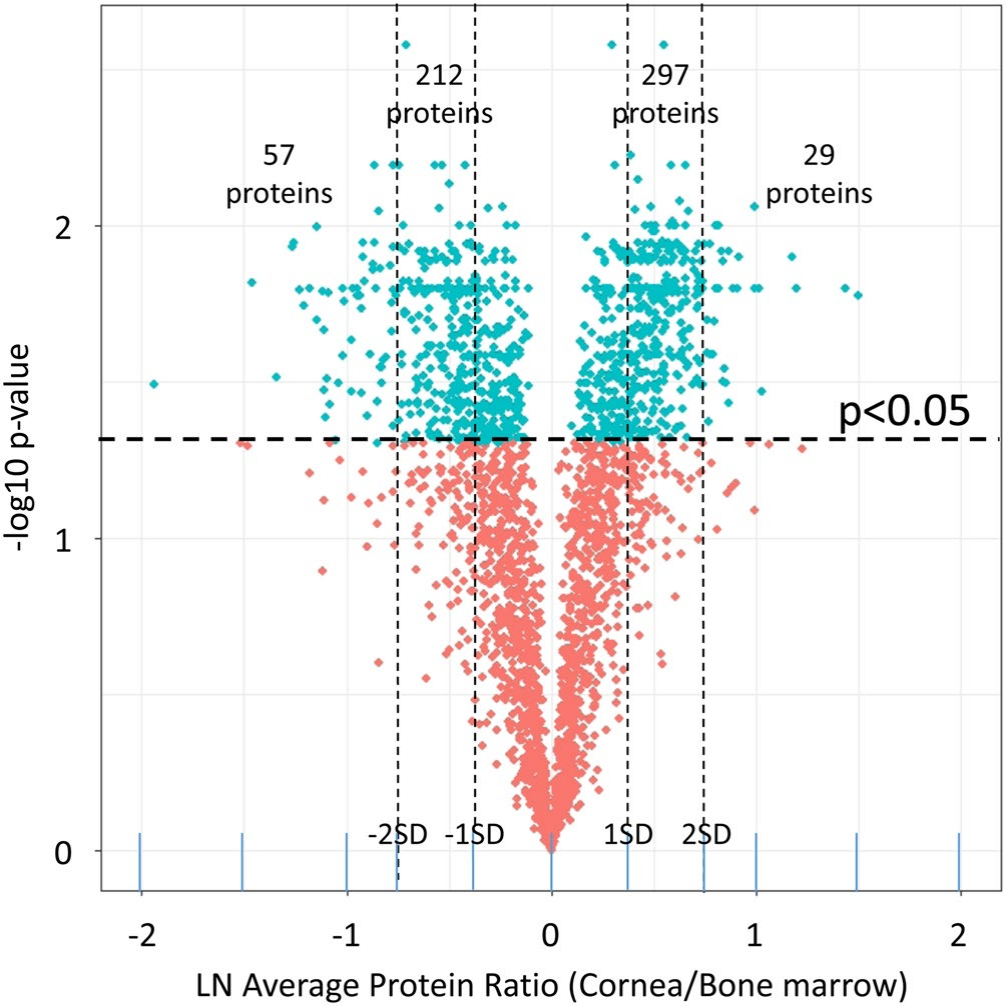
Differentially expressed myofibroblast proteins. This volcano plot shows LN average protein ratios (cornea/BM) versus *p* values (paired t-test adjusted for multiple testing) for 2329 proteins quantified in ≥ 3 paired myofibroblast specimen. Vertical dashed lines represent protein fold changes above and below the mean by 1 standard deviations (SD), with proteins above the horizontal line (*p* values < 0.05) considered differentially expressed.

Cornea and BM were isolated from four New Zealand white rabbits and four myofibroblast primary cultures were generated from each tissue. Myofibroblast identity and homogeneity were confirmed by immunocytochemistry (Fig. 1) and paired cornea- and BM-derived myofibroblast specimens were analyzed by LC MS/MS iTRAQ technology, yielding a total of 2420 proteins quantified, of which 2329 were quantified in ≥ 3 rabbits. Proteomic results are summarized in Table 1 and presented in detail for each rabbit in Supplemental Tables S1–S4. These results include protein accession numbers and descriptions, gene symbols, protein ratios (cornea/BM), number of unique peptides, number of summed peptide intensities, percent sequence coverage, database identification category, and percent identity between rabbit and human proteins. The proteomic results from all four paired samples were similar in quality and exhibited near-to-normal distributions (Fig. 2) and therefore the quantitation was suitable for averaging. The mean relative abundance of proteins quantified in all the myofibroblast samples is presented in Supplemental Table S5, including sample frequency, standard error of the mean, and moderated *p* values adjusted for multiple testing, A total 673 DE proteins were identified, as illustrated by Volcano plot (Fig. 3) and itemized in Supplemental Table S5 with color coding. Criteria for identifying DE proteins included: (i) quantification in ≥ 3 of the paired myofibroblast samples; (ii) an average protein ratio above or below the mean by at least 1 standard deviation (SD), and (iii) an average ratio with an adjusted *p* value ≤ 0.05.

**Table 1:** Rabbit myofibroblast proteomic analysis summary.

Rabbit cornea- and bone marrow-derived myofibroblast primary cultures	4 each
Total proteins quantified with ≥ 2 peptides	2420
Proteins derived from ≥ 3 rabbits and identified with ≥ 2 peptides	2329
Total differentially expressed myofibroblast proteins identified	673

Primary cultures of cornea- and BM-derived myofibroblast were obtained from four rabbits and paired specimens analyzed by LC MS/MS iTRAQ technology. Criteria for differentially expressed myofibroblast proteins included protein ratios (cornea/bone marrow) ≥ 1 SD from the mean with *p* ≤ 0.05 in ≥ 3 rabbits.

### Proteomic comparison of myofibroblasts from cornea or BM

Comparative proteomic analysis of myofibroblasts was performed with 2329 proteins quantified in ≥ 3 of the four paired cornea and BM myofibroblast samples. Three hundred sixty proteins (~ 15%) were found significantly more abundant in cornea- than BM-derived myofibroblasts and are considered to be differentially expressed (Table S5). Thirty-three of these 360 proteins exhibited ratios ≥ 2 SD from the mean. Three hundred thirteen DE proteins were found significantly more abundant in BM- than cornea-derived myofibroblasts (Table S5), including 63 proteins exhibiting ratios ≥ 2 SD from the mean. Proteomic differences between these two types of myofibroblasts are suggested by a comparison of 30 proteins that are significantly more abundant in cornea-(Table 2) or BM-(Table 3) derived progenitor cells. Bioinformatic comparison of all DE proteins more abundant in either the cornea-derived myofibroblasts (Table 4) or BM-derived myofibroblasts (Table 5) support significant differences in the molecular and cellular functions of these two types of myofibroblasts.

**Table 2:** Differentially expressed proteins proteins significantly more abundant in rabbit myofibroblasts from cornea than from BM.

Rabbit accession UniProt	Human accession UniProt	Gene symbol	Protein	Sample frequency	Linear ratio cornea/bone marrow	% identity rabbit and human proteins	Adjusted *p* value^D^
G1SIW5	Q92820	GGH	Folate gamma-glutamyl hydrolase	4	4.78	82	3.5E−04
G1U4P8	Q9BXN1	ASPN	Asporin	4	3.05	88	3.5E−04
G1SDA2	P29762	CRABP1	FABP domain-containCellular retinoic acid-binding protein 1ing protein	4	2.89	99	1.7E−04
G1TW43	J3QSU6	TNC	Tenascin	4	2.73	74	1.7 E−04
G1ST69	P20700	LMNB1	Lamin-B1	4	2.73	98	1.7 E−04
G1T380	Q02388	COL7A1	Collagen alpha-1(VII) chain	4	2.73	87	1.6 E−03
G1SJF4	A0A0A0MR51	FADS1	Acyl-CoA (8–3)-desaturase	4	2.66	93	4.7E−04
G1T3Y8	P10809	HSPD1	60 kDa heat shock protein, mitochondrial	4	2.66	99	1.7E−04
G1SJW7	J3QLE5	SNRPN	Small nuclear ribonucleoprotein-associated protein	4	2.57	100	1.7E−04
G1SK42	P21980	TGM2	Protein-glutamine gamma-glutamyltransferase 2	4	2.55	87	2.8E−04
G1SEF5	Q8NAV1	PRPF38A	Pre-mRNA-splicing factor 38A	4	2.52	100	3.3E−04
G1SNX5	B4DY09	ILF2	Interleukin enhancer-binding factor 2	4	2.45	100	1.7E−04
G1T2X0	Q99541	PLIN2	Perilipin-2	4	2.45	88	2.6E−03
G1SI79	P51991	HNRNPA3	Heterogeneous nuclear ribonucleoprotein A3	4	2.44	100	1.7E−04
G1SFC6	A0A087WV29	NAT10	RNA cytidine acetyltransferase	4	2.41	96	2.2E−04
G1TD26	D6RAA6	TMEM33	Transmembrane protein 33 (Fragment)	4	2.40	99	1.7E−04
G1SUP9	P46087	NOP2	Probable 28S rRNA (cytosine(4447)-C(5))-methyltransferase	4	2.40	77	1.9E−04
P00389	P16435	POR	NADPH–cytochrome P450 reductase	4	2.37	92	2.5E−04
G1TLW3	J3KTA4	DDX5	Probable ATP-dependent RNA helicase DDX5	4	2.35	96	1.7E−04
G1SDW8	H0Y2P0	CD44	CD44 antigen (Fragment)	4	2.34	91	5.2E−04
G1T4M2	O60264	SMARCA5	SWI/SNF-related matrix-associated actin-dependent regulator of chromatin subfamily A member 5	4	2.321	100	2.5E−04
G1T2K5	I3L1L3	MYBBP1A	Myb-binding protein 1A (Fragment)	4	2.318	69	1.9E−04
G1SI26	Q4VC31	CCDC58	Coiled-coil domain-containing protein 58	4	2.277	95	1.7E−04
G1TX84	Q9Y5J1	UTP18	U3 small nucleolar RNA-associated protein 18 homolog	4	2.269	89	1.7E−04
G1SMB3	Q9NV31	IMP3	U3 small nucleolar ribonucleoprotein protein IMP3	4	2.260	99	1.7E−04
G1SE74	Q7KZ85	SUPT6H	Transcription elongation factor SPT6	4	2.247	99	2.8E−04
G1T3Y0	P98082	DAB2	Disabled homolog 2	4	2.233	90	3.3E−04
G1SIJ6	Q13308	PTK7	Inactive tyrosine-protein kinase 7	4	2.232	93	1.7E−04
G1TD41	G8JLB6	HNRNPH1	Heterogeneous nuclear ribonucleoprotein H	4	2.226	98	1.7E−04
G1T6T0	J3KNJ3	NAALAD2	N-acetylated-alpha-linked acidic dipeptidase 2	4	2.215	89	1.9E−04

The above 30 proteins were selected from 360 differentially expressed proteins more abundant in rabbit myofibroblasts from cornea than from BM. Each exhibited a protein ratio ≥ 1 SD from the mean and an adjusted *p*values ≤ 0.05 in ≥ 3 paired myofibroblast samples. All differentially expressed proteins are illustrated in Fig. 2 and identified in Supplemental Table 5.

**Table 3:** Differentially expressed proteins proteins significantly more abundant in rabbit myofibroblasts from bm than from cornea.

Rabbit accession UniProt	Human accession UniProt	Gene symbol	Protein	Sample frequency	Linear ratio bone marrow/cornea	% identity rabbit and human proteins	Adjusted *p* value^D^
G1SMS2	Q8WX93	PALLD	Palladin	4	4.43	87	2.0E−04
G1T5T8	O00151	PDLIM1	PDZ and LIM domain protein 1	4	3.32	92	1.7E−04
G1SZ00	P21291	CSRP1	Cysteine and glycine-rich protein 1	4	3.08	98	3.2E−02
G1SW77	Q8NC51	SERBP1	Plasminogen activator inhibitor 1 RNA-binding protein	4	3.05	99	1.7E−04
G1T387	Q14247	CTTN	Src substrate cortactin	4	2.96	88	1.7E−04
G1SKS8	E7EVA0	MAP4	Microtubule-associated protein	4	2.94	85	3.4E−04
G1TAJ3	P00338	LDHA	L-lactate dehydrogenase A chain	4	2.94	94	1.9E−03
G1TYY5	Q14847	LASP1	LIM and SH3 domain protein 1	4	2.90	94	1.7E−04
G1SUI9	E7EX44	CALD1	Caldesmon	4	2.89	84	1.7E−04
G1T315	A0A2R8Y2R1	SGCE	Epsilon-sarcoglycan (Fragment)	4	2.80	88	1.7E−04
G1SYJ4	P06733	ENO1	Alpha-enolase	4	2.76	96	9.6E−04
G1T4F9	E9PR44	CRYAB	Alpha-crystallin B chain (Fragment)	4	2.74	98	1.0E−02
G1U634	P08473	MME	Neprilysin	4	2.74	94	1.5E−03
G1T5B6	P12107	COL11A1	Collagen alpha-1(XI) chain	4	2.63	98	1.1E−03
G1SW82	H0Y9Y3	SYNPO2	Synaptopodin-2 (Fragment)	4	2.61	80	3.9E−04
G1TN29	Q86UU1	PHLDB1	Pleckstrin homology-like domain family B member 1	4	2.54	89	1.9E−04
G1T7Z6	P00558	PGK1	Phosphoglycerate kinase 1	4	2.49	99	4.7E−04
G1SPQ9	O94875	SORBS2	Sorbin and SH3 domain-containing protein 2	4	2.47	90	1.7E−04
G1T8J0	P02461	COL3A1	Collagen alpha-1(III) chain	4	2.46	92	3.8E−03
G1SRR2	E9PMP7	LMO7	LIM domain only protein 7 (Fragment)	4	2.43	92	1.9E−04
G1SPY1	E9PGM4	GBE1	1,4-alpha-glucan-branching enzyme	4	2.40	93	2.5E−04
G1SPY1	E9PGM4	GBE1	1,4-alpha-glucan-branching enzyme	4	2.40	70	2.5E−04
G1SUY3	F8VQR7	CSRP2	Cysteine and glycine-rich protein 2	4	2.39	100	1.9E−04
G1SFG6	A0A0A0MTS2	GPI	Glucose-6-phosphate isomerase (Fragment)	4	2.37	93	6.1E−04
G1TE78	Q15121	PEA15	Astrocytic phosphoprotein PEA-15	4	2.36	100	2.0E−04
P35748	P35749	MYH11	Myosin-11	4	2.34	97	1.1E−02
G1TA83	P09525	ANXA4	Annexin A4	4	2.31	94	1.7E−04
G1TRY5	P13797	PLS3	Plastin-3	4	2.31	100	1.7E−04
G1T2K1	O95340	PAPSS2	Bifunctional 3 ~ -phosphoadenosine 5 ~ -phosphosulfate synthase 2	4	2.22	94	4.5E−04
G1SHL8	P42224	STAT1	Signal transducer and activator of transcription 1-alpha/beta	4	2.22	95	1.7E−04

The above 30 proteins were selected from 313 differentially expressed proteins more abundant in rabbit myofibroblasts derived from BM than from cornea. Each exhibits a protein ratio ≥ 1 SD from the mean and an adjusted *p* values ≤ 0.05 in ≥ 3 paired myofibroblast samples. All differentially expressed proteins are illustrated in Fig. 2 and identified in Supplemental Table 5.

**Table 4:** Bioinformatic analyses of proteins more abundant in cornea-derived myofibroblasts.

	BH *p* value
*Top canonical pathways*	
Mitochondrial dysfunction	1.9E−10
Oxidative phosphorylation	3.9E−09
Sirtuin signaling pathway	5.0E−08
*Top molecular and cellular functions*	
Processing of RNA	5.1E−74
Splicing of mRNA	3.4E−56
Transport of mRNA	3.2E−16
Export of mRNA	4.8E−16
Translation	3.6E−14
Translation of protein	3.6E−13
Homologous recombination of cells	8.7E−11
DNA recombination	8.1E−07

Bioinformatic properties of 360 differentially expressed proteins more abundant in cornea- than BM-derived myofibroblasts were determined using Ingenuity Pathway Analysis. BH *p* value refers to the false discovery rate^37^.

**Table 5:** Bioinformatic analyses of proteins more abundant in BM-derived myofibroblasts.

	BH *p* value
*Top canonical pathways*	
Actin cytoskeleton signaling	7.9E−09
Glycolysis I	7.9E−09
Integrin signalling	1.6E−08
Remodeling of epithelial adherens junctions	1.6E−08
*Top molecular and cellular functions*	
Organization of cytoplasm	4.2E−24
Degranulation of cells	7.1E−21
Organization of cytoskeleton	1.3E−20
Microtubule dynamics	4.3E−15
Fibrogenesis	4.3E−15
Formation of filaments	1.0E−14
Cell movement	1.5E−12
Formation of cellular protrusions	4.7E−11

Bioinformatic properties of 313 differentially expressed proteins more abundant in BM- than cornea-derived myofibroblasts were determined using Ingenuity Pathway Analysis. BH *p* value refers to the false discovery rate^37^.

### Independent evidence supporting the iTRAQ protein quantitation

**Figure 4:**
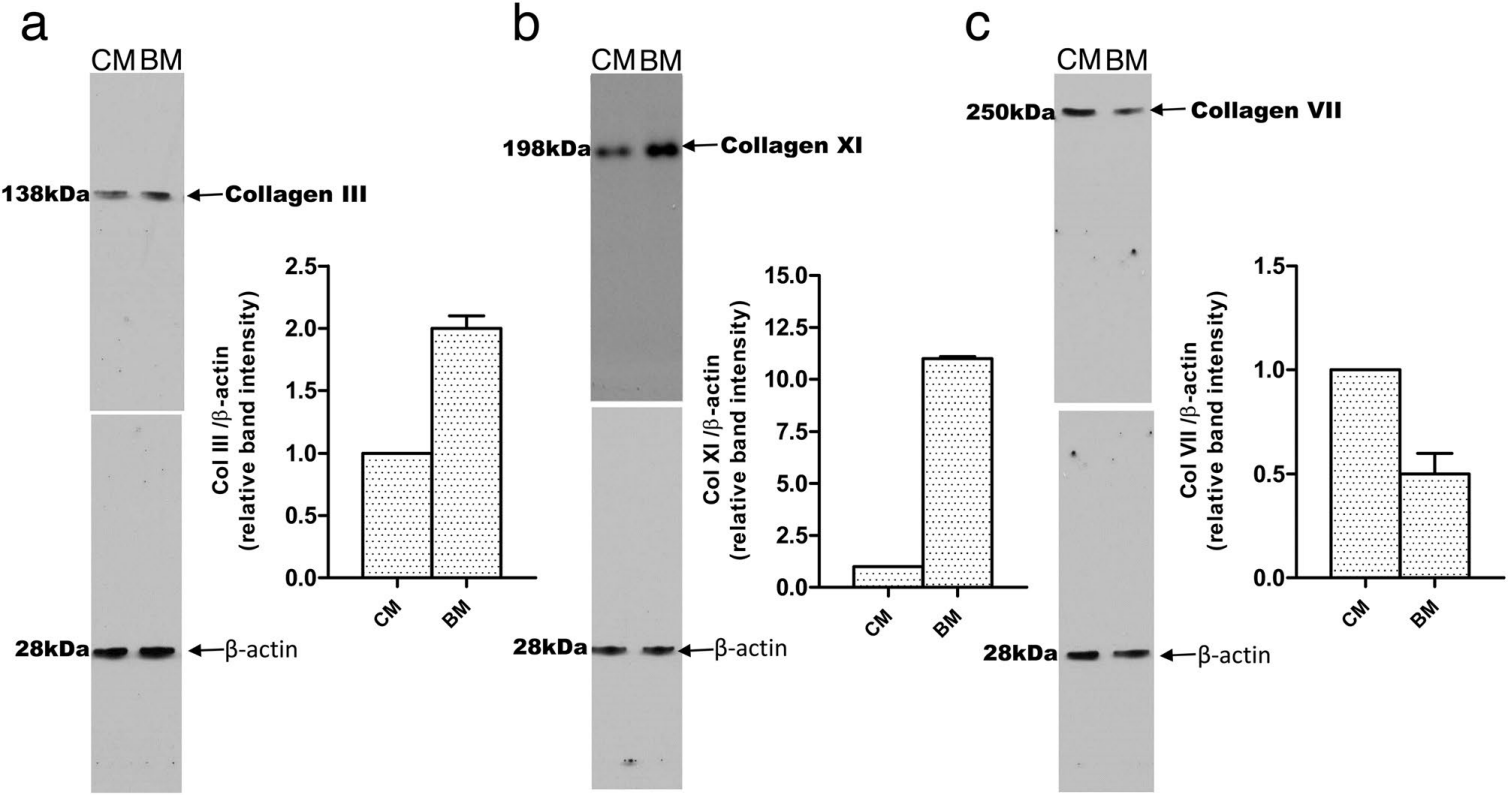
Western blot analysis. Western blot analysis was used to evaluate the relative amount of three proteins in cornea derived and BM derived myofibroblast. (**a**) Collagen type III, (**b**) collagen type XI, and (**c**) collagen type VII expression measured by western blotting (upper panels in **a**, **b**, and **c**). A representative full-length western blot of the three performed for each protein is shown. Beta actin western blots were used to demonstrate equivalent loading (lower panels in **a**, **b**, and **c**). Each blot underwent densitometric analysis using Image J software (NIH, Bethesda, MD) of each of the three western blots from different experiments (n = 3); error bars reflect mean ± SD. All three differences were statistically significant (*P* < 0.05). *CM* corneal keratocyte-derived myofibroblast; *BM* BM-derived myofibroblasts.

Western blot analysis was used to independently corroborate the iTRAQ protein quantitation by analysis of three proteins likely to be involved in fibrosis mediated by myofibroblasts (collagen type III, collagen type VII and collagen type XI.) Consistent with the iTRAQ quantitation, immunoblot results confirmed that collagen type III and collagen type XI were decreased (Fig. 4A, B) and collagen type VII was elevated (Fig. 4C) in cornea-derived myofibroblasts compared to BM-derived myofibroblasts, respectively.

## Discussion

Myofibroblasts are critical mediators of fibrosis that may occur in most organs of animals after injury^1^. Typically, at least two progenitor cells to myofibroblasts have been found to participate in the pathophysiology of fibrosis in each organ evaluated^7–9,13^. In the present study, we have compared the proteome of corneal keratocyte-derived myofibroblasts to that of BM-derived myofibroblasts—both of which have been shown to be major progenitors to myofibroblasts in fibrosis that occurs after corneal injury^6,13^. The keratocyte-derived and BM-derived myofibroblasts used in this study were at similar stages of myofibroblast differentiation as cell culture procedures were carefully coordinated and 100% of the cells in all cultures were α-SMA+^16^.

The present study used quantitative proteomics technology to identify DE proteins in myofibroblasts derived from cornea and BM progenitors. The data indicates about 29% of the proteins quantified were differentially expressed between these two types of myofibroblasts. Proteomic differences were confirmed by Western blot analysis of three proteins likely to be involved in the pathophysiology of fibrosis, namely collagen type III, collagen type VII and collagen type XI. Clues to progenitor-dependent differences in myofibroblasts were suggested by bioinformatic analysis of the DE proteins. Canonical pathways involving mitochondrial dysfunction, oxidative phosphorylation and sirtuin signaling were implicated as most predominant in cornea-derived cells (Table 4), and pathways involving glycolysis I, integrin signaling and remodeling of epithelial adherens junctions as most predominant in BM-derived cells (Table 5). Molecular and cellular functions of cornea- and BM-derived myofibroblasts as projected from Ingenuity Pathway Analysis of the DE proteins, also were significantly different. RNA processing, mRNA splicing, transport and export, protein synthesis, and homologous cell recombination were the top functions implicated by bioinformatic analysis of DE proteins more abundant in cornea-derived myofibroblasts (Table 4). Top functions of DE proteins more abundant in BM-derived myofibroblasts were bioinformatically centered on cellular organization, degranulation of cells, microtubule dynamics, fibrogenesis, cell movement, and formation of filaments and cell protrusions (Table 5). For example, since BM-derived myofibroblasts produce much more collagen type XI and collagen type III, they likely contribute greatly to structure and strength of fibrotic tissue in the cornea and may contribute most of the collagen type III deposited in the cornea after injuries^28^. Conversely, since corneal keratocyte-derived myofibroblasts produce more collagen type VII, they may be more likely to modulate cytokine production by adjacent fibroblasts in the healing stroma^29^.

The bioinformatic results support the likelihood of progenitor-dependent functional differences in myofibroblasts, however, potentially limiting factors warrant acknowledgement. These factors include the relatively small sample size employed, namely only four rabbits, and the fact that the rabbit protein sequence databases are not well developed and are incompletely curated. Nevertheless, rabbits are close phylogenetic relatives of humans^22^ and utilization of the human UniProt sequence database, one of the most complete and well-curated databases available, supports the reliability of the protein identifications. Notably, the overall level of significant proteomic differences (~ 29%) observed between cornea- and BM-derived myofibroblasts is 2–3× greater than the variability reported from repetitive proteomic analysis of a variety of normal tissues^30,31^. Furthermore, the levels of three different collagens thought to contribute to corneal fibrosis, were confirmed to be different between keratocyte-derived myofibroblasts and BM-derived myofibroblasts. It’s possible that the BM-derived myofibroblasts analyzed in this study could have been heterogeneous if they were derived from both bone marrow fibrocytes and bone marrow stromal cells after stimulation with TGF beta-1^32,33^. Another possible limitation is that these two types of myofibroblasts were cultured and characterized in vitro, and it is not certain that the cells generated in vivo and in vitro are the same. However, the expression of vimentin, α-SMA and desmin found for both myofibroblast types in vitro was similar to findings after fibrosis-producing injuries in situ^34^.

The results of this study suggest that the myofibroblasts derived from different progenitors contribute differentially, and perhaps additively, to the fibrosis response to injury in the cornea. This further suggests that the character of the fibrotic tissue may vary depending on the relative contributions of the myofibroblast progenitors. For example, after anterior corneal injury produced by irregular phototherapeutic keratectomy (PTK) to inhibit epithelial basement membrane regeneration in mice, 30 to 70% of myofibroblasts were derived from BM-derived progenitors, with the remaining myofibroblasts developed from keratocyte-derived progenitors^6^, although some possibly developed from Schwann cells^35^. How the variation in myofibroblasts would affect properties of fibrosis such as contractility, opacity or persistence in the cornea remains unknown. A recent in vitro study^36^ showed that the numbers of alpha-smooth muscle actin+ myofibroblasts generated from either keratocyte-derived precursors or BM-derived precursor cells were higher when both cells were co-cultured together in a culture flask (juxtacrine) as compared to when BM-derived precursor cells and keratocyte-derived precursor cells were co-culture in different compartments of a Transwell System (paracrine). This suggests that the presence of the two different myofibroblasts cells in the stroma after injury may potentiate the overall fibrosis response. Our current proteomic and bioinformatic results suggest that BM-derived myofibroblasts may be more prone than cornea-derived precursors to impact cellular organization and the formation of excessive cellular and extracellular material characteristic of fibrosis. Hopefully, the findings of this study will stimulate future research to better understand the contributions of myofibroblasts from different precursors to the fibrosis response, and toward the development of more effective therapies to fibrotic tissue damage.

## Supplementary information

Supplementary information.

## Data Availability

Data generated during this study are included as Supplemental Tables S1–S5 and as Supplemental Figure S1. The original mass spectra are publicly available from MassIVE (https://massive.ucsd.edu) using the identifier MSV000084599.
