# Supplementary material for: Quantitative proteomic comparison of myofibroblasts derived from bone marrow and cornea
Source: Sci Rep. 2020 Oct 7;10:16717. doi: 10.1038/s41598-020-73686-w (PMC7541534; doi:10.1038/s41598-020-73686-w)
Supplement: Supplementary file 1 — Supplementary information. [file 41598_2020_73686_MOESM1_ESM.pdf]

SUPPLEMENTARY MATERIALS

Quantitative Proteomic Comparison of Myofibroblasts Derived from Bone Marrow and Cornea

Paramananda Saikia[#1] Jack S. Crabb[#1,2], Luciana L. Dibbin[1], Madison J. Juszczak[1], Belinda Willard[2], Geeng-Fu Jang [1,2], Thomas Michael Shiju[1], John W. Crabb[1,2,3*], and Steven E. Wilson[1,3*]

Cole Eye Institute[1] and Lerner Research Institute[2], Cleveland Clinic, and the Cleveland Clinic Lerner College of Medicine of Case Western Reserve University[3], Cleveland, OH 44195

# These authors contributed equally to this work

Full length Western blots and beta-actin loading western blots

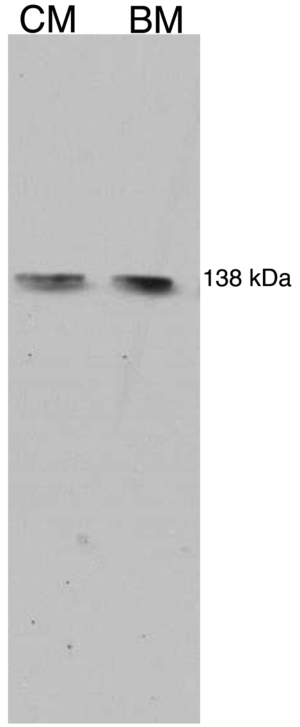

Collagen type III full length WB

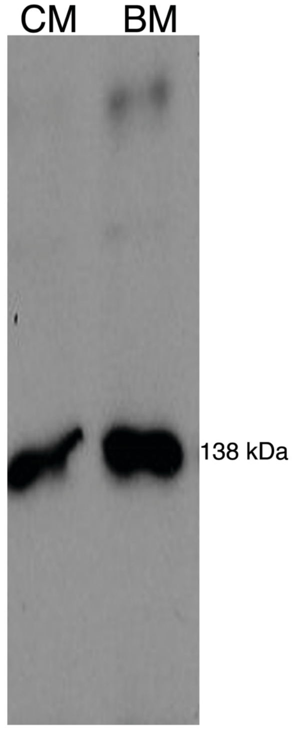

Collagen type III full length WB

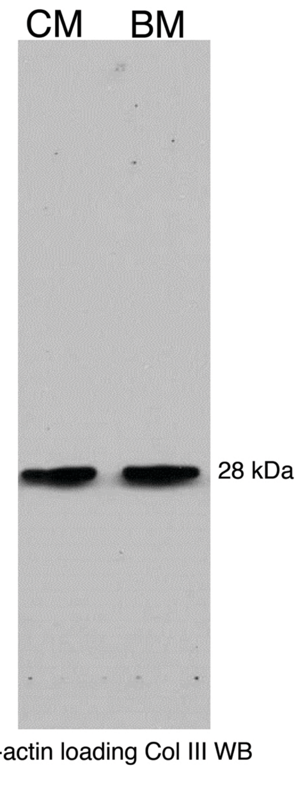

β-actin loading Col III WB

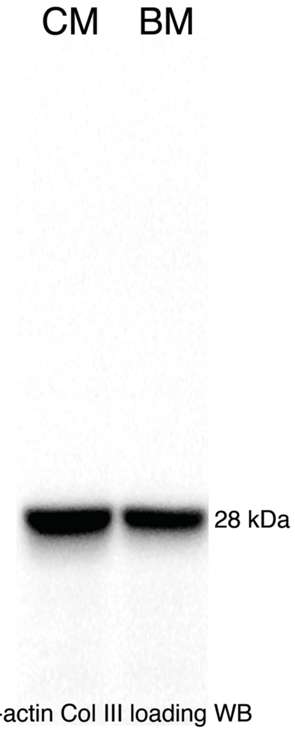

β-actin Col III loading WB

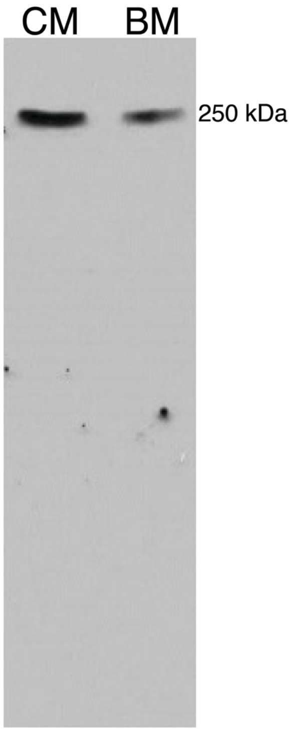

Collagen type VII full length WB

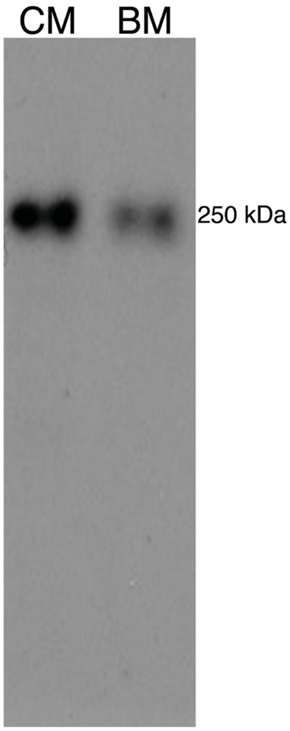

Collagen type VII full length WB

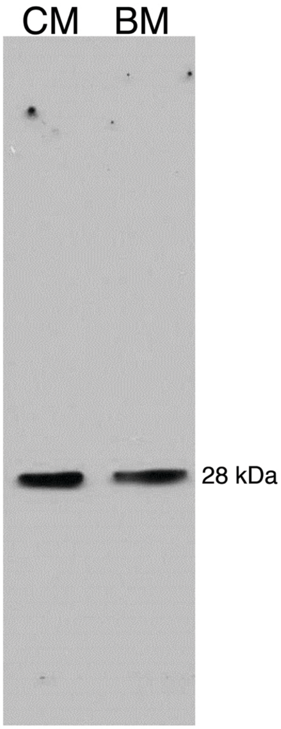

β-actin loading COL VII WB

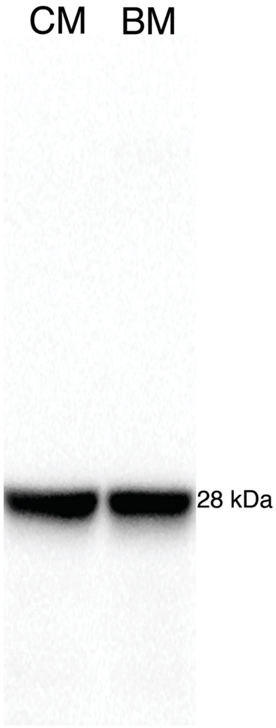

β-actin loading COL VII WB

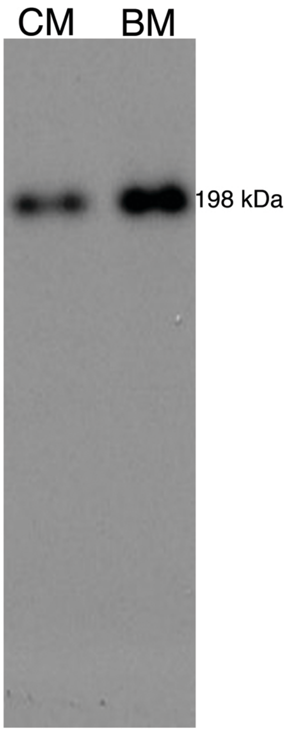

Collagen type XI full length WB

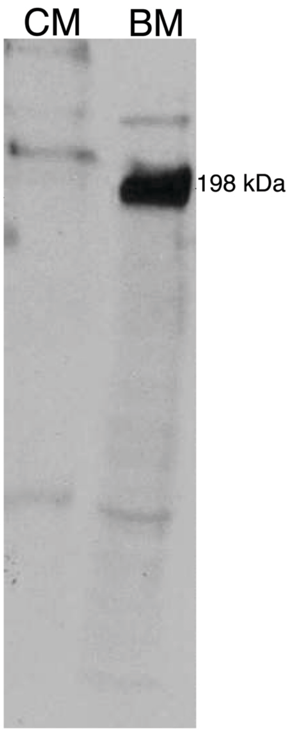

Collagen type XI full length WB

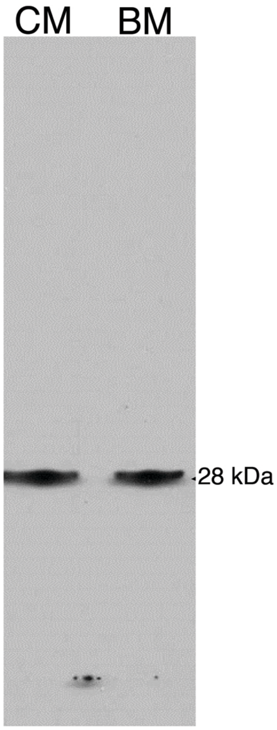

β-actin loading COL XI WB

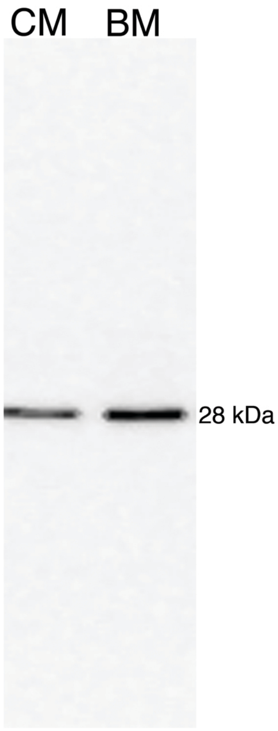

β-actin loading COL XI WB

Supplemental Table S1
Myofibroblasts from Rabbit Cornea & Bone Marrow
Relative Protein Abundance, Rabbit 24
Orbitrap Fusion Lumos Tribrid LC MS/MS iTRAQ Results

Total Proteins Quantified = 2383; Median Protein iTRAQ Ratio =1.018; Mean Protein iTRAQ Ratio = 1.000; Standard Deviation (SD) = 0.428
Yellow = 1SD from the mean; Brown = 2SD from the mean

Rabbit Accession UniProt	Human Accession UniProt	Gene Symbol	Rabbit Protein Description	Human Protein Description	% identity[A]	Database Identification category[B]	Linear Ratio Cornea/Bone marrow	Peptides[C]	N[D]	% Sequence Coverage
	A0A2R8Y7G9	H3.Y		Histone domain-containing protein		4	149.1586	2	2	29
G1TEP2		FAM210B	DUF1279 domain-containing protein			1	5.3405	2	2	12
G1T380	Q02388	COL7A1	Uncharacterized protein	Collagen alpha-1(VII) chain	87	3	4.7064	16	23	8
G1SJF4	A0A0A0MR51	FADS1	Fatty acid desaturase 1	Acyl-CoA (8-3)-desaturase	93	2	4.2217	3	5	6
G1SIW5	Q92820	GGH	Folate gamma-glutamyl hydrolase	Gamma-glutamyl hydrolase	82	2	4.0061	6	16	28
	P02765	AHSG		Alpha-2-HS-glycoprotein		4	3.7941	2	2	5
G1SEF5	Q8NAV1	PRPF38A	PRP38_assoc domain-containing protein	Pre-mRNA-splicing factor 38A	100	2	3.6595	2	5	9
G1TG89	P62244	RPS15A	Uncharacterized protein	40S ribosomal protein S15a	100	3	3.5950	7	24	55
O19105		SLC1A5	Neutral amino acid transporter B(0)			1	3.5849	4	6	14
	P16104	H2AFX		Histone H2AX		4	3.2614	5	23	53
G1T3Y8	P10809	HSPD1	Uncharacterized protein	60 kDa heat shock protein, mitochondrial	99	3	3.0559	34	56	75
G1SK42	P21980	TGM2	TGc domain-containing protein	Protein-glutamine gamma-glutamyltransferase 2	87	2	2.8688	22	74	51
G1TW43	J3QSU6	TNC	Uncharacterized protein	Tenascin	74	3	2.8301	6	8	3
G1SUP9	P46087	NOP2	SAM_MT_RSMB_NOP domain-containing protein	Probable 28S rRNA (cytosine(4447)-C(5))-methyltransferase	77	2	2.8237	6	6	12
G1SI79	P51991	HNRNPA3	Uncharacterized protein	Heterogeneous nuclear ribonucleoprotein A3	100	3	2.7804	8	22	25
	A0A087X2B1	RBFOX1		RNA binding protein fox-1 homolog		4	2.7518	2	3	3
G1TD26	D6RAA6	TMEM33	Uncharacterized protein	Transmembrane protein 33 (Fragment)	99	3	2.7463	3	6	12
G1U4P8	Q9BXN1	ASPN	LRRNT domain-containing protein	Asporin	88	2	2.6716	5	6	19
G1SJW7	J3QLE5	SNRPN	Small nuclear ribonucleoprotein-associated protein	Small nuclear ribonucleoprotein-associated protein N (Fragment)	100	2	2.6596	6	11	19
G1SLK4	Q8WVX9	FAR1	Fatty acyl-CoA reductase	Fatty acyl-CoA reductase 1	93	2	2.6440	2	2	7
G1SDA2	P29762	CRABP1	FABP domain-containing protein	Cellular retinoic acid-binding protein 1	99	2	2.5951	5	19	41
G1TMU1	Q13151	HNRNPA0	Uncharacterized protein	Heterogeneous nuclear ribonucleoprotein A0	78	3	2.5610	2	3	10
G1SIJ6	Q13308	PTK7	Protein tyrosine kinase 7 (inactive)	Inactive tyrosine-protein kinase 7	93	2	2.5555	28	59	39
G1TB57		MRPS35	MRP-S28 domain-containing protein			1	2.5527	3	4	16
	B1ANM7	FAF1		FAS-associated factor 1		4	2.5445	3	3	7
G1TV19	H3BPE7	FUS	FUS RNA binding protein	RNA-binding protein FUS	80	2	2.5251	5	3	9
A0A140TAV7	J3KPF3	SLC3A2	4F2 cell-surface antigen heavy chain	4F2 cell-surface antigen heavy chain	81	2	2.4855	20	106	57
G1TCA0	Q96KR1	ZFR	DZF domain-containing protein	Zinc finger RNA-binding protein	99	2	2.4809	3	2	4
G1TUE1		ATP1B1	Sodium/potassium-transporting ATPase subunit beta			1	2.4634	4	5	15
G1SMB3	Q9NV31	IMP3	S4 RNA-binding domain-containing protein	U3 small nucleolar ribonucleoprotein protein IMP3	99	2	2.4514	2	2	15
G1T6T0	J3KNJ3	NAALAD2	Uncharacterized protein	N-acetylated-alpha-linked acidic dipeptidase 2	89	3	2.4329	11	18	23
G1SNF2	P13995	MTHFD2	Uncharacterized protein	Bifunctional methylenetetrahydrofolate dehydrogenase/cyclohydrolase, mitochondrial	93	3	2.4265	12	34	57
P00389	P16435	POR	NADPH--cytochrome P450 reductase	NADPH--cytochrome P450 reductase	92	2	2.4164	27	70	49
G1ST69	P20700	LMNB1	Uncharacterized protein	Lamin-B1	98	3	2.4154	19	38	42
G1T6N3		NPC2	ML domain-containing protein			1	2.4035	3	6	21
	Q9Y2Z2	MTO1		Protein MTO1 homolog, mitochondrial		4	2.3509	2	2	4
G1SW67	O75367	H2AFY	Core histone macro-H2A	Core histone macro-H2A.1	91	2	2.3482	13	3	54
G1SKM2	P35555	FBN1	Uncharacterized protein	Fibrillin-1	97	3	2.3426	6	6	2
G1SNX5	B4DY09	ILF2	Interleukin enhancer binding factor 2	Interleukin enhancer-binding factor 2	100	2	2.3113	17	70	58
G1SI26	Q4VC31	CCDC58	Uncharacterized protein	Coiled-coil domain-containing protein 58	95	3	2.3067	3	7	26
G1TXA3	P82921	MRPS21	Uncharacterized protein	28S ribosomal protein S21, mitochondrial	93	3	2.3002	3	3	38
G1T8H6	K7EK07	H3F3B	Histone H3	Histone H3 (Fragment)	98	2	2.2754	7	23	52
G1T5I0		SLC7A1	Solute carrier family 7 member 1			1	2.2403	4	13	11
G1TU85		FADS3	Cytochrome b5 heme-binding domain-containing protein			1	2.2265	2	2	10
G1ST15	Q13825	AUH	AU RNA binding methylglutaconyl-CoA hydratase	Methylglutaconyl-CoA hydratase, mitochondrial	95	2	2.2247	2	2	7
G1SN06	O60488	ACSL4	AMP-binding domain-containing protein	Long-chain-fatty-acid--CoA ligase 4	98	2	2.2053	11	20	21
	H0Y6E7	RBMX		RNA-binding motif protein, X chromosome (Fragment)		4	2.2053	3	9	12
G1SPY7	O14807	MRAS	Uncharacterized protein	Ras-related protein M-Ras	100	3	2.2053	2	3	16
G1TAH7	P29401	TKT	TRANSKETOLASE_1 domain-containing protein	Transketolase	94	2	2.1869	20	46	49
	Q08945	SSRP1		FACT complex subunit SSRP1		4	2.1565	3	3	9
G1SUS6	D6RD69	SAR1B	Uncharacterized protein	GTP-binding protein SAR1b (Fragment)	98	3	2.1537	4	4	25

G1SDK8	Q13564	NAE1	NEDD8-activating enzyme E1 regulatory subunit	NEDD8-activating enzyme E1 regulatory subunit	96	2	2.1509	3	3	11
G1T3Y0	P98082	DAB2	PID domain-containing protein	Disabled homolog 2	90	2	2.1334	5	7	9
G1TZB9	Q13595	TRA2A	RRM domain-containing protein	Transformer-2 protein homolog alpha	100	2	2.1261	3	2	16
G1SQ07		DTYMK	Thymidylate_kin domain-containing protein			1	2.1224	2	2	10
G1TAU6		SERPINE1	SERPIN domain-containing protein			1	2.1196	2	2	7
G1SF95	H0Y8G5	HNRNPD	Heterogeneous nuclear ribonucleoprotein D	Heterogeneous nuclear ribonucleoprotein D0 (Fragment)	99	2	2.1141	7	16	26
G1TAK1		COA3	Coiled-coil_56 domain-containing protein			1	2.1049	2	5	19
G1SKI8	Q6DKI1	RPL7L1	Uncharacterized protein	60S ribosomal protein L7-like 1	85	3	2.0929	3	4	17
	P35268	RPL22		60S ribosomal protein L22		4	2.0855	2	9	32
G1TJS2	Q8IV08	PLD3	Phospholipase D family member 3	Phospholipase D3	89	2	2.0809	5	2	18
G1TX84	Q9Y5J1	UTP18	WD_REPEATS_REGION domain-containing protein	U3 small nucleolar RNA-associated protein 18 homolog	89	2	2.0708	4	4	12
G1TEI2		FDX1	2Fe-2S ferredoxin-type domain-containing protein			1	2.0671	2	3	20
G1SD44	Q13505	MTX1	Uncharacterized protein	Metaxin-1	84	3	2.0652	6	8	21
G1TE69	J3KTL2	SRSF1	Uncharacterized protein	Serine/arginine-rich-splicing factor 1	100	3	2.0588	6	10	25
G1T017		SLC1A4	Solute carrier family 1 member 4			1	2.0514	3	6	9
G1THR4		GADD45GIP1	GADD45G interacting protein 1			1	2.0431	2	5	13
G1T5A2	P08648	ITGA5	Integrin_alpha2 domain-containing protein	Integrin alpha-5	91	2	2.0394	10	13	13
	A0A2R8Y7C0	HBA2		Hemoglobin subunit alpha (Fragment)		4	2.0394	3	2	23
G1T5A0	P23921	RRM1	Ribonucleoside-diphosphate reductase	Ribonucleoside-diphosphate reductase large subunit	98	2	2.0357	3	3	7
	Q9Y5L4	TIMM13		Mitochondrial import inner membrane translocase subunit Tim13		4	2.0348	3	4	37
G1TD41	G8JLB6	HNRNPH1	Uncharacterized protein	Heterogeneous nuclear ribonucleoprotein H	98	3	2.0247	12	14	39
G1SS51		MRPS25	L51_S25_CI-B8 domain-containing protein			1	2.0228	3	3	22
	P31040	SDHA		Succinate dehydrogenase [ubiquinone] flavoprotein subunit, mitochondrial		4	2.0182	13	7	34
G1TLW3	J3KTA4	DDX5	Uncharacterized protein	Probable ATP-dependent RNA helicase DDX5	96	3	2.0155	21	30	42
G1TQJ5		DEK	SAP domain-containing protein			1	2.0127	2	2	6
G1SIE6	P29279	CCN2	Cellular communication network factor 2	CCN family member 2	90	2	2.0090	11	15	32
	A0A024R4M0	RPS9		40S ribosomal protein S9		4	2.0072	12	19	40
G1TB39	P29353	SHC1	Uncharacterized protein	SHC-transforming protein 1	97	3	2.0072	2	2	6
G1TBV4	Q14566	MCM6	DNA helicase	DNA replication licensing factor MCM6	97	2	1.9970	2	2	4
G1SL60	Q15393	SF3B3	CPSF_A domain-containing protein	Splicing factor 3B subunit 3	98	2	1.9934	17	27	20
G1T8M9	P42704	LRPPRC	PPR_long domain-containing protein	Leucine-rich PPR motif-containing protein, mitochondrial	81	2	1.9786	51	85	47
G1SFQ3		MRPL2	Ribosomal_L2_C domain-containing protein			1	1.9731	3	4	19
	F6RGN5	SLC25A10		Mitochondrial dicarboxylate carrier		4	1.9712	3	6	11
G1SDW8	H0Y2P0	CD44	Link domain-containing protein	CD44 antigen (Fragment)	91	2	1.9675	10	18	17
	K7EKE6	LONP1		Lon protease homolog, mitochondrial		4	1.9657	19	46	28
G1SD25	Q5SRE5	NUP188	Nucleoporin 188	Nucleoporin NUP188 homolog	94	2	1.9546	10	11	9
P12345	P00505	GOT2	Aspartate aminotransferase, mitochondrial	Aspartate aminotransferase, mitochondrial	94	2	1.9528	18	98	46
	Q13247	SRSF6		Serine/arginine-rich splicing factor 6		4	1.9519	3	4	10
G1T4L3	Q01780	EXOSC10	HRDC domain-containing protein	Exosome component 10	89	2	1.9519	2	3	4
G1SIF2	Q16822	PCK2	Uncharacterized protein	Phosphoenolpyruvate carboxykinase [GTP], mitochondrial	95	3	1.9454	23	75	47
G1T7B5	Q9H0S4	DDX47	Uncharacterized protein	Probable ATP-dependent RNA helicase DDX47	97	3	1.9408	2	5	6
G1SP02	A8MT40	PDPR	Uncharacterized protein	Pyruvate dehydrogenase phosphatase regulatory subunit, mitochondrial	95	3	1.9399	4	7	7
	A0A0G2JL54	C4B_2		Complement C4-B		4	1.9353	3	2	1
G1SWU9		FAM20B	Fam20C domain-containing protein			1	1.9335	2	4	7
G1TA04	Q9UMS4	PRPF19	Uncharacterized protein	Pre-mRNA-processing factor 19	96	3	1.9316	5	9	16
	P26368	U2AF2		Splicing factor U2AF 65 kDa subunit		4	1.9288	8	14	29
G1TDQ3	J3QT28	BUB3	WD_REPEATS_REGION domain-containing protein	Mitotic checkpoint protein BUB3 (Fragment)	99	2	1.9261	4	6	15
U3KMH9	P40926	MDH2	Malate dehydrogenase	Malate dehydrogenase, mitochondrial	94	2	1.9086	15	65	60
G1U5D4	F5GY32	PUS1	tRNA pseudouridine synthase	tRNA pseudouridine synthase A (Fragment)	78	2	1.9076	2	3	5
	Q9Y4W6	AFG3L2		AFG3-like protein 2		4	1.9040	11	20	19
G1T2X0	Q99541	PLIN2	Perilipin	Perilipin-2	88	2	1.9030	9	16	28
G1SMH6	Q9P0M6	H2AFY2	Core histone macro-H2A	Core histone macro-H2A.2	99	2	1.9030	6	5	23
G1SSH0	A0A087WUB9	CTNNBL1	DUF1716 domain-containing protein	Beta-catenin-like protein 1	96	2	1.9012	5	7	12
G1SPY4		ASRGL1	Asparaginase like 1			1	1.9003	3	4	11
G1TDQ1	Q9H0C8	ILKAP	PPM-type phosphatase domain-containing protein	Integrin-linked kinase-associated serine/threonine phosphatase 2C	94	2	1.8994	2	3	8
G1SXC8		NXF1	Nuclear RNA export factor 1			1	1.8947	3	3	8
G1SIP2	A0A3B3ITJ4	HNRNPL	Uncharacterized protein	Heterogeneous nuclear ribonucleoprotein L (Fragment)	93	3	1.8929	18	41	53

G1SLK6	J3KMX2	SMARCD2	SWIB domain-containing protein	SWI/SNF-related matrix-associated actin-dependent regulator of chromatin subfamily D member 2	98	2	1.8920	3	2	10
G1T1P3	Q9H845	ACAD9	Uncharacterized protein	Acyl-CoA dehydrogenase family member 9, mitochondrial	88	3	1.8911	9	15	25
G1SS37	A0A494C1M4	ALDH1L2	10-formyltetrahydrofolate dehydrogenase	10-formyltetrahydrofolate dehydrogenase	96	2	1.8874	44	105	66
G1U6B2		ALAD	Delta-aminolevulinic acid dehydratase			1	1.8846	2	2	15
G1U018		IGF2R	Insulin like growth factor 2 receptor			1	1.8837	9	11	4
G1SZ76	P05455	SSB	Lupus La protein homolog	Lupus La protein	92	2	1.8800	16	30	41
	A0A0U1RQF0	FASN		Fatty acid synthase		4	1.8782	8	12	4
G1T7L0		CTSC	Pept_C1 domain-containing protein			1	1.8782	5	5	20
G1SXR7		SLIRP	RRM domain-containing protein			1	1.8782	3	8	34
	F8VV64	TNS2		Tensin-2		4	1.8772	2	3	2
G1SFH4		MRPL24	KOW domain-containing protein			1	1.8754	4	7	32
G1SUQ9	P11387	TOP1	DNA topoisomerase I	DNA topoisomerase 1	97	2	1.8680	7	16	10
G1T5X6		HMGCL	Pyruvate carboxyltransferase domain-containing protein			1	1.8680	2	5	11
G1TRL8	J3QLI9	SNRPD1	Small nuclear ribonucleoprotein Sm D1	Small nuclear ribonucleoprotein Sm D1	100	2	1.8662	3	11	28
G1SY19	O15118	NPC1	SSD domain-containing protein	NPC intracellular cholesterol transporter 1	91	2	1.8616	3	3	3
G1T2K5	I3L1L3	MYBBP1A	Uncharacterized protein	Myb-binding protein 1A (Fragment)	69	3	1.8606	34	92	37
G1SI37	D6RF87	ACSF2	Uncharacterized protein	Acyl-CoA synthetase family member 2, mitochondrial (Fragment)	82	3	1.8597	18	33	51
G1TN86	C9IZG4	CUTA	Uncharacterized protein	Protein CutA	93	3	1.8597	2	2	18
G1SPN1		NUDCD2	CS domain-containing protein			1	1.8588	2	3	17
G1T120	S4R369	MRPL37	Uncharacterized protein	39S ribosomal protein L37, mitochondrial	84	3	1.8570	6	8	19
G1STC6		WDR3	WD_REPEATS_REGION domain-containing protein			1	1.8551	3	3	5
G1T7J5		NCSTN	Ncstrn_small domain-containing protein			1	1.8496	7	17	16
G1SR36	C9JG87	MRPL39	Uncharacterized protein	39S ribosomal protein L39, mitochondrial (Fragment)	86	3	1.8385	5	5	12
G1SE74	Q7KZ85	SUPT6H	Transcription elongation factor spt6	Transcription elongation factor SPT6	99	2	1.8367	5	6	4
G1SLM0	A0A087WTT1	PABPC1	Polyadenylate-binding protein	Polyadenylate-binding protein	99	2	1.8348	18	18	38
G1T157	Q9Y376	CAB39	Uncharacterized protein	Calcium-binding protein 39	99	3	1.8321	3	2	8
G1SW10	O94776	MTA2	Uncharacterized protein	Metastasis-associated protein MTA2	99	3	1.8293	6	5	12
G1TA05	A2AE48	TRIM26	Uncharacterized protein	Tripartite motif-containing protein 26 (Fragment)	92	3	1.8265	2	2	4
	H0YEN5	RPS2		40S ribosomal protein S2 (Fragment)		4	1.8247	10	6	54
G1SU17		NSDHL	3Beta_HSD domain-containing protein			1	1.8247	2	2	10
G1SSJ7	P35232	PHB	PHB domain-containing protein	Prohibitin	100	2	1.8229	17	47	81
G1SMI2	Q9Y305	ACOT9	Acyl-CoA thioesterase 9	Acyl-coenzyme A thioesterase 9, mitochondrial	83	2	1.8210	13	12	33
G1T7H4	Q8TDN6	BRIX1	Brix domain-containing protein	Ribosome biogenesis protein BRX1 homolog	93	2	1.8210	4	6	13
G1T8E0	A3KMH1	VWA8	VWFA domain-containing protein	von Willebrand factor A domain-containing protein 8	92	2	1.8183	7	10	5
G1STZ4	P11413	G6PD	Glucose-6-phosphate 1-dehydrogenase	Glucose-6-phosphate 1-dehydrogenase	94	2	1.8136	5	9	11
G1SHL0		SBNO1	Strawberry notch homolog 1			1	1.8100	2	2	3
G1TBR6	Q69YN4	VIRMA	VIR_N domain-containing protein	Protein virilizer homolog	98	2	1.8054	5	5	4
G1SHU8	D3YTB1	RPL32	Uncharacterized protein	60S ribosomal protein L32 (Fragment)	88	3	1.8044	4	9	29
G1SIW1	O43143	DHX15	Uncharacterized protein	Pre-mRNA-splicing factor ATP-dependent RNA helicase DHX15	99	3	1.8035	14	24	24
G1SMY1	A0A1W2PQ51	DDX17	Uncharacterized protein	Probable ATP-dependent RNA helicase DDX17	99	3	1.7998	20	24	38
G1TI40	P62316	SNRPD2	Small nuclear ribonucleoprotein Sm D2	Small nuclear ribonucleoprotein Sm D2	100	2	1.7943	6	19	48
	P38919	EIF4A3		Eukaryotic initiation factor 4A-III		4	1.7915	13	19	40
G1SFE6	P08579	SNRPB2	Uncharacterized protein	U2 small nuclear ribonucleoprotein B~~	96	3	1.7878	3	2	16
G1SE36	R4GMU1	H6PD	GDH/6PGL endoplasmic bifunctional protein	GDH/6PGL endoplasmic bifunctional protein	85	2	1.7832	8	7	15
G1T4Z1	Q07954	LRP1	Uncharacterized protein	Prolow-density lipoprotein receptor-related protein 1	98	3	1.7786	81	10	26
G1TCZ8	P30153	PPP2R1A	Protein phosphatase 2 scaffold subunit Aalpha	Serine/threonine-protein phosphatase 2A 65 kDa regulatory subunit A alpha isoform	90	2	1.7786	15	3	37
G1TKJ4	P61601	NCALD	Uncharacterized protein	Neurocalcin-delta	100	3	1.7731	6	9	38
	Q9Y2Q5	LAMTOR2		Ragulator complex protein LAMTOR2		4	1.7722	2	3	22
G1SZF7	P48735	IDH2	Isocitrate dehydrogenase [NADP]	Isocitrate dehydrogenase [NADP], mitochondrial	96	2	1.7676	20	124	47
G1TWL0	P22626	HNRNPA2B1	Uncharacterized protein	Heterogeneous nuclear ribonucleoproteins A2/B1	99	3	1.7666	10	25	28
G1SFR8	P25398	RPS12	40S ribosomal protein S12	40S ribosomal protein S12	100	2	1.7657	4	12	32
G1SDV5		NOL9	CLP1_P domain-containing protein			1	1.7611	2	2	4
G1SQR7	P61970	NUTF2	NTF2 domain-containing protein	Nuclear transport factor 2	100	2	1.7574	2	2	17
	Q15428	SF3A2		Splicing factor 3A subunit 2		4	1.7556	2	2	4
G1U1Q1		THBS2	Thrombospondin 2			1	1.7537	5	6	8
G1U9B4	P62995	TRA2B	RRM domain-containing protein	Transformer-2 protein homolog beta	100	2	1.7491	4	7	16
G1T6M1	Q96DA6	DNAJC19	J domain-containing protein	Mitochondrial import inner membrane translocase subunit TIM14	100	2	1.7473	2	3	19

G1SRW4	Q9Y6C2	EMILIN1	Elastin microfibril interfacer 1	EMILIN-1	87	2	1.7464	5	5	6
G1TE50	Q9Y221	NIP7	60S ribosome subunit biogenesis protein NIP7 homolog	60S ribosome subunit biogenesis protein NIP7 homolog	97	2	1.7455	2	2	16
	H0Y2W2	ATAD3A		ATPase family AAA domain-containing protein 3A (Fragment)		4	1.7445	5	10	12
G1SI76		MRPL43	L51_S25_CI-B8 domain-containing protein			1	1.7436	3	3	19
G1T5M0	P04181	OAT	Uncharacterized protein	Ornithine aminotransferase, mitochondrial	91	3	1.7390	16	55	54
G1SQ11	P54886	ALDH18A1	Delta-1-pyrroline-5-carboxylate synthase	Delta-1-pyrroline-5-carboxylate synthase	97	2	1.7325	30	76	53
G1SR61	H7BXY3	DHX30	Uncharacterized protein	ATP-dependent RNA helicase DHX30	98	3	1.7325	7	13	12
	O60518	RANBP6		Ran-binding protein 6		4	1.7316	2	8	3
G1TRH3	Q9UBR2	CTSZ	Pept_C1 domain-containing protein	Cathepsin Z	86	2	1.7307	7	34	30
G1TKL0	M0QZG7	SNRPA	Small nuclear ribonucleoprotein polypeptide A	U1 small nuclear ribonucleoprotein A (Fragment)	60	2	1.7307	4	3	14
G1T9U7	Q8TD30	GPT2	Glutamic--pyruvic transaminase 2	Alanine aminotransferase 2	97	2	1.7261	5	2	21
G1T8P3	P08621	SNRNP70	Small nuclear ribonucleoprotein U1 subunit 70	U1 small nuclear ribonucleoprotein 70 kDa	92	2	1.7261	5	8	11
G1U0Y6		ECI1	Enoyl-CoA delta isomerase 1			1	1.7206	9	21	63
G1U862	Q01650	SLC7A5	Large neutral amino acids transporter small subunit 1	Large neutral amino acids transporter small subunit 1	94	2	1.7187	4	8	14
G1SFH9	A0A2R8Y543	CTNNB1	Uncharacterized protein	Catenin beta-1	100	3	1.7178	25	57	46
G1TGX2		ZCCHC8	Zinc finger CCHC-type containing 8			1	1.7160	2	4	5
G1SLC2	O00303	EIF3F	Eukaryotic translation initiation factor 3 subunit F	Eukaryotic translation initiation factor 3 subunit F	99	2	1.7141	9	25	44
G1SJ32	A0A0A0MTB8	WDR36	WD_REPEATS_REGION domain-containing protein	WD repeat-containing protein 36	94	2	1.7123	2	2	4
G1TC03	Q13263	TRIM28	Tripartite motif containing 28	Transcription intermediary factor 1-beta	96	2	1.7114	16	16	25
G1TCU1	I3L2K5	ZC3H7A	Uncharacterized protein	Zinc finger CCCH domain-containing protein 7A (Fragment)	95	3	1.7114	2	2	2
G1STP6	P35221	CTNNA1	Catenin alpha-1	Catenin alpha-1	99	2	1.7104	31	77	48
G1TTN9	Q9P258	RCC2	Regulator of chromosome condensation 2	Protein RCC2	99	2	1.7104	4	6	13
G1U2E6	J3KPX7	PHB2	PHB domain-containing protein	Prohibitin-2	99	2	1.7086	17	109	61
	Q15366-3	PCBP2		Isoform 3 of Poly(rC)-binding protein 2		4	1.7058	13	3	56
G1SV32	P18124	RPL7	Uncharacterized protein	60S ribosomal protein L7	98	3	1.7049	13	14	46
P35953	P98155	VLDLR	Very low-density lipoprotein receptor	Very low-density lipoprotein receptor	97	2	1.7049	10	14	16
G1SFC6	A0A087WV29	NAT10	RNA cytidine acetyltransferase	RNA cytidine acetyltransferase	96	2	1.7031	6	4	11
G1T9M7	E9PK47	PYGL	Alpha-1,4 glucan phosphorylase	Alpha-1,4 glucan phosphorylase	94	2	1.7021	15	12	25
	A6NG10	WBP2		WW domain-binding protein 2		4	1.7012	2	2	7
G1SGC2	P0C0S5	H2AFZ	Histone H2A	Histone H2A.Z	98	2	1.6975	5	16	54
	Q13045	FLII		Protein flightless-1 homolog		4	1.6948	6	5	6
G1T520	Q99615	DNAJC7	Uncharacterized protein	DnaJ homolog subfamily C member 7	98	3	1.6948	3	3	10
G1SQ38	E5RHK8	DNM3	Uncharacterized protein	Dynamin-3	97	3	1.6938	9	2	12
B7NZN9	P51571	SSR4	Signal sequence receptor, delta (Predicted)	Translocon-associated protein subunit delta	97	2	1.6911	5	10	36
G1SL16	A0A0B4J1Z1	SRSF7	Uncharacterized protein	Serine/arginine-rich-splicing factor 7	100	3	1.6892	4	4	31
G1TEN4	Q9HD33	MRPL47	Uncharacterized protein	39S ribosomal protein L47, mitochondrial	79	3	1.6883	5	7	19
G1SUR4		ZADH2	PKS_ER domain-containing protein			1	1.6883	3	3	18
G1STI3	Q07666	KHDRBS1	KH domain-containing protein	KH domain-containing, RNA-binding, signal transduction-associated protein 1	99	2	1.6874	4	10	12
G1TAE2	Q15717	ELAVL1	ELAV-like protein	ELAV-like protein 1	99	2	1.6837	10	41	32
G1SRH7	P84103	SRSF3	RRM domain-containing protein	Serine/arginine-rich splicing factor 3	100	2	1.6837	4	5	24
G1SZF9	O75976	CPD	Uncharacterized protein	Carboxypeptidase D	91	3	1.6828	5	5	5
G1TB02	E7EPS8	PTPRM	Uncharacterized protein	Receptor-type tyrosine-protein phosphatase mu	98	3	1.6800	3	3	3
G1SJK7		CYP27A1	Sterol 26-hydroxylase, mitochondrial			1	1.6763	2	5	8
G1TIT4		PTTG1IP	PTTG1 interacting protein			1	1.6763	2	3	11
	A0A0C4DFX9	NELFA		Negative elongation factor A		4	1.6754	2	2	7
G1TGI6	A0A0C4DG17	RPSA	40S ribosomal protein SA	40S ribosomal protein SA	98	2	1.6736	10	33	46
G1SEK1	P62140	PPP1CB	Serine/threonine-protein phosphatase	Serine/threonine-protein phosphatase PP1-beta catalytic subunit	100	2	1.6708	9	3	35
G1SSK8	B4DJV2	CS	Citrate synthase	Citrate synthase	96	2	1.6699	18	100	53
G1T920	P49903	SEPHS1	Uncharacterized protein	Selenide, water dikinase 1	100	3	1.6662	5	5	20
P30947	P08238	HSP90AB1	Heat shock protein HSP 90-beta	Heat shock protein HSP 90-beta	99	2	1.6653	34	100	61
	Q9NTZ6	RBM12		RNA-binding protein 12		4	1.6653	2	3	5
G1T890	Q8NE86	MCU	MCU domain-containing protein	Calcium uniporter protein, mitochondrial	98	2	1.6644	10	18	33
G1SUA4	J3KS05	CBX1	Uncharacterized protein	Chromobox protein homolog 1 (Fragment)	98	3	1.6644	4	14	39
G1SZK8	P41223	BUD31	Uncharacterized protein	Protein BUD31 homolog	100	3	1.6588	2	2	19
G1U1X6		NT5C3A	5~-nucleotidase			1	1.6579	2	3	9
G1U7I9	A0A0G2JJZ9	DDX39B	Uncharacterized protein	Spliceosome RNA helicase DDX39B (Fragment)	95	3	1.6570	14	33	46
G1SNS1	Q14692	BMS1	Bms1-type G domain-containing protein	Ribosome biogenesis protein BMS1 homolog	88	2	1.6570	5	5	7

G1TX03	A0A0J9YW13	RBM8A	RNA-binding protein 8A	RNA-binding protein 8A (Fragment)	100	2	1.6570	5	8	49
	Q8N201	INTS1		Integrator complex subunit 1		4	1.6570	4	5	2
G1TMU2	P52597	HNRNPF	Uncharacterized protein	Heterogeneous nuclear ribonucleoprotein F	99	3	1.6515	11	179	44
P62139	P62136	PPP1CA	Serine/threonine-protein phosphatase PP1-alpha catalytic subunit	Serine/threonine-protein phosphatase PP1-alpha catalytic subunit	100	2	1.6515	11	2	51
G1U636	G3V153	CAPRIN1	Uncharacterized protein	Caprin-1	98	3	1.6515	7	25	12
G1SGL3		SRSF11	RRM domain-containing protein			1	1.6505	2	4	5
G1SQA8	P06576	ATP5F1B	ATP synthase subunit beta	ATP synthase subunit beta, mitochondrial	97	2	1.6487	29	463	74
	A0A087WTP3	KHSRP		Far upstream element-binding protein 2		4	1.6487	11	11	17
G1STQ6	Q9Y617	PSAT1	Phosphoserine aminotransferase	Phosphoserine aminotransferase	94	2	1.6478	9	16	22
G1SSF2		ENG	Endoglin			1	1.6432	5	9	12
G1TZN7		COX5A	Cytochrome c oxidase subunit 5A			1	1.6432	2	2	7
G1TTA5	H0Y8C3	MTCH1	Uncharacterized protein	Mitochondrial carrier homolog 1 (Fragment)	93	3	1.6404	7	23	23
G1SHE2		GGCX	HTTM domain-containing protein			1	1.6404	2	3	3
G1SCR7	E9PF10	NUP155	Uncharacterized protein	Nuclear pore complex protein Nup155	90	3	1.6385	14	20	16
G1SZI5	P62269	RPS18	Uncharacterized protein	40S ribosomal protein S18	100	3	1.6376	9	19	44
G1T116	Q96TA2	YME1L1	AAA domain-containing protein	ATP-dependent zinc metalloprotease YME1L1	89	2	1.6349	4	5	9
G1SIM3	O95831	AIFM1	Uncharacterized protein	Apoptosis-inducing factor 1, mitochondrial	96	3	1.6321	8	12	22
G1SRF7	P38646	HSPA9	Uncharacterized protein	Stress-70 protein, mitochondrial	99	3	1.6303	36	411	62
G1T2U6	A0A0C4DG89	DDX46	Uncharacterized protein	Probable ATP-dependent RNA helicase DDX46	99	3	1.6266	6	5	8
	O94973	AP2A2		AP-2 complex subunit alpha-2		4	1.6238	27	37	42
G1SRX2	Q15029	EFTUD2	Tr-type G domain-containing protein	116 kDa U5 small nuclear ribonucleoprotein component	100	2	1.6238	18	39	31
G1T3P4	Q9NRL2	BAZ1A	Bromodomain adjacent to zinc finger domain 1A	Bromodomain adjacent to zinc finger domain protein 1A	89	2	1.6238	3	2	2
G1SJG9	Q6P4Q7	CNNM4	Uncharacterized protein	Metal transporter CNNM4	94	3	1.6229	3	3	5
	P31153	MAT2A		S-adenosylmethionine synthase isoform type-2		4	1.6229	2	2	9
G1SZ00	P21291	CSRP1	Uncharacterized protein	Cysteine and glycine-rich protein 1	98	3	1.6220	9	2	48
	B7WP74	CWC22		Pre-mRNA-splicing factor CWC22 homolog (Fragment)		4	1.6210	3	2	7
G1U4C2	Q76M96	CCDC80	Uncharacterized protein	Coiled-coil domain-containing protein 80	86	3	1.6201	5	6	6
G1SIC7	Q01432	AMPD3	AMP deaminase	AMP deaminase 3	96	2	1.6192	3	3	5
G1SR13	Q9UJZ1	STOML2	PHB domain-containing protein	Stomatin-like protein 2, mitochondrial	96	2	1.6183	15	31	54
G1SKZ8	P62906	RPL10A	Ribosomal protein	60S ribosomal protein L10a	100	2	1.6164	13	44	53
G1T4M2	O60264	SMARCA5	Uncharacterized protein	SWI/SNF-related matrix-associated actin-dependent regulator of chromatin subfamily A member 5	100	3	1.6164	5	10	8
G1SKZ3	P12004	PCNA	Proliferating cell nuclear antigen	Proliferating cell nuclear antigen	99	2	1.6155	2	5	15
G1SJY0		MRPS5	S5 DRBM domain-containing protein			1	1.6146	4	4	12
G1TUB8	Q5VVC8	RPL11	Uncharacterized protein	60S ribosomal protein L11	100	3	1.6127	5	13	34
G1T2N4	E7ET15	U2SURP	Uncharacterized protein	U2 snRNP-associated SURP motif-containing protein	99	3	1.6109	6	11	8
G1TAR0		RPF2	Brix domain-containing protein			1	1.6100	3	4	14
	Q9NTI5-2	PDS5B		Isoform 2 of Sister chromatid cohesion protein PDS5 homolog B		4	1.6063	2	2	2
G1SL42	Q9BPW8	NIPSNAP1	NIPSNAP domain-containing protein	Protein NipSnap homolog 1	95	2	1.6054	5	4	26
	Q8WX92	NELFB		Negative elongation factor B		4	1.6017	3	4	6
G1SST6		ARMC10	Arm_2 domain-containing protein			1	1.5998	2	3	12
G1SKK1		DUT	dUTPase domain-containing protein			1	1.5989	2	5	14
G1SY30	O75643	SNRNP200	Uncharacterized protein	U5 small nuclear ribonucleoprotein 200 kDa helicase	100	3	1.5980	39	61	30
G1T657	A0A087WUK2	HNRNPDL	Heterogeneous nuclear ribonucleoprotein D like	Heterogeneous nuclear ribonucleoprotein D-like	92	2	1.5980	6	16	18
	Q01105-2	SET		Isoform 2 of Protein SET		4	1.5980	6	12	36
G1U3G0	Q9C0C9	UBE2O	Ubiquitin conjugating enzyme E2 O	(E3-independent) E2 ubiquitin-conjugating enzyme	96	2	1.5980	3	3	4
	I3L3B0	C1QBP		Complement component 1 Q subcomponent-binding protein, mitochondrial		4	1.5980	2	22	32
G1SDX3	E9PCY5	TOP2B	DNA topoisomerase 2	DNA topoisomerase 2 (Fragment)	99	2	1.5971	14	21	12
G1T364	Q8NDH3	NPEPL1	Aminopeptidase like 1	Probable aminopeptidase NPEPL1	90	2	1.5962	3	6	9
G1TID3		ITPRIP	Inositol 1,4,5-trisphosphate receptor interacting protein			1	1.5943	2	3	5
G1STF9	Q13347	EIF3I	Eukaryotic translation initiation factor 3 subunit I	Eukaryotic translation initiation factor 3 subunit I	100	2	1.5934	10	10	41
G1TNV7	O75600	GCAT	Glycine C-acetyltransferase	2-amino-3-ketobutyrate coenzyme A ligase, mitochondrial	94	2	1.5934	7	6	27
	P62166	NCS1		Neuronal calcium sensor 1		4	1.5925	2	2	24
	O43752	STX6		Syntaxin-6		4	1.5906	2	2	13
G1TES6	Q99714	HSD17B10	Uncharacterized protein	3-hydroxyacyl-CoA dehydrogenase type-2	92	3	1.5879	13	67	84
G1SHZ2	Q86X83	COMMD2	COMM domain-containing protein	COMM domain-containing protein 2	97	2	1.5879	2	3	17
G1SDD3		KCTD10	BTB domain-containing protein			1	1.5879	2	2	9
G1SKF1	P07996	THBS1	Uncharacterized protein	Thrombospondin-1	97	3	1.5869	34	74	37

G1TAV2		SMCHD1	SMC hinge domain-containing protein			1	1.5860	3	3	2
G1STF8		TBRG4	RAP domain-containing protein			1	1.5860	3	4	7
G1U450		XYLB	Xylulokinase			1	1.5860	2	2	6
	Q5R3B4	MPC2		Mitochondrial pyruvate carrier (Fragment)		4	1.5851	3	4	32
G1TDH4	P30048	PRDX3	Thioredoxin domain-containing protein	Thioredoxin-dependent peroxide reductase, mitochondrial	86	2	1.5842	8	25	36
G1T5G8	Q9HC07	TMEM165	GDT1 family protein	Transmembrane protein 165	91	2	1.5833	2	11	9
G1SMM7	P62318	SNRPD3	Small nuclear ribonucleoprotein Sm D3	Small nuclear ribonucleoprotein Sm D3	100	2	1.5814	3	5	29
G1U2T2	H0YIB4	SRSF9	Uncharacterized protein	Serine/arginine-rich-splicing factor 9 (Fragment)	65	3	1.5814	3	4	15
G1T8B3	Q8IZL8	PELP1	Uncharacterized protein	Proline-, glutamic acid- and leucine-rich protein 1	91	3	1.5786	5	8	6
G1SL97	Q15397	PUM3	PUM-HD domain-containing protein	Pumilio homolog 3	91	2	1.5750	6	7	16
Q28888		DCN	Decorin			1	1.5750	4	3	16
	Q9NRP0	OSTC		Oligosaccharyltransferase complex subunit OSTC		4	1.5750	2	6	13
G1T5A6	Q5T4D3	TMTC4	Transmembrane and tetratricopeptide repeat containing 4	Protein O-mannosyl-transferase TMTC4	92	2	1.5740	5	6	11
G1U4Y5		FCGRT	Fc fragment of IgG receptor and transporter			1	1.5740	2	2	7
G1SNV4	Q12874	SF3A3	Matrin-type domain-containing protein	Splicing factor 3A subunit 3	100	2	1.5722	11	24	33
	Q9BXP5	SRRT		Serrate RNA effector molecule homolog		4	1.5722	7	4	7
G1SJI6		MRPS23	MRP-S23 domain-containing protein			1	1.5713	5	6	34
G1SQR1	Q93096	PTP4A1	TYR_PHOSPHATASE_2 domain-containing protein	Protein tyrosine phosphatase type IVA 1	100	2	1.5713	2	3	17
	O96008	TOMM40		Mitochondrial import receptor subunit TOM40 homolog		4	1.5676	9	15	37
G1TD99	A0A1B0GUX9	PCCA	Uncharacterized protein	Propionyl-CoA carboxylase alpha chain, mitochondrial (Fragment)	96	3	1.5648	8	11	15
G1SIJ7	Q9NR30	DDX21	Uncharacterized protein	Nucleolar RNA helicase 2	89	3	1.5621	16	33	24
G1U9R6	P02751	FN1	Fibronectin	Fibronectin	70	2	1.5602	69	150	46
G1SZE8		UTP20	DRIM domain-containing protein			1	1.5593	7	13	4
G1U3Q0	Q93050	ATP6V0A1	V-type proton ATPase subunit a	V-type proton ATPase 116 kDa subunit a isoform 1	96	2	1.5575	12	3	20
G1SRP7	O00505	KPNA3	Importin subunit alpha	Importin subunit alpha-4	99	2	1.5575	8	11	27
G1TEN9	Q8TB61	SLC35B2	Uncharacterized protein	Adenosine 3~-phospho 5~-phosphosulfate transporter 1	89	3	1.5556	4	7	9
G1TGK9	Q08211	DHX9	Uncharacterized protein	ATP-dependent RNA helicase A	93	3	1.5519	21	39	31
G1SQF9	Q8WXF1	PSPC1	Uncharacterized protein	Paraspeckle component 1	98	3	1.5519	6	9	15
G1TVG7	O75533	SF3B1	SF3b1 domain-containing protein	Splicing factor 3B subunit 1	99	2	1.5492	20	42	27
G1SQW0	E9PIE4	MTCH2	Uncharacterized protein	Mitochondrial carrier homolog 2 (Fragment)	92	3	1.5492	6	16	29
G1ST81	Q9P035	HACD3	Very-long-chain (3R)-3-hydroxyacyl-CoA dehydratase	Very-long-chain (3R)-3-hydroxyacyl-CoA dehydratase 3	96	2	1.5482	4	7	18
G1TMM7	H0YN26	ANP32A	LRRcap domain-containing protein	Acidic leucine-rich nuclear phosphoprotein 32 family member A	93	2	1.5473	7	9	39
G1SM50	Q15427	SF3B4	Uncharacterized protein	Splicing factor 3B subunit 4	100	3	1.5473	4	8	18
G1SUY2	P05091	ALDH2	Aldedh domain-containing protein	Aldehyde dehydrogenase, mitochondrial	91	2	1.5436	12	20	36
G1TC48	A0A0A0MRA5	HNRNPUL1	Uncharacterized protein	Heterogeneous nuclear ribonucleoprotein U-like protein 1	95	3	1.5436	6	8	10
G1SEQ2		PBDC1	Polysacc_synt_4 domain-containing protein			1	1.5436	3	3	16
G1TCT4	O95155	UBE4B	U-box domain-containing protein	Ubiquitin conjugation factor E4 B	95	2	1.5436	2	2	3
G1SDP7	H0Y8P4	UTP15	UTP15, small subunit processome component	U3 small nucleolar RNA-associated protein 15 homolog (Fragment)	92	2	1.5436	2	2	4
G1TTY7	M0R1A7	RPL18A	60S ribosomal protein L18a	60S ribosomal protein L18a	90	2	1.5418	4	4	19
G1SDD0	P30084	ECHS1	Uncharacterized protein	Enoyl-CoA hydratase, mitochondrial	86	3	1.5399	9	16	45
P67828	P48729	CSNK1A1	Casein kinase I isoform alpha	Casein kinase I isoform alpha	100	2	1.5390	4	6	13
G1T9E3	E9PLP8	CSTF3	Suf domain-containing protein	Cleavage stimulation factor subunit 3	100	2	1.5390	3	5	7
G1SKD9	P53597	SUCLG1	Succinate--CoA ligase [ADP/GDP-forming] subunit alpha, mitochondrial	Succinate--CoA ligase [ADP/GDP-forming] subunit alpha, mitochondrial	96	2	1.5372	7	29	25
	Q12931	TRAP1		Heat shock protein 75 kDa, mitochondrial		4	1.5363	7	2	12
G1SRF1	J3KT10	NUP85	Nuclear pore complex protein Nup85	Nuclear pore complex protein Nup85	84	2	1.5353	7	13	16
G1SED9	B0QY89	EIF3L	Eukaryotic translation initiation factor 3 subunit L	Eukaryotic translation initiation factor 3 subunit L	97	2	1.5335	18	30	41
G1SMY6	O14980	XPO1	Importin N-terminal domain-containing protein	Exportin-1	99	2	1.5335	16	24	25
G1TKC4	Q15046	KARS	AA_TRNA_LIGASE_II domain-containing protein	Lysine--tRNA ligase	88	2	1.5326	13	17	32
G1U194	J3KPP4	LUC7L3	Uncharacterized protein	Cisplatin resistance-associated overexpressed protein, isoform CRA_b	98	3	1.5307	3	4	8
G1T2A9	Q14315	FLNC	Uncharacterized protein	Filamin-C	90	3	1.5298	73	100	40
G1SJB4	P63244	RACK1	WD_REPEATS_REGION domain-containing protein	Receptor of activated protein C kinase 1	100	2	1.5289	16	45	73
G1SHH0	Q14139	UBE4A	Ubiquitination factor E4A	Ubiquitin conjugation factor E4 A	98	2	1.5289	3	6	7
G1T8D7	Q8IUX7	AEBP1	F5/8 type C domain-containing protein	Adipocyte enhancer-binding protein 1	76	2	1.5270	5	6	8
G1T2J6	O75746	SLC25A12	Solute carrier family 25 member 12	Calcium-binding mitochondrial carrier protein Aralar1	95	2	1.5261	12	2	34
G1TPN3	D6R9P3	HNRNPAB	Uncharacterized protein	Heterogeneous nuclear ribonucleoprotein A/B	96	3	1.5261	9	80	37
G1TDK8	F8VXC8	SMARCC2	Uncharacterized protein	SWI/SNF complex subunit SMARCC2	93	3	1.5261	8	4	10
G1SEC9	Q9HAV0	GNB4	WD_REPEATS_REGION domain-containing protein	Guanine nucleotide-binding protein subunit beta-4	99	2	1.5215	8	6	28

G1U5U0	Q9Y2X3	NOP58	Nop domain-containing protein	Nucleolar protein 58	92	2	1.5206	11	3	29
G1TME5	Q9H857	NT5DC2	Uncharacterized protein	5~-nucleotidase domain-containing protein 2	86	3	1.5206	4	6	13
	P49750-1	YLPM1		Isoform 1 of YLP motif-containing protein 1		4	1.5206	4	5	4
	O75937	DNAJC8		DnaJ homolog subfamily C member 8		4	1.5206	2	2	7
G1SG61		TPST2	Protein-tyrosine sulfotransferase			1	1.5206	2	2	5
G1SJ30		WDR43	WD_REPEATS_REGION domain-containing protein			1	1.5197	2	2	4
G1SHG0	P62899	RPL31	Uncharacterized protein	60S ribosomal protein L31	100	3	1.5187	6	13	45
G1SGX6		SDR39U1	DUF1731 domain-containing protein			1	1.5160	2	2	14
G1TNM3	P23396	RPS3	KH type-2 domain-containing protein	40S ribosomal protein S3	100	2	1.5132	17	47	62
G1SV12	P16219	ACADS	Uncharacterized protein	Short-chain specific acyl-CoA dehydrogenase, mitochondrial	92	3	1.5132	13	20	51
	B4DQT1	MAEA		Macrophage erythroblast attacher		4	1.5132	3	3	8
G1SQ45	A0A0C4DG98	THOC2	Uncharacterized protein	THO complex subunit 2	98	3	1.5123	4	5	5
G1SP32	Q6NVY1	HIBCH	3-hydroxyisobutyryl-CoA hydrolase, mitochondrial	3-hydroxyisobutyryl-CoA hydrolase, mitochondrial	84	2	1.5105	8	8	25
	P62304	SNRPE		Small nuclear ribonucleoprotein E		4	1.5086	3	13	52
G1SLW8	Q7L2H7	EIF3M	Eukaryotic translation initiation factor 3 subunit M	Eukaryotic translation initiation factor 3 subunit M	100	2	1.5077	12	27	48
G1SME4	Q9BWF3	RBM4	RNA-binding protein 4	RNA-binding protein 4	99	2	1.5068	7	7	20
G1TL80	Q9Y3B4	SF3B6	RRM domain-containing protein	Splicing factor 3B subunit 6	100	2	1.5040	2	6	21
G1SWA6	A0A2R8Y4T4	SMARCE1	HMG box domain-containing protein	SWI/SNF-related matrix-associated actin-dependent regulator of chromatin subfamily E member 1	95	2	1.5031	3	3	9
G1SLR8	Q9NZI8	IGF2BP1	Uncharacterized protein	Insulin-like growth factor 2 mRNA-binding protein 1	99	3	1.5003	3	2	7
	P30533	LRPAP1		Alpha-2-macroglobulin receptor-associated protein		4	1.5003	2	4	5
G1SXW0	P43304	GPD2	Glycerol-3-phosphate dehydrogenase	Glycerol-3-phosphate dehydrogenase, mitochondrial	96	2	1.4985	16	30	34
G1TG15		FAM210A	DUF1279 domain-containing protein			1	1.4985	2	3	13
G1SUP4	J3QRS9	ZNF207	Uncharacterized protein	BUB3-interacting and GLEBS motif-containing protein ZNF207	100	3	1.4975	3	4	8
G1T011	Q9Y5B9	SUPT16H	Uncharacterized protein	FACT complex subunit SPT16	100	3	1.4920	7	10	10
G1STS0	Q9Y2S7	POLDIP2	ApaG domain-containing protein	Polymerase delta-interacting protein 2	98	2	1.4911	6	5	26
G1SLV3	P42285	MTREX	Uncharacterized protein	Exosome RNA helicase MTR4	99	3	1.4902	8	15	11
G1SPF1	Q5JTH9	RRP12	NUC173 domain-containing protein	RRP12-like protein	92	2	1.4902	7	10	8
G1SJV2	Q7L523	RRAGA	Uncharacterized protein	Ras-related GTP-binding protein A	100	3	1.4902	4	8	18
G1SSC9	O95373	IPO7	Importin N-terminal domain-containing protein	Importin-7	100	2	1.4893	18	43	29
G1SFI7	P49915	GMPS	Uncharacterized protein	GMP synthase [glutamine-hydrolyzing]	99	3	1.4893	6	6	10
G1T3R1	Q86SX6	GLRX5	Glutaredoxin 5	Glutaredoxin-related protein 5, mitochondrial	84	2	1.4893	3	4	25
G1TIR7	O95777	LSM8	U6 snRNA-associated Sm-like protein LSm8	U6 snRNA-associated Sm-like protein LSm8	99	2	1.4856	3	32	52
G1T7P9	Q96DI7	SNRNP40	WD_REPEATS_REGION domain-containing protein	U5 small nuclear ribonucleoprotein 40 kDa protein	99	2	1.4828	2	3	13
G1SML5	Q9H9B4	SFXN1	Sidoreflexin	Sideroflexin-1	94	2	1.4810	11	26	53
	Q9P0J1	PDP1		[Pyruvate dehydrogenase [acetyl-transferring]]-phosphatase 1, mitochondrial		4	1.4810	2	3	5
G1TUX2	A2A274	ACO2	Aconitate hydratase, mitochondrial	Aconitate hydratase, mitochondrial	94	2	1.4800	25	56	42
G1TVW1	O60568	PLOD3	Procollagen-lysine,2-oxoglutarate 5-dioxygenase 3	Multifunctional procollagen lysine hydroxylase and glycosyltransferase LH3	94	2	1.4800	13	11	31
G1SEM4		THADA	DUF2428 domain-containing protein			1	1.4800	2	2	1
	Q8NHH9-2	ATL2		Isoform 2 of Atlastin-2		4	1.4782	4	7	11
G1TUD2	Q9UBI6	GNG12	Guanine nucleotide-binding protein subunit gamma	Guanine nucleotide-binding protein G(I)/G(S)/G(O) subunit gamma-12	100	2	1.4782	4	5	67
G1THY5	Q9Y3C6	PPIL1	Peptidyl-prolyl cis-trans isomerase	Peptidyl-prolyl cis-trans isomerase-like 1	100	2	1.4782	2	2	29
G1SM77	P36542	ATP5F1C	ATP synthase subunit gamma	ATP synthase subunit gamma, mitochondrial	94	2	1.4764	11	99	43
G1STR6	Q96FN9	DTD2	Uncharacterized protein	D-aminoacyl-tRNA deacylase 2	93	3	1.4764	2	3	19
G1TFE0	H0YHA7	RPL18	Ribosomal_L18e/L15P domain-containing protein	60S ribosomal protein L18 (Fragment)	90	2	1.4754	3	9	22
	P43897	TSFM		Elongation factor Ts, mitochondrial		4	1.4754	3	4	17
G1TX53		NDUFA8	NADH dehydrogenase [ubiquinone] 1 alpha subcomplex subunit 8			1	1.4754	2	2	9
G1T6I6	Q12906	ILF3	Interleukin enhancer binding factor 3	Interleukin enhancer-binding factor 3	96	2	1.4717	21	34	33
G1SYB9		ITGA3	Integrin subunit alpha 3			1	1.4708	2	5	4
G1T3X2	H7BZW6	SAP18	Histone deacetylase complex subunit SAP18	Histone deacetylase complex subunit SAP18 (Fragment)	98	2	1.4708	2	3	15
G1T2J0	P14923	JUP	Uncharacterized protein	Junction plakoglobin	99	3	1.4699	5	10	10
G1SG11		COX4I1	Cytochrome c oxidase subunit 4 isoform 1, mitochondrial			1	1.4681	3	3	21
G1TCH9	A0A2R8Y473	ABCB7	Uncharacterized protein	ATP-binding cassette sub-family B member 7, mitochondrial	95	3	1.4653	7	13	13
G1SGY8	Q3ZCQ8	TIMM50	Mitochondrial import inner membrane translocase subunit TIM50	Mitochondrial import inner membrane translocase subunit TIM50	96	2	1.4653	6	13	23
G1SPW1	Q29RF7	PDS5A	Uncharacterized protein	Sister chromatid cohesion protein PDS5 homolog A	99	3	1.4644	3	3	3
G1SPL1	A0A286YF22	PHGDH	D-3-phosphoglycerate dehydrogenase	D-3-phosphoglycerate dehydrogenase	93	2	1.4635	26	100	61
G1U448	E9PEB5	FUBP1	Uncharacterized protein	Far upstream element-binding protein 1	93	3	1.4616	10	8	16
G1SVJ8	Q96Q05	TRAPPC9	Uncharacterized protein	Trafficking protein particle complex subunit 9	87	3	1.4616	2	2	2

G1T0I5	Q5JRX3	PITRM1	M16C_associated domain-containing protein	Presequence protease, mitochondrial	89	2	1.4607	27	43	37
G1T2M9	P09486	SPARC	SPARC	SPARC	94	2	1.4607	13	26	45
G1T7V5	E9PEX6	DLD	Dihydrolipoyl dehydrogenase	Dihydrolipoyl dehydrogenase	91	2	1.4598	11	3	32
	Q58FF6	HSP90AB4P		Putative heat shock protein HSP 90-beta 4		4	1.4598	4	9	9
G1T696	Q09161	NCBP1	MIF4G domain-containing protein	Nuclear cap-binding protein subunit 1	99	2	1.4570	9	12	19
	H0Y5K5	ERGIC3		Endoplasmic reticulum-Golgi intermediate compartment protein 3 (Fragment)		4	1.4561	2	2	5
G1SLJ9	Q9P0I2	EMC3	ER membrane protein complex subunit 3	ER membrane protein complex subunit 3	99	2	1.4552	3	3	20
G1SX50		RBM19	RNA binding motif protein 19			1	1.4552	2	2	4
G1SNY0	E9PJD9	RPL27A	Ribosomal_L18e/L15P domain-containing protein	60S ribosomal protein L27a	98	2	1.4542	3	5	22
G1TBL0	E7ETZ4	BZW2	Basic leucine zipper and W2 domains 2	Basic leucine zipper and W2 domain-containing protein 2 (Fragment)	100	2	1.4533	7	9	20
G1T888	Q9P2R3	ANKFY1	Ankyrin repeat and FYVE domain containing 1	Rabankyrin-5	96	2	1.4533	5	2	7
G1SG07	Q8IWT6	LRRC8A	Leucine rich repeat containing 8 VRAC subunit A	Volume-regulated anion channel subunit LRRC8A	98	2	1.4533	3	4	8
G1SIP9	H0Y9G6	MRPL3	Uncharacterized protein	39S ribosomal protein L3, mitochondrial (Fragment)	89	3	1.4515	4	4	21
P80912		HINT1	Histidine triad nucleotide-binding protein 1			1	1.4515	2	2	28
G1TSP3	Q9NZ01	TECR	Uncharacterized protein	Very-long-chain enoyl-CoA reductase	98	3	1.4505	5	12	15
G1SU13	Q5VW52	GPAM	Glycerol-3-phosphate acyltransferase 1, mitochondrial	Glycerol-3-phosphate acyltransferase 1, mitochondrial	94	2	1.4505	3	8	8
G1T9T5	P36957	DLST	Lipoyl-binding domain-containing protein	Dihydrolipoyllysine-residue succinyltransferase component of 2-oxoglutarate dehydrogenase complex, mitochondrial	91	2	1.4496	12	28	35
G1T301	A0A0A0MRN4	ZNF326	Uncharacterized protein	DBIRD complex subunit ZNF326	96	3	1.4487	4	5	10
	P19623	SRM		Spermidine synthase		4	1.4487	3	4	17
U3KMD4	Q8IYU8	MICU2	Uncharacterized protein	Calcium uptake protein 2, mitochondrial	83	3	1.4478	5	3	25
G1SUR8	B7ZBJ4	CAB39L	Uncharacterized protein	Calcium-binding protein 39-like	98	3	1.4469	3	3	12
G1SQB6	Q9NRX1	PNO1	KH domain-containing protein	RNA-binding protein PNO1	96	2	1.4450	2	7	14
G1SKT4	P25705	ATP5F1A	ATP synthase subunit alpha	ATP synthase subunit alpha, mitochondrial	98	2	1.4413	29	386	62
G1SGG6	P48449	LSS	Terpene cyclase/mutase family member	Lanosterol synthase	89	2	1.4413	12	20	20
G1TWS0	B3KY94	CDIPT	CDP-diacylglycerol--inositol 3-phosphatidyltransferase	CDP-diacylglycerol--inositol 3-phosphatidyltransferase	75	2	1.4413	4	6	22
G1SU33		NSUN2	NOP2/Sun RNA methyltransferase family member 2			1	1.4413	3	3	7
G1SKQ8	Q13601	KRR1	KRR1 small subunit processome component	KRR1 small subunit processome component homolog	95	2	1.4413	2	3	7
G1SRS0	D6RBS5	ELMOD2	ELMO domain-containing protein	ELMO domain-containing protein 2 (Fragment)	93	2	1.4404	2	2	6
G1T359	P28331	NDUFS1	Uncharacterized protein	NADH-ubiquinone oxidoreductase 75 kDa subunit, mitochondrial	98	3	1.4395	18	31	37
G1SE65	A0A0C4DG95	PXK	Uncharacterized protein	PX domain-containing protein kinase-like protein	94	3	1.4386	2	2	7
G1SKP2	H0Y8C6	IPO5	Importin N-terminal domain-containing protein	Importin-5 (Fragment)	99	2	1.4376	28	56	42
G1T5H0	A0A3B3IU24	HTRA1	PDZ domain-containing protein	Serine protease HTRA1	94	2	1.4330	2	3	10
G1T0N4	P06748	NPM1	Uncharacterized protein	Nucleophosmin	89	3	1.4303	11	50	38
G1TBN5		TOR1AIP2	Torsin 1A interacting protein 2			1	1.4303	2	4	6
G1SLP3	H3BMM9	RNPS1	RNA binding protein with serine rich domain 1	RNA-binding protein with serine-rich domain 1 (Fragment)	99	2	1.4294	2	3	14
G1T524	P05141	SLC25A5	Uncharacterized protein	ADP/ATP translocase 2	98	3	1.4275	22	20	69
G1SGY1	Q86TB9	PATL1	PAT1 domain-containing protein	Protein PAT1 homolog 1	97	2	1.4247	2	2	4
G1SVD5	P55265	ADAR	Uncharacterized protein	Double-stranded RNA-specific adenosine deaminase	80	3	1.4238	18	29	23
G1SWD1	M0QXL5	FBL	Fibrillarin	rRNA 2~-O-methyltransferase fibrillarin (Fragment)	97	2	1.4220	10	63	66
G1TH59	Q14498	RBM39	Uncharacterized protein	RNA-binding protein 39	91	3	1.4201	7	9	21
G1SZ23	E9PCG9	BDH1	Uncharacterized protein	D-beta-hydroxybutyrate dehydrogenase, mitochondrial	87	3	1.4192	9	14	32
G1TW66		DMAC2	Distal membrane arm assembly complex 2			1	1.4183	2	3	17
G1U0B5	Q9Y570	PPME1	Protein phosphatase methylesterase 1	Protein phosphatase methylesterase 1	96	2	1.4174	3	4	16
G1TSL1	A0A087WZN1	IDH3B	Isocitrate dehydrogenase [NAD] subunit, mitochondrial	Isocitrate dehydrogenase [NAD] subunit, mitochondrial	95	2	1.4155	14	21	42
G1T237	F8VVM2	SLC25A3	Uncharacterized protein	Phosphate carrier protein, mitochondrial	86	3	1.4146	14	110	48
G1SJN5		MAN2A1	Alpha-mannosidase			1	1.4146	8	9	12
G1TM60		NDUFA9	Epimerase domain-containing protein			1	1.4146	6	11	20
G1T9F6		NDUFA6	NADH:ubiquinone oxidoreductase subunit A6			1	1.4128	2	2	19
G1T7W7	P46977	STT3A	Uncharacterized protein	Dolichyl-diphosphooligosaccharide--protein glycosyltransferase subunit STT3A	100	3	1.4118	15	51	26
G1T3S2	P54753	EPHB3	Uncharacterized protein	Ephrin type-B receptor 3	98	3	1.4118	9	17	15
U3KM30	Q969X5	ERGIC1	Uncharacterized protein	Endoplasmic reticulum-Golgi intermediate compartment protein 1	99	3	1.4118	3	4	18
G1SSX2	Q7Z6Z7	HUWE1	HECT, UBA and WWE domain containing 1, E3 ubiquitin protein ligase	E3 ubiquitin-protein ligase HUWE1	96	2	1.4109	19	21	8
G1TMZ2		MRPS7	Ribosomal_S7 domain-containing protein			1	1.4100	5	6	29
G1SYR5		EIF2B2	Translation initiation factor eIF-2B subunit beta			1	1.4100	2	3	8
G1TTN7	O00139	KIF2A	Kinesin-like protein	Kinesin-like protein KIF2A	94	2	1.4072	4	3	6
G1TBL1	C9JPE1	SLC25A20	Uncharacterized protein	Mitochondrial carnitine/acylcarnitine carrier protein	92	3	1.4054	5	15	22
G1SGG2	V9GYM8	ARHGEF2	Uncharacterized protein	Rho guanine nucleotide exchange factor 2	95	3	1.4035	13	21	21

G1T3N1	Q9NVP1	DDX18	RNA helicase	ATP-dependent RNA helicase DDX18	87	2	1.4035	6	9	12
G1SI29	P49411	TUFM	Elongation factor Tu	Elongation factor Tu, mitochondrial	93	2	1.4026	17	94	41
G1SIV7	O43776	NARS	AA_TRNA_LIGASE_II domain-containing protein	Asparagine--tRNA ligase, cytoplasmic	91	2	1.4017	15	36	36
G1SQJ2		FOCAD	DUF3730 domain-containing protein			1	1.4017	8	9	7
G1U2E5	P08243	ASNS	Asparagine synthetase [glutamine-hydrolyzing]	Asparagine synthetase [glutamine-hydrolyzing]	87	2	1.3989	8	8	19
G1SML9	P31689	DNAJA1	Uncharacterized protein	DnaJ homolog subfamily A member 1	100	3	1.3989	8	17	34
G1T8H7	A0A2R8YCL1	GRB10	Growth factor receptor bound protein 10	Growth factor receptor-bound protein 10	90	2	1.3989	2	2	4
G1TAI0		KPNA2	Importin subunit alpha			1	1.3980	3	6	11
G1SWK4	B4E1G1	DERL1	Derlin	Derlin	99	2	1.3971	2	3	10
G1TTM6	Q99797	MIPEP	Peptidase_M3 domain-containing protein	Mitochondrial intermediate peptidase	93	2	1.3962	6	5	15
G1SZZ2	P49458	SRP9	Signal recognition particle 9 kDa protein	Signal recognition particle 9 kDa protein	92	2	1.3962	3	4	30
G1TEI1	P20645	M6PR	Uncharacterized protein	Cation-dependent mannose-6-phosphate receptor	95	3	1.3953	2	3	8
G1SMX7	O43731	KDELR3	ER lumen protein-retaining receptor	ER lumen protein-retaining receptor 3	98	2	1.3943	3	2	17
G1SHV1	Q9Y333	LSM2	U6 snRNA-associated Sm-like protein LSm2	U6 snRNA-associated Sm-like protein LSm2	100	2	1.3934	2	4	27
G1T5H2	Q9H0D6	XRN2	5~-3~ exoribonuclease	5~-3~ exoribonuclease 2	98	2	1.3916	8	10	14
G1T2Y5	P12270	TPR	TPR_MLP1_2 domain-containing protein	Nucleoprotein TPR	97	2	1.3906	26	37	14
G1SUF4	O15269	SPTLC1	Aminotran_1_2 domain-containing protein	Serine palmitoyltransferase 1	95	2	1.3879	6	12	15
G1SN16	P27695	APEX1	DNA-(apurinic or apyrimidinic site) lyase	DNA-(apurinic or apyrimidinic site) lyase	95	2	1.3879	5	13	28
G1SQK0	A0A087X1A5	STAU1	Uncharacterized protein	Double-stranded RNA-binding protein Staufen homolog 1	89	3	1.3870	2	2	4
G1THH7		SUN2	SUN domain-containing protein			1	1.3860	7	11	16
G1STX9		COQ8B	Coenzyme Q8B			1	1.3860	3	4	15
G1SIQ9	Q9GZL7	WDR12	Ribosome biogenesis protein WDR12	Ribosome biogenesis protein WDR12	97	2	1.3860	3	2	15
G1TEZ1	G3V198	NUP160	Uncharacterized protein	Nuclear pore complex protein Nup160 (Fragment)	93	3	1.3842	14	23	18
G1SPT2	P26196	DDX6	Uncharacterized protein	Probable ATP-dependent RNA helicase DDX6	99	3	1.3833	13	21	39
G1SZ44	B8ZZL8	HSPE1	Uncharacterized protein	10 kDa heat shock protein, mitochondrial	100	3	1.3833	7	7	58
G1SXI9		COX6B1	Cytochrome c oxidase subunit			1	1.3833	2	3	29
	P62424	RPL7A		60S ribosomal protein L7a		4	1.3814	12	35	45
G1T3L2	K7ERF1	EIF3K	Eukaryotic translation initiation factor 3 subunit K	Eukaryotic translation initiation factor 3 subunit K	87	2	1.3814	7	13	44
G1TG28	P80723	BASP1	Brain abundant membrane attached signal protein 1	Brain acid soluble protein 1	61	2	1.3796	5	6	51
G1SF56		MICAL2	Microtubule associated monooxygenase, calponin and LIM domain containing 2			1	1.3796	3	4	6
G1SVT4	P51665	PSMD7	MPN domain-containing protein	26S proteasome non-ATPase regulatory subunit 7	99	2	1.3787	9	20	38
G1SMI7	V9GYL9	DAP3	Uncharacterized protein	28S ribosomal protein S29, mitochondrial (Fragment)	86	3	1.3777	8	9	28
G1SMR7	P30050	RPL12	Uncharacterized protein	60S ribosomal protein L12	100	3	1.3777	8	22	53
G1SPK4	P05388	RPLP0	60S acidic ribosomal protein P0	60S acidic ribosomal protein P0	98	2	1.3759	13	9	68
G1SD24	A0A494BZU6	PARN	R3H domain-containing protein	Poly(A)-specific ribonuclease PARN (Fragment)	96	2	1.3759	2	2	5
G1SHF3		NIT1	CN hydrolase domain-containing protein			1	1.3750	3	4	14
G1SGH2		MRPL15	Ribosomal_L18e/L15P domain-containing protein			1	1.3750	2	2	9
G1SUC8	P60228	EIF3E	Eukaryotic translation initiation factor 3 subunit E	Eukaryotic translation initiation factor 3 subunit E	100	2	1.3741	14	26	41
G1T1D9	Q92905	COPS5	MPN domain-containing protein	COP9 signalosome complex subunit 5	100	2	1.3741	7	12	31
	Q10570	CPSF1		Cleavage and polyadenylation specificity factor subunit 1		4	1.3741	2	2	2
G1SD91	E9PFD2	UMPS	OMPdecase domain-containing protein	Uridine 5~-monophosphate synthase	92	2	1.3741	2	2	6
G1SZZ1	Q9H0V1	TMEM168	Transmembrane protein 168	Transmembrane protein 168	96	2	1.3731	5	6	13
G1T8F7	A0A494C128	NOP56	Nop domain-containing protein	Nucleolar protein 56	98	2	1.3722	16	22	39
G1SYT7	G3V0E4	PMPCB	Uncharacterized protein	Mitochondrial-processing peptidase subunit beta	94	3	1.3722	10	19	34
G1SY93	P63000	RAC1	Rac family small GTPase 1	Ras-related C3 botulinum toxin substrate 1	90	2	1.3722	6	10	31
G1SE49	Q9UID3	VPS51	Uncharacterized protein	Vacuolar protein sorting-associated protein 51 homolog	96	3	1.3722	6	7	15
G1TAN9	P21399	ACO1	Cytoplasmic aconitate hydratase	Cytoplasmic aconitate hydratase	93	2	1.3704	5	7	9
	P18583-2	SON		Isoform A of Protein SON		4	1.3676	2	2	1
G1TJW3	Q15233	NONO	Uncharacterized protein	Non-POU domain-containing octamer-binding protein	99	3	1.3648	12	44	33
G1SGJ5	Q92600	CNOT9	Uncharacterized protein	CCR4-NOT transcription complex subunit 9	97	3	1.3630	2	3	7
G1TQD4		RALY	RRM domain-containing protein			1	1.3630	2	2	6
G1TTL1	A0A0B4J1W3	NAA15	Uncharacterized protein	N-alpha-acetyltransferase 15, NatA auxiliary subunit	99	3	1.3612	7	10	13
G1STH4	Q14344	GNA13	Uncharacterized protein	Guanine nucleotide-binding protein subunit alpha-13	86	3	1.3612	3	5	11
G1SQG1	H0YK61	EMC4	ER membrane protein complex subunit 4	ER membrane protein complex subunit 4	97	2	1.3602	2	3	32
G1TBC0	A0A499FI31	SART3	Uncharacterized protein	Squamous cell carcinoma antigen recognized by T-cells 3	89	3	1.3602	2	3	4
G1U7L4	P11021	HSPA5	Heat shock protein family A (Hsp70) member 5	Endoplasmic reticulum chaperone BiP	99	2	1.3593	34	761	62
G1T3H5		EIF2B3	NTP_transferase domain-containing protein			1	1.3593	4	4	12

G1T5L3	Q9H9J2	MRPL44	Uncharacterized protein	39S ribosomal protein L44, mitochondrial	90	3	1.3575	3	4	15
G1TYH7	Q9HDC9	APMAP	Adipocyte plasma membrane associated protein	Adipocyte plasma membrane-associated protein	94	2	1.3565	12	29	34
G1TDA9	O94766	B3GAT3	Galactosylgalactosylxylosylprotein 3-beta-glucuronosyltransferase	Galactosylgalactosylxylosylprotein 3-beta-glucuronosyltransferase 3	96	2	1.3565	4	4	12
G1SCW9		CHPF2	Hexosyltransferase			1	1.3565	4	4	10
G1SWF3		DHODH	Dihydroorotate dehydrogenase (quinone), mitochondrial			1	1.3565	4	4	15
	Q03252	LMNB2		Lamin-B2		4	1.3556	8	6	11
G1TDX2	A0A3B3ISY9	AGK	Acylglycerol kinase	Acylglycerol kinase, mitochondrial	94	2	1.3556	5	6	32
G1SYC5	Q9NQZ2	UTP3	Sas10 domain-containing protein	Something about silencing protein 10	82	2	1.3547	3	4	8
G1SSN2		SIRT5	NAD-dependent protein deacylase sirtuin-5, mitochondrial			1	1.3547	2	2	10
P67873	Q5SRQ6	CSNK2B	Casein kinase II subunit beta	Casein kinase II subunit beta	100	2	1.3529	7	10	51
G1T5I9	F8WJN3	CPSF6	RRM domain-containing protein	Cleavage and polyadenylation-specificity factor subunit 6	99	2	1.3519	3	4	9
G1SE30		EPS8	SH3 domain-containing protein			1	1.3519	2	4	5
G1THL2		FTL	Ferritin			1	1.3519	2	2	17
G1TAB7	A0A2R8Y3X5	OPA1	Dynamin-type G domain-containing protein	Dynamin-like 120 kDa protein, mitochondrial	94	2	1.3510	26	51	32
G1SGP1	P31930	UQCRC1	Uncharacterized protein	Cytochrome b-c1 complex subunit 1, mitochondrial	93	3	1.3510	12	23	42
G1TD91	O43809	NUDT21	Nudix hydrolase domain-containing protein	Cleavage and polyadenylation specificity factor subunit 5	100	2	1.3510	4	9	30
G1SJ66	C9JME2	FARP1	Uncharacterized protein	FERM, ARHGEF and pleckstrin domain-containing protein 1	90	3	1.3501	28	61	38
	K7EIE8	MBD3		Methyl-CpG binding domain protein 3, isoform CRA_b		4	1.3501	2	2	14
G1U150	M0QXU7	TIMM44	Mitochondrial import inner membrane translocase subunit TIM44	Mitochondrial import inner membrane translocase subunit TIM44 (Fragment)	83	2	1.3492	7	13	18
G1SZA1	B4DHE8	MSI2	Uncharacterized protein	RNA-binding protein Musashi homolog 2	94	3	1.3492	4	5	18
G1T4Z2	P53396	ACLY	ATP-citrate synthase	ATP-citrate synthase	98	2	1.3483	17	31	23
G1T6J2		APOO	MICOS complex subunit			1	1.3483	5	8	46
G1TBC1	P14625	HSP90B1	Endoplasmin	Endoplasmin	94	2	1.3473	43	921	55
G1SYC1	P83111	LACTB	Uncharacterized protein	Serine beta-lactamase-like protein LACTB, mitochondrial	89	3	1.3455	4	8	12
G1TE34	P06756	ITGAV	Integrin_alpha2 domain-containing protein	Integrin alpha-V	95	2	1.3446	26	92	34
G1SWI3	P45880	VDAC2	Voltage-dependent anion-selective channel protein 2	Voltage-dependent anion-selective channel protein 2	99	2	1.3446	13	59	63
G1TCX6	P08240	SRPRA	SRP54 domain-containing protein	Signal recognition particle receptor subunit alpha	99	2	1.3446	11	13	25
G1SXZ9	Q86WA6	BPHL	Biphenyl hydrolase like	Valacyclovir hydrolase	89	2	1.3446	7	9	25
	E7EVJ3	NDST1		Bifunctional heparan sulfate N-deacetylase/N-sulfotransferase 1		4	1.3436	2	2	3
G1SK22	P62979	RPS27A	Ubiquitin-like domain-containing protein	Ubiquitin-40S ribosomal protein S27a	100	2	1.3427	8	44	47
G1T8T0		FRMD6	FERM domain-containing protein			1	1.3427	2	2	4
G1SGB5	P23246	SFPQ	Splicing factor proline and glutamine rich	Splicing factor, proline- and glutamine-rich	100	2	1.3418	13	26	21
G1SM91		FAH	Fumarylacetoacetase			1	1.3418	2	2	7
G1SFV7	Q16531	DDB1	Damage specific DNA binding protein 1	DNA damage-binding protein 1	100	2	1.3409	18	25	19
G1SWD9	B4DKY1	CARS	Cysteinyl-tRNA synthetase	Cysteine--tRNA ligase, cytoplasmic	68	2	1.3409	12	23	20
G1TIP5	C9JCC6	DRAP1	CBFD_NFYB_HMF domain-containing protein	Dr1-associated corepressor	87	2	1.3400	2	3	11
U3KPI1		MPHOSPH10	U3 small nucleolar ribonucleoprotein protein MPP10			1	1.3400	2	4	4
G1T2V2	D6RF62	PAICS	AIRC domain-containing protein	Multifunctional protein ADE2	93	2	1.3381	8	8	32
G1SUU2	A0A087WXS7	ASNA1	ATPase ASNA1	ATPase ASNA1	93	2	1.3372	9	12	44
	Q9NRZ7	AGPAT3		1-acyl-sn-glycerol-3-phosphate acyltransferase gamma		4	1.3372	2	3	4
G1SXN0		PES1	Pescadillo homolog			1	1.3363	2	4	6
G1SCK0	Q6P2Q9	PRPF8	MPN domain-containing protein	Pre-mRNA-processing-splicing factor 8	100	2	1.3344	35	52	22
G1SCN8	P49368	CCT3	T-complex protein 1 subunit gamma	T-complex protein 1 subunit gamma	98	2	1.3326	27	50	64
P62497	P62495	ETF1	Eukaryotic peptide chain release factor subunit 1	Eukaryotic peptide chain release factor subunit 1	100	2	1.3326	15	35	52
G1SMC8	Q14997	PSME4	Uncharacterized protein	Proteasome activator complex subunit 4	98	3	1.3326	5	4	5
G1TPZ3	Q96GK7	FAHD2A	FAA_hydrolase domain-containing protein	Fumarylacetoacetate hydrolase domain-containing protein 2A	90	2	1.3326	3	3	12
G1U516	E9PHV4	POLR2D	RPOL4c domain-containing protein	DNA-directed RNA polymerase II subunit RPB4	76	2	1.3326	2	6	25
G1TZQ6		NDUFA10	NADH dehydrogenase [ubiquinone] 1 alpha subcomplex subunit 10, mitochondrial			1	1.3317	4	7	19
G1T568	Q92973	TNPO1	Transportin 1	Transportin-1	93	2	1.3307	14	11	21
	A0A087WX29	TARDBP		TAR DNA-binding protein 43 (Fragment)		4	1.3307	7	6	47
G1T9N2	O75947	ATP5PD	ATP synthase subunit d, mitochondrial	ATP synthase subunit d, mitochondrial	91	2	1.3298	10	23	70
G1SDL0	Q96DX4	RSPRY1	Uncharacterized protein	RING finger and SPRY domain-containing protein 1	93	3	1.3261	4	6	16
G1SEC8	Q8IWA4	MFN1	Dynamin-type G domain-containing protein	Mitofusin-1	92	2	1.3252	8	14	17
G1T701		DBT	Dihydrolipoamide acetyltransferase component of pyruvate dehydrogenase complex			1	1.3252	2	3	5
	M0R261	PGLS		6-phosphogluconolactonase (Fragment)		4	1.3252	2	4	13
P20647	P16615	ATP2A2	Sarcoplasmic/endoplasmic reticulum calcium ATPase 2	Sarcoplasmic/endoplasmic reticulum calcium ATPase 2	98	2	1.3243	29	5	36
G1SFD8	Q9BUQ8	DDX23	Uncharacterized protein	Probable ATP-dependent RNA helicase DDX23	99	3	1.3243	7	7	11

G1SZ37	P31937	HIBADH	3-hydroxyisobutyrate dehydrogenase	3-hydroxyisobutyrate dehydrogenase, mitochondrial	96	2	1.3243	6	10	32
G1SG80	F8VZG5	AK2	Adenylate kinase 2, mitochondrial	Adenylate kinase 2, mitochondrial	92	2	1.3234	9	25	47
G1SGY0	O00267	SUPT5H	Transcription elongation factor SPT5	Transcription elongation factor SPT5	98	2	1.3234	8	8	12
G1SQH0	P61254	RPL26	KOW domain-containing protein	60S ribosomal protein L26	100	2	1.3234	6	10	28
G1T0U4	Q9UIG0	BAZ1B	Bromodomain adjacent to zinc finger domain 1B	Tyrosine-protein kinase BAZ1B	94	2	1.3234	4	3	4
G1T7I3	Q13085	ACACA	Uncharacterized protein	Acetyl-CoA carboxylase 1	98	3	1.3225	12	13	7
G1T647		GCLM	Glutamate-cysteine ligase modifier subunit			1	1.3225	3	4	16
G1T6L0	Q5T3Q7	HEATR1	BP28CT domain-containing protein	HEAT repeat-containing protein 1	93	2	1.3215	11	15	10
	Q9H0B6	KLC2		Kinesin light chain 2		4	1.3215	5	2	12
G1T958		EBP	EXPERA domain-containing protein			1	1.3215	2	3	8
G1T2F2	P23284	PPIB	Peptidyl-prolyl cis-trans isomerase	Peptidyl-prolyl cis-trans isomerase B	94	2	1.3206	7	11	31
G1TA40	H0YEP5	SMPD1	Sphingomyelin phosphodiesterase	Sphingomyelin phosphodiesterase (Fragment)	78	2	1.3206	5	8	14
	K7ER00	FARSA		Phenylalanine--tRNA ligase alpha subunit		4	1.3206	3	2	6
G1TQ57	H3BP71	RNF40	E3 ubiquitin protein ligase	E3 ubiquitin protein ligase	92	2	1.3206	3	3	6
G1SEK2		PPM1F	PPM-type phosphatase domain-containing protein			1	1.3206	2	3	11
G1T9W3	K4DI93	CUL4B	CULLIN_2 domain-containing protein	Cullin 4B, isoform CRA_e	100	2	1.3197	10	9	14
	M0QYZ2	AP2S1		AP complex subunit sigma		4	1.3197	5	12	33
	A0A1B0GV23	CTSD		Cathepsin D		4	1.3188	6	21	14
G1SLI8	A0A0A0MSJ0	DDX42	Uncharacterized protein	ATP-dependent RNA helicase DDX42	96	3	1.3188	3	7	7
G1SH80	O14874	BCKDK	Protein-serine/threonine kinase	[3-methyl-2-oxobutanoate dehydrogenase [lipoamide]] kinase, mitochondrial	97	2	1.3188	2	9	6
G1TUU9		TRIP12	Thyroid hormone receptor interactor 12			1	1.3178	8	10	6
G1SNE9	A0A0G2JQ41	ABR	Uncharacterized protein	Active breakpoint cluster region-related protein (Fragment)	100	3	1.3178	7	5	18
G1SXK8	F8WE74	SLC25A17	Uncharacterized protein	Peroxisomal membrane protein PMP34	96	3	1.3178	4	6	21
O18757	Q6NUK1	SLC25A24	Calcium-binding mitochondrial carrier protein SCaMC-1	Calcium-binding mitochondrial carrier protein SCaMC-1	95	2	1.3169	14	5	43
	A0A0J9YVP6	PUF60		Poly(U)-binding-splicing factor PUF60 (Fragment)		4	1.3160	8	14	23
G1T9R8	Q16222	UAP1	Uncharacterized protein	UDP-N-acetylhexosamine pyrophosphorylase	96	3	1.3160	5	6	14
G1SF78	Q96RL7	VPS13A	Vacuolar protein sorting 13 homolog A	Vacuolar protein sorting-associated protein 13A	89	2	1.3160	4	5	3
G1SEW1	R4GMQ1	KDM1A	Lysine-specific histone demethylase	Lysine-specific histone demethylase	96	2	1.3160	3	8	7
G1T9V1	P11177	PDHB	Pyruvate dehydrogenase E1 component subunit beta	Pyruvate dehydrogenase E1 component subunit beta, mitochondrial	97	2	1.3142	10	31	39
G1SS22		XRCC6	Ku domain-containing protein			1	1.3142	2	3	5
G1U971		EIF3C	Eukaryotic translation initiation factor 3 subunit C			1	1.3114	16	28	24
	P0DN76	U2AF1L5		Splicing factor U2AF 35 kDa subunit-like protein		4	1.3114	3	6	23
G1SH10	H3BVG0	NUP93	Nuclear pore complex protein Nup93	Nuclear pore complex protein Nup93	99	2	1.3105	14	20	24
	Q12873	CHD3		Chromodomain-helicase-DNA-binding protein 3		4	1.3105	7	2	6
G1SRY1	Q8TCS8	PNPT1	S1 motif domain-containing protein	Polyribonucleotide nucleotidyltransferase 1, mitochondrial	94	2	1.3105	7	14	14
G1SF97		MRPL46	MRP-L46 domain-containing protein			1	1.3105	4	11	18
G1TKC9	Q15582	TGFBI	Transforming growth factor-beta-induced protein ig-h3	Transforming growth factor-beta-induced protein ig-h3	93	2	1.3086	5	6	16
G1SR19	P20594	NPR2	Guanylate cyclase	Atrial natriuretic peptide receptor 2	95	2	1.3059	7	10	10
G1SES8	G5E9V5	MRPS22	Uncharacterized protein	28S ribosomal protein S22, mitochondrial	84	3	1.3059	2	2	6
	O95202	LETM1		Mitochondrial proton/calcium exchanger protein		4	1.3040	7	13	10
G1SMZ5	Q14152	EIF3A	Eukaryotic translation initiation factor 3 subunit A	Eukaryotic translation initiation factor 3 subunit A	93	2	1.3031	31	60	27
G1TRS0		DKC1	PUA domain-containing protein			1	1.3022	5	7	14
G1TUP1	O75340	PDCD6	Programmed cell death 6	Programmed cell death protein 6	99	2	1.3022	5	14	29
G1SHS8	A0A087WY55	VTA1	Uncharacterized protein	Chromosome 6 open reading frame 55, isoform CRA_b	87	3	1.3022	3	5	15
B7NZG9	Q8N3U4	STAG2	Stromal antigen 2 isoform a (Predicted)	Cohesin subunit SA-2	97	2	1.3013	5	7	8
G1SHL9	Q15006	EMC2	TPR_REGION domain-containing protein	ER membrane protein complex subunit 2	99	2	1.3003	6	14	34
G1SQ57	Q8N6T3	ARFGAP1	Arf-GAP domain-containing protein	ADP-ribosylation factor GTPase-activating protein 1	78	2	1.3003	5	5	21
G1SDJ7	B1ANR0	PABPC4	Polyadenylate-binding protein	Polyadenylate-binding protein	92	2	1.2994	17	12	27
G1TT27	E9PKZ0	RPL8	Ribosomal_L2_C domain-containing protein	60S ribosomal protein L8 (Fragment)	100	2	1.2994	6	12	35
	G3V1C3	API5		Apoptosis inhibitor 5		4	1.2985	8	18	27
G1SCY8	Q9UKD2	MRTO4	Ribosome assembly factor mrt4	mRNA turnover protein 4 homolog	96	2	1.2985	2	3	10
G1STH0	Q15459	SF3A1	Uncharacterized protein	Splicing factor 3A subunit 1	98	3	1.2966	8	13	13
B7NZJ1		CPNE1	Copine I, isoform 8 (Predicted)			1	1.2966	3	4	11
G1TEB0	Q9UKX5	ITGA11	VWFA domain-containing protein	Integrin alpha-11	91	2	1.2957	20	42	24
G1SI71		PAIP1	MIF4G domain-containing protein			1	1.2957	2	3	6
G1SKW4		KIDINS220	Kinase D interacting substrate 220			1	1.2930	8	8	7
G1SDN3	K7EM18	EIF1	SUI1 domain-containing protein	Eukaryotic translation initiation factor 1	100	2	1.2920	5	7	44

G1SSL5	Q14CX7	NAA25	TPR_REGION domain-containing protein	N-alpha-acetyltransferase 25, NatB auxiliary subunit	96	2	1.2920	5	6	11
G1T3V3	Q6R327	RICTOR	Uncharacterized protein	Rapamycin-insensitive companion of mTOR	98	3	1.2911	4	6	4
G1SQ54	A0A087WT44	HMOX2	Heme oxygenase	Heme oxygenase 2	89	2	1.2893	7	9	33
G1TX78	Q15388	TOMM20	Uncharacterized protein	Mitochondrial import receptor subunit TOM20 homolog	100	3	1.2865	3	27	28
G1T2G3	Q5VTR2	RNF20	E3 ubiquitin protein ligase	E3 ubiquitin-protein ligase BRE1A	98	2	1.2847	6	4	10
P0CL18		EIF2D	Eukaryotic translation initiation factor 2D			1	1.2837	2	3	8
G1SIB2	A0A087WVM4	MTHFD1L	Uncharacterized protein	Monofunctional C1-tetrahydrofolate synthase, mitochondrial	92	3	1.2828	9	22	13
G1SLI0	Q96A33	CCDC47	Uncharacterized protein	Coiled-coil domain-containing protein 47	98	3	1.2819	8	12	22
G1TDB3	P62851	RPS25	Uncharacterized protein	40S ribosomal protein S25	100	3	1.2819	3	5	16
G1SF26	P53675	CLTCL1	Clathrin heavy chain	Clathrin heavy chain 2	91	2	1.2810	17	4	13
G1TBS2	Q9Y265	RUVBL1	RuvB-like helicase	RuvB-like 1	100	2	1.2810	14	26	47
G1TLE4	P62879	GNB2	WD_REPEATS_REGION domain-containing protein	Guanine nucleotide-binding protein G(I)/G(S)/G(T) subunit beta-2	100	2	1.2801	11	6	43
G1TVK1		NOTCH2	Notch 2			1	1.2801	4	5	3
G1T1L7	A0A087WWS1	THOC1	Death domain-containing protein	THO complex subunit 1	98	2	1.2791	3	3	10
P30946	P07900	HSP90AA1	Heat shock protein HSP 90-alpha	Heat shock protein HSP 90-alpha	94	2	1.2773	36	271	63
O77622		CCT6	T-complex protein 1 subunit zeta			1	1.2773	23	15	54
G1SI85	A0A0A0MS41	SFXN3	Uncharacterized protein	Sidoreflexin	94	3	1.2773	13	19	39
Q9GKX2		DHRS4	Dehydrogenase/reductase SDR family member 4 (Fragment)			1	1.2773	8	14	38
G1SW11		CERCAM	Cerebral endothelial cell adhesion molecule			1	1.2773	6	9	16
G1TIB4	P62857	RPS28	Ribosomal protein S28	40S ribosomal protein S28	100	2	1.2773	3	7	46
G1T994	D6RGG3	COL12A1	Collagen alpha-1(XII) chain	Collagen alpha-1(XII) chain	95	2	1.2764	117	679	55
G1SCT6	E9PB90	HK2	Uncharacterized protein	Hexokinase-2	96	3	1.2764	10	3	16
A0A0A0MQQ6	D6RBW1	EIF4E	Eukaryotic translation initiation factor 4E	Eukaryotic translation initiation factor 4E	98	2	1.2764	3	5	20
G1T312		ACAD8	Acyl-CoA dehydrogenase family member 8			1	1.2764	2	2	5
	P52815	MRPL12		39S ribosomal protein L12, mitochondrial		4	1.2764	2	3	13
G1SQZ4	Q9Y230	RUVBL2	RuvB-like helicase	RuvB-like 2	99	2	1.2736	15	39	49
G1STU7	Q5QNZ2	ATP5PB	Uncharacterized protein	ATP synthase F(0) complex subunit B1, mitochondrial	84	3	1.2727	12	47	24
	P54725	RAD23A		UV excision repair protein RAD23 homolog A		4	1.2727	5	13	23
	P60468	SEC61B		Protein transport protein Sec61 subunit beta		4	1.2727	3	5	52
G1SVQ8	F8WF48	SEC62	Uncharacterized protein	Translocation protein SEC62	100	3	1.2727	2	3	5
Q9N0Z6	P05023	ATP1A1	Sodium/potassium-transporting ATPase subunit alpha-1	Sodium/potassium-transporting ATPase subunit alpha-1	98	2	1.2708	28	70	36
G1TVY5	C9JZR2	CTNND1	Uncharacterized protein	Catenin delta-1	97	3	1.2708	19	46	35
G1TSS7	P05106	ITGB3	Integrin beta	Integrin beta-3	95	2	1.2708	5	5	9
G1T0U8	Q9UHG3	PCYOX1	Prenylcys_lyase domain-containing protein	Prenylcysteine oxidase 1	86	2	1.2699	10	28	31
G1SVH1	A0A0U1RRK1	MICU1	Uncharacterized protein	Calcium uptake protein 1, mitochondrial	94	3	1.2690	5	17	18
Q28618	P67809	YBX1	Nuclease-sensitive element-binding protein 1	Nuclease-sensitive element-binding protein 1	99	2	1.2690	4	93	22
	E9PM12	TCIRG1		V-type proton ATPase subunit a (Fragment)		4	1.2690	2	2	9
G1TQ79		CHCHD6	MICOS complex subunit			1	1.2681	3	3	15
G1SYD3	P54577	YARS	Tyrosine--tRNA ligase	Tyrosine--tRNA ligase, cytoplasmic	96	2	1.2672	20	34	44
G1SHI9	A0A0D9SFS3	OGDH	Transket_pyr domain-containing protein	2-oxoglutarate dehydrogenase, mitochondrial	95	2	1.2662	29	65	40
G1U383	A0A087WYN9	DHX29	ATP-dependent RNA helicase DHX29	ATP-dependent RNA helicase DHX29	95	2	1.2662	7	6	6
G1T0M2		TRMT10C	SAM-dependent MTase TRM10-type domain-containing protein			1	1.2662	4	5	15
G1T361	Q96I99	SUCLG2	Succinate--CoA ligase [GDP-forming] subunit beta, mitochondrial	Succinate--CoA ligase [GDP-forming] subunit beta, mitochondrial	97	2	1.2653	17	38	49
G1T7A8	Q9Y646	CPQ	Peptidase_M28 domain-containing protein	Carboxypeptidase Q	87	2	1.2653	2	2	4
G1SRD2	P28330	ACADL	Uncharacterized protein	Long-chain specific acyl-CoA dehydrogenase, mitochondrial	82	3	1.2644	5	83	17
G1U1H3	P61018	RAB4B	Uncharacterized protein	Ras-related protein Rab-4B	100	3	1.2644	4	6	31
G1SRZ0		TOR2A	Torsin family 2 member A			1	1.2644	2	3	20
G1SSB5	C9JLU1	POLR2H	DNA-directed RNA polymerases I, II, and III subunit RPABC3	DNA-directed RNA polymerases I, II, and III subunit RPABC3 (Fragment)	100	2	1.2625	2	2	29
G1T7J9	B1AV70	YIPF6	Protein YIPF	Protein YIPF (Fragment)	75	2	1.2616	4	9	12
P19943	P05387	RPLP2	60S acidic ribosomal protein P2 (Fragment)	60S acidic ribosomal protein P2	100	2	1.2607	8	22	64
G1TMS5	P48643	CCT5	Uncharacterized protein	T-complex protein 1 subunit epsilon	99	3	1.2589	31	87	70
G1SQP9	E7EMS6	COMT	Catechol-O-methyltransferase	Catechol O-methyltransferase (Fragment)	78	2	1.2589	10	34	52
G1T235	P28072	PSMB6	Proteasome subunit beta	Proteasome subunit beta type-6	97	2	1.2589	6	12	40
G1TKE3	X6RJP6	TAGLN2	Transgelin	Transgelin-2 (Fragment)	77	2	1.2589	6	11	40
G1T720	F5GXX5	DAD1	Dolichyl-diphosphooligosaccharide--protein glycosyltransferase subunit DAD1	Dolichyl-diphosphooligosaccharide--protein glycosyltransferase subunit DAD1	75	2	1.2589	3	5	38
G1SVB6	P00367	GLUD1	Glutamate dehydrogenase	Glutamate dehydrogenase 1, mitochondrial	98	2	1.2579	23	166	64
G1TDI0	Q9UQ80	PA2G4	Peptidase_M24 domain-containing protein	Proliferation-associated protein 2G4	98	2	1.2570	12	71	43

G1T2R2	A0A3B3ITU8	ITPR1	Uncharacterized protein	Inositol 1,4,5-trisphosphate receptor type 1	97	3	1.2570	4	2	2
G1U6N8	Q14980	NUMA1	Nuclear mitotic apparatus protein 1	Nuclear mitotic apparatus protein 1	90	2	1.2561	21	28	15
G1SHK6	Q5SSJ5	HP1BP3	Uncharacterized protein	Heterochromatin protein 1-binding protein 3	93	3	1.2561	11	22	26
G1TBW2	E7EQB8	IDH3G	Isocitrate dehydrogenase [NAD] subunit, mitochondrial	Isocitrate dehydrogenase [NAD] subunit, mitochondrial	94	2	1.2561	6	8	27
G1SCS8		ANO6	Anoctamin			1	1.2561	5	6	8
G1SPB2		RNMT	mRNA cap guanine-N7 methyltransferase			1	1.2552	4	4	11
G1SDN9	Q9P2B2	PTGFRN	Uncharacterized protein	Prostaglandin F2 receptor negative regulator	91	3	1.2543	6	11	8
G1TEI0		PARP1	Poly [ADP-ribose] polymerase			1	1.2533	3	3	5
G1SSA2	Q13200	PSMD2	26S proteasome non-ATPase regulatory subunit 2	26S proteasome non-ATPase regulatory subunit 2	99	2	1.2524	30	85	47
G1SRI8	P19367	HK1	Uncharacterized protein	Hexokinase-1	96	3	1.2524	27	50	33
G1SDT9	P49754	VPS41	Vacuolar protein sorting-associated protein 41 homolog	Vacuolar protein sorting-associated protein 41 homolog	98	2	1.2524	5	6	8
G1SRP2	Q5SWX8	ODR4	Uncharacterized protein	Protein odr-4 homolog	89	3	1.2506	12	13	43
P42675	E9PCB6	NLN	Neurolysin, mitochondrial	Neurolysin, mitochondrial	94	2	1.2506	6	12	15
G1T2G6	Q9P265	DIP2B	DMAP-interaction domain-containing protein	Disco-interacting protein 2 homolog B	99	2	1.2506	2	2	2
U3KN22	Q9Y2Q3	GSTK1	Glutathione S-transferase kappa	Glutathione S-transferase kappa 1	79	2	1.2496	9	19	47
G1T855	C9J5X1	IGF1R	Tyrosine-protein kinase receptor	Tyrosine-protein kinase receptor	93	2	1.2496	8	13	10
G1SGA5	Q9HCJ6	VAT1L	PKS_ER domain-containing protein	Synaptic vesicle membrane protein VAT-1 homolog-like	96	2	1.2496	3	2	10
G1T0W7	M0R1B0	EMC8	ER membrane protein complex subunit 8	ER membrane protein complex subunit 8 (Fragment)	98	2	1.2478	3	3	42
G1SQL1	Q9HAU5	UPF2	Uncharacterized protein	Regulator of nonsense transcripts 2	97	3	1.2478	2	2	2
G1T2G4	P05198	EIF2S1	Eukaryotic translation initiation factor 2 subunit 1	Eukaryotic translation initiation factor 2 subunit 1	99	2	1.2460	14	32	53
G1T8Y0		COASY	CTP_transf_like domain-containing protein			1	1.2460	2	2	5
G1TBS4	P20020	ATP2B1	Calcium-transporting ATPase	Plasma membrane calcium-transporting ATPase 1	97	2	1.2441	6	5	7
G1SPR9	P04844	RPN2	Dolichyl-diphosphooligosaccharide--protein glycosyltransferase subunit 2	Dolichyl-diphosphooligosaccharide--protein glycosyltransferase subunit 2	93	2	1.2432	25	93	68
G1U5B3	C9JIZ6	PSAP	Prosaposin	Prosaposin	78	2	1.2432	13	47	40
G1U0U5	E9PDE8	HSPA4L	Uncharacterized protein	Heat shock 70 kDa protein 4L	91	3	1.2432	5	4	8
G1TFM5	M0R0F0	RPS5	Ribosomal_S7 domain-containing protein	40S ribosomal protein S5 (Fragment)	100	2	1.2423	8	62	56
G1U9U0	P50991	CCT4	T-complex protein 1 subunit delta	T-complex protein 1 subunit delta	99	2	1.2414	23	58	56
G1TH06	F6WQW2	RANBP1	RAN binding protein 1	Ran-specific GTPase-activating protein	91	2	1.2414	4	7	28
G1TE39	Q9NYU1	UGGT2	Uncharacterized protein	UDP-glucose:glycoprotein glucosyltransferase 2	84	3	1.2395	22	23	22
P00169	P00167	CYB5A	Cytochrome b5	Cytochrome b5	90	2	1.2395	7	14	80
G1SLD7	Q9NXF1	TEX10	Ipi1_N domain-containing protein	Testis-expressed protein 10	94	2	1.2395	3	4	5
G1SV40	P83436	COG7	Uncharacterized protein	Conserved oligomeric Golgi complex subunit 7	93	3	1.2386	5	8	10
G1SE56	F6T1Q0	PDE12	Endo/exonuclease/phosphatase domain-containing protein	2~,5~-phosphodiesterase 12	90	2	1.2386	3	2	10
G1T5A5		RTN4IP1	PKS_ER domain-containing protein			1	1.2386	2	3	12
U3KPB2	P61009	SPCS3	Signal peptidase complex subunit 3	Signal peptidase complex subunit 3	100	2	1.2386	2	4	13
G1T1C4		HSD17B7	Hydroxysteroid 17-beta dehydrogenase 7			1	1.2367	3	3	16
G1STL1	Q8WUA2	PPIL4	Peptidyl-prolyl cis-trans isomerase	Peptidyl-prolyl cis-trans isomerase-like 4	97	2	1.2367	2	3	6
G1T3A6	A0A1W2PQH3	ME2	Malic enzyme	Malic enzyme	92	2	1.2358	16	14	40
G1T2V6	Q99747	NAPG	Uncharacterized protein	Gamma-soluble NSF attachment protein	98	3	1.2358	3	3	8
	Q8NBJ5	COLGALT1		Procollagen galactosyltransferase 1		4	1.2340	7	37	14
G1U5M7	A0A3B3IUA2	SNU13	Ribonucloprotein	Ribonucloprotein	100	2	1.2340	4	11	23
G1SCE7	P55084	HADHB	Uncharacterized protein	Trifunctional enzyme subunit beta, mitochondrial	94	3	1.2331	14	17	42
G1SYL8	I3L0M9	ELOB	Elongin B	Elongin-B (Fragment)	82	2	1.2331	4	6	43
	A0A087WUC6	SPCS2		Signal peptidase complex subunit 2		4	1.2321	6	16	33
G1U484		LLGL1	LLGL scribble cell polarity complex component 1			1	1.2321	2	2	3
G1SYI2	P62873	GNB1	WD_REPEATS_REGION domain-containing protein	Guanine nucleotide-binding protein G(I)/G(S)/G(T) subunit beta-1	100	2	1.2294	12	47	45
G1SMP5	Q5T160	RARS2	DALR_1 domain-containing protein	Probable arginine--tRNA ligase, mitochondrial	93	2	1.2294	6	6	10
G1SE12	P13804	ETFA	Electron transfer flavoprotein subunit alpha	Electron transfer flavoprotein subunit alpha, mitochondrial	95	2	1.2285	11	17	56
G1TFL3	J3KQE5	RAN	GTP-binding nuclear protein Ran	GTP-binding nuclear protein Ran (Fragment)	96	2	1.2285	9	23	36
G1TBH1	A0A1W2PPT5	POLR2B	DNA-directed RNA polymerase subunit beta	DNA-directed RNA polymerase subunit beta	100	2	1.2285	6	7	7
G1U2J5	Q8NE01	CNNM3	Uncharacterized protein	Metal transporter CNNM3	88	3	1.2285	2	2	7
G1SIJ2	P24752	ACAT1	Uncharacterized protein	Acetyl-CoA acetyltransferase, mitochondrial	92	3	1.2275	13	22	47
G1SY50	E7EPT4	NDUFV2	Uncharacterized protein	NADH dehydrogenase [ubiquinone] flavoprotein 2, mitochondrial	98	3	1.2275	4	7	21
P29694	P26641	EEF1G	Elongation factor 1-gamma	Elongation factor 1-gamma	98	2	1.2257	21	80	64
G1T9V4	G3V5Z7	PSMA6	Proteasome subunit alpha type	Proteasome subunit alpha type	97	2	1.2257	10	60	47
G1SZM2	A0A0A0MRG2	APP	Amyloid-beta A4 protein	Amyloid-beta precursor protein	99	2	1.2257	7	8	11
G1T1B6	Q16832	DDR2	Uncharacterized protein	Discoidin domain-containing receptor 2	97	3	1.2248	5	6	9

G1U276	A0A2R8Y6Y7	SUCLA2	Succinate--CoA ligase [ADP-forming] subunit beta, mitochondrial	Succinate--CoA ligase [ADP-forming] subunit beta, mitochondrial	91	2	1.2238	13	22	38
G1SYL3	E9PFH4	TNPO3	Xpo1 domain-containing protein	Transportin-3	96	2	1.2238	6	10	12
G1T5H5	Q15050	RRS1	Ribosome biogenesis regulatory protein	Ribosome biogenesis regulatory protein homolog	92	2	1.2238	5	6	20
G1SDL7		ARG2	Arginase			1	1.2238	2	2	8
G1SKE2	E7ETC0	TIAL1	TIA1 cytotoxic granule associated RNA binding protein like 1	Nucleolysin TIAR	98	2	1.2229	2	6	8
G1SWN4	Q9H832	UBE2Z	UBIQUITIN_CONJUGAT_2 domain-containing protein	Ubiquitin-conjugating enzyme E2 Z	96	2	1.2220	3	3	11
O19048	Q15365	PCBP1	Poly(rC)-binding protein 1	Poly(rC)-binding protein 1	100	2	1.2211	14	25	64
G1SFU0	P51149	RAB7A	Ras-related protein Rab-7a	Ras-related protein Rab-7a	100	2	1.2211	12	25	61
G1SK67	Q13547	HDAC1	Histone deacetylase	Histone deacetylase 1	99	2	1.2211	6	4	23
G1T336	A0A0D9SF53	DDX3X	Uncharacterized protein	ATP-dependent RNA helicase DDX3X	99	3	1.2202	19	43	31
G1TXS5	Q9UGP8	SEC63	J domain-containing protein	Translocation protein SEC63 homolog	93	2	1.2192	12	22	21
	P08574	CYC1		Cytochrome c1, heme protein, mitochondrial		4	1.2192	4	5	16
G1SV81	Q9BTE1	DCTN5	Uncharacterized protein	Dynactin subunit 5	100	3	1.2192	2	2	9
G1T1X4	O43395	PRPF3	PWI domain-containing protein	U4/U6 small nuclear ribonucleoprotein Prp3	100	2	1.2192	2	2	7
A0A140TAV6		HBB2	Globin A1			1	1.2183	2	2	13
B7NZS4	O75175	CNOT3	CCR4-NOT transcription complex, subunit 3 (Predicted)	CCR4-NOT transcription complex subunit 3	97	2	1.2174	3	5	9
G1T594	G0XQ39	STIM1	Stromal interaction molecule 1	STIM1L	98	2	1.2174	2	3	3
G1TWU8	O43264	ZW10	Uncharacterized protein	Centromere/kinetochore protein zw10 homolog	89	3	1.2155	10	14	20
	A0A087WVZ9	POLR2E		DNA-directed RNA polymerases I, II, and III subunit RPABC1		4	1.2146	2	3	17
G1TCT3	F8VPD4	CAD	Carbamoyl-phosphate synthetase 2, aspartate transcarbamylase, and dihydroorotase	CAD protein	94	2	1.2137	18	7	13
G1SCF6		YARS2	Tyrosine--tRNA ligase			1	1.2137	5	5	20
G1TST9	A0A2R8Y212	CHD4	Uncharacterized protein	Chromodomain-helicase-DNA-binding protein 4	97	3	1.2128	20	18	18
G1U7X2	Q15637	SF1	CCHC-type domain-containing protein	Splicing factor 1	99	2	1.2128	4	8	12
G1U115	Q15008	PSMD6	PCI domain-containing protein	26S proteasome non-ATPase regulatory subunit 6	99	2	1.2119	16	39	47
G1SGE5	A0A087WZR9	PYCR2	Pyrroline-5-carboxylate reductase	Pyrroline-5-carboxylate reductase	94	2	1.2119	6	5	26
G1TQA4		EPM2AIP1	EPM2A interacting protein 1			1	1.2119	4	5	10
G1SV05	P34932	HSPA4	Uncharacterized protein	Heat shock 70 kDa protein 4	97	3	1.2109	25	23	45
G1SQA4	Q6UWP7	LCLAT1	PlsC domain-containing protein	Lysocardiolipin acyltransferase 1	89	2	1.2109	5	5	15
G1T0L9	P04843	RPN1	Dolichyl-diphosphooligosaccharide--protein glycosyltransferase subunit 1	Dolichyl-diphosphooligosaccharide--protein glycosyltransferase subunit 1	97	2	1.2100	28	231	53
G1SZD2	P53701	HCCS	Cytochrome c heme lyase	Cytochrome c-type heme lyase	83	2	1.2100	4	7	17
G1SDN4	O60506-3	SYNCRIP	Uncharacterized protein	Isoform 3 of Heterogeneous nuclear ribonucleoprotein Q	100	3	1.2091	22	50	49
G1T7R4	P18084	ITGB5	Integrin beta	Integrin beta-5	93	2	1.2082	5	6	8
G1SD98	Q13523	PRPF4B	Pre-mRNA processing factor 4B	Serine/threonine-protein kinase PRP4 homolog	98	2	1.2082	4	6	4
G1SHA4		TEP1	Telomerase associated protein 1			1	1.2073	5	8	3
G1SQU5	Q9UP95	SLC12A4	Solute carrier family 12 member 4	Solute carrier family 12 member 4	97	2	1.2063	13	23	16
G1TJX7	P50552	VASP	Vasodilator stimulated phosphoprotein	Vasodilator-stimulated phosphoprotein	87	2	1.2063	2	5	6
	P50454	SERPINH1		Serpin H1		4	1.2054	15	30	35
G1T3D9	P62241	RPS8	40S ribosomal protein S8	40S ribosomal protein S8	94	2	1.2054	6	22	37
G1T6T8	O94906	PRPF6	Uncharacterized protein	Pre-mRNA-processing factor 6	96	3	1.2045	5	5	7
G1SNR2	Q969S9	GFM2	Ribosome-releasing factor 2, mitochondrial	Ribosome-releasing factor 2, mitochondrial	88	2	1.2045	4	4	10
Q9TT15	P21796	VDAC1	Voltage-dependent anion-selective channel protein 1	Voltage-dependent anion-selective channel protein 1	100	2	1.2036	18	405	82
G1T866	Q96CS3	FAF2	UBX domain-containing protein	FAS-associated factor 2	99	2	1.2026	7	18	24
G1T9P2		CARKD	ATP-dependent (S)-NAD(P)H-hydrate dehydratase			1	1.1999	6	11	31
G1SLM1	Q9HB71	CACYBP	Uncharacterized protein	Calcyclin-binding protein	92	3	1.1999	5	4	22
G1T1R4		NR3C1	Glucocorticoid receptor			1	1.1999	2	3	6
G1SRN1		LAS1L	LAS1 like, ribosome biogenesis factor			1	1.1990	4	3	11
G1TEE3	G3V5Z3	PPP4R3A	SMK-1 domain-containing protein	Serine/threonine-protein phosphatase 4 regulatory subunit 3A	100	2	1.1990	4	7	9
G1SZ15	A8K878	MANF	Mesencephalic astrocyte derived neurotrophic factor	Mesencephalic astrocyte-derived neurotrophic factor	97	2	1.1980	6	16	30
G1SF08	C9K025	RPL35A	Uncharacterized protein	60S ribosomal protein L35a (Fragment)	99	3	1.1980	3	2	23
G1SWW7	B4DJK0	SRSF5	Uncharacterized protein	Serine/arginine-rich-splicing factor 5	100	3	1.1980	2	4	8
G1SFS8	Q7KZF4	SND1	Staphylococcal nuclease domain-containing protein	Staphylococcal nuclease domain-containing protein 1	97	2	1.1971	35	87	54
G1T8P4	P05386	RPLP1	Uncharacterized protein	60S acidic ribosomal protein P1	97	3	1.1971	4	10	72
B6V9S9	P78371	CCT2	Chaperonin-containing T-complex polypeptide beta subunit	T-complex protein 1 subunit beta	99	2	1.1962	27	157	68
G1SQU6	O75489	NDUFS3	Complex1_30kDa domain-containing protein	NADH dehydrogenase [ubiquinone] iron-sulfur protein 3, mitochondrial	91	2	1.1953	4	4	21
G1U9S7	P17987	TCP1	Uncharacterized protein	T-complex protein 1 subunit alpha	97	3	1.1944	26	370	62
P27124	Q02790	FKBP4	Peptidyl-prolyl cis-trans isomerase FKBP4	Peptidyl-prolyl cis-trans isomerase FKBP4	91	2	1.1944	9	13	26
G1SW97	A0A0A0MT83	IVD	Uncharacterized protein	Isovaleryl-CoA dehydrogenase isoform 1	95	3	1.1944	7	10	17

G1SEE0	Q09028	RBBP4	RB binding protein 4, chromatin remodeling factor	Histone-binding protein RBBP4	100	2	1.1944	6	3	23
G1SRV1	K7ELV2	SEH1L	Uncharacterized protein	Nucleoporin SEH1 (Fragment)	92	3	1.1944	5	6	18
G1SD54	Q99720	SIGMAR1	Uncharacterized protein	Sigma non-opioid intracellular receptor 1	97	3	1.1944	4	5	23
	B5MDE0	RFT1		Protein RFT1 homolog		4	1.1925	2	3	4
G1T095		TMEM147	Transmembrane protein 147			1	1.1916	2	2	18
G1SR03	P55072	VCP	Uncharacterized protein	Transitional endoplasmic reticulum ATPase	100	3	1.1897	39	182	69
G1T619		MCUB	MCU domain-containing protein			1	1.1888	3	3	14
G1TCE9		HSDL2	SCP2 domain-containing protein			1	1.1879	8	16	28
G1SCQ1		AKR7L	Aldo_ket_red domain-containing protein			1	1.1870	7	8	35
U3KM62		PTGS2	Prostaglandin G/H synthase 2			1	1.1870	6	7	14
G1SG37	G5E9T8	GOSR1	Golgi SNAP receptor complex member 1	Golgi SNAP receptor complex member 1 (Fragment)	99	2	1.1870	3	6	20
G1SME1		NOP14	NOP14 nucleolar protein			1	1.1870	3	5	6
	Q9UNK0	STX8		Syntaxin-8		4	1.1870	2	2	12
	Q92900	UPF1		Regulator of nonsense transcripts 1		4	1.1861	18	25	23
G1TRG8	P04899	GNAI2	Uncharacterized protein	Guanine nucleotide-binding protein G(i) subunit alpha-2	98	3	1.1861	15	18	50
G1TIT1	O75477	ERLIN1	PHB domain-containing protein	Erlin-1	98	2	1.1861	11	9	41
G1T519	P25789	PSMA4	Proteasome subunit alpha type	Proteasome subunit alpha type-4	100	2	1.1861	10	21	58
G1U0M5	H7C1W1	PXDN	Peroxidasin	Peroxidasin homolog (Fragment)	93	2	1.1861	5	3	5
G1T578	E9PHY0	ACP2	Uncharacterized protein	Lysosomal acid phosphatase	94	3	1.1861	4	7	14
G1SWE5		WDR61	WD repeat domain 61			1	1.1861	2	3	12
G1TE27	X6R9L0	DNAJC3	Uncharacterized protein	DnaJ homolog subfamily C member 3	88	3	1.1851	13	5	33
G1SLK2	P62195	PSMC5	AAA domain-containing protein	26S proteasome regulatory subunit 8	100	2	1.1833	14	27	43
G1SSL0	P30040	ERP29	Endoplasmic reticulum resident protein 29	Endoplasmic reticulum resident protein 29	93	2	1.1833	9	35	46
G1SZ59	P60842	EIF4A1	Eukaryotic initiation factor 4A-I	Eukaryotic initiation factor 4A-I	100	2	1.1824	23	189	76
G1U013	H7C3P9	COPS3	PCI domain-containing protein	COP9 signalosome complex subunit 3	92	2	1.1824	4	5	18
	P56385	ATP5ME		ATP synthase subunit e, mitochondrial		4	1.1824	3	3	22
G1SSL8		HEATR5A	HEAT repeat containing 5A			1	1.1815	4	3	3
G1SP33		SYMPK	Symplekin			1	1.1815	2	2	3
G1SZI6	Q86X10	RALGAPB	Rap-GAP domain-containing protein	Ral GTPase-activating protein subunit beta	97	2	1.1805	4	3	3
	A0A0C4DGG8	CCAR1		Cell division cycle and apoptosis regulator protein 1 (Fragment)		4	1.1805	3	3	5
	P62701	RPS4X		40S ribosomal protein S4, X isoform		4	1.1796	15	39	52
G1T7Q2		LOXL2	Lysyl oxidase like 2			1	1.1796	6	7	9
G1SXB8	Q10567	AP1B1	AP complex subunit beta	AP-1 complex subunit beta-1	98	2	1.1787	32	8	45
G1SQV5	O15371	EIF3D	Eukaryotic translation initiation factor 3 subunit D	Eukaryotic translation initiation factor 3 subunit D	99	2	1.1787	13	23	39
G1TN13	A8CTZ0	ITSN1	Intersectin 1	Intersectin 1 short form variant 13	88	2	1.1787	5	5	12
G1SVD7		SYNJ2BP-COX	PDZ domain-containing protein			1	1.1778	2	2	15
G1SKK0	P22102	GART	Trifunctional purine biosynthetic protein adenosine-3	Trifunctional purine biosynthetic protein adenosine-3	89	2	1.1768	6	9	12
G1TAL6	Q10471	GALNT2	Polypeptide N-acetylgalactosaminyltransferase	Polypeptide N-acetylgalactosaminyltransferase 2	99	2	1.1759	10	18	23
G1SRZ8	A5YKK6	CNOT1	Uncharacterized protein	CCR4-NOT transcription complex subunit 1	100	3	1.1759	8	9	5
	J3QQX2	ARHGDIA		Rho GDP-dissociation inhibitor 1		4	1.1759	5	7	18
G1SGI8	O95881	TXNDC12	Thioredoxin domain-containing protein	Thioredoxin domain-containing protein 12	95	2	1.1759	5	10	37
	P68402	PAFAH1B2		Platelet-activating factor acetylhydrolase IB subunit beta		4	1.1759	4	11	37
G1SKF7	Q02878	RPL6	60S ribosomal protein L6	60S ribosomal protein L6	89	2	1.1750	11	39	40
G1SIA3	Q8N3C0	ASCC3	Uncharacterized protein	Activating signal cointegrator 1 complex subunit 3	94	3	1.1741	7	8	5
G1TA69	Q8TBA6	GOLGA5	Uncharacterized protein	Golgin subfamily A member 5	83	3	1.1741	4	4	6
G1U6Y3		MEAK7	MTOR associated protein, eak-7 homolog			1	1.1741	3	4	16
G1SV60	J3KS45	TMCO1	Calcium load-activated calcium channel	Calcium load-activated calcium channel (Fragment)	95	2	1.1741	2	2	13
G1SNZ3	P55060	CSE1L	Chromosome segregation 1 like	Exportin-2	99	2	1.1732	13	35	26
G1SHI2		MYD88	Myeloid differentiation primary response protein MyD88			1	1.1732	2	2	9
G1U4H9	O14907	TAX1BP3	Tax1-binding protein 3	Tax1-binding protein 3	100	2	1.1732	2	4	28
	P13639	EEF2		Elongation factor 2		4	1.1722	37	281	58
G1SPD2	Q16401	PSMD5	Uncharacterized protein	26S proteasome non-ATPase regulatory subunit 5	92	3	1.1722	12	15	43
G1SDU6	P26639	TARS	AA_TRNA_LIGASE_II domain-containing protein	Threonine--tRNA ligase, cytoplasmic	96	2	1.1713	17	27	28
G1TUK6	O14656	TOR1A	Torsin family 1 member A	Torsin-1A	90	2	1.1713	7	8	25
U3KM71		ATP5MG	ATP synthase subunit			1	1.1704	5	17	62
	A0A3B3IRT8	SSR1		Translocon-associated protein subunit alpha		4	1.1695	4	78	21
G1T970	P30038	ALDH4A1	Multifunctional fusion protein	Delta-1-pyrroline-5-carboxylate dehydrogenase, mitochondrial	91	2	1.1685	11	23	31

G9GLC3		ATP1B3	Sodium/potassium-transporting ATPase subunit beta-3			1	1.1685	3	5	15
G1U4G9	O00299	CLIC1	Chloride intracellular channel protein	Chloride intracellular channel protein 1	98	2	1.1676	6	11	42
	Q96PU8	QKI		Protein quaking		4	1.1676	4	6	16
G1U3V0	Q9BX68	HINT2	HIT domain-containing protein	Histidine triad nucleotide-binding protein 2, mitochondrial	94	2	1.1667	4	7	41
G1T5C5	E5RHW4	ERLIN2	PHB domain-containing protein	Erlin-2 (Fragment)	98	2	1.1658	8	5	36
G1SDD2	B7Z2Y2	COG2	Uncharacterized protein	Conserved oligomeric Golgi complex subunit 2	91	3	1.1649	6	10	15
G1U8P2	J3KTE4	RPL19	Ribosomal protein L19	Ribosomal protein L19	98	2	1.1639	7	15	30
G1TBW7	P50148	GNAQ	Uncharacterized protein	Guanine nucleotide-binding protein G(q) subunit alpha	99	3	1.1630	11	16	38
G1SVU0	C9JAZ1	MTX2	Uncharacterized protein	Metaxin-2 (Fragment)	89	3	1.1630	4	13	37
	A0A0A0MRA3	TTN		Titin		4	1.1630	2	2	0
G1SSM6	Q8WUM0	NUP133	Nucleoporin_C domain-containing protein	Nuclear pore complex protein Nup133	91	2	1.1621	14	20	23
G1SUT8	O95486	SEC24A	Uncharacterized protein	Protein transport protein Sec24A	93	3	1.1621	5	8	7
G1SLE1		HARS2	AA_TRNA_LIGASE_II domain-containing protein			1	1.1621	4	3	10
G1TTB5	A0A0D9SEM4	SRSF4	Serine and arginine rich splicing factor 4	Serine/arginine-rich-splicing factor 4 (Fragment)	75	2	1.1621	3	2	8
G1SI20	O75874	IDH1	Isocitrate dehydrogenase [NADP]	Isocitrate dehydrogenase [NADP] cytoplasmic	97	2	1.1612	19	17	49
G1T3M3	Q9UL25	RAB21	Uncharacterized protein	Ras-related protein Rab-21	97	3	1.1593	5	13	30
G1SN52	E7ESC6	XPO7	Exportin 7	Exportin-7	99	2	1.1593	5	9	7
G1U5Z2		TK2	dNK domain-containing protein			1	1.1593	2	2	11
G1T7X6	G3XAI2	LAMB1	Uncharacterized protein	Laminin subunit beta-1	91	3	1.1584	3	4	3
G1SYD6	P02545	LMNA	Uncharacterized protein	Prelamin-A/C	98	3	1.1575	33	90	52
G1T813	P08559	PDHA1	Pyruvate dehydrogenase E1 component subunit alpha	Pyruvate dehydrogenase E1 component subunit alpha, somatic form, mitochondrial	99	2	1.1575	12	22	35
	P30044	PRDX5		Peroxiredoxin-5, mitochondrial		4	1.1575	5	9	33
G1TCY7		EIF2B4	Translation initiation factor eIF-2B subunit delta			1	1.1575	3	3	8
G1SR15		CD109	CD109 molecule			1	1.1575	2	3	2
G1SCW7	Q9UG63	ABCF2	Uncharacterized protein	ATP-binding cassette sub-family F member 2	99	3	1.1556	6	9	11
G1T573	H0Y3P2	EIF4G2	Eukaryotic translation initiation factor 4 gamma 2	Eukaryotic translation initiation factor 4 gamma 2	95	2	1.1547	18	33	26
G1T3H3	Q8IXI2	RHOT1	Mitochondrial Rho GTPase	Mitochondrial Rho GTPase 1	99	2	1.1547	7	8	13
G1TNH0	O14939	PLD2	Phospholipase D2	Phospholipase D2	89	2	1.1538	3	2	7
	Q9NYU2	UGGT1		UDP-glucose:glycoprotein glucosyltransferase 1		4	1.1529	19	5	19
G1SHK7	A0A0C4DGV4	LAMTOR5	Uncharacterized protein	Hepatitis B virus x interacting protein	100	3	1.1529	3	7	31
G1SWA0		RIPOR1	RHO family interacting cell polarization regulator 1			1	1.1529	3	3	5
A0A140TAW0	O43852	CALU	Calumenin	Calumenin	99	2	1.1520	15	90	64
G1SXF1		NFU1	Nfu_N domain-containing protein			1	1.1520	4	11	22
G1SEV2	P30101	PDIA3	Protein disulfide-isomerase	Protein disulfide-isomerase A3	96	2	1.1510	30	226	45
	A0A0A6YYJ8	LUC7L2		Putative RNA-binding protein Luc7-like 2		4	1.1510	4	7	11
G1SSR8	O76031	CLPX	Uncharacterized protein	ATP-dependent Clp protease ATP-binding subunit clpX-like, mitochondrial	97	3	1.1501	5	4	13
G1TJR5	Q9NZL4	HSPBP1	HSPA (Hsp70) binding protein 1	Hsp70-binding protein 1	96	2	1.1501	4	7	22
G1T2G5	Q5NDL2	EOGT	Uncharacterized protein	EGF domain-specific O-linked N-acetylglucosamine transferase	91	3	1.1492	8	10	18
G1SLS8	Q9NX62	IMPAD1	Uncharacterized protein	Inositol monophosphatase 3	96	3	1.1492	6	8	21
G1SRY7	K7ESP4	DCAKD	Uncharacterized protein	Dephospho-CoA kinase domain-containing protein (Fragment)	92	3	1.1492	4	7	29
G1T2N8	O43592	XPOT	Exportin-T	Exportin-T	99	2	1.1492	4	7	6
G1T9F3	Q14974	KPNB1	Importin N-terminal domain-containing protein	Importin subunit beta-1	99	2	1.1483	28	102	50
G1TD24	Q9NRY4	ARHGAP35	Rho GTPase activating protein 35	Rho GTPase-activating protein 35	98	2	1.1483	4	3	3
G1U3S3		KIAA2013	KIAA2013			1	1.1483	4	4	11
G1SLT8	P31942	HNRNPH3	Uncharacterized protein	Heterogeneous nuclear ribonucleoprotein H3	100	3	1.1474	6	6	24
G1SZ66	I3L295	MPDU1	Uncharacterized protein	Mannose-P-dolichol utilization defect 1 isoform 2	89	3	1.1474	3	5	15
G1T0V4	F5H013	SNRPG	Small nuclear ribonucleoprotein G	Small nuclear ribonucleoprotein G	98	2	1.1474	3	19	58
G1T1L4		GRWD1	WD_REPEATS_REGION domain-containing protein			1	1.1464	3	4	13
Q8HZQ5	E7EQR4	EZR	Ezrin	Ezrin	94	2	1.1455	15	11	28
G1T1T8	O96005	CLPTM1	CLPTM1, transmembrane protein	Cleft lip and palate transmembrane protein 1	97	2	1.1455	11	26	21
G1T085		SVIL	HP domain-containing protein			1	1.1455	4	7	4
G1SRQ2	A0A087WTB8	UCHL3	Ubiquitin carboxyl-terminal hydrolase	Ubiquitin carboxyl-terminal hydrolase	98	2	1.1455	3	4	21
G1T279	Q9UHW5	GPN3	GPN-loop GTPase 3	GPN-loop GTPase 3	96	2	1.1455	2	3	12
O46373	P12235	SLC25A4	ADP/ATP translocase 1	ADP/ATP translocase 1	96	2	1.1446	18	25	71
G1TTK6	E7ETT1	PCCB	Uncharacterized protein	Propionyl-CoA carboxylase beta chain, mitochondrial	86	3	1.1446	9	14	28
G1T9J3	A2RRP1	NBAS	Uncharacterized protein	Neuroblastoma-amplified sequence	90	3	1.1437	19	28	12
G1SFN5	E9PFR3	PPP2R5D	Serine/threonine-protein phosphatase 2A 56 kDa regulatory subunit	Serine/threonine-protein phosphatase 2A 56 kDa regulatory subunit	98	2	1.1437	6	10	14

G1T7I0	Q96PU5	NEDD4L	E3 ubiquitin-protein ligase	E3 ubiquitin-protein ligase NEDD4-like	96	2	1.1437	3	4	5
G1SN68	A0A1B0GVU9	QARS	Uncharacterized protein	Glutamine--tRNA ligase (Fragment)	92	3	1.1427	25	41	43
G1SGX4	P62249	RPS16	Uncharacterized protein	40S ribosomal protein S16	100	3	1.1427	7	13	37
G1SK61	Q9UIW2	PLXNA1	Plexin A1	Plexin-A1	91	2	1.1427	3	4	4
G1SUJ1	P24390	KDELR1	ER lumen protein-retaining receptor	ER lumen protein-retaining receptor 1	96	2	1.1418	3	2	21
G1TWC3		TMX1	Thioredoxin domain-containing protein			1	1.1409	5	5	19
	K7EK33	DAZAP1		DAZ-associated protein 1		4	1.1409	4	6	18
Q28611		UGT1	UDP-glucuronosyltransferase 1-6			1	1.1400	10	13	24
G1T897	B4DP72	COQ5	2-methoxy-6-polyprenyl-1,4-benzoquinol methylase, mitochondrial	2-methoxy-6-polyprenyl-1,4-benzoquinol methylase, mitochondrial	77	2	1.1400	2	3	12
G1STD4	Q9BSR8	YIPF4	Protein YIPF	Protein YIPF4	99	2	1.1400	2	2	9
G1T4X8	P49721	PSMB2	Proteasome subunit beta	Proteasome subunit beta type-2	99	2	1.1391	8	44	60
G1T645	Q96S52	PIGS	Uncharacterized protein	GPI transamidase component PIG-S	85	3	1.1391	6	11	17
G1TMD8		PPP5C	Serine/threonine-protein phosphatase			1	1.1391	3	3	10
G1T6G1		MMAB	Corrinoid adenosyltransferase			1	1.1381	3	4	19
G1T0H9	O60645	EXOC3	Exocyst complex component 3	Exocyst complex component 3	95	2	1.1372	3	4	5
G1SU02	A0A1W2PP11	PARL	Rhomboid domain-containing protein	Presenilins-associated rhomboid-like protein, mitochondrial	94	2	1.1372	2	2	6
G1SU30	A0A024RCR6	BAG6	Ubiquitin-like domain-containing protein	BAG6	91	2	1.1354	8	8	12
G1SH30	Q8WVY7	UBLCP1	Uncharacterized protein	Ubiquitin-like domain-containing CTD phosphatase 1	100	3	1.1354	2	2	10
G1TRL5	A0A087WUT6	EIF5B	Tr-type G domain-containing protein	Eukaryotic translation initiation factor 5B	95	2	1.1345	21	44	22
G1T156	Q9HB40	SCPEP1	Carboxypeptidase	Retinoid-inducible serine carboxypeptidase	86	2	1.1345	11	20	26
G1SUM3	Q8TBC4	UBA3	E2_bind domain-containing protein	NEDD8-activating enzyme E1 catalytic subunit	99	2	1.1345	5	6	21
G1SP24	E7EQB9	POLR1C	RPOLD domain-containing protein	DNA-directed RNA polymerases I and III subunit RPAC1	84	2	1.1345	4	7	25
P98049		MT-CO2	Cytochrome c oxidase subunit 2			1	1.1345	3	4	20
G1SZI0		SPG7	SPG7, paraplegin matrix AAA peptidase subunit			1	1.1345	3	3	10
G1U0A4	P21810	BGN	Biglycan	Biglycan	94	2	1.1335	12	15	46
G1SEF1		NDUFC2	NADH dehydrogenase [ubiquinone] 1 subunit C2			1	1.1335	2	2	19
G1T297	Q9UBV2	SEL1L	Fibronectin type-II domain-containing protein	Protein sel-1 homolog 1	97	2	1.1326	15	24	30
G1U684	A0A087X1W8	CADM1	Uncharacterized protein	Cell adhesion molecule 1	95	3	1.1326	9	14	34
U3KM96	P61224	RAP1B	Uncharacterized protein	Ras-related protein Rap-1b	100	3	1.1317	11	6	73
G1SEF8	M0R0Y2	NAPA	Uncharacterized protein	Alpha-soluble NSF attachment protein	84	3	1.1317	10	15	49
G1U3I5		ECH1	Enoyl-CoA hydratase 1			1	1.1317	9	10	37
G1TS38	H0YF06	CCDC90B	Uncharacterized protein	Coiled-coil domain-containing protein 90B, mitochondrial (Fragment)	94	3	1.1317	2	3	18
G1U4R5	A0A0C4DGS1	DDOST	Dolichyl-diphosphooligosaccharide--protein glycosyltransferase 48 kDa subunit	Dolichyl-diphosphooligosaccharide--protein glycosyltransferase 48 kDa subunit	94	2	1.1308	14	440	39
G1SYB4	P60953	CDC42	Uncharacterized protein	Cell division control protein 42 homolog	100	3	1.1308	7	27	49
G1SH86		TBCD	Tubulin folding cofactor D			1	1.1298	13	21	17
G1T511	A0A3F2YNY6	PRPF40A	Pre-mRNA processing factor 40 homolog A	Pre-mRNA-processing factor 40 homolog A	83	2	1.1298	5	6	7
G1SPX2	Q6KC79	NIPBL	Nipped-B protein	Nipped-B-like protein	98	2	1.1298	2	2	1
G1T964		TMED1	GOLD domain-containing protein			1	1.1289	3	4	27
G1TQR9		ZSWIM8	SWIM-type domain-containing protein			1	1.1289	2	3	2
G1SWU1	P55809	OXCT1	Succinyl-CoA:3-ketoacid-coenzyme A transferase	Succinyl-CoA:3-ketoacid coenzyme A transferase 1, mitochondrial	94	2	1.1280	18	109	52
G1T4Q8	J3KNQ4	PARVA	Uncharacterized protein	Alpha-parvin	91	3	1.1280	11	26	34
G1SZJ5	O15212	PFDN6	Uncharacterized protein	Prefoldin subunit 6	100	3	1.1280	5	7	38
	Q96PK6-5	RBM14		Isoform 5 of RNA-binding protein 14		4	1.1280	5	3	24
G1TC33	P35580	MYH10	Uncharacterized protein	Myosin-10	99	3	1.1271	102	310	54
G1TAM8	P61289	PSME3	Uncharacterized protein	Proteasome activator complex subunit 3	95	3	1.1271	8	10	37
G1TCU4	H3BUU9	CDH11	Uncharacterized protein	Cadherin-11	99	3	1.1271	3	7	9
G1T4H6		MTMR6	Myotubularin phosphatase domain-containing protein			1	1.1243	3	2	4
G1TWD8		SELENOO	Selenoprotein O			1	1.1234	3	4	18
G1SHV9	P49720	PSMB3	Proteasome subunit beta	Proteasome subunit beta type-3	99	2	1.1215	6	18	39
G1T9W8	Q8TC07	TBC1D15	Rab-GAP TBC domain-containing protein	TBC1 domain family member 15	92	2	1.1215	2	2	3
G1SL80		UROD	Uroporphyrinogen decarboxylase			1	1.1206	4	5	19
G1SVP9	G3V4T2	PABPN1	RRM domain-containing protein	Polyadenylate-binding protein 2	99	2	1.1206	2	2	6
G1TZI2		PGAM5	PGAM family member 5, mitochondrial serine/threonine protein phosphatase			1	1.1206	2	2	9
	W4VSQ9	TRIP10		Cdc42-interacting protein 4		4	1.1206	2	2	7
G1SJQ2	Q92896	GLG1	Uncharacterized protein	Golgi apparatus protein 1	97	3	1.1197	43	73	42
G1SM52	Q96AG4	LRRC59	Uncharacterized protein	Leucine-rich repeat-containing protein 59	97	3	1.1197	13	24	48
G1SGQ0	Q9UBS4	DNAJB11	J domain-containing protein	DnaJ homolog subfamily B member 11	98	2	1.1197	9	22	35

G1SX71		AGPAT5	1-acylglycerol-3-phosphate O-acyltransferase 5			1	1.1197	2	3	11
G1SJX1	Q16537	PPP2R5E	Serine/threonine-protein phosphatase 2A 56 kDa regulatory subunit	Serine/threonine-protein phosphatase 2A 56 kDa regulatory subunit epsilon isoform	100	2	1.1188	4	7	13
G1TA11	P54136	RARS	Uncharacterized protein	Arginine--tRNA ligase, cytoplasmic	92	3	1.1179	23	46	43
G1SRA8	P41091	EIF2S3	Eukaryotic translation initiation factor 2 subunit 3	Eukaryotic translation initiation factor 2 subunit 3	92	2	1.1169	12	27	36
G1SFG8	A0A096LNH6	DOCK1	Uncharacterized protein	Dedicator of cytokinesis protein 1	95	3	1.1169	4	7	4
G1SL85	A0A1B0GTM3	ASAH1	Uncharacterized protein	Acid ceramidase	81	3	1.1160	7	11	27
G1TY83		NLRX1	NLR family member X1			1	1.1160	3	4	6
G1SXX9	A0A0C4DGH3	NUMBL	NUMB like, endocytic adaptor protein	Numb-like protein	93	2	1.1160	2	2	4
G1T9S4	H0YDD4	DLAT	Acetyltransferase component of pyruvate dehydrogenase complex	Acetyltransferase component of pyruvate dehydrogenase complex (Fragment)	90	2	1.1151	11	22	25
G1SRD1		EXD2	3~-5~ exonuclease domain-containing protein			1	1.1151	4	4	9
	U3KQK1	LSM4		U6 snRNA-associated Sm-like protein LSm4		4	1.1151	2	5	13
G1SFG0	A0A087WVP1	FAT1	Uncharacterized protein	Protocadherin Fat 1	92	3	1.1142	11	14	4
	P29992	GNA11		Guanine nucleotide-binding protein subunit alpha-11		4	1.1142	6	2	22
G1STU4	E9PIE3	CAVIN3	Uncharacterized protein	Caveolae-associated protein 3	78	3	1.1142	4	5	15
	P39019	RPS19		40S ribosomal protein S19		4	1.1133	11	35	57
G1TS78	A0A075B6F6	HM13	Uncharacterized protein	Minor histocompatibility antigen H13 (Fragment)	84	3	1.1123	7	19	21
G1T134		ABHD11	Abhydrolase domain containing 11			1	1.1123	3	4	16
G1SXX5	A0A2R8Y4F5	HADHA	Uncharacterized protein	Trifunctional enzyme subunit alpha, mitochondrial	86	3	1.1114	30	150	52
G1SP21	H0Y9A1	YIPF3	Uncharacterized protein	Protein YIPF3 (Fragment)	72	3	1.1105	3	7	16
G1SGJ4		SRR	PALP domain-containing protein			1	1.1096	2	3	5
G1TA59	P50213	IDH3A	Isocitrate dehydrogenase [NAD] subunit, mitochondrial	Isocitrate dehydrogenase [NAD] subunit alpha, mitochondrial	98	2	1.1086	12	37	40
	A0A096LP07	GPS1		COP9 signalosome complex subunit 1		4	1.1086	4	4	10
	P55010	EIF5		Eukaryotic translation initiation factor 5		4	1.1077	5	9	15
G1SZW0	Q7L576	CYFIP1	Cytoplasmic FMR1-interacting protein	Cytoplasmic FMR1-interacting protein 1	98	2	1.1068	18	31	17
G1SRJ7	P08253	MMP2	72 kDa type IV collagenase	72 kDa type IV collagenase	95	2	1.1068	7	11	19
G1TKQ8	A0A0D9SG77	UBE3A	Ubiquitin-protein ligase E3A	Ubiquitin-protein ligase E3A	97	2	1.1068	6	6	11
	Q16394	EXT1		Exostosin-1		4	1.1059	2	3	5
G1SZ72	P51116	FXR2	Uncharacterized protein	Fragile X mental retardation syndrome-related protein 2	98	3	1.1050	8	7	20
G1SCF0	P13807	GYS1	Glycogen [starch] synthase	Glycogen [starch] synthase, muscle	96	2	1.1050	3	4	6
	Q8N122	RPTOR		Regulatory-associated protein of mTOR		4	1.1050	2	2	3
G1T925		GPX8	Glutathione peroxidase			1	1.1031	4	7	20
G1TPY7		SUMF2	FGE-sulfatase domain-containing protein			1	1.1031	3	2	13
G1SCY3		UBR4	UBR-type domain-containing protein			1	1.1022	46	77	17
G1STG2	Q9Y5M8	SRPRB	SRP receptor subunit beta	Signal recognition particle receptor subunit beta	94	2	1.1022	11	19	49
B7NZM4	Q9Y295	DRG1	Developmentally regulated GTP binding protein 1 (Predicted)	Developmentally-regulated GTP-binding protein 1	100	2	1.1022	9	20	35
G1T069	P57740	NUP107	Nuclear pore complex protein	Nuclear pore complex protein Nup107	94	2	1.1022	9	21	16
	P37198	NUP62		Nuclear pore glycoprotein p62		4	1.1022	4	5	11
G1SLF8	J3KN16	ECPAS	Vac14_Fab1_bd domain-containing protein	Proteasome adapter and scaffold protein ECM29	97	2	1.1013	18	31	17
G1T923	A0A1B0GTB0	ATP6AP2	Uncharacterized protein	Renin receptor (Fragment)	84	3	1.1013	4	4	21
G1SMZ9		ICMT	Protein-S-isoprenylcysteine O-methyltransferase			1	1.1013	2	2	11
	A0A0U1RRM4	PTBP1		Polypyrimidine tract-binding protein 1		4	1.1004	10	56	29
G1T3N8	Q13155	AIMP2	Uncharacterized protein	Aminoacyl tRNA synthase complex-interacting multifunctional protein 2	87	3	1.0994	12	17	68
G1TAA4	O75844	ZMPSTE24	CAAX prenyl protease	CAAX prenyl protease 1 homolog	96	2	1.0994	3	5	9
G1T673	Q99653	CHP1	Calcineurin like EF-hand protein 1	Calcineurin B homologous protein 1	98	2	1.0994	2	4	17
G1SUD2	Q6UW02	CYP20A1	Uncharacterized protein	Cytochrome P450 20A1	88	3	1.0985	18	26	46
G1TE61	O60684	KPNA6	Importin subunit alpha	Importin subunit alpha-7	99	2	1.0985	10	21	34
G1SEX6	Q9H2U1	DHX36	Uncharacterized protein	ATP-dependent DNA/RNA helicase DHX36	94	3	1.0985	3	3	5
G1ST95	B3KS98	EIF3H	Eukaryotic translation initiation factor 3 subunit H	Eukaryotic translation initiation factor 3 subunit H	98	2	1.0957	8	15	37
G1SGR9	A0A494C1K3	GTF2I	General transcription factor IIi	General transcription factor II-I	97	2	1.0957	4	6	9
	E9PAV3-2	NACA		Isoform skNAC-2 of Nascent polypeptide-associated complex subunit alpha, muscle-specific form		4	1.0948	6	32	11
G1T5J9	O75306	NDUFS2	Complex1_49kDa domain-containing protein	NADH dehydrogenase [ubiquinone] iron-sulfur protein 2, mitochondrial	94	2	1.0948	6	10	23
G1TN89	P98160	HSPG2	Heparan sulfate proteoglycan 2	Basement membrane-specific heparan sulfate proteoglycan core protein	90	2	1.0939	30	52	10
G1T0T5	F5GYF7	COPS7A	PCI domain-containing protein	COP9 signalosome complex subunit 7a (Fragment)	100	2	1.0939	2	4	10
G1SDM6	O75165	DNAJC13	J domain-containing protein	DnaJ homolog subfamily C member 13	98	2	1.0930	27	35	17
G1SNQ9	A0A0A0MTJ9	NCEH1	Uncharacterized protein	Neutral cholesterol ester hydrolase 1	88	3	1.0930	9	10	32
	A6XGL3	PRSS1		Protease serine 1		4	1.0930	2	11	8
G1SYS5	A0A1B0GW77	ALDH7A1	Aldedh domain-containing protein	Alpha-aminoadipic semialdehyde dehydrogenase	93	2	1.0921	9	32	30

G1SWS6	A0A3B3IRN5	FMOD	Fibromodulin	Fibromodulin	92	2	1.0921	2	2	7
G1SVV6	Q02750	MAP2K1	Dual-specificity mitogen-activated protein kinase kinase 1	Dual specificity mitogen-activated protein kinase kinase 1	94	2	1.0911	5	11	21
G1SPZ2	Q13107	USP4	Ubiquitin carboxyl-terminal hydrolase	Ubiquitin carboxyl-terminal hydrolase 4	89	2	1.0911	3	8	7
G1SZ03	P55884	EIF3B	Eukaryotic translation initiation factor 3 subunit B	Eukaryotic translation initiation factor 3 subunit B	98	2	1.0902	24	9	41
G1T5V3	P61106	RAB14	Uncharacterized protein	Ras-related protein Rab-14	100	3	1.0902	12	30	73
G1T7L7		KDELC2	CAP10 domain-containing protein			1	1.0902	10	15	25
G1SRB1	Q16134	ETFDH	4Fe-4S ferredoxin-type domain-containing protein	Electron transfer flavoprotein-ubiquinone oxidoreductase, mitochondrial	94	2	1.0902	9	12	22
G1T3M5	O43324	EEF1E1	GST C-terminal domain-containing protein	Eukaryotic translation elongation factor 1 epsilon-1	95	2	1.0902	6	8	44
G1TD98		GSR	Glutathione reductase			1	1.0902	2	4	7
G1SQB1	P22033	MMUT	B12-binding domain-containing protein	Methylmalonyl-CoA mutase, mitochondrial	96	2	1.0893	2	2	4
	Q969V3	NCLN		Nicalin		4	1.0884	7	10	18
G1SL07	Q9NUP9	LIN7C	Protein lin-7 homolog	Protein lin-7 homolog C	99	2	1.0884	5	4	29
G1STE3	A0A0A0MQX8	MBNL1	Uncharacterized protein	Muscleblind-like protein 1	100	3	1.0884	3	4	7
P06813	A0A075B7C0	CAPNS1	Calpain small subunit 1	Calpain small subunit 1 (Fragment)	96	2	1.0875	10	21	69
G1SYI3	A0A087X1B2	USP39	Uncharacterized protein	U4/U6.U5 tri-snRNP-associated protein 2	99	3	1.0875	5	7	14
G1SQ70	P01023	A2M	Uncharacterized protein	Alpha-2-macroglobulin	78	3	1.0875	4	3	3
G1T2C3	O75534	CSDE1	Uncharacterized protein	Cold shock domain-containing protein E1	99	3	1.0865	15	20	21
G1SDA8	P25786	PSMA1	Proteasome endopeptidase complex	Proteasome subunit alpha type-1	100	2	1.0865	10	22	49
G1T2N2		LTN1	RING-type domain-containing protein			1	1.0865	4	3	4
B7NZS0		MYADM	Myeloid-associated differentiation marker (Predicted)			1	1.0865	2	8	10
G1SFH8		PDCL3	Phosducin domain-containing protein			1	1.0865	2	3	11
G1SN37	O43172	PRPF4	WD_REPEATS_REGION domain-containing protein	U4/U6 small nuclear ribonucleoprotein Prp4	99	2	1.0856	3	4	9
P15253	P27797	CALR	Calreticulin	Calreticulin	96	2	1.0847	21	568	67
G1TVN1	I3L4X2	ABCC1	ATP binding cassette subfamily C member 1	Multidrug resistance-associated protein 1 (Fragment)	91	2	1.0847	16	39	16
G1SIJ8	Q8TEX9	IPO4	Importin N-terminal domain-containing protein	Importin-4	89	2	1.0838	6	9	10
G1T2F5	Q86X52	CHSY1	Hexosyltransferase	Chondroitin sulfate synthase 1	93	2	1.0819	3	2	7
Q28647		PPP2R5B	Serine/threonine-protein phosphatase 2A 56 kDa regulatory subunit beta isoform			1	1.0819	2	2	7
G1SV51	Q08378	GOLGA3	Golgin A3	Golgin subfamily A member 3	85	2	1.0810	12	16	16
G1T373	B1ALA9	PRPS1	Pribosyltran_N domain-containing protein	Ribose-phosphate pyrophosphokinase 1	88	2	1.0810	4	13	19
G1SN85	A0A0D9SEN1	FAP	Uncharacterized protein	Prolyl endopeptidase FAP	95	3	1.0801	27	87	40
G1T6M2		DHRS7B	Dehydrogenase/reductase 7B			1	1.0801	6	11	22
G1SE01	A0A0A0MSI8	EXOC5	Exocyst complex component 5	Exocyst complex component 5	98	2	1.0801	4	4	7
G1TEU8		TBCC	C-CAP/cofactor C-like domain-containing protein			1	1.0801	2	2	7
G1T3E6	A0A024R442	DNPEP	Uncharacterized protein	Aspartyl aminopeptidase	91	3	1.0792	9	11	30
G1SDH8	Q5T9L3	WLS	Uncharacterized protein	Protein wntless homolog	98	3	1.0792	5	7	12
G1U6X6	P55795	HNRNPH2	Uncharacterized protein	Heterogeneous nuclear ribonucleoprotein H2	100	3	1.0782	10	11	34
G1T0F6	Q9UHB9	SRP68	Signal recognition particle subunit SRP68	Signal recognition particle subunit SRP68	95	2	1.0773	9	16	18
G1T6L7	F8WAR4	CHCHD3	MICOS complex subunit	MICOS complex subunit	78	2	1.0773	3	4	9
G1U9T1	Q99832	CCT7	T-complex protein 1 subunit eta	T-complex protein 1 subunit eta	97	2	1.0764	26	9	60
G1TJV3	Q9H7Z7	PTGES2	Prostaglandin E synthase 2	Prostaglandin E synthase 2	79	2	1.0764	4	3	19
G1SJK0		APOOL	MICOS complex subunit			1	1.0764	3	9	23
G1SVV2	Q9NZM1	MYOF	Uncharacterized protein	Myoferlin	94	3	1.0755	68	126	44
G1SWY6	Q15075	EEA1	Early endosome antigen 1	Early endosome antigen 1	87	2	1.0755	32	21	28
G1SRA9	A0A0A0MTH3	ILK	Uncharacterized protein	Integrin-linked protein kinase	93	3	1.0755	11	17	27
G1THT8	J3QT54	CPSF7	Cleavage and polyadenylation specific factor 7	Cleavage and polyadenylation-specificity factor subunit 7 (Fragment)	95	2	1.0755	4	3	16
	O95747	OXSR1		Serine/threonine-protein kinase OSR1		4	1.0755	4	6	12
G1TJA8		FNBP1	Formin binding protein 1			1	1.0755	3	2	4
G1SVA3	O00231	PSMD11	PCI domain-containing protein	26S proteasome non-ATPase regulatory subunit 11	100	2	1.0745	17	37	56
G1SL03	Q9BY32	ITPA	Inosine triphosphate pyrophosphatase	Inosine triphosphate pyrophosphatase	92	2	1.0745	4	2	42
G1T4D2		ACOX1	Acyl-coenzyme A oxidase			1	1.0736	12	7	27
G1T7D1		HIST1H1C	H15 domain-containing protein			1	1.0736	10	23	33
G1TNU3		STX16	t-SNARE coiled-coil homology domain-containing protein			1	1.0736	5	4	23
G1SK33	P05556	ITGB1	Integrin beta	Integrin beta-1	94	2	1.0727	17	51	26
G1T3S1	A0A087X2I1	PSMC6	AAA domain-containing protein	26S proteasome regulatory subunit 10B	100	2	1.0718	12	25	42
G1T1U7	A0A494C1T2	MTHFD1	Uncharacterized protein	C-1-tetrahydrofolate synthase, cytoplasmic (Fragment)	90	3	1.0699	14	21	20
G1SQ93	Q9UKV8	AGO2	Protein argonaute-2	Protein argonaute-2	98	2	1.0699	3	3	6
G1ST56	Q96P70	IPO9	Importin N-terminal domain-containing protein	Importin-9	99	2	1.0690	6	11	15

G1T103	F8VQQ3	C12orf10	Uncharacterized protein	UPF0160 protein MYG1, mitochondrial	77	3	1.0681	4	7	16
A0A494BI13		NAAA	N-acylethanolamine-hydrolyzing acid amidase			1	1.0681	2	2	8
G1SHZ8	P50990	CCT8	Uncharacterized protein	T-complex protein 1 subunit theta	97	3	1.0663	33	72	71
G1SG68	O00629	KPNA4	Importin subunit alpha	Importin subunit alpha-3	99	2	1.0653	7	19	24
G1SGN0	Q15042	RAB3GAP1	Uncharacterized protein	Rab3 GTPase-activating protein catalytic subunit	94	3	1.0644	10	8	17
G1SG42	Q52LJ0	FAM98B	Uncharacterized protein	Protein FAM98B	94	3	1.0644	9	8	34
G1T7T2	Q15424	SAFB	Scaffold attachment factor B	Scaffold attachment factor B1	86	2	1.0644	6	6	14
G1SJE4	Q92556	ELMO1	ELMO domain-containing protein	Engulfment and cell motility protein 1	97	2	1.0635	6	9	13
G1T0Z8	Q99471	PFDN5	Uncharacterized protein	Prefoldin subunit 5	99	3	1.0635	4	5	44
G1SSV4	A0A087WT80	PLCB1	1-phosphatidylinositol 4,5-bisphosphate phosphodiesterase	1-phosphatidylinositol 4,5-bisphosphate phosphodiesterase	97	2	1.0635	3	3	5
	F5H039	GPHN		Molybdopterin molybdenumtransferase		4	1.0635	2	3	5
G1TCK9	A0A0A0MSX9	IARS	Uncharacterized protein	Isoleucine--tRNA ligase, cytoplasmic	94	3	1.0626	33	67	34
G1SI95	O00291	HIP1	Uncharacterized protein	Huntingtin-interacting protein 1	91	3	1.0626	15	16	24
G1SRE8		XRCC5	Ku domain-containing protein			1	1.0626	7	14	20
G1SFK3		RPA1	Replication protein A subunit			1	1.0626	2	2	7
	A6NM71	WDR45		PRA1 family protein		4	1.0616	3	5	9
G1SF36	Q13423	NNT	Uncharacterized protein	NAD(P) transhydrogenase, mitochondrial	97	3	1.0607	25	60	28
G1SQY8	Q9Y266	NUDC	CS domain-containing protein	Nuclear migration protein nudC	97	2	1.0607	10	12	31
G1TNL6	P50479	PDLIM4	Uncharacterized protein	PDZ and LIM domain protein 4	86	3	1.0598	7	9	33
G1TCE2	H7C0R7	CYB5R1	NADH-cytochrome b5 reductase	NADH-cytochrome b5 reductase 1 (Fragment)	77	2	1.0598	6	10	28
G1U5M4	J3QQS9	SLC16A3	Solute carrier family 16 member 3	Monocarboxylate transporter 4 (Fragment)	92	2	1.0589	3	4	6
G1T918	P28070	PSMB4	Proteasome subunit beta	Proteasome subunit beta type-4	95	2	1.0580	8	37	49
G1T3Z6	P31323	PRKAR2B	Uncharacterized protein	cAMP-dependent protein kinase type II-beta regulatory subunit	97	3	1.0580	7	8	27
G1SVY6	Q9BTX1	NDC1	Uncharacterized protein	Nucleoporin NDC1	88	3	1.0580	2	2	5
G1SW24	P49588	AARS	AA_TRNA_LIGASE_II_ALA domain-containing protein	Alanine--tRNA ligase, cytoplasmic	93	2	1.0570	33	26	47
G1SM15	A0A087WY71	AP2M1	MHD domain-containing protein	AP-2 complex subunit mu	100	2	1.0570	15	27	42
G1SPB6	E7EPM6	ACSL1	AMP-binding domain-containing protein	Long-chain-fatty-acid--CoA ligase 1	80	2	1.0570	5	11	23
G1TIM0	G3V155	TMX2	Thioredoxin domain-containing protein	Thioredoxin domain containing 14, isoform CRA_a	94	2	1.0570	4	4	13
G1SE46	O95159	ZFPL1	Zinc finger protein like 1	Zinc finger protein-like 1	92	2	1.0561	4	4	15
G1SSX5	P56192	MARS	Uncharacterized protein	Methionine--tRNA ligase, cytoplasmic	93	3	1.0543	14	29	21
B7NZD2	Q5VWC4	PSMD4	Proteasome 26S subunit, non-ATPase, 4 (Predicted)	26S proteasome non-ATPase regulatory subunit 4	99	2	1.0543	8	18	27
G1T2R3		EFL1	Tr-type G domain-containing protein			1	1.0543	3	3	6
G1TM00	O75436	VPS26A	VPS26, retromer complex component A	Vacuolar protein sorting-associated protein 26A	99	2	1.0543	2	2	10
G1SNY3	Q68E01	INTS3	Uncharacterized protein	Integrator complex subunit 3	99	3	1.0534	6	6	12
G1T617	P49756	RBM25	Uncharacterized protein	RNA-binding protein 25	99	3	1.0534	5	4	9
G1T239	F5GYQ1	ATP6V0D1	V-type proton ATPase subunit	V-type proton ATPase subunit	90	2	1.0524	8	12	31
G1SZG3	Q8NEW0	SLC30A7	Uncharacterized protein	Zinc transporter 7	97	3	1.0524	4	3	18
G1SKF5		ERBIN	Erbb2 interacting protein			1	1.0515	5	6	9
G1T0R4	Q96JC1	VPS39	CNH domain-containing protein	Vam6/Vps39-like protein	97	2	1.0515	3	2	5
G1SDN8	Q92791	P3H4	Uncharacterized protein	Endoplasmic reticulum protein SC65	88	3	1.0506	5	9	18
G1U797	P51114	FXR1	Uncharacterized protein	Fragile X mental retardation syndrome-related protein 1	91	3	1.0497	12	10	19
G1TLQ8	R4GNH3	PSMC3	AAA domain-containing protein	26S proteasome regulatory subunit 6A	100	2	1.0487	21	50	65
G1TKE0	Q9P2X0	DPM3	Dolichol-phosphate mannosyltransferase subunit 3	Dolichol-phosphate mannosyltransferase subunit 3	95	2	1.0487	2	2	24
G1SEM0	Q96AC1	FERMT2	PH domain-containing protein	Fermitin family homolog 2	100	2	1.0469	22	41	47
G1SZ14	P25788	PSMA3	Proteasome endopeptidase complex	Proteasome subunit alpha type-3	99	2	1.0469	8	20	26
G1SQT2	Q6P2E9	EDC4	WD_REPEATS_REGION domain-containing protein	Enhancer of mRNA-decapping protein 4	96	2	1.0460	3	4	5
	P40429	RPL13A		60S ribosomal protein L13a		4	1.0451	9	12	37
G1TAF8	P31948	STIP1	Uncharacterized protein	Stress-induced-phosphoprotein 1	96	3	1.0441	26	45	52
G1SMI6	Q13162	PRDX4	Thioredoxin domain-containing protein	Peroxiredoxin-4	96	2	1.0432	10	18	41
G1T840	Q92598	HSPH1	Uncharacterized protein	Heat shock protein 105 kDa	96	3	1.0423	17	23	28
G1SYF9	Q9Y613	FHOD1	Uncharacterized protein	FH1/FH2 domain-containing protein 1	87	3	1.0423	5	6	9
G1SI54		ILVBL	IlvB acetolactate synthase like			1	1.0423	5	6	19
G1TIS2		TIMM17B	Mitochondrial import inner membrane translocase subunit TIM17			1	1.0423	2	3	22
G1TRZ2		LAMP1	Lysosomal associated membrane protein 1			1	1.0414	5	18	14
G1T6N8		ALG12	Mannosyltransferase			1	1.0405	2	2	9
G1SWN7	Q4G0N4	NADK2	NAD kinase 2, mitochondrial	NAD kinase 2, mitochondrial	92	2	1.0395	5	5	14
G1SG63	P55011	SLC12A2	Uncharacterized protein	Solute carrier family 12 member 2	97	3	1.0395	3	2	4

G1U354	R4GMR5	PSMD8	PCI domain-containing protein	26S proteasome non-ATPase regulatory subunit 8	97	2	1.0386	9	16	33
G1SZR8	P51858	HDGF	Heparin binding growth factor	Hepatoma-derived growth factor	96	2	1.0386	5	6	22
G1SVK5	P26447	S100A4	Protein S100	Protein S100-A4	98	2	1.0386	4	4	36
Q01971	P61019	RAB2A	Ras-related protein Rab-2A	Ras-related protein Rab-2A	100	2	1.0377	12	45	67
G1T810	A0A0A0MTN0	CUL2	CULLIN_2 domain-containing protein	Cullin-2	99	2	1.0377	5	6	10
G1T419	Q92621	NUP205	Uncharacterized protein	Nuclear pore complex protein Nup205	96	3	1.0368	24	14	19
G1TS36	B8ZZG1	MPP6	Uncharacterized protein	Membrane protein, palmitoylated 6 (MAGUK p55 subfamily member 6), isoform CRA_a	96	3	1.0358	5	4	10
A0A0A0MQP7	P62736	ACTA2	Actin, aortic smooth muscle	Actin, aortic smooth muscle	100	2	1.0349	30	###	71
G1TZC9	A0A087WXU3	ESYT2	Extended synaptotagmin 2	Extended synaptotagmin-2	92	2	1.0349	13	7	25
G1T9L2	Q86VN1	VPS36	GLUE N-terminal domain-containing protein	Vacuolar protein-sorting-associated protein 36	97	2	1.0340	4	5	18
G1T4N5	P42345	MTOR	Serine/threonine-protein kinase mTOR	Serine/threonine-protein kinase mTOR	99	2	1.0331	16	17	11
	A0A087X0W8	RELA		Transcription factor p65		4	1.0331	2	2	7
G1SU97	H0YD97	PDHX	Dihydrolipoamide acetyltransferase component of pyruvate dehydrogenase complex	Pyruvate dehydrogenase protein X component, mitochondrial (Fragment)	89	2	1.0322	4	7	10
G1SLD5	A0A087X054	HYOU1	Hypoxia up-regulated 1	Hypoxia up-regulated protein 1	87	2	1.0312	29	52	38
G1T242		IKBIP	IKBKB interacting protein			1	1.0312	15	24	39
G1TQR2	P24534	EEF1B2	Uncharacterized protein	Elongation factor 1-beta	98	3	1.0312	10	24	65
G1TCB7	Q5LJA9	UCHL5	Ubiquitin carboxyl-terminal hydrolase	Ubiquitin carboxyl-terminal hydrolase (Fragment)	91	2	1.0312	7	7	17
G1SPJ7		UTP4	WD_REPEATS_REGION domain-containing protein			1	1.0312	2	2	5
G1T4H3	Q15084	PDIA6	Uncharacterized protein	Protein disulfide-isomerase A6	93	3	1.0294	17	116	52
G1SNI4	O94760	DDAH1	Uncharacterized protein	N(G),N(G)-dimethylarginine dimethylaminohydrolase 1	96	3	1.0294	6	6	24
G1TWK1	E9PGZ4	SACM1L	SAC domain-containing protein	Phosphatidylinositide phosphatase SAC1	98	2	1.0285	14	26	34
G1TEG1	O95470	SGPL1	Uncharacterized protein	Sphingosine-1-phosphate lyase 1	90	3	1.0285	9	17	22
G1SJF1	A0A499FIZ0	WDR26	Uncharacterized protein	WD repeat-containing protein 26	99	3	1.0285	7	7	18
G1SR63		PREB	WD_REPEATS_REGION domain-containing protein			1	1.0285	3	7	15
G1TWP4	A0A140T936	VARS	GST C-terminal domain-containing protein	Valine--tRNA ligase (Fragment)	92	2	1.0275	18	27	18
G1SF32	O94826	TOMM70	TPR_REGION domain-containing protein	Mitochondrial import receptor subunit TOM70	97	2	1.0275	16	40	24
G1SCY7	Q8N766	EMC1	EMC1_C domain-containing protein	ER membrane protein complex subunit 1	96	2	1.0275	15	24	23
G1T3S4		MECR	PKS_ER domain-containing protein			1	1.0275	5	9	34
G1TA37	O75531	BANF1	Uncharacterized protein	Barrier-to-autointegration factor	100	3	1.0275	4	12	57
G1SEI8		NUP214	Nup214_FG domain-containing protein			1	1.0275	4	9	4
	O43290	SART1		U4/U6.U5 tri-snRNP-associated protein 1		4	1.0275	2	3	5
	J3KN75	TBC1D8B		TBC1 domain family member 8B		4	1.0275	2	2	3
G1SFC1	H0Y6I0	GOLGA4	Golgin A4	Golgin subfamily A member 4 (Fragment)	77	2	1.0266	12	18	7
G1TH33	P38117	ETFB	Electron transfer flavoprotein subunit beta	Electron transfer flavoprotein subunit beta	95	2	1.0266	10	7	47
G1SWR1	O43818	RRP9	WD_REPEATS_REGION domain-containing protein	U3 small nucleolar RNA-interacting protein 2	94	2	1.0266	5	6	13
G1T2Z8	B4DUC8	MTAP	S-methyl-5~-thioadenosine phosphorylase	S-methyl-5~-thioadenosine phosphorylase	99	2	1.0266	4	6	27
G1TPC8	P08962	CD63	Tetraspanin	CD63 antigen	78	2	1.0257	4	9	15
G1TKG2		ISOC2	Isochorismatase domain containing 2			1	1.0257	4	7	43
G1T196	Q13033	STRN3	WD_REPEATS_REGION domain-containing protein	Striatin-3	92	2	1.0257	4	4	12
G1SNM8	Q8N5M9	JAGN1	Uncharacterized protein	Protein jagunal homolog 1	96	3	1.0257	3	4	17
G1TBK0		ANKMY2	Ankyrin repeat and MYND domain containing 2			1	1.0257	2	3	5
G1U724	Q9UBE0	SAE1	SUMO1 activating enzyme subunit 1	SUMO-activating enzyme subunit 1	88	2	1.0248	8	13	26
G1SMW3	Q92615	LARP4B	La ribonucleoprotein domain family member 4B	La-related protein 4B	88	2	1.0248	3	3	8
G1TGY9		VPS11	VPS11, CORVET/HOPS core subunit			1	1.0248	2	2	3
G1SZ91		FABP5	Lipocln_cytosolic_FA-bd_dom domain-containing protein			1	1.0229	4	10	37
G1T887		GPR107	G protein-coupled receptor 107			1	1.0229	2	3	9
G1T193		UGT3A2	UDP-glucuronosyltransferase			1	1.0229	2	2	6
G1SVF2	A0A087WW66	PSMD1	26S proteasome non-ATPase regulatory subunit 1	26S proteasome non-ATPase regulatory subunit 1	99	2	1.0220	29	7	44
G1TGF1	Q15185	PTGES3	Prostaglandin E synthase 3	Prostaglandin E synthase 3	100	2	1.0220	5	8	52
	Q9NZN4	EHD2		EH domain-containing protein 2		4	1.0202	11	16	27
G1SSV2	Q9UL01	DSE	DUF4962 domain-containing protein	Dermatan-sulfate epimerase	96	2	1.0202	8	8	15
G1SNI2	Q12841	FSTL1	Kazal-like domain-containing protein	Follistatin-related protein 1	94	2	1.0202	8	23	32
G1SJ43	Q9H3S7	PTPN23	Uncharacterized protein	Tyrosine-protein phosphatase non-receptor type 23	91	3	1.0202	7	6	5
G1T3A2	Q9Y3A6	TMED5	GOLD domain-containing protein	Transmembrane emp24 domain-containing protein 5	97	2	1.0202	5	12	24
G1TI97	Q13769	THOC5	Uncharacterized protein	THO complex subunit 5 homolog	97	3	1.0202	3	5	8
G1SLQ3	P15559	NQO1	Flavodoxin_2 domain-containing protein	NAD(P)H dehydrogenase [quinone] 1	89	2	1.0193	10	19	48
G1SW36	H0YDT8	EMC7	DUF2012 domain-containing protein	ER membrane protein complex subunit 7 (Fragment)	79	2	1.0193	4	4	24

G1TX94	A0A0R4J2E8	MATR3	Uncharacterized protein	Matrin-3	99	3	1.0183	17	30	28
G1SU80	O60462	NRP2	Neuropilin	Neuropilin-2	95	2	1.0183	17	21	25
G1T1G2	A0A1B0GWA2	AGPS	Alkylglycerone-phosphate synthase	Alkylglycerone-phosphate synthase (Fragment)	96	2	1.0183	7	13	17
G1SU71	P20618	PSMB1	Proteasome subunit beta	Proteasome subunit beta type-1	94	2	1.0156	8	12	42
	A0A0A0MTU3	TMEM259		Membralin		4	1.0156	2	2	8
G1SFE0	O00487	PSMD14	MPN domain-containing protein	26S proteasome non-ATPase regulatory subunit 14	100	2	1.0146	7	14	45
G1SJH8	K7EP90	RBM42	RRM domain-containing protein	RNA-binding protein 42	97	2	1.0146	2	2	8
G1SEN5	Q8IZ07	ANKRD13A	Ankyrin repeat domain 13A	Ankyrin repeat domain-containing protein 13A	94	2	1.0128	2	3	5
G1SYC3		FBXL8	F-box domain-containing protein			1	1.0119	2	3	10
G1SKK5		PEX6	Peroxisomal biogenesis factor 6			1	1.0119	2	3	6
G1T5H7		TMEM132B	Transmembrane protein 132B			1	1.0119	2	2	4
G1TYV6	Q9HD20	ATP13A1	Cation-transporting ATPase	Manganese-transporting ATPase 13A1	95	2	1.0110	12	16	15
G1T0N5	Q00577	PURA	Purine rich element binding protein A	Transcriptional activator protein Pur-alpha	91	2	1.0110	7	13	40
G1T159	P62070	RRAS2	Uncharacterized protein	Ras-related protein R-Ras2	97	3	1.0091	6	7	33
G1SHJ3		KDELC1	CAP10 domain-containing protein			1	1.0091	5	8	14
G1U1E6		GCSH	Glycine cleavage system H protein			1	1.0091	2	4	33
G1SJW8	H3BND8	USP7	Uncharacterized protein	Ubiquitin carboxyl-terminal hydrolase (Fragment)	99	3	1.0082	9	8	16
G1U864		TRRAP	Transformation/transcription domain associated protein			1	1.0082	3	3	1
G1SVM1	Q14764	MVP	Uncharacterized protein	Major vault protein	91	3	1.0073	35	111	57
G1SS70	P61247	RPS3A	40S ribosomal protein S3a	40S ribosomal protein S3a	100	2	1.0073	15	91	55
G1SZQ7	Q7L1Q6	BZW1	W2 domain-containing protein	Basic leucine zipper and W2 domain-containing protein 1	100	2	1.0073	12	21	39
G1TEY6	O00178	GTPBP1	Tr-type G domain-containing protein	GTP-binding protein 1	94	2	1.0073	5	8	12
G1TEN1	H7C5S0	ACTL6A	Uncharacterized protein	Actin-like protein 6A (Fragment)	99	3	1.0073	4	5	15
G1T4N4	A0A0B4J210	LARP1	HTH La-type RNA-binding domain-containing protein	La-related protein 1 (Fragment)	93	2	1.0064	5	10	8
G1SPH7	Q9P2I0	CPSF2	Cleavage and polyadenylation specificity factor subunit 2	Cleavage and polyadenylation specificity factor subunit 2	99	2	1.0064	4	6	6
G1SDL9	Q9UEU0	VTI1B	t-SNARE coiled-coil homology domain-containing protein	Vesicle transport through interaction with t-SNAREs homolog 1B	93	2	1.0064	4	5	24
G1SDA4	P53618	COPB1	Coatomer subunit beta	Coatomer subunit beta	99	2	1.0054	37	202	54
G1TFZ7	O15355	PPM1G	PPM-type phosphatase domain-containing protein	Protein phosphatase 1G	97	2	1.0054	3	5	10
	A0A087X1G7	SELENOF		Selenoprotein F		4	1.0045	3	14	22
G1TK72	F8W8H5	RAB24	Uncharacterized protein	Ras-related protein Rab-24	98	3	1.0045	2	2	13
U3KNL7	Q06210	GFPT1	Uncharacterized protein	Glutamine--fructose-6-phosphate aminotransferase [isomerizing] 1	95	3	1.0036	15	4	40
	E9PB61	ALYREF		THO complex subunit 4		4	1.0027	5	51	25
G1T798	Q12846	STX4	t-SNARE coiled-coil homology domain-containing protein	Syntaxin-4	96	2	1.0027	4	7	27
G1SI94		CRLF3	Cytokine receptor like factor 3			1	1.0027	2	2	14
G1TKY3	P17655	CAPN2	Calpain-2 catalytic subunit	Calpain-2 catalytic subunit	94	2	1.0008	22	42	49
G1T4K8	P32189	GK	Uncharacterized protein	Glycerol kinase	97	3	1.0008	11	19	23
G1SQ03	Q8WWI5	SLC44A1	Solute carrier family 44 member 1	Choline transporter-like protein 1	93	2	1.0008	3	4	7
G1SX32	O00743	PPP6C	Serine/threonine-protein phosphatase	Serine/threonine-protein phosphatase 6 catalytic subunit	100	2	1.0008	2	2	16
G1TGH1		D2HGDH	D-2-hydroxyglutarate dehydrogenase			1	0.9999	4	7	13
G1TFB5	Q9NP72	RAB18	Uncharacterized protein	Ras-related protein Rab-18	98	3	0.9990	8	20	49
G1SIL2	Q9UNW1	MINPP1	Multiple inositol-polyphosphate phosphatase 1	Multiple inositol polyphosphate phosphatase 1	87	2	0.9990	4	6	16
	Q9Y224	RTRAF		RNA transcription, translation and transport factor protein		4	0.9981	10	43	50
G1U207	Q86Y82	STX12	t-SNARE coiled-coil homology domain-containing protein	Syntaxin-12	96	2	0.9981	10	19	55
G1TE08	H7C3P6	NUP98	Peptidase S59 domain-containing protein	Nuclear pore complex protein Nup98-Nup96 (Fragment)	90	2	0.9981	9	12	7
U3KM64		CLTA	Clathrin light chain			1	0.9981	7	27	28
G1T7T0	Q9Y2G5	POFUT2	Protein O-fucosyltransferase 2	GDP-fucose protein O-fucosyltransferase 2	84	2	0.9981	3	4	13
G1SU75	O95140	MFN2	Mitofusin 2	Mitofusin-2	92	2	0.9971	10	14	18
G1SUP8	P67812	SEC11A	Signal peptidase complex catalytic subunit SEC11	Signal peptidase complex catalytic subunit SEC11A	100	2	0.9971	6	9	40
G1SF00	Q9BSJ2	TUBGCP2	Gamma-tubulin complex component	Gamma-tubulin complex component 2	90	2	0.9971	5	5	7
G1T044	A0A3B3IUC4	GLA	Alpha-galactosidase	Alpha-galactosidase	75	2	0.9971	3	3	7
G1TNT7		DDX27	DEAD-box helicase 27			1	0.9962	2	2	3
G1T5Y2	Q9NUQ9	FAM49B	Uncharacterized protein	Protein FAM49B	100	3	0.9953	3	4	16
G1SUQ4		DNTTIP2	Fcf2 domain-containing protein			1	0.9953	2	3	4
G1TA15	Q9P2J5	LARS	Uncharacterized protein	Leucine--tRNA ligase, cytoplasmic	95	3	0.9944	25	49	29
G1T7Y5	H0Y368	DPM1	Dolichol-phosphate mannosyltransferase subunit 1	Dolichol-phosphate mannosyltransferase subunit 1 (Fragment)	83	2	0.9944	5	5	23
G1U6X4	F8VQZ7	METAP2	Methionine aminopeptidase 2	Methionine aminopeptidase 2	94	2	0.9944	5	21	17
G1SV75	H3BNK3	NDUFAB1	Acyl carrier protein	Acyl carrier protein (Fragment)	63	2	0.9944	2	3	10

G1TDJ9	F6Y5H0	RBMS1	RNA binding motif single stranded interacting protein 1	RNA-binding motif, single-stranded-interacting protein 1	97	2	0.9944	2	3	14
Q9TT13	Q9Y277	VDAC3	Voltage-dependent anion-selective channel protein 3	Voltage-dependent anion-selective channel protein 3	98	2	0.9935	12	29	54
G1T1F0	A0A2R8Y811	RPS14	Uncharacterized protein	40S ribosomal protein S14 (Fragment)	100	3	0.9935	5	10	37
G1SQM7	P53999	SUB1	PC4 domain-containing protein	Activated RNA polymerase II transcriptional coactivator p15	97	2	0.9935	4	7	22
G1T8H5		POLR2C	RPOLD domain-containing protein			1	0.9935	2	3	11
G1SL02	P63010	AP2B1	AP complex subunit beta	AP-2 complex subunit beta	98	2	0.9925	41	63	57
G1TNW8	Q9Y512	SAMM50	SAMM50 sorting and assembly machinery component	Sorting and assembly machinery component 50 homolog	96	2	0.9925	9	9	35
G1T6E6		NOC3L	Nucleolar complex protein 3 homolog			1	0.9916	4	6	9
P62493	P62491	RAB11A	Ras-related protein Rab-11A	Ras-related protein Rab-11A	100	2	0.9907	11	37	59
G1STS3	Q96T76	MMS19	Uncharacterized protein	MMS19 nucleotide excision repair protein homolog	93	3	0.9907	8	10	16
G1SJR4	Q15363	TMED2	Transmembrane p24 trafficking protein 2	Transmembrane emp24 domain-containing protein 2	99	2	0.9907	8	29	64
G1T093	Q9NZJ4-2	SACS	Uncharacterized protein	Isoform 2 of Sacsin	96	3	0.9907	5	4	2
G1SKG9	A0A087X2H1	HECTD1	Uncharacterized protein	E3 ubiquitin-protein ligase HECTD1	99	3	0.9898	7	11	4
G1TE13	P84095	RHOG	Uncharacterized protein	Rho-related GTP-binding protein RhoG	99	3	0.9898	6	9	41
	O15031	PLXNB2		Plexin-B2		4	0.9888	7	7	6
G1SH42	Q5VSL9	STRIP1	Uncharacterized protein	Striatin-interacting protein 1	98	3	0.9888	4	4	12
G1SJ37	F8W9S7	GAPVD1	Uncharacterized protein	GTPase-activating protein and VPS9 domain-containing protein 1	95	3	0.9888	3	4	4
G1TRV7		QPCTL	Glutaminyl-peptide cyclotransferase like			1	0.9879	6	9	32
G1SGM3	A0A0J9YXF2	PON2	Uncharacterized protein	Paraoxonase 2, isoform CRA_a	93	3	0.9870	12	26	58
G1T4M1		LAMC1	Laminin subunit gamma 1			1	0.9870	4	9	9
G1TVT0	P19338	NCL	Nucleolin	Nucleolin	83	2	0.9861	23	23	34
G1TBU9	A0A0B4J2A4	ACAA2	Uncharacterized protein	3-ketoacyl-CoA thiolase, mitochondrial	89	3	0.9861	15	57	64
G1T4T7	Q9Y2A7	NCKAP1	Uncharacterized protein	Nck-associated protein 1	100	3	0.9861	15	24	21
G1SGM2	O14744	PRMT5	Protein arginine N-methyltransferase 5	Protein arginine N-methyltransferase 5	98	2	0.9861	6	9	19
	A0A494C0A9	CBFB		Core-binding factor subunit beta		4	0.9861	4	5	31
G1T6D4	O00232	PSMD12	PCI domain-containing protein	26S proteasome non-ATPase regulatory subunit 12	99	2	0.9852	16	49	48
P43236	P43235	CTSK	Cathepsin K	Cathepsin K	94	2	0.9852	5	13	22
G1SVA1	F2Z2X4	XPO4	CRM1_C domain-containing protein	Exportin-4	99	2	0.9852	5	5	10
G1TED6	P08758	ANXA5	Annexin	Annexin A5	93	2	0.9842	19	53	65
G1TBX7	P11233	RALA	Uncharacterized protein	Ras-related protein Ral-A	100	3	0.9842	8	6	50
	I3L0A0	1EM189-UBE2V1		HCG2044781		4	0.9842	6	2	23
G1SFQ7		TUBG1	Tubulin gamma chain			1	0.9842	5	7	17
G1T0Z6	Q9Y4X5	ARIH1	RBR-type E3 ubiquitin transferase	E3 ubiquitin-protein ligase ARIH1	98	2	0.9842	3	3	10
G1U222	A0A384DVK7	ARHGEF10L	Rho guanine nucleotide exchange factor 10 like	Rho guanine nucleotide exchange factor 10-like protein (Fragment)	93	2	0.9842	2	2	4
G1SQ90	A0A2R8Y5H3	COL4A3BP	Collagen type IV alpha 3 binding protein	Collagen type IV alpha-3-binding protein (Fragment)	96	2	0.9842	2	2	6
G1TW04	Q16643	DBN1	Drebrin 1	Drebrin	63	2	0.9833	12	92	28
G1TE47		COG8	Conserved oligomeric Golgi complex subunit 8			1	0.9824	5	6	12
G1T9V7		SRP19	Signal recognition particle 19			1	0.9815	2	4	26
G1SM82	E9PGC0	RASA1	Uncharacterized protein	Ras GTPase-activating protein 1	96	3	0.9805	8	10	13
G1SF45		SEC24B	SEC24 homolog B, COPII coat complex component			1	0.9805	3	4	4
G1T748	Q5VZU9	TPP2	Uncharacterized protein	Tripeptidyl-peptidase 2	97	3	0.9787	26	35	25
G1SES9	P31939	ATIC	MGS domain-containing protein	Bifunctional purine biosynthesis protein PURH	94	2	0.9787	17	34	44
G1SYV0	P35998	PSMC2	AAA domain-containing protein	26S proteasome regulatory subunit 7	100	2	0.9787	17	38	46
	G3V0I5	NDUFV1		NADH dehydrogenase [ubiquinone] flavoprotein 1, mitochondrial		4	0.9787	6	9	27
G1SK29		MTIF2	Tr-type G domain-containing protein			1	0.9787	3	4	8
G1SL41		GUSB	Beta-glucuronidase			1	0.9778	11	24	30
G1T8S0	Q9C0D5	TANC1	Uncharacterized protein	Protein TANC1	85	3	0.9778	8	8	9
G1SPG6	A6NEM5	PIGK	GPI-anchor transamidase	GPI-anchor transamidase	85	2	0.9778	4	7	22
P40826	P54578	USP14	Ubiquitin carboxyl-terminal hydrolase 14	Ubiquitin carboxyl-terminal hydrolase 14	97	2	0.9769	10	17	29
	Q5T8U5	SURF4		Surfeit 4		4	0.9769	4	76	29
G1SJJ2	Q9NZ32	ACTR10	Uncharacterized protein	Actin-related protein 10	96	3	0.9759	10	24	40
G1SFE9	H0YJG7	AHSA1	Aha1_N domain-containing protein	Activator of 90 kDa heat shock protein ATPase homolog 1 (Fragment)	95	2	0.9759	3	4	14
G1TEM4	Q9UEW8	STK39	Protein kinase domain-containing protein	STE20/SPS1-related proline-alanine-rich protein kinase	95	2	0.9759	2	2	4
G1SZ12	E7EPB3	RPL14	Ribosomal_L14e domain-containing protein	60S ribosomal protein L14	96	2	0.9750	6	12	27
G1TLK9	P49748	ACADVL	Uncharacterized protein	Very long-chain specific acyl-CoA dehydrogenase, mitochondrial	87	3	0.9741	22	61	49
G1SCY4	P52907	CAPZA1	F-actin-capping protein subunit alpha	F-actin-capping protein subunit alpha-1	96	2	0.9732	10	28	58
G1T643	A0A1W2PNX8	UNC45A	Unc-45 myosin chaperone A	Protein unc-45 homolog A	94	2	0.9732	8	14	14

G1SQT0	P60510	PPP4C	Serine/threonine-protein phosphatase	Serine/threonine-protein phosphatase 4 catalytic subunit	100	2	0.9732	3	3	14
G1SXR1		PRELP	LRRNT domain-containing protein			1	0.9723	4	6	17
G1T4J2	F5H228	TRIO	Uncharacterized protein	Triple functional domain protein	99	3	0.9723	4	6	4
G1SZ63	P49189	ALDH9A1	Aldedh domain-containing protein	4-trimethylaminobutyraldehyde dehydrogenase	94	2	0.9713	8	12	29
G1SH09	Q5VIR6	VPS53	VPS53, GARP complex subunit	Vacuolar protein sorting-associated protein 53 homolog	95	2	0.9713	4	4	11
G1TUY5		OCIAD1	OCIA domain-containing protein			1	0.9713	3	3	15
G1SGX3	P22059	OSBP	Oxysterol-binding protein	Oxysterol-binding protein 1	97	2	0.9695	8	12	12
G1TTU6	E7ERH2	SKP1	Uncharacterized protein	S-phase kinase-associated protein 1 (Fragment)	100	3	0.9695	6	56	56
G1SL62	P07355	ANXA2	Annexin	Annexin A2	98	2	0.9686	26	65	71
G1SK52	Q5T6H7	XPNPEP1	Uncharacterized protein	Xaa-Pro aminopeptidase 1	97	3	0.9686	10	12	25
G1SY96	Q9UJS0	SLC25A13	Uncharacterized protein	Calcium-binding mitochondrial carrier protein Aralar2	97	3	0.9676	11	3	28
G1SM64	Q5T985	ITIH2	Uncharacterized protein	Inter-alpha-trypsin inhibitor heavy chain H2	85	3	0.9676	5	5	8
G1SZH8	Q8N1B4	VPS52	Uncharacterized protein	Vacuolar protein sorting-associated protein 52 homolog	99	3	0.9676	4	6	12
G1SVW9		VEZT	Vezatin domain-containing protein			1	0.9676	2	2	4
G1SEW3	P49792	RANBP2	RAN binding protein 2	E3 SUMO-protein ligase RanBP2	84	2	0.9667	17	31	8
G1SGS7	O94874	UFL1	Uncharacterized protein	E3 UFM1-protein ligase 1	94	3	0.9667	16	26	29
	P09497-2	CLTB		Isoform Non-brain of Clathrin light chain B		4	0.9667	5	10	21
G1SET5		CEMIP2	G8 domain-containing protein			1	0.9667	4	4	5
G1TQ31	A0A2R8YD58	CSNK2A1	Casein kinase II subunit alpha	Casein kinase II subunit alpha	97	2	0.9667	4	5	21
	O14818	PSMA7		Proteasome subunit alpha type-7		4	0.9658	9	18	44
G1U7Q6	H7C1D4	TSN	Uncharacterized protein	Translin (Fragment)	99	3	0.9658	3	48	22
G1SXI8		PCID2	PCI domain containing 2			1	0.9658	2	2	6
G1SK48	K7ES02	BLMH	Bleomycin hydrolase	Bleomycin hydrolase (Fragment)	92	2	0.9640	8	13	26
G1T7U4	Q9Y4E8	USP15	Ubiquitin carboxyl-terminal hydrolase	Ubiquitin carboxyl-terminal hydrolase 15	99	2	0.9630	4	4	7
G1U460	Q02252	ALDH6A1	Aldedh domain-containing protein	Methylmalonate-semialdehyde dehydrogenase [acylating], mitochondrial	95	2	0.9621	8	17	26
G1T601		CLYBL	HpcH_HpaI domain-containing protein			1	0.9621	4	7	19
G1SM01		AKAP9	A-kinase anchor protein 9			1	0.9621	3	4	1
G1U8J5		ATP5PF	ATP synthase-coupling factor 6, mitochondrial			1	0.9621	3	3	33
G1SKJ8		TCOF1	LisH domain-containing protein			1	0.9621	2	2	3
G1TB98	Q15293	RCN1	Uncharacterized protein	Reticulocalbin-1	85	3	0.9612	12	10	45
G1SST7	Q96SL4	GPX7	Glutathione peroxidase	Glutathione peroxidase 7	92	2	0.9612	6	9	36
G1T0H0	Q9NX46	ADPRHL2	ADP-ribosylhydrolase like 2	ADP-ribose glycohydrolase ARH3	95	2	0.9603	3	3	12
G1SMK4		UCKL1	Uridine-cytidine kinase 1 like 1			1	0.9603	2	3	6
G1SI98	O60502	OGA	Uncharacterized protein	Protein O-GlcNAcase	99	3	0.9594	3	3	3
G1SER3	Q02978	SLC25A11	Uncharacterized protein	Mitochondrial 2-oxoglutarate/malate carrier protein	97	3	0.9584	8	57	37
G1SIE8	A0A3B3ISG5	IDE	Uncharacterized protein	Insulin-degrading enzyme	96	3	0.9584	7	7	9
A7X8X3		HPRT	Hypoxanthine phosphoribosyltransferase			1	0.9584	6	9	31
G1SNM5	A0A2R8Y566	RELCH	LisH domain-containing protein	RAB11-binding protein RELCH	96	2	0.9584	3	3	5
G1SQN6	J3KN59	BNIP2	CRAL-TRIO domain-containing protein	BCL2/adenovirus E1B 19 kDa protein-interacting protein 2	94	2	0.9584	2	3	11
G1SM24		MDN1	Midasin			1	0.9584	2	2	1
G1SFV1	A0A499FI48	PDIA4	Protein disulfide-isomerase	Protein disulfide-isomerase	91	2	0.9575	29	385	49
G1SZE0	Q96QK1	VPS35	Vacuolar protein sorting-associated protein 35	Vacuolar protein sorting-associated protein 35	100	2	0.9575	22	49	37
G1T9L6		TOR1AIP1	Torsin 1A interacting protein 1			1	0.9575	4	7	9
G1SGD9		ATG7	Ubiquitin-like modifier-activating enzyme ATG7			1	0.9575	2	2	6
G1T5U1		GPN1	GPN-loop GTPase			1	0.9575	2	2	17
G1SNM1	P41250	GARS	Uncharacterized protein	Glycine--tRNA ligase	95	3	0.9566	22	41	39
G1TBR5	B4DR61	SEC61A1	Plug_translocon domain-containing protein	Protein transport protein Sec61 subunit alpha isoform 1	100	2	0.9557	11	79	39
G1SJU2	O60306	AQR	RNA helicase aquarius	RNA helicase aquarius	96	2	0.9557	3	3	4
G1TCS8	P62820	RAB1A	Uncharacterized protein	Ras-related protein Rab-1A	100	3	0.9547	13	152	74
G1SH26	F6TLX2	GLOD4	Glyoxalase domain containing 4	Glyoxalase domain-containing protein 4	81	2	0.9547	7	10	23
G1SEU9	Q9UHA4	LAMTOR3	Uncharacterized protein	Ragulator complex protein LAMTOR3	98	3	0.9547	4	6	40
G1TX74	Q6IBS0	TWF2	Uncharacterized protein	Twinfilin-2	95	3	0.9547	4	5	17
G1SX73	E5RGS4	PFDN1	Uncharacterized protein	Prefoldin subunit 1	88	3	0.9538	4	7	30
G1TE76	Q15056	EIF4H	Eukaryotic translation initiation factor 4H	Eukaryotic translation initiation factor 4H	92	2	0.9538	3	10	23
G1T5R3		TCF25	Transcription factor 25			1	0.9538	3	2	10
	H0Y507	SH3PXD2A		SH3 and PX domain-containing protein 2A (Fragment)		4	0.9538	2	2	3
G1TBL6	A0A087WVQ6	CLTC	Clathrin heavy chain	Clathrin heavy chain	99	2	0.9529	80	565	63

G1TLD3	Q02818	NUCB1	Nucleobindin 1	Nucleobindin-1	87	2	0.9529	17	23	45
	K7EPT8	GFAP		Glial fibrillary acidic protein (Fragment)		4	0.9529	3	209	15
G1SSV0	Q9H1E5	TMX4	Thioredoxin domain-containing protein	Thioredoxin-related transmembrane protein 4	84	2	0.9529	3	4	12
G1U9C1	P50570	DNM2	Dynamin 2	Dynamin-2	96	2	0.9520	24	7	36
G1SPA6	Q32P28	P3H1	Fe2OG dioxygenase domain-containing protein	Prolyl 3-hydroxylase 1	91	2	0.9520	24	47	52
G1T3R5		ERO1A	Endoplasmic reticulum oxidoreductase 1 alpha			1	0.9511	15	32	34
G1SFC5	Q86VS8	HOOK3	Calponin-homology (CH) domain-containing protein	Protein Hook homolog 3	99	2	0.9511	14	27	22
G1SYX4	P50281	MMP14	Matrix metalloproteinase-14	Matrix metalloproteinase-14	95	2	0.9511	5	6	11
G1SYA5	X6RA14	ESD	S-formylglutathione hydrolase	S-formylglutathione hydrolase	88	2	0.9501	8	16	45
G1SIP1	Q96LJ7	DHRS1	Uncharacterized protein	Dehydrogenase/reductase SDR family member 1	87	3	0.9501	6	6	30
G1SXJ6		CUL4A	CULLIN_2 domain-containing protein			1	0.9492	10	6	17
G1TII2	O95197	RTN3	Reticulon	Reticulon-3	72	2	0.9492	4	6	6
G1SP22	Q99943	AGPAT1	1-acyl-sn-glycerol-3-phosphate acyltransferase	1-acyl-sn-glycerol-3-phosphate acyltransferase alpha	98	2	0.9492	3	3	13
G1T2Y2		MAVS	CARD_2 domain-containing protein			1	0.9492	2	3	6
G1TL06	P39023	RPL3	Uncharacterized protein	60S ribosomal protein L3	98	3	0.9483	17	194	44
G1T2I7	Q9HCJ1	ANKH	Uncharacterized protein	Progressive ankylosis protein homolog	99	3	0.9483	3	4	13
G1SG72	P61221	ABCE1	Uncharacterized protein	ATP-binding cassette sub-family E member 1	100	3	0.9474	14	25	32
G1SJU1	O75051	PLXNA2	Sema domain-containing protein	Plexin-A2	98	2	0.9474	3	3	2
G1SDC6	Q9NXC5	MIOS	zinc_ribbon_16 domain-containing protein	GATOR complex protein MIOS	98	2	0.9474	2	2	2
G1SW61	Q2TAY7	SMU1	Uncharacterized protein	WD40 repeat-containing protein SMU1	100	3	0.9455	7	11	17
G1TM22		UGT1A1	UDP-glucuronosyltransferase			1	0.9455	7	2	22
G1SFQ6	Q9NSK0	KLC4	Uncharacterized protein	Kinesin light chain 4	97	3	0.9455	4	2	10
G1TP15	O43242	PSMD3	PCI domain-containing protein	26S proteasome non-ATPase regulatory subunit 3	98	2	0.9446	20	37	43
G1T057	D6RCE2	TTC37	Uncharacterized protein	Tetratricopeptide repeat protein 37 (Fragment)	83	3	0.9446	7	13	6
G1TER0	Q9NV70	EXOC1	Sec3-PIP2_bind domain-containing protein	Exocyst complex component 1	98	2	0.9446	6	9	14
G1SS85	Q9UBQ7	GRHPR	Uncharacterized protein	Glyoxylate reductase/hydroxypyruvate reductase	90	3	0.9446	5	8	28
G1SW89	Q9C0E8	LNPK	zinc_ribbon_10 domain-containing protein	Endoplasmic reticulum junction formation protein lunapark	89	2	0.9446	2	2	5
G1U6H0	P27824	CANX	Uncharacterized protein	Calnexin	95	3	0.9437	16	59	30
G1SDH3	E9PIG4	PRCP	Uncharacterized protein	Lysosomal Pro-X carboxypeptidase (Fragment)	89	3	0.9437	8	12	30
G1T0H3	P40763	STAT3	Signal transducer and activator of transcription	Signal transducer and activator of transcription 3	100	2	0.9437	8	14	17
G1SIA6	Q5T2E6	ARMH3	DUF1741 domain-containing protein	Armadillo-like helical domain-containing protein 3	99	2	0.9437	4	5	11
G1SJQ6	O00519	FAAH	Amidase domain-containing protein	Fatty-acid amide hydrolase 1	91	2	0.9437	4	6	11
	K7ELL7	PRKCSH		Glucosidase 2 subunit beta		4	0.9428	2	5	4
G1SRL3	P30419	NMT1	Glycylpeptide N-tetradecanoyltransferase	Glycylpeptide N-tetradecanoyltransferase 1	98	2	0.9418	7	10	19
G1U8C4	H0YKK6	PSME1	Uncharacterized protein	Proteasome activator complex subunit 1	98	3	0.9418	2	2	27
	V9GYD0	ARL2-SNX15		ARL2-SNX15 readthrough (NMD candidate)		4	0.9409	3	5	32
G1SMM4		DKK3	Dickkopf_N domain-containing protein			1	0.9409	3	8	14
G1T9M9	P11142	HSPA8	Uncharacterized protein	Heat shock cognate 71 kDa protein	100	3	0.9400	32	714	71
G1TDJ3	Q9BSJ8	ESYT1	Uncharacterized protein	Extended synaptotagmin-1	89	3	0.9400	30	44	41
G1TRH5	Q9ULV4	CORO1C	Coronin	Coronin-1C	97	2	0.9391	19	72	44
G1T3D1	Q14789	GOLGB1	Uncharacterized protein	Golgin subfamily B member 1	85	3	0.9382	32	43	15
G1SPG2		TRIP11	GRIP domain-containing protein			1	0.9382	16	18	11
G1SEF9	P17661	DES	IF rod domain-containing protein	Desmin	99	2	0.9382	8	8	10
G1STY8	G3V5E4	GNPNAT1	Glucosamine 6-phosphate N-acetyltransferase	Glucosamine 6-phosphate N-acetyltransferase	100	2	0.9382	4	4	20
G1SJ41	Q9Y6Y8	SEC23IP	DDHD domain-containing protein	SEC23-interacting protein	89	2	0.9363	14	23	16
G1TYK8	Q13492	PICALM	Phosphatidylinositol binding clathrin assembly protein	Phosphatidylinositol-binding clathrin assembly protein	97	2	0.9363	12	31	27
G1T4P8	O76003	GLRX3	Glutaredoxin 3	Glutaredoxin-3	93	2	0.9363	10	19	46
G1T7Q3	F5H157	RAB35	Uncharacterized protein	Ras-related protein Rab-35 (Fragment)	91	3	0.9363	6	20	40
G1SEV7	Q9NX55	HYPK	Uncharacterized protein	Huntingtin-interacting protein K	98	3	0.9363	4	7	42
G1SCP0	O00203	AP3B1	AP-3 complex subunit beta	AP-3 complex subunit beta-1	88	2	0.9354	13	17	16
G1T6B3	Q9Y3F4	STRAP	WD_REPEATS_REGION domain-containing protein	Serine-threonine kinase receptor-associated protein	98	2	0.9354	13	33	52
G1TPM1	Q96SB3	PPP1R9B	PDZ domain-containing protein	Neurabin-2	99	2	0.9354	6	11	15
G1T3Z2	Q9UNZ2	NSFL1C	Uncharacterized protein	NSFL1 cofactor p47	97	3	0.9354	5	7	19
	H3BQQ9	UBE2I		SUMO-conjugating enzyme UBC9 (Fragment)		4	0.9345	3	6	40
U3KPG6		ICAM1	Intercellular adhesion molecule 1			1	0.9335	8	15	20
G1TWK7	Q14696	MESD	Uncharacterized protein	LRP chaperone MESD	90	3	0.9335	4	7	22
G1SR53	P04066	FUCA1	Alpha-L-fucosidase	Tissue alpha-L-fucosidase	83	2	0.9326	10	29	34

G1SR28	Q15102	PAFAH1B3	SGNH_hydro domain-containing protein	Platelet-activating factor acetylhydrolase IB subunit gamma	97	2	0.9326	5	8	31
G1STJ3		PPIE	Peptidyl-prolyl cis-trans isomerase E			1	0.9326	2	2	13
G1U2R2	E9PLK3	NPEPPS	Aminopeptidase	Aminopeptidase	98	2	0.9317	16	25	27
G1T7F5	A0A1C7CYY0	ADD2	Aldolase_II domain-containing protein	Beta-adducin (Fragment)	93	2	0.9317	2	2	8
	Q9Y6I3	EPN1		Epsin-1		4	0.9317	2	3	4
G1T7I8		RIC8A	RIC8 guanine nucleotide exchange factor A			1	0.9308	4	5	11
G1TKY7		OGFOD3	2-oxoglutarate and iron dependent oxygenase domain containing 3			1	0.9308	3	3	13
G1SQ52	P52788	SMS	PABS domain-containing protein	Spermine synthase	99	2	0.9308	3	6	18
G1U0Q2	K7EJ78	RPS15	Uncharacterized protein	40S ribosomal protein S15	99	3	0.9308	2	15	28
G1U1M3	Q12904	AIMP1	tRNA-binding domain-containing protein	Aminoacyl tRNA synthase complex-interacting multifunctional protein 1	91	2	0.9299	8	15	36
G1SCU8		OTUD6B	OTU domain-containing protein			1	0.9299	3	3	19
G1SCF4	G8JLD5	DNM1L	Uncharacterized protein	Dynamin-1-like protein	93	3	0.9289	13	27	26
G1TY34		ITGA4	Integrin_alpha2 domain-containing protein			1	0.9289	4	7	7
	A0A0A0MRE1	EXOC7		Exocyst complex component 7 (Fragment)		4	0.9289	3	6	7
	P02458	COL2A1		Collagen alpha-1(II) chain		4	0.9280	4	2	5
G1T3Q2	Q96HY6	DDRGK1	Uncharacterized protein	DDRGK domain-containing protein 1	90	3	0.9271	6	15	25
G1T6S6		ATP6V1F	V-type proton ATPase subunit F			1	0.9271	4	11	54
G1TIA2		RIOX1	JmjC domain-containing protein			1	0.9271	3	3	10
G1SYR9	B9A067	IMMT	MICOS complex subunit MIC60	MICOS complex subunit MIC60	89	2	0.9262	31	57	50
P68105	P68104	EEF1A1	Elongation factor 1-alpha 1	Elongation factor 1-alpha 1	100	2	0.9262	22	816	65
G1SPR5	P16278	GLB1	Glyco_hydro_35 domain-containing protein	Beta-galactosidase	82	2	0.9262	15	27	33
G1TEA8	P12268	IMPDH2	Inosine-5~-monophosphate dehydrogenase	Inosine-5~-monophosphate dehydrogenase 2	99	2	0.9253	9	16	29
G1SMZ8	Q96JJ7	TMX3	Thioredoxin domain-containing protein	Protein disulfide-isomerase TMX3	92	2	0.9253	8	8	28
G1SZA3	Q9H2M9	RAB3GAP2	Uncharacterized protein	Rab3 GTPase-activating protein non-catalytic subunit	94	3	0.9234	13	13	20
G1SPJ5	Q92616	GCN1	TOG domain-containing protein	eIF-2-alpha kinase activator GCN1	96	2	0.9225	46	83	27
G1SDR2	P24844	MYL9	Uncharacterized protein	Myosin regulatory light polypeptide 9	99	3	0.9225	10	19	80
G1TEK3	Q8IWJ2	GCC2	GRIP domain-containing protein	GRIP and coiled-coil domain-containing protein 2	86	2	0.9225	9	16	8
G1TM81	A0A0B4J2C3	TPT1	Translationally-controlled tumor protein	Translationally-controlled tumor protein	98	2	0.9225	5	67	34
G1TBG2	Q96KP1	EXOC2	Exocyst complex component 2	Exocyst complex component 2	94	2	0.9225	4	5	6
G1STW7	Q9NSD9	FARSB	B5 domain-containing protein	Phenylalanine--tRNA ligase beta subunit	95	2	0.9216	11	17	22
G1T670		SORT1	Proteasome subunit alpha type			1	0.9216	10	20	48
G1T168	P46783	RPS10	S10_plectin domain-containing protein	40S ribosomal protein S10	100	2	0.9197	10	15	40
	P20908	COL5A1		Collagen alpha-1(V) chain		4	0.9197	9	3	6
G1SW40	E7EMB1	SWAP70	PH domain-containing protein	Switch-associated protein 70	77	2	0.9197	7	24	16
G1T4Q9	P28074	PSMB5	Proteasome subunit beta	Proteasome subunit beta type-5	98	2	0.9188	9	48	47
G1SYE7	H0Y8R1	GRSF1	Uncharacterized protein	G-rich sequence factor 1 (Fragment)	96	3	0.9188	3	5	14
G1TDC3	Q14697	GANAB	Gal_mutarotas_2 domain-containing protein	Neutral alpha-glucosidase AB	91	2	0.9179	30	236	46
G1U0B3	P53007	SLC25A1	Solute carrier family 25 member 1	Tricarboxylate transport protein, mitochondrial	86	2	0.9179	10	25	48
	Q9NUY8	TBC1D23		TBC1 domain family member 23		4	0.9179	3	5	6
G1TDN6		KRT5	IF rod domain-containing protein			1	0.9170	5	2	8
G1SIS5	O15498	YKT6	Uncharacterized protein	Synaptobrevin homolog YKT6	96	3	0.9170	5	6	36
G1SJX5	A0A087WTF3	ANK3	Ankyrin 3	Ankyrin-3 (Fragment)	93	2	0.9170	3	5	4
G1SIW8	Q08257	CRYZ	PKS_ER domain-containing protein	Quinone oxidoreductase	87	2	0.9160	7	24	39
G1SL98	Q53EP0	FNDC3B	Uncharacterized protein	Fibronectin type III domain-containing protein 3B	97	3	0.9151	15	4	20
P13642	P49591	SARS	Serine--tRNA ligase, cytoplasmic	Serine--tRNA ligase, cytoplasmic	97	2	0.9151	14	24	32
P41035	P20042	EIF2S2	Eukaryotic translation initiation factor 2 subunit 2	Eukaryotic translation initiation factor 2 subunit 2	98	2	0.9151	12	26	53
G1SYC9	H0YNE9	RAB8B	Uncharacterized protein	Ras-related protein Rab-8B (Fragment)	99	3	0.9151	5	4	19
G1SPB8	Q99733	NAP1L4	Uncharacterized protein	Nucleosome assembly protein 1-like 4	93	3	0.9142	9	17	40
G1T550	P51148	RAB5C	Uncharacterized protein	Ras-related protein Rab-5C	91	3	0.9142	8	28	53
G1TA41	Q7L7X3	TAOK1	Protein kinase domain-containing protein	Serine/threonine-protein kinase TAO1	100	2	0.9142	4	3	6
G1T5N5	C9IZ01	GFM1	Elongation factor G, mitochondrial	Elongation factor G, mitochondrial	93	2	0.9133	8	9	19
G1TBC4	G3V126	ATP6V1H	V-type proton ATPase subunit H	V-type proton ATPase subunit H	99	2	0.9133	6	8	23
G1THI9	Q9UKB1	FBXW11	Uncharacterized protein	F-box/WD repeat-containing protein 11	96	3	0.9133	2	2	7
G1SQD1	E7EUU4	EIF4G1	Eukaryotic translation initiation factor 4 gamma 1	Eukaryotic translation initiation factor 4 gamma 1	94	2	0.9124	21	35	15
G1U535	Q9HBH5	RDH14	Uncharacterized protein	Retinol dehydrogenase 14	91	3	0.9124	4	3	13
G1TPZ1		LGALS1	Galectin			1	0.9114	9	39	75
G1T194	Q9ULX6	AKAP8L	A-kinase anchoring protein 8 like	A-kinase anchor protein 8-like	86	2	0.9114	4	6	10

G1TPB1		CRAT	Carnitine O-acetyltransferase			1	0.9114	2	2	8
G1TUD6	P43686	PSMC4	Proteasome 26S subunit, ATPase 4	26S proteasome regulatory subunit 6B	100	2	0.9105	12	55	34
	Q9H0U4	RAB1B		Ras-related protein Rab-1B		4	0.9105	12	14	69
G1SN17		PPP4R1	WRNPLPNID domain-containing protein			1	0.9105	4	4	8
G1U9I8	P04264	KRT1	IF rod domain-containing protein	Keratin, type II cytoskeletal 1	86	2	0.9096	20	36	39
G1T9H0	Q8NCA5	FAM98A	Uncharacterized protein	Protein FAM98A	92	3	0.9096	8	11	22
G1TBC9		TMEM119	Transmembrane protein 119			1	0.9096	2	2	17
G1SLU5	Q9UKZ1	CNOT11	CCR4-NOT transcription complex subunit 11	CCR4-NOT transcription complex subunit 11	79	2	0.9087	2	2	6
G1SXL9		XPNPEP3	AMP_N domain-containing protein			1	0.9087	2	2	9
G1T726	A0A0A0MSE2	HADH	Uncharacterized protein	Hydroxyacyl-coenzyme A dehydrogenase, mitochondrial	92	3	0.9077	10	15	43
G1T2I4	P07814	EPRS	Glutamyl-prolyl-tRNA synthetase	Bifunctional glutamate/proline--tRNA ligase	89	2	0.9068	54	70	46
G1SLZ8	Q06124	PTPN11	Tyrosine-protein phosphatase non-receptor type	Tyrosine-protein phosphatase non-receptor type 11	98	2	0.9059	6	6	15
G1SET0	P35606	COPB2	Coatomer subunit beta~	Coatomer subunit beta~	98	2	0.9050	24	58	38
	P10301	RRAS		Ras-related protein R-Ras		4	0.9050	6	4	38
G1T3P1	K7EKP8	ACOT7	Uncharacterized protein	Cytosolic acyl coenzyme A thioester hydrolase (Fragment)	98	3	0.9050	5	5	23
G1U7C7	Q5GLZ8	HERC4	HECT domain-containing protein	Probable E3 ubiquitin-protein ligase HERC4	96	2	0.9050	3	3	6
G1T466	A0AVT1	UBA6	UBA_e1_C domain-containing protein	Ubiquitin-like modifier-activating enzyme 6	93	2	0.9041	7	6	10
G1U522	P13861	PRKAR2A	Uncharacterized protein	cAMP-dependent protein kinase type II-alpha regulatory subunit	90	3	0.9031	10	12	34
G1U2R1	Q99805	TM9SF2	Transmembrane 9 superfamily member	Transmembrane 9 superfamily member 2	93	2	0.9022	6	20	16
G1SIB1	P29083	GTF2E1	HTH TFE/IIEalpha-type domain-containing protein	General transcription factor IIE subunit 1	94	2	0.9013	2	2	9
G1SSZ1	Q01658	DR1	CBFD_NFYB_HMF domain-containing protein	Protein Dr1	100	2	0.9004	2	3	21
G1T0Q6	Q9UQE7	SMC3	Structural maintenance of chromosomes protein	Structural maintenance of chromosomes protein 3	100	2	0.8995	8	7	9
Q95MN6		PLP2	Proteolipid protein 2			1	0.8995	3	7	34
G1T089		THUMPD3	THUMP domain containing 3			1	0.8995	3	3	9
G1SRL4		NAGA	Alpha-galactosidase			1	0.8985	14	29	47
G1SE28	C9JNW5	RPL24	TRASH domain-containing protein	60S ribosomal protein L24	100	2	0.8985	6	10	34
G1SZW5	Q8TD16	BICD2	Uncharacterized protein	Protein bicaudal D homolog 2	95	3	0.8985	4	5	7
G1TQD3	Q01968	OCRL	Rho-GAP domain-containing protein	Inositol polyphosphate 5-phosphatase OCRL	95	2	0.8985	3	4	5
G1T1Y3	A0A384DVU0	PNPLA6	Patatin like phospholipase domain containing 6	Neuropathy target esterase	98	2	0.8985	3	2	5
G1T198	Q8TDX7	NEK7	Protein kinase domain-containing protein	Serine/threonine-protein kinase Nek7	98	2	0.8976	3	4	15
G1T845	I3L0N3	NSF	Uncharacterized protein	Vesicle-fusing ATPase	99	3	0.8967	17	31	33
G1U5L3	P49257	LMAN1	L-type lectin-like domain-containing protein	Protein ERGIC-53	91	2	0.8967	15	66	40
G1SZ93	Q9NSE4	IARS2	Uncharacterized protein	Isoleucine--tRNA ligase, mitochondrial	90	3	0.8967	14	16	22
G1SVW7	G3V1U5	GOLT1B	Uncharacterized protein	Golgi transport 1 homolog B (S. cerevisiae), isoform CRA_c	100	3	0.8967	3	24	17
G1SM04	Q13188	STK3	Uncharacterized protein	Serine/threonine-protein kinase 3	99	3	0.8967	2	2	5
G1SYN4		PTX3	LamGL domain-containing protein			1	0.8958	11	20	29
G1SPN3	Q14573	ITPR3	Inositol 1,4,5-trisphosphate receptor type 3	Inositol 1,4,5-trisphosphate receptor type 3	95	2	0.8958	8	9	6
G1TAD1	Q7RTS9	DYM	Uncharacterized protein	Dymeclin	97	3	0.8958	4	7	13
G1TM86	A2AB27	GNL1	G protein nucleolar 1 (putative)	Guanine nucleotide-binding protein-like 1 (Fragment)	98	2	0.8958	2	4	5
G1U2W0	B4E321	OS9	OS9, endoplasmic reticulum lectin	Protein OS-9	79	2	0.8948	3	2	5
G1ST51		CLIC2	Chloride intracellular channel protein			1	0.8939	4	4	23
G1TRS4	O95302	FKBP9	Peptidylprolyl isomerase	Peptidyl-prolyl cis-trans isomerase FKBP9	97	2	0.8930	17	136	41
G1TCY1	Q16513	PKN2	Uncharacterized protein	Serine/threonine-protein kinase N2	97	3	0.8921	4	3	4
G1TCP2	A0A087X1E4	ARFIP2	AH domain-containing protein	Arfaptin-2	91	2	0.8921	2	5	9
G1SNZ8		GORASP1	GRASP55_65 domain-containing protein			1	0.8921	2	3	7
G1TT64	Q68EM7	ARHGAP17	Uncharacterized protein	Rho GTPase-activating protein 17	91	3	0.8912	4	4	8
G1U3S6	O15258	RER1	Protein RER1	Protein RER1	95	2	0.8912	2	4	13
	Q9UL15	BAG5		BAG family molecular chaperone regulator 5		4	0.8902	2	2	7
	Q6WCQ1	MPRIP		Myosin phosphatase Rho-interacting protein		4	0.8893	16	2	20
G1SRB6	Q08752	PPID	Peptidylprolyl isomerase D	Peptidyl-prolyl cis-trans isomerase D	93	2	0.8893	9	24	27
G1T365	Q9H269	VPS16	Vacuolar protein sorting-associated protein 16 homolog	Vacuolar protein sorting-associated protein 16 homolog	98	2	0.8893	6	9	15
G1SZR6	Q13618	CUL3	CULLIN_2 domain-containing protein	Cullin-3	100	2	0.8884	13	28	23
G1SKS9	A0A087WSW9	TXNRD1	Glutaredoxin domain-containing protein	Thioredoxin reductase 1, cytoplasmic	94	2	0.8884	10	10	27
G1TA48	Q9H223	EHD4	Uncharacterized protein	EH domain-containing protein 4	97	3	0.8875	14	19	37
G1SKT3	Q9NUJ1	ABHD10	AB hydrolase-1 domain-containing protein	Mycophenolic acid acyl-glucuronide esterase, mitochondrial	88	2	0.8875	8	13	36
G1TTJ1	Q6UWP2	DHRS11	Uncharacterized protein	Dehydrogenase/reductase SDR family member 11	92	3	0.8875	4	5	21
	J3KN01	AFDN		Afadin		4	0.8865	2	3	2

G1SDG2	P48739	PITPNB	Phosphatidylinositol transfer protein beta	Phosphatidylinositol transfer protein beta isoform	97	2	0.8856	4	4	14
G1T0B0	Q9H3H3	C11orf68	Uncharacterized protein	UPF0696 protein C11orf68	93	3	0.8856	2	3	13
G1TYU5		QTRT1	Queuine tRNA-ribosyltransferase			1	0.8856	2	3	12
G1SNS3	Q6P4E1	CASC4	Uncharacterized protein	Protein CASC4	89	3	0.8847	7	8	16
	G8JLG1	SMC1A		Structural maintenance of chromosomes protein		4	0.8847	5	5	6
G1SV22	P30086	PEBP1	Phosphatidylethanolamine-binding protein 1	Phosphatidylethanolamine-binding protein 1	89	2	0.8838	5	7	37
G1U304		GALNS	Galactosamine (N-acetyl)-6-sulfatase			1	0.8838	2	5	7
G1TDQ5	A0A2R8Y891	PFKM	ATP-dependent 6-phosphofructokinase	ATP-dependent 6-phosphofructokinase	97	2	0.8829	5	7	10
G1TB71	Q14008	CKAP5	Uncharacterized protein	Cytoskeleton-associated protein 5	97	3	0.8819	18	25	15
G1SSL2	F8VXJ7	CNPY2	Saposin B-type domain-containing protein	Protein canopy homolog 2 (Fragment)	99	2	0.8819	7	25	56
G1SQ96	Q5H9R7	PPP6R3	Uncharacterized protein	Serine/threonine-protein phosphatase 6 regulatory subunit 3	92	3	0.8810	7	6	13
G1ST05	Q15120	PDK3	Protein-serine/threonine kinase	[Pyruvate dehydrogenase (acetyl-transferring)] kinase isozyme 3, mitochondrial	99	2	0.8810	6	9	26
G1TA82	Q12768	WASHC5	Uncharacterized protein	WASH complex subunit 5	97	3	0.8801	6	12	9
G1SMP3	A0A2R8Y6F8	CASK	Uncharacterized protein	Peripheral plasma membrane protein CASK	97	3	0.8792	12	15	17
G1ST38	Q9Y673	ALG5	ALG5, dolichyl-phosphate beta-glucosyltransferase	Dolichyl-phosphate beta-glucosyltransferase	93	2	0.8792	6	10	21
G1TD16	P48426	PIP4K2A	PIPK domain-containing protein	Phosphatidylinositol 5-phosphate 4-kinase type-2 alpha	100	2	0.8792	2	2	5
G1SHS4	A0A0C4DGX4	CUL1	CULLIN_2 domain-containing protein	Cullin-1	97	2	0.8783	5	10	11
G1SLC0	Q9BS26	ERP44	Thioredoxin domain-containing protein	Endoplasmic reticulum resident protein 44	97	2	0.8764	13	32	42
G1TD47	Q8N3E9	PLCD3	Phosphoinositide phospholipase C	1-phosphatidylinositol 4,5-bisphosphate phosphodiesterase delta-3	88	2	0.8764	4	5	9
G1SIA1	A0A087WWA3	KIF1B	Uncharacterized protein	Kinesin-like protein KIF1B	96	3	0.8764	3	6	2
Q28740		BSG	Basigin			1	0.8764	2	4	10
G1SEJ4	P21281	ATP6V1B2	Vacuolar proton pump subunit B	V-type proton ATPase subunit B, brain isoform	99	2	0.8755	18	38	55
G1SKV7	Q96KP4	CNDP2	M20_dimer domain-containing protein	Cytosolic non-specific dipeptidase	90	2	0.8755	12	18	38
G1SQU0	P15586	GNS	N-acetylglucosamine-6-sulfatase	N-acetylglucosamine-6-sulfatase	95	2	0.8755	10	14	19
G1SNP4	A0A3B3ITZ9	THRAP3	Uncharacterized protein	Thyroid hormone receptor-associated protein 3	95	3	0.8755	6	8	8
G1SMB5		VPS37C	VPS37 C-terminal domain-containing protein			1	0.8755	4	4	21
G1SRI2	Q6P996	PDXDC1	Uncharacterized protein	Pyridoxal-dependent decarboxylase domain-containing protein 1	89	3	0.8746	17	32	35
	P63241	EIF5A		Eukaryotic translation initiation factor 5A-1		4	0.8746	9	221	66
G1SW44	H7C286	NAGK	BcrAD_BadFG domain-containing protein	N-acetyl-D-glucosamine kinase	97	2	0.8746	6	10	27
G1T6S9	P53367	ARFIP1	AH domain-containing protein	Arfaptin-1	98	2	0.8746	4	5	15
G1SNL7	Q9H1I8	ASCC2	Activating signal cointegrator 1 complex subunit 2	Activating signal cointegrator 1 complex subunit 2	86	2	0.8746	3	4	6
G1SWM8	A0A0U1RQQ9	SCYL2	Protein kinase domain-containing protein	SCY1-like protein 2	94	2	0.8746	2	2	6
G1T2W1	O76094	SRP72	Signal recognition particle subunit SRP72	Signal recognition particle subunit SRP72	98	2	0.8736	9	15	20
G1SPI7	Q92538	GBF1	SEC7 domain-containing protein	Golgi-specific brefeldin A-resistance guanine nucleotide exchange factor 1	96	2	0.8727	18	23	14
G1TI53	A0A0A0MRM8	MYO6	Uncharacterized protein	Unconventional myosin-VI	94	3	0.8727	7	13	8
G1T706	O00442	RTCA	Uncharacterized protein	RNA 3~-terminal phosphate cyclase	96	3	0.8718	5	6	19
G1T9V2	A0A2R8Y852	CUX1	Cut like homeobox 1	Homeobox protein cut-like	87	2	0.8718	4	5	5
	P37837	TALDO1		Transaldolase		4	0.8718	3	6	11
G1SZ19	Q9Y3L5	RAP2C	Uncharacterized protein	Ras-related protein Rap-2c	100	3	0.8709	6	3	38
G1SH85	A0A2R8YF87	VPS33A	Uncharacterized protein	Vacuolar protein sorting-associated protein 33A	91	3	0.8690	8	9	22
G1T276		ALDH3A2	Aldehyde dehydrogenase			1	0.8690	7	11	21
G1T579	Q6NUQ1	RINT1	Uncharacterized protein	RAD50-interacting protein 1	92	3	0.8690	7	11	15
	O15143	ARPC1B		Actin-related protein 2/3 complex subunit 1B		4	0.8690	3	6	14
G1SH66		RBM3	RRM domain-containing protein			1	0.8690	3	6	37
G1T302		THYN1	EVE domain-containing protein			1	0.8690	2	7	10
G1T329	J3QQY1	CDK5RAP3	Uncharacterized protein	CDK5 regulatory subunit-associated protein 3 (Fragment)	88	3	0.8681	9	14	25
G1T416	Q9UP83	COG5	Uncharacterized protein	Conserved oligomeric Golgi complex subunit 5	91	3	0.8681	5	5	11
	Q9NWU2	GID8		Glucose-induced degradation protein 8 homolog		4	0.8681	5	10	41
G1U7M0	Q96AJ9	VTI1A	t-SNARE coiled-coil homology domain-containing protein	Vesicle transport through interaction with t-SNAREs homolog 1A	94	2	0.8681	3	3	17
G1T850	B4E0K5	MAPK14	Mitogen-activated protein kinase	Mitogen-activated protein kinase	100	2	0.8672	2	2	13
G1SCE6	Q9Y223	GNE	Epimerase_2 domain-containing protein	Bifunctional UDP-N-acetylglucosamine 2-epimerase/N-acetylmannosamine kinase	100	2	0.8663	10	25	27
G1T8P7	A0A2R8YFH5	SEC23B	Protein transport protein SEC23	Protein transport protein SEC23	95	2	0.8663	8	9	15
G1TE64	O95758	PTBP3	Uncharacterized protein	Polypyrimidine tract-binding protein 3	97	3	0.8663	5	2	20
G1SJG0	P28300	LOX	Uncharacterized protein	Protein-lysine 6-oxidase	88	3	0.8663	4	6	14
G1T534	E9PDM8	SEC24D	SEC24 homolog D, COPII coat complex component	Protein transport protein Sec24D	92	2	0.8654	8	2	8
G1T1V9	P54652	HSPA2	Uncharacterized protein	Heat shock-related 70 kDa protein 2	98	3	0.8644	26	39	58
G1SST9	P54727	RAD23B	Uncharacterized protein	UV excision repair protein RAD23 homolog B	94	3	0.8644	12	15	42

G1T2I5	F8W914	RTN4	Reticulon	Reticulon	98	2	0.8644	8	277	13
G1SWK5	A0A1W2PNP0	PIGT	Uncharacterized protein	GPI transamidase component PIG-T (Fragment)	86	3	0.8644	3	4	8
	M0QXF7	MYDGF		Myeloid-derived growth factor (Fragment)		4	0.8644	2	2	19
G1T7I4	P53621	COPA	Coatomer subunit alpha	Coatomer subunit alpha	99	2	0.8635	48	103	52
G1TDF6	O75955	FLOT1	PHB domain-containing protein	Flotillin-1	99	2	0.8635	13	23	40
G1T846	P14868	DARS	AA_TRNA_LIGASE_II domain-containing protein	Aspartate--tRNA ligase, cytoplasmic	98	2	0.8635	10	21	22
G1SRT1	Q9UKG1	APPL1	Adaptor protein, phosphotyrosine interacting with PH domain and leucine zipper 1	DCC-interacting protein 13-alpha	98	2	0.8635	8	10	19
G1TS73	H0YBP1	PTK2	Protein tyrosine kinase 2	Focal adhesion kinase 1 (Fragment)	89	2	0.8617	4	4	6
G1SM31	Q9P2G1	ANKIB1	RBR-type E3 ubiquitin transferase	Ankyrin repeat and IBR domain-containing protein 1	93	2	0.8617	2	4	4
G1T501		CCAR2	Cell cycle and apoptosis regulator 2			1	0.8607	2	4	6
G1TQR0	P12814-3	ACTN1	Uncharacterized protein	Isoform 3 of Alpha-actinin-1	97	3	0.8598	59	116	79
P00883	P04075	ALDOA	Fructose-bisphosphate aldolase A	Fructose-bisphosphate aldolase A	98	2	0.8598	24	111	76
G1SW57	P21589	NT5E	Uncharacterized protein	5~-nucleotidase	89	3	0.8598	10	13	21
G1SJL0		TIMM29	Translocase of inner mitochondrial membrane 29			1	0.8598	2	3	16
G1TCY4	Q9Y5X3	SNX5	Sorting nexin	Sorting nexin-5	98	2	0.8589	4	8	12
G1TSL5	C9J6N9	UFD1	Uncharacterized protein	Ubiquitin recognition factor in ER-associated degradation protein 1 (Fragment)	100	3	0.8589	3	3	13
G1SLS3	O43747	AP1G1	AP-1 complex subunit gamma	AP-1 complex subunit gamma-1	100	2	0.8580	8	13	16
G1SQ23	P42025	ACTR1B	Uncharacterized protein	Beta-centractin	99	3	0.8580	7	4	25
G1SFX7	P62330	ARF6	Uncharacterized protein	ADP-ribosylation factor 6	100	3	0.8580	5	9	33
G1T3U3	A0A0G2JRV3	SMARCB1	SWI/SNF related, matrix associated, actin dependent regulator of chromatin, subfamily b, member 1	SWI/SNF-related matrix-associated actin-dependent regulator of chromatin subfamily B member 1	85	2	0.8580	4	6	26
G1U0T4		HSD17B14	Hydroxysteroid 17-beta dehydrogenase 14			1	0.8580	2	3	9
G1SIB6	M0R192	BLVRB	Biliverdin reductase B	Flavin reductase (NADPH)	73	2	0.8571	7	31	51
G1T4Z0	Q96PY5	FMNL2	Formin like 2	Formin-like protein 2	97	2	0.8571	7	4	8
G1TZQ5	G5EA31	SEC24C	Uncharacterized protein	Protein transport protein Sec24C	93	3	0.8571	6	8	10
G1T593	F8VVA7	COPZ1	Clat_adaptor_s domain-containing protein	Coatomer subunit zeta-1	88	2	0.8571	4	13	45
G1T725	Q8TB40	ABHD4	AB hydrolase-1 domain-containing protein	(Lyso)-N-acylphosphatidylethanolamine lipase	97	2	0.8571	2	3	8
	P35527	KRT9		Keratin, type I cytoskeletal 9		4	0.8561	19	39	44
G1SLJ8	Q8IY81	FTSJ3	pre-rRNA processing protein FTSJ3	pre-rRNA 2~-O-ribose RNA methyltransferase FTSJ3	85	2	0.8561	7	9	19
G1T8C2	F8W7U8	MRE11	Double-strand break repair protein	Double-strand break repair protein	93	2	0.8561	4	3	9
G1SMG5	P15170	GSPT1	Tr-type G domain-containing protein	Eukaryotic peptide chain release factor GTP-binding subunit ERF3A	99	2	0.8543	11	18	25
G1TA78	P23381	WARS	Tryptophan--tRNA ligase, cytoplasmic	Tryptophan--tRNA ligase, cytoplasmic	89	2	0.8543	11	25	31
G1SJ23	P07384	CAPN1	Calpain-1 catalytic subunit	Calpain-1 catalytic subunit	90	2	0.8515	10	11	25
G1SH95	P30622	CLIP1	CAP-Gly domain containing linker protein 1	CAP-Gly domain-containing linker protein 1	85	2	0.8506	12	11	10
G1SW65		ATP5MC2	ATP-synt_C domain-containing protein			1	0.8506	2	2	21
G1SVZ8		C9orf64	Queuosine salvage protein			1	0.8506	2	2	7
G1SWK3	P49902	NT5C2	Uncharacterized protein	Cytosolic purine 5~-nucleotidase	100	3	0.8497	8	15	21
G1SZT8	P55735	SEC13	WD_REPEATS_REGION domain-containing protein	Protein SEC13 homolog	95	2	0.8497	8	16	38
G1SCE1	Q9H4G4	GLIPR2	SCP domain-containing protein	Golgi-associated plant pathogenesis-related protein 1	97	2	0.8497	4	4	41
G1SMM1		USP47	USP domain-containing protein			1	0.8497	2	3	3
G1SM51	Q9UNM6	PSMD13	Proteasome 26S subunit, non-ATPase 13	26S proteasome non-ATPase regulatory subunit 13	92	2	0.8488	15	11	46
G1SM05	Q9NR31	SAR1A	Uncharacterized protein	GTP-binding protein SAR1a	99	3	0.8488	6	13	41
G1SUN1	O75915	ARL6IP5	PRA1 family protein	PRA1 family protein 3	96	2	0.8488	5	24	24
	Q13363	CTBP1		C-terminal-binding protein 1		4	0.8488	4	4	10
G1U2B5		PDXK	Phos_pyr_kin domain-containing protein			1	0.8488	3	3	19
G1T8D4	D6REX3	SEC31A	WD_REPEATS_REGION domain-containing protein	Protein transport protein Sec31A	91	2	0.8478	29	64	35
G1T0S0	Q8NBN3	TMEM87A	Uncharacterized protein	Transmembrane protein 87A	96	3	0.8478	4	6	9
G1TTS1		FUNDC2	FUN14 domain containing 2			1	0.8478	2	3	12
G1T7Y7	B1AK87	CAPZB	F-actin-capping protein subunit beta	F-actin-capping protein subunit beta	100	2	0.8469	11	37	54
G1T450		RO60	TROVE domain-containing protein			1	0.8469	3	6	7
G1SRB7	A0A2R8Y7U1	TPP1	Peptidase S53 domain-containing protein	Tripeptidyl-peptidase 1 (Fragment)	93	2	0.8460	7	14	23
G1TC10	P61081	UBE2M	UBIQUITIN_CONJUGAT_2 domain-containing protein	NEDD8-conjugating enzyme Ubc12	100	2	0.8460	7	8	51
G1U1E5	O60826	CCDC22	Coiled-coil domain containing 22	Coiled-coil domain-containing protein 22	75	2	0.8460	6	7	12
G1T782	Q9Y6N5	SQOR	Pyr_redox_2 domain-containing protein	Sulfide:quinone oxidoreductase, mitochondrial	93	2	0.8460	6	14	18
G1TWB9		TBC1D5	Rab-GAP TBC domain-containing protein			1	0.8460	2	3	4
G1SQ22	Q9Y678	COPG1	Coatomer subunit gamma	Coatomer subunit gamma-1	99	2	0.8451	35	161	58
G1T0K1	Q9BTV4	TMEM43	Uncharacterized protein	Transmembrane protein 43	92	3	0.8451	10	20	39
G1SYN5	J3QRU4	VAMP2	V-SNARE coiled-coil homology domain-containing protein	Vesicle-associated membrane protein 2	99	2	0.8451	4	4	42

G1SGB3		ELP1	Elongator complex protein 1			1	0.8451	2	3	4
B7NZM8	Q04917	YWHAH	Tyrosine 3-monooxygenase/tryptophan 5-monooxygenase activation protein, eta polypeptide (Predicted)	14-3-3 protein eta	99	2	0.8442	14	35	65
	Q63ZY3	KANK2		KN motif and ankyrin repeat domain-containing protein 2		4	0.8442	9	7	13
G1T6E8	F5H442	TSG101	Uncharacterized protein	Tumor susceptibility gene 101 protein	99	3	0.8442	6	8	25
G1TE96	O43813	LANCL1	Uncharacterized protein	Glutathione S-transferase LANCL1	95	3	0.8442	4	8	18
G1SU24	O60503	ADCY9	Adenylate cyclase 9	Adenylate cyclase type 9	92	2	0.8442	2	2	4
G1SPL8		NGB	Neuroglobin			1	0.8432	2	2	18
G1TRA4	P01111	NRAS	Uncharacterized protein	GTPase NRas	100	3	0.8423	7	3	53
G1SGL4	Q9Y276	BCS1L	Uncharacterized protein	Mitochondrial chaperone BCS1	96	3	0.8423	6	6	26
G1T3L5	O43719	HTATSF1	Uncharacterized protein	HIV Tat-specific factor 1	82	3	0.8423	3	3	6
G1SSS4		CLCN6	Chloride transport protein 6			1	0.8423	2	2	4
G1SMG1		KANK1	KN motif and ankyrin repeat domains 1			1	0.8414	6	5	10
G1SPV8	O94829	IPO13	Importin N-terminal domain-containing protein	Importin-13	100	2	0.8414	2	2	3
G1TJ79		KYAT1	Aminotran_1_2 domain-containing protein			1	0.8414	2	2	10
G1TUU3	Q5JXR6	ZNFX1	Uncharacterized protein	NFX1-type zinc finger-containing protein 1	90	3	0.8414	2	2	2
G1T7H0	A0A1W2PPS1	HNRNPU	Uncharacterized protein	Heterogeneous nuclear ribonucleoprotein U	96	3	0.8405	22	58	33
G1SNE8	P31949	S100A11	Protein S100	Protein S100-A11	87	2	0.8405	5	44	65
G1T1T4		ADPGK	ADP dependent glucokinase			1	0.8395	9	16	27
G1SXH7	P12931	SRC	Tyrosine-protein kinase	Proto-oncogene tyrosine-protein kinase Src	96	2	0.8395	5	6	14
	Q96HP0	DOCK6		Dedicator of cytokinesis protein 6		4	0.8395	3	5	2
G1TRM4	P62280	RPS11	Ribosomal_S17_N domain-containing protein	40S ribosomal protein S11	100	2	0.8386	9	20	55
G1SVW5	P36578	RPL4	Ribos_L4_asso_C domain-containing protein	60S ribosomal protein L4	96	2	0.8377	14	47	36
G1SM62	O43252	PAPSS1	Uncharacterized protein	Bifunctional 3~-phosphoadenosine 5~-phosphosulfate synthase 1	99	3	0.8377	9	17	24
G1SM65		GALNT16	Polypeptide N-acetylgalactosaminyltransferase			1	0.8377	3	9	16
G1TBJ4	Q92597	NDRG1	Uncharacterized protein	Protein NDRG1	96	3	0.8377	3	3	17
G1SFC4		LMF2	Lipase maturation factor			1	0.8368	3	7	7
G1T1R9	Q96A65	EXOC4	Sec8_exocyst domain-containing protein	Exocyst complex component 4	95	2	0.8359	11	15	19
G1SID3	Q709C8	VPS13C	Vacuolar protein sorting 13 homolog C	Vacuolar protein sorting-associated protein 13C	88	2	0.8349	10	10	4
	A0A0A0MSZ1	MARK3		Non-specific serine/threonine protein kinase		4	0.8349	2	2	4
G1SX42	Q9Y3P9	RABGAP1	Uncharacterized protein	Rab GTPase-activating protein 1	97	3	0.8349	2	2	3
G1T7T6	O60763	USO1	Uncharacterized protein	General vesicular transport factor p115	95	3	0.8340	22	41	35
G1SQL0	P62191	PSMC1	AAA domain-containing protein	26S proteasome regulatory subunit 4	100	2	0.8331	17	33	43
G1TXS2	A0A0D9SFK2	MYO18A	Uncharacterized protein	Unconventional myosin-XVIIIa	94	3	0.8331	10	13	7
G1SIG2	P46108	CRK	Uncharacterized protein	Adapter molecule crk	99	3	0.8331	7	3	37
G1TAW7		EIF2A	Eukaryotic translation initiation factor 2A			1	0.8331	3	5	9
G1SL46		PSMD9	PDZ domain-containing protein			1	0.8331	3	3	15
G1SES2		NAA10	N-acetyltransferase domain-containing protein			1	0.8322	2	2	9
G1SUL3		TMF1	TMF_TATA_bd domain-containing protein			1	0.8322	2	2	3
G1SJN4	Q9BT78	COPS4	PCI domain-containing protein	COP9 signalosome complex subunit 4	100	2	0.8313	12	28	49
G1TA50	E7EQ61	UBA5	ThiF domain-containing protein	Ubiquitin-like modifier-activating enzyme 5	93	2	0.8313	6	10	27
G1T652	P09972	ALDOC	Fructose-bisphosphate aldolase	Fructose-bisphosphate aldolase C	99	2	0.8303	9	8	30
G1SKM5	Q8TAT6	NPLOC4	NPL4 homolog, ubiquitin recognition factor	Nuclear protein localization protein 4 homolog	92	2	0.8303	5	6	12
G1T7P8	Q9UIV1	CNOT7	Uncharacterized protein	CCR4-NOT transcription complex subunit 7	100	3	0.8303	3	4	16
G1SL38	F8VS81	TWF1	Twinfilin actin binding protein 1	Twinfilin-1 (Fragment)	96	2	0.8294	7	15	25
G1SII9	Q5QJ74	TBCEL	Ubiquitin-like domain-containing protein	Tubulin-specific chaperone cofactor E-like protein	99	2	0.8294	2	2	8
G1SCI5	F5H6E2	MYO1C	Uncharacterized protein	Unconventional myosin-Ic	92	3	0.8285	40	99	46
P63169	F8VRV5	DYNLL1	Dynein light chain 1, cytoplasmic	Dynein light chain	100	2	0.8285	2	14	43
G1SV13	P22314	UBA1	Ubiquitin-like modifier-activating enzyme 1	Ubiquitin-like modifier-activating enzyme 1	97	2	0.8266	30	75	48
G1SS33	P36543	ATP6V1E1	Uncharacterized protein	V-type proton ATPase subunit E 1	99	3	0.8266	11	17	58
G1SP89	J3QLD9	FLOT2	PHB domain-containing protein	Flotillin-2	97	2	0.8266	11	16	34
G1TP30	H0Y4Q3	RANGAP1	RanGAP1_C domain-containing protein	Ran GTPase-activating protein 1 (Fragment)	80	2	0.8266	7	20	20
G1SUY5	E7EVZ5	PCYOX1L	Prenylcys_lyase domain-containing protein	Prenylcysteine oxidase-like	95	2	0.8266	4	8	15
	A0A024RBG1	NUDT4B		Diphosphoinositol polyphosphate phosphohydrolase NUDT4B		4	0.8257	2	2	21
G1T4A5	P02452	COL1A1	Collagen alpha-1(I) chain	Collagen alpha-1(I) chain	91	2	0.8239	34	27	45
G1SSF0	A3KFL2	EXOSC2	Uncharacterized protein	Exosome complex component RRP4	92	3	0.8239	3	2	15
	A0A0J9YXC7	LIMS4		LIM and senescent cell antigen-like-containing domain protein		4	0.8239	2	4	7
U3KMI4	P62834	RAP1A	Uncharacterized protein	Ras-related protein Rap-1A	100	3	0.8230	8	2	65

G1SMM5	O60884	DNAJA2	Uncharacterized protein	DnaJ homolog subfamily A member 2	100	3	0.8230	6	13	29
	Q9UNE7	STUB1		E3 ubiquitin-protein ligase CHIP		4	0.8230	2	2	9
G1SML4	P28288	ABCD3	Uncharacterized protein	ATP-binding cassette sub-family D member 3	96	3	0.8220	10	10	21
G1SG41		TBL2	WD_REPEATS_REGION domain-containing protein			1	0.8220	5	6	11
G1T6P0	Q9H7D0	DOCK5	Dedicator of cytokinesis 5	Dedicator of cytokinesis protein 5	96	2	0.8220	3	4	3
G1SLQ4	B4DXW1	ACTR3	Uncharacterized protein	Actin-related protein 3	100	3	0.8193	17	43	71
G1T7B1	Q9H3P7	ACBD3	Uncharacterized protein	Golgi resident protein GCP60	96	3	0.8193	7	7	21
G1SG59	Q7L9L4	MOB1B	Uncharacterized protein	MOB kinase activator 1B	100	3	0.8193	2	7	15
G1SK00		USP5	Ubiquitin carboxyl-terminal hydrolase			1	0.8184	21	41	38
G1SS73	Q92499	DDX1	Uncharacterized protein	ATP-dependent RNA helicase DDX1	98	3	0.8184	20	41	37
G1SDY5	P63104	YWHAZ	14_3_3 domain-containing protein	14-3-3 protein zeta/delta	100	2	0.8184	15	120	65
G1SIH3	A0A2R8Y5A6	ATXN2	Uncharacterized protein	Ataxin-2	94	3	0.8184	5	9	5
G1SIT5	F2Z388	RPL35	Uncharacterized protein	60S ribosomal protein L35	93	3	0.8184	3	5	25
G1T545		BTBD9	Lactoylglutathione lyase			1	0.8174	4	6	33
G1SIL8	A0A087WWM4	GMPR2	GMP reductase	GMP reductase	90	2	0.8165	3	4	13
G1SCL6	A0A494C1J1	SPECC1L	Calponin-homology (CH) domain-containing protein	Cytospin-A	90	2	0.8156	12	15	15
G1T8X7	Q96JH7	VCPIP1	OTU domain-containing protein	Deubiquitinating protein VCIP135	96	2	0.8156	2	3	2
G1TJY2		CHID1	Chitinase domain containing 1			1	0.8147	10	19	53
G1SFH6	Q9UBT2	UBA2	Uncharacterized protein	SUMO-activating enzyme subunit 2	97	3	0.8147	10	9	27
G1TVU4	Q96D15	RCN3	Reticulocalbin 3	Reticulocalbin-3	78	2	0.8147	9	39	51
G1SMT7		NHLRC2	Thioredoxin domain-containing protein			1	0.8147	3	3	9
G1T0B4	Q9Y3B3	TMED7	GOLD domain-containing protein	Transmembrane emp24 domain-containing protein 7	92	2	0.8137	7	20	52
G1T2I6	P45877	PPIC	Peptidyl-prolyl cis-trans isomerase	Peptidyl-prolyl cis-trans isomerase C	92	2	0.8137	6	24	48
G1SQ80	Q8TDZ2	MICAL1	Uncharacterized protein	[F-actin]-monooxygenase MICAL1	83	3	0.8137	4	4	8
G1T6P5	Q9NVJ2	ARL8B	ADP ribosylation factor like GTPase 8B	ADP-ribosylation factor-like protein 8B	100	2	0.8128	7	31	45
G1U3B8	K7ER96	TXNL1	PITH domain-containing protein	Thioredoxin-like protein 1 (Fragment)	100	2	0.8128	5	5	30
G1SD02	E7EM64	COPS6	COP9 signalosome subunit 6	COP9 signalosome complex subunit 6	97	2	0.8110	3	5	11
	A0A494C1N0	FKBP2		Peptidylprolyl isomerase		4	0.8101	2	7	17
	Q9UBF2	COPG2		Coatomer subunit gamma-2		4	0.8091	12	3	18
G1TDI6		BLOC1S6	Biogenesis of lysosome-related organelles complex 1 subunit 6			1	0.8091	2	2	25
G1SFU4	Q9UIQ6	LNPEP	Uncharacterized protein	Leucyl-cystinyl aminopeptidase	90	3	0.8082	14	20	17
G1SL53	Q66K14	TBC1D9B	Uncharacterized protein	TBC1 domain family member 9B	89	3	0.8073	3	2	3
P11974-2		PKM	Isoform M2 of Pyruvate kinase PKM			1	0.8064	38	464	81
G1SUX1		TIMP3	Metalloproteinase inhibitor 3			1	0.8064	2	2	13
G1SWK8	Q5TBG5	PSMB7	Proteasome subunit beta	Proteasome subunit beta (Fragment)	84	2	0.8055	6	61	25
P27115		MGAT1	Alpha-1,3-mannosyl-glycoprotein 2-beta-N-acetylglucosaminyltransferase			1	0.8055	3	3	12
G1T0G0		P3H2	Fe2OG dioxygenase domain-containing protein			1	0.8055	2	3	4
G1SP48	O60701	UGDH	UDP-glucose 6-dehydrogenase	UDP-glucose 6-dehydrogenase	96	2	0.8045	17	4	51
G1T5J8	Q9UJW0	DCTN4	Uncharacterized protein	Dynactin subunit 4	97	3	0.8027	6	8	29
G1T2L1	A0A024RA52	PSMA2	Proteasome subunit alpha type	Proteasome subunit alpha type	100	2	0.8018	11	31	59
G1SZN0	Q9NZB2	FAM120A	Uncharacterized protein	Constitutive coactivator of PPAR-gamma-like protein 1	94	3	0.8018	5	6	6
G1T332		GOT1	Aspartate aminotransferase			1	0.8018	3	4	10
G1SXB6	B4DVA9	POGLUT1	CAP10 domain-containing protein	Protein O-glucosyltransferase 1	96	2	0.8018	3	3	14
G1TBW9	Q96FJ2	DYNLL2	Dynein light chain	Dynein light chain 2, cytoplasmic	100	2	0.8018	2	12	33
G1SMI4	Q15276	RABEP1	Uncharacterized protein	Rab GTPase-binding effector protein 1	96	3	0.8008	2	3	4
	A0A2U3U034	ARSB		Arylsulfatase B		4	0.7999	4	5	12
G1SWN1	Q96H20	SNF8	Vacuolar-sorting protein SNF8	Vacuolar-sorting protein SNF8	100	2	0.7999	3	3	24
G1SXN1	Q9BXB4	OSBPL11	Oxysterol-binding protein	Oxysterol-binding protein-related protein 11	93	2	0.7999	2	4	4
	Q92696	RABGGTA		Geranylgeranyl transferase type-2 subunit alpha		4	0.7999	2	2	6
	P35908	KRT2		Keratin, type II cytoskeletal 2 epidermal		4	0.7990	10	7	25
G1T9V6	Q9UPU5	USP24	Ubiquitin specific peptidase 24	Ubiquitin carboxyl-terminal hydrolase 24	98	2	0.7990	7	7	5
G1T6W7	P04040	CAT	Catalase	Catalase	91	2	0.7990	4	4	16
G1T147	Q8IZ52	CHPF	Hexosyltransferase	Chondroitin sulfate synthase 2	95	2	0.7990	3	3	12
P21195	P07237	P4HB	Protein disulfide-isomerase	Protein disulfide-isomerase	91	2	0.7981	34	674	72
G1TCW5	A0A494C165	PEPD	Peptidase D	Xaa-Pro dipeptidase (Fragment)	79	2	0.7972	7	11	22
G1SJ20	A0A1W2PNV3	GOSR2	Uncharacterized protein	Golgi SNAP receptor complex member 2 (Fragment)	92	3	0.7972	5	10	37
O46638	Q00688	FKBP3	Peptidyl-prolyl cis-trans isomerase FKBP3	Peptidyl-prolyl cis-trans isomerase FKBP3	96	2	0.7972	4	7	21

G1SLF1		ADH5	S-(hydroxymethyl)glutathione dehydrogenase			1	0.7962	4	4	12
G1TJC8		PEX5	TPR_REGION domain-containing protein			1	0.7962	2	2	5
G1T9D6	C9J5C3	PDCD10	Uncharacterized protein	Programmed cell death protein 10 (Fragment)	99	3	0.7953	7	8	48
G1SD27	B7ZC38	SH3GLB2	SH3 domain containing GRB2 like, endophilin B2	Endophilin-B2	92	2	0.7953	2	2	6
G1SXQ0	A0A0A0MTN3	GSTM3	Glutathione S-transferase	Glutathione S-transferase	90	2	0.7944	10	23	37
G1SN95	A0A087WSV8	NUCB2	Nucleobindin 2	Nucleobindin 2, isoform CRA_b	93	2	0.7935	18	36	47
G1SRJ6		AFAP1	Actin filament associated protein 1			1	0.7935	3	6	7
G1T7R2	P62258	YWHAE	14_3_3 domain-containing protein	14-3-3 protein epsilon	100	2	0.7925	18	195	78
G1T369	C9J8R4	DCUN1D1	DCN1-like protein	DCN1-like protein (Fragment)	100	2	0.7925	2	3	13
G1T3K1		DESI2	DUF862 domain-containing protein			1	0.7925	2	3	29
G1SXS3	Q13573	SNW1	SKIP_SNW domain-containing protein	SNW domain-containing protein 1	100	2	0.7925	2	2	8
G1SN14	Q86VP6	CAND1	TIP120 domain-containing protein	Cullin-associated NEDD8-dissociated protein 1	100	2	0.7916	32	53	36
Q09YN4	P47755	CAPZA2	F-actin-capping protein subunit alpha-2	F-actin-capping protein subunit alpha-2	98	2	0.7916	8	15	50
G1TM48	Q5JRA6	MIA3	SH3 domain-containing protein	Transport and Golgi organization protein 1 homolog	71	2	0.7916	5	5	3
G1SJH1	H7BXE3	SLTM	RRM domain-containing protein	SAFB-like transcription modulator (Fragment)	92	2	0.7916	3	3	4
G1SIB0	Q12907	LMAN2	L-type lectin-like domain-containing protein	Vesicular integral-membrane protein VIP36	98	2	0.7907	6	15	23
G1TKH3		SOD1	Superoxide dismutase [Cu-Zn]			1	0.7907	3	119	35
G1T3V0	A0A024R4E5	HDLBP	Uncharacterized protein	High density lipoprotein binding protein (Vigilin), isoform CRA_a	97	3	0.7898	45	107	47
G1U800	Q8NC56	LEMD2	MSC domain-containing protein	LEM domain-containing protein 2	79	2	0.7898	5	3	19
G1SY00	P42858	HTT	Uncharacterized protein	Huntingtin	91	3	0.7898	4	4	3
G1SCW0	A8MT72	RTN1	Reticulon	Reticulon	98	2	0.7898	2	3	3
G1SKL7	Q99536	VAT1	Vesicle amine transport 1	Synaptic vesicle membrane protein VAT-1 homolog	78	2	0.7889	9	17	35
G1T087	Q93034	CUL5	Cullin-5	Cullin-5	100	2	0.7889	4	15	10
G1T2K6	Q8NBU5	ATAD1	AAA domain-containing protein	ATPase family AAA domain-containing protein 1	100	2	0.7889	2	6	10
G1U5A6	A0A286YFF8	MON2	Uncharacterized protein	Protein MON2 homolog	98	3	0.7879	6	7	6
P47823		EIF2B5	Translation initiation factor eIF-2B subunit epsilon			1	0.7879	5	9	14
G1T8X3		NEU1	Sialidase domain-containing protein			1	0.7879	4	6	18
U3KP45		SYNE1	Spectrin repeat containing nuclear envelope protein 1			1	0.7879	4	6	1
G1U826	O75348	ATP6V1G1	V-type proton ATPase subunit G	V-type proton ATPase subunit G 1	97	2	0.7879	3	7	39
G1U8F0	O95782	AP2A1	AP-2 complex subunit alpha	AP-2 complex subunit alpha-1	98	2	0.7870	32	5	46
G1SDL3	P54802	NAGLU	Uncharacterized protein	Alpha-N-acetylglucosaminidase	88	3	0.7861	15	19	29
G1SZW8	M0R165	EPS15L1	Epidermal growth factor receptor pathway substrate 15 like 1	Epidermal growth factor receptor substrate 15-like 1	88	2	0.7861	9	12	16
G1TZV3	E7ENJ6	AP1M1	Adaptor related protein complex 1 subunit mu 1	AP-1 complex subunit mu-1	75	2	0.7861	8	14	33
G1TXW6		GPNMB	PKD domain-containing protein			1	0.7861	2	5	5
G1SHF1	Q9UHV9	PFDN2	Uncharacterized protein	Prefoldin subunit 2	99	3	0.7852	4	8	36
G1T5Z7	B3KR49	MAPK3	Mitogen-activated protein kinase	Mitogen-activated protein kinase 3	97	2	0.7843	6	3	37
G1SJC7	Q9UN86	G3BP2	Uncharacterized protein	Ras GTPase-activating protein-binding protein 2	100	3	0.7843	5	6	10
G1SLA2	Q92604	LPGAT1	Lysophosphatidylglycerol acyltransferase 1	Acyl-CoA:lysophosphatidylglycerol acyltransferase 1	94	2	0.7843	5	7	17
G1TD36	Q6PML9	SLC30A9	Uncharacterized protein	Zinc transporter 9	96	3	0.7843	4	4	8
G1TCM0	J3KQ34	COPS7B	PCI domain-containing protein	COP9 signalosome complex subunit 7b	97	2	0.7833	3	7	19
G1U7C5	Q9P2E9	RRBP1	Uncharacterized protein	Ribosome-binding protein 1	83	3	0.7824	44	103	38
G1THP8	P46734	MAP2K3	Protein kinase domain-containing protein	Dual specificity mitogen-activated protein kinase kinase 3	97	2	0.7824	6	8	29
G1SXY5		STX2	Syntaxin 2			1	0.7815	3	4	21
U3KMP1	P61026	RAB10	Uncharacterized protein	Ras-related protein Rab-10	100	3	0.7806	9	10	54
G1TVG8	O15173	PGRMC2	Cytochrome b5 heme-binding domain-containing protein	Membrane-associated progesterone receptor component 2	97	2	0.7806	8	10	33
G1TAM3		TBCB	CAP-Gly domain-containing protein			1	0.7796	2	2	11
G1T108	Q9NRG9	AAAS	WD_REPEATS_REGION domain-containing protein	Aladin	94	2	0.7787	6	8	17
G1T295		EPHX1	Epoxide hydrolase			1	0.7787	5	6	15
G1SSN9	Q96S59	RANBP9	Uncharacterized protein	Ran-binding protein 9	97	3	0.7787	4	5	15
G1TGK3	B4DP31	PRPSAP1	Pribosyltran_N domain-containing protein	Phosphoribosyl pyrophosphate synthase-associated protein 1	99	2	0.7787	3	3	13
G1SP36	Q9HAV4	XPO5	Importin N-terminal domain-containing protein	Exportin-5	95	2	0.7778	3	6	7
G1T9Y4	E5RIU9	CHMP7	Uncharacterized protein	Charged multivesicular body protein 7 (Fragment)	69	3	0.7778	2	3	9
G1TBH6	Q9P0S9	TMEM14C	Uncharacterized protein	Transmembrane protein 14C	92	3	0.7769	3	3	66
U3KNB6	P48444	ARCN1	Coatomer subunit delta	Coatomer subunit delta	97	2	0.7750	18	34	40
G1T860	M0R0P8	MYO9B	Myosin IXB	Unconventional myosin-IXb	83	2	0.7750	13	11	13
G1SP51	P62277	RPS13	Ribosomal_S13_N domain-containing protein	40S ribosomal protein S13	100	2	0.7750	8	16	46
G1TEW4	Q93008	USP9X	USP domain-containing protein	Probable ubiquitin carboxyl-terminal hydrolase FAF-X	99	2	0.7741	35	57	21

G1T0Y9	Q13561	DCTN2	Uncharacterized protein	Dynactin subunit 2	97	3	0.7732	10	26	43
G1T358	Q96AG3	SLC25A46	Uncharacterized protein	Solute carrier family 25 member 46	94	3	0.7732	3	5	18
G1T8E2	A0A3B3ITW1	GSK3B	Protein kinase domain-containing protein	Glycogen synthase kinase-3 beta	87	2	0.7732	2	3	10
G1SWC9	H3BMU1	IST1	Uncharacterized protein	IST1 homolog (Fragment)	100	3	0.7723	5	7	21
G1SIN4	X6RLX0	ERC1	FIP-RBD domain-containing protein	ELKS/Rab6-interacting/CAST family member 1	98	2	0.7714	14	20	15
G1SL52	A0A0A0MS45	COG4	Cog4 domain-containing protein	Conserved oligomeric Golgi complex subunit 4	94	2	0.7714	10	13	19
G1SR27	P62993	GRB2	Uncharacterized protein	Growth factor receptor-bound protein 2	100	3	0.7714	3	5	22
G1SIT9	P31946	YWHAB	14_3_3 domain-containing protein	14-3-3 protein beta/alpha	100	2	0.7704	16	53	76
G1SS79	P29966	MARCKS	Uncharacterized protein	Myristoylated alanine-rich C-kinase substrate	87	3	0.7695	4	9	25
G1T4K5	A0A1B0GTW1	TJP2	Uncharacterized protein	Tight junction protein ZO-2	87	3	0.7677	5	5	8
	O14617	AP3D1		AP-3 complex subunit delta-1		4	0.7658	8	12	9
G1TNY9		TRADD	Death domain-containing protein			1	0.7658	2	3	10
	A0A3B3ISV4	VKORC1L1		Vitamin K epoxide reductase complex subunit 1-like protein 1		4	0.7658	2	3	9
Q29502	Q13177	PAK2	Serine/threonine-protein kinase PAK 2	Serine/threonine-protein kinase PAK 2	98	2	0.7640	8	5	25
G1SYK3	P45985	MAP2K4	Protein kinase domain-containing protein	Dual specificity mitogen-activated protein kinase kinase 4	99	2	0.7640	3	5	10
G1U0Y0	A0A2Q3DQE3	CAMK2G	Protein kinase domain-containing protein	Calcium/calmodulin-dependent protein kinase (CaM kinase) II gamma, isoform CRA_d	91	2	0.7631	7	5	23
G1SMS3	P61160	ACTR2	Actin-related protein 2	Actin-related protein 2	100	2	0.7621	13	111	48
G1T7N4	P41743	PRKCI	Protein kinase C	Protein kinase C iota type	99	2	0.7621	2	3	7
G1SFZ8	Q9UMX0	UBQLN1	Ubiquilin 1	Ubiquilin-1	86	2	0.7612	8	12	26
G1T3U1	Q9H8Y8	GORASP2	GRASP55_65 domain-containing protein	Golgi reassembly-stacking protein 2	91	2	0.7612	6	15	18
G1SR93	B8ZZA2	FAM126A	Uncharacterized protein	Hyccin	98	3	0.7612	2	2	6
G1SYV9	Q9Y490	TLN1	Uncharacterized protein	Talin-1	99	3	0.7603	108	42	61
G1T1V0	P13645	KRT10	IF rod domain-containing protein	Keratin, type I cytoskeletal 10	95	2	0.7594	22	59	51
G1SCT9	A0A087WU53	MAGT1	Uncharacterized protein	Magnesium transporter protein 1	97	3	0.7594	7	8	24
G1SNE1	Q6DKJ4	NXN	Thioredoxin domain-containing protein	Nucleoredoxin	98	2	0.7585	8	24	30
G1U9U1	P50995	ANXA11	Annexin	Annexin A11	92	2	0.7585	7	9	17
G1TP81	Q9Y6W5	WASF2	WH2 domain-containing protein	Wiskott-Aldrich syndrome protein family member 2	94	2	0.7585	4	6	10
G1TIW9		FBXO6	F-box protein 6			1	0.7585	3	3	11
G1SLD6	B3KWE1	HARS	Uncharacterized protein	Histidine--tRNA ligase, cytoplasmic	97	3	0.7566	7	9	19
G1SWM7	Q5W0J6	ECHDC3	Uncharacterized protein	Enoyl-CoA hydratase domain-containing protein 3, mitochondrial (Fragment)	90	3	0.7566	2	4	9
G1SZP4	I6L894	ANK2	Ankyrin 2	Ankyrin-2	88	2	0.7557	3	3	1
G1T8U2		GMDS	GDP-mannose 4,6-dehydratase			1	0.7557	2	3	13
G1SN09	Q9Y5P6	GMPPB	NTP_transferase domain-containing protein	Mannose-1-phosphate guanyltransferase beta	99	2	0.7548	4	4	22
G1SPM5	P61163	ACTR1A	Uncharacterized protein	Alpha-centractin	100	3	0.7538	11	18	40
Q9XS70	Q9BR76	CORO1B	Coronin-1B	Coronin-1B	93	2	0.7538	9	27	27
G1T2F8	H0YJH8	ATP6V1D	V-type proton ATPase subunit D	V-type proton ATPase subunit D (Fragment)	99	2	0.7538	3	15	23
G1T4S5	Q8NFW8	CMAS	Uncharacterized protein	N-acylneuraminate cytidylyltransferase	96	3	0.7529	5	5	11
G1SGR0	Q8N3P4	VPS8	Uncharacterized protein	Vacuolar protein sorting-associated protein 8 homolog	93	3	0.7520	2	3	3
G1TPW2		MRI1	Methylthioribose-1-phosphate isomerase			1	0.7511	4	7	22
	O43765	SGTA		Small glutamine-rich tetratricopeptide repeat-containing protein alpha		4	0.7511	2	3	8
G1SCI0	Q14204	DYNC1H1	Dynein cytoplasmic 1 heavy chain 1	Cytoplasmic dynein 1 heavy chain 1	98	2	0.7492	121	73	52
G1TSK4		AARS2	AA_TRNA_LIGASE_II_ALA domain-containing protein			1	0.7492	2	3	4
G1SMK9	Q9ULC3	RAB23	Uncharacterized protein	Ras-related protein Rab-23	97	3	0.7483	4	8	22
G1SZU0		URB2	Urb2 domain-containing protein			1	0.7483	2	2	2
G1TF32	F5H6I7	ATL3	Atlastin GTPase 3	Atlastin-3	95	2	0.7474	15	45	44
G1SZD6	P27348	YWHAQ	14-3-3 protein theta	14-3-3 protein theta	99	2	0.7465	16	121	68
	A8MZF9	DRG2		Developmentally-regulated GTP-binding protein 2		4	0.7465	3	6	14
G1SP97	P51884	LUM	Lumican	Lumican	91	2	0.7455	4	7	19
G1TC19		CPT2	Carnitine palmitoyltransferase 2			1	0.7446	2	2	5
G1SUP1	A0A0D9SEY1	MAP4K4	Mitogen-activated protein kinase kinase kinase kinase 4	Mitogen-activated protein kinase kinase kinase kinase 4	91	2	0.7437	6	6	7
G1TDN4	P10644	PRKAR1A	Uncharacterized protein	cAMP-dependent protein kinase type I-alpha regulatory subunit	99	3	0.7437	4	8	17
G1TB45	Q86W92	PPFIBP1	Uncharacterized protein	Liprin-beta-1	87	3	0.7419	22	38	33
G1T4C9	Q8IVL6	P3H3	Prolyl 3-hydroxylase 3	Prolyl 3-hydroxylase 3	88	2	0.7419	14	24	29
G1STX3	Q8WVM8	SCFD1	Uncharacterized protein	Sec1 family domain-containing protein 1	96	3	0.7409	16	25	42
G1SQF7	Q14554	PDIA5	Uncharacterized protein	Protein disulfide-isomerase A5	92	3	0.7409	10	18	29
G1T090	G3V180	DPP3	Dipeptidyl peptidase 3	Dipeptidyl peptidase 3	94	2	0.7409	9	17	24
G1SH25	P40616	ARL1	Uncharacterized protein	ADP-ribosylation factor-like protein 1	99	3	0.7409	5	16	38

G1TSV3	Q8ND76	CCNY	Cyclin Y	Cyclin-Y	99	2	0.7409	2	3	9
G1SGY2	H0YC15	PTPN12	Tyrosine-protein phosphatase non-receptor type 12	Tyrosine-protein phosphatase non-receptor type 12 (Fragment)	90	2	0.7409	2	3	21
G1SF82	P62072	TIMM10	zf-Tim10_DDP domain-containing protein	Mitochondrial import inner membrane translocase subunit Tim10	100	2	0.7409	2	5	36
G1SZX4	Q14571	ITPR2	Uncharacterized protein	Inositol 1,4,5-trisphosphate receptor type 2	97	3	0.7400	13	11	9
Q28717	F6WIT2	PTPA	Serine/threonine-protein phosphatase 2A activator	Serine/threonine-protein phosphatase 2A activator	97	2	0.7400	8	11	41
G1TB18	Q07960	ARHGAP1	Uncharacterized protein	Rho GTPase-activating protein 1	95	3	0.7400	5	8	16
	J3KR44	OTUB1		Ubiquitin thioesterase		4	0.7400	5	8	34
G1SHH1		PPOX	Protoporphyrinogen oxidase			1	0.7400	2	2	8
G1TMQ8	H0Y9V7	ATP2C1	Calcium-transporting ATPase	Calcium-transporting ATPase type 2C member 1 (Fragment)	99	2	0.7391	4	5	9
G1SXG8	F8VU90	FKBP11	Peptidylprolyl isomerase	Peptidylprolyl isomerase	95	2	0.7382	4	9	31
P63150	P63151	PPP2R2A	Serine/threonine-protein phosphatase 2A 55 kDa regulatory subunit B alpha isoform	Serine/threonine-protein phosphatase 2A 55 kDa regulatory subunit B alpha isoform	100	2	0.7382	4	7	15
G1TUH9	Q14195	DPYSL3	Amidohydro-rel domain-containing protein	Dihydropyrimidinase-related protein 3	98	2	0.7373	17	24	38
G1SJB9	Q14257	RCN2	Reticulocalbin 2	Reticulocalbin-2	90	2	0.7373	12	126	56
G1SJ77	Q9Y696	CLIC4	Chloride intracellular channel protein	Chloride intracellular channel protein 4	100	2	0.7363	11	39	58
G1TV43	O60218	AKR1B10	Aldo_ket_red domain-containing protein	Aldo-keto reductase family 1 member B10	86	2	0.7363	10	17	31
G1TI71	A0A087WYS1	UGP2	UTP--glucose-1-phosphate uridylyltransferase	UTP--glucose-1-phosphate uridylyltransferase	99	2	0.7354	24	64	62
G1SQ12	Q8TDJ6	DMXL2	WD_REPEATS_REGION domain-containing protein	DmX-like protein 2	93	2	0.7354	2	2	1
G1TBA4	Q86UY8	NT5DC3	Uncharacterized protein	5~-nucleotidase domain-containing protein 3	97	3	0.7336	9	15	26
G1SDZ0		CTSA	Carboxypeptidase			1	0.7336	5	7	11
G1T933	Q05707	COL14A1	Uncharacterized protein	Collagen alpha-1(XIV) chain	94	3	0.7326	34	64	27
G1U8V2	Q96DZ1	ERLEC1	Uncharacterized protein	Endoplasmic reticulum lectin 1	98	3	0.7326	3	6	8
G1SS77	Q15477	SKIV2L	Uncharacterized protein	Helicase SKI2W	95	3	0.7317	7	9	7
G1SD49	X6RCK5	DCTN3	Uncharacterized protein	Dynactin subunit 3 (Fragment)	82	3	0.7317	4	5	24
U3KMU7	A0A024R571	EHD1	EH domain containing 1	EH domain-containing protein 1	99	2	0.7308	15	15	35
G1T9N3	B5MBZ0	EML4	Uncharacterized protein	Echinoderm microtubule-associated protein-like 4	92	3	0.7308	4	7	5
	M0QXB5	ETHE1		Persulfide dioxygenase ETHE1, mitochondrial		4	0.7308	2	2	14
G1U998		NOL6	Nucleolar protein 6			1	0.7299	3	11	7
G1SIP6		CISD1	ZnF_CDGSH domain-containing protein			1	0.7299	2	2	20
G1SHS7	O75083	WDR1	WD_REPEATS_REGION domain-containing protein	WD repeat-containing protein 1	94	2	0.7290	21	50	51
G1SZ34	Q9Y5S2	CDC42BPB	CDC42 binding protein kinase beta	Serine/threonine-protein kinase MRCK beta	92	2	0.7290	10	10	11
G1SHI0	H0Y4D4	ACAA1	Acetyl-CoA acyltransferase 1	3-ketoacyl-CoA thiolase, peroxisomal (Fragment)	62	2	0.7290	8	7	34
G1SJ61	Q9Y608	LRRFIP2	Uncharacterized protein	Leucine-rich repeat flightless-interacting protein 2	91	3	0.7290	7	9	13
G1SL68	P35579	MYH9	Uncharacterized protein	Myosin-9	96	3	0.7280	119	268	57
G1STY4		PLBD2	Phospholipase B-like			1	0.7280	5	15	16
G1TA10		TMEM109	Transmembrane protein 109			1	0.7280	3	6	9
G1TB49		APOA1BP	NAD(P)H-hydrate epimerase			1	0.7271	2	5	15
G1TYL5		DNPH1	2~-deoxynucleoside 5~-phosphate N-hydrolase 1			1	0.7271	2	2	31
P00939	P60174	TPI1	Triosephosphate isomerase	Triosephosphate isomerase	93	2	0.7262	15	59	72
G1SVH8	Q13636	RAB31	Uncharacterized protein	Ras-related protein Rab-31	95	3	0.7262	6	8	36
G1SX70	Q9NRW1	RAB6B	Uncharacterized protein	Ras-related protein Rab-6B	100	3	0.7225	7	2	45
G1SWW6	F5H459	AP3S1	Clat_adaptor_s domain-containing protein	AP complex subunit sigma	95	2	0.7216	2	3	14
G1TPN2	K7EQA9	CDC37	Cell division cycle 37	Hsp90 co-chaperone Cdc37 (Fragment)	57	2	0.7207	5	2	17
U3KMD1	A0A0A0MRI2	SNX6	Vps5 domain-containing protein	Sorting nexin	99	2	0.7197	11	106	28
	P17612	PRKACA		cAMP-dependent protein kinase catalytic subunit alpha		4	0.7197	6	4	27
G1T284	Q8TCJ2	STT3B	Uncharacterized protein	Dolichyl-diphosphooligosaccharide--protein glycosyltransferase subunit STT3B	100	3	0.7197	5	2	9
P41982	P04179	SOD2	Superoxide dismutase [Mn], mitochondrial (Fragment)	Superoxide dismutase [Mn], mitochondrial	92	2	0.7197	4	23	25
G1TS42		AGL	Glycogen debranching enzyme			1	0.7197	3	2	4
G1SVG6	P21359	NF1	Uncharacterized protein	Neurofibromin	99	3	0.7179	4	6	3
	P62854	RPS26		40S ribosomal protein S26		4	0.7179	2	11	21
G1T1D7	B1ALD9	POSTN	Uncharacterized protein	Periostin	90	3	0.7170	16	36	34
	A0A0A0MT60	FKBP15		Peptidylprolyl isomerase (Fragment)		4	0.7170	3	3	3
	A0A087WY85	UBE2D3		Ubiquitin-conjugating enzyme E2 D3		4	0.7170	2	4	20
G1T2Z5	A0A087WTA8	COL1A2	Collagen alpha-2(I) chain	Collagen alpha-2(I) chain	94	2	0.7161	28	170	33
G1SZM0	Q9BRT3	MIEN1	Uncharacterized protein	Migration and invasion enhancer 1	95	3	0.7161	2	2	16
G1SDU1	Q96A49	SYAP1	BSD domain-containing protein	Synapse-associated protein 1	89	2	0.7161	2	5	14
	Q08209-2	PPP3CA		Isoform 2 of Serine/threonine-protein phosphatase 2B catalytic subunit alpha isoform		4	0.7151	9	16	27
G1SMF4	Q9UHP3	USP25	USP domain-containing protein	Ubiquitin carboxyl-terminal hydrolase 25	94	2	0.7151	4	6	6

G1TUC2	P62633	CNBP	Uncharacterized protein	Cellular nucleic acid-binding protein	100	3	0.7151	2	4	13
G1SUK0	O75940	SMNDC1	Tudor domain-containing protein	Survival of motor neuron-related-splicing factor 30	99	2	0.7142	2	2	13
G1SE57	P18085	ARF4	Uncharacterized protein	ADP-ribosylation factor 4	95	3	0.7133	10	20	76
G1U886	A0A2R8YGH5	AP1S1	AP complex subunit sigma	AP complex subunit sigma	100	2	0.7133	4	3	33
G1TXN1		NIT2	CN hydrolase domain-containing protein			1	0.7133	3	3	14
G1TZP0	P61981	YWHAG	14_3_3 domain-containing protein	14-3-3 protein gamma	99	2	0.7124	13	54	70
G1U446	Q14677	CLINT1	ENTH domain-containing protein	Clathrin interactor 1	97	2	0.7124	9	15	17
G1SMA1	P49755	TMED10	Transmembrane emp24 domain-containing protein 10	Transmembrane emp24 domain-containing protein 10	95	2	0.7124	8	291	41
G1SQR6	Q15417	CNN3	Calponin	Calponin-3	98	2	0.7115	15	510	55
G1TWQ3		SIRT2	NAD-dependent protein deacetylase			1	0.7105	5	5	19
G1SVH0	P23526	AHCY	AdoHcyase_NAD domain-containing protein	Adenosylhomocysteinase	97	2	0.7096	15	43	36
G1SQ02	A0A0A0MSI0	PRDX1	Thioredoxin domain-containing protein	Peroxiredoxin-1 (Fragment)	98	2	0.7096	10	17	57
	Q8WXF7-2	ATL1		Isoform 2 of Atlastin-1		4	0.7096	5	8	15
G1TEU5	O75131	CPNE3	Uncharacterized protein	Copine-3	96	3	0.7096	3	3	7
G1T4H0	Q9NYL9	TMOD3	Uncharacterized protein	Tropomodulin-3	93	3	0.7087	15	27	50
G1SCZ9	Q92575	UBXN4	UBX domain-containing protein	UBX domain-containing protein 4	94	2	0.7087	4	6	14
G1SXT1	O43865	AHCYL1	AdoHcyase_NAD domain-containing protein	S-adenosylhomocysteine hydrolase-like protein 1	100	2	0.7087	3	6	7
G1STQ7		TMEM97	Transmembrane protein 97			1	0.7087	2	2	12
G1T5I3	A0A0C4DFM1	TM9SF4	Transmembrane 9 superfamily member	Transmembrane 9 superfamily member	100	2	0.7078	4	8	10
G1STX4	P61011	SRP54	Signal recognition particle 54 kDa protein	Signal recognition particle 54 kDa protein	99	2	0.7068	13	16	38
G1TB17	A0A1B0GWF8	ADSL	Adenylosuccinate lyase	Adenylosuccinate lyase (Fragment)	95	2	0.7068	4	3	13
G1TIZ5	Q8NBS9	TXNDC5	Uncharacterized protein	Thioredoxin domain-containing protein 5	87	3	0.7059	13	29	39
G1SJZ4	J3KQ32	OLA1	Obg-like ATPase 1	Obg-like ATPase 1	100	2	0.7059	9	19	34
G1SE87		ARSE	Arylsulfatase E			1	0.7059	3	3	7
G1U0Q7		SEPT2	Septin-type G domain-containing protein			1	0.7050	13	59	54
G1T2T9	P20339	RAB5A	Uncharacterized protein	Ras-related protein Rab-5A	99	3	0.7050	8	11	54
G1TR92		SYDE1	Synapse defective Rho GTPase homolog 1			1	0.7050	3	4	7
G1SKT1	Q7Z7H5	TMED4	GOLD domain-containing protein	Transmembrane emp24 domain-containing protein 4	94	2	0.7041	7	15	44
G1SNL4	Q9Y385	UBE2J1	UBIQUITIN_CONJUGAT_2 domain-containing protein	Ubiquitin-conjugating enzyme E2 J1	94	2	0.7041	3	3	14
G1T974	A0A3B3IRK6	MOGS	Mannosyl-oligosaccharide glucosidase	Mannosyl-oligosaccharide glucosidase (Fragment)	86	2	0.7022	9	12	20
G1T6E9	Q8N5K1	CISD2	ZnF_CDGSH domain-containing protein	CDGSH iron-sulfur domain-containing protein 2	99	2	0.7022	4	8	32
G1SP34	O15144	ARPC2	Arp2/3 complex 34 kDa subunit	Actin-related protein 2/3 complex subunit 2	100	2	0.7013	15	31	54
G1STP3	O75718	CRTAP	Uncharacterized protein	Cartilage-associated protein	94	3	0.7013	11	17	32
G1TJC3	Q9BUF5	TUBB6	Tubulin beta chain	Tubulin beta-6 chain	97	2	0.6995	17	24	57
G1SUV0	Q9NW15	ANO10	Anoctamin	Anoctamin-10	94	2	0.6995	5	13	12
G1T5H8	E7EX17	EIF4B	RRM domain-containing protein	Eukaryotic translation initiation factor 4B	93	2	0.6995	3	8	10
G1TBN2	Q96II5	ARAF	Uncharacterized protein	ARAF protein	95	3	0.6995	2	2	4
G1U101	J3KPF0	HECTD4	HECT domain-containing protein	Probable E3 ubiquitin-protein ligase HECTD4	97	2	0.6995	2	5	1
G1SY70	F5H365	SEC23A	Protein transport protein SEC23	Protein transport protein SEC23	96	2	0.6985	22	42	47
G1SNQ2	Q8WVF1	OSCP1	Uncharacterized protein	Protein OSCP1	87	3	0.6958	2	3	8
G1SE61	O75369	FLNB	Filamin-B	Filamin-B	97	2	0.6949	106	546	61
G1T8H8	P08183	ABCB1	Uncharacterized protein	ATP-dependent translocase ABCB1	88	3	0.6949	5	15	7
G1SDT0	Q562R1	ACTBL2	Uncharacterized protein	Beta-actin-like protein 2	97	3	0.6939	14	16	41
G1SY84	Q13409-2	DYNC1I2	WD_REPEATS_REGION domain-containing protein	Isoform 2B of Cytoplasmic dynein 1 intermediate chain 2	92	2	0.6930	9	2	29
G1TBY1		CTSB	Pept_C1 domain-containing protein			1	0.6930	7	63	30
G1TD38	H3BVG8	VPS35L	Uncharacterized protein	VPS35 endosomal protein sorting factor-like	93	3	0.6930	6	10	4
G1SN05	Q14240	EIF4A2	Uncharacterized protein	Eukaryotic initiation factor 4A-II	100	3	0.6921	14	15	49
G1T078	O43294	TGFB1I1	Uncharacterized protein	Transforming growth factor beta-1-induced transcript 1 protein	95	3	0.6921	4	7	11
G1U2Q7		COL8A1	Collagen alpha-1(VIII) chain			1	0.6921	3	4	5
G1TEA5	P36959	GMPR	GMP reductase	GMP reductase 1	97	2	0.6921	2	3	13
G1SHK8	O94925	GLS	ANK_REP_REGION domain-containing protein	Glutaminase kidney isoform, mitochondrial	97	2	0.6912	21	11	48
O77708	D6R938	CAMK2D	Calcium/calmodulin-dependent protein kinase type II subunit delta	Calcium/calmodulin-dependent protein kinase (CaM kinase) II delta, isoform CRA_e	92	2	0.6912	12	18	33
G1T8H1	O95573	ACSL3	AMP-binding domain-containing protein	Long-chain-fatty-acid--CoA ligase 3	96	2	0.6912	7	5	14
G1T107	P61088	UBE2N	UBIQUITIN_CONJUGAT_2 domain-containing protein	Ubiquitin-conjugating enzyme E2 N	100	2	0.6912	5	10	59
G1SNP9	P45954	ACADSB	Acyl-CoA dehydrogenase short/branched chain	Short/branched chain specific acyl-CoA dehydrogenase, mitochondrial	88	2	0.6903	18	144	55
G1SHX1	P68036	UBE2L3	Ubiquitin conjugating enzyme E2 L3	Ubiquitin-conjugating enzyme E2 L3	96	2	0.6903	6	12	57
G1SEH1		PLSCR3	Phospholipid scramblase			1	0.6903	2	6	13

G1SR49		SEPT10	Septin-type G domain-containing protein			1	0.6893	6	11	17
G1T0E5		SNAP23	Synaptosomal-associated protein			1	0.6893	3	4	23
G1TYW1		TPD52L2	TPD52 like 2			1	0.6893	3	5	13
G1SWR0	H3BP20	HEXA	Beta-hexosaminidase	Beta-hexosaminidase	85	2	0.6884	12	19	36
G1T275	Q8WU90	ZC3H15	Uncharacterized protein	Zinc finger CCCH domain-containing protein 15	98	3	0.6884	3	3	9
G1T5Q4	Q9H446	RWDD1	RWD domain-containing protein	RWD domain-containing protein 1	91	2	0.6875	4	7	27
G1SYY0	Q96SK2	TMEM209	Uncharacterized protein	Transmembrane protein 209	96	3	0.6875	3	6	12
G1TIZ2	Q02809	PLOD1	Procollagen-lysine,2-oxoglutarate 5-dioxygenase 1	Procollagen-lysine,2-oxoglutarate 5-dioxygenase 1	93	2	0.6866	26	19	41
G1TJ80	Q9Y3Q3	TMED3	Transmembrane p24 trafficking protein 3	Transmembrane emp24 domain-containing protein 3	91	2	0.6856	4	15	40
G1T967	A0A0U1RRB6	EXOC6B	Exocyst complex component	Exocyst complex component	99	2	0.6847	7	10	15
G1THV8	Q9BT09	CNPY3	DUF3456 domain-containing protein	Protein canopy homolog 3	92	2	0.6847	6	7	26
G1T1X2	A0A087WZF1	LPP	Uncharacterized protein	Lipoma-preferred partner	89	3	0.6847	4	5	8
G1T7D0		SRRM2	Serine/arginine repetitive matrix 2			1	0.6847	2	3	1
G1T0H7	A0A087WWM0	TRAPPC3	Trafficking protein particle complex subunit	Trafficking protein particle complex subunit	94	2	0.6838	4	8	27
G1TBU8	P52306	RAP1GDS1	Uncharacterized protein	Rap1 GTPase-GDP dissociation stimulator 1	97	3	0.6829	6	7	15
G1SQG5	P40925	MDH1	Malate dehydrogenase	Malate dehydrogenase, cytoplasmic	97	2	0.6820	12	16	57
G1T6Q9	O15400	STX7	t-SNARE coiled-coil homology domain-containing protein	Syntaxin-7	95	2	0.6810	7	18	48
G1THW3	A0A3B3IUC0	ITM2B	BRICHOS domain-containing protein	Integral membrane protein 2B	96	2	0.6810	5	9	31
U3KN73	Q9UBQ0	VPS29	Vacuolar protein sorting-associated protein 29	Vacuolar protein sorting-associated protein 29	100	2	0.6792	6	10	37
G1T1G5	Q9H4A6	GOLPH3	Uncharacterized protein	Golgi phosphoprotein 3	99	3	0.6792	5	6	32
G1SP83	Q9Y2D0	CA5B	Carbonic anhydrase 5B	Carbonic anhydrase 5B, mitochondrial	93	2	0.6783	10	33	46
	O14908	GIPC1		PDZ domain-containing protein GIPC1		4	0.6783	4	6	16
G1T7H6	Q6UX71	PLXDC2	PSI domain-containing protein	Plexin domain-containing protein 2	94	2	0.6783	4	10	9
G1U949	Q15691	MAPRE1	Uncharacterized protein	Microtubule-associated protein RP/EB family member 1	99	3	0.6774	8	24	43
G1SER8	P07737	PFN1	Profilin	Profilin-1	94	2	0.6774	7	133	75
G1SD48	A0A0G2JLB3	GBA	Glucosylceramidase	Glucosylceramidase	90	2	0.6774	5	8	20
	P21333	FLNA		Filamin-A		4	0.6764	105	13	51
G1U670	A0A087WW40	SH3GLB1	Uncharacterized protein	Endophilin-B1	95	3	0.6764	2	3	6
G1SFP0	P28838	LAP3	CYTOSOL_AP domain-containing protein	Cytosol aminopeptidase	92	2	0.6737	17	30	52
Q6SQH4	P60903	S100a10	Protein S100-A10	Protein S100-A10	100	2	0.6737	4	12	35
G1U9D3	Q9UK41	VPS28	Vacuolar protein sorting-associated protein 28 homolog	Vacuolar protein sorting-associated protein 28 homolog	93	2	0.6737	4	4	29
G1TU12	Q5H907	MAGED2	MAGE domain-containing protein	Melanoma antigen family D, 2, isoform CRA_d	79	2	0.6727	5	4	10
G1TMP7	A0A0D9SG72	STXBP1	Syntaxin binding protein 1	Syntaxin-binding protein 1	99	2	0.6718	4	4	10
G1T8R1		RAP1GAP2	Platelet-activating factor acetylhydrolase IB subunit alpha			1	0.6709	8	8	24
G1SSV1	Q99627	COPS8	PCI domain-containing protein	COP9 signalosome complex subunit 8	99	2	0.6700	4	12	38
G1SZR7	Q86UP2	KTN1	Uncharacterized protein	Kinectin	90	3	0.6691	46	84	40
G1T146	E7ESY4	MTA1	Metastasis associated 1	Metastasis-associated protein MTA1	88	2	0.6681	4	3	9
G1TEH2	P21283	ATP6V1C1	V-type proton ATPase subunit C	V-type proton ATPase subunit C 1	99	2	0.6663	7	10	20
G1T006	Q9UMX5	NENF	Neudesin neurotrophic factor	Neudesin	98	2	0.6663	3	6	19
G1SNY5	H0YK42	SNX1	PX domain-containing protein	Sorting nexin-1	96	2	0.6654	9	14	20
G1SLX0	G3V4P8	GMFB	Glia maturation factor	Glia maturation factor beta (Fragment)	99	2	0.6645	3	7	30
G1TM35	Q9Y3D6	FIS1	Fission, mitochondrial 1	Mitochondrial fission 1 protein	86	2	0.6635	4	5	15
G1SCQ0		EDEM3	alpha-1,2-Mannosidase			1	0.6635	2	2	4
P58776	A7XZE4	TPM2	Tropomyosin beta chain	Beta tropomyosin isoform	94	2	0.6617	24	27	63
	Q9UHD8	SEPTIN9		Septin-9		4	0.6617	10	7	21
G1SXU7	F8WA11	CLASP1	Cytoplasmic linker associated protein 1	CLIP-associating protein 1	93	2	0.6617	4	3	4
G1T432	Q01518	CAP1	Adenylyl cyclase-associated protein	Adenylyl cyclase-associated protein 1	96	2	0.6608	24	174	65
G1TI39	P35241	RDX	FERM domain-containing protein	Radixin	99	2	0.6608	17	16	31
G1SIX1	Q9H2G2	SLK	Uncharacterized protein	STE20-like serine/threonine-protein kinase	88	3	0.6608	6	8	12
G1SDW3		CDS2	Phosphatidate cytidylyltransferase			1	0.6608	3	4	10
G1SWY3	A0A1B0GUZ7	EFR3A	Uncharacterized protein	Protein EFR3 homolog A	98	3	0.6608	2	4	5
G1SMU8	Q9NQP4	PFDN4	Prefoldin subunit 4	Prefoldin subunit 4	99	2	0.6608	2	3	20
G1T3J0		SLC26A7	Anion exchange transporter			1	0.6598	2	2	7
	Q16181	SEPTIN7		Septin-7		4	0.6589	19	54	57
G1TZ31	Q9Y680	FKBP7	Peptidylprolyl isomerase	Peptidyl-prolyl cis-trans isomerase FKBP7	77	2	0.6580	11	27	44
G1SXI1		TTC27	TPR_REGION domain-containing protein			1	0.6580	3	4	7
G1SXK6	Q9NR12	PDLIM7	PDZ and LIM domain 7	PDZ and LIM domain protein 7	92	2	0.6571	8	24	26

U3KNY1	Q12797	ASPH	Aspartate beta-hydroxylase	Aspartyl/asparaginyl beta-hydroxylase	96	2	0.6552	19	13	56
G1TCS1		MTMR9	Myotubularin phosphatase domain-containing protein			1	0.6552	2	3	6
G1SY85	Q9Y2T2	AP3M1	MHD domain-containing protein	AP-3 complex subunit mu-1	99	2	0.6543	9	17	42
G1TZ26		GUK1	Guanylate kinase 1			1	0.6543	3	6	24
G1SFH5	O00469-2	PLOD2	Fe2OG dioxygenase domain-containing protein		90	2	0.6525	30	63	47
G1T8H3	P51570	GALK1	Uncharacterized protein	Galactokinase	92	3	0.6525	10	13	42
G1TAK5		WNK4	WNK lysine deficient protein kinase 4			1	0.6525	3	3	2
G1U9R8	P06396-2	GSN	Uncharacterized protein	Isoform 2 of Gelsolin	95	3	0.6506	19	42	41
G1SWU8	A0A0C4DGW6	C5orf51	Uncharacterized protein	UPF0600 protein C5orf51	96	3	0.6497	3	4	45
G1SE41	Q9NWM8	FKBP14	Peptidylprolyl isomerase	Peptidyl-prolyl cis-trans isomerase FKBP14	94	2	0.6488	4	8	23
G1SKY8	Q96A57	TMEM230	Uncharacterized protein	Transmembrane protein 230	98	3	0.6488	2	3	22
	P06753-5	TPM3		Isoform 5 of Tropomyosin alpha-3 chain		4	0.6479	21	27	62
G1SXL6	O94915	FRYL	Uncharacterized protein	Protein furry homolog-like	97	3	0.6479	4	6	2
G1TAJ3	P00338	LDHA	L-lactate dehydrogenase	L-lactate dehydrogenase A chain	94	2	0.6469	21	226	66
G1TWU1		TOR1B	Torsin			1	0.6469	5	5	19
G1TS93	Q15435	PPP1R7	LRRcap domain-containing protein	Protein phosphatase 1 regulatory subunit 7	95	2	0.6460	10	8	46
G1SD09	A0A0J9YYJ0	CNPY4	Canopy FGF signaling regulator 4	Protein canopy homolog 4 (Fragment)	97	2	0.6460	6	16	35
G1TDH8		CLCN5	Chloride channel protein			1	0.6460	4	6	13
	Q8TBX8	PIP4K2C		Phosphatidylinositol 5-phosphate 4-kinase type-2 gamma		4	0.6460	4	3	18
P30801		S100A6	Protein S100-A6			1	0.6451	7	22	56
G1SIP7	O14976	GAK	Cyclin G associated kinase	Cyclin-G-associated kinase	77	2	0.6451	4	4	4
G1T5R0		TUBGCP3	Gamma-tubulin complex component			1	0.6451	2	2	5
G1SZP0	O60784	TOM1	Uncharacterized protein	Target of Myb protein 1	88	3	0.6433	11	26	44
	P17858	PFKL		ATP-dependent 6-phosphofructokinase, liver type		4	0.6414	8	11	12
	P32119	PRDX2		Peroxiredoxin-2		4	0.6414	5	10	31
G1SDU5	H3BPE1	MACF1	Uncharacterized protein	Microtubule-actin cross-linking factor 1, isoforms 1/2/3/5	88	3	0.6405	203	165	38
G1SPN9	P36405	ARL3	Uncharacterized protein	ADP-ribosylation factor-like protein 3	99	3	0.6396	2	3	9
G1SGV5	Q8NE71	ABCF1	Uncharacterized protein	ATP-binding cassette sub-family F member 1	92	3	0.6386	11	13	27
G1TMM0	A0A087WW43	ITIH3	Inter-alpha-trypsin inhibitor heavy chain H3	Inter-alpha-trypsin inhibitor heavy chain H3	91	2	0.6386	4	11	5
G1TEG8	Q8WUM4	PDCD6IP	BRO1 domain-containing protein	Programmed cell death 6-interacting protein	94	2	0.6377	24	95	36
G1T7Z0	P52209	PGD	6-phosphogluconate dehydrogenase, decarboxylating	6-phosphogluconate dehydrogenase, decarboxylating	93	2	0.6350	14	18	31
G1TCD4	E5RGF9	FAM114A2	Uncharacterized protein	Protein FAM114A2 (Fragment)	80	3	0.6350	5	4	18
B7NZQ6	P31150	GDI1	Rab GDP dissociation inhibitor	Rab GDP dissociation inhibitor alpha	99	2	0.6331	16	22	48
G1TVW5	Q96IJ6	GMPPA	NTP_transferase domain-containing protein	Mannose-1-phosphate guanyltransferase alpha	96	2	0.6331	7	8	30
G1SHM2		CLPTM1L	CLPTM1 like			1	0.6331	5	5	22
G1SLF5	H0YG54	REXO2	Exonuclease domain-containing protein	Oligoribonuclease, mitochondrial	98	2	0.6331	3	4	22
G1SFF5	O15270	SPTLC2	Aminotran_1_2 domain-containing protein	Serine palmitoyltransferase 2	98	2	0.6331	3	4	8
G1SWL6	Q9P0K7	RAI14	ANK_REP_REGION domain-containing protein	Ankycorbin	91	2	0.6313	24	43	32
G1TE37		ATP6AP1	ATPase H+ transporting accessory protein 1			1	0.6304	2	2	10
G1SZR4	H7BZ14	PPIL3	Peptidyl-prolyl cis-trans isomerase	Peptidyl-prolyl cis-trans isomerase (Fragment)	100	2	0.6304	2	3	12
	Q8NBZ7	UXS1		UDP-glucuronic acid decarboxylase 1		4	0.6294	2	3	10
G1TBT4	Q9HB90	RRAGC	Uncharacterized protein	Ras-related GTP-binding protein C	98	3	0.6285	5	9	23
	Q14318	FKBP8		Peptidyl-prolyl cis-trans isomerase FKBP8		4	0.6285	3	6	15
G1SDQ5	Q96JG6	VPS50	Uncharacterized protein	Syndetin	98	3	0.6276	4	8	9
	Q9UQ13	SHOC2		Leucine-rich repeat protein SHOC-2		4	0.6276	2	4	10
G1SIT6	Q9BPX5	ARPC5L	Actin-related protein 2/3 complex subunit 5	Actin-related protein 2/3 complex subunit 5-like protein	98	2	0.6257	7	11	63
G1SWD8	P42356	PI4KA	Uncharacterized protein	Phosphatidylinositol 4-kinase alpha	98	3	0.6248	10	5	8
G1SMT2		FAM129B	Family with sequence similarity 129 member B			1	0.6239	17	44	36
G1SDF2	A0A0A0MRJ6	PCMT1	Protein-L-isoaspartate O-methyltransferase	Protein-L-isoaspartate O-methyltransferase	96	2	0.6239	5	7	21
G1SGK1		GIGYF2	GRB10 interacting GYF protein 2			1	0.6239	3	3	5
G1SCP7	P46940	IQGAP1	Uncharacterized protein	Ras GTPase-activating-like protein IQGAP1	97	3	0.6221	75	237	60
G1SZK4	Q13442	PDAP1	PDGFA associated protein 1	28 kDa heat- and acid-stable phosphoprotein	96	2	0.6221	2	4	16
G1TAB2		GM2A	ML domain-containing protein			1	0.6211	4	8	22
G1TDU0	P33176	KIF5B	Kinesin-like protein	Kinesin-1 heavy chain	99	2	0.6175	28	7	41
G1T2C4	Q01995	TAGLN	Transgelin	Transgelin	99	2	0.6175	11	164	58
G1SPF5	P18669	PGAM1	Uncharacterized protein	Phosphoglycerate mutase 1	100	3	0.6165	13	90	67
G1TVQ3		DYNLRB1	Dynein light chain roadblock			1	0.6165	3	4	51

G1T188	Q9UIJ7	AK3	GTP:AMP phosphotransferase AK3, mitochondrial	GTP:AMP phosphotransferase AK3, mitochondrial	93	2	0.6156	11	22	57
G1TR42		RNPEP	Leuk-A4-hydro_C domain-containing protein			1	0.6156	5	5	10
G1T8L2	P05997	COL5A2	Uncharacterized protein	Collagen alpha-2(V) chain	96	3	0.6138	15	31	17
G1SUK4		MPI	Mannose-6-phosphate isomerase			1	0.6138	3	3	12
G1TME7		CSTB	Cystatin B			1	0.6119	3	4	36
G1T281		SNTA1	Alpha-1-syntrophin			1	0.6119	2	7	8
G1T8R3	P46939	UTRN	Uncharacterized protein	Utrophin	91	3	0.6110	53	70	24
G1U3X5	P46821	MAP1B	Uncharacterized protein	Microtubule-associated protein 1B	88	3	0.6110	15	8	10
G1TT75	O00264	PGRMC1	Cytochrome b5 heme-binding domain-containing protein	Membrane-associated progesterone receptor component 1	93	2	0.6110	7	9	29
G1SNP8	C9JJP5	TFG	PB1 domain-containing protein	Protein TFG (Fragment)	97	2	0.6110	7	16	23
G1TET2	P13796	LCP1	Uncharacterized protein	Plastin-2	98	3	0.6092	19	24	46
G1T6T5	E9PPQ5	CHORDC1	Uncharacterized protein	Cysteine and histidine-rich domain-containing protein 1	94	3	0.6092	2	2	9
P51662	P04083	ANXA1	Annexin A1	Annexin A1	91	2	0.6082	20	22	61
	Q9UJY5	GGA1		ADP-ribosylation factor-binding protein GGA1		4	0.6082	3	5	9
	A0A2R8Y3S6	SNX27		Sorting nexin-27 (Fragment)		4	0.6082	2	3	20
G1TEM7	D6RA82	ANXA3	Annexin	Annexin	93	2	0.6073	13	21	51
G1TMW2		TOLLIP	Toll interacting protein			1	0.6073	4	5	25
	Q8WUW1	BRK1		Protein BRICK1		4	0.6073	2	3	23
G1TZ40	Q5M775	SPECC1	Calponin-homology (CH) domain-containing protein	Cytospin-B	88	2	0.6073	2	5	3
	O14964	HGS		Hepatocyte growth factor-regulated tyrosine kinase substrate		4	0.6064	5	9	13
G1U634	P08473	MME	Neprilysin	Neprilysin	94	2	0.6045	41	6	61
G1T345		UBR1	E3 ubiquitin-protein ligase			1	0.6045	2	2	2
G1T6C0	P61020	RAB5B	Uncharacterized protein	Ras-related protein Rab-5B	100	3	0.6036	9	40	56
G1SJS2	Q14232	EIF2B1	Uncharacterized protein	Translation initiation factor eIF-2B subunit alpha	96	3	0.6036	4	13	31
G1SSP0		STRN	WD_REPEATS_REGION domain-containing protein			1	0.6036	3	4	11
G1U6H4	O95865	DDAH2	Uncharacterized protein	N(G),N(G)-dimethylarginine dimethylaminohydrolase 2	97	3	0.6027	9	9	56
G1SQF2	O43237	DYNC1LI2	Uncharacterized protein	Cytoplasmic dynein 1 light intermediate chain 2	98	3	0.6027	9	14	37
G1T277	F8VR50	ARPC3	Actin-related protein 2/3 complex subunit 3	Actin-related protein 2/3 complex subunit 3 (Fragment)	100	2	0.6018	3	9	17
G1U0I7	H3BSK9	ATXN2L	Ataxin 2 like	Ataxin-2-like protein (Fragment)	99	2	0.6009	5	5	25
G1SFG7	P48681	NES	Nestin	Nestin	60	2	0.5999	28	135	31
G1SCX4	Q15052	ARHGEF6	Uncharacterized protein	Rho guanine nucleotide exchange factor 6	94	3	0.5999	2	3	4
G1TYN0	Q9BVK6	TMED9	Transmembrane p24 trafficking protein 9	Transmembrane emp24 domain-containing protein 9	80	2	0.5990	9	9	35
G1SRF5	Q9UPN7	PPP6R1	Protein phosphatase 6 regulatory subunit 1	Serine/threonine-protein phosphatase 6 regulatory subunit 1	84	2	0.5990	4	4	9
	O43707	ACTN4		Alpha-actinin-4		4	0.5972	52	74	64
G1STD5		SUCO	SUN domain containing ossification factor			1	0.5972	2	4	3
G1T8Z0	P30041	PRDX6	Thioredoxin domain-containing protein	Peroxiredoxin-6	95	2	0.5963	15	33	64
G1SYD2		AK4	Adenylate kinase 4, mitochondrial			1	0.5963	6	8	41
G1T6D1	P62829	RPL23	Uncharacterized protein	60S ribosomal protein L23	100	3	0.5963	6	14	57
G1SNC4	Q5T9B7	AK1	Adenylate kinase isoenzyme 1	Adenylate kinase isoenzyme 1	94	2	0.5963	2	4	12
	Q7Z406	MYH14		Myosin-14		4	0.5953	14	63	8
G1TYA7	P07195	LDHB	L-lactate dehydrogenase	L-lactate dehydrogenase B chain	100	2	0.5944	17	179	58
G1T5Y1	P28482	MAPK1	Mitogen-activated protein kinase	Mitogen-activated protein kinase 1	98	2	0.5944	11	9	44
G1SI83	Q99584	S100A13	S_100 domain-containing protein	Protein S100-A13	91	2	0.5944	3	5	27
G1T8B5	Q9H1E3	NUCKS1	Nuclear casein kinase and cyclin dependent kinase substrate 1	Nuclear ubiquitous casein and cyclin-dependent kinase substrate 1	94	2	0.5944	2	3	9
G1SPZ7		GPX1	Glutathione peroxidase			1	0.5935	5	6	51
G1TX63		CDC42BPA	Non-specific serine/threonine protein kinase			1	0.5935	4	2	4
G1SQ10	Q9NQT8	KIF13B	Uncharacterized protein	Kinesin-like protein KIF13B	89	3	0.5926	13	4	9
G1T5E6	O60749	SNX2	PX domain-containing protein	Sorting nexin-2	98	2	0.5916	11	11	28
B7NZQ3		RA_m006_jsm824	Deoxyribonuclease			1	0.5916	6	15	28
G1SN22	Q9Y5K6	CD2AP	Uncharacterized protein	CD2-associated protein	91	3	0.5907	2	2	5
G1SLL1	A0A087X0K9	TJP1	Uncharacterized protein	Tight junction protein ZO-1	82	3	0.5898	21	27	19
G1SWS9	P08670	VIM	IF rod domain-containing protein	Vimentin	97	2	0.5889	50	###	88
G1SH88	Q8N8S7	ENAH	ENAH, actin regulator	Protein enabled homolog	99	2	0.5880	10	2	28
	P02533	KRT14		Keratin, type I cytoskeletal 14		4	0.5880	5	4	14
	P78536	ADAM17		Disintegrin and metalloproteinase domain-containing protein 17		4	0.5880	3	3	6
	H0Y5B4	RPL36A		60S ribosomal protein L36a		4	0.5880	3	6	16
G1TV76	Q9Y4D1	DAAM1	Uncharacterized protein	Disheveled-associated activator of morphogenesis 1	97	3	0.5861	4	6	6

G1SCP8	P26038	MSN	FERM domain-containing protein	Moesin	94	2	0.5852	33	23	59
G1SXE6	Q9Y6G9	DYNC1LI1	Uncharacterized protein	Cytoplasmic dynein 1 light intermediate chain 1	94	3	0.5852	9	10	26
Q09YN6	C9JKI3	CAV1	Caveolin-1	Caveolin (Fragment)	96	2	0.5824	3	4	21
G1SYJ4	P06733	ENO1	Uncharacterized protein	Alpha-enolase	96	3	0.5806	24	143	72
G1SKD5	Q92783	STAM	Uncharacterized protein	Signal transducing adapter molecule 1	96	3	0.5806	5	12	14
G1U7S8	F8W6C2	SPATS2L	Uncharacterized protein	SPATS2-like protein (Fragment)	100	3	0.5797	4	4	9
G1SSW2	B4E0Y9	STK26	Protein kinase domain-containing protein	Serine/threonine-protein kinase 26	95	2	0.5778	5	3	19
G1TPC5		TPD52	Tumor protein D52			1	0.5778	3	6	19
G1U3F3		NEXN	Ig-like domain-containing protein			1	0.5769	22	28	36
G1SCT1	A0A499FJL1	PREP	Uncharacterized protein	Prolyl endopeptidase	96	3	0.5769	19	35	41
G1U8T9	Q96CX2	KCTD12	Potassium channel tetramerization domain containing 12	BTB/POZ domain-containing protein KCTD12	97	2	0.5751	3	3	17
G1TRX7	P23743	DGKA	Diacylglycerol kinase	Diacylglycerol kinase alpha	93	2	0.5741	4	5	9
G1U974	A0A1C7CYX9	DPYSL2	Amidohydro-rel domain-containing protein	Dihydropyrimidinase-related protein 2	98	2	0.5732	19	38	51
G1SMY7	C9JIF9	APEH	Acylamino-acid-releasing enzyme	Acylamino-acid-releasing enzyme	93	2	0.5732	7	11	18
P53787		EEF1D	Elongation factor 1-delta			1	0.5732	4	16	24
	Q96BM9	ARL8A		ADP-ribosylation factor-like protein 8A		4	0.5723	7	2	45
G1T3S7	O60476	MAN1A2	alpha-1,2-Mannosidase	Mannosyl-oligosaccharide 1,2-alpha-mannosidase IB	95	2	0.5723	2	2	4
G1T182	Q86UE4	MTDH	Metadherin	Protein LYRIC	88	2	0.5705	4	6	18
G1SMP6	A0A1B0GVV3	RILPL1	Rab interacting lysosomal protein like 1	RILP-like protein 1	93	2	0.5695	4	6	16
G1U4A0		FRMD8	FERM domain containing 8			1	0.5695	2	2	9
G1TC70	O14974	PPP1R12A	Protein phosphatase 1 regulatory subunit	Protein phosphatase 1 regulatory subunit 12A	94	2	0.5668	13	33	16
G1TRI7	A0A0C4DG51	PNPLA8	Calcium-independent phospholipase A2-gamma	Calcium-independent phospholipase A2-gamma (Fragment)	90	2	0.5668	2	2	4
G1TGA8	O75396	SEC22B	Uncharacterized protein	Vesicle-trafficking protein SEC22b	98	3	0.5658	10	33	48
G1T3I9	P20073	ANXA7	Annexin	Annexin A7	93	2	0.5649	10	13	20
G1U9R0	A8MX94	GSTP1	Uncharacterized protein	Glutathione S-transferase P	71	3	0.5649	10	90	49
G1TBW1		TXNDC17	DUF953 domain-containing protein			1	0.5649	3	8	31
G1SJ56	P18206	VCL	Uncharacterized protein	Vinculin	99	3	0.5640	54	176	59
G1SFG6	A0A0A0MTS2	GPI	Glucose-6-phosphate isomerase	Glucose-6-phosphate isomerase (Fragment)	93	2	0.5640	22	130	56
G1SUK5	O43583	DENR	Density-regulated protein	Density-regulated protein	97	2	0.5640	2	5	9
G1SI13		SDC2	Syndecan			1	0.5603	3	4	11
	P25098	GRK2		Beta-adrenergic receptor kinase 1		4	0.5603	2	2	6
G1SZI7		CROT	Carn_acyltransf domain-containing protein			1	0.5585	2	2	4
G1SR77	P23634	ATP2B4	Calcium-transporting ATPase	Plasma membrane calcium-transporting ATPase 4	87	2	0.5575	9	10	10
G1TQV4	F6SKB8	NECAP2	DUF1681 domain-containing protein	Adaptin ear-binding coat-associated protein 2	95	2	0.5566	2	2	8
G1TAC4		GLRX	Glutaredoxin-1			1	0.5557	3	9	38
G1T4P7	Q9Y4G6	TLN2	Talin 2	Talin-2	98	2	0.5548	19	9	11
G1SVB5	J3QRN6	MYO1D	Uncharacterized protein	Unconventional myosin-Id	95	3	0.5548	15	21	20
Q28685	Q14118	DAG1	Dystroglycan	Dystroglycan	94	2	0.5548	3	4	4
G1STJ8	G3V394	MYO5A	Uncharacterized protein	Unconventional myosin-Va	96	3	0.5539	17	4	12
G1SE51	A0A087X2D8	SPAG9	Sperm associated antigen 9	C-Jun-amino-terminal kinase-interacting protein 4	93	2	0.5539	10	22	13
G1T0J3	E9PDF6	MYO1B	Uncharacterized protein	Unconventional myosin-Ib	96	3	0.5529	17	28	22
G1TN33		SEPT8	Septin-type G domain-containing protein			1	0.5520	8	10	24
G1SZ47	P62266	RPS23	Uncharacterized protein	40S ribosomal protein S23	100	3	0.5511	6	104	48
G1T7Z6	P00558	PGK1	Phosphoglycerate kinase	Phosphoglycerate kinase 1	99	2	0.5493	26	83	72
G1SP27	Q16204	CCDC6	Uncharacterized protein	Coiled-coil domain-containing protein 6	93	3	0.5493	14	31	31
G1SUF5	Q8TF66	LRRC15	LRRCT domain-containing protein	Leucine-rich repeat-containing protein 15	90	2	0.5493	6	9	18
G1T3D7		NANS	AFP-like domain-containing protein			1	0.5493	4	5	14
G1SF47		SEPT11	Septin-type G domain-containing protein			1	0.5483	12	15	34
G1TPH5	Q9Y6D5	ARFGEF2	ADP ribosylation factor guanine nucleotide exchange factor 2	Brefeldin A-inhibited guanine nucleotide-exchange protein 2	96	2	0.5483	9	9	8
G1TR97	A0A0A0MSA7	EIF4G3	Uncharacterized protein	Eukaryotic translation initiation factor 4 gamma 3	89	3	0.5483	8	11	8
G1T5W7	Q14376	GALE	NAD(P)-bd_dom domain-containing protein	UDP-glucose 4-epimerase	95	2	0.5474	7	13	27
G1SGL0	Q92629	SGCD	Uncharacterized protein	Delta-sarcoglycan	98	3	0.5465	3	5	14
	P51178	PLCD1		1-phosphatidylinositol 4,5-bisphosphate phosphodiesterase delta-1		4	0.5456	2	3	3
G1T7T8	P47712	PLA2G4A	Phospholipase A2	Cytosolic phospholipase A2	94	2	0.5446	27	126	54
G1T634	Q9Y5X1	SNX9	Sorting nexin	Sorting nexin-9	89	2	0.5437	7	8	23
Q9TTC6	P62937	PPIA	Peptidyl-prolyl cis-trans isomerase A	Peptidyl-prolyl cis-trans isomerase A	96	2	0.5391	16	22	87
	Q15149	PLEC		Plectin		4	0.5382	84	112	21

G1SN21	P00491	PNP	Purine nucleoside phosphorylase	Purine nucleoside phosphorylase	90	2	0.5382	13	28	62
G1TLL6	Q9UNH6	SNX7	PX domain-containing protein	Sorting nexin-7	96	2	0.5373	4	4	15
G1SV03	Q14165	MLEC	Malectin domain-containing protein	Malectin	95	2	0.5364	8	14	32
G1TMV1	P60981	DSTN	ADF-H domain-containing protein	Destrin	100	2	0.5345	9	72	58
G1SX37	Q9Y281	CFL2	Cofilin 2	Cofilin-2	91	2	0.5336	8	14	55
	H0Y7A7	CALM2		Calmodulin-2 (Fragment)		4	0.5336	7	51	49
G1TER4	P33897	ABCD1	Uncharacterized protein	ATP-binding cassette sub-family D member 1	94	3	0.5327	4	4	8
G1U4V6		CACNA2D1	Voltage-dependent calcium channel subunit alpha-2/delta-1			1	0.5317	9	2	13
G1U723		PGER5	3alpha/17beta/20alpha-hydroxysteroid dehydrogenase			1	0.5317	5	8	20
G1SEN8		SCCPDH	Sacchrp_dh_NADP domain-containing protein			1	0.5317	5	8	24
G1SNH7	P09417	QDPR	Quinoid dihydropteridine reductase	Dihydropteridine reductase	92	2	0.5317	4	6	23
Q8WN94		DBI	Acyl-CoA-binding protein			1	0.5317	3	4	39
G1TV31	O15511	ARPC5	Actin-related protein 2/3 complex subunit 5	Actin-related protein 2/3 complex subunit 5	95	2	0.5299	8	50	54
G1TBZ5	E9PCX2	AKR1B1	Aldo-keto reductase family 1 member B1	Aldo-keto reductase family 1 member B1	85	2	0.5281	6	15	29
	Q9NP61	ARFGAP3		ADP-ribosylation factor GTPase-activating protein 3		4	0.5262	2	2	4
G1SH05	P07437	TUBB	Tubulin beta chain	Tubulin beta chain	100	2	0.5253	23	122	71
G1SZ18	A0A0C4DFT3	DLG1	Uncharacterized protein	Disks large homolog 1	93	3	0.5235	4	5	7
G1T4N8	Q8TF42	UBASH3B	Uncharacterized protein	Ubiquitin-associated and SH3 domain-containing protein B	98	3	0.5225	2	2	5
G1TAP1	Q15181	PPA1	Uncharacterized protein	Inorganic pyrophosphatase	96	3	0.5207	8	12	48
G1SJ87	Q9H2D6	TRIOBP	TRIO and F-actin binding protein	TRIO and F-actin-binding protein	73	2	0.5207	3	4	2
G1SPV0	P61086	UBE2K	Uncharacterized protein	Ubiquitin-conjugating enzyme E2 K	100	3	0.5198	4	9	35
G1SN11	Q01082	SPTBN1	Spectrin beta chain	Spectrin beta chain, non-erythrocytic 1	99	2	0.5188	75	14	44
G1SCR0	H0YFI1	LAMTOR1	Uncharacterized protein	Ragulator complex protein LAMTOR1 (Fragment)	100	3	0.5188	2	3	47
G1SXU2	Q96HC4	PDLIM5	Uncharacterized protein	PDZ and LIM domain protein 5	88	3	0.5179	10	28	22
G1T9X4	O95980	RECK	Reversion inducing cysteine rich protein with kazal motifs	Reversion-inducing cysteine-rich protein with Kazal motifs	95	2	0.5170	3	5	3
Q6TYA7		GJA1	Gap junction alpha-1 protein			1	0.5170	2	4	6
G1U2E3	Q9H444	CHMP4B	Uncharacterized protein	Charged multivesicular body protein 4b	100	3	0.5142	7	8	40
	P22392-2	NME2		Isoform 3 of Nucleoside diphosphate kinase B		4	0.5133	7	24	35
G1T7D9		FNTB	Protein farnesyltransferase subunit beta			1	0.5124	3	2	6
	Q6NZI2	CAVIN1		Caveolae-associated protein 1		4	0.5115	7	13	19
G1SPY1	E9PGM4	GBE1	Aamy domain-containing protein	1,4-alpha-glucan-branching enzyme	93	2	0.5105	21	39	42
G1SYL5	Q7Z4N8	P4HA3	Fe2OG dioxygenase domain-containing protein	Prolyl 4-hydroxylase subunit alpha-3	92	2	0.5105	15	38	43
G1T9P1		PEAK1	Pseudopodium enriched atypical kinase 1			1	0.5105	3	3	3
G1SMX4	A0A0G2JH68	DIAPH1	Uncharacterized protein	Protein diaphanous homolog 1	90	3	0.5078	15	26	22
G1SEX8	Q9P299	COPZ2	Clat_adaptor_s domain-containing protein	Coatomer subunit zeta-2	84	2	0.5078	4	6	23
G1SXR6	O60282	KIF5C	Kinesin-like protein	Kinesin heavy chain isoform 5C	99	2	0.5059	20	13	29
G1T4Q7		ATP11C	Phospholipid-transporting ATPase			1	0.5059	2	5	3
G1SPB4		HYAL1	Hyaluronidase			1	0.5059	2	2	8
G1T8K2	A0A0U1RR22	PACSIN2	Uncharacterized protein	Protein kinase C and casein kinase substrate in neurons protein 2 (Fragment)	93	3	0.5050	8	8	23
G1SQ30		POMGNT2	Fibronectin type-III domain-containing protein			1	0.5050	2	6	6
P40144		ADCY5	Adenylate cyclase type 5			1	0.5032	3	4	4
G1T659	Q9UHY7	ENOPH1	Enolase-phosphatase E1	Enolase-phosphatase E1	97	2	0.5032	2	2	12
G1T0H8	Q01813	PFKP	ATP-dependent 6-phosphofructokinase	ATP-dependent 6-phosphofructokinase, platelet type	93	2	0.5023	22	35	42
G1SVI9	Q9P0L0	VAPA	MSP domain-containing protein	Vesicle-associated membrane protein-associated protein A	84	2	0.5023	9	18	40
	Q16658	FSCN1		Fascin		4	0.4995	10	9	23
G1SIB9	P11216	PYGB	Alpha-1,4 glucan phosphorylase	Glycogen phosphorylase, brain form	95	2	0.4986	31	24	45
G1SJ72		CSPG4	Chondroitin sulfate proteoglycan 4			1	0.4986	19	25	17
	P68371	TUBB4B		Tubulin beta-4B chain		4	0.4967	22	24	71
P48738	Q00169	PITPNA	Phosphatidylinositol transfer protein alpha isoform	Phosphatidylinositol transfer protein alpha isoform	99	2	0.4967	6	6	24
G1SQK1		SERPINB6	SERPIN domain-containing protein			1	0.4958	3	4	17
G1TER3	A0A2R8Y5M6	BCAP31	Uncharacterized protein	B-cell receptor-associated protein 31	81	3	0.4940	11	19	53
G1T512		CNP	2~,3~-cyclic nucleotide 3~ phosphodiesterase			1	0.4921	2	4	7
G1SIC4	I3L294	ABHD12	Abhydrolase domain containing 12	Lysophosphatidylserine lipase ABHD12 (Fragment)	99	2	0.4894	4	6	18
G1SHR7	Q9P0J7	KCMF1	C2H2-type domain-containing protein	E3 ubiquitin-protein ligase KCMF1	99	2	0.4894	2	3	9
G1T2K1	O95340	PAPSS2	Uncharacterized protein	Bifunctional 3~-phosphoadenosine 5~-phosphosulfate synthase 2	94	3	0.4866	5	10	14
G1SEA7	Q13131	PRKAA1	Non-specific serine/threonine protein kinase	5~-AMP-activated protein kinase catalytic subunit alpha-1	97	2	0.4866	3	3	8
G1SH63		GSS	Glutathione synthetase			1	0.4866	2	2	6

G1TNB4		PARP3	Poly [ADP-ribose] polymerase			1	0.4866	2	2	7
G1SHN4	Q96JB2	COG3	Uncharacterized protein	Conserved oligomeric Golgi complex subunit 3	96	3	0.4857	4	5	10
G1T9Q5		SLC44A2	Solute carrier family 44 member 2			1	0.4829	3	6	6
G1SMK8	O95816	BAG2	BAG domain-containing protein	BAG family molecular chaperone regulator 2	98	2	0.4811	6	9	29
G1SUY3	F8VQR7	CSRP2	Uncharacterized protein	Cysteine and glycine-rich protein 2	100	3	0.4811	6	15	38
G1T060	A0A2R8YD50	HSD17B4	Uncharacterized protein	Peroxisomal multifunctional enzyme type 2	89	3	0.4801	9	14	21
	P56377	AP1S2		AP-1 complex subunit sigma-2		4	0.4801	4	5	33
G1U411	F8WBG8	DBNL	Uncharacterized protein	Drebrin-like protein	95	3	0.4792	9	19	31
G1U5Q7		ARPC4	Actin-related protein 2/3 complex subunit 4			1	0.4792	7	36	46
G1TK63	P22413	ENPP1	Uncharacterized protein	Ectonucleotide pyrophosphatase/phosphodiesterase family member 1	89	3	0.4783	18	27	30
G1TI02	Q9BRP8	PYM1	PYM homolog 1, exon junction complex associated factor	Partner of Y14 and mago	91	2	0.4783	2	4	6
G1SNT8	P08133	ANXA6	Annexin	Annexin A6	96	2	0.4765	33	64	48
G1SVY8	P12277	CKB	Creatine kinase B-type	Creatine kinase B-type	97	2	0.4728	19	219	70
G1T823	H3BT58	COTL1	Coactosin like F-actin binding protein 1	Coactosin-like protein	96	2	0.4718	3	9	27
G1SUU7	Q9Y3I0	RTCB	tRNA-splicing ligase RtcB homolog	tRNA-splicing ligase RtcB homolog	100	2	0.4709	16	3	43
G1SNT1	O75822	EIF3J	Eukaryotic translation initiation factor 3 subunit J	Eukaryotic translation initiation factor 3 subunit J	92	2	0.4709	11	18	48
G1T004	H0Y987	PGM3	Phosphoacetylglucosamine mutase	Phosphoacetylglucosamine mutase	90	2	0.4709	8	22	27
G1ST64		LMOD1	Leiomodin 1			1	0.4709	5	6	9
G1T9W9		ATG3	Autophagy-related protein 3			1	0.4700	2	2	8
G1TBS1	Q99497	PARK7	DJ-1_PfpI domain-containing protein	Protein/nucleic acid deglycase DJ-1	96	2	0.4691	11	21	72
G1SHL8	P42224	STAT1	Signal transducer and activator of transcription	Signal transducer and activator of transcription 1-alpha/beta	95	2	0.4635	13	18	27
G1SHB9	P40121	CAPG	Uncharacterized protein	Macrophage-capping protein	94	3	0.4635	9	21	44
G1SN76	O94804	STK10	Serine/threonine kinase 10	Serine/threonine-protein kinase 10	90	2	0.4608	2	5	5
G1SG54	A0A0U1RQT1	ACAP2	Arf-GAP with coiled-coil, ANK repeat and PH domain-containing protein 2	Arf-GAP with coiled-coil, ANK repeat and PH domain-containing protein 2 (Fragment)	99	2	0.4599	4	4	11
G1T705	P00387	CYB5R3	NADH-cytochrome b5 reductase	NADH-cytochrome b5 reductase 3	90	2	0.4589	12	47	57
G1TBX9	Q9H8M7	MINDY3	DUF4205 domain-containing protein	Ubiquitin carboxyl-terminal hydrolase MINDY-3	99	2	0.4589	3	3	13
G1SLH7	P42566	EPS15	Uncharacterized protein	Epidermal growth factor receptor substrate 15	93	3	0.4543	4	5	6
G1TKE7		IQGAP2	IQ motif containing GTPase activating protein 2			1	0.4534	4	2	3
G1TBU2		KIAA1217	KIAA1217			1	0.4525	2	2	2
G1T6S4	F8W9J4	DST	Dystonin	Dystonin	62	2	0.4516	13	7	4
G1TFI4	Q96K17	BTF3L4	Transcription factor BTF3	Transcription factor BTF3 homolog 4	100	2	0.4516	3	4	39
G1SYM3		CD9	Tetraspanin			1	0.4497	3	4	7
G1SDP2	Q96AY3	FKBP10	FK506-binding protein	Peptidyl-prolyl cis-trans isomerase FKBP10	93	2	0.4488	17	26	40
G1TF67		CORO1A	Coronin			1	0.4460	3	2	10
G1T473	F8VQE1	LIMA1	LIM zinc-binding domain-containing protein	LIM domain and actin-binding protein 1	85	2	0.4451	22	41	38
G1TQJ4	Q01433	AMPD2	AMP deaminase	AMP deaminase 2	97	2	0.4396	3	4	5
G1T3U5	Q9UDY4	DNAJB4	J domain-containing protein	DnaJ homolog subfamily B member 4	96	2	0.4341	4	5	21
	H7BZL4	GPC1		Glypican-1 (Fragment)		4	0.4322	5	5	29
G1SG55	O43491	EPB41L2	FERM domain-containing protein	Band 4.1-like protein 2	90	2	0.4313	38	91	43
G1SY88	P55263	ADK	PfkB domain-containing protein	Adenosine kinase	98	2	0.4304	8	14	38
G1SUZ7		ARSA	Sulfatase domain-containing protein			1	0.4285	4	3	18
G1SKT2	E9PGF6	PHLDB2	PH domain-containing protein	Pleckstrin homology-like domain family B member 2	65	2	0.4276	8	10	8
G1TBJ8		RB1CC1	RB1 inducible coiled-coil 1			1	0.4239	3	3	3
G1SUV2		CAMLG	Calcium signal-modulating cyclophilin ligand			1	0.4239	2	5	16
G1SN43	F8W930	IGF2BP2	Uncharacterized protein	Insulin-like growth factor 2 mRNA-binding protein 2	98	3	0.4202	7	5	15
	E9PS68	PC		Pyruvate carboxylase, mitochondrial		4	0.4202	2	2	6
G1T2Q8	Q96M27	PRRC1	NTPase_I-T domain-containing protein	Protein PRRC1	90	2	0.4184	7	14	22
G1SPD1		MTPN	ANK_REP_REGION domain-containing protein			1	0.4184	2	5	32
G1U7J6		PRKCA	Protein kinase C			1	0.4184	2	2	6
G1T8J0	P02461	COL3A1	Uncharacterized protein	Collagen alpha-1(III) chain	92	3	0.4165	17	29	15
G1T8C8	A0A087X0R6	SNX12	Sorting nexin 12	Sorting nexin-12	100	2	0.4165	2	3	13
G1TN08	P37235	HPCAL1	Uncharacterized protein	Hippocalcin-like protein 1	99	3	0.4147	6	5	34
G1T346	Q13813-3	SPTAN1	Uncharacterized protein	Isoform 3 of Spectrin alpha chain, non-erythrocytic 1	97	3	0.4129	108	30	55
G1SZJ7	A0A2R8YEC9	MANBA	Uncharacterized protein	Beta-mannosidase	75	3	0.4064	7	10	11
G1SNP7	E9PGC8	MAP1A	Uncharacterized protein	Microtubule-associated protein 1A	79	3	0.4036	6	5	4
G1SZH6	Q9BZF1	OSBPL8	Oxysterol-binding protein	Oxysterol-binding protein-related protein 8	99	2	0.3990	7	10	11
G1T6L5	O75368	SH3BGRL	Uncharacterized protein	SH3 domain-binding glutamic acid-rich-like protein	98	3	0.3990	5	10	31

	B4DDF4	CNN2		Calponin		4	0.3972	5	4	18
G1U7U3		NME1	Nucleoside diphosphate kinase			1	0.3963	7	9	57
G1SP54		LTA4H	Leukotriene A(4) hydrolase			1	0.3944	4	6	10
	P33241	LSP1		Lymphocyte-specific protein 1		4	0.3889	3	4	9
G1U7Y3	E7END4	LOXL3	Uncharacterized protein	Lysyl oxidase homolog 3	87	3	0.3880	3	3	5
G1TRY5	P13797	PLS3	Uncharacterized protein	Plastin-3	100	3	0.3871	37	151	77
G1SYE0		ABHD14B	AB hydrolase-1 domain-containing protein			1	0.3815	4	7	21
	Q9Y2V2	CARHSP1		Calcium-regulated heat-stable protein 1		4	0.3797	2	3	18
G1STV0		CAST	Calpastatin			1	0.3742	12	24	21
G1SPL3	G3V583	FAM177A1	Uncharacterized protein	Protein FAM177A1 (Fragment)	93	3	0.3742	2	2	12
G1SFW9		ACIN1	SAP domain-containing protein			1	0.3686	2	2	2
G1TP25	Q5VU77	UBAP2L	Ubiquitin associated protein 2 like	Ubiquitin-associated protein 2-like (Fragment)	95	2	0.3668	3	4	4
G1SWW4		SYNPO	Synaptopodin			1	0.3659	3	7	6
G1T5P0	Q8NDI1	EHBP1	Uncharacterized protein	EH domain-binding protein 1	94	3	0.3640	2	3	3
G1SY87		CD200	Ig-like domain-containing protein			1	0.3594	2	3	7
G1TBQ6	O95292	VAPB	VAMP associated protein B and C	Vesicle-associated membrane protein-associated protein B/C	90	2	0.3566	3	3	17
G1TMI5		RABAC1	PRA1 family protein			1	0.3557	2	3	17
G1SPQ9	O94875	SORBS2	Uncharacterized protein	Sorbin and SH3 domain-containing protein 2	90	3	0.3530	17	25	19
G1TYY5	Q14847	LASP1	LIM and SH3 domain protein 1	LIM and SH3 domain protein 1	94	2	0.3401	6	9	23
G1T5B6	P12107	COL11A1	Fibrillar collagen NC1 domain-containing protein	Collagen alpha-1(XI) chain	98	2	0.3373	29	54	24
G1SD83		ITGA6	Integrin_alpha2 domain-containing protein			1	0.3281	2	6	2
G1T3V2	P04792	HSPB1	SHSP domain-containing protein	Heat shock protein beta-1	91	2	0.3244	8	22	52
	P32418	SLC8A1		Sodium/calcium exchanger 1		4	0.3225	2	3	4
P62943	P62942	FKBP1A	Peptidyl-prolyl cis-trans isomerase FKBP1A	Peptidyl-prolyl cis-trans isomerase FKBP1A	100	2	0.3207	2	6	25
G1TN29	Q86UU1	PHLDB1	Pleckstrin homology like domain family B member 1	Pleckstrin homology-like domain family B member 1	89	2	0.3143	8	3	6
G1SSL3	P29373	CRABP2	FABP domain-containing protein	Cellular retinoic acid-binding protein 2	90	2	0.3124	3	3	27
G1TA83	P09525	ANXA4	Annexin	Annexin A4	94	2	0.3087	13	27	51
G1SW82	H0Y9Y3	SYNPO2	PDZ domain-containing protein	Synaptopodin-2 (Fragment)	80	2	0.3078	3	5	4
G1SI22		AKAP12	A-kinase anchoring protein 12			1	0.3060	11	15	17
G1TNJ2	Q15942	ZYX	Uncharacterized protein	Zyxin	87	3	0.3032	6	23	15
G1SRD9	A0A1C7CYX8	FAM107B	Uncharacterized protein	Protein FAM107B (Fragment)	96	3	0.2967	2	2	16
	P51911	CNN1		Calponin-1		4	0.2931	7	8	33
G1TE78	Q15121	PEA15	DED domain-containing protein	Astrocytic phosphoprotein PEA-15	100	2	0.2848	5	4	43
G1SRR2	E9PMP7	LMO7	Uncharacterized protein	LIM domain only protein 7 (Fragment)	70	3	0.2820	30	39	24
G1SUI9	E7EX44	CALD1	Uncharacterized protein	Caldesmon	84	3	0.2792	24	103	39
G1T013	P48163	ME1	Malic enzyme	NADP-dependent malic enzyme	95	2	0.2737	6	17	22
G1TK30		CSAD	Cysteine sulfinic acid decarboxylase			1	0.2663	4	9	18
G1T387	Q14247	CTTN	Cortactin	Src substrate cortactin	88	2	0.2580	5	3	13
G1U1H1	Q00535	CDK5	Protein kinase domain-containing protein	Cyclin-dependent-like kinase 5	100	2	0.2534	3	2	12
G1TA72		HS1BP3	PX domain-containing protein			1	0.2479	2	2	6
G1T5T8	O00151	PDLIM1	Uncharacterized protein	PDZ and LIM domain protein 1	92	3	0.2461	14	31	60
G1SMS2	Q8WX93	PALLD	Uncharacterized protein	Palladin	87	3	0.2433	20	43	20
G1T315	A0A2R8Y2R1	SGCE	CADG domain-containing protein	Epsilon-sarcoglycan (Fragment)	88	2	0.2313	2	6	7
G1T6W4		ANXA8	Annexin			1	0.2212	18	72	66
G1SW77	Q8NC51	SERBP1	HABP4_PAI-RBP1 domain-containing protein	Plasminogen activator inhibitor 1 RNA-binding protein	99	2	0.2212	7	17	19
	Q09666	AHNAK		Neuroblast differentiation-associated protein AHNAK		4	0.2156	33	48	6
G1SKQ9		SEPT6	Septin-type G domain-containing protein			1	0.2156	8	3	22
G1SVE3		PRUNE2	CRAL-TRIO domain-containing protein			1	0.2129	4	4	2
G1SKS8	E7EVA0	MAP4	Microtubule-associated protein	Microtubule-associated protein	85	2	0.2110	25	48	30
P15541		ANPEP	Aminopeptidase N			1	0.1871	11	14	19
P35748	P35749	MYH11	Myosin-11	Myosin-11	97	2	0.1410	42	38	29
G1T4F9	E9PR44	CRYAB	Alpha-crystallin B chain	Alpha-crystallin B chain (Fragment)	98	2	0.1180	6	13	44

A. Percent sequence Identity (rounded to the nearest integer) between the indicated rabbit and human proteins from blast analysis of the rabbit and human UniProt databases.
B. Database Identification Categories: Category 1, Characterized in the rabbit database only; Category 2, Characterized in both the rabbit and human databases; Category 3, Uncharacterized in the rabbit database but characterized in the human database; Category 4, Characterized in the human database only.
C. Number of unique peptides quantified.
D. Number of peptide intensities summed for protein quantitation.

Supplemental Table S2
Myofibroblasts from Rabbit Cornea & Bone Marrow
Relative Protein Abundance, Rabbit 25
Orbitrap Fusion Lumos Tribrid LC MS/MS iTRAQ Results

Total Proteins Quantified = 2320; Median Protein iTRAQ Ratio =1.003; Mean Protein iTRAQ Ratio = 1.000; Standard Deviation (SD) = 0.356
Yellow = 1SD from the mean; Brown = 2SD from the mean

Rabbit Accession UniProt	Human Accession UniProt	Gene Symbol	Rabbit Protein Description	Human Protein Description	% identity[A]	Database Identification category[B]	Linear Ratio Cornea/Bone marrow	Peptides[C]	N[D]	% sequence coverage
	A0A2R8Y7G9	H3.Y		Histone domain-containing protein		4	4.87	2	3	29
	P02765	AHSG		Alpha-2-HS-glycoprotein		4	3.95	2	2	5
G1T2X0	Q99541	PLIN2	Perilipin	Perilipin-2	88	2	3.03	9	14	28
O19105		SLC1A5	Neutral amino acid transporter B(0)			1	3.00	4	5	14
G1TVK1		NOTCH2	Notch 2			1	2.85	4	5	3
G1TKV4		HIST2H3D	Histone H3			1	2.84	7	26	52
G1SIW5	Q92820	GGH	Folate gamma-glutamyl hydrolase	Gamma-glutamyl hydrolase	82	2	2.71	6	15	28
G1SDW8	H0Y2P0	CD44	Link domain-containing protein	CD44 antigen (Fragment)	91	2	2.70	10	18	17
	A0A2R8Y7C0	HBA2		Hemoglobin subunit alpha (Fragment)		4	2.67	3	2	23
G1SDL7		ARG2	Arginase			1	2.65	2	2	8
G1SK42	P21980	TGM2	TGc domain-containing protein	Protein-glutamine gamma-glutamyltransferase 2	87	2	2.53	22	69	51
	P16104	H2AFX		Histone H2AX		4	2.50	5	22	53
G1U450		XYLB	Xylulokinase			1	2.50	2	2	6
G1SFC6	A0A087WV29	NAT10	RNA cytidine acetyltransferase	RNA cytidine acetyltransferase	96	2	2.46	6	3	11
G1T2K5	I3L1L3	MYBBP1A	Uncharacterized protein	Myb-binding protein 1A (Fragment)	69	3	2.44	34	83	37
G1T3Y0	P98082	DAB2	PID domain-containing protein	Disabled homolog 2	90	2	2.41	5	5	9
G1SL97	Q15397	PUM3	PUM-HD domain-containing protein	Pumilio homolog 3	91	2	2.40	6	5	16
G1ST69	P20700	LMNB1	Uncharacterized protein	Lamin-B1	98	3	2.39	19	33	42
G1SJ30		WDR43	WD_REPEATS_REGION domain-containing protein			1	2.32	2	2	4
G1SJW7	J3QLE5	SNRPN	Small nuclear ribonucleoprotein-associated protein	Small nuclear ribonucleoprotein-associated protein N (Fragment)	100	2	2.32	6	10	19
G1TW43	J3QSU6	TNC	Uncharacterized protein	Tenascin	74	3	2.31	6	6	3
G1SIJ7	Q9NR30	DDX21	Uncharacterized protein	Nucleolar RNA helicase 2	89	3	2.30	16	27	24
G1T1B6	Q16832	DDR2	Uncharacterized protein	Discoidin domain-containing receptor 2	97	3	2.26	5	5	9
G1SXN0		PES1	Pescadillo homolog			1	2.25	2	3	6
G1TX84	Q9Y5J1	UTP18	WD_REPEATS_REGION domain-containing protein	U3 small nucleolar RNA-associated protein 18 homolog	89	2	2.23	4	4	12
G1TLW3	J3KTA4	DDX5	Uncharacterized protein	Probable ATP-dependent RNA helicase DDX5	96	3	2.21	21	28	42
G1T594	G0XQ39	STIM1	Stromal interaction molecule 1	STIM1L	98	2	2.21	2	3	3
G1TUE1		ATP1B1	Sodium/potassium-transporting ATPase subunit beta			1	2.20	4	4	15
G1SDA2	P29762	CRABP1	FABP domain-containing protein	Cellular retinoic acid-binding protein 1	99	2	2.20	5	14	41
G1SVK5	P26447	S100A4	Protein S100	Protein S100-A4	98	2	2.19	4	4	36
G1STC6		WDR3	WD_REPEATS_REGION domain-containing protein			1	2.19	3	3	5
G1SNX5	B4DY09	ILF2	Interleukin enhancer binding factor 2	Interleukin enhancer-binding factor 2	100	2	2.17	17	67	58
G1TEP2		FAM210B	DUF1279 domain-containing protein			1	2.16	2	2	12
G1T017		SLC1A4	Solute carrier family 1 member 4			1	2.14	3	6	9
G1SNS1	Q14692	BMS1	Bms1-type G domain-containing protein	Ribosome biogenesis protein BMS1 homolog	88	2	2.13	5	4	7
G1SUP9	P46087	NOP2	SAM_MT_RSMB_NOP domain-containing protein	Probable 28S rRNA (cytosine(4447)-C(5))-methyltransferase	77	2	2.13	6	5	12
G1SZE8		UTP20	DRIM domain-containing protein			1	2.13	7	11	4
G1T4M2	O60264	SMARCA5	Uncharacterized protein	SWI/SNF-related matrix-associated actin-dependent regulator of chromatin subfamily A member 5	100	3	2.11	5	9	8
G1SE30		EPS8	SH3 domain-containing protein			1	2.11	2	4	5
G1SWD1	M0QXL5	FBL	Fibrillarin	rRNA 2~-O-methyltransferase fibrillarin (Fragment)	97	2	2.10	10	63	66
G1T5I0		SLC7A1	Solute carrier family 7 member 1			1	2.10	4	9	11
G1TD41	G8JLB6	HNRNPH1	Uncharacterized protein	Heterogeneous nuclear ribonucleoprotein H	98	3	2.09	12	11	39
G1TD26	D6RAA6	TMEM33	Uncharacterized protein	Transmembrane protein 33 (Fragment)	99	3	2.07	3	6	12
G1TL80	Q9Y3B4	SF3B6	RRM domain-containing protein	Splicing factor 3B subunit 6	100	2	2.07	2	6	21
G1SI79	P51991	HNRNPA3	Uncharacterized protein	Heterogeneous nuclear ribonucleoprotein A3	100	3	2.06	8	22	25
G1TNT7		DDX27	DEAD-box helicase 27			1	2.05	2	2	3
G1SQR1	Q93096	PTP4A1	TYR_PHOSPHATASE_2 domain-containing protein	Protein tyrosine phosphatase type IVA 1	100	2	2.04	2	3	17
G1TAR0		RPF2	Brix domain-containing protein			1	2.04	3	3	14
	Q969V3	NCLN		Nicalin		4	2.02	7	11	18
G1SMB3	Q9NV31	IMP3	S4 RNA-binding domain-containing protein	U3 small nucleolar ribonucleoprotein protein IMP3	99	2	2.02	2	2	15
P00389	P16435	POR	NADPH--cytochrome P450 reductase	NADPH--cytochrome P450 reductase	92	2	2.00	27	65	49

G1SQ07		DTYMK	Thymidylate_kin domain-containing protein			1	1.99	2	2	10
G1TI40	P62316	SNRPD2	Small nuclear ribonucleoprotein Sm D2	Small nuclear ribonucleoprotein Sm D2	100	2	1.97	6	15	48
G1SIJ6	Q13308	PTK7	Protein tyrosine kinase 7 (inactive)	Inactive tyrosine-protein kinase 7	93	2	1.97	28	60	39
	H0Y6E7	RBMX		RNA-binding motif protein, X chromosome (Fragment)		4	1.96	3	9	12
G1TC03	Q13263	TRIM28	Tripartite motif containing 28	Transcription intermediary factor 1-beta	96	2	1.96	16	13	25
G1U4P8	Q9BXN1	ASPN	LRRNT domain-containing protein	Asporin	88	2	1.95	5	6	19
G1TB02	E7EPS8	PTPRM	Uncharacterized protein	Receptor-type tyrosine-protein phosphatase mu	98	3	1.95	3	3	3
G1TPN3	D6R9P3	HNRNPAB	Uncharacterized protein	Heterogeneous nuclear ribonucleoprotein A/B	96	3	1.95	9	71	37
G1T9U7	Q8TD30	GPT2	Glutamic--pyruvic transaminase 2	Alanine aminotransferase 2	97	2	1.94	5	2	21
G1SZ76	P05455	SSB	Lupus La protein homolog	Lupus La protein	92	2	1.94	16	30	41
G1SEF5	Q8NAV1	PRPF38A	PRP38_assoc domain-containing protein	Pre-mRNA-splicing factor 38A	100	2	1.94	2	4	9
G1T4L3	Q01780	EXOSC10	HRDC domain-containing protein	Exosome component 10	89	2	1.94	2	3	4
G1T6T0	J3KNJ3	NAALAD2	Uncharacterized protein	N-acetylated-alpha-linked acidic dipeptidase 2	89	3	1.94	11	14	23
G1SLK6	J3KMX2	SMARCD2	SWIB domain-containing protein	SWI/SNF-related matrix-associated actin-dependent regulator of chromatin subfamily D member 2	98	2	1.93	3	2	10
G1SF56		MICAL2	Microtubule associated monooxygenase, calponin and LIM domain containing 2			1	1.92	3	4	6
U3KPI1		MPHOSPH10	U3 small nucleolar ribonucleoprotein protein MPP10			1	1.90	2	3	4
G1SKI8	Q6DKI1	RPL7L1	Uncharacterized protein	60S ribosomal protein L7-like 1	85	3	1.90	3	4	17
A0A140TAV7	J3KPF3	SLC3A2	4F2 cell-surface antigen heavy chain	4F2 cell-surface antigen heavy chain	81	2	1.90	20	106	57
G1SG11		COX4I1	Cytochrome c oxidase subunit 4 isoform 1, mitochondrial			1	1.90	3	2	21
G1TE69	J3KTL2	SRSF1	Uncharacterized protein	Serine/arginine-rich-splicing factor 1	100	3	1.89	6	9	25
G1TBS4	P20020	ATP2B1	Calcium-transporting ATPase	Plasma membrane calcium-transporting ATPase 1	97	2	1.88	6	4	7
G1SPF1	Q5JTH9	RRP12	NUC173 domain-containing protein	RRP12-like protein	92	2	1.88	7	10	8
	Q10570	CPSF1		Cleavage and polyadenylation specificity factor subunit 1		4	1.88	2	2	2
G1TG89	P62244	RPS15A	Uncharacterized protein	40S ribosomal protein S15a	100	3	1.87	7	24	55
G1TZB9	Q13595	TRA2A	RRM domain-containing protein	Transformer-2 protein homolog alpha	100	2	1.87	3	2	16
G1U5U0	Q9Y2X3	NOP58	Nop domain-containing protein	Nucleolar protein 58	92	2	1.87	11	3	29
G1SQ52	P52788	SMS	PABS domain-containing protein	Spermine synthase	99	2	1.87	3	5	18
G1SPY7	O14807	MRAS	Uncharacterized protein	Ras-related protein M-Ras	100	3	1.86	2	3	16
G1SJG9	Q6P4Q7	CNNM4	Uncharacterized protein	Metal transporter CNNM4	94	3	1.86	3	3	5
G1TS78	A0A075B6F6	HM13	Uncharacterized protein	Minor histocompatibility antigen H13 (Fragment)	84	3	1.86	7	20	21
G1SP22	Q99943	AGPAT1	1-acyl-sn-glycerol-3-phosphate acyltransferase	1-acyl-sn-glycerol-3-phosphate acyltransferase alpha	98	2	1.85	3	4	13
G1ST81	Q9P035	HACD3	Very-long-chain (3R)-3-hydroxyacyl-CoA dehydratase	Very-long-chain (3R)-3-hydroxyacyl-CoA dehydratase 3	96	2	1.85	4	7	18
G1TMU1	Q13151	HNRNPA0	Uncharacterized protein	Heterogeneous nuclear ribonucleoprotein A0	78	3	1.84	2	3	10
P35953	P98155	VLDLR	Very low-density lipoprotein receptor	Very low-density lipoprotein receptor	97	2	1.84	10	13	16
G1TRS0		DKC1	PUA domain-containing protein			1	1.84	5	7	14
	P38919	EIF4A3		Eukaryotic initiation factor 4A-III		4	1.83	13	16	40
G1U018		IGF2R	Insulin like growth factor 2 receptor			1	1.83	9	10	4
G1SGC2	P0C0S5	H2AFZ	Histone H2A	Histone H2A.Z	98	2	1.83	5	18	54
G1T5H5	Q15050	RRS1	Ribosome biogenesis regulatory protein	Ribosome biogenesis regulatory protein homolog	92	2	1.83	5	6	20
G1T3Y8	P10809	HSPD1	Uncharacterized protein	60 kDa heat shock protein, mitochondrial	99	3	1.83	34	52	75
G1TU85		FADS3	Cytochrome b5 heme-binding domain-containing protein			1	1.83	2	2	10
G1TRL8	J3QLI9	SNRPD1	Small nuclear ribonucleoprotein Sm D1	Small nuclear ribonucleoprotein Sm D1	100	2	1.83	3	7	28
G1TTM6	Q99797	MIPEP	Peptidase_M3 domain-containing protein	Mitochondrial intermediate peptidase	93	2	1.82	6	5	15
G1SZK8	P41223	BUD31	Uncharacterized protein	Protein BUD31 homolog	100	3	1.82	2	2	19
G1TV19	H3BPE7	FUS	FUS RNA binding protein	RNA-binding protein FUS	80	2	1.82	5	3	9
G1SLP3	H3BMM9	RNPS1	RNA binding protein with serine rich domain 1	RNA-binding protein with serine-rich domain 1 (Fragment)	99	2	1.81	2	3	14
G1T8F7	A0A494C128	NOP56	Nop domain-containing protein	Nucleolar protein 56	98	2	1.81	16	22	39
G1SPW1	Q29RF7	PDS5A	Uncharacterized protein	Sister chromatid cohesion protein PDS5 homolog A	99	3	1.80	3	2	3
G1T0I5	Q5JRX3	PITRM1	M16C_associated domain-containing protein	Presequence protease, mitochondrial	89	2	1.80	27	44	37
G1SMY1	A0A1W2PQ51	DDX17	Uncharacterized protein	Probable ATP-dependent RNA helicase DDX17	99	3	1.79	20	23	38
G1T7H4	Q8TDN6	BRIX1	Brix domain-containing protein	Ribosome biogenesis protein BRX1 homolog	93	2	1.79	4	6	13
G1U5M7	A0A3B3IUA2	SNU13	Ribonucleoprotein	Ribonucleoprotein	100	2	1.79	4	11	23
	A0A087WVZ9	POLR2E		DNA-directed RNA polymerases I, II, and III subunit RPABC1		4	1.79	2	3	17
G1TBV4	Q14566	MCM6	DNA helicase	DNA replication licensing factor MCM6	97	2	1.79	2	2	4
G1T8P3	P08621	SNRNP70	Small nuclear ribonucleoprotein U1 subunit 70	U1 small nuclear ribonucleoprotein 70 kDa	92	2	1.78	5	8	11
G1SUQ9	P11387	TOP1	DNA topoisomerase I	DNA topoisomerase 1	97	2	1.78	7	16	10
G1TNV7	O75600	GCAT	Glycine C-acetyltransferase	2-amino-3-ketobutyrate coenzyme A ligase, mitochondrial	94	2	1.77	7	6	27

G1SKQ8	Q13601	KRR1	KRR1 small subunit processome component	KRR1 small subunit processome component homolog	95	2	1.77	2	3	7
G1TA04	Q9UMS4	PRPF19	Uncharacterized protein	Pre-mRNA-processing factor 19	96	3	1.77	5	9	16
G1SL16	A0A0B4J1Z1	SRSF7	Uncharacterized protein	Serine/arginine-rich-splicing factor 7	100	3	1.77	4	4	31
G1SZA1	B4DHE8	MSI2	Uncharacterized protein	RNA-binding protein Musashi homolog 2	94	3	1.76	4	3	18
G1T8M9	P42704	LRPPRC	PPR_long domain-containing protein	Leucine-rich PPR motif-containing protein, mitochondrial	81	2	1.76	51	85	47
G1TKC9	Q15582	TGFBI	Transforming growth factor-beta-induced protein ig-h3	Transforming growth factor-beta-induced protein ig-h3	93	2	1.76	5	5	16
G1TJW3	Q15233	NONO	Uncharacterized protein	Non-POU domain-containing octamer-binding protein	99	3	1.76	12	39	33
G1T1P3	Q9H845	ACAD9	Uncharacterized protein	Acyl-CoA dehydrogenase family member 9, mitochondrial	88	3	1.76	9	12	25
G1TID3		ITPRIP	Inositol 1,4,5-trisphosphate receptor interacting protein			1	1.76	2	3	5
	A0A0C4DFX9	NELFA		Negative elongation factor A		4	1.75	2	2	7
G1TDQ3	J3QT28	BUB3	WD_REPEATS_REGION domain-containing protein	Mitotic checkpoint protein BUB3 (Fragment)	99	2	1.75	4	6	15
G1TKC4	Q15046	KARS	AA_TRNA_LIGASE_II domain-containing protein	Lysine--tRNA ligase	88	2	1.75	13	17	32
G1TVN1	I3L4X2	ABCC1	ATP binding cassette subfamily C member 1	Multidrug resistance-associated protein 1 (Fragment)	91	2	1.75	16	39	16
G1SQB6	Q9NRX1	PNO1	KH domain-containing protein	RNA-binding protein PNO1	96	2	1.75	2	7	14
G1SPN3	Q14573	ITPR3	Inositol 1,4,5-trisphosphate receptor type 3	Inositol 1,4,5-trisphosphate receptor type 3	95	2	1.74	8	8	6
	Q9NTZ6	RBM12		RNA-binding protein 12		4	1.74	2	3	5
G1TWL0	P22626	HNRNPA2B1	Uncharacterized protein	Heterogeneous nuclear ribonucleoproteins A2/B1	99	3	1.74	10	24	28
G1T6I6	Q12906	ILF3	Interleukin enhancer binding factor 3	Interleukin enhancer-binding factor 3	96	2	1.74	21	31	33
G1T1L7	A0A087WWS1	THOC1	Death domain-containing protein	THO complex subunit 1	98	2	1.74	3	3	10
G1SE74	Q7KZ85	SUPT6H	Transcription elongation factor spt6	Transcription elongation factor SPT6	99	2	1.73	5	5	4
G1SE36	R4GMU1	H6PD	GDH/6PGL endoplasmic bifunctional protein	GDH/6PGL endoplasmic bifunctional protein	85	2	1.73	8	5	15
G1SXW0	P43304	GPD2	Glycerol-3-phosphate dehydrogenase	Glycerol-3-phosphate dehydrogenase, mitochondrial	96	2	1.73	16	27	34
G1SGP1	P31930	UQCRC1	Uncharacterized protein	Cytochrome b-c1 complex subunit 1, mitochondrial	93	3	1.73	12	18	42
G1SEM4		THADA	DUF2428 domain-containing protein			1	1.73	2	2	1
	Q9BXP5	SRRT		Serrate RNA effector molecule homolog		4	1.72	7	4	7
G1T3N1	Q9NVP1	DDX18	RNA helicase	ATP-dependent RNA helicase DDX18	87	2	1.72	6	7	12
G1T6L0	Q5T3Q7	HEATR1	BP28CT domain-containing protein	HEAT repeat-containing protein 1	93	2	1.72	11	12	10
G1TYH7	Q9HDC9	APMAP	Adipocyte plasma membrane associated protein	Adipocyte plasma membrane-associated protein	94	2	1.71	12	29	34
G1SUA4	J3KS05	CBX1	Uncharacterized protein	Chromobox protein homolog 1 (Fragment)	98	3	1.71	4	12	39
	H0Y2W2	ATAD3A		ATPase family AAA domain-containing protein 3A (Fragment)		4	1.71	5	10	12
G1T9F6		NDUFA6	NADH:ubiquinone oxidoreductase subunit A6			1	1.71	2	2	19
G1SX50		RBM19	RNA binding motif protein 19			1	1.70	2	2	4
Q8HZQ5	E7EQR4	EZR	Ezrin	Ezrin	94	2	1.70	15	9	28
G1TB57		MRPS35	MRP-S28 domain-containing protein			1	1.70	3	4	16
G1SSJ7	P35232	PHB	PHB domain-containing protein	Prohibitin	100	2	1.70	17	48	81
G1SHV1	Q9Y333	LSM2	U6 snRNA-associated Sm-like protein LSm2	U6 snRNA-associated Sm-like protein LSm2	100	2	1.70	2	3	27
G1TC48	A0A0A0MRA5	HNRNPUL1	Uncharacterized protein	Heterogeneous nuclear ribonucleoprotein U-like protein 1	95	3	1.69	6	8	10
G1SN06	O60488	ACSL4	AMP-binding domain-containing protein	Long-chain-fatty-acid--CoA ligase 4	98	2	1.69	11	17	21
G1SI26	Q4VC31	CCDC58	Uncharacterized protein	Coiled-coil domain-containing protein 58	95	3	1.68	3	7	26
G1SYC5	Q9NQZ2	UTP3	Sas10 domain-containing protein	Something about silencing protein 10	82	2	1.68	3	3	8
	Q9NTI5-2	PDS5B		Isoform 2 of Sister chromatid cohesion protein PDS5 homolog B		4	1.68	2	2	2
	Q13247	SRSF6		Serine/arginine-rich splicing factor 6		4	1.68	3	4	10
	A0A087X2B1	RBFOX1		RNA binding protein fox-1 homolog		4	1.68	2	3	3
G1SLR8	Q9NZI8	IGF2BP1	Uncharacterized protein	Insulin-like growth factor 2 mRNA-binding protein 1	99	3	1.68	3	2	7
G1TBR6	Q69YN4	VIRMA	VIR_N domain-containing protein	Protein virilizer homolog	98	2	1.68	5	5	4
G1TX03	A0A0J9YW13	RBM8A	RNA-binding protein 8A	RNA-binding protein 8A (Fragment)	100	2	1.68	5	7	49
G1SJY0		MRPS5	S5 DRBM domain-containing protein			1	1.67	4	4	12
P98049		MT-CO2	Cytochrome c oxidase subunit 2			1	1.67	3	5	20
G1TAK1		COA3	Coiled-coil_56 domain-containing protein			1	1.67	2	5	19
G1SL60	Q15393	SF3B3	CPSF_A domain-containing protein	Splicing factor 3B subunit 3	98	2	1.67	17	25	20
G1SDP7	H0Y8P4	UTP15	UTP15, small subunit processome component	U3 small nucleolar RNA-associated protein 15 homolog (Fragment)	92	2	1.67	2	2	4
G1SIF2	Q16822	PCK2	Uncharacterized protein	Phosphoenolpyruvate carboxykinase [GTP], mitochondrial	95	3	1.67	23	73	47
G1T8H1	O95573	ACSL3	AMP-binding domain-containing protein	Long-chain-fatty-acid--CoA ligase 3	96	2	1.67	7	5	14
G1SGG2	V9GYM8	ARHGEF2	Uncharacterized protein	Rho guanine nucleotide exchange factor 2	95	3	1.67	13	19	21
G1SPY4		ASRGL1	Asparaginase like 1			1	1.67	3	4	11
G1SY30	O75643	SNRNP200	Uncharacterized protein	U5 small nuclear ribonucleoprotein 200 kDa helicase	100	3	1.67	39	60	30
	P02458	COL2A1		Collagen alpha-1(II) chain		4	1.66	4	2	5

G1SNV4	Q12874	SF3A3	Matrin-type domain-containing protein	Splicing factor 3A subunit 3	100	2	1.66	11	21	33
G1SYB9		ITGA3	Integrin subunit alpha 3			1	1.66	2	5	4
G1SW61	Q2TAY7	SMU1	Uncharacterized protein	WD40 repeat-containing protein SMU1	100	3	1.65	7	11	17
G1SJF4	A0A0A0MR51	FADS1	Fatty acid desaturase 1	Acyl-CoA (8-3)-desaturase	93	2	1.65	3	5	6
G1T0U4	Q9UIG0	BAZ1B	Bromodomain adjacent to zinc finger domain 1B	Tyrosine-protein kinase BAZ1B	94	2	1.65	4	3	4
G1TX53		NDUFA8	NADH dehydrogenase [ubiquinone] 1 alpha subcomplex subunit 8			1	1.65	2	2	9
G1T8H6	K7EK07	H3F3B	Histone H3	Histone H3 (Fragment)	98	2	1.64	7	22	52
G1SCR7	E9PF10	NUP155	Uncharacterized protein	Nuclear pore complex protein Nup155	90	3	1.64	14	19	16
G1SIM3	O95831	AIFM1	Uncharacterized protein	Apoptosis-inducing factor 1, mitochondrial	96	3	1.64	8	11	22
G1SCY8	Q9UKD2	MRTO4	Ribosome assembly factor mrt4	mRNA turnover protein 4 homolog	96	2	1.64	2	3	10
G1SM50	Q15427	SF3B4	Uncharacterized protein	Splicing factor 3B subunit 4	100	3	1.64	4	7	18
G1U862	Q01650	SLC7A5	Large neutral amino acids transporter small subunit 1	Large neutral amino acids transporter small subunit 1	94	2	1.64	4	9	14
G1SXR7		SLIRP	RRM domain-containing protein			1	1.64	3	8	34
G1TQJ5		DEK	SAP domain-containing protein			1	1.64	2	2	6
G1SD44	Q13505	MTX1	Uncharacterized protein	Metaxin-1	84	3	1.64	6	8	21
	I3L3B0	C1QBP		Complement component 1 Q subcomponent-binding protein, mitochondrial		4	1.63	2	13	32
G1T2U6	A0A0C4DG89	DDX46	Uncharacterized protein	Probable ATP-dependent RNA helicase DDX46	99	3	1.63	6	4	8
Q28618	P67809	YBX1	Nuclease-sensitive element-binding protein 1	Nuclease-sensitive element-binding protein 1	99	2	1.63	4	96	22
G1SIW1	O43143	DHX15	Uncharacterized protein	Pre-mRNA-splicing factor ATP-dependent RNA helicase DHX15	99	3	1.63	14	21	24
G1SFE6	P08579	SNRPB2	Uncharacterized protein	U2 small nuclear ribonucleoprotein B~~	96	3	1.63	3	2	16
G1TTN9	Q9P258	RCC2	Regulator of chromosome condensation 2	Protein RCC2	99	2	1.63	4	4	13
	P26368	U2AF2		Splicing factor U2AF 65 kDa subunit		4	1.63	8	12	29
G1SSH0	A0A087WUB9	CTNNBL1	DUF1716 domain-containing protein	Beta-catenin-like protein 1	96	2	1.62	5	7	12
G1TMU2	P52597	HNRNPF	Uncharacterized protein	Heterogeneous nuclear ribonucleoprotein F	99	3	1.62	11	172	44
G1TES6	Q99714	HSD17B10	Uncharacterized protein	3-hydroxyacyl-CoA dehydrogenase type-2	92	3	1.62	13	65	84
G1T3X2	H7BZW6	SAP18	Histone deacetylase complex subunit SAP18	Histone deacetylase complex subunit SAP18 (Fragment)	98	2	1.62	2	3	15
G1SV60	J3KS45	TMCO1	Calcium load-activated calcium channel	Calcium load-activated calcium channel (Fragment)	95	2	1.62	2	2	13
G1SU13	Q5VW52	GPAM	Glycerol-3-phosphate acyltransferase 1, mitochondrial	Glycerol-3-phosphate acyltransferase 1, mitochondrial	94	2	1.62	3	5	8
G1STI3	Q07666	KHDRBS1	KH domain-containing protein	KH domain-containing, RNA-binding, signal transduction-associated protein 1	99	2	1.62	4	10	12
	Q15428	SF3A2		Splicing factor 3A subunit 2		4	1.61	2	2	4
G1SR13	Q9UJZ1	STOML2	PHB domain-containing protein	Stomatin-like protein 2, mitochondrial	96	2	1.61	15	26	54
G1T5I9	F8WJN3	CPSF6	RRM domain-containing protein	Cleavage and polyadenylation-specificity factor subunit 6	99	2	1.61	3	4	9
G1SXC8		NXF1	Nuclear RNA export factor 1			1	1.61	3	3	8
	O60518	RANBP6		Ran-binding protein 6		4	1.61	2	7	3
G1T696	Q09161	NCBP1	MIF4G domain-containing protein	Nuclear cap-binding protein subunit 1	99	2	1.61	9	9	19
G1TAH7	P29401	TKT	TRANSKETOLASE_1 domain-containing protein	Transketolase	94	2	1.61	20	45	49
	Q8WX92	NELFB		Negative elongation factor B		4	1.61	3	4	6
G1THH7		SUN2	SUN domain-containing protein			1	1.61	7	10	16
G1TE50	Q9Y221	NIP7	60S ribosome subunit biogenesis protein NIP7 homolog	60S ribosome subunit biogenesis protein NIP7 homolog	97	2	1.61	2	2	16
G1U1X6		NT5C3A	5~-nucleotidase			1	1.60	2	2	9
G1TIR7	O95777	LSM8	U6 snRNA-associated Sm-like protein LSm8	U6 snRNA-associated Sm-like protein LSm8	99	2	1.60	3	52	52
G1SWU9		FAM20B	Fam20C domain-containing protein			1	1.60	2	4	7
G1TXA3	P82921	MRPS21	Uncharacterized protein	28S ribosomal protein S21, mitochondrial	93	3	1.60	3	3	38
	A0A3B3IRT8	SSR1		Translocon-associated protein subunit alpha		4	1.60	4	75	21
G1SMM7	P62318	SNRPD3	Small nuclear ribonucleoprotein Sm D3	Small nuclear ribonucleoprotein Sm D3	100	2	1.60	3	5	29
G1T7B5	Q9H0S4	DDX47	Uncharacterized protein	Probable ATP-dependent RNA helicase DDX47	97	3	1.59	2	5	6
G1SDJ7	B1ANR0	PABPC4	Polyadenylate-binding protein	Polyadenylate-binding protein	92	2	1.59	17	12	27
G1SLV3	P42285	MTREX	Uncharacterized protein	Exosome RNA helicase MTR4	99	3	1.59	8	15	11
G1SNF2	P13995	MTHFD2	Uncharacterized protein	Bifunctional methylenetetrahydrofolate dehydrogenase/cyclohydrolase, mitochondrial	93	3	1.58	12	28	57
	F6RGN5	SLC25A10		Mitochondrial dicarboxylate carrier		4	1.58	3	6	11
G1SJ32	A0A0A0MTB8	WDR36	WD_REPEATS_REGION domain-containing protein	WD repeat-containing protein 36	94	2	1.58	2	2	4
	Q9Y2Z2	MTO1		Protein MTO1 homolog, mitochondrial		4	1.58	2	2	4
	O96008	TOMM40		Mitochondrial import receptor subunit TOM40 homolog		4	1.58	9	14	37
G1T6T8	O94906	PRPF6	Uncharacterized protein	Pre-mRNA-processing factor 6	96	3	1.58	5	5	7
G1SRZ0		TOR2A	Torsin family 2 member A			1	1.58	2	3	20
G1SRF1	J3KT10	NUP85	Nuclear pore complex protein Nup85	Nuclear pore complex protein Nup85	84	2	1.58	7	10	16
G1TZN7		COX5A	Cytochrome c oxidase subunit 5A			1	1.57	2	2	7

G1U9B4	P62995	TRA2B	RRM domain-containing protein	Transformer-2 protein homolog beta	100	2	1.57	4	8	16
	Q01105-2	SET		Isoform 2 of Protein SET		4	1.57	6	7	36
G1T2A9	Q14315	FLNC	Uncharacterized protein	Filamin-C	90	3	1.57	73	93	40
G1SQZ4	Q9Y230	RUVBL2	RuvB-like helicase	RuvB-like 2	99	2	1.57	15	41	49
G1SW10	O94776	MTA2	Uncharacterized protein	Metastasis-associated protein MTA2	99	3	1.57	6	5	12
G1T5H2	Q9H0D6	XRN2	5~-3~ exoribonuclease	5~-3~ exoribonuclease 2	98	2	1.56	8	10	14
	A0A0C4DGG8	CCAR1		Cell division cycle and apoptosis regulator protein 1 (Fragment)		4	1.56	3	3	5
G1SQ54	A0A087WT44	HMOX2	Heme oxygenase	Heme oxygenase 2	89	2	1.56	7	10	33
	A0A087WTP3	KHSRP		Far upstream element-binding protein 2		4	1.56	11	11	17
G1T617	P49756	RBM25	Uncharacterized protein	RNA-binding protein 25	99	3	1.56	5	4	9
G1SME4	Q9BWF3	RBM4	RNA-binding protein 4	RNA-binding protein 4	99	2	1.55	7	7	20
G1TGK9	Q08211	DHX9	Uncharacterized protein	ATP-dependent RNA helicase A	93	3	1.55	21	32	31
G1SCT6	E9PB90	HK2	Uncharacterized protein	Hexokinase-2	96	3	1.55	10	3	16
G1SQA4	Q6UWP7	LCLAT1	PlsC domain-containing protein	Lysocardiolipin acyltransferase 1	89	2	1.55	5	6	15
G1SCK0	Q6P2Q9	PRPF8	MPN domain-containing protein	Pre-mRNA-processing-splicing factor 8	100	2	1.55	35	47	22
G1SF08	C9K025	RPL35A	Uncharacterized protein	60S ribosomal protein L35a (Fragment)	99	3	1.55	3	2	23
	Q08945	SSRP1		FACT complex subunit SSRP1		4	1.54	3	3	9
G1TKJ4	P61601	NCALD	Uncharacterized protein	Neurocalcin-delta	100	3	1.54	6	7	38
G1TCA0	Q96KR1	ZFR	DZF domain-containing protein	Zinc finger RNA-binding protein	99	2	1.54	3	2	4
G1SS51		MRPS25	L51_S25_CI-B8 domain-containing protein			1	1.54	3	3	22
G1SMI7	V9GYL9	DAP3	Uncharacterized protein	28S ribosomal protein S29, mitochondrial (Fragment)	86	3	1.54	8	7	28
G1SZR8	P51858	HDGF	Heparin binding growth factor	Hepatoma-derived growth factor	96	2	1.54	5	4	22
G1SWW7	B4DJK0	SRSF5	Uncharacterized protein	Serine/arginine-rich-splicing factor 5	100	3	1.54	2	4	8
G1SFH9	A0A2R8Y543	CTNNB1	Uncharacterized protein	Catenin beta-1	100	3	1.54	25	53	46
G1SIP2	A0A3B3ITJ4	HNRNPL	Uncharacterized protein	Heterogeneous nuclear ribonucleoprotein L (Fragment)	93	3	1.54	18	37	53
G1T116	Q96TA2	YME1L1	AAA domain-containing protein	ATP-dependent zinc metalloprotease YME1L1	89	2	1.53	4	6	9
G1TCU1	I3L2K5	ZC3H7A	Uncharacterized protein	Zinc finger CCCH domain-containing protein 7A (Fragment)	95	3	1.53	2	2	2
G1TBS2	Q9Y265	RUVBL1	RuvB-like helicase	RuvB-like 1	100	2	1.53	14	27	47
G1T120	S4R369	MRPL37	Uncharacterized protein	39S ribosomal protein L37, mitochondrial	84	3	1.52	6	4	19
G1SLJ8	Q8IY81	FTSJ3	pre-rRNA processing protein FTSJ3	pre-rRNA 2~-O-ribose RNA methyltransferase FTSJ3	85	2	1.52	7	6	19
G1SUP4	J3QRS9	ZNF207	Uncharacterized protein	BUB3-interacting and GLEBS motif-containing protein ZNF207	100	3	1.52	3	4	8
G1T8B3	Q8IZL8	PELP1	Uncharacterized protein	Proline-, glutamic acid- and leucine-rich protein 1	91	3	1.52	5	8	6
G1SUS6	D6RD69	SAR1B	Uncharacterized protein	GTP-binding protein SAR1b (Fragment)	98	3	1.52	4	2	25
G1TI97	Q13769	THOC5	Uncharacterized protein	THO complex subunit 5 homolog	97	3	1.52	3	3	8
G1SQ45	A0A0C4DG98	THOC2	Uncharacterized protein	THO complex subunit 2	98	3	1.52	4	4	5
	U3KQK1	LSM4		U6 snRNA-associated Sm-like protein LSm4		4	1.52	2	5	13
G1SLK4	Q8WVX9	FAR1	Fatty acyl-CoA reductase	Fatty acyl-CoA reductase 1	93	2	1.51	2	2	7
G1SWF3		DHODH	Dihydroorotate dehydrogenase (quinone), mitochondrial			1	1.51	4	4	15
P27124	Q02790	FKBP4	Peptidyl-prolyl cis-trans isomerase FKBP4	Peptidyl-prolyl cis-trans isomerase FKBP4	91	2	1.51	9	10	26
G1THL2		FTL	Ferritin			1	1.51	2	2	17
	K7EIE8	MBD3		Methyl-CpG binding domain protein 3, isoform CRA_b		4	1.51	2	2	14
	Q9NRZ7	AGPAT3		1-acyl-sn-glycerol-3-phosphate acyltransferase gamma		4	1.51	2	3	4
G1SFD8	Q9BUQ8	DDX23	Uncharacterized protein	Probable ATP-dependent RNA helicase DDX23	99	3	1.50	7	6	11
G1T2Y5	P12270	TPR	TPR_MLP1_2 domain-containing protein	Nucleoprotein TPR	97	2	1.50	26	33	14
U3KM30	Q969X5	ERGIC1	Uncharacterized protein	Endoplasmic reticulum-Golgi intermediate compartment protein 1	99	3	1.50	3	4	18
G1SEF1		NDUFC2	NADH dehydrogenase [ubiquinone] 1 subunit C2			1	1.50	2	2	19
G1SZZ1	Q9H0V1	TMEM168	Transmembrane protein 168	Transmembrane protein 168	96	2	1.50	5	6	13
G1T6E6		NOC3L	Nucleolar complex protein 3 homolog			1	1.50	4	2	9
	Q96PK6-5	RBM14		Isoform 5 of RNA-binding protein 14		4	1.50	5	2	24
G1T011	Q9Y5B9	SUPT16H	Uncharacterized protein	FACT complex subunit SPT16	100	3	1.50	7	8	10
G1TVG7	O75533	SF3B1	SF3b1 domain-containing protein	Splicing factor 3B subunit 1	99	2	1.50	20	36	27
G1TH59	Q14498	RBM39	Uncharacterized protein	RNA-binding protein 39	91	3	1.50	7	9	21
G1SF95	H0Y8G5	HNRNPD	Heterogeneous nuclear ribonucleoprotein D	Heterogeneous nuclear ribonucleoprotein D0 (Fragment)	99	2	1.50	7	15	26
G1SUF4	O15269	SPTLC1	Aminotran_1_2 domain-containing protein	Serine palmitoyltransferase 1	95	2	1.50	6	11	15
G1SGA5	Q9HCJ6	VAT1L	PKS_ER domain-containing protein	Synaptic vesicle membrane protein VAT-1 homolog-like	96	2	1.50	3	2	10
G1U2E6	J3KPX7	PHB2	PHB domain-containing protein	Prohibitin-2	99	2	1.50	17	109	61
G1SGI8	O95881	TXNDC12	Thioredoxin domain-containing protein	Thioredoxin domain-containing protein 12	95	2	1.50	5	10	37

G1T134		ABHD11	Abhydrolase domain containing 11			1	1.49	3	2	16
G1SR61	H7BXY3	DHX30	Uncharacterized protein	ATP-dependent RNA helicase DHX30	98	3	1.49	7	12	12
G1TZQ6		NDUFA10	NADH dehydrogenase [ubiquinone] 1 alpha subcomplex subunit 10, mitochondrial			1	1.49	4	7	19
	P35268	RPL22		60S ribosomal protein L22		4	1.49	2	7	32
G1U864		TRRAP	Transformation/transcription domain associated protein			1	1.49	3	3	1
G1U6N8	Q14980	NUMA1	Nuclear mitotic apparatus protein 1	Nuclear mitotic apparatus protein 1	90	2	1.49	21	26	15
G1SHL0		SBNO1	Strawberry notch homolog 1			1	1.49	2	2	3
G1SQ11	P54886	ALDH18A1	Delta-1-pyrroline-5-carboxylate synthase	Delta-1-pyrroline-5-carboxylate synthase	97	2	1.49	30	73	53
G1SQB1	P22033	MMUT	B12-binding domain-containing protein	Methylmalonyl-CoA mutase, mitochondrial	96	2	1.49	2	2	4
G1SDX3	E9PCY5	TOP2B	DNA topoisomerase 2	DNA topoisomerase 2 (Fragment)	99	2	1.48	14	21	12
G1SWK4	B4E1G1	DERL1	Derlin	Derlin	99	2	1.48	2	3	10
G1SQU6	O75489	NDUFS3	Complex1_30kDa domain-containing protein	NADH dehydrogenase [ubiquinone] iron-sulfur protein 3, mitochondrial	91	2	1.48	4	3	21
G1SUR4		ZADH2	PKS_ER domain-containing protein			1	1.48	3	2	18
G1SI76		MRPL43	L51_S25_CI-B8 domain-containing protein			1	1.48	3	3	19
G1T5H0	A0A3B3IU24	HTRA1	PDZ domain-containing protein	Serine protease HTRA1	94	2	1.48	2	3	10
G1T359	P28331	NDUFS1	Uncharacterized protein	NADH-ubiquinone oxidoreductase 75 kDa subunit, mitochondrial	98	3	1.48	18	29	37
G1SUQ4		DNTTIP2	Fcf2 domain-containing protein			1	1.47	2	3	4
G1TEN4	Q9HD33	MRPL47	Uncharacterized protein	39S ribosomal protein L47, mitochondrial	79	3	1.47	5	7	19
G1SP02	A8MT40	PDPR	Uncharacterized protein	Pyruvate dehydrogenase phosphatase regulatory subunit, mitochondrial	95	3	1.47	4	3	7
G1SRF7	P38646	HSPA9	Uncharacterized protein	Stress-70 protein, mitochondrial	99	3	1.47	36	418	62
	K7EKE6	LONP1		Lon protease homolog, mitochondrial		4	1.47	19	44	28
G1THY5	Q9Y3C6	PPIL1	Peptidyl-prolyl cis-trans isomerase	Peptidyl-prolyl cis-trans isomerase-like 1	100	2	1.47	2	2	29
G1SRH7	P84103	SRSF3	RRM domain-containing protein	Serine/arginine-rich splicing factor 3	100	2	1.47	4	5	24
G1U4G9	O00299	CLIC1	Chloride intracellular channel protein	Chloride intracellular channel protein 1	98	2	1.47	6	9	42
	Q12931	TRAP1		Heat shock protein 75 kDa, mitochondrial		4	1.47	7	2	12
G1SGY1	Q86TB9	PATL1	PAT1 domain-containing protein	Protein PAT1 homolog 1	97	2	1.46	2	2	4
G1TIT1	O75477	ERLIN1	PHB domain-containing protein	Erlin-1	98	2	1.46	11	8	41
G1SVW9		VEZT	Vezatin domain-containing protein			1	1.46	2	2	4
G1TUK6	O14656	TOR1A	Torsin family 1 member A	Torsin-1A	90	2	1.46	7	7	25
G1STP6	P35221	CTNNA1	Catenin alpha-1	Catenin alpha-1	99	2	1.46	31	69	48
G1TG28	P80723	BASP1	Brain abundant membrane attached signal protein 1	Brain acid soluble protein 1	61	2	1.46	5	6	51
G1U7I9	A0A0G2JJZ9	DDX39B	Uncharacterized protein	Spliceosome RNA helicase DDX39B (Fragment)	95	3	1.46	14	29	46
A0A0A0MQQ6	D6RBW1	EIF4E	Eukaryotic translation initiation factor 4E	Eukaryotic translation initiation factor 4E	98	2	1.46	3	3	20
G1TAE2	Q15717	ELAVL1	ELAV-like protein	ELAV-like protein 1	99	2	1.46	10	42	32
G1TUB8	Q5VVC8	RPL11	Uncharacterized protein	60S ribosomal protein L11	100	3	1.46	5	13	34
G1SGY8	Q3ZCQ8	TIMM50	Mitochondrial import inner membrane translocase subunit TIM50	Mitochondrial import inner membrane translocase subunit TIM50	96	2	1.46	6	11	23
G1SM52	Q96AG4	LRRC59	Uncharacterized protein	Leucine-rich repeat-containing protein 59	97	3	1.45	13	23	48
G1T0N4	P06748	NPM1	Uncharacterized protein	Nucleophosmin	89	3	1.45	11	43	38
G1T7D1		HIST1H1C	H15 domain-containing protein			1	1.45	10	22	33
	P62304	SNRPE		Small nuclear ribonucleoprotein E		4	1.45	3	13	52
	Q8N201	INTS1		Integrator complex subunit 1		4	1.45	4	4	2
G1TX78	Q15388	TOMM20	Uncharacterized protein	Mitochondrial import receptor subunit TOM20 homolog	100	3	1.45	3	26	28
G1SMH6	Q9P0M6	H2AFY2	Core histone macro-H2A	Core histone macro-H2A.2	99	2	1.45	6	5	23
B7NZN9	P51571	SSR4	Signal sequence receptor, delta (Predicted)	Translocon-associated protein subunit delta	97	2	1.45	5	10	36
G1T647		GCLM	Glutamate-cysteine ligase modifier subunit			1	1.45	3	4	16
G1SF97		MRPL46	MRP-L46 domain-containing protein			1	1.45	4	11	18
	Q13045	FLII		Protein flightless-1 homolog		4	1.45	6	5	6
G1SP24	E7EQB9	POLR1C	RPOLD domain-containing protein	DNA-directed RNA polymerases I and III subunit RPAC1	84	2	1.45	4	6	25
	P31040	SDHA		Succinate dehydrogenase [ubiquinone] flavoprotein subunit, mitochondrial		4	1.45	13	7	34
G1U2E5	P08243	ASNS	Asparagine synthetase [glutamine-hydrolyzing]	Asparagine synthetase [glutamine-hydrolyzing]	87	2	1.44	8	6	19
G1SES8	G5E9V5	MRPS22	Uncharacterized protein	28S ribosomal protein S22, mitochondrial	84	3	1.44	2	2	6
G1SLM0	A0A087WTT1	PABPC1	Polyadenylate-binding protein	Polyadenylate-binding protein	99	2	1.44	18	18	38
G1SRY1	Q8TCS8	PNPT1	S1 motif domain-containing protein	Polyribonucleotide nucleotidyltransferase 1, mitochondrial	94	2	1.44	7	11	14
G1TQ57	H3BP71	RNF40	E3 ubiquitin protein ligase	E3 ubiquitin protein ligase	92	2	1.44	3	2	6
G1STZ4	P11413	G6PD	Glucose-6-phosphate 1-dehydrogenase	Glucose-6-phosphate 1-dehydrogenase	94	2	1.44	5	9	11
G1SDL0	Q96DX4	RSPRY1	Uncharacterized protein	RING finger and SPRY domain-containing protein 1	93	3	1.44	4	4	16
G1TM60		NDUFA9	Epimerase domain-containing protein			1	1.44	6	10	20

G1SQP9	E7EMS6	COMT	Catechol-O-methyltransferase	Catechol O-methyltransferase (Fragment)	78	2	1.43	10	29	52
G1T5L3	Q9H9J2	MRPL44	Uncharacterized protein	39S ribosomal protein L44, mitochondrial	90	3	1.43	3	4	15
G1TEZ1	G3V198	NUP160	Uncharacterized protein	Nuclear pore complex protein Nup160 (Fragment)	93	3	1.43	14	21	18
G1SHJ3		KDELC1	CAP10 domain-containing protein			1	1.43	5	6	14
G1SIP9	H0Y9G6	MRPL3	Uncharacterized protein	39S ribosomal protein L3, mitochondrial (Fragment)	89	3	1.43	4	4	21
G1SJI6		MRPS23	MRP-S23 domain-containing protein			1	1.43	5	6	34
G1SUY2	P05091	ALDH2	Aldedh domain-containing protein	Aldehyde dehydrogenase, mitochondrial	91	2	1.43	12	18	36
G1SD25	Q5SRE5	NUP188	Nucleoporin 188	Nucleoporin NUP188 homolog	94	2	1.43	10	10	9
G1SU17		NSDHL	3Beta_HSD domain-containing protein			1	1.43	2	2	10
G1SIQ9	Q9GZL7	WDR12	Ribosome biogenesis protein WDR12	Ribosome biogenesis protein WDR12	97	2	1.43	3	2	15
G1T7L0		CTSC	Pept_C1 domain-containing protein			1	1.42	5	4	20
G1SHG0	P62899	RPL31	Uncharacterized protein	60S ribosomal protein L31	100	3	1.42	6	13	45
G1SKP2	H0Y8C6	IPO5	Importin N-terminal domain-containing protein	Importin-5 (Fragment)	99	2	1.42	28	48	42
G1TFE0	H0YHA7	RPL18	Ribosomal_L18e/L15P domain-containing protein	60S ribosomal protein L18 (Fragment)	90	2	1.42	3	9	22
G1TFZ7	O15355	PPM1G	PPM-type phosphatase domain-containing protein	Protein phosphatase 1G	97	2	1.42	3	2	10
U3KPG6		ICAM1	Intercellular adhesion molecule 1			1	1.42	8	14	20
G1SFH4		MRPL24	KOW domain-containing protein			1	1.42	4	7	32
G1T301	A0A0A0MRN4	ZNF326	Uncharacterized protein	DBIRD complex subunit ZNF326	96	3	1.42	4	4	10
G1SSM6	Q8WUM0	NUP133	Nucleoporin_C domain-containing protein	Nuclear pore complex protein Nup133	91	2	1.41	14	19	23
G1TTY7	M0R1A7	RPL18A	60S ribosomal protein L18a	60S ribosomal protein L18a	90	2	1.41	4	4	19
G1SHL9	Q15006	EMC2	TPR_REGION domain-containing protein	ER membrane protein complex subunit 2	99	2	1.41	6	13	34
G1SZ44	B8ZZL8	HSPE1	Uncharacterized protein	10 kDa heat shock protein, mitochondrial	100	3	1.41	7	7	58
G1STH0	Q15459	SF3A1	Uncharacterized protein	Splicing factor 3A subunit 1	98	3	1.41	8	11	13
G1TDH4	P30048	PRDX3	Thioredoxin domain-containing protein	Thioredoxin-dependent peroxide reductase, mitochondrial	86	2	1.41	8	24	36
G1T925		GPX8	Glutathione peroxidase			1	1.41	4	6	20
G1T890	Q8NE86	MCU	MCU domain-containing protein	Calcium uniporter protein, mitochondrial	98	2	1.41	10	18	33
	G3V1C3	API5		Apoptosis inhibitor 5		4	1.41	8	17	27
G1T069	P57740	NUP107	Nuclear pore complex protein	Nuclear pore complex protein Nup107	94	2	1.41	9	18	16
	A0A0J9YVP6	PUF60		Poly(U)-binding-splicing factor PUF60 (Fragment)		4	1.41	8	13	23
G1TDJ9	F6Y5H0	RBMS1	RNA binding motif single stranded interacting protein 1	RNA-binding motif, single-stranded-interacting protein 1	97	2	1.41	2	3	14
G1T1R4		NR3C1	Glucocorticoid receptor			1	1.41	2	3	6
G1TA05	A2AE48	TRIM26	Uncharacterized protein	Tripartite motif-containing protein 26 (Fragment)	92	3	1.41	2	2	4
G1SGL3		SRSF11	RRM domain-containing protein			1	1.41	2	4	5
G1T0V4	F5H013	SNRPG	Small nuclear ribonucleoprotein G	Small nuclear ribonucleoprotein G	98	2	1.41	3	18	58
G1SCW7	Q9UG63	ABCF2	Uncharacterized protein	ATP-binding cassette sub-family F member 2	99	3	1.40	6	9	11
G1U800	Q8NC56	LEMD2	MSC domain-containing protein	LEM domain-containing protein 2	79	2	1.40	5	3	19
G1SV32	P18124	RPL7	Uncharacterized protein	60S ribosomal protein L7	98	3	1.40	13	14	46
G1SDN4	O60506-3	SYNCRIP	Uncharacterized protein	Isoform 3 of Heterogeneous nuclear ribonucleoprotein Q	100	3	1.40	22	48	49
G1TTL1	A0A0B4J1W3	NAA15	Uncharacterized protein	N-alpha-acetyltransferase 15, NatA auxiliary subunit	99	3	1.40	7	11	13
P30947	P08238	HSP90AB1	Heat shock protein HSP 90-beta	Heat shock protein HSP 90-beta	99	2	1.40	34	93	61
G1SRX2	Q15029	EFTUD2	Tr-type G domain-containing protein	116 kDa U5 small nuclear ribonucleoprotein component	100	2	1.40	18	34	31
G1SKZ8	P62906	RPL10A	Ribosomal protein	60S ribosomal protein L10a	100	2	1.40	13	45	53
	B1ANM7	FAF1		FAS-associated factor 1		4	1.40	3	3	7
	A0A0U1RQF0	FASN		Fatty acid synthase		4	1.40	8	12	4
G1U6X4	F8VQZ7	METAP2	Methionine aminopeptidase 2	Methionine aminopeptidase 2	94	2	1.40	5	21	17
G1SFI7	P49915	GMPS	Uncharacterized protein	GMP synthase [glutamine-hydrolyzing]	99	3	1.40	6	6	10
G1SFQ3		MRPL2	Ribosomal_L2_C domain-containing protein			1	1.40	3	4	19
G1SD24	A0A494BZU6	PARN	R3H domain-containing protein	Poly(A)-specific ribonuclease PARN (Fragment)	96	2	1.40	2	2	5
G1SLD7	Q9NXF1	TEX10	Ipi1_N domain-containing protein	Testis-expressed protein 10	94	2	1.40	3	4	5
	P37198	NUP62		Nuclear pore glycoprotein p62		4	1.39	4	4	11
G1T2N4	E7ET15	U2SURP	Uncharacterized protein	U2 snRNP-associated SURP motif-containing protein	99	3	1.39	6	11	8
G1TV43	O60218	AKR1B10	Aldo_ket_red domain-containing protein	Aldo-keto reductase family 1 member B10	86	2	1.39	10	18	31
G1SU33		NSUN2	NOP2/Sun RNA methyltransferase family member 2			1	1.39	3	3	7
	B7WP74	CWC22		Pre-mRNA-splicing factor CWC22 homolog (Fragment)		4	1.39	3	2	7
	K7EK33	DAZAP1		DAZ-associated protein 1		4	1.39	4	6	18
G1TTB5	A0A0D9SEM4	SRSF4	Serine and arginine rich splicing factor 4	Serine/arginine-rich-splicing factor 4 (Fragment)	75	2	1.39	3	2	8
G1SYD6	P02545	LMNA	Uncharacterized protein	Prelamin-A/C	98	3	1.39	33	90	52

G1T095		TMEM147	Transmembrane protein 147			1	1.39	2	2	18
G1TJR5	Q9NZL4	HSPBP1	HSPA (Hsp70) binding protein 1	Hsp70-binding protein 1	96	2	1.39	4	7	22
	P62424	RPL7A		60S ribosomal protein L7a		4	1.39	12	34	45
	P43897	TSFM		Elongation factor Ts, mitochondrial		4	1.39	3	4	17
G1T194	Q9ULX6	AKAP8L	A-kinase anchoring protein 8 like	A-kinase anchor protein 8-like	86	2	1.38	4	5	10
	Q9P0J1	PDP1		[Pyruvate dehydrogenase [acetyl-transferring]]-phosphatase 1, mitochondrial		4	1.38	2	3	5
G1T380	Q02388	COL7A1	Uncharacterized protein	Collagen alpha-1(VII) chain	87	3	1.38	16	24	8
	A0A024R4M0	RPS9		40S ribosomal protein S9		4	1.38	12	19	40
	Q12873	CHD3		Chromodomain-helicase-DNA-binding protein 3		4	1.38	7	2	6
G1SSF2		ENG	Endoglin			1	1.38	5	9	12
G1SFG0	A0A087WVP1	FAT1	Uncharacterized protein	Protocadherin Fat 1	92	3	1.38	11	12	4
G1SMI2	Q9Y305	ACOT9	Acyl-CoA thioesterase 9	Acyl-coenzyme A thioesterase 9, mitochondrial	83	2	1.38	13	11	33
G1T1L4		GRWD1	WD_REPEATS_REGION domain-containing protein			1	1.38	3	3	13
G1TEN1	H7C5S0	ACTL6A	Uncharacterized protein	Actin-like protein 6A (Fragment)	99	3	1.38	4	4	15
G1TDQ1	Q9H0C8	ILKAP	PPM-type phosphatase domain-containing protein	Integrin-linked kinase-associated serine/threonine phosphatase 2C	94	2	1.38	2	3	8
G1SR36	C9JG87	MRPL39	Uncharacterized protein	39S ribosomal protein L39, mitochondrial (Fragment)	86	3	1.38	5	5	12
G1SST6		ARMC10	Arm_2 domain-containing protein			1	1.37	2	3	12
G1T6M1	Q96DA6	DNAJC19	J domain-containing protein	Mitochondrial import inner membrane translocase subunit TIM14	100	2	1.37	2	3	19
P12345	P00505	GOT2	Aspartate aminotransferase, mitochondrial	Aspartate aminotransferase, mitochondrial	94	2	1.37	18	94	46
G1SWA6	A0A2R8Y4T4	SMARCE1	HMG box domain-containing protein	SWI/SNF-related matrix-associated actin-dependent regulator of chromatin subfamily E member 1	95	2	1.37	3	3	9
G1U2T2	H0YIB4	SRSF9	Uncharacterized protein	Serine/arginine-rich-splicing factor 9 (Fragment)	65	3	1.37	3	4	15
G1U535	Q9HBH5	RDH14	Uncharacterized protein	Retinol dehydrogenase 14	91	3	1.37	4	3	13
U3KMH9	P40926	MDH2	Malate dehydrogenase	Malate dehydrogenase, mitochondrial	94	2	1.37	15	64	60
	B5MDE0	RFT1		Protein RFT1 homolog		4	1.37	2	3	4
G1SGY0	O00267	SUPT5H	Transcription elongation factor SPT5	Transcription elongation factor SPT5	98	2	1.37	8	7	12
G1SJW8	H3BND8	USP7	Uncharacterized protein	Ubiquitin carboxyl-terminal hydrolase (Fragment)	99	3	1.37	9	8	16
G1SEX6	Q9H2U1	DHX36	Uncharacterized protein	ATP-dependent DNA/RNA helicase DHX36	94	3	1.37	3	3	5
	Q16394	EXT1		Exostosin-1		4	1.37	2	3	5
G1TAV2		SMCHD1	SMC hinge domain-containing protein			1	1.37	3	3	2
G1U448	E9PEB5	FUBP1	Uncharacterized protein	Far upstream element-binding protein 1	93	3	1.36	10	7	16
G1TMM7	H0YN26	ANP32A	LRRcap domain-containing protein	Acidic leucine-rich nuclear phosphoprotein 32 family member A	93	2	1.36	7	9	39
G1STF9	Q13347	EIF3I	Eukaryotic translation initiation factor 3 subunit I	Eukaryotic translation initiation factor 3 subunit I	100	2	1.36	10	11	41
G1SD98	Q13523	PRPF4B	Pre-mRNA processing factor 4B	Serine/threonine-protein kinase PRP4 homolog	98	2	1.36	4	5	4
G1U636	G3V153	CAPRIN1	Uncharacterized protein	Caprin-1	98	3	1.36	7	21	12
G1STS0	Q9Y2S7	POLDIP2	ApaG domain-containing protein	Polymerase delta-interacting protein 2	98	2	1.36	6	5	26
G1SF32	O94826	TOMM70	TPR_REGION domain-containing protein	Mitochondrial import receptor subunit TOM70	97	2	1.36	16	38	24
G1TEI2		FDX1	2Fe-2S ferredoxin-type domain-containing protein			1	1.36	2	3	20
G1TD99	A0A1B0GUX9	PCCA	Uncharacterized protein	Propionyl-CoA carboxylase alpha chain, mitochondrial (Fragment)	96	3	1.35	8	10	15
	Q9NYU2	UGGT1		UDP-glucose:glycoprotein glucosyltransferase 1		4	1.35	19	3	19
G1TCU4	H3BUU9	CDH11	Uncharacterized protein	Cadherin-11	99	3	1.35	3	7	9
G1SGB5	P23246	SFPQ	Splicing factor proline and glutamine rich	Splicing factor, proline- and glutamine-rich	100	2	1.35	13	23	21
	Q9Y5L4	TIMM13		Mitochondrial import inner membrane translocase subunit Tim13		4	1.35	3	4	37
G1SQG1	H0YK61	EMC4	ER membrane protein complex subunit 4	ER membrane protein complex subunit 4	97	2	1.35	2	3	32
G1SHE2		GGCX	HTTM domain-containing protein			1	1.35	2	3	3
	P19623	SRM		Spermidine synthase		4	1.35	3	2	17
G1SKM2	P35555	FBN1	Uncharacterized protein	Fibrillin-1	97	3	1.35	6	6	2
G1T5J9	O75306	NDUFS2	Complex1_49kDa domain-containing protein	NADH dehydrogenase [ubiquinone] iron-sulfur protein 2, mitochondrial	94	2	1.35	6	10	23
	Q9Y4W6	AFG3L2		AFG3-like protein 2		4	1.35	11	21	19
G1SLC2	O00303	EIF3F	Eukaryotic translation initiation factor 3 subunit F	Eukaryotic translation initiation factor 3 subunit F	99	2	1.35	9	24	44
G1SMR7	P30050	RPL12	Uncharacterized protein	60S ribosomal protein L12	100	3	1.35	8	21	53
G1U0U5	E9PDE8	HSPA4L	Uncharacterized protein	Heat shock 70 kDa protein 4L	91	3	1.34	5	4	8
G1TEI0		PARP1	Poly [ADP-ribose] polymerase			1	1.34	3	3	5
G1SGJ5	Q92600	CNOT9	Uncharacterized protein	CCR4-NOT transcription complex subunit 9	97	3	1.34	2	3	7
G1T2I5	F8W914	RTN4	Reticulon	Reticulon	98	2	1.34	8	272	13
G1T3U3	A0A0G2JRV3	SMARCB1	SWI/SNF related, matrix associated, actin dependent regulator of chromatin, subfamily b, member 1	SWI/SNF-related matrix-associated actin-dependent regulator of chromatin subfamily B member 1	85	2	1.34	4	5	26
G1T657	A0A087WUK2	HNRNPDL	Heterogeneous nuclear ribonucleoprotein D like	Heterogeneous nuclear ribonucleoprotein D-like	92	2	1.34	6	14	18
G1U194	J3KPP4	LUC7L3	Uncharacterized protein	Cisplatin resistance-associated overexpressed protein, isoform CRA_b	98	3	1.34	3	4	8

G1SRN1		LAS1L	LAS1 like, ribosome biogenesis factor			1	1.34	4	3	11
	O95202	LETM1		Mitochondrial proton/calcium exchanger protein		4	1.34	7	11	10
	G3V0I5	NDUFV1		NADH dehydrogenase [ubiquinone] flavoprotein 1, mitochondrial		4	1.34	6	10	27
G1TT27	E9PKZ0	RPL8	Ribosomal_L2_C domain-containing protein	60S ribosomal protein L8 (Fragment)	100	2	1.34	6	11	35
G1TEG1	O95470	SGPL1	Uncharacterized protein	Sphingosine-1-phosphate lyase 1	90	3	1.34	9	16	22
G1SSC9	O95373	IPO7	Importin N-terminal domain-containing protein	Importin-7	100	2	1.34	18	39	29
	Q9H0B6	KLC2		Kinesin light chain 2		4	1.34	5	2	12
G1SQF9	Q8WXF1	PSPC1	Uncharacterized protein	Paraspeckle component 1	98	3	1.34	6	7	15
G1TE08	H7C3P6	NUP98	Peptidase S59 domain-containing protein	Nuclear pore complex protein Nup98-Nup96 (Fragment)	90	2	1.33	9	10	7
G1SFU0	P51149	RAB7A	Ras-related protein Rab-7a	Ras-related protein Rab-7a	100	2	1.33	12	24	61
	Q5T8U5	SURF4		Surfeit 4		4	1.33	4	83	29
G1SRS0	D6RBS5	ELMOD2	ELMO domain-containing protein	ELMO domain-containing protein 2 (Fragment)	93	2	1.33	2	2	6
G1SJQ6	O00519	FAAH	Amidase domain-containing protein	Fatty-acid amide hydrolase 1	91	2	1.33	4	6	11
G1SVD5	P55265	ADAR	Uncharacterized protein	Double-stranded RNA-specific adenosine deaminase	80	3	1.33	18	26	23
G1SXI9		COX6B1	Cytochrome c oxidase subunit			1	1.33	2	3	29
G1SXK8	F8WE74	SLC25A17	Uncharacterized protein	Peroxisomal membrane protein PMP34	96	3	1.33	4	6	21
U3KPB2	P61009	SPCS3	Signal peptidase complex subunit 3	Signal peptidase complex subunit 3	100	2	1.33	2	5	13
G1SFG7	P48681	NES	Nestin	Nestin	60	2	1.33	28	128	31
G1TSS7	P05106	ITGB3	Integrin beta	Integrin beta-3	95	2	1.33	5	5	9
G1SG07	Q8IWT6	LRRC8A	Leucine rich repeat containing 8 VRAC subunit A	Volume-regulated anion channel subunit LRRC8A	98	2	1.32	3	3	8
G1T511	A0A3F2YNY6	PRPF40A	Pre-mRNA processing factor 40 homolog A	Pre-mRNA-processing factor 40 homolog A	83	2	1.32	5	4	7
P42675	E9PCB6	NLN	Neurolysin, mitochondrial	Neurolysin, mitochondrial	94	2	1.32	6	10	15
G1T5A6	Q5T4D3	TMTC4	Transmembrane and tetratricopeptide repeat containing 4	Protein O-mannosyl-transferase TMTC4	92	2	1.32	5	3	11
G1SSF0	A3KFL2	EXOSC2	Uncharacterized protein	Exosome complex component RRP4	92	3	1.32	3	2	15
G1TD91	O43809	NUDT21	Nudix hydrolase domain-containing protein	Cleavage and polyadenylation specificity factor subunit 5	100	2	1.32	4	7	30
G1T7J9	B1AV70	YIPF6	Protein YIPF	Protein YIPF (Fragment)	75	2	1.32	4	9	12
G1TKL0	M0QZG7	SNRPA	Small nuclear ribonucleoprotein polypeptide A	U1 small nuclear ribonucleoprotein A (Fragment)	60	2	1.32	4	3	14
G1T0W7	M0R1B0	EMC8	ER membrane protein complex subunit 8	ER membrane protein complex subunit 8 (Fragment)	98	2	1.32	3	2	42
G1STX9		COQ8B	Coenzyme Q8B			1	1.32	3	4	15
G1U383	A0A087WYN9	DHX29	ATP-dependent RNA helicase DHX29	ATP-dependent RNA helicase DHX29	95	2	1.32	7	5	6
G1ST15	Q13825	AUH	AU RNA binding methylglutaconyl-CoA hydratase	Methylglutaconyl-CoA hydratase, mitochondrial	95	2	1.32	2	2	7
G1T159	P62070	RRAS2	Uncharacterized protein	Ras-related protein R-Ras2	97	3	1.32	6	6	33
	J3KN01	AFDN		Afadin		4	1.31	2	3	2
G1T1G2	A0A1B0GWA2	AGPS	Alkylglycerone-phosphate synthase	Alkylglycerone-phosphate synthase (Fragment)	96	2	1.31	7	13	17
G1SUC8	P60228	EIF3E	Eukaryotic translation initiation factor 3 subunit E	Eukaryotic translation initiation factor 3 subunit E	100	2	1.31	14	24	41
B7NZM8	Q04917	YWHAH	Tyrosine 3-monooxygenase/tryptophan 5-monooxygenase activation protein, eta polypeptide (Predicted)	14-3-3 protein eta	99	2	1.31	14	34	65
G1SIV7	O43776	NARS	AA_TRNA_LIGASE_II domain-containing protein	Asparagine--tRNA ligase, cytoplasmic	91	2	1.31	15	33	36
A0A140TAV6		HBB2	Globin A1			1	1.31	2	2	13
	P08574	CYC1		Cytochrome c1, heme protein, mitochondrial		4	1.31	4	5	16
G1T701		DBT	Dihydrolipoamide acetyltransferase component of pyruvate dehydrogenase complex			1	1.31	2	3	5
G1TMZ2		MRPS7	Ribosomal_S7 domain-containing protein			1	1.31	5	6	29
G1SY50	E7EPT4	NDUFV2	Uncharacterized protein	NADH dehydrogenase [ubiquinone] flavoprotein 2, mitochondrial	98	3	1.31	4	8	21
	A0A087WUC6	SPCS2		Signal peptidase complex subunit 2		4	1.31	6	15	33
	O75937	DNAJC8		DnaJ homolog subfamily C member 8		4	1.31	2	2	7
G1T5A2	P08648	ITGA5	Integrin_alpha2 domain-containing protein	Integrin alpha-5	91	2	1.31	10	13	13
	P49750-1	YLPM1		Isoform 1 of YLP motif-containing protein 1		4	1.31	4	5	4
G1T673	Q99653	CHP1	Calcineurin like EF-hand protein 1	Calcineurin B homologous protein 1	98	2	1.30	2	3	17
G1T958		EBP	EXPERA domain-containing protein			1	1.30	2	3	8
G1TBL0	E7ETZ4	BZW2	Basic leucine zipper and W2 domains 2	Basic leucine zipper and W2 domain-containing protein 2 (Fragment)	100	2	1.30	7	11	20
G1SLI0	Q96A33	CCDC47	Uncharacterized protein	Coiled-coil domain-containing protein 47	98	3	1.30	8	13	22
G1TCT3	F8VPD4	CAD	Carbamoyl-phosphate synthetase 2, aspartate transcarbamylase, and dihydroorotase	CAD protein	94	2	1.30	18	5	13
G1SSX5	P56192	MARS	Uncharacterized protein	Methionine--tRNA ligase, cytoplasmic	93	3	1.30	14	25	21
G1TNM3	P23396	RPS3	KH type-2 domain-containing protein	40S ribosomal protein S3	100	2	1.30	17	44	62
G1T5C5	E5RHW4	ERLIN2	PHB domain-containing protein	Erlin-2 (Fragment)	98	2	1.30	8	3	36
G1SH10	H3BVG0	NUP93	Nuclear pore complex protein Nup93	Nuclear pore complex protein Nup93	99	2	1.30	14	16	24
	Q96PU8	QKI		Protein quaking		4	1.30	4	4	16
	E9PB61	ALYREF		THO complex subunit 4		4	1.30	5	48	25

G1TCX6	P08240	SRPRA	SRP54 domain-containing protein	Signal recognition particle receptor subunit alpha	99	2	1.30	11	13	25
G1T5G8	Q9HC07	TMEM165	GDT1 family protein	Transmembrane protein 165	91	2	1.30	2	9	9
G1SDH8	Q5T9L3	WLS	Uncharacterized protein	Protein wntless homolog	98	3	1.30	5	4	12
G1SI29	P49411	TUFM	Elongation factor Tu	Elongation factor Tu, mitochondrial	93	2	1.30	17	103	41
G1T4D2		ACOX1	Acyl-coenzyme A oxidase			1	1.30	12	7	27
G1SU24	O60503	ADCY9	Adenylate cyclase 9	Adenylate cyclase type 9	92	2	1.30	2	2	4
	Q58FF6	HSP90AB4P		Putative heat shock protein HSP 90-beta 4		4	1.30	4	8	9
O79428		MT-ND2	NADH-ubiquinone oxidoreductase chain 2			1	1.30	2	2	10
G1SMZ5	Q14152	EIF3A	Eukaryotic translation initiation factor 3 subunit A	Eukaryotic translation initiation factor 3 subunit A	93	2	1.30	31	59	27
G1SZZ2	P49458	SRP9	Signal recognition particle 9 kDa protein	Signal recognition particle 9 kDa protein	92	2	1.30	3	4	30
G1T7T2	Q15424	SAFB	Scaffold attachment factor B	Scaffold attachment factor B1	86	2	1.29	6	7	14
G1TDX2	A0A3B3ISY9	AGK	Acylglycerol kinase	Acylglycerol kinase, mitochondrial	94	2	1.29	5	6	32
G1SYT7	G3V0E4	PMPCB	Uncharacterized protein	Mitochondrial-processing peptidase subunit beta	94	3	1.29	10	17	34
G1SJX5	A0A087WTF3	ANK3	Ankyrin 3	Ankyrin-3 (Fragment)	93	2	1.29	3	3	4
O46638	Q00688	FKBP3	Peptidyl-prolyl cis-trans isomerase FKBP3	Peptidyl-prolyl cis-trans isomerase FKBP3	96	2	1.29	4	7	21
P67828	P48729	CSNK1A1	Casein kinase I isoform alpha	Casein kinase I isoform alpha	100	2	1.29	4	6	13
G1SG63	P55011	SLC12A2	Uncharacterized protein	Solute carrier family 12 member 2	97	3	1.29	3	2	4
G1SED9	B0QY89	EIF3L	Eukaryotic translation initiation factor 3 subunit L	Eukaryotic translation initiation factor 3 subunit L	97	2	1.29	18	27	41
G1STJ3		PPIE	Peptidyl-prolyl cis-trans isomerase E			1	1.29	2	2	13
G1SPH7	Q9P2I0	CPSF2	Cleavage and polyadenylation specificity factor subunit 2	Cleavage and polyadenylation specificity factor subunit 2	99	2	1.29	4	6	6
G1STG2	Q9Y5M8	SRPRB	SRP receptor subunit beta	Signal recognition particle receptor subunit beta	94	2	1.29	11	19	49
G1STD4	Q9BSR8	YIPF4	Protein YIPF	Protein YIPF4	99	2	1.29	2	2	9
G1SSB5	C9JLU1	POLR2H	DNA-directed RNA polymerases I, II, and III subunit RPABC3	DNA-directed RNA polymerases I, II, and III subunit RPABC3 (Fragment)	100	2	1.29	2	2	29
G1T0M2		TRMT10C	SAM-dependent MTase TRM10-type domain-containing protein			1	1.28	4	4	15
G1SEC9	Q9HAV0	GNB4	WD_REPEATS_REGION domain-containing protein	Guanine nucleotide-binding protein subunit beta-4	99	2	1.28	8	6	28
G1SRV1	K7ELV2	SEH1L	Uncharacterized protein	Nucleoporin SEH1 (Fragment)	92	3	1.28	5	5	18
G1SDV5		NOL9	CLP1_P domain-containing protein			1	1.28	2	2	4
G1TXS5	Q9UGP8	SEC63	J domain-containing protein	Translocation protein SEC63 homolog	93	2	1.28	12	20	21
G1TEN9	Q8TB61	SLC35B2	Uncharacterized protein	Adenosine 3~-phospho 5~-phosphosulfate transporter 1	89	3	1.28	4	7	9
G1T3R1	Q86SX6	GLRX5	Glutaredoxin 5	Glutaredoxin-related protein 5, mitochondrial	84	2	1.28	3	4	25
G1TFM5	M0R0F0	RPS5	Ribosomal_S7 domain-containing protein	40S ribosomal protein S5 (Fragment)	100	2	1.28	8	60	56
G1TGI6	A0A0C4DG17	RPSA	40S ribosomal protein SA	40S ribosomal protein SA	98	2	1.28	10	31	46
	P0DN76	U2AF1L5		Splicing factor U2AF 35 kDa subunit-like protein		4	1.28	3	5	23
G1SGH2		MRPL15	Ribosomal_L18e/L15P domain-containing protein			1	1.28	2	2	9
G1T8P4	P05386	RPLP1	Uncharacterized protein	60S acidic ribosomal protein P1	97	3	1.28	4	7	72
G1TA37	O75531	BANF1	Uncharacterized protein	Barrier-to-autointegration factor	100	3	1.28	4	12	57
G1SEI8		NUP214	Nup214_FG domain-containing protein			1	1.28	4	5	4
G1TAA4	O75844	ZMPSTE24	CAAX prenyl protease	CAAX prenyl protease 1 homolog	96	2	1.28	3	5	9
G1T1T8	O96005	CLPTM1	CLPTM1, transmembrane protein	Cleft lip and palate transmembrane protein 1	97	2	1.28	11	24	21
G1T3L2	K7ERF1	EIF3K	Eukaryotic translation initiation factor 3 subunit K	Eukaryotic translation initiation factor 3 subunit K	87	2	1.28	7	13	44
G1SEW1	R4GMQ1	KDM1A	Lysine-specific histone demethylase	Lysine-specific histone demethylase	96	2	1.28	3	7	7
G1SHK6	Q5SSJ5	HP1BP3	Uncharacterized protein	Heterochromatin protein 1-binding protein 3	93	3	1.27	11	20	26
G1TTN7	O00139	KIF2A	Kinesin-like protein	Kinesin-like protein KIF2A	94	2	1.27	4	3	6
G1SQY8	Q9Y266	NUDC	CS domain-containing protein	Nuclear migration protein nudC	97	2	1.27	10	12	31
G1TDI0	Q9UQ80	PA2G4	Peptidase_M24 domain-containing protein	Proliferation-associated protein 2G4	98	2	1.27	12	68	43
G1T7I8		RIC8A	RIC8 guanine nucleotide exchange factor A			1	1.27	4	6	11
G1TQR2	P24534	EEF1B2	Uncharacterized protein	Elongation factor 1-beta	98	3	1.27	10	25	65
G1SUD2	Q6UW02	CYP20A1	Uncharacterized protein	Cytochrome P450 20A1	88	3	1.27	18	25	46
G1T5R3		TCF25	Transcription factor 25			1	1.27	3	2	10
G1T970	P30038	ALDH4A1	Multifunctional fusion protein	Delta-1-pyrroline-5-carboxylate dehydrogenase, mitochondrial	91	2	1.27	11	20	31
G1SD54	Q99720	SIGMAR1	Uncharacterized protein	Sigma non-opioid intracellular receptor 1	97	3	1.27	4	5	23
G1SSL5	Q14CX7	NAA25	TPR_REGION domain-containing protein	N-alpha-acetyltransferase 25, NatB auxiliary subunit	96	2	1.27	5	6	11
G1SUU2	A0A087WXS7	ASNA1	ATPase ASNA1	ATPase ASNA1	93	2	1.27	9	11	44
G1SKW4		KIDINS220	Kinase D interacting substrate 220			1	1.27	8	6	7
G1SN16	P27695	APEX1	DNA-(apurinic or apyrimidinic site) lyase	DNA-(apurinic or apyrimidinic site) lyase	95	2	1.26	5	10	28
O77622		CCT6	T-complex protein 1 subunit zeta			1	1.26	23	15	54
G1SLF5	H0YG54	REXO2	Exonuclease domain-containing protein	Oligoribonuclease, mitochondrial	98	2	1.26	3	4	22

G1T1U7	A0A494C1T2	MTHFD1	Uncharacterized protein	C-1-tetrahydrofolate synthase, cytoplasmic (Fragment)	90	3	1.26	14	18	20
G1TVT0	P19338	NCL	Nucleolin	Nucleolin	83	2	1.26	23	23	34
	A0A0U1RRM4	PTBP1		Polypyrimidine tract-binding protein 1		4	1.26	10	53	29
G1TIM0	G3V155	TMX2	Thioredoxin domain-containing protein	Thioredoxin domain containing 14, isoform CRA_a	94	2	1.26	4	4	13
G1T3S2	P54753	EPHB3	Uncharacterized protein	Ephrin type-B receptor 3	98	3	1.26	9	15	15
G1T2V6	Q99747	NAPG	Uncharacterized protein	Gamma-soluble NSF attachment protein	98	3	1.26	3	3	8
	A0A0A0MTU3	TMEM259		Membralin		4	1.26	2	2	8
G1SSL0	P30040	ERP29	Endoplasmic reticulum resident protein 29	Endoplasmic reticulum resident protein 29	93	2	1.26	9	35	46
G1TY34		ITGA4	Integrin_alpha2 domain-containing protein			1	1.26	4	5	7
G1TEE3	G3V5Z3	PPP4R3A	SMK-1 domain-containing protein	Serine/threonine-protein phosphatase 4 regulatory subunit 3A	100	2	1.26	4	8	9
P29694	P26641	EEF1G	Elongation factor 1-gamma	Elongation factor 1-gamma	98	2	1.26	21	66	64
G1T419	Q92621	NUP205	Uncharacterized protein	Nuclear pore complex protein Nup205	96	3	1.25	24	15	19
G1SYC1	P83111	LACTB	Uncharacterized protein	Serine beta-lactamase-like protein LACTB, mitochondrial	89	3	1.25	4	7	12
G1SM24		MDN1	Midasin			1	1.25	2	2	1
G1TAM8	P61289	PSME3	Uncharacterized protein	Proteasome activator complex subunit 3	95	3	1.25	8	11	37
G1SKF7	Q02878	RPL6	60S ribosomal protein L6	60S ribosomal protein L6	89	2	1.25	11	40	40
G1SZI5	P62269	RPS18	Uncharacterized protein	40S ribosomal protein S18	100	3	1.25	9	19	44
G1T2G5	Q5NDL2	EOGT	Uncharacterized protein	EGF domain-specific O-linked N-acetylglucosamine transferase	91	3	1.25	8	10	18
G1TCB7	Q5LJA9	UCHL5	Ubiquitin carboxyl-terminal hydrolase	Ubiquitin carboxyl-terminal hydrolase (Fragment)	91	2	1.25	7	6	17
G1SNM8	Q8N5M9	JAGN1	Uncharacterized protein	Protein jagunal homolog 1	96	3	1.25	3	4	17
	P62701	RPS4X		40S ribosomal protein S4, X isoform		4	1.25	15	37	52
G1SIB2	A0A087WVM4	MTHFD1L	Uncharacterized protein	Monofunctional C1-tetrahydrofolate synthase, mitochondrial	92	3	1.25	9	21	13
G1SEW3	P49792	RANBP2	RAN binding protein 2	E3 SUMO-protein ligase RanBP2	84	2	1.25	17	29	8
G1SQH0	P61254	RPL26	KOW domain-containing protein	60S ribosomal protein L26	100	2	1.25	6	11	28
	B4DQT1	MAEA		Macrophage erythroblast attacher		4	1.25	3	3	8
G1TAB7	A0A2R8Y3X5	OPA1	Dynamin-type G domain-containing protein	Dynamin-like 120 kDa protein, mitochondrial	94	2	1.25	26	47	32
G1SG61		TPST2	Protein-tyrosine sulfotransferase			1	1.25	2	2	5
G1TVY5	C9JZR2	CTNND1	Uncharacterized protein	Catenin delta-1	97	3	1.25	19	42	35
G1SU75	O95140	MFN2	Mitofusin 2	Mitofusin-2	92	2	1.25	10	12	18
G1SVH1	A0A0U1RRK1	MICU1	Uncharacterized protein	Calcium uptake protein 1, mitochondrial	94	3	1.24	5	17	18
G1STL1	Q8WUA2	PPIL4	Peptidyl-prolyl cis-trans isomerase	Peptidyl-prolyl cis-trans isomerase-like 4	97	2	1.24	2	3	6
G1SCY7	Q8N766	EMC1	EMC1_C domain-containing protein	ER membrane protein complex subunit 1	96	2	1.24	15	24	23
G1TQD4		RALY	RRM domain-containing protein			1	1.24	2	2	6
	Q9NZN4	EHD2		EH domain-containing protein 2		4	1.24	11	16	27
G1SPK4	P05388	RPLP0	60S acidic ribosomal protein P0	60S acidic ribosomal protein P0	98	2	1.24	13	8	68
G1TI71	A0A087WYS1	UGP2	UTP--glucose-1-phosphate uridylyltransferase	UTP--glucose-1-phosphate uridylyltransferase	99	2	1.24	24	61	62
B7NZM4	Q9Y295	DRG1	Developmentally regulated GTP binding protein 1 (Predicted)	Developmentally-regulated GTP-binding protein 1	100	2	1.24	9	19	35
G1SK29		MTIF2	Tr-type G domain-containing protein			1	1.24	3	4	8
G1SFR8	P25398	RPS12	40S ribosomal protein S12	40S ribosomal protein S12	100	2	1.24	4	12	32
G1T920	P49903	SEPHS1	Uncharacterized protein	Selenide, water dikinase 1	100	3	1.24	5	5	20
	P18583-2	SON		Isoform A of Protein SON		4	1.24	2	2	1
G1SRE8		XRCC5	Ku domain-containing protein			1	1.24	7	14	20
G1SVQ8	F8WF48	SEC62	Uncharacterized protein	Translocation protein SEC62	100	3	1.24	2	3	5
G1STR6	Q96FN9	DTD2	Uncharacterized protein	D-aminoacyl-tRNA deacylase 2	93	3	1.24	2	3	19
G1TCY7		EIF2B4	Translation initiation factor eIF-2B subunit delta			1	1.24	3	3	8
	H3BQQ9	UBE2I		SUMO-conjugating enzyme UBC9 (Fragment)		4	1.23	3	3	40
G1SJC7	Q9UN86	G3BP2	Uncharacterized protein	Ras GTPase-activating protein-binding protein 2	100	3	1.23	5	6	10
G1T4K8	P32189	GK	Uncharacterized protein	Glycerol kinase	97	3	1.23	11	19	23
G1SW36	H0YDT8	EMC7	DUF2012 domain-containing protein	ER membrane protein complex subunit 7 (Fragment)	79	2	1.23	4	4	24
G1U797	P51114	FXR1	Uncharacterized protein	Fragile X mental retardation syndrome-related protein 1	91	3	1.23	12	10	19
G1TIP5	C9JCC6	DRAP1	CBFD_NFYB_HMF domain-containing protein	Dr1-associated corepressor	87	2	1.23	2	3	11
P62497	P62495	ETF1	Eukaryotic peptide chain release factor subunit 1	Eukaryotic peptide chain release factor subunit 1	100	2	1.23	15	32	52
G1SPJ5	Q92616	GCN1	TOG domain-containing protein	eIF-2-alpha kinase activator GCN1	96	2	1.23	46	82	27
G1SGG6	P48449	LSS	Terpene cyclase/mutase family member	Lanosterol synthase	89	2	1.23	12	17	20
P19943	P05387	RPLP2	60S acidic ribosomal protein P2 (Fragment)	60S acidic ribosomal protein P2	100	2	1.23	8	20	64
G1U3Q0	Q93050	ATP6V0A1	V-type proton ATPase subunit a	V-type proton ATPase 116 kDa subunit a isoform 1	96	2	1.23	12	3	20
G1SKZ3	P12004	PCNA	Proliferating cell nuclear antigen	Proliferating cell nuclear antigen	99	2	1.23	2	3	15

G1TDK8	F8VXC8	SMARCC2	Uncharacterized protein	SWI/SNF complex subunit SMARCC2	93	3	1.23	8	5	10
G1SLI8	A0A0A0MSJ0	DDX42	Uncharacterized protein	ATP-dependent RNA helicase DDX42	96	3	1.23	3	6	7
G1TA69	Q8TBA6	GOLGA5	Uncharacterized protein	Golgin subfamily A member 5	83	3	1.23	4	3	6
G1T2G3	Q5VTR2	RNF20	E3 ubiquitin protein ligase	E3 ubiquitin-protein ligase BRE1A	98	2	1.23	6	4	10
G1U724	Q9UBE0	SAE1	SUMO1 activating enzyme subunit 1	SUMO-activating enzyme subunit 1	88	2	1.23	8	12	26
G1U2J5	Q8NE01	CNNM3	Uncharacterized protein	Metal transporter CNNM3	88	3	1.23	2	2	7
G1T2V2	D6RF62	PAICS	AIRC domain-containing protein	Multifunctional protein ADE2	93	2	1.23	8	7	32
G1U1H3	P61018	RAB4B	Uncharacterized protein	Ras-related protein Rab-4B	100	3	1.23	4	6	31
G1TSP3	Q9NZ01	TECR	Uncharacterized protein	Very-long-chain enoyl-CoA reductase	98	3	1.23	5	12	15
G1TST9	A0A2R8Y212	CHD4	Uncharacterized protein	Chromodomain-helicase-DNA-binding protein 4	97	3	1.23	20	16	18
G1SCF6		YARS2	Tyrosine--tRNA ligase			1	1.23	5	4	20
G1T108	Q9NRG9	AAAS	WD_REPEATS_REGION domain-containing protein	Aladin	94	2	1.22	6	7	17
G1T9E3	E9PLP8	CSTF3	Suf domain-containing protein	Cleavage stimulation factor subunit 3	100	2	1.22	3	3	7
G1SQV5	O15371	EIF3D	Eukaryotic translation initiation factor 3 subunit D	Eukaryotic translation initiation factor 3 subunit D	99	2	1.22	13	21	39
	A0A0A6YYJ8	LUC7L2		Putative RNA-binding protein Luc7-like 2		4	1.22	4	7	11
G1U0A4	P21810	BGN	Biglycan	Biglycan	94	2	1.22	12	15	46
G1SF82	P62072	TIMM10	zf-Tim10_DDP domain-containing protein	Mitochondrial import inner membrane translocase subunit Tim10	100	2	1.22	2	4	36
G1U9T1	Q99832	CCT7	T-complex protein 1 subunit eta	T-complex protein 1 subunit eta	97	2	1.22	26	9	60
G1T619		MCUB	MCU domain-containing protein			1	1.22	3	3	14
G1SVP9	G3V4T2	PABPN1	RRM domain-containing protein	Polyadenylate-binding protein 2	99	2	1.22	2	2	6
G1SIA3	Q8N3C0	ASCC3	Uncharacterized protein	Activating signal cointegrator 1 complex subunit 3	94	3	1.22	7	7	5
G1T9T5	P36957	DLST	Lipoyl-binding domain-containing protein	Dihydrolipoyllysine-residue succinyltransferase component of 2-oxoglutarate dehydrogenase complex, mitochondrial	91	2	1.22	12	28	35
G1U971		EIF3C	Eukaryotic translation initiation factor 3 subunit C			1	1.22	16	26	24
G1TPZ3	Q96GK7	FAHD2A	FAA_hydrolase domain-containing protein	Fumarylacetoacetate hydrolase domain-containing protein 2A	90	2	1.22	3	3	12
B7NZG9	Q8N3U4	STAG2	Stromal antigen 2 isoform a (Predicted)	Cohesin subunit SA-2	97	2	1.22	5	7	8
G1T6E9	Q8N5K1	CISD2	ZnF_CDGSH domain-containing protein	CDGSH iron-sulfur domain-containing protein 2	99	2	1.22	4	8	32
G1SLW8	Q7L2H7	EIF3M	Eukaryotic translation initiation factor 3 subunit M	Eukaryotic translation initiation factor 3 subunit M	100	2	1.22	12	28	48
G1TRH3	Q9UBR2	CTSZ	Pept_C1 domain-containing protein	Cathepsin Z	86	2	1.22	7	36	30
G1SXF1		NFU1	Nfu_N domain-containing protein			1	1.22	4	10	22
G1T5V3	P61106	RAB14	Uncharacterized protein	Ras-related protein Rab-14	100	3	1.22	12	28	73
G1T8E0	A3KMH1	VWA8	VWFA domain-containing protein	von Willebrand factor A domain-containing protein 8	92	2	1.22	7	10	5
	P13639	EEF2		Elongation factor 2		4	1.22	37	271	58
G1SZ03	P55884	EIF3B	Eukaryotic translation initiation factor 3 subunit B	Eukaryotic translation initiation factor 3 subunit B	98	2	1.21	24	8	41
G1SFK3		RPA1	Replication protein A subunit			1	1.21	2	2	7
G1SES2		NAA10	N-acetyltransferase domain-containing protein			1	1.21	2	2	9
G1SG80	F8VZG5	AK2	Adenylate kinase 2, mitochondrial	Adenylate kinase 2, mitochondrial	92	2	1.21	9	25	47
G1ST95	B3KS98	EIF3H	Eukaryotic translation initiation factor 3 subunit H	Eukaryotic translation initiation factor 3 subunit H	98	2	1.21	8	14	37
U3KNL7	Q06210	GFPT1	Uncharacterized protein	Glutamine--fructose-6-phosphate aminotransferase [isomerizing] 1	95	3	1.21	15	4	40
G1TBX7	P11233	RALA	Uncharacterized protein	Ras-related protein Ral-A	100	3	1.21	8	6	50
G1SRA8	P41091	EIF2S3	Eukaryotic translation initiation factor 2 subunit 3	Eukaryotic translation initiation factor 2 subunit 3	92	2	1.21	12	23	36
G1SGM2	O14744	PRMT5	Protein arginine N-methyltransferase 5	Protein arginine N-methyltransferase 5	98	2	1.21	6	10	19
G1TWC3		TMX1	Thioredoxin domain-containing protein			1	1.21	5	6	19
G1SLM1	Q9HB71	CACYBP	Uncharacterized protein	Calcyclin-binding protein	92	3	1.21	5	3	22
G1SFC1	H0Y6I0	GOLGA4	Golgin A4	Golgin subfamily A member 4 (Fragment)	77	2	1.21	12	16	7
G1SSK8	B4DJV2	CS	Citrate synthase	Citrate synthase	96	2	1.21	18	89	53
G1T2G4	P05198	EIF2S1	Eukaryotic translation initiation factor 2 subunit 1	Eukaryotic translation initiation factor 2 subunit 1	99	2	1.21	14	30	53
G1TWU8	O43264	ZW10	Uncharacterized protein	Centromere/kinetochore protein zw10 homolog	89	3	1.21	10	12	20
G1ST38	Q9Y673	ALG5	ALG5, dolichyl-phosphate beta-glucosyltransferase	Dolichyl-phosphate beta-glucosyltransferase	93	2	1.21	6	10	21
G1U9S7	P17987	TCP1	Uncharacterized protein	T-complex protein 1 subunit alpha	97	3	1.21	26	385	62
G1TWP4	A0A140T936	VARS	GST C-terminal domain-containing protein	Valine--tRNA ligase (Fragment)	92	2	1.21	18	30	18
G1SUX1		TIMP3	Metalloproteinase inhibitor 3			1	1.21	2	2	13
G1SMY6	O14980	XPO1	Importin N-terminal domain-containing protein	Exportin-1	99	2	1.21	16	19	25
G1TQ31	A0A2R8YD58	CSNK2A1	Casein kinase II subunit alpha	Casein kinase II subunit alpha	97	2	1.20	4	5	21
G1TG15		FAM210A	DUF1279 domain-containing protein			1	1.20	2	3	13
G1T2N8	O43592	XPOT	Exportin-T	Exportin-T	99	2	1.20	4	5	6
Q9TT15	P21796	VDAC1	Voltage-dependent anion-selective channel protein 1	Voltage-dependent anion-selective channel protein 1	100	2	1.20	18	410	82
G1SGR9	A0A494C1K3	GTF2I	General transcription factor IIi	General transcription factor II-I	97	2	1.20	4	6	9

G1SNE9	A0A0G2JQ41	ABR	Uncharacterized protein	Active breakpoint cluster region-related protein (Fragment)	100	3	1.20	7	4	18
G1SVB6	P00367	GLUD1	Glutamate dehydrogenase	Glutamate dehydrogenase 1, mitochondrial	98	2	1.20	23	174	64
G1TMD8		PPP5C	Serine/threonine-protein phosphatase			1	1.20	3	2	10
G1STH4	Q14344	GNA13	Uncharacterized protein	Guanine nucleotide-binding protein subunit alpha-13	86	3	1.20	3	5	11
G1TAW7		EIF2A	Eukaryotic translation initiation factor 2A			1	1.20	3	4	9
G1SYE7	H0Y8R1	GRSF1	Uncharacterized protein	G-rich sequence factor 1 (Fragment)	96	3	1.20	3	4	14
G1T855	C9J5X1	IGF1R	Tyrosine-protein kinase receptor	Tyrosine-protein kinase receptor	93	2	1.20	8	11	10
G1T3M3	Q9UL25	RAB21	Uncharacterized protein	Ras-related protein Rab-21	97	3	1.20	5	12	30
G1SVY6	Q9BTX1	NDC1	Uncharacterized protein	Nucleoporin NDC1	88	3	1.20	2	2	5
G1SPR9	P04844	RPN2	Dolichyl-diphosphooligosaccharide--protein glycosyltransferase subunit 2	Dolichyl-diphosphooligosaccharide--protein glycosyltransferase subunit 2	93	2	1.20	25	86	68
G1TKQ8	A0A0D9SG77	UBE3A	Ubiquitin-protein ligase E3A	Ubiquitin-protein ligase E3A	97	2	1.20	6	5	11
G1SML5	Q9H9B4	SFXN1	Sidoreflexin	Sideroflexin-1	94	2	1.19	11	26	53
G1SYI2	P62873	GNB1	WD_REPEATS_REGION domain-containing protein	Guanine nucleotide-binding protein G(I)/G(S)/G(T) subunit beta-1	100	2	1.19	12	47	45
G1SJU1	O75051	PLXNA2	Sema domain-containing protein	Plexin-A2	98	2	1.19	3	3	2
G1TB17	A0A1B0GWF8	ADSL	Adenylosuccinate lyase	Adenylosuccinate lyase (Fragment)	95	2	1.19	4	2	13
G1T4Z1	Q07954	LRP1	Uncharacterized protein	Prolow-density lipoprotein receptor-related protein 1	98	3	1.19	81	8	26
G1T1Y3	A0A384DVU0	PNPLA6	Patatin like phospholipase domain containing 6	Neuropathy target esterase	98	2	1.19	3	2	5
G1SYL3	E9PFH4	TNPO3	Xpo1 domain-containing protein	Transportin-3	96	2	1.19	6	8	12
G1T103	F8VQQ3	C12orf10	Uncharacterized protein	UPF0160 protein MYG1, mitochondrial	77	3	1.19	4	7	16
G1TWS0	B3KY94	CDIPT	CDP-diacylglycerol--inositol 3-phosphatidyltransferase	CDP-diacylglycerol--inositol 3-phosphatidyltransferase	75	2	1.19	4	6	22
G1SSR8	O76031	CLPX	Uncharacterized protein	ATP-dependent Clp protease ATP-binding subunit clpX-like, mitochondrial	97	3	1.19	5	4	13
G1SML9	P31689	DNAJA1	Uncharacterized protein	DnaJ homolog subfamily A member 1	100	3	1.19	8	20	34
G1SK61	Q9UIW2	PLXNA1	Plexin A1	Plexin-A1	91	2	1.19	3	3	4
G1TE34	P06756	ITGAV	Integrin_alpha2 domain-containing protein	Integrin alpha-V	95	2	1.19	26	87	34
G1T720	F5GXX5	DAD1	Dolichyl-diphosphooligosaccharide--protein glycosyltransferase subunit DAD1	Dolichyl-diphosphooligosaccharide--protein glycosyltransferase subunit DAD1	75	2	1.19	3	5	38
G1T9M7	E9PK47	PYGL	Alpha-1,4 glucan phosphorylase	Alpha-1,4 glucan phosphorylase	94	2	1.19	15	12	25
G1U9U0	P50991	CCT4	T-complex protein 1 subunit delta	T-complex protein 1 subunit delta	99	2	1.19	23	54	56
G1T7V5	E9PEX6	DLD	Dihydrolipoyl dehydrogenase	Dihydrolipoyl dehydrogenase	91	2	1.19	11	3	32
G1TIS2		TIMM17B	Mitochondrial import inner membrane translocase subunit TIM17			1	1.19	2	3	22
G1SNZ3	P55060	CSE1L	Chromosome segregation 1 like	Exportin-2	99	2	1.19	13	31	26
P47823		EIF2B5	Translation initiation factor eIF-2B subunit epsilon			1	1.19	5	9	14
G1SKF5		ERBIN	Erbb2 interacting protein			1	1.19	5	3	9
G1SL42	Q9BPW8	NIPSNAP1	NIPSNAP domain-containing protein	Protein NipSnap homolog 1	95	2	1.19	5	3	26
G1TTK6	E7ETT1	PCCB	Uncharacterized protein	Propionyl-CoA carboxylase beta chain, mitochondrial	86	3	1.18	9	14	28
G1TB39	P29353	SHC1	Uncharacterized protein	SHC-transforming protein 1	97	3	1.18	2	2	6
G1TCH9	A0A2R8Y473	ABCB7	Uncharacterized protein	ATP-binding cassette sub-family B member 7, mitochondrial	95	3	1.18	7	11	13
P67873	Q5SRQ6	CSNK2B	Casein kinase II subunit beta	Casein kinase II subunit beta	100	2	1.18	7	10	51
G1TUX2	A2A274	ACO2	Aconitate hydratase, mitochondrial	Aconitate hydratase, mitochondrial	94	2	1.18	25	53	42
G1SLQ3	P15559	NQO1	Flavodoxin_2 domain-containing protein	NAD(P)H dehydrogenase [quinone] 1	89	2	1.18	10	17	48
G1SEE0	Q09028	RBBP4	RB binding protein 4, chromatin remodeling factor	Histone-binding protein RBBP4	100	2	1.18	6	2	23
G1T9J3	A2RRP1	NBAS	Uncharacterized protein	Neuroblastoma-amplified sequence	90	3	1.18	19	26	12
G1TJV3	Q9H7Z7	PTGES2	Prostaglandin E synthase 2	Prostaglandin E synthase 2	79	2	1.18	4	3	19
G1SM82	E9PGC0	RASA1	Uncharacterized protein	Ras GTPase-activating protein 1	96	3	1.18	8	10	13
G1T0F6	Q9UHB9	SRP68	Signal recognition particle subunit SRP68	Signal recognition particle subunit SRP68	95	2	1.18	9	15	18
G1SKK1		DUT	dUTPase domain-containing protein			1	1.18	2	3	14
G1TBH1	A0A1W2PPT5	POLR2B	DNA-directed RNA polymerase subunit beta	DNA-directed RNA polymerase subunit beta	100	2	1.18	6	7	7
G1SRB1	Q16134	ETFDH	4Fe-4S ferredoxin-type domain-containing protein	Electron transfer flavoprotein-ubiquinone oxidoreductase, mitochondrial	94	2	1.18	9	9	22
G1SVM1	Q14764	MVP	Uncharacterized protein	Major vault protein	91	3	1.18	35	102	57
G1TUU9		TRIP12	Thyroid hormone receptor interactor 12			1	1.18	8	8	6
G1T568	Q92973	TNPO1	Transportin 1	Transportin-1	93	2	1.18	14	12	21
G1SFH6	Q9UBT2	UBA2	Uncharacterized protein	SUMO-activating enzyme subunit 2	97	3	1.18	10	9	27
G1T9V1	P11177	PDHB	Pyruvate dehydrogenase E1 component subunit beta	Pyruvate dehydrogenase E1 component subunit beta, mitochondrial	97	2	1.18	10	31	39
	E9PAV3-2	NACA		Isoform skNAC-2 of Nascent polypeptide-associated complex subunit alpha, muscle-specific form		4	1.18	6	35	11
G1SPG2		TRIP11	GRIP domain-containing protein			1	1.18	16	15	11
G1TRL5	A0A087WUT6	EIF5B	Tr-type G domain-containing protein	Eukaryotic translation initiation factor 5B	95	2	1.17	21	44	22
G1T8H5		POLR2C	RPOLD domain-containing protein			1	1.17	2	3	11
G1SJB4	P63244	RACK1	WD_REPEATS_REGION domain-containing protein	Receptor of activated protein C kinase 1	100	2	1.17	16	45	73

G1SHU8	D3YTB1	RPL32	Uncharacterized protein	60S ribosomal protein L32 (Fragment)	88	3	1.17	4	10	29
G1T0Q6	Q9UQE7	SMC3	Structural maintenance of chromosomes protein	Structural maintenance of chromosomes protein 3	100	2	1.17	8	5	9
G1SRZ8	A5YKK6	CNOT1	Uncharacterized protein	CCR4-NOT transcription complex subunit 1	100	3	1.17	8	8	5
G1STF8		TBRG4	RAP domain-containing protein			1	1.17	3	3	7
G1THT8	J3QT54	CPSF7	Cleavage and polyadenylation specific factor 7	Cleavage and polyadenylation-specificity factor subunit 7 (Fragment)	95	2	1.17	4	3	16
G1TZI2		PGAM5	PGAM family member 5, mitochondrial serine/threonine protein phosphatase			1	1.17	2	2	9
	G8JLG1	SMC1A		Structural maintenance of chromosomes protein		4	1.17	5	5	6
	A0A087WX29	TARDBP		TAR DNA-binding protein 43 (Fragment)		4	1.17	7	2	47
G1SZM2	A0A0A0MRG2	APP	Amyloid-beta A4 protein	Amyloid-beta precursor protein	99	2	1.17	7	8	11
G1SU30	A0A024RCR6	BAG6	Ubiquitin-like domain-containing protein	BAG6	91	2	1.17	8	7	12
G1SCN8	P49368	CCT3	T-complex protein 1 subunit gamma	T-complex protein 1 subunit gamma	98	2	1.17	27	50	64
G1SJ66	C9JME2	FARP1	Uncharacterized protein	FERM, ARHGEF and pleckstrin domain-containing protein 1	90	3	1.17	28	57	38
G1U7L4	P11021	HSPA5	Heat shock protein family A (Hsp70) member 5	Endoplasmic reticulum chaperone BiP	99	2	1.17	34	780	62
G1SYD3	P54577	YARS	Tyrosine--tRNA ligase	Tyrosine--tRNA ligase, cytoplasmic	96	2	1.17	20	36	44
G1T888	Q9P2R3	ANKFY1	Ankyrin repeat and FYVE domain containing 1	Rabankyrin-5	96	2	1.17	5	2	7
G1T4A5	P02452	COL1A1	Collagen alpha-1(I) chain	Collagen alpha-1(I) chain	91	2	1.17	34	25	45
G1T550	P51148	RAB5C	Uncharacterized protein	Ras-related protein Rab-5C	91	3	1.17	8	24	53
G1T2J0	P14923	JUP	Uncharacterized protein	Junction plakoglobin	99	3	1.17	5	10	10
G1T7W7	P46977	STT3A	Uncharacterized protein	Dolichyl-diphosphooligosaccharide--protein glycosyltransferase subunit STT3A	100	3	1.17	15	43	26
G1SQ57	Q8N6T3	ARFGAP1	Arf-GAP domain-containing protein	ADP-ribosylation factor GTPase-activating protein 1	78	2	1.17	5	4	21
G1STW7	Q9NSD9	FARSB	B5 domain-containing protein	Phenylalanine--tRNA ligase beta subunit	95	2	1.17	11	15	22
G1T5M0	P04181	OAT	Uncharacterized protein	Ornithine aminotransferase, mitochondrial	91	3	1.17	16	53	54
G1SH66		RBM3	RRM domain-containing protein			1	1.17	3	4	37
G1TDA9	O94766	B3GAT3	Galactosylgalactosylxylosylprotein 3-beta-glucuronosyltransferase	Galactosylgalactosylxylosylprotein 3-beta-glucuronosyltransferase 3	96	2	1.17	4	4	12
	Q03252	LMNB2		Lamin-B2		4	1.17	8	7	11
	E7EVJ3	NDST1		Bifunctional heparan sulfate N-deacetylase/N-sulfotransferase 1		4	1.17	2	2	3
G1T813	P08559	PDHA1	Pyruvate dehydrogenase E1 component subunit alpha	Pyruvate dehydrogenase E1 component subunit alpha, somatic form, mitochondrial	99	2	1.17	12	19	35
G1U3S3		KIAA2013	KIAA2013			1	1.17	4	4	11
G1STE3	A0A0A0MQX8	MBNL1	Uncharacterized protein	Muscleblind-like protein 1	100	3	1.17	3	4	7
G1T9L6		TOR1AIP1	Torsin 1A interacting protein 1			1	1.17	4	5	9
G1SMK9	Q9ULC3	RAB23	Uncharacterized protein	Ras-related protein Rab-23	97	3	1.16	4	7	22
G1SW89	Q9C0E8	LNPK	zinc_ribbon_10 domain-containing protein	Endoplasmic reticulum junction formation protein lunapark	89	2	1.16	2	2	5
G1SGM3	A0A0J9YXF2	PON2	Uncharacterized protein	Paraoxonase 2, isoform CRA_a	93	3	1.16	12	26	58
G1TCT4	O95155	UBE4B	U-box domain-containing protein	Ubiquitin conjugation factor E4 B	95	2	1.16	2	2	3
G1TTA5	H0Y8C3	MTCH1	Uncharacterized protein	Mitochondrial carrier homolog 1 (Fragment)	93	3	1.16	7	21	23
Q9N0Z6	P05023	ATP1A1	Sodium/potassium-transporting ATPase subunit alpha-1	Sodium/potassium-transporting ATPase subunit alpha-1	98	2	1.16	28	68	36
G1SME1		NOP14	NOP14 nucleolar protein			1	1.16	3	5	6
	Q9UL15	BAG5		BAG family molecular chaperone regulator 5		4	1.16	2	2	7
G1SZW5	Q8TD16	BICD2	Uncharacterized protein	Protein bicaudal D homolog 2	95	3	1.16	4	5	7
G1SFV7	Q16531	DDB1	Damage specific DNA binding protein 1	DNA damage-binding protein 1	100	2	1.16	18	27	19
G1SRY7	K7ESP4	DCAKD	Uncharacterized protein	Dephospho-CoA kinase domain-containing protein (Fragment)	92	3	1.16	4	7	29
G1SEV7	Q9NX55	HYPK	Uncharacterized protein	Huntingtin-interacting protein K	98	3	1.16	4	6	42
G1T573	H0Y3P2	EIF4G2	Eukaryotic translation initiation factor 4 gamma 2	Eukaryotic translation initiation factor 4 gamma 2	95	2	1.16	18	32	26
G1U115	Q15008	PSMD6	PCI domain-containing protein	26S proteasome non-ATPase regulatory subunit 6	99	2	1.16	16	38	47
G1TWK1	E9PGZ4	SACM1L	SAC domain-containing protein	Phosphatidylinositide phosphatase SAC1	98	2	1.16	14	25	34
G1U7M0	Q96AJ9	VTI1A	t-SNARE coiled-coil homology domain-containing protein	Vesicle transport through interaction with t-SNAREs homolog 1A	94	2	1.16	3	3	17
G1SI95	O00291	HIP1	Uncharacterized protein	Huntingtin-interacting protein 1	91	3	1.16	15	10	24
G1T840	Q92598	HSPH1	Uncharacterized protein	Heat shock protein 105 kDa	96	3	1.16	17	22	28
G1TEI1	P20645	M6PR	Uncharacterized protein	Cation-dependent mannose-6-phosphate receptor	95	3	1.16	2	3	8
G1SV05	P34932	HSPA4	Uncharacterized protein	Heat shock 70 kDa protein 4	97	3	1.15	25	21	45
G1TRI7	A0A0C4DG51	PNPLA8	Calcium-independent phospholipase A2-gamma	Calcium-independent phospholipase A2-gamma (Fragment)	90	2	1.15	2	2	4
G1SH80	O14874	BCKDK	Protein-serine/threonine kinase	[3-methyl-2-oxobutanoate dehydrogenase [lipoamide]] kinase, mitochondrial	97	2	1.15	2	9	6
G1SS85	Q9UBQ7	GRHPR	Uncharacterized protein	Glyoxylate reductase/hydroxypyruvate reductase	90	3	1.15	5	7	28
G1SH42	Q5VSL9	STRIP1	Uncharacterized protein	Striatin-interacting protein 1	98	3	1.15	4	3	12
G1TCE9		HSDL2	SCP2 domain-containing protein			1	1.15	8	15	28
G1TCK9	A0A0A0MSX9	IARS	Uncharacterized protein	Isoleucine--tRNA ligase, cytoplasmic	94	3	1.15	33	62	34
G1SQ93	Q9UKV8	AGO2	Protein argonaute-2	Protein argonaute-2	98	2	1.15	3	3	6

G1T9S4	H0YDD4	DLAT	Acetyltransferase component of pyruvate dehydrogenase complex	Acetyltransferase component of pyruvate dehydrogenase complex (Fragment)	90	2	1.15	11	21	25
G1U998		NOL6	Nucleolar protein 6			1	1.15	3	9	7
G1SEQ2		PBDC1	Polysacc_synt_4 domain-containing protein			1	1.15	3	3	16
G1T157	Q9Y376	CAB39	Uncharacterized protein	Calcium-binding protein 39	99	3	1.15	3	2	8
G1T336	A0A0D9SF53	DDX3X	Uncharacterized protein	ATP-dependent RNA helicase DDX3X	99	3	1.15	19	42	31
G1SEC8	Q8IWA4	MFN1	Dynamin-type G domain-containing protein	Mitofusin-1	92	2	1.15	8	14	17
G1T0E5		SNAP23	Synaptosomal-associated protein			1	1.15	3	4	23
G1T2T9	P20339	RAB5A	Uncharacterized protein	Ras-related protein Rab-5A	99	3	1.15	8	8	54
G1SV12	P16219	ACADS	Uncharacterized protein	Short-chain specific acyl-CoA dehydrogenase, mitochondrial	92	3	1.15	13	19	51
G1T006	Q9UMX5	NENF	Neudesin neurotrophic factor	Neudesin	98	2	1.15	3	7	19
G1TAL6	Q10471	GALNT2	Polypeptide N-acetylgalactosaminyltransferase	Polypeptide N-acetylgalactosaminyltransferase 2	99	2	1.15	10	15	23
	P35527	KRT9		Keratin, type I cytoskeletal 9		4	1.15	19	37	44
G1U150	M0QXU7	TIMM44	Mitochondrial import inner membrane translocase subunit TIM44	Mitochondrial import inner membrane translocase subunit TIM44 (Fragment)	83	2	1.15	7	11	18
G1TBW7	P50148	GNAQ	Uncharacterized protein	Guanine nucleotide-binding protein G(q) subunit alpha	99	3	1.14	11	14	38
G1TA15	Q9P2J5	LARS	Uncharacterized protein	Leucine--tRNA ligase, cytoplasmic	95	3	1.14	25	48	29
G1SM77	P36542	ATP5F1C	ATP synthase subunit gamma	ATP synthase subunit gamma, mitochondrial	94	2	1.14	11	102	43
G1T9P2		CARKD	ATP-dependent (S)-NAD(P)H-hydrate dehydratase			1	1.14	6	9	31
G1SZ66	I3L295	MPDU1	Uncharacterized protein	Mannose-P-dolichol utilization defect 1 isoform 2	89	3	1.14	3	6	15
	P52815	MRPL12		39S ribosomal protein L12, mitochondrial		4	1.14	2	3	13
U3KMP1	P61026	RAB10	Uncharacterized protein	Ras-related protein Rab-10	100	3	1.14	9	10	54
U3KM96	P61224	RAP1B	Uncharacterized protein	Ras-related protein Rap-1b	100	3	1.14	11	6	73
G1U3G0	Q9C0C9	UBE2O	Ubiquitin conjugating enzyme E2 O	(E3-independent) E2 ubiquitin-conjugating enzyme	96	2	1.14	3	3	4
G1SP32	Q6NVY1	HIBCH	3-hydroxyisobutyryl-CoA hydrolase, mitochondrial	3-hydroxyisobutyryl-CoA hydrolase, mitochondrial	84	2	1.14	8	9	25
	P40429	RPL13A		60S ribosomal protein L13a		4	1.14	9	13	37
G1T798	Q12846	STX4	t-SNARE coiled-coil homology domain-containing protein	Syntaxin-4	96	2	1.14	4	7	27
G1SKE2	E7ETC0	TIAL1	TIA1 cytotoxic granule associated RNA binding protein like 1	Nucleolysin TIAR	98	2	1.14	2	4	8
G1TSK4		AARS2	AA_TRNA_LIGASE_II_ALA domain-containing protein			1	1.14	2	3	4
G1SS37	A0A494C1M4	ALDH1L2	10-formyltetrahydrofolate dehydrogenase	10-formyltetrahydrofolate dehydrogenase	96	2	1.14	44	93	66
G1T3M5	O43324	EEF1E1	GST C-terminal domain-containing protein	Eukaryotic translation elongation factor 1 epsilon-1	95	2	1.14	6	7	44
G1SLE1		HARS2	AA_TRNA_LIGASE_II domain-containing protein			1	1.14	4	3	10
G1SPT2	P26196	DDX6	Uncharacterized protein	Probable ATP-dependent RNA helicase DDX6	99	3	1.14	13	20	39
G1SJE4	Q92556	ELMO1	ELMO domain-containing protein	Engulfment and cell motility protein 1	97	2	1.14	6	8	13
G1TSL1	A0A087WZN1	IDH3B	Isocitrate dehydrogenase [NAD] subunit, mitochondrial	Isocitrate dehydrogenase [NAD] subunit, mitochondrial	95	2	1.14	14	19	42
G1SE65	A0A0C4DG95	PXK	Uncharacterized protein	PX domain-containing protein kinase-like protein	94	3	1.14	2	2	7
G1SYR5		EIF2B2	Translation initiation factor eIF-2B subunit beta			1	1.14	2	3	8
G1TGY9		VPS11	VPS11, CORVET/HOPS core subunit			1	1.14	2	2	3
G1TEK3	Q8IWJ2	GCC2	GRIP domain-containing protein	GRIP and coiled-coil domain-containing protein 2	86	2	1.14	9	14	8
G1SG42	Q52LJ0	FAM98B	Uncharacterized protein	Protein FAM98B	94	3	1.13	9	7	34
G1T8C2	F8W7U8	MRE11	Double-strand break repair protein	Double-strand break repair protein	93	2	1.13	4	2	9
G1SNR2	Q969S9	GFM2	Ribosome-releasing factor 2, mitochondrial	Ribosome-releasing factor 2, mitochondrial	88	2	1.13	4	4	10
G1TA11	P54136	RARS	Uncharacterized protein	Arginine--tRNA ligase, cytoplasmic	92	3	1.13	23	43	43
G1T524	P05141	SLC25A5	Uncharacterized protein	ADP/ATP translocase 2	98	3	1.13	22	20	69
G1SZI0		SPG7	SPG7, paraplegin matrix AAA peptidase subunit			1	1.13	3	3	10
G1SR03	P55072	VCP	Uncharacterized protein	Transitional endoplasmic reticulum ATPase	100	3	1.13	39	164	69
G1T5Y2	Q9NUQ9	FAM49B	Uncharacterized protein	Protein FAM49B	100	3	1.13	3	3	16
G1TFB5	Q9NP72	RAB18	Uncharacterized protein	Ras-related protein Rab-18	98	3	1.13	8	14	49
G1TYV6	Q9HD20	ATP13A1	Cation-transporting ATPase	Manganese-transporting ATPase 13A1	95	2	1.13	12	14	15
G1SHZ8	P50990	CCT8	Uncharacterized protein	T-complex protein 1 subunit theta	97	3	1.13	33	72	71
O18757	Q6NUK1	SLC25A24	Calcium-binding mitochondrial carrier protein SCaMC-1	Calcium-binding mitochondrial carrier protein SCaMC-1	95	2	1.13	14	5	43
G1SQA8	P06576	ATP5F1B	ATP synthase subunit beta	ATP synthase subunit beta, mitochondrial	97	2	1.13	29	531	74
G1SYL8	I3L0M9	ELOB	Elongin B	Elongin-B (Fragment)	82	2	1.13	4	4	43
B6V9S9	P78371	CCT2	Chaperonin-containing T-complex polypeptide beta subunit	T-complex protein 1 subunit beta	99	2	1.13	27	152	68
G1T7Q3	F5H157	RAB35	Uncharacterized protein	Ras-related protein Rab-35 (Fragment)	91	3	1.13	6	19	40
G1TAF8	P31948	STIP1	Uncharacterized protein	Stress-induced-phosphoprotein 1	96	3	1.13	26	42	52
G1TMS5	P48643	CCT5	Uncharacterized protein	T-complex protein 1 subunit epsilon	99	3	1.13	31	82	70
G1SUP8	P67812	SEC11A	Signal peptidase complex catalytic subunit SEC11	Signal peptidase complex catalytic subunit SEC11A	100	2	1.13	6	9	40
G1SNY3	Q68E01	INTS3	Uncharacterized protein	Integrator complex subunit 3	99	3	1.13	6	5	12

	A6XGL3	PRSS1		Protease serine 1		4	1.13	2	11	8
G1T237	F8VVM2	SLC25A3	Uncharacterized protein	Phosphate carrier protein, mitochondrial	86	3	1.13	14	112	48
G1SGV5	Q8NE71	ABCF1	Uncharacterized protein	ATP-binding cassette sub-family F member 1	92	3	1.13	11	13	27
G1TMQ8	H0Y9V7	ATP2C1	Calcium-transporting ATPase	Calcium-transporting ATPase type 2C member 1 (Fragment)	99	2	1.13	4	5	9
G1TS38	H0YF06	CCDC90B	Uncharacterized protein	Coiled-coil domain-containing protein 90B, mitochondrial (Fragment)	94	3	1.13	2	2	18
G1SMP5	Q5T160	RARS2	DALR_1 domain-containing protein	Probable arginine--tRNA ligase, mitochondrial	93	2	1.13	6	6	10
U3KM71		ATP5MG	ATP synthase subunit			1	1.12	5	15	62
G1U4R5	A0A0C4DGS1	DDOST	Dolichyl-diphosphooligosaccharide--protein glycosyltransferase 48 kDa subunit	Dolichyl-diphosphooligosaccharide--protein glycosyltransferase 48 kDa subunit	94	2	1.12	14	442	39
G1T866	Q96CS3	FAF2	UBX domain-containing protein	FAS-associated factor 2	99	2	1.12	7	17	24
G1SDD3		KCTD10	BTB domain-containing protein			1	1.12	2	2	9
	F8VV64	TNS2		Tensin-2		4	1.12	2	3	2
G1SMZ9		ICMT	Protein-S-isoprenylcysteine O-methyltransferase			1	1.12	2	2	11
G1SSL2	F8VXJ7	CNPY2	Saposin B-type domain-containing protein	Protein canopy homolog 2 (Fragment)	99	2	1.12	7	23	56
G1SQT0	P60510	PPP4C	Serine/threonine-protein phosphatase	Serine/threonine-protein phosphatase 4 catalytic subunit	100	2	1.12	3	2	14
G1SP51	P62277	RPS13	Ribosomal_S13_N domain-containing protein	40S ribosomal protein S13	100	2	1.12	8	16	46
G1TX74	Q6IBS0	TWF2	Uncharacterized protein	Twinfilin-2	95	3	1.12	4	3	17
G1T810	A0A0A0MTN0	CUL2	CULLIN_2 domain-containing protein	Cullin-2	99	2	1.12	5	6	10
G1SDN3	K7EM18	EIF1	SUI1 domain-containing protein	Eukaryotic translation initiation factor 1	100	2	1.12	5	6	44
G1TRG8	P04899	GNAI2	Uncharacterized protein	Guanine nucleotide-binding protein G(i) subunit alpha-2	98	3	1.12	15	18	50
G1SDK8	Q13564	NAE1	NEDD8-activating enzyme E1 regulatory subunit	NEDD8-activating enzyme E1 regulatory subunit	96	2	1.12	3	3	11
G1SI54		ILVBL	IlvB acetolactate synthase like			1	1.12	5	5	19
G1SI94		CRLF3	Cytokine receptor like factor 3			1	1.12	2	2	14
Q9GKX2		DHRS4	Dehydrogenase/reductase SDR family member 4 (Fragment)			1	1.12	8	14	38
G1T659	Q9UHY7	ENOPH1	Enolase-phosphatase E1	Enolase-phosphatase E1	97	2	1.12	2	2	12
G1T7Y5	H0Y368	DPM1	Dolichol-phosphate mannosyltransferase subunit 1	Dolichol-phosphate mannosyltransferase subunit 1 (Fragment)	83	2	1.11	5	6	23
G1SZ59	P60842	EIF4A1	Eukaryotic initiation factor 4A-I	Eukaryotic initiation factor 4A-I	100	2	1.11	23	189	76
	A6NM71	WDR45		PRA1 family protein		4	1.11	3	3	9
G1T146	E7ESY4	MTA1	Metastasis associated 1	Metastasis-associated protein MTA1	88	2	1.11	4	2	9
Q28888		DCN	Decorin			1	1.11	4	3	16
G1TLE4	P62879	GNB2	WD_REPEATS_REGION domain-containing protein	Guanine nucleotide-binding protein G(I)/G(S)/G(T) subunit beta-2	100	2	1.11	11	7	43
G1SGL4	Q9Y276	BCS1L	Uncharacterized protein	Mitochondrial chaperone BCS1	96	3	1.11	6	5	26
G1SR63		PREB	WD_REPEATS_REGION domain-containing protein			1	1.11	3	7	15
G1T093	Q9NZJ4-2	SACS	Uncharacterized protein	Isoform 2 of Sacsin	96	3	1.11	5	4	2
G1SF78	Q96RL7	VPS13A	Vacuolar protein sorting 13 homolog A	Vacuolar protein sorting-associated protein 13A	89	2	1.11	4	5	3
G1TP30	H0Y4Q3	RANGAP1	RanGAP1_C domain-containing protein	Ran GTPase-activating protein 1 (Fragment)	80	2	1.11	7	18	20
	O43290	SART1		U4/U6.U5 tri-snRNP-associated protein 1		4	1.11	2	2	5
	P56385	ATP5ME		ATP synthase subunit e, mitochondrial		4	1.11	3	3	22
G1SXR1		PRELP	LRRNT domain-containing protein			1	1.11	4	6	17
G1T0L9	P04843	RPN1	Dolichyl-diphosphooligosaccharide--protein glycosyltransferase subunit 1	Dolichyl-diphosphooligosaccharide--protein glycosyltransferase subunit 1	97	2	1.11	28	231	53
G1T297	Q9UBV2	SEL1L	Fibronectin type-II domain-containing protein	Protein sel-1 homolog 1	97	2	1.11	15	21	30
G1SPB6	E7EPM6	ACSL1	AMP-binding domain-containing protein	Long-chain-fatty-acid--CoA ligase 1	80	2	1.11	5	9	23
G1SW97	A0A0A0MT83	IVD	Uncharacterized protein	Isovaleryl-CoA dehydrogenase isoform 1	95	3	1.11	7	10	17
G1SZ19	Q9Y3L5	RAP2C	Uncharacterized protein	Ras-related protein Rap-2c	100	3	1.11	6	4	38
G1T3D9	P62241	RPS8	40S ribosomal protein S8	40S ribosomal protein S8	94	2	1.11	6	22	37
G1SCW9		CHPF2	Hexosyltransferase			1	1.11	4	5	10
G1SXJ6		CUL4A	CULLIN_2 domain-containing protein			1	1.11	10	7	17
	H0Y5K5	ERGIC3		Endoplasmic reticulum-Golgi intermediate compartment protein 3 (Fragment)		4	1.11	2	2	5
G1U0Y6		ECI1	Enoyl-CoA delta isomerase 1			1	1.10	9	17	63
G1SFN5	E9PFR3	PPP2R5D	Serine/threonine-protein phosphatase 2A 56 kDa regulatory subunit	Serine/threonine-protein phosphatase 2A 56 kDa regulatory subunit	98	2	1.10	6	9	14
G1TBN5		TOR1AIP2	Torsin 1A interacting protein 2			1	1.10	2	4	6
G1U2W0	B4E321	OS9	OS9, endoplasmic reticulum lectin	Protein OS-9	79	2	1.10	3	2	5
G1SYC9	H0YNE9	RAB8B	Uncharacterized protein	Ras-related protein Rab-8B (Fragment)	99	3	1.10	5	5	19
G1SDT9	P49754	VPS41	Vacuolar protein sorting-associated protein 41 homolog	Vacuolar protein sorting-associated protein 41 homolog	98	2	1.10	5	6	8
G1SFE9	H0YJG7	AHSA1	Aha1_N domain-containing protein	Activator of 90 kDa heat shock protein ATPase homolog 1 (Fragment)	95	2	1.10	3	4	14
	H0YEN5	RPS2		40S ribosomal protein S2 (Fragment)		4	1.10	10	6	54
G1T9F3	Q14974	KPNB1	Importin N-terminal domain-containing protein	Importin subunit beta-1	99	2	1.10	28	93	50
G1SNN0		CSGALNACT2	Hexosyltransferase			1	1.10	2	2	7

G1U276	A0A2R8Y6Y7	SUCLA2	Succinate--CoA ligase [ADP-forming] subunit beta, mitochondrial	Succinate--CoA ligase [ADP-forming] subunit beta, mitochondrial	91	2	1.10	13	21	38
G1SJ20	A0A1W2PNV3	GOSR2	Uncharacterized protein	Golgi SNAP receptor complex member 2 (Fragment)	92	3	1.10	5	10	37
G1T275	Q8WU90	ZC3H15	Uncharacterized protein	Zinc finger CCCH domain-containing protein 15	98	3	1.10	3	2	9
G1T3N8	Q13155	AIMP2	Uncharacterized protein	Aminoacyl tRNA synthase complex-interacting multifunctional protein 2	87	3	1.10	12	21	68
	Q96HP0	DOCK6		Dedicator of cytokinesis protein 6		4	1.10	3	5	2
G1SEF8	M0R0Y2	NAPA	Uncharacterized protein	Alpha-soluble NSF attachment protein	84	3	1.10	10	13	49
G1SQL1	Q9HAU5	UPF2	Uncharacterized protein	Regulator of nonsense transcripts 2	97	3	1.10	2	2	2
U3KM62		PTGS2	Prostaglandin G/H synthase 2			1	1.10	6	7	14
G1SWR1	O43818	RRP9	WD_REPEATS_REGION domain-containing protein	U3 small nucleolar RNA-interacting protein 2	94	2	1.10	5	6	13
G1SE46	O95159	ZFPL1	Zinc finger protein like 1	Zinc finger protein-like 1	92	2	1.10	4	4	15
	K7ER00	FARSA		Phenylalanine--tRNA ligase alpha subunit		4	1.10	3	2	6
	Q9Y2Q5	LAMTOR2		Ragulator complex protein LAMTOR2		4	1.10	2	2	22
G1SKD9	P53597	SUCLG1	Succinate--CoA ligase [ADP/GDP-forming] subunit alpha, mitochondrial	Succinate--CoA ligase [ADP/GDP-forming] subunit alpha, mitochondrial	96	2	1.10	7	26	25
G1SNZ8		GORASP1	GRASP55_65 domain-containing protein			1	1.10	2	2	7
G1SIJ8	Q8TEX9	IPO4	Importin N-terminal domain-containing protein	Importin-4	89	2	1.10	6	8	10
G1SP21	H0Y9A1	YIPF3	Uncharacterized protein	Protein YIPF3 (Fragment)	72	3	1.10	3	9	16
G1SDA8	P25786	PSMA1	Proteasome endopeptidase complex	Proteasome subunit alpha type-1	100	2	1.10	10	22	49
G1TZC9	A0A087WXU3	ESYT2	Extended synaptotagmin 2	Extended synaptotagmin-2	92	2	1.09	13	7	25
G1U6X6	P55795	HNRNPH2	Uncharacterized protein	Heterogeneous nuclear ribonucleoprotein H2	100	3	1.09	10	10	34
G1TPM1	Q96SB3	PPP1R9B	PDZ domain-containing protein	Neurabin-2	99	2	1.09	6	6	15
G1SVT4	P51665	PSMD7	MPN domain-containing protein	26S proteasome non-ATPase regulatory subunit 7	99	2	1.09	9	18	38
G1TFL3	J3KQE5	RAN	GTP-binding nuclear protein Ran	GTP-binding nuclear protein Ran (Fragment)	96	2	1.09	9	24	36
G1SYI3	A0A087X1B2	USP39	Uncharacterized protein	U4/U6.U5 tri-snRNP-associated protein 2	99	3	1.09	5	7	14
G1T329	J3QQY1	CDK5RAP3	Uncharacterized protein	CDK5 regulatory subunit-associated protein 3 (Fragment)	88	3	1.09	9	13	25
G1TWU1		TOR1B	Torsin			1	1.09	5	4	19
G1TUD2	Q9UBI6	GNG12	Guanine nucleotide-binding protein subunit gamma	Guanine nucleotide-binding protein G(I)/G(S)/G(O) subunit gamma-12	100	2	1.09	4	5	67
G1U484		LLGL1	LLGL scribble cell polarity complex component 1			1	1.09	2	2	3
Q01971	P61019	RAB2A	Ras-related protein Rab-2A	Ras-related protein Rab-2A	100	2	1.09	12	45	67
G1T923	A0A1B0GTB0	ATP6AP2	Uncharacterized protein	Renin receptor (Fragment)	84	3	1.09	4	4	21
P41035	P20042	EIF2S2	Eukaryotic translation initiation factor 2 subunit 2	Eukaryotic translation initiation factor 2 subunit 2	98	2	1.09	12	25	53
G1SHF3		NIT1	CN hydrolase domain-containing protein			1	1.09	3	4	14
G1SL46		PSMD9	PDZ domain-containing protein			1	1.09	3	2	15
	Q9H0U4	RAB1B		Ras-related protein Rab-1B		4	1.09	12	10	69
	Q92900	UPF1		Regulator of nonsense transcripts 1		4	1.09	18	26	23
G1U222	A0A384DVK7	ARHGEF10L	Rho guanine nucleotide exchange factor 10 like	Rho guanine nucleotide exchange factor 10-like protein (Fragment)	93	2	1.09	2	2	4
G1SNI4	O94760	DDAH1	Uncharacterized protein	N(G),N(G)-dimethylarginine dimethylaminohydrolase 1	96	3	1.09	6	6	24
G1SRJ6		AFAP1	Actin filament associated protein 1			1	1.09	3	7	7
G1U6B2		ALAD	Delta-aminolevulinic acid dehydratase			1	1.09	2	2	15
G1TKY7		OGFOD3	2-oxoglutarate and iron dependent oxygenase domain containing 3			1	1.09	3	3	13
G1T2W1	O76094	SRP72	Signal recognition particle subunit SRP72	Signal recognition particle subunit SRP72	98	2	1.09	9	16	20
G1SYY0	Q96SK2	TMEM209	Uncharacterized protein	Transmembrane protein 209	96	3	1.09	3	6	12
G1T4Z2	P53396	ACLY	ATP-citrate synthase	ATP-citrate synthase	98	2	1.09	17	30	23
G1SKT4	P25705	ATP5F1A	ATP synthase subunit alpha	ATP synthase subunit alpha, mitochondrial	98	2	1.09	29	377	62
G1SLS8	Q9NX62	IMPAD1	Uncharacterized protein	Inositol monophosphatase 3	96	3	1.09	6	8	21
G1T2K6	Q8NBU5	ATAD1	AAA domain-containing protein	ATPase family AAA domain-containing protein 1	100	2	1.09	2	4	10
	P29992	GNA11		Guanine nucleotide-binding protein subunit alpha-11		4	1.09	6	2	22
G1TVG8	O15173	PGRMC2	Cytochrome b5 heme-binding domain-containing protein	Membrane-associated progesterone receptor component 2	97	2	1.09	8	10	33
G1TBR5	B4DR61	SEC61A1	Plug_translocon domain-containing protein	Protein transport protein Sec61 subunit alpha isoform 1	100	2	1.09	11	79	39
G1T9W3	K4DI93	CUL4B	CULLIN_2 domain-containing protein	Cullin 4B, isoform CRA_e	100	2	1.08	10	5	14
G1SE56	F6T1Q0	PDE12	Endo/exonuclease/phosphatase domain-containing protein	2~,5~-phosphodiesterase 12	90	2	1.08	3	2	10
G1TBX9	Q9H8M7	MINDY3	DUF4205 domain-containing protein	Ubiquitin carboxyl-terminal hydrolase MINDY-3	99	2	1.08	3	3	13
G1TH06	F6WQW2	RANBP1	RAN binding protein 1	Ran-specific GTPase-activating protein	91	2	1.08	4	7	28
G1SWA0		RIPOR1	RHO family interacting cell polarization regulator 1			1	1.08	3	3	5
O46373	P12235	SLC25A4	ADP/ATP translocase 1	ADP/ATP translocase 1	96	2	1.08	18	25	71
G1STU7	Q5QNZ2	ATP5PB	Uncharacterized protein	ATP synthase F(0) complex subunit B1, mitochondrial	84	3	1.08	12	47	24
G1SLF8	J3KN16	ECPAS	Vac14_Fab1_bd domain-containing protein	Proteasome adapter and scaffold protein ECM29	97	2	1.08	18	29	17
G1SZ23	E9PCG9	BDH1	Uncharacterized protein	D-beta-hydroxybutyrate dehydrogenase, mitochondrial	87	3	1.08	9	12	32

G1SMB5		VPS37C	VPS37 C-terminal domain-containing protein			1	1.08	4	4	21
G1TW04	Q16643	DBN1	Drebrin 1	Drebrin	63	2	1.08	12	95	28
G1U3I5		ECH1	Enoyl-CoA hydratase 1			1	1.08	9	9	37
P62139	P62136	PPP1CA	Serine/threonine-protein phosphatase PP1-alpha catalytic subunit	Serine/threonine-protein phosphatase PP1-alpha catalytic subunit	100	2	1.08	11	2	51
G1T6L7	F8WAR4	CHCHD3	MICOS complex subunit	MICOS complex subunit	78	2	1.08	3	4	9
G1SJ37	F8W9S7	GAPVD1	Uncharacterized protein	GTPase-activating protein and VPS9 domain-containing protein 1	95	3	1.08	3	4	4
G1T7P8	Q9UIV1	CNOT7	Uncharacterized protein	CCR4-NOT transcription complex subunit 7	100	3	1.08	3	3	16
G1SLJ9	Q9P0I2	EMC3	ER membrane protein complex subunit 3	ER membrane protein complex subunit 3	99	2	1.07	3	3	20
G1SXB8	Q10567	AP1B1	AP complex subunit beta	AP-1 complex subunit beta-1	98	2	1.07	32	8	45
G1SKK5		PEX6	Peroxisomal biogenesis factor 6			1	1.07	2	3	6
G1T168	P46783	RPS10	S10_plectin domain-containing protein	40S ribosomal protein S10	100	2	1.07	10	15	40
G1U7X2	Q15637	SF1	CCHC-type domain-containing protein	Splicing factor 1	99	2	1.07	4	5	12
G1U4V6		CACNA2D1	Voltage-dependent calcium channel subunit alpha-2/delta-1			1	1.07	9	2	13
G1SG68	O00629	KPNA4	Importin subunit alpha	Importin subunit alpha-3	99	2	1.07	7	15	24
G1TM48	Q5JRA6	MIA3	SH3 domain-containing protein	Transport and Golgi organization protein 1 homolog	71	2	1.07	5	4	3
G1TS36	B8ZZG1	MPP6	Uncharacterized protein	Membrane protein, palmitoylated 6 (MAGUK p55 subfamily member 6), isoform CRA_a	96	3	1.07	5	4	10
G1SVU0	C9JAZ1	MTX2	Uncharacterized protein	Metaxin-2 (Fragment)	89	3	1.07	4	13	37
G1TLQ8	R4GNH3	PSMC3	AAA domain-containing protein	26S proteasome regulatory subunit 6A	100	2	1.07	21	46	65
G1U354	R4GMR5	PSMD8	PCI domain-containing protein	26S proteasome non-ATPase regulatory subunit 8	97	2	1.07	9	14	33
G1SXB6	B4DVA9	POGLUT1	CAP10 domain-containing protein	Protein O-glucosyltransferase 1	96	2	1.07	3	3	14
G1SIX1	Q9H2G2	SLK	Uncharacterized protein	STE20-like serine/threonine-protein kinase	88	3	1.07	6	7	12
G1T9V2	A0A2R8Y852	CUX1	Cut like homeobox 1	Homeobox protein cut-like	87	2	1.07	4	5	5
G1SS79	P29966	MARCKS	Uncharacterized protein	Myristoylated alanine-rich C-kinase substrate	87	3	1.07	4	5	25
G1SM62	O43252	PAPSS1	Uncharacterized protein	Bifunctional 3~-phosphoadenosine 5~-phosphosulfate synthase 1	99	3	1.07	9	15	24
G1SJX1	Q16537	PPP2R5E	Serine/threonine-protein phosphatase 2A 56 kDa regulatory subunit	Serine/threonine-protein phosphatase 2A 56 kDa regulatory subunit epsilon isoform	100	2	1.07	4	6	13
G1TLK9	P49748	ACADVL	Uncharacterized protein	Very long-chain specific acyl-CoA dehydrogenase, mitochondrial	87	3	1.07	22	60	49
G1SFE0	O00487	PSMD14	MPN domain-containing protein	26S proteasome non-ATPase regulatory subunit 14	100	2	1.07	7	10	45
G1U0M5	H7C1W1	PXDN	Peroxidasin	Peroxidasin homolog (Fragment)	93	2	1.07	5	3	5
G1SHH0	Q14139	UBE4A	Ubiquitination factor E4A	Ubiquitin conjugation factor E4 A	98	2	1.07	3	6	7
G1SM91		FAH	Fumarylacetoacetase			1	1.07	2	2	7
G1U0B5	Q9Y570	PPME1	Protein phosphatase methylesterase 1	Protein phosphatase methylesterase 1	96	2	1.07	3	4	16
U3KMI4	P62834	RAP1A	Uncharacterized protein	Ras-related protein Rap-1A	100	3	1.07	8	2	65
G1TBC0	A0A499FI31	SART3	Uncharacterized protein	Squamous cell carcinoma antigen recognized by T-cells 3	89	3	1.07	2	3	4
G1U8V2	Q96DZ1	ERLEC1	Uncharacterized protein	Endoplasmic reticulum lectin 1	98	3	1.07	3	7	8
G1SMC8	Q14997	PSME4	Uncharacterized protein	Proteasome activator complex subunit 4	98	3	1.07	5	3	5
G1SIS5	O15498	YKT6	Uncharacterized protein	Synaptobrevin homolog YKT6	96	3	1.07	5	6	36
G1SCS8		ANO6	Anoctamin			1	1.06	5	5	8
G1SSX2	Q7Z6Z7	HUWE1	HECT, UBA and WWE domain containing 1, E3 ubiquitin protein ligase	E3 ubiquitin-protein ligase HUWE1	96	2	1.06	19	15	8
G1SZG3	Q8NEW0	SLC30A7	Uncharacterized protein	Zinc transporter 7	97	3	1.06	4	4	18
G1U684	A0A087X1W8	CADM1	Uncharacterized protein	Cell adhesion molecule 1	95	3	1.06	9	11	34
G1SN68	A0A1B0GVU9	QARS	Uncharacterized protein	Glutamine--tRNA ligase (Fragment)	92	3	1.06	25	38	43
G1SZD2	P53701	HCCS	Cytochrome c heme lyase	Cytochrome c-type heme lyase	83	2	1.06	4	6	17
G1T9V7		SRP19	Signal recognition particle 19			1	1.06	2	2	26
G1SLA2	Q92604	LPGAT1	Lysophosphatidylglycerol acyltransferase 1	Acyl-CoA:lysophosphatidylglycerol acyltransferase 1	94	2	1.06	5	6	17
G1TME5	Q9H857	NT5DC2	Uncharacterized protein	5~-nucleotidase domain-containing protein 2	86	3	1.06	4	3	13
G1SK22	P62979	RPS27A	Ubiquitin-like domain-containing protein	Ubiquitin-40S ribosomal protein S27a	100	2	1.06	8	48	47
G1SGX6		SDR39U1	DUF1731 domain-containing protein			1	1.06	2	2	14
G1SFS8	Q7KZF4	SND1	Staphylococcal nuclease domain-containing protein	Staphylococcal nuclease domain-containing protein 1	97	2	1.06	35	80	54
G1T9N2	O75947	ATP5PD	ATP synthase subunit d, mitochondrial	ATP synthase subunit d, mitochondrial	91	2	1.06	10	23	70
G1SLT8	P31942	HNRNPH3	Uncharacterized protein	Heterogeneous nuclear ribonucleoprotein H3	100	3	1.06	6	6	24
G1TVW1	O60568	PLOD3	Procollagen-lysine,2-oxoglutarate 5-dioxygenase 3	Multifunctional procollagen lysine hydroxylase and glycosyltransferase LH3	94	2	1.06	13	10	31
P30946	P07900	HSP90AA1	Heat shock protein HSP 90-alpha	Heat shock protein HSP 90-alpha	94	2	1.06	36	264	63
G1SJN5		MAN2A1	Alpha-mannosidase			1	1.06	8	8	12
G1T0U8	Q9UHG3	PCYOX1	Prenylcys_lyase domain-containing protein	Prenylcysteine oxidase 1	86	2	1.06	10	25	31
G1SX03	Q8TBQ9	TMEM167A	Protein kish	Protein kish-A	96	2	1.06	2	3	46
G1T9R8	Q16222	UAP1	Uncharacterized protein	UDP-N-acetylhexosamine pyrophosphorylase	96	3	1.06	5	6	14
G1T3Q2	Q96HY6	DDRGK1	Uncharacterized protein	DDRGK domain-containing protein 1	90	3	1.05	6	16	25

G1SN95	A0A087WSV8	NUCB2	Nucleobindin 2	Nucleobindin 2, isoform CRA_b	93	2	1.05	18	34	47
G1T2F2	P23284	PPIB	Peptidyl-prolyl cis-trans isomerase	Peptidyl-prolyl cis-trans isomerase B	94	2	1.05	7	8	31
G1T4J2	F5H228	TRIO	Uncharacterized protein	Triple functional domain protein	99	3	1.05	4	5	4
G1SIP6		CISD1	ZnF_CDGSH domain-containing protein			1	1.05	2	2	20
G1SX71		AGPAT5	1-acylglycerol-3-phosphate O-acyltransferase 5			1	1.05	2	3	11
G1SUP1	A0A0D9SEY1	MAP4K4	Mitogen-activated protein kinase kinase kinase kinase 4	Mitogen-activated protein kinase kinase kinase kinase 4	91	2	1.05	6	7	7
G1SZI7		CROT	Carn_acyltransf domain-containing protein			1	1.05	2	2	4
G1TCS8	P62820	RAB1A	Uncharacterized protein	Ras-related protein Rab-1A	100	3	1.05	13	143	74
G1TNU3		STX16	t-SNARE coiled-coil homology domain-containing protein			1	1.05	5	5	23
	O15031	PLXNB2		Plexin-B2		4	1.05	7	6	6
G1TDB3	P62851	RPS25	Uncharacterized protein	40S ribosomal protein S25	100	3	1.05	3	5	16
G1T643	A0A1W2PNX8	UNC45A	Unc-45 myosin chaperone A	Protein unc-45 homolog A	94	2	1.05	8	14	14
G1T520	Q99615	DNAJC7	Uncharacterized protein	DnaJ homolog subfamily C member 7	98	3	1.05	3	3	10
G1SMI6	Q13162	PRDX4	Thioredoxin domain-containing protein	Peroxiredoxin-4	96	2	1.05	10	17	41
G1SJ43	Q9H3S7	PTPN23	Uncharacterized protein	Tyrosine-protein phosphatase non-receptor type 23	91	3	1.05	7	5	5
G1SUR8	B7ZBJ4	CAB39L	Uncharacterized protein	Calcium-binding protein 39-like	98	3	1.05	3	3	12
G1TQA4		EPM2AIP1	EPM2A interacting protein 1			1	1.05	4	5	10
G1SE12	P13804	ETFA	Electron transfer flavoprotein subunit alpha	Electron transfer flavoprotein subunit alpha, mitochondrial	95	2	1.05	11	14	56
G1TH33	P38117	ETFB	Electron transfer flavoprotein subunit beta	Electron transfer flavoprotein subunit beta	95	2	1.05	10	6	47
G1SU97	H0YD97	PDHX	Dihydrolipoamide acetyltransferase component of pyruvate dehydrogenase complex	Pyruvate dehydrogenase protein X component, mitochondrial (Fragment)	89	2	1.05	4	6	10
G1U3S6	O15258	RER1	Protein RER1	Protein RER1	95	2	1.05	2	2	13
G1T3H5		EIF2B3	NTP_transferase domain-containing protein			1	1.05	4	4	12
G1SPG6	A6NEM5	PIGK	GPI-anchor transamidase	GPI-anchor transamidase	85	2	1.05	4	6	22
G1TR92		SYDE1	Synapse defective Rho GTPase homolog 1			1	1.05	3	4	7
G1SQT2	Q6P2E9	EDC4	WD_REPEATS_REGION domain-containing protein	Enhancer of mRNA-decapping protein 4	96	2	1.04	3	3	5
G1SGS7	O94874	UFL1	Uncharacterized protein	E3 UFM1-protein ligase 1	94	3	1.04	16	24	29
G1T501		CCAR2	Cell cycle and apoptosis regulator 2			1	1.04	2	4	6
G1TN86	C9IZG4	CUTA	Uncharacterized protein	Protein CutA	93	3	1.04	2	2	18
G1SSV4	A0A087WT80	PLCB1	1-phosphatidylinositol 4,5-bisphosphate phosphodiesterase	1-phosphatidylinositol 4,5-bisphosphate phosphodiesterase	97	2	1.04	3	3	5
G1TB98	Q15293	RCN1	Uncharacterized protein	Reticulocalbin-1	85	3	1.04	12	9	45
G1SDD0	P30084	ECHS1	Uncharacterized protein	Enoyl-CoA hydratase, mitochondrial	86	3	1.04	9	16	45
G1SRW4	Q9Y6C2	EMILIN1	Elastin microfibril interfacer 1	EMILIN-1	87	2	1.04	5	5	6
G1STS3	Q96T76	MMS19	Uncharacterized protein	MMS19 nucleotide excision repair protein homolog	93	3	1.04	8	7	16
	I3L0A0	TMEM189-UBE2V1		HCG2044781		4	1.04	6	3	23
G1SUL3		TMF1	TMF_TATA_bd domain-containing protein			1	1.04	2	2	3
G1TA59	P50213	IDH3A	Isocitrate dehydrogenase [NAD] subunit, mitochondrial	Isocitrate dehydrogenase [NAD] subunit alpha, mitochondrial	98	2	1.04	12	31	40
G1SVA3	O00231	PSMD11	PCI domain-containing protein	26S proteasome non-ATPase regulatory subunit 11	100	2	1.04	17	34	56
G1T593	F8VVA7	COPZ1	Clat_adaptor_s domain-containing protein	Coatomer subunit zeta-1	88	2	1.04	4	11	45
P27115		MGAT1	Alpha-1,3-mannosyl-glycoprotein 2-beta-N-acetylglucosaminyltransferase			1	1.04	3	3	12
	Q9NRP0	OSTC		Oligosaccharyltransferase complex subunit OSTC		4	1.04	2	6	13
G1SJL0		TIMM29	Translocase of inner mitochondrial membrane 29			1	1.04	2	2	16
G1SG72	P61221	ABCE1	Uncharacterized protein	ATP-binding cassette sub-family E member 1	100	3	1.04	14	19	32
G1SI37	D6RF87	ACSF2	Uncharacterized protein	Acyl-CoA synthetase family member 2, mitochondrial (Fragment)	82	3	1.04	18	31	51
G1U013	H7C3P9	COPS3	PCI domain-containing protein	COP9 signalosome complex subunit 3	92	2	1.04	4	5	18
G1SRP7	O00505	KPNA3	Importin subunit alpha	Importin subunit alpha-4	99	2	1.04	8	10	27
G1SGX4	P62249	RPS16	Uncharacterized protein	40S ribosomal protein S16	100	3	1.04	7	13	37
G1TE76	Q15056	EIF4H	Eukaryotic translation initiation factor 4H	Eukaryotic translation initiation factor 4H	92	2	1.04	3	7	23
G1SVF2	A0A087WW66	PSMD1	26S proteasome non-ATPase regulatory subunit 1	26S proteasome non-ATPase regulatory subunit 1	99	2	1.04	29	7	44
G1TJ79		KYAT1	Aminotran_1_2 domain-containing protein			1	1.04	2	2	10
G1SLK2	P62195	PSMC5	AAA domain-containing protein	26S proteasome regulatory subunit 8	100	2	1.04	14	23	43
G1TP15	O43242	PSMD3	PCI domain-containing protein	26S proteasome non-ATPase regulatory subunit 3	98	2	1.04	20	37	43
G1SNP4	A0A3B3ITZ9	THRAP3	Uncharacterized protein	Thyroid hormone receptor-associated protein 3	95	3	1.04	6	8	8
G1T6M2		DHRS7B	Dehydrogenase/reductase 7B			1	1.04	6	10	22
P40144		ADCY5	Adenylate cyclase type 5			1	1.04	3	4	4
Q95MN6		PLP2	Proteolipid protein 2			1	1.04	3	7	34
G1T3S1	A0A087X2I1	PSMC6	AAA domain-containing protein	26S proteasome regulatory subunit 10B	100	2	1.04	12	24	42
G1SV51	Q08378	GOLGA3	Golgin A3	Golgin subfamily A member 3	85	2	1.04	12	12	16

G1T279	Q9UHW5	GPN3	GPN-loop GTPase 3	GPN-loop GTPase 3	96	2	1.04	2	3	12
G1SK67	Q13547	HDAC1	Histone deacetylase	Histone deacetylase 1	99	2	1.04	6	2	23
G1SMW3	Q92615	LARP4B	La ribonucleoprotein domain family member 4B	La-related protein 4B	88	2	1.04	3	3	8
G1SXI8		PCID2	PCI domain containing 2			1	1.04	2	2	6
G1T579	Q6NUQ1	RINT1	Uncharacterized protein	RAD50-interacting protein 1	92	3	1.04	7	8	15
	Q9UNK0	STX8		Syntaxin-8		4	1.04	2	2	12
Q28611		UGT1	UDP-glucuronosyltransferase 1-6			1	1.04	10	13	24
G1SIB1	P29083	GTF2E1	HTH TFE/IIEalpha-type domain-containing protein	General transcription factor IIE subunit 1	94	2	1.03	2	2	9
G1SPN1		NUDCD2	CS domain-containing protein			1	1.03	2	3	17
G1TE13	P84095	RHOG	Uncharacterized protein	Rho-related GTP-binding protein RhoG	99	3	1.03	6	8	41
G1TKE0	Q9P2X0	DPM3	Dolichol-phosphate mannosyltransferase subunit 3	Dolichol-phosphate mannosyltransferase subunit 3	95	2	1.03	2	2	24
G1SHK7	A0A0C4DGV 4	LAMTOR5	Uncharacterized protein	Hepatitis B virus x interacting protein	100	3	1.03	3	5	31
G1T4N5	P42345	MTOR	Serine/threonine-protein kinase mTOR	Serine/threonine-protein kinase mTOR	99	2	1.03	16	17	11
G1T3Z6	P31323	PRKAR2B	Uncharacterized protein	cAMP-dependent protein kinase type II-beta regulatory subunit	97	3	1.03	7	7	27
G1SSA2	Q13200	PSMD2	26S proteasome non-ATPase regulatory subunit 2	26S proteasome non-ATPase regulatory subunit 2	99	2	1.03	30	77	47
G1U886	A0A2R8YGH 5	AP1S1	AP complex subunit sigma	AP complex subunit sigma	100	2	1.03	4	3	33
G1SXZ9	Q86WA6	BPHL	Biphenyl hydrolase like	Valacyclovir hydrolase	89	2	1.03	7	7	25
G1SL07	Q9NUP9	LIN7C	Protein lin-7 homolog	Protein lin-7 homolog C	99	2	1.03	5	4	29
G1T2C3	O75534	CSDE1	Uncharacterized protein	Cold shock domain-containing protein E1	99	3	1.03	15	20	21
	A6NG10	WBP2		WW domain-binding protein 2		4	1.03	2	2	7
G1T5N5	C9IZ01	GFM1	Elongation factor G, mitochondrial	Elongation factor G, mitochondrial	93	2	1.03	8	9	19
G1SCY3		UBR4	UBR-type domain-containing protein			1	1.03	46	71	17
G1SIP1	Q96LJ7	DHRS1	Uncharacterized protein	Dehydrogenase/reductase SDR family member 1	87	3	1.03	6	6	30
	Q5R3B4	MPC2		Mitochondrial pyruvate carrier (Fragment)		4	1.03	3	4	32
G1SYV0	P35998	PSMC2	AAA domain-containing protein	26S proteasome regulatory subunit 7	100	2	1.03	17	36	46
G1T193		UGT3A2	UDP-glucuronosyltransferase			1	1.03	2	2	6
G1SWI3	P45880	VDAC2	Voltage-dependent anion-selective channel protein 2	Voltage-dependent anion-selective channel protein 2	99	2	1.03	13	65	63
P20647	P16615	ATP2A2	Sarcoplasmic/endoplasmic reticulum calcium ATPase 2	Sarcoplasmic/endoplasmic reticulum calcium ATPase 2	98	2	1.03	29	5	36
G1T1C4		HSD17B7	Hydroxysteroid 17-beta dehydrogenase 7			1	1.03	3	3	16
	Q9NUY8	TBC1D23		TBC1 domain family member 23		4	1.03	3	5	6
G1T6E8	F5H442	TSG101	Uncharacterized protein	Tumor susceptibility gene 101 protein	99	3	1.03	6	8	25
	Q8NBJ5	COLGALT1		Procollagen galactosyltransferase 1		4	1.02	7	34	14
G1SR19	P20594	NPR2	Guanylate cyclase	Atrial natriuretic peptide receptor 2	95	2	1.02	7	10	10
G1SH95	P30622	CLIP1	CAP-Gly domain containing linker protein 1	CAP-Gly domain-containing linker protein 1	85	2	1.02	12	11	10
G1T7J5		NCSTN	Ncstrn_small domain-containing protein			1	1.02	7	17	16
G1SY93	P63000	RAC1	Rac family small GTPase 1	Ras-related C3 botulinum toxin substrate 1	90	2	1.02	6	10	31
G1SVW5	P36578	RPL4	Ribos_L4_asso_C domain-containing protein	60S ribosomal protein L4	96	2	1.02	14	46	36
G1TA10		TMEM109	Transmembrane protein 109			1	1.02	3	6	9
G1U1M3	Q12904	AIMP1	tRNA-binding domain-containing protein	Aminoacyl tRNA synthase complex-interacting multifunctional protein 1	91	2	1.02	8	14	36
	Q8NHH9-2	ATL2		Isoform 2 of Atlastin-2		4	1.02	4	8	11
G1T9M9	P11142	HSPA8	Uncharacterized protein	Heat shock cognate 71 kDa protein	100	3	1.02	32	678	71
G1SRP2	Q5SWX8	ODR4	Uncharacterized protein	Protein odr-4 homolog	89	3	1.02	12	14	43
G1SRB6	Q08752	PPID	Peptidylprolyl isomerase D	Peptidyl-prolyl cis-trans isomerase D	93	2	1.02	9	22	27
G1T3H3	Q8IXI2	RHOT1	Mitochondrial Rho GTPase	Mitochondrial Rho GTPase 1	99	2	1.02	7	8	13
G1T3V3	Q6R327	RICTOR	Uncharacterized protein	Rapamycin-insensitive companion of mTOR	98	3	1.02	4	6	4
G1SVV6	Q02750	MAP2K1	Dual-specificity mitogen-activated protein kinase kinase 1	Dual specificity mitogen-activated protein kinase kinase 1	94	2	1.02	5	10	21
	P39019	RPS19		40S ribosomal protein S19		4	1.02	11	34	57
G1SHI9	A0A0D9SFS3	OGDH	Transket_pyr domain-containing protein	2-oxoglutarate dehydrogenase, mitochondrial	95	2	1.02	29	62	40
	K7ELL7	PRKCSH		Glucosidase 2 subunit beta		4	1.02	2	5	4
G1SUN1	O75915	ARL6IP5	PRA1 family protein	PRA1 family protein 3	96	2	1.02	5	24	24
G1SN52	E7ESC6	XPO7	Exportin 7	Exportin-7	99	2	1.02	5	8	7
G1TBK0		ANKMY2	Ankyrin repeat and MYND domain containing 2			1	1.02	2	3	5
G1TE61	O60684	KPNA6	Importin subunit alpha	Importin subunit alpha-7	99	2	1.02	10	19	34
G1T2R3		EFL1	Tr-type G domain-containing protein			1	1.02	3	2	6
G1T6J2		APOO	MICOS complex subunit			1	1.02	5	8	46
G1SJN4	Q9BT78	COPS4	PCI domain-containing protein	COP9 signalosome complex subunit 4	100	2	1.02	12	26	49
G1T6P0	Q9H7D0	DOCK5	Dedicator of cytokinesis 5	Dedicator of cytokinesis protein 5	96	2	1.02	3	3	3

G1T3L5	O43719	HTATSF1	Uncharacterized protein	HIV Tat-specific factor 1	82	3	1.02	3	3	6
G1T242		IKBIP	IKBKB interacting protein			1	1.02	15	23	39
G1T9V4	G3V5Z7	PSMA6	Proteasome subunit alpha type	Proteasome subunit alpha type	97	2	1.02	10	56	47
G1SZR6	Q13618	CUL3	CULLIN_2 domain-containing protein	Cullin-3	100	2	1.01	13	26	23
G1T645	Q96S52	PIGS	Uncharacterized protein	GPI transamidase component PIG-S	85	3	1.01	6	10	17
G1T4X8	P49721	PSMB2	Proteasome subunit beta	Proteasome subunit beta type-2	99	2	1.01	8	42	60
	O43752	STX6		Syntaxin-6		4	1.01	2	2	13
G1T0S0	Q8NBN3	TMEM87A	Uncharacterized protein	Transmembrane protein 87A	96	3	1.01	4	5	9
G1SIH3	A0A2R8Y5A6	ATXN2	Uncharacterized protein	Ataxin-2	94	3	1.01	5	8	5
	Q9NWU2	GID8		Glucose-induced degradation protein 8 homolog		4	1.01	5	6	41
G1TBU9	A0A0B4J2A4	ACAA2	Uncharacterized protein	3-ketoacyl-CoA thiolase, mitochondrial	89	3	1.01	15	52	64
G1SIW8	Q08257	CRYZ	PKS_ER domain-containing protein	Quinone oxidoreductase	87	2	1.01	7	21	39
G1T6D4	O00232	PSMD12	PCI domain-containing protein	26S proteasome non-ATPase regulatory subunit 12	99	2	1.01	16	45	48
G1TVU4	Q96D15	RCN3	Reticulocalbin 3	Reticulocalbin-3	78	2	1.01	9	37	51
G1SD02	E7EM64	COPS6	COP9 signalosome subunit 6	COP9 signalosome complex subunit 6	97	2	1.01	3	5	11
G1T0H9	O60645	EXOC3	Exocyst complex component 3	Exocyst complex component 3	95	2	1.01	3	4	5
G1SRL3	P30419	NMT1	Glycylpeptide N-tetradecanoyltransferase	Glycylpeptide N-tetradecanoyltransferase 1	98	2	1.01	7	9	19
G1TGF1	Q15185	PTGES3	Prostaglandin E synthase 3	Prostaglandin E synthase 3	100	2	1.01	5	8	52
G1TEM4	Q9UEW8	STK39	Protein kinase domain-containing protein	STE20/SPS1-related proline-alanine-rich protein kinase	95	2	1.01	2	2	4
G1SD91	E9PFD2	UMPS	OMPdecase domain-containing protein	Uridine 5~-monophosphate synthase	92	2	1.01	2	2	6
G1SJK0		APOOL	MICOS complex subunit			1	1.01	3	8	23
G1ST56	Q96P70	IPO9	Importin N-terminal domain-containing protein	Importin-9	99	2	1.01	6	9	15
G1SF45		SEC24B	SEC24 homolog B, COPII coat complex component			1	1.01	3	4	4
G1SQU5	Q9UP95	SLC12A4	Solute carrier family 12 member 4	Solute carrier family 12 member 4	97	2	1.01	13	21	16
G1TKG2		ISOC2	Isochorismatase domain containing 2			1	1.00	4	6	43
G1SHS4	A0A0C4DGX4	CUL1	CULLIN_2 domain-containing protein	Cullin-1	97	2	1.00	5	7	11
G1SGQ0	Q9UBS4	DNAJB11	J domain-containing protein	DnaJ homolog subfamily B member 11	98	2	1.00	9	21	35
G1T3S7	O60476	MAN1A2	alpha-1,2-Mannosidase	Mannosyl-oligosaccharide 1,2-alpha-mannosidase IB	95	2	1.00	2	2	4
G1SG41		TBL2	WD_REPEATS_REGION domain-containing protein			1	1.00	5	6	11
G1SEN5	Q8IZ07	ANKRD13A	Ankyrin repeat domain 13A	Ankyrin repeat domain-containing protein 13A	94	2	1.00	2	2	5
G1T3D1	Q14789	GOLGB1	Uncharacterized protein	Golgin subfamily B member 1	85	3	1.00	32	43	15
G1T0G0		P3H2	Fe2OG dioxygenase domain-containing protein			1	1.00	2	2	4
G1SS70	P61247	RPS3A	40S ribosomal protein S3a	40S ribosomal protein S3a	100	2	1.00	15	87	55
	A0A0A0MRA3	TTN		Titin		4	1.00	2	2	0
G1T7I3	Q13085	ACACA	Uncharacterized protein	Acetyl-CoA carboxylase 1	98	3	1.00	12	12	7
G1SVV2	Q9NZM1	MYOF	Uncharacterized protein	Myoferlin	94	3	1.00	68	110	44
G1SH78		VKORC1	VKc domain-containing protein			1	1.00	2	3	29
G1T846	P14868	DARS	AA_TRNA_LIGASE_II domain-containing protein	Aspartate--tRNA ligase, cytoplasmic	98	2	1.00	10	20	22
G1SXG8	F8VU90	FKBP11	Peptidylprolyl isomerase	Peptidylprolyl isomerase	95	2	1.00	4	10	31
G1SKK0	P22102	GART	Trifunctional purine biosynthetic protein adenosine-3	Trifunctional purine biosynthetic protein adenosine-3	89	2	1.00	6	8	12
G1TAI0		KPNA2	Importin subunit alpha			1	1.00	3	6	11
G1SPD2	Q16401	PSMD5	Uncharacterized protein	26S proteasome non-ATPase regulatory subunit 5	92	3	1.00	12	13	43
G1SDU6	P26639	TARS	AA_TRNA_LIGASE_II domain-containing protein	Threonine--tRNA ligase, cytoplasmic	96	2	1.00	17	25	28
G1T239	F5GYQ1	ATP6V0D1	V-type proton ATPase subunit	V-type proton ATPase subunit	90	2	1.00	8	10	31
G1SCY4	P52907	CAPZA1	F-actin-capping protein subunit alpha	F-actin-capping protein subunit alpha-1	96	2	1.00	10	27	58
G1SF00	Q9BSJ2	TUBGCP2	Gamma-tubulin complex component	Gamma-tubulin complex component 2	90	2	1.00	5	5	7
G1T6W7	P04040	CAT	Catalase	Catalase	91	2	1.00	4	2	16
	P30044	PRDX5		Peroxiredoxin-5, mitochondrial		4	1.00	5	9	33
P62493	P62491	RAB11A	Ras-related protein Rab-11A	Ras-related protein Rab-11A	100	2	1.00	11	34	59
G1TL06	P39023	RPL3	Uncharacterized protein	60S ribosomal protein L3	98	3	1.00	17	191	44
G1SM04	Q13188	STK3	Uncharacterized protein	Serine/threonine-protein kinase 3	99	3	1.00	2	2	5
G1TCM0	J3KQ34	COPS7B	PCI domain-containing protein	COP9 signalosome complex subunit 7b	97	2	1.00	3	7	19
G1SLZ8	Q06124	PTPN11	Tyrosine-protein phosphatase non-receptor type	Tyrosine-protein phosphatase non-receptor type 11	98	2	1.00	6	3	15
G1SZA3	Q9H2M9	RAB3GAP2	Uncharacterized protein	Rab3 GTPase-activating protein non-catalytic subunit	94	3	1.00	13	11	20
G1SFF5	O15270	SPTLC2	Aminotran_1_2 domain-containing protein	Serine palmitoyltransferase 2	98	2	1.00	3	5	8
G1TRV7		QPCTL	Glutaminyl-peptide cyclotransferase like			1	0.99	6	6	32
G1SPB2		RNMT	mRNA cap guanine-N7 methyltransferase			1	0.99	4	4	11

G1SXI1		TTC27	TPR_REGION domain-containing protein			1	0.99	3	3	7
G1SJ72		CSPG4	Chondroitin sulfate proteoglycan 4			1	0.99	19	19	17
G1T3E6	A0A024R442	DNPEP	Uncharacterized protein	Aspartyl aminopeptidase	91	3	0.99	9	11	30
G1SLD5	A0A087X054	HYOU1	Hypoxia up-regulated 1	Hypoxia up-regulated protein 1	87	2	0.99	29	45	38
G1SZI6	Q86X10	RALGAPB	Rap-GAP domain-containing protein	Ral GTPase-activating protein subunit beta	97	2	0.99	4	2	3
G1SVJ8	Q96Q05	TRAPPC9	Uncharacterized protein	Trafficking protein particle complex subunit 9	87	3	0.99	2	2	2
G1SMG5	P15170	GSPT1	Tr-type G domain-containing protein	Eukaryotic peptide chain release factor GTP-binding subunit ERF3A	99	2	0.99	11	16	25
G1SUK4		MPI	Mannose-6-phosphate isomerase			1	0.99	3	2	12
G1TJS2	Q8IV08	PLD3	Phospholipase D family member 3	Phospholipase D3	89	2	0.99	5	2	18
G1TE39	Q9NYU1	UGGT2	Uncharacterized protein	UDP-glucose:glycoprotein glucosyltransferase 2	84	3	0.99	22	25	22
G1TPN2	K7EQA9	CDC37	Cell division cycle 37	Hsp90 co-chaperone Cdc37 (Fragment)	57	2	0.99	5	2	17
G1SCI5	F5H6E2	MYO1C	Uncharacterized protein	Unconventional myosin-Ic	92	3	0.99	40	100	46
G1SWN7	Q4G0N4	NADK2	NAD kinase 2, mitochondrial	NAD kinase 2, mitochondrial	92	2	0.99	5	5	14
G1T0H0	Q9NX46	ADPRHL2	ADP-ribosylhydrolase like 2	ADP-ribose glycohydrolase ARH3	95	2	0.99	3	3	12
	O94973	AP2A2		AP-2 complex subunit alpha-2		4	0.99	27	31	42
G1T6N8		ALG12	Mannosyltransferase			1	0.99	2	2	9
G1SI85	A0A0A0MS41	SFXN3	Uncharacterized protein	Sidoreflexin	94	3	0.99	13	17	39
G1T2I4	P07814	EPRS	Glutamyl-prolyl-tRNA synthetase	Bifunctional glutamate/proline--tRNA ligase	89	2	0.99	54	67	46
G1U1E6		GCSH	Glycine cleavage system H protein			1	0.99	2	4	33
G1SLU5	Q9UKZ1	CNOT11	CCR4-NOT transcription complex subunit 11	CCR4-NOT transcription complex subunit 11	79	2	0.99	2	2	6
P63169	F8VRV5	DYNLL1	Dynein light chain 1, cytoplasmic	Dynein light chain	100	2	0.99	2	11	43
G1SVW7	G3V1U5	GOLT1B	Uncharacterized protein	Golgi transport 1 homolog B (S. cerevisiae), isoform CRA_c	100	3	0.99	3	26	17
G1T534	E9PDM8	SEC24D	SEC24 homolog D, COPII coat complex component	Protein transport protein Sec24D	92	2	0.99	8	2	8
G1SQD1	E7EUU4	EIF4G1	Eukaryotic translation initiation factor 4 gamma 1	Eukaryotic translation initiation factor 4 gamma 1	94	2	0.99	21	33	15
G1SHS8	A0A087WY55	VTA1	Uncharacterized protein	Chromosome 6 open reading frame 55, isoform CRA_b	87	3	0.99	3	5	15
G1SJG0	P28300	LOX	Uncharacterized protein	Protein-lysine 6-oxidase	88	3	0.98	4	5	14
G1TXN1		NIT2	CN hydrolase domain-containing protein			1	0.98	3	3	14
G1T6B3	Q9Y3F4	STRAP	WD_REPEATS_REGION domain-containing protein	Serine-threonine kinase receptor-associated protein	98	2	0.98	13	33	52
G1SII9	Q5QJ74	TBCEL	Ubiquitin-like domain-containing protein	Tubulin-specific chaperone cofactor E-like protein	99	2	0.98	2	2	8
G1SP97	P51884	LUM	Lumican	Lumican	91	2	0.98	4	7	19
G1SWD9	B4DKY1	CARS	Cysteinyl-tRNA synthetase	Cysteine--tRNA ligase, cytoplasmic	68	2	0.98	12	23	20
G1T2F5	Q86X52	CHSY1	Hexosyltransferase	Chondroitin sulfate synthase 1	93	2	0.98	3	2	7
G1SV40	P83436	COG7	Uncharacterized protein	Conserved oligomeric Golgi complex subunit 7	93	3	0.98	5	8	10
G1SE01	A0A0A0MSI8	EXOC5	Exocyst complex component 5	Exocyst complex component 5	98	2	0.98	4	4	7
G1T373	B1ALA9	PRPS1	Pribosyltran_N domain-containing protein	Ribose-phosphate pyrophosphokinase 1	88	2	0.98	4	13	19
G1SQK0	A0A087X1A5	STAU1	Uncharacterized protein	Double-stranded RNA-binding protein Staufen homolog 1	89	3	0.98	2	2	4
G1U6H0	P27824	CANX	Uncharacterized protein	Calnexin	95	3	0.98	16	49	30
G1T5R0		TUBGCP3	Gamma-tubulin complex component			1	0.98	2	2	5
G1T332		GOT1	Aspartate aminotransferase			1	0.98	3	4	10
G1U522	P13861	PRKAR2A	Uncharacterized protein	cAMP-dependent protein kinase type II-alpha regulatory subunit	90	3	0.98	10	10	34
G1SFQ7		TUBG1	Tubulin gamma chain			1	0.98	5	7	17
B7NZS0		MYADM	Myeloid-associated differentiation marker (Predicted)			1	0.98	2	9	10
G1SCU8		OTUD6B	OTU domain-containing protein			1	0.98	3	3	19
G1TZQ5	G5EA31	SEC24C	Uncharacterized protein	Protein transport protein Sec24C	93	3	0.98	6	9	10
G1U207	Q86Y82	STX12	t-SNARE coiled-coil homology domain-containing protein	Syntaxin-12	96	2	0.98	10	16	55
G1SP48	O60701	UGDH	UDP-glucose 6-dehydrogenase	UDP-glucose 6-dehydrogenase	96	2	0.98	17	4	51
G1SHM2		CLPTM1L	CLPTM1 like			1	0.98	5	5	22
G1U5Z2		TK2	dNK domain-containing protein			1	0.98	2	2	11
Q9TT13	Q9Y277	VDAC3	Voltage-dependent anion-selective channel protein 3	Voltage-dependent anion-selective channel protein 3	98	2	0.98	12	30	54
G1SVY8	P12277	CKB	Creatine kinase B-type	Creatine kinase B-type	97	2	0.98	19	211	70
G1SPI7	Q92538	GBF1	SEC7 domain-containing protein	Golgi-specific brefeldin A-resistance guanine nucleotide exchange factor 1	96	2	0.98	18	20	14
G1TAP1	Q15181	PPA1	Uncharacterized protein	Inorganic pyrophosphatase	96	3	0.98	8	13	48
G1TIT4		PTTG1IP	PTTG1 interacting protein			1	0.98	2	3	11
G1STX4	P61011	SRP54	Signal recognition particle 54 kDa protein	Signal recognition particle 54 kDa protein	99	2	0.98	13	15	38
G1SRQ2	A0A087WTB8	UCHL3	Ubiquitin carboxyl-terminal hydrolase	Ubiquitin carboxyl-terminal hydrolase	98	2	0.98	3	4	21
G1SYA5	X6RA14	ESD	S-formylglutathione hydrolase	S-formylglutathione hydrolase	88	2	0.98	8	16	45
G1SHH1		PPOX	Protoporphyrinogen oxidase			1	0.98	2	2	8

G1TA41	Q7L7X3	TAOK1	Protein kinase domain-containing protein	Serine/threonine-protein kinase TAO1	100	2	0.98	4	3	6
G1T4H6		MTMR6	Myotubularin phosphatase domain-containing protein			1	0.97	3	2	4
G1SFH8		PDCL3	Phosducin domain-containing protein			1	0.97	2	3	11
	P37837	TALDO1		Transaldolase		4	0.97	3	6	11
G1SJR4	Q15363	TMED2	Transmembrane p24 trafficking protein 2	Transmembrane emp24 domain-containing protein 2	99	2	0.97	8	31	64
G1SNL4	Q9Y385	UBE2J1	UBIQUITIN_CONJUGAT_2 domain-containing protein	Ubiquitin-conjugating enzyme E2 J1	94	2	0.97	3	4	14
G1T7U4	Q9Y4E8	USP15	Ubiquitin carboxyl-terminal hydrolase	Ubiquitin carboxyl-terminal hydrolase 15	99	2	0.97	4	3	7
G1TDF6	O75955	FLOT1	PHB domain-containing protein	Flotillin-1	99	2	0.97	13	21	40
	Q9UNE7	STUB1		E3 ubiquitin-protein ligase CHIP		4	0.97	2	2	9
G1SES9	P31939	ATIC	MGS domain-containing protein	Bifunctional purine biosynthesis protein PURH	94	2	0.97	17	30	44
G1TSV3	Q8ND76	CCNY	Cyclin Y	Cyclin-Y	99	2	0.97	2	3	9
G1SQG5	P40925	MDH1	Malate dehydrogenase	Malate dehydrogenase, cytoplasmic	97	2	0.97	12	16	57
G1TNW8	Q9Y512	SAMM50	SAMM50 sorting and assembly machinery component	Sorting and assembly machinery component 50 homolog	96	2	0.97	9	9	35
G1SKG9	A0A087X2H1	HECTD1	Uncharacterized protein	E3 ubiquitin-protein ligase HECTD1	99	3	0.97	7	10	4
G1T7T0	Q9Y2G5	POFUT2	Protein O-fucosyltransferase 2	GDP-fucose protein O-fucosyltransferase 2	84	2	0.97	3	3	13
G1TE37		ATP6AP1	ATPase H+ transporting accessory protein 1			1	0.97	2	2	10
	O95747	OXSR1		Serine/threonine-protein kinase OSR1		4	0.97	4	7	12
G1SQ90	A0A2R8Y5H3	COL4A3BP	Collagen type IV alpha 3 binding protein	Collagen type IV alpha-3-binding protein (Fragment)	96	2	0.97	2	2	6
G1SGN0	Q15042	RAB3GAP1	Uncharacterized protein	Rab3 GTPase-activating protein catalytic subunit	94	3	0.97	10	9	17
G1SSN9	Q96S59	RANBP9	Uncharacterized protein	Ran-binding protein 9	97	3	0.97	4	4	15
G1SCE6	Q9Y223	GNE	Epimerase_2 domain-containing protein	Bifunctional UDP-N-acetylglucosamine 2-epimerase/N-acetylmannosamine kinase	100	2	0.97	10	25	27
G1SLD6	B3KWE1	HARS	Uncharacterized protein	Histidine--tRNA ligase, cytoplasmic	97	3	0.97	7	8	19
G1SMG1		KANK1	KN motif and ankyrin repeat domains 1			1	0.97	6	5	10
G1SR15		CD109	CD109 molecule			1	0.97	2	3	2
P00169	P00167	CYB5A	Cytochrome b5	Cytochrome b5	90	2	0.97	7	13	80
G1TE27	X6R9L0	DNAJC3	Uncharacterized protein	DnaJ homolog subfamily C member 3	88	3	0.97	13	4	33
G1TDJ3	Q9BSJ8	ESYT1	Uncharacterized protein	Extended synaptotagmin-1	89	3	0.97	30	43	41
G1SL52	A0A0A0MS45	COG4	Cog4 domain-containing protein	Conserved oligomeric Golgi complex subunit 4	94	2	0.96	10	10	19
G1U0Q2	K7EJ78	RPS15	Uncharacterized protein	40S ribosomal protein S15	99	3	0.96	2	14	28
G1U4C2	Q76M96	CCDC80	Uncharacterized protein	Coiled-coil domain-containing protein 80	86	3	0.96	5	4	6
G1T2G6	Q9P265	DIP2B	DMAP-interaction domain-containing protein	Disco-interacting protein 2 homolog B	99	2	0.96	2	2	2
G1SQJ2		FOCAD	DUF3730 domain-containing protein			1	0.96	8	8	7
G1SKM5	Q8TAT6	NPLOC4	NPL4 homolog, ubiquitin recognition factor	Nuclear protein localization protein 4 homolog	92	2	0.96	5	5	12
G1TTU6	E7ERH2	SKP1	Uncharacterized protein	S-phase kinase-associated protein 1 (Fragment)	100	3	0.96	6	55	56
G1TCP2	A0A087X1E4	ARFIP2	AH domain-containing protein	Arfaptin-2	91	2	0.96	2	5	9
	V9GYD0	ARL2-SNX15		ARL2-SNX15 readthrough (NMD candidate)		4	0.96	3	5	32
G1T6P5	Q9NVJ2	ARL8B	ADP ribosylation factor like GTPase 8B	ADP-ribosylation factor-like protein 8B	100	2	0.96	7	31	45
G1TI39	P35241	RDX	FERM domain-containing protein	Radixin	99	2	0.96	17	16	31
G1T5H7		TMEM132B	Transmembrane protein 132B			1	0.96	2	2	4
G1SIJ2	P24752	ACAT1	Uncharacterized protein	Acetyl-CoA acetyltransferase, mitochondrial	92	3	0.96	13	21	47
G1TC19		CPT2	Carnitine palmitoyltransferase 2			1	0.96	2	2	5
G1TD24	Q9NRY4	ARHGAP35	Rho GTPase activating protein 35	Rho GTPase-activating protein 35	98	2	0.96	4	4	3
G1TEY6	O00178	GTPBP1	Tr-type G domain-containing protein	GTP-binding protein 1	94	2	0.96	5	6	12
G1TBW2	E7EQB8	IDH3G	Isocitrate dehydrogenase [NAD] subunit, mitochondrial	Isocitrate dehydrogenase [NAD] subunit, mitochondrial	94	2	0.96	6	10	27
G1TCY1	Q16513	PKN2	Uncharacterized protein	Serine/threonine-protein kinase N2	97	3	0.96	4	3	4
G1SEK1	P62140	PPP1CB	Serine/threonine-protein phosphatase	Serine/threonine-protein phosphatase PP1-beta catalytic subunit	100	2	0.96	9	3	35
G1U9D3	Q9UK41	VPS28	Vacuolar protein sorting-associated protein 28 homolog	Vacuolar protein sorting-associated protein 28 homolog	93	2	0.96	4	4	29
P0CL18		EIF2D	Eukaryotic translation initiation factor 2D			1	0.96	2	3	8
	O43765	SGTA		Small glutamine-rich tetratricopeptide repeat-containing protein alpha		4	0.96	2	3	8
G1T235	P28072	PSMB6	Proteasome subunit beta	Proteasome subunit beta type-6	97	2	0.96	6	10	40
	Q9Y224	RTRAF		RNA transcription, translation and transport factor protein		4	0.96	10	41	50
G1SXT1	O43865	AHCYL1	AdoHcyase_NAD domain-containing protein	S-adenosylhomocysteine hydrolase-like protein 1	100	2	0.96	3	6	7
G1TE47		COG8	Conserved oligomeric Golgi complex subunit 8			1	0.96	5	4	12
P43236	P43235	CTSK	Cathepsin K	Cathepsin K	94	2	0.95	5	11	22
G1U101	J3KPF0	HECTD4	HECT domain-containing protein	Probable E3 ubiquitin-protein ligase HECTD4	97	2	0.95	2	5	1
G1TBC1	P14625	HSP90B1	Endoplasmin	Endoplasmin	94	2	0.95	43	877	55
G1SZ14	P25788	PSMA3	Proteasome endopeptidase complex	Proteasome subunit alpha type-3	99	2	0.95	8	19	26

G1SJH8	K7EP90	RBM42	RRM domain-containing protein	RNA-binding protein 42	97	2	0.95	2	2	8
G1SFC4		LMF2	Lipase maturation factor			1	0.95	3	5	7
	P54725	RAD23A		UV excision repair protein RAD23 homolog A		4	0.95	5	12	23
G1T365	Q9H269	VPS16	Vacuolar protein sorting-associated protein 16 homolog	Vacuolar protein sorting-associated protein 16 homolog	98	2	0.95	6	9	15
G1TA82	Q12768	WASHC5	Uncharacterized protein	WASH complex subunit 5	97	3	0.95	6	11	9
G1SYS5	A0A1B0GW77	ALDH7A1	Aldedh domain-containing protein	Alpha-aminoadipic semialdehyde dehydrogenase	93	2	0.95	9	30	30
G1U1Q1		THBS2	Thrombospondin 2			1	0.95	5	6	8
G1SRD2	P28330	ACADL	Uncharacterized protein	Long-chain specific acyl-CoA dehydrogenase, mitochondrial	82	3	0.95	5	82	17
G1SM15	A0A087WY71	AP2M1	MHD domain-containing protein	AP-2 complex subunit mu	100	2	0.95	15	24	42
G1TZ26		GUK1	Guanylate kinase 1			1	0.95	3	6	24
G1T364	Q8NDH3	NPEPL1	Aminopeptidase like 1	Probable aminopeptidase NPEPL1	90	2	0.95	3	5	9
G1SVD7		SYNJ2BP-COX16	PDZ domain-containing protein			1	0.95	2	2	15
G1TIA2		RIOX1	JmjC domain-containing protein			1	0.95	3	4	10
G1SMZ8	Q96JJ7	TMX3	Thioredoxin domain-containing protein	Protein disulfide-isomerase TMX3	92	2	0.95	8	8	28
G1T918	P28070	PSMB4	Proteasome subunit beta	Proteasome subunit beta type-4	95	2	0.95	8	37	49
G1T748	Q5VZU9	TPP2	Uncharacterized protein	Tripeptidyl-peptidase 2	97	3	0.95	26	31	25
G1SWY6	Q15075	EEA1	Early endosome antigen 1	Early endosome antigen 1	87	2	0.95	32	20	28
G1SYF9	Q9Y613	FHOD1	Uncharacterized protein	FH1/FH2 domain-containing protein 1	87	3	0.95	5	5	9
G1SWE5		WDR61	WD repeat domain 61			1	0.95	2	3	12
Q9GLC3		ATP1B3	Sodium/potassium-transporting ATPase subunit beta-3			1	0.95	3	4	15
G1SXX5	A0A2R8Y4F5	HADHA	Uncharacterized protein	Trifunctional enzyme subunit alpha, mitochondrial	86	3	0.95	30	150	52
G1SIE8	A0A3B3ISG5	IDE	Uncharacterized protein	Insulin-degrading enzyme	96	3	0.95	7	9	9
O19048	Q15365	PCBP1	Poly(rC)-binding protein 1	Poly(rC)-binding protein 1	100	2	0.95	14	26	64
	P30533	LRPAP1		Alpha-2-macroglobulin receptor-associated protein		4	0.95	2	4	5
G1SNQ9	A0A0A0MTJ9	NCEH1	Uncharacterized protein	Neutral cholesterol ester hydrolase 1	88	3	0.95	9	9	32
G1T0K1	Q9BTV4	TMEM43	Uncharacterized protein	Transmembrane protein 43	92	3	0.95	10	18	39
G1U3B8	K7ER96	TXNL1	PITH domain-containing protein	Thioredoxin-like protein 1 (Fragment)	100	2	0.95	5	4	30
G1T994	D6RGG3	COL12A1	Collagen alpha-1(XII) chain	Collagen alpha-1(XII) chain	95	2	0.94	117	647	55
G1T0T5	F5GYF7	COPS7A	PCI domain-containing protein	COP9 signalosome complex subunit 7a (Fragment)	100	2	0.94	2	4	10
G1T0N5	Q00577	PURA	Purine rich element binding protein A	Transcriptional activator protein Pur-alpha	91	2	0.94	7	10	40
P13642	P49591	SARS	Serine--tRNA ligase, cytoplasmic	Serine--tRNA ligase, cytoplasmic	97	2	0.94	14	25	32
G1T4S5	Q8NFW8	CMAS	Uncharacterized protein	N-acylneuraminate cytidylyltransferase	96	3	0.94	5	5	11
	F5H039	GPHN		Molybdopterin molybdenumtransferase		4	0.94	2	3	5
G1TX94	A0A0R4J2E8	MATR3	Uncharacterized protein	Matrin-3	99	3	0.94	17	31	28
G1T5I3	A0A0C4DFM1	TM9SF4	Transmembrane 9 superfamily member	Transmembrane 9 superfamily member	100	2	0.94	4	6	10
G1T7F5	A0A1C7CYY0	ADD2	Aldolase_II domain-containing protein	Beta-adducin (Fragment)	93	2	0.94	2	2	8
G1U3V0	Q9BX68	HINT2	HIT domain-containing protein	Histidine triad nucleotide-binding protein 2, mitochondrial	94	2	0.94	4	6	41
G1U0T4		HSD17B14	Hydroxysteroid 17-beta dehydrogenase 14			1	0.94	2	3	9
G1TUD6	P43686	PSMC4	Proteasome 26S subunit, ATPase 4	26S proteasome regulatory subunit 6B	100	2	0.94	12	51	34
P30801		S100A6	Protein S100-A6			1	0.94	7	22	56
G1U2R1	Q99805	TM9SF2	Transmembrane 9 superfamily member	Transmembrane 9 superfamily member 2	93	2	0.94	6	22	16
G1T0R4	Q96JC1	VPS39	CNH domain-containing protein	Vam6/Vps39-like protein	97	2	0.94	3	3	5
G1SGD9		ATG7	Ubiquitin-like modifier-activating enzyme ATG7			1	0.94	2	2	6
G1T4P8	O76003	GLRX3	Glutaredoxin 3	Glutaredoxin-3	93	2	0.94	10	17	46
G1SUM3	Q8TBC4	UBA3	E2_bind domain-containing protein	NEDD8-activating enzyme E1 catalytic subunit	99	2	0.94	5	6	21
G1SDM6	O75165	DNAJC13	J domain-containing protein	DnaJ homolog subfamily C member 13	98	2	0.94	27	33	17
	A0A024RBG1	NUDT4B		Diphosphoinositol polyphosphate phosphohydrolase NUDT4B		4	0.94	2	2	21
G1SWC9	H3BMU1	IST1	Uncharacterized protein	IST1 homolog (Fragment)	100	3	0.94	5	7	21
G1SSV2	Q9UL01	DSE	DUF4962 domain-containing protein	Dermatan-sulfate epimerase	96	2	0.94	8	8	15
G1SMP6	A0A1B0GVV3	RILPL1	Rab interacting lysosomal protein like 1	RILP-like protein 1	93	2	0.94	4	5	16
G1T8P7	A0A2R8YFH5	SEC23B	Protein transport protein SEC23	Protein transport protein SEC23	95	2	0.94	8	8	15
G1T7L7		KDELC2	CAP10 domain-containing protein			1	0.94	10	14	25
G1T725	Q8TB40	ABHD4	AB hydrolase-1 domain-containing protein	(Lyso)-N-acylphosphatidylethanolamine lipase	97	2	0.94	2	3	8
G1SVH0	P23526	AHCY	AdoHcyase_NAD domain-containing protein	Adenosylhomocysteinase	97	2	0.94	15	41	36
	M0QYZ2	AP2S1		AP complex subunit sigma		4	0.94	5	12	33
G1T8Y0		COASY	CTP_transf_like domain-containing protein			1	0.94	2	2	5
	Q9UBF2	COPG2		Coatomer subunit gamma-2		4	0.93	12	4	18

G1TC33	P35580	MYH10	Uncharacterized protein	Myosin-10	99	3	0.93	102	297	54
P63150	P63151	PPP2R2A	Serine/threonine-protein phosphatase 2A 55 kDa regulatory subunit B alpha isoform	Serine/threonine-protein phosphatase 2A 55 kDa regulatory subunit B alpha isoform	100	2	0.93	4	7	15
G1SER3	Q02978	SLC25A11	Uncharacterized protein	Mitochondrial 2-oxoglutarate/malate carrier protein	97	3	0.93	8	56	37
G1T276		ALDH3A2	Aldehyde dehydrogenase			1	0.93	7	10	21
G1SJU2	O60306	AQR	RNA helicase aquarius	RNA helicase aquarius	96	2	0.93	3	2	4
G1T7I0	Q96PU5	NEDD4L	E3 ubiquitin-protein ligase	E3 ubiquitin-protein ligase NEDD4-like	96	2	0.93	3	3	5
G1SM51	Q9UNM6	PSMD13	Proteasome 26S subunit, non-ATPase 13	26S proteasome non-ATPase regulatory subunit 13	92	2	0.93	15	8	46
G1SUT8	O95486	SEC24A	Uncharacterized protein	Protein transport protein Sec24A	93	3	0.93	5	7	7
G1SHZ2	Q86X83	COMMD2	COMM domain-containing protein	COMM domain-containing protein 2	97	2	0.93	2	4	17
G1U4Y5		FCGRT	Fc fragment of IgG receptor and transporter			1	0.93	2	2	7
G1SG37	G5E9T8	GOSR1	Golgi SNAP receptor complex member 1	Golgi SNAP receptor complex member 1 (Fragment)	99	2	0.93	3	6	20
B7NZD2	Q5VWC4	PSMD4	Proteasome 26S subunit, non-ATPase, 4 (Predicted)	26S proteasome non-ATPase regulatory subunit 4	99	2	0.93	8	16	27
	A0A087X1G7	SELENOF		Selenoprotein F		4	0.93	3	14	22
G1T466	A0AVT1	UBA6	UBA_e1_C domain-containing protein	Ubiquitin-like modifier-activating enzyme 6	93	2	0.93	7	5	10
G1SFG8	A0A096LNH6	DOCK1	Uncharacterized protein	Dedicator of cytokinesis protein 1	95	3	0.93	4	5	4
G1T3A6	A0A1W2PQH3	ME2	Malic enzyme	Malic enzyme	92	2	0.93	16	14	40
G1T089		THUMPD3	THUMP domain containing 3			1	0.93	3	2	9
G1SZW0	Q7L576	CYFIP1	Cytoplasmic FMR1-interacting protein	Cytoplasmic FMR1-interacting protein 1	98	2	0.93	18	31	17
G1SCT9	A0A087WU53	MAGT1	Uncharacterized protein	Magnesium transporter protein 1	97	3	0.93	7	9	24
G1SY19	O15118	NPC1	SSD domain-containing protein	NPC intracellular cholesterol transporter 1	91	2	0.93	3	4	3
G1SKF1	P07996	THBS1	Uncharacterized protein	Thrombospondin-1	97	3	0.93	34	69	37
G1SXL9		XPNPEP3	AMP_N domain-containing protein			1	0.93	2	2	9
G1U8J5		ATP5PF	ATP synthase-coupling factor 6, mitochondrial			1	0.93	3	3	33
G1T1D9	Q92905	COPS5	MPN domain-containing protein	COP9 signalosome complex subunit 5	100	2	0.93	7	9	31
G1SSV1	Q99627	COPS8	PCI domain-containing protein	COP9 signalosome complex subunit 8	99	2	0.93	4	11	38
G1SRI8	P19367	HK1	Uncharacterized protein	Hexokinase-1	96	3	0.93	27	49	33
Q28740		BSG	Basigin			1	0.93	2	4	10
	P63241	EIF5A		Eukaryotic translation initiation factor 5A-1		4	0.93	9	218	66
G1SP36	Q9HAV4	XPO5	Importin N-terminal domain-containing protein	Exportin-5	95	2	0.93	3	6	7
G1T312		ACAD8	Acyl-CoA dehydrogenase family member 8			1	0.93	2	2	5
G1TK72	F8W8H5	RAB24	Uncharacterized protein	Ras-related protein Rab-24	98	3	0.93	2	3	13
G1T302		THYN1	EVE domain-containing protein			1	0.92	2	7	10
G1TBJ8		RB1CC1	RB1 inducible coiled-coil 1			1	0.92	3	2	3
	Q9Y6I3	EPN1		Epsin-1		4	0.92	2	3	4
G1T7Q2		LOXL2	Lysyl oxidase like 2			1	0.92	6	5	9
	A0A0A0MSZ1	MARK3		Non-specific serine/threonine protein kinase		4	0.92	2	2	4
G1SL80		UROD	Uroporphyrinogen decarboxylase			1	0.92	4	4	19
G1TQ79		CHCHD6	MICOS complex subunit			1	0.92	3	3	15
G1T974	A0A3B3IRK6	MOGS	Mannosyl-oligosaccharide glucosidase	Mannosyl-oligosaccharide glucosidase (Fragment)	86	2	0.92	9	12	20
G1SRF5	Q9UPN7	PPP6R1	Protein phosphatase 6 regulatory subunit 1	Serine/threonine-protein phosphatase 6 regulatory subunit 1	84	2	0.92	4	3	9
G1SJS2	Q14232	EIF2B1	Uncharacterized protein	Translation initiation factor eIF-2B subunit alpha	96	3	0.92	4	12	31
G1TYK8	Q13492	PICALM	Phosphatidylinositol binding clathrin assembly protein	Phosphatidylinositol-binding clathrin assembly protein	97	2	0.92	12	31	27
G1T4Q9	P28074	PSMB5	Proteasome subunit beta	Proteasome subunit beta type-5	98	2	0.92	9	50	47
G1T4K5	A0A1B0GTW1	TJP2	Uncharacterized protein	Tight junction protein ZO-2	87	3	0.92	5	4	8
G1SVA1	F2Z2X4	XPO4	CRM1_C domain-containing protein	Exportin-4	99	2	0.92	5	4	10
G1SZP4	I6L894	ANK2	Ankyrin 2	Ankyrin-2	88	2	0.92	3	3	1
G1SYR9	B9A067	IMMT	MICOS complex subunit MIC60	MICOS complex subunit MIC60	89	2	0.92	31	55	50
U3KMD4	Q8IYU8	MICU2	Uncharacterized protein	Calcium uptake protein 2, mitochondrial	83	3	0.92	5	3	25
G1SPV0	P61086	UBE2K	Uncharacterized protein	Ubiquitin-conjugating enzyme E2 K	100	3	0.92	4	5	35
G1T0Z6	Q9Y4X5	ARIH1	RBR-type E3 ubiquitin transferase	E3 ubiquitin-protein ligase ARIH1	98	2	0.92	3	2	10
G1SST9	P54727	RAD23B	Uncharacterized protein	UV excision repair protein RAD23 homolog B	94	3	0.92	12	15	42
G1SJ41	Q9Y6Y8	SEC23IP	DDHD domain-containing protein	SEC23-interacting protein	89	2	0.92	14	21	16
G1TC10	P61081	UBE2M	UBIQUITIN_CONJUGAT_2 domain-containing protein	NEDD8-conjugating enzyme Ubc12	100	2	0.92	7	7	51
G1SUV0	Q9NW15	ANO10	Anoctamin	Anoctamin-10	94	2	0.92	5	12	12
G1SCQ0		EDEM3	alpha-1,2-Mannosidase			1	0.92	2	2	4
G1T887		GPR107	G protein-coupled receptor 107			1	0.92	2	4	9
G1SU71	P20618	PSMB1	Proteasome subunit beta	Proteasome subunit beta type-1	94	2	0.92	8	12	42

G1TJX7	P50552	VASP	Vasodilator stimulated phosphoprotein	Vasodilator-stimulated phosphoprotein	87	2	0.92	2	5	6
G1T2Z5	A0A087WTA8	COL1A2	Collagen alpha-2(I) chain	Collagen alpha-2(I) chain	94	2	0.92	28	176	33
G1SK00		USP5	Ubiquitin carboxyl-terminal hydrolase			1	0.92	21	38	38
G1TM00	O75436	VPS26A	VPS26, retromer complex component A	Vacuolar protein sorting-associated protein 26A	99	2	0.92	2	2	10
G1SCE7	P55084	HADHB	Uncharacterized protein	Trifunctional enzyme subunit beta, mitochondrial	94	3	0.91	14	16	42
G1SHV9	P49720	PSMB3	Proteasome subunit beta	Proteasome subunit beta type-3	99	2	0.91	6	15	39
G1U4H9	O14907	TAX1BP3	Tax1-binding protein 3	Tax1-binding protein 3	100	2	0.91	2	4	28
P15541		ANPEP	Aminopeptidase N			1	0.91	11	15	19
G1SG59	Q7L9L4	MOB1B	Uncharacterized protein	MOB kinase activator 1B	100	3	0.91	2	6	15
G1TY83		NLRX1	NLR family member X1			1	0.91	3	3	6
G1SWN1	Q96H20	SNF8	Vacuolar-sorting protein SNF8	Vacuolar-sorting protein SNF8	100	2	0.91	3	3	24
G1SH25	P40616	ARL1	Uncharacterized protein	ADP-ribosylation factor-like protein 1	99	3	0.91	5	16	38
G1SW65		ATP5MC2	ATP-synt_C domain-containing protein			1	0.91	2	2	21
P41982	P04179	SOD2	Superoxide dismutase [Mn], mitochondrial (Fragment)	Superoxide dismutase [Mn], mitochondrial	92	2	0.91	4	22	25
P68105	P68104	EEF1A1	Elongation factor 1-alpha 1	Elongation factor 1-alpha 1	100	2	0.91	22	823	65
Q28717	F6WIT2	PTPA	Serine/threonine-protein phosphatase 2A activator	Serine/threonine-protein phosphatase 2A activator	97	2	0.91	8	13	41
G1SJV2	Q7L523	RRAGA	Uncharacterized protein	Ras-related GTP-binding protein A	100	3	0.91	4	8	18
G1T9H0	Q8NCA5	FAM98A	Uncharacterized protein	Protein FAM98A	92	3	0.91	8	12	22
	A0A087X0W8	RELA		Transcription factor p65		4	0.91	2	2	7
G1T8T0		FRMD6	FERM domain-containing protein			1	0.91	2	2	4
G1T2R2	A0A3B3ITU8	ITPR1	Uncharacterized protein	Inositol 1,4,5-trisphosphate receptor type 1	97	3	0.91	4	2	2
G1TXS2	A0A0D9SFK2	MYO18A	Uncharacterized protein	Unconventional myosin-XVIIIa	94	3	0.91	10	9	7
G1SVG6	P21359	NF1	Uncharacterized protein	Neurofibromin	99	3	0.91	4	6	3
U3KN73	Q9UBQ0	VPS29	Vacuolar protein sorting-associated protein 29	Vacuolar protein sorting-associated protein 29	100	2	0.91	6	8	37
G1TA78	P23381	WARS	Tryptophan--tRNA ligase, cytoplasmic	Tryptophan--tRNA ligase, cytoplasmic	89	2	0.91	11	25	31
G1SDD2	B7Z2Y2	COG2	Uncharacterized protein	Conserved oligomeric Golgi complex subunit 2	91	3	0.91	6	10	15
G1SY96	Q9UJS0	SLC25A13	Uncharacterized protein	Calcium-binding mitochondrial carrier protein Aralar2	97	3	0.91	11	4	28
G1TYL5		DNPH1	2~-deoxynucleoside 5~-phosphate N-hydrolase 1			1	0.91	2	2	31
G1TIW9		FBXO6	F-box protein 6			1	0.91	3	3	11
G1SPV8	O94829	IPO13	Importin N-terminal domain-containing protein	Importin-13	100	2	0.91	2	2	3
G1SCZ9	Q92575	UBXN4	UBX domain-containing protein	UBX domain-containing protein 4	94	2	0.91	4	6	14
G1SJ61	Q9Y608	LRRFIP2	Uncharacterized protein	Leucine-rich repeat flightless-interacting protein 2	91	3	0.90	7	7	13
P40826	P54578	USP14	Ubiquitin carboxyl-terminal hydrolase 14	Ubiquitin carboxyl-terminal hydrolase 14	97	2	0.90	10	17	29
G1T9V6	Q9UPU5	USP24	Ubiquitin specific peptidase 24	Ubiquitin carboxyl-terminal hydrolase 24	98	2	0.90	7	6	5
G1T8X7	Q96JH7	VCPIP1	OTU domain-containing protein	Deubiquitinating protein VCIP135	96	2	0.90	2	3	2
G1T5X6		HMGCL	Pyruvate carboxyltransferase domain-containing protein			1	0.90	2	3	11
G1SJB9	Q14257	RCN2	Reticulocalbin 2	Reticulocalbin-2	90	2	0.90	12	131	56
G1SMM5	O60884	DNAJA2	Uncharacterized protein	DnaJ homolog subfamily A member 2	100	3	0.90	6	11	29
G1SQM7	P53999	SUB1	PC4 domain-containing protein	Activated RNA polymerase II transcriptional coactivator p15	97	2	0.90	4	7	22
G1SCF0	P13807	GYS1	Glycogen [starch] synthase	Glycogen [starch] synthase, muscle	96	2	0.90	3	4	6
G1TE96	O43813	LANCL1	Uncharacterized protein	Glutathione S-transferase LANCL1	95	3	0.90	4	9	18
G1TQD3	Q01968	OCRL	Rho-GAP domain-containing protein	Inositol polyphosphate 5-phosphatase OCRL	95	2	0.90	3	2	5
G1T8K2	A0A0U1RR22	PACSIN2	Uncharacterized protein	Protein kinase C and casein kinase substrate in neurons protein 2 (Fragment)	93	3	0.90	8	7	23
G1T8E2	A0A3B3ITW1	GSK3B	Protein kinase domain-containing protein	Glycogen synthase kinase-3 beta	87	2	0.90	2	2	10
G1TZV3	E7ENJ6	AP1M1	Adaptor related protein complex 1 subunit mu 1	AP-1 complex subunit mu-1	75	2	0.90	8	11	33
G1THI9	Q9UKB1	FBXW11	Uncharacterized protein	F-box/WD repeat-containing protein 11	96	3	0.90	2	2	7
	J3KR44	OTUB1		Ubiquitin thioesterase		4	0.90	5	7	34
G1U8P2	J3KTE4	RPL19	Ribosomal protein L19	Ribosomal protein L19	98	2	0.90	7	15	30
G1SWN4	Q9H832	UBE2Z	UBIQUITIN_CONJUGAT_2 domain-containing protein	Ubiquitin-conjugating enzyme E2 Z	96	2	0.90	3	4	11
U3KM64		CLTA	Clathrin light chain			1	0.90	7	26	28
	P62166	NCS1		Neuronal calcium sensor 1		4	0.90	2	2	24
G1TUY5		OCIAD1	OCIA domain-containing protein			1	0.90	3	4	15
G1SI71		PAIP1	MIF4G domain-containing protein			1	0.90	2	3	6
G1TUP1	O75340	PDCD6	Programmed cell death 6	Programmed cell death protein 6	99	2	0.90	5	14	29
G1SZ72	P51116	FXR2	Uncharacterized protein	Fragile X mental retardation syndrome-related protein 2	98	3	0.90	8	5	20
G1SML4	P28288	ABCD3	Uncharacterized protein	ATP-binding cassette sub-family D member 3	96	3	0.90	10	11	21
G1T0B0	Q9H3H3	C11orf68	Uncharacterized protein	UPF0696 protein C11orf68	93	3	0.90	2	3	13

	Q13363	CTBP1		C-terminal-binding protein 1		4	0.90	4	4	10
G1T369	C9J8R4	DCUN1D1	DCN1-like protein	DCN1-like protein (Fragment)	100	2	0.90	2	2	13
G1T5H8	E7EX17	EIF4B	RRM domain-containing protein	Eukaryotic translation initiation factor 4B	93	2	0.90	3	7	10
G1TT64	Q68EM7	ARHGAP17	Uncharacterized protein	Rho GTPase-activating protein 17	91	3	0.90	4	4	8
G1SZQ7	Q7L1Q6	BZW1	W2 domain-containing protein	Basic leucine zipper and W2 domain-containing protein 1	100	2	0.90	12	19	39
G1SZF9	O75976	CPD	Uncharacterized protein	Carboxypeptidase D	91	3	0.90	5	5	5
G1TTS1		FUNDC2	FUN14 domain containing 2			1	0.90	2	3	12
G1TET2	P13796	LCP1	Uncharacterized protein	Plastin-2	98	3	0.90	19	22	46
G1T4Q8	J3KNQ4	PARVA	Uncharacterized protein	Alpha-parvin	91	3	0.90	11	25	34
	A0A096LP07	GPS1		COP9 signalosome complex subunit 1		4	0.90	4	3	10
G1SRA9	A0A0A0MTH3	ILK	Uncharacterized protein	Integrin-linked protein kinase	93	3	0.90	11	17	27
G1T519	P25789	PSMA4	Proteasome subunit alpha type	Proteasome subunit alpha type-4	100	2	0.90	10	20	58
	P60468	SEC61B		Protein transport protein Sec61 subunit beta		4	0.89	3	5	52
G1TB71	Q14008	CKAP5	Uncharacterized protein	Cytoskeleton-associated protein 5	97	3	0.89	18	24	15
G1TDU0	P33176	KIF5B	Kinesin-like protein	Kinesin-1 heavy chain	99	2	0.89	28	6	41
G1TD47	Q8N3E9	PLCD3	Phosphoinositide phospholipase C	1-phosphatidylinositol 4,5-bisphosphate phosphodiesterase delta-3	88	2	0.89	4	5	9
G1SJ77	Q9Y696	CLIC4	Chloride intracellular channel protein	Chloride intracellular channel protein 4	100	2	0.89	11	35	58
B7NZJ1		CPNE1	Copine I, isoform 8 (Predicted)			1	0.89	3	3	11
	O14818	PSMA7		Proteasome subunit alpha type-7		4	0.89	9	19	44
G1TB18	Q07960	ARHGAP1	Uncharacterized protein	Rho GTPase-activating protein 1	95	3	0.89	5	7	16
G1T7H0	A0A1W2PPS1	HNRNPU	Uncharacterized protein	Heterogeneous nuclear ribonucleoprotein U	96	3	0.89	22	55	33
G1SQ02	A0A0A0MSI0	PRDX1	Thioredoxin domain-containing protein	Peroxiredoxin-1 (Fragment)	98	2	0.89	10	15	57
G1SQL0	P62191	PSMC1	AAA domain-containing protein	26S proteasome regulatory subunit 4	100	2	0.89	17	29	43
G1SCQ1		AKR7L	Aldo_ket_red domain-containing protein			1	0.89	7	8	35
G1T090	G3V180	DPP3	Dipeptidyl peptidase 3	Dipeptidyl peptidase 3	94	2	0.89	9	16	24
G1SZ37	P31937	HIBADH	3-hydroxyisobutyrate dehydrogenase	3-hydroxyisobutyrate dehydrogenase, mitochondrial	96	2	0.89	6	6	32
G1SEU9	Q9UHA4	LAMTOR3	Uncharacterized protein	Ragulator complex protein LAMTOR3	98	3	0.89	4	6	40
G1T8U2		GMDS	GDP-mannose 4,6-dehydratase			1	0.89	2	3	13
G1TPY7		SUMF2	FGE-sulfatase domain-containing protein			1	0.89	3	2	13
G1TEU8		TBCC	C-CAP/cofactor C-like domain-containing protein			1	0.89	2	2	7
G1SK48	K7ES02	BLMH	Bleomycin hydrolase	Bleomycin hydrolase (Fragment)	92	2	0.89	8	11	26
G1SNS3	Q6P4E1	CASC4	Uncharacterized protein	Protein CASC4	89	3	0.89	7	9	16
G1SFC5	Q86VS8	HOOK3	Calponin-homology (CH) domain-containing protein	Protein Hook homolog 3	99	2	0.89	14	26	22
G1SSV0	Q9H1E5	TMX4	Thioredoxin domain-containing protein	Thioredoxin-related transmembrane protein 4	84	2	0.89	3	5	12
G1T8H8	P08183	ABCB1	Uncharacterized protein	ATP-dependent translocase ABCB1	88	3	0.89	5	16	7
G1TDC3	Q14697	GANAB	Gal_mutarotas_2 domain-containing protein	Neutral alpha-glucosidase AB	91	2	0.89	30	229	46
G1TD98		GSR	Glutathione reductase			1	0.89	2	3	7
G1SK33	P05556	ITGB1	Integrin beta	Integrin beta-1	94	2	0.89	17	48	26
G1T3S4		MECR	PKS_ER domain-containing protein			1	0.89	5	7	34
G1SQW0	E9PIE4	MTCH2	Uncharacterized protein	Mitochondrial carrier homolog 2 (Fragment)	92	3	0.89	6	15	29
G1SFV1	A0A499FI48	PDIA4	Protein disulfide-isomerase	Protein disulfide-isomerase	91	2	0.89	29	377	49
G1TCZ8	P30153	PPP2R1A	Protein phosphatase 2 scaffold subunit Aalpha	Serine/threonine-protein phosphatase 2A 65 kDa regulatory subunit A alpha isoform	90	2	0.89	15	3	37
G1SZU0		URB2	Urb2 domain-containing protein			1	0.89	2	2	2
G1SD09	A0A0J9YYJ0	CNPY4	Canopy FGF signaling regulator 4	Protein canopy homolog 4 (Fragment)	97	2	0.89	6	13	35
G1SRB7	A0A2R8Y7U1	TPP1	Peptidase S53 domain-containing protein	Tripeptidyl-peptidase 1 (Fragment)	93	2	0.89	7	14	23
G1T9L2	Q86VN1	VPS36	GLUE N-terminal domain-containing protein	Vacuolar protein-sorting-associated protein 36	97	2	0.89	4	4	18
	Q8WUW1	BRK1		Protein BRICK1		4	0.88	2	3	23
B7NZS4	O75175	CNOT3	CCR4-NOT transcription complex, subunit 3 (Predicted)	CCR4-NOT transcription complex subunit 3	97	2	0.88	3	4	9
G1U7Q6	H7C1D4	TSN	Uncharacterized protein	Translin (Fragment)	99	3	0.88	3	47	22
G1SYB4	P60953	CDC42	Uncharacterized protein	Cell division control protein 42 homolog	100	3	0.88	7	25	49
G1SQN6	J3KN59	BNIP2	CRAL-TRIO domain-containing protein	BCL2/adenovirus E1B 19 kDa protein-interacting protein 2	94	2	0.88	2	3	11
G1SIE6	P29279	CCN2	Cellular communication network factor 2	CCN family member 2	90	2	0.88	11	16	32
G1U1E5	O60826	CCDC22	Coiled-coil domain containing 22	Coiled-coil domain-containing protein 22	75	2	0.88	6	6	12
G1SMM4		DKK3	Dickkopf_N domain-containing protein			1	0.88	3	8	14
G1T284	Q8TCJ2	STT3B	Uncharacterized protein	Dolichyl-diphosphooligosaccharide--protein glycosyltransferase subunit STT3B	100	3	0.88	5	2	9
G1TN13	A8CTZ0	ITSN1	Intersectin 1	Intersectin 1 short form variant 13	88	2	0.88	5	4	12
G1T0Z8	Q99471	PFDN5	Uncharacterized protein	Prefoldin subunit 5	99	3	0.88	4	5	44

G1SL62	P07355	ANXA2	Annexin	Annexin A2	98	2	0.88	26	56	71
U3KMU7	A0A024R571	EHD1	EH domain containing 1	EH domain-containing protein 1	99	2	0.88	15	14	35
G1SPA6	Q32P28	P3H1	Fe2OG dioxygenase domain-containing protein	Prolyl 3-hydroxylase 1	91	2	0.88	24	44	52
G1SH86		TBCD	Tubulin folding cofactor D			1	0.88	13	16	17
G1SHF1	Q9UHV9	PFDN2	Uncharacterized protein	Prefoldin subunit 2	99	3	0.88	4	8	36
G1SET5		CEMIP2	G8 domain-containing protein			1	0.88	4	2	5
G1T2N2		LTN1	RING-type domain-containing protein			1	0.88	4	3	4
	P10301	RRAS		Ras-related protein R-Ras		4	0.88	6	5	38
G1T361	Q96I99	SUCLG2	Succinate--CoA ligase [GDP-forming] subunit beta, mitochondrial	Succinate--CoA ligase [GDP-forming] subunit beta, mitochondrial	97	2	0.88	17	34	49
G1TM81	A0A0B4J2C3	TPT1	Translationally-controlled tumor protein	Translationally-controlled tumor protein	98	2	0.88	5	67	34
G1SIG2	P46108	CRK	Uncharacterized protein	Adapter molecule crk	99	3	0.88	7	3	37
G1TD36	Q6PML9	SLC30A9	Uncharacterized protein	Zinc transporter 9	96	3	0.88	4	5	8
G1TPC8	P08962	CD63	Tetraspanin	CD63 antigen	78	2	0.87	4	9	15
G1SM65		GALNT16	Polypeptide N-acetylgalactosaminyltransferase			1	0.87	3	8	16
G1SI20	O75874	IDH1	Isocitrate dehydrogenase [NADP]	Isocitrate dehydrogenase [NADP] cytoplasmic	97	2	0.87	19	16	49
G1THP8	P46734	MAP2K3	Protein kinase domain-containing protein	Dual specificity mitogen-activated protein kinase kinase 3	97	2	0.87	6	9	29
G1T601		CLYBL	HpcH_HpaI domain-containing protein			1	0.87	4	6	19
	Q15366-3	PCBP2		Isoform 3 of Poly(rC)-binding protein 2		4	0.87	13	3	56
G1T4H3	Q15084	PDIA6	Uncharacterized protein	Protein disulfide-isomerase A6	93	3	0.87	17	109	52
G1T1D7	B1ALD9	POSTN	Uncharacterized protein	Periostin	90	3	0.87	16	36	34
G1SH30	Q8WVY7	UBLCP1	Uncharacterized protein	Ubiquitin-like domain-containing CTD phosphatase 1	100	3	0.87	2	2	10
G1SMF4	Q9UHP3	USP25	USP domain-containing protein	Ubiquitin carboxyl-terminal hydrolase 25	94	2	0.87	4	5	6
G1SM01		AKAP9	A-kinase anchor protein 9			1	0.87	3	3	1
G1ST51		CLIC2	Chloride intracellular channel protein			1	0.87	4	4	23
G1SSZ1	Q01658	DR1	CBFD_NFYB_HMF domain-containing protein	Protein Dr1	100	2	0.87	2	3	21
P80912		HINT1	Histidine triad nucleotide-binding protein 1			1	0.87	2	2	28
A7X8X3		HPRT	Hypoxanthine phosphoribosyltransferase			1	0.87	6	9	31
G1T845	I3L0N3	NSF	Uncharacterized protein	Vesicle-fusing ATPase	99	3	0.87	17	30	33
G1TBG2	Q96KP1	EXOC2	Exocyst complex component 2	Exocyst complex component 2	94	2	0.87	4	5	6
G1SZE0	Q96QK1	VPS35	Vacuolar protein sorting-associated protein 35	Vacuolar protein sorting-associated protein 35	100	2	0.87	22	47	37
U3KNY1	Q12797	ASPH	Aspartate beta-hydroxylase	Aspartyl/asparaginyl beta-hydroxylase	96	2	0.87	19	9	56
	A0A0J9YXC7	LIMS4		LIM and senescent cell antigen-like-containing domain protein		4	0.87	2	4	7
G1STX3	Q8WVM8	SCFD1	Uncharacterized protein	Sec1 family domain-containing protein 1	96	3	0.87	16	25	42
G1SNL7	Q9H1I8	ASCC2	Activating signal cointegrator 1 complex subunit 2	Activating signal cointegrator 1 complex subunit 2	86	2	0.87	3	4	6
G1T6S6		ATP6V1F	V-type proton ATPase subunit F			1	0.87	4	11	54
G1TW66		DMAC2	Distal membrane arm assembly complex 2			1	0.87	2	3	17
	A0A0A0MRE1	EXOC7		Exocyst complex component 7 (Fragment)		4	0.87	3	6	7
G1SEH1		PLSCR3	Phospholipid scramblase			1	0.87	2	4	13
G1TBU8	P52306	RAP1GDS1	Uncharacterized protein	Rap1 GTPase-GDP dissociation stimulator 1	97	3	0.87	6	8	15
G1SNM5	A0A2R8Y566	RELCH	LisH domain-containing protein	RAB11-binding protein RELCH	96	2	0.87	3	3	5
G1T670		SORT1	Proteasome subunit alpha type			1	0.87	10	19	48
G1SXY5		STX2	Syntaxin 2			1	0.87	3	3	21
G1T0H7	A0A087WWM0	TRAPPC3	Trafficking protein particle complex subunit	Trafficking protein particle complex subunit	94	2	0.87	4	8	27
G1SJZ4	J3KQ32	OLA1	Obg-like ATPase 1	Obg-like ATPase 1	100	2	0.87	9	16	34
G1SDG2	P48739	PITPNB	Phosphatidylinositol transfer protein beta	Phosphatidylinositol transfer protein beta isoform	97	2	0.87	4	4	14
G1TE64	O95758	PTBP3	Uncharacterized protein	Polypyrimidine tract-binding protein 3	97	3	0.87	5	3	20
G1SXH7	P12931	SRC	Tyrosine-protein kinase	Proto-oncogene tyrosine-protein kinase Src	96	2	0.87	5	6	14
U3KN22	Q9Y2Q3	GSTK1	Glutathione S-transferase kappa	Glutathione S-transferase kappa 1	79	2	0.87	9	18	47
G1TDN4	P10644	PRKAR1A	Uncharacterized protein	cAMP-dependent protein kinase type I-alpha regulatory subunit	99	3	0.87	4	8	17
G1U7C5	Q9P2E9	RRBP1	Uncharacterized protein	Ribosome-binding protein 1	83	3	0.87	44	97	38
G1SS77	Q15477	SKIV2L	Uncharacterized protein	Helicase SKI2W	95	3	0.87	7	9	7
G1SQF7	Q14554	PDIA5	Uncharacterized protein	Protein disulfide-isomerase A5	92	3	0.87	10	17	29
G1SGE5	A0A087WZR9	PYCR2	Pyrroline-5-carboxylate reductase	Pyrroline-5-carboxylate reductase	94	2	0.87	6	6	26
G1TCE2	H7C0R7	CYB5R1	NADH-cytochrome b5 reductase	NADH-cytochrome b5 reductase 1 (Fragment)	77	2	0.86	6	9	28
G1SEV2	P30101	PDIA3	Protein disulfide-isomerase	Protein disulfide-isomerase A3	96	2	0.86	30	215	45
	A8MZF9	DRG2		Developmentally-regulated GTP-binding protein 2		4	0.86	3	6	14
G1TA48	Q9H223	EHD4	Uncharacterized protein	EH domain-containing protein 4	97	3	0.86	14	19	37

G1T2Z8	B4DUC8	MTAP	S-methyl-5~-thioadenosine phosphorylase	S-methyl-5~-thioadenosine phosphorylase	99	2	0.86	4	6	27
G1SZK4	Q13442	PDAP1	PDGFA associated protein 1	28 kDa heat- and acid-stable phosphoprotein	96	2	0.86	2	4	16
G1TAN9	P21399	ACO1	Cytoplasmic aconitate hydratase	Cytoplasmic aconitate hydratase	93	2	0.86	5	6	9
G1SQ38	E5RHK8	DNM3	Uncharacterized protein	Dynamin-3	97	3	0.86	9	2	12
G1T0B4	Q9Y3B3	TMED7	GOLD domain-containing protein	Transmembrane emp24 domain-containing protein 7	92	2	0.86	7	20	52
G1SKS9	A0A087WSW9	TXNRD1	Glutaredoxin domain-containing protein	Thioredoxin reductase 1, cytoplasmic	94	2	0.86	10	10	27
G1SDA4	P53618	COPB1	Coatomer subunit beta	Coatomer subunit beta	99	2	0.86	37	194	54
	P68402	PAFAH1B2		Platelet-activating factor acetylhydrolase IB subunit beta		4	0.86	4	10	37
G1SZJ5	O15212	PFDN6	Uncharacterized protein	Prefoldin subunit 6	100	3	0.86	5	7	38
G1SWM8	A0A0U1RQQ9	SCYL2	Protein kinase domain-containing protein	SCY1-like protein 2	94	2	0.86	2	2	6
	P09497-2	CLTB		Isoform Non-brain of Clathrin light chain B		4	0.86	5	9	21
G1T4N4	A0A0B4J210	LARP1	HTH La-type RNA-binding domain-containing protein	La-related protein 1 (Fragment)	93	2	0.86	5	11	8
G1SUU7	Q9Y3I0	RTCB	tRNA-splicing ligase RtcB homolog	tRNA-splicing ligase RtcB homolog	100	2	0.86	16	3	43
A0A0A0MQP7	P62736	ACTA2	Actin, aortic smooth muscle	Actin, aortic smooth muscle	100	2	0.86	30	###	71
G1U2B5		PDXK	Phos_pyr_kin domain-containing protein			1	0.86	3	3	19
G1SZH8	Q8N1B4	VPS52	Uncharacterized protein	Vacuolar protein sorting-associated protein 52 homolog	99	3	0.86	4	3	12
G1SGJ4		SRR	PALP domain-containing protein			1	0.86	2	3	5
G1SH09	Q5VIR6	VPS53	VPS53, GARP complex subunit	Vacuolar protein sorting-associated protein 53 homolog	95	2	0.86	4	5	11
G1SGK1		GIGYF2	GRB10 interacting GYF protein 2			1	0.86	3	2	5
G1SGR0	Q8N3P4	VPS8	Uncharacterized protein	Vacuolar protein sorting-associated protein 8 homolog	93	3	0.86	2	3	3
G1SMP3	A0A2R8Y6F8	CASK	Uncharacterized protein	Peripheral plasma membrane protein CASK	97	3	0.86	12	14	17
G1SPB8	Q99733	NAP1L4	Uncharacterized protein	Nucleosome assembly protein 1-like 4	93	3	0.86	9	14	40
G1T0H3	P40763	STAT3	Signal transducer and activator of transcription	Signal transducer and activator of transcription 3	100	2	0.86	8	12	17
G1SZ34	Q9Y5S2	CDC42BPB	CDC42 binding protein kinase beta	Serine/threonine-protein kinase MRCK beta	92	2	0.86	10	10	11
G1SZ93	Q9NSE4	IARS2	Uncharacterized protein	Isoleucine--tRNA ligase, mitochondrial	90	3	0.86	14	14	22
G1TEB0	Q9UKX5	ITGA11	VWFA domain-containing protein	Integrin alpha-11	91	2	0.86	20	40	24
G1TGK3	B4DP31	PRPSAP1	Pribosyltran_N domain-containing protein	Phosphoribosyl pyrophosphate synthase-associated protein 1	99	2	0.86	3	3	13
G1SWK8	Q5TBG5	PSMB7	Proteasome subunit beta	Proteasome subunit beta (Fragment)	84	2	0.86	6	59	25
	P55010	EIF5		Eukaryotic translation initiation factor 5		4	0.85	5	8	15
G1SQR7	P61970	NUTF2	NTF2 domain-containing protein	Nuclear transport factor 2	100	2	0.85	2	2	17
G1TB45	Q86W92	PPFIBP1	Uncharacterized protein	Liprin-beta-1	87	3	0.85	22	39	33
G1T3P1	K7EKP8	ACOT7	Uncharacterized protein	Cytosolic acyl coenzyme A thioester hydrolase (Fragment)	98	3	0.85	5	5	23
G1SQ96	Q5H9R7	PPP6R3	Uncharacterized protein	Serine/threonine-protein phosphatase 6 regulatory subunit 3	92	3	0.85	7	8	13
G1T7N4	P41743	PRKCI	Protein kinase C	Protein kinase C iota type	99	2	0.85	2	2	7
G1SJJ2	Q9NZ32	ACTR10	Uncharacterized protein	Actin-related protein 10	96	3	0.85	10	23	40
G1U8F0	O95782	AP2A1	AP-2 complex subunit alpha	AP-2 complex subunit alpha-1	98	2	0.85	32	3	46
G1U7C7	Q5GLZ8	HERC4	HECT domain-containing protein	Probable E3 ubiquitin-protein ligase HERC4	96	2	0.85	3	3	6
	M0R261	PGLS		6-phosphogluconolactonase (Fragment)		4	0.85	2	4	13
G1T044	A0A3B3IUC4	GLA	Alpha-galactosidase	Alpha-galactosidase	75	2	0.85	3	3	7
G1T4T7	Q9Y2A7	NCKAP1	Uncharacterized protein	Nck-associated protein 1	100	3	0.85	15	18	21
G1T9Q5		SLC44A2	Solute carrier family 44 member 2			1	0.85	3	5	6
G1TBW1		TXNDC17	DUF953 domain-containing protein			1	0.85	3	8	31
G1SZR7	Q86UP2	KTN1	Uncharacterized protein	Kinectin	90	3	0.85	46	80	40
G1SWK5	A0A1W2PNP0	PIGT	Uncharacterized protein	GPI transamidase component PIG-T (Fragment)	86	3	0.85	3	4	8
	P20908	COL5A1		Collagen alpha-1(V) chain		4	0.85	9	3	6
G1SCE1	Q9H4G4	GLIPR2	SCP domain-containing protein	Golgi-associated plant pathogenesis-related protein 1	97	2	0.85	4	5	41
G1T6G1		MMAB	Corrinoid adenosyltransferase			1	0.85	3	3	19
G1SPL1	A0A286YF22	PHGDH	D-3-phosphoglycerate dehydrogenase	D-3-phosphoglycerate dehydrogenase	93	2	0.85	26	97	61
G1T2L1	A0A024RA52	PSMA2	Proteasome subunit alpha type	Proteasome subunit alpha type	100	2	0.85	11	32	59
G1TBL6	A0A087WVQ6	CLTC	Clathrin heavy chain	Clathrin heavy chain	99	2	0.85	80	522	63
G1SF26	P53675	CLTCL1	Clathrin heavy chain	Clathrin heavy chain 2	91	2	0.85	17	4	13
G1SL02	P63010	AP2B1	AP complex subunit beta	AP-2 complex subunit beta	98	2	0.85	41	56	57
G1SIA6	Q5T2E6	ARMH3	DUF1741 domain-containing protein	Armadillo-like helical domain-containing protein 3	99	2	0.85	4	4	11
G1T9P1		PEAK1	Pseudopodium enriched atypical kinase 1			1	0.85	3	3	3
G1SV13	P22314	UBA1	Ubiquitin-like modifier-activating enzyme 1	Ubiquitin-like modifier-activating enzyme 1	97	2	0.85	30	69	48
G1TP81	Q9Y6W5	WASF2	WH2 domain-containing protein	Wiskott-Aldrich syndrome protein family member 2	94	2	0.85	4	5	10
G1SZN0	Q9NZB2	FAM120A	Uncharacterized protein	Constitutive coactivator of PPAR-gamma-like protein 1	94	3	0.85	5	6	6

G1SJF1	A0A499FIZ0	WDR26	Uncharacterized protein	WD repeat-containing protein 26	99	3	0.85	7	5	18
G1T3V0	A0A024R4E5	HDLBP	Uncharacterized protein	High density lipoprotein binding protein (Vigilin), isoform CRA_a	97	3	0.85	45	105	47
G1T4Z0	Q96PY5	FMNL2	Formin like 2	Formin-like protein 2	97	2	0.84	7	4	8
G1SP89	J3QLD9	FLOT2	PHB domain-containing protein	Flotillin-2	97	2	0.84	11	16	34
G1STQ6	Q9Y617	PSAT1	Phosphoserine aminotransferase	Phosphoserine aminotransferase	94	2	0.84	9	16	22
G1TYN0	Q9BVK6	TMED9	Transmembrane p24 trafficking protein 9	Transmembrane emp24 domain-containing protein 9	80	2	0.84	9	9	35
G1SH85	A0A2R8YF87	VPS33A	Uncharacterized protein	Vacuolar protein sorting-associated protein 33A	91	3	0.84	8	9	22
G1SS73	Q92499	DDX1	Uncharacterized protein	ATP-dependent RNA helicase DDX1	98	3	0.84	20	38	37
G1T5W7	Q14376	GALE	NAD(P)-bd_dom domain-containing protein	UDP-glucose 4-epimerase	95	2	0.84	7	13	27
G1TEA8	P12268	IMPDH2	Inosine-5~-monophosphate dehydrogenase	Inosine-5~-monophosphate dehydrogenase 2	99	2	0.84	9	17	29
G1SPL8		NGB	Neuroglobin			1	0.84	2	2	18
G1TYU5		QTRT1	Queuine tRNA-ribosyltransferase			1	0.84	2	3	12
G1SCP0	O00203	AP3B1	AP-3 complex subunit beta	AP-3 complex subunit beta-1	88	2	0.84	13	17	16
G1THV8	Q9BT09	CNPY3	DUF3456 domain-containing protein	Protein canopy homolog 3	92	2	0.84	6	5	26
G1TIB4	P62857	RPS28	Ribosomal protein S28	40S ribosomal protein S28	100	2	0.84	3	8	46
G1SE51	A0A087X2D8	SPAG9	Sperm associated antigen 9	C-Jun-amino-terminal kinase-interacting protein 4	93	2	0.84	10	19	13
P15253	P27797	CALR	Calreticulin	Calreticulin	96	2	0.84	21	556	67
G1T5J8	Q9UJW0	DCTN4	Uncharacterized protein	Dynactin subunit 4	97	3	0.84	6	6	29
G1T450		RO60	TROVE domain-containing protein			1	0.84	3	6	7
G1T3Z2	Q9UNZ2	NSFL1C	Uncharacterized protein	NSFL1 cofactor p47	97	3	0.84	5	7	19
G1SMA1	P49755	TMED10	Transmembrane emp24 domain-containing protein 10	Transmembrane emp24 domain-containing protein 10	95	2	0.84	8	286	41
G1SIB6	M0R192	BLVRB	Biliverdin reductase B	Flavin reductase (NADPH)	73	2	0.84	7	32	51
G1SL38	F8VS81	TWF1	Twinfilin actin binding protein 1	Twinfilin-1 (Fragment)	96	2	0.84	7	13	25
G1T147	Q8IZ52	CHPF	Hexosyltransferase	Chondroitin sulfate synthase 2	95	2	0.84	3	3	12
Q29502	Q13177	PAK2	Serine/threonine-protein kinase PAK 2	Serine/threonine-protein kinase PAK 2	98	2	0.84	8	6	25
G1TQR9		ZSWIM8	SWIM-type domain-containing protein			1	0.84	2	3	2
G1SW24	P49588	AARS	AA_TRNA_LIGASE_II_ALA domain-containing protein	Alanine--tRNA ligase, cytoplasmic	93	2	0.84	33	25	47
G1SK52	Q5T6H7	XPNPEP1	Uncharacterized protein	Xaa-Pro aminopeptidase 1	97	3	0.84	10	11	25
G1SZ15	A8K878	MANF	Mesencephalic astrocyte derived neurotrophic factor	Mesencephalic astrocyte-derived neurotrophic factor	97	2	0.83	6	15	30
G1SKT1	Q7Z7H5	TMED4	GOLD domain-containing protein	Transmembrane emp24 domain-containing protein 4	94	2	0.83	7	14	44
G1SLS3	O43747	AP1G1	AP-1 complex subunit gamma	AP-1 complex subunit gamma-1	100	2	0.83	8	11	16
G1TER0	Q9NV70	EXOC1	Sec3-PIP2_bind domain-containing protein	Exocyst complex component 1	98	2	0.83	6	8	14
G1SD83		ITGA6	Integrin_alpha2 domain-containing protein			1	0.83	2	6	2
G1TWK7	Q14696	MESD	Uncharacterized protein	LRP chaperone MESD	90	3	0.83	4	6	22
G1TBL1	C9JPE1	SLC25A20	Uncharacterized protein	Mitochondrial carnitine/acylcarnitine carrier protein	92	3	0.83	5	16	22
Q28685	Q14118	DAG1	Dystroglycan	Dystroglycan	94	2	0.83	3	4	4
G1T3U1	Q9H8Y8	GORASP2	GRASP55_65 domain-containing protein	Golgi reassembly-stacking protein 2	91	2	0.83	6	15	18
G1SDU5	H3BPE1	MACF1	Uncharacterized protein	Microtubule-actin cross-linking factor 1, isoforms 1/2/3/5	88	3	0.83	203	152	38
G1T1T4		ADPGK	ADP dependent glucokinase			1	0.83	9	17	27
	Q8WXF7-2	ATL1		Isoform 2 of Atlastin-1		4	0.83	5	5	15
P06813	A0A075B7C0	CAPNS1	Calpain small subunit 1	Calpain small subunit 1 (Fragment)	96	2	0.83	10	20	69
G1T1F0	A0A2R8Y811	RPS14	Uncharacterized protein	40S ribosomal protein S14 (Fragment)	100	3	0.83	5	9	37
G1T057	D6RCE2	TTC37	Uncharacterized protein	Tetratricopeptide repeat protein 37 (Fragment)	83	3	0.83	7	12	6
G1SMK4		UCKL1	Uridine-cytidine kinase 1 like 1			1	0.83	2	3	6
G1SLL1	A0A087X0K9	TJP1	Uncharacterized protein	Tight junction protein ZO-1	82	3	0.83	21	27	19
G1T7Y7	B1AK87	CAPZB	F-actin-capping protein subunit beta	F-actin-capping protein subunit beta	100	2	0.83	11	35	54
G1SR93	B8ZZA2	FAM126A	Uncharacterized protein	Hyccin	98	3	0.83	2	2	6
G1TEJ4	Q86VW0	SESTD1	SEC14 and spectrin domain containing 1	SEC14 domain and spectrin repeat-containing protein 1	95	2	0.83	2	4	8
G1SL98	Q53EP0	FNDC3B	Uncharacterized protein	Fibronectin type III domain-containing protein 3B	97	3	0.83	15	4	20
G1SZM0	Q9BRT3	MIEN1	Uncharacterized protein	Migration and invasion enhancer 1	95	3	0.83	2	2	16
G1TM86	A2AB27	GNL1	G protein nucleolar 1 (putative)	Guanine nucleotide-binding protein-like 1 (Fragment)	98	2	0.83	2	3	5
G1SIB0	Q12907	LMAN2	L-type lectin-like domain-containing protein	Vesicular integral-membrane protein VIP36	98	2	0.82	6	14	23
G1SRT1	Q9UKG1	APPL1	Adaptor protein, phosphotyrosine interacting with PH domain and leucine zipper 1	DCC-interacting protein 13-alpha	98	2	0.82	8	6	19
G1T087	Q93034	CUL5	Cullin-5	Cullin-5	100	2	0.82	4	13	10
G1SWY3	A0A1B0GUZ7	EFR3A	Uncharacterized protein	Protein EFR3 homolog A	98	3	0.82	2	3	5
G1T1R9	Q96A65	EXOC4	Sec8_exocyst domain-containing protein	Exocyst complex component 4	95	2	0.82	11	14	19
G1SNM1	P41250	GARS	Uncharacterized protein	Glycine--tRNA ligase	95	3	0.82	22	40	39

G1T7B1	Q9H3P7	ACBD3	Uncharacterized protein	Golgi resident protein GCP60	96	3	0.82	7	6	21
G1T6C0	P61020	RAB5B	Uncharacterized protein	Ras-related protein Rab-5B	100	3	0.82	9	37	56
	J3KN75	TBC1D8B		TBC1 domain family member 8B		4	0.82	2	2	3
G1SL53	Q66K14	TBC1D9B	Uncharacterized protein	TBC1 domain family member 9B	89	3	0.82	3	2	3
G1T4H0	Q9NYL9	TMOD3	Uncharacterized protein	Tropomodulin-3	93	3	0.82	15	24	50
G1SRJ7	P08253	MMP2	72 kDa type IV collagenase	72 kDa type IV collagenase	95	2	0.82	7	9	19
G1SGY2	H0YC15	PTPN12	Tyrosine-protein phosphatase non-receptor type 12	Tyrosine-protein phosphatase non-receptor type 12 (Fragment)	90	2	0.82	2	3	21
G1TKY3	P17655	CAPN2	Calpain-2 catalytic subunit	Calpain-2 catalytic subunit	94	2	0.82	22	38	49
G1T9Y4	E5RIU9	CHMP7	Uncharacterized protein	Charged multivesicular body protein 7 (Fragment)	69	3	0.82	2	3	9
G1U3X5	P46821	MAP1B	Uncharacterized protein	Microtubule-associated protein 1B	88	3	0.82	15	9	10
G1SZR4	H7BZ14	PPIL3	Peptidyl-prolyl cis-trans isomerase	Peptidyl-prolyl cis-trans isomerase (Fragment)	100	2	0.82	2	3	12
G1SST7	Q96SL4	GPX7	Glutathione peroxidase	Glutathione peroxidase 7	92	2	0.82	6	9	36
G1SN17		PPP4R1	WRNPLPNID domain-containing protein			1	0.82	4	2	8
G1T8S0	Q9C0D5	TANC1	Uncharacterized protein	Protein TANC1	85	3	0.82	8	5	9
U3KNB6	P48444	ARCN1	Coatomer subunit delta	Coatomer subunit delta	97	2	0.82	18	32	40
G1SZT8	P55735	SEC13	WD_REPEATS_REGION domain-containing protein	Protein SEC13 homolog	95	2	0.82	8	16	38
	Q8N3D4	EHBP1L1		EH domain-binding protein 1-like protein 1		4	0.82	2	2	2
G1SEM0	Q96AC1	FERMT2	PH domain-containing protein	Fermitin family homolog 2	100	2	0.82	22	40	47
	M0QXF7	MYDGF		Myeloid-derived growth factor (Fragment)		4	0.82	2	2	19
G1T706	O00442	RTCA	Uncharacterized protein	RNA 3~-terminal phosphate cyclase	96	3	0.82	5	6	19
G1T7I4	P53621	COPA	Coatomer subunit alpha	Coatomer subunit alpha	99	2	0.82	48	96	52
	A0A0G2JL54	C4B_2		Complement C4-B		4	0.82	3	2	1
G1SZ91		FABP5	Lipocln_cytosolic_FA-bd_dom domain-containing protein			1	0.82	4	11	37
G1SGX3	P22059	OSBP	Oxysterol-binding protein	Oxysterol-binding protein 1	97	2	0.82	8	9	12
G1T964		TMED1	GOLD domain-containing protein			1	0.82	3	6	27
G1T860	M0R0P8	MYO9B	Myosin IXB	Unconventional myosin-IXb	83	2	0.82	13	9	13
G1SV22	P30086	PEBP1	Phosphatidylethanolamine-binding protein 1	Phosphatidylethanolamine-binding protein 1	89	2	0.81	5	8	37
G1SHI2		MYD88	Myeloid differentiation primary response protein MyD88			1	0.81	2	2	9
G1TM22		UGT1A1	UDP-glucuronosyltransferase			1	0.81	7	2	22
G1T196	Q13033	STRN3	WD_REPEATS_REGION domain-containing protein	Striatin-3	92	2	0.81	4	3	12
G1T3A2	Q9Y3A6	TMED5	GOLD domain-containing protein	Transmembrane emp24 domain-containing protein 5	97	2	0.81	5	12	24
G1TED6	P08758	ANXA5	Annexin	Annexin A5	93	2	0.81	19	50	65
G1TPB1		CRAT	Carnitine O-acetyltransferase			1	0.81	2	2	8
G1T4M1		LAMC1	Laminin subunit gamma 1			1	0.81	4	9	9
G1T358	Q96AG3	SLC25A46	Uncharacterized protein	Solute carrier family 25 member 46	94	3	0.81	3	5	18
G1T416	Q9UP83	COG5	Uncharacterized protein	Conserved oligomeric Golgi complex subunit 5	91	3	0.81	5	5	11
G1SU80	O60462	NRP2	Neuropilin	Neuropilin-2	95	2	0.81	17	18	25
G1SQ23	P42025	ACTR1B	Uncharacterized protein	Beta-centractin	99	3	0.81	7	4	25
G1SYM3		CD9	Tetraspanin			1	0.81	3	4	7
G1U6Y3		MEAK7	MTOR associated protein, eak-7 homolog			1	0.81	3	2	16
G1TLD3	Q02818	NUCB1	Nucleobindin 1	Nucleobindin-1	87	2	0.81	17	20	45
	P56377	AP1S2		AP-1 complex subunit sigma-2		4	0.81	4	4	33
G1SWM7	Q5W0J6	ECHDC3	Uncharacterized protein	Enoyl-CoA hydratase domain-containing protein 3, mitochondrial (Fragment)	90	3	0.81	2	4	9
G1TI02	Q9BRP8	PYM1	PYM homolog 1, exon junction complex associated factor	Partner of Y14 and mago	91	2	0.81	2	4	6
G1TAK5		WNK4	WNK lysine deficient protein kinase 4			1	0.81	3	3	2
	Q15149	PLEC		Plectin		4	0.81	84	113	21
G1SCT1	A0A499FJL1	PREP	Uncharacterized protein	Prolyl endopeptidase	96	3	0.81	19	33	41
P51662	P04083	ANXA1	Annexin A1	Annexin A1	91	2	0.80	20	20	61
G1SQU0	P15586	GNS	N-acetylglucosamine-6-sulfatase	N-acetylglucosamine-6-sulfatase	95	2	0.80	10	13	19
G1SF36	Q13423	NNT	Uncharacterized protein	NAD(P) transhydrogenase, mitochondrial	97	3	0.80	25	60	28
G1TSL5	C9J6N9	UFD1	Uncharacterized protein	Ubiquitin recognition factor in ER-associated degradation protein 1 (Fragment)	100	3	0.80	3	3	13
G1TII2	O95197	RTN3	Reticulon	Reticulon-3	72	2	0.80	4	5	6
G1T5Q4	Q9H446	RWDD1	RWD domain-containing protein	RWD domain-containing protein 1	91	2	0.80	4	6	27
G1T933	Q05707	COL14A1	Uncharacterized protein	Collagen alpha-1(XIV) chain	94	3	0.80	34	57	27
G1T7R4	P18084	ITGB5	Integrin beta	Integrin beta-5	93	2	0.80	5	6	8
G1TPW2		MRI1	Methylthioribose-1-phosphate isomerase			1	0.80	4	5	22
	P62854	RPS26		40S ribosomal protein S26		4	0.80	2	11	21

G1TJA8		FNBP1	Formin binding protein 1			1	0.80	3	2	4
G1TQV4	F6SKB8	NECAP2	DUF1681 domain-containing protein	Adaptin ear-binding coat-associated protein 2	95	2	0.80	2	2	8
G1SX73	E5RGS4	PFDN1	Uncharacterized protein	Prefoldin subunit 1	88	3	0.80	4	7	30
G1T3J0		SLC26A7	Anion exchange transporter			1	0.80	2	2	7
G1STP3	O75718	CRTAP	Uncharacterized protein	Cartilage-associated protein	94	3	0.80	11	16	32
G1STJ8	G3V394	MYO5A	Uncharacterized protein	Unconventional myosin-Va	96	3	0.80	17	3	12
G1T8D7	Q8IUX7	AEBP1	F5/8 type C domain-containing protein	Adipocyte enhancer-binding protein 1	76	2	0.80	5	5	8
G1TNH0	O14939	PLD2	Phospholipase D2	Phospholipase D2	89	2	0.80	3	2	7
	A0A2R8Y3S6	SNX27		Sorting nexin-27 (Fragment)		4	0.80	2	3	20
	E9PM12	TCIRG1		V-type proton ATPase subunit a (Fragment)		4	0.80	2	2	9
G1T6W4		ANXA8	Annexin			1	0.80	18	66	66
G1TB49		APOA1BP	NAD(P)H-hydrate epimerase			1	0.80	2	5	15
G1TBC4	G3V126	ATP6V1H	V-type proton ATPase subunit H	V-type proton ATPase subunit H	99	2	0.80	6	9	23
G1SMX4	A0A0G2JH68	DIAPH1	Uncharacterized protein	Protein diaphanous homolog 1	90	3	0.80	15	22	22
G1SSN2		SIRT5	NAD-dependent protein deacylase sirtuin-5, mitochondrial			1	0.80	2	2	10
G1SQ12	Q8TDJ6	DMXL2	WD_REPEATS_REGION domain-containing protein	DmX-like protein 2	93	2	0.79	2	2	1
G1T652	P09972	ALDOC	Fructose-bisphosphate aldolase	Fructose-bisphosphate aldolase C	99	2	0.79	9	7	30
G1TDQ5	A0A2R8Y891	PFKM	ATP-dependent 6-phosphofructokinase	ATP-dependent 6-phosphofructokinase	97	2	0.79	5	7	10
G1T7T6	O60763	USO1	Uncharacterized protein	General vesicular transport factor p115	95	3	0.79	22	37	35
G1SN22	Q9Y5K6	CD2AP	Uncharacterized protein	CD2-associated protein	91	3	0.79	2	2	5
G1U9C1	P50570	DNM2	Dynamin 2	Dynamin-2	96	2	0.79	24	6	36
G1U5L3	P49257	LMAN1	L-type lectin-like domain-containing protein	Protein ERGIC-53	91	2	0.79	15	64	40
G1SHA4		TEP1	Telomerase associated protein 1			1	0.79	5	8	3
G1SM31	Q9P2G1	ANKIB1	RBR-type E3 ubiquitin transferase	Ankyrin repeat and IBR domain-containing protein 1	93	2	0.79	2	4	4
G1SFX7	P62330	ARF6	Uncharacterized protein	ADP-ribosylation factor 6	100	3	0.79	5	9	33
G1SS33	P36543	ATP6V1E1	Uncharacterized protein	V-type proton ATPase subunit E 1	99	3	0.79	11	16	58
G1TJC8		PEX5	TPR_REGION domain-containing protein			1	0.79	2	2	5
	P17612	PRKACA		cAMP-dependent protein kinase catalytic subunit alpha		4	0.79	6	3	27
G1TZ40	Q5M775	SPECC1	Calponin-homology (CH) domain-containing protein	Cytospin-B	88	2	0.79	2	4	3
G1TEM7	D6RA82	ANXA3	Annexin	Annexin	93	2	0.79	13	20	51
G1SCP8	P26038	MSN	FERM domain-containing protein	Moesin	94	2	0.79	33	21	59
Q28647		PPP2R5B	Serine/threonine-protein phosphatase 2A 56 kDa regulatory subunit beta isoform			1	0.79	2	2	7
G1T4Q7		ATP11C	Phospholipid-transporting ATPase			1	0.79	2	4	3
	A0A494C1N0	FKBP2		Peptidylprolyl isomerase		4	0.79	2	7	17
	Q14318	FKBP8		Peptidyl-prolyl cis-trans isomerase FKBP8		4	0.79	3	6	15
G1SN14	Q86VP6	CAND1	TIP120 domain-containing protein	Cullin-associated NEDD8-dissociated protein 1	100	2	0.79	32	47	36
G1SDC6	Q9NXC5	MIOS	zinc_ribbon_16 domain-containing protein	GATOR complex protein MIOS	98	2	0.79	2	2	2
G1SCL6	A0A494C1J1	SPECC1L	Calponin-homology (CH) domain-containing protein	Cytospin-A	90	2	0.79	12	14	15
G1SN76	O94804	STK10	Serine/threonine kinase 10	Serine/threonine-protein kinase 10	90	2	0.79	2	4	5
G1SDU1	Q96A49	SYAP1	BSD domain-containing protein	Synapse-associated protein 1	89	2	0.79	2	5	14
G1T578	E9PHY0	ACP2	Uncharacterized protein	Lysosomal acid phosphatase	94	3	0.79	4	8	14
G1SR77	P23634	ATP2B4	Calcium-transporting ATPase	Plasma membrane calcium-transporting ATPase 4	87	2	0.79	9	8	10
G1STQ7		TMEM97	Transmembrane protein 97			1	0.79	2	2	12
G1STY8	G3V5E4	GNPNAT1	Glucosamine 6-phosphate N-acetyltransferase	Glucosamine 6-phosphate N-acetyltransferase	100	2	0.78	4	4	20
G1SXL6	O94915	FRYL	Uncharacterized protein	Protein furry homolog-like	97	3	0.78	4	6	2
G1SIL2	Q9UNW1	MINPP1	Multiple inositol-polyphosphate phosphatase 1	Multiple inositol polyphosphate phosphatase 1	87	2	0.78	4	6	16
G1SNY0	E9PJD9	RPL27A	Ribosomal_L18e/L15P domain-containing protein	60S ribosomal protein L27a	98	2	0.78	3	5	22
A0A140TAW0	O43852	CALU	Calumenin	Calumenin	99	2	0.78	15	84	64
G1SCF4	G8JLD5	DNM1L	Uncharacterized protein	Dynamin-1-like protein	93	3	0.78	13	26	26
G1TF32	F5H6I7	ATL3	Atlastin GTPase 3	Atlastin-3	95	2	0.78	15	43	44
G1SV81	Q9BTE1	DCTN5	Uncharacterized protein	Dynactin subunit 5	100	3	0.78	2	2	9
G1T8H3	P51570	GALK1	Uncharacterized protein	Galactokinase	92	3	0.78	10	12	42
G1SW44	H7C286	NAGK	BcrAD_BadFG domain-containing protein	N-acetyl-D-glucosamine kinase	97	2	0.78	6	8	27
G1SW11		CERCAM	Cerebral endothelial cell adhesion molecule			1	0.78	6	9	16
G1SZW8	M0R165	EPS15L1	Epidermal growth factor receptor pathway substrate 15 like 1	Epidermal growth factor receptor substrate 15-like 1	88	2	0.78	9	8	16
	H0Y507	SH3PXD2A		SH3 and PX domain-containing protein 2A (Fragment)		4	0.78	2	2	3
	A0A494C0A9	CBFB		Core-binding factor subunit beta		4	0.78	4	5	31

G1TCD4	E5RGF9	FAM114A2	Uncharacterized protein	Protein FAM114A2 (Fragment)	80	3	0.78	5	2	18
G1TS93	Q15435	PPP1R7	LRRcap domain-containing protein	Protein phosphatase 1 regulatory subunit 7	95	2	0.78	10	9	46
G1SPZ2	Q13107	USP4	Ubiquitin carboxyl-terminal hydrolase	Ubiquitin carboxyl-terminal hydrolase 4	89	2	0.78	3	5	7
G1T8D4	D6REX3	SEC31A	WD_REPEATS_REGION domain-containing protein	Protein transport protein Sec31A	91	2	0.78	29	58	35
G1T107	P61088	UBE2N	UBIQUITIN_CONJUGAT_2 domain-containing protein	Ubiquitin-conjugating enzyme E2 N	100	2	0.78	5	9	59
G1SI22		AKAP12	A-kinase anchoring protein 12			1	0.78	11	12	17
G1SNE8	P31949	S100A11	Protein S100	Protein S100-A11	87	2	0.78	5	46	65
G1TPC5		TPD52	Tumor protein D52			1	0.78	3	6	19
G1SLF1		ADH5	S-(hydroxymethyl)glutathione dehydrogenase			1	0.78	4	5	12
G1TR97	A0A0A0MSA7	EIF4G3	Uncharacterized protein	Eukaryotic translation initiation factor 4 gamma 3	89	3	0.78	8	10	8
	Q8TBX8	PIP4K2C		Phosphatidylinositol 5-phosphate 4-kinase type-2 gamma		4	0.78	4	3	18
G1SIP7	O14976	GAK	Cyclin G associated kinase	Cyclin-G-associated kinase	77	2	0.78	4	4	4
G1T9W8	Q8TC07	TBC1D15	Rab-GAP TBC domain-containing protein	TBC1 domain family member 15	92	2	0.78	2	2	3
G1SDL9	Q9UEU0	VTI1B	t-SNARE coiled-coil homology domain-containing protein	Vesicle transport through interaction with t-SNAREs homolog 1B	93	2	0.78	4	6	24
	O14617	AP3D1		AP-3 complex subunit delta-1		4	0.77	8	11	9
G1SDW3		CDS2	Phosphatidate cytidylyltransferase			1	0.77	3	4	10
G1SHX1	P68036	UBE2L3	Ubiquitin conjugating enzyme E2 L3	Ubiquitin-conjugating enzyme E2 L3	96	2	0.77	6	11	57
G1U2R2	E9PLK3	NPEPPS	Aminopeptidase	Aminopeptidase	98	2	0.77	16	24	27
G1T6D1	P62829	RPL23	Uncharacterized protein	60S ribosomal protein L23	100	3	0.77	6	14	57
G1T7R2	P62258	YWHAE	14_3_3 domain-containing protein	14-3-3 protein epsilon	100	2	0.77	18	196	78
G1TBZ5	E9PCX2	AKR1B1	Aldo-keto reductase family 1 member B1	Aldo-keto reductase family 1 member B1	85	2	0.77	6	15	29
G1TD38	H3BVG8	VPS35L	Uncharacterized protein	VPS35 endosomal protein sorting factor-like	93	3	0.77	6	9	4
G1U9U1	P50995	ANXA11	Annexin	Annexin A11	92	2	0.77	7	9	17
G1SMX7	O43731	KDELR3	ER lumen protein-retaining receptor	ER lumen protein-retaining receptor 3	98	2	0.77	3	2	17
G1SWU1	P55809	OXCT1	Succinyl-CoA:3-ketoacid-coenzyme A transferase	Succinyl-CoA:3-ketoacid coenzyme A transferase 1, mitochondrial	94	2	0.77	18	105	52
G1SET0	P35606	COPB2	Coatomer subunit beta~	Coatomer subunit beta~	98	2	0.77	24	56	38
G1T182	Q86UE4	MTDH	Metadherin	Protein LYRIC	88	2	0.77	4	6	18
G1SID3	Q709C8	VPS13C	Vacuolar protein sorting 13 homolog C	Vacuolar protein sorting-associated protein 13C	88	2	0.77	10	10	4
G1TV76	Q9Y4D1	DAAM1	Uncharacterized protein	Disheveled-associated activator of morphogenesis 1	97	3	0.77	4	5	6
G1T6S4	F8W9J4	DST	Dystonin	Dystonin	62	2	0.77	13	6	4
G1SIT5	F2Z388	RPL35	Uncharacterized protein	60S ribosomal protein L35	93	3	0.77	3	5	25
G1SSL8		HEATR5A	HEAT repeat containing 5A			1	0.77	4	3	3
G1T9D6	C9J5C3	PDCD10	Uncharacterized protein	Programmed cell death protein 10 (Fragment)	99	3	0.77	7	8	48
	H0Y5B4	RPL36A		60S ribosomal protein L36a		4	0.77	3	6	16
G1TAD1	Q7RTS9	DYM	Uncharacterized protein	Dymeclin	97	3	0.77	4	7	13
G1SIN4	X6RLX0	ERC1	FIP-RBD domain-containing protein	ELKS/Rab6-interacting/CAST family member 1	98	2	0.77	14	20	15
G1TYW1		TPD52L2	TPD52 like 2			1	0.77	3	5	13
G1SDQ5	Q96JG6	VPS50	Uncharacterized protein	Syndetin	98	3	0.77	4	6	9
G1SJ23	P07384	CAPN1	Calpain-1 catalytic subunit	Calpain-1 catalytic subunit	90	2	0.77	10	11	25
G1T6T5	E9PPQ5	CHORDC1	Uncharacterized protein	Cysteine and histidine-rich domain-containing protein 1	94	3	0.77	2	2	9
G1SLC0	Q9BS26	ERP44	Thioredoxin domain-containing protein	Endoplasmic reticulum resident protein 44	97	2	0.77	13	29	42
G1SEJ4	P21281	ATP6V1B2	Vacuolar proton pump subunit B	V-type proton ATPase subunit B, brain isoform	99	2	0.77	18	37	55
G1SJQ2	Q92896	GLG1	Uncharacterized protein	Golgi apparatus protein 1	97	3	0.76	43	70	42
G1TBS1	Q99497	PARK7	DJ-1_PfpI domain-containing protein	Protein/nucleic acid deglycase DJ-1	96	2	0.76	11	21	72
G1TMP7	A0A0D9SG72	STXBP1	Syntaxin binding protein 1	Syntaxin-binding protein 1	99	2	0.76	4	4	10
G1SWS6	A0A3B3IRN5	FMOD	Fibromodulin	Fibromodulin	92	2	0.76	2	2	7
G1SRI2	Q6P996	PDXDC1	Uncharacterized protein	Pyridoxal-dependent decarboxylase domain-containing protein 1	89	3	0.76	17	28	35
G1SZF7	P48735	IDH2	Isocitrate dehydrogenase [NADP]	Isocitrate dehydrogenase [NADP], mitochondrial	96	2	0.76	20	126	47
G1T2F8	H0YJH8	ATP6V1D	V-type proton ATPase subunit D	V-type proton ATPase subunit D (Fragment)	99	2	0.76	3	14	23
G1SFU4	Q9UIQ6	LNPEP	Uncharacterized protein	Leucyl-cystinyl aminopeptidase	90	3	0.76	14	19	17
G1SY70	F5H365	SEC23A	Protein transport protein SEC23	Protein transport protein SEC23	96	2	0.76	22	37	47
G1U949	Q15691	MAPRE1	Uncharacterized protein	Microtubule-associated protein RP/EB family member 1	99	3	0.76	8	21	43
G1TJ80	Q9Y3Q3	TMED3	Transmembrane p24 trafficking protein 3	Transmembrane emp24 domain-containing protein 3	91	2	0.76	4	14	40
G1SKY8	Q96A57	TMEM230	Uncharacterized protein	Transmembrane protein 230	98	3	0.76	2	3	22
G1SMT2		FAM129B	Family with sequence similarity 129 member B			1	0.76	17	41	36
G1SPM5	P61163	ACTR1A	Uncharacterized protein	Alpha-centractin	100	3	0.75	11	15	40
G1SIA1	A0A087WWA3	KIF1B	Uncharacterized protein	Kinesin-like protein KIF1B	96	3	0.75	3	6	2

G1SX42	Q9Y3P9	RABGAP1	Uncharacterized protein	Rab GTPase-activating protein 1	97	3	0.75	2	2	3
G1T2M9	P09486	SPARC	SPARC	SPARC	94	2	0.75	13	25	45
G1SMS3	P61160	ACTR2	Actin-related protein 2	Actin-related protein 2	100	2	0.75	13	108	48
G1SIL8	A0A087WWM4	GMPR2	GMP reductase	GMP reductase	90	2	0.75	3	2	13
G1T198	Q8TDX7	NEK7	Protein kinase domain-containing protein	Serine/threonine-protein kinase Nek7	98	2	0.75	3	3	15
G1SEK2		PPM1F	PPM-type phosphatase domain-containing protein			1	0.75	2	3	11
G1TIZ5	Q8NBS9	TXNDC5	Uncharacterized protein	Thioredoxin domain-containing protein 5	87	3	0.75	13	28	39
G1TD16	P48426	PIP4K2A	PIPK domain-containing protein	Phosphatidylinositol 5-phosphate 4-kinase type-2 alpha	100	2	0.75	2	2	5
G1T782	Q9Y6N5	SQOR	Pyr_redox_2 domain-containing protein	Sulfide:quinone oxidoreductase, mitochondrial	93	2	0.75	6	14	18
G1U5A6	A0A286YFF8	MON2	Uncharacterized protein	Protein MON2 homolog	98	3	0.75	6	7	6
G1TGH1		D2HGDH	D-2-hydroxyglutarate dehydrogenase			1	0.75	4	7	13
G1SWD8	P42356	PI4KA	Uncharacterized protein	Phosphatidylinositol 4-kinase alpha	98	3	0.75	10	5	8
U3KP45		SYNE1	Spectrin repeat containing nuclear envelope protein 1			1	0.75	4	5	1
G1SE49	Q9UID3	VPS51	Uncharacterized protein	Vacuolar protein sorting-associated protein 51 homolog	96	3	0.75	6	6	15
G1SP83	Q9Y2D0	CA5B	Carbonic anhydrase 5B	Carbonic anhydrase 5B, mitochondrial	93	2	0.75	10	33	46
G1TVW5	Q96IJ6	GMPPA	NTP_transferase domain-containing protein	Mannose-1-phosphate guanyltransferase alpha	96	2	0.75	7	9	30
G1TI53	A0A0A0MRM8	MYO6	Uncharacterized protein	Unconventional myosin-VI	94	3	0.75	7	11	8
G1SPB4		HYAL1	Hyaluronidase			1	0.75	2	2	8
G1SQ22	Q9Y678	COPG1	Coatomer subunit gamma	Coatomer subunit gamma-1	99	2	0.74	35	164	58
Q9TTC6	P62937	PPIA	Peptidyl-prolyl cis-trans isomerase A	Peptidyl-prolyl cis-trans isomerase A	96	2	0.74	16	23	87
G1SEN8		SCCPDH	Sacchrp_dh_NADP domain-containing protein			1	0.74	5	6	24
G1STU4	E9PIE3	CAVIN3	Uncharacterized protein	Caveolae-associated protein 3	78	3	0.74	4	5	15
G1TEU5	O75131	CPNE3	Uncharacterized protein	Copine-3	96	3	0.74	3	2	7
G1TT75	O00264	PGRMC1	Cytochrome b5 heme-binding domain-containing protein	Membrane-associated progesterone receptor component 1	93	2	0.74	7	9	29
G1TMI5		RABAC1	PRA1 family protein			1	0.74	2	3	17
	O15143	ARPC1B		Actin-related protein 2/3 complex subunit 1B		4	0.74	3	6	14
G1TRH5	Q9ULV4	CORO1C	Coronin	Coronin-1C	97	2	0.74	19	72	44
G1SPZ7		GPX1	Glutathione peroxidase			1	0.74	5	6	51
G1TDN6		KRT5	IF rod domain-containing protein			1	0.74	5	2	8
G1TBA4	Q86UY8	NT5DC3	Uncharacterized protein	5~-nucleotidase domain-containing protein 3	97	3	0.74	9	14	26
G1TGA8	O75396	SEC22B	Uncharacterized protein	Vesicle-trafficking protein SEC22b	98	3	0.74	10	32	48
G1SYN5	J3QRU4	VAMP2	V-SNARE coiled-coil homology domain-containing protein	Vesicle-associated membrane protein 2	99	2	0.74	4	4	42
G1SCW0	A8MT72	RTN1	Reticulon	Reticulon	98	2	0.74	2	3	3
G1SKD5	Q92783	STAM	Uncharacterized protein	Signal transducing adapter molecule 1	96	3	0.74	5	11	14
G1SR53	P04066	FUCA1	Alpha-L-fucosidase	Tissue alpha-L-fucosidase	83	2	0.74	10	25	34
G1SDN8	Q92791	P3H4	Uncharacterized protein	Endoplasmic reticulum protein SC65	88	3	0.74	5	9	18
G1SLQ4	B4DXW1	ACTR3	Uncharacterized protein	Actin-related protein 3	100	3	0.74	17	41	71
G1SFP0	P28838	LAP3	CYTOSOL_AP domain-containing protein	Cytosol aminopeptidase	92	2	0.74	17	28	52
G1T5Y1	P28482	MAPK1	Mitogen-activated protein kinase	Mitogen-activated protein kinase 1	98	2	0.74	11	9	44
G1TEG8	Q8WUM4	PDCD6IP	BRO1 domain-containing protein	Programmed cell death 6-interacting protein	94	2	0.74	24	92	36
G1T6S9	P53367	ARFIP1	AH domain-containing protein	Arfaptin-1	98	2	0.74	4	5	15
G1SJH1	H7BXE3	SLTM	RRM domain-containing protein	SAFB-like transcription modulator (Fragment)	92	2	0.74	3	2	4
G1SD49	X6RCK5	DCTN3	Uncharacterized protein	Dynactin subunit 3 (Fragment)	82	3	0.73	4	5	24
G1T967	A0A0U1RRB6	EXOC6B	Exocyst complex component	Exocyst complex component	99	2	0.73	7	8	15
G1SR28	Q15102	PAFAH1B3	SGNH_hydro domain-containing protein	Platelet-activating factor acetylhydrolase IB subunit gamma	97	2	0.73	5	5	31
	P50454	SERPINH1		Serpin H1		4	0.73	15	30	35
G1T295		EPHX1	Epoxide hydrolase			1	0.73	5	6	15
G1TEW4	Q93008	USP9X	USP domain-containing protein	Probable ubiquitin carboxyl-terminal hydrolase FAF-X	99	2	0.73	35	52	21
Q9XS70	Q9BR76	CORO1B	Coronin-1B	Coronin-1B	93	2	0.73	9	26	27
G1SWL6	Q9P0K7	RAI14	ANK_REP_REGION domain-containing protein	Ankycorbin	91	2	0.73	24	42	32
G1T8R1		RAP1GAP2	Platelet-activating factor acetylhydrolase IB subunit alpha			1	0.73	8	8	24
G1SSL3	P29373	CRABP2	FABP domain-containing protein	Cellular retinoic acid-binding protein 2	90	2	0.73	3	3	27
G1T3R5		ERO1A	Endoplasmic reticulum oxidoreductase 1 alpha			1	0.73	15	31	34
G1U1H1	Q00535	CDK5	Protein kinase domain-containing protein	Cyclin-dependent-like kinase 5	100	2	0.73	3	2	12
	A0A2U3U034	ARSB		Arylsulfatase B		4	0.73	4	5	12
G1SIT9	P31946	YWHAB	14_3_3 domain-containing protein	14-3-3 protein beta/alpha	100	2	0.73	16	49	76
G1SMY7	C9JIF9	APEH	Acylamino-acid-releasing enzyme	Acylamino-acid-releasing enzyme	93	2	0.73	7	9	18

	J3QQX2	ARHGDIA		Rho GDP-dissociation inhibitor 1		4	0.73	5	7	18
G1SUF5	Q8TF66	LRRC15	LRRCT domain-containing protein	Leucine-rich repeat-containing protein 15	90	2	0.73	6	8	18
G1SFZ8	Q9UMX0	UBQLN1	Ubiquilin 1	Ubiquilin-1	86	2	0.73	8	9	26
G1T3V2	P04792	HSPB1	SHSP domain-containing protein	Heat shock protein beta-1	91	2	0.73	8	20	52
G1TCW5	A0A494C165	PEPD	Peptidase D	Xaa-Pro dipeptidase (Fragment)	79	2	0.73	7	11	22
G1SZ12	E7EPB3	RPL14	Ribosomal_L14e domain-containing protein	60S ribosomal protein L14	96	2	0.73	6	12	27
G1T078	O43294	TGFB1I1	Uncharacterized protein	Transforming growth factor beta-1-induced transcript 1 protein	95	3	0.73	4	6	11
Q09YN4	P47755	CAPZA2	F-actin-capping protein subunit alpha-2	F-actin-capping protein subunit alpha-2	98	2	0.73	8	12	50
	O14908	GIPC1		PDZ domain-containing protein GIPC1		4	0.73	4	6	16
G1T2I6	P45877	PPIC	Peptidyl-prolyl cis-trans isomerase	Peptidyl-prolyl cis-trans isomerase C	92	2	0.73	6	20	48
G1SYV9	Q9Y490	TLN1	Uncharacterized protein	Talin-1	99	3	0.73	108	35	61
G1SN05	Q14240	EIF4A2	Uncharacterized protein	Eukaryotic initiation factor 4A-II	100	3	0.72	14	15	49
G1SWK3	P49902	NT5C2	Uncharacterized protein	Cytosolic purine 5~-nucleotidase	100	3	0.72	8	14	21
G1SKV7	Q96KP4	CNDP2	M20_dimer domain-containing protein	Cytosolic non-specific dipeptidase	90	2	0.72	12	16	38
G1T634	Q9Y5X1	SNX9	Sorting nexin	Sorting nexin-9	89	2	0.72	7	8	23
G1TPH5	Q9Y6D5	ARFGEF2	ADP ribosylation factor guanine nucleotide exchange factor 2	Brefeldin A-inhibited guanine nucleotide-exchange protein 2	96	2	0.72	9	10	8
G1TBT4	Q9HB90	RRAGC	Uncharacterized protein	Ras-related GTP-binding protein C	98	3	0.72	5	7	23
G1TZ31	Q9Y680	FKBP7	Peptidylprolyl isomerase	Peptidyl-prolyl cis-trans isomerase FKBP7	77	2	0.72	11	26	44
G1U9I8	P04264	KRT1	IF rod domain-containing protein	Keratin, type II cytoskeletal 1	86	2	0.72	20	35	39
G1SZ18	A0A0C4DFT3	DLG1	Uncharacterized protein	Disks large homolog 1	93	3	0.72	4	5	7
	Q63ZY3	KANK2		KN motif and ankyrin repeat domain-containing protein 2		4	0.72	9	6	13
G1SDF2	A0A0A0MRJ6	PCMT1	Protein-L-isoaspartate O-methyltransferase	Protein-L-isoaspartate O-methyltransferase	96	2	0.72	5	7	21
	A0A3B3ISV4	VKORC1L1		Vitamin K epoxide reductase complex subunit 1-like protein 1		4	0.72	2	3	9
G1TRS4	O95302	FKBP9	Peptidylprolyl isomerase	Peptidyl-prolyl cis-trans isomerase FKBP9	97	2	0.72	17	134	41
G1TS73	H0YBP1	PTK2	Protein tyrosine kinase 2	Focal adhesion kinase 1 (Fragment)	89	2	0.72	4	4	6
G1SM05	Q9NR31	SAR1A	Uncharacterized protein	GTP-binding protein SAR1a	99	3	0.72	6	9	41
G1SVI9	Q9P0L0	VAPA	MSP domain-containing protein	Vesicle-associated membrane protein-associated protein A	84	2	0.72	9	20	40
G1TJY2		CHID1	Chitinase domain containing 1			1	0.72	10	18	53
G1SDT0	Q562R1	ACTBL2	Uncharacterized protein	Beta-actin-like protein 2	97	3	0.72	14	18	41
G1SQ80	Q8TDZ2	MICAL1	Uncharacterized protein	[F-actin]-monooxygenase MICAL1	83	3	0.72	4	4	8
G1U3F3		NEXN	Ig-like domain-containing protein			1	0.72	22	26	36
G1SDY5	P63104	YWHAZ	14_3_3 domain-containing protein	14-3-3 protein zeta/delta	100	2	0.72	15	152	65
G1SIC4	I3L294	ABHD12	Abhydrolase domain containing 12	Lysophosphatidylserine lipase ABHD12 (Fragment)	99	2	0.71	4	6	18
G1U9R6	P02751	FN1	Fibronectin	Fibronectin	70	2	0.71	69	143	46
G1T7D9		FNTB	Protein farnesyltransferase subunit beta			1	0.71	3	2	6
G1SH26	F6TLX2	GLOD4	Glyoxalase domain containing 4	Glyoxalase domain-containing protein 4	81	2	0.71	7	10	23
G1SN09	Q9Y5P6	GMPPB	NTP_transferase domain-containing protein	Mannose-1-phosphate guanyltransferase beta	99	2	0.71	4	3	22
G1SD27	B7ZC38	SH3GLB2	SH3 domain containing GRB2 like, endophilin B2	Endophilin-B2	92	2	0.71	2	2	6
G1T5Z7	B3KR49	MAPK3	Mitogen-activated protein kinase	Mitogen-activated protein kinase 3	97	2	0.71	6	3	37
G1SV03	Q14165	MLEC	Malectin domain-containing protein	Malectin	95	2	0.71	8	14	32
G1TR42		RNPEP	Leuk-A4-hydro_C domain-containing protein			1	0.71	5	5	10
	A0A0A0MT60	FKBP15		Peptidylprolyl isomerase (Fragment)		4	0.71	3	2	3
G1SHI0	H0Y4D4	ACAA1	Acetyl-CoA acyltransferase 1	3-ketoacyl-CoA thiolase, peroxisomal (Fragment)	62	2	0.71	8	7	34
	Q9UJY5	GGA1		ADP-ribosylation factor-binding protein GGA1		4	0.71	3	4	9
G1T8X3		NEU1	Sialidase domain-containing protein			1	0.71	4	5	18
G1TJC3	Q9BUF5	TUBB6	Tubulin beta chain	Tubulin beta-6 chain	97	2	0.71	17	20	57
G1SUK5	O43583	DENR	Density-regulated protein	Density-regulated protein	97	2	0.71	2	4	9
G1SE28	C9JNW5	RPL24	TRASH domain-containing protein	60S ribosomal protein L24	100	2	0.71	6	10	34
G1TS42		AGL	Glycogen debranching enzyme			1	0.71	3	2	4
G1SHS7	O75083	WDR1	WD_REPEATS_REGION domain-containing protein	WD repeat-containing protein 1	94	2	0.71	21	49	51
G1TWB9		TBC1D5	Rab-GAP TBC domain-containing protein			1	0.71	2	3	4
G1TZP0	P61981	YWHAG	14_3_3 domain-containing protein	14-3-3 protein gamma	99	2	0.70	13	49	70
G1T8L2	P05997	COL5A2	Uncharacterized protein	Collagen alpha-2(V) chain	96	3	0.70	15	30	17
G1TBC9		TMEM119	Transmembrane protein 119			1	0.70	2	2	17
Q6SQH4	P60903	S100a10	Protein S100-A10	Protein S100-A10	100	2	0.70	4	11	35
G1TWQ3		SIRT2	NAD-dependent protein deacetylase			1	0.70	5	5	19
G1TRM4	P62280	RPS11	Ribosomal_S17_N domain-containing protein	40S ribosomal protein S11	100	2	0.70	9	20	55

G1SNP8	C9JJP5	TFG	PB1 domain-containing protein	Protein TFG (Fragment)	97	2	0.70	7	16	23
G1SY85	Q9Y2T2	AP3M1	MHD domain-containing protein	AP-3 complex subunit mu-1	99	2	0.70	9	12	42
	Q08209-2	PPP3CA		Isoform 2 of Serine/threonine-protein phosphatase 2B catalytic subunit alpha isoform		4	0.70	9	15	27
G1U446	Q14677	CLINT1	ENTH domain-containing protein	Clathrin interactor 1	97	2	0.70	9	12	17
G1TNY9		TRADD	Death domain-containing protein			1	0.70	2	3	10
G1TFI4	Q96K17	BTF3L4	Transcription factor BTF3	Transcription factor BTF3 homolog 4	100	2	0.70	3	4	39
G1SXQ0	A0A0A0MTN3	GSTM3	Glutathione S-transferase	Glutathione S-transferase	90	2	0.70	10	22	37
G1TEH2	P21283	ATP6V1C1	V-type proton ATPase subunit C	V-type proton ATPase subunit C 1	99	2	0.70	7	10	20
G1TBW9	Q96FJ2	DYNLL2	Dynein light chain	Dynein light chain 2, cytoplasmic	100	2	0.70	2	10	33
G1SVH8	Q13636	RAB31	Uncharacterized protein	Ras-related protein Rab-31	95	3	0.70	6	6	36
G1SWW6	F5H459	AP3S1	Clat_adaptor_s domain-containing protein	AP complex subunit sigma	95	2	0.70	2	3	14
B7NZQ3		RA_m006_jsm824E4r	Deoxyribonuclease			1	0.69	6	13	28
G1T346	Q13813-3	SPTAN1	Uncharacterized protein	Isoform 3 of Spectrin alpha chain, non-erythrocytic 1	97	3	0.69	108	31	55
G1SKL7	Q99536	VAT1	Vesicle amine transport 1	Synaptic vesicle membrane protein VAT-1 homolog	78	2	0.69	9	17	35
G1SP34	O15144	ARPC2	Arp2/3 complex 34 kDa subunit	Actin-related protein 2/3 complex subunit 2	100	2	0.69	15	30	54
G1SNI2	Q12841	FSTL1	Kazal-like domain-containing protein	Follistatin-related protein 1	94	2	0.69	8	23	32
G1U826	O75348	ATP6V1G1	V-type proton ATPase subunit G	V-type proton ATPase subunit G 1	97	2	0.69	3	7	39
G1U7Y3	E7END4	LOXL3	Uncharacterized protein	Lysyl oxidase homolog 3	87	3	0.69	3	3	5
A0A494BI13		NAAA	N-acylethanolamine-hydrolyzing acid amidase			1	0.69	2	2	8
G1T4C9	Q8IVL6	P3H3	Prolyl 3-hydroxylase 3	Prolyl 3-hydroxylase 3	88	2	0.69	14	21	29
G1SQ03	Q8WWI5	SLC44A1	Solute carrier family 44 member 1	Choline transporter-like protein 1	93	2	0.69	3	4	7
G1SKT3	Q9NUJ1	ABHD10	AB hydrolase-1 domain-containing protein	Mycophenolic acid acyl-glucuronide esterase, mitochondrial	88	2	0.69	8	9	36
	O14964	HGS		Hepatocyte growth factor-regulated tyrosine kinase substrate		4	0.69	5	6	13
G1SL68	P35579	MYH9	Uncharacterized protein	Myosin-9	96	3	0.69	119	283	57
G1U460	Q02252	ALDH6A1	Aldedh domain-containing protein	Methylmalonate-semialdehyde dehydrogenase [acylating], mitochondrial	95	2	0.69	8	16	26
G1U0B3	P53007	SLC25A1	Solute carrier family 25 member 1	Tricarboxylate transport protein, mitochondrial	86	2	0.69	10	24	48
G1SNY5	H0YK42	SNX1	PX domain-containing protein	Sorting nexin-1	96	2	0.69	9	13	20
G1T6Q9	O15400	STX7	t-SNARE coiled-coil homology domain-containing protein	Syntaxin-7	95	2	0.69	7	14	48
G1TCY4	Q9Y5X3	SNX5	Sorting nexin	Sorting nexin-5	98	2	0.68	4	8	12
G1TPZ1		LGALS1	Galectin			1	0.68	9	34	75
G1SNH7	P09417	QDPR	Quinoid dihydropteridine reductase	Dihydropteridine reductase	92	2	0.68	4	5	23
G1SX32	O00743	PPP6C	Serine/threonine-protein phosphatase	Serine/threonine-protein phosphatase 6 catalytic subunit	100	2	0.68	2	2	16
G1U670	A0A087WW40	SH3GLB1	Uncharacterized protein	Endophilin-B1	95	3	0.68	2	3	6
G1TQJ4	Q01433	AMPD2	AMP deaminase	AMP deaminase 2	97	2	0.68	3	5	5
	Q16181	SEPTIN7		Septin-7		4	0.68	19	52	57
G1TA50	E7EQ61	UBA5	ThiF domain-containing protein	Ubiquitin-like modifier-activating enzyme 5	93	2	0.68	6	8	27
G1TMW2		TOLLIP	Toll interacting protein			1	0.68	4	5	25
G1SN11	Q01082	SPTBN1	Spectrin beta chain	Spectrin beta chain, non-erythrocytic 1	99	2	0.68	75	13	44
G1SH88	Q8N8S7	ENAH	ENAH, actin regulator	Protein enabled homolog	99	2	0.68	10	2	28
G1T726	A0A0A0MSE2	HADH	Uncharacterized protein	Hydroxyacyl-coenzyme A dehydrogenase, mitochondrial	92	3	0.68	10	14	43
G1SUY5	E7EVZ5	PCYOX1L	Prenylcys_lyase domain-containing protein	Prenylcysteine oxidase-like	95	2	0.68	4	8	15
G1T3I9	P20073	ANXA7	Annexin	Annexin A7	93	2	0.68	10	13	20
G1TKE7		IQGAP2	IQ motif containing GTPase activating protein 2			1	0.68	4	2	3
G1SJ87	Q9H2D6	TRIOBP	TRIO and F-actin binding protein	TRIO and F-actin-binding protein	73	2	0.68	3	3	2
G1T3U5	Q9UDY4	DNAJB4	J domain-containing protein	DnaJ homolog subfamily B member 4	96	2	0.68	4	4	21
P53787		EEF1D	Elongation factor 1-delta			1	0.68	4	15	24
G1TRX7	P23743	DGKA	Diacylglycerol kinase	Diacylglycerol kinase alpha	93	2	0.67	4	5	9
G1TTJ1	Q6UWP2	DHRS11	Uncharacterized protein	Dehydrogenase/reductase SDR family member 11	92	3	0.67	4	5	21
G1T9X4	O95980	RECK	Reversion inducing cysteine rich protein with kazal motifs	Reversion-inducing cysteine-rich protein with Kazal motifs	95	2	0.67	3	4	3
G1TN89	P98160	HSPG2	Heparan sulfate proteoglycan 2	Basement membrane-specific heparan sulfate proteoglycan core protein	90	2	0.67	30	51	10
G1SQK1		SERPINB6	SERPIN domain-containing protein			1	0.67	3	4	17
G1SXU7	F8WA11	CLASP1	Cytoplasmic linker associated protein 1	CLIP-associating protein 1	93	2	0.67	4	2	4
G1TVQ3		DYNLRB1	Dynein light chain roadblock			1	0.67	3	4	51
G1T7H6	Q6UX71	PLXDC2	PSI domain-containing protein	Plexin domain-containing protein 2	94	2	0.67	4	9	9
G1TIZ2	Q02809	PLOD1	Procollagen-lysine,2-oxoglutarate 5-dioxygenase 1	Procollagen-lysine,2-oxoglutarate 5-dioxygenase 1	93	2	0.67	26	18	41
G1SZ63	P49189	ALDH9A1	Aldedh domain-containing protein	4-trimethylaminobutyraldehyde dehydrogenase	94	2	0.67	8	10	29
G1SCI0	Q14204	DYNC1H1	Dynein cytoplasmic 1 heavy chain 1	Cytoplasmic dynein 1 heavy chain 1	98	2	0.67	121	75	52

G1SE61	O75369	FLNB	Filamin-B	Filamin-B	97	2	0.67	106	526	61
G1U8C4	H0YKK6	PSME1	Uncharacterized protein	Proteasome activator complex subunit 1	98	3	0.67	2	2	27
G1T004	H0Y987	PGM3	Phosphoacetylglucosamine mutase	Phosphoacetylglucosamine mutase	90	2	0.67	8	18	27
G1SHN4	Q96JB2	COG3	Uncharacterized protein	Conserved oligomeric Golgi complex subunit 3	96	3	0.67	4	4	10
G1T4P7	Q9Y4G6	TLN2	Talin 2	Talin-2	98	2	0.67	19	9	11
G1T1V9	P54652	HSPA2	Uncharacterized protein	Heat shock-related 70 kDa protein 2	98	3	0.67	26	34	58
G1SER8	P07737	PFN1	Profilin	Profilin-1	94	2	0.67	7	133	75
	Q9UHD8	SEPTIN9		Septin-9		4	0.67	10	7	21
G1TAM3		TBCB	CAP-Gly domain-containing protein			1	0.67	2	2	11
	H0Y7A7	CALM2		Calmodulin-2 (Fragment)		4	0.66	7	56	49
G1SY84	Q13409-2	DYNC1I2	WD_REPEATS_REGION domain-containing protein	Isoform 2B of Cytoplasmic dynein 1 intermediate chain 2	92	2	0.66	9	2	29
G1T345		UBR1	E3 ubiquitin-protein ligase			1	0.66	2	2	2
G1T432	Q01518	CAP1	Adenylyl cyclase-associated protein	Adenylyl cyclase-associated protein 1	96	2	0.66	24	173	65
B7NZQ6	P31150	GDI1	Rab GDP dissociation inhibitor	Rab GDP dissociation inhibitor alpha	99	2	0.66	16	18	48
G1T7Z0	P52209	PGD	6-phosphogluconate dehydrogenase, decarboxylating	6-phosphogluconate dehydrogenase, decarboxylating	93	2	0.66	14	16	31
G1SI13		SDC2	Syndecan			1	0.66	3	4	11
P00883	P04075	ALDOA	Fructose-bisphosphate aldolase A	Fructose-bisphosphate aldolase A	98	2	0.66	24	110	76
G1SNE1	Q6DKJ4	NXN	Thioredoxin domain-containing protein	Nucleoredoxin	98	2	0.66	8	23	30
G1TQR0	P12814-3	ACTN1	Uncharacterized protein	Isoform 3 of Alpha-actinin-1	97	3	0.66	59	115	79
G1SCP7	P46940	IQGAP1	Uncharacterized protein	Ras GTPase-activating-like protein IQGAP1	97	3	0.66	75	224	60
G1TNL6	P50479	PDLIM4	Uncharacterized protein	PDZ and LIM domain protein 4	86	3	0.66	7	10	33
G1SL85	A0A1B0GTM3	ASAH1	Uncharacterized protein	Acid ceramidase	81	3	0.66	7	11	27
G1TU12	Q5H907	MAGED2	MAGE domain-containing protein	Melanoma antigen family D, 2, isoform CRA_d	79	2	0.66	5	3	10
G1TRZ2		LAMP1	Lysosomal associated membrane protein 1			1	0.66	5	18	14
	Q96BM9	ARL8A		ADP-ribosylation factor-like protein 8A		4	0.66	7	2	45
G1SCR0	H0YFI1	LAMTOR1	Uncharacterized protein	Ragulator complex protein LAMTOR1 (Fragment)	100	3	0.66	2	3	47
G1U2E3	Q9H444	CHMP4B	Uncharacterized protein	Charged multivesicular body protein 4b	100	3	0.66	7	8	40
G1U304		GALNS	Galactosamine (N-acetyl)-6-sulfatase			1	0.66	2	5	7
G1SPN9	P36405	ARL3	Uncharacterized protein	ADP-ribosylation factor-like protein 3	99	3	0.65	2	3	9
G1U5M4	J3QQS9	SLC16A3	Solute carrier family 16 member 3	Monocarboxylate transporter 4 (Fragment)	92	2	0.65	3	4	6
G1U8T9	Q96CX2	KCTD12	Potassium channel tetramerization domain containing 12	BTB/POZ domain-containing protein KCTD12	97	2	0.65	3	3	17
G1T8C8	A0A087X0R6	SNX12	Sorting nexin 12	Sorting nexin-12	100	2	0.65	2	3	13
G1SHR7	Q9P0J7	KCMF1	C2H2-type domain-containing protein	E3 ubiquitin-protein ligase KCMF1	99	2	0.65	2	3	9
G1U0Q7		SEPT2	Septin-type G domain-containing protein			1	0.65	13	60	54
G1T1X2	A0A087WZF1	LPP	Uncharacterized protein	Lipoma-preferred partner	89	3	0.65	4	5	8
G1T5P0	Q8NDI1	EHBP1	Uncharacterized protein	EH domain-binding protein 1	94	3	0.65	2	4	3
G1SR49		SEPT10	Septin-type G domain-containing protein			1	0.65	6	11	17
G1T8R3	P46939	UTRN	Uncharacterized protein	Utrophin	91	3	0.65	53	62	24
G1SE57	P18085	ARF4	Uncharacterized protein	ADP-ribosylation factor 4	95	3	0.65	10	20	76
G1T0Y9	Q13561	DCTN2	Uncharacterized protein	Dynactin subunit 2	97	3	0.65	10	23	43
G1SDN9	Q9P2B2	PTGFRN	Uncharacterized protein	Prostaglandin F2 receptor negative regulator	91	3	0.65	6	9	8
G1TM35	Q9Y3D6	FIS1	Fission, mitochondrial 1	Mitochondrial fission 1 protein	86	2	0.65	4	4	15
G1T3D7		NANS	AFP-like domain-containing protein			1	0.65	4	6	14
G1SEX8	Q9P299	COPZ2	Clat_adaptor_s domain-containing protein	Coatomer subunit zeta-2	84	2	0.64	4	6	23
U3KMD1	A0A0A0MRI2	SNX6	Vps5 domain-containing protein	Sorting nexin	99	2	0.64	11	112	28
G1TDH8		CLCN5	Chloride channel protein			1	0.64	4	5	13
G1SHK8	O94925	GLS	ANK_REP_REGION domain-containing protein	Glutaminase kidney isoform, mitochondrial	97	2	0.64	21	11	48
G1T5E6	O60749	SNX2	PX domain-containing protein	Sorting nexin-2	98	2	0.64	11	10	28
	O43707	ACTN4		Alpha-actinin-4		4	0.64	52	70	64
	P22392-2	NME2		Isoform 3 of Nucleoside diphosphate kinase B		4	0.64	7	24	35
G1T6N3		NPC2	ML domain-containing protein			1	0.64	3	6	21
G1U723		PGER5	3alpha/17beta/20alpha-hydroxysteroid dehydrogenase			1	0.64	5	5	20
G1SSP0		STRN	WD_REPEATS_REGION domain-containing protein			1	0.64	3	4	11
G1TAB2		GM2A	ML domain-containing protein			1	0.64	4	7	22
G1TUH9	Q14195	DPYSL3	Amidohydro-rel domain-containing protein	Dihydropyrimidinase-related protein 3	98	2	0.64	17	25	38
G1SGL0	Q92629	SGCD	Uncharacterized protein	Delta-sarcoglycan	98	3	0.64	3	5	14
G1T281		SNTA1	Alpha-1-syntrophin			1	0.64	2	6	8

G1TS18		BSDC1	BSD domain containing 1			1	0.64	3	3	13
G1U974	A0A1C7CYX9	DPYSL2	Amidohydro-rel domain-containing protein	Dihydropyrimidinase-related protein 2	98	2	0.64	19	37	51
G1SN85	A0A0D9SEN1	FAP	Uncharacterized protein	Prolyl endopeptidase FAP	95	3	0.64	27	87	40
G1SWR0	H3BP20	HEXA	Beta-hexosaminidase	Beta-hexosaminidase	85	2	0.64	12	16	36
G1SR27	P62993	GRB2	Uncharacterized protein	Growth factor receptor-bound protein 2	100	3	0.63	3	4	22
G1T060	A0A2R8YD50	HSD17B4	Uncharacterized protein	Peroxisomal multifunctional enzyme type 2	89	3	0.63	9	14	21
G1SP54		LTA4H	Leukotriene A(4) hydrolase			1	0.63	4	3	10
	A0A087WY85	UBE2D3		Ubiquitin-conjugating enzyme E2 D3		4	0.63	2	3	20
G1SDR2	P24844	MYL9	Uncharacterized protein	Myosin regulatory light polypeptide 9	99	3	0.63	10	18	80
G1T277	F8VR50	ARPC3	Actin-related protein 2/3 complex subunit 3	Actin-related protein 2/3 complex subunit 3 (Fragment)	100	2	0.63	3	9	17
G1U0Y0	A0A2Q3DQE3	CAMK2G	Protein kinase domain-containing protein	Calcium/calmodulin-dependent protein kinase (CaM kinase) II gamma, isoform CRA_d	91	2	0.63	7	4	23
G1TBY1		CTSB	Pept_C1 domain-containing protein			1	0.63	7	55	30
G1SIT6	Q9BPX5	ARPC5L	Actin-related protein 2/3 complex subunit 5	Actin-related protein 2/3 complex subunit 5-like protein	98	2	0.63	7	9	63
	A0A1B0GV23	CTSD		Cathepsin D		4	0.63	6	21	14
P48738	Q00169	PITPNA	Phosphatidylinositol transfer protein alpha isoform	Phosphatidylinositol transfer protein alpha isoform	99	2	0.63	6	6	24
G1U7S8	F8W6C2	SPATS2L	Uncharacterized protein	SPATS2-like protein (Fragment)	100	3	0.63	4	4	9
G1SQ70	P01023	A2M	Uncharacterized protein	Alpha-2-macroglobulin	78	3	0.63	4	3	3
G1SXE6	Q9Y6G9	DYNC1LI1	Uncharacterized protein	Cytoplasmic dynein 1 light intermediate chain 1	94	3	0.63	9	9	26
G1TK63	P22413	ENPP1	Uncharacterized protein	Ectonucleotide pyrophosphatase/phosphodiesterase family member 1	89	3	0.63	18	25	30
G1T545		BTBD9	Lactoylglutathione lyase			1	0.62	4	6	33
G1U5B3	C9JIZ6	PSAP	Prosaposin	Prosaposin	78	2	0.62	13	47	40
	P21333	FLNA		Filamin-A		4	0.62	105	12	51
G1T1G5	Q9H4A6	GOLPH3	Uncharacterized protein	Golgi phosphoprotein 3	99	3	0.62	5	5	32
	P02533	KRT14		Keratin, type I cytoskeletal 14		4	0.62	5	4	14
G1U0I7	H3BSK9	ATXN2L	Ataxin 2 like	Ataxin-2-like protein (Fragment)	99	2	0.62	5	5	25
	K7EPT8	GFAP		Glial fibrillary acidic protein (Fragment)		4	0.62	3	210	15
G1SEA7	Q13131	PRKAA1	Non-specific serine/threonine protein kinase	5~-AMP-activated protein kinase catalytic subunit alpha-1	97	2	0.62	3	3	8
G1TA40	H0YEP5	SMPD1	Sphingomyelin phosphodiesterase	Sphingomyelin phosphodiesterase (Fragment)	78	2	0.62	5	6	14
G1SW40	E7EMB1	SWAP70	PH domain-containing protein	Switch-associated protein 70	77	2	0.62	7	23	16
	Q7Z406	MYH14		Myosin-14		4	0.62	14	62	8
G1SLH7	P42566	EPS15	Uncharacterized protein	Epidermal growth factor receptor substrate 15	93	3	0.62	4	5	6
G1TKE3	X6RJP6	TAGLN2	Transgelin	Transgelin-2 (Fragment)	77	2	0.62	6	11	40
G1TX63		CDC42BPA	Non-specific serine/threonine protein kinase			1	0.62	4	2	4
G1U411	F8WBG8	DBNL	Uncharacterized protein	Drebrin-like protein	95	3	0.62	9	17	31
G1SN21	P00491	PNP	Purine nucleoside phosphorylase	Purine nucleoside phosphorylase	90	2	0.62	13	26	62
G1SJK7		CYP27A1	Sterol 26-hydroxylase, mitochondrial			1	0.62	2	3	8
G1SNP9	P45954	ACADSB	Acyl-CoA dehydrogenase short/branched chain	Short/branched chain specific acyl-CoA dehydrogenase, mitochondrial	88	2	0.61	18	137	55
G1TUC2	P62633	CNBP	Uncharacterized protein	Cellular nucleic acid-binding protein	100	3	0.61	2	4	13
G1SD48	A0A0G2JLB3	GBA	Glucosylceramidase	Glucosylceramidase	90	2	0.61	5	7	20
P11974-2		PKM	Isoform M2 of Pyruvate kinase PKM			1	0.61	38	440	81
G1SZD6	P27348	YWHAQ	14-3-3 protein theta	14-3-3 protein theta	99	2	0.61	16	144	68
G1T085		SVIL	HP domain-containing protein			1	0.61	4	6	4
G1T2I7	Q9HCJ1	ANKH	Uncharacterized protein	Progressive ankylosis protein homolog	99	3	0.60	3	4	13
G1SE87		ARSE	Arylsulfatase E			1	0.60	3	3	7
G1T512		CNP	2~,3~-cyclic nucleotide 3~ phosphodiesterase			1	0.60	2	3	7
G1SPR5	P16278	GLB1	Glyco_hydro_35 domain-containing protein	Beta-galactosidase	82	2	0.60	15	24	33
G1TC70	O14974	PPP1R12A	Protein phosphatase 1 regulatory subunit	Protein phosphatase 1 regulatory subunit 12A	94	2	0.60	13	33	16
G1SY00	P42858	HTT	Uncharacterized protein	Huntingtin	91	3	0.60	4	3	3
G1TBH6	Q9P0S9	TMEM14C	Uncharacterized protein	Transmembrane protein 14C	92	3	0.60	3	3	66
G1SJ56	P18206	VCL	Uncharacterized protein	Vinculin	99	3	0.60	54	169	59
G1T7T8	P47712	PLA2G4A	Phospholipase A2	Cytosolic phospholipase A2	94	2	0.60	27	134	54
G1SPL3	G3V583	FAM177A1	Uncharacterized protein	Protein FAM177A1 (Fragment)	93	3	0.60	2	2	12
B7NZG7	O60493	SNX3	Sorting nexin 3 (Predicted)	Sorting nexin-3	100	2	0.60	2	4	17
G1SNT1	O75822	EIF3J	Eukaryotic translation initiation factor 3 subunit J	Eukaryotic translation initiation factor 3 subunit J	92	2	0.60	11	15	48
G1SN43	F8W930	IGF2BP2	Uncharacterized protein	Insulin-like growth factor 2 mRNA-binding protein 2	98	3	0.60	7	6	15
G1TYA7	P07195	LDHB	L-lactate dehydrogenase	L-lactate dehydrogenase B chain	100	2	0.60	17	183	58
G1STD5		SUCO	SUN domain containing ossification factor			1	0.59	2	4	3

	Q16658	FSCN1		Fascin		4	0.59	10	9	23
	P51178	PLCD1		1-phosphatidylinositol 4,5-bisphosphate phosphodiesterase delta-1		4	0.59	2	3	3
P21195	P07237	P4HB	Protein disulfide-isomerase	Protein disulfide-isomerase	91	2	0.59	34	603	72
G1T4F9	E9PR44	CRYAB	Alpha-crystallin B chain	Alpha-crystallin B chain (Fragment)	98	2	0.59	6	13	44
G1T013	P48163	ME1	Malic enzyme	NADP-dependent malic enzyme	95	2	0.59	6	18	22
O77708	D6R938	CAMK2D	Calcium/calmodulin-dependent protein kinase type II subunit delta	Calcium/calmodulin-dependent protein kinase (CaM kinase) II delta, isoform CRA e	92	2	0.59	12	12	33
Q6TYA7		GJA1	Gap junction alpha-1 protein			1	0.59	2	4	6
G1SPF5	P18669	PGAM1	Uncharacterized protein	Phosphoglycerate mutase 1	100	3	0.59	13	89	67
G1U2Q7		COL8A1	Collagen alpha-1(VIII) chain			1	0.59	3	4	5
G1SEF9	P17661	DES	IF rod domain-containing protein	Desmin	99	2	0.59	8	8	10
G1TAC4		GLRX	Glutaredoxin-1			1	0.59	3	9	38
G1T7X6	G3XAI2	LAMB1	Uncharacterized protein	Laminin subunit beta-1	91	3	0.59	3	2	3
G1SF47		SEPT11	Septin-type G domain-containing protein			1	0.59	12	14	34
G1U9R8	P06396-2	GSN	Uncharacterized protein	Isoform 2 of Gelsolin	95	3	0.59	19	40	41
G1SXR6	O60282	KIF5C	Kinesin-like protein	Kinesin heavy chain isoform 5C	99	2	0.58	20	11	29
G1U7U3		NME1	Nucleoside diphosphate kinase			1	0.58	7	7	57
G1SG55	O43491	EPB41L2	FERM domain-containing protein	Band 4.1-like protein 2	90	2	0.58	38	91	43
G1SZH6	Q9BZF1	OSBPL8	Oxysterol-binding protein	Oxysterol-binding protein-related protein 8	99	2	0.58	7	10	11
G1SFH5	O00469-2	PLOD2	Fe2OG dioxygenase domain-containing protein		90	2	0.58	30	54	47
G1TN33		SEPT8	Septin-type G domain-containing protein			1	0.58	8	11	24
G1T2K1	O95340	PAPSS2	Uncharacterized protein	Bifunctional 3~-phosphoadenosine 5~-phosphosulfate synthase 2	94	3	0.58	5	9	14
G1U5Q7		ARPC4	Actin-related protein 2/3 complex subunit 4			1	0.58	7	35	46
G1SL41		GUSB	Beta-glucuronidase			1	0.57	11	20	30
G1SZ47	P62266	RPS23	Uncharacterized protein	40S ribosomal protein S23	100	3	0.57	6	105	48
	P68371	TUBB4B		Tubulin beta-4B chain		4	0.57	22	23	71
G1SX37	Q9Y281	CFL2	Cofilin 2	Cofilin-2	91	2	0.57	8	13	55
G1T156	Q9HB40	SCPEP1	Carboxypeptidase	Retinoid-inducible serine carboxypeptidase	86	2	0.57	11	21	26
G1SUV2		CAMLG	Calcium signal-modulating cyclophilin ligand			1	0.57	2	5	16
G1SWW4		SYNPO	Synaptopodin			1	0.57	3	6	6
G1SZP0	O60784	TOM1	Uncharacterized protein	Target of Myb protein 1	88	3	0.57	11	25	44
Q09YN6	C9JKI3	CAV1	Caveolin-1	Caveolin (Fragment)	96	2	0.57	3	4	21
G1SLX0	G3V4P8	GMFB	Glia maturation factor	Glia maturation factor beta (Fragment)	99	2	0.57	3	5	30
	P32119	PRDX2		Peroxiredoxin-2		4	0.56	5	10	31
G1SYX4	P50281	MMP14	Matrix metalloproteinase-14	Matrix metalloproteinase-14	95	2	0.56	5	6	11
G1ST05	Q15120	PDK3	Protein-serine/threonine kinase	[Pyruvate dehydrogenase (acetyl-transferring)] kinase isozyme 3, mitochondrial	99	2	0.56	6	9	26
G1SNC4	Q5T9B7	AK1	Adenylate kinase isoenzyme 1	Adenylate kinase isoenzyme 1	94	2	0.56	2	4	12
G1U6H4	O95865	DDAH2	Uncharacterized protein	N(G),N(G)-dimethylarginine dimethylaminohydrolase 2	97	3	0.56	9	10	56
G1SPD1		MTPN	ANK_REP_REGION domain-containing protein			1	0.56	2	5	32
G1SG54	A0A0U1RQT1	ACAP2	Arf-GAP with coiled-coil, ANK repeat and PH domain-containing protein 2	Arf-GAP with coiled-coil, ANK repeat and PH domain-containing protein 2 (Fragment)	99	2	0.55	4	3	11
G1SXK6	Q9NR12	PDLIM7	PDZ and LIM domain 7	PDZ and LIM domain protein 7	92	2	0.55	8	24	26
G1U9R0	A8MX94	GSTP1	Uncharacterized protein	Glutathione S-transferase P	71	3	0.55	10	86	49
G1TMM0	A0A087WW43	ITIH3	Inter-alpha-trypsin inhibitor heavy chain H3	Inter-alpha-trypsin inhibitor heavy chain H3	91	2	0.55	4	11	5
G1TKH3		SOD1	Superoxide dismutase [Cu-Zn]			1	0.55	3	107	35
G1ST64		LMOD1	Leiomodin 1			1	0.55	5	6	9
G1T0H8	Q01813	PFKP	ATP-dependent 6-phosphofructokinase	ATP-dependent 6-phosphofructokinase, platelet type	93	2	0.55	22	34	42
G1SKT2	E9PGF6	PHLDB2	PH domain-containing protein	Pleckstrin homology-like domain family B member 2	65	2	0.55	8	8	8
G1T7A8	Q9Y646	CPQ	Peptidase_M28 domain-containing protein	Carboxypeptidase Q	87	2	0.55	2	2	4
G1SMK8	O95816	BAG2	BAG domain-containing protein	BAG family molecular chaperone regulator 2	98	2	0.55	6	9	29
G1T823	H3BT58	COTL1	Coactosin like F-actin binding protein 1	Coactosin-like protein	96	2	0.55	3	9	27
	H7BZL4	GPC1		Glypican-1 (Fragment)		4	0.55	5	5	29
	Q6WCQ1	MPRIP		Myosin phosphatase Rho-interacting protein		4	0.54	16	2	20
	Q6NZI2	CAVIN1		Caveolae-associated protein 1		4	0.54	7	10	19
G1TLL6	Q9UNH6	SNX7	PX domain-containing protein	Sorting nexin-7	96	2	0.54	4	3	15
G1TER3	A0A2R8Y5M6	BCAP31	Uncharacterized protein	B-cell receptor-associated protein 31	81	3	0.54	11	22	53
G1SM64	Q5T985	ITIH2	Uncharacterized protein	Inter-alpha-trypsin inhibitor heavy chain H2	85	3	0.53	5	4	8
G1THW3	A0A3B3IUC0	ITM2B	BRICHOS domain-containing protein	Integral membrane protein 2B	96	2	0.53	5	10	31
G1SIB9	P11216	PYGB	Alpha-1,4 glucan phosphorylase	Glycogen phosphorylase, brain form	95	2	0.53	31	23	45

G1TN08	P37235	HPCAL1	Uncharacterized protein	Hippocalcin-like protein 1	99	3	0.53	6	5	34
G1SDZ0		CTSA	Carboxypeptidase			1	0.53	5	6	11
G1STY4		PLBD2	Phospholipase B-like			1	0.53	5	14	16
G1SRL4		NAGA	Alpha-galactosidase			1	0.53	14	30	47
G1T0J3	E9PDF6	MYO1B	Uncharacterized protein	Unconventional myosin-Ib	96	3	0.53	17	25	22
G1SNT8	P08133	ANXA6	Annexin	Annexin A6	96	2	0.53	33	61	48
G1TME7		CSTB	Cystatin B			1	0.52	3	4	36
G1SE41	Q9NWM8	FKBP14	Peptidylprolyl isomerase	Peptidyl-prolyl cis-trans isomerase FKBP14	94	2	0.52	4	6	23
G1SY88	P55263	ADK	PfkB domain-containing protein	Adenosine kinase	98	2	0.52	8	13	38
G1SX70	Q9NRW1	RAB6B	Uncharacterized protein	Ras-related protein Rab-6B	100	3	0.52	7	2	45
G1TXW6		GPNMB	PKD domain-containing protein			1	0.52	2	5	5
G1SHB9	P40121	CAPG	Uncharacterized protein	Macrophage-capping protein	94	3	0.51	9	19	44
G1TV31	O15511	ARPC5	Actin-related protein 2/3 complex subunit 5	Actin-related protein 2/3 complex subunit 5	95	2	0.51	8	39	54
	Q9NP61	ARFGAP3		ADP-ribosylation factor GTPase-activating protein 3		4	0.51	2	2	4
G1SUZ7		ARSA	Sulfatase domain-containing protein			1	0.51	4	3	18
G1SQ10	Q9NQT8	KIF13B	Uncharacterized protein	Kinesin-like protein KIF13B	89	3	0.51	13	4	9
G1T473	F8VQE1	LIMA1	LIM zinc-binding domain-containing protein	LIM domain and actin-binding protein 1	85	2	0.51	22	37	38
	P06753-5	TPM3		Isoform 5 of Tropomyosin alpha-3 chain		4	0.51	21	22	62
G1SP27	Q16204	CCDC6	Uncharacterized protein	Coiled-coil domain-containing protein 6	93	3	0.51	14	30	31
G1T9N3	B5MBZ0	EML4	Uncharacterized protein	Echinoderm microtubule-associated protein-like 4	92	3	0.51	4	7	5
P62943	P62942	FKBP1A	Peptidyl-prolyl cis-trans isomerase FKBP1A	Peptidyl-prolyl cis-trans isomerase FKBP1A	100	2	0.51	2	6	25
	P33241	LSP1		Lymphocyte-specific protein 1		4	0.51	3	5	9
G1TP25	Q5VU77	UBAP2L	Ubiquitin associated protein 2 like	Ubiquitin-associated protein 2-like (Fragment)	95	2	0.51	3	4	4
G1SDH3	E9PIG4	PRCP	Uncharacterized protein	Lysosomal Pro-X carboxypeptidase (Fragment)	89	3	0.50	8	13	30
G1T2Q8	Q96M27	PRRC1	NTPase_I-T domain-containing protein	Protein PRRC1	90	2	0.50	7	14	22
G1SI83	Q99584	S100A13	S_100 domain-containing protein	Protein S100-A13	91	2	0.50	3	5	27
G1SDL3	P54802	NAGLU	Uncharacterized protein	Alpha-N-acetylglucosaminidase	88	3	0.50	15	18	29
G1TCM9	Q9H299	SH3BGRL3	SH3 domain-binding glutamic acid-rich-like protein	SH3 domain-binding glutamic acid-rich-like protein 3	100	2	0.50	2	6	52
G1T705	P00387	CYB5R3	NADH-cytochrome b5 reductase	NADH-cytochrome b5 reductase 3	90	2	0.50	12	46	57
G1TK30		CSAD	Cysteine sulfinic acid decarboxylase			1	0.50	4	9	18
P00939	P60174	TPI1	Triosephosphate isomerase	Triosephosphate isomerase	93	2	0.50	15	59	72
G1SVB5	J3QRN6	MYO1D	Uncharacterized protein	Unconventional myosin-Id	95	3	0.49	15	19	20
Q8WN94		DBI	Acyl-CoA-binding protein			1	0.49	3	4	39
	P35908	KRT2		Keratin, type II cytoskeletal 2 epidermal		4	0.49	10	7	25
P35748	P35749	MYH11	Myosin-11	Myosin-11	97	2	0.49	42	39	29
G1SDP2	Q96AY3	FKBP10	FK506-binding protein	Peptidyl-prolyl cis-trans isomerase FKBP10	93	2	0.49	17	23	40
G1T8Z0	P30041	PRDX6	Thioredoxin domain-containing protein	Peroxiredoxin-6	95	2	0.49	15	33	64
G1TBJ4	Q92597	NDRG1	Uncharacterized protein	Protein NDRG1	96	3	0.49	3	4	17
G1SQR6	Q15417	CNN3	Calponin	Calponin-3	98	2	0.48	15	553	55
G1T4N8	Q8TF42	UBASH3B	Uncharacterized protein	Ubiquitin-associated and SH3 domain-containing protein B	98	3	0.48	2	2	5
G1SQF2	O43237	DYNC1LI2	Uncharacterized protein	Cytoplasmic dynein 1 light intermediate chain 2	98	3	0.48	9	13	37
G1SZX4	Q14571	ITPR2	Uncharacterized protein	Inositol 1,4,5-trisphosphate receptor type 2	97	3	0.48	13	10	9
G1T6L5	O75368	SH3BGRL	Uncharacterized protein	SH3 domain-binding glutamic acid-rich-like protein	98	3	0.48	5	10	31
G1SFG6	A0A0A0MTS2	GPI	Glucose-6-phosphate isomerase	Glucose-6-phosphate isomerase (Fragment)	93	2	0.48	22	122	56
G1STV0		CAST	Calpastatin			1	0.47	12	20	21
G1SH05	P07437	TUBB	Tubulin beta chain	Tubulin beta chain	100	2	0.47	23	122	71
G1SWS9	P08670	VIM	IF rod domain-containing protein	Vimentin	97	2	0.47	50	###	88
G1TMV1	P60981	DSTN	ADF-H domain-containing protein	Destrin	100	2	0.47	9	71	58
G1SYE0		ABHD14B	AB hydrolase-1 domain-containing protein			1	0.47	4	7	21
G1SNP7	E9PGC8	MAP1A	Uncharacterized protein	Microtubule-associated protein 1A	79	3	0.46	6	4	4
G1SW57	P21589	NT5E	Uncharacterized protein	5~-nucleotidase	89	3	0.46	10	12	21
G1TER4	P33897	ABCD1	Uncharacterized protein	ATP-binding cassette sub-family D member 1	94	3	0.46	4	3	8
G1T8B5	Q9H1E3	NUCKS1	Nuclear casein kinase and cyclin dependent kinase substrate 1	Nuclear ubiquitous casein and cyclin-dependent kinase substrate 1	94	2	0.46	2	3	9
G1TBQ6	O95292	VAPB	VAMP associated protein B and C	Vesicle-associated membrane protein-associated protein B/C	90	2	0.45	3	3	17
G1T188	Q9UIJ7	AK3	GTP:AMP phosphotransferase AK3, mitochondrial	GTP:AMP phosphotransferase AK3, mitochondrial	93	2	0.45	11	22	57
G1SYL5	Q7Z4N8	P4HA3	Fe2OG dioxygenase domain-containing protein	Prolyl 4-hydroxylase subunit alpha-3	92	2	0.45	15	38	43
G1TE78	Q15121	PEA15	DED domain-containing protein	Astrocytic phosphoprotein PEA-15	100	2	0.44	5	4	43

G1SZJ7	A0A2R8YEC9	MANBA	Uncharacterized protein	Beta-mannosidase	75	3	0.44	7	8	11
G1SYN4		PTX3	LamGL domain-containing protein			1	0.44	11	20	29
G1TA83	P09525	ANXA4	Annexin	Annexin A4	94	2	0.43	13	25	51
G1U7J6		PRKCA	Protein kinase C			1	0.43	2	2	6
G1T7Z6	P00558	PGK1	Phosphoglycerate kinase	Phosphoglycerate kinase 1	99	2	0.43	26	87	72
G1SY87		CD200	Ig-like domain-containing protein			1	0.42	2	4	7
G1TNJ2	Q15942	ZYX	Uncharacterized protein	Zyxin	87	3	0.42	6	21	15
G1SXU2	Q96HC4	PDLIM5	Uncharacterized protein	PDZ and LIM domain protein 5	88	3	0.42	10	28	22
G1TRY5	P13797	PLS3	Uncharacterized protein	Plastin-3	100	3	0.42	37	141	77
	B4DDF4	CNN2		Calponin		4	0.41	5	5	18
G1SHL8	P42224	STAT1	Signal transducer and activator of transcription	Signal transducer and activator of transcription 1-alpha/beta	95	2	0.41	13	18	27
G1SVE3		PRUNE2	CRAL-TRIO domain-containing protein			1	0.41	4	4	2
G1T315	A0A2R8Y2R1	SGCE	CADG domain-containing protein	Epsilon-sarcoglycan (Fragment)	88	2	0.41	2	6	7
G1SPY1	E9PGM4	GBE1	Aamy domain-containing protein	1,4-alpha-glucan-branching enzyme	93	2	0.40	21	37	42
G1SYJ4	P06733	ENO1	Uncharacterized protein	Alpha-enolase	96	3	0.40	24	133	72
G1TAJ3	P00338	LDHA	L-lactate dehydrogenase	L-lactate dehydrogenase A chain	94	2	0.40	21	217	66
P58776	A7XZE4	TPM2	Tropomyosin beta chain	Beta tropomyosin isoform	94	2	0.39	24	26	63
G1T1V0	P13645	KRT10	IF rod domain-containing protein	Keratin, type I cytoskeletal 10	95	2	0.39	22	61	51
G1SRR2	E9PMP7	LMO7	Uncharacterized protein	LIM domain only protein 7 (Fragment)	70	3	0.39	30	42	24
G1SKQ9		SEPT6	Septin-type G domain-containing protein			1	0.38	8	3	22
G1SSW2	B4E0Y9	STK26	Protein kinase domain-containing protein	Serine/threonine-protein kinase 26	95	2	0.38	5	3	19
	P17858	PFKL		ATP-dependent 6-phosphofructokinase, liver type		4	0.38	8	11	12
G1U634	P08473	MME	Neprilysin	Neprilysin	94	2	0.38	41	6	61
G1SRD9	A0A1C7CYX8	FAM107B	Uncharacterized protein	Protein FAM107B (Fragment)	96	3	0.38	2	2	16
G1TN29	Q86UU1	PHLDB1	Pleckstrin homology like domain family B member 1	Pleckstrin homology-like domain family B member 1	89	2	0.37	8	3	6
G1SUY3	F8VQR7	CSRP2	Uncharacterized protein	Cysteine and glycine-rich protein 2	100	3	0.37	6	15	38
G1SW77	Q8NC51	SERBP1	HABP4_PAI-RBP1 domain-containing protein	Plasminogen activator inhibitor 1 RNA-binding protein	99	2	0.36	7	15	19
	P51911	CNN1		Calponin-1		4	0.36	7	8	33
G1T2C4	Q01995	TAGLN	Transgelin	Transgelin	99	2	0.36	11	170	58
G1T387	Q14247	CTTN	Cortactin	Src substrate cortactin	88	2	0.35	5	2	13
G1TYY5	Q14847	LASP1	LIM and SH3 domain protein 1	LIM and SH3 domain protein 1	94	2	0.35	6	9	23
G1SPQ9	O94875	SORBS2	Uncharacterized protein	Sorbin and SH3 domain-containing protein 2	90	3	0.35	17	23	19
G1SYD2		AK4	Adenylate kinase 4, mitochondrial			1	0.34	6	8	41
G1SKS8	E7EVA0	MAP4	Microtubule-associated protein	Microtubule-associated protein	85	2	0.34	25	47	30
	Q9Y2V2	CARHSP1		Calcium-regulated heat-stable protein 1		4	0.33	2	2	18
G1TBU2		KIAA1217	KIAA1217			1	0.33	2	2	2
G1T5T8	O00151	PDLIM1	Uncharacterized protein	PDZ and LIM domain protein 1	92	3	0.33	14	29	60
G1T5B6	P12107	COL11A1	Fibrillar collagen NC1 domain-containing protein	Collagen alpha-1(XI) chain	98	2	0.32	29	58	24
	P32418	SLC8A1		Sodium/calcium exchanger 1		4	0.32	2	3	4
G1SUI9	E7EX44	CALD1	Uncharacterized protein	Caldesmon	84	3	0.32	24	103	39
	Q09666	AHNAK		Neuroblast differentiation-associated protein AHNAK		4	0.29	33	48	6
G1SW82	H0Y9Y3	SYNPO2	PDZ domain-containing protein	Synaptopodin-2 (Fragment)	80	2	0.29	3	4	4
G1T8J0	P02461	COL3A1	Uncharacterized protein	Collagen alpha-1(III) chain	92	3	0.29	17	30	15
G1SZ00	P21291	CSRP1	Uncharacterized protein	Cysteine and glycine-rich protein 1	98	3	0.28	9	2	48
G1TA72		HS1BP3	PX domain-containing protein			1	0.28	2	2	6
G1SMS2	Q8WX93	PALLD	Uncharacterized protein	Palladin	87	3	0.20	20	39	20

A. Percent sequence Identity (rounded to the nearest integer) between the indicated rabbit and human proteins from blastp analysis of the rabbit and human UniProt databases.
B. Database Identification Categories: Category 1, Characterized in the rabbit database only; Category 2, Characterized in both the rabbit and human databases; Category 3, Uncharacterized in the rabbit database but characterized in the human database; Category 4, Characterized in the human database only.
C. Number of unique peptides quantified.
D. Number of peptide intensities summed for protein quantitation.

Supplemental Table S3
Myofibroblasts from Rabbit Cornea & Bone Marrow
Relative Protein Abundance, Rabbit 26
Orbitrap Fusion Lumos Tribrid LC MS/MS iTRAQ Results

Total Proteins Quantified = 2405; Median Protein iTRAQ Ratio =0.997; Mean Protein iTRAQ Ratio = 1.000; Standard Deviation (SD) = 0.439
Yellow = 1SD from the mean; Brown = 2SD from the mean; Green = p ≤ 0.05

Rabbit Accession UniProt	Human Accession UniProt	Gene Symbol	Rabbit Protein Description	Human Protein Description	% identity[A]	Database Identification category[B]	Linear Ratio Cornea/Bone marrow	Peptides[C]	N[D]	% sequence coverage
G1SIW5	Q92820	GGH	Folate gamma-glutamyl hydrolase	Gamma-glutamyl hydrolase	82	2	8.37	6	15	28
G1U4P8	Q9BXN1	ASPN	LRRNT domain-containing protein	Asporin	88	2	5.68	5	6	19
	A0A2R8Y7G9	H3.Y		Histone domain-containing protein		4	5.45	2	3	29
	P02765	AHSG		Alpha-2-HS-glycoprotein		4	5.30	2	2	5
G1SZ44	B8ZZL8	HSPE1	Uncharacterized protein	10 kDa heat shock protein, mitochondrial	100	3	4.67	7	7	58
	A0A2R8Y7C0	HBA2		Hemoglobin subunit alpha (Fragment)		4	4.57	3	2	23
G1SDL7		ARG2	Arginase			1	4.55	2	2	8
G1SDA2	P29762	CRABP1	FABP domain-containing protein	Cellular retinoic acid-binding protein 1	99	2	4.30	5	19	41
G1SVK5	P26447	S100A4	Protein S100	Protein S100-A4	98	2	4.13	4	4	36
G1TKC9	Q15582	TGFBI	Transforming growth factor-beta-induced protein ig-h3	Transforming growth factor-beta-induced protein ig-h3	93	2	3.93	5	6	16
G1TUE1		ATP1B1	Sodium/potassium-transporting ATPase subunit beta			1	3.87	4	5	15
G1TX53		NDUFA8	NADH dehydrogenase [ubiquinone] 1 alpha subcomplex subunit 8			1	3.70	2	2	9
G1U0A4	P21810	BGN	Biglycan	Biglycan	94	2	3.60	12	17	46
G1TKV4		HIST2H3D	Histone H3			1	3.60	7	30	52
G1TW43	J3QSU6	TNC	Uncharacterized protein	Tenascin	74	3	3.40	6	8	3
G1T3Y8	P10809	HSPD1	Uncharacterized protein	60 kDa heat shock protein, mitochondrial	99	3	3.36	34	57	75
O19105		SLC1A5	Neutral amino acid transporter B(0)			1	3.34	4	6	14
G1SXR1		PRELP	LRRNT domain-containing protein			1	3.12	4	6	17
G1T594	G0XQ39	STIM1	Stromal interaction molecule 1	STIM1L	98	2	3.12	2	3	3
G1TAK1		COA3	Coiled-coil_56 domain-containing protein			1	3.05	2	5	19
G1T2X0	Q99541	PLIN2	Perilipin	Perilipin-2	88	2	2.94	9	15	28
Q28888		DCN	Decorin			1	2.92	4	3	16
G1SP97	P51884	LUM	Lumican	Lumican	91	2	2.88	4	6	19
G1T9F6		NDUFA6	NADH:ubiquinone oxidoreductase subunit A6			1	2.80	2	2	19
G1SLF6	P25205	MCM3	DNA helicase	DNA replication licensing factor MCM3	97	2	2.77	2	2	4
P98049		MT-CO2	Cytochrome c oxidase subunit 2			1	2.76	3	5	20
G1TSS7	P05106	ITGB3	Integrin beta	Integrin beta-3	95	2	2.73	5	5	9
A0A140TAV6		HBB2	Globin A1			1	2.70	2	2	13
G1T3P4	Q9NRL2	BAZ1A	Bromodomain adjacent to zinc finger domain 1A	Bromodomain adjacent to zinc finger domain protein 1A	89	2	2.62	3	2	2
G1SGP1	P31930	UQCRC1	Uncharacterized protein	Cytochrome b-c1 complex subunit 1, mitochondrial	93	3	2.62	12	21	42
G1T9I6	A8MZB2	NAA20	N-acetyltransferase domain-containing protein	N-acetyltransferase 5 (ARD1 homolog, S. cerevisiae), isoform CRA_a	92	2	2.60	2	2	17
G1T7D1		HIST1H1C	H15 domain-containing protein			1	2.59	10	23	33
G1T017		SLC1A4	Solute carrier family 1 member 4			1	2.54	3	6	9
G1SQ07		DTYMK	Thymidylate_kin domain-containing protein			1	2.54	2	2	10
G1SXW0	P43304	GPD2	Glycerol-3-phosphate dehydrogenase	Glycerol-3-phosphate dehydrogenase, mitochondrial	96	2	2.53	16	31	34
G1TZQ6		NDUFA10	NADH dehydrogenase [ubiquinone] 1 alpha subcomplex subunit 10, mitochondrial			1	2.52	4	8	19
G1T0I5	Q5JRX3	PITRM1	M16C_associated domain-containing protein	Presequence protease, mitochondrial	89	2	2.52	27	46	37
	P16104	H2AFX		Histone H2AX		4	2.51	5	24	53
G1SJW7	J3QLE5	SNRPN	Small nuclear ribonucleoprotein-associated protein	Small nuclear ribonucleoprotein-associated protein N (Fragment)	100	2	2.50	6	10	19
G1ST69	P20700	LMNB1	Uncharacterized protein	Lamin-B1	98	3	2.50	19	37	42
G1SW67	O75367	H2AFY	Core histone macro-H2A	Core histone macro-H2A.1	91	2	2.49	13	3	54
G1SG11		COX4I1	Cytochrome c oxidase subunit 4 isoform 1, mitochondrial			1	2.48	3	3	21
G1T5H0	A0A3B3IU24	HTRA1	PDZ domain-containing protein	Serine protease HTRA1	94	2	2.41	2	3	10
G1TID3		ITPRIP	Inositol 1,4,5-trisphosphate receptor interacting protein			1	2.37	2	3	5
G1T4M2	O60264	SMARCA5	Uncharacterized protein	SWI/SNF-related matrix-associated actin-dependent regulator of chromatin subfamily A member 5	100	3	2.36	5	10	8
G1SUY2	P05091	ALDH2	Aldedh domain-containing protein	Aldehyde dehydrogenase, mitochondrial	91	2	2.36	12	21	36
G1SJF4	A0A0A0MR51	FADS1	Fatty acid desaturase 1	Acyl-CoA (8-3)-desaturase	93	2	2.35	3	5	6
G1SFC6	A0A087WV29	NAT10	RNA cytidine acetyltransferase	RNA cytidine acetyltransferase	96	2	2.34	6	4	11
G1TD26	D6RAA6	TMEM33	Uncharacterized protein	Transmembrane protein 33 (Fragment)	99	3	2.31	3	6	12
G1TXA3	P82921	MRPS21	Uncharacterized protein	28S ribosomal protein S21, mitochondrial	93	3	2.30	3	3	38
G1TL80	Q9Y3B4	SF3B6	RRM domain-containing protein	Splicing factor 3B subunit 6	100	2	2.30	2	6	21
G1TLW3	J3KTA4	DDX5	Uncharacterized protein	Probable ATP-dependent RNA helicase DDX5	96	3	2.29	21	30	42
G1SMB3	Q9NV31	IMP3	S4 RNA-binding domain-containing protein	U3 small nucleolar ribonucleoprotein protein IMP3	99	2	2.28	2	2	15
G1TDN6		KRT5	IF rod domain-containing protein			1	2.28	5	2	8

G1SSJ7	P35232	PHB	PHB domain-containing protein	Prohibitin	100	2	2.27	17	51	81
G1SJY0		MRPS5	S5 DRBM domain-containing protein			1	2.26	4	4	12
G1SQ52	P52788	SMS	PABS domain-containing protein	Spermine synthase	99	2	2.25	3	7	18
G1SE30		EPS8	SH3 domain-containing protein			1	2.24	2	4	5
G1T2K5	I3L1L3	MYBBP1A	Uncharacterized protein	Myb-binding protein 1A (Fragment)	69	3	2.23	34	94	37
G1SNX5	B4DY09	ILF2	Interleukin enhancer binding factor 2	Interleukin enhancer-binding factor 2	100	2	2.22	17	74	58
G1SF08	C9K025	RPL35A	Uncharacterized protein	60S ribosomal protein L35a (Fragment)	99	3	2.22	3	2	23
G1SNS1	Q14692	BMS1	Bms1-type G domain-containing protein	Ribosome biogenesis protein BMS1 homolog	88	2	2.20	5	5	7
U3KMH9	P40926	MDH2	Malate dehydrogenase	Malate dehydrogenase, mitochondrial	94	2	2.19	15	67	60
G1SR13	Q9UJZ1	STOML2	PHB domain-containing protein	Stomatin-like protein 2, mitochondrial	96	2	2.19	15	30	54
G1SXI9		COX6B1	Cytochrome c oxidase subunit			1	2.19	2	3	29
G1TDA7	Q99459	CDC5L	Uncharacterized protein	Cell division cycle 5-like protein	99	3	2.18	3	2	4
G1SI26	Q4VC31	CCDC58	Uncharacterized protein	Coiled-coil domain-containing protein 58	95	3	2.18	3	7	26
G1T3Y0	P98082	DAB2	PID domain-containing protein	Disabled homolog 2	90	2	2.16	5	6	9
G1SEF1		NDUFC2	NADH dehydrogenase [ubiquinone] 1 subunit C2			1	2.15	2	2	19
G1SPY4		ASRGL1	Asparaginase like 1			1	2.14	3	4	11
G1T1B6	Q16832	DDR2	Uncharacterized protein	Discoidin domain-containing receptor 2	97	3	2.14	5	6	9
G1SLK6	J3KMX2	SMARCD2	SWIB domain-containing protein	SWI/SNF-related matrix-associated actin-dependent regulator of chromatin subfamily D member 2	98	2	2.13	3	3	10
G1SIJ7	Q9NR30	DDX21	Uncharacterized protein	Nucleolar RNA helicase 2	89	3	2.12	16	33	24
G1SCY8	Q9UKD2	MRTO4	Ribosome assembly factor mrt4	mRNA turnover protein 4 homolog	96	2	2.12	2	3	10
G1SIM3	O95831	AIFM1	Uncharacterized protein	Apoptosis-inducing factor 1, mitochondrial	96	3	2.12	8	12	22
P12345	P00505	GOT2	Aspartate aminotransferase, mitochondrial	Aspartate aminotransferase, mitochondrial	94	2	2.12	18	96	46
G1TES6	Q99714	HSD17B10	Uncharacterized protein	3-hydroxyacyl-CoA dehydrogenase type-2	92	3	2.11	13	68	84
G1T8M9	P42704	LRPPRC	PPR_long domain-containing protein	Leucine-rich PPR motif-containing protein, mitochondrial	81	2	2.11	51	90	47
U3KMD4	Q8IYU8	MICU2	Uncharacterized protein	Calcium uptake protein 2, mitochondrial	83	3	2.11	5	3	25
	Q9NTI5-2	PDS5B		Isoform 2 of Sister chromatid cohesion protein PDS5 homolog B		4	2.10	2	2	2
G1T359	P28331	NDUFS1	Uncharacterized protein	NADH-ubiquinone oxidoreductase 75 kDa subunit, mitochondrial	98	3	2.10	18	30	37
G1T1P3	Q9H845	ACAD9	Uncharacterized protein	Acyl-CoA dehydrogenase family member 9, mitochondrial	88	3	2.10	9	15	25
	H0Y2W2	ATAD3A		ATPase family AAA domain-containing protein 3A (Fragment)		4	2.10	5	10	12
G1SLR8	Q9NZI8	IGF2BP1	Uncharacterized protein	Insulin-like growth factor 2 mRNA-binding protein 1	99	3	2.10	3	2	7
G1TWL0	P22626	HNRNPA2B1	Uncharacterized protein	Heterogeneous nuclear ribonucleoproteins A2/B1	99	3	2.09	10	25	28
A0A140TAV7	J3KPF3	SLC3A2	4F2 cell-surface antigen heavy chain	4F2 cell-surface antigen heavy chain	81	2	2.09	20	113	57
G1T380	Q02388	COL7A1	Uncharacterized protein	Collagen alpha-1(VII) chain	87	3	2.09	16	24	8
G1U2E6	J3KPX7	PHB2	PHB domain-containing protein	Prohibitin-2	99	2	2.08	17	115	61
	O96008	TOMM40		Mitochondrial import receptor subunit TOM40 homolog		4	2.08	9	15	37
G1SXR7		SLIRP	RRM domain-containing protein			1	2.08	3	7	34
G1T5I0		SLC7A1	Solute carrier family 7 member 1			1	2.07	4	12	11
G1SE74	Q7KZ85	SUPT6H	Transcription elongation factor spt6	Transcription elongation factor SPT6	99	2	2.07	5	6	4
G1SWD1	M0QXL5	FBL	Fibrillarin	rRNA 2~-O-methyltransferase fibrillarin (Fragment)	97	2	2.06	10	65	66
G1SZE8		UTP20	DRIM domain-containing protein			1	2.06	7	12	4
G1TAR0		RPF2	Brix domain-containing protein			1	2.06	3	4	14
	Q9Y2Z2	MTO1		Protein MTO1 homolog, mitochondrial		4	2.05	2	3	4
G1TRL8	J3QLI9	SNRPD1	Small nuclear ribonucleoprotein Sm D1	Small nuclear ribonucleoprotein Sm D1	100	2	2.05	3	11	28
G1TZN7		COX5A	Cytochrome c oxidase subunit 5A			1	2.04	2	2	7
G1TRS0		DKC1	PUA domain-containing protein			1	2.04	5	7	14
G1TEN4	Q9HD33	MRPL47	Uncharacterized protein	39S ribosomal protein L47, mitochondrial	79	3	2.04	5	7	19
G1TX84	Q9Y5J1	UTP18	WD_REPEATS_REGION domain-containing protein	U3 small nucleolar RNA-associated protein 18 homolog	89	2	2.04	4	4	12
G1SGC2	P0C0S5	H2AFZ	Histone H2A	Histone H2A.Z	98	2	2.04	5	18	54
G1SI79	P51991	HNRNPA3	Uncharacterized protein	Heterogeneous nuclear ribonucleoprotein A3	100	3	2.04	8	22	25
G1SL97	Q15397	PUM3	PUM-HD domain-containing protein	Pumilio homolog 3	91	2	2.03	6	8	16
G1TNV7	O75600	GCAT	Glycine C-acetyltransferase	2-amino-3-ketobutyrate coenzyme A ligase, mitochondrial	94	2	2.03	7	7	27
G1TB57		MRPS35	MRP-S28 domain-containing protein			1	2.03	3	4	16
G1TD41	G8JLB6	HNRNPH1	Uncharacterized protein	Heterogeneous nuclear ribonucleoprotein H	98	3	2.03	12	11	39
G1STC6		WDR3	WD_REPEATS_REGION domain-containing protein			1	2.02	3	4	5
P14519	P34897	SHMT2	Serine hydroxymethyltransferase, mitochondrial	Serine hydroxymethyltransferase, mitochondrial	95	2	2.02	22	159	51
G1SRF7	P38646	HSPA9	Uncharacterized protein	Stress-70 protein, mitochondrial	99	3	2.02	36	431	62
G1SYC5	Q9NQZ2	UTP3	Sas10 domain-containing protein	Something about silencing protein 10	82	2	2.01	3	4	8
G1T9U7	Q8TD30	GPT2	Glutamic--pyruvic transaminase 2	Alanine aminotransferase 2	97	2	2.01	5	2	21
G1TKJ4	P61601	NCALD	Uncharacterized protein	Neurocalcin-delta	100	3	2.01	6	8	38
G1SUP9	P46087	NOP2	SAM_MT_RSMB_NOP domain-containing protein	Probable 28S rRNA (cytosine(4447)-C(5))-methyltransferase	77	2	2.00	6	6	12

G1SXN0		PES1	Pescadillo homolog			1	2.00	2	3	6
G1TZB9	Q13595	TRA2A	RRM domain-containing protein	Transformer-2 protein homolog alpha	100	2	1.99	3	2	16
G1TM60		NDUFA9	Epimerase domain-containing protein			1	1.99	6	11	20
G1THH7		SUN2	SUN domain-containing protein			1	1.99	7	10	16
G1ST81	Q9P035	HACD3	Very-long-chain (3R)-3-hydroxyacyl-CoA dehydratase	Very-long-chain (3R)-3-hydroxyacyl-CoA dehydratase 3	96	2	1.99	4	7	18
G1SJ30		WDR43	WD_REPEATS_REGION domain-containing protein			1	1.98	2	2	4
G1T7H4	Q8TDN6	BRIX1	Brix domain-containing protein	Ribosome biogenesis protein BRX1 homolog	93	2	1.98	4	6	13
O79428		MT-ND2	NADH-ubiquinone oxidoreductase chain 2			1	1.98	2	2	10
G1SZ76	P05455	SSB	Lupus La protein homolog	Lupus La protein	92	2	1.98	16	30	41
G1T890	Q8NE86	MCU	MCU domain-containing protein	Calcium uniporter protein, mitochondrial	98	2	1.98	10	19	33
G1SU13	Q5VW52	GPAM	Glycerol-3-phosphate acyltransferase 1, mitochondrial	Glycerol-3-phosphate acyltransferase 1, mitochondrial	94	2	1.97	3	7	8
	I3L3B0	C1QBP		Complement component 1 Q subcomponent-binding protein, mitochondrial		4	1.97	2	21	32
G1SZK8	P41223	BUD31	Uncharacterized protein	Protein BUD31 homolog	100	3	1.97	2	2	19
G1SIJ6	Q13308	PTK7	Protein tyrosine kinase 7 (inactive)	Inactive tyrosine-protein kinase 7	93	2	1.97	28	62	39
G1SEF5	Q8NAV1	PRPF38A	PRP38_assoc domain-containing protein	Pre-mRNA-splicing factor 38A	100	2	1.97	2	5	9
G1TE34	P06756	ITGAV	Integrin_alpha2 domain-containing protein	Integrin alpha-V	95	2	1.96	26	93	34
G1SI76		MRPL43	L51_S25_CI-B8 domain-containing protein			1	1.96	3	3	19
G1SQB6	Q9NRX1	PNO1	KH domain-containing protein	RNA-binding protein PNO1	96	2	1.96	2	7	14
G1TTM6	Q99797	MIPEP	Peptidase_M3 domain-containing protein	Mitochondrial intermediate peptidase	93	2	1.96	6	6	15
G1SN06	O60488	ACSL4	AMP-binding domain-containing protein	Long-chain-fatty-acid--CoA ligase 4	98	2	1.95	11	21	21
G1SQR1	Q93096	PTP4A1	TYR_PHOSPHATASE_2 domain-containing protein	Protein tyrosine phosphatase type IVA 1	100	2	1.95	2	3	17
G1T4L3	Q01780	EXOSC10	HRDC domain-containing protein	Exosome component 10	89	2	1.95	2	4	4
G1T5J9	O75306	NDUFS2	Complex1_49kDa domain-containing protein	NADH dehydrogenase [ubiquinone] iron-sulfur protein 2, mitochondrial	94	2	1.94	6	10	23
G1SVB6	P00367	GLUD1	Glutamate dehydrogenase	Glutamate dehydrogenase 1, mitochondrial	98	2	1.94	23	173	64
G1THR4		GADD45GIP1	GADD45G interacting protein 1			1	1.93	2	5	13
G1SI37	D6RF87	ACSF2	Uncharacterized protein	Acyl-CoA synthetase family member 2, mitochondrial (Fragment)	82	3	1.93	18	31	51
G1SD44	Q13505	MTX1	Uncharacterized protein	Metaxin-1	84	3	1.93	6	8	21
G1TI40	P62316	SNRPD2	Small nuclear ribonucleoprotein Sm D2	Small nuclear ribonucleoprotein Sm D2	100	2	1.92	6	18	48
	K7EKE6	LONP1		Lon protease homolog, mitochondrial		4	1.92	19	44	28
G1SE36	R4GMU1	H6PD	GDH/6PGL endoplasmic bifunctional protein	GDH/6PGL endoplasmic bifunctional protein	85	2	1.92	8	7	15
G1T0U4	Q9UIG0	BAZ1B	Bromodomain adjacent to zinc finger domain 1B	Tyrosine-protein kinase BAZ1B	94	2	1.92	4	4	4
G1TB02	E7EPS8	PTPRM	Uncharacterized protein	Receptor-type tyrosine-protein phosphatase mu	98	3	1.91	3	3	3
G1SK42	P21980	TGM2	TGc domain-containing protein	Protein-glutamine gamma-glutamyltransferase 2	87	2	1.91	22	75	51
G1SF56		MICAL2	Microtubule associated monooxygenase, calponin and LIM domain containing 2			1	1.91	3	4	6
G1SUA4	J3KS05	CBX1	Uncharacterized protein	Chromobox protein homolog 1 (Fragment)	98	3	1.90	4	12	39
G1T2A9	Q14315	FLNC	Uncharacterized protein	Filamin-C	90	3	1.90	73	102	40
G1U5U0	Q9Y2X3	NOP58	Nop domain-containing protein	Nucleolar protein 58	92	2	1.89	11	5	29
G1TD99	A0A1B0GUX9	PCCA	Uncharacterized protein	Propionyl-CoA carboxylase alpha chain, mitochondrial (Fragment)	96	3	1.89	8	12	15
	P08574	CYC1		Cytochrome c1, heme protein, mitochondrial		4	1.89	4	4	16
G1SUG5		DHX37	DEAH-box helicase 37			1	1.89	2	2	3
G1U1X6		NT5C3A	5~-nucleotidase			1	1.89	2	3	9
G1TBS4	P20020	ATP2B1	Calcium-transporting ATPase	Plasma membrane calcium-transporting ATPase 1	97	2	1.88	6	5	7
G1SPY7	O14807	MRAS	Uncharacterized protein	Ras-related protein M-Ras	100	3	1.88	2	3	16
G1U5M7	A0A3B3IUA2	SNU13	Ribonucleoprotein	Ribonucleoprotein	100	2	1.88	4	12	23
G1SGH2		MRPL15	Ribosomal_L18e/L15P domain-containing protein			1	1.87	2	2	9
G1T8H1	O95573	ACSL3	AMP-binding domain-containing protein	Long-chain-fatty-acid--CoA ligase 3	96	2	1.87	7	6	14
G1T701		DBT	Dihydrolipoamide acetyltransferase component of pyruvate dehydrogenase complex			1	1.87	2	3	5
G1TPN3	D6R9P3	HNRNPAB	Uncharacterized protein	Heterogeneous nuclear ribonucleoprotein A/B	96	3	1.87	9	80	37
G1SUQ9	P11387	TOP1	DNA topoisomerase I	DNA topoisomerase 1	97	2	1.87	7	15	10
	Q9BXP5	SRRT		Serrate RNA effector molecule homolog		4	1.86	7	4	7
G1SDP7	H0Y8P4	UTP15	UTP15, small subunit processome component	U3 small nucleolar RNA-associated protein 15 homolog (Fragment)	92	2	1.86	2	2	4
G1U862	Q01650	SLC7A5	Large neutral amino acids transporter small subunit 1	Large neutral amino acids transporter small subunit 1	94	2	1.86	4	9	14
	Q10570	CPSF1		Cleavage and polyadenylation specificity factor subunit 1		4	1.86	2	2	2
G1TDQ3	J3QT28	BUB3	WD_REPEATS_REGION domain-containing protein	Mitotic checkpoint protein BUB3 (Fragment)	99	2	1.85	4	6	15
G1ST15	Q13825	AUH	AU RNA binding methylglutaconyl-CoA hydratase	Methylglutaconyl-CoA hydratase, mitochondrial	95	2	1.85	2	2	7
G1TMU1	Q13151	HNRNPA0	Uncharacterized protein	Heterogeneous nuclear ribonucleoprotein A0	78	3	1.85	2	3	10
G1SDW8	H0Y2P0	CD44	Link domain-containing protein	CD44 antigen (Fragment)	91	2	1.85	10	18	17
G1SYC1	P83111	LACTB	Uncharacterized protein	Serine beta-lactamase-like protein LACTB, mitochondrial	89	3	1.85	4	9	12
G1TQJ5		DEK	SAP domain-containing protein			1	1.84	2	2	6
G1SWF3		DHODH	Dihydroorotate dehydrogenase (quinone), mitochondrial			1	1.84	4	4	15

	P38919	EIF4A3		Eukaryotic initiation factor 4A-III		4	1.84	13	19	40
G1SRE6	A0A2R8Y420	EPB41	FERM domain-containing protein	Protein 4.1	95	2	1.84	3	6	5
	H0Y6E7	RBMX		RNA-binding motif protein, X chromosome (Fragment)		4	1.84	3	9	12
	Q9Y4W6	AFG3L2		AFG3-like protein 2		4	1.84	11	22	19
G1SHL0		SBNO1	Strawberry notch homolog 1			1	1.83	2	2	3
G1SL60	Q15393	SF3B3	CPSF_A domain-containing protein	Splicing factor 3B subunit 3	98	2	1.83	17	27	20
G1TC42		AXL	AXL receptor tyrosine kinase			1	1.82	2	3	3
G1SKQ8	Q13601	KRR1	KRR1 small subunit processome component	KRR1 small subunit processome component homolog	95	2	1.82	2	3	7
G1SPF1	Q5JTH9	RRP12	NUC173 domain-containing protein	RRP12-like protein	92	2	1.82	7	10	8
G1TA04	Q9UMS4	PRPF19	Uncharacterized protein	Pre-mRNA-processing factor 19	96	3	1.82	5	9	16
G1TC03	Q13263	TRIM28	Tripartite motif containing 28	Transcription intermediary factor 1-beta	96	2	1.82	16	15	25
G1SIF2	Q16822	PCK2	Uncharacterized protein	Phosphoenolpyruvate carboxykinase [GTP], mitochondrial	95	3	1.82	23	76	47
G1SPW1	Q29RF7	PDS5A	Uncharacterized protein	Sister chromatid cohesion protein PDS5 homolog A	99	3	1.81	3	3	3
G1SM50	Q15427	SF3B4	Uncharacterized protein	Splicing factor 3B subunit 4	100	3	1.81	4	9	18
G1SWA6	A0A2R8Y4T4	SMARCE1	HMG box domain-containing protein	SWI/SNF-related matrix-associated actin-dependent regulator of chromatin subfamily E member 1	95	2	1.81	3	3	9
G1TV43	O60218	AKR1B10	Aldo_ket_red domain-containing protein	Aldo-keto reductase family 1 member B10	86	2	1.81	10	19	31
	O95202	LETM1		Mitochondrial proton/calcium exchanger protein		4	1.80	7	13	10
	P43897	TSFM		Elongation factor Ts, mitochondrial		4	1.80	3	4	17
G1TE69	J3KTL2	SRSF1	Uncharacterized protein	Serine/arginine-rich-splicing factor 1	100	3	1.80	6	11	25
G1TG89	P62244	RPS15A	Uncharacterized protein	40S ribosomal protein S15a	100	3	1.80	7	24	55
G1SGY8	Q3ZCQ8	TIMM50	Mitochondrial import inner membrane translocase subunit TIM50	Mitochondrial import inner membrane translocase subunit TIM50	96	2	1.80	6	13	23
G1T970	P30038	ALDH4A1	Multifunctional fusion protein	Delta-1-pyrroline-5-carboxylate dehydrogenase, mitochondrial	91	2	1.80	11	25	31
B7NZG9	Q8N3U4	STAG2	Stromal antigen 2 isoform a (Predicted)	Cohesin subunit SA-2	97	2	1.79	5	6	8
G1SYP7		POLR1A	DNA-directed RNA polymerase subunit			1	1.79	2	4	2
	G3V0I5	NDUFV1		NADH dehydrogenase [ubiquinone] flavoprotein 1, mitochondrial		4	1.79	6	9	27
G1TG28	P80723	BASP1	Brain abundant membrane attached signal protein 1	Brain acid soluble protein 1	61	2	1.78	5	6	51
G1TET2	P13796	LCP1	Uncharacterized protein	Plastin-2	98	3	1.78	19	20	46
G1SMY1	A0A1W2PQ51	DDX17	Uncharacterized protein	Probable ATP-dependent RNA helicase DDX17	99	3	1.78	20	22	38
G1TEP2		FAM210B	DUF1279 domain-containing protein			1	1.78	2	2	12
G1T1L7	A0A087WWS1	THOC1	Death domain-containing protein	THO complex subunit 1	98	2	1.77	3	3	10
G1SKK1		DUT	dUTPase domain-containing protein			1	1.77	2	5	14
G1SUP4	J3QRS9	ZNF207	Uncharacterized protein	BUB3-interacting and GLEBS motif-containing protein ZNF207	100	3	1.77	3	4	8
G1STJ3		PPIE	Peptidyl-prolyl cis-trans isomerase E			1	1.77	2	2	13
G1SIQ9	Q9GZL7	WDR12	Ribosome biogenesis protein WDR12	Ribosome biogenesis protein WDR12	97	2	1.77	3	2	15
G1TG15		FAM210A	DUF1279 domain-containing protein			1	1.77	2	3	13
G1TBV4	Q14566	MCM6	DNA helicase	DNA replication licensing factor MCM6	97	2	1.77	2	2	4
G1T134		ABHD11	Abhydrolase domain containing 11			1	1.76	3	3	16
G1T7V5	E9PEX6	DLD	Dihydrolipoyl dehydrogenase	Dihydrolipoyl dehydrogenase	91	2	1.76	11	3	32
G1SI29	P49411	TUFM	Elongation factor Tu	Elongation factor Tu, mitochondrial	93	2	1.76	17	107	41
G1TDQ1	Q9H0C8	ILKAP	PPM-type phosphatase domain-containing protein	Integrin-linked kinase-associated serine/threonine phosphatase 2C	94	2	1.76	2	3	8
G1SJ32	A0A0A0MTB8	WDR36	WD_REPEATS_REGION domain-containing protein	WD repeat-containing protein 36	94	2	1.76	2	2	4
G1T6T0	J3KNJ3	NAALAD2	Uncharacterized protein	N-acetylated-alpha-linked acidic dipeptidase 2	89	3	1.76	11	18	23
P00389	P16435	POR	NADPH--cytochrome P450 reductase	NADPH--cytochrome P450 reductase	92	2	1.76	27	70	49
G1SUR4		ZADH2	PKS_ER domain-containing protein			1	1.75	3	2	18
G1SY50	E7EPT4	NDUFV2	Uncharacterized protein	NADH dehydrogenase [ubiquinone] flavoprotein 2, mitochondrial	98	3	1.75	4	9	21
G1SQU6	O75489	NDUFS3	Complex1_30kDa domain-containing protein	NADH dehydrogenase [ubiquinone] iron-sulfur protein 3, mitochondrial	91	2	1.75	4	5	21
	Q12873	CHD3		Chromodomain-helicase-DNA-binding protein 3		4	1.75	7	2	6
G1SXC8		NXF1	Nuclear RNA export factor 1			1	1.75	3	4	8
G1T5I9	F8WJN3	CPSF6	RRM domain-containing protein	Cleavage and polyadenylation-specificity factor subunit 6	99	2	1.75	3	4	9
G1SNR2	Q969S9	GFM2	Ribosome-releasing factor 2, mitochondrial	Ribosome-releasing factor 2, mitochondrial	88	2	1.74	4	4	10
G1SL16	A0A0B4J1Z1	SRSF7	Uncharacterized protein	Serine/arginine-rich-splicing factor 7	100	3	1.74	4	4	31
G1SF95	H0Y8G5	HNRNPD	Heterogeneous nuclear ribonucleoprotein D	Heterogeneous nuclear ribonucleoprotein D0 (Fragment)	99	2	1.74	7	16	26
P35953	P98155	VLDLR	Very low-density lipoprotein receptor	Very low-density lipoprotein receptor	97	2	1.74	10	13	16
G1SKI8	Q6DKI1	RPL7L1	Uncharacterized protein	60S ribosomal protein L7-like 1	85	3	1.74	3	5	17
	A0A087WVZ9	POLR2E		DNA-directed RNA polymerases I, II, and III subunit RPABC1		4	1.73	2	3	17
G1SFQ3		MRPL2	Ribosomal_L2_C domain-containing protein			1	1.73	3	5	19
G1SP24	E7EQB9	POLR1C	RPOLD domain-containing protein	DNA-directed RNA polymerases I and III subunit RPAC1	84	2	1.73	4	7	25
G1T011	Q9Y5B9	SUPT16H	Uncharacterized protein	FACT complex subunit SPT16	100	3	1.73	7	10	10
G1T9T5	P36957	DLST	Lipoyl-binding domain-containing protein	Dihydrolipoyllysine-residue succinyltransferase component of 2-oxoglutarate dehydrogenase complex, mitochondrial	91	2	1.73	12	29	35
G1T120	S4R369	MRPL37	Uncharacterized protein	39S ribosomal protein L37, mitochondrial	84	3	1.73	6	8	19

	K7EIE8	MBD3		Methyl-CpG binding domain protein 3, isoform CRA_b		4	1.72	2	2	14
G1U2E5	P08243	ASNS	Asparagine synthetase [glutamine-hydrolyzing]	Asparagine synthetase [glutamine-hydrolyzing]	87	2	1.72	8	7	19
	P02458	COL2A1		Collagen alpha-1(II) chain		4	1.72	4	2	5
G1SRN1		LAS1L	LAS1 like, ribosome biogenesis factor			1	1.72	4	4	11
G1SF97		MRPL46	MRP-L46 domain-containing protein			1	1.72	4	11	18
G1SQB1	P22033	MMUT	B12-binding domain-containing protein	Methylmalonyl-CoA mutase, mitochondrial	96	2	1.71	2	2	4
G1SLV3	P42285	MTREX	Uncharacterized protein	Exosome RNA helicase MTR4	99	3	1.71	8	16	11
	Q9NZN4	EHD2		EH domain-containing protein 2		4	1.71	11	17	27
G1SLJ8	Q8IY81	FTSJ3	pre-rRNA processing protein FTSJ3	pre-rRNA 2~-O-ribose RNA methyltransferase FTSJ3	85	2	1.71	7	8	19
G1T5H5	Q15050	RRS1	Ribosome biogenesis regulatory protein	Ribosome biogenesis regulatory protein homolog	92	2	1.71	5	6	20
G1TNT7		DDX27	DEAD-box helicase 27			1	1.71	2	2	3
G1SMP5	Q5T160	RARS2	DALR_1 domain-containing protein	Probable arginine--tRNA ligase, mitochondrial	93	2	1.71	6	5	10
G1U018		IGF2R	Insulin like growth factor 2 receptor			1	1.70	9	10	4
G1TA05	A2AE48	TRIM26	Uncharacterized protein	Tripartite motif-containing protein 26 (Fragment)	92	3	1.70	2	2	4
G1SSK8	B4DJV2	CS	Citrate synthase	Citrate synthase	96	2	1.70	18	106	53
G1T5L3	Q9H9J2	MRPL44	Uncharacterized protein	39S ribosomal protein L44, mitochondrial	90	3	1.70	3	5	15
G1SSF2		ENG	Endoglin			1	1.70	5	9	12
G1TE50	Q9Y221	NIP7	60S ribosome subunit biogenesis protein NIP7 homolog	60S ribosome subunit biogenesis protein NIP7 homolog	97	2	1.70	2	2	16
G1TGK9	Q08211	DHX9	Uncharacterized protein	ATP-dependent RNA helicase A	93	3	1.70	21	40	31
G1STZ4	P11413	G6PD	Glucose-6-phosphate 1-dehydrogenase	Glucose-6-phosphate 1-dehydrogenase	94	2	1.69	5	9	11
G1TSL1	A0A087WZN1	IDH3B	Isocitrate dehydrogenase [NAD] subunit, mitochondrial	Isocitrate dehydrogenase [NAD] subunit, mitochondrial	95	2	1.69	14	20	42
G1U864		TRRAP	Transformation/transcription domain associated protein			1	1.69	3	3	1
G1TV19	H3BPE7	FUS	FUS RNA binding protein	RNA-binding protein FUS	80	2	1.69	5	3	9
G1SMI2	Q9Y305	ACOT9	Acyl-CoA thioesterase 9	Acyl-coenzyme A thioesterase 9, mitochondrial	83	2	1.68	13	11	33
G1SR36	C9JG87	MRPL39	Uncharacterized protein	39S ribosomal protein L39, mitochondrial (Fragment)	86	3	1.68	5	5	12
G1SD24	A0A494BZU6	PARN	R3H domain-containing protein	Poly(A)-specific ribonuclease PARN (Fragment)	96	2	1.68	2	2	5
G1SES8	G5E9V5	MRPS22	Uncharacterized protein	28S ribosomal protein S22, mitochondrial	84	3	1.67	2	2	6
U3KPI1		MPHOSPH10	U3 small nucleolar ribonucleoprotein protein MPP10			1	1.67	2	3	4
G1TS78	A0A075B6F6	HM13	Uncharacterized protein	Minor histocompatibility antigen H13 (Fragment)	84	3	1.67	7	21	21
G1SDE1		MTG1	Mitochondrial GTPase 1			1	1.67	2	2	11
G1TAH7	P29401	TKT	TRANSKETOLASE_1 domain-containing protein	Transketolase	94	2	1.67	20	49	49
G1T958		EBP	EXPERA domain-containing protein			1	1.67	2	3	8
G1SHJ3		KDELC1	CAP10 domain-containing protein			1	1.67	5	6	14
G1T1L4		GRWD1	WD_REPEATS_REGION domain-containing protein			1	1.66	3	3	13
G1T8F7	A0A494C128	NOP56	Nop domain-containing protein	Nucleolar protein 56	98	2	1.66	16	22	39
G1SIW1	O43143	DHX15	Uncharacterized protein	Pre-mRNA-splicing factor ATP-dependent RNA helicase DHX15	99	3	1.66	14	24	24
G1T3N1	Q9NVP1	DDX18	RNA helicase	ATP-dependent RNA helicase DDX18	87	2	1.66	6	8	12
	Q13247	SRSF6		Serine/arginine-rich splicing factor 6		4	1.66	3	4	10
G1T8P3	P08621	SNRNP70	Small nuclear ribonucleoprotein U1 subunit 70	U1 small nuclear ribonucleoprotein 70 kDa	92	2	1.66	5	8	11
	Q9P0J1	PDP1		[Pyruvate dehydrogenase [acetyl-transferring]]-phosphatase 1, mitochondrial		4	1.66	2	3	5
	Q9NTZ6	RBM12		RNA-binding protein 12		4	1.66	2	3	5
G1TAV2		SMCHD1	SMC hinge domain-containing protein			1	1.66	3	3	2
G1SY30	O75643	SNRNP200	Uncharacterized protein	U5 small nuclear ribonucleoprotein 200 kDa helicase	100	3	1.66	39	65	30
G1T8H6	K7EK07	H3F3B	Histone H3	Histone H3 (Fragment)	98	2	1.65	7	27	52
G1TH59	Q14498	RBM39	Uncharacterized protein	RNA-binding protein 39	91	3	1.65	7	9	21
Q8HZQ5	E7EQR4	EZR	Ezrin	Ezrin	94	2	1.65	15	11	28
G1TC48	A0A0A0MRA5	HNRNPUL1	Uncharacterized protein	Heterogeneous nuclear ribonucleoprotein U-like protein 1	95	3	1.65	6	9	10
G1TMU2	P52597	HNRNPF	Uncharacterized protein	Heterogeneous nuclear ribonucleoprotein F	99	3	1.65	11	182	44
	Q8WX92	NELFB		Negative elongation factor B		4	1.65	3	4	6
G1T1R4		NR3C1	Glucocorticoid receptor			1	1.65	2	3	6
G1SS51		MRPS25	L51_S25_CI-B8 domain-containing protein			1	1.64	3	3	22
G1T116	Q96TA2	YME1L1	AAA domain-containing protein	ATP-dependent zinc metalloprotease YME1L1	89	2	1.64	4	6	9
G1SP32	Q6NVY1	HIBCH	3-hydroxyisobutyryl-CoA hydrolase, mitochondrial	3-hydroxyisobutyryl-CoA hydrolase, mitochondrial	84	2	1.64	8	9	25
G1SNV4	Q12874	SF3A3	Matrin-type domain-containing protein	Splicing factor 3A subunit 3	100	2	1.64	11	24	33
G1TIR7	O95777	LSM8	U6 snRNA-associated Sm-like protein LSm8	U6 snRNA-associated Sm-like protein LSm8	99	2	1.64	3	51	52
G1SKZ3	P12004	PCNA	Proliferating cell nuclear antigen	Proliferating cell nuclear antigen	99	2	1.64	2	3	15
G1TDX2	A0A3B3ISY9	AGK	Acylglycerol kinase	Acylglycerol kinase, mitochondrial	94	2	1.64	5	6	32
G1SP33		SYMPK	Symplekin			1	1.64	2	2	3
G1SSH0	A0A087WUB9	CTNNBL1	DUF1716 domain-containing protein	Beta-catenin-like protein 1	96	2	1.64	5	7	12
G1SRD2	P28330	ACADL	Uncharacterized protein	Long-chain specific acyl-CoA dehydrogenase, mitochondrial	82	3	1.64	5	84	17

G1SMI7	V9GYL9	DAP3	Uncharacterized protein	28S ribosomal protein S29, mitochondrial (Fragment)	86	3	1.64	8	9	28
	A0A0C4DFX9	NELFA		Negative elongation factor A		4	1.64	2	2	7
	P31040	SDHA		Succinate dehydrogenase [ubiquinone] flavoprotein subunit, mitochondrial		4	1.64	13	7	34
G1TTN9	Q9P258	RCC2	Regulator of chromosome condensation 2	Protein RCC2	99	2	1.64	4	5	13
	O60518	RANBP6		Ran-binding protein 6		4	1.64	2	8	3
P27124	Q02790	FKBP4	Peptidyl-prolyl cis-trans isomerase FKBP4	Peptidyl-prolyl cis-trans isomerase FKBP4	91	2	1.63	9	12	26
G1SRY1	Q8TCS8	PNPT1	S1 motif domain-containing protein	Polyribonucleotide nucleotidyltransferase 1, mitochondrial	94	2	1.63	7	14	14
G1T813	P08559	PDHA1	Pyruvate dehydrogenase E1 component subunit alpha	Pyruvate dehydrogenase E1 component subunit alpha, somatic form, mitochondrial	99	2	1.63	12	23	35
G1SMM7	P62318	SNRPD3	Small nuclear ribonucleoprotein Sm D3	Small nuclear ribonucleoprotein Sm D3	100	2	1.63	3	5	29
G1U3I5		ECH1	Enoyl-CoA hydratase 1			1	1.63	9	10	37
G1SFG7	P48681	NES	Nestin	Nestin	60	2	1.62	28	149	31
	Q16394	EXT1		Exostosin-1		4	1.62	2	3	5
G1SCR7	E9PF10	NUP155	Uncharacterized protein	Nuclear pore complex protein Nup155	90	3	1.62	14	22	16
G1SV12	P16219	ACADS	Uncharacterized protein	Short-chain specific acyl-CoA dehydrogenase, mitochondrial	92	3	1.62	13	21	51
	Q08945	SSRP1		FACT complex subunit SSRP1		4	1.62	3	3	9
G1STI3	Q07666	KHDRBS1	KH domain-containing protein	KH domain-containing, RNA-binding, signal transduction-associated protein 1	99	2	1.62	4	10	12
G1T617	P49756	RBM25	Uncharacterized protein	RNA-binding protein 25	99	3	1.62	5	4	9
G1SRF1	J3KT10	NUP85	Nuclear pore complex protein Nup85	Nuclear pore complex protein Nup85	84	2	1.62	7	12	16
G1SH66		RBM3	RRM domain-containing protein			1	1.62	3	6	37
G1SW61	Q2TAY7	SMU1	Uncharacterized protein	WD40 repeat-containing protein SMU1	100	3	1.62	7	11	17
G1T6E6		NOC3L	Nucleolar complex protein 3 homolog			1	1.62	4	6	9
G1SHE2		GGCX	HTTM domain-containing protein			1	1.62	2	3	3
G1TI97	Q13769	THOC5	Uncharacterized protein	THO complex subunit 5 homolog	97	3	1.62	3	5	8
G1SVH1	A0A0U1RRK1	MICU1	Uncharacterized protein	Calcium uptake protein 1, mitochondrial	94	3	1.61	5	20	18
G1SKM2	P35555	FBN1	Uncharacterized protein	Fibrillin-1	97	3	1.61	6	7	2
G1T3X2	H7BZW6	SAP18	Histone deacetylase complex subunit SAP18	Histone deacetylase complex subunit SAP18 (Fragment)	98	2	1.61	2	3	15
	Q01105-2	SET		Isoform 2 of Protein SET		4	1.60	6	10	36
G1T2U6	A0A0C4DG89	DDX46	Uncharacterized protein	Probable ATP-dependent RNA helicase DDX46	99	3	1.60	6	5	8
G1SQ11	P54886	ALDH18A1	Delta-1-pyrroline-5-carboxylate synthase	Delta-1-pyrroline-5-carboxylate synthase	97	2	1.60	30	79	53
G1T6L0	Q5T3Q7	HEATR1	BP28CT domain-containing protein	HEAT repeat-containing protein 1	93	2	1.60	11	14	10
G1SFE6	P08579	SNRPB2	Uncharacterized protein	U2 small nuclear ribonucleoprotein B~~	96	3	1.60	3	2	16
G1TU85		FADS3	Cytochrome b5 heme-binding domain-containing protein			1	1.60	2	2	10
G1SCK0	Q6P2Q9	PRPF8	MPN domain-containing protein	Pre-mRNA-processing-splicing factor 8	100	2	1.60	35	56	22
G1SJI6		MRPS23	MRP-S23 domain-containing protein			1	1.60	5	6	34
G1T5A2	P08648	ITGA5	Integrin_alpha2 domain-containing protein	Integrin alpha-5	91	2	1.60	10	13	13
	Q15428	SF3A2		Splicing factor 3A subunit 2		4	1.60	2	2	4
G1SKW4		KIDINS220	Kinase D interacting substrate 220			1	1.60	8	9	7
U3KPG6		ICAM1	Intercellular adhesion molecule 1			1	1.60	8	14	20
G1TBS2	Q9Y265	RUVBL1	RuvB-like helicase	RuvB-like 1	100	2	1.60	14	27	47
	Q5R3B4	MPC2		Mitochondrial pyruvate carrier (Fragment)		4	1.59	3	4	32
G1T3A6	A0A1W2PQH3	ME2	Malic enzyme	Malic enzyme	92	2	1.59	16	14	40
G1T9V1	P11177	PDHB	Pyruvate dehydrogenase E1 component subunit beta	Pyruvate dehydrogenase E1 component subunit beta, mitochondrial	97	2	1.59	10	33	39
	A0A3B3IRT8	SSR1		Translocon-associated protein subunit alpha		4	1.59	4	76	21
G1TX03	A0A0J9YW13	RBM8A	RNA-binding protein 8A	RNA-binding protein 8A (Fragment)	100	2	1.59	5	8	49
G1SPJ7		UTP4	WD_REPEATS_REGION domain-containing protein			1	1.59	2	2	5
G1SS37	A0A494C1M4	ALDH1L2	10-formyltetrahydrofolate dehydrogenase	10-formyltetrahydrofolate dehydrogenase	96	2	1.59	44	102	66
	A0A087WTP3	KHSRP		Far upstream element-binding protein 2		4	1.59	11	12	17
G1SDX3	E9PCY5	TOP2B	DNA topoisomerase 2	DNA topoisomerase 2 (Fragment)	99	2	1.59	14	22	12
G1U6N8	Q14980	NUMA1	Nuclear mitotic apparatus protein 1	Nuclear mitotic apparatus protein 1	90	2	1.59	21	28	15
G1U1H3	P61018	RAB4B	Uncharacterized protein	Ras-related protein Rab-4B	100	3	1.59	4	6	31
G1TBH1	A0A1W2PPT5	POLR2B	DNA-directed RNA polymerase subunit beta	DNA-directed RNA polymerase subunit beta	100	2	1.58	6	8	7
G1TQ57	H3BP71	RNF40	E3 ubiquitin protein ligase	E3 ubiquitin protein ligase	92	2	1.58	3	3	6
G1T2Y5	P12270	TPR	TPR_MLP1_2 domain-containing protein	Nucleoprotein TPR	97	2	1.58	26	36	14
G1T6I6	Q12906	ILF3	Interleukin enhancer binding factor 3	Interleukin enhancer-binding factor 3	96	2	1.58	21	33	33
G1SFH4		MRPL24	KOW domain-containing protein			1	1.58	4	7	32
G1TJW3	Q15233	NONO	Uncharacterized protein	Non-POU domain-containing octamer-binding protein	99	3	1.58	12	44	33
G1T6T8	O94906	PRPF6	Uncharacterized protein	Pre-mRNA-processing factor 6	96	3	1.58	5	7	7
G1TFE0	H0YHA7	RPL18	Ribosomal_L18e/L15P domain-containing protein	60S ribosomal protein L18 (Fragment)	90	2	1.58	3	9	22
G1SGL3		SRSF11	RRM domain-containing protein			1	1.58	2	4	5
	A0A087X2B1	RBFOX1		RNA binding protein fox-1 homolog		4	1.57	2	3	3

G1SGB5	P23246	SFPQ	Splicing factor proline and glutamine rich	Splicing factor, proline- and glutamine-rich	100	2	1.57	13	25	21
	P0DN76	U2AF1L5		Splicing factor U2AF 35 kDa subunit-like protein		4	1.57	3	5	23
G1U9B4	P62995	TRA2B	RRM domain-containing protein	Transformer-2 protein homolog beta	100	2	1.57	4	8	16
G1SGA5	Q9HCJ6	VAT1L	PKS_ER domain-containing protein	Synaptic vesicle membrane protein VAT-1 homolog-like	96	2	1.57	3	3	10
	Q12931	TRAP1		Heat shock protein 75 kDa, mitochondrial		4	1.57	7	2	12
G1SLK4	Q8WVX9	FAR1	Fatty acyl-CoA reductase	Fatty acyl-CoA reductase 1	93	2	1.57	2	2	7
G1SHV1	Q9Y333	LSM2	U6 snRNA-associated Sm-like protein LSm2	U6 snRNA-associated Sm-like protein LSm2	100	2	1.57	2	5	27
G1U7I9	A0A0G2JJZ9	DDX39B	Uncharacterized protein	Spliceosome RNA helicase DDX39B (Fragment)	95	3	1.57	14	32	46
G1U4G9	O00299	CLIC1	Chloride intracellular channel protein	Chloride intracellular channel protein 1	98	2	1.56	6	11	42
G1SDD0	P30084	ECHS1	Uncharacterized protein	Enoyl-CoA hydratase, mitochondrial	86	3	1.56	9	16	45
G1U5D4	F5GY32	PUS1	tRNA pseudouridine synthase	tRNA pseudouridine synthase A (Fragment)	78	2	1.56	2	3	5
G1TMZ2		MRPS7	Ribosomal_S7 domain-containing protein			1	1.56	5	5	29
G1TAB7	A0A2R8Y3X5	OPA1	Dynamin-type G domain-containing protein	Dynamin-like 120 kDa protein, mitochondrial	94	2	1.56	26	48	32
G1SZR8	P51858	HDGF	Heparin binding growth factor	Hepatoma-derived growth factor	96	2	1.56	5	5	22
	Q8N201	INTS1		Integrator complex subunit 1		4	1.56	4	5	2
G1T3R1	Q86SX6	GLRX5	Glutaredoxin 5	Glutaredoxin-related protein 5, mitochondrial	84	2	1.56	3	4	25
G1SDL0	Q96DX4	RSPRY1	Uncharacterized protein	RING finger and SPRY domain-containing protein 1	93	3	1.56	4	5	16
G1TUB8	Q5VVC8	RPL11	Uncharacterized protein	60S ribosomal protein L11	100	3	1.56	5	13	34
G1SIP9	H0Y9G6	MRPL3	Uncharacterized protein	39S ribosomal protein L3, mitochondrial (Fragment)	89	3	1.56	4	4	21
G1TTK6	E7ETT1	PCCB	Uncharacterized protein	Propionyl-CoA carboxylase beta chain, mitochondrial	86	3	1.56	9	14	28
G1TBR6	Q69YN4	VIRMA	VIR_N domain-containing protein	Protein virilizer homolog	98	2	1.56	5	5	4
G1T5G8	Q9HC07	TMEM165	GDT1 family protein	Transmembrane protein 165	91	2	1.55	2	8	9
G1U194	J3KPP4	LUC7L3	Uncharacterized protein	Cisplatin resistance-associated overexpressed protein, isoform CRA_b	98	3	1.55	3	4	8
G1THY5	Q9Y3C6	PPIL1	Peptidyl-prolyl cis-trans isomerase	Peptidyl-prolyl cis-trans isomerase-like 1	100	2	1.55	2	2	29
G1SGY1	Q86TB9	PATL1	PAT1 domain-containing protein	Protein PAT1 homolog 1	97	2	1.55	2	2	4
G1TCH9	A0A2R8Y473	ABCB7	Uncharacterized protein	ATP-binding cassette sub-family B member 7, mitochondrial	95	3	1.55	7	13	13
G1STX9		COQ8B	Coenzyme Q8B			1	1.55	3	5	15
G1T5H2	Q9H0D6	XRN2	5~-3~ exoribonuclease	5~-3~ exoribonuclease 2	98	2	1.55	8	11	14
G1T696	Q09161	NCBP1	MIF4G domain-containing protein	Nuclear cap-binding protein subunit 1	99	2	1.55	9	12	19
G1STS0	Q9Y2S7	POLDIP2	ApaG domain-containing protein	Polymerase delta-interacting protein 2	98	2	1.55	6	6	26
G1SD54	Q99720	SIGMAR1	Uncharacterized protein	Sigma non-opioid intracellular receptor 1	97	3	1.55	4	6	23
G1T0V4	F5H013	SNRPG	Small nuclear ribonucleoprotein G	Small nuclear ribonucleoprotein G	98	2	1.54	3	20	58
G1TYH7	Q9HDC9	APMAP	Adipocyte plasma membrane associated protein	Adipocyte plasma membrane-associated protein	94	2	1.54	12	31	34
G1SNF2	P13995	MTHFD2	Uncharacterized protein	Bifunctional methylenetetrahydrofolate dehydrogenase/cyclohydrolase, mitochondrial	93	3	1.54	12	32	57
G1SZA1	B4DHE8	MSI2	Uncharacterized protein	RNA-binding protein Musashi homolog 2	94	3	1.54	4	3	18
G1SR61	H7BXY3	DHX30	Uncharacterized protein	ATP-dependent RNA helicase DHX30	98	3	1.54	7	13	12
G1TKC4	Q15046	KARS	AA_TRNA_LIGASE_II domain-containing protein	Lysine--tRNA ligase	88	2	1.54	13	18	32
G1SQA8	P06576	ATP5F1B	ATP synthase subunit beta	ATP synthase subunit beta, mitochondrial	97	2	1.53	29	538	74
	U3KQK1	LSM4		U6 snRNA-associated Sm-like protein LSm4		4	1.53	2	5	13
G1SV75	H3BNK3	NDUFAB1	Acyl carrier protein	Acyl carrier protein (Fragment)	63	2	1.53	2	3	10
B7NZN9	P51571	SSR4	Signal sequence receptor, delta (Predicted)	Translocon-associated protein subunit delta	97	2	1.53	5	10	36
G1TEI0		PARP1	Poly [ADP-ribose] polymerase			1	1.53	3	3	5
G1TCU1	I3L2K5	ZC3H7A	Uncharacterized protein	Zinc finger CCCH domain-containing protein 7A (Fragment)	95	3	1.53	2	2	2
G1TME5	Q9H857	NT5DC2	Uncharacterized protein	5~-nucleotidase domain-containing protein 2	86	3	1.53	4	4	13
G1TTB5	A0A0D9SEM4	SRSF4	Serine and arginine rich splicing factor 4	Serine/arginine-rich-splicing factor 4 (Fragment)	75	2	1.53	3	2	8
G1TTY7	M0R1A7	RPL18A	60S ribosomal protein L18a	60S ribosomal protein L18a	90	2	1.53	4	4	19
G1SPR9	P04844	RPN2	Dolichyl-diphosphooligosaccharide--protein glycosyltransferase subunit 2	Dolichyl-diphosphooligosaccharide--protein glycosyltransferase subunit 2	93	2	1.52	25	96	68
G1SK29		MTIF2	Tr-type G domain-containing protein			1	1.52	3	4	8
U3KM71		ATP5MG	ATP synthase subunit			1	1.52	5	17	62
G1SP02	A8MT40	PDPR	Uncharacterized protein	Pyruvate dehydrogenase phosphatase regulatory subunit, mitochondrial	95	3	1.52	4	7	7
G1SQA4	Q6UWP7	LCLAT1	PlsC domain-containing protein	Lysocardiolipin acyltransferase 1	89	2	1.52	5	6	15
G1TJV3	Q9H7Z7	PTGES2	Prostaglandin E synthase 2	Prostaglandin E synthase 2	79	2	1.52	4	5	19
G1T524	P05141	SLC25A5	Uncharacterized protein	ADP/ATP translocase 2	98	3	1.52	22	20	69
G1TX78	Q15388	TOMM20	Uncharacterized protein	Mitochondrial import receptor subunit TOM20 homolog	100	3	1.52	3	27	28
	Q969V3	NCLN		Nicalin		4	1.51	7	11	18
G1SHI9	A0A0D9SFS3	OGDH	Transket_pyr domain-containing protein	2-oxoglutarate dehydrogenase, mitochondrial	95	2	1.51	29	69	40
G1SUF4	O15269	SPTLC1	Aminotran_1_2 domain-containing protein	Serine palmitoyltransferase 1	95	2	1.51	6	12	15
G1SM77	P36542	ATP5F1C	ATP synthase subunit gamma	ATP synthase subunit gamma, mitochondrial	94	2	1.51	11	105	43
G1SKD9	P53597	SUCLG1	Succinate--CoA ligase [ADP/GDP-forming] subunit alpha, mitochondrial	Succinate--CoA ligase [ADP/GDP-forming] subunit alpha, mitochondrial	96	2	1.51	7	27	25
G1SQP9	E7EMS6	COMT	Catechol-O-methyltransferase	Catechol O-methyltransferase (Fragment)	78	2	1.51	10	35	52

G1SFD8	Q9BUQ8	DDX23	Uncharacterized protein	Probable ATP-dependent RNA helicase DDX23	99	3	1.51	7	7	11
G1SUS6	D6RD69	SAR1B	Uncharacterized protein	GTP-binding protein SAR1b (Fragment)	98	3	1.51	4	4	25
G1SLP3	H3BMM9	RNPS1	RNA binding protein with serine rich domain 1	RNA-binding protein with serine-rich domain 1 (Fragment)	99	2	1.51	2	3	14
	A0A0U1RQF0	FASN		Fatty acid synthase		4	1.50	8	12	4
G1TVG7	O75533	SF3B1	SF3b1 domain-containing protein	Splicing factor 3B subunit 1	99	2	1.50	20	42	27
G1SH10	H3BVG0	NUP93	Nuclear pore complex protein Nup93	Nuclear pore complex protein Nup93	99	2	1.50	14	19	24
G1SV60	J3KS45	TMCO1	Calcium load-activated calcium channel	Calcium load-activated calcium channel (Fragment)	95	2	1.50	2	2	13
G1SIP2	A0A3B3ITJ4	HNRNPL	Uncharacterized protein	Heterogeneous nuclear ribonucleoprotein L (Fragment)	93	3	1.50	18	40	53
G1SQ54	A0A087WT44	HMOX2	Heme oxygenase	Heme oxygenase 2	89	2	1.50	7	10	33
	P37198	NUP62		Nuclear pore glycoprotein p62		4	1.50	4	4	11
G1U636	G3V153	CAPRIN1	Uncharacterized protein	Caprin-1	98	3	1.50	7	25	12
G1SUQ4		DNTTIP2	Fcf2 domain-containing protein			1	1.50	2	3	4
G1U0U5	E9PDE8	HSPA4L	Uncharacterized protein	Heat shock 70 kDa protein 4L	91	3	1.49	5	4	8
	A0A0A6YYJ8	LUC7L2		Putative RNA-binding protein Luc7-like 2		4	1.49	4	7	11
G1T5A5		RTN4IP1	PKS_ER domain-containing protein			1	1.49	2	3	12
	A0A0C4DGG8	CCAR1		Cell division cycle and apoptosis regulator protein 1 (Fragment)		4	1.49	3	3	5
G1TF67		CORO1A	Coronin			1	1.49	3	2	10
G1SEX6	Q9H2U1	DHX36	Uncharacterized protein	ATP-dependent DNA/RNA helicase DHX36	94	3	1.49	3	4	5
G1SX50		RBM19	RNA binding motif protein 19			1	1.49	2	2	4
G1U276	A0A2R8Y6Y7	SUCLA2	Succinate--CoA ligase [ADP-forming] subunit beta, mitochondrial	Succinate--CoA ligase [ADP-forming] subunit beta, mitochondrial	91	2	1.49	13	23	38
G1T9E3	E9PLP8	CSTF3	Suf domain-containing protein	Cleavage stimulation factor subunit 3	100	2	1.49	3	5	7
G1STU7	Q5QNZ2	ATP5PB	Uncharacterized protein	ATP synthase F(0) complex subunit B1, mitochondrial	84	3	1.48	12	45	24
G1SG80	F8VZG5	AK2	Adenylate kinase 2, mitochondrial	Adenylate kinase 2, mitochondrial	92	2	1.48	9	25	47
G1T2G5	Q5NDL2	EOGT	Uncharacterized protein	EGF domain-specific O-linked N-acetylglucosamine transferase	91	3	1.48	8	10	18
G1TDH4	P30048	PRDX3	Thioredoxin domain-containing protein	Thioredoxin-dependent peroxide reductase, mitochondrial	86	2	1.48	8	25	36
Q9GKX2		DHRS4	Dehydrogenase/reductase SDR family member 4 (Fragment)			1	1.48	8	13	38
G1T9S4	H0YDD4	DLAT	Acetyltransferase component of pyruvate dehydrogenase complex	Acetyltransferase component of pyruvate dehydrogenase complex (Fragment)	90	2	1.48	11	22	25
G1SW97	A0A0A0MT83	IVD	Uncharacterized protein	Isovaleryl-CoA dehydrogenase isoform 1	95	3	1.48	7	10	17
G1T069	P57740	NUP107	Nuclear pore complex protein	Nuclear pore complex protein Nup107	94	2	1.48	9	22	16
	P26368	U2AF2		Splicing factor U2AF 65 kDa subunit		4	1.47	8	16	29
G1T8E0	A3KMH1	VWA8	VWFA domain-containing protein	von Willebrand factor A domain-containing protein 8	92	2	1.47	7	10	5
G1SEC9	Q9HAV0	GNB4	WD_REPEATS_REGION domain-containing protein	Guanine nucleotide-binding protein subunit beta-4	99	2	1.47	8	6	28
G1TEE3	G3V5Z3	PPP4R3A	SMK-1 domain-containing protein	Serine/threonine-protein phosphatase 4 regulatory subunit 3A	100	2	1.47	4	8	9
G1SEM4		THADA	DUF2428 domain-containing protein			1	1.47	2	2	1
G1SGY0	O00267	SUPT5H	Transcription elongation factor SPT5	Transcription elongation factor SPT5	98	2	1.47	8	8	12
G1U6X4	F8VQZ7	METAP2	Methionine aminopeptidase 2	Methionine aminopeptidase 2	94	2	1.47	5	21	17
G1SQ45	A0A0C4DG98	THOC2	Uncharacterized protein	THO complex subunit 2	98	3	1.46	4	5	5
G1TEI2		FDX1	2Fe-2S ferredoxin-type domain-containing protein			1	1.46	2	3	20
G1SEE0	Q09028	RBBP4	RB binding protein 4, chromatin remodeling factor	Histone-binding protein RBBP4	100	2	1.46	6	5	23
G1TEN1	H7C5S0	ACTL6A	Uncharacterized protein	Actin-like protein 6A (Fragment)	99	3	1.46	4	6	15
G1U998		NOL6	Nucleolar protein 6			1	1.46	3	10	7
G1T6L7	F8WAR4	CHCHD3	MICOS complex subunit	MICOS complex subunit	78	2	1.46	3	4	9
G1SQZ4	Q9Y230	RUVBL2	RuvB-like helicase	RuvB-like 2	99	2	1.45	15	52	49
G1TIT1	O75477	ERLIN1	PHB domain-containing protein	Erlin-1	98	2	1.45	11	8	41
G1U450		XYLB	Xylulokinase			1	1.45	2	2	6
G1TAE2	Q15717	ELAVL1	ELAV-like protein	ELAV-like protein 1	99	2	1.45	10	42	32
G1SXF1		NFU1	Nfu_N domain-containing protein			1	1.45	4	9	22
G1SML5	Q9H9B4	SFXN1	Sidoreflexin	Sideroflexin-1	94	2	1.45	11	27	53
G1SW10	O94776	MTA2	Uncharacterized protein	Metastasis-associated protein MTA2	99	3	1.45	6	4	12
G1T7B5	Q9H0S4	DDX47	Uncharacterized protein	Probable ATP-dependent RNA helicase DDX47	97	3	1.44	2	5	6
G1T0M2		TRMT10C	SAM-dependent MTase TRM10-type domain-containing protein			1	1.44	4	5	15
G1TAU6		SERPINE1	SERPIN domain-containing protein			1	1.44	2	2	7
G1T657	A0A087WUK2	HNRNPDL	Heterogeneous nuclear ribonucleoprotein D like	Heterogeneous nuclear ribonucleoprotein D-like	92	2	1.44	6	15	18
G1SWW7	B4DJK0	SRSF5	Uncharacterized protein	Serine/arginine-rich-splicing factor 5	100	3	1.44	2	4	8
G1T933	Q05707	COL14A1	Uncharacterized protein	Collagen alpha-1(XIV) chain	94	3	1.44	34	65	27
G1SSM6	Q8WUM0	NUP133	Nucleoporin_C domain-containing protein	Nuclear pore complex protein Nup133	91	2	1.44	14	20	23
G1SD25	Q5SRE5	NUP188	Nucleoporin 188	Nucleoporin NUP188 homolog	94	2	1.44	10	11	9
G1SL42	Q9BPW8	NIPSNAP1	NIPSNAP domain-containing protein	Protein NipSnap homolog 1	95	2	1.44	5	4	26
G1T9L6		TOR1AIP1	Torsin 1A interacting protein 1			1	1.44	4	6	9
G1SFI7	P49915	GMPS	Uncharacterized protein	GMP synthase [glutamine-hydrolyzing]	99	3	1.44	6	6	10

	A0A0J9YVP6	PUF60		Poly(U)-binding-splicing factor PUF60 (Fragment)		4	1.44	8	14	23
G1TDJ9	F6Y5H0	RBMS1	RNA binding motif single stranded interacting protein 1	RNA-binding motif, single-stranded-interacting protein 1	97	2	1.44	2	3	14
G1SIB2	A0A087WVM4	MTHFD1L	Uncharacterized protein	Monofunctional C1-tetrahydrofolate synthase, mitochondrial	92	3	1.43	9	23	13
G1SKP2	H0Y8C6	IPO5	Importin N-terminal domain-containing protein	Importin-5 (Fragment)	99	2	1.43	28	58	42
G1U0Y6		ECI1	Enoyl-CoA delta isomerase 1			1	1.43	9	19	63
G1SYT7	G3V0E4	PMPCB	Uncharacterized protein	Mitochondrial-processing peptidase subunit beta	94	3	1.43	10	21	34
G1TEZ1	G3V198	NUP160	Uncharacterized protein	Nuclear pore complex protein Nup160 (Fragment)	93	3	1.42	14	21	18
	A0A024R4M0	RPS9		40S ribosomal protein S9		4	1.42	12	18	40
G1T0Q6	Q9UQE7	SMC3	Structural maintenance of chromosomes protein	Structural maintenance of chromosomes protein 3	100	2	1.42	8	8	9
	K7EK33	DAZAP1		DAZ-associated protein 1		4	1.42	4	6	18
G1SLM0	A0A087WTT1	PABPC1	Polyadenylate-binding protein	Polyadenylate-binding protein	99	2	1.42	18	18	38
G1SRD1		EXD2	3~-5~ exonuclease domain-containing protein			1	1.41	4	3	9
G1SRH7	P84103	SRSF3	RRM domain-containing protein	Serine/arginine-rich splicing factor 3	100	2	1.41	4	5	24
G1SQH0	P61254	RPL26	KOW domain-containing protein	60S ribosomal protein L26	100	2	1.41	6	11	28
G1SJW8	H3BND8	USP7	Uncharacterized protein	Ubiquitin carboxyl-terminal hydrolase (Fragment)	99	3	1.41	9	10	16
G1SE12	P13804	ETFA	Electron transfer flavoprotein subunit alpha	Electron transfer flavoprotein subunit alpha, mitochondrial	95	2	1.41	11	16	56
G1T237	F8VVM2	SLC25A3	Uncharacterized protein	Phosphate carrier protein, mitochondrial	86	3	1.41	14	115	48
G1T2J6	O75746	SLC25A12	Solute carrier family 25 member 12	Calcium-binding mitochondrial carrier protein Aralar1	95	2	1.41	12	2	34
G1T1G2	A0A1B0GWA2	AGPS	Alkylglycerone-phosphate synthase	Alkylglycerone-phosphate synthase (Fragment)	96	2	1.41	7	13	17
G1SU97	H0YD97	PDHX	Dihydrolipoamide acetyltransferase component of pyruvate dehydrogenase complex	Pyruvate dehydrogenase protein X component, mitochondrial (Fragment)	89	2	1.41	4	7	10
G1T095		TMEM147	Transmembrane protein 147			1	1.41	2	2	18
G1SV32	P18124	RPL7	Uncharacterized protein	60S ribosomal protein L7	98	3	1.41	13	14	46
G1U800	Q8NC56	LEMD2	MSC domain-containing protein	LEM domain-containing protein 2	79	2	1.40	5	3	19
G1SWK4	B4E1G1	DERL1	Derlin	Derlin	99	2	1.40	2	3	10
G1T6M1	Q96DA6	DNAJC19	J domain-containing protein	Mitochondrial import inner membrane translocase subunit TIM14	100	2	1.40	2	3	19
G1SPX2	Q6KC79	NIPBL	Nipped-B protein	Nipped-B-like protein	98	2	1.40	2	2	1
U3KM30	Q969X5	ERGIC1	Uncharacterized protein	Endoplasmic reticulum-Golgi intermediate compartment protein 1	99	3	1.40	3	4	18
G1U2T2	H0YIB4	SRSF9	Uncharacterized protein	Serine/arginine-rich-splicing factor 9 (Fragment)	65	3	1.40	3	4	15
G1T994	D6RGG3	COL12A1	Collagen alpha-1(XII) chain	Collagen alpha-1(XII) chain	95	2	1.40	117	677	55
G1SRX2	Q15029	EFTUD2	Tr-type G domain-containing protein	116 kDa U5 small nuclear ribonucleoprotein component	100	2	1.40	18	42	31
G1TNM3	P23396	RPS3	KH type-2 domain-containing protein	40S ribosomal protein S3	100	2	1.40	17	47	62
G1TBL0	E7ETZ4	BZW2	Basic leucine zipper and W2 domains 2	Basic leucine zipper and W2 domain-containing protein 2 (Fragment)	100	2	1.40	7	10	20
G1SHK6	Q5SSJ5	HP1BP3	Uncharacterized protein	Heterochromatin protein 1-binding protein 3	93	3	1.39	11	22	26
G1SSB5	C9JLU1	POLR2H	DNA-directed RNA polymerases I, II, and III subunit RPABC3	DNA-directed RNA polymerases I, II, and III subunit RPABC3 (Fragment)	100	2	1.39	2	2	29
G1SRZ0		TOR2A	Torsin family 2 member A			1	1.39	2	3	20
G1SD98	Q13523	PRPF4B	Pre-mRNA processing factor 4B	Serine/threonine-protein kinase PRP4 homolog	98	2	1.39	4	6	4
G1SGI8	O95881	TXNDC12	Thioredoxin domain-containing protein	Thioredoxin domain-containing protein 12	95	2	1.39	5	10	37
G1SHL9	Q15006	EMC2	TPR_REGION domain-containing protein	ER membrane protein complex subunit 2	99	2	1.39	6	13	34
G1STL1	Q8WUA2	PPIL4	Peptidyl-prolyl cis-trans isomerase	Peptidyl-prolyl cis-trans isomerase-like 4	97	2	1.39	2	3	6
G1TST9	A0A2R8Y212	CHD4	Uncharacterized protein	Chromodomain-helicase-DNA-binding protein 4	97	3	1.39	20	20	18
G1SW36	H0YDT8	EMC7	DUF2012 domain-containing protein	ER membrane protein complex subunit 7 (Fragment)	79	2	1.39	4	4	24
G1SME4	Q9BWF3	RBM4	RNA-binding protein 4	RNA-binding protein 4	99	2	1.39	7	7	20
G1TCT4	O95155	UBE4B	U-box domain-containing protein	Ubiquitin conjugation factor E4 B	95	2	1.39	2	2	3
G1STH0	Q15459	SF3A1	Uncharacterized protein	Splicing factor 3A subunit 1	98	3	1.39	8	11	13
G1SEW3	P49792	RANBP2	RAN binding protein 2	E3 SUMO-protein ligase RanBP2	84	2	1.38	17	33	8
G1T5A0	P23921	RRM1	Ribonucleoside-diphosphate reductase	Ribonucleoside-diphosphate reductase large subunit	98	2	1.38	3	4	7
G1TA37	O75531	BANF1	Uncharacterized protein	Barrier-to-autointegration factor	100	3	1.38	4	12	57
G1SDV5		NOL9	CLP1_P domain-containing protein			1	1.38	2	2	4
G1U2B5		PDXK	Phos_pyr_kin domain-containing protein			1	1.38	3	3	19
G1TCX6	P08240	SRPRA	SRP54 domain-containing protein	Signal recognition particle receptor subunit alpha	99	2	1.38	11	15	25
G1TUX2	A2A274	ACO2	Aconitate hydratase, mitochondrial	Aconitate hydratase, mitochondrial	94	2	1.38	25	55	42
G1TIS2		TIMM17B	Mitochondrial import inner membrane translocase subunit TIM17			1	1.38	2	3	22
G1SCF6		YARS2	Tyrosine--tRNA ligase			1	1.38	5	5	20
G1TVN1	I3L4X2	ABCC1	ATP binding cassette subfamily C member 1	Multidrug resistance-associated protein 1 (Fragment)	91	2	1.38	16	39	16
G1SKT4	P25705	ATP5F1A	ATP synthase subunit alpha	ATP synthase subunit alpha, mitochondrial	98	2	1.38	29	386	62
G1TCC8	O75934	BCAS2	Uncharacterized protein	Pre-mRNA-splicing factor SPF27	100	3	1.38	2	3	16
G1SPH7	Q9P2I0	CPSF2	Cleavage and polyadenylation specificity factor subunit 2	Cleavage and polyadenylation specificity factor subunit 2	99	2	1.38	4	6	6
G1T2N4	E7ET15	U2SURP	Uncharacterized protein	U2 snRNP-associated SURP motif-containing protein	99	3	1.38	6	12	8
G1TE08	H7C3P6	NUP98	Peptidase S59 domain-containing protein	Nuclear pore complex protein Nup98-Nup96 (Fragment)	90	2	1.38	9	11	7
G1U6X6	P55795	HNRNPH2	Uncharacterized protein	Heterogeneous nuclear ribonucleoprotein H2	100	3	1.38	10	11	34

O46373	P12235	SLC25A4	ADP/ATP translocase 1	ADP/ATP translocase 1	96	2	1.38	18	26	71
G1STF9	Q13347	EIF3I	Eukaryotic translation initiation factor 3 subunit I	Eukaryotic translation initiation factor 3 subunit I	100	2	1.37	10	11	41
G1TAW7		EIF2A	Eukaryotic translation initiation factor 2A			1	1.37	3	5	9
P42675	E9PCB6	NLN	Neurolysin, mitochondrial	Neurolysin, mitochondrial	94	2	1.37	6	12	15
G1T8H5		POLR2C	RPOLD domain-containing protein			1	1.37	2	3	11
	P49750-1	YLPM1		Isoform 1 of YLP motif-containing protein 1		4	1.37	4	6	4
G1SCT6	E9PB90	HK2	Uncharacterized protein	Hexokinase-2	96	3	1.37	10	3	16
G1SZG3	Q8NEW0	SLC30A7	Uncharacterized protein	Zinc transporter 7	97	3	1.37	4	4	18
G1T8C2	F8W7U8	MRE11	Double-strand break repair protein	Double-strand break repair protein	93	2	1.37	4	3	9
G1T6P0	Q9H7D0	DOCK5	Dedicator of cytokinesis 5	Dedicator of cytokinesis protein 5	96	2	1.37	3	4	3
G1T3S2	P54753	EPHB3	Uncharacterized protein	Ephrin type-B receptor 3	98	3	1.37	9	17	15
P30801		S100A6	Protein S100-A6			1	1.37	7	22	56
G1SX71		AGPAT5	1-acylglycerol-3-phosphate O-acyltransferase 5			1	1.37	2	3	11
G1SZ23	E9PCG9	BDH1	Uncharacterized protein	D-beta-hydroxybutyrate dehydrogenase, mitochondrial	87	3	1.37	9	13	32
G1TS38	H0YF06	CCDC90B	Uncharacterized protein	Coiled-coil domain-containing protein 90B, mitochondrial (Fragment)	94	3	1.37	2	3	18
G1SMH6	Q9P0M6	H2AFY2	Core histone macro-H2A	Core histone macro-H2A.2	99	2	1.37	6	5	23
G1SYD6	P02545	LMNA	Uncharacterized protein	Prelamin-A/C	98	3	1.37	33	91	52
G1SLQ3	P15559	NQO1	Flavodoxin_2 domain-containing protein	NAD(P)H dehydrogenase [quinone] 1	89	2	1.37	10	20	48
	G3V1C3	API5		Apoptosis inhibitor 5		4	1.37	8	18	27
G1T6E9	Q8N5K1	CISD2	ZnF_CDGSH domain-containing protein	CDGSH iron-sulfur domain-containing protein 2	99	2	1.37	4	8	32
G1TUK6	O14656	TOR1A	Torsin family 1 member A	Torsin-1A	90	2	1.36	7	7	25
G1SDJ7	B1ANR0	PABPC4	Polyadenylate-binding protein	Polyadenylate-binding protein	92	2	1.36	17	13	27
G1SWU1	P55809	OXCT1	Succinyl-CoA:3-ketoacid-coenzyme A transferase	Succinyl-CoA:3-ketoacid coenzyme A transferase 1, mitochondrial	94	2	1.36	18	109	52
G1T2I5	F8W914	RTN4	Reticulon	Reticulon	98	2	1.36	8	281	13
G1SQF9	Q8WXF1	PSPC1	Uncharacterized protein	Paraspeckle component 1	98	3	1.36	6	8	15
	P56385	ATP5ME		ATP synthase subunit e, mitochondrial		4	1.36	3	3	22
	P19623	SRM		Spermidine synthase		4	1.36	3	4	17
G1SHG0	P62899	RPL31	Uncharacterized protein	60S ribosomal protein L31	100	3	1.36	6	14	45
	A0A087WUC6	SPCS2		Signal peptidase complex subunit 2		4	1.36	6	16	33
G1SYL3	E9PFH4	TNPO3	Xpo1 domain-containing protein	Transportin-3	96	2	1.36	6	9	12
G1TTL1	A0A0B4J1W3	NAA15	Uncharacterized protein	N-alpha-acetyltransferase 15, NatA auxiliary subunit	99	3	1.36	7	12	13
G1SRV1	K7ELV2	SEH1L	Uncharacterized protein	Nucleoporin SEH1 (Fragment)	92	3	1.36	5	6	18
G1SDD3		KCTD10	BTB domain-containing protein			1	1.35	2	2	9
G1SI22		AKAP12	A-kinase anchoring protein 12			1	1.35	11	16	17
G1T5A6	Q5T4D3	TMTC4	Transmembrane and tetratricopeptide repeat containing 4	Protein O-mannosyl-transferase TMTC4	92	2	1.35	5	5	11
G1SST6		ARMC10	Arm_2 domain-containing protein			1	1.35	2	3	12
G1TY34		ITGA4	Integrin_alpha2 domain-containing protein			1	1.35	4	6	7
G1T9N2	O75947	ATP5PD	ATP synthase subunit d, mitochondrial	ATP synthase subunit d, mitochondrial	91	2	1.35	10	23	70
G1T855	C9J5X1	IGF1R	Tyrosine-protein kinase receptor	Tyrosine-protein kinase receptor	93	2	1.35	8	15	10
G1SU33		NSUN2	NOP2/Sun RNA methyltransferase family member 2			1	1.35	3	3	7
G1STP6	P35221	CTNNA1	Catenin alpha-1	Catenin alpha-1	99	2	1.35	31	81	48
	O75937	DNAJC8		DnaJ homolog subfamily C member 8		4	1.35	2	2	7
G1TCE9		HSDL2	SCP2 domain-containing protein			1	1.35	8	17	28
G1SNE9	A0A0G2JQ41	ABR	Uncharacterized protein	Active breakpoint cluster region-related protein (Fragment)	100	3	1.35	7	7	18
G1T4Z1	Q07954	LRP1	Uncharacterized protein	Prolow-density lipoprotein receptor-related protein 1	98	3	1.35	81	11	26
G1T194	Q9ULX6	AKAP8L	A-kinase anchoring protein 8 like	A-kinase anchor protein 8-like	86	2	1.35	4	5	10
	Q96PK6-5	RBM14		Isoform 5 of RNA-binding protein 14		4	1.34	5	3	24
G1TSP3	Q9NZ01	TECR	Uncharacterized protein	Very-long-chain enoyl-CoA reductase	98	3	1.34	5	12	15
G1TTA5	H0Y8C3	MTCH1	Uncharacterized protein	Mitochondrial carrier homolog 1 (Fragment)	93	3	1.34	7	24	23
G1SF82	P62072	TIMM10	zf-Tim10_DDP domain-containing protein	Mitochondrial import inner membrane translocase subunit Tim10	100	2	1.34	2	5	36
	E9PB61	ALYREF		THO complex subunit 4		4	1.34	5	53	25
G1SLI0	Q96A33	CCDC47	Uncharacterized protein	Coiled-coil domain-containing protein 47	98	3	1.34	8	14	22
	P62424	RPL7A		60S ribosomal protein L7a		4	1.34	12	35	45
G1U150	M0QXU7	TIMM44	Mitochondrial import inner membrane translocase subunit TIM44	Mitochondrial import inner membrane translocase subunit TIM44 (Fragment)	83	2	1.34	7	12	18
G1SSL5	Q14CX7	NAA25	TPR_REGION domain-containing protein	N-alpha-acetyltransferase 25, NatB auxiliary subunit	96	2	1.34	5	6	11
G1SVW9		VEZT	Vezatin domain-containing protein			1	1.34	2	2	4
G1SVD7		SYNJ2BP-COX16	PDZ domain-containing protein			1	1.34	2	3	15
A0A0A0MQQ6	D6RBW1	EIF4E	Eukaryotic translation initiation factor 4E	Eukaryotic translation initiation factor 4E	98	2	1.34	3	5	20
G1SFG0	A0A087WVP1	FAT1	Uncharacterized protein	Protocadherin Fat 1	92	3	1.34	11	15	4
G1T7T2	Q15424	SAFB	Scaffold attachment factor B	Scaffold attachment factor B1	86	2	1.33	6	8	14

U3KPB2	P61009	SPCS3	Signal peptidase complex subunit 3	Signal peptidase complex subunit 3	100	2	1.33	2	5	13
G1TAA4	O75844	ZMPSTE24	CAAX prenyl protease	CAAX prenyl protease 1 homolog	96	2	1.33	3	4	9
G1SFH9	A0A2R8Y543	CTNNB1	Uncharacterized protein	Catenin beta-1	100	3	1.33	25	56	46
G1TA59	P50213	IDH3A	Isocitrate dehydrogenase [NAD] subunit, mitochondrial	Isocitrate dehydrogenase [NAD] subunit alpha, mitochondrial	98	2	1.33	12	38	40
G1SU17		NSDHL	3Beta_HSD domain-containing protein			1	1.33	2	2	10
Q9TT15	P21796	VDAC1	Voltage-dependent anion-selective channel protein 1	Voltage-dependent anion-selective channel protein 1	100	2	1.33	18	416	82
G1SLT8	P31942	HNRNPH3	Uncharacterized protein	Heterogeneous nuclear ribonucleoprotein H3	100	3	1.33	6	6	24
G1SFV7	Q16531	DDB1	Damage specific DNA binding protein 1	DNA damage-binding protein 1	100	2	1.33	18	26	19
	P62304	SNRPE		Small nuclear ribonucleoprotein E		4	1.33	3	12	52
G1SDN4	O60506-3	SYNCRIP	Uncharacterized protein	Isoform 3 of Heterogeneous nuclear ribonucleoprotein Q	100	3	1.33	22	49	49
G1SJQ6	O00519	FAAH	Amidase domain-containing protein	Fatty-acid amide hydrolase 1	91	2	1.32	4	6	11
G1TBU9	A0A0B4J2A4	ACAA2	Uncharacterized protein	3-ketoacyl-CoA thiolase, mitochondrial	89	3	1.32	15	61	64
G1T2K6	Q8NBU5	ATAD1	AAA domain-containing protein	ATPase family AAA domain-containing protein 1	100	2	1.32	2	5	10
G1SKZ8	P62906	RPL10A	Ribosomal protein	60S ribosomal protein L10a	100	2	1.32	13	46	53
G1TJR5	Q9NZL4	HSPBP1	HSPA (Hsp70) binding protein 1	Hsp70-binding protein 1	96	2	1.32	4	7	22
G1TIP5	C9JCC6	DRAP1	CBFD_NFYB_HMF domain-containing protein	Dr1-associated corepressor	87	2	1.32	2	3	11
G1T647		GCLM	Glutamate-cysteine ligase modifier subunit			1	1.32	3	4	16
G1T1Y3	A0A384DVU0	PNPLA6	Patatin like phospholipase domain containing 6	Neuropathy target esterase	98	2	1.32	3	3	5
G1SED9	B0QY89	EIF3L	Eukaryotic translation initiation factor 3 subunit L	Eukaryotic translation initiation factor 3 subunit L	97	2	1.32	18	29	41
P62497	P62495	ETF1	Eukaryotic peptide chain release factor subunit 1	Eukaryotic peptide chain release factor subunit 1	100	2	1.32	15	36	52
G1SFR8	P25398	RPS12	40S ribosomal protein S12	40S ribosomal protein S12	100	2	1.31	4	12	32
G1TWS0	B3KY94	CDIPT	CDP-diacylglycerol--inositol 3-phosphatidyltransferase	CDP-diacylglycerol--inositol 3-phosphatidyltransferase	75	2	1.31	4	7	22
G1T925		GPX8	Glutathione peroxidase			1	1.31	4	7	20
O46638	Q00688	FKBP3	Peptidyl-prolyl cis-trans isomerase FKBP3	Peptidyl-prolyl cis-trans isomerase FKBP3	96	2	1.31	4	7	21
G1U516	E9PHV4	POLR2D	RPOL4c domain-containing protein	DNA-directed RNA polymerase II subunit RPB4	76	2	1.31	2	4	25
G1SCW7	Q9UG63	ABCF2	Uncharacterized protein	ATP-binding cassette sub-family F member 2	99	3	1.31	6	8	11
G1TXS5	Q9UGP8	SEC63	J domain-containing protein	Translocation protein SEC63 homolog	93	2	1.31	12	22	21
	F6RGN5	SLC25A10		Mitochondrial dicarboxylate carrier		4	1.31	3	6	11
G1SGG6	P48449	LSS	Terpene cyclase/mutase family member	Lanosterol synthase	89	2	1.31	12	20	20
G1TCA0	Q96KR1	ZFR	DZF domain-containing protein	Zinc finger RNA-binding protein	99	2	1.31	3	2	4
	B4DQT1	MAEA		Macrophage erythroblast attacher		4	1.30	3	3	8
G1SVY6	Q9BTX1	NDC1	Uncharacterized protein	Nucleoporin NDC1	88	3	1.30	2	2	5
	Q13363	CTBP1		C-terminal-binding protein 1		4	1.30	4	3	10
G1SPK4	P05388	RPLP0	60S acidic ribosomal protein P0	60S acidic ribosomal protein P0	98	2	1.30	13	9	68
G1TZI2		PGAM5	PGAM family member 5, mitochondrial serine/threonine protein phosphatase			1	1.30	2	2	9
G1T3D9	P62241	RPS8	40S ribosomal protein S8	40S ribosomal protein S8	94	2	1.30	6	22	37
	Q9Y5L4	TIMM13		Mitochondrial import inner membrane translocase subunit Tim13		4	1.30	3	4	37
G1TGX2		ZCCHC8	Zinc finger CCHC-type containing 8			1	1.30	2	4	5
G1T3U3	A0A0G2JRV3	SMARCB1	SWI/SNF related, matrix associated, actin dependent regulator of chromatin, subfamily b, member 1	SWI/SNF-related matrix-associated actin-dependent regulator of chromatin subfamily B member 1	85	2	1.30	4	7	26
G1T6J2		APOO	MICOS complex subunit			1	1.30	5	8	46
G1SMR7	P30050	RPL12	Uncharacterized protein	60S ribosomal protein L12	100	3	1.30	8	22	53
G1T920	P49903	SEPHS1	Uncharacterized protein	Selenide, water dikinase 1	100	3	1.30	5	6	20
G1SLC2	O00303	EIF3F	Eukaryotic translation initiation factor 3 subunit F	Eukaryotic translation initiation factor 3 subunit F	99	2	1.29	9	23	44
G1TEG1	O95470	SGPL1	Uncharacterized protein	Sphingosine-1-phosphate lyase 1	90	3	1.29	9	17	22
G1SCY7	Q8N766	EMC1	EMC1_C domain-containing protein	ER membrane protein complex subunit 1	96	2	1.29	15	23	23
G1SMY6	O14980	XPO1	Importin N-terminal domain-containing protein	Exportin-1	99	2	1.29	16	26	25
G1SXZ9	Q86WA6	BPHL	Biphenyl hydrolase like	Valacyclovir hydrolase	89	2	1.29	7	9	25
	Q9NRZ7	AGPAT3		1-acyl-sn-glycerol-3-phosphate acyltransferase gamma		4	1.29	2	3	4
G1SU75	O95140	MFN2	Mitofusin 2	Mitofusin-2	92	2	1.29	10	15	18
G1TDK8	F8VXC8	SMARCC2	Uncharacterized protein	SWI/SNF complex subunit SMARCC2	93	3	1.29	8	4	10
G1SIV7	O43776	NARS	AA_TRNA_LIGASE_II domain-containing protein	Asparagine--tRNA ligase, cytoplasmic	91	2	1.29	15	35	36
G1SDH8	Q5T9L3	WLS	Uncharacterized protein	Protein wntless homolog	98	3	1.29	5	6	12
G1SSL0	P30040	ERP29	Endoplasmic reticulum resident protein 29	Endoplasmic reticulum resident protein 29	93	2	1.28	9	37	46
G1T7P9	Q96DI7	SNRNP40	WD_REPEATS_REGION domain-containing protein	U5 small nuclear ribonucleoprotein 40 kDa protein	99	2	1.28	2	3	13
G1SUC8	P60228	EIF3E	Eukaryotic translation initiation factor 3 subunit E	Eukaryotic translation initiation factor 3 subunit E	100	2	1.28	14	26	41
G1SEC8	Q8IWA4	MFN1	Dynamin-type G domain-containing protein	Mitofusin-1	92	2	1.28	8	13	17
	Q13045	FLII		Protein flightless-1 homolog		4	1.28	6	5	6
O18757	Q6NUK1	SLC25A24	Calcium-binding mitochondrial carrier protein SCaMC-1	Calcium-binding mitochondrial carrier protein SCaMC-1	95	2	1.28	14	5	43
G1SLD7	Q9NXF1	TEX10	Ipi1_N domain-containing protein	Testis-expressed protein 10	94	2	1.28	3	4	5
G1TIT4		PTTG1IP	PTTG1 interacting protein			1	1.28	2	3	11

G1SEW1	R4GMQ1	KDM1A	Lysine-specific histone demethylase	Lysine-specific histone demethylase	96	2	1.28	3	7	7
G1T419	Q92621	NUP205	Uncharacterized protein	Nuclear pore complex protein Nup205	96	3	1.28	24	16	19
G1SLF5	H0YG54	REXO2	Exonuclease domain-containing protein	Oligoribonuclease, mitochondrial	98	2	1.28	3	5	22
	O43290	SART1		U4/U6.U5 tri-snRNP-associated protein 1		4	1.28	2	2	5
G1SG61		TPST2	Protein-tyrosine sulfotransferase			1	1.28	2	2	5
G1SYI3	A0A087X1B2	USP39	Uncharacterized protein	U4/U6.U5 tri-snRNP-associated protein 2	99	3	1.28	5	7	14
G1SYB9		ITGA3	Integrin subunit alpha 3			1	1.28	2	5	4
G1TVK1		NOTCH2	Notch 2			1	1.28	4	5	3
G1T093	Q9NZJ4-2	SACS	Uncharacterized protein	Isoform 2 of Sacsin	96	3	1.27	5	4	2
G1TH33	P38117	ETFB	Electron transfer flavoprotein subunit beta	Electron transfer flavoprotein subunit beta	95	2	1.27	10	7	47
G1SK67	Q13547	HDAC1	Histone deacetylase	Histone deacetylase 1	99	2	1.27	6	4	23
G1SHU8	D3YTB1	RPL32	Uncharacterized protein	60S ribosomal protein L32 (Fragment)	88	3	1.27	4	10	29
G1SRB1	Q16134	ETFDH	4Fe-4S ferredoxin-type domain-containing protein	Electron transfer flavoprotein-ubiquinone oxidoreductase, mitochondrial	94	2	1.27	9	12	22
G1SZI5	P62269	RPS18	Uncharacterized protein	40S ribosomal protein S18	100	3	1.27	9	19	44
G1T619		MCUB	MCU domain-containing protein			1	1.27	3	3	14
G1SLI8	A0A0A0MSJ0	DDX42	Uncharacterized protein	ATP-dependent RNA helicase DDX42	96	3	1.27	3	6	7
G1T8H7	A0A2R8YCL1	GRB10	Growth factor receptor bound protein 10	Growth factor receptor-bound protein 10	90	2	1.27	2	2	4
G1SGG2	V9GYM8	ARHGEF2	Uncharacterized protein	Rho guanine nucleotide exchange factor 2	95	3	1.27	13	22	21
G1SR19	P20594	NPR2	Guanylate cyclase	Atrial natriuretic peptide receptor 2	95	2	1.27	7	9	10
G1T4Z2	P53396	ACLY	ATP-citrate synthase	ATP-citrate synthase	98	2	1.27	17	30	23
	P40429	RPL13A		60S ribosomal protein L13a		4	1.27	9	13	37
G1SML9	P31689	DNAJA1	Uncharacterized protein	DnaJ homolog subfamily A member 1	100	3	1.27	8	22	34
G1SVQ8	F8WF48	SEC62	Uncharacterized protein	Translocation protein SEC62	100	3	1.27	2	3	5
G1SF32	O94826	TOMM70	TPR_REGION domain-containing protein	Mitochondrial import receptor subunit TOM70	97	2	1.27	16	41	24
G1SRW4	Q9Y6C2	EMILIN1	Elastin microfibril interfacer 1	EMILIN-1	87	2	1.26	5	5	6
G1TMM7	H0YN26	ANP32A	LRRcap domain-containing protein	Acidic leucine-rich nuclear phosphoprotein 32 family member A	93	2	1.26	7	10	39
G1T5V3	P61106	RAB14	Uncharacterized protein	Ras-related protein Rab-14	100	3	1.26	12	30	73
G1TD36	Q6PML9	SLC30A9	Uncharacterized protein	Zinc transporter 9	96	3	1.26	4	5	8
G1SSC9	O95373	IPO7	Importin N-terminal domain-containing protein	Importin-7	100	2	1.26	18	40	29
G1SM52	Q96AG4	LRRC59	Uncharacterized protein	Leucine-rich repeat-containing protein 59	97	3	1.26	13	26	48
G1T4A5	P02452	COL1A1	Collagen alpha-1(I) chain	Collagen alpha-1(I) chain	91	2	1.26	34	27	45
G1SIA3	Q8N3C0	ASCC3	Uncharacterized protein	Activating signal cointegrator 1 complex subunit 3	94	3	1.25	7	8	5
G1SPB6	E7EPM6	ACSL1	AMP-binding domain-containing protein	Long-chain-fatty-acid--CoA ligase 1	80	2	1.25	5	10	23
G1U3V0	Q9BX68	HINT2	HIT domain-containing protein	Histidine triad nucleotide-binding protein 2, mitochondrial	94	2	1.25	4	6	41
G1SE65	A0A0C4DG95	PXK	Uncharacterized protein	PX domain-containing protein kinase-like protein	94	3	1.25	2	2	7
G1SW65		ATP5MC2	ATP-synt_C domain-containing protein			1	1.25	2	2	21
G1T2G3	Q5VTR2	RNF20	E3 ubiquitin protein ligase	E3 ubiquitin-protein ligase BRE1A	98	2	1.25	6	4	10
G1TGI6	A0A0C4DG17	RPSA	40S ribosomal protein SA	40S ribosomal protein SA	98	2	1.25	10	32	46
G1TBW2	E7EQB8	IDH3G	Isocitrate dehydrogenase [NAD] subunit, mitochondrial	Isocitrate dehydrogenase [NAD] subunit, mitochondrial	94	2	1.25	6	9	27
G1T511	A0A3F2YNY6	PRPF40A	Pre-mRNA processing factor 40 homolog A	Pre-mRNA-processing factor 40 homolog A	83	2	1.25	5	5	7
G1SK22	P62979	RPS27A	Ubiquitin-like domain-containing protein	Ubiquitin-40S ribosomal protein S27a	100	2	1.25	8	51	47
G1U797	P51114	FXR1	Uncharacterized protein	Fragile X mental retardation syndrome-related protein 1	91	3	1.25	12	10	19
G1SVU0	C9JAZ1	MTX2	Uncharacterized protein	Metaxin-2 (Fragment)	89	3	1.25	4	14	37
G1TD91	O43809	NUDT21	Nudix hydrolase domain-containing protein	Cleavage and polyadenylation specificity factor subunit 5	100	2	1.25	4	9	30
G1TB17	A0A1B0GWF8	ADSL	Adenylosuccinate lyase	Adenylosuccinate lyase (Fragment)	95	2	1.25	4	3	13
G1SFU0	P51149	RAB7A	Ras-related protein Rab-7a	Ras-related protein Rab-7a	100	2	1.25	12	25	61
G1TNW8	Q9Y512	SAMM50	SAMM50 sorting and assembly machinery component	Sorting and assembly machinery component 50 homolog	96	2	1.25	9	10	35
G1TA10		TMEM109	Transmembrane protein 109			1	1.25	3	6	9
G1U383	A0A087WYN9	DHX29	ATP-dependent RNA helicase DHX29	ATP-dependent RNA helicase DHX29	95	2	1.25	7	7	6
G1SWU9		FAM20B	Fam20C domain-containing protein			1	1.25	2	4	7
G1SCT9	A0A087WU53	MAGT1	Uncharacterized protein	Magnesium transporter protein 1	97	3	1.25	7	8	24
G1U724	Q9UBE0	SAE1	SUMO1 activating enzyme subunit 1	SUMO-activating enzyme subunit 1	88	2	1.25	8	14	26
G1SH80	O14874	BCKDK	Protein-serine/threonine kinase	[3-methyl-2-oxobutanoate dehydrogenase [lipoamide]] kinase, mitochondrial	97	2	1.24	2	9	6
G1SK61	Q9UIW2	PLXNA1	Plexin A1	Plexin-A1	91	2	1.24	3	4	4
G1SJH8	K7EP90	RBM42	RRM domain-containing protein	RNA-binding protein 42	97	2	1.24	2	3	8
G1T782	Q9Y6N5	SQOR	Pyr_redox_2 domain-containing protein	Sulfide:quinone oxidoreductase, mitochondrial	93	2	1.24	6	14	18
G1T5Y2	Q9NUQ9	FAM49B	Uncharacterized protein	Protein FAM49B	100	3	1.24	3	2	16
G1TFZ7	O15355	PPM1G	PPM-type phosphatase domain-containing protein	Protein phosphatase 1G	97	2	1.24	3	5	10
	P62701	RPS4X		40S ribosomal protein S4, X isoform		4	1.24	15	38	52
	Q58FF6	HSP90AB4P		Putative heat shock protein HSP 90-beta 4		4	1.24	4	10	9

G1SRS0	D6RBS5	ELMOD2	ELMO domain-containing protein	ELMO domain-containing protein 2 (Fragment)	93	2	1.24	2	2	6
G1SG07	Q8IWT6	LRRC8A	Leucine rich repeat containing 8 VRAC subunit A	Volume-regulated anion channel subunit LRRC8A	98	2	1.24	3	4	8
G1TVT0	P19338	NCL	Nucleolin	Nucleolin	83	2	1.24	23	23	34
G1SIC7	Q01432	AMPD3	AMP deaminase	AMP deaminase 3	96	2	1.24	3	2	5
G1SN16	P27695	APEX1	DNA-(apurinic or apyrimidinic site) lyase	DNA-(apurinic or apyrimidinic site) lyase	95	2	1.24	5	13	28
G1SJ66	C9JME2	FARP1	Uncharacterized protein	FERM, ARHGEF and pleckstrin domain-containing protein 1	90	3	1.23	28	63	38
G1U2J5	Q8NE01	CNNM3	Uncharacterized protein	Metal transporter CNNM3	88	3	1.23	2	2	7
G1T1U7	A0A494C1T2	MTHFD1	Uncharacterized protein	C-1-tetrahydrofolate synthase, cytoplasmic (Fragment)	90	3	1.23	14	20	20
G1T897	B4DP72	COQ5	2-methoxy-6-polyprenyl-1,4-benzoquinol methylase, mitochondrial	2-methoxy-6-polyprenyl-1,4-benzoquinol methylase, mitochondrial	77	2	1.23	2	3	12
G1SLW8	Q7L2H7	EIF3M	Eukaryotic translation initiation factor 3 subunit M	Eukaryotic translation initiation factor 3 subunit M	100	2	1.23	12	27	48
G1SL03	Q9BY32	ITPA	Inosine triphosphate pyrophosphatase	Inosine triphosphate pyrophosphatase	92	2	1.23	4	3	42
P67828	P48729	CSNK1A1	Casein kinase I isoform alpha	Casein kinase I isoform alpha	100	2	1.23	4	6	13
G1SEI8		NUP214	Nup214_FG domain-containing protein			1	1.23	4	9	4
G1SWS6	A0A3B3IRN5	FMOD	Fibromodulin	Fibromodulin	92	2	1.23	2	2	7
P30947	P08238	HSP90AB1	Heat shock protein HSP 90-beta	Heat shock protein HSP 90-beta	99	2	1.23	34	102	61
G1TB39	P29353	SHC1	Uncharacterized protein	SHC-transforming protein 1	97	3	1.23	2	2	6
G1TQ31	A0A2R8YD58	CSNK2A1	Casein kinase II subunit alpha	Casein kinase II subunit alpha	97	2	1.23	4	6	21
G1SME1		NOP14	NOP14 nucleolar protein			1	1.23	3	5	6
G1T0N4	P06748	NPM1	Uncharacterized protein	Nucleophosmin	89	3	1.23	11	48	38
G1T5R3		TCF25	Transcription factor 25			1	1.23	3	3	10
G1SLM1	Q9HB71	CACYBP	Uncharacterized protein	Calcyclin-binding protein	92	3	1.23	5	4	22
G1T9M7	E9PK47	PYGL	Alpha-1,4 glucan phosphorylase	Alpha-1,4 glucan phosphorylase	94	2	1.23	15	12	25
	P35268	RPL22		60S ribosomal protein L22		4	1.23	2	9	32
	E9PAV3-2	NACA		Isoform skNAC-2 of Nascent polypeptide-associated complex subunit alpha, muscle-specific form		4	1.22	6	35	11
G1SVP9	G3V4T2	PABPN1	RRM domain-containing protein	Polyadenylate-binding protein 2	99	2	1.22	2	2	6
	G8JLG1	SMC1A		Structural maintenance of chromosomes protein		4	1.22	5	6	6
G1TPZ3	Q96GK7	FAHD2A	FAA_hydrolase domain-containing protein	Fumarylacetoacetate hydrolase domain-containing protein 2A	90	2	1.22	3	3	12
	P18583-2	SON		Isoform A of Protein SON		4	1.22	2	2	1
G1SRA8	P41091	EIF2S3	Eukaryotic translation initiation factor 2 subunit 3	Eukaryotic translation initiation factor 2 subunit 3	92	2	1.22	12	27	36
G1SZZ2	P49458	SRP9	Signal recognition particle 9 kDa protein	Signal recognition particle 9 kDa protein	92	2	1.22	3	4	30
G1T301	A0A0A0MRN4	ZNF326	Uncharacterized protein	DBIRD complex subunit ZNF326	96	3	1.22	4	4	10
G1T673	Q99653	CHP1	Calcineurin like EF-hand protein 1	Calcineurin B homologous protein 1	98	2	1.22	2	4	17
G1T5M0	P04181	OAT	Uncharacterized protein	Ornithine aminotransferase, mitochondrial	91	3	1.22	16	56	54
G1SI85	A0A0A0MS41	SFXN3	Uncharacterized protein	Sidoreflexin	94	3	1.22	13	19	39
G1TBH6	Q9P0S9	TMEM14C	Uncharacterized protein	Transmembrane protein 14C	92	3	1.22	3	4	66
G1SVD5	P55265	ADAR	Uncharacterized protein	Double-stranded RNA-specific adenosine deaminase	80	3	1.21	18	29	23
	P52815	MRPL12		39S ribosomal protein L12, mitochondrial		4	1.21	2	3	13
G1T9P2		CARKD	ATP-dependent (S)-NAD(P)H-hydrate dehydratase			1	1.21	6	11	31
G1SNY3	Q68E01	INTS3	Uncharacterized protein	Integrator complex subunit 3	99	3	1.21	6	6	12
G1TT27	E9PKZ0	RPL8	Ribosomal_L2_C domain-containing protein	60S ribosomal protein L8 (Fragment)	100	2	1.21	6	12	35
G1TCB7	Q5LJA9	UCHL5	Ubiquitin carboxyl-terminal hydrolase	Ubiquitin carboxyl-terminal hydrolase (Fragment)	91	2	1.21	7	7	17
G1TTS1		FUNDC2	FUN14 domain containing 2			1	1.21	2	3	12
	B1ANM7	FAF1		FAS-associated factor 1		4	1.21	3	3	7
G1T006	Q9UMX5	NENF	Neudesin neurotrophic factor	Neudesin	98	2	1.21	3	4	19
	B5MDE0	RFT1		Protein RFT1 homolog		4	1.21	2	3	4
G1STG2	Q9Y5M8	SRPRB	SRP receptor subunit beta	Signal recognition particle receptor subunit beta	94	2	1.21	11	20	49
O77622		CCT6	T-complex protein 1 subunit zeta			1	1.21	23	15	54
G1SUD2	Q6UW02	CYP20A1	Uncharacterized protein	Cytochrome P450 20A1	88	3	1.21	18	23	46
G1SGR9	A0A494C1K3	GTF2I	General transcription factor IIi	General transcription factor II-I	97	2	1.21	4	6	9
G1TFM5	M0R0F0	RPS5	Ribosomal_S7 domain-containing protein	40S ribosomal protein S5 (Fragment)	100	2	1.21	8	64	56
G1SNZ3	P55060	CSE1L	Chromosome segregation 1 like	Exportin-2	99	2	1.21	13	35	26
G1SMZ5	Q14152	EIF3A	Eukaryotic translation initiation factor 3 subunit A	Eukaryotic translation initiation factor 3 subunit A	93	2	1.21	31	60	27
G1SQG1	H0YK61	EMC4	ER membrane protein complex subunit 4	ER membrane protein complex subunit 4	97	2	1.21	2	3	32
G1SU02	A0A1W2PP11	PARL	Rhomboid domain-containing protein	Presenilins-associated rhomboid-like protein, mitochondrial	94	2	1.21	2	2	6
G1T8D7	Q8IUX7	AEBP1	F5/8 type C domain-containing protein	Adipocyte enhancer-binding protein 1	76	2	1.21	5	6	8
G1SZM2	A0A0A0MRG2	APP	Amyloid-beta A4 protein	Amyloid-beta precursor protein	99	2	1.21	7	9	11
G1SSS4		CLCN6	Chloride transport protein 6			1	1.21	2	2	4
G1T5N5	C9IZ01	GFM1	Elongation factor G, mitochondrial	Elongation factor G, mitochondrial	93	2	1.21	8	11	19
U3KMP1	P61026	RAB10	Uncharacterized protein	Ras-related protein Rab-10	100	3	1.21	9	10	54
G1SG63	P55011	SLC12A2	Uncharacterized protein	Solute carrier family 12 member 2	97	3	1.21	3	2	4

G1U9T1	Q99832	CCT7	T-complex protein 1 subunit eta	T-complex protein 1 subunit eta	97	2	1.21	26	8	60
G1SSR8	O76031	CLPX	Uncharacterized protein	ATP-dependent Clp protease ATP-binding subunit clpX-like, mitochondrial	97	3	1.21	5	4	13
G1T601		CLYBL	HpcH_HpaI domain-containing protein			1	1.21	4	7	19
G1SRY7	K7ESP4	DCAKD	Uncharacterized protein	Dephospho-CoA kinase domain-containing protein (Fragment)	92	3	1.21	4	7	29
G1SGM2	O14744	PRMT5	Protein arginine N-methyltransferase 5	Protein arginine N-methyltransferase 5	98	2	1.20	6	10	19
G1SRE8		XRCC5	Ku domain-containing protein			1	1.20	7	16	20
G1U7L4	P11021	HSPA5	Heat shock protein family A (Hsp70) member 5	Endoplasmic reticulum chaperone BiP	99	2	1.20	34	801	62
	H3BQQ9	UBE2I		SUMO-conjugating enzyme UBC9 (Fragment)		4	1.20	3	7	40
G1SYA5	X6RA14	ESD	S-formylglutathione hydrolase	S-formylglutathione hydrolase	88	2	1.20	8	18	45
G1T8B3	Q8IZL8	PELP1	Uncharacterized protein	Proline-, glutamic acid- and leucine-rich protein 1	91	3	1.20	5	8	6
G1TAM8	P61289	PSME3	Uncharacterized protein	Proteasome activator complex subunit 3	95	3	1.20	8	10	37
G1SUU2	A0A087WXS7	ASNA1	ATPase ASNA1	ATPase ASNA1	93	2	1.20	9	12	44
G1SZF7	P48735	IDH2	Isocitrate dehydrogenase [NADP]	Isocitrate dehydrogenase [NADP], mitochondrial	96	2	1.20	20	129	47
G1T7W7	P46977	STT3A	Uncharacterized protein	Dolichyl-diphosphooligosaccharide--protein glycosyltransferase subunit STT3A	100	3	1.20	15	51	26
G1TVY5	C9JZR2	CTNND1	Uncharacterized protein	Catenin delta-1	97	3	1.20	19	47	35
G1SYR9	B9A067	IMMT	MICOS complex subunit MIC60	MICOS complex subunit MIC60	89	2	1.20	31	58	50
Q28618	P67809	YBX1	Nuclease-sensitive element-binding protein 1	Nuclease-sensitive element-binding protein 1	99	2	1.20	4	93	22
G1T2J0	P14923	JUP	Uncharacterized protein	Junction plakoglobin	99	3	1.20	5	10	10
G1T7U4	Q9Y4E8	USP15	Ubiquitin carboxyl-terminal hydrolase	Ubiquitin carboxyl-terminal hydrolase 15	99	2	1.20	4	3	7
G1T8Y0		COASY	CTP_transf_like domain-containing protein			1	1.19	2	3	5
G1SXX5	A0A2R8Y4F5	HADHA	Uncharacterized protein	Trifunctional enzyme subunit alpha, mitochondrial	86	3	1.19	30	151	52
G1TBK0		ANKMY2	Ankyrin repeat and MYND domain containing 2			1	1.19	2	3	5
G1SF00	Q9BSJ2	TUBGCP2	Gamma-tubulin complex component	Gamma-tubulin complex component 2	90	2	1.19	5	4	7
	P30044	PRDX5		Peroxiredoxin-5, mitochondrial		4	1.19	5	9	33
G1T3L2	K7ERF1	EIF3K	Eukaryotic translation initiation factor 3 subunit K	Eukaryotic translation initiation factor 3 subunit K	87	2	1.19	7	13	44
P67873	Q5SRQ6	CSNK2B	Casein kinase II subunit beta	Casein kinase II subunit beta	100	2	1.19	7	10	51
	P13639	EEF2		Elongation factor 2		4	1.19	37	288	58
G1T361	Q96I99	SUCLG2	Succinate--CoA ligase [GDP-forming] subunit beta, mitochondrial	Succinate--CoA ligase [GDP-forming] subunit beta, mitochondrial	97	2	1.19	17	39	49
G1TLK9	P49748	ACADVL	Uncharacterized protein	Very long-chain specific acyl-CoA dehydrogenase, mitochondrial	87	3	1.19	22	64	49
	A6XGL3	PRSS1		Protease serine 1		4	1.19	2	11	8
	J3KN01	AFDN		Afadin		4	1.19	2	3	2
G1ST56	Q96P70	IPO9	Importin N-terminal domain-containing protein	Importin-9	99	2	1.18	6	13	15
G1SMK9	Q9ULC3	RAB23	Uncharacterized protein	Ras-related protein Rab-23	97	3	1.18	4	6	22
G1T0H0	Q9NX46	ADPRHL2	ADP-ribosylhydrolase like 2	ADP-ribose glycohydrolase ARH3	95	2	1.18	3	5	12
U3KM96	P61224	RAP1B	Uncharacterized protein	Ras-related protein Rap-1b	100	3	1.18	11	7	73
G1SIP6		CISD1	ZnF_CDGSH domain-containing protein			1	1.18	2	2	20
G1U971		EIF3C	Eukaryotic translation initiation factor 3 subunit C			1	1.18	16	29	24
G1SQY8	Q9Y266	NUDC	CS domain-containing protein	Nuclear migration protein nudC	97	2	1.18	10	12	31
G1SM62	O43252	PAPSS1	Uncharacterized protein	Bifunctional 3~-phosphoadenosine 5~-phosphosulfate synthase 1	99	3	1.18	9	17	24
G1ST95	B3KS98	EIF3H	Eukaryotic translation initiation factor 3 subunit H	Eukaryotic translation initiation factor 3 subunit H	98	2	1.18	8	16	37
G1SCE7	P55084	HADHB	Uncharacterized protein	Trifunctional enzyme subunit beta, mitochondrial	94	3	1.18	14	16	42
G1TP30	H0Y4Q3	RANGAP1	RanGAP1_C domain-containing protein	Ran GTPase-activating protein 1 (Fragment)	80	2	1.18	7	19	20
	P37837	TALDO1		Transaldolase		4	1.18	3	6	11
G1SKF7	Q02878	RPL6	60S ribosomal protein L6	60S ribosomal protein L6	89	2	1.18	11	40	40
G1TS36	B8ZZG1	MPP6	Uncharacterized protein	Membrane protein, palmitoylated 6 (MAGUK p55 subfamily member 6), isoform CRA_a	96	3	1.18	5	4	10
	A0A0U1RRM4	PTBP1		Polypyrimidine tract-binding protein 1		4	1.17	10	60	29
G1T9R8	Q16222	UAP1	Uncharacterized protein	UDP-N-acetylhexosamine pyrophosphorylase	96	3	1.17	5	6	14
G1SIB1	P29083	GTF2E1	HTH TFE/IIEalpha-type domain-containing protein	General transcription factor IIE subunit 1	94	2	1.17	2	2	9
G1SJU1	O75051	PLXNA2	Sema domain-containing protein	Plexin-A2	98	2	1.17	3	3	2
G1SIJ2	P24752	ACAT1	Uncharacterized protein	Acetyl-CoA acetyltransferase, mitochondrial	92	3	1.17	13	21	47
G1T7I3	Q13085	ACACA	Uncharacterized protein	Acetyl-CoA carboxylase 1	98	3	1.17	12	13	7
G1U0T4		HSD17B14	Hydroxysteroid 17-beta dehydrogenase 14			1	1.17	2	3	9
G1TL06	P39023	RPL3	Uncharacterized protein	60S ribosomal protein L3	98	3	1.17	17	197	44
G1TBC0	A0A499FI31	SART3	Uncharacterized protein	Squamous cell carcinoma antigen recognized by T-cells 3	89	3	1.17	2	3	4
	P31153	MAT2A		S-adenosylmethionine synthase isoform type-2		4	1.17	2	2	9
Q9N0Z6	P05023	ATP1A1	Sodium/potassium-transporting ATPase subunit alpha-1	Sodium/potassium-transporting ATPase subunit alpha-1	98	2	1.17	28	72	36
G1SNM8	Q8N5M9	JAGN1	Uncharacterized protein	Protein jagunal homolog 1	96	3	1.17	3	4	17
G1SLA2	Q92604	LPGAT1	Lysophosphatidylglycerol acyltransferase 1	Acyl-CoA:lysophosphatidylglycerol acyltransferase 1	94	2	1.17	5	7	17
G1SDN3	K7EM18	EIF1	SUI1 domain-containing protein	Eukaryotic translation initiation factor 1	100	2	1.17	5	7	44
G1TBW7	P50148	GNAQ	Uncharacterized protein	Guanine nucleotide-binding protein G(q) subunit alpha	99	3	1.17	11	15	38

G1SYD3	P54577	YARS	Tyrosine--tRNA ligase	Tyrosine--tRNA ligase, cytoplasmic	96	2	1.17	20	36	44
G1SIE8	A0A3B3ISG5	IDE	Uncharacterized protein	Insulin-degrading enzyme	96	3	1.16	7	8	9
G1TDI0	Q9UQ80	PA2G4	Peptidase_M24 domain-containing protein	Proliferation-associated protein 2G4	98	2	1.16	12	71	43
G1SGL4	Q9Y276	BCS1L	Uncharacterized protein	Mitochondrial chaperone BCS1	96	3	1.16	6	7	26
G1SJG9	Q6P4Q7	CNNM4	Uncharacterized protein	Metal transporter CNNM4	94	3	1.16	3	3	5
G1T1T8	O96005	CLPTM1	CLPTM1, transmembrane protein	Cleft lip and palate transmembrane protein 1	97	2	1.16	11	26	21
G1U1Q1		THBS2	Thrombospondin 2			1	1.16	5	7	8
G1TCU4	H3BUU9	CDH11	Uncharacterized protein	Cadherin-11	99	3	1.16	3	8	9
	P02533	KRT14		Keratin, type I cytoskeletal 14		4	1.16	5	3	14
G1SS22		XRCC6	Ku domain-containing protein			1	1.16	2	2	5
G1SM24		MDN1	Midasin			1	1.16	2	2	1
G1T923	A0A1B0GTB0	ATP6AP2	Uncharacterized protein	Renin receptor (Fragment)	84	3	1.16	4	4	21
G1SPB2		RNMT	mRNA cap guanine-N7 methyltransferase			1	1.16	4	4	11
G1T193		UGT3A2	UDP-glucuronosyltransferase			1	1.16	2	2	6
G1T146	E7ESY4	MTA1	Metastasis associated 1	Metastasis-associated protein MTA1	88	2	1.16	4	4	9
G1T0W7	M0R1B0	EMC8	ER membrane protein complex subunit 8	ER membrane protein complex subunit 8 (Fragment)	98	2	1.16	3	3	42
G1SVV2	Q9NZM1	MYOF	Uncharacterized protein	Myoferlin	94	3	1.16	68	128	44
B7NZM4	Q9Y295	DRG1	Developmentally regulated GTP binding protein 1 (Predicted)	Developmentally-regulated GTP-binding protein 1	100	2	1.16	9	22	35
G1T2V6	Q99747	NAPG	Uncharacterized protein	Gamma-soluble NSF attachment protein	98	3	1.16	3	3	8
G1SJB4	P63244	RACK1	WD_REPEATS_REGION domain-containing protein	Receptor of activated protein C kinase 1	100	2	1.16	16	46	73
G1TUU9		TRIP12	Thyroid hormone receptor interactor 12			1	1.16	8	11	6
G1STH4	Q14344	GNA13	Uncharacterized protein	Guanine nucleotide-binding protein subunit alpha-13	86	3	1.15	3	5	11
G1STF8		TBRG4	RAP domain-containing protein			1	1.15	3	3	7
P20647	P16615	ATP2A2	Sarcoplasmic/endoplasmic reticulum calcium ATPase 2	Sarcoplasmic/endoplasmic reticulum calcium ATPase 2	98	2	1.15	29	5	36
G1SR63		PREB	WD_REPEATS_REGION domain-containing protein			1	1.15	3	7	15
G1T7I8		RIC8A	RIC8 guanine nucleotide exchange factor A			1	1.15	4	5	11
G1U0B5	Q9Y570	PPME1	Protein phosphatase methylesterase 1	Protein phosphatase methylesterase 1	96	2	1.15	3	4	16
G1TFL3	J3KQE5	RAN	GTP-binding nuclear protein Ran	GTP-binding nuclear protein Ran (Fragment)	96	2	1.15	9	23	36
G1U9U0	P50991	CCT4	T-complex protein 1 subunit delta	T-complex protein 1 subunit delta	99	2	1.15	23	58	56
G1SXJ6		CUL4A	CULLIN_2 domain-containing protein			1	1.15	10	8	17
G1TJX7	P50552	VASP	Vasodilator stimulated phosphoprotein	Vasodilator-stimulated phosphoprotein	87	2	1.15	2	5	6
G1STE3	A0A0A0MQX8	MBNL1	Uncharacterized protein	Muscleblind-like protein 1	100	3	1.15	3	4	7
G1T3H3	Q8IXI2	RHOT1	Mitochondrial Rho GTPase	Mitochondrial Rho GTPase 1	99	2	1.15	7	9	13
G1SQL1	Q9HAU5	UPF2	Uncharacterized protein	Regulator of nonsense transcripts 2	97	3	1.15	2	2	2
G1TCT3	F8VPD4	CAD	Carbamoyl-phosphate synthetase 2, aspartate transcarbamylase, and dihydroorotase	CAD protein	94	2	1.14	18	6	13
G1SGJ5	Q92600	CNOT9	Uncharacterized protein	CCR4-NOT transcription complex subunit 9	97	3	1.14	2	3	7
G1T2N8	O43592	XPOT	Exportin-T	Exportin-T	99	2	1.14	4	6	6
	Q03252	LMNB2		Lamin-B2		4	1.14	8	7	11
G1SHH1		PPOX	Protoporphyrinogen oxidase			1	1.14	2	2	8
G1U8J5		ATP5PF	ATP synthase-coupling factor 6, mitochondrial			1	1.14	3	3	33
G1U448	E9PEB5	FUBP1	Uncharacterized protein	Far upstream element-binding protein 1	93	3	1.14	10	6	16
G1TZ26		GUK1	Guanylate kinase 1			1	1.14	3	6	24
	Q9H0B6	KLC2		Kinesin light chain 2		4	1.14	5	2	12
G1U9S7	P17987	TCP1	Uncharacterized protein	T-complex protein 1 subunit alpha	97	3	1.14	26	389	62
G1SQV5	O15371	EIF3D	Eukaryotic translation initiation factor 3 subunit D	Eukaryotic translation initiation factor 3 subunit D	99	2	1.14	13	28	39
G1U7X2	Q15637	SF1	CCHC-type domain-containing protein	Splicing factor 1	99	2	1.14	4	7	12
G1THL2		FTL	Ferritin			1	1.14	2	2	17
G1T6W7	P04040	CAT	Catalase	Catalase	91	2	1.14	4	4	16
G1SCW9		CHPF2	Hexosyltransferase			1	1.14	4	5	10
G1SN37	O43172	PRPF4	WD_REPEATS_REGION domain-containing protein	U4/U6 small nuclear ribonucleoprotein Prp4	99	2	1.14	3	3	9
G1SJU2	O60306	AQR	RNA helicase aquarius	RNA helicase aquarius	96	2	1.14	3	3	4
G1T568	Q92973	TNPO1	Transportin 1	Transportin-1	93	2	1.14	14	12	21
G1T888	Q9P2R3	ANKFY1	Ankyrin repeat and FYVE domain containing 1	Rabankyrin-5	96	2	1.13	5	2	7
G1SZ03	P55884	EIF3B	Eukaryotic translation initiation factor 3 subunit B	Eukaryotic translation initiation factor 3 subunit B	98	2	1.13	24	9	41
G1TBR5	B4DR61	SEC61A1	Plug_translocon domain-containing protein	Protein transport protein Sec61 subunit alpha isoform 1	100	2	1.13	11	81	39
G1TMD8		PPP5C	Serine/threonine-protein phosphatase			1	1.13	3	3	10
G1SES2		NAA10	N-acetyltransferase domain-containing protein			1	1.13	2	2	9
G1T4D2		ACOX1	Acyl-coenzyme A oxidase			1	1.13	12	7	27
G1U222	A0A384DVK7	ARHGEF10L	Rho guanine nucleotide exchange factor 10 like	Rho guanine nucleotide exchange factor 10-like protein (Fragment)	93	2	1.13	2	2	4
G1SW89	Q9C0E8	LNPK	zinc_ribbon_10 domain-containing protein	Endoplasmic reticulum junction formation protein lunapark	89	2	1.13	2	2	5

G1T840	Q92598	HSPH1	Uncharacterized protein	Heat shock protein 105 kDa	96	3	1.13	17	23	28
G1TWK1	E9PGZ4	SACM1L	SAC domain-containing protein	Phosphatidylinositide phosphatase SAC1	98	2	1.13	14	26	34
G1TKQ8	A0A0D9SG77	UBE3A	Ubiquitin-protein ligase E3A	Ubiquitin-protein ligase E3A	97	2	1.13	6	7	11
G1U4R5	A0A0C4DGS1	DDOST	Dolichyl-diphosphooligosaccharide--protein glycosyltransferase 48 kDa subunit	Dolichyl-diphosphooligosaccharide--protein glycosyltransferase 48 kDa subunit	94	2	1.13	14	452	39
G1SPJ5	Q92616	GCN1	TOG domain-containing protein	eIF-2-alpha kinase activator GCN1	96	2	1.13	46	84	27
G1SPN1		NUDCD2	CS domain-containing protein			1	1.12	2	3	17
G1T7Q3	F5H157	RAB35	Uncharacterized protein	Ras-related protein Rab-35 (Fragment)	91	3	1.12	6	20	40
G1TA69	Q8TBA6	GOLGA5	Uncharacterized protein	Golgin subfamily A member 5	83	3	1.12	4	3	6
G1T2V2	D6RF62	PAICS	AIRC domain-containing protein	Multifunctional protein ADE2	93	2	1.12	8	9	32
G1SM82	E9PGC0	RASA1	Uncharacterized protein	Ras GTPase-activating protein 1	96	3	1.12	8	10	13
G1SQ93	Q9UKV8	AGO2	Protein argonaute-2	Protein argonaute-2	98	2	1.12	3	3	6
G1SFC1	H0Y6I0	GOLGA4	Golgin A4	Golgin subfamily A member 4 (Fragment)	77	2	1.12	12	17	7
G1T0E5		SNAP23	Synaptosomal-associated protein			1	1.12	3	5	23
G1SGV5	Q8NE71	ABCF1	Uncharacterized protein	ATP-binding cassette sub-family F member 1	92	3	1.12	11	13	27
P62139	P62136	PPP1CA	Serine/threonine-protein phosphatase PP1-alpha catalytic subunit	Serine/threonine-protein phosphatase PP1-alpha catalytic subunit	100	2	1.12	11	2	51
G1TWP4	A0A140T936	VARS	GST C-terminal domain-containing protein	Valine--tRNA ligase (Fragment)	92	2	1.12	18	32	18
G1T336	A0A0D9SF53	DDX3X	Uncharacterized protein	ATP-dependent RNA helicase DDX3X	99	3	1.12	19	44	31
U3KN22	Q9Y2Q3	GSTK1	Glutathione S-transferase kappa	Glutathione S-transferase kappa 1	79	2	1.12	9	18	47
G1SXK8	F8WE74	SLC25A17	Uncharacterized protein	Peroxisomal membrane protein PMP34	96	3	1.12	4	6	21
G1TWU8	O43264	ZW10	Uncharacterized protein	Centromere/kinetochore protein zw10 homolog	89	3	1.12	10	15	20
G1TRL5	A0A087WUT6	EIF5B	Tr-type G domain-containing protein	Eukaryotic translation initiation factor 5B	95	2	1.12	21	45	22
G1SJE4	Q92556	ELMO1	ELMO domain-containing protein	Engulfment and cell motility protein 1	97	2	1.12	6	9	13
G1SER3	Q02978	SLC25A11	Uncharacterized protein	Mitochondrial 2-oxoglutarate/malate carrier protein	97	3	1.12	8	58	37
G1T643	A0A1W2PNX8	UNC45A	Unc-45 myosin chaperone A	Protein unc-45 homolog A	94	2	1.12	8	16	14
G1T3K1		DESI2	DUF862 domain-containing protein			1	1.12	2	3	29
G1TTN7	O00139	KIF2A	Kinesin-like protein	Kinesin-like protein KIF2A	94	2	1.12	4	3	6
G1SFH6	Q9UBT2	UBA2	Uncharacterized protein	SUMO-activating enzyme subunit 2	97	3	1.12	10	10	27
G1SPG2		TRIP11	GRIP domain-containing protein			1	1.11	16	20	11
G1TMQ8	H0Y9V7	ATP2C1	Calcium-transporting ATPase	Calcium-transporting ATPase type 2C member 1 (Fragment)	99	2	1.11	4	6	9
G1SJ37	F8W9S7	GAPVD1	Uncharacterized protein	GTPase-activating protein and VPS9 domain-containing protein 1	95	3	1.11	3	4	4
G1SWN7	Q4G0N4	NADK2	NAD kinase 2, mitochondrial	NAD kinase 2, mitochondrial	92	2	1.11	5	5	14
G1U3Q0	Q93050	ATP6V0A1	V-type proton ATPase subunit a	V-type proton ATPase 116 kDa subunit a isoform 1	96	2	1.11	12	3	20
G1U115	Q15008	PSMD6	PCI domain-containing protein	26S proteasome non-ATPase regulatory subunit 6	99	2	1.11	16	38	47
G1T5X6		HMGCL	Pyruvate carboxyltransferase domain-containing protein			1	1.11	2	5	11
G1SIH3	A0A2R8Y5A6	ATXN2	Uncharacterized protein	Ataxin-2	94	3	1.11	5	9	5
G1SRI8	P19367	HK1	Uncharacterized protein	Hexokinase-1	96	3	1.11	27	50	33
G1SSX5	P56192	MARS	Uncharacterized protein	Methionine--tRNA ligase, cytoplasmic	93	3	1.11	14	28	21
G1SJK0		APOOL	MICOS complex subunit			1	1.11	3	9	23
G1T108	Q9NRG9	AAAS	WD_REPEATS_REGION domain-containing protein	Aladin	94	2	1.11	6	8	17
G1SZW5	Q8TD16	BICD2	Uncharacterized protein	Protein bicaudal D homolog 2	95	3	1.11	4	6	7
	B7WP74	CWC22		Pre-mRNA-splicing factor CWC22 homolog (Fragment)		4	1.11	3	3	7
G1T2G4	P05198	EIF2S1	Eukaryotic translation initiation factor 2 subunit 1	Eukaryotic translation initiation factor 2 subunit 1	99	2	1.11	14	37	53
G1SKK0	P22102	GART	Trifunctional purine biosynthetic protein adenosine-3	Trifunctional purine biosynthetic protein adenosine-3	89	2	1.11	6	10	12
G1SIJ8	Q8TEX9	IPO4	Importin N-terminal domain-containing protein	Importin-4	89	2	1.11	6	8	10
G1TN86	C9IZG4	CUTA	Uncharacterized protein	Protein CutA	93	3	1.10	2	3	18
G1TLE4	P62879	GNB2	WD_REPEATS_REGION domain-containing protein	Guanine nucleotide-binding protein G(I)/G(S)/G(T) subunit beta-2	100	2	1.10	11	6	43
P43236	P43235	CTSK	Cathepsin K	Cathepsin K	94	2	1.10	5	13	22
G1SHS4	A0A0C4DGX4	CUL1	CULLIN_2 domain-containing protein	Cullin-1	97	2	1.10	5	10	11
G1TM48	Q5JRA6	MIA3	SH3 domain-containing protein	Transport and Golgi organization protein 1 homolog	71	2	1.10	5	5	3
G1TEB0	Q9UKX5	ITGA11	VWFA domain-containing protein	Integrin alpha-11	91	2	1.10	20	39	24
	Q96PU8	QKI		Protein quaking		4	1.10	4	7	16
G1T159	P62070	RRAS2	Uncharacterized protein	Ras-related protein R-Ras2	97	3	1.10	6	6	33
G1SIS5	O15498	YKT6	Uncharacterized protein	Synaptobrevin homolog YKT6	96	3	1.10	5	6	36
G1SM15	A0A087WY71	AP2M1	MHD domain-containing protein	AP-2 complex subunit mu	100	2	1.10	15	26	42
G1T3S4		MECR	PKS_ER domain-containing protein			1	1.10	5	9	34
G1T810	A0A0A0MTN0	CUL2	CULLIN_2 domain-containing protein	Cullin-2	99	2	1.10	5	4	10
G1T866	Q96CS3	FAF2	UBX domain-containing protein	FAS-associated factor 2	99	2	1.10	7	20	24
G1SZ66	I3L295	MPDU1	Uncharacterized protein	Mannose-P-dolichol utilization defect 1 isoform 2	89	3	1.10	3	6	15
	H0YEN5	RPS2		40S ribosomal protein S2 (Fragment)		4	1.10	10	6	54
G1SYY0	Q96SK2	TMEM209	Uncharacterized protein	Transmembrane protein 209	96	3	1.10	3	5	12

G1T720	F5GXX5	DAD1	Dolichyl-diphosphooligosaccharide--protein glycosyltransferase subunit DAD1	Dolichyl-diphosphooligosaccharide--protein glycosyltransferase subunit DAD1	75	2	1.10	3	5	38
G1U3X5	P46821	MAP1B	Uncharacterized protein	Microtubule-associated protein 1B	88	3	1.10	15	8	10
	P62166	NCS1		Neuronal calcium sensor 1		4	1.10	2	2	24
G1TEI1	P20645	M6PR	Uncharacterized protein	Cation-dependent mannose-6-phosphate receptor	95	3	1.10	2	3	8
P62493	P62491	RAB11A	Ras-related protein Rab-11A	Ras-related protein Rab-11A	100	2	1.10	11	40	59
Q9TT13	Q9Y277	VDAC3	Voltage-dependent anion-selective channel protein 3	Voltage-dependent anion-selective channel protein 3	98	2	1.10	12	31	54
G1ST38	Q9Y673	ALG5	ALG5, dolichyl-phosphate beta-glucosyltransferase	Dolichyl-phosphate beta-glucosyltransferase	93	2	1.10	6	9	21
G1SZ59	P60842	EIF4A1	Eukaryotic initiation factor 4A-I	Eukaryotic initiation factor 4A-I	100	2	1.10	23	208	76
G1SMC8	Q14997	PSME4	Uncharacterized protein	Proteasome activator complex subunit 4	98	3	1.10	5	5	5
G1SNN0		CSGALNACT2	Hexosyltransferase			1	1.10	2	2	7
G1SK33	P05556	ITGB1	Integrin beta	Integrin beta-1	94	2	1.10	17	48	26
G1TIA2		RIOX1	JmjC domain-containing protein			1	1.10	3	4	10
G1T103	F8VQQ3	C12orf10	Uncharacterized protein	UPF0160 protein MYG1, mitochondrial	77	3	1.09	4	7	16
G1SP36	Q9HAV4	XPO5	Importin N-terminal domain-containing protein	Exportin-5	95	2	1.09	3	6	7
G1SUP8	P67812	SEC11A	Signal peptidase complex catalytic subunit SEC11	Signal peptidase complex catalytic subunit SEC11A	100	2	1.09	6	9	40
G1SR03	P55072	VCP	Uncharacterized protein	Transitional endoplasmic reticulum ATPase	100	3	1.09	39	189	69
G1SYS5	A0A1B0GW77	ALDH7A1	Aldedh domain-containing protein	Alpha-aminoadipic semialdehyde dehydrogenase	93	2	1.09	9	30	30
G1SMZ8	Q96JJ7	TMX3	Thioredoxin domain-containing protein	Protein disulfide-isomerase TMX3	92	2	1.09	8	9	28
	A0A0A0MRA3	TTN		Titin		4	1.09	2	2	0
G1T0U8	Q9UHG3	PCYOX1	Prenylcys_lyase domain-containing protein	Prenylcysteine oxidase 1	86	2	1.09	10	28	31
G1SFK3		RPA1	Replication protein A subunit			1	1.09	2	2	7
G1SCN8	P49368	CCT3	T-complex protein 1 subunit gamma	T-complex protein 1 subunit gamma	98	2	1.09	27	54	64
G1SIW8	Q08257	CRYZ	PKS_ER domain-containing protein	Quinone oxidoreductase	87	2	1.09	7	25	39
G1TEK3	Q8IWJ2	GCC2	GRIP domain-containing protein	GRIP and coiled-coil domain-containing protein 2	86	2	1.09	9	16	8
G1SQU5	Q9UP95	SLC12A4	Solute carrier family 12 member 4	Solute carrier family 12 member 4	97	2	1.09	13	23	16
G1TYV6	Q9HD20	ATP13A1	Cation-transporting ATPase	Manganese-transporting ATPase 13A1	95	2	1.09	12	14	15
G1SHK7	A0A0C4DGV4	LAMTOR5	Uncharacterized protein	Hepatitis B virus x interacting protein	100	3	1.09	3	5	31
G1TGH1		D2HGDH	D-2-hydroxyglutarate dehydrogenase			1	1.09	4	7	13
G1T9J3	A2RRP1	NBAS	Uncharacterized protein	Neuroblastoma-amplified sequence	90	3	1.09	19	28	12
G1TZC9	A0A087WXU3	ESYT2	Extended synaptotagmin 2	Extended synaptotagmin-2	92	2	1.09	13	7	25
G1TH06	F6WQW2	RANBP1	RAN binding protein 1	Ran-specific GTPase-activating protein	91	2	1.09	4	7	28
G1TAN9	P21399	ACO1	Cytoplasmic aconitate hydratase	Cytoplasmic aconitate hydratase	93	2	1.08	5	5	9
	H0Y5K5	ERGIC3		Endoplasmic reticulum-Golgi intermediate compartment protein 3 (Fragment)		4	1.08	2	2	5
G1T157	Q9Y376	CAB39	Uncharacterized protein	Calcium-binding protein 39	99	3	1.08	3	2	8
G1TUY5		OCIAD1	OCIA domain-containing protein			1	1.08	3	5	15
G1TIM0	G3V155	TMX2	Thioredoxin domain-containing protein	Thioredoxin domain containing 14, isoform CRA_a	94	2	1.08	4	4	13
G1SN95	A0A087WSV8	NUCB2	Nucleobindin 2	Nucleobindin 2, isoform CRA_b	93	2	1.08	18	35	47
G1TNH0	O14939	PLD2	Phospholipase D2	Phospholipase D2	89	2	1.08	3	2	7
Q01971	P61019	RAB2A	Ras-related protein Rab-2A	Ras-related protein Rab-2A	100	2	1.08	12	46	67
B7NZM8	Q04917	YWHAH	Tyrosine 3-monooxygenase/tryptophan 5-monooxygenase activation protein, eta polypeptide (Predicted)	14-3-3 protein eta	99	2	1.08	14	38	65
G1TGF1	Q15185	PTGES3	Prostaglandin E synthase 3	Prostaglandin E synthase 3	100	2	1.08	5	8	52
G1SP51	P62277	RPS13	Ribosomal_S13_N domain-containing protein	40S ribosomal protein S13	100	2	1.08	8	16	46
G1U535	Q9HBH5	RDH14	Uncharacterized protein	Retinol dehydrogenase 14	91	3	1.08	4	3	13
G1SNP4	A0A3B3ITZ9	THRAP3	Uncharacterized protein	Thyroid hormone receptor-associated protein 3	95	3	1.08	6	8	8
G1SSV4	A0A087WT80	PLCB1	1-phosphatidylinositol 4,5-bisphosphate phosphodiesterase	1-phosphatidylinositol 4,5-bisphosphate phosphodiesterase	97	2	1.08	3	4	5
G1TQR9		ZSWIM8	SWIM-type domain-containing protein			1	1.08	2	3	2
G1SEQ2		PBDC1	Polysacc_synt_4 domain-containing protein			1	1.08	3	3	16
P63169	F8VRV5	DYNLL1	Dynein light chain 1, cytoplasmic	Dynein light chain	100	2	1.08	2	13	43
G1SLS8	Q9NX62	IMPAD1	Uncharacterized protein	Inositol monophosphatase 3	96	3	1.08	6	8	21
G1TEM4	Q9UEW8	STK39	Protein kinase domain-containing protein	STE20/SPS1-related proline-alanine-rich protein kinase	95	2	1.08	2	2	4
G1SPN3	Q14573	ITPR3	Inositol 1,4,5-trisphosphate receptor type 3	Inositol 1,4,5-trisphosphate receptor type 3	95	2	1.08	8	10	6
G1TKG2		ISOC2	Isochorismatase domain containing 2			1	1.08	4	5	43
G1SFC4		LMF2	Lipase maturation factor			1	1.08	3	8	7
	A0A0A0MTU3	TMEM259		Membralin		4	1.08	2	2	8
G1SFE9	H0YJG7	AHSA1	Aha1_N domain-containing protein	Activator of 90 kDa heat shock protein ATPase homolog 1 (Fragment)	95	2	1.07	3	5	14
G1SZ37	P31937	HIBADH	3-hydroxyisobutyrate dehydrogenase	3-hydroxyisobutyrate dehydrogenase, mitochondrial	96	2	1.07	6	11	32
G1SV05	P34932	HSPA4	Uncharacterized protein	Heat shock 70 kDa protein 4	97	3	1.07	25	23	45
G1SYC9	H0YNE9	RAB8B	Uncharacterized protein	Ras-related protein Rab-8B (Fragment)	99	3	1.07	5	5	19
G1T0H3	P40763	STAT3	Signal transducer and activator of transcription	Signal transducer and activator of transcription 3	100	2	1.07	8	16	17
G1SUN1	O75915	ARL6IP5	PRA1 family protein	PRA1 family protein 3	96	2	1.07	5	24	24

G1TDB3	P62851	RPS25	Uncharacterized protein	40S ribosomal protein S25	100	3	1.07	3	5	16
G1SLJ9	Q9P0I2	EMC3	ER membrane protein complex subunit 3	ER membrane protein complex subunit 3	99	2	1.07	3	4	20
G1SSX2	Q7Z6Z7	HUWE1	HECT, UBA and WWE domain containing 1, E3 ubiquitin protein ligase	E3 ubiquitin-protein ligase HUWE1	96	2	1.07	19	21	8
G1SJX1	Q16537	PPP2R5E	Serine/threonine-protein phosphatase 2A 56 kDa regulatory subunit	Serine/threonine-protein phosphatase 2A 56 kDa regulatory subunit epsilon isoform	100	2	1.07	4	7	13
G1T5C5	E5RHW4	ERLIN2	PHB domain-containing protein	Erlin-2 (Fragment)	98	2	1.07	8	6	36
G1SJ43	Q9H3S7	PTPN23	Uncharacterized protein	Tyrosine-protein phosphatase non-receptor type 23	91	3	1.07	7	7	5
G1TE76	Q15056	EIF4H	Eukaryotic translation initiation factor 4H	Eukaryotic translation initiation factor 4H	92	2	1.07	3	10	23
G1T3L5	O43719	HTATSF1	Uncharacterized protein	HIV Tat-specific factor 1	82	3	1.07	3	3	6
G1SSN9	Q96S59	RANBP9	Uncharacterized protein	Ran-binding protein 9	97	3	1.07	4	6	15
G1SPT2	P26196	DDX6	Uncharacterized protein	Probable ATP-dependent RNA helicase DDX6	99	3	1.07	13	22	39
G1SFF5	O15270	SPTLC2	Aminotran_1_2 domain-containing protein	Serine palmitoyltransferase 2	98	2	1.07	3	5	8
G1SZH8	Q8N1B4	VPS52	Uncharacterized protein	Vacuolar protein sorting-associated protein 52 homolog	99	3	1.07	4	3	12
G1T9W3	K4DI93	CUL4B	CULLIN_2 domain-containing protein	Cullin 4B, isoform CRA_e	100	2	1.07	10	7	14
G1SG42	Q52LJ0	FAM98B	Uncharacterized protein	Protein FAM98B	94	3	1.07	9	6	34
G1T168	P46783	RPS10	S10_plectin domain-containing protein	40S ribosomal protein S10	100	2	1.07	10	17	40
G1SQM7	P53999	SUB1	PC4 domain-containing protein	Activated RNA polymerase II transcriptional coactivator p15	97	2	1.07	4	7	22
G1SI98	O60502	OGA	Uncharacterized protein	Protein O-GlcNAcase	99	3	1.07	3	3	3
	Q9NYU2	UGGT1		UDP-glucose:glycoprotein glucosyltransferase 1		4	1.07	19	5	19
G1T0L9	P04843	RPN1	Dolichyl-diphosphooligosaccharide--protein glycosyltransferase subunit 1	Dolichyl-diphosphooligosaccharide--protein glycosyltransferase subunit 1	97	2	1.07	28	238	53
G1U0Q2	K7EJ78	RPS15	Uncharacterized protein	40S ribosomal protein S15	99	3	1.07	2	16	28
G1T284	Q8TCJ2	STT3B	Uncharacterized protein	Dolichyl-diphosphooligosaccharide--protein glycosyltransferase subunit STT3B	100	3	1.07	5	2	9
G1TUD2	Q9UBI6	GNG12	Guanine nucleotide-binding protein subunit gamma	Guanine nucleotide-binding protein G(I)/G(S)/G(O) subunit gamma-12	100	2	1.06	4	5	67
G1T573	H0Y3P2	EIF4G2	Eukaryotic translation initiation factor 4 gamma 2	Eukaryotic translation initiation factor 4 gamma 2	95	2	1.06	18	32	26
	M0QXF7	MYDGF		Myeloid-derived growth factor (Fragment)		4	1.06	2	2	19
	Q5T8U5	SURF4		Surfeit 4		4	1.06	4	87	29
G1SNI4	O94760	DDAH1	Uncharacterized protein	N(G),N(G)-dimethylarginine dimethylaminohydrolase 1	96	3	1.06	6	6	24
G1T3Q2	Q96HY6	DDRGK1	Uncharacterized protein	DDRGK domain-containing protein 1	90	3	1.06	6	17	25
G1U8V2	Q96DZ1	ERLEC1	Uncharacterized protein	Endoplasmic reticulum lectin 1	98	3	1.06	3	7	8
G1TBL1	C9JPE1	SLC25A20	Uncharacterized protein	Mitochondrial carnitine/acylcarnitine carrier protein	92	3	1.06	5	13	22
G1T358	Q96AG3	SLC25A46	Uncharacterized protein	Solute carrier family 25 member 46	94	3	1.06	3	5	18
G1SVM1	Q14764	MVP	Uncharacterized protein	Major vault protein	91	3	1.06	35	109	57
G1T534	E9PDM8	SEC24D	SEC24 homolog D, COPII coat complex component	Protein transport protein Sec24D	92	2	1.06	8	2	8
G1SCX4	Q15052	ARHGEF6	Uncharacterized protein	Rho guanine nucleotide exchange factor 6	94	3	1.06	2	4	4
G1SES9	P31939	ATIC	MGS domain-containing protein	Bifunctional purine biosynthesis protein PURH	94	2	1.06	17	35	44
B7NZS0		MYADM	Myeloid-associated differentiation marker (Predicted)			1	1.06	2	9	10
G1T501		CCAR2	Cell cycle and apoptosis regulator 2			1	1.06	2	4	6
G1SXI8		PCID2	PCI domain containing 2			1	1.06	2	2	6
G1SL46		PSMD9	PDZ domain-containing protein			1	1.06	3	3	15
G1SQ57	Q8N6T3	ARFGAP1	Arf-GAP domain-containing protein	ADP-ribosylation factor GTPase-activating protein 1	78	2	1.06	5	4	21
G1T3H5		EIF2B3	NTP_transferase domain-containing protein			1	1.06	4	3	12
G1T4K8	P32189	GK	Uncharacterized protein	Glycerol kinase	97	3	1.06	11	20	23
G1SVT4	P51665	PSMD7	MPN domain-containing protein	26S proteasome non-ATPase regulatory subunit 7	99	2	1.06	9	20	38
G1SI54		ILVBL	IlvB acetolactate synthase like			1	1.06	5	8	19
G1SWR1	O43818	RRP9	WD_REPEATS_REGION domain-containing protein	U3 small nucleolar RNA-interacting protein 2	94	2	1.06	5	6	13
G1T3M3	Q9UL25	RAB21	Uncharacterized protein	Ras-related protein Rab-21	97	3	1.06	5	12	30
G1T297	Q9UBV2	SEL1L	Fibronectin type-II domain-containing protein	Protein sel-1 homolog 1	97	2	1.06	15	26	30
B6V9S9	P78371	CCT2	Chaperonin-containing T-complex polypeptide beta subunit	T-complex protein 1 subunit beta	99	2	1.05	27	165	68
G1SZD2	P53701	HCCS	Cytochrome c heme lyase	Cytochrome c-type heme lyase	83	2	1.05	4	7	17
G1SF78	Q96RL7	VPS13A	Vacuolar protein sorting 13 homolog A	Vacuolar protein sorting-associated protein 13A	89	2	1.05	4	5	3
G1T9F3	Q14974	KPNB1	Importin N-terminal domain-containing protein	Importin subunit beta-1	99	2	1.05	28	100	50
G1SQT0	P60510	PPP4C	Serine/threonine-protein phosphatase	Serine/threonine-protein phosphatase 4 catalytic subunit	100	2	1.05	3	3	14
G1SRZ8	A5YKK6	CNOT1	Uncharacterized protein	CCR4-NOT transcription complex subunit 1	100	3	1.05	8	8	5
P0CL18		EIF2D	Eukaryotic translation initiation factor 2D			1	1.05	2	3	8
G1SDA8	P25786	PSMA1	Proteasome endopeptidase complex	Proteasome subunit alpha type-1	100	2	1.05	10	24	49
U3KNL7	Q06210	GFPT1	Uncharacterized protein	Glutamine--fructose-6-phosphate aminotransferase [isomerizing] 1	95	3	1.05	15	4	40
G1TPM1	Q96SB3	PPP1R9B	PDZ domain-containing protein	Neurabin-2	99	2	1.05	6	9	15
G1STD4	Q9BSR8	YIPF4	Protein YIPF	Protein YIPF4	99	2	1.05	2	2	9
G1SZI0		SPG7	SPG7, paraplegin matrix AAA peptidase subunit			1	1.05	3	3	10
G1T550	P51148	RAB5C	Uncharacterized protein	Ras-related protein Rab-5C	91	3	1.05	8	30	53
G1SS70	P61247	RPS3A	40S ribosomal protein S3a	40S ribosomal protein S3a	100	2	1.05	15	91	55

G1T7J9	B1AV70	YIPF6	Protein YIPF	Protein YIPF (Fragment)	75	2	1.05	4	7	12
G1SFG8	A0A096LNH6	DOCK1	Uncharacterized protein	Dedicator of cytokinesis protein 1	95	3	1.05	4	6	4
G1T7R4	P18084	ITGB5	Integrin beta	Integrin beta-5	93	2	1.05	5	6	8
G1SS79	P29966	MARCKS	Uncharacterized protein	Myristoylated alanine-rich C-kinase substrate	87	3	1.05	4	11	25
P19943	P05387	RPLP2	60S acidic ribosomal protein P2 (Fragment)	60S acidic ribosomal protein P2	100	2	1.05	8	25	64
	P29992	GNA11		Guanine nucleotide-binding protein subunit alpha-11		4	1.04	6	2	22
G1TFB5	Q9NP72	RAB18	Uncharacterized protein	Ras-related protein Rab-18	98	3	1.04	8	17	49
G1SUL3		TMF1	TMF_TATA_bd domain-containing protein			1	1.04	2	2	3
B7NZS4	O75175	CNOT3	CCR4-NOT transcription complex, subunit 3 (Predicted)	CCR4-NOT transcription complex subunit 3	97	2	1.04	3	5	9
G1TLQ8	R4GNH3	PSMC3	AAA domain-containing protein	26S proteasome regulatory subunit 6A	100	2	1.04	21	51	65
G1SXS3	Q13573	SNW1	SKIP_SNW domain-containing protein	SNW domain-containing protein 1	100	2	1.04	2	2	8
G1U4Y5		FCGRT	Fc fragment of IgG receptor and transporter			1	1.04	2	2	7
G1SEV7	Q9NX55	HYPK	Uncharacterized protein	Huntingtin-interacting protein K	98	3	1.04	4	7	42
G1SVV6	Q02750	MAP2K1	Dual-specificity mitogen-activated protein kinase kinase 1	Dual specificity mitogen-activated protein kinase kinase 1	94	2	1.04	5	10	21
G1TBX9	Q9H8M7	MINDY3	DUF4205 domain-containing protein	Ubiquitin carboxyl-terminal hydrolase MINDY-3	99	2	1.04	3	3	13
	Q9NUY8	TBC1D23		TBC1 domain family member 23		4	1.04	3	5	6
G1U3G0	Q9C0C9	UBE2O	Ubiquitin conjugating enzyme E2 O	(E3-independent) E2 ubiquitin-conjugating enzyme	96	2	1.04	3	3	4
G1SHF3		NIT1	CN hydrolase domain-containing protein			1	1.04	3	3	14
G1TEN9	Q8TB61	SLC35B2	Uncharacterized protein	Adenosine 3~-phospho 5~-phosphosulfate transporter 1	89	3	1.04	4	7	9
G1TPC8	P08962	CD63	Tetraspanin	CD63 antigen	78	2	1.04	4	9	15
G1SYC3		FBXL8	F-box domain-containing protein			1	1.04	2	3	10
G1SJ20	A0A1W2PNV3	GOSR2	Uncharacterized protein	Golgi SNAP receptor complex member 2 (Fragment)	92	3	1.04	5	11	37
G1THP8	P46734	MAP2K3	Protein kinase domain-containing protein	Dual specificity mitogen-activated protein kinase kinase 3	97	2	1.04	6	10	29
G1SZZ1	Q9H0V1	TMEM168	Transmembrane protein 168	Transmembrane protein 168	96	2	1.04	5	5	13
	I3L0A0	TMEM189-UBE2V1		HCG2044781		4	1.04	6	2	23
G1SWI3	P45880	VDAC2	Voltage-dependent anion-selective channel protein 2	Voltage-dependent anion-selective channel protein 2	99	2	1.04	13	66	63
G1TCY7		EIF2B4	Translation initiation factor eIF-2B subunit delta			1	1.04	3	3	8
G1TWD8		SELENOO	Selenoprotein O			1	1.04	3	4	18
G1SKJ8		TCOF1	LisH domain-containing protein			1	1.04	2	2	3
G1SLE1		HARS2	AA_TRNA_LIGASE_II domain-containing protein			1	1.04	4	3	10
G1T6G1		MMAB	Corrinoid adenosyltransferase			1	1.04	3	4	19
G1SFV1	A0A499FI48	PDIA4	Protein disulfide-isomerase	Protein disulfide-isomerase	91	2	1.04	29	398	49
G1TD24	Q9NRY4	ARHGAP35	Rho GTPase activating protein 35	Rho GTPase-activating protein 35	98	2	1.04	4	3	3
G1T2R3		EFL1	Tr-type G domain-containing protein			1	1.04	3	2	6
G1ST64		LMOD1	Leiomodin 1			1	1.04	5	5	9
G1SDK8	Q13564	NAE1	NEDD8-activating enzyme E1 regulatory subunit	NEDD8-activating enzyme E1 regulatory subunit	96	2	1.04	3	3	11
	A0A087WX29	TARDBP		TAR DNA-binding protein 43 (Fragment)		4	1.04	7	5	47
G1T242		IKBIP	IKBKB interacting protein			1	1.04	15	24	39
G1TVG8	O15173	PGRMC2	Cytochrome b5 heme-binding domain-containing protein	Membrane-associated progesterone receptor component 2	97	2	1.04	8	10	33
G1SP83	Q9Y2D0	CA5B	Carbonic anhydrase 5B	Carbonic anhydrase 5B, mitochondrial	93	2	1.03	10	35	46
G1SSL2	F8VXJ7	CNPY2	Saposin B-type domain-containing protein	Protein canopy homolog 2 (Fragment)	99	2	1.03	7	26	56
G1SPV2	J3QRU1	YES1	Tyrosine-protein kinase	Tyrosine-protein kinase	97	2	1.03	5	3	13
G1TNU3		STX16	t-SNARE coiled-coil homology domain-containing protein			1	1.03	5	5	23
G1TX74	Q6IBS0	TWF2	Uncharacterized protein	Twinfilin-2	95	3	1.03	4	5	17
G1SLD5	A0A087X054	HYOU1	Hypoxia up-regulated 1	Hypoxia up-regulated protein 1	87	2	1.03	29	51	38
G1TDN4	P10644	PRKAR1A	Uncharacterized protein	cAMP-dependent protein kinase type I-alpha regulatory subunit	99	3	1.03	4	8	17
G1U0M5	H7C1W1	PXDN	Peroxidasin	Peroxidasin homolog (Fragment)	93	2	1.03	5	3	5
G1SHZ8	P50990	CCT8	Uncharacterized protein	T-complex protein 1 subunit theta	97	3	1.03	33	75	71
G1SQ90	A0A2R8Y5H3	COL4A3BP	Collagen type IV alpha 3 binding protein	Collagen type IV alpha-3-binding protein (Fragment)	96	2	1.03	2	2	6
G1TED6	P08758	ANXA5	Annexin	Annexin A5	93	2	1.03	19	55	65
G1T7J5		NCSTN	Ncstrn_small domain-containing protein			1	1.03	7	18	16
G1TC19		CPT2	Carnitine palmitoyltransferase 2			1	1.03	2	2	5
P29694	P26641	EEF1G	Elongation factor 1-gamma	Elongation factor 1-gamma	98	2	1.03	21	81	64
G1STW7	Q9NSD9	FARSB	B5 domain-containing protein	Phenylalanine--tRNA ligase beta subunit	95	2	1.03	11	17	22
G1SEF8	M0R0Y2	NAPA	Uncharacterized protein	Alpha-soluble NSF attachment protein	84	3	1.03	10	18	49
	A6NG10	WBP2		WW domain-binding protein 2		4	1.03	2	2	7
G1SN52	E7ESC6	XPO7	Exportin 7	Exportin-7	99	2	1.03	5	9	7
G1SS85	Q9UBQ7	GRHPR	Uncharacterized protein	Glyoxylate reductase/hydroxypyruvate reductase	90	3	1.03	5	9	28
G1SYE7	H0Y8R1	GRSF1	Uncharacterized protein	G-rich sequence factor 1 (Fragment)	96	3	1.03	3	5	14
G1SH30	Q8WVY7	UBLCP1	Uncharacterized protein	Ubiquitin-like domain-containing CTD phosphatase 1	100	3	1.03	2	2	10

G1TBX7	P11233	RALA	Uncharacterized protein	Ras-related protein Ral-A	100	3	1.03	8	6	50
G1SG41		TBL2	WD_REPEATS_REGION domain-containing protein			1	1.03	5	6	11
G1T4J2	F5H228	TRIO	Uncharacterized protein	Triple functional domain protein	99	3	1.03	4	5	4
G1SZP4	I6L894	ANK2	Ankyrin 2	Ankyrin-2	88	2	1.02	3	3	1
G1TAP1	Q15181	PPA1	Uncharacterized protein	Inorganic pyrophosphatase	96	3	1.02	8	13	48
G1TP15	O43242	PSMD3	PCI domain-containing protein	26S proteasome non-ATPase regulatory subunit 3	98	2	1.02	20	36	43
G1TMS5	P48643	CCT5	Uncharacterized protein	T-complex protein 1 subunit epsilon	99	3	1.02	31	91	70
G1TCM0	J3KQ34	COPS7B	PCI domain-containing protein	COP9 signalosome complex subunit 7b	97	2	1.02	3	8	19
G1SRQ2	A0A087WTB8	UCHL3	Ubiquitin carboxyl-terminal hydrolase	Ubiquitin carboxyl-terminal hydrolase	98	2	1.02	3	5	21
	Q9H0U4	RAB1B		Ras-related protein Rab-1B		4	1.02	12	13	69
G1U7M0	Q96AJ9	VTI1A	t-SNARE coiled-coil homology domain-containing protein	Vesicle transport through interaction with t-SNAREs homolog 1A	94	2	1.02	3	5	17
G1T275	Q8WU90	ZC3H15	Uncharacterized protein	Zinc finger CCCH domain-containing protein 15	98	3	1.02	3	3	9
G1SG72	P61221	ABCE1	Uncharacterized protein	ATP-binding cassette sub-family E member 1	100	3	1.02	14	23	32
G1SG68	O00629	KPNA4	Importin subunit alpha	Importin subunit alpha-3	99	2	1.02	7	18	24
G1TVW1	O60568	PLOD3	Procollagen-lysine,2-oxoglutarate 5-dioxygenase 3	Multifunctional procollagen lysine hydroxylase and glycosyltransferase LH3	94	2	1.02	13	11	31
G1U5Z2		TK2	dNK domain-containing protein			1	1.02	2	2	11
	Q8NHH9-2	ATL2		Isoform 2 of Atlastin-2		4	1.02	4	8	11
G1U3F3		NEXN	Ig-like domain-containing protein			1	1.02	22	32	36
G1SWD9	B4DKY1	CARS	Cysteinyl-tRNA synthetase	Cysteine--tRNA ligase, cytoplasmic	68	2	1.02	12	23	20
	Q8NBJ5	COLGALT1		Procollagen galactosyltransferase 1		4	1.02	7	34	14
G1T2C3	O75534	CSDE1	Uncharacterized protein	Cold shock domain-containing protein E1	99	3	1.02	15	23	21
G1SFS8	Q7KZF4	SND1	Staphylococcal nuclease domain-containing protein	Staphylococcal nuclease domain-containing protein 1	97	2	1.02	35	83	54
G1SYL8	I3L0M9	ELOB	Elongin B	Elongin-B (Fragment)	82	2	1.02	4	6	43
G1SY93	P63000	RAC1	Rac family small GTPase 1	Ras-related C3 botulinum toxin substrate 1	90	2	1.02	6	10	31
	W4VSQ9	TRIP10		Cdc42-interacting protein 4		4	1.02	2	2	7
G1TE39	Q9NYU1	UGGT2	Uncharacterized protein	UDP-glucose:glycoprotein glucosyltransferase 2	84	3	1.02	22	24	22
G1T0F6	Q9UHB9	SRP68	Signal recognition particle subunit SRP68	Signal recognition particle subunit SRP68	95	2	1.02	9	15	18
G1T6M2		DHRS7B	Dehydrogenase/reductase 7B			1	1.02	6	11	22
G1SD91	E9PFD2	UMPS	OMPdecase domain-containing protein	Uridine 5~-monophosphate synthase	92	2	1.02	2	2	6
G1SJ77	Q9Y696	CLIC4	Chloride intracellular channel protein	Chloride intracellular channel protein 4	100	2	1.01	11	42	58
	Q96HP0	DOCK6		Dedicator of cytokinesis protein 6		4	1.01	3	4	2
G1SUK0	O75940	SMNDC1	Tudor domain-containing protein	Survival of motor neuron-related-splicing factor 30	99	2	1.01	2	2	13
G1T2F5	Q86X52	CHSY1	Hexosyltransferase	Chondroitin sulfate synthase 1	93	2	1.01	3	2	7
G1T7P8	Q9UIV1	CNOT7	Uncharacterized protein	CCR4-NOT transcription complex subunit 7	100	3	1.01	3	3	16
G1T2F2	P23284	PPIB	Peptidyl-prolyl cis-trans isomerase	Peptidyl-prolyl cis-trans isomerase B	94	2	1.01	7	10	31
	P39019	RPS19		40S ribosomal protein S19		4	1.01	11	34	57
G1TJY2		CHID1	Chitinase domain containing 1			1	1.01	10	19	53
G1U3S3		KIAA2013	KIAA2013			1	1.01	4	5	11
G1STS3	Q96T76	MMS19	Uncharacterized protein	MMS19 nucleotide excision repair protein homolog	93	3	1.01	8	11	16
	Q9NRP0	OSTC		Oligosaccharyltransferase complex subunit OSTC		4	1.01	2	5	13
G1SVW5	P36578	RPL4	Ribos_L4_asso_C domain-containing protein	60S ribosomal protein L4	96	2	1.01	14	45	36
G1TDA9	O94766	B3GAT3	Galactosylgalactosylxylosylprotein 3-beta-glucuronosyltransferase	Galactosylgalactosylxylosylprotein 3-beta-glucuronosyltransferase 3	96	2	1.01	4	4	12
G1SQ38	E5RHK8	DNM3	Uncharacterized protein	Dynamin-3	97	3	1.01	9	2	12
P80912		HINT1	Histidine triad nucleotide-binding protein 1			1	1.01	2	2	28
	M0QYZ2	AP2S1		AP complex subunit sigma		4	1.01	5	13	33
G1TYL5		DNPH1	2~-deoxynucleoside 5~-phosphate N-hydrolase 1			1	1.01	2	2	31
G1SGX4	P62249	RPS16	Uncharacterized protein	40S ribosomal protein S16	100	3	1.01	7	13	37
G1T364	Q8NDH3	NPEPL1	Aminopeptidase like 1	Probable aminopeptidase NPEPL1	90	2	1.01	3	6	9
G1T1F0	A0A2R8Y811	RPS14	Uncharacterized protein	40S ribosomal protein S14 (Fragment)	100	3	1.01	5	12	37
	O43765	SGTA		Small glutamine-rich tetratricopeptide repeat-containing protein alpha		4	1.01	2	3	8
G1TBN5		TOR1AIP2	Torsin 1A interacting protein 2			1	1.01	2	4	6
G1SFN5	E9PFR3	PPP2R5D	Serine/threonine-protein phosphatase 2A 56 kDa regulatory subunit	Serine/threonine-protein phosphatase 2A 56 kDa regulatory subunit	98	2	1.01	6	10	14
G1TXW6		GPNMB	PKD domain-containing protein			1	1.01	2	4	5
G1THT8	J3QT54	CPSF7	Cleavage and polyadenylation specific factor 7	Cleavage and polyadenylation-specificity factor subunit 7 (Fragment)	95	2	1.00	4	5	16
G1SZR6	Q13618	CUL3	CULLIN_2 domain-containing protein	Cullin-3	100	2	1.00	13	28	23
G1TCY1	Q16513	PKN2	Uncharacterized protein	Serine/threonine-protein kinase N2	97	3	1.00	4	3	4
G1T8X7	Q96JH7	VCPIP1	OTU domain-containing protein	Deubiquitinating protein VCIP135	96	2	1.00	2	3	2
G1SYI2	P62873	GNB1	WD_REPEATS_REGION domain-containing protein	Guanine nucleotide-binding protein G(I)/G(S)/G(T) subunit beta-1	100	2	1.00	12	47	45
G1SL80		UROD	Uroporphyrinogen decarboxylase			1	1.00	4	5	19
G1SGM3	A0A0J9YXF2	PON2	Uncharacterized protein	Paraoxonase 2, isoform CRA_a	93	3	1.00	12	26	58

G1SRB6	Q08752	PPID	Peptidylprolyl isomerase D	Peptidyl-prolyl cis-trans isomerase D	93	2	1.00	9	25	27
G1TI39	P35241	RDX	FERM domain-containing protein	Radixin	99	2	1.00	17	16	31
G1TAI0		KPNA2	Importin subunit alpha			1	1.00	3	6	11
G1TRG8	P04899	GNAI2	Uncharacterized protein	Guanine nucleotide-binding protein G(i) subunit alpha-2	98	3	1.00	15	19	50
G1TQA4		EPM2AIP1	EPM2A interacting protein 1			1	1.00	4	5	10
G1T3N8	Q13155	AIMP2	Uncharacterized protein	Aminoacyl tRNA synthase complex-interacting multifunctional protein 2	87	3	1.00	12	21	68
G1SE56	F6T1Q0	PDE12	Endo/exonuclease/phosphatase domain-containing protein	2~,5~-phosphodiesterase 12	90	2	1.00	3	3	10
	Q9UL15	BAG5		BAG family molecular chaperone regulator 5		4	1.00	2	2	7
G1SLK2	P62195	PSMC5	AAA domain-containing protein	26S proteasome regulatory subunit 8	100	2	1.00	14	28	43
G1SKG9	A0A087X2H1	HECTD1	Uncharacterized protein	E3 ubiquitin-protein ligase HECTD1	99	3	1.00	7	11	4
G1T312		ACAD8	Acyl-CoA dehydrogenase family member 8			1	0.99	2	2	5
G1SKK5		PEX6	Peroxisomal biogenesis factor 6			1	0.99	2	3	6
G1SIT5	F2Z388	RPL35	Uncharacterized protein	60S ribosomal protein L35	93	3	0.99	3	5	25
	Q92900	UPF1		Regulator of nonsense transcripts 1		4	0.99	18	27	23
P40144		ADCY5	Adenylate cyclase type 5			1	0.99	3	4	4
G1SI95	O00291	HIP1	Uncharacterized protein	Huntingtin-interacting protein 1	91	3	0.99	15	15	24
G1SY96	Q9UJS0	SLC25A13	Uncharacterized protein	Calcium-binding mitochondrial carrier protein Aralar2	97	3	0.99	11	5	28
G1TWU1		TOR1B	Torsin			1	0.99	5	5	19
G1SU24	O60503	ADCY9	Adenylate cyclase 9	Adenylate cyclase type 9	92	2	0.99	2	2	4
G1TYK8	Q13492	PICALM	Phosphatidylinositol binding clathrin assembly protein	Phosphatidylinositol-binding clathrin assembly protein	97	2	0.99	12	32	27
G1SVY8	P12277	CKB	Creatine kinase B-type	Creatine kinase B-type	97	2	0.99	19	227	70
G1SSA2	Q13200	PSMD2	26S proteasome non-ATPase regulatory subunit 2	26S proteasome non-ATPase regulatory subunit 2	99	2	0.99	30	86	47
G1SLU5	Q9UKZ1	CNOT11	CCR4-NOT transcription complex subunit 11	CCR4-NOT transcription complex subunit 11	79	2	0.99	2	2	6
G1SMM4		DKK3	Dickkopf_N domain-containing protein			1	0.99	3	8	14
G1SLF8	J3KN16	ECPAS	Vac14_Fab1_bd domain-containing protein	Proteasome adapter and scaffold protein ECM29	97	2	0.99	18	32	17
G1T279	Q9UHW5	GPN3	GPN-loop GTPase 3	GPN-loop GTPase 3	96	2	0.99	2	4	12
G1SYH5		NPTN	Neuroplastin			1	0.99	2	2	8
	E9PM12	TCIRG1		V-type proton ATPase subunit a (Fragment)		4	0.99	2	2	9
G1TRH3	Q9UBR2	CTSZ	Pept_C1 domain-containing protein	Cathepsin Z	86	2	0.99	7	36	30
G1U354	R4GMR5	PSMD8	PCI domain-containing protein	26S proteasome non-ATPase regulatory subunit 8	97	2	0.99	9	16	33
G1T9M9	P11142	HSPA8	Uncharacterized protein	Heat shock cognate 71 kDa protein	100	3	0.99	32	713	71
G1TJ79		KYAT1	Aminotran_1_2 domain-containing protein			1	0.99	2	2	10
P27115		MGAT1	Alpha-1,3-mannosyl-glycoprotein 2-beta-N-acetylglucosaminyltransferase			1	0.99	3	3	12
G1T1X4	O43395	PRPF3	PWI domain-containing protein	U4/U6 small nuclear ribonucleoprotein Prp3	100	2	0.99	2	2	7
G1TCS8	P62820	RAB1A	Uncharacterized protein	Ras-related protein Rab-1A	100	3	0.99	13	152	74
G1SYF9	Q9Y613	FHOD1	Uncharacterized protein	FH1/FH2 domain-containing protein 1	87	3	0.99	5	6	9
G1SWA0		RIPOR1	RHO family interacting cell polarization regulator 1			1	0.99	3	4	5
G1SWN4	Q9H832	UBE2Z	UBIQUITIN_CONJUGAT_2 domain-containing protein	Ubiquitin-conjugating enzyme E2 Z	96	2	0.99	3	4	11
G1T4Q7		ATP11C	Phospholipid-transporting ATPase			1	0.99	2	5	3
P41982	P04179	SOD2	Superoxide dismutase [Mn], mitochondrial (Fragment)	Superoxide dismutase [Mn], mitochondrial	92	2	0.99	4	22	25
	F8VV64	TNS2		Tensin-2		4	0.99	2	3	2
G1SD02	E7EM64	COPS6	COP9 signalosome subunit 6	COP9 signalosome complex subunit 6	97	2	0.98	3	5	11
G1SZ91		FABP5	Lipocln_cytosolic_FA-bd_dom domain-containing protein			1	0.98	4	11	37
G1SM04	Q13188	STK3	Uncharacterized protein	Serine/threonine-protein kinase 3	99	3	0.98	2	2	5
G1T198	Q8TDX7	NEK7	Protein kinase domain-containing protein	Serine/threonine-protein kinase Nek7	98	2	0.98	3	4	15
G1SNY0	E9PJD9	RPL27A	Ribosomal_L18e/L15P domain-containing protein	60S ribosomal protein L27a	98	2	0.98	3	5	22
G1T6B3	Q9Y3F4	STRAP	WD_REPEATS_REGION domain-containing protein	Serine-threonine kinase receptor-associated protein	98	2	0.98	13	33	52
O19048	Q15365	PCBP1	Poly(rC)-binding protein 1	Poly(rC)-binding protein 1	100	2	0.98	14	27	64
G1TEY6	O00178	GTPBP1	Tr-type G domain-containing protein	GTP-binding protein 1	94	2	0.98	5	9	12
G1T2R2	A0A3B3ITU8	ITPR1	Uncharacterized protein	Inositol 1,4,5-trisphosphate receptor type 1	97	3	0.98	4	2	2
G1U4H9	O14907	TAX1BP3	Tax1-binding protein 3	Tax1-binding protein 3	100	2	0.98	2	4	28
G1T3E6	A0A024R442	DNPEP	Uncharacterized protein	Aspartyl aminopeptidase	91	3	0.98	9	10	30
G1T1C4		HSD17B7	Hydroxysteroid 17-beta dehydrogenase 7			1	0.98	3	3	16
P30946	P07900	HSP90AA1	Heat shock protein HSP 90-alpha	Heat shock protein HSP 90-alpha	94	2	0.98	36	276	63
G1T2Z8	B4DUC8	MTAP	S-methyl-5~-thioadenosine phosphorylase	S-methyl-5~-thioadenosine phosphorylase	99	2	0.98	4	7	27
G1T6E8	F5H442	TSG101	Uncharacterized protein	Tumor susceptibility gene 101 protein	99	3	0.98	6	8	25
G1SUR8	B7ZBJ4	CAB39L	Uncharacterized protein	Calcium-binding protein 39-like	98	3	0.98	3	3	12
G1TDF6	O75955	FLOT1	PHB domain-containing protein	Flotillin-1	99	2	0.98	13	23	40
G1TQ79		CHCHD6	MICOS complex subunit			1	0.98	3	3	15
G1T6D4	O00232	PSMD12	PCI domain-containing protein	26S proteasome non-ATPase regulatory subunit 12	99	2	0.98	16	43	48

	O43752	STX6		Syntaxin-6		4	0.98	2	2	13
	Q9Y2Q5	LAMTOR2		Ragulator complex protein LAMTOR2		4	0.98	2	2	22
G1T725	Q8TB40	ABHD4	AB hydrolase-1 domain-containing protein	(Lyso)-N-acylphosphatidylethanolamine lipase	97	2	0.98	2	3	8
G1SU30	A0A024RCR6	BAG6	Ubiquitin-like domain-containing protein	BAG6	91	2	0.98	8	9	12
G1TA15	Q9P2J5	LARS	Uncharacterized protein	Leucine--tRNA ligase, cytoplasmic	95	3	0.98	25	49	29
	Q15366-3	PCBP2		Isoform 3 of Poly(rC)-binding protein 2		4	0.98	13	4	56
G1T798	Q12846	STX4	t-SNARE coiled-coil homology domain-containing protein	Syntaxin-4	96	2	0.98	4	6	27
G1SVF2	A0A087WW66	PSMD1	26S proteasome non-ATPase regulatory subunit 1	26S proteasome non-ATPase regulatory subunit 1	99	2	0.97	29	8	44
	O94973	AP2A2		AP-2 complex subunit alpha-2		4	0.97	27	41	42
G1T520	Q99615	DNAJC7	Uncharacterized protein	DnaJ homolog subfamily C member 7	98	3	0.97	3	3	10
G1T9V7		SRP19	Signal recognition particle 19			1	0.97	2	3	26
G1T7Y5	H0Y368	DPM1	Dolichol-phosphate mannosyltransferase subunit 1	Dolichol-phosphate mannosyltransferase subunit 1 (Fragment)	83	2	0.97	5	6	23
G1SJB9	Q14257	RCN2	Reticulocalbin 2	Reticulocalbin-2	90	2	0.97	12	135	56
	Q9NWU2	GID8		Glucose-induced degradation protein 8 homolog		4	0.97	5	10	41
G1TXN1		NIT2	CN hydrolase domain-containing protein			1	0.97	3	3	14
G1SWY6	Q15075	EEA1	Early endosome antigen 1	Early endosome antigen 1	87	2	0.97	32	21	28
G1SDC6	Q9NXC5	MIOS	zinc_ribbon_16 domain-containing protein	GATOR complex protein MIOS	98	2	0.97	2	2	2
G1T2Z5	A0A087WTA8	COL1A2	Collagen alpha-2(I) chain	Collagen alpha-2(I) chain	94	2	0.97	28	177	33
G1TQR2	P24534	EEF1B2	Uncharacterized protein	Elongation factor 1-beta	98	3	0.97	10	26	65
G1SEN8		SCCPDH	Sacchrp_dh_NADP domain-containing protein			1	0.97	5	9	24
G1T5U1		GPN1	GPN-loop GTPase			1	0.97	2	2	17
G1U7C7	Q5GLZ8	HERC4	HECT domain-containing protein	Probable E3 ubiquitin-protein ligase HERC4	96	2	0.97	3	3	6
G1SJN5		MAN2A1	Alpha-mannosidase			1	0.97	8	8	12
G1SFE0	O00487	PSMD14	MPN domain-containing protein	26S proteasome non-ATPase regulatory subunit 14	100	2	0.97	7	13	45
G1SNL4	Q9Y385	UBE2J1	UBIQUITIN_CONJUGAT_2 domain-containing protein	Ubiquitin-conjugating enzyme E2 J1	94	2	0.97	3	4	14
G1SRL3	P30419	NMT1	Glycylpeptide N-tetradecanoyltransferase	Glycylpeptide N-tetradecanoyltransferase 1	98	2	0.97	7	9	19
	V9GYD0	ARL2-SNX15		ARL2-SNX15 readthrough (NMD candidate)		4	0.96	3	6	32
G1SJN4	Q9BT78	COPS4	PCI domain-containing protein	COP9 signalosome complex subunit 4	100	2	0.96	12	28	49
G1T3V3	Q6R327	RICTOR	Uncharacterized protein	Rapamycin-insensitive companion of mTOR	98	3	0.96	4	6	4
G1T239	F5GYQ1	ATP6V0D1	V-type proton ATPase subunit	V-type proton ATPase subunit	90	2	0.96	8	12	31
G1T2G6	Q9P265	DIP2B	DMAP-interaction domain-containing protein	Disco-interacting protein 2 homolog B	99	2	0.96	2	2	2
P41035	P20042	EIF2S2	Eukaryotic translation initiation factor 2 subunit 2	Eukaryotic translation initiation factor 2 subunit 2	98	2	0.96	12	26	53
G1SLD6	B3KWE1	HARS	Uncharacterized protein	Histidine--tRNA ligase, cytoplasmic	97	3	0.96	7	9	19
G1SDN9	Q9P2B2	PTGFRN	Uncharacterized protein	Prostaglandin F2 receptor negative regulator	91	3	0.96	6	10	8
G1SGR0	Q8N3P4	VPS8	Uncharacterized protein	Vacuolar protein sorting-associated protein 8 homolog	93	3	0.96	2	3	3
G1SXB8	Q10567	AP1B1	AP complex subunit beta	AP-1 complex subunit beta-1	98	2	0.96	32	6	45
G1TA11	P54136	RARS	Uncharacterized protein	Arginine--tRNA ligase, cytoplasmic	92	3	0.96	23	46	43
G1U8P2	J3KTE4	RPL19	Ribosomal protein L19	Ribosomal protein L19	98	2	0.96	7	16	30
G1TRV7		QPCTL	Glutaminyl-peptide cyclotransferase like			1	0.96	6	10	32
G1T6N8		ALG12	Mannosyltransferase			1	0.96	2	2	9
G1SCS8		ANO6	Anoctamin			1	0.96	5	7	8
G1T593	F8VVA7	COPZ1	Clat_adaptor_s domain-containing protein	Coatomer subunit zeta-1	88	2	0.96	4	13	45
G1SIY9		ALDH5A1	Succinate-semialdehyde dehydrogenase			1	0.96	2	2	9
G1SEK1	P62140	PPP1CB	Serine/threonine-protein phosphatase	Serine/threonine-protein phosphatase PP1-beta catalytic subunit	100	2	0.96	9	3	35
G1SGE5	A0A087WZR9	PYCR2	Pyrroline-5-carboxylate reductase	Pyrroline-5-carboxylate reductase	94	2	0.96	6	6	26
G1T4N5	P42345	MTOR	Serine/threonine-protein kinase mTOR	Serine/threonine-protein kinase mTOR	99	2	0.96	16	17	11
G1SPG6	A6NEM5	PIGK	GPI-anchor transamidase	GPI-anchor transamidase	85	2	0.96	4	9	22
G1SNE8	P31949	S100A11	Protein S100	Protein S100-A11	87	2	0.96	5	46	65
G1SEN5	Q8IZ07	ANKRD13A	Ankyrin repeat domain 13A	Ankyrin repeat domain-containing protein 13A	94	2	0.96	2	3	5
Q95MN6		PLP2	Proteolipid protein 2			1	0.96	3	7	34
G1TTU6	E7ERH2	SKP1	Uncharacterized protein	S-phase kinase-associated protein 1 (Fragment)	100	3	0.96	6	58	56
G1SML4	P28288	ABCD3	Uncharacterized protein	ATP-binding cassette sub-family D member 3	96	3	0.96	10	10	21
P00169	P00167	CYB5A	Cytochrome b5	Cytochrome b5	90	2	0.96	7	16	80
G1SQT2	Q6P2E9	EDC4	WD_REPEATS_REGION domain-containing protein	Enhancer of mRNA-decapping protein 4	96	2	0.96	3	4	5
G1SG37	G5E9T8	GOSR1	Golgi SNAP receptor complex member 1	Golgi SNAP receptor complex member 1 (Fragment)	99	2	0.96	3	7	20
G1SQR7	P61970	NUTF2	NTF2 domain-containing protein	Nuclear transport factor 2	100	2	0.96	2	2	17
G1TE13	P84095	RHOG	Uncharacterized protein	Rho-related GTP-binding protein RhoG	99	3	0.96	6	7	41
G1TA41	Q7L7X3	TAOK1	Protein kinase domain-containing protein	Serine/threonine-protein kinase TAO1	100	2	0.96	4	4	6
G1SWE5		WDR61	WD repeat domain 61			1	0.96	2	3	12
	F5H039	GPHN		Molybdopterin molybdenumtransferase		4	0.96	2	3	5

G1SVA3	O00231	PSMD11	PCI domain-containing protein	26S proteasome non-ATPase regulatory subunit 11	100	2	0.96	17	38	56
G1SGS7	O94874	UFL1	Uncharacterized protein	E3 UFM1-protein ligase 1	94	3	0.96	16	28	29
G1TUD6	P43686	PSMC4	Proteasome 26S subunit, ATPase 4	26S proteasome regulatory subunit 6B	100	2	0.95	12	57	34
G1SUM3	Q8TBC4	UBA3	E2_bind domain-containing protein	NEDD8-activating enzyme E1 catalytic subunit	99	2	0.95	5	7	21
G1TKY7		OGFOD3	2-oxoglutarate and iron dependent oxygenase domain containing 3			1	0.95	3	3	13
G1T0K1	Q9BTV4	TMEM43	Uncharacterized protein	Transmembrane protein 43	92	3	0.95	10	21	39
G1SKM5	Q8TAT6	NPLOC4	NPL4 homolog, ubiquitin recognition factor	Nuclear protein localization protein 4 homolog	92	2	0.95	5	6	12
G1SKF5		ERBIN	Erbb2 interacting protein			1	0.95	5	5	9
G1T3D1	Q14789	GOLGB1	Uncharacterized protein	Golgin subfamily B member 1	85	3	0.95	32	46	15
G1SV51	Q08378	GOLGA3	Golgin A3	Golgin subfamily A member 3	85	2	0.95	12	16	16
G1SNQ9	A0A0A0MTJ9	NCEH1	Uncharacterized protein	Neutral cholesterol ester hydrolase 1	88	3	0.95	9	11	32
G1T7H0	A0A1W2PPS1	HNRNPU	Uncharacterized protein	Heterogeneous nuclear ribonucleoprotein U	96	3	0.95	22	62	33
G1SRP2	Q5SWX8	ODR4	Uncharacterized protein	Protein odr-4 homolog	89	3	0.95	12	15	43
G1SH42	Q5VSL9	STRIP1	Uncharacterized protein	Striatin-interacting protein 1	98	3	0.95	4	4	12
G1SYR5		EIF2B2	Translation initiation factor eIF-2B subunit beta			1	0.95	2	3	8
G1SPV0	P61086	UBE2K	Uncharacterized protein	Ubiquitin-conjugating enzyme E2 K	100	3	0.95	4	7	35
G1TGY9		VPS11	VPS11, CORVET/HOPS core subunit			1	0.95	2	2	3
G1U013	H7C3P9	COPS3	PCI domain-containing protein	COP9 signalosome complex subunit 3	92	2	0.95	4	6	18
G1SGQ0	Q9UBS4	DNAJB11	J domain-containing protein	DnaJ homolog subfamily B member 11	98	2	0.95	9	20	35
	K7ER00	FARSA		Phenylalanine--tRNA ligase alpha subunit		4	0.95	3	2	6
G1SQG5	P40925	MDH1	Malate dehydrogenase	Malate dehydrogenase, cytoplasmic	97	2	0.95	12	17	57
G1SDU6	P26639	TARS	AA_TRNA_LIGASE_II domain-containing protein	Threonine--tRNA ligase, cytoplasmic	96	2	0.95	17	27	28
G1TA78	P23381	WARS	Tryptophan--tRNA ligase, cytoplasmic	Tryptophan--tRNA ligase, cytoplasmic	89	2	0.95	11	25	31
G1SSV1	Q99627	COPS8	PCI domain-containing protein	COP9 signalosome complex subunit 8	99	2	0.95	4	13	38
U3KM62		PTGS2	Prostaglandin G/H synthase 2			1	0.95	6	7	14
	A0A087X0W8	RELA		Transcription factor p65		4	0.95	2	2	7
G1T7L0		CTSC	Pept_C1 domain-containing protein			1	0.94	5	5	20
G1SMG5	P15170	GSPT1	Tr-type G domain-containing protein	Eukaryotic peptide chain release factor GTP-binding subunit ERF3A	99	2	0.94	11	18	25
G1T3S1	A0A087X2I1	PSMC6	AAA domain-containing protein	26S proteasome regulatory subunit 10B	100	2	0.94	12	25	42
P47823		EIF2B5	Translation initiation factor eIF-2B subunit epsilon			1	0.94	5	10	14
G1T0H9	O60645	EXOC3	Exocyst complex component 3	Exocyst complex component 3	95	2	0.94	3	4	5
G1T579	Q6NUQ1	RINT1	Uncharacterized protein	RAD50-interacting protein 1	92	3	0.94	7	12	15
G1SIX1	Q9H2G2	SLK	Uncharacterized protein	STE20-like serine/threonine-protein kinase	88	3	0.94	6	8	12
G1TM00	O75436	VPS26A	VPS26, retromer complex component A	Vacuolar protein sorting-associated protein 26A	99	2	0.94	2	2	10
G1T329	J3QQY1	CDK5RAP3	Uncharacterized protein	CDK5 regulatory subunit-associated protein 3 (Fragment)	88	3	0.94	9	14	25
G1T5J8	Q9UJW0	DCTN4	Uncharacterized protein	Dynactin subunit 4	97	3	0.94	6	8	29
G1T3M5	O43324	EEF1E1	GST C-terminal domain-containing protein	Eukaryotic translation elongation factor 1 epsilon-1	95	2	0.94	6	8	44
G1SQD1	E7EUU4	EIF4G1	Eukaryotic translation initiation factor 4 gamma 1	Eukaryotic translation initiation factor 4 gamma 1	94	2	0.94	21	36	15
	A8MZF9	DRG2		Developmentally-regulated GTP-binding protein 2		4	0.94	3	7	14
G1SMW3	Q92615	LARP4B	La ribonucleoprotein domain family member 4B	La-related protein 4B	88	2	0.94	3	3	8
G1TDJ3	Q9BSJ8	ESYT1	Uncharacterized protein	Extended synaptotagmin-1	89	3	0.94	30	46	41
G1TRA4	P01111	NRAS	Uncharacterized protein	GTPase NRas	100	3	0.94	7	3	53
G1SUV0	Q9NW15	ANO10	Anoctamin	Anoctamin-10	94	2	0.94	5	11	12
G1T7T0	Q9Y2G5	POFUT2	Protein O-fucosyltransferase 2	GDP-fucose protein O-fucosyltransferase 2	84	2	0.94	3	4	13
	H0Y5B4	RPL36A		60S ribosomal protein L36a		4	0.94	3	6	16
G1SMB5		VPS37C	VPS37 C-terminal domain-containing protein			1	0.94	4	6	21
G1T0T5	F5GYF7	COPS7A	PCI domain-containing protein	COP9 signalosome complex subunit 7a (Fragment)	100	2	0.94	2	4	10
G1SMI6	Q13162	PRDX4	Thioredoxin domain-containing protein	Peroxiredoxin-4	96	2	0.94	10	17	41
G1SP21	H0Y9A1	YIPF3	Uncharacterized protein	Protein YIPF3 (Fragment)	72	3	0.94	3	14	16
G1T0B0	Q9H3H3	C11orf68	Uncharacterized protein	UPF0696 protein C11orf68	93	3	0.93	2	3	13
G1TAL6	Q10471	GALNT2	Polypeptide N-acetylgalactosaminyltransferase	Polypeptide N-acetylgalactosaminyltransferase 2	99	2	0.93	10	18	23
G1SNZ8		GORASP1	GRASP55_65 domain-containing protein			1	0.93	2	3	7
G1SKE2	E7ETC0	TIAL1	TIA1 cytotoxic granule associated RNA binding protein like 1	Nucleolysin TIAR	98	2	0.93	2	6	8
G1TY83		NLRX1	NLR family member X1			1	0.93	3	4	6
G1TUP1	O75340	PDCD6	Programmed cell death 6	Programmed cell death protein 6	99	2	0.93	5	14	29
Q9TTC6	P62937	PPIA	Peptidyl-prolyl cis-trans isomerase A	Peptidyl-prolyl cis-trans isomerase A	96	2	0.93	16	23	87
G1TQD4		RALY	RRM domain-containing protein			1	0.93	2	2	6
	P62854	RPS26		40S ribosomal protein S26		4	0.93	2	11	21
G1TM22		UGT1A1	UDP-glucuronosyltransferase			1	0.93	7	2	22
G1SYV0	P35998	PSMC2	AAA domain-containing protein	26S proteasome regulatory subunit 7	100	2	0.93	17	40	46

	Q9UNK0	STX8		Syntaxin-8		4	0.93	2	2	12
G1SDM6	O75165	DNAJC13	J domain-containing protein	DnaJ homolog subfamily C member 13	98	2	0.93	27	36	17
G1T7N4	P41743	PRKCI	Protein kinase C	Protein kinase C iota type	99	2	0.93	2	2	7
G1SCY3		UBR4	UBR-type domain-containing protein			1	0.93	46	78	17
G1SQF7	Q14554	PDIA5	Uncharacterized protein	Protein disulfide-isomerase A5	92	3	0.93	10	18	29
G1TE61	O60684	KPNA6	Importin subunit alpha	Importin subunit alpha-7	99	2	0.93	10	17	34
G1U2W0	B4E321	OS9	OS9, endoplasmic reticulum lectin	Protein OS-9	79	2	0.93	3	2	5
G1SR15		CD109	CD109 molecule			1	0.93	2	3	2
G1SHZ2	Q86X83	COMMD2	COMM domain-containing protein	COMM domain-containing protein 2	97	2	0.93	2	4	17
G1SXN1	Q9BXB4	OSBPL11	Oxysterol-binding protein	Oxysterol-binding protein-related protein 11	93	2	0.93	2	4	4
G1SIE6	P29279	CCN2	Cellular communication network factor 2	CCN family member 2	90	2	0.93	11	16	32
G1THI9	Q9UKB1	FBXW11	Uncharacterized protein	F-box/WD repeat-containing protein 11	96	3	0.93	2	2	7
G1SQW0	E9PIE4	MTCH2	Uncharacterized protein	Mitochondrial carrier homolog 2 (Fragment)	92	3	0.93	6	15	29
	A0A087X1G7	SELENOF		Selenoprotein F		4	0.93	3	15	22
G1T089		THUMPD3	THUMP domain containing 3			1	0.93	3	3	9
G1SVJ8	Q96Q05	TRAPPC9	Uncharacterized protein	Trafficking protein particle complex subunit 9	87	3	0.93	2	2	2
G1TE37		ATP6AP1	ATPase H+ transporting accessory protein 1			1	0.93	2	2	10
	P54725	RAD23A		UV excision repair protein RAD23 homolog A		4	0.93	5	12	23
G1T5R0		TUBGCP3	Gamma-tubulin complex component			1	0.93	2	3	5
G1SN85	A0A0D9SEN1	FAP	Uncharacterized protein	Prolyl endopeptidase FAP	95	3	0.92	27	89	40
G1SF36	Q13423	NNT	Uncharacterized protein	NAD(P) transhydrogenase, mitochondrial	97	3	0.92	25	60	28
	P68402	PAFAH1B2		Platelet-activating factor acetylhydrolase IB subunit beta		4	0.92	4	11	37
G1T2W1	O76094	SRP72	Signal recognition particle subunit SRP72	Signal recognition particle subunit SRP72	98	2	0.92	9	15	20
G1SZN0	Q9NZB2	FAM120A	Uncharacterized protein	Constitutive coactivator of PPAR-gamma-like protein 1	94	3	0.92	5	6	6
	P63241	EIF5A		Eukaryotic translation initiation factor 5A-1		4	0.92	9	221	66
G1T7L7		KDELC2	CAP10 domain-containing protein			1	0.92	10	16	25
G1TX94	A0A0R4J2E8	MATR3	Uncharacterized protein	Matrin-3	99	3	0.92	17	33	28
G1TBU8	P52306	RAP1GDS1	Uncharacterized protein	Rap1 GTPase-GDP dissociation stimulator 1	97	3	0.92	6	8	15
G1SQK0	A0A087X1A5	STAU1	Uncharacterized protein	Double-stranded RNA-binding protein Staufen homolog 1	89	3	0.92	2	2	4
G1SH95	P30622	CLIP1	CAP-Gly domain containing linker protein 1	CAP-Gly domain-containing linker protein 1	85	2	0.92	12	12	10
G1T659	Q9UHY7	ENOPH1	Enolase-phosphatase E1	Enolase-phosphatase E1	97	2	0.92	2	2	12
G1TBC1	P14625	HSP90B1	Endoplasmin	Endoplasmin	94	2	0.92	43	950	55
G1U9D3	Q9UK41	VPS28	Vacuolar protein sorting-associated protein 28 homolog	Vacuolar protein sorting-associated protein 28 homolog	93	2	0.92	4	5	29
G1TW04	Q16643	DBN1	Drebrin 1	Drebrin	63	2	0.92	12	99	28
G1T846	P14868	DARS	AA_TRNA_LIGASE_II domain-containing protein	Aspartate--tRNA ligase, cytoplasmic	98	2	0.92	10	21	22
G1T4Q8	J3KNQ4	PARVA	Uncharacterized protein	Alpha-parvin	91	3	0.92	11	27	34
	O15031	PLXNB2		Plexin-B2		4	0.92	7	8	6
G1TCK9	A0A0A0MSX9	IARS	Uncharacterized protein	Isoleucine--tRNA ligase, cytoplasmic	94	3	0.92	33	64	34
G1SUJ1	P24390	KDELR1	ER lumen protein-retaining receptor	ER lumen protein-retaining receptor 1	96	2	0.92	3	2	21
G1U484		LLGL1	LLGL scribble cell polarity complex component 1			1	0.92	2	2	3
G1SN68	A0A1B0GVU9	QARS	Uncharacterized protein	Glutamine--tRNA ligase (Fragment)	92	3	0.92	25	42	43
G1SHH0	Q14139	UBE4A	Ubiquitination factor E4A	Ubiquitin conjugation factor E4 A	98	2	0.92	3	6	7
G1U2R1	Q99805	TM9SF2	Transmembrane 9 superfamily member	Transmembrane 9 superfamily member 2	93	2	0.92	6	23	16
G1T9V2	A0A2R8Y852	CUX1	Cut like homeobox 1	Homeobox protein cut-like	87	2	0.92	4	5	5
G1SI20	O75874	IDH1	Isocitrate dehydrogenase [NADP]	Isocitrate dehydrogenase [NADP] cytoplasmic	97	2	0.92	19	17	49
G1TWC3		TMX1	Thioredoxin domain-containing protein			1	0.92	5	7	19
G1SMQ8	Q9P253	VPS18	Pep3_Vps18 domain-containing protein	Vacuolar protein sorting-associated protein 18 homolog	97	2	0.92	2	2	3
G1SEM0	Q96AC1	FERMT2	PH domain-containing protein	Fermitin family homolog 2	100	2	0.92	22	42	47
G1SCQ0		EDEM3	alpha-1,2-Mannosidase			1	0.91	2	2	4
G1TWK7	Q14696	MESD	Uncharacterized protein	LRP chaperone MESD	90	3	0.91	4	7	22
G1TAF8	P31948	STIP1	Uncharacterized protein	Stress-induced-phosphoprotein 1	96	3	0.91	26	46	52
G1T4P8	O76003	GLRX3	Glutaredoxin 3	Glutaredoxin-3	93	2	0.91	10	19	46
	P30533	LRPAP1		Alpha-2-macroglobulin receptor-associated protein		4	0.91	2	4	5
G1T235	P28072	PSMB6	Proteasome subunit beta	Proteasome subunit beta type-6	97	2	0.91	6	13	40
G1T2T9	P20339	RAB5A	Uncharacterized protein	Ras-related protein Rab-5A	99	3	0.91	8	12	54
G1SZI6	Q86X10	RALGAPB	Rap-GAP domain-containing protein	Ral GTPase-activating protein subunit beta	97	2	0.91	4	3	3
G1SZ93	Q9NSE4	IARS2	Uncharacterized protein	Isoleucine--tRNA ligase, mitochondrial	90	3	0.91	14	17	22
G1SCI5	F5H6E2	MYO1C	Uncharacterized protein	Unconventional myosin-Ic	92	3	0.91	40	104	46
G1U6B2		ALAD	Delta-aminolevulinic acid dehydratase			1	0.91	2	2	15
G1SM31	Q9P2G1	ANKIB1	RBR-type E3 ubiquitin transferase	Ankyrin repeat and IBR domain-containing protein 1	93	2	0.91	2	4	4

G1SZA3	Q9H2M9	RAB3GAP2	Uncharacterized protein	Rab3 GTPase-activating protein non-catalytic subunit	94	3	0.91	13	12	20
	Q9UNE7	STUB1		E3 ubiquitin-protein ligase CHIP		4	0.91	2	2	9
U3KP45		SYNE1	Spectrin repeat containing nuclear envelope protein 1			1	0.91	4	6	1
G1SJL0		TIMM29	Translocase of inner mitochondrial membrane 29			1	0.91	2	3	16
G1SMM5	O60884	DNAJA2	Uncharacterized protein	DnaJ homolog subfamily A member 2	100	3	0.91	6	13	29
G1SGN0	Q15042	RAB3GAP1	Uncharacterized protein	Rab3 GTPase-activating protein catalytic subunit	94	3	0.91	10	9	17
G1SUX1		TIMP3	Metalloproteinase inhibitor 3			1	0.91	2	2	13
G1TCE2	H7C0R7	CYB5R1	NADH-cytochrome b5 reductase	NADH-cytochrome b5 reductase 1 (Fragment)	77	2	0.91	6	8	28
G1SRP7	O00505	KPNA3	Importin subunit alpha	Importin subunit alpha-4	99	2	0.91	8	10	27
	H0Y507	SH3PXD2A		SH3 and PX domain-containing protein 2A (Fragment)		4	0.91	2	2	3
G1SPI7	Q92538	GBF1	SEC7 domain-containing protein	Golgi-specific brefeldin A-resistance guanine nucleotide exchange factor 1	96	2	0.90	18	22	14
	Q8WUW1	BRK1		Protein BRICK1		4	0.90	2	3	23
G1T8E2	A0A3B3ITW1	GSK3B	Protein kinase domain-containing protein	Glycogen synthase kinase-3 beta	87	2	0.90	2	3	10
G1SUP1	A0A0D9SEY1	MAP4K4	Mitogen-activated protein kinase kinase kinase kinase 4	Mitogen-activated protein kinase kinase kinase kinase 4	91	2	0.90	6	8	7
G1SZR4	H7BZ14	PPIL3	Peptidyl-prolyl cis-trans isomerase	Peptidyl-prolyl cis-trans isomerase (Fragment)	100	2	0.90	2	3	12
G1SVH8	Q13636	RAB31	Uncharacterized protein	Ras-related protein Rab-31	95	3	0.90	6	8	36
G1SNM5	A0A2R8Y566	RELCH	LisH domain-containing protein	RAB11-binding protein RELCH	96	2	0.90	3	3	5
G1T8P4	P05386	RPLP1	Uncharacterized protein	60S acidic ribosomal protein P1	97	3	0.90	4	10	72
G1TZQ5	G5EA31	SEC24C	Uncharacterized protein	Protein transport protein Sec24C	93	3	0.90	6	8	10
G1SRJ6		AFAP1	Actin filament associated protein 1			1	0.90	3	7	7
G1SZQ7	Q7L1Q6	BZW1	W2 domain-containing protein	Basic leucine zipper and W2 domain-containing protein 1	100	2	0.90	12	18	39
G1SPD2	Q16401	PSMD5	Uncharacterized protein	26S proteasome non-ATPase regulatory subunit 5	92	3	0.90	12	15	43
G1SSV0	Q9H1E5	TMX4	Thioredoxin domain-containing protein	Thioredoxin-related transmembrane protein 4	84	2	0.90	3	4	12
P06813	A0A075B7C0	CAPNS1	Calpain small subunit 1	Calpain small subunit 1 (Fragment)	96	2	0.90	10	22	69
G1SL98	Q53EP0	FNDC3B	Uncharacterized protein	Fibronectin type III domain-containing protein 3B	97	3	0.90	15	4	20
G1TC33	P35580	MYH10	Uncharacterized protein	Myosin-10	99	3	0.90	102	327	54
G1SRA9	A0A0A0MTH3	ILK	Uncharacterized protein	Integrin-linked protein kinase	93	3	0.90	11	16	27
G1STR6	Q96FN9	DTD2	Uncharacterized protein	D-aminoacyl-tRNA deacylase 2	93	3	0.90	2	3	19
G1U101	J3KPF0	HECTD4	HECT domain-containing protein	Probable E3 ubiquitin-protein ligase HECTD4	97	2	0.90	2	5	1
G1T8K2	A0A0U1RR22	PACSIN2	Uncharacterized protein	Protein kinase C and casein kinase substrate in neurons protein 2 (Fragment)	93	3	0.90	8	10	23
G1SE46	O95159	ZFPL1	Zinc finger protein like 1	Zinc finger protein-like 1	92	2	0.90	4	4	15
G1U9U1	P50995	ANXA11	Annexin	Annexin A11	92	2	0.90	7	9	17
G1SG59	Q7L9L4	MOB1B	Uncharacterized protein	MOB kinase activator 1B	100	3	0.90	2	6	15
G1SH86		TBCD	Tubulin folding cofactor D			1	0.90	13	20	17
G1SDT9	P49754	VPS41	Vacuolar protein sorting-associated protein 41 homolog	Vacuolar protein sorting-associated protein 41 homolog	98	2	0.90	5	6	8
G1SP89	J3QLD9	FLOT2	PHB domain-containing protein	Flotillin-2	97	2	0.90	11	16	34
G1U460	Q02252	ALDH6A1	Aldedh domain-containing protein	Methylmalonate-semialdehyde dehydrogenase [acylating], mitochondrial	95	2	0.89	8	17	26
	A0A0J9YXC7	LIMS4		LIM and senescent cell antigen-like-containing domain protein		4	0.89	2	4	7
G1TIB4	P62857	RPS28	Ribosomal protein S28	40S ribosomal protein S28	100	2	0.89	3	8	46
G1SJ23	P07384	CAPN1	Calpain-1 catalytic subunit	Calpain-1 catalytic subunit	90	2	0.89	10	10	25
G1T332		GOT1	Aspartate aminotransferase			1	0.89	3	4	10
P63150	P63151	PPP2R2A	Serine/threonine-protein phosphatase 2A 55 kDa regulatory subunit B alpha isoform	Serine/threonine-protein phosphatase 2A 55 kDa regulatory subunit B alpha isoform	100	2	0.89	4	7	15
Q28717	F6WIT2	PTPA	Serine/threonine-protein phosphatase 2A activator	Serine/threonine-protein phosphatase 2A activator	97	2	0.89	8	13	41
G1TK72	F8W8H5	RAB24	Uncharacterized protein	Ras-related protein Rab-24	98	3	0.89	2	3	13
G1SJF1	A0A499FIZ0	WDR26	Uncharacterized protein	WD repeat-containing protein 26	99	3	0.89	7	8	18
G1SJ61	Q9Y608	LRRFIP2	Uncharacterized protein	Leucine-rich repeat flightless-interacting protein 2	91	3	0.89	7	9	13
G1T645	Q96S52	PIGS	Uncharacterized protein	GPI transamidase component PIG-S	85	3	0.89	6	11	17
G1SF45		SEC24B	SEC24 homolog B, COPII coat complex component			1	0.89	3	4	4
G1SMZ9		ICMT	Protein-S-isoprenylcysteine O-methyltransferase			1	0.89	2	2	11
G1T3A2	Q9Y3A6	TMED5	GOLD domain-containing protein	Transmembrane emp24 domain-containing protein 5	97	2	0.89	5	13	24
P40826	P54578	USP14	Ubiquitin carboxyl-terminal hydrolase 14	Ubiquitin carboxyl-terminal hydrolase 14	97	2	0.89	10	17	29
G1U0I7	H3BSK9	ATXN2L	Ataxin 2 like	Ataxin-2-like protein (Fragment)	99	2	0.89	5	3	25
G1SZI7		CROT	Carn_acyltransf domain-containing protein			1	0.89	2	2	4
G1SE01	A0A0A0MSI8	EXOC5	Exocyst complex component 5	Exocyst complex component 5	98	2	0.89	4	4	7
G1U3B8	K7ER96	TXNL1	PITH domain-containing protein	Thioredoxin-like protein 1 (Fragment)	100	2	0.89	5	5	30
A0A0A0MQP7	P62736	ACTA2	Actin, aortic smooth muscle	Actin, aortic smooth muscle	100	2	0.89	30	###	71
G1TPB1		CRAT	Carnitine O-acetyltransferase			1	0.89	2	2	8
B7NZD2	Q5VWC4	PSMD4	Proteasome 26S subunit, non-ATPase, 4 (Predicted)	26S proteasome non-ATPase regulatory subunit 4	99	2	0.89	8	19	27
Q28611		UGT1	UDP-glucuronosyltransferase 1-6			1	0.89	10	14	24
G1SE49	Q9UID3	VPS51	Uncharacterized protein	Vacuolar protein sorting-associated protein 51 homolog	96	3	0.89	6	7	15

G1U522	P13861	PRKAR2A	Uncharacterized protein	cAMP-dependent protein kinase type II-alpha regulatory subunit	90	3	0.89	10	14	34
G1SMG1		KANK1	KN motif and ankyrin repeat domains 1			1	0.89	6	7	10
	Q9Y224	RTRAF		RNA transcription, translation and transport factor protein		4	0.89	10	43	50
G1T5H7		TMEM132B	Transmembrane protein 132B			1	0.89	2	2	4
G1SII9	Q5QJ74	TBCEL	Ubiquitin-like domain-containing protein	Tubulin-specific chaperone cofactor E-like protein	99	2	0.89	2	2	8
G1T4X8	P49721	PSMB2	Proteasome subunit beta	Proteasome subunit beta type-2	99	2	0.89	8	44	60
G1SRJ7	P08253	MMP2	72 kDa type IV collagenase	72 kDa type IV collagenase	95	2	0.88	7	9	19
G1SVH0	P23526	AHCY	AdoHcyase_NAD domain-containing protein	Adenosylhomocysteinase	97	2	0.88	15	47	36
G1SJV2	Q7L523	RRAGA	Uncharacterized protein	Ras-related GTP-binding protein A	100	3	0.88	4	9	18
G1SWN1	Q96H20	SNF8	Vacuolar-sorting protein SNF8	Vacuolar-sorting protein SNF8	100	2	0.88	3	3	24
G1SIP1	Q96LJ7	DHRS1	Uncharacterized protein	Dehydrogenase/reductase SDR family member 1	87	3	0.88	6	6	30
G1T4H3	Q15084	PDIA6	Uncharacterized protein	Protein disulfide-isomerase A6	93	3	0.88	17	122	52
G1TBZ5	E9PCX2	AKR1B1	Aldo-keto reductase family 1 member B1	Aldo-keto reductase family 1 member B1	85	2	0.88	6	16	29
G1T4S5	Q8NFW8	CMAS	Uncharacterized protein	N-acylneuraminate cytidylyltransferase	96	3	0.88	5	5	11
	A0A0A0MT60	FKBP15		Peptidylprolyl isomerase (Fragment)		4	0.88	3	3	3
G1TUU3	Q5JXR6	ZNFX1	Uncharacterized protein	NFX1-type zinc finger-containing protein 1	90	3	0.88	2	2	2
G1T3P1	K7EKP8	ACOT7	Uncharacterized protein	Cytosolic acyl coenzyme A thioester hydrolase (Fragment)	98	3	0.88	5	5	23
G1T6P5	Q9NVJ2	ARL8B	ADP ribosylation factor like GTPase 8B	ADP-ribosylation factor-like protein 8B	100	2	0.88	7	33	45
	A0A024RBG1	NUDT4B		Diphosphoinositol polyphosphate phosphohydrolase NUDT4B		4	0.88	2	2	21
G1T2I4	P07814	EPRS	Glutamyl-prolyl-tRNA synthetase	Bifunctional glutamate/proline--tRNA ligase	89	2	0.88	54	69	46
	M0R261	PGLS		6-phosphogluconolactonase (Fragment)		4	0.88	2	4	13
G1T9V4	G3V5Z7	PSMA6	Proteasome subunit alpha type	Proteasome subunit alpha type	97	2	0.88	10	58	47
G1SCF4	G8JLD5	DNM1L	Uncharacterized protein	Dynamin-1-like protein	93	3	0.88	13	29	26
G1SFC5	Q86VS8	HOOK3	Calponin-homology (CH) domain-containing protein	Protein Hook homolog 3	99	2	0.88	14	28	22
G1TSL5	C9J6N9	UFD1	Uncharacterized protein	Ubiquitin recognition factor in ER-associated degradation protein 1 (Fragment)	100	3	0.88	3	3	13
G1TA82	Q12768	WASHC5	Uncharacterized protein	WASH complex subunit 5	97	3	0.88	6	12	9
G1SXL9		XPNPEP3	AMP_N domain-containing protein			1	0.88	2	2	9
G1SV03	Q14165	MLEC	Malectin domain-containing protein	Malectin	95	2	0.88	8	14	32
	J3KR44	OTUB1		Ubiquitin thioesterase		4	0.88	5	7	34
G1SMM1		USP47	USP domain-containing protein			1	0.88	2	3	3
G1U8C4	H0YKK6	PSME1	Uncharacterized protein	Proteasome activator complex subunit 1	98	3	0.88	2	2	27
G1SVA1	F2Z2X4	XPO4	CRM1_C domain-containing protein	Exportin-4	99	2	0.88	5	6	10
G1SCY4	P52907	CAPZA1	F-actin-capping protein subunit alpha	F-actin-capping protein subunit alpha-1	96	2	0.87	10	27	58
G1T1D7	B1ALD9	POSTN	Uncharacterized protein	Periostin	90	3	0.87	16	36	34
G1T0B4	Q9Y3B3	TMED7	GOLD domain-containing protein	Transmembrane emp24 domain-containing protein 7	92	2	0.87	7	19	52
G1T9P1		PEAK1	Pseudopodium enriched atypical kinase 1			1	0.87	3	4	3
G1SKT3	Q9NUJ1	ABHD10	AB hydrolase-1 domain-containing protein	Mycophenolic acid acyl-glucuronide esterase, mitochondrial	88	2	0.87	8	11	36
G1SDW3		CDS2	Phosphatidate cytidylyltransferase			1	0.87	3	4	10
G1THV8	Q9BT09	CNPY3	DUF3456 domain-containing protein	Protein canopy homolog 3	92	2	0.87	6	7	26
G1SI71		PAIP1	MIF4G domain-containing protein			1	0.87	2	3	6
G1TE96	O43813	LANCL1	Uncharacterized protein	Glutathione S-transferase LANCL1	95	3	0.87	4	10	18
G1SY19	O15118	NPC1	SSD domain-containing protein	NPC intracellular cholesterol transporter 1	91	2	0.87	3	4	3
G1SHF1	Q9UHV9	PFDN2	Uncharacterized protein	Prefoldin subunit 2	99	3	0.87	4	8	36
G1T2I7	Q9HCJ1	ANKH	Uncharacterized protein	Progressive ankylosis protein homolog	99	3	0.87	3	5	13
G1U8F0	O95782	AP2A1	AP-2 complex subunit alpha	AP-2 complex subunit alpha-1	98	2	0.87	32	5	46
G1SSV2	Q9UL01	DSE	DUF4962 domain-containing protein	Dermatan-sulfate epimerase	96	2	0.87	8	8	15
G1T726	A0A0A0MSE2	HADH	Uncharacterized protein	Hydroxyacyl-coenzyme A dehydrogenase, mitochondrial	92	3	0.87	10	13	43
G1SEU9	Q9UHA4	LAMTOR3	Uncharacterized protein	Ragulator complex protein LAMTOR3	98	3	0.87	4	6	40
G1TKY3	P17655	CAPN2	Calpain-2 catalytic subunit	Calpain-2 catalytic subunit	94	2	0.87	22	44	49
G1T4Q9	P28074	PSMB5	Proteasome subunit beta	Proteasome subunit beta type-5	98	2	0.87	9	53	47
G1SLZ8	Q06124	PTPN11	Tyrosine-protein phosphatase non-receptor type	Tyrosine-protein phosphatase non-receptor type 11	98	2	0.87	6	6	15
	A0A0A0MRE1	EXOC7		Exocyst complex component 7 (Fragment)		4	0.87	3	6	7
G1TC10	P61081	UBE2M	UBIQUITIN_CONJUGAT_2 domain-containing protein	NEDD8-conjugating enzyme Ubc12	100	2	0.87	7	7	51
G1T373	B1ALA9	PRPS1	Pribosyltran_N domain-containing protein	Ribose-phosphate pyrophosphokinase 1	88	2	0.87	4	13	19
G1T1D9	Q92905	COPS5	MPN domain-containing protein	COP9 signalosome complex subunit 5	100	2	0.87	7	11	31
G1SCP8	P26038	MSN	FERM domain-containing protein	Moesin	94	2	0.87	33	23	59
G1SGX6		SDR39U1	DUF1731 domain-containing protein			1	0.87	2	2	14
	M0QXB5	ETHE1		Persulfide dioxygenase ETHE1, mitochondrial		4	0.87	2	2	14
G1U1M3	Q12904	AIMP1	tRNA-binding domain-containing protein	Aminoacyl tRNA synthase complex-interacting multifunctional protein 1	91	2	0.86	8	14	36
G1TJC8		PEX5	TPR_REGION domain-containing protein			1	0.86	2	2	5

G1TUH9	Q14195	DPYSL3	Amidohydro-rel domain-containing protein	Dihydropyrimidinase-related protein 3	98	2	0.86	17	25	38
	O95747	OXSR1		Serine/threonine-protein kinase OSR1		4	0.86	4	7	12
G1SHK8	O94925	GLS	ANK_REP_REGION domain-containing protein	Glutaminase kidney isoform, mitochondrial	97	2	0.86	21	12	48
G1SJC7	Q9UN86	G3BP2	Uncharacterized protein	Ras GTPase-activating protein-binding protein 2	100	3	0.86	5	6	10
G1SCU8		OTUD6B	OTU domain-containing protein			1	0.86	3	3	19
G1TIZ5	Q8NBS9	TXNDC5	Uncharacterized protein	Thioredoxin domain-containing protein 5	87	3	0.86	13	24	39
G1T7F5	A0A1C7CYY0	ADD2	Aldolase_II domain-containing protein	Beta-adducin (Fragment)	93	2	0.86	2	2	8
G1SWC9	H3BMU1	IST1	Uncharacterized protein	IST1 homolog (Fragment)	100	3	0.86	5	7	21
G1SGB3		ELP1	Elongator complex protein 1			1	0.86	2	3	4
G1TPZ1		LGALS1	Galectin			1	0.86	9	39	75
Q29502	Q13177	PAK2	Serine/threonine-protein kinase PAK 2	Serine/threonine-protein kinase PAK 2	98	2	0.86	8	5	25
G1STQ6	Q9Y617	PSAT1	Phosphoserine aminotransferase	Phosphoserine aminotransferase	94	2	0.86	9	16	22
G1SXB6	B4DVA9	POGLUT1	CAP10 domain-containing protein	Protein O-glucosyltransferase 1	96	2	0.86	3	3	14
U3KMI4	P62834	RAP1A	Uncharacterized protein	Ras-related protein Rap-1A	100	3	0.86	8	2	65
G1T9L2	Q86VN1	VPS36	GLUE N-terminal domain-containing protein	Vacuolar protein-sorting-associated protein 36	97	2	0.86	4	5	18
G1SZ34	Q9Y5S2	CDC42BPB	CDC42 binding protein kinase beta	Serine/threonine-protein kinase MRCK beta	92	2	0.86	10	10	11
G1TE47		COG8	Conserved oligomeric Golgi complex subunit 8			1	0.86	5	6	12
G1TRM4	P62280	RPS11	Ribosomal_S17_N domain-containing protein	40S ribosomal protein S11	100	2	0.86	9	20	55
	Q9UQ13	SHOC2		Leucine-rich repeat protein SHOC-2		4	0.86	2	4	10
G1SXT1	O43865	AHCYL1	AdoHcyase_NAD domain-containing protein	S-adenosylhomocysteine hydrolase-like protein 1	100	2	0.86	3	6	7
G1TE27	X6R9L0	DNAJC3	Uncharacterized protein	DnaJ homolog subfamily C member 3	88	3	0.86	13	4	33
G1SK00		USP5	Ubiquitin carboxyl-terminal hydrolase			1	0.86	21	39	38
G1U4C2	Q76M96	CCDC80	Uncharacterized protein	Coiled-coil domain-containing protein 80	86	3	0.86	5	5	6
G1T369	C9J8R4	DCUN1D1	DCN1-like protein	DCN1-like protein (Fragment)	100	2	0.86	2	3	13
G1U0B3	P53007	SLC25A1	Solute carrier family 25 member 1	Tricarboxylate transport protein, mitochondrial	86	2	0.86	10	27	48
	A0A096LP07	GPS1		COP9 signalosome complex subunit 1		4	0.85	4	4	10
G1SPA6	Q32P28	P3H1	Fe2OG dioxygenase domain-containing protein	Prolyl 3-hydroxylase 1	91	2	0.85	24	47	52
G1SMI4	Q15276	RABEP1	Uncharacterized protein	Rab GTPase-binding effector protein 1	96	3	0.85	2	3	4
B7NZJ1		CPNE1	Copine I, isoform 8 (Predicted)			1	0.85	3	5	11
G1TKE0	Q9P2X0	DPM3	Dolichol-phosphate mannosyltransferase subunit 3	Dolichol-phosphate mannosyltransferase subunit 3	95	2	0.85	2	2	24
	Q9Y6I3	EPN1		Epsin-1		4	0.85	2	3	4
G1SNM1	P41250	GARS	Uncharacterized protein	Glycine--tRNA ligase	95	3	0.85	22	42	39
G1U6Y3		MEAK7	MTOR associated protein, eak-7 homolog			1	0.85	3	4	16
G1T182	Q86UE4	MTDH	Metadherin	Protein LYRIC	88	2	0.85	4	6	18
	Q8N122	RPTOR		Regulatory-associated protein of mTOR		4	0.85	2	2	3
	A0A0A0MSZ1	MARK3		Non-specific serine/threonine protein kinase		4	0.85	2	2	4
G1SCE6	Q9Y223	GNE	Epimerase_2 domain-containing protein	Bifunctional UDP-N-acetylglucosamine 2-epimerase/N-acetylmannosamine kinase	100	2	0.85	10	25	27
G1T4N4	A0A0B4J210	LARP1	HTH La-type RNA-binding domain-containing protein	La-related protein 1 (Fragment)	93	2	0.85	5	11	8
G1SFH8		PDCL3	Phosducin domain-containing protein			1	0.85	2	3	11
G1TEU8		TBCC	C-CAP/cofactor C-like domain-containing protein			1	0.85	2	2	7
G1T974	A0A3B3IRK6	MOGS	Mannosyl-oligosaccharide glucosidase	Mannosyl-oligosaccharide glucosidase (Fragment)	86	2	0.85	9	12	20
G1TXS2	A0A0D9SFK2	MYO18A	Uncharacterized protein	Unconventional myosin-XVIIIa	94	3	0.85	10	12	7
P68105	P68104	EEF1A1	Elongation factor 1-alpha 1	Elongation factor 1-alpha 1	100	2	0.85	22	864	65
G1SM65		GALNT16	Polypeptide N-acetylgalactosaminyltransferase			1	0.85	3	8	16
G1SZ15	A8K878	MANF	Mesencephalic astrocyte derived neurotrophic factor	Mesencephalic astrocyte-derived neurotrophic factor	97	2	0.85	6	16	30
G1T5I3	A0A0C4DFM1	TM9SF4	Transmembrane 9 superfamily member	Transmembrane 9 superfamily member	100	2	0.85	4	7	10
G1U6H0	P27824	CANX	Uncharacterized protein	Calnexin	95	3	0.85	16	57	30
G1U3S6	O15258	RER1	Protein RER1	Protein RER1	95	2	0.85	2	4	13
G1TPN2	K7EQA9	CDC37	Cell division cycle 37	Hsp90 co-chaperone Cdc37 (Fragment)	57	2	0.85	5	2	17
G1STX3	Q8WVM8	SCFD1	Uncharacterized protein	Sec1 family domain-containing protein 1	96	3	0.85	16	27	42
G1SL02	P63010	AP2B1	AP complex subunit beta	AP-2 complex subunit beta	98	2	0.85	41	63	57
G1SDG2	P48739	PITPNB	Phosphatidylinositol transfer protein beta	Phosphatidylinositol transfer protein beta isoform	97	2	0.85	4	4	14
G1U7C5	Q9P2E9	RRBP1	Uncharacterized protein	Ribosome-binding protein 1	83	3	0.85	44	105	38
G1TKL0	M0QZG7	SNRPA	Small nuclear ribonucleoprotein polypeptide A	U1 small nuclear ribonucleoprotein A (Fragment)	60	2	0.85	4	3	14
G1SZ72	P51116	FXR2	Uncharacterized protein	Fragile X mental retardation syndrome-related protein 2	98	3	0.85	8	7	20
Q28647		PPP2R5B	Serine/threonine-protein phosphatase 2A 56 kDa regulatory subunit beta isoform			1	0.85	2	2	7
G1U207	Q86Y82	STX12	t-SNARE coiled-coil homology domain-containing protein	Syntaxin-12	96	2	0.85	10	17	55
P15253	P27797	CALR	Calreticulin	Calreticulin	96	2	0.85	21	587	67
G1T302		THYN1	EVE domain-containing protein			1	0.85	2	5	10
G1SPZ2	Q13107	USP4	Ubiquitin carboxyl-terminal hydrolase	Ubiquitin carboxyl-terminal hydrolase 4	89	2	0.85	3	8	7

G1TR97	A0A0A0MSA7	EIF4G3	Uncharacterized protein	Eukaryotic translation initiation factor 4 gamma 3	89	3	0.84	8	11	8
G1SZ14	P25788	PSMA3	Proteasome endopeptidase complex	Proteasome subunit alpha type-3	99	2	0.84	8	19	26
G1SZU0		URB2	Urb2 domain-containing protein			1	0.84	2	2	2
G1U684	A0A087X1W8	CADM1	Uncharacterized protein	Cell adhesion molecule 1	95	3	0.84	9	17	34
G1T4H6		MTMR6	Myotubularin phosphatase domain-containing protein			1	0.84	3	2	4
G1SM51	Q9UNM6	PSMD13	Proteasome 26S subunit, non-ATPase 13	26S proteasome non-ATPase regulatory subunit 13	92	2	0.84	15	10	46
G1T748	Q5VZU9	TPP2	Uncharacterized protein	Tripeptidyl-peptidase 2	97	3	0.84	26	34	25
G1TSV3	Q8ND76	CCNY	Cyclin Y	Cyclin-Y	99	2	0.84	2	3	9
G1SQJ2		FOCAD	DUF3730 domain-containing protein			1	0.84	8	9	7
G1SXL6	O94915	FRYL	Uncharacterized protein	Protein furry homolog-like	97	3	0.84	4	6	2
G1TD98		GSR	Glutathione reductase			1	0.84	2	4	7
	P10301	RRAS		Ras-related protein R-Ras		4	0.84	6	6	38
G1T0H7	A0A087WWM0	TRAPPC3	Trafficking protein particle complex subunit	Trafficking protein particle complex subunit	94	2	0.84	4	7	27
G1SQN6	J3KN59	BNIP2	CRAL-TRIO domain-containing protein	BCL2/adenovirus E1B 19 kDa protein-interacting protein 2	94	2	0.84	2	3	11
G1SPL1	A0A286YF22	PHGDH	D-3-phosphoglycerate dehydrogenase	D-3-phosphoglycerate dehydrogenase	93	2	0.84	26	102	61
G1TJS2	Q8IV08	PLD3	Phospholipase D family member 3	Phospholipase D3	89	2	0.84	5	3	18
G1T3Z6	P31323	PRKAR2B	Uncharacterized protein	cAMP-dependent protein kinase type II-beta regulatory subunit	97	3	0.84	7	8	27
G1SZW0	Q7L576	CYFIP1	Cytoplasmic FMR1-interacting protein	Cytoplasmic FMR1-interacting protein 1	98	2	0.84	18	30	17
G1SEV2	P30101	PDIA3	Protein disulfide-isomerase	Protein disulfide-isomerase A3	96	2	0.84	30	226	45
G1TCZ8	P30153	PPP2R1A	Protein phosphatase 2 scaffold subunit Aalpha	Serine/threonine-protein phosphatase 2A 65 kDa regulatory subunit A alpha isoform	90	2	0.84	15	3	37
G1TB18	Q07960	ARHGAP1	Uncharacterized protein	Rho GTPase-activating protein 1	95	3	0.84	5	6	16
G1SH26	F6TLX2	GLOD4	Glyoxalase domain containing 4	Glyoxalase domain-containing protein 4	81	2	0.84	7	11	23
G1SV13	P22314	UBA1	Ubiquitin-like modifier-activating enzyme 1	Ubiquitin-like modifier-activating enzyme 1	97	2	0.84	30	75	48
	H0Y7A7	CALM2		Calmodulin-2 (Fragment)		4	0.84	7	62	49
A0A140TAW0	O43852	CALU	Calumenin	Calumenin	99	2	0.84	15	87	64
G1SH09	Q5VIR6	VPS53	VPS53, GARP complex subunit	Vacuolar protein sorting-associated protein 53 homolog	95	2	0.84	4	8	11
G1SP22	Q99943	AGPAT1	1-acyl-sn-glycerol-3-phosphate acyltransferase	1-acyl-sn-glycerol-3-phosphate acyltransferase alpha	98	2	0.83	3	5	13
G1SHV9	P49720	PSMB3	Proteasome subunit beta	Proteasome subunit beta type-3	99	2	0.83	6	16	39
G1SWY3	A0A1B0GUZ7	EFR3A	Uncharacterized protein	Protein EFR3 homolog A	98	3	0.83	2	2	5
G1TZV3	E7ENJ6	AP1M1	Adaptor related protein complex 1 subunit mu 1	AP-1 complex subunit mu-1	75	2	0.83	8	13	33
G1SDU5	H3BPE1	MACF1	Uncharacterized protein	Microtubule-actin cross-linking factor 1, isoforms 1/2/3/5	88	3	0.83	203	174	38
G1TXZ1		RIC1	RIC1 domain-containing protein			1	0.83	2	2	2
	Q14318	FKBP8		Peptidyl-prolyl cis-trans isomerase FKBP8		4	0.83	3	7	15
G1SFQ7		TUBG1	Tubulin gamma chain			1	0.83	5	7	17
	K7ELL7	PRKCSH		Glucosidase 2 subunit beta		4	0.83	2	5	4
G1SXG8	F8VU90	FKBP11	Peptidylprolyl isomerase	Peptidylprolyl isomerase	95	2	0.83	4	10	31
G1TVU4	Q96D15	RCN3	Reticulocalbin 3	Reticulocalbin-3	78	2	0.83	9	39	51
G1SDU1	Q96A49	SYAP1	BSD domain-containing protein	Synapse-associated protein 1	89	2	0.83	2	5	14
G1SYV9	Q9Y490	TLN1	Uncharacterized protein	Talin-1	99	3	0.83	108	46	61
G1SXI1		TTC27	TPR_REGION domain-containing protein			1	0.83	3	4	7
G1SP48	O60701	UGDH	UDP-glucose 6-dehydrogenase	UDP-glucose 6-dehydrogenase	96	2	0.83	17	4	51
G1SN22	Q9Y5K6	CD2AP	Uncharacterized protein	CD2-associated protein	91	3	0.83	2	2	5
G1SQL0	P62191	PSMC1	AAA domain-containing protein	26S proteasome regulatory subunit 4	100	2	0.83	17	34	43
G1SV40	P83436	COG7	Uncharacterized protein	Conserved oligomeric Golgi complex subunit 7	93	3	0.83	5	7	10
G1SM91		FAH	Fumarylacetoacetase			1	0.83	2	2	7
G1SST9	P54727	RAD23B	Uncharacterized protein	UV excision repair protein RAD23 homolog B	94	3	0.83	12	18	42
G1T719		TSR1	Bms1-type G domain-containing protein			1	0.83	2	4	4
G1SK52	Q5T6H7	XPNPEP1	Uncharacterized protein	Xaa-Pro aminopeptidase 1	97	3	0.83	10	13	25
G1TBL6	A0A087WVQ6	CLTC	Clathrin heavy chain	Clathrin heavy chain	99	2	0.82	80	579	63
G1SJK7		CYP27A1	Sterol 26-hydroxylase, mitochondrial			1	0.82	2	5	8
G1SGY2	H0YC15	PTPN12	Tyrosine-protein phosphatase non-receptor type 12	Tyrosine-protein phosphatase non-receptor type 12 (Fragment)	90	2	0.82	2	3	21
G1SLF1		ADH5	S-(hydroxymethyl)glutathione dehydrogenase			1	0.82	4	6	12
G1SSZ1	Q01658	DR1	CBFD_NFYB_HMF domain-containing protein	Protein Dr1	100	2	0.82	2	3	21
G1SHI2		MYD88	Myeloid differentiation primary response protein MyD88			1	0.82	2	2	9
G1T706	O00442	RTCA	Uncharacterized protein	RNA 3~-terminal phosphate cyclase	96	3	0.82	5	6	19
G1SQ80	Q8TDZ2	MICAL1	Uncharacterized protein	[F-actin]-monooxygenase MICAL1	83	3	0.82	4	4	8
G1SN17		PPP4R1	WRNPLPNID domain-containing protein			1	0.82	4	3	8
G1SXH7	P12931	SRC	Tyrosine-protein kinase	Proto-oncogene tyrosine-protein kinase Src	96	2	0.82	5	6	14
G1TI71	A0A087WYS1	UGP2	UTP--glucose-1-phosphate uridylyltransferase	UTP--glucose-1-phosphate uridylyltransferase	99	2	0.82	24	68	62
G1T147	Q8IZ52	CHPF	Hexosyltransferase	Chondroitin sulfate synthase 2	95	2	0.82	3	3	12

G1T860	M0R0P8	MYO9B	Myosin IXB	Unconventional myosin-IXb	83	2	0.82	13	13	13
G1SWM8	A0A0U1RQQ9	SCYL2	Protein kinase domain-containing protein	SCY1-like protein 2	94	2	0.82	2	2	6
G1TQJ4	Q01433	AMPD2	AMP deaminase	AMP deaminase 2	97	2	0.82	3	3	5
U3KM64		CLTA	Clathrin light chain			1	0.82	7	28	28
G1SH25	P40616	ARL1	Uncharacterized protein	ADP-ribosylation factor-like protein 1	99	3	0.82	5	16	38
G1SK48	K7ES02	BLMH	Bleomycin hydrolase	Bleomycin hydrolase (Fragment)	92	2	0.82	8	13	26
G1SJR4	Q15363	TMED2	Transmembrane p24 trafficking protein 2	Transmembrane emp24 domain-containing protein 2	99	2	0.82	8	31	64
G1SJ72		CSPG4	Chondroitin sulfate proteoglycan 4			1	0.82	19	28	17
G1SJS2	Q14232	EIF2B1	Uncharacterized protein	Translation initiation factor eIF-2B subunit alpha	96	3	0.82	4	11	31
G1T967	A0A0U1RRB6	EXOC6B	Exocyst complex component	Exocyst complex component	99	2	0.82	7	5	15
G1T7Q2		LOXL2	Lysyl oxidase like 2			1	0.82	6	6	9
G1SRB7	A0A2R8Y7U1	TPP1	Peptidase S53 domain-containing protein	Tripeptidyl-peptidase 1 (Fragment)	93	2	0.82	7	16	23
G1T276		ALDH3A2	Aldehyde dehydrogenase			1	0.82	7	10	21
G1U1E5	O60826	CCDC22	Coiled-coil domain containing 22	Coiled-coil domain-containing protein 22	75	2	0.82	6	8	12
G1U1H1	Q00535	CDK5	Protein kinase domain-containing protein	Cyclin-dependent-like kinase 5	100	2	0.82	3	2	12
G1SJZ4	J3KQ32	OLA1	Obg-like ATPase 1	Obg-like ATPase 1	100	2	0.82	9	16	34
G1T8P7	A0A2R8YFH5	SEC23B	Protein transport protein SEC23	Protein transport protein SEC23	95	2	0.82	8	9	15
G1SCZ9	Q92575	UBXN4	UBX domain-containing protein	UBX domain-containing protein 4	94	2	0.82	4	7	14
	P55010	EIF5		Eukaryotic translation initiation factor 5		4	0.82	5	8	15
G1SDR2	P24844	MYL9	Uncharacterized protein	Myosin regulatory light polypeptide 9	99	3	0.82	10	20	80
G1T7I0	Q96PU5	NEDD4L	E3 ubiquitin-protein ligase	E3 ubiquitin-protein ligase NEDD4-like	96	2	0.82	3	4	5
G1U2M9		SAMD9	Sterile alpha motif domain containing 9			1	0.82	2	2	2
G1SDQ5	Q96JG6	VPS50	Uncharacterized protein	Syndetin	98	3	0.82	4	8	9
G1TDC3	Q14697	GANAB	Gal_mutarotas_2 domain-containing protein	Neutral alpha-glucosidase AB	91	2	0.82	30	247	46
	E7EVJ3	NDST1		Bifunctional heparan sulfate N-deacetylase/N-sulfotransferase 1		4	0.82	2	2	3
G1SJ41	Q9Y6Y8	SEC23IP	DDHD domain-containing protein	SEC23-interacting protein	89	2	0.82	14	21	16
G1T4H0	Q9NYL9	TMOD3	Uncharacterized protein	Tropomodulin-3	93	3	0.82	15	30	50
G1SCR0	H0YFI1	LAMTOR1	Uncharacterized protein	Ragulator complex protein LAMTOR1 (Fragment)	100	3	0.81	2	4	47
G1T4Z0	Q96PY5	FMNL2	Formin like 2	Formin-like protein 2	97	2	0.81	7	4	8
G1TQV4	F6SKB8	NECAP2	DUF1681 domain-containing protein	Adaptin ear-binding coat-associated protein 2	95	2	0.81	2	2	8
G1SQ96	Q5H9R7	PPP6R3	Uncharacterized protein	Serine/threonine-protein phosphatase 6 regulatory subunit 3	92	3	0.81	7	8	13
G1SPN9	P36405	ARL3	Uncharacterized protein	ADP-ribosylation factor-like protein 3	99	3	0.81	2	3	9
G1SE51	A0A087X2D8	SPAG9	Sperm associated antigen 9	C-Jun-amino-terminal kinase-interacting protein 4	93	2	0.81	10	20	13
G1SEH1		PLSCR3	Phospholipid scramblase			1	0.81	2	6	13
G1SSN2		SIRT5	NAD-dependent protein deacylase sirtuin-5, mitochondrial			1	0.81	2	2	10
G1SZE0	Q96QK1	VPS35	Vacuolar protein sorting-associated protein 35	Vacuolar protein sorting-associated protein 35	100	2	0.81	22	48	37
G1T3Z2	Q9UNZ2	NSFL1C	Uncharacterized protein	NSFL1 cofactor p47	97	3	0.81	5	7	19
G1SMP3	A0A2R8Y6F8	CASK	Uncharacterized protein	Peripheral plasma membrane protein CASK	97	3	0.81	12	15	17
G1TEU5	O75131	CPNE3	Uncharacterized protein	Copine-3	96	3	0.81	3	3	7
G1SPB8	Q99733	NAP1L4	Uncharacterized protein	Nucleosome assembly protein 1-like 4	93	3	0.81	9	16	40
G1SX32	O00743	PPP6C	Serine/threonine-protein phosphatase	Serine/threonine-protein phosphatase 6 catalytic subunit	100	2	0.81	2	2	16
G1T365	Q9H269	VPS16	Vacuolar protein sorting-associated protein 16 homolog	Vacuolar protein sorting-associated protein 16 homolog	98	2	0.81	6	9	15
G1SL07	Q9NUP9	LIN7C	Protein lin-7 homolog	Protein lin-7 homolog C	99	2	0.81	5	4	29
G1T845	I3L0N3	NSF	Uncharacterized protein	Vesicle-fusing ATPase	99	3	0.81	17	31	33
G1T0S0	Q8NBN3	TMEM87A	Uncharacterized protein	Transmembrane protein 87A	96	3	0.81	4	7	9
G1U2Q7		COL8A1	Collagen alpha-1(VIII) chain			1	0.81	3	4	5
G1SHX1	P68036	UBE2L3	Ubiquitin conjugating enzyme E2 L3	Ubiquitin-conjugating enzyme E2 L3	96	2	0.81	6	12	57
G1SDD2	B7Z2Y2	COG2	Uncharacterized protein	Conserved oligomeric Golgi complex subunit 2	91	3	0.81	6	11	15
G1TM86	A2AB27	GNL1	G protein nucleolar 1 (putative)	Guanine nucleotide-binding protein-like 1 (Fragment)	98	2	0.81	2	4	5
G1SU71	P20618	PSMB1	Proteasome subunit beta	Proteasome subunit beta type-1	94	2	0.81	8	11	42
G1T0Z6	Q9Y4X5	ARIH1	RBR-type E3 ubiquitin transferase	E3 ubiquitin-protein ligase ARIH1	98	2	0.80	3	3	10
	Q9UJY5	GGA1		ADP-ribosylation factor-binding protein GGA1		4	0.80	3	5	9
G1T090	G3V180	DPP3	Dipeptidyl peptidase 3	Dipeptidyl peptidase 3	94	2	0.80	9	19	24
G1SMP6	A0A1B0GVV3	RILPL1	Rab interacting lysosomal protein like 1	RILP-like protein 1	93	2	0.80	4	6	16
G1T670		SORT1	Proteasome subunit alpha type			1	0.80	10	20	48
G1TDI6		BLOC1S6	Biogenesis of lysosome-related organelles complex 1 subunit 6			1	0.80	2	2	25
G1SF26	P53675	CLTCL1	Clathrin heavy chain	Clathrin heavy chain 2	91	2	0.80	17	6	13
G1TS93	Q15435	PPP1R7	LRRcap domain-containing protein	Protein phosphatase 1 regulatory subunit 7	95	2	0.80	10	9	46
G1TDU0	P33176	KIF5B	Kinesin-like protein	Kinesin-1 heavy chain	99	2	0.80	28	5	41
G1T466	A0AVT1	UBA6	UBA_e1_C domain-containing protein	Ubiquitin-like modifier-activating enzyme 6	93	2	0.80	7	6	10

G1TDH8		CLCN5	Chloride channel protein			1	0.80	4	6	13
	Q8TBX8	PIP4K2C		Phosphatidylinositol 5-phosphate 4-kinase type-2 gamma		4	0.80	4	4	18
	O14908	GIPC1		PDZ domain-containing protein GIPC1		4	0.80	4	6	16
A7X8X3		HPRT	Hypoxanthine phosphoribosyltransferase			1	0.80	6	7	31
G1T2M9	P09486	SPARC	SPARC	SPARC	94	2	0.80	13	29	45
G1SL38	F8VS81	TWF1	Twinfilin actin binding protein 1	Twinfilin-1 (Fragment)	96	2	0.80	7	14	25
G1STX4	P61011	SRP54	Signal recognition particle 54 kDa protein	Signal recognition particle 54 kDa protein	99	2	0.80	13	19	38
G1SJQ2	Q92896	GLG1	Uncharacterized protein	Golgi apparatus protein 1	97	3	0.80	43	75	42
G1SL53	Q66K14	TBC1D9B	Uncharacterized protein	TBC1 domain family member 9B	89	3	0.80	3	2	3
Q28685	Q14118	DAG1	Dystroglycan	Dystroglycan	94	2	0.80	3	4	4
G1SKS0	Q92747	ARPC1A	Actin-related protein 2/3 complex subunit	Actin-related protein 2/3 complex subunit 1A	100	2	0.79	3	5	13
G1TKE3	X6RJP6	TAGLN2	Transgelin	Transgelin-2 (Fragment)	77	2	0.79	6	12	40
G1SYB4	P60953	CDC42	Uncharacterized protein	Cell division control protein 42 homolog	100	3	0.79	7	27	49
G1SWK5	A0A1W2PNP0	PIGT	Uncharacterized protein	GPI transamidase component PIG-T (Fragment)	86	3	0.79	3	5	8
G1TBT4	Q9HB90	RRAGC	Uncharacterized protein	Ras-related GTP-binding protein C	98	3	0.79	5	10	23
G1SGD9		ATG7	Ubiquitin-like modifier-activating enzyme ATG7			1	0.79	2	2	6
G1T044	A0A3B3IUC4	GLA	Alpha-galactosidase	Alpha-galactosidase	75	2	0.79	3	3	7
	O14964	HGS		Hepatocyte growth factor-regulated tyrosine kinase substrate		4	0.79	5	8	13
G1SFU4	Q9UIQ6	LNPEP	Uncharacterized protein	Leucyl-cystinyl aminopeptidase	90	3	0.79	14	19	17
G1SKF1	P07996	THBS1	Uncharacterized protein	Thrombospondin-1	97	3	0.79	34	75	37
P15541		ANPEP	Aminopeptidase N			1	0.79	11	13	19
G1SI94		CRLF3	Cytokine receptor like factor 3			1	0.79	2	3	14
G1SH63		GSS	Glutathione synthetase			1	0.79	2	2	6
G1T918	P28070	PSMB4	Proteasome subunit beta	Proteasome subunit beta type-4	95	2	0.79	8	38	49
G1T634	Q9Y5X1	SNX9	Sorting nexin	Sorting nexin-9	89	2	0.79	7	9	23
G1SHS8	A0A087WY55	VTA1	Uncharacterized protein	Chromosome 6 open reading frame 55, isoform CRA_b	87	3	0.79	3	5	15
G1T8H8	P08183	ABCB1	Uncharacterized protein	ATP-dependent translocase ABCB1	88	3	0.79	5	15	7
G1TJA8		FNBP1	Formin binding protein 1			1	0.79	3	2	4
G1SZR7	Q86UP2	KTN1	Uncharacterized protein	Kinectin	90	3	0.79	46	85	40
G1T0Z8	Q99471	PFDN5	Uncharacterized protein	Prefoldin subunit 5	99	3	0.79	4	6	44
U3KN73	Q9UBQ0	VPS29	Vacuolar protein sorting-associated protein 29	Vacuolar protein sorting-associated protein 29	100	2	0.79	6	10	37
G1T9H0	Q8NCA5	FAM98A	Uncharacterized protein	Protein FAM98A	92	3	0.79	8	12	22
	Q63ZY3	KANK2		KN motif and ankyrin repeat domain-containing protein 2		4	0.79	9	7	13
U3KMU7	A0A024R571	EHD1	EH domain containing 1	EH domain-containing protein 1	99	2	0.79	15	14	35
G1T964		TMED1	GOLD domain-containing protein			1	0.79	3	6	27
G1SNI2	Q12841	FSTL1	Kazal-like domain-containing protein	Follistatin-related protein 1	94	2	0.79	8	22	32
G1SS73	Q92499	DDX1	Uncharacterized protein	ATP-dependent RNA helicase DDX1	98	3	0.78	20	43	37
G1SET5		CEMIP2	G8 domain-containing protein			1	0.78	4	3	5
G1SPV8	O94829	IPO13	Importin N-terminal domain-containing protein	Importin-13	100	2	0.78	2	2	3
G1SMT7		NHLRC2	Thioredoxin domain-containing protein			1	0.78	3	2	9
G1TRI7	A0A0C4DG51	PNPLA8	Calcium-independent phospholipase A2-gamma	Calcium-independent phospholipase A2-gamma (Fragment)	90	2	0.78	2	2	4
G1SRF5	Q9UPN7	PPP6R1	Protein phosphatase 6 regulatory subunit 1	Serine/threonine-protein phosphatase 6 regulatory subunit 1	84	2	0.78	4	5	9
G1SCP0	O00203	AP3B1	AP-3 complex subunit beta	AP-3 complex subunit beta-1	88	2	0.78	13	17	16
G1TT64	Q68EM7	ARHGAP17	Uncharacterized protein	Rho GTPase-activating protein 17	91	3	0.78	4	5	8
G1T188	Q9UIJ7	AK3	GTP:AMP phosphotransferase AK3, mitochondrial	GTP:AMP phosphotransferase AK3, mitochondrial	93	2	0.78	11	21	57
G1SVZ8		C9orf64	Queuosine salvage protein			1	0.78	2	2	7
G1SMU8	Q9NQP4	PFDN4	Prefoldin subunit 4	Prefoldin subunit 4	99	2	0.78	2	3	20
G1T2L1	A0A024RA52	PSMA2	Proteasome subunit alpha type	Proteasome subunit alpha type	100	2	0.78	11	36	59
G1SJX5	A0A087WTF3	ANK3	Ankyrin 3	Ankyrin-3 (Fragment)	93	2	0.78	3	6	4
G1SR93	B8ZZA2	FAM126A	Uncharacterized protein	Hyccin	98	3	0.78	2	2	6
G1T4M1		LAMC1	Laminin subunit gamma 1			1	0.78	4	10	9
P51662	P04083	ANXA1	Annexin A1	Annexin A1	91	2	0.78	20	22	61
G1TB71	Q14008	CKAP5	Uncharacterized protein	Cytoskeleton-associated protein 5	97	3	0.78	18	26	15
G1TPW2		MRI1	Methylthioribose-1-phosphate isomerase			1	0.78	4	5	22
G1TPY7		SUMF2	FGE-sulfatase domain-containing protein			1	0.78	3	3	13
G1T107	P61088	UBE2N	UBIQUITIN_CONJUGAT_2 domain-containing protein	Ubiquitin-conjugating enzyme E2 N	100	2	0.78	5	12	59
G1T6S6		ATP6V1F	V-type proton ATPase subunit F			1	0.78	4	12	54
G1SSL8		HEATR5A	HEAT repeat containing 5A			1	0.78	4	3	3
G1SV22	P30086	PEBP1	Phosphatidylethanolamine-binding protein 1	Phosphatidylethanolamine-binding protein 1	89	2	0.78	5	8	37
G1U723		PGER5	3alpha/17beta/20alpha-hydroxysteroid dehydrogenase			1	0.78	5	5	20

G1T9Y4	E5RIU9	CHMP7	Uncharacterized protein	Charged multivesicular body protein 7 (Fragment)	69	3	0.78	2	3	9
G1U9C1	P50570	DNM2	Dynamin 2	Dynamin-2	96	2	0.78	24	7	36
G1T5H8	E7EX17	EIF4B	RRM domain-containing protein	Eukaryotic translation initiation factor 4B	93	2	0.78	3	8	10
G1SLS3	O43747	AP1G1	AP-1 complex subunit gamma	AP-1 complex subunit gamma-1	100	2	0.77	8	13	16
G1TQD3	Q01968	OCRL	Rho-GAP domain-containing protein	Inositol polyphosphate 5-phosphatase OCRL	95	2	0.77	3	4	5
G1TEJ4	Q86VW0	SESTD1	SEC14 and spectrin domain containing 1	SEC14 domain and spectrin repeat-containing protein 1	95	2	0.77	2	6	8
G1U7Q6	H7C1D4	TSN	Uncharacterized protein	Translin (Fragment)	99	3	0.77	3	49	22
G1SIA6	Q5T2E6	ARMH3	DUF1741 domain-containing protein	Armadillo-like helical domain-containing protein 3	99	2	0.77	4	5	11
G1TM35	Q9Y3D6	FIS1	Fission, mitochondrial 1	Mitochondrial fission 1 protein	86	2	0.77	4	5	15
P35566		MARCKSL1	MARCKS-related protein			1	0.77	2	2	25
G1SQ30		POMGNT2	Fibronectin type-III domain-containing protein			1	0.77	2	6	6
G1T6C0	P61020	RAB5B	Uncharacterized protein	Ras-related protein Rab-5B	100	3	0.77	9	39	56
G1SCL6	A0A494C1J1	SPECC1L	Calponin-homology (CH) domain-containing protein	Cytospin-A	90	2	0.77	12	15	15
G1T087	Q93034	CUL5	Cullin-5	Cullin-5	100	2	0.77	4	15	10
G1T0N5	Q00577	PURA	Purine rich element binding protein A	Transcriptional activator protein Pur-alpha	91	2	0.77	7	14	40
G1SW24	P49588	AARS	AA_TRNA_LIGASE_II_ALA domain-containing protein	Alanine--tRNA ligase, cytoplasmic	93	2	0.77	33	25	47
G1TB49		APOA1BP	NAD(P)H-hydrate epimerase			1	0.77	2	5	15
G1T4T7	Q9Y2A7	NCKAP1	Uncharacterized protein	Nck-associated protein 1	100	3	0.77	15	25	21
G1SWK8	Q5TBG5	PSMB7	Proteasome subunit beta	Proteasome subunit beta (Fragment)	84	2	0.77	6	63	25
G1SKS9	A0A087WSW9	TXNRD1	Glutaredoxin domain-containing protein	Thioredoxin reductase 1, cytoplasmic	94	2	0.77	10	10	27
G1SHI0	H0Y4D4	ACAA1	Acetyl-CoA acyltransferase 1	3-ketoacyl-CoA thiolase, peroxisomal (Fragment)	62	2	0.77	8	8	34
G1TER0	Q9NV70	EXOC1	Sec3-PIP2_bind domain-containing protein	Exocyst complex component 1	98	2	0.77	6	9	14
G1SVG6	P21359	NF1	Uncharacterized protein	Neurofibromin	99	3	0.77	4	7	3
G1TT75	O00264	PGRMC1	Cytochrome b5 heme-binding domain-containing protein	Membrane-associated progesterone receptor component 1	93	2	0.77	7	10	29
G1TF32	F5H6I7	ATL3	Atlastin GTPase 3	Atlastin-3	95	2	0.77	15	52	44
G1SZ19	Q9Y3L5	RAP2C	Uncharacterized protein	Ras-related protein Rap-2c	100	3	0.77	6	3	38
G1SPB4		HYAL1	Hyaluronidase			1	0.77	2	2	8
G1STP3	O75718	CRTAP	Uncharacterized protein	Cartilage-associated protein	94	3	0.77	11	14	32
G1TB98	Q15293	RCN1	Uncharacterized protein	Reticulocalbin-1	85	3	0.77	12	9	45
G1T6D1	P62829	RPL23	Uncharacterized protein	60S ribosomal protein L23	100	3	0.77	6	14	57
G1T1R9	Q96A65	EXOC4	Sec8_exocyst domain-containing protein	Exocyst complex component 4	95	2	0.77	11	15	19
G1T5Y1	P28482	MAPK1	Mitogen-activated protein kinase	Mitogen-activated protein kinase 1	98	2	0.77	11	6	44
G1SZM0	Q9BRT3	MIEN1	Uncharacterized protein	Migration and invasion enhancer 1	95	3	0.77	2	2	16
G1SMX7	O43731	KDELR3	ER lumen protein-retaining receptor	ER lumen protein-retaining receptor 3	98	2	0.77	3	2	17
G1T2N2		LTN1	RING-type domain-containing protein			1	0.77	4	3	4
G1TCP2	A0A087X1E4	ARFIP2	AH domain-containing protein	Arfaptin-2	91	2	0.76	2	5	9
G1TIW9		FBXO6	F-box protein 6			1	0.76	3	3	11
G1SZJ5	O15212	PFDN6	Uncharacterized protein	Prefoldin subunit 6	100	3	0.76	5	7	38
G1SQU0	P15586	GNS	N-acetylglucosamine-6-sulfatase	N-acetylglucosamine-6-sulfatase	95	2	0.76	10	16	19
G1SX73	E5RGS4	PFDN1	Uncharacterized protein	Prefoldin subunit 1	88	3	0.76	4	7	30
G1TBJ8		RB1CC1	RB1 inducible coiled-coil 1			1	0.76	3	3	3
G1SCT1	A0A499FJL1	PREP	Uncharacterized protein	Prolyl endopeptidase	96	3	0.76	19	37	41
G1TR92		SYDE1	Synapse defective Rho GTPase homolog 1			1	0.76	3	4	7
G1SVW7	G3V1U5	GOLT1B	Uncharacterized protein	Golgi transport 1 homolog B (S. cerevisiae), isoform CRA_c	100	3	0.76	3	27	17
G1TEM7	D6RA82	ANXA3	Annexin	Annexin	93	2	0.76	13	20	51
G1T9A1	Q96J02	ITCH	E3 ubiquitin-protein ligase	E3 ubiquitin-protein ligase Itchy homolog	93	2	0.76	2	4	4
G1T578	E9PHY0	ACP2	Uncharacterized protein	Lysosomal acid phosphatase	94	3	0.76	4	7	14
G1SHM2		CLPTM1L	CLPTM1 like			1	0.76	5	5	22
G1SL52	A0A0A0MS45	COG4	Cog4 domain-containing protein	Conserved oligomeric Golgi complex subunit 4	94	2	0.76	10	15	19
G1T9Q5		SLC44A2	Solute carrier family 44 member 2			1	0.76	3	4	6
G1SUF5	Q8TF66	LRRC15	LRRCT domain-containing protein	Leucine-rich repeat-containing protein 15	90	2	0.76	6	11	18
G1U886	A0A2R8YGH5	AP1S1	AP complex subunit sigma	AP complex subunit sigma	100	2	0.76	4	4	33
G1T3V0	A0A024R4E5	HDLBP	Uncharacterized protein	High density lipoprotein binding protein (Vigilin), isoform CRA_a	97	3	0.76	45	109	47
G1SMX4	A0A0G2JH68	DIAPH1	Uncharacterized protein	Protein diaphanous homolog 1	90	3	0.76	15	27	22
G1SUU7	Q9Y3I0	RTCB	tRNA-splicing ligase RtcB homolog	tRNA-splicing ligase RtcB homolog	100	2	0.76	16	3	43
G1T1X2	A0A087WZF1	LPP	Uncharacterized protein	Lipoma-preferred partner	89	3	0.76	4	5	8
G1U949	Q15691	MAPRE1	Uncharacterized protein	Microtubule-associated protein RP/EB family member 1	99	3	0.76	8	25	43
G1TD47	Q8N3E9	PLCD3	Phosphoinositide phospholipase C	1-phosphatidylinositol 4,5-bisphosphate phosphodiesterase delta-3	88	2	0.76	4	6	9
G1T7Y7	B1AK87	CAPZB	F-actin-capping protein subunit beta	F-actin-capping protein subunit beta	100	2	0.75	11	34	54
G1TR42		RNPEP	Leuk-A4-hydro_C domain-containing protein			1	0.75	5	7	10

P48738	Q00169	PITPNA	Phosphatidylinositol transfer protein alpha isoform	Phosphatidylinositol transfer protein alpha isoform	99	2	0.75	6	6	24
G1TS73	H0YBP1	PTK2	Protein tyrosine kinase 2	Focal adhesion kinase 1 (Fragment)	89	2	0.75	4	5	6
G1SHA4		TEP1	Telomerase associated protein 1			1	0.75	5	8	3
G1TN89	P98160	HSPG2	Heparan sulfate proteoglycan 2	Basement membrane-specific heparan sulfate proteoglycan core protein	90	2	0.75	30	55	10
G1TEA8	P12268	IMPDH2	Inosine-5~-monophosphate dehydrogenase	Inosine-5~-monophosphate dehydrogenase 2	99	2	0.75	9	20	29
G1SUY5	E7EVZ5	PCYOX1L	Prenylcys_lyase domain-containing protein	Prenylcysteine oxidase-like	95	2	0.75	4	9	15
G1TBW1		TXNDC17	DUF953 domain-containing protein			1	0.75	3	7	31
	A0A087WY85	UBE2D3		Ubiquitin-conjugating enzyme E2 D3		4	0.75	2	4	20
G1T5Z7	B3KR49	MAPK3	Mitogen-activated protein kinase	Mitogen-activated protein kinase 3	97	2	0.75	6	3	37
G1T887		GPR107	G protein-coupled receptor 107			1	0.75	2	5	9
G1SE28	C9JNW5	RPL24	TRASH domain-containing protein	60S ribosomal protein L24	100	2	0.75	6	10	34
G1T0R4	Q96JC1	VPS39	CNH domain-containing protein	Vam6/Vps39-like protein	97	2	0.75	3	3	5
G1TA48	Q9H223	EHD4	Uncharacterized protein	EH domain-containing protein 4	97	3	0.75	14	20	37
G1SIN4	X6RLX0	ERC1	FIP-RBD domain-containing protein	ELKS/Rab6-interacting/CAST family member 1	98	2	0.75	14	21	15
G1SUT8	O95486	SEC24A	Uncharacterized protein	Protein transport protein Sec24A	93	3	0.75	5	8	7
G1SH85	A0A2R8YF87	VPS33A	Uncharacterized protein	Vacuolar protein sorting-associated protein 33A	91	3	0.75	8	12	22
	A6NM71	WDR45		PRA1 family protein		4	0.75	3	4	9
G1T519	P25789	PSMA4	Proteasome subunit alpha type	Proteasome subunit alpha type-4	100	2	0.75	10	22	58
G1TGK3	B4DP31	PRPSAP1	Pribosyltran_N domain-containing protein	Phosphoribosyl pyrophosphate synthase-associated protein 1	99	2	0.75	3	3	13
G1TE64	O95758	PTBP3	Uncharacterized protein	Polypyrimidine tract-binding protein 3	97	3	0.75	5	2	20
G1TYU5		QTRT1	Queuine tRNA-ribosyltransferase			1	0.75	2	3	12
	A0A494C0A9	CBFB		Core-binding factor subunit beta		4	0.74	4	6	31
G1T8U2		GMDS	GDP-mannose 4,6-dehydratase			1	0.74	2	4	13
G1TJC3	Q9BUF5	TUBB6	Tubulin beta chain	Tubulin beta-6 chain	97	2	0.74	17	23	57
G1SJG0	P28300	LOX	Uncharacterized protein	Protein-lysine 6-oxidase	88	3	0.74	4	6	14
G1SZT8	P55735	SEC13	WD_REPEATS_REGION domain-containing protein	Protein SEC13 homolog	95	2	0.74	8	17	38
G1STQ7		TMEM97	Transmembrane protein 97			1	0.74	2	2	12
G1SDA4	P53618	COPB1	Coatomer subunit beta	Coatomer subunit beta	99	2	0.74	37	202	54
G1SIA1	A0A087WWA3	KIF1B	Uncharacterized protein	Kinesin-like protein KIF1B	96	3	0.74	3	5	2
G1TB45	Q86W92	PPFIBP1	Uncharacterized protein	Liprin-beta-1	87	3	0.74	22	41	33
G1T085		SVIL	HP domain-containing protein			1	0.74	4	7	4
G1SNP9	P45954	ACADSB	Acyl-CoA dehydrogenase short/branched chain	Short/branched chain specific acyl-CoA dehydrogenase, mitochondrial	88	2	0.74	18	149	55
	Q9UBF2	COPG2		Coatomer subunit gamma-2		4	0.74	12	4	18
G1SLC0	Q9BS26	ERP44	Thioredoxin domain-containing protein	Endoplasmic reticulum resident protein 44	97	2	0.74	13	36	42
B7NZQ3		RA_m006_jsm824E4r	Deoxyribonuclease			1	0.74	6	15	28
G1SIB0	Q12907	LMAN2	L-type lectin-like domain-containing protein	Vesicular integral-membrane protein VIP36	98	2	0.74	6	15	23
G1TWB9		TBC1D5	Rab-GAP TBC domain-containing protein			1	0.74	2	3	4
G1SM01		AKAP9	A-kinase anchor protein 9			1	0.74	3	4	1
G1TBG2	Q96KP1	EXOC2	Exocyst complex component 2	Exocyst complex component 2	94	2	0.74	4	5	6
G1TNJ2	Q15942	ZYX	Uncharacterized protein	Zyxin	87	3	0.74	6	26	15
G1TP25	Q5VU77	UBAP2L	Ubiquitin associated protein 2 like	Ubiquitin-associated protein 2-like (Fragment)	95	2	0.74	3	4	4
G1SCF0	P13807	GYS1	Glycogen [starch] synthase	Glycogen [starch] synthase, muscle	96	2	0.74	3	4	6
G1T078	O43294	TGFB1I1	Uncharacterized protein	Transforming growth factor beta-1-induced transcript 1 protein	95	3	0.74	4	7	11
G1TBA4	Q86UY8	NT5DC3	Uncharacterized protein	5~-nucleotidase domain-containing protein 3	97	3	0.74	9	15	26
G1TJ80	Q9Y3Q3	TMED3	Transmembrane p24 trafficking protein 3	Transmembrane emp24 domain-containing protein 3	91	2	0.74	4	15	40
G1TAC4		GLRX	Glutaredoxin-1			1	0.74	3	9	38
G1SNY5	H0YK42	SNX1	PX domain-containing protein	Sorting nexin-1	96	2	0.73	9	14	20
G1SYN5	J3QRU4	VAMP2	V-SNARE coiled-coil homology domain-containing protein	Vesicle-associated membrane protein 2	99	2	0.73	4	4	42
G1TN13	A8CTZ0	ITSN1	Intersectin 1	Intersectin 1 short form variant 13	88	2	0.73	5	5	12
G1SLQ4	B4DXW1	ACTR3	Uncharacterized protein	Actin-related protein 3	100	3	0.73	17	47	71
G1T9W8	Q8TC07	TBC1D15	Rab-GAP TBC domain-containing protein	TBC1 domain family member 15	92	2	0.73	2	2	3
G1TMI5		RABAC1	PRA1 family protein			1	0.73	2	3	17
G1TS42		AGL	Glycogen debranching enzyme			1	0.73	3	2	4
G1TBN2	Q96II5	ARAF	Uncharacterized protein	ARAF protein	95	3	0.73	2	2	4
G1SFX7	P62330	ARF6	Uncharacterized protein	ADP-ribosylation factor 6	100	3	0.73	5	9	33
G1SIG2	P46108	CRK	Uncharacterized protein	Adapter molecule crk	99	3	0.73	7	4	37
G1SL68	P35579	MYH9	Uncharacterized protein	Myosin-9	96	3	0.73	119	317	57
G1SZK4	Q13442	PDAP1	PDGFA associated protein 1	28 kDa heat- and acid-stable phosphoprotein	96	2	0.73	2	4	16
G1TNY9		TRADD	Death domain-containing protein			1	0.73	2	3	10
G1SQ12	Q8TDJ6	DMXL2	WD_REPEATS_REGION domain-containing protein	DmX-like protein 2	93	2	0.73	2	2	1

G1SM05	Q9NR31	SAR1A	Uncharacterized protein	GTP-binding protein SAR1a	99	3	0.73	6	14	41
G1TZ40	Q5M775	SPECC1	Calponin-homology (CH) domain-containing protein	Cytospin-B	88	2	0.73	2	5	3
G1TAK5		WNK4	WNK lysine deficient protein kinase 4			1	0.73	3	3	2
G1T6T5	E9PPQ5	CHORDC1	Uncharacterized protein	Cysteine and histidine-rich domain-containing protein 1	94	3	0.73	2	2	9
G1SIP7	O14976	GAK	Cyclin G associated kinase	Cyclin-G-associated kinase	77	2	0.73	4	4	4
G1T450		RO60	TROVE domain-containing protein			1	0.73	3	6	7
G1T5Q4	Q9H446	RWDD1	RWD domain-containing protein	RWD domain-containing protein 1	91	2	0.73	4	7	27
	Q8NBZ7	UXS1		UDP-glucuronic acid decarboxylase 1		4	0.73	2	3	10
	P23142	FBLN1		Fibulin-1		4	0.72	2	2	3
G1SRT1	Q9UKG1	APPL1	Adaptor protein, phosphotyrosine interacting with PH domain and leucine zipper 1	DCC-interacting protein 13-alpha	98	2	0.72	8	10	19
G1ST51		CLIC2	Chloride intracellular channel protein			1	0.72	4	5	23
Q9XS70	Q9BR76	CORO1B	Coronin-1B	Coronin-1B	93	2	0.72	9	28	27
G1TEW4	Q93008	USP9X	USP domain-containing protein	Probable ubiquitin carboxyl-terminal hydrolase FAF-X	99	2	0.72	35	57	21
G1T1T4		ADPGK	ADP dependent glucokinase			1	0.72	9	19	27
G1SSL3	P29373	CRABP2	FABP domain-containing protein	Cellular retinoic acid-binding protein 2	90	2	0.72	3	3	27
G1STJ8	G3V394	MYO5A	Uncharacterized protein	Unconventional myosin-Va	96	3	0.72	17	4	12
	O15143	ARPC1B		Actin-related protein 2/3 complex subunit 1B		4	0.72	3	6	14
G1STU4	E9PIE3	CAVIN3	Uncharacterized protein	Caveolae-associated protein 3	78	3	0.72	4	5	15
G1SXX9	A0A0C4DGH3	NUMBL	NUMB like, endocytic adaptor protein	Numb-like protein	93	2	0.72	2	2	4
G1SFP0	P28838	LAP3	CYTOSOL_AP domain-containing protein	Cytosol aminopeptidase	92	2	0.72	17	28	52
G1SGX3	P22059	OSBP	Oxysterol-binding protein	Oxysterol-binding protein 1	97	2	0.72	8	12	12
G1SDY5	P63104	YWHAZ	14_3_3 domain-containing protein	14-3-3 protein zeta/delta	100	2	0.72	15	171	65
G1T8T0		FRMD6	FERM domain-containing protein			1	0.72	2	2	4
G1T9D6	C9J5C3	PDCD10	Uncharacterized protein	Programmed cell death protein 10 (Fragment)	99	3	0.72	7	8	48
G1T7H6	Q6UX71	PLXDC2	PSI domain-containing protein	Plexin domain-containing protein 2	94	2	0.72	4	10	9
	O14818	PSMA7		Proteasome subunit alpha type-7		4	0.72	9	20	44
G1U670	A0A087WW40	SH3GLB1	Uncharacterized protein	Endophilin-B1	95	3	0.72	2	3	6
G1SKY8	Q96A57	TMEM230	Uncharacterized protein	Transmembrane protein 230	98	3	0.72	2	3	22
	Q15149	PLEC		Plectin		4	0.72	84	118	21
	P17612	PRKACA		cAMP-dependent protein kinase catalytic subunit alpha		4	0.72	6	4	27
G1T8R1		RAP1GAP2	Platelet-activating factor acetylhydrolase IB subunit alpha			1	0.72	8	7	24
U3KNB6	P48444	ARCN1	Coatomer subunit delta	Coatomer subunit delta	97	2	0.72	18	32	40
G1T295		EPHX1	Epoxide hydrolase			1	0.72	5	6	15
G1SCQ1		AKR7L	Aldo_ket_red domain-containing protein			1	0.71	7	8	35
G1SN05	Q14240	EIF4A2	Uncharacterized protein	Eukaryotic initiation factor 4A-II	100	3	0.71	14	17	49
G1SYK3	P45985	MAP2K4	Protein kinase domain-containing protein	Dual specificity mitogen-activated protein kinase kinase 4	99	2	0.71	3	3	10
G1SKT1	Q7Z7H5	TMED4	GOLD domain-containing protein	Transmembrane emp24 domain-containing protein 4	94	2	0.71	7	16	44
G1SIT9	P31946	YWHAB	14_3_3 domain-containing protein	14-3-3 protein beta/alpha	100	2	0.71	16	58	76
	P25098	GRK2		Beta-adrenergic receptor kinase 1		4	0.71	2	2	6
P35748	P35749	MYH11	Myosin-11	Myosin-11	97	2	0.71	42	38	29
G1SI13		SDC2	Syndecan			1	0.71	3	4	11
G1SMS3	P61160	ACTR2	Actin-related protein 2	Actin-related protein 2	100	2	0.71	13	121	48
Q9GLC3		ATP1B3	Sodium/potassium-transporting ATPase subunit beta-3			1	0.71	3	5	15
G1STY8	G3V5E4	GNPNAT1	Glucosamine 6-phosphate N-acetyltransferase	Glucosamine 6-phosphate N-acetyltransferase	100	2	0.71	4	4	20
P13642	P49591	SARS	Serine--tRNA ligase, cytoplasmic	Serine--tRNA ligase, cytoplasmic	97	2	0.71	14	29	32
G1TCM9	Q9H299	SH3BGRL3	SH3 domain-binding glutamic acid-rich-like protein	SH3 domain-binding glutamic acid-rich-like protein 3	100	2	0.71	2	6	52
G1SMK4		UCKL1	Uridine-cytidine kinase 1 like 1			1	0.71	2	3	6
G1SN14	Q86VP6	CAND1	TIP120 domain-containing protein	Cullin-associated NEDD8-dissociated protein 1	100	2	0.71	32	54	36
G1TII2	O95197	RTN3	Reticulon	Reticulon-3	72	2	0.71	4	6	6
G1TYW1		TPD52L2	TPD52 like 2			1	0.71	3	5	13
G1SPZ7		GPX1	Glutathione peroxidase			1	0.71	5	6	51
G1TCY4	Q9Y5X3	SNX5	Sorting nexin	Sorting nexin-5	98	2	0.71	4	7	12
G1SD09	A0A0J9YYJ0	CNPY4	Canopy FGF signaling regulator 4	Protein canopy homolog 4 (Fragment)	97	2	0.70	6	16	35
G1T060	A0A2R8YD50	HSD17B4	Uncharacterized protein	Peroxisomal multifunctional enzyme type 2	89	3	0.70	9	15	21
G1TM81	A0A0B4J2C3	TPT1	Translationally-controlled tumor protein	Translationally-controlled tumor protein	98	2	0.70	5	68	34
G1SMF4	Q9UHP3	USP25	USP domain-containing protein	Ubiquitin carboxyl-terminal hydrolase 25	94	2	0.70	4	6	6
G1TI02	Q9BRP8	PYM1	PYM homolog 1, exon junction complex associated factor	Partner of Y14 and mago	91	2	0.70	2	4	6
G1SJ56	P18206	VCL	Uncharacterized protein	Vinculin	99	3	0.70	54	176	59
G1T416	Q9UP83	COG5	Uncharacterized protein	Conserved oligomeric Golgi complex subunit 5	91	3	0.70	5	6	11
G1TW66		DMAC2	Distal membrane arm assembly complex 2			1	0.70	2	3	17

G1SER8	P07737	PFN1	Profilin	Profilin-1	94	2	0.70	7	134	75
G1T4K5	A0A1B0GTW1	TJP2	Uncharacterized protein	Tight junction protein ZO-2	87	3	0.70	5	5	8
G1SU80	O60462	NRP2	Neuropilin	Neuropilin-2	95	2	0.70	17	22	25
G1SN76	O94804	STK10	Serine/threonine kinase 10	Serine/threonine-protein kinase 10	90	2	0.70	2	5	5
	A0A0G2JL54	C4B_2		Complement C4-B		4	0.70	3	2	1
G1SET0	P35606	COPB2	Coatomer subunit beta~	Coatomer subunit beta~	98	2	0.70	24	57	38
G1SMA1	P49755	TMED10	Transmembrane emp24 domain-containing protein 10	Transmembrane emp24 domain-containing protein 10	95	2	0.70	8	296	41
G1TBS1	Q99497	PARK7	DJ-1_PfpI domain-containing protein	Protein/nucleic acid deglycase DJ-1	96	2	0.70	11	22	72
G1SNQ2	Q8WVF1	OSCP1	Uncharacterized protein	Protein OSCP1	87	3	0.70	2	3	8
G1TER4	P33897	ABCD1	Uncharacterized protein	ATP-binding cassette sub-family D member 1	94	3	0.70	4	4	8
G1SV81	Q9BTE1	DCTN5	Uncharacterized protein	Dynactin subunit 5	100	3	0.70	2	2	9
G1SS77	Q15477	SKIV2L	Uncharacterized protein	Helicase SKI2W	95	3	0.70	7	9	7
G1SGJ4		SRR	PALP domain-containing protein			1	0.70	2	3	5
G1T6S9	P53367	ARFIP1	AH domain-containing protein	Arfaptin-1	98	2	0.70	4	6	15
G1SPL8		NGB	Neuroglobin			1	0.70	2	3	18
G1SZ12	E7EPB3	RPL14	Ribosomal_L14e domain-containing protein	60S ribosomal protein L14	96	2	0.70	6	12	27
G1TD38	H3BVG8	VPS35L	Uncharacterized protein	VPS35 endosomal protein sorting factor-like	93	3	0.70	6	10	4
P62943	P62942	FKBP1A	Peptidyl-prolyl cis-trans isomerase FKBP1A	Peptidyl-prolyl cis-trans isomerase FKBP1A	100	2	0.70	2	6	25
G1U5L3	P49257	LMAN1	L-type lectin-like domain-containing protein	Protein ERGIC-53	91	2	0.70	15	67	40
G1T8D4	D6REX3	SEC31A	WD_REPEATS_REGION domain-containing protein	Protein transport protein Sec31A	91	2	0.70	29	64	35
G1SQ02	A0A0A0MSI0	PRDX1	Thioredoxin domain-containing protein	Peroxiredoxin-1 (Fragment)	98	2	0.70	10	17	57
G1SE61	O75369	FLNB	Filamin-B	Filamin-B	97	2	0.69	106	546	61
G1SNE1	Q6DKJ4	NXN	Thioredoxin domain-containing protein	Nucleoredoxin	98	2	0.69	8	24	30
	P60468	SEC61B		Protein transport protein Sec61 subunit beta		4	0.69	3	5	52
G1T3V2	P04792	HSPB1	SHSP domain-containing protein	Heat shock protein beta-1	91	2	0.69	8	22	52
G1SKD5	Q92783	STAM	Uncharacterized protein	Signal transducing adapter molecule 1	96	3	0.69	5	12	14
G1TRX7	P23743	DGKA	Diacylglycerol kinase	Diacylglycerol kinase alpha	93	2	0.69	4	5	9
G1T4C9	Q8IVL6	P3H3	Prolyl 3-hydroxylase 3	Prolyl 3-hydroxylase 3	88	2	0.69	14	24	29
G1U2R2	E9PLK3	NPEPPS	Aminopeptidase	Aminopeptidase	98	2	0.69	16	26	27
G1T4P7	Q9Y4G6	TLN2	Talin 2	Talin-2	98	2	0.69	19	10	11
G1SFZ8	Q9UMX0	UBQLN1	Ubiquilin 1	Ubiquilin-1	86	2	0.69	8	10	26
G1SW11		CERCAM	Cerebral endothelial cell adhesion molecule			1	0.69	6	9	16
G1T3S7	O60476	MAN1A2	alpha-1,2-Mannosidase	Mannosyl-oligosaccharide 1,2-alpha-mannosidase IB	95	2	0.69	2	2	4
G1T0G0		P3H2	Fe2OG dioxygenase domain-containing protein			1	0.69	2	3	4
G1TQR0	P12814-3	ACTN1	Uncharacterized protein	Isoform 3 of Alpha-actinin-1	97	3	0.69	59	118	79
G1SIL2	Q9UNW1	MINPP1	Multiple inositol-polyphosphate phosphatase 1	Multiple inositol polyphosphate phosphatase 1	87	2	0.69	4	6	16
G1T6S4	F8W9J4	DST	Dystonin	Dystonin	62	2	0.68	13	7	4
G1T8H3	P51570	GALK1	Uncharacterized protein	Galactokinase	92	3	0.68	10	14	42
G1TMP7	A0A0D9SG72	STXBP1	Syntaxin binding protein 1	Syntaxin-binding protein 1	99	2	0.68	4	6	10
G1SZW8	M0R165	EPS15L1	Epidermal growth factor receptor pathway substrate 15 like 1	Epidermal growth factor receptor substrate 15-like 1	88	2	0.68	9	13	16
G1SLL1	A0A087X0K9	TJP1	Uncharacterized protein	Tight junction protein ZO-1	82	3	0.68	21	29	19
G1SST7	Q96SL4	GPX7	Glutathione peroxidase	Glutathione peroxidase 7	92	2	0.68	6	9	36
G1SDN8	Q92791	P3H4	Uncharacterized protein	Endoplasmic reticulum protein SC65	88	3	0.68	5	9	18
G1T8S0	Q9C0D5	TANC1	Uncharacterized protein	Protein TANC1	85	3	0.68	8	10	9
G1U7S8	F8W6C2	SPATS2L	Uncharacterized protein	SPATS2-like protein (Fragment)	100	3	0.68	4	5	9
G1SXY5		STX2	Syntaxin 2			1	0.68	3	4	21
G1SHS7	O75083	WDR1	WD_REPEATS_REGION domain-containing protein	WD repeat-containing protein 1	94	2	0.68	21	52	51
G1TP81	Q9Y6W5	WASF2	WH2 domain-containing protein	Wiskott-Aldrich syndrome protein family member 2	94	2	0.68	4	5	10
G1SPL3	G3V583	FAM177A1	Uncharacterized protein	Protein FAM177A1 (Fragment)	93	3	0.68	2	2	12
G1U2E3	Q9H444	CHMP4B	Uncharacterized protein	Charged multivesicular body protein 4b	100	3	0.68	7	8	40
G1SWL6	Q9P0K7	RAI14	ANK_REP_REGION domain-containing protein	Ankycorbin	91	2	0.68	24	42	32
G1SJ87	Q9H2D6	TRIOBP	TRIO and F-actin binding protein	TRIO and F-actin-binding protein	73	2	0.68	3	5	2
G1SDL9	Q9UEU0	VTI1B	t-SNARE coiled-coil homology domain-containing protein	Vesicle transport through interaction with t-SNAREs homolog 1B	93	2	0.68	4	8	24
	O14617	AP3D1		AP-3 complex subunit delta-1		4	0.67	8	12	9
G1SWD8	P42356	PI4KA	Uncharacterized protein	Phosphatidylinositol 4-kinase alpha	98	3	0.67	10	5	8
G1SX42	Q9Y3P9	RABGAP1	Uncharacterized protein	Rab GTPase-activating protein 1	97	3	0.67	2	2	3
G1SQ23	P42025	ACTR1B	Uncharacterized protein	Beta-centractin	99	3	0.67	7	4	25
	P35908	KRT2		Keratin, type II cytoskeletal 2 epidermal		4	0.67	10	9	25
G1TDQ5	A0A2R8Y891	PFKM	ATP-dependent 6-phosphofructokinase	ATP-dependent 6-phosphofructokinase	97	2	0.67	5	7	10
G1T7D0		SRRM2	Serine/arginine repetitive matrix 2			1	0.67	2	2	1

G1T057	D6RCE2	TTC37	Uncharacterized protein	Tetratricopeptide repeat protein 37 (Fragment)	83	3	0.67	7	14	6
	A0A494C1N0	FKBP2		Peptidylprolyl isomerase		4	0.67	2	7	17
G1T7I4	P53621	COPA	Coatomer subunit alpha	Coatomer subunit alpha	99	2	0.67	48	102	52
G1TRZ2		LAMP1	Lysosomal associated membrane protein 1			1	0.67	5	18	14
G1TRH5	Q9ULV4	CORO1C	Coronin	Coronin-1C	97	2	0.67	19	78	44
G1SXU7	F8WA11	CLASP1	Cytoplasmic linker associated protein 1	CLIP-associating protein 1	93	2	0.67	4	2	4
G1SIC4	I3L294	ABHD12	Abhydrolase domain containing 12	Lysophosphatidylserine lipase ABHD12 (Fragment)	99	2	0.67	4	6	18
	O43707	ACTN4		Alpha-actinin-4		4	0.67	52	73	64
U3KNY1	Q12797	ASPH	Aspartate beta-hydroxylase	Aspartyl/asparaginyl beta-hydroxylase	96	2	0.67	19	12	56
U3KMD1	A0A0A0MRI2	SNX6	Vps5 domain-containing protein	Sorting nexin	99	2	0.67	11	108	28
G1SJH1	H7BXE3	SLTM	RRM domain-containing protein	SAFB-like transcription modulator (Fragment)	92	2	0.67	3	2	4
G1SR27	P62993	GRB2	Uncharacterized protein	Growth factor receptor-bound protein 2	100	3	0.66	3	5	22
G1SW44	H7C286	NAGK	BcrAD_BadFG domain-containing protein	N-acetyl-D-glucosamine kinase	97	2	0.66	6	11	27
	E9PS68	PC		Pyruvate carboxylase, mitochondrial		4	0.66	2	2	6
G1SQK1		SERPINB6	SERPIN domain-containing protein			1	0.66	3	4	17
G1T2F8	H0YJH8	ATP6V1D	V-type proton ATPase subunit D	V-type proton ATPase subunit D (Fragment)	99	2	0.66	3	14	23
G1T8L2	P05997	COL5A2	Uncharacterized protein	Collagen alpha-2(V) chain	96	3	0.66	15	32	17
G1T5W7	Q14376	GALE	NAD(P)-bd_dom domain-containing protein	UDP-glucose 4-epimerase	95	2	0.66	7	14	27
G1SD27	B7ZC38	SH3GLB2	SH3 domain containing GRB2 like, endophilin B2	Endophilin-B2	92	2	0.66	2	2	6
G1SID3	Q709C8	VPS13C	Vacuolar protein sorting 13 homolog C	Vacuolar protein sorting-associated protein 13C	88	2	0.66	10	11	4
G1SNS3	Q6P4E1	CASC4	Uncharacterized protein	Protein CASC4	89	3	0.66	7	9	16
G1SZF9	O75976	CPD	Uncharacterized protein	Carboxypeptidase D	91	3	0.66	5	5	5
G1SWK3	P49902	NT5C2	Uncharacterized protein	Cytosolic purine 5~-nucleotidase	100	3	0.66	8	14	21
G1SMY7	C9JIF9	APEH	Acylamino-acid-releasing enzyme	Acylamino-acid-releasing enzyme	93	2	0.66	7	11	18
G1SXQ0	A0A0A0MTN3	GSTM3	Glutathione S-transferase	Glutathione S-transferase	90	2	0.66	10	24	37
	P50454	SERPINH1		Serpin H1		4	0.66	15	30	35
G1T7R2	P62258	YWHAE	14_3_3 domain-containing protein	14-3-3 protein epsilon	100	2	0.66	18	204	78
G1T432	Q01518	CAP1	Adenylyl cyclase-associated protein	Adenylyl cyclase-associated protein 1	96	2	0.66	24	173	65
	P09497-2	CLTB		Isoform Non-brain of Clathrin light chain B		4	0.66	5	10	21
G1T3U1	Q9H8Y8	GORASP2	GRASP55_65 domain-containing protein	Golgi reassembly-stacking protein 2	91	2	0.66	6	15	18
G1T7B1	Q9H3P7	ACBD3	Uncharacterized protein	Golgi resident protein GCP60	96	3	0.65	7	8	21
	J3QQX2	ARHGDIA		Rho GDP-dissociation inhibitor 1		4	0.65	5	7	18
G1SP34	O15144	ARPC2	Arp2/3 complex 34 kDa subunit	Actin-related protein 2/3 complex subunit 2	100	2	0.65	15	32	54
G1TPH5	Q9Y6D5	ARFGEF2	ADP ribosylation factor guanine nucleotide exchange factor 2	Brefeldin A-inhibited guanine nucleotide-exchange protein 2	96	2	0.65	9	10	8
G1U9R8	P06396-2	GSN	Uncharacterized protein	Isoform 2 of Gelsolin	95	3	0.65	19	43	41
G1SIB6	M0R192	BLVRB	Biliverdin reductase B	Flavin reductase (NADPH)	73	2	0.65	7	32	51
G1U5A6	A0A286YFF8	MON2	Uncharacterized protein	Protein MON2 homolog	98	3	0.65	6	7	6
G1TFI4	Q96K17	BTF3L4	Transcription factor BTF3	Transcription factor BTF3 homolog 4	100	2	0.65	3	4	39
G1SEJ4	P21281	ATP6V1B2	Vacuolar proton pump subunit B	V-type proton ATPase subunit B, brain isoform	99	2	0.65	18	43	55
G1SMT2		FAM129B	Family with sequence similarity 129 member B			1	0.65	17	44	36
G1T7Z0	P52209	PGD	6-phosphogluconate dehydrogenase, decarboxylating	6-phosphogluconate dehydrogenase, decarboxylating	93	2	0.65	14	18	31
	P21333	FLNA		Filamin-A		4	0.65	105	13	51
G1TME7		CSTB	Cystatin B			1	0.65	3	4	36
G1TCD4	E5RGF9	FAM114A2	Uncharacterized protein	Protein FAM114A2 (Fragment)	80	3	0.65	5	4	18
G1TD16	P48426	PIP4K2A	PIPK domain-containing protein	Phosphatidylinositol 5-phosphate 4-kinase type-2 alpha	100	2	0.65	2	2	5
G1SP54		LTA4H	Leukotriene A(4) hydrolase			1	0.65	4	6	10
G1TEG8	Q8WUM4	PDCD6IP	BRO1 domain-containing protein	Programmed cell death 6-interacting protein	94	2	0.65	24	95	36
G1TPC5		TPD52	Tumor protein D52			1	0.65	3	6	19
G1T345		UBR1	E3 ubiquitin-protein ligase			1	0.65	2	2	2
	A0A2U3U034	ARSB		Arylsulfatase B		4	0.64	4	4	12
G1SR53	P04066	FUCA1	Alpha-L-fucosidase	Tissue alpha-L-fucosidase	83	2	0.64	10	29	34
	Q92696	RABGGTA		Geranylgeranyl transferase type-2 subunit alpha		4	0.64	2	2	6
G1SKV7	Q96KP4	CNDP2	M20_dimer domain-containing protein	Cytosolic non-specific dipeptidase	90	2	0.64	12	21	38
G1SN11	Q01082	SPTBN1	Spectrin beta chain	Spectrin beta chain, non-erythrocytic 1	99	2	0.64	75	14	44
G1T004	H0Y987	PGM3	Phosphoacetylglucosamine mutase	Phosphoacetylglucosamine mutase	90	2	0.64	8	21	27
G1T7T6	O60763	USO1	Uncharacterized protein	General vesicular transport factor p115	95	3	0.64	22	43	35
	Q7Z406	MYH14		Myosin-14		4	0.64	14	62	8
G1TWQ3		SIRT2	NAD-dependent protein deacetylase			1	0.64	5	5	19
G1SKL7	Q99536	VAT1	Vesicle amine transport 1	Synaptic vesicle membrane protein VAT-1 homolog	78	2	0.64	9	17	35
G1TX63		CDC42BPA	Non-specific serine/threonine protein kinase			1	0.64	4	2	4

G1T196	Q13033	STRN3	WD_REPEATS_REGION domain-containing protein	Striatin-3	92	2	0.64	4	4	12
G1TZP0	P61981	YWHAG	14_3_3 domain-containing protein	14-3-3 protein gamma	99	2	0.64	13	53	70
G1SQ22	Q9Y678	COPG1	Coatomer subunit gamma	Coatomer subunit gamma-1	99	2	0.64	35	172	58
G1T4G0	Q96N67	DOCK7	Uncharacterized protein	Dedicator of cytokinesis protein 7	97	3	0.64	11	3	10
	Q8WXF7-2	ATL1		Isoform 2 of Atlastin-1		4	0.64	5	7	15
	J3KN75	TBC1D8B		TBC1 domain family member 8B		4	0.64	2	2	3
G1TMW2		TOLLIP	Toll interacting protein			1	0.64	4	4	25
G1SGK1		GIGYF2	GRB10 interacting GYF protein 2			1	0.63	3	4	5
G1SJJ2	Q9NZ32	ACTR10	Uncharacterized protein	Actin-related protein 10	96	3	0.63	10	22	40
	Q16181	SEPTIN7		Septin-7		4	0.63	19	55	57
Q09YN4	P47755	CAPZA2	F-actin-capping protein subunit alpha-2	F-actin-capping protein subunit alpha-2	98	2	0.63	8	14	50
G1SWU8	A0A0C4DGW6	C5orf51	Uncharacterized protein	UPF0600 protein C5orf51	96	3	0.63	3	3	45
	P20908	COL5A1		Collagen alpha-1(V) chain		4	0.63	9	3	6
G1SS33	P36543	ATP6V1E1	Uncharacterized protein	V-type proton ATPase subunit E 1	99	3	0.63	11	15	58
G1U9R6	P02751	FN1	Fibronectin	Fibronectin	70	2	0.63	69	158	46
G1TCW2		OSTF1	Osteoclast stimulating factor 1			1	0.63	2	4	15
G1SR28	Q15102	PAFAH1B3	SGNH_hydro domain-containing protein	Platelet-activating factor acetylhydrolase IB subunit gamma	97	2	0.63	5	7	31
G1SNL7	Q9H1I8	ASCC2	Activating signal cointegrator 1 complex subunit 2	Activating signal cointegrator 1 complex subunit 2	86	2	0.63	3	3	6
G1TBC4	G3V126	ATP6V1H	V-type proton ATPase subunit H	V-type proton ATPase subunit H	99	2	0.63	6	12	23
G1TBY1		CTSB	Pept_C1 domain-containing protein			1	0.63	7	60	30
G1TEH2	P21283	ATP6V1C1	V-type proton ATPase subunit C	V-type proton ATPase subunit C 1	99	2	0.63	7	10	20
G1SY85	Q9Y2T2	AP3M1	MHD domain-containing protein	AP-3 complex subunit mu-1	99	2	0.62	9	15	42
G1T3I9	P20073	ANXA7	Annexin	Annexin A7	93	2	0.62	10	12	20
G1SR49		SEPT10	Septin-type G domain-containing protein			1	0.62	6	11	17
G1TRS4	O95302	FKBP9	Peptidylprolyl isomerase	Peptidyl-prolyl cis-trans isomerase FKBP9	97	2	0.62	17	141	41
G1SN09	Q9Y5P6	GMPPB	NTP_transferase domain-containing protein	Mannose-1-phosphate guanyltransferase beta	99	2	0.62	4	4	22
G1TBC9		TMEM119	Transmembrane protein 119			1	0.62	2	3	17
G1SZ63	P49189	ALDH9A1	Aldedh domain-containing protein	4-trimethylaminobutyraldehyde dehydrogenase	94	2	0.62	8	12	29
G1SYM3		CD9	Tetraspanin			1	0.62	3	4	7
G1U446	Q14677	CLINT1	ENTH domain-containing protein	Clathrin interactor 1	97	2	0.62	9	14	17
G1SKE0	Q15555	MAPRE2	Uncharacterized protein	Microtubule-associated protein RP/EB family member 2	98	3	0.62	2	2	11
G1STD5		SUCO	SUN domain containing ossification factor			1	0.62	2	4	3
G1TC70	O14974	PPP1R12A	Protein phosphatase 1 regulatory subunit	Protein phosphatase 1 regulatory subunit 12A	94	2	0.62	13	35	16
G1SNH7	P09417	QDPR	Quinoid dihydropteridine reductase	Dihydropteridine reductase	92	2	0.62	4	6	23
G1SCW0	A8MT72	RTN1	Reticulon	Reticulon	98	2	0.62	2	3	3
G1SY70	F5H365	SEC23A	Protein transport protein SEC23	Protein transport protein SEC23	96	2	0.62	22	44	47
G1T9V6	Q9UPU5	USP24	Ubiquitin specific peptidase 24	Ubiquitin carboxyl-terminal hydrolase 24	98	2	0.62	7	8	5
G1T823	H3BT58	COTL1	Coactosin like F-actin binding protein 1	Coactosin-like protein	96	2	0.62	3	9	27
G1U0Y0	A0A2Q3DQE3	CAMK2G	Protein kinase domain-containing protein	Calcium/calmodulin-dependent protein kinase (CaM kinase) II gamma, isoform CRA_d	91	2	0.61	7	5	23
G1SUK5	O43583	DENR	Density-regulated protein	Density-regulated protein	97	2	0.61	2	5	9
G1U5B3	C9JIZ6	PSAP	Prosaposin	Prosaposin	78	2	0.61	13	47	40
G1T7D9		FNTB	Protein farnesyltransferase subunit beta			1	0.61	3	2	6
G1TAB2		GM2A	ML domain-containing protein			1	0.61	4	8	22
G1U5Q7		ARPC4	Actin-related protein 2/3 complex subunit 4			1	0.61	7	37	46
G1TZ31	Q9Y680	FKBP7	Peptidylprolyl isomerase	Peptidyl-prolyl cis-trans isomerase FKBP7	77	2	0.61	11	27	44
G1SCE1	Q9H4G4	GLIPR2	SCP domain-containing protein	Golgi-associated plant pathogenesis-related protein 1	97	2	0.61	4	5	41
G1SDF2	A0A0A0MRJ6	PCMT1	Protein-L-isoaspartate O-methyltransferase	Protein-L-isoaspartate O-methyltransferase	96	2	0.61	5	7	21
G1SVI9	Q9P0L0	VAPA	MSP domain-containing protein	Vesicle-associated membrane protein-associated protein A	84	2	0.61	9	21	40
Q09YN6	C9JKI3	CAV1	Caveolin-1	Caveolin (Fragment)	96	2	0.61	3	4	21
G1T6Q9	O15400	STX7	t-SNARE coiled-coil homology domain-containing protein	Syntaxin-7	95	2	0.61	7	18	48
	P35527	KRT9		Keratin, type I cytoskeletal 9		4	0.61	19	37	44
	Q96BM9	ARL8A		ADP-ribosylation factor-like protein 8A		4	0.61	7	2	45
G1TVW5	Q96IJ6	GMPPA	NTP_transferase domain-containing protein	Mannose-1-phosphate guanyltransferase alpha	96	2	0.61	7	10	30
G1T1G5	Q9H4A6	GOLPH3	Uncharacterized protein	Golgi phosphoprotein 3	99	3	0.61	5	6	32
O77708	D6R938	CAMK2D	Calcium/calmodulin-dependent protein kinase type II subunit delta	Calcium/calmodulin-dependent protein kinase (CaM kinase) II delta, isoform CRA_e	92	2	0.60	12	18	33
G1SNP8	C9JJP5	TFG	PB1 domain-containing protein	Protein TFG (Fragment)	97	2	0.60	7	16	23
	Q6WCQ1	MPRIP		Myosin phosphatase Rho-interacting protein		4	0.60	16	2	20
G1TI53	A0A0A0MRM8	MYO6	Uncharacterized protein	Unconventional myosin-VI	94	3	0.60	7	13	8
G1TLL6	Q9UNH6	SNX7	PX domain-containing protein	Sorting nexin-7	96	2	0.60	4	4	15
G1SDT0	Q562R1	ACTBL2	Uncharacterized protein	Beta-actin-like protein 2	97	3	0.60	14	17	41

G1T1V0	P13645	KRT10	IF rod domain-containing protein	Keratin, type I cytoskeletal 10	95	2	0.60	22	59	51
G1T8C8	A0A087X0R6	SNX12	Sorting nexin 12	Sorting nexin-12	100	2	0.60	2	3	13
G1SPR5	P16278	GLB1	Glyco_hydro_35 domain-containing protein	Beta-galactosidase	82	2	0.59	15	28	33
G1TCW5	A0A494C165	PEPD	Peptidase D	Xaa-Pro dipeptidase (Fragment)	79	2	0.59	7	11	22
G1TA50	E7EQ61	UBA5	ThiF domain-containing protein	Ubiquitin-like modifier-activating enzyme 5	93	2	0.59	6	10	27
G1U4A0		FRMD8	FERM domain containing 8			1	0.59	2	2	9
G1SG54	A0A0U1RQT1	ACAP2	Arf-GAP with coiled-coil, ANK repeat and PH domain-containing protein 2	Arf-GAP with coiled-coil, ANK repeat and PH domain-containing protein 2 (Fragment)	99	2	0.59	4	5	11
G1SR77	P23634	ATP2B4	Calcium-transporting ATPase	Plasma membrane calcium-transporting ATPase 4	87	2	0.59	9	9	10
G1SSP0		STRN	WD_REPEATS_REGION domain-containing protein			1	0.59	3	5	11
G1SWW6	F5H459	AP3S1	Clat_adaptor_s domain-containing protein	AP complex subunit sigma	95	2	0.59	2	3	14
G1SCI0	Q14204	DYNC1H1	Dynein cytoplasmic 1 heavy chain 1	Cytoplasmic dynein 1 heavy chain 1	98	2	0.59	121	80	52
G1TF20		PLEKHO2	PH domain-containing protein			1	0.59	2	2	13
G1TTJ1	Q6UWP2	DHRS11	Uncharacterized protein	Dehydrogenase/reductase SDR family member 11	92	3	0.59	4	5	21
G1T9X4	O95980	RECK	Reversion inducing cysteine rich protein with kazal motifs	Reversion-inducing cysteine-rich protein with Kazal motifs	95	2	0.59	3	6	3
G1SZ47	P62266	RPS23	Uncharacterized protein	40S ribosomal protein S23	100	3	0.59	6	105	48
G1SL62	P07355	ANXA2	Annexin	Annexin A2	98	2	0.59	26	60	71
	P56377	AP1S2		AP-1 complex subunit sigma-2		4	0.58	4	5	33
G1TV31	O15511	ARPC5	Actin-related protein 2/3 complex subunit 5	Actin-related protein 2/3 complex subunit 5	95	2	0.58	8	49	54
G1SY00	P42858	HTT	Uncharacterized protein	Huntingtin	91	3	0.58	4	4	3
G1SIT6	Q9BPX5	ARPC5L	Actin-related protein 2/3 complex subunit 5	Actin-related protein 2/3 complex subunit 5-like protein	98	2	0.58	7	11	63
G1U411	F8WBG8	DBNL	Uncharacterized protein	Drebrin-like protein	95	3	0.58	9	19	31
G1U9I8	P04264	KRT1	IF rod domain-containing protein	Keratin, type II cytoskeletal 1	86	2	0.58	20	36	39
G1SPM5	P61163	ACTR1A	Uncharacterized protein	Alpha-centractin	100	3	0.58	11	15	40
G1T8X3		NEU1	Sialidase domain-containing protein			1	0.58	4	6	18
G1U0Q7		SEPT2	Septin-type G domain-containing protein			1	0.58	13	61	54
G1SLX0	G3V4P8	GMFB	Glia maturation factor	Glia maturation factor beta (Fragment)	99	2	0.58	3	7	30
G1SCP7	P46940	IQGAP1	Uncharacterized protein	Ras GTPase-activating-like protein IQGAP1	97	3	0.58	75	250	60
G1SD83		ITGA6	Integrin_alpha2 domain-containing protein			1	0.58	2	6	2
G1SUV2		CAMLG	Calcium signal-modulating cyclophilin ligand			1	0.58	2	6	16
G1SGL0	Q92629	SGCD	Uncharacterized protein	Delta-sarcoglycan	98	3	0.58	3	6	14
G1SZP0	O60784	TOM1	Uncharacterized protein	Target of Myb protein 1	88	3	0.58	11	28	44
G1T9W9		ATG3	Autophagy-related protein 3			1	0.58	2	2	8
G1T512		CNP	2~,3~-cyclic nucleotide 3~ phosphodiesterase			1	0.58	2	3	7
G1T277	F8VR50	ARPC3	Actin-related protein 2/3 complex subunit 3	Actin-related protein 2/3 complex subunit 3 (Fragment)	100	2	0.57	3	9	17
	Q08209-2	PPP3CA		Isoform 2 of Serine/threonine-protein phosphatase 2B catalytic subunit alpha isoform		4	0.57	9	15	27
G1T8R3	P46939	UTRN	Uncharacterized protein	Utrophin	91	3	0.57	53	68	24
G1SZ18	A0A0C4DFT3	DLG1	Uncharacterized protein	Disks large homolog 1	93	3	0.57	4	5	7
G1SY84	Q13409-2	DYNC1I2	WD_REPEATS_REGION domain-containing protein	Isoform 2B of Cytoplasmic dynein 1 intermediate chain 2	92	2	0.57	9	2	29
G1TU12	Q5H907	MAGED2	MAGE domain-containing protein	Melanoma antigen family D, 2, isoform CRA_d	79	2	0.57	5	4	10
P53787		EEF1D	Elongation factor 1-delta			1	0.57	4	16	24
G1SNT1	O75822	EIF3J	Eukaryotic translation initiation factor 3 subunit J	Eukaryotic translation initiation factor 3 subunit J	92	2	0.57	11	16	48
G1T346	Q13813-3	SPTAN1	Uncharacterized protein	Isoform 3 of Spectrin alpha chain, non-erythrocytic 1	97	3	0.57	108	29	55
G1SL41		GUSB	Beta-glucuronidase			1	0.57	11	26	30
G1TYA7	P07195	LDHB	L-lactate dehydrogenase	L-lactate dehydrogenase B chain	100	2	0.57	17	183	58
	P68371	TUBB4B		Tubulin beta-4B chain		4	0.57	22	25	71
G1SEK2		PPM1F	PPM-type phosphatase domain-containing protein			1	0.56	2	3	11
G1SHN4	Q96JB2	COG3	Uncharacterized protein	Conserved oligomeric Golgi complex subunit 3	96	3	0.56	4	4	10
B7NZG7	O60493	SNX3	Sorting nexin 3 (Predicted)	Sorting nexin-3	100	2	0.56	2	4	17
G1SEA7	Q13131	PRKAA1	Non-specific serine/threonine protein kinase	5~-AMP-activated protein kinase catalytic subunit alpha-1	97	2	0.56	3	3	8
G1SXK6	Q9NR12	PDLIM7	PDZ and LIM domain 7	PDZ and LIM domain protein 7	92	2	0.56	8	24	26
G1SW57	P21589	NT5E	Uncharacterized protein	5~-nucleotidase	89	3	0.56	10	10	21
G1SEX8	Q9P299	COPZ2	Clat_adaptor_s domain-containing protein	Coatomer subunit zeta-2	84	2	0.56	4	6	23
G1T3D7		NANS	AFP-like domain-containing protein			1	0.56	4	7	14
G1SZH6	Q9BZF1	OSBPL8	Oxysterol-binding protein	Oxysterol-binding protein-related protein 8	99	2	0.56	7	10	11
	P22392-2	NME2		Isoform 3 of Nucleoside diphosphate kinase B		4	0.56	7	25	35
G1SN21	P00491	PNP	Purine nucleoside phosphorylase	Purine nucleoside phosphorylase	90	2	0.55	13	30	62
G1SNC4	Q5T9B7	AK1	Adenylate kinase isoenzyme 1	Adenylate kinase isoenzyme 1	94	2	0.55	2	4	12
G1SQ03	Q8WWI5	SLC44A1	Solute carrier family 44 member 1	Choline transporter-like protein 1	93	2	0.55	3	4	7
G1SPD1		MTPN	ANK_REP_REGION domain-containing protein			1	0.55	2	5	32
	Q6NZI2	CAVIN1		Caveolae-associated protein 1		4	0.55	7	13	19

G1SQ70	P01023	A2M	Uncharacterized protein	Alpha-2-macroglobulin	78	3	0.55	4	3	3
G1SD49	X6RCK5	DCTN3	Uncharacterized protein	Dynactin subunit 3 (Fragment)	82	3	0.55	4	5	24
B7NZQ6	P31150	GDI1	Rab GDP dissociation inhibitor	Rab GDP dissociation inhibitor alpha	99	2	0.55	16	23	48
G1SKT2	E9PGF6	PHLDB2	PH domain-containing protein	Pleckstrin homology-like domain family B member 2	65	2	0.55	8	11	8
G1TIZ2	Q02809	PLOD1	Procollagen-lysine,2-oxoglutarate 5-dioxygenase 1	Procollagen-lysine,2-oxoglutarate 5-dioxygenase 1	93	2	0.55	26	20	41
G1U974	A0A1C7CYX9	DPYSL2	Amidohydro-rel domain-containing protein	Dihydropyrimidinase-related protein 2	98	2	0.55	19	41	51
G1SE41	Q9NWM8	FKBP14	Peptidylprolyl isomerase	Peptidyl-prolyl cis-trans isomerase FKBP14	94	2	0.55	4	7	23
G1TBW9	Q96FJ2	DYNLL2	Dynein light chain	Dynein light chain 2, cytoplasmic	100	2	0.55	2	12	33
G1SH88	Q8N8S7	ENAH	ENAH, actin regulator	Protein enabled homolog	99	2	0.55	10	2	28
G1T652	P09972	ALDOC	Fructose-bisphosphate aldolase	Fructose-bisphosphate aldolase C	99	2	0.54	9	8	30
G1T0Y9	Q13561	DCTN2	Uncharacterized protein	Dynactin subunit 2	97	3	0.54	10	25	43
G1SIL8	A0A087WWM4	GMPR2	GMP reductase	GMP reductase	90	2	0.54	3	4	13
G1T1V9	P54652	HSPA2	Uncharacterized protein	Heat shock-related 70 kDa protein 2	98	3	0.54	26	38	58
G1U7Y3	E7END4	LOXL3	Uncharacterized protein	Lysyl oxidase homolog 3	87	3	0.54	3	3	5
G1TLD3	Q02818	NUCB1	Nucleobindin 1	Nucleobindin-1	87	2	0.53	17	22	45
G1T156	Q9HB40	SCPEP1	Carboxypeptidase	Retinoid-inducible serine carboxypeptidase	86	2	0.53	11	21	26
G1SWW4		SYNPO	Synaptopodin			1	0.53	3	6	6
G1SW40	E7EMB1	SWAP70	PH domain-containing protein	Switch-associated protein 70	77	2	0.53	7	25	16
Q8WN94		DBI	Acyl-CoA-binding protein			1	0.53	3	5	39
	Q9UHD8	SEPTIN9		Septin-9		4	0.53	10	7	21
G1THW3	A0A3B3IUC0	ITM2B	BRICHOS domain-containing protein	Integral membrane protein 2B	96	2	0.53	5	10	31
G1TAD1	Q7RTS9	DYM	Uncharacterized protein	Dymeclin	97	3	0.53	4	7	13
G1SYX4	P50281	MMP14	Matrix metalloproteinase-14	Matrix metalloproteinase-14	95	2	0.53	5	7	11
G1TAM3		TBCB	CAP-Gly domain-containing protein			1	0.53	2	2	11
	A0A2R8Y3S6	SNX27		Sorting nexin-27 (Fragment)		4	0.53	2	3	20
G1SRI2	Q6P996	PDXDC1	Uncharacterized protein	Pyridoxal-dependent decarboxylase domain-containing protein 1	89	3	0.53	17	35	35
G1TGA8	O75396	SEC22B	Uncharacterized protein	Vesicle-trafficking protein SEC22b	98	3	0.53	10	32	48
G1U826	O75348	ATP6V1G1	V-type proton ATPase subunit G	V-type proton ATPase subunit G 1	97	2	0.53	3	7	39
G1SN43	F8W930	IGF2BP2	Uncharacterized protein	Insulin-like growth factor 2 mRNA-binding protein 2	98	3	0.52	7	6	15
G1TKE7		IQGAP2	IQ motif containing GTPase activating protein 2			1	0.52	4	2	3
G1TVQ3		DYNLRB1	Dynein light chain roadblock			1	0.52	3	4	51
G1U9R0	A8MX94	GSTP1	Uncharacterized protein	Glutathione S-transferase P	71	3	0.52	10	88	49
Q28740		BSG	Basigin			1	0.52	2	4	10
G1SI83	Q99584	S100A13	S_100 domain-containing protein	Protein S100-A13	91	2	0.52	3	5	27
G1SHR7	Q9P0J7	KCMF1	C2H2-type domain-containing protein	E3 ubiquitin-protein ligase KCMF1	99	2	0.52	2	3	9
G1T281		SNTA1	Alpha-1-syntrophin			1	0.52	2	7	8
G1T5E6	O60749	SNX2	PX domain-containing protein	Sorting nexin-2	98	2	0.52	11	11	28
G1SXE6	Q9Y6G9	DYNC1LI1	Uncharacterized protein	Cytoplasmic dynein 1 light intermediate chain 1	94	3	0.52	9	12	26
G1SVE3		PRUNE2	CRAL-TRIO domain-containing protein			1	0.52	4	4	2
	A0A3B3ISV4	VKORC1L1		Vitamin K epoxide reductase complex subunit 1-like protein 1		4	0.51	2	3	9
G1SE57	P18085	ARF4	Uncharacterized protein	ADP-ribosylation factor 4	95	3	0.51	10	23	76
G1T1M9		HBS1L	Tr-type G domain-containing protein			1	0.51	2	4	5
G1SZD6	P27348	YWHAQ	14-3-3 protein theta	14-3-3 protein theta	99	2	0.51	16	159	68
G1SUK4		MPI	Mannose-6-phosphate isomerase			1	0.51	3	3	12
G1SRL4		NAGA	Alpha-galactosidase			1	0.51	14	31	47
	P51911	CNN1		Calponin-1		4	0.51	7	8	33
G1U304		GALNS	Galactosamine (N-acetyl)-6-sulfatase			1	0.51	2	5	7
G1T7T8	P47712	PLA2G4A	Phospholipase A2	Cytosolic phospholipase A2	94	2	0.51	27	140	54
G1SNT8	P08133	ANXA6	Annexin	Annexin A6	96	2	0.51	33	66	48
G1TNL6	P50479	PDLIM4	Uncharacterized protein	PDZ and LIM domain protein 4	86	3	0.51	7	10	33
G1TYN0	Q9BVK6	TMED9	Transmembrane p24 trafficking protein 9	Transmembrane emp24 domain-containing protein 9	80	2	0.50	9	10	35
	P33241	LSP1		Lymphocyte-specific protein 1		4	0.50	3	5	9
G1T545		BTBD9	Lactoylglutathione lyase			1	0.50	4	6	33
G1SD48	A0A0G2JLB3	GBA	Glucosylceramidase	Glucosylceramidase	90	2	0.50	5	9	20
G1SFH5	O00469-2	PLOD2	Fe2OG dioxygenase domain-containing protein		90	2	0.50	30	59	47
G1SMK8	O95816	BAG2	BAG domain-containing protein	BAG family molecular chaperone regulator 2	98	2	0.50	6	9	29
G1T3U5	Q9UDY4	DNAJB4	J domain-containing protein	DnaJ homolog subfamily B member 4	96	2	0.50	4	5	21
G1U8T9	Q96CX2	KCTD12	Potassium channel tetramerization domain containing 12	BTB/POZ domain-containing protein KCTD12	97	2	0.50	3	3	17
G1T473	F8VQE1	LIMA1	LIM zinc-binding domain-containing protein	LIM domain and actin-binding protein 1	85	2	0.50	22	40	38
G1SSW2	B4E0Y9	STK26	Protein kinase domain-containing protein	Serine/threonine-protein kinase 26	95	2	0.50	5	3	19

G1T6W4		ANXA8	Annexin			1	0.50	18	102	66
G1TN33		SEPT8	Septin-type G domain-containing protein			1	0.49	8	11	24
G1SVB5	J3QRN6	MYO1D	Uncharacterized protein	Unconventional myosin-Id	95	3	0.49	15	21	20
G1T6L5	O75368	SH3BGRL	Uncharacterized protein	SH3 domain-binding glutamic acid-rich-like protein	98	3	0.49	5	10	31
G1TER3	A0A2R8Y5M6	BCAP31	Uncharacterized protein	B-cell receptor-associated protein 31	81	3	0.49	11	22	53
G1SLH7	P42566	EPS15	Uncharacterized protein	Epidermal growth factor receptor substrate 15	93	3	0.49	4	6	6
G1TNB4		PARP3	Poly [ADP-ribose] polymerase			1	0.49	2	2	7
G1SWR0	H3BP20	HEXA	Beta-hexosaminidase	Beta-hexosaminidase	85	2	0.48	12	23	36
G1T705	P00387	CYB5R3	NADH-cytochrome b5 reductase	NADH-cytochrome b5 reductase 3	90	2	0.48	12	51	57
G1TEA5	P36959	GMPR	GMP reductase	GMP reductase 1	97	2	0.48	2	3	13
G1SDL3	P54802	NAGLU	Uncharacterized protein	Alpha-N-acetylglucosaminidase	88	3	0.48	15	19	29
G1TK63	P22413	ENPP1	Uncharacterized protein	Ectonucleotide pyrophosphatase/phosphodiesterase family member 1	89	3	0.48	18	26	30
G1SG55	O43491	EPB41L2	FERM domain-containing protein	Band 4.1-like protein 2	90	2	0.48	38	90	43
G1SNP7	E9PGC8	MAP1A	Uncharacterized protein	Microtubule-associated protein 1A	79	3	0.48	6	5	4
G1T2I6	P45877	PPIC	Peptidyl-prolyl cis-trans isomerase	Peptidyl-prolyl cis-trans isomerase C	92	2	0.48	6	24	48
G1TMV1	P60981	DSTN	ADF-H domain-containing protein	Destrin	100	2	0.48	9	74	58
G1U7U3		NME1	Nucleoside diphosphate kinase			1	0.48	7	9	57
A0A494BI13		NAAA	N-acylethanolamine-hydrolyzing acid amidase			1	0.47	2	2	8
	P32119	PRDX2		Peroxiredoxin-2		4	0.47	5	9	31
P00883	P04075	ALDOA	Fructose-bisphosphate aldolase A	Fructose-bisphosphate aldolase A	98	2	0.47	24	114	76
G1SDZ0		CTSA	Carboxypeptidase			1	0.47	5	7	11
G1TK30		CSAD	Cysteine sulfinic acid decarboxylase			1	0.47	4	9	18
G1SDP2	Q96AY3	FKBP10	FK506-binding protein	Peptidyl-prolyl cis-trans isomerase FKBP10	93	2	0.47	17	26	40
G1SX37	Q9Y281	CFL2	Cofilin 2	Cofilin-2	91	2	0.47	8	13	55
G1TS18		BSDC1	BSD domain containing 1			1	0.47	3	5	13
G1TV76	Q9Y4D1	DAAM1	Uncharacterized protein	Disheveled-associated activator of morphogenesis 1	97	3	0.47	4	5	6
G1SYE0		ABHD14B	AB hydrolase-1 domain-containing protein			1	0.47	4	6	21
G1SW82	H0Y9Y3	SYNPO2	PDZ domain-containing protein	Synaptopodin-2 (Fragment)	80	2	0.46	3	4	4
G1T7X6	G3XAI2	LAMB1	Uncharacterized protein	Laminin subunit beta-1	91	3	0.46	3	3	3
G1SH05	P07437	TUBB	Tubulin beta chain	Tubulin beta chain	100	2	0.46	23	125	71
G1TE78	Q15121	PEA15	DED domain-containing protein	Astrocytic phosphoprotein PEA-15	100	2	0.46	5	4	43
G1SXU2	Q96HC4	PDLIM5	Uncharacterized protein	PDZ and LIM domain protein 5	88	3	0.46	10	28	22
G1T6N3		NPC2	ML domain-containing protein			1	0.46	3	6	21
G1U4V6		CACNA2D1	Voltage-dependent calcium channel subunit alpha-2/delta-1			1	0.46	9	3	13
G1T8Z0	P30041	PRDX6	Thioredoxin domain-containing protein	Peroxiredoxin-6	95	2	0.45	15	34	64
G1T013	P48163	ME1	Malic enzyme	NADP-dependent malic enzyme	95	2	0.45	6	19	22
G1SRD9	A0A1C7CYX8	FAM107B	Uncharacterized protein	Protein FAM107B (Fragment)	96	3	0.45	2	2	16
G1SP27	Q16204	CCDC6	Uncharacterized protein	Coiled-coil domain-containing protein 6	93	3	0.45	14	34	31
G1SIB9	P11216	PYGB	Alpha-1,4 glucan phosphorylase	Glycogen phosphorylase, brain form	95	2	0.45	31	23	45
P58776	A7XZE4	TPM2	Tropomyosin beta chain	Beta tropomyosin isoform	94	2	0.45	24	28	63
G1T8B5	Q9H1E3	NUCKS1	Nuclear casein kinase and cyclin dependent kinase substrate 1	Nuclear ubiquitous casein and cyclin-dependent kinase substrate 1	94	2	0.44	2	3	9
G1ST05	Q15120	PDK3	Protein-serine/threonine kinase	[Pyruvate dehydrogenase (acetyl-transferring)] kinase isozyme 3, mitochondrial	99	2	0.44	6	9	26
G1SY88	P55263	ADK	PfkB domain-containing protein	Adenosine kinase	98	2	0.44	8	17	38
G1TKH3		SOD1	Superoxide dismutase [Cu-Zn]			1	0.44	3	121	35
G1SQF2	O43237	DYNC1LI2	Uncharacterized protein	Cytoplasmic dynein 1 light intermediate chain 2	98	3	0.44	9	17	37
G1TA83	P09525	ANXA4	Annexin	Annexin A4	94	2	0.43	13	27	51
G1T9N3	B5MBZ0	EML4	Uncharacterized protein	Echinoderm microtubule-associated protein-like 4	92	3	0.43	4	7	5
G1SF47		SEPT11	Septin-type G domain-containing protein			1	0.43	12	15	34
	Q9NP61	ARFGAP3		ADP-ribosylation factor GTPase-activating protein 3		4	0.43	2	2	4
G1SE87		ARSE	Arylsulfatase E			1	0.43	3	3	7
G1STV0		CAST	Calpastatin			1	0.43	12	21	21
Q6TYA7		GJA1	Gap junction alpha-1 protein			1	0.43	2	4	6
G1SPF5	P18669	PGAM1	Uncharacterized protein	Phosphoglycerate mutase 1	100	3	0.43	13	91	67
	Q16658	FSCN1		Fascin		4	0.43	10	11	23
G1SZX4	Q14571	ITPR2	Uncharacterized protein	Inositol 1,4,5-trisphosphate receptor type 2	97	3	0.43	13	11	9
G1SXR6	O60282	KIF5C	Kinesin-like protein	Kinesin heavy chain isoform 5C	99	2	0.43	20	13	29
G1SHB9	P40121	CAPG	Uncharacterized protein	Macrophage-capping protein	94	3	0.42	9	21	44
G1T3R5		ERO1A	Endoplasmic reticulum oxidoreductase 1 alpha			1	0.42	15	31	34
G1TN08	P37235	HPCAL1	Uncharacterized protein	Hippocalcin-like protein 1	99	3	0.42	6	5	34
	P06753-5	TPM3		Isoform 5 of Tropomyosin alpha-3 chain		4	0.42	21	25	62

G1SQ10	Q9NQT8	KIF13B	Uncharacterized protein	Kinesin-like protein KIF13B	89	3	0.42	13	4	9
P11974-2		PKM	Isoform M2 of Pyruvate kinase PKM			1	0.42	38	469	81
	B4DDF4	CNN2		Calponin		4	0.41	5	5	18
Q6SQH4	P60903	S100a10	Protein S100-A10	Protein S100-A10	100	2	0.41	4	13	35
G1SHL8	P42224	STAT1	Signal transducer and activator of transcription	Signal transducer and activator of transcription 1-alpha/beta	95	2	0.41	13	17	27
G1TRY5	P13797	PLS3	Uncharacterized protein	Plastin-3	100	3	0.41	37	157	77
G1T3J0		SLC26A7	Anion exchange transporter			1	0.41	2	2	7
G1SPQ9	O94875	SORBS2	Uncharacterized protein	Sorbin and SH3 domain-containing protein 2	90	3	0.41	17	23	19
G1T2C4	Q01995	TAGLN	Transgelin	Transgelin	99	2	0.41	11	170	58
G1SM64	Q5T985	ITIH2	Uncharacterized protein	Inter-alpha-trypsin inhibitor heavy chain H2	85	3	0.41	5	4	8
G1T7A8	Q9Y646	CPQ	Peptidase_M28 domain-containing protein	Carboxypeptidase Q	87	2	0.41	2	2	4
G1SL85	A0A1B0GTM3	ASAH1	Uncharacterized protein	Acid ceramidase	81	3	0.40	7	11	27
G1T0J3	E9PDF6	MYO1B	Uncharacterized protein	Unconventional myosin-Ib	96	3	0.40	17	28	22
G1TMM0	A0A087WW43	ITIH3	Inter-alpha-trypsin inhibitor heavy chain H3	Inter-alpha-trypsin inhibitor heavy chain H3	91	2	0.40	4	11	5
G1U7J6		PRKCA	Protein kinase C			1	0.40	2	2	6
G1SYL5	Q7Z4N8	P4HA3	Fe2OG dioxygenase domain-containing protein	Prolyl 4-hydroxylase subunit alpha-3	92	2	0.39	15	40	43
	A0A1B0GV23	CTSD		Cathepsin D		4	0.39	6	20	14
P21195	P07237	P4HB	Protein disulfide-isomerase	Protein disulfide-isomerase	91	2	0.39	34	696	72
	P51178	PLCD1		1-phosphatidylinositol 4,5-bisphosphate phosphodiesterase delta-1		4	0.39	2	3	3
G1TA72		HS1BP3	PX domain-containing protein			1	0.39	2	2	6
	P32418	SLC8A1		Sodium/calcium exchanger 1		4	0.39	2	3	4
	K7EPT8	GFAP		Glial fibrillary acidic protein (Fragment)		4	0.39	3	209	15
G1SRR2	E9PMP7	LMO7	Uncharacterized protein	LIM domain only protein 7 (Fragment)	70	3	0.39	30	41	24
G1SW77	Q8NC51	SERBP1	HABP4_PAI-RBP1 domain-containing protein	Plasminogen activator inhibitor 1 RNA-binding protein	99	2	0.38	7	16	19
G1T2Q8	Q96M27	PRRC1	NTPase_I-T domain-containing protein	Protein PRRC1	90	2	0.38	7	13	22
G1T4N8	Q8TF42	UBASH3B	Uncharacterized protein	Ubiquitin-associated and SH3 domain-containing protein B	98	3	0.38	2	2	5
G1SUZ7		ARSA	Sulfatase domain-containing protein			1	0.38	4	5	18
G1SQR6	Q15417	CNN3	Calponin	Calponin-3	98	2	0.38	15	579	55
G1SKS8	E7EVA0	MAP4	Microtubule-associated protein	Microtubule-associated protein	85	2	0.37	25	48	30
	Q9Y2V2	CARHSP1		Calcium-regulated heat-stable protein 1		4	0.37	2	3	18
G1T0H8	Q01813	PFKP	ATP-dependent 6-phosphofructokinase	ATP-dependent 6-phosphofructokinase, platelet type	93	2	0.36	22	36	42
G1TBQ6	O95292	VAPB	VAMP associated protein B and C	Vesicle-associated membrane protein-associated protein B/C	90	2	0.36	3	3	17
G1T387	Q14247	CTTN	Cortactin	Src substrate cortactin	88	2	0.36	5	3	13
G1T5P0	Q8NDI1	EHBP1	Uncharacterized protein	EH domain-binding protein 1	94	3	0.36	2	4	3
G1SWS9	P08670	VIM	IF rod domain-containing protein	Vimentin	97	2	0.36	50	###	88
G1TBJ4	Q92597	NDRG1	Uncharacterized protein	Protein NDRG1	96	3	0.35	3	4	17
G1TA40	H0YEP5	SMPD1	Sphingomyelin phosphodiesterase	Sphingomyelin phosphodiesterase (Fragment)	78	2	0.35	5	6	14
G1U6H4	O95865	DDAH2	Uncharacterized protein	N(G),N(G)-dimethylarginine dimethylaminohydrolase 2	97	3	0.35	9	10	56
G1SZJ7	A0A2R8YEC9	MANBA	Uncharacterized protein	Beta-mannosidase	75	3	0.34	7	8	11
G1SDH3	E9PIG4	PRCP	Uncharacterized protein	Lysosomal Pro-X carboxypeptidase (Fragment)	89	3	0.33	8	14	30
G1SUY3	F8VQR7	CSRP2	Uncharacterized protein	Cysteine and glycine-rich protein 2	100	3	0.33	6	13	38
G1T2K1	O95340	PAPSS2	Uncharacterized protein	Bifunctional 3~-phosphoadenosine 5~-phosphosulfate synthase 2	94	3	0.33	5	8	14
G1SEF9	P17661	DES	IF rod domain-containing protein	Desmin	99	2	0.33	8	8	10
G1STY4		PLBD2	Phospholipase B-like			1	0.33	5	14	16
G1SX70	Q9NRW1	RAB6B	Uncharacterized protein	Ras-related protein Rab-6B	100	3	0.32	7	2	45
G1T5T8	O00151	PDLIM1	Uncharacterized protein	PDZ and LIM domain protein 1	92	3	0.31	14	30	60
G1SUI9	E7EX44	CALD1	Uncharacterized protein	Caldesmon	84	3	0.31	24	106	39
	Q09666	AHNAK		Neuroblast differentiation-associated protein AHNAK		4	0.30	33	48	6
P00939	P60174	TPI1	Triosephosphate isomerase	Triosephosphate isomerase	93	2	0.30	15	60	72
G1TN29	Q86UU1	PHLDB1	Pleckstrin homology like domain family B member 1	Pleckstrin homology-like domain family B member 1	89	2	0.30	8	4	6
G1SKQ9		SEPT6	Septin-type G domain-containing protein			1	0.30	8	3	22
G1T4F9	E9PR44	CRYAB	Alpha-crystallin B chain	Alpha-crystallin B chain (Fragment)	98	2	0.29	6	13	44
G1SPY1	E9PGM4	GBE1	Aamy domain-containing protein	1,4-alpha-glucan-branching enzyme	93	2	0.28	21	41	42
G1SFG6	A0A0A0MTS2	GPI	Glucose-6-phosphate isomerase	Glucose-6-phosphate isomerase (Fragment)	93	2	0.28	22	134	56
G1U5M4	J3QQS9	SLC16A3	Solute carrier family 16 member 3	Monocarboxylate transporter 4 (Fragment)	92	2	0.28	3	4	6
G1T315	A0A2R8Y2R1	SGCE	CADG domain-containing protein	Epsilon-sarcoglycan (Fragment)	88	2	0.27	2	5	7
G1T7Z6	P00558	PGK1	Phosphoglycerate kinase	Phosphoglycerate kinase 1	99	2	0.26	26	90	72
G1TYY5	Q14847	LASP1	LIM and SH3 domain protein 1	LIM and SH3 domain protein 1	94	2	0.26	6	9	23
	H7BZL4	GPC1		Glypican-1 (Fragment)		4	0.25	5	6	29
G1TBU2		KIAA1217	KIAA1217			1	0.25	2	2	2

G1T8J0	P02461	COL3A1	Uncharacterized protein	Collagen alpha-1(III) chain	92	3	0.24	17	34	15
G1SYN4		PTX3	LamGL domain-containing protein			1	0.23	11	22	29
G1SYJ4	P06733	ENO1	Uncharacterized protein	Alpha-enolase	96	3	0.22	24	144	72
G1T5B6	P12107	COL11A1	Fibrillar collagen NC1 domain-containing protein	Collagen alpha-1(XI) chain	98	2	0.21	29	69	24
G1SYD2		AK4	Adenylate kinase 4, mitochondrial			1	0.21	6	9	41
	P17858	PFKL		ATP-dependent 6-phosphofructokinase, liver type		4	0.20	8	11	12
G1SMS2	Q8WX93	PALLD	Uncharacterized protein	Palladin	87	3	0.20	20	41	20
G1SZ00	P21291	CSRP1	Uncharacterized protein	Cysteine and glycine-rich protein 1	98	3	0.18	9	2	48
G1TAJ3	P00338	LDHA	L-lactate dehydrogenase	L-lactate dehydrogenase A chain	94	2	0.16	21	226	66
G1U634	P08473	MME	Neprilysin	Neprilysin	94	2	0.15	41	5	61
G1SY87		CD200	Ig-like domain-containing protein			1	0.14	2	4	7

A. Percent sequence Identity (rounded to the nearest integer) between the indicated rabbit and human proteins from blastp analysis of the rabbit and human UniProt databases.
B. Database Identification Categories: Category 1, Characterized in the rabbit database only; Category 2, Characterized in both the rabbit and human databases; Category 3, Uncharacterized in the rabbit database but characterized in the human database; Category 4, Characterized in the human database only.
C. Number of unique peptides quantified.
D. Number of peptide intensities summed for protein quantitation.

Supplemental Table S4
Myofibroblasts from Rabbit Cornea & Bone Marrow
Relative Protein Abundance, Rabbit 27
Orbitrap Fusion Lumos Tribrid LC MS/MS iTRAQ Results

Total Proteins Quantified = 2307; Median Protein iTRAQ Ratio =1.002; Mean Protein iTRAQ Ratio = 1.000; Standard Deviation (SD) = 0.398
Yellow = 1SD from the mean; Brown = 2SD from the mean; Green = p ≤ 0.05

Rabbit Accession UniProt	Human Accession UniProt	Gene Symbol	Rabbit Protein Description	Human Protein Description	% identity[A]	Database Identification category[B]	Linear Ratio Cornea/Bone marrow	Peptides[C]	N[D]	% sequence coverage
	P02765	AHSG		Alpha-2-HS-glycoprotein		4	4.30	2	2	5
G1SIW5	Q92820	GGH	Folate gamma-glutamyl hydrolase	Gamma-glutamyl hydrolase	82	2	4.15	6	15	28
G1TEP2		FAM210B	DUF1279 domain-containing protein			1	3.88	2	2	12
	A0A2R8Y7G9	H3.Y		Histone domain-containing protein		4	3.60	2	3	29
	P02458	COL2A1		Collagen alpha-1(II) chain		4	3.45	4	2	5
	A0A024RBG1	NUDT4B		Diphosphoinositol polyphosphate phosphohydrolase NUDT4B		4	3.28	2	2	21
G1T380	Q02388	COL7A1	Uncharacterized protein	Collagen alpha-1(VII) chain	87	3	3.18	16	23	8
G1TUE1		ATP1B1	Sodium/potassium-transporting ATPase subunit beta			1	3.13	4	4	15
G1TAK1		COA3	Coiled-coil_56 domain-containing protein			1	2.85	2	5	19
G1TX53		NDUFA8	NADH dehydrogenase [ubiquinone] 1 alpha subcomplex subunit 8			1	2.81	2	2	9
G1ST69	P20700	LMNB1	Uncharacterized protein	Lamin-B1	98	3	2.77	19	35	42
O19105		SLC1A5	Neutral amino acid transporter B(0)			1	2.76	4	5	14
G1SE74	Q7KZ85	SUPT6H	Transcription elongation factor spt6	Transcription elongation factor SPT6	99	2	2.63	5	7	4
P00389	P16435	POR	NADPH--cytochrome P450 reductase	NADPH--cytochrome P450 reductase	92	2	2.52	27	63	49
P98049		MT-CO2	Cytochrome c oxidase subunit 2			1	2.50	3	5	20
G1SEF1		NDUFC2	NADH dehydrogenase [ubiquinone] 1 subunit C2			1	2.49	2	2	19
G1T4M2	O60264	SMARCA5	Uncharacterized protein	SWI/SNF-related matrix-associated actin-dependent regulator of chromatin subfamily A member 5	100	3	2.42	5	9	8
G1TZQ6		NDUFA10	NADH dehydrogenase [ubiquinone] 1 alpha subcomplex subunit 10, mitochondrial			1	2.38	4	7	19
	I3L3B0	C1QBP		Complement component 1 Q subcomponent-binding protein, mitochondrial		4	2.37	2	14	32
G1SF56		MICAL2	Microtubule associated monooxygenase, calponin and LIM domain containing 2			1	2.36	3	4	6
G1SDA2	P29762	CRABP1	FABP domain-containing protein	Cellular retinoic acid-binding protein 1	99	2	2.34	5	16	41
G1SGP1	P31930	UQCRC1	Uncharacterized protein	Cytochrome b-c1 complex subunit 1, mitochondrial	93	3	2.32	12	16	42
G1SJF4	A0A0A0MR51	FADS1	Fatty acid desaturase 1	Acyl-CoA (8-3)-desaturase	93	2	2.31	3	5	6
G1U4P8	Q9BXN1	ASPN	LRRNT domain-containing protein	Asporin	88	2	2.31	5	6	19
G1SNX5	B4DY09	ILF2	Interleukin enhancer binding factor 2	Interleukin enhancer-binding factor 2	100	2	2.30	17	62	58
	Q10570	CPSF1		Cleavage and polyadenylation specificity factor subunit 1		4	2.29	2	2	2
G1SI26	Q4VC31	CCDC58	Uncharacterized protein	Coiled-coil domain-containing protein 58	95	3	2.29	3	7	26
G1SFC6	A0A087WV29	NAT10	RNA cytidine acetyltransferase	RNA cytidine acetyltransferase	96	2	2.28	6	3	11
G1SXI9		COX6B1	Cytochrome c oxidase subunit			1	2.27	2	3	29
G1T1L7	A0A087WWS1	THOC1	Death domain-containing protein	THO complex subunit 1	98	2	2.27	3	3	10
G1SXN0		PES1	Pescadillo homolog			1	2.23	2	3	6
P35953	P98155	VLDLR	Very low-density lipoprotein receptor	Very low-density lipoprotein receptor	97	2	2.22	10	13	16
G1SUY2	P05091	ALDH2	Aldedh domain-containing protein	Aldehyde dehydrogenase, mitochondrial	91	2	2.22	12	21	36
G1T1P3	Q9H845	ACAD9	Uncharacterized protein	Acyl-CoA dehydrogenase family member 9, mitochondrial	88	3	2.20	9	12	25
G1T3Y8	P10809	HSPD1	Uncharacterized protein	60 kDa heat shock protein, mitochondrial	99	3	2.20	34	43	75
G1T017		SLC1A4	Solute carrier family 1 member 4			1	2.20	3	6	9
G1SI79	P51991	HNRNPA3	Uncharacterized protein	Heterogeneous nuclear ribonucleoprotein A3	100	3	2.18	8	22	25
G1SN37	O43172	PRPF4	WD_REPEATS_REGION domain-containing protein	U4/U6 small nuclear ribonucleoprotein Prp4	99	2	2.18	3	3	9
G1SCY8	Q9UKD2	MRTO4	Ribosome assembly factor mrt4	mRNA turnover protein 4 homolog	96	2	2.17	2	3	10
G1TPN3	D6R9P3	HNRNPAB	Uncharacterized protein	Heterogeneous nuclear ribonucleoprotein A/B	96	3	2.17	9	74	37
G1SEF5	Q8NAV1	PRPF38A	PRP38_assoc domain-containing protein	Pre-mRNA-splicing factor 38A	100	2	2.17	2	4	9
G1SKW4		KIDINS220	Kinase D interacting substrate 220			1	2.16	8	6	7
G1SG11		COX4I1	Cytochrome c oxidase subunit 4 isoform 1, mitochondrial			1	2.16	3	2	21
G1T6T0	J3KNJ3	NAALAD2	Uncharacterized protein	N-acetylated-alpha-linked acidic dipeptidase 2	89	3	2.13	11	14	23
G1TLW3	J3KTA4	DDX5	Uncharacterized protein	Probable ATP-dependent RNA helicase DDX5	96	3	2.13	21	29	42
G1SIJ7	Q9NR30	DDX21	Uncharacterized protein	Nucleolar RNA helicase 2	89	3	2.13	16	27	24
G1SNS1	Q14692	BMS1	Bms1-type G domain-containing protein	Ribosome biogenesis protein BMS1 homolog	88	2	2.12	5	5	7
U3KMH9	P40926	MDH2	Malate dehydrogenase	Malate dehydrogenase, mitochondrial	94	2	2.12	15	64	60
G1SJW7	J3QLE5	SNRPN	Small nuclear ribonucleoprotein-associated protein	Small nuclear ribonucleoprotein-associated protein N (Fragment)	100	2	2.10	6	10	19
G1TD41	G8JLB6	HNRNPH1	Uncharacterized protein	Heterogeneous nuclear ribonucleoprotein H	98	3	2.10	12	11	39
G1TM60		NDUFA9	Epimerase domain-containing protein			1	2.09	6	10	20
G1TL80	Q9Y3B4	SF3B6	RRM domain-containing protein	Splicing factor 3B subunit 6	100	2	2.09	2	6	21

G1T9F6		NDUFA6	NADH:ubiquinone oxidoreductase subunit A6			1	2.08	2	2	19
G1SQB1	P22033	MMUT	B12-binding domain-containing protein	Methylmalonyl-CoA mutase, mitochondrial	96	2	2.08	2	2	4
G1T594	G0XQ39	STIM1	Stromal interaction molecule 1	STIM1L	98	2	2.08	2	3	3
G1SYC1	P83111	LACTB	Uncharacterized protein	Serine beta-lactamase-like protein LACTB, mitochondrial	89	3	2.07	4	7	12
	H0Y6E7	RBMX		RNA-binding motif protein, X chromosome (Fragment)		4	2.07	3	9	12
G1SIM3	O95831	AIFM1	Uncharacterized protein	Apoptosis-inducing factor 1, mitochondrial	96	3	2.06	8	12	22
G1SKK1		DUT	dUTPase domain-containing protein			1	2.05	2	5	14
G1TW43	J3QSU6	TNC	Uncharacterized protein	Tenascin	74	3	2.05	6	6	3
G1SK29		MTIF2	Tr-type G domain-containing protein			1	2.05	3	4	8
G1TAR0		RPF2	Brix domain-containing protein			1	2.05	3	3	14
G1TID3		ITPRIP	Inositol 1,4,5-trisphosphate receptor interacting protein			1	2.05	2	3	5
G1TYH7	Q9HDC9	APMAP	Adipocyte plasma membrane associated protein	Adipocyte plasma membrane-associated protein	94	2	2.04	12	29	34
G1SE36	R4GMU1	H6PD	GDH/6PGL endoplasmic bifunctional protein	GDH/6PGL endoplasmic bifunctional protein	85	2	2.04	8	5	15
G1SP32	Q6NVY1	HIBCH	3-hydroxyisobutyryl-CoA hydrolase, mitochondrial	3-hydroxyisobutyryl-CoA hydrolase, mitochondrial	84	2	2.04	8	9	25
G1TX84	Q9Y5J1	UTP18	WD_REPEATS_REGION domain-containing protein	U3 small nucleolar RNA-associated protein 18 homolog	89	2	2.04	4	4	12
G1SXR7		SLIRP	RRM domain-containing protein			1	2.03	3	6	34
G1U5U0	Q9Y2X3	NOP58	Nop domain-containing protein	Nucleolar protein 58	92	2	2.03	11	4	29
G1SYC5	Q9NQZ2	UTP3	Sas10 domain-containing protein	Something about silencing protein 10	82	2	2.02	3	3	8
G1SWA6	A0A2R8Y4T4	SMARCE1	HMG box domain-containing protein	SWI/SNF-related matrix-associated actin-dependent regulator of chromatin subfamily E member 1	95	2	2.02	3	3	9
G1T9U7	Q8TD30	GPT2	Glutamic--pyruvic transaminase 2	Alanine aminotransferase 2	97	2	2.02	5	2	21
G1SLR8	Q9NZI8	IGF2BP1	Uncharacterized protein	Insulin-like growth factor 2 mRNA-binding protein 1	99	3	2.02	3	2	7
G1T2K5	I3L1L3	MYBBP1A	Uncharacterized protein	Myb-binding protein 1A (Fragment)	69	3	2.02	34	84	37
G1SZ44	B8ZZL8	HSPE1	Uncharacterized protein	10 kDa heat shock protein, mitochondrial	100	3	2.02	7	7	58
G1SUP9	P46087	NOP2	SAM_MT_RSMB_NOP domain-containing protein	Probable 28S rRNA (cytosine(4447)-C(5))-methyltransferase	77	2	2.02	6	5	12
G1T6E6		NOC3L	Nucleolar complex protein 3 homolog			1	2.01	4	4	9
G1SK42	P21980	TGM2	TGc domain-containing protein	Protein-glutamine gamma-glutamyltransferase 2	87	2	2.01	22	66	51
G1SSJ7	P35232	PHB	PHB domain-containing protein	Prohibitin	100	2	2.00	17	47	81
G1SL97	Q15397	PUM3	PUM-HD domain-containing protein	Pumilio homolog 3	91	2	1.99	6	5	16
G1SKM2	P35555	FBN1	Uncharacterized protein	Fibrillin-1	97	3	1.99	6	6	2
G1SGH2		MRPL15	Ribosomal_L18e/L15P domain-containing protein			1	1.99	2	2	9
G1SM50	Q15427	SF3B4	Uncharacterized protein	Splicing factor 3B subunit 4	100	3	1.99	4	6	18
G1T0U4	Q9UIG0	BAZ1B	Bromodomain adjacent to zinc finger domain 1B	Tyrosine-protein kinase BAZ1B	94	2	1.97	4	2	4
G1SIF2	Q16822	PCK2	Uncharacterized protein	Phosphoenolpyruvate carboxykinase [GTP], mitochondrial	95	3	1.97	23	74	47
G1TKC9	Q15582	TGFBI	Transforming growth factor-beta-induced protein ig-h3	Transforming growth factor-beta-induced protein ig-h3	93	2	1.97	5	5	16
G1SZE8		UTP20	DRIM domain-containing protein			1	1.97	7	11	4
G1T5J9	O75306	NDUFS2	Complex1_49kDa domain-containing protein	NADH dehydrogenase [ubiquinone] iron-sulfur protein 2, mitochondrial	94	2	1.96	6	10	23
G1T359	P28331	NDUFS1	Uncharacterized protein	NADH-ubiquinone oxidoreductase 75 kDa subunit, mitochondrial	98	3	1.96	18	27	37
G1T8M9	P42704	LRPPRC	PPR_long domain-containing protein	Leucine-rich PPR motif-containing protein, mitochondrial	81	2	1.96	51	85	47
G1SQ07		DTYMK	Thymidylate_kin domain-containing protein			1	1.95	2	2	10
G1TBS4	P20020	ATP2B1	Calcium-transporting ATPase	Plasma membrane calcium-transporting ATPase 1	97	2	1.95	6	5	7
G1SXW0	P43304	GPD2	Glycerol-3-phosphate dehydrogenase	Glycerol-3-phosphate dehydrogenase, mitochondrial	96	2	1.95	16	25	34
	Q16394	EXT1		Exostosin-1		4	1.94	2	3	5
G1T0I5	Q5JRX3	PITRM1	M16C_associated domain-containing protein	Presequence protease, mitochondrial	89	2	1.94	27	42	37
G1TD99	A0A1B0GUX9	PCCA	Uncharacterized protein	Propionyl-CoA carboxylase alpha chain, mitochondrial (Fragment)	96	3	1.94	8	10	15
G1SDW8	H0Y2P0	CD44	Link domain-containing protein	CD44 antigen (Fragment)	91	2	1.94	10	18	17
G1TD26	D6RAA6	TMEM33	Uncharacterized protein	Transmembrane protein 33 (Fragment)	99	3	1.93	3	6	12
G1T4L3	Q01780	EXOSC10	HRDC domain-containing protein	Exosome component 10	89	2	1.93	2	4	4
G1TE69	J3KTL2	SRSF1	Uncharacterized protein	Serine/arginine-rich-splicing factor 1	100	3	1.93	6	10	25
G1SRN1		LAS1L	LAS1 like, ribosome biogenesis factor			1	1.93	4	3	11
G1SLK4	Q8WVX9	FAR1	Fatty acyl-CoA reductase	Fatty acyl-CoA reductase 1	93	2	1.93	2	2	7
G1TV19	H3BPE7	FUS	FUS RNA binding protein	RNA-binding protein FUS	80	2	1.92	5	3	9
G1T701		DBT	Dihydrolipoamide acetyltransferase component of pyruvate dehydrogenase complex			1	1.92	2	3	5
	P31040	SDHA		Succinate dehydrogenase [ubiquinone] flavoprotein subunit, mitochondrial		4	1.92	13	7	34
G1TVK1		NOTCH2	Notch 2			1	1.91	4	5	3
G1SI76		MRPL43	L51_S25_CI-B8 domain-containing protein			1	1.91	3	3	19
G1TSS7	P05106	ITGB3	Integrin beta	Integrin beta-3	95	2	1.90	5	5	9
G1SIJ6	Q13308	PTK7	Protein tyrosine kinase 7 (inactive)	Inactive tyrosine-protein kinase 7	93	2	1.90	28	57	39
G1SWA0		RIPOR1	RHO family interacting cell polarization regulator 1			1	1.90	3	3	5
G1T1B6	Q16832	DDR2	Uncharacterized protein	Discoidin domain-containing receptor 2	97	3	1.90	5	5	9

G1SXC8		NXF1	Nuclear RNA export factor 1			1	1.89	3	3	8
G1TNV7	O75600	GCAT	Glycine C-acetyltransferase	2-amino-3-ketobutyrate coenzyme A ligase, mitochondrial	94	2	1.88	7	5	27
G1T0Q6	Q9UQE7	SMC3	Structural maintenance of chromosomes protein	Structural maintenance of chromosomes protein 3	100	2	1.88	8	5	9
G1TI40	P62316	SNRPD2	Small nuclear ribonucleoprotein Sm D2	Small nuclear ribonucleoprotein Sm D2	100	2	1.88	6	15	48
G1T2A9	Q14315	FLNC	Uncharacterized protein	Filamin-C	90	3	1.88	73	96	40
G1SIQ9	Q9GZL7	WDR12	Ribosome biogenesis protein WDR12	Ribosome biogenesis protein WDR12	97	2	1.88	3	2	15
	P38919	EIF4A3		Eukaryotic initiation factor 4A-III		4	1.88	13	15	40
G1TX78	Q15388	TOMM20	Uncharacterized protein	Mitochondrial import receptor subunit TOM20 homolog	100	3	1.88	3	26	28
G1TKJ4	P61601	NCALD	Uncharacterized protein	Neurocalcin-delta	100	3	1.88	6	7	38
G1SLK6	J3KMX2	SMARCD2	SWIB domain-containing protein	SWI/SNF-related matrix-associated actin-dependent regulator of chromatin subfamily D member 2	98	2	1.88	3	2	10
G1T4A5	P02452	COL1A1	Collagen alpha-1(I) chain	Collagen alpha-1(I) chain	91	2	1.88	34	27	45
G1T958		EBP	EXPERA domain-containing protein			1	1.87	2	3	8
	Q9P0J1	PDP1		[Pyruvate dehydrogenase [acetyl-transferring]]-phosphatase 1, mitochondrial		4	1.87	2	3	5
G1SN06	O60488	ACSL4	AMP-binding domain-containing protein	Long-chain-fatty-acid--CoA ligase 4	98	2	1.87	11	16	21
G1SRF7	P38646	HSPA9	Uncharacterized protein	Stress-70 protein, mitochondrial	99	3	1.86	36	406	62
G1T5I9	F8WJN3	CPSF6	RRM domain-containing protein	Cleavage and polyadenylation-specificity factor subunit 6	99	2	1.86	3	3	9
G1SDL7		ARG2	Arginase			1	1.85	2	2	8
G1SL16	A0A0B4J1Z1	SRSF7	Uncharacterized protein	Serine/arginine-rich-splicing factor 7	100	3	1.85	4	4	31
G1TA04	Q9UMS4	PRPF19	Uncharacterized protein	Pre-mRNA-processing factor 19	96	3	1.84	5	9	16
G1TJW3	Q15233	NONO	Uncharacterized protein	Non-POU domain-containing octamer-binding protein	99	3	1.84	12	39	33
G1SR13	Q9UJZ1	STOML2	PHB domain-containing protein	Stomatin-like protein 2, mitochondrial	96	2	1.83	15	25	54
	H0Y2W2	ATAD3A		ATPase family AAA domain-containing protein 3A (Fragment)		4	1.83	5	10	12
U3KPG6		ICAM1	Intercellular adhesion molecule 1			1	1.83	8	15	20
	Q9BXP5	SRRT		Serrate RNA effector molecule homolog		4	1.83	7	4	7
P12345	P00505	GOT2	Aspartate aminotransferase, mitochondrial	Aspartate aminotransferase, mitochondrial	94	2	1.83	18	90	46
G1SP02	A8MT40	PDPR	Uncharacterized protein	Pyruvate dehydrogenase phosphatase regulatory subunit, mitochondrial	95	3	1.83	4	4	7
G1THH7		SUN2	SUN domain-containing protein			1	1.82	7	8	16
G1SF95	H0Y8G5	HNRNPD	Heterogeneous nuclear ribonucleoprotein D	Heterogeneous nuclear ribonucleoprotein D0 (Fragment)	99	2	1.82	7	15	26
	Q13247	SRSF6		Serine/arginine-rich splicing factor 6		4	1.82	3	4	10
U3KMD4	Q8IYU8	MICU2	Uncharacterized protein	Calcium uptake protein 2, mitochondrial	83	3	1.82	5	3	25
G1SPW1	Q29RF7	PDS5A	Uncharacterized protein	Sister chromatid cohesion protein PDS5 homolog A	99	3	1.82	3	3	3
G1TNT7		DDX27	DEAD-box helicase 27			1	1.82	2	2	3
A0A140TAV7	J3KPF3	SLC3A2	4F2 cell-surface antigen heavy chain	4F2 cell-surface antigen heavy chain	81	2	1.81	20	100	57
G1T5I0		SLC7A1	Solute carrier family 7 member 1			1	1.81	4	6	11
G1SMB3	Q9NV31	IMP3	S4 RNA-binding domain-containing protein	U3 small nucleolar ribonucleoprotein protein IMP3	99	2	1.81	2	2	15
G1TEN4	Q9HD33	MRPL47	Uncharacterized protein	39S ribosomal protein L47, mitochondrial	79	3	1.80	5	7	19
G1SSK8	B4DJV2	CS	Citrate synthase	Citrate synthase	96	2	1.80	18	90	53
	O95202	LETM1		Mitochondrial proton/calcium exchanger protein		4	1.80	7	13	10
G1T9V1	P11177	PDHB	Pyruvate dehydrogenase E1 component subunit beta	Pyruvate dehydrogenase E1 component subunit beta, mitochondrial	97	2	1.80	10	28	39
G1T970	P30038	ALDH4A1	Multifunctional fusion protein	Delta-1-pyrroline-5-carboxylate dehydrogenase, mitochondrial	91	2	1.79	11	22	31
G1TRL8	J3QLI9	SNRPD1	Small nuclear ribonucleoprotein Sm D1	Small nuclear ribonucleoprotein Sm D1	100	2	1.79	3	8	28
G1SPY7	O14807	MRAS	Uncharacterized protein	Ras-related protein M-Ras	100	3	1.79	2	3	16
G1SQU6	O75489	NDUFS3	Complex1_30kDa domain-containing protein	NADH dehydrogenase [ubiquinone] iron-sulfur protein 3, mitochondrial	91	2	1.78	4	4	21
G1SHL9	Q15006	EMC2	TPR_REGION domain-containing protein	ER membrane protein complex subunit 2	99	2	1.78	6	10	34
G1SQ54	A0A087WT44	HMOX2	Heme oxygenase	Heme oxygenase 2	89	2	1.78	7	10	33
G1TZB9	Q13595	TRA2A	RRM domain-containing protein	Transformer-2 protein homolog alpha	100	2	1.78	3	2	16
G1TB57		MRPS35	MRP-S28 domain-containing protein			1	1.78	3	4	16
G1SDX3	E9PCY5	TOP2B	DNA topoisomerase 2	DNA topoisomerase 2 (Fragment)	99	2	1.78	14	21	12
G1U018		IGF2R	Insulin like growth factor 2 receptor			1	1.77	9	9	4
	A0A0C4DGG8	CCAR1		Cell division cycle and apoptosis regulator protein 1 (Fragment)		4	1.77	3	3	5
G1SD44	Q13505	MTX1	Uncharacterized protein	Metaxin-1	84	3	1.76	6	7	21
G1SUP4	J3QRS9	ZNF207	Uncharacterized protein	BUB3-interacting and GLEBS motif-containing protein ZNF207	100	3	1.76	3	4	8
G1SW61	Q2TAY7	SMU1	Uncharacterized protein	WD40 repeat-containing protein SMU1	100	3	1.75	7	11	17
G1SMM7	P62318	SNRPD3	Small nuclear ribonucleoprotein Sm D3	Small nuclear ribonucleoprotein Sm D3	100	2	1.75	3	4	29
G1U2E6	J3KPX7	PHB2	PHB domain-containing protein	Prohibitin-2	99	2	1.75	17	108	61
G1T7V5	E9PEX6	DLD	Dihydrolipoyl dehydrogenase	Dihydrolipoyl dehydrogenase	91	2	1.75	11	3	32
G1TMU2	P52597	HNRNPF	Uncharacterized protein	Heterogeneous nuclear ribonucleoprotein F	99	3	1.75	11	172	44
G1TTK6	E7ETT1	PCCB	Uncharacterized protein	Propionyl-CoA carboxylase beta chain, mitochondrial	86	3	1.75	9	14	28
G1SUQ9	P11387	TOP1	DNA topoisomerase I	DNA topoisomerase 1	97	2	1.75	7	16	10

G1SMI7	V9GYL9	DAP3	Uncharacterized protein	28S ribosomal protein S29, mitochondrial (Fragment)	86	3	1.74	8	7	28
G1T8P3	P08621	SNRNP70	Small nuclear ribonucleoprotein U1 subunit 70	U1 small nuclear ribonucleoprotein 70 kDa	92	2	1.74	5	8	11
	Q9Y2Z2	MTO1		Protein MTO1 homolog, mitochondrial		4	1.74	2	3	4
G1U6N8	Q14980	NUMA1	Nuclear mitotic apparatus protein 1	Nuclear mitotic apparatus protein 1	90	2	1.74	21	24	15
G1STJ3		PPIE	Peptidyl-prolyl cis-trans isomerase E			1	1.74	2	2	13
G1T6T8	O94906	PRPF6	Uncharacterized protein	Pre-mRNA-processing factor 6	96	3	1.74	5	6	7
G1T617	P49756	RBM25	Uncharacterized protein	RNA-binding protein 25	99	3	1.74	5	4	9
G1STC6		WDR3	WD_REPEATS_REGION domain-containing protein			1	1.74	3	3	5
G1SMY1	A0A1W2PQ5 1	DDX17	Uncharacterized protein	Probable ATP-dependent RNA helicase DDX17	99	3	1.73	20	25	38
G1SSF2		ENG	Endoglin			1	1.73	5	9	12
G1SRY1	Q8TCS8	PNPT1	S1 motif domain-containing protein	Polyribonucleotide nucleotidyltransferase 1, mitochondrial	94	2	1.73	7	9	14
G1SFE6	P08579	SNRPB2	Uncharacterized protein	U2 small nuclear ribonucleoprotein B~~	96	3	1.73	3	2	16
G1T7H4	Q8TDN6	BRIX1	Brix domain-containing protein	Ribosome biogenesis protein BRX1 homolog	93	2	1.73	4	6	13
G1SY50	E7EPT4	NDUFV2	Uncharacterized protein	NADH dehydrogenase [ubiquinone] flavoprotein 2, mitochondrial	98	3	1.73	4	9	21
G1SKI8	Q6DKI1	RPL7L1	Uncharacterized protein	60S ribosomal protein L7-like 1	85	3	1.73	3	5	17
G1TGK9	Q08211	DHX9	Uncharacterized protein	ATP-dependent RNA helicase A	93	3	1.72	21	32	31
G1TIR7	O95777	LSM8	U6 snRNA-associated Sm-like protein LSm8	U6 snRNA-associated Sm-like protein LSm8	99	2	1.72	3	50	52
G1TWL0	P22626	HNRNPA2B 1	Uncharacterized protein	Heterogeneous nuclear ribonucleoproteins A2/B1	99	3	1.72	10	25	28
G1T9T5	P36957	DLST	Lipoyl-binding domain-containing protein	Dihydrolipoyllysine-residue succinyltransferase component of 2-oxoglutarate dehydrogenase complex, mitochondrial	91	2	1.72	12	29	35
G1SNR2	Q969S9	GFM2	Ribosome-releasing factor 2, mitochondrial	Ribosome-releasing factor 2, mitochondrial	88	2	1.72	4	4	10
G1SL60	Q15393	SF3B3	CPSF_A domain-containing protein	Splicing factor 3B subunit 3	98	2	1.72	17	25	20
G1T6I6	Q12906	ILF3	Interleukin enhancer binding factor 3	Interleukin enhancer-binding factor 3	96	2	1.71	21	30	33
G1T116	Q96TA2	YME1L1	AAA domain-containing protein	ATP-dependent zinc metalloprotease YME1L1	89	2	1.71	4	5	9
G1T8H1	O95573	ACSL3	AMP-binding domain-containing protein	Long-chain-fatty-acid--CoA ligase 3	96	2	1.71	7	5	14
G1T3N1	Q9NVP1	DDX18	RNA helicase	ATP-dependent RNA helicase DDX18	87	2	1.71	6	7	12
G1T0H0	Q9NX46	ADPRHL2	ADP-ribosylhydrolase like 2	ADP-ribose glycohydrolase ARH3	95	2	1.70	3	3	12
G1SD24	A0A494BZU6	PARN	R3H domain-containing protein	Poly(A)-specific ribonuclease PARN (Fragment)	96	2	1.70	2	2	5
G1TI97	Q13769	THOC5	Uncharacterized protein	THO complex subunit 5 homolog	97	3	1.70	3	3	8
	P43897	TSFM		Elongation factor Ts, mitochondrial		4	1.70	3	5	17
G1TUX2	A2A274	ACO2	Aconitate hydratase, mitochondrial	Aconitate hydratase, mitochondrial	94	2	1.70	25	51	42
G1SME4	Q9BWF3	RBM4	RNA-binding protein 4	RNA-binding protein 4	99	2	1.69	7	7	20
G1SWF3		DHODH	Dihydroorotate dehydrogenase (quinone), mitochondrial			1	1.69	4	4	15
G1SIP2	A0A3B3ITJ4	HNRNPL	Uncharacterized protein	Heterogeneous nuclear ribonucleoprotein L (Fragment)	93	3	1.69	18	33	53
G1SST6		ARMC10	Arm_2 domain-containing protein			1	1.69	2	3	12
G1T3S2	P54753	EPHB3	Uncharacterized protein	Ephrin type-B receptor 3	98	3	1.69	9	15	15
G1T5H2	Q9H0D6	XRN2	5~-3~ exoribonuclease	5~-3~ exoribonuclease 2	98	2	1.68	8	10	14
G1SVB6	P00367	GLUD1	Glutamate dehydrogenase	Glutamate dehydrogenase 1, mitochondrial	98	2	1.68	23	163	64
G1ST81	Q9P035	HACD3	Very-long-chain (3R)-3-hydroxyacyl-CoA dehydratase	Very-long-chain (3R)-3-hydroxyacyl-CoA dehydratase 3	96	2	1.68	4	7	18
G1T2Y5	P12270	TPR	TPR_MLP1_2 domain-containing protein	Nucleoprotein TPR	97	2	1.68	26	35	14
G1TTM6	Q99797	MIPEP	Peptidase_M3 domain-containing protein	Mitochondrial intermediate peptidase	93	2	1.68	6	5	15
G1T5A6	Q5T4D3	TMTC4	Transmembrane and tetratricopeptide repeat containing 4	Protein O-mannosyl-transferase TMTC4	92	2	1.68	5	4	11
G1SQF9	Q8WXF1	PSPC1	Uncharacterized protein	Paraspeckle component 1	98	3	1.68	6	6	15
G1SHL0		SBNO1	Strawberry notch homolog 1			1	1.68	2	2	3
G1SPH7	Q9P2I0	CPSF2	Cleavage and polyadenylation specificity factor subunit 2	Cleavage and polyadenylation specificity factor subunit 2	99	2	1.68	4	6	6
	A0A3B3IRT8	SSR1		Translocon-associated protein subunit alpha		4	1.68	4	79	21
G1TU85		FADS3	Cytochrome b5 heme-binding domain-containing protein			1	1.68	2	2	10
G1STI3	Q07666	KHDRBS1	KH domain-containing protein	KH domain-containing, RNA-binding, signal transduction-associated protein 1	99	2	1.68	4	10	12
G1T5L3	Q9H9J2	MRPL44	Uncharacterized protein	39S ribosomal protein L44, mitochondrial	90	3	1.67	3	4	15
G1SI37	D6RF87	ACSF2	Uncharacterized protein	Acyl-CoA synthetase family member 2, mitochondrial (Fragment)	82	3	1.67	18	32	51
G1TQJ5		DEK	SAP domain-containing protein			1	1.67	2	2	6
	P08574	CYC1		Cytochrome c1, heme protein, mitochondrial		4	1.67	4	6	16
G1TDH4	P30048	PRDX3	Thioredoxin domain-containing protein	Thioredoxin-dependent peroxide reductase, mitochondrial	86	2	1.67	8	23	36
G1TMU1	Q13151	HNRNPA0	Uncharacterized protein	Heterogeneous nuclear ribonucleoprotein A0	78	3	1.67	2	3	10
G1SUA4	J3KS05	CBX1	Uncharacterized protein	Chromobox protein homolog 1 (Fragment)	98	3	1.67	4	12	39
	Q9Y4W6	AFG3L2		AFG3-like protein 2		4	1.66	11	19	19
G1T3X2	H7BZW6	SAP18	Histone deacetylase complex subunit SAP18	Histone deacetylase complex subunit SAP18 (Fragment)	98	2	1.66	2	3	15
G1TZN7		COX5A	Cytochrome c oxidase subunit 5A			1	1.66	2	2	7
G1SIW1	O43143	DHX15	Uncharacterized protein	Pre-mRNA-splicing factor ATP-dependent RNA helicase DHX15	99	3	1.66	14	22	24
G1SWW7	B4DJK0	SRSF5	Uncharacterized protein	Serine/arginine-rich-splicing factor 5	100	3	1.66	2	4	8

O79428		MT-ND2	NADH-ubiquinone oxidoreductase chain 2			1	1.65	2	2	10
G1TXA3	P82921	MRPS21	Uncharacterized protein	28S ribosomal protein S21, mitochondrial	93	3	1.65	3	3	38
G1TS78	A0A075B6F6	HM13	Uncharacterized protein	Minor histocompatibility antigen H13 (Fragment)	84	3	1.65	7	18	21
	G3V0I5	NDUFV1		NADH dehydrogenase [ubiquinone] flavoprotein 1, mitochondrial		4	1.65	6	9	27
G1T923	A0A1B0GTB0	ATP6AP2	Uncharacterized protein	Renin receptor (Fragment)	84	3	1.64	4	4	21
G1TWD8		SELENOO	Selenoprotein O			1	1.64	3	4	18
G1T4K8	P32189	GK	Uncharacterized protein	Glycerol kinase	97	3	1.64	11	17	23
G1T6L0	Q5T3Q7	HEATR1	BP28CT domain-containing protein	HEAT repeat-containing protein 1	93	2	1.64	11	11	10
G1T3A6	A0A1W2PQH3	ME2	Malic enzyme	Malic enzyme	92	2	1.64	16	14	40
G1TVN1	I3L4X2	ABCC1	ATP binding cassette subfamily C member 1	Multidrug resistance-associated protein 1 (Fragment)	91	2	1.64	16	39	16
G1TTB5	A0A0D9SEM4	SRSF4	Serine and arginine rich splicing factor 4	Serine/arginine-rich-splicing factor 4 (Fragment)	75	2	1.64	3	2	8
G1TH59	Q14498	RBM39	Uncharacterized protein	RNA-binding protein 39	91	3	1.64	7	9	21
G1T2G3	Q5VTR2	RNF20	E3 ubiquitin protein ligase	E3 ubiquitin-protein ligase BRE1A	98	2	1.64	6	3	10
G1T011	Q9Y5B9	SUPT16H	Uncharacterized protein	FACT complex subunit SPT16	100	3	1.64	7	7	10
	Q9NTI5-2	PDS5B		Isoform 2 of Sister chromatid cohesion protein PDS5 homolog B		4	1.63	2	2	2
G1SJQ6	O00519	FAAH	Amidase domain-containing protein	Fatty-acid amide hydrolase 1	91	2	1.63	4	6	11
G1SNV4	Q12874	SF3A3	Matrin-type domain-containing protein	Splicing factor 3A subunit 3	100	2	1.63	11	21	33
G1TAE2	Q15717	ELAVL1	ELAV-like protein	ELAV-like protein 1	99	2	1.63	10	43	32
G1TE50	Q9Y221	NIP7	60S ribosome subunit biogenesis protein NIP7 homolog	60S ribosome subunit biogenesis protein NIP7 homolog	97	2	1.63	2	2	16
G1SSL2	F8VXJ7	CNPY2	Saposin B-type domain-containing protein	Protein canopy homolog 2 (Fragment)	99	2	1.63	7	23	56
G1STS0	Q9Y2S7	POLDIP2	ApaG domain-containing protein	Polymerase delta-interacting protein 2	98	2	1.63	6	6	26
B7NZG9	Q8N3U4	STAG2	Stromal antigen 2 isoform a (Predicted)	Cohesin subunit SA-2	97	2	1.62	5	6	8
G1TES6	Q99714	HSD17B10	Uncharacterized protein	3-hydroxyacyl-CoA dehydrogenase type-2	92	3	1.62	13	65	84
	K7EIE8	MBD3		Methyl-CpG binding domain protein 3, isoform CRA_b		4	1.62	2	2	14
G1SRH7	P84103	SRSF3	RRM domain-containing protein	Serine/arginine-rich splicing factor 3	100	2	1.62	4	5	24
G1SFG0	A0A087WVP1	FAT1	Uncharacterized protein	Protocadherin Fat 1	92	3	1.62	11	12	4
	F6RGN5	SLC25A10		Mitochondrial dicarboxylate carrier		4	1.62	3	6	11
G1T813	P08559	PDHA1	Pyruvate dehydrogenase E1 component subunit alpha	Pyruvate dehydrogenase E1 component subunit alpha, somatic form, mitochondrial	99	2	1.62	12	20	35
G1SY30	O75643	SNRNP200	Uncharacterized protein	U5 small nuclear ribonucleoprotein 200 kDa helicase	100	3	1.62	39	59	30
G1TBR6	Q69YN4	VIRMA	VIR_N domain-containing protein	Protein virilizer homolog	98	2	1.62	5	4	4
G1SFQ3		MRPL2	Ribosomal_L2_C domain-containing protein			1	1.61	3	5	19
G1TC48	A0A0A0MRA5	HNRNPUL1	Uncharacterized protein	Heterogeneous nuclear ribonucleoprotein U-like protein 1	95	3	1.61	6	7	10
G1TX03	A0A0J9YW13	RBM8A	RNA-binding protein 8A	RNA-binding protein 8A (Fragment)	100	2	1.61	5	6	49
G1U1H3	P61018	RAB4B	Uncharacterized protein	Ras-related protein Rab-4B	100	3	1.61	4	6	31
G1SIJ2	P24752	ACAT1	Uncharacterized protein	Acetyl-CoA acetyltransferase, mitochondrial	92	3	1.61	13	21	47
G1SQA8	P06576	ATP5F1B	ATP synthase subunit beta	ATP synthase subunit beta, mitochondrial	97	2	1.61	29	483	74
G1TIT1	O75477	ERLIN1	PHB domain-containing protein	Erlin-1	98	2	1.61	11	8	41
G1SG80	F8VZG5	AK2	Adenylate kinase 2, mitochondrial	Adenylate kinase 2, mitochondrial	92	2	1.61	9	25	47
G1SGI8	O95881	TXNDC12	Thioredoxin domain-containing protein	Thioredoxin domain-containing protein 12	95	2	1.60	5	10	37
G1TDQ3	J3QT28	BUB3	WD_REPEATS_REGION domain-containing protein	Mitotic checkpoint protein BUB3 (Fragment)	99	2	1.60	4	6	15
G1TEN1	H7C5S0	ACTL6A	Uncharacterized protein	Actin-like protein 6A (Fragment)	99	3	1.60	4	4	15
G1SV12	P16219	ACADS	Uncharacterized protein	Short-chain specific acyl-CoA dehydrogenase, mitochondrial	92	3	1.60	13	21	51
G1TS36	B8ZZG1	MPP6	Uncharacterized protein	Membrane protein, palmitoylated 6 (MAGUK p55 subfamily member 6), isoform CRA_a	96	3	1.60	5	4	10
G1U5M7	A0A3B3IUA2	SNU13	Ribonucleoprotein	Ribonucleoprotein	100	2	1.60	4	11	23
	Q969V3	NCLN		Nicalin		4	1.59	7	11	18
G1SSL0	P30040	ERP29	Endoplasmic reticulum resident protein 29	Endoplasmic reticulum resident protein 29	93	2	1.59	9	34	46
G1T890	Q8NE86	MCU	MCU domain-containing protein	Calcium uniporter protein, mitochondrial	98	2	1.59	10	18	33
G1SDH8	Q5T9L3	WLS	Uncharacterized protein	Protein wntless homolog	98	3	1.59	5	4	12
G1SFD8	Q9BUQ8	DDX23	Uncharacterized protein	Probable ATP-dependent RNA helicase DDX23	99	3	1.59	7	5	11
G1SJY0		MRPS5	S5 DRBM domain-containing protein			1	1.59	4	4	12
G1U1Q1		THBS2	Thrombospondin 2			1	1.59	5	6	8
G1SCK0	Q6P2Q9	PRPF8	MPN domain-containing protein	Pre-mRNA-processing-splicing factor 8	100	2	1.59	35	45	22
G1SZ76	P05455	SSB	Lupus La protein homolog	Lupus La protein	92	2	1.59	16	30	41
G1T994	D6RGG3	COL12A1	Collagen alpha-1(XII) chain	Collagen alpha-1(XII) chain	95	2	1.58	117	656	55
G1SW10	O94776	MTA2	Uncharacterized protein	Metastasis-associated protein MTA2	99	3	1.58	6	5	12
G1SJ30		WDR43	WD_REPEATS_REGION domain-containing protein			1	1.58	2	2	4
G1T3Y0	P98082	DAB2	PID domain-containing protein	Disabled homolog 2	90	2	1.58	5	4	9
G1T3U3	A0A0G2JRV3	SMARCB1	SWI/SNF related, matrix associated, actin dependent regulator of chromatin, subfamily b, member 1	SWI/SNF-related matrix-associated actin-dependent regulator of chromatin subfamily B member 1	85	2	1.58	4	5	26
G1SEX6	Q9H2U1	DHX36	Uncharacterized protein	ATP-dependent DNA/RNA helicase DHX36	94	3	1.58	3	3	5

G1SMP5	Q5T160	RARS2	DALR_1 domain-containing protein	Probable arginine--tRNA ligase, mitochondrial	93	2	1.58	6	6	10
G1SPF1	Q5JTH9	RRP12	NUC173 domain-containing protein	RRP12-like protein	92	2	1.58	7	10	8
G1SKD9	P53597	SUCLG1	Succinate--CoA ligase [ADP/GDP-forming] subunit alpha, mitochondrial	Succinate--CoA ligase [ADP/GDP-forming] subunit alpha, mitochondrial	96	2	1.58	7	28	25
G1TAL6	Q10471	GALNT2	Polypeptide N-acetylgalactosaminyltransferase	Polypeptide N-acetylgalactosaminyltransferase 2	99	2	1.58	10	15	23
G1T134		ABHD11	Abhydrolase domain containing 11			1	1.57	3	2	16
G1SF97		MRPL46	MRP-L46 domain-containing protein			1	1.57	4	11	18
G1TVG7	O75533	SF3B1	SF3b1 domain-containing protein	Splicing factor 3B subunit 1	99	2	1.57	20	37	27
G1SLV3	P42285	MTREX	Uncharacterized protein	Exosome RNA helicase MTR4	99	3	1.57	8	14	11
G1SZK8	P41223	BUD31	Uncharacterized protein	Protein BUD31 homolog	100	3	1.57	2	2	19
G1T3R1	Q86SX6	GLRX5	Glutaredoxin 5	Glutaredoxin-related protein 5, mitochondrial	84	2	1.56	3	4	25
G1SCX4	Q15052	ARHGEF6	Uncharacterized protein	Rho guanine nucleotide exchange factor 6	94	3	1.56	2	4	4
G1SGY8	Q3ZCQ8	TIMM50	Mitochondrial import inner membrane translocase subunit TIM50	Mitochondrial import inner membrane translocase subunit TIM50	96	2	1.56	6	11	23
G1T2N4	E7ET15	U2SURP	Uncharacterized protein	U2 snRNP-associated SURP motif-containing protein	99	3	1.56	6	11	8
G1T1R4		NR3C1	Glucocorticoid receptor			1	1.56	2	3	6
	Q96PK6-5	RBM14		Isoform 5 of RNA-binding protein 14		4	1.56	5	2	24
G1TC03	Q13263	TRIM28	Tripartite motif containing 28	Transcription intermediary factor 1-beta	96	2	1.56	16	14	25
G1TRH3	Q9UBR2	CTSZ	Pept_C1 domain-containing protein	Cathepsin Z	86	2	1.56	7	34	30
G1SJI6		MRPS23	MRP-S23 domain-containing protein			1	1.56	5	6	34
G1SRF1	J3KT10	NUP85	Nuclear pore complex protein Nup85	Nuclear pore complex protein Nup85	84	2	1.56	7	10	16
G1SKQ8	Q13601	KRR1	KRR1 small subunit processome component	KRR1 small subunit processome component homolog	95	2	1.56	2	3	7
G1SZA1	B4DHE8	MSI2	Uncharacterized protein	RNA-binding protein Musashi homolog 2	94	3	1.56	4	3	18
G1U7M0	Q96AJ9	VTI1A	t-SNARE coiled-coil homology domain-containing protein	Vesicle transport through interaction with t-SNAREs homolog 1A	94	2	1.55	3	3	17
G1SSH0	A0A087WUB9	CTNNBL1	DUF1716 domain-containing protein	Beta-catenin-like protein 1	96	2	1.55	5	6	12
G1TUK6	O14656	TOR1A	Torsin family 1 member A	Torsin-1A	90	2	1.55	7	7	25
G1TXS5	Q9UGP8	SEC63	J domain-containing protein	Translocation protein SEC63 homolog	93	2	1.55	12	20	21
	A0A087WUC6	SPCS2		Signal peptidase complex subunit 2		4	1.55	6	17	33
G1SZ93	Q9NSE4	IARS2	Uncharacterized protein	Isoleucine--tRNA ligase, mitochondrial	90	3	1.55	14	13	22
G1U1X6		NT5C3A	5~-nucleotidase			1	1.55	2	2	9
G1SP24	E7EQB9	POLR1C	RPOLD domain-containing protein	DNA-directed RNA polymerases I and III subunit RPAC1	84	2	1.55	4	7	25
G1SDL0	Q96DX4	RSPRY1	Uncharacterized protein	RING finger and SPRY domain-containing protein 1	93	3	1.55	4	4	16
	O96008	TOMM40		Mitochondrial import receptor subunit TOM40 homolog		4	1.55	9	14	37
G1T301	A0A0A0MRN4	ZNF326	Uncharacterized protein	DBIRD complex subunit ZNF326	96	3	1.55	4	4	10
G1SJW8	H3BND8	USP7	Uncharacterized protein	Ubiquitin carboxyl-terminal hydrolase (Fragment)	99	3	1.55	9	8	16
G1SR36	C9JG87	MRPL39	Uncharacterized protein	39S ribosomal protein L39, mitochondrial (Fragment)	86	3	1.54	5	5	12
G1SFH9	A0A2R8Y543	CTNNB1	Uncharacterized protein	Catenin beta-1	100	3	1.54	25	55	46
	Q03252	LMNB2		Lamin-B2		4	1.54	8	6	11
	P26368	U2AF2		Splicing factor U2AF 65 kDa subunit		4	1.54	8	12	29
	K7EKE6	LONP1		Lon protease homolog, mitochondrial		4	1.54	19	42	28
G1STP6	P35221	CTNNA1	Catenin alpha-1	Catenin alpha-1	99	2	1.54	31	69	48
G1T146	E7ESY4	MTA1	Metastasis associated 1	Metastasis-associated protein MTA1	88	2	1.54	4	2	9
G1SS51		MRPS25	L51_S25_CI-B8 domain-containing protein			1	1.53	3	2	22
G1ST15	Q13825	AUH	AU RNA binding methylglutaconyl-CoA hydratase	Methylglutaconyl-CoA hydratase, mitochondrial	95	2	1.53	2	2	7
G1T6L7	F8WAR4	CHCHD3	MICOS complex subunit	MICOS complex subunit	78	2	1.53	3	4	9
G1T696	Q09161	NCBP1	MIF4G domain-containing protein	Nuclear cap-binding protein subunit 1	99	2	1.53	9	9	19
G1U998		NOL6	Nucleolar protein 6			1	1.53	3	9	7
G1T2I5	F8W914	RTN4	Reticulon	Reticulon	98	2	1.53	8	270	13
G1T069	P57740	NUP107	Nuclear pore complex protein	Nuclear pore complex protein Nup107	94	2	1.53	9	17	16
G1SD54	Q99720	SIGMAR1	Uncharacterized protein	Sigma non-opioid intracellular receptor 1	97	3	1.53	4	6	23
G1SR61	H7BXY3	DHX30	Uncharacterized protein	ATP-dependent RNA helicase DHX30	98	3	1.52	7	12	12
G1SMI2	Q9Y305	ACOT9	Acyl-CoA thioesterase 9	Acyl-coenzyme A thioesterase 9, mitochondrial	83	2	1.52	13	11	33
G1TEI2		FDX1	2Fe-2S ferredoxin-type domain-containing protein			1	1.52	2	3	20
G1TAB7	A0A2R8Y3X5	OPA1	Dynamin-type G domain-containing protein	Dynamin-like 120 kDa protein, mitochondrial	94	2	1.52	26	48	32
G1SLI0	Q96A33	CCDC47	Uncharacterized protein	Coiled-coil domain-containing protein 47	98	3	1.52	8	12	22
G1SHV1	Q9Y333	LSM2	U6 snRNA-associated Sm-like protein LSm2	U6 snRNA-associated Sm-like protein LSm2	100	2	1.52	2	3	27
G1T120	S4R369	MRPL37	Uncharacterized protein	39S ribosomal protein L37, mitochondrial	84	3	1.52	6	5	19
G1T0N4	P06748	NPM1	Uncharacterized protein	Nucleophosmin	89	3	1.52	11	44	38
G1STH0	Q15459	SF3A1	Uncharacterized protein	Splicing factor 3A subunit 1	98	3	1.52	8	11	13
G1TRS0		DKC1	PUA domain-containing protein			1	1.51	5	7	14
G1SRX2	Q15029	EFTUD2	Tr-type G domain-containing protein	116 kDa U5 small nuclear ribonucleoprotein component	100	2	1.51	18	33	31

G1T511	A0A3F2YNY6	PRPF40A	Pre-mRNA processing factor 40 homolog A	Pre-mRNA-processing factor 40 homolog A	83	2	1.51	5	5	7
G1TZI2		PGAM5	PGAM family member 5, mitochondrial serine/threonine protein phosphatase			1	1.51	2	2	9
G1U2T2	H0YIB4	SRSF9	Uncharacterized protein	Serine/arginine-rich-splicing factor 9 (Fragment)	65	3	1.51	3	4	15
	J3KN01	AFDN		Afadin		4	1.51	2	3	2
G1SVH1	A0A0U1RRK1	MICU1	Uncharacterized protein	Calcium uptake protein 1, mitochondrial	94	3	1.51	5	15	18
G1TJV3	Q9H7Z7	PTGES2	Prostaglandin E synthase 2	Prostaglandin E synthase 2	79	2	1.51	4	5	19
G1STX9		COQ8B	Coenzyme Q8B			1	1.51	3	3	15
G1THT8	J3QT54	CPSF7	Cleavage and polyadenylation specific factor 7	Cleavage and polyadenylation-specificity factor subunit 7 (Fragment)	95	2	1.51	4	2	16
G1U7L4	P11021	HSPA5	Heat shock protein family A (Hsp70) member 5	Endoplasmic reticulum chaperone BiP	99	2	1.51	34	775	62
G1SVD5	P55265	ADAR	Uncharacterized protein	Double-stranded RNA-specific adenosine deaminase	80	3	1.51	18	27	23
G1SV60	J3KS45	TMCO1	Calcium load-activated calcium channel	Calcium load-activated calcium channel (Fragment)	95	2	1.50	2	2	13
G1T4D2		ACOX1	Acyl-coenzyme A oxidase			1	1.50	12	6	27
G1U6X4	F8VQZ7	METAP2	Methionine aminopeptidase 2	Methionine aminopeptidase 2	94	2	1.50	5	21	17
G1SFG7	P48681	NES	Nestin	Nestin	60	2	1.50	28	151	31
	P37198	NUP62		Nuclear pore glycoprotein p62		4	1.50	4	4	11
G1SLJ8	Q8IY81	FTSJ3	pre-rRNA processing protein FTSJ3	pre-rRNA 2~-O-ribose RNA methyltransferase FTSJ3	85	2	1.50	7	7	19
G1SU13	Q5VW52	GPAM	Glycerol-3-phosphate acyltransferase 1, mitochondrial	Glycerol-3-phosphate acyltransferase 1, mitochondrial	94	2	1.50	3	7	8
G1SZF7	P48735	IDH2	Isocitrate dehydrogenase [NADP]	Isocitrate dehydrogenase [NADP], mitochondrial	96	2	1.50	20	126	47
G1TH33	P38117	ETFB	Electron transfer flavoprotein subunit beta	Electron transfer flavoprotein subunit beta	95	2	1.50	10	7	47
G1TSL1	A0A087WZN1	IDH3B	Isocitrate dehydrogenase [NAD] subunit, mitochondrial	Isocitrate dehydrogenase [NAD] subunit, mitochondrial	95	2	1.50	14	19	42
G1TCA0	Q96KR1	ZFR	DZF domain-containing protein	Zinc finger RNA-binding protein	99	2	1.50	3	2	4
G1SI29	P49411	TUFM	Elongation factor Tu	Elongation factor Tu, mitochondrial	93	2	1.50	17	106	41
G1SHJ3		KDELC1	CAP10 domain-containing protein			1	1.50	5	6	14
G1SZZ1	Q9H0V1	TMEM168	Transmembrane protein 168	Transmembrane protein 168	96	2	1.50	5	6	13
G1T7B5	Q9H0S4	DDX47	Uncharacterized protein	Probable ATP-dependent RNA helicase DDX47	97	3	1.50	2	5	6
G1T5M0	P04181	OAT	Uncharacterized protein	Ornithine aminotransferase, mitochondrial	91	3	1.50	16	49	54
G1SS37	A0A494C1M4	ALDH1L2	10-formyltetrahydrofolate dehydrogenase	10-formyltetrahydrofolate dehydrogenase	96	2	1.49	44	86	66
G1THR4		GADD45GIP1	GADD45G interacting protein 1			1	1.49	2	5	13
G1SFH4		MRPL24	KOW domain-containing protein			1	1.49	4	7	32
	B7WP74	CWC22		Pre-mRNA-splicing factor CWC22 homolog (Fragment)		4	1.49	3	2	7
G1TE34	P06756	ITGAV	Integrin_alpha2 domain-containing protein	Integrin alpha-V	95	2	1.49	26	88	34
G1SL42	Q9BPW8	NIPSNAP1	NIPSNAP domain-containing protein	Protein NipSnap homolog 1	95	2	1.48	5	3	26
G1SIP9	H0Y9G6	MRPL3	Uncharacterized protein	39S ribosomal protein L3, mitochondrial (Fragment)	89	3	1.48	4	3	21
G1SR19	P20594	NPR2	Guanylate cyclase	Atrial natriuretic peptide receptor 2	95	2	1.48	7	9	10
G1SRV1	K7ELV2	SEH1L	Uncharacterized protein	Nucleoporin SEH1 (Fragment)	92	3	1.48	5	5	18
G1SQ52	P52788	SMS	PABS domain-containing protein	Spermine synthase	99	2	1.48	3	5	18
G1STD5		SUCO	SUN domain containing ossification factor			1	1.48	2	4	3
G1T194	Q9ULX6	AKAP8L	A-kinase anchoring protein 8 like	A-kinase anchor protein 8-like	86	2	1.48	4	5	10
G1SGG6	P48449	LSS	Terpene cyclase/mutase family member	Lanosterol synthase	89	2	1.48	12	19	20
G1TDK8	F8VXC8	SMARCC2	Uncharacterized protein	SWI/SNF complex subunit SMARCC2	93	3	1.48	8	4	10
G1SDP7	H0Y8P4	UTP15	UTP15, small subunit processome component	U3 small nucleolar RNA-associated protein 15 homolog (Fragment)	92	2	1.48	2	2	4
G1U886	A0A2R8YGH5	AP1S1	AP complex subunit sigma	AP complex subunit sigma	100	2	1.48	4	4	33
G1SGB5	P23246	SFPQ	Splicing factor proline and glutamine rich	Splicing factor, proline- and glutamine-rich	100	2	1.48	13	22	21
G1SSB5	C9JLU1	POLR2H	DNA-directed RNA polymerases I, II, and III subunit RPABC3	DNA-directed RNA polymerases I, II, and III subunit RPABC3 (Fragment)	100	2	1.47	2	2	29
G1U862	Q01650	SLC7A5	Large neutral amino acids transporter small subunit 1	Large neutral amino acids transporter small subunit 1	94	2	1.47	4	9	14
	U3KQK1	LSM4		U6 snRNA-associated Sm-like protein LSm4		4	1.47	2	5	13
G1T8E0	A3KMH1	VWA8	VWFA domain-containing protein	von Willebrand factor A domain-containing protein 8	92	2	1.47	7	10	5
G1SH80	O14874	BCKDK	Protein-serine/threonine kinase	[3-methyl-2-oxobutanoate dehydrogenase [lipoamide]] kinase, mitochondrial	97	2	1.47	2	9	6
G1T6E9	Q8N5K1	CISD2	ZnF_CDGSH domain-containing protein	CDGSH iron-sulfur domain-containing protein 2	99	2	1.47	4	8	32
G1T1G2	A0A1B0GWA2	AGPS	Alkylglycerone-phosphate synthase	Alkylglycerone-phosphate synthase (Fragment)	96	2	1.46	7	10	17
U3KM30	Q969X5	ERGIC1	Uncharacterized protein	Endoplasmic reticulum-Golgi intermediate compartment protein 1	99	3	1.46	3	4	18
G1T2U6	A0A0C4DG89	DDX46	Uncharacterized protein	Probable ATP-dependent RNA helicase DDX46	99	3	1.46	6	5	8
G1STG2	Q9Y5M8	SRPRB	SRP receptor subunit beta	Signal recognition particle receptor subunit beta	94	2	1.46	11	17	49
G1SEE0	Q09028	RBBP4	RB binding protein 4, chromatin remodeling factor	Histone-binding protein RBBP4	100	2	1.46	6	3	23
G1TBC9		TMEM119	Transmembrane protein 119			1	1.46	2	2	17
G1SLJ9	Q9P0I2	EMC3	ER membrane protein complex subunit 3	ER membrane protein complex subunit 3	99	2	1.46	3	4	20
G1STU7	Q5QNZ2	ATP5PB	Uncharacterized protein	ATP synthase F(0) complex subunit B1, mitochondrial	84	3	1.46	12	46	24
G1SQ11	P54886	ALDH18A1	Delta-1-pyrroline-5-carboxylate synthase	Delta-1-pyrroline-5-carboxylate synthase	97	2	1.46	30	69	53
G1SM77	P36542	ATP5F1C	ATP synthase subunit gamma	ATP synthase subunit gamma, mitochondrial	94	2	1.45	11	101	43

G1U3I5		ECH1	Enoyl-CoA hydratase 1			1	1.45	9	10	37
G1SF32	O94826	TOMM70	TPR_REGION domain-containing protein	Mitochondrial import receptor subunit TOM70	97	2	1.45	16	41	24
G1U276	A0A2R8Y6Y7	SUCLA2	Succinate--CoA ligase [ADP-forming] subunit beta, mitochondrial	Succinate--CoA ligase [ADP-forming] subunit beta, mitochondrial	91	2	1.45	13	22	38
	Q8N201	INTS1		Integrator complex subunit 1		4	1.45	4	3	2
G1SZW5	Q8TD16	BICD2	Uncharacterized protein	Protein bicaudal D homolog 2	95	3	1.45	4	4	7
G1TB02	E7EPS8	PTPRM	Uncharacterized protein	Receptor-type tyrosine-protein phosphatase mu	98	3	1.44	3	3	3
G1SCF6		YARS2	Tyrosine--tRNA ligase			1	1.44	5	4	20
G1SW36	H0YDT8	EMC7	DUF2012 domain-containing protein	ER membrane protein complex subunit 7 (Fragment)	79	2	1.44	4	4	24
G1SEC8	Q8IWA4	MFN1	Dynamin-type G domain-containing protein	Mitofusin-1	92	2	1.44	8	14	17
G1T9N2	O75947	ATP5PD	ATP synthase subunit d, mitochondrial	ATP synthase subunit d, mitochondrial	91	2	1.44	10	22	70
G1SFC1	H0Y6I0	GOLGA4	Golgin A4	Golgin subfamily A member 4 (Fragment)	77	2	1.44	12	14	7
G1SW97	A0A0A0MT83	IVD	Uncharacterized protein	Isovaleryl-CoA dehydrogenase isoform 1	95	3	1.44	7	10	17
G1TCE9		HSDL2	SCP2 domain-containing protein			1	1.44	8	16	28
G1SJ32	A0A0A0MTB8	WDR36	WD_REPEATS_REGION domain-containing protein	WD repeat-containing protein 36	94	2	1.44	2	2	4
U3KM71		ATP5MG	ATP synthase subunit			1	1.44	5	16	62
G1T657	A0A087WUK2	HNRNPDL	Heterogeneous nuclear ribonucleoprotein D like	Heterogeneous nuclear ribonucleoprotein D-like	92	2	1.44	6	15	18
G1SUF4	O15269	SPTLC1	Aminotran_1_2 domain-containing protein	Serine palmitoyltransferase 1	95	2	1.44	6	12	15
	P49750-1	YLPM1		Isoform 1 of YLP motif-containing protein 1		4	1.44	4	4	4
G1TBW7	P50148	GNAQ	Uncharacterized protein	Guanine nucleotide-binding protein G(q) subunit alpha	99	3	1.44	11	14	38
G1SSM6	Q8WUM0	NUP133	Nucleoporin_C domain-containing protein	Nuclear pore complex protein Nup133	91	2	1.44	14	17	23
	Q9NTZ6	RBM12		RNA-binding protein 12		4	1.44	2	3	5
G1TCU4	H3BUU9	CDH11	Uncharacterized protein	Cadherin-11	99	3	1.44	3	7	9
G1SWU9		FAM20B	Fam20C domain-containing protein			1	1.43	2	4	7
G1TIT4		PTTG1IP	PTTG1 interacting protein			1	1.43	2	3	11
	P0DN76	U2AF1L5		Splicing factor U2AF 35 kDa subunit-like protein		4	1.43	3	3	23
G1TCU1	I3L2K5	ZC3H7A	Uncharacterized protein	Zinc finger CCCH domain-containing protein 7A (Fragment)	95	3	1.43	2	2	2
G1SRS0	D6RBS5	ELMOD2	ELMO domain-containing protein	ELMO domain-containing protein 2 (Fragment)	93	2	1.43	2	2	6
G1SRZ0		TOR2A	Torsin family 2 member A			1	1.43	2	3	20
G1SYB9		ITGA3	Integrin subunit alpha 3			1	1.43	2	5	4
G1T361	Q96I99	SUCLG2	Succinate--CoA ligase [GDP-forming] subunit beta, mitochondrial	Succinate--CoA ligase [GDP-forming] subunit beta, mitochondrial	97	2	1.43	17	35	49
G1TDX2	A0A3B3ISY9	AGK	Acylglycerol kinase	Acylglycerol kinase, mitochondrial	94	2	1.43	5	6	32
G1SXF1		NFU1	Nfu_N domain-containing protein			1	1.43	4	10	22
G1T2K6	Q8NBU5	ATAD1	AAA domain-containing protein	ATPase family AAA domain-containing protein 1	100	2	1.43	2	4	10
G1SXZ9	Q86WA6	BPHL	Biphenyl hydrolase like	Valacyclovir hydrolase	89	2	1.43	7	7	25
G1T8F7	A0A494C128	NOP56	Nop domain-containing protein	Nucleolar protein 56	98	2	1.43	16	21	39
G1TWS0	B3KY94	CDIPT	CDP-diacylglycerol--inositol 3-phosphatidyltransferase	CDP-diacylglycerol--inositol 3-phosphatidyltransferase	75	2	1.42	4	5	22
G1SWD1	M0QXL5	FBL	Fibrillarin	rRNA 2~-O-methyltransferase fibrillarin (Fragment)	97	2	1.42	10	63	66
	A0A0J9YVP6	PUF60		Poly(U)-binding-splicing factor PUF60 (Fragment)		4	1.42	8	13	23
G1T2G5	Q5NDL2	EOGT	Uncharacterized protein	EGF domain-specific O-linked N-acetylglucosamine transferase	91	3	1.42	8	9	18
G1T0W7	M0R1B0	EMC8	ER membrane protein complex subunit 8	ER membrane protein complex subunit 8 (Fragment)	98	2	1.42	3	3	42
	Q8WX92	NELFB		Negative elongation factor B		4	1.42	3	4	6
G1SZ23	E9PCG9	BDH1	Uncharacterized protein	D-beta-hydroxybutyrate dehydrogenase, mitochondrial	87	3	1.42	9	12	32
	A0A0A6YYJ8	LUC7L2		Putative RNA-binding protein Luc7-like 2		4	1.42	4	7	11
G1TEZ1	G3V198	NUP160	Uncharacterized protein	Nuclear pore complex protein Nup160 (Fragment)	93	3	1.42	14	19	18
G1T7T2	Q15424	SAFB	Scaffold attachment factor B	Scaffold attachment factor B1	86	2	1.42	6	7	14
G1SYT7	G3V0E4	PMPCB	Uncharacterized protein	Mitochondrial-processing peptidase subunit beta	94	3	1.41	10	17	34
G1TE08	H7C3P6	NUP98	Peptidase S59 domain-containing protein	Nuclear pore complex protein Nup98-Nup96 (Fragment)	90	2	1.41	9	10	7
G1SHI9	A0A0D9SFS3	OGDH	Transket_pyr domain-containing protein	2-oxoglutarate dehydrogenase, mitochondrial	95	2	1.41	29	59	40
G1T6J2		APOO	MICOS complex subunit			1	1.41	5	8	46
G1STL1	Q8WUA2	PPIL4	Peptidyl-prolyl cis-trans isomerase	Peptidyl-prolyl cis-trans isomerase-like 4	97	2	1.41	2	3	6
G1T2Z5	A0A087WTA8	COL1A2	Collagen alpha-2(I) chain	Collagen alpha-2(I) chain	94	2	1.41	28	171	33
G1T8H6	K7EK07	H3F3B	Histone H3	Histone H3 (Fragment)	98	2	1.41	7	12	52
G1T159	P62070	RRAS2	Uncharacterized protein	Ras-related protein R-Ras2	97	3	1.41	6	7	33
P42675	E9PCB6	NLN	Neurolysin, mitochondrial	Neurolysin, mitochondrial	94	2	1.41	6	10	15
G1SPY4		ASRGL1	Asparaginase like 1			1	1.40	3	4	11
G1U9B4	P62995	TRA2B	RRM domain-containing protein	Transformer-2 protein homolog beta	100	2	1.40	4	8	16
G1SVQ8	F8WF48	SEC62	Uncharacterized protein	Translocation protein SEC62	100	3	1.40	2	3	5
G1U5D4	F5GY32	PUS1	tRNA pseudouridine synthase	tRNA pseudouridine synthase A (Fragment)	78	2	1.40	2	3	5
G1SDN4	O60506-3	SYNCRIP	Uncharacterized protein	Isoform 3 of Heterogeneous nuclear ribonucleoprotein Q	100	3	1.40	22	44	49

G1TBL0	E7ETZ4	BZW2	Basic leucine zipper and W2 domains 2	Basic leucine zipper and W2 domain-containing protein 2 (Fragment)	100	2	1.40	7	8	20
G1SQ45	A0A0C4DG98	THOC2	Uncharacterized protein	THO complex subunit 2	98	3	1.40	4	4	5
G1T933	Q05707	COL14A1	Uncharacterized protein	Collagen alpha-1(XIV) chain	94	3	1.40	34	59	27
G1T9V2	A0A2R8Y852	CUX1	Cut like homeobox 1	Homeobox protein cut-like	87	2	1.40	4	5	5
G1U8V2	Q96DZ1	ERLEC1	Uncharacterized protein	Endoplasmic reticulum lectin 1	98	3	1.40	3	7	8
G1TBC1	P14625	HSP90B1	Endoplasmin	Endoplasmin	94	2	1.40	43	854	55
G1SDJ7	B1ANR0	PABPC4	Polyadenylate-binding protein	Polyadenylate-binding protein	92	2	1.40	17	11	27
G1T3M3	Q9UL25	RAB21	Uncharacterized protein	Ras-related protein Rab-21	97	3	1.39	5	10	30
G1T524	P05141	SLC25A5	Uncharacterized protein	ADP/ATP translocase 2	98	3	1.39	22	20	69
G1SMM4		DKK3	Dickkopf_N domain-containing protein			1	1.39	3	8	14
G1T7J9	B1AV70	YIPF6	Protein YIPF	Protein YIPF (Fragment)	75	2	1.39	4	8	12
G1TMZ2		MRPS7	Ribosomal_S7 domain-containing protein			1	1.39	5	6	29
G1SGY1	Q86TB9	PATL1	PAT1 domain-containing protein	Protein PAT1 homolog 1	97	2	1.39	2	2	4
G1TCH9	A0A2R8Y473	ABCB7	Uncharacterized protein	ATP-binding cassette sub-family B member 7, mitochondrial	95	3	1.39	7	10	13
G1TNU3		STX16	t-SNARE coiled-coil homology domain-containing protein			1	1.39	5	5	23
G1T237	F8VVM2	SLC25A3	Uncharacterized protein	Phosphate carrier protein, mitochondrial	86	3	1.39	14	111	48
	A0A087WVZ9	POLR2E		DNA-directed RNA polymerases I, II, and III subunit RPABC1		4	1.39	2	2	17
G1TD36	Q6PML9	SLC30A9	Uncharacterized protein	Zinc transporter 9	96	3	1.39	4	5	8
G1SJB9	Q14257	RCN2	Reticulocalbin 2	Reticulocalbin-2	90	2	1.39	12	121	56
G1TQD4		RALY	RRM domain-containing protein			1	1.38	2	2	6
G1TA59	P50213	IDH3A	Isocitrate dehydrogenase [NAD] subunit, mitochondrial	Isocitrate dehydrogenase [NAD] subunit alpha, mitochondrial	98	2	1.38	12	35	40
G1SX50		RBM19	RNA binding motif protein 19			1	1.38	2	2	4
G1U3V0	Q9BX68	HINT2	HIT domain-containing protein	Histidine triad nucleotide-binding protein 2, mitochondrial	94	2	1.38	4	7	41
G1SU17		NSDHL	3Beta_HSD domain-containing protein			1	1.38	2	2	10
G1TCX6	P08240	SRPRA	SRP54 domain-containing protein	Signal recognition particle receptor subunit alpha	99	2	1.38	11	12	25
O46373	P12235	SLC25A4	ADP/ATP translocase 1	ADP/ATP translocase 1	96	2	1.38	18	25	71
G1T9S4	H0YDD4	DLAT	Acetyltransferase component of pyruvate dehydrogenase complex	Acetyltransferase component of pyruvate dehydrogenase complex (Fragment)	90	2	1.38	11	21	25
G1SR63		PREB	WD_REPEATS_REGION domain-containing protein			1	1.38	3	6	15
G1SGY0	O00267	SUPT5H	Transcription elongation factor SPT5	Transcription elongation factor SPT5	98	2	1.38	8	7	12
G1SU75	O95140	MFN2	Mitofusin 2	Mitofusin-2	92	2	1.38	10	11	18
G1TST9	A0A2R8Y212	CHD4	Uncharacterized protein	Chromodomain-helicase-DNA-binding protein 4	97	3	1.37	20	15	18
G1T6M1	Q96DA6	DNAJC19	J domain-containing protein	Mitochondrial import inner membrane translocase subunit TIM14	100	2	1.37	2	3	19
G1SCR7	E9PF10	NUP155	Uncharacterized protein	Nuclear pore complex protein Nup155	90	3	1.37	14	18	16
G1SLP3	H3BMM9	RNPS1	RNA binding protein with serine rich domain 1	RNA-binding protein with serine-rich domain 1 (Fragment)	99	2	1.37	2	3	14
G1SRD2	P28330	ACADL	Uncharacterized protein	Long-chain specific acyl-CoA dehydrogenase, mitochondrial	82	3	1.37	5	82	17
G1T5A2	P08648	ITGA5	Integrin_alpha2 domain-containing protein	Integrin alpha-5	91	2	1.37	10	13	13
U3KPB2	P61009	SPCS3	Signal peptidase complex subunit 3	Signal peptidase complex subunit 3	100	2	1.37	2	5	13
G1T6G1		MMAB	Corrinoid adenosyltransferase			1	1.37	3	3	19
G1T5H5	Q15050	RRS1	Ribosome biogenesis regulatory protein	Ribosome biogenesis regulatory protein homolog	92	2	1.37	5	7	20
G1SP36	Q9HAV4	XPO5	Importin N-terminal domain-containing protein	Exportin-5	95	2	1.37	3	6	7
G1SUK4		MPI	Mannose-6-phosphate isomerase			1	1.37	3	2	12
G1SWN7	Q4G0N4	NADK2	NAD kinase 2, mitochondrial	NAD kinase 2, mitochondrial	92	2	1.37	5	5	14
G1TA69	Q8TBA6	GOLGA5	Uncharacterized protein	Golgin subfamily A member 5	83	3	1.37	4	3	6
G1SHH0	Q14139	UBE4A	Ubiquitination factor E4A	Ubiquitin conjugation factor E4 A	98	2	1.37	3	6	7
G1SI85	A0A0A0MS41	SFXN3	Uncharacterized protein	Sidoreflexin	94	3	1.36	13	16	39
G1SM52	Q96AG4	LRRC59	Uncharacterized protein	Leucine-rich repeat-containing protein 59	97	3	1.36	13	24	48
G1T3S4		MECR	PKS_ER domain-containing protein			1	1.36	5	7	34
G1SML5	Q9H9B4	SFXN1	Sidoreflexin	Sideroflexin-1	94	2	1.36	11	25	53
G1TBU9	A0A0B4J2A4	ACAA2	Uncharacterized protein	3-ketoacyl-CoA thiolase, mitochondrial	89	3	1.36	15	52	64
	Q08945	SSRP1		FACT complex subunit SSRP1		4	1.36	3	3	9
G1TYV6	Q9HD20	ATP13A1	Cation-transporting ATPase	Manganese-transporting ATPase 13A1	95	2	1.36	12	13	15
	P20908	COL5A1		Collagen alpha-1(V) chain		4	1.36	9	3	6
G1SUD2	Q6UW02	CYP20A1	Uncharacterized protein	Cytochrome P450 20A1	88	3	1.36	18	25	46
	Q96HP0	DOCK6		Dedicator of cytokinesis protein 6		4	1.36	3	5	2
G1T8D7	Q8IUX7	AEBP1	F5/8 type C domain-containing protein	Adipocyte enhancer-binding protein 1	76	2	1.36	5	6	8
	Q12873	CHD3		Chromodomain-helicase-DNA-binding protein 3		4	1.36	7	2	6
G1TEK3	Q8IWJ2	GCC2	GRIP domain-containing protein	GRIP and coiled-coil domain-containing protein 2	86	2	1.36	9	12	8
G1SWU1	P55809	OXCT1	Succinyl-CoA:3-ketoacid-coenzyme A transferase	Succinyl-CoA:3-ketoacid coenzyme A transferase 1, mitochondrial	94	2	1.36	18	95	52
G1SYE7	H0Y8R1	GRSF1	Uncharacterized protein	G-rich sequence factor 1 (Fragment)	96	3	1.36	3	2	14

G1T7J5		NCSTN	Ncstrn_small domain-containing protein			1	1.35	7	16	16
G1TAV2		SMCHD1	SMC hinge domain-containing protein			1	1.35	3	3	2
G1U1E6		GCSH	Glycine cleavage system H protein			1	1.35	2	4	33
	Q96PU8	QKI		Protein quaking		4	1.35	4	6	16
	A0A2R8Y7C0	HBA2		Hemoglobin subunit alpha (Fragment)		4	1.35	3	2	23
G1SGL3		SRSF11	RRM domain-containing protein			1	1.35	2	4	5
G1T419	Q92621	NUP205	Uncharacterized protein	Nuclear pore complex protein Nup205	96	3	1.35	24	14	19
G1TA10		TMEM109	Transmembrane protein 109			1	1.35	3	5	9
G1TD91	O43809	NUDT21	Nudix hydrolase domain-containing protein	Cleavage and polyadenylation specificity factor subunit 5	100	2	1.35	4	7	30
	Q9Y5L4	TIMM13		Mitochondrial import inner membrane translocase subunit Tim13		4	1.35	3	4	37
G1SCW9		CHPF2	Hexosyltransferase			1	1.35	4	4	10
	A0A0U1RQF0	FASN		Fatty acid synthase		4	1.35	8	12	4
G1SD98	Q13523	PRPF4B	Pre-mRNA processing factor 4B	Serine/threonine-protein kinase PRP4 homolog	98	2	1.34	4	6	4
G1U6X6	P55795	HNRNPH2	Uncharacterized protein	Heterogeneous nuclear ribonucleoprotein H2	100	3	1.34	10	10	34
G1SGQ0	Q9UBS4	DNAJB11	J domain-containing protein	DnaJ homolog subfamily B member 11	98	2	1.34	9	21	35
G1SQG1	H0YK61	EMC4	ER membrane protein complex subunit 4	ER membrane protein complex subunit 4	97	2	1.34	2	3	32
P41982	P04179	SOD2	Superoxide dismutase [Mn], mitochondrial (Fragment)	Superoxide dismutase [Mn], mitochondrial	92	2	1.34	4	21	25
G1T5R3		TCF25	Transcription factor 25			1	1.34	3	2	10
G1SNF2	P13995	MTHFD2	Uncharacterized protein	Bifunctional methylenetetrahydrofolate dehydrogenase/cyclohydrolase, mitochondrial	93	3	1.34	12	30	57
G1TM48	Q5JRA6	MIA3	SH3 domain-containing protein	Transport and Golgi organization protein 1 homolog	71	2	1.34	5	5	3
	Q9NWU2	GID8		Glucose-induced degradation protein 8 homolog		4	1.34	5	4	41
G1STQ6	Q9Y617	PSAT1	Phosphoserine aminotransferase	Phosphoserine aminotransferase	94	2	1.34	9	16	22
G1T8C2	F8W7U8	MRE11	Double-strand break repair protein	Double-strand break repair protein	93	2	1.34	4	2	9
	Q8NBJ5	COLGALT1		Procollagen galactosyltransferase 1		4	1.34	7	34	14
P27124	Q02790	FKBP4	Peptidyl-prolyl cis-trans isomerase FKBP4	Peptidyl-prolyl cis-trans isomerase FKBP4	91	2	1.34	9	11	26
G1U800	Q8NC56	LEMD2	MSC domain-containing protein	LEM domain-containing protein 2	79	2	1.34	5	3	19
Q28618	P67809	YBX1	Nuclease-sensitive element-binding protein 1	Nuclease-sensitive element-binding protein 1	99	2	1.34	4	94	22
G1SPN3	Q14573	ITPR3	Inositol 1,4,5-trisphosphate receptor type 3	Inositol 1,4,5-trisphosphate receptor type 3	95	2	1.33	8	7	6
G1U636	G3V153	CAPRIN1	Uncharacterized protein	Caprin-1	98	3	1.33	7	22	12
G1SKT4	P25705	ATP5F1A	ATP synthase subunit alpha	ATP synthase subunit alpha, mitochondrial	98	2	1.33	29	372	62
G1SDD0	P30084	ECHS1	Uncharacterized protein	Enoyl-CoA hydratase, mitochondrial	86	3	1.33	9	16	45
	P16104	H2AFX		Histone H2AX		4	1.33	5	23	53
G1T1Y3	A0A384DVU0	PNPLA6	Patatin like phospholipase domain containing 6	Neuropathy target esterase	98	2	1.33	3	2	5
G1T4Z1	Q07954	LRP1	Uncharacterized protein	Prolow-density lipoprotein receptor-related protein 1	98	3	1.33	81	10	26
G1TCE2	H7C0R7	CYB5R1	NADH-cytochrome b5 reductase	NADH-cytochrome b5 reductase 1 (Fragment)	77	2	1.33	6	7	28
G1TKE0	Q9P2X0	DPM3	Dolichol-phosphate mannosyltransferase subunit 3	Dolichol-phosphate mannosyltransferase subunit 3	95	2	1.33	2	2	24
G1TDJ9	F6Y5H0	RBMS1	RNA binding motif single stranded interacting protein 1	RNA-binding motif, single-stranded-interacting protein 1	97	2	1.33	2	3	14
	Q15428	SF3A2		Splicing factor 3A subunit 2		4	1.33	2	2	4
	Q9NYU2	UGGT1		UDP-glucose:glycoprotein glucosyltransferase 1		4	1.33	19	4	19
G1T8H5		POLR2C	RPOLD domain-containing protein			1	1.33	2	2	11
	A0A0C4DFX9	NELFA		Negative elongation factor A		4	1.33	2	2	7
G1U2E5	P08243	ASNS	Asparagine synthetase [glutamine-hydrolyzing]	Asparagine synthetase [glutamine-hydrolyzing]	87	2	1.33	8	7	19
G1SRB1	Q16134	ETFDH	4Fe-4S ferredoxin-type domain-containing protein	Electron transfer flavoprotein-ubiquinone oxidoreductase, mitochondrial	94	2	1.33	9	9	22
G1T2X0	Q99541	PLIN2	Perilipin	Perilipin-2	88	2	1.32	9	14	28
	Q9NRZ7	AGPAT3		1-acyl-sn-glycerol-3-phosphate acyltransferase gamma		4	1.32	2	3	4
G1T925		GPX8	Glutathione peroxidase			1	1.32	4	7	20
G1SCY7	Q8N766	EMC1	EMC1_C domain-containing protein	ER membrane protein complex subunit 1	96	2	1.32	15	23	23
G1SGC2	P0C0S5	H2AFZ	Histone H2A	Histone H2A.Z	98	2	1.32	5	17	54
G1SEV2	P30101	PDIA3	Protein disulfide-isomerase	Protein disulfide-isomerase A3	96	2	1.32	30	214	45
G1T4H3	Q15084	PDIA6	Uncharacterized protein	Protein disulfide-isomerase A6	93	3	1.32	17	106	52
O18757	Q6NUK1	SLC25A24	Calcium-binding mitochondrial carrier protein SCaMC-1	Calcium-binding mitochondrial carrier protein SCaMC-1	95	2	1.32	14	5	43
G1TEG1	O95470	SGPL1	Uncharacterized protein	Sphingosine-1-phosphate lyase 1	90	3	1.32	9	16	22
G1T3L5	O43719	HTATSF1	Uncharacterized protein	HIV Tat-specific factor 1	82	3	1.32	3	3	6
G1SNI4	O94760	DDAH1	Uncharacterized protein	N(G),N(G)-dimethylarginine dimethylaminohydrolase 1	96	3	1.32	6	6	24
G1T1T8	O96005	CLPTM1	CLPTM1, transmembrane protein	Cleft lip and palate transmembrane protein 1	97	2	1.32	11	24	21
G1T5V3	P61106	RAB14	Uncharacterized protein	Ras-related protein Rab-14	100	3	1.32	12	28	73
Q9GKX2		DHRS4	Dehydrogenase/reductase SDR family member 4 (Fragment)			1	1.32	8	12	38
G1T7I0	Q96PU5	NEDD4L	E3 ubiquitin-protein ligase	E3 ubiquitin-protein ligase NEDD4-like	96	2	1.31	3	3	5
G1U864		TRRAP	Transformation/transcription domain associated protein			1	1.31	3	3	1

G1SJG9	Q6P4Q7	CNNM4	Uncharacterized protein	Metal transporter CNNM4	94	3	1.31	3	3	5
P43236	P43235	CTSK	Cathepsin K	Cathepsin K	94	2	1.31	5	11	22
G1T720	F5GXX5	DAD1	Dolichyl-diphosphooligosaccharide--protein glycosyltransferase subunit DAD1	Dolichyl-diphosphooligosaccharide--protein glycosyltransferase subunit DAD1	75	2	1.31	3	5	38
G1U7I9	A0A0G2JJZ9	DDX39B	Uncharacterized protein	Spliceosome RNA helicase DDX39B (Fragment)	95	3	1.31	14	29	46
G1SU97	H0YD97	PDHX	Dihydrolipoamide acetyltransferase component of pyruvate dehydrogenase complex	Pyruvate dehydrogenase protein X component, mitochondrial (Fragment)	89	2	1.31	4	6	10
G1SPR9	P04844	RPN2	Dolichyl-diphosphooligosaccharide--protein glycosyltransferase subunit 2	Dolichyl-diphosphooligosaccharide--protein glycosyltransferase subunit 2	93	2	1.31	25	79	68
G1SNZ8		GORASP1	GRASP55_65 domain-containing protein			1	1.31	2	2	7
	A0A087X2B1	RBFOX1		RNA binding protein fox-1 homolog		4	1.31	2	3	3
G1SFF5	O15270	SPTLC2	Aminotran_1_2 domain-containing protein	Serine palmitoyltransferase 2	98	2	1.31	3	5	8
G1SGJ5	Q92600	CNOT9	Uncharacterized protein	CCR4-NOT transcription complex subunit 9	97	3	1.31	2	3	7
G1SH10	H3BVG0	NUP93	Nuclear pore complex protein Nup93	Nuclear pore complex protein Nup93	99	2	1.31	14	17	24
G1U0Y6		ECI1	Enoyl-CoA delta isomerase 1			1	1.31	9	16	63
G1SK61	Q9UIW2	PLXNA1	Plexin A1	Plexin-A1	91	2	1.31	3	2	4
	G3V1C3	API5		Apoptosis inhibitor 5		4	1.31	8	17	27
G1T7W7	P46977	STT3A	Uncharacterized protein	Dolichyl-diphosphooligosaccharide--protein glycosyltransferase subunit STT3A	100	3	1.31	15	41	26
	E9PB61	ALYREF		THO complex subunit 4		4	1.31	5	45	25
G1TVU4	Q96D15	RCN3	Reticulocalbin 3	Reticulocalbin-3	78	2	1.31	9	38	51
G1T2F2	P23284	PPIB	Peptidyl-prolyl cis-trans isomerase	Peptidyl-prolyl cis-trans isomerase B	94	2	1.30	7	10	31
G1T5C5	E5RHW4	ERLIN2	PHB domain-containing protein	Erlin-2 (Fragment)	98	2	1.30	8	3	36
G1T0V4	F5H013	SNRPG	Small nuclear ribonucleoprotein G	Small nuclear ribonucleoprotein G	98	2	1.30	3	19	58
	Q5T8U5	SURF4		Surfeit 4		4	1.30	4	80	29
G1SF82	P62072	TIMM10	zf-Tim10_DDP domain-containing protein	Mitochondrial import inner membrane translocase subunit Tim10	100	2	1.30	2	4	36
G1SFU0	P51149	RAB7A	Ras-related protein Rab-7a	Ras-related protein Rab-7a	100	2	1.30	12	22	61
G1T0S0	Q8NBN3	TMEM87A	Uncharacterized protein	Transmembrane protein 87A	96	3	1.30	4	5	9
G1SD25	Q5SRE5	NUP188	Nucleoporin 188	Nucleoporin NUP188 homolog	94	2	1.30	10	10	9
G1T601		CLYBL	HpcH_HpaI domain-containing protein			1	1.30	4	5	19
G1SHK6	Q5SSJ5	HP1BP3	Uncharacterized protein	Heterochromatin protein 1-binding protein 3	93	3	1.30	11	19	26
G1SLA2	Q92604	LPGAT1	Lysophosphatidylglycerol acyltransferase 1	Acyl-CoA:lysophosphatidylglycerol acyltransferase 1	94	2	1.30	5	6	17
G1SLM0	A0A087WTT1	PABPC1	Polyadenylate-binding protein	Polyadenylate-binding protein	99	2	1.30	18	18	38
G1U8C4	H0YKK6	PSME1	Uncharacterized protein	Proteasome activator complex subunit 1	98	3	1.30	2	2	27
G1T0L9	P04843	RPN1	Dolichyl-diphosphooligosaccharide--protein glycosyltransferase subunit 1	Dolichyl-diphosphooligosaccharide--protein glycosyltransferase subunit 1	97	2	1.30	28	228	53
G1SE12	P13804	ETFA	Electron transfer flavoprotein subunit alpha	Electron transfer flavoprotein subunit alpha, mitochondrial	95	2	1.30	11	18	56
G1T093	Q9NZJ4-2	SACS	Uncharacterized protein	Isoform 2 of Sacsin	96	3	1.30	5	4	2
G1SNY3	Q68E01	INTS3	Uncharacterized protein	Integrator complex subunit 3	99	3	1.30	6	5	12
G1SVP9	G3V4T2	PABPN1	RRM domain-containing protein	Polyadenylate-binding protein 2	99	2	1.30	2	2	6
G1SSV0	Q9H1E5	TMX4	Thioredoxin domain-containing protein	Thioredoxin-related transmembrane protein 4	84	2	1.29	3	3	12
G1SLS8	Q9NX62	IMPAD1	Uncharacterized protein	Inositol monophosphatase 3	96	3	1.29	6	7	21
G1SIB2	A0A087WVM4	MTHFD1L	Uncharacterized protein	Monofunctional C1-tetrahydrofolate synthase, mitochondrial	92	3	1.29	9	22	13
G1T798	Q12846	STX4	t-SNARE coiled-coil homology domain-containing protein	Syntaxin-4	96	2	1.29	4	4	27
G1T108	Q9NRG9	AAAS	WD_REPEATS_REGION domain-containing protein	Aladin	94	2	1.29	6	4	17
G1U0T4		HSD17B14	Hydroxysteroid 17-beta dehydrogenase 14			1	1.28	2	3	9
G1TBH1	A0A1W2PPT5	POLR2B	DNA-directed RNA polymerase subunit beta	DNA-directed RNA polymerase subunit beta	100	2	1.28	6	8	7
	A0A087WTP3	KHSRP		Far upstream element-binding protein 2		4	1.28	11	11	17
G1TEI0		PARP1	Poly [ADP-ribose] polymerase			1	1.28	3	3	5
G1TFE0	H0YHA7	RPL18	Ribosomal_L18e/L15P domain-containing protein	60S ribosomal protein L18 (Fragment)	90	2	1.28	3	9	22
G1SZD2	P53701	HCCS	Cytochrome c heme lyase	Cytochrome c-type heme lyase	83	2	1.28	4	6	17
G1T7L7		KDELC2	CAP10 domain-containing protein			1	1.28	10	15	25
G1SYI3	A0A087X1B2	USP39	Uncharacterized protein	U4/U6.U5 tri-snRNP-associated protein 2	99	3	1.28	5	7	14
	B4DQT1	MAEA		Macrophage erythroblast attacher		4	1.28	3	3	8
	Q12931	TRAP1		Heat shock protein 75 kDa, mitochondrial		4	1.28	7	2	12
Q9TT15	P21796	VDAC1	Voltage-dependent anion-selective channel protein 1	Voltage-dependent anion-selective channel protein 1	100	2	1.28	18	404	82
G1U383	A0A087WYN9	DHX29	ATP-dependent RNA helicase DHX29	ATP-dependent RNA helicase DHX29	95	2	1.28	7	4	6
	O75937	DNAJC8		DnaJ homolog subfamily C member 8		4	1.28	2	2	7
G1T855	C9J5X1	IGF1R	Tyrosine-protein kinase receptor	Tyrosine-protein kinase receptor	93	2	1.28	8	12	10
G1T329	J3QQY1	CDK5RAP3	Uncharacterized protein	CDK5 regulatory subunit-associated protein 3 (Fragment)	88	3	1.28	9	13	25
G1SCT6	E9PB90	HK2	Uncharacterized protein	Hexokinase-2	96	3	1.28	10	3	16
G1T9E3	E9PLP8	CSTF3	Suf domain-containing protein	Cleavage stimulation factor subunit 3	100	2	1.27	3	3	7
G1T1L4		GRWD1	WD_REPEATS_REGION domain-containing protein			1	1.27	3	4	13
G1SYD6	P02545	LMNA	Uncharacterized protein	Prelamin-A/C	98	3	1.27	33	84	52

G1TG89	P62244	RPS15A	Uncharacterized protein	40S ribosomal protein S15a	100	3	1.27	7	24	55
G1TS38	H0YF06	CCDC90B	Uncharacterized protein	Coiled-coil domain-containing protein 90B, mitochondrial (Fragment)	94	3	1.27	2	2	18
G1THL2		FTL	Ferritin			1	1.27	2	2	17
P27115		MGAT1	Alpha-1,3-mannosyl-glycoprotein 2-beta-N-acetylglucosaminyltransferase			1	1.27	3	3	12
G1SLE1		HARS2	AA_TRNA_LIGASE_II domain-containing protein			1	1.27	4	3	10
G1U4R5	A0A0C4DGS1	DDOST	Dolichyl-diphosphooligosaccharide--protein glycosyltransferase 48 kDa subunit	Dolichyl-diphosphooligosaccharide--protein glycosyltransferase 48 kDa subunit	94	2	1.27	14	433	39
G1SEW1	R4GMQ1	KDM1A	Lysine-specific histone demethylase	Lysine-specific histone demethylase	96	2	1.27	3	7	7
G1SFV1	A0A499FI48	PDIA4	Protein disulfide-isomerase	Protein disulfide-isomerase	91	2	1.27	29	379	49
G1U194	J3KPP4	LUC7L3	Uncharacterized protein	Cisplatin resistance-associated overexpressed protein, isoform CRA_b	98	3	1.27	3	4	8
U3KM62		PTGS2	Prostaglandin G/H synthase 2			1	1.27	6	7	14
B7NZN9	P51571	SSR4	Signal sequence receptor, delta (Predicted)	Translocon-associated protein subunit delta	97	2	1.27	5	9	36
G1SEI8		NUP214	Nup214_FG domain-containing protein			1	1.27	4	6	4
G1SEC9	Q9HAV0	GNB4	WD_REPEATS_REGION domain-containing protein	Guanine nucleotide-binding protein subunit beta-4	99	2	1.26	8	6	28
G1TWU8	O43264	ZW10	Uncharacterized protein	Centromere/kinetochore protein zw10 homolog	89	3	1.26	10	13	20
G1T7P8	Q9UIV1	CNOT7	Uncharacterized protein	CCR4-NOT transcription complex subunit 7	100	3	1.26	3	3	16
G1SE30		EPS8	SH3 domain-containing protein			1	1.26	2	4	5
G1TME5	Q9H857	NT5DC2	Uncharacterized protein	5~-nucleotidase domain-containing protein 2	86	3	1.26	4	4	13
G1SCQ0		EDEM3	alpha-1,2-Mannosidase			1	1.26	2	2	4
G1SRI8	P19367	HK1	Uncharacterized protein	Hexokinase-1	96	3	1.26	27	49	33
G1SMW3	Q92615	LARP4B	La ribonucleoprotein domain family member 4B	La-related protein 4B	88	2	1.26	3	2	8
G1SVU0	C9JAZ1	MTX2	Uncharacterized protein	Metaxin-2 (Fragment)	89	3	1.26	4	12	37
G1T647		GCLM	Glutamate-cysteine ligase modifier subunit			1	1.26	3	4	16
G1SKF1	P07996	THBS1	Uncharacterized protein	Thrombospondin-1	97	3	1.26	34	72	37
G1T501		CCAR2	Cell cycle and apoptosis regulator 2			1	1.26	2	4	6
G1SIB1	P29083	GTF2E1	HTH TFE/IIEalpha-type domain-containing protein	General transcription factor IIE subunit 1	94	2	1.26	2	2	9
G1T006	Q9UMX5	NENF	Neudesin neurotrophic factor	Neudesin	98	2	1.26	3	7	19
G1TDQ1	Q9H0C8	ILKAP	PPM-type phosphatase domain-containing protein	Integrin-linked kinase-associated serine/threonine phosphatase 2C	94	2	1.25	2	3	8
G1SIL2	Q9UNW1	MINPP1	Multiple inositol-polyphosphate phosphatase 1	Multiple inositol polyphosphate phosphatase 1	87	2	1.25	4	6	16
G1SGR9	A0A494C1K3	GTF2I	General transcription factor IIi	General transcription factor II-I	97	2	1.25	4	6	9
G1T4M1		LAMC1	Laminin subunit gamma 1			1	1.25	4	9	9
G1TMQ8	H0Y9V7	ATP2C1	Calcium-transporting ATPase	Calcium-transporting ATPase type 2C member 1 (Fragment)	99	2	1.25	4	4	9
G1TE39	Q9NYU1	UGGT2	Uncharacterized protein	UDP-glucose:glycoprotein glucosyltransferase 2	84	3	1.25	22	22	22
G1TVW1	O60568	PLOD3	Procollagen-lysine,2-oxoglutarate 5-dioxygenase 3	Multifunctional procollagen lysine hydroxylase and glycosyltransferase LH3	94	2	1.25	13	10	31
G1TVY5	C9JZR2	CTNND1	Uncharacterized protein	Catenin delta-1	97	3	1.25	19	40	35
G1U3X5	P46821	MAP1B	Uncharacterized protein	Microtubule-associated protein 1B	88	3	1.24	15	9	10
G1TLK9	P49748	ACADVL	Uncharacterized protein	Very long-chain specific acyl-CoA dehydrogenase, mitochondrial	87	3	1.24	22	54	49
G1SES8	G5E9V5	MRPS22	Uncharacterized protein	28S ribosomal protein S22, mitochondrial	84	3	1.24	2	2	6
G1SLQ3	P15559	NQO1	Flavodoxin_2 domain-containing protein	NAD(P)H dehydrogenase [quinone] 1	89	2	1.24	10	17	48
G1TBS2	Q9Y265	RUVBL1	RuvB-like helicase	RuvB-like 1	100	2	1.24	14	26	47
G1T6P0	Q9H7D0	DOCK5	Dedicator of cytokinesis 5	Dedicator of cytokinesis protein 5	96	2	1.24	3	3	3
G1SQP9	E7EMS6	COMT	Catechol-O-methyltransferase	Catechol O-methyltransferase (Fragment)	78	2	1.24	10	29	52
G1T7Y5	H0Y368	DPM1	Dolichol-phosphate mannosyltransferase subunit 1	Dolichol-phosphate mannosyltransferase subunit 1 (Fragment)	83	2	1.24	5	6	23
G1TPZ3	Q96GK7	FAHD2A	FAA_hydrolase domain-containing protein	Fumarylacetoacetate hydrolase domain-containing protein 2A	90	2	1.24	3	3	12
G1TEE3	G3V5Z3	PPP4R3A	SMK-1 domain-containing protein	Serine/threonine-protein phosphatase 4 regulatory subunit 3A	100	2	1.24	4	8	9
G1T5H0	A0A3B3IU24	HTRA1	PDZ domain-containing protein	Serine protease HTRA1	94	2	1.23	2	3	10
G1T0U8	Q9UHG3	PCYOX1	Prenylcys_lyase domain-containing protein	Prenylcysteine oxidase 1	86	2	1.23	10	25	31
G1SW11		CERCAM	Cerebral endothelial cell adhesion molecule			1	1.23	6	10	16
G1SLT8	P31942	HNRNPH3	Uncharacterized protein	Heterogeneous nuclear ribonucleoprotein H3	100	3	1.23	6	6	24
	A0A0U1RRM4	PTBP1		Polypyrimidine tract-binding protein 1		4	1.23	10	55	29
G1STD4	Q9BSR8	YIPF4	Protein YIPF	Protein YIPF4	99	2	1.23	2	2	9
G1SI95	O00291	HIP1	Uncharacterized protein	Huntingtin-interacting protein 1	91	3	1.23	15	13	24
G1TY83		NLRX1	NLR family member X1			1	1.23	3	3	6
G1U0M5	H7C1W1	PXDN	Peroxidasin	Peroxidasin homolog (Fragment)	93	2	1.23	5	3	5
G1T9J3	A2RRP1	NBAS	Uncharacterized protein	Neuroblastoma-amplified sequence	90	3	1.23	19	26	12
G1T719		TSR1	Bms1-type G domain-containing protein			1	1.23	2	4	4
G1SMH6	Q9P0M6	H2AFY2	Core histone macro-H2A	Core histone macro-H2A.2	99	2	1.23	6	5	23
	P62304	SNRPE		Small nuclear ribonucleoprotein E		4	1.23	3	12	52
G1T2M9	P09486	SPARC	SPARC	SPARC	94	2	1.23	13	22	45
G1T673	Q99653	CHP1	Calcineurin like EF-hand protein 1	Calcineurin B homologous protein 1	98	2	1.22	2	3	17

U3KM96	P61224	RAP1B	Uncharacterized protein	Ras-related protein Rap-1b	100	3	1.22	11	7	73
G1T0E5		SNAP23	Synaptosomal-associated protein			1	1.22	3	4	23
G1SZ03	P55884	EIF3B	Eukaryotic translation initiation factor 3 subunit B	Eukaryotic translation initiation factor 3 subunit B	98	2	1.22	24	8	41
G1U150	M0QXU7	TIMM44	Mitochondrial import inner membrane translocase subunit TIM44	Mitochondrial import inner membrane translocase subunit TIM44 (Fragment)	83	2	1.22	7	12	18
G1T5N5	C9IZ01	GFM1	Elongation factor G, mitochondrial	Elongation factor G, mitochondrial	93	2	1.22	8	10	19
G1TLE4	P62879	GNB2	WD_REPEATS_REGION domain-containing protein	Guanine nucleotide-binding protein G(I)/G(S)/G(T) subunit beta-2	100	2	1.22	11	6	43
G1TBN5		TOR1AIP2	Torsin 1A interacting protein 2			1	1.22	2	4	6
G1SGG2	V9GYM8	ARHGEF2	Uncharacterized protein	Rho guanine nucleotide exchange factor 2	95	3	1.22	13	20	21
G1T242		IKBIP	IKBKB interacting protein			1	1.22	15	22	39
G1T6W7	P04040	CAT	Catalase	Catalase	91	2	1.22	4	2	16
G1T659	Q9UHY7	ENOPH1	Enolase-phosphatase E1	Enolase-phosphatase E1	97	2	1.22	2	2	12
G1SQZ4	Q9Y230	RUVBL2	RuvB-like helicase	RuvB-like 2	99	2	1.22	15	46	49
G1TNW8	Q9Y512	SAMM50	SAMM50 sorting and assembly machinery component	Sorting and assembly machinery component 50 homolog	96	2	1.22	9	9	35
G1SGX6		SDR39U1	DUF1731 domain-containing protein			1	1.22	2	2	14
G1SV32	P18124	RPL7	Uncharacterized protein	60S ribosomal protein L7	98	3	1.22	13	13	46
U3KNY1	Q12797	ASPH	Aspartate beta-hydroxylase	Aspartyl/asparaginyl beta-hydroxylase	96	2	1.22	19	9	56
G1TIM0	G3V155	TMX2	Thioredoxin domain-containing protein	Thioredoxin domain containing 14, isoform CRA_a	94	2	1.22	4	4	13
G1SZG3	Q8NEW0	SLC30A7	Uncharacterized protein	Zinc transporter 7	97	3	1.21	4	4	18
G1SRJ6		AFAP1	Actin filament associated protein 1			1	1.21	3	7	7
G1SLI8	A0A0A0MSJ0	DDX42	Uncharacterized protein	ATP-dependent RNA helicase DDX42	96	3	1.21	3	4	7
G1T297	Q9UBV2	SEL1L	Fibronectin type-II domain-containing protein	Protein sel-1 homolog 1	97	2	1.21	15	20	30
A0A140TAW0	O43852	CALU	Calumenin	Calumenin	99	2	1.21	15	78	64
G1U535	Q9HBH5	RDH14	Uncharacterized protein	Retinol dehydrogenase 14	91	3	1.21	4	3	13
G1SRW4	Q9Y6C2	EMILIN1	Elastin microfibril interfacer 1	EMILIN-1	87	2	1.21	5	5	6
G1TBC0	A0A499FI31	SART3	Uncharacterized protein	Squamous cell carcinoma antigen recognized by T-cells 3	89	3	1.21	2	3	4
	K7EK33	DAZAP1		DAZ-associated protein 1		4	1.21	4	4	18
G1T2J0	P14923	JUP	Uncharacterized protein	Junction plakoglobin	99	3	1.21	5	10	10
G1SUL3		TMF1	TMF_TATA_bd domain-containing protein			1	1.21	2	2	3
G1TJS2	Q8IV08	PLD3	Phospholipase D family member 3	Phospholipase D3	89	2	1.21	5	2	18
G1TSP3	Q9NZ01	TECR	Uncharacterized protein	Very-long-chain enoyl-CoA reductase	98	3	1.21	5	12	15
G1TRV7		QPCTL	Glutaminyl-peptide cyclotransferase like			1	1.21	6	6	32
G1T5G8	Q9HC07	TMEM165	GDT1 family protein	Transmembrane protein 165	91	2	1.21	2	11	9
G1TQ31	A0A2R8YD58	CSNK2A1	Casein kinase II subunit alpha	Casein kinase II subunit alpha	97	2	1.20	4	5	21
Q28888		DCN	Decorin			1	1.20	4	3	16
G1T866	Q96CS3	FAF2	UBX domain-containing protein	FAS-associated factor 2	99	2	1.20	7	19	24
G1T5X6		HMGCL	Pyruvate carboxyltransferase domain-containing protein			1	1.20	2	3	11
G1SYR9	B9A067	IMMT	MICOS complex subunit MIC60	MICOS complex subunit MIC60	89	2	1.20	31	56	50
G1TKY7		OGFOD3	2-oxoglutarate and iron dependent oxygenase domain containing 3			1	1.20	3	3	13
G1T276		ALDH3A2	Aldehyde dehydrogenase			1	1.20	7	10	21
G1SQU5	Q9UP95	SLC12A4	Solute carrier family 12 member 4	Solute carrier family 12 member 4	97	2	1.20	13	21	16
P40144		ADCY5	Adenylate cyclase type 5			1	1.20	3	4	4
G1SUC8	P60228	EIF3E	Eukaryotic translation initiation factor 3 subunit E	Eukaryotic translation initiation factor 3 subunit E	100	2	1.20	14	24	41
G1U797	P51114	FXR1	Uncharacterized protein	Fragile X mental retardation syndrome-related protein 1	91	3	1.20	12	11	19
P15253	P27797	CALR	Calreticulin	Calreticulin	96	2	1.20	21	531	67
G1STF9	Q13347	EIF3I	Eukaryotic translation initiation factor 3 subunit I	Eukaryotic translation initiation factor 3 subunit I	100	2	1.20	10	11	41
G1TFB5	Q9NP72	RAB18	Uncharacterized protein	Ras-related protein Rab-18	98	3	1.20	8	14	49
G1SJH8	K7EP90	RBM42	RRM domain-containing protein	RNA-binding protein 42	97	2	1.20	2	3	8
G1SZZ2	P49458	SRP9	Signal recognition particle 9 kDa protein	Signal recognition particle 9 kDa protein	92	2	1.20	3	4	30
G1T284	Q8TCJ2	STT3B	Uncharacterized protein	Dolichyl-diphosphooligosaccharide--protein glycosyltransferase subunit STT3B	100	3	1.20	5	2	9
	A6XGL3	PRSS1		Protease serine 1		4	1.20	2	11	8
G1T9P2		CARKD	ATP-dependent (S)-NAD(P)H-hydrate dehydratase			1	1.19	6	8	31
G1SG07	Q8IWT6	LRRC8A	Leucine rich repeat containing 8 VRAC subunit A	Volume-regulated anion channel subunit LRRC8A	98	2	1.19	3	3	8
G1TQ79		CHCHD6	MICOS complex subunit			1	1.19	3	3	15
G1SUF5	Q8TF66	LRRC15	LRRCT domain-containing protein	Leucine-rich repeat-containing protein 15	90	2	1.19	6	7	18
G1TVT0	P19338	NCL	Nucleolin	Nucleolin	83	2	1.19	23	23	34
G1SUK0	O75940	SMNDC1	Tudor domain-containing protein	Survival of motor neuron-related-splicing factor 30	99	2	1.19	2	2	13
G1TC33	P35580	MYH10	Uncharacterized protein	Myosin-10	99	3	1.19	102	308	54
	B5MDE0	RFT1		Protein RFT1 homolog		4	1.19	2	3	4
P00169	P00167	CYB5A	Cytochrome b5	Cytochrome b5	90	2	1.19	7	13	80

G1STH4	Q14344	GNA13	Uncharacterized protein	Guanine nucleotide-binding protein subunit alpha-13	86	3	1.19	3	5	11
G1TTL1	A0A0B4J1W3	NAA15	Uncharacterized protein	N-alpha-acetyltransferase 15, NatA auxiliary subunit	99	3	1.19	7	11	13
G1TCT4	O95155	UBE4B	U-box domain-containing protein	Ubiquitin conjugation factor E4 B	95	2	1.19	2	2	3
G1SPG6	A6NEM5	PIGK	GPI-anchor transamidase	GPI-anchor transamidase	85	2	1.19	4	6	22
G1SPB6	E7EPM6	ACSL1	AMP-binding domain-containing protein	Long-chain-fatty-acid--CoA ligase 1	80	2	1.19	5	10	23
G1T3Q2	Q96HY6	DDRGK1	Uncharacterized protein	DDRGK domain-containing protein 1	90	3	1.19	6	13	25
	Q13045	FLII		Protein flightless-1 homolog		4	1.19	6	4	6
G1TEI1	P20645	M6PR	Uncharacterized protein	Cation-dependent mannose-6-phosphate receptor	95	3	1.19	2	3	8
G1T3H3	Q8IXI2	RHOT1	Mitochondrial Rho GTPase	Mitochondrial Rho GTPase 1	99	2	1.19	7	8	13
G1U7X2	Q15637	SF1	CCHC-type domain-containing protein	Splicing factor 1	99	2	1.19	4	6	12
G1TRG8	P04899	GNAI2	Uncharacterized protein	Guanine nucleotide-binding protein G(i) subunit alpha-2	98	3	1.19	15	18	50
G1TP30	H0Y4Q3	RANGAP1	RanGAP1_C domain-containing protein	Ran GTPase-activating protein 1 (Fragment)	80	2	1.18	7	20	20
G1SEN8		SCCPDH	Sacchrp_dh_NADP domain-containing protein			1	1.18	5	6	24
G1SML4	P28288	ABCD3	Uncharacterized protein	ATP-binding cassette sub-family D member 3	96	3	1.18	10	10	21
G1T4Z2	P53396	ACLY	ATP-citrate synthase	ATP-citrate synthase	98	2	1.18	17	29	23
G1SZF9	O75976	CPD	Uncharacterized protein	Carboxypeptidase D	91	3	1.18	5	5	5
G1SMK9	Q9ULC3	RAB23	Uncharacterized protein	Ras-related protein Rab-23	97	3	1.18	4	5	22
G1U3S3		KIAA2013	KIAA2013			1	1.18	4	4	11
G1SGL4	Q9Y276	BCS1L	Uncharacterized protein	Mitochondrial chaperone BCS1	96	3	1.18	6	5	26
G1TA05	A2AE48	TRIM26	Uncharacterized protein	Tripartite motif-containing protein 26 (Fragment)	92	3	1.18	2	2	4
G1SJQ2	Q92896	GLG1	Uncharacterized protein	Golgi apparatus protein 1	97	3	1.18	43	71	42
G1SLF5	H0YG54	REXO2	Exonuclease domain-containing protein	Oligoribonuclease, mitochondrial	98	2	1.18	3	4	22
G1T5Y2	Q9NUQ9	FAM49B	Uncharacterized protein	Protein FAM49B	100	3	1.17	3	3	16
	P29992	GNA11		Guanine nucleotide-binding protein subunit alpha-11		4	1.17	6	2	22
G1SEW3	P49792	RANBP2	RAN binding protein 2	E3 SUMO-protein ligase RanBP2	84	2	1.17	17	28	8
G1SF78	Q96RL7	VPS13A	Vacuolar protein sorting 13 homolog A	Vacuolar protein sorting-associated protein 13A	89	2	1.17	4	5	3
G1U448	E9PEB5	FUBP1	Uncharacterized protein	Far upstream element-binding protein 1	93	3	1.17	10	7	16
G1SUU2	A0A087WXS7	ASNA1	ATPase ASNA1	ATPase ASNA1	93	2	1.17	9	11	44
Q8HZQ5	E7EQR4	EZR	Ezrin	Ezrin	94	2	1.17	15	7	28
G1SF36	Q13423	NNT	Uncharacterized protein	NAD(P) transhydrogenase, mitochondrial	97	3	1.17	25	61	28
G1SWK4	B4E1G1	DERL1	Derlin	Derlin	99	2	1.17	2	3	10
G1TTA5	H0Y8C3	MTCH1	Uncharacterized protein	Mitochondrial carrier homolog 1 (Fragment)	93	3	1.17	7	21	23
G1T920	P49903	SEPHS1	Uncharacterized protein	Selenide, water dikinase 1	100	3	1.17	5	5	20
G1SMI6	Q13162	PRDX4	Thioredoxin domain-containing protein	Peroxiredoxin-4	96	2	1.17	10	16	41
	P30044	PRDX5		Peroxiredoxin-5, mitochondrial		4	1.17	5	8	33
G1TBR5	B4DR61	SEC61A1	Plug_translocon domain-containing protein	Protein transport protein Sec61 subunit alpha isoform 1	100	2	1.17	11	79	39
G1SIH3	A0A2R8Y5A6	ATXN2	Uncharacterized protein	Ataxin-2	94	3	1.17	5	8	5
	O15031	PLXNB2		Plexin-B2		4	1.17	7	5	6
G1SGS7	O94874	UFL1	Uncharacterized protein	E3 UFM1-protein ligase 1	94	3	1.16	16	21	29
G1TKG2		ISOC2	Isochorismatase domain containing 2			1	1.16	4	6	43
G1T7L0		CTSC	Pept_C1 domain-containing protein			1	1.16	5	4	20
G1STZ4	P11413	G6PD	Glucose-6-phosphate 1-dehydrogenase	Glucose-6-phosphate 1-dehydrogenase	94	2	1.16	5	9	11
G1SXL9		XPNPEP3	AMP_N domain-containing protein			1	1.16	2	2	9
G1SLD7	Q9NXF1	TEX10	Ipi1_N domain-containing protein	Testis-expressed protein 10	94	2	1.16	3	4	5
G1SDV5		NOL9	CLP1_P domain-containing protein			1	1.16	2	2	4
G1SU33		NSUN2	NOP2/Sun RNA methyltransferase family member 2			1	1.16	3	2	7
G1SGA5	Q9HCJ6	VAT1L	PKS_ER domain-containing protein	Synaptic vesicle membrane protein VAT-1 homolog-like	96	2	1.16	3	2	10
G1SXX5	A0A2R8Y4F5	HADHA	Uncharacterized protein	Trifunctional enzyme subunit alpha, mitochondrial	86	3	1.16	30	138	52
G1TW04	Q16643	DBN1	Drebrin 1	Drebrin	63	2	1.16	12	96	28
U3KN22	Q9Y2Q3	GSTK1	Glutathione S-transferase kappa	Glutathione S-transferase kappa 1	79	2	1.16	9	19	47
	Q9Y2Q5	LAMTOR2		Ragulator complex protein LAMTOR2		4	1.16	2	2	22
G1T3S7	O60476	MAN1A2	alpha-1,2-Mannosidase	Mannosyl-oligosaccharide 1,2-alpha-mannosidase IB	95	2	1.16	2	2	4
G1SES2		NAA10	N-acetyltransferase domain-containing protein			1	1.16	2	2	9
G1TWK1	E9PGZ4	SACM1L	SAC domain-containing protein	Phosphatidylinositide phosphatase SAC1	98	2	1.16	14	23	34
G1SW89	Q9C0E8	LNPK	zinc_ribbon_10 domain-containing protein	Endoplasmic reticulum junction formation protein lunapark	89	2	1.16	2	2	5
G1SER3	Q02978	SLC25A11	Uncharacterized protein	Mitochondrial 2-oxoglutarate/malate carrier protein	97	3	1.16	8	56	37
G1T4Q7		ATP11C	Phospholipid-transporting ATPase			1	1.16	2	3	3
G1SV51	Q08378	GOLGA3	Golgin A3	Golgin subfamily A member 3	85	2	1.16	12	13	16
G1SCE7	P55084	HADHB	Uncharacterized protein	Trifunctional enzyme subunit beta, mitochondrial	94	3	1.15	14	13	42

G1SZ37	P31937	HIBADH	3-hydroxyisobutyrate dehydrogenase	3-hydroxyisobutyrate dehydrogenase, mitochondrial	96	2	1.15	6	7	32
G1SCT9	A0A087WU53	MAGT1	Uncharacterized protein	Magnesium transporter protein 1	97	3	1.15	7	8	24
G1U6H0	P27824	CANX	Uncharacterized protein	Calnexin	95	3	1.15	16	51	30
G1T8B3	Q8IZL8	PELP1	Uncharacterized protein	Proline-, glutamic acid- and leucine-rich protein 1	91	3	1.15	5	7	6
G1TEN9	Q8TB61	SLC35B2	Uncharacterized protein	Adenosine 3~-phospho 5~-phosphosulfate transporter 1	89	3	1.15	4	7	9
G1SJ66	C9JME2	FARP1	Uncharacterized protein	FERM, ARHGEF and pleckstrin domain-containing protein 1	90	3	1.15	28	57	38
G1SZR8	P51858	HDGF	Heparin binding growth factor	Hepatoma-derived growth factor	96	2	1.15	5	5	22
G1SKZ8	P62906	RPL10A	Ribosomal protein	60S ribosomal protein L10a	100	2	1.15	13	44	53
	A0A024R4M0	RPS9		40S ribosomal protein S9		4	1.15	12	17	40
G1SHE2		GGCX	HTTM domain-containing protein			1	1.15	2	3	3
G1SN95	A0A087WSV8	NUCB2	Nucleobindin 2	Nucleobindin 2, isoform CRA_b	93	2	1.15	18	29	47
G1SJK0		APOOL	MICOS complex subunit			1	1.15	3	8	23
G1SQA4	Q6UWP7	LCLAT1	PlsC domain-containing protein	Lysocardiolipin acyltransferase 1	89	2	1.15	5	5	15
G1SMR7	P30050	RPL12	Uncharacterized protein	60S ribosomal protein L12	100	3	1.15	8	22	53
	A0A087X1G7	SELENOF		Selenoprotein F		4	1.15	3	12	22
G1SG63	P55011	SLC12A2	Uncharacterized protein	Solute carrier family 12 member 2	97	3	1.15	3	2	4
G1SIE6	P29279	CCN2	Cellular communication network factor 2	CCN family member 2	90	2	1.15	11	16	32
G1SQR1	Q93096	PTP4A1	TYR_PHOSPHATASE_2 domain-containing protein	Protein tyrosine phosphatase type IVA 1	100	2	1.14	2	3	17
G1SKF7	Q02878	RPL6	60S ribosomal protein L6	60S ribosomal protein L6	89	2	1.14	11	40	40
G1SY96	Q9UJS0	SLC25A13	Uncharacterized protein	Calcium-binding mitochondrial carrier protein Aralar2	97	3	1.14	11	4	28
G1SFS8	Q7KZF4	SND1	Staphylococcal nuclease domain-containing protein	Staphylococcal nuclease domain-containing protein 1	97	2	1.14	35	80	54
G1U2W0	B4E321	OS9	OS9, endoplasmic reticulum lectin	Protein OS-9	79	2	1.14	3	2	5
G1SWI3	P45880	VDAC2	Voltage-dependent anion-selective channel protein 2	Voltage-dependent anion-selective channel protein 2	99	2	1.14	13	63	63
G1TKC4	Q15046	KARS	AA_TRNA_LIGASE_II domain-containing protein	Lysine--tRNA ligase	88	2	1.14	13	17	32
G1T7X6	G3XAI2	LAMB1	Uncharacterized protein	Laminin subunit beta-1	91	3	1.14	3	2	3
G1SZ15	A8K878	MANF	Mesencephalic astrocyte derived neurotrophic factor	Mesencephalic astrocyte-derived neurotrophic factor	97	2	1.14	6	15	30
G1T0H7	A0A087WWM0	TRAPPC3	Trafficking protein particle complex subunit	Trafficking protein particle complex subunit	94	2	1.14	4	7	27
G1TUY5		OCIAD1	OCIA domain-containing protein			1	1.14	3	3	15
G1T312		ACAD8	Acyl-CoA dehydrogenase family member 8			1	1.14	2	2	5
G1SIP6		CISD1	ZnF_CDGSH domain-containing protein			1	1.14	2	2	20
G1STF8		TBRG4	RAP domain-containing protein			1	1.14	3	3	7
G1SNP4	A0A3B3ITZ9	THRAP3	Uncharacterized protein	Thyroid hormone receptor-associated protein 3	95	3	1.14	6	8	8
G1U0A4	P21810	BGN	Biglycan	Biglycan	94	2	1.14	12	17	46
B7NZS4	O75175	CNOT3	CCR4-NOT transcription complex, subunit 3 (Predicted)	CCR4-NOT transcription complex subunit 3	97	2	1.14	3	5	9
G1TE27	X6R9L0	DNAJC3	Uncharacterized protein	DnaJ homolog subfamily C member 3	88	3	1.14	13	3	33
G1TB98	Q15293	RCN1	Uncharacterized protein	Reticulocalbin-1	85	3	1.14	12	9	45
G1SVY6	Q9BTX1	NDC1	Uncharacterized protein	Nucleoporin NDC1	88	3	1.13	2	2	5
	G8JLG1	SMC1A		Structural maintenance of chromosomes protein		4	1.13	5	6	6
	Q92900	UPF1		Regulator of nonsense transcripts 1		4	1.13	18	26	23
G1T7I3	Q13085	ACACA	Uncharacterized protein	Acetyl-CoA carboxylase 1	98	3	1.13	12	11	7
G1SCE6	Q9Y223	GNE	Epimerase_2 domain-containing protein	Bifunctional UDP-N-acetylglucosamine 2-epimerase/N-acetylmannosamine kinase	100	2	1.13	10	24	27
G1SRZ8	A5YKK6	CNOT1	Uncharacterized protein	CCR4-NOT transcription complex subunit 1	100	3	1.13	8	7	5
G1ST38	Q9Y673	ALG5	ALG5, dolichyl-phosphate beta-glucosyltransferase	Dolichyl-phosphate beta-glucosyltransferase	93	2	1.13	6	10	21
G1TGH1		D2HGDH	D-2-hydroxyglutarate dehydrogenase			1	1.13	4	7	13
G1SNM8	Q8N5M9	JAGN1	Uncharacterized protein	Protein jagunal homolog 1	96	3	1.13	3	4	17
G1SPG2		TRIP11	GRIP domain-containing protein			1	1.13	16	17	11
G1SZM2	A0A0A0MRG2	APP	Amyloid-beta A4 protein	Amyloid-beta precursor protein	99	2	1.13	7	7	11
G1SXB6	B4DVA9	POGLUT1	CAP10 domain-containing protein	Protein O-glucosyltransferase 1	96	2	1.13	3	3	14
G1TBX7	P11233	RALA	Uncharacterized protein	Ras-related protein Ral-A	100	3	1.13	8	6	50
G1SP21	H0Y9A1	YIPF3	Uncharacterized protein	Protein YIPF3 (Fragment)	72	3	1.13	3	7	16
P62139	P62136	PPP1CA	Serine/threonine-protein phosphatase PP1-alpha catalytic subunit	Serine/threonine-protein phosphatase PP1-alpha catalytic subunit	100	2	1.13	11	2	51
G1TTN9	Q9P258	RCC2	Regulator of chromosome condensation 2	Protein RCC2	99	2	1.13	4	4	13
G1SQ57	Q8N6T3	ARFGAP1	Arf-GAP domain-containing protein	ADP-ribosylation factor GTPase-activating protein 1	78	2	1.13	5	4	21
G1T619		MCUB	MCU domain-containing protein			1	1.13	3	3	14
	Q9H0U4	RAB1B		Ras-related protein Rab-1B		4	1.13	12	10	69
G1SCW7	Q9UG63	ABCF2	Uncharacterized protein	ATP-binding cassette sub-family F member 2	99	3	1.12	6	8	11
P20647	P16615	ATP2A2	Sarcoplasmic/endoplasmic reticulum calcium ATPase 2	Sarcoplasmic/endoplasmic reticulum calcium ATPase 2	98	2	1.12	29	3	36
	Q01105-2	SET		Isoform 2 of Protein SET		4	1.12	6	9	36
G1SKK5		PEX6	Peroxisomal biogenesis factor 6			1	1.12	2	3	6

G1SZA3	Q9H2M9	RAB3GAP2	Uncharacterized protein	Rab3 GTPase-activating protein non-catalytic subunit	94	3	1.12	13	12	20
G1SM04	Q13188	STK3	Uncharacterized protein	Serine/threonine-protein kinase 3	99	3	1.12	2	2	5
	P56377	AP1S2		AP-1 complex subunit sigma-2		4	1.12	4	4	33
G1U971		EIF3C	Eukaryotic translation initiation factor 3 subunit C			1	1.12	16	25	24
G1TBX9	Q9H8M7	MINDY3	DUF4205 domain-containing protein	Ubiquitin carboxyl-terminal hydrolase MINDY-3	99	2	1.12	3	3	13
U3KMP1	P61026	RAB10	Uncharacterized protein	Ras-related protein Rab-10	100	3	1.12	9	9	54
	P18583-2	SON		Isoform A of Protein SON		4	1.12	2	2	1
G1TAH7	P29401	TKT	TRANSKETOLASE_1 domain-containing protein	Transketolase	94	2	1.12	20	43	49
Q9N0Z6	P05023	ATP1A1	Sodium/potassium-transporting ATPase subunit alpha-1	Sodium/potassium-transporting ATPase subunit alpha-1	98	2	1.12	28	66	36
G1SSL5	Q14CX7	NAA25	TPR_REGION domain-containing protein	N-alpha-acetyltransferase 25, NatB auxiliary subunit	96	2	1.12	5	6	11
G1TYL5		DNPH1	2~-deoxynucleoside 5~-phosphate N-hydrolase 1			1	1.12	2	2	31
G1SLD5	A0A087X054	HYOU1	Hypoxia up-regulated 1	Hypoxia up-regulated protein 1	87	2	1.12	29	43	38
G1SVD7		SYNJ2BP-COX16	PDZ domain-containing protein			1	1.12	2	2	15
G1SRP2	Q5SWX8	ODR4	Uncharacterized protein	Protein odr-4 homolog	89	3	1.12	12	12	43
G1T550	P51148	RAB5C	Uncharacterized protein	Ras-related protein Rab-5C	91	3	1.12	8	27	53
G1SJL0		TIMM29	Translocase of inner mitochondrial membrane 29			1	1.12	2	2	16
	A0A494C1N0	FKBP2		Peptidylprolyl isomerase		4	1.11	2	6	17
G1TX94	A0A0R4J2E8	MATR3	Uncharacterized protein	Matrin-3	99	3	1.11	17	30	28
Q9TT13	Q9Y277	VDAC3	Voltage-dependent anion-selective channel protein 3	Voltage-dependent anion-selective channel protein 3	98	2	1.11	12	31	54
G1TZ40	Q5M775	SPECC1	Calponin-homology (CH) domain-containing protein	Cytospin-B	88	2	1.11	2	4	3
G1SK67	Q13547	HDAC1	Histone deacetylase	Histone deacetylase 1	99	2	1.11	6	2	23
G1SZP4	I6L894	ANK2	Ankyrin 2	Ankyrin-2	88	2	1.11	3	3	1
G1SJK7		CYP27A1	Sterol 26-hydroxylase, mitochondrial			1	1.11	2	4	8
G1TAA4	O75844	ZMPSTE24	CAAX prenyl protease	CAAX prenyl protease 1 homolog	96	2	1.11	3	5	9
B7NZM4	Q9Y295	DRG1	Developmentally regulated GTP binding protein 1 (Predicted)	Developmentally-regulated GTP-binding protein 1	100	2	1.11	9	17	35
G1SRJ7	P08253	MMP2	72 kDa type IV collagenase	72 kDa type IV collagenase	95	2	1.11	7	9	19
G1TDN4	P10644	PRKAR1A	Uncharacterized protein	cAMP-dependent protein kinase type I-alpha regulatory subunit	99	3	1.11	4	8	17
G1T1C4		HSD17B7	Hydroxysteroid 17-beta dehydrogenase 7			1	1.11	3	3	16
G1U2R1	Q99805	TM9SF2	Transmembrane 9 superfamily member	Transmembrane 9 superfamily member 2	93	2	1.11	6	23	16
G1T3D1	Q14789	GOLGB1	Uncharacterized protein	Golgin subfamily B member 1	85	3	1.11	32	41	15
G1TVG8	O15173	PGRMC2	Cytochrome b5 heme-binding domain-containing protein	Membrane-associated progesterone receptor component 2	97	2	1.11	8	10	33
G1U0B5	Q9Y570	PPME1	Protein phosphatase methylesterase 1	Protein phosphatase methylesterase 1	96	2	1.11	3	4	16
Q28611		UGT1	UDP-glucuronosyltransferase 1-6			1	1.10	10	13	24
G1SZI7		CROT	Carn_acyltransf domain-containing protein			1	1.10	2	2	4
G1SKF5		ERBIN	Erbb2 interacting protein			1	1.10	5	2	9
G1SHK7	A0A0C4DGV4	LAMTOR5	Uncharacterized protein	Hepatitis B virus x interacting protein	100	3	1.10	3	5	31
G1TFZ7	O15355	PPM1G	PPM-type phosphatase domain-containing protein	Protein phosphatase 1G	97	2	1.10	3	3	10
G1SZI0		SPG7	SPG7, paraplegin matrix AAA peptidase subunit			1	1.10	3	3	10
	Q13363	CTBP1		C-terminal-binding protein 1		4	1.10	4	3	10
G1T2T9	P20339	RAB5A	Uncharacterized protein	Ras-related protein Rab-5A	99	3	1.10	8	5	54
G1T2C3	O75534	CSDE1	Uncharacterized protein	Cold shock domain-containing protein E1	99	3	1.10	15	17	21
	P52815	MRPL12		39S ribosomal protein L12, mitochondrial		4	1.10	2	3	13
G1T579	Q6NUQ1	RINT1	Uncharacterized protein	RAD50-interacting protein 1	92	3	1.10	7	7	15
G1SGM3	A0A0J9YXF2	PON2	Uncharacterized protein	Paraoxonase 2, isoform CRA_a	93	3	1.10	12	22	58
P67828	P48729	CSNK1A1	Casein kinase I isoform alpha	Casein kinase I isoform alpha	100	2	1.10	4	6	13
G1SVV2	Q9NZM1	MYOF	Uncharacterized protein	Myoferlin	94	3	1.10	68	118	44
Q01971	P61019	RAB2A	Ras-related protein Rab-2A	Ras-related protein Rab-2A	100	2	1.10	12	43	67
G1SI54		ILVBL	IlvB acetolactate synthase like			1	1.10	5	7	19
G1SE56	F6T1Q0	PDE12	Endo/exonuclease/phosphatase domain-containing protein	2~,5~-phosphodiesterase 12	90	2	1.10	3	2	10
A0A0A0MQQ6	D6RBW1	EIF4E	Eukaryotic translation initiation factor 4E	Eukaryotic translation initiation factor 4E	98	2	1.10	3	3	20
	Q5R3B4	MPC2		Mitochondrial pyruvate carrier (Fragment)		4	1.10	3	4	32
G1T0F6	Q9UHB9	SRP68	Signal recognition particle subunit SRP68	Signal recognition particle subunit SRP68	95	2	1.10	9	12	18
G1T4N8	Q8TF42	UBASH3B	Uncharacterized protein	Ubiquitin-associated and SH3 domain-containing protein B	98	3	1.10	2	2	5
G1TT27	E9PKZ0	RPL8	Ribosomal_L2_C domain-containing protein	60S ribosomal protein L8 (Fragment)	100	2	1.10	6	12	35
G1T239	F5GYQ1	ATP6V0D1	V-type proton ATPase subunit	V-type proton ATPase subunit	90	2	1.09	8	10	31
G1TE13	P84095	RHOG	Uncharacterized protein	Rho-related GTP-binding protein RhoG	99	3	1.09	6	8	41
G1SH95	P30622	CLIP1	CAP-Gly domain containing linker protein 1	CAP-Gly domain-containing linker protein 1	85	2	1.09	12	11	10
G1SJ20	A0A1W2PNV3	GOSR2	Uncharacterized protein	Golgi SNAP receptor complex member 2 (Fragment)	92	3	1.09	5	10	37
G1SQT0	P60510	PPP4C	Serine/threonine-protein phosphatase	Serine/threonine-protein phosphatase 4 catalytic subunit	100	2	1.09	3	2	14

P67873	Q5SRQ6	CSNK2B	Casein kinase II subunit beta	Casein kinase II subunit beta	100	2	1.09	7	10	51
G1SWY6	Q15075	EEA1	Early endosome antigen 1	Early endosome antigen 1	87	2	1.09	32	21	28
G1SMZ5	Q14152	EIF3A	Eukaryotic translation initiation factor 3 subunit A	Eukaryotic translation initiation factor 3 subunit A	93	2	1.09	31	57	27
G1TDC3	Q14697	GANAB	Gal_mutarotas_2 domain-containing protein	Neutral alpha-glucosidase AB	91	2	1.09	30	228	46
	P50454	SERPINH1		Serpin H1		4	1.09	15	30	35
G1SNE9	A0A0G2JQ41	ABR	Uncharacterized protein	Active breakpoint cluster region-related protein (Fragment)	100	3	1.09	7	4	18
G1TDA9	O94766	B3GAT3	Galactosylgalactosylxylosylprotein 3-beta-glucuronosyltransferase	Galactosylgalactosylxylosylprotein 3-beta-glucuronosyltransferase 3	96	2	1.09	4	4	12
	Q9UNK0	STX8		Syntaxin-8		4	1.09	2	2	12
	Q9NRP0	OSTC		Oligosaccharyltransferase complex subunit OSTC		4	1.09	2	6	13
G1T5I3	A0A0C4DFM1	TM9SF4	Transmembrane 9 superfamily member	Transmembrane 9 superfamily member	100	2	1.09	4	6	10
G1SFI7	P49915	GMPS	Uncharacterized protein	GMP synthase [glutamine-hydrolyzing]	99	3	1.09	6	6	10
G1SL07	Q9NUP9	LIN7C	Protein lin-7 homolog	Protein lin-7 homolog C	99	2	1.09	5	4	29
G1TRI7	A0A0C4DG51	PNPLA8	Calcium-independent phospholipase A2-gamma	Calcium-independent phospholipase A2-gamma (Fragment)	90	2	1.09	2	2	4
G1STP3	O75718	CRTAP	Uncharacterized protein	Cartilage-associated protein	94	3	1.09	11	15	32
G1THI9	Q9UKB1	FBXW11	Uncharacterized protein	F-box/WD repeat-containing protein 11	96	3	1.09	2	2	7
G1TBW2	E7EQB8	IDH3G	Isocitrate dehydrogenase [NAD] subunit, mitochondrial	Isocitrate dehydrogenase [NAD] subunit, mitochondrial	94	2	1.09	6	10	27
G1T095		TMEM147	Transmembrane protein 147			1	1.09	2	2	18
G1TRS4	O95302	FKBP9	Peptidylprolyl isomerase	Peptidyl-prolyl cis-trans isomerase FKBP9	97	2	1.09	17	134	41
G1SK33	P05556	ITGB1	Integrin beta	Integrin beta-1	94	2	1.09	17	44	26
G1SIA3	Q8N3C0	ASCC3	Uncharacterized protein	Activating signal cointegrator 1 complex subunit 3	94	3	1.09	7	7	5
G1TJY2		CHID1	Chitinase domain containing 1			1	1.09	10	16	53
G1SLW8	Q7L2H7	EIF3M	Eukaryotic translation initiation factor 3 subunit M	Eukaryotic translation initiation factor 3 subunit M	100	2	1.09	12	26	48
G1SUS6	D6RD69	SAR1B	Uncharacterized protein	GTP-binding protein SAR1b (Fragment)	98	3	1.09	4	2	25
G1SNQ9	A0A0A0MTJ9	NCEH1	Uncharacterized protein	Neutral cholesterol ester hydrolase 1	88	3	1.09	9	11	32
G1SXK8	F8WE74	SLC25A17	Uncharacterized protein	Peroxisomal membrane protein PMP34	96	3	1.09	4	6	21
G1SDD3		KCTD10	BTB domain-containing protein			1	1.08	2	2	9
	P56385	ATP5ME		ATP synthase subunit e, mitochondrial		4	1.08	3	3	22
G1SZ34	Q9Y5S2	CDC42BPB	CDC42 binding protein kinase beta	Serine/threonine-protein kinase MRCK beta	92	2	1.08	10	8	11
G1SSL3	P29373	CRABP2	FABP domain-containing protein	Cellular retinoic acid-binding protein 2	90	2	1.08	3	3	27
G1SFC4		LMF2	Lipase maturation factor			1	1.08	3	5	7
G1ST95	B3KS98	EIF3H	Eukaryotic translation initiation factor 3 subunit H	Eukaryotic translation initiation factor 3 subunit H	98	2	1.08	8	14	37
	H0Y5K5	ERGIC3		Endoplasmic reticulum-Golgi intermediate compartment protein 3 (Fragment)		4	1.08	2	2	5
G1TZC9	A0A087WXU3	ESYT2	Extended synaptotagmin 2	Extended synaptotagmin-2	92	2	1.08	13	7	25
G1TWK7	Q14696	MESD	Uncharacterized protein	LRP chaperone MESD	90	3	1.08	4	6	22
G1SHH1		PPOX	Protoporphyrinogen oxidase			1	1.08	2	2	8
	A6NM71	WDR45		PRA1 family protein		4	1.08	3	6	9
G1SP22	Q99943	AGPAT1	1-acyl-sn-glycerol-3-phosphate acyltransferase	1-acyl-sn-glycerol-3-phosphate acyltransferase alpha	98	2	1.08	3	5	13
G1SSV2	Q9UL01	DSE	DUF4962 domain-containing protein	Dermatan-sulfate epimerase	96	2	1.08	8	8	15
G1T888	Q9P2R3	ANKFY1	Ankyrin repeat and FYVE domain containing 1	Rabankyrin-5	96	2	1.08	5	2	7
A0A0A0MQP7	P62736	ACTA2	Actin, aortic smooth muscle	Actin, aortic smooth muscle	100	2	1.08	30	967	71
	B1ANM7	FAF1		FAS-associated factor 1		4	1.08	3	3	7
G1T5H7		TMEM132B	Transmembrane protein 132B			1	1.08	2	2	4
G1TG15		FAM210A	DUF1279 domain-containing protein			1	1.08	2	3	13
G1U3F3		NEXN	Ig-like domain-containing protein			1	1.08	22	25	36
G1TX74	Q6IBS0	TWF2	Uncharacterized protein	Twinfilin-2	95	3	1.08	4	4	17
G1SGV5	Q8NE71	ABCF1	Uncharacterized protein	ATP-binding cassette sub-family F member 1	92	3	1.08	11	14	27
G1U4G9	O00299	CLIC1	Chloride intracellular channel protein	Chloride intracellular channel protein 1	98	2	1.08	6	9	42
G1TCS8	P62820	RAB1A	Uncharacterized protein	Ras-related protein Rab-1A	100	3	1.08	13	139	74
G1SUP8	P67812	SEC11A	Signal peptidase complex catalytic subunit SEC11	Signal peptidase complex catalytic subunit SEC11A	100	2	1.08	6	9	40
G1SMC8	Q14997	PSME4	Uncharacterized protein	Proteasome activator complex subunit 4	98	3	1.07	5	4	5
G1SQV5	O15371	EIF3D	Eukaryotic translation initiation factor 3 subunit D	Eukaryotic translation initiation factor 3 subunit D	99	2	1.07	13	22	39
G1TQR9		ZSWIM8	SWIM-type domain-containing protein			1	1.07	2	3	2
G1SUN1	O75915	ARL6IP5	PRA1 family protein	PRA1 family protein 3	96	2	1.07	5	21	24
G1SYY0	Q96SK2	TMEM209	Uncharacterized protein	Transmembrane protein 209	96	3	1.07	3	5	12
G1SUQ4		DNTTIP2	Fcf2 domain-containing protein			1	1.07	2	3	4
G1TWC3		TMX1	Thioredoxin domain-containing protein			1	1.07	5	7	19
G1SSC9	O95373	IPO7	Importin N-terminal domain-containing protein	Importin-7	100	2	1.07	18	36	29
P62493	P62491	RAB11A	Ras-related protein Rab-11A	Ras-related protein Rab-11A	100	2	1.07	11	36	59
G1TTN7	O00139	KIF2A	Kinesin-like protein	Kinesin-like protein KIF2A	94	2	1.07	4	3	6

G1SPA6	Q32P28	P3H1	Fe2OG dioxygenase domain-containing protein	Prolyl 3-hydroxylase 1	91	2	1.07	24	43	52
G1SVK5	P26447	S100A4	Protein S100	Protein S100-A4	98	2	1.07	4	4	36
G1TBL1	C9JPE1	SLC25A20	Uncharacterized protein	Mitochondrial carnitine/acylcarnitine carrier protein	92	3	1.07	5	15	22
G1SYL3	E9PFH4	TNPO3	Xpo1 domain-containing protein	Transportin-3	96	2	1.07	6	7	12
G1U460	Q02252	ALDH6A1	Aldedh domain-containing protein	Methylmalonate-semialdehyde dehydrogenase [acylating], mitochondrial	95	2	1.07	8	17	26
G1U4C2	Q76M96	CCDC80	Uncharacterized protein	Coiled-coil domain-containing protein 80	86	3	1.07	5	6	6
G1T9W3	K4DI93	CUL4B	CULLIN_2 domain-containing protein	Cullin 4B, isoform CRA_e	100	2	1.07	10	5	14
G1SRY7	K7ESP4	DCAKD	Uncharacterized protein	Dephospho-CoA kinase domain-containing protein (Fragment)	92	3	1.07	4	7	29
G1SCZ9	Q92575	UBXN4	UBX domain-containing protein	UBX domain-containing protein 4	94	2	1.07	4	5	14
G1U450		XYLB	Xylulokinase			1	1.07	2	2	6
	A0A0G2JL54	C4B_2		Complement C4-B		4	1.06	3	2	1
G1SQ90	A0A2R8Y5H3	COL4A3BP	Collagen type IV alpha 3 binding protein	Collagen type IV alpha-3-binding protein (Fragment)	96	2	1.06	2	2	6
G1SEV7	Q9NX55	HYPK	Uncharacterized protein	Huntingtin-interacting protein K	98	3	1.06	4	6	42
G1TPM1	Q96SB3	PPP1R9B	PDZ domain-containing protein	Neurabin-2	99	2	1.06	6	9	15
	O43752	STX6		Syntaxin-6		4	1.06	2	2	13
G1TPY7		SUMF2	FGE-sulfatase domain-containing protein			1	1.06	3	2	13
G1SN16	P27695	APEX1	DNA-(apurinic or apyrimidinic site) lyase	DNA-(apurinic or apyrimidinic site) lyase	95	2	1.06	5	11	28
G1SED9	B0QY89	EIF3L	Eukaryotic translation initiation factor 3 subunit L	Eukaryotic translation initiation factor 3 subunit L	97	2	1.06	18	25	41
G1T6N3		NPC2	ML domain-containing protein			1	1.06	3	6	21
G1U7C5	Q9P2E9	RRBP1	Uncharacterized protein	Ribosome-binding protein 1	83	3	1.06	44	97	38
G1SKE2	E7ETC0	TIAL1	TIA1 cytotoxic granule associated RNA binding protein like 1	Nucleolysin TIAR	98	2	1.06	2	4	8
G1SG61		TPST2	Protein-tyrosine sulfotransferase			1	1.06	2	2	5
	H3BQQ9	UBE2I		SUMO-conjugating enzyme UBC9 (Fragment)		4	1.06	3	3	40
G1SQB6	Q9NRX1	PNO1	KH domain-containing protein	RNA-binding protein PNO1	96	2	1.06	2	7	14
G1TUU9		TRIP12	Thyroid hormone receptor interactor 12			1	1.06	8	7	6
G1SYS5	A0A1B0GW77	ALDH7A1	Aldedh domain-containing protein	Alpha-aminoadipic semialdehyde dehydrogenase	93	2	1.06	9	32	30
G1TR92		SYDE1	Synapse defective Rho GTPase homolog 1			1	1.06	3	4	7
G1SZR7	Q86UP2	KTN1	Uncharacterized protein	Kinectin	90	3	1.06	46	77	40
	E9PAV3-2	NACA		Isoform skNAC-2 of Nascent polypeptide-associated complex subunit alpha, muscle-specific form		4	1.06	6	31	11
G1SSN9	Q96S59	RANBP9	Uncharacterized protein	Ran-binding protein 9	97	3	1.06	4	5	15
G1SIE8	A0A3B3ISG5	IDE	Uncharacterized protein	Insulin-degrading enzyme	96	3	1.06	7	8	9
G1T810	A0A0A0MTN0	CUL2	CULLIN_2 domain-containing protein	Cullin-2	99	2	1.05	5	6	10
G1SJN5		MAN2A1	Alpha-mannosidase			1	1.05	8	9	12
G1T3H5		EIF2B3	NTP_transferase domain-containing protein			1	1.05	4	4	12
	Q8WXF7-2	ATL1		Isoform 2 of Atlastin-1		4	1.05	5	5	15
G1TC19		CPT2	Carnitine palmitoyltransferase 2			1	1.05	2	2	5
G1T840	Q92598	HSPH1	Uncharacterized protein	Heat shock protein 105 kDa	96	3	1.05	17	20	28
	A0A0A0MRA3	TTN		Titin		4	1.05	2	2	0
G1SRE8		XRCC5	Ku domain-containing protein			1	1.05	7	13	20
G1SQF7	Q14554	PDIA5	Uncharacterized protein	Protein disulfide-isomerase A5	92	3	1.05	10	14	29
G1SLC2	O00303	EIF3F	Eukaryotic translation initiation factor 3 subunit F	Eukaryotic translation initiation factor 3 subunit F	99	2	1.05	9	23	44
G1T9H0	Q8NCA5	FAM98A	Uncharacterized protein	Protein FAM98A	92	3	1.05	8	10	22
G1TIS2		TIMM17B	Mitochondrial import inner membrane translocase subunit TIM17			1	1.05	2	3	22
G1SXJ6		CUL4A	CULLIN_2 domain-containing protein			1	1.05	10	7	17
G1TGI6	A0A0C4DG17	RPSA	40S ribosomal protein SA	40S ribosomal protein SA	98	2	1.05	10	31	46
G1SFC5	Q86VS8	HOOK3	Calponin-homology (CH) domain-containing protein	Protein Hook homolog 3	99	2	1.05	14	25	22
G1SXR1		PRELP	LRRNT domain-containing protein			1	1.05	4	6	17
G1SQL1	Q9HAU5	UPF2	Uncharacterized protein	Regulator of nonsense transcripts 2	97	3	1.05	2	2	2
G1T336	A0A0D9SF53	DDX3X	Uncharacterized protein	ATP-dependent RNA helicase DDX3X	99	3	1.05	19	42	31
G1SUY5	E7EVZ5	PCYOX1L	Prenylcys_lyase domain-containing protein	Prenylcysteine oxidase-like	95	2	1.05	4	8	15
G1SQU0	P15586	GNS	N-acetylglucosamine-6-sulfatase	N-acetylglucosamine-6-sulfatase	95	2	1.04	10	15	19
G1SJ61	Q9Y608	LRRFIP2	Uncharacterized protein	Leucine-rich repeat flightless-interacting protein 2	91	3	1.04	7	7	13
G1T9M7	E9PK47	PYGL	Alpha-1,4 glucan phosphorylase	Alpha-1,4 glucan phosphorylase	94	2	1.04	15	11	25
G1SE46	O95159	ZFPL1	Zinc finger protein like 1	Zinc finger protein-like 1	92	2	1.04	4	4	15
G1SHM2		CLPTM1L	CLPTM1 like			1	1.04	5	5	22
G1T7I8		RIC8A	RIC8 guanine nucleotide exchange factor A			1	1.04	4	5	11
G1T8L2	P05997	COL5A2	Uncharacterized protein	Collagen alpha-2(V) chain	96	3	1.04	15	31	17
G1SG37	G5E9T8	GOSR1	Golgi SNAP receptor complex member 1	Golgi SNAP receptor complex member 1 (Fragment)	99	2	1.04	3	7	20
G1TA37	O75531	BANF1	Uncharacterized protein	Barrier-to-autointegration factor	100	3	1.04	4	11	57

G1T887		GPR107	G protein-coupled receptor 107			1	1.04	2	3	9
G1T7Q3	F5H157	RAB35	Uncharacterized protein	Ras-related protein Rab-35 (Fragment)	91	3	1.04	6	18	40
G1SDM6	O75165	DNAJC13	J domain-containing protein	DnaJ homolog subfamily C member 13	98	2	1.04	27	31	17
G1TAW7		EIF2A	Eukaryotic translation initiation factor 2A			1	1.04	3	5	9
G1SH66		RBM3	RRM domain-containing protein			1	1.04	3	4	37
G1T3L2	K7ERF1	EIF3K	Eukaryotic translation initiation factor 3 subunit K	Eukaryotic translation initiation factor 3 subunit K	87	2	1.04	7	12	44
G1SYI2	P62873	GNB1	WD_REPEATS_REGION domain-containing protein	Guanine nucleotide-binding protein G(I)/G(S)/G(T) subunit beta-1	100	2	1.04	12	44	45
G1TDI0	Q9UQ80	PA2G4	Peptidase_M24 domain-containing protein	Proliferation-associated protein 2G4	98	2	1.04	12	70	43
G1T6M2		DHRS7B	Dehydrogenase/reductase 7B			1	1.04	6	11	22
G1U2J5	Q8NE01	CNNM3	Uncharacterized protein	Metal transporter CNNM3	88	3	1.03	2	2	7
G1TCT3	F8VPD4	CAD	Carbamoyl-phosphate synthetase 2, aspartate transcarbamylase, and dihydroorotase	CAD protein	94	2	1.03	18	5	13
G1SSX2	Q7Z6Z7	HUWE1	HECT, UBA and WWE domain containing 1, E3 ubiquitin protein ligase	E3 ubiquitin-protein ligase HUWE1	96	2	1.03	19	16	8
G1T147	Q8IZ52	CHPF	Hexosyltransferase	Chondroitin sulfate synthase 2	95	2	1.03	3	3	12
G1TCM0	J3KQ34	COPS7B	PCI domain-containing protein	COP9 signalosome complex subunit 7b	97	2	1.03	3	8	19
G1TAI0		KPNA2	Importin subunit alpha			1	1.03	3	6	11
G1SJC7	Q9UN86	G3BP2	Uncharacterized protein	Ras GTPase-activating protein-binding protein 2	100	3	1.03	5	6	10
G1TWU1		TOR1B	Torsin			1	1.03	5	4	19
G1SMB5		VPS37C	VPS37 C-terminal domain-containing protein			1	1.03	4	5	21
G1TG28	P80723	BASP1	Brain abundant membrane attached signal protein 1	Brain acid soluble protein 1	61	2	1.03	5	6	51
G1SML9	P31689	DNAJA1	Uncharacterized protein	DnaJ homolog subfamily A member 1	100	3	1.03	8	17	34
G1TDF6	O75955	FLOT1	PHB domain-containing protein	Flotillin-1	99	2	1.03	13	21	40
G1TT75	O00264	PGRMC1	Cytochrome b5 heme-binding domain-containing protein	Membrane-associated progesterone receptor component 1	93	2	1.03	7	7	29
G1TAM8	P61289	PSME3	Uncharacterized protein	Proteasome activator complex subunit 3	95	3	1.03	8	11	37
G1T534	E9PDM8	SEC24D	SEC24 homolog D, COPII coat complex component	Protein transport protein Sec24D	92	2	1.03	8	2	8
G1T782	Q9Y6N5	SQOR	Pyr_redox_2 domain-containing protein	Sulfide:quinone oxidoreductase, mitochondrial	93	2	1.03	6	14	18
G1SMZ8	Q96JJ7	TMX3	Thioredoxin domain-containing protein	Protein disulfide-isomerase TMX3	92	2	1.03	8	8	28
	Q9NUY8	TBC1D23		TBC1 domain family member 23		4	1.03	3	3	6
G1SGN0	Q15042	RAB3GAP1	Uncharacterized protein	Rab3 GTPase-activating protein catalytic subunit	94	3	1.03	10	7	17
G1TA41	Q7L7X3	TAOK1	Protein kinase domain-containing protein	Serine/threonine-protein kinase TAO1	100	2	1.03	4	3	6
	O94973	AP2A2		AP-2 complex subunit alpha-2		4	1.03	27	34	42
G1SVW7	G3V1U5	GOLT1B	Uncharacterized protein	Golgi transport 1 homolog B (S. cerevisiae), isoform CRA_c	100	3	1.03	3	26	17
G1TKQ8	A0A0D9SG77	UBE3A	Ubiquitin-protein ligase E3A	Ubiquitin-protein ligase E3A	97	2	1.03	6	5	11
G1SD09	A0A0J9YYJ0	CNPY4	Canopy FGF signaling regulator 4	Protein canopy homolog 4 (Fragment)	97	2	1.03	6	13	35
G1SWY3	A0A1B0GUZ7	EFR3A	Uncharacterized protein	Protein EFR3 homolog A	98	3	1.03	2	2	5
G1SG68	O00629	KPNA4	Importin subunit alpha	Importin subunit alpha-3	99	2	1.03	7	16	24
G1SHF3		NIT1	CN hydrolase domain-containing protein			1	1.03	3	4	14
G1T2F5	Q86X52	CHSY1	Hexosyltransferase	Chondroitin sulfate synthase 1	93	2	1.03	3	2	7
G1SEK1	P62140	PPP1CB	Serine/threonine-protein phosphatase	Serine/threonine-protein phosphatase PP1-beta catalytic subunit	100	2	1.03	9	3	35
G1T974	A0A3B3IRK6	MOGS	Mannosyl-oligosaccharide glucosidase	Mannosyl-oligosaccharide glucosidase (Fragment)	86	2	1.02	9	11	20
G1T193		UGT3A2	UDP-glucuronosyltransferase			1	1.02	2	2	6
G1SYF9	Q9Y613	FHOD1	Uncharacterized protein	FH1/FH2 domain-containing protein 1	87	3	1.02	5	5	9
G1SHG0	P62899	RPL31	Uncharacterized protein	60S ribosomal protein L31	100	3	1.02	6	14	45
G1TE37		ATP6AP1	ATPase H+ transporting accessory protein 1			1	1.02	2	2	10
G1SSR8	O76031	CLPX	Uncharacterized protein	ATP-dependent Clp protease ATP-binding subunit clpX-like, mitochondrial	97	3	1.02	5	4	13
G1SVJ8	Q96Q05	TRAPPC9	Uncharacterized protein	Trafficking protein particle complex subunit 9	87	3	1.02	2	2	2
G1TM22		UGT1A1	UDP-glucuronosyltransferase			1	1.02	7	2	22
G1U9D3	Q9UK41	VPS28	Vacuolar protein sorting-associated protein 28 homolog	Vacuolar protein sorting-associated protein 28 homolog	93	2	1.02	4	5	29
G1ST56	Q96P70	IPO9	Importin N-terminal domain-containing protein	Importin-9	99	2	1.02	6	9	15
G1SJU1	O75051	PLXNA2	Sema domain-containing protein	Plexin-A2	98	2	1.02	3	3	2
	K7ELL7	PRKCSH		Glucosidase 2 subunit beta		4	1.02	2	5	4
G1SD49	X6RCK5	DCTN3	Uncharacterized protein	Dynactin subunit 3 (Fragment)	82	3	1.02	4	4	24
G1U101	J3KPF0	HECTD4	HECT domain-containing protein	Probable E3 ubiquitin-protein ligase HECTD4	97	2	1.02	2	5	1
G1SZI6	Q86X10	RALGAPB	Rap-GAP domain-containing protein	Ral GTPase-activating protein subunit beta	97	2	1.02	4	2	3
G1SQK1		SERPINB6	SERPIN domain-containing protein			1	1.02	3	3	17
G1TB39	P29353	SHC1	Uncharacterized protein	SHC-transforming protein 1	97	3	1.02	2	2	6
G1U5Z2		TK2	dNK domain-containing protein			1	1.02	2	2	11
G1T4J2	F5H228	TRIO	Uncharacterized protein	Triple functional domain protein	99	3	1.02	4	5	4
G1TCB7	Q5LJA9	UCHL5	Ubiquitin carboxyl-terminal hydrolase	Ubiquitin carboxyl-terminal hydrolase (Fragment)	91	2	1.02	7	7	17
G1SKP2	H0Y8C6	IPO5	Importin N-terminal domain-containing protein	Importin-5 (Fragment)	99	2	1.02	28	45	42

G1U684	A0A087X1W8	CADM1	Uncharacterized protein	Cell adhesion molecule 1	95	3	1.02	9	15	34
G1TJ80	Q9Y3Q3	TMED3	Transmembrane p24 trafficking protein 3	Transmembrane emp24 domain-containing protein 3	91	2	1.02	4	14	40
G1SFV7	Q16531	DDB1	Damage specific DNA binding protein 1	DNA damage-binding protein 1	100	2	1.02	18	24	19
	Q9H0B6	KLC2		Kinesin light chain 2		4	1.02	5	2	12
G1SZ66	I3L295	MPDU1	Uncharacterized protein	Mannose-P-dolichol utilization defect 1 isoform 2	89	3	1.02	3	6	15
G1U724	Q9UBE0	SAE1	SUMO1 activating enzyme subunit 1	SUMO-activating enzyme subunit 1	88	2	1.02	8	12	26
G1SJR4	Q15363	TMED2	Transmembrane p24 trafficking protein 2	Transmembrane emp24 domain-containing protein 2	99	2	1.02	8	27	64
	P62424	RPL7A		60S ribosomal protein L7a		4	1.01	12	34	45
G1TQV4	F6SKB8	NECAP2	DUF1681 domain-containing protein	Adaptin ear-binding coat-associated protein 2	95	2	1.01	2	2	8
G1SIP7	O14976	GAK	Cyclin G associated kinase	Cyclin-G-associated kinase	77	2	1.01	4	4	4
G1SQ93	Q9UKV8	AGO2	Protein argonaute-2	Protein argonaute-2	98	2	1.01	3	3	6
G1TQA4		EPM2AIP1	EPM2A interacting protein 1			1	1.01	4	5	10
G1SDW3		CDS2	Phosphatidate cytidylyltransferase			1	1.01	3	4	10
G1T7T0	Q9Y2G5	POFUT2	Protein O-fucosyltransferase 2	GDP-fucose protein O-fucosyltransferase 2	84	2	1.01	3	3	13
G1U4Y5		FCGRT	Fc fragment of IgG receptor and transporter			1	1.01	2	2	7
G1ST64		LMOD1	Leiomodin 1			1	1.01	5	6	9
G1T643	A0A1W2PNX8	UNC45A	Unc-45 myosin chaperone A	Protein unc-45 homolog A	94	2	1.01	8	13	14
	P30533	LRPAP1		Alpha-2-macroglobulin receptor-associated protein		4	1.01	2	4	5
G1TUB8	Q5VVC8	RPL11	Uncharacterized protein	60S ribosomal protein L11	100	3	1.01	5	13	34
G1TSV3	Q8ND76	CCNY	Cyclin Y	Cyclin-Y	99	2	1.01	2	3	9
G1SG41		TBL2	WD_REPEATS_REGION domain-containing protein			1	1.01	5	6	11
G1SPT2	P26196	DDX6	Uncharacterized protein	Probable ATP-dependent RNA helicase DDX6	99	3	1.01	13	21	39
G1SJ37	F8W9S7	GAPVD1	Uncharacterized protein	GTPase-activating protein and VPS9 domain-containing protein 1	95	3	1.01	3	4	4
G1SQW0	E9PIE4	MTCH2	Uncharacterized protein	Mitochondrial carrier homolog 2 (Fragment)	92	3	1.01	6	16	29
	E9PM12	TCIRG1		V-type proton ATPase subunit a (Fragment)		4	1.01	2	2	9
G1TWP4	A0A140T936	VARS	GST C-terminal domain-containing protein	Valine--tRNA ligase (Fragment)	92	2	1.01	18	29	18
G1SEF8	M0R0Y2	NAPA	Uncharacterized protein	Alpha-soluble NSF attachment protein	84	3	1.00	10	16	49
	A0A087WX29	TARDBP		TAR DNA-binding protein 43 (Fragment)		4	1.00	7	3	47
G1T0M2		TRMT10C	SAM-dependent MTase TRM10-type domain-containing protein			1	1.00	4	4	15
G1TGY9		VPS11	VPS11, CORVET/HOPS core subunit			1	1.00	2	2	3
G1TUD2	Q9UBI6	GNG12	Guanine nucleotide-binding protein subunit gamma	Guanine nucleotide-binding protein G(I)/G(S)/G(O) subunit gamma-12	100	2	1.00	4	5	67
G1TY34		ITGA4	Integrin_alpha2 domain-containing protein			1	1.00	4	5	7
P35566		MARCKSL1	MARCKS-related protein			1	1.00	2	2	25
G1T2V6	Q99747	NAPG	Uncharacterized protein	Gamma-soluble NSF attachment protein	98	3	1.00	3	3	8
Q95MN6		PLP2	Proteolipid protein 2			1	1.00	3	5	34
G1SLC0	Q9BS26	ERP44	Thioredoxin domain-containing protein	Endoplasmic reticulum resident protein 44	97	2	1.00	13	31	42
	P02533	KRT14		Keratin, type I cytoskeletal 14		4	1.00	5	4	14
G1TRA4	P01111	NRAS	Uncharacterized protein	GTPase NRas	100	3	1.00	7	3	53
G1SEH1		PLSCR3	Phospholipid scramblase			1	1.00	2	4	13
G1SDN9	Q9P2B2	PTGFRN	Uncharacterized protein	Prostaglandin F2 receptor negative regulator	91	3	1.00	6	7	8
	P51911	CNN1		Calponin-1		4	1.00	7	8	33
G1SZR6	Q13618	CUL3	CULLIN_2 domain-containing protein	Cullin-3	100	2	1.00	13	26	23
G1SM82	E9PGC0	RASA1	Uncharacterized protein	Ras GTPase-activating protein 1	96	3	1.00	8	9	13
G1STX3	Q8WVM8	SCFD1	Uncharacterized protein	Sec1 family domain-containing protein 1	96	3	1.00	16	24	42
G1T568	Q92973	TNPO1	Transportin 1	Transportin-1	93	2	1.00	14	11	21
G1SUV0	Q9NW15	ANO10	Anoctamin	Anoctamin-10	94	2	1.00	5	11	12
P47823		EIF2B5	Translation initiation factor eIF-2B subunit epsilon			1	1.00	5	8	14
G1SPB4		HYAL1	Hyaluronidase			1	1.00	2	2	8
G1SMY6	O14980	XPO1	Importin N-terminal domain-containing protein	Exportin-1	99	2	1.00	16	17	25
P30947	P08238	HSP90AB1	Heat shock protein HSP 90-beta	Heat shock protein HSP 90-beta	99	2	1.00	34	91	61
G1SGM2	O14744	PRMT5	Protein arginine N-methyltransferase 5	Protein arginine N-methyltransferase 5	98	2	1.00	6	9	19
G1U207	Q86Y82	STX12	t-SNARE coiled-coil homology domain-containing protein	Syntaxin-12	96	2	1.00	10	18	55
G1T7H0	A0A1W2PPS1	HNRNPU	Uncharacterized protein	Heterogeneous nuclear ribonucleoprotein U	96	3	1.00	22	54	33
G1TR97	A0A0A0MSA7	EIF4G3	Uncharacterized protein	Eukaryotic translation initiation factor 4 gamma 3	89	3	1.00	8	10	8
G1TMI5		RABAC1	PRA1 family protein			1	1.00	2	4	17
P62497	P62495	ETF1	Eukaryotic peptide chain release factor subunit 1	Eukaryotic peptide chain release factor subunit 1	100	2	1.00	15	31	52
G1THV8	Q9BT09	CNPY3	DUF3456 domain-containing protein	Protein canopy homolog 3	92	2	0.99	6	6	26
G1SL46		PSMD9	PDZ domain-containing protein			1	0.99	3	3	15
G1SMA1	P49755	TMED10	Transmembrane emp24 domain-containing protein 10	Transmembrane emp24 domain-containing protein 10	95	2	0.99	8	280	41

G1SY19	O15118	NPC1	SSD domain-containing protein	NPC intracellular cholesterol transporter 1	91	2	0.99	3	4	3
G1TCP2	A0A087X1E4	ARFIP2	AH domain-containing protein	Arfaptin-2	91	2	0.99	2	5	9
G1T3U5	Q9UDY4	DNAJB4	J domain-containing protein	DnaJ homolog subfamily B member 4	96	2	0.99	4	4	21
G1SDN8	Q92791	P3H4	Uncharacterized protein	Endoplasmic reticulum protein SC65	88	3	0.99	5	8	18
G1U115	Q15008	PSMD6	PCI domain-containing protein	26S proteasome non-ATPase regulatory subunit 6	99	2	0.99	16	36	47
G1T3A2	Q9Y3A6	TMED5	GOLD domain-containing protein	Transmembrane emp24 domain-containing protein 5	97	2	0.99	5	13	24
G1TZQ5	G5EA31	SEC24C	Uncharacterized protein	Protein transport protein Sec24C	93	3	0.99	6	9	10
G1SJG0	P28300	LOX	Uncharacterized protein	Protein-lysine 6-oxidase	88	3	0.99	4	6	14
G1STE3	A0A0A0MQX8	MBNL1	Uncharacterized protein	Muscleblind-like protein 1	100	3	0.99	3	4	7
	P19623	SRM		Spermidine synthase		4	0.99	3	2	17
G1TPC8	P08962	CD63	Tetraspanin	CD63 antigen	78	2	0.99	4	9	15
G1SFQ6	Q9NSK0	KLC4	Uncharacterized protein	Kinesin light chain 4	97	3	0.99	4	2	10
G1STS3	Q96T76	MMS19	Uncharacterized protein	MMS19 nucleotide excision repair protein homolog	93	3	0.98	8	10	16
G1TE47		COG8	Conserved oligomeric Golgi complex subunit 8			1	0.98	5	5	12
G1TCY7		EIF2B4	Translation initiation factor eIF-2B subunit delta			1	0.98	3	3	8
G1SFH6	Q9UBT2	UBA2	Uncharacterized protein	SUMO-activating enzyme subunit 2	97	3	0.98	10	7	27
G1TBK0		ANKMY2	Ankyrin repeat and MYND domain containing 2			1	0.98	2	3	5
G1TB71	Q14008	CKAP5	Uncharacterized protein	Cytoskeleton-associated protein 5	97	3	0.98	18	24	15
G1T8Y0		COASY	CTP_transf_like domain-containing protein			1	0.98	2	2	5
G1T2G4	P05198	EIF2S1	Eukaryotic translation initiation factor 2 subunit 1	Eukaryotic translation initiation factor 2 subunit 1	99	2	0.98	14	33	53
G1SZN0	Q9NZB2	FAM120A	Uncharacterized protein	Constitutive coactivator of PPAR-gamma-like protein 1	94	3	0.98	5	6	6
G1SR15		CD109	CD109 molecule			1	0.98	2	3	2
G1SJE4	Q92556	ELMO1	ELMO domain-containing protein	Engulfment and cell motility protein 1	97	2	0.98	6	8	13
G1TJ79		KYAT1	Aminotran_1_2 domain-containing protein			1	0.98	2	2	10
G1TCZ8	P30153	PPP2R1A	Protein phosphatase 2 scaffold subunit Aalpha	Serine/threonine-protein phosphatase 2A 65 kDa regulatory subunit A alpha isoform	90	2	0.98	15	3	37
G1SM01		AKAP9	A-kinase anchor protein 9			1	0.98	3	3	1
G1T593	F8VVA7	COPZ1	Clat_adaptor_s domain-containing protein	Coatomer subunit zeta-1	88	2	0.98	4	11	45
G1TE76	Q15056	EIF4H	Eukaryotic translation initiation factor 4H	Eukaryotic translation initiation factor 4H	92	2	0.98	3	6	23
G1SP89	J3QLD9	FLOT2	PHB domain-containing protein	Flotillin-2	97	2	0.97	11	15	34
G1T0R4	Q96JC1	VPS39	CNH domain-containing protein	Vam6/Vps39-like protein	97	2	0.97	3	2	5
	Q8NHH9-2	ATL2		Isoform 2 of Atlastin-2		4	0.97	4	7	11
G1T0T5	F5GYF7	COPS7A	PCI domain-containing protein	COP9 signalosome complex subunit 7a (Fragment)	100	2	0.97	2	4	10
G1T0H9	O60645	EXOC3	Exocyst complex component 3	Exocyst complex component 3	95	2	0.97	3	3	5
G1SS85	Q9UBQ7	GRHPR	Uncharacterized protein	Glyoxylate reductase/hydroxypyruvate reductase	90	3	0.97	5	8	28
G1T8X3		NEU1	Sialidase domain-containing protein			1	0.97	4	5	18
G1SQK0	A0A087X1A5	STAU1	Uncharacterized protein	Double-stranded RNA-binding protein Staufen homolog 1	89	3	0.97	2	2	4
G1SDT9	P49754	VPS41	Vacuolar protein sorting-associated protein 41 homolog	Vacuolar protein sorting-associated protein 41 homolog	98	2	0.97	5	6	8
G1T4K5	A0A1B0GTW1	TJP2	Uncharacterized protein	Tight junction protein ZO-2	87	3	0.97	5	4	8
G1TMM7	H0YN26	ANP32A	LRRcap domain-containing protein	Acidic leucine-rich nuclear phosphoprotein 32 family member A	93	2	0.97	7	9	39
G1SP83	Q9Y2D0	CA5B	Carbonic anhydrase 5B	Carbonic anhydrase 5B, mitochondrial	93	2	0.97	10	32	46
G1SSX5	P56192	MARS	Uncharacterized protein	Methionine--tRNA ligase, cytoplasmic	93	3	0.97	14	26	21
G1SG59	Q7L9L4	MOB1B	Uncharacterized protein	MOB kinase activator 1B	100	3	0.97	2	6	15
G1SPB2		RNMT	mRNA cap guanine-N7 methyltransferase			1	0.97	4	4	11
O46638	Q00688	FKBP3	Peptidyl-prolyl cis-trans isomerase FKBP3	Peptidyl-prolyl cis-trans isomerase FKBP3	96	2	0.97	4	6	21
	O14964	HGS		Hepatocyte growth factor-regulated tyrosine kinase substrate		4	0.97	5	9	13
G1SEQ2		PBDC1	Polysacc_synt_4 domain-containing protein			1	0.97	3	3	16
G1TYK8	Q13492	PICALM	Phosphatidylinositol binding clathrin assembly protein	Phosphatidylinositol-binding clathrin assembly protein	97	2	0.97	12	28	27
G1TXN1		NIT2	CN hydrolase domain-containing protein			1	0.97	3	3	14
G1SKT3	Q9NUJ1	ABHD10	AB hydrolase-1 domain-containing protein	Mycophenolic acid acyl-glucuronide esterase, mitochondrial	88	2	0.96	8	7	36
G1U9S7	P17987	TCP1	Uncharacterized protein	T-complex protein 1 subunit alpha	97	3	0.96	26	374	62
G1SR03	P55072	VCP	Uncharacterized protein	Transitional endoplasmic reticulum ATPase	100	3	0.96	39	177	69
G1SXB8	Q10567	AP1B1	AP complex subunit beta	AP-1 complex subunit beta-1	98	2	0.96	32	5	45
G1SPI7	Q92538	GBF1	SEC7 domain-containing protein	Golgi-specific brefeldin A-resistance guanine nucleotide exchange factor 1	96	2	0.96	18	21	14
G1TNH0	O14939	PLD2	Phospholipase D2	Phospholipase D2	89	2	0.96	3	2	7
G1SNL4	Q9Y385	UBE2J1	UBIQUITIN_CONJUGAT_2 domain-containing protein	Ubiquitin-conjugating enzyme E2 J1	94	2	0.96	3	4	14
G1SQT2	Q6P2E9	EDC4	WD_REPEATS_REGION domain-containing protein	Enhancer of mRNA-decapping protein 4	96	2	0.96	3	3	5
G1U7C7	Q5GLZ8	HERC4	HECT domain-containing protein	Probable E3 ubiquitin-protein ligase HERC4	96	2	0.96	3	3	6
G1TCS1		MTMR9	Myotubularin phosphatase domain-containing protein			1	0.96	2	2	6
G1SPN1		NUDCD2	CS domain-containing protein			1	0.96	2	3	17

G1SCU8		OTUD6B	OTU domain-containing protein			1	0.96	3	3	19
G1SWR1	O43818	RRP9	WD_REPEATS_REGION domain-containing protein	U3 small nucleolar RNA-interacting protein 2	94	2	0.96	5	6	13
G1SH30	Q8WVY7	UBLCP1	Uncharacterized protein	Ubiquitin-like domain-containing CTD phosphatase 1	100	3	0.96	2	2	10
G1SHS4	A0A0C4DGX4	CUL1	CULLIN_2 domain-containing protein	Cullin-1	97	2	0.96	5	8	11
G1SW65		ATP5MC2	ATP-synt_C domain-containing protein			1	0.96	2	2	21
G1T0B0	Q9H3H3	C11orf68	Uncharacterized protein	UPF0696 protein C11orf68	93	3	0.96	2	3	13
G1T3Z6	P31323	PRKAR2B	Uncharacterized protein	cAMP-dependent protein kinase type II-beta regulatory subunit	97	3	0.96	7	7	27
G1SIN4	X6RLX0	ERC1	FIP-RBD domain-containing protein	ELKS/Rab6-interacting/CAST family member 1	98	2	0.96	14	20	15
U3KMI4	P62834	RAP1A	Uncharacterized protein	Ras-related protein Rap-1A	100	3	0.96	8	2	65
	A0A0A0MSZ1	MARK3		Non-specific serine/threonine protein kinase		4	0.96	2	2	4
G1SRB7	A0A2R8Y7U1	TPP1	Peptidase S53 domain-containing protein	Tripeptidyl-peptidase 1 (Fragment)	93	2	0.96	7	14	23
	Q58FF6	HSP90AB4P		Putative heat shock protein HSP 90-beta 4		4	0.96	4	8	9
G1T0N5	Q00577	PURA	Purine rich element binding protein A	Transcriptional activator protein Pur-alpha	91	2	0.96	7	13	40
G1SIX1	Q9H2G2	SLK	Uncharacterized protein	STE20-like serine/threonine-protein kinase	88	3	0.96	6	7	12
G1T9L6		TOR1AIP1	Torsin 1A interacting protein 1			1	0.96	4	5	9
G1SE01	A0A0A0MSI8	EXOC5	Exocyst complex component 5	Exocyst complex component 5	98	2	0.95	4	4	7
G1SLL1	A0A087X0K9	TJP1	Uncharacterized protein	Tight junction protein ZO-1	82	3	0.95	21	26	19
G1SF00	Q9BSJ2	TUBGCP2	Gamma-tubulin complex component	Gamma-tubulin complex component 2	90	2	0.95	5	5	7
G1T103	F8VQQ3	C12orf10	Uncharacterized protein	UPF0160 protein MYG1, mitochondrial	77	3	0.95	4	7	16
G1U013	H7C3P9	COPS3	PCI domain-containing protein	COP9 signalosome complex subunit 3	92	2	0.95	4	4	18
G1T5J8	Q9UJW0	DCTN4	Uncharacterized protein	Dynactin subunit 4	97	3	0.95	6	7	29
G1SUP1	A0A0D9SEY1	MAP4K4	Mitogen-activated protein kinase kinase kinase kinase 4	Mitogen-activated protein kinase kinase kinase kinase 4	91	2	0.95	6	7	7
G1SZH6	Q9BZF1	OSBPL8	Oxysterol-binding protein	Oxysterol-binding protein-related protein 8	99	2	0.95	7	10	11
G1SM15	A0A087WY71	AP2M1	MHD domain-containing protein	AP-2 complex subunit mu	100	2	0.95	15	23	42
G1SQ70	P01023	A2M	Uncharacterized protein	Alpha-2-macroglobulin	78	3	0.95	4	3	3
G1SL52	A0A0A0MS45	COG4	Cog4 domain-containing protein	Conserved oligomeric Golgi complex subunit 4	94	2	0.95	10	10	19
G1SIW8	Q08257	CRYZ	PKS_ER domain-containing protein	Quinone oxidoreductase	87	2	0.95	7	19	39
G1T182	Q86UE4	MTDH	Metadherin	Protein LYRIC	88	2	0.95	4	6	18
G1TBA4	Q86UY8	NT5DC3	Uncharacterized protein	5~-nucleotidase domain-containing protein 3	97	3	0.95	9	12	26
G1TI02	Q9BRP8	PYM1	PYM homolog 1, exon junction complex associated factor	Partner of Y14 and mago	91	2	0.95	2	4	6
G1TER4	P33897	ABCD1	Uncharacterized protein	ATP-binding cassette sub-family D member 1	94	3	0.95	4	3	8
G1SXL6	O94915	FRYL	Uncharacterized protein	Protein furry homolog-like	97	3	0.95	4	5	2
G1TRL5	A0A087WUT6	EIF5B	Tr-type G domain-containing protein	Eukaryotic translation initiation factor 5B	95	2	0.95	21	41	22
G1SM65		GALNT16	Polypeptide N-acetylgalactosaminyltransferase			1	0.95	3	8	16
G1SYC9	H0YNE9	RAB8B	Uncharacterized protein	Ras-related protein Rab-8B (Fragment)	99	3	0.95	5	5	19
G1SJB4	P63244	RACK1	WD_REPEATS_REGION domain-containing protein	Receptor of activated protein C kinase 1	100	2	0.95	16	45	73
G1SU30	A0A024RCR6	BAG6	Ubiquitin-like domain-containing protein	BAG6	91	2	0.95	8	8	12
G1SK22	P62979	RPS27A	Ubiquitin-like domain-containing protein	Ubiquitin-40S ribosomal protein S27a	100	2	0.95	8	45	47
G1SH42	Q5VSL9	STRIP1	Uncharacterized protein	Striatin-interacting protein 1	98	3	0.95	4	3	12
G1SIC4	I3L294	ABHD12	Abhydrolase domain containing 12	Lysophosphatidylserine lipase ABHD12 (Fragment)	99	2	0.95	4	5	18
G1SD02	E7EM64	COPS6	COP9 signalosome subunit 6	COP9 signalosome complex subunit 6	97	2	0.95	3	5	11
G1SRD9	A0A1C7CYX8	FAM107B	Uncharacterized protein	Protein FAM107B (Fragment)	96	3	0.95	2	2	16
G1T8E2	A0A3B3ITW1	GSK3B	Protein kinase domain-containing protein	Glycogen synthase kinase-3 beta	87	2	0.95	2	2	10
G1SM62	O43252	PAPSS1	Uncharacterized protein	Bifunctional 3~-phosphoadenosine 5~-phosphosulfate synthase 1	99	3	0.95	9	15	24
G1SQH0	P61254	RPL26	KOW domain-containing protein	60S ribosomal protein L26	100	2	0.95	6	11	28
G1T7D0		SRRM2	Serine/arginine repetitive matrix 2			1	0.95	2	2	1
G1SUX1		TIMP3	Metalloproteinase inhibitor 3			1	0.95	2	2	13
G1TZ26		GUK1	Guanylate kinase 1			1	0.94	3	6	24
G1SJ43	Q9H3S7	PTPN23	Uncharacterized protein	Tyrosine-protein phosphatase non-receptor type 23	91	3	0.94	7	5	5
G1TMD8		PPP5C	Serine/threonine-protein phosphatase			1	0.94	3	2	10
G1TAN9	P21399	ACO1	Cytoplasmic aconitate hydratase	Cytoplasmic aconitate hydratase	93	2	0.94	5	6	9
G1T279	Q9UHW5	GPN3	GPN-loop GTPase 3	GPN-loop GTPase 3	96	2	0.94	2	3	12
G1SVM1	Q14764	MVP	Uncharacterized protein	Major vault protein	91	3	0.94	35	98	57
G1SGE5	A0A087WZR9	PYCR2	Pyrroline-5-carboxylate reductase	Pyrroline-5-carboxylate reductase	94	2	0.94	6	6	26
	P35908	KRT2		Keratin, type II cytoskeletal 2 epidermal		4	0.94	10	7	25
G1TA15	Q9P2J5	LARS	Uncharacterized protein	Leucine--tRNA ligase, cytoplasmic	95	3	0.94	25	44	29
G1U5L3	P49257	LMAN1	L-type lectin-like domain-containing protein	Protein ERGIC-53	91	2	0.94	15	63	40
G1SNC4	Q5T9B7	AK1	Adenylate kinase isoenzyme 1	Adenylate kinase isoenzyme 1	94	2	0.94	2	4	12
G1T6P5	Q9NVJ2	ARL8B	ADP ribosylation factor like GTPase 8B	ADP-ribosylation factor-like protein 8B	100	2	0.94	7	31	45

G1TII2	O95197	RTN3	Reticulon	Reticulon-3	72	2	0.94	4	5	6
B7NZS0		MYADM	Myeloid-associated differentiation marker (Predicted)			1	0.94	2	8	10
G1SKT1	Q7Z7H5	TMED4	GOLD domain-containing protein	Transmembrane emp24 domain-containing protein 4	94	2	0.94	7	16	44
G1T7R4	P18084	ITGB5	Integrin beta	Integrin beta-5	93	2	0.94	5	6	8
G1T7N4	P41743	PRKCI	Protein kinase C	Protein kinase C iota type	99	2	0.94	2	2	7
G1SM51	Q9UNM6	PSMD13	Proteasome 26S subunit, non-ATPase 13	26S proteasome non-ATPase regulatory subunit 13	92	2	0.94	15	9	46
G1SY93	P63000	RAC1	Rac family small GTPase 1	Ras-related C3 botulinum toxin substrate 1	90	2	0.94	6	10	31
G1TA11	P54136	RARS	Uncharacterized protein	Arginine--tRNA ligase, cytoplasmic	92	3	0.94	23	44	43
G1T2W1	O76094	SRP72	Signal recognition particle subunit SRP72	Signal recognition particle subunit SRP72	98	2	0.94	9	14	20
G1T4S5	Q8NFW8	CMAS	Uncharacterized protein	N-acylneuraminate cytidylyltransferase	96	3	0.93	5	5	11
G1SV05	P34932	HSPA4	Uncharacterized protein	Heat shock 70 kDa protein 4	97	3	0.93	25	22	45
	E7EVJ3	NDST1		Bifunctional heparan sulfate N-deacetylase/N-sulfotransferase 1		4	0.93	2	2	3
G1T9P1		PEAK1	Pseudopodium enriched atypical kinase 1			1	0.93	3	3	3
G1SR93	B8ZZA2	FAM126A	Uncharacterized protein	Hyccin	98	3	0.93	2	2	6
G1TEB0	Q9UKX5	ITGA11	VWFA domain-containing protein	Integrin alpha-11	91	2	0.93	20	40	24
	O60518	RANBP6		Ran-binding protein 6		4	0.93	2	7	3
G1SWM8	A0A0U1RQQ9	SCYL2	Protein kinase domain-containing protein	SCY1-like protein 2	94	2	0.93	2	2	6
G1U0B3	P53007	SLC25A1	Solute carrier family 25 member 1	Tricarboxylate transport protein, mitochondrial	86	2	0.93	10	23	48
G1T369	C9J8R4	DCUN1D1	DCN1-like protein	DCN1-like protein (Fragment)	100	2	0.93	2	3	13
G1TXS2	A0A0D9SFK2	MYO18A	Uncharacterized protein	Unconventional myosin-XVIIIa	94	3	0.93	10	10	7
G1SDA8	P25786	PSMA1	Proteasome endopeptidase complex	Proteasome subunit alpha type-1	100	2	0.93	10	24	49
G1TTY7	M0R1A7	RPL18A	60S ribosomal protein L18a	60S ribosomal protein L18a	90	2	0.93	4	4	19
G1U222	A0A384DVK7	ARHGEF10L	Rho guanine nucleotide exchange factor 10 like	Rho guanine nucleotide exchange factor 10-like protein (Fragment)	93	2	0.93	2	2	4
G1TCY1	Q16513	PKN2	Uncharacterized protein	Serine/threonine-protein kinase N2	97	3	0.93	4	3	4
G1SFG8	A0A096LNH6	DOCK1	Uncharacterized protein	Dedicator of cytokinesis protein 1	95	3	0.93	4	5	4
G1SLF8	J3KN16	ECPAS	Vac14_Fab1_bd domain-containing protein	Proteasome adapter and scaffold protein ECM29	97	2	0.93	18	27	17
G1SPL8		NGB	Neuroglobin			1	0.93	2	2	18
G1SES9	P31939	ATIC	MGS domain-containing protein	Bifunctional purine biosynthesis protein PURH	94	2	0.93	17	27	44
G1SDN3	K7EM18	EIF1	SUI1 domain-containing protein	Eukaryotic translation initiation factor 1	100	2	0.93	5	7	44
G1U9U0	P50991	CCT4	T-complex protein 1 subunit delta	T-complex protein 1 subunit delta	99	2	0.93	23	52	56
G1U9T1	Q99832	CCT7	T-complex protein 1 subunit eta	T-complex protein 1 subunit eta	97	2	0.93	26	7	60
G1STR6	Q96FN9	DTD2	Uncharacterized protein	D-aminoacyl-tRNA deacylase 2	93	3	0.93	2	3	19
G1T2V2	D6RF62	PAICS	AIRC domain-containing protein	Multifunctional protein ADE2	93	2	0.93	8	7	32
G1SJH1	H7BXE3	SLTM	RRM domain-containing protein	SAFB-like transcription modulator (Fragment)	92	2	0.93	3	3	4
G1SCN8	P49368	CCT3	T-complex protein 1 subunit gamma	T-complex protein 1 subunit gamma	98	2	0.93	27	54	64
	P37837	TALDO1		Transaldolase		4	0.93	3	6	11
G1SFQ7		TUBG1	Tubulin gamma chain			1	0.93	5	7	17
G1TRZ2		LAMP1	Lysosomal associated membrane protein 1			1	0.92	5	17	14
G1STQ7		TMEM97	Transmembrane protein 97			1	0.92	2	2	12
G1SJF1	A0A499FIZ0	WDR26	Uncharacterized protein	WD repeat-containing protein 26	99	3	0.92	7	5	18
G1U3G0	Q9C0C9	UBE2O	Ubiquitin conjugating enzyme E2 O	(E3-independent) E2 ubiquitin-conjugating enzyme	96	2	0.92	3	3	4
G1SIB0	Q12907	LMAN2	L-type lectin-like domain-containing protein	Vesicular integral-membrane protein VIP36	98	2	0.92	6	14	23
G1SDD2	B7Z2Y2	COG2	Uncharacterized protein	Conserved oligomeric Golgi complex subunit 2	91	3	0.92	6	10	15
G1SXG8	F8VU90	FKBP11	Peptidylprolyl isomerase	Peptidylprolyl isomerase	95	2	0.92	4	8	31
G1T726	A0A0A0MSE2	HADH	Uncharacterized protein	Hydroxyacyl-coenzyme A dehydrogenase, mitochondrial	92	3	0.92	10	11	43
	O43290	SART1		U4/U6.U5 tri-snRNP-associated protein 1		4	0.92	2	2	5
	M0QYZ2	AP2S1		AP complex subunit sigma		4	0.92	5	11	33
G1U1E5	O60826	CCDC22	Coiled-coil domain containing 22	Coiled-coil domain-containing protein 22	75	2	0.92	6	8	12
G1SZ72	P51116	FXR2	Uncharacterized protein	Fragile X mental retardation syndrome-related protein 2	98	3	0.92	8	5	20
G1TD24	Q9NRY4	ARHGAP35	Rho GTPase activating protein 35	Rho GTPase-activating protein 35	98	2	0.92	4	4	3
G1TTS1		FUNDC2	FUN14 domain containing 2			1	0.92	2	3	12
G1T1U7	A0A494C1T2	MTHFD1	Uncharacterized protein	C-1-tetrahydrofolate synthase, cytoplasmic (Fragment)	90	3	0.92	14	18	20
G1SN52	E7ESC6	XPO7	Exportin 7	Exportin-7	99	2	0.92	5	8	7
G1SG42	Q52LJ0	FAM98B	Uncharacterized protein	Protein FAM98B	94	3	0.92	9	6	34
G1SRL3	P30419	NMT1	Glycylpeptide N-tetradecanoyltransferase	Glycylpeptide N-tetradecanoyltransferase 1	98	2	0.92	7	9	19
G1SJV2	Q7L523	RRAGA	Uncharacterized protein	Ras-related GTP-binding protein A	100	3	0.92	4	8	18
G1T0K1	Q9BTV4	TMEM43	Uncharacterized protein	Transmembrane protein 43	92	3	0.92	10	19	39
G1T9R8	Q16222	UAP1	Uncharacterized protein	UDP-N-acetylhexosamine pyrophosphorylase	96	3	0.92	5	6	14
G1T725	Q8TB40	ABHD4	AB hydrolase-1 domain-containing protein	(Lyso)-N-acylphosphatidylethanolamine lipase	97	2	0.92	2	3	8

G1U3Q0	Q93050	ATP6V0A1	V-type proton ATPase subunit a	V-type proton ATPase 116 kDa subunit a isoform 1	96	2	0.92	12	3	20
G1SN05	Q14240	EIF4A2	Uncharacterized protein	Eukaryotic initiation factor 4A-II	100	3	0.92	14	14	49
G1SDC6	Q9NXC5	MIOS	zinc_ribbon_16 domain-containing protein	GATOR complex protein MIOS	98	2	0.92	2	2	2
G1T1D7	B1ALD9	POSTN	Uncharacterized protein	Periostin	90	3	0.92	16	35	34
G1SYD3	P54577	YARS	Tyrosine--tRNA ligase	Tyrosine--tRNA ligase, cytoplasmic	96	2	0.92	20	33	44
G1SCS8		ANO6	Anoctamin			1	0.92	5	5	8
G1SZ59	P60842	EIF4A1	Eukaryotic initiation factor 4A-I	Eukaryotic initiation factor 4A-I	100	2	0.92	23	190	76
G1SF45		SEC24B	SEC24 homolog B, COPII coat complex component			1	0.92	3	4	4
G1TF67		CORO1A	Coronin			1	0.91	3	2	10
	O14908	GIPC1		PDZ domain-containing protein GIPC1		4	0.91	4	6	16
G1SST7	Q96SL4	GPX7	Glutathione peroxidase	Glutathione peroxidase 7	92	2	0.91	6	9	36
G1TE61	O60684	KPNA6	Importin subunit alpha	Importin subunit alpha-7	99	2	0.91	10	16	34
	O43765	SGTA		Small glutamine-rich tetratricopeptide repeat-containing protein alpha		4	0.91	2	3	8
G1T6N8		ALG12	Mannosyltransferase			1	0.91	2	2	9
G1STW7	Q9NSD9	FARSB	B5 domain-containing protein	Phenylalanine--tRNA ligase beta subunit	95	2	0.91	11	17	22
G1SL02	P63010	AP2B1	AP complex subunit beta	AP-2 complex subunit beta	98	2	0.91	41	56	57
U3KM64		CLTA	Clathrin light chain			1	0.91	7	27	28
	Q9UNE7	STUB1		E3 ubiquitin-protein ligase CHIP		4	0.91	2	2	9
A0A140TAV6		HBB2	Globin A1			1	0.91	2	2	13
G1TDN6		KRT5	IF rod domain-containing protein			1	0.91	5	2	8
O19048	Q15365	PCBP1	Poly(rC)-binding protein 1	Poly(rC)-binding protein 1	100	2	0.91	14	25	64
G1T9V4	G3V5Z7	PSMA6	Proteasome subunit alpha type	Proteasome subunit alpha type	97	2	0.91	10	56	47
G1SXI1		TTC27	TPR_REGION domain-containing protein			1	0.91	3	4	7
G1SV40	P83436	COG7	Uncharacterized protein	Conserved oligomeric Golgi complex subunit 7	93	3	0.91	5	7	10
P63169	F8VRV5	DYNLL1	Dynein light chain 1, cytoplasmic	Dynein light chain	100	2	0.91	2	6	43
G1TCK9	A0A0A0MSX9	IARS	Uncharacterized protein	Isoleucine--tRNA ligase, cytoplasmic	94	3	0.91	33	54	34
G1SDR2	P24844	MYL9	Uncharacterized protein	Myosin regulatory light polypeptide 9	99	3	0.91	10	17	80
G1SN68	A0A1B0GVU9	QARS	Uncharacterized protein	Glutamine--tRNA ligase (Fragment)	92	3	0.91	25	36	43
G1T6B3	Q9Y3F4	STRAP	WD_REPEATS_REGION domain-containing protein	Serine-threonine kinase receptor-associated protein	98	2	0.91	13	29	52
G1SWK5	A0A1W2PNP0	PIGT	Uncharacterized protein	GPI transamidase component PIG-T (Fragment)	86	3	0.91	3	3	8
G1U4H9	O14907	TAX1BP3	Tax1-binding protein 3	Tax1-binding protein 3	100	2	0.91	2	4	28
G1T3M5	O43324	EEF1E1	GST C-terminal domain-containing protein	Eukaryotic translation elongation factor 1 epsilon-1	95	2	0.91	6	7	44
G1SMX7	O43731	KDELR3	ER lumen protein-retaining receptor	ER lumen protein-retaining receptor 3	98	2	0.91	3	2	17
G1T4C9	Q8IVL6	P3H3	Prolyl 3-hydroxylase 3	Prolyl 3-hydroxylase 3	88	2	0.91	14	20	29
G1SJ87	Q9H2D6	TRIOBP	TRIO and F-actin binding protein	TRIO and F-actin-binding protein	73	2	0.91	3	3	2
G1SET5		CEMIP2	G8 domain-containing protein			1	0.91	4	2	5
G1SCL6	A0A494C1J1	SPECC1L	Calponin-homology (CH) domain-containing protein	Cytospin-A	90	2	0.91	12	14	15
G1TPB1		CRAT	Carnitine O-acetyltransferase			1	0.90	2	2	8
G1SXI8		PCID2	PCI domain containing 2			1	0.90	2	2	6
G1T9V7		SRP19	Signal recognition particle 19			1	0.90	2	2	26
G1TIP5	C9JCC6	DRAP1	CBFD_NFYB_HMF domain-containing protein	Dr1-associated corepressor	87	2	0.90	2	3	11
G1SPV0	P61086	UBE2K	Uncharacterized protein	Ubiquitin-conjugating enzyme E2 K	100	3	0.90	4	6	35
G1SZQ7	Q7L1Q6	BZW1	W2 domain-containing protein	Basic leucine zipper and W2 domain-containing protein 1	100	2	0.90	12	16	39
G1T0B4	Q9Y3B3	TMED7	GOLD domain-containing protein	Transmembrane emp24 domain-containing protein 7	92	2	0.90	7	19	52
G1TIZ5	Q8NBS9	TXNDC5	Uncharacterized protein	Thioredoxin domain-containing protein 5	87	3	0.90	13	24	39
G1T3N8	Q13155	AIMP2	Uncharacterized protein	Aminoacyl tRNA synthase complex-interacting multifunctional protein 2	87	3	0.90	12	22	68
G1SJU2	O60306	AQR	RNA helicase aquarius	RNA helicase aquarius	96	2	0.90	3	2	4
G1U8J5		ATP5PF	ATP synthase-coupling factor 6, mitochondrial			1	0.90	3	3	33
G1SN85	A0A0D9SEN1	FAP	Uncharacterized protein	Prolyl endopeptidase FAP	95	3	0.90	27	85	40
G1SI20	O75874	IDH1	Isocitrate dehydrogenase [NADP]	Isocitrate dehydrogenase [NADP] cytoplasmic	97	2	0.90	19	16	49
G1SKM5	Q8TAT6	NPLOC4	NPL4 homolog, ubiquitin recognition factor	Nuclear protein localization protein 4 homolog	92	2	0.90	5	5	12
G1T235	P28072	PSMB6	Proteasome subunit beta	Proteasome subunit beta type-6	97	2	0.90	6	12	40
G1TV43	O60218	AKR1B10	Aldo_ket_red domain-containing protein	Aldo-keto reductase family 1 member B10	86	2	0.90	10	18	31
G1SRA8	P41091	EIF2S3	Eukaryotic translation initiation factor 2 subunit 3	Eukaryotic translation initiation factor 2 subunit 3	92	2	0.90	12	23	36
G1SNP9	P45954	ACADSB	Acyl-CoA dehydrogenase short/branched chain	Short/branched chain specific acyl-CoA dehydrogenase, mitochondrial	88	2	0.90	18	132	55
G1SJ41	Q9Y6Y8	SEC23IP	DDHD domain-containing protein	SEC23-interacting protein	89	2	0.90	14	21	16
	F8VV64	TNS2		Tensin-2		4	0.90	2	3	2
G1SMZ9		ICMT	Protein-S-isoprenylcysteine O-methyltransferase			1	0.90	2	2	11
G1T4Q9	P28074	PSMB5	Proteasome subunit beta	Proteasome subunit beta type-5	98	2	0.90	9	52	47

G1T8H8	P08183	ABCB1	Uncharacterized protein	ATP-dependent translocase ABCB1	88	3	0.90	5	14	7
G1T9F3	Q14974	KPNB1	Importin N-terminal domain-containing protein	Importin subunit beta-1	99	2	0.90	28	92	50
G1SQ96	Q5H9R7	PPP6R3	Uncharacterized protein	Serine/threonine-protein phosphatase 6 regulatory subunit 3	92	3	0.90	7	7	13
G1SLK2	P62195	PSMC5	AAA domain-containing protein	26S proteasome regulatory subunit 8	100	2	0.90	14	24	43
G1SHN4	Q96JB2	COG3	Uncharacterized protein	Conserved oligomeric Golgi complex subunit 3	96	3	0.90	4	5	10
	Q9UBF2	COPG2		Coatomer subunit gamma-2		4	0.90	12	4	18
G1SJ72		CSPG4	Chondroitin sulfate proteoglycan 4			1	0.90	19	23	17
G1TBJ8		RB1CC1	RB1 inducible coiled-coil 1			1	0.90	3	2	3
G1T044	A0A3B3IUC4	GLA	Alpha-galactosidase	Alpha-galactosidase	75	2	0.89	3	3	7
G1SHK8	O94925	GLS	ANK_REP_REGION domain-containing protein	Glutaminase kidney isoform, mitochondrial	97	2	0.89	21	11	48
G1TE64	O95758	PTBP3	Uncharacterized protein	Polypyrimidine tract-binding protein 3	97	3	0.89	5	2	20
G1T2N8	O43592	XPOT	Exportin-T	Exportin-T	99	2	0.89	4	5	6
P30946	P07900	HSP90AA1	Heat shock protein HSP 90-alpha	Heat shock protein HSP 90-alpha	94	2	0.89	36	255	63
G1SIV7	O43776	NARS	AA_TRNA_LIGASE_II domain-containing protein	Asparagine--tRNA ligase, cytoplasmic	91	2	0.89	15	35	36
G1U0U5	E9PDE8	HSPA4L	Uncharacterized protein	Heat shock 70 kDa protein 4L	91	3	0.89	5	4	8
G1TJR5	Q9NZL4	HSPBP1	HSPA (Hsp70) binding protein 1	Hsp70-binding protein 1	96	2	0.89	4	6	22
G1STJ8	G3V394	MYO5A	Uncharacterized protein	Unconventional myosin-Va	96	3	0.89	17	3	12
G1SSN2		SIRT5	NAD-dependent protein deacylase sirtuin-5, mitochondrial			1	0.89	2	2	10
	Q14318	FKBP8		Peptidyl-prolyl cis-trans isomerase FKBP8		4	0.89	3	6	15
	P62166	NCS1		Neuronal calcium sensor 1		4	0.89	2	2	24
G1TBU8	P52306	RAP1GDS1	Uncharacterized protein	Rap1 GTPase-GDP dissociation stimulator 1	97	3	0.89	6	8	15
G1SL38	F8VS81	TWF1	Twinfilin actin binding protein 1	Twinfilin-1 (Fragment)	96	2	0.89	7	12	25
G1T4N5	P42345	MTOR	Serine/threonine-protein kinase mTOR	Serine/threonine-protein kinase mTOR	99	2	0.89	16	16	11
G1SIP1	Q96LJ7	DHRS1	Uncharacterized protein	Dehydrogenase/reductase SDR family member 1	87	3	0.89	6	6	30
G1T645	Q96S52	PIGS	Uncharacterized protein	GPI transamidase component PIG-S	85	3	0.89	6	6	17
G1SJX1	Q16537	PPP2R5E	Serine/threonine-protein phosphatase 2A 56 kDa regulatory subunit	Serine/threonine-protein phosphatase 2A 56 kDa regulatory subunit epsilon isoform	100	2	0.89	4	5	13
G1SZ19	Q9Y3L5	RAP2C	Uncharacterized protein	Ras-related protein Rap-2c	100	3	0.89	6	4	38
G1T7U4	Q9Y4E8	USP15	Ubiquitin carboxyl-terminal hydrolase	Ubiquitin carboxyl-terminal hydrolase 15	99	2	0.89	4	3	7
G1T365	Q9H269	VPS16	Vacuolar protein sorting-associated protein 16 homolog	Vacuolar protein sorting-associated protein 16 homolog	98	2	0.89	6	8	15
G1SLS3	O43747	AP1G1	AP-1 complex subunit gamma	AP-1 complex subunit gamma-1	100	2	0.89	8	11	16
B6V9S9	P78371	CCT2	Chaperonin-containing T-complex polypeptide beta subunit	T-complex protein 1 subunit beta	99	2	0.89	27	147	68
G1TBT4	Q9HB90	RRAGC	Uncharacterized protein	Ras-related GTP-binding protein C	98	3	0.89	5	8	23
G1SHZ8	P50990	CCT8	Uncharacterized protein	T-complex protein 1 subunit theta	97	3	0.89	33	70	71
G1SZW8	M0R165	EPS15L1	Epidermal growth factor receptor pathway substrate 15 like 1	Epidermal growth factor receptor substrate 15-like 1	88	2	0.89	9	10	16
G1SPJ5	Q92616	GCN1	TOG domain-containing protein	eIF-2-alpha kinase activator GCN1	96	2	0.89	46	82	27
G1T1X2	A0A087WZF1	LPP	Uncharacterized protein	Lipoma-preferred partner	89	3	0.89	4	6	8
	Q9Y224	RTRAF		RNA transcription, translation and transport factor protein		4	0.89	10	42	50
G1T8S0	Q9C0D5	TANC1	Uncharacterized protein	Protein TANC1	85	3	0.89	8	5	9
	A6NG10	WBP2		WW domain-binding protein 2		4	0.89	2	2	7
G1SRL4		NAGA	Alpha-galactosidase			1	0.88	14	29	47
G1TIZ2	Q02809	PLOD1	Procollagen-lysine,2-oxoglutarate 5-dioxygenase 1	Procollagen-lysine,2-oxoglutarate 5-dioxygenase 1	93	2	0.88	26	18	41
	P62701	RPS4X		40S ribosomal protein S4, X isoform		4	0.88	15	36	52
G1T6E8	F5H442	TSG101	Uncharacterized protein	Tumor susceptibility gene 101 protein	99	3	0.88	6	8	25
G1SNS3	Q6P4E1	CASC4	Uncharacterized protein	Protein CASC4	89	3	0.88	7	9	16
G1TMS5	P48643	CCT5	Uncharacterized protein	T-complex protein 1 subunit epsilon	99	3	0.88	31	85	70
G1SYM3		CD9	Tetraspanin			1	0.88	3	4	7
	A0A1B0GV23	CTSD		Cathepsin D		4	0.88	6	18	14
G1T846	P14868	DARS	AA_TRNA_LIGASE_II domain-containing protein	Aspartate--tRNA ligase, cytoplasmic	98	2	0.88	10	21	22
G1T9M9	P11142	HSPA8	Uncharacterized protein	Heat shock cognate 71 kDa protein	100	3	0.88	32	668	71
G1SMG1		KANK1	KN motif and ankyrin repeat domains 1			1	0.88	6	4	10
G1SDK8	Q13564	NAE1	NEDD8-activating enzyme E1 regulatory subunit	NEDD8-activating enzyme E1 regulatory subunit	96	2	0.88	3	3	11
G1SFE9	H0YJG7	AHSA1	Aha1_N domain-containing protein	Activator of 90 kDa heat shock protein ATPase homolog 1 (Fragment)	95	2	0.88	3	4	14
G1SRP7	O00505	KPNA3	Importin subunit alpha	Importin subunit alpha-4	99	2	0.88	8	6	27
G1TW66		DMAC2	Distal membrane arm assembly complex 2			1	0.88	2	2	17
G1SIJ8	Q8TEX9	IPO4	Importin N-terminal domain-containing protein	Importin-4	89	2	0.88	6	8	10
G1SWK8	Q5TBG5	PSMB7	Proteasome subunit beta	Proteasome subunit beta (Fragment)	84	2	0.88	6	62	25
B7NZQ3		RA_m006_jsm824E4r	Deoxyribonuclease			1	0.88	6	13	28
G1T964		TMED1	GOLD domain-containing protein			1	0.88	3	6	27
G1T157	Q9Y376	CAB39	Uncharacterized protein	Calcium-binding protein 39	99	3	0.88	3	2	8

	P13639	EEF2		Elongation factor 2		4	0.88	37	261	58
	A0A0A0MRE1	EXOC7		Exocyst complex component 7 (Fragment)		4	0.88	3	6	7
G1SU80	O60462	NRP2	Neuropilin	Neuropilin-2	95	2	0.88	17	20	25
G1TP15	O43242	PSMD3	PCI domain-containing protein	26S proteasome non-ATPase regulatory subunit 3	98	2	0.88	20	33	43
G1T7Q2		LOXL2	Lysyl oxidase like 2			1	0.88	6	6	9
G1TD98		GSR	Glutathione reductase			1	0.88	2	3	7
G1SEU9	Q9UHA4	LAMTOR3	Uncharacterized protein	Ragulator complex protein LAMTOR3	98	3	0.88	4	6	40
G1SFU4	Q9UIQ6	LNPEP	Uncharacterized protein	Leucyl-cystinyl aminopeptidase	90	3	0.88	14	18	17
	Q9UQ13	SHOC2		Leucine-rich repeat protein SHOC-2		4	0.88	2	4	10
G1TZV3	E7ENJ6	AP1M1	Adaptor related protein complex 1 subunit mu 1	AP-1 complex subunit mu-1	75	2	0.88	8	13	33
G1SZ18	A0A0C4DFT3	DLG1	Uncharacterized protein	Disks large homolog 1	93	3	0.88	4	4	7
G1SFE0	O00487	PSMD14	MPN domain-containing protein	26S proteasome non-ATPase regulatory subunit 14	100	2	0.88	7	10	45
P29694	P26641	EEF1G	Elongation factor 1-gamma	Elongation factor 1-gamma	98	2	0.88	21	73	64
G1SL53	Q66K14	TBC1D9B	Uncharacterized protein	TBC1 domain family member 9B	89	3	0.88	3	2	3
G1TZ31	Q9Y680	FKBP7	Peptidylprolyl isomerase	Peptidyl-prolyl cis-trans isomerase FKBP7	77	2	0.88	11	26	44
G1SZH8	Q8N1B4	VPS52	Uncharacterized protein	Vacuolar protein sorting-associated protein 52 homolog	99	3	0.88	4	3	12
G1T275	Q8WU90	ZC3H15	Uncharacterized protein	Zinc finger CCCH domain-containing protein 15	98	3	0.88	3	2	9
G1SL98	Q53EP0	FNDC3B	Uncharacterized protein	Fibronectin type III domain-containing protein 3B	97	3	0.87	15	4	20
G1T4H0	Q9NYL9	TMOD3	Uncharacterized protein	Tropomodulin-3	93	3	0.87	15	22	50
G1T573	H0Y3P2	EIF4G2	Eukaryotic translation initiation factor 4 gamma 2	Eukaryotic translation initiation factor 4 gamma 2	95	2	0.87	18	33	26
G1U522	P13861	PRKAR2A	Uncharacterized protein	cAMP-dependent protein kinase type II-alpha regulatory subunit	90	3	0.87	10	10	34
G1T748	Q5VZU9	TPP2	Uncharacterized protein	Tripeptidyl-peptidase 2	97	3	0.87	26	31	25
G1SG72	P61221	ABCE1	Uncharacterized protein	ATP-binding cassette sub-family E member 1	100	3	0.87	14	22	32
G1TQR2	P24534	EEF1B2	Uncharacterized protein	Elongation factor 1-beta	98	3	0.87	10	23	65
O77622		CCT6	T-complex protein 1 subunit zeta			1	0.87	23	15	54
G1T520	Q99615	DNAJC7	Uncharacterized protein	DnaJ homolog subfamily C member 7	98	3	0.87	3	3	10
G1SU71	P20618	PSMB1	Proteasome subunit beta	Proteasome subunit beta type-1	94	2	0.87	8	11	42
G1SPD2	Q16401	PSMD5	Uncharacterized protein	26S proteasome non-ATPase regulatory subunit 5	92	3	0.87	12	13	43
	Q96BM9	ARL8A		ADP-ribosylation factor-like protein 8A		4	0.87	7	2	45
G1SCY4	P52907	CAPZA1	F-actin-capping protein subunit alpha	F-actin-capping protein subunit alpha-1	96	2	0.87	10	22	58
G1SSV1	Q99627	COPS8	PCI domain-containing protein	COP9 signalosome complex subunit 8	99	2	0.87	4	11	38
G1T087	Q93034	CUL5	Cullin-5	Cullin-5	100	2	0.87	4	15	10
G1SQY8	Q9Y266	NUDC	CS domain-containing protein	Nuclear migration protein nudC	97	2	0.87	10	11	31
G1SNZ3	P55060	CSE1L	Chromosome segregation 1 like	Exportin-2	99	2	0.87	13	29	26
G1T9Q5		SLC44A2	Solute carrier family 44 member 2			1	0.87	3	5	6
G1TN86	C9IZG4	CUTA	Uncharacterized protein	Protein CutA	93	3	0.86	2	2	18
	Q9UJY5	GGA1		ADP-ribosylation factor-binding protein GGA1		4	0.86	3	4	9
G1TEY6	O00178	GTPBP1	Tr-type G domain-containing protein	GTP-binding protein 1	94	2	0.86	5	6	12
B7NZM8	Q04917	YWHAH	Tyrosine 3-monooxygenase/tryptophan 5-monooxygenase activation protein, eta polypeptide (Predicted)	14-3-3 protein eta	99	2	0.86	14	33	65
G1T7Y7	B1AK87	CAPZB	F-actin-capping protein subunit beta	F-actin-capping protein subunit beta	100	2	0.86	11	35	54
G1T512		CNP	2~,3~-cyclic nucleotide 3~ phosphodiesterase			1	0.86	2	3	7
Q28647		PPP2R5B	Serine/threonine-protein phosphatase 2A 56 kDa regulatory subunit beta isoform			1	0.86	2	2	7
G1T6C0	P61020	RAB5B	Uncharacterized protein	Ras-related protein Rab-5B	100	3	0.86	9	33	56
G1SLU5	Q9UKZ1	CNOT11	CCR4-NOT transcription complex subunit 11	CCR4-NOT transcription complex subunit 11	79	2	0.86	2	2	6
	P55010	EIF5		Eukaryotic translation initiation factor 5		4	0.86	5	8	15
G1TLD3	Q02818	NUCB1	Nucleobindin 1	Nucleobindin-1	87	2	0.86	17	19	45
G1SVA3	O00231	PSMD11	PCI domain-containing protein	26S proteasome non-ATPase regulatory subunit 11	100	2	0.86	17	37	56
G1TEM4	Q9UEW8	STK39	Protein kinase domain-containing protein	STE20/SPS1-related proline-alanine-rich protein kinase	95	2	0.86	2	2	4
G1SGR0	Q8N3P4	VPS8	Uncharacterized protein	Vacuolar protein sorting-associated protein 8 homolog	93	3	0.86	2	3	3
G1SF26	P53675	CLTCL1	Clathrin heavy chain	Clathrin heavy chain 2	91	2	0.86	17	4	13
P58776	A7XZE4	TPM2	Tropomyosin beta chain	Beta tropomyosin isoform	94	2	0.86	24	28	63
G1T578	E9PHY0	ACP2	Uncharacterized protein	Lysosomal acid phosphatase	94	3	0.86	4	8	14
G1SJN4	Q9BT78	COPS4	PCI domain-containing protein	COP9 signalosome complex subunit 4	100	2	0.86	12	23	49
G1U8F0	O95782	AP2A1	AP-2 complex subunit alpha	AP-2 complex subunit alpha-1	98	2	0.86	32	3	46
G1SEJ4	P21281	ATP6V1B2	Vacuolar proton pump subunit B	V-type proton ATPase subunit B, brain isoform	99	2	0.86	18	33	55
G1TJA8		FNBP1	Formin binding protein 1			1	0.86	3	2	4
G1SDU5	H3BPE1	MACF1	Uncharacterized protein	Microtubule-actin cross-linking factor 1, isoforms 1/2/3/5	88	3	0.86	203	149	38
G1TLQ8	R4GNH3	PSMC3	AAA domain-containing protein	26S proteasome regulatory subunit 6A	100	2	0.86	21	48	65
G1SSA2	Q13200	PSMD2	26S proteasome non-ATPase regulatory subunit 2	26S proteasome non-ATPase regulatory subunit 2	99	2	0.86	30	76	47

G1TI39	P35241	RDX	FERM domain-containing protein	Radixin	99	2	0.86	17	16	31
G1SQD1	E7EUU4	EIF4G1	Eukaryotic translation initiation factor 4 gamma 1	Eukaryotic translation initiation factor 4 gamma 1	94	2	0.86	21	33	15
G1SVG6	P21359	NF1	Uncharacterized protein	Neurofibromin	99	3	0.86	4	6	3
	P54725	RAD23A		UV excision repair protein RAD23 homolog A		4	0.86	5	11	23
G1T466	A0AVT1	UBA6	UBA_e1_C domain-containing protein	Ubiquitin-like modifier-activating enzyme 6	93	2	0.86	7	6	10
G1T2I7	Q9HCJ1	ANKH	Uncharacterized protein	Progressive ankylosis protein homolog	99	3	0.85	3	4	13
G1TDH8		CLCN5	Chloride channel protein			1	0.85	4	4	13
G1SGD9		ATG7	Ubiquitin-like modifier-activating enzyme ATG7			1	0.85	2	2	6
G1SVT4	P51665	PSMD7	MPN domain-containing protein	26S proteasome non-ATPase regulatory subunit 7	99	2	0.85	9	15	38
G1SL80		UROD	Uroporphyrinogen decarboxylase			1	0.85	4	4	19
G1SQ38	E5RHK8	DNM3	Uncharacterized protein	Dynamin-3	97	3	0.85	9	2	12
G1SR53	P04066	FUCA1	Alpha-L-fucosidase	Tissue alpha-L-fucosidase	83	2	0.85	10	26	34
G1TD47	Q8N3E9	PLCD3	Phosphoinositide phospholipase C	1-phosphatidylinositol 4,5-bisphosphate phosphodiesterase delta-3	88	2	0.85	4	5	9
G1SVF2	A0A087WW66	PSMD1	26S proteasome non-ATPase regulatory subunit 1	26S proteasome non-ATPase regulatory subunit 1	99	2	0.85	29	8	44
G1SCY3		UBR4	UBR-type domain-containing protein			1	0.85	46	72	17
G1TRH5	Q9ULV4	CORO1C	Coronin	Coronin-1C	97	2	0.85	19	69	44
G1T4H6		MTMR6	Myotubularin phosphatase domain-containing protein			1	0.85	3	2	4
G1SQR7	P61970	NUTF2	NTF2 domain-containing protein	Nuclear transport factor 2	100	2	0.85	2	2	17
G1SXH7	P12931	SRC	Tyrosine-protein kinase	Proto-oncogene tyrosine-protein kinase Src	96	2	0.85	5	5	14
G1TBY1		CTSB	Pept_C1 domain-containing protein			1	0.85	7	50	30
G1TKL0	M0QZG7	SNRPA	Small nuclear ribonucleoprotein polypeptide A	U1 small nuclear ribonucleoprotein A (Fragment)	60	2	0.85	4	3	14
G1SII9	Q5QJ74	TBCEL	Ubiquitin-like domain-containing protein	Tubulin-specific chaperone cofactor E-like protein	99	2	0.85	2	2	8
G1SRT1	Q9UKG1	APPL1	Adaptor protein, phosphotyrosine interacting with PH domain and leucine zipper 1	DCC-interacting protein 13-alpha	98	2	0.85	8	7	19
G1TBL6	A0A087WVQ6	CLTC	Clathrin heavy chain	Clathrin heavy chain	99	2	0.85	80	521	63
G1T2I4	P07814	EPRS	Glutamyl-prolyl-tRNA synthetase	Bifunctional glutamate/proline--tRNA ligase	89	2	0.85	54	64	46
G1SWS6	A0A3B3IRN5	FMOD	Fibromodulin	Fibromodulin	92	2	0.85	2	2	7
G1T302		THYN1	EVE domain-containing protein			1	0.85	2	5	10
G1SHS8	A0A087WY55	VTA1	Uncharacterized protein	Chromosome 6 open reading frame 55, isoform CRA_b	87	3	0.85	3	5	15
G1SFN5	E9PFR3	PPP2R5D	Serine/threonine-protein phosphatase 2A 56 kDa regulatory subunit	Serine/threonine-protein phosphatase 2A 56 kDa regulatory subunit	98	2	0.85	6	9	14
G1TFM5	M0R0F0	RPS5	Ribosomal_S7 domain-containing protein	40S ribosomal protein S5 (Fragment)	100	2	0.85	8	59	56
G1SJX5	A0A087WTF3	ANK3	Ankyrin 3	Ankyrin-3 (Fragment)	93	2	0.85	3	3	4
P41035	P20042	EIF2S2	Eukaryotic translation initiation factor 2 subunit 2	Eukaryotic translation initiation factor 2 subunit 2	98	2	0.84	12	25	53
U3KNL7	Q06210	GFPT1	Uncharacterized protein	Glutamine--fructose-6-phosphate aminotransferase [isomerizing] 1	95	3	0.84	15	4	40
	Q15366-3	PCBP2		Isoform 3 of Poly(rC)-binding protein 2		4	0.84	13	3	56
G1SIS5	O15498	YKT6	Uncharacterized protein	Synaptobrevin homolog YKT6	96	3	0.84	5	6	36
G1T860	M0R0P8	MYO9B	Myosin IXB	Unconventional myosin-IXb	83	2	0.84	13	9	13
G1T2I6	P45877	PPIC	Peptidyl-prolyl cis-trans isomerase	Peptidyl-prolyl cis-trans isomerase C	92	2	0.84	6	18	48
G1SWL6	Q9P0K7	RAI14	ANK_REP_REGION domain-containing protein	Ankycorbin	91	2	0.84	24	43	32
G1SCQ1		AKR7L	Aldo_ket_red domain-containing protein			1	0.84	7	6	35
G1U1H1	Q00535	CDK5	Protein kinase domain-containing protein	Cyclin-dependent-like kinase 5	100	2	0.84	3	2	12
	P21333	FLNA		Filamin-A		4	0.84	105	11	51
G1T3U1	Q9H8Y8	GORASP2	GRASP55_65 domain-containing protein	Golgi reassembly-stacking protein 2	91	2	0.84	6	15	18
G1T358	Q96AG3	SLC25A46	Uncharacterized protein	Solute carrier family 25 member 46	94	3	0.84	3	4	18
G1T0H3	P40763	STAT3	Signal transducer and activator of transcription	Signal transducer and activator of transcription 3	100	2	0.84	8	13	17
G1TDI6		BLOC1S6	Biogenesis of lysosome-related organelles complex 1 subunit 6			1	0.84	2	2	25
G1SKG9	A0A087X2H1	HECTD1	Uncharacterized protein	E3 ubiquitin-protein ligase HECTD1	99	3	0.84	7	8	4
G1SIA1	A0A087WWA3	KIF1B	Uncharacterized protein	Kinesin-like protein KIF1B	96	3	0.84	3	5	2
G1SDL9	Q9UEU0	VTI1B	t-SNARE coiled-coil homology domain-containing protein	Vesicle transport through interaction with t-SNAREs homolog 1B	93	2	0.84	4	6	24
G1SMP3	A0A2R8Y6F8	CASK	Uncharacterized protein	Peripheral plasma membrane protein CASK	97	3	0.84	12	14	17
G1SH09	Q5VIR6	VPS53	VPS53, GARP complex subunit	Vacuolar protein sorting-associated protein 53 homolog	95	2	0.84	4	4	11
Q9GLC3		ATP1B3	Sodium/potassium-transporting ATPase subunit beta-3			1	0.84	3	4	15
G1THP8	P46734	MAP2K3	Protein kinase domain-containing protein	Dual specificity mitogen-activated protein kinase kinase 3	97	2	0.84	6	8	29
G1T845	I3L0N3	NSF	Uncharacterized protein	Vesicle-fusing ATPase	99	3	0.84	17	29	33
G1SYN5	J3QRU4	VAMP2	V-SNARE coiled-coil homology domain-containing protein	Vesicle-associated membrane protein 2	99	2	0.84	4	4	42
G1STX4	P61011	SRP54	Signal recognition particle 54 kDa protein	Signal recognition particle 54 kDa protein	99	2	0.84	13	14	38
G1SYB4	P60953	CDC42	Uncharacterized protein	Cell division control protein 42 homolog	100	3	0.83	7	24	49
G1TXW6		GPNMB	PKD domain-containing protein			1	0.83	2	5	5
G1SCI5	F5H6E2	MYO1C	Uncharacterized protein	Unconventional myosin-Ic	92	3	0.83	40	99	46
	Q9UL15	BAG5		BAG family molecular chaperone regulator 5		4	0.83	2	2	7

G1TUP1	O75340	PDCD6	Programmed cell death 6	Programmed cell death protein 6	99	2	0.83	5	15	29
G1SYV0	P35998	PSMC2	AAA domain-containing protein	26S proteasome regulatory subunit 7	100	2	0.83	17	33	46
G1SE49	Q9UID3	VPS51	Uncharacterized protein	Vacuolar protein sorting-associated protein 51 homolog	96	3	0.83	6	7	15
G1T1T4		ADPGK	ADP dependent glucokinase			1	0.83	9	14	27
G1TT64	Q68EM7	ARHGAP17	Uncharacterized protein	Rho GTPase-activating protein 17	91	3	0.83	4	3	8
G1SQ12	Q8TDJ6	DMXL2	WD_REPEATS_REGION domain-containing protein	DmX-like protein 2	93	2	0.83	2	2	1
G1SFH5	O00469-2	PLOD2	Fe2OG dioxygenase domain-containing protein		90	2	0.83	30	57	47
G1TYN0	Q9BVK6	TMED9	Transmembrane p24 trafficking protein 9	Transmembrane emp24 domain-containing protein 9	80	2	0.83	9	8	35
G1TM00	O75436	VPS26A	VPS26, retromer complex component A	Vacuolar protein sorting-associated protein 26A	99	2	0.83	2	2	10
G1T3E6	A0A024R442	DNPEP	Uncharacterized protein	Aspartyl aminopeptidase	91	3	0.83	9	9	30
	Q9NZN4	EHD2		EH domain-containing protein 2		4	0.83	11	14	27
G1SYR5		EIF2B2	Translation initiation factor eIF-2B subunit beta			1	0.83	2	3	8
G1SWN1	Q96H20	SNF8	Vacuolar-sorting protein SNF8	Vacuolar-sorting protein SNF8	100	2	0.83	3	3	24
G1T295		EPHX1	Epoxide hydrolase			1	0.83	5	6	15
G1SCW0	A8MT72	RTN1	Reticulon	Reticulon	98	2	0.83	2	3	3
G1SDQ5	Q96JG6	VPS50	Uncharacterized protein	Syndetin	98	3	0.83	4	6	9
	P23142	FBLN1		Fibulin-1		4	0.83	2	2	3
G1T3S1	A0A087X2I1	PSMC6	AAA domain-containing protein	26S proteasome regulatory subunit 10B	100	2	0.83	12	25	42
G1TFL3	J3KQE5	RAN	GTP-binding nuclear protein Ran	GTP-binding nuclear protein Ran (Fragment)	96	2	0.83	9	24	36
G1TB18	Q07960	ARHGAP1	Uncharacterized protein	Rho GTPase-activating protein 1	95	3	0.83	5	7	16
G1SIA6	Q5T2E6	ARMH3	DUF1741 domain-containing protein	Armadillo-like helical domain-containing protein 3	99	2	0.83	4	4	11
G1STY8	G3V5E4	GNPNAT1	Glucosamine 6-phosphate N-acetyltransferase	Glucosamine 6-phosphate N-acetyltransferase	100	2	0.83	4	4	20
G1T8P7	A0A2R8YFH5	SEC23B	Protein transport protein SEC23	Protein transport protein SEC23	95	2	0.83	8	7	15
G1U6B2		ALAD	Delta-aminolevulinic acid dehydratase			1	0.83	2	2	15
G1TF32	F5H6I7	ATL3	Atlastin GTPase 3	Atlastin-3	95	2	0.83	15	45	44
G1T2Z8	B4DUC8	MTAP	S-methyl-5~-thioadenosine phosphorylase	S-methyl-5~-thioadenosine phosphorylase	99	2	0.83	4	6	27
G1SHV9	P49720	PSMB3	Proteasome subunit beta	Proteasome subunit beta type-3	99	2	0.83	6	18	39
G1TH06	F6WQW2	RANBP1	RAN binding protein 1	Ran-specific GTPase-activating protein	91	2	0.83	4	7	28
G1U3B8	K7ER96	TXNL1	PITH domain-containing protein	Thioredoxin-like protein 1 (Fragment)	100	2	0.83	5	5	30
G1SUR8	B7ZBJ4	CAB39L	Uncharacterized protein	Calcium-binding protein 39-like	98	3	0.82	3	3	12
G1U3S6	O15258	RER1	Protein RER1	Protein RER1	95	2	0.82	2	2	13
G1TQJ4	Q01433	AMPD2	AMP deaminase	AMP deaminase 2	97	2	0.82	3	3	5
G1T4Q8	J3KNQ4	PARVA	Uncharacterized protein	Alpha-parvin	91	3	0.82	11	26	34
G1SH85	A0A2R8YF87	VPS33A	Uncharacterized protein	Vacuolar protein sorting-associated protein 33A	91	3	0.82	8	8	22
G1U304		GALNS	Galactosamine (N-acetyl)-6-sulfatase			1	0.82	2	5	7
G1SLD6	B3KWE1	HARS	Uncharacterized protein	Histidine--tRNA ligase, cytoplasmic	97	3	0.82	7	6	19
G1T3Z2	Q9UNZ2	NSFL1C	Uncharacterized protein	NSFL1 cofactor p47	97	3	0.82	5	7	19
G1T8K2	A0A0U1RR22	PACSIN2	Uncharacterized protein	Protein kinase C and casein kinase substrate in neurons protein 2 (Fragment)	93	3	0.82	8	7	23
G1T3V3	Q6R327	RICTOR	Uncharacterized protein	Rapamycin-insensitive companion of mTOR	98	3	0.82	4	6	4
	Q9Y6I3	EPN1		Epsin-1		4	0.82	2	3	4
G1U2B5		PDXK	Phos_pyr_kin domain-containing protein			1	0.82	3	3	19
	P10301	RRAS		Ras-related protein R-Ras		4	0.82	6	5	38
G1TCM9	Q9H299	SH3BGRL3	SH3 domain-binding glutamic acid-rich-like protein	SH3 domain-binding glutamic acid-rich-like protein 3	100	2	0.82	2	6	52
	F5H039	GPHN		Molybdopterin molybdenumtransferase		4	0.82	2	4	5
G1T4T7	Q9Y2A7	NCKAP1	Uncharacterized protein	Nck-associated protein 1	100	3	0.82	15	21	21
G1T416	Q9UP83	COG5	Uncharacterized protein	Conserved oligomeric Golgi complex subunit 5	91	3	0.82	5	5	11
G1TDJ3	Q9BSJ8	ESYT1	Uncharacterized protein	Extended synaptotagmin-1	89	3	0.82	30	45	41
G1SSV4	A0A087WT80	PLCB1	1-phosphatidylinositol 4,5-bisphosphate phosphodiesterase	1-phosphatidylinositol 4,5-bisphosphate phosphodiesterase	97	2	0.82	3	3	5
Q29502	Q13177	PAK2	Serine/threonine-protein kinase PAK 2	Serine/threonine-protein kinase PAK 2	98	2	0.82	8	6	25
G1TN13	A8CTZ0	ITSN1	Intersectin 1	Intersectin 1 short form variant 13	88	2	0.81	5	5	12
G1SME1		NOP14	NOP14 nucleolar protein			1	0.81	3	5	6
	H0YEN5	RPS2		40S ribosomal protein S2 (Fragment)		4	0.81	10	5	54
G1SKS9	A0A087WSW9	TXNRD1	Glutaredoxin domain-containing protein	Thioredoxin reductase 1, cytoplasmic	94	2	0.81	10	10	27
G1SFR8	P25398	RPS12	40S ribosomal protein S12	40S ribosomal protein S12	100	2	0.81	4	12	32
G1TNM3	P23396	RPS3	KH type-2 domain-containing protein	40S ribosomal protein S3	100	2	0.81	17	41	62
G1SUM3	Q8TBC4	UBA3	E2_bind domain-containing protein	NEDD8-activating enzyme E1 catalytic subunit	99	2	0.81	5	5	21
G1T6S6		ATP6V1F	V-type proton ATPase subunit F			1	0.81	4	8	54
G1SYL8	I3L0M9	ELOB	Elongin B	Elongin-B (Fragment)	82	2	0.81	4	4	43
G1SGY2	H0YC15	PTPN12	Tyrosine-protein phosphatase non-receptor type 12	Tyrosine-protein phosphatase non-receptor type 12 (Fragment)	90	2	0.81	2	3	21

G1TIA2		RIOX1	JmjC domain-containing protein			1	0.81	3	4	10
G1SPR5	P16278	GLB1	Glyco_hydro_35 domain-containing protein	Beta-galactosidase	82	2	0.81	15	24	33
G1T6D4	O00232	PSMD12	PCI domain-containing protein	26S proteasome non-ATPase regulatory subunit 12	99	2	0.81	16	41	48
G1T196	Q13033	STRN3	WD_REPEATS_REGION domain-containing protein	Striatin-3	92	2	0.81	4	4	12
Q28685	Q14118	DAG1	Dystroglycan	Dystroglycan	94	2	0.81	3	4	4
G1SKK0	P22102	GART	Trifunctional purine biosynthetic protein adenosine-3	Trifunctional purine biosynthetic protein adenosine-3	89	2	0.81	6	8	12
G1T373	B1ALA9	PRPS1	Pribosyltran_N domain-containing protein	Ribose-phosphate pyrophosphokinase 1	88	2	0.81	4	11	19
G1SWD8	P42356	PI4KA	Uncharacterized protein	Phosphatidylinositol 4-kinase alpha	98	3	0.81	10	5	8
G1SQ30		POMGNT2	Fibronectin type-III domain-containing protein			1	0.81	2	6	6
G1SZR4	H7BZ14	PPIL3	Peptidyl-prolyl cis-trans isomerase	Peptidyl-prolyl cis-trans isomerase (Fragment)	100	2	0.81	2	3	12
G1SRF5	Q9UPN7	PPP6R1	Protein phosphatase 6 regulatory subunit 1	Serine/threonine-protein phosphatase 6 regulatory subunit 1	84	2	0.81	4	3	9
G1T918	P28070	PSMB4	Proteasome subunit beta	Proteasome subunit beta type-4	95	2	0.81	8	37	49
G1SUU7	Q9Y3I0	RTCB	tRNA-splicing ligase RtcB homolog	tRNA-splicing ligase RtcB homolog	100	2	0.81	16	3	43
G1T5H8	E7EX17	EIF4B	RRM domain-containing protein	Eukaryotic translation initiation factor 4B	93	2	0.81	3	8	10
G1TET2	P13796	LCP1	Uncharacterized protein	Plastin-2	98	3	0.81	19	18	46
G1U484		LLGL1	LLGL scribble cell polarity complex component 1			1	0.81	2	2	3
G1SZM0	Q9BRT3	MIEN1	Uncharacterized protein	Migration and invasion enhancer 1	95	3	0.81	2	2	16
G1TER3	A0A2R8Y5M6	BCAP31	Uncharacterized protein	B-cell receptor-associated protein 31	81	3	0.81	11	20	53
G1SV03	Q14165	MLEC	Malectin domain-containing protein	Malectin	95	2	0.81	8	14	32
G1STU4	E9PIE3	CAVIN3	Uncharacterized protein	Caveolae-associated protein 3	78	3	0.80	4	5	15
G1T156	Q9HB40	SCPEP1	Carboxypeptidase	Retinoid-inducible serine carboxypeptidase	86	2	0.80	11	20	26
G1SQM7	P53999	SUB1	PC4 domain-containing protein	Activated RNA polymerase II transcriptional coactivator p15	97	2	0.80	4	7	22
U3KN73	Q9UBQ0	VPS29	Vacuolar protein sorting-associated protein 29	Vacuolar protein sorting-associated protein 29	100	2	0.80	6	9	37
G1SL85	A0A1B0GTM3	ASAH1	Uncharacterized protein	Acid ceramidase	81	3	0.80	7	9	27
G1SRA9	A0A0A0MTH3	ILK	Uncharacterized protein	Integrin-linked protein kinase	93	3	0.80	11	16	27
G1T1V0	P13645	KRT10	IF rod domain-containing protein	Keratin, type I cytoskeletal 10	95	2	0.80	22	53	51
	P35527	KRT9		Keratin, type I cytoskeletal 9		4	0.80	19	28	44
G1SRB6	Q08752	PPID	Peptidylprolyl isomerase D	Peptidyl-prolyl cis-trans isomerase D	93	2	0.80	9	25	27
G1T5R0		TUBGCP3	Gamma-tubulin complex component			1	0.80	2	2	5
G1SJS2	Q14232	EIF2B1	Uncharacterized protein	Translation initiation factor eIF-2B subunit alpha	96	3	0.80	4	13	31
G1SD48	A0A0G2JLB3	GBA	Glucosylceramidase	Glucosylceramidase	90	2	0.80	5	8	20
G1SP97	P51884	LUM	Lumican	Lumican	91	2	0.80	4	5	19
	Q15149	PLEC		Plectin		4	0.80	84	109	21
G1SE51	A0A087X2D8	SPAG9	Sperm associated antigen 9	C-Jun-amino-terminal kinase-interacting protein 4	93	2	0.80	10	16	13
G1T4P8	O76003	GLRX3	Glutaredoxin 3	Glutaredoxin-3	93	2	0.80	10	18	46
G1SLM1	Q9HB71	CACYBP	Uncharacterized protein	Calcyclin-binding protein	92	3	0.80	5	3	22
G1T8X7	Q96JH7	VCPIP1	OTU domain-containing protein	Deubiquitinating protein VCIP135	96	2	0.80	2	3	2
G1T364	Q8NDH3	NPEPL1	Aminopeptidase like 1	Probable aminopeptidase NPEPL1	90	2	0.80	3	5	9
G1TMP7	A0A0D9SG72	STXBP1	Syntaxin binding protein 1	Syntaxin-binding protein 1	99	2	0.80	4	5	10
G1TEU8		TBCC	C-CAP/cofactor C-like domain-containing protein			1	0.80	2	2	7
G1T332		GOT1	Aspartate aminotransferase			1	0.80	3	4	10
G1U354	R4GMR5	PSMD8	PCI domain-containing protein	26S proteasome non-ATPase regulatory subunit 8	97	2	0.80	9	14	33
G1SDU1	Q96A49	SYAP1	BSD domain-containing protein	Synapse-associated protein 1	89	2	0.80	2	5	14
G1SYK3	P45985	MAP2K4	Protein kinase domain-containing protein	Dual specificity mitogen-activated protein kinase kinase 4	99	2	0.80	3	3	10
P21195	P07237	P4HB	Protein disulfide-isomerase	Protein disulfide-isomerase	91	2	0.80	34	614	72
G1SDT0	Q562R1	ACTBL2	Uncharacterized protein	Beta-actin-like protein 2	97	3	0.80	14	15	41
G1TBC4	G3V126	ATP6V1H	V-type proton ATPase subunit H	V-type proton ATPase subunit H	99	2	0.80	6	6	23
G1T9Y4	E5RIU9	CHMP7	Uncharacterized protein	Charged multivesicular body protein 7 (Fragment)	69	3	0.80	2	3	9
G1SNN0		CSGALNACT2	Hexosyltransferase			1	0.80	2	2	7
G1SS73	Q92499	DDX1	Uncharacterized protein	ATP-dependent RNA helicase DDX1	98	3	0.79	20	38	37
G1SMM5	O60884	DNAJA2	Uncharacterized protein	DnaJ homolog subfamily A member 2	100	3	0.79	6	12	29
G1U6Y3		MEAK7	MTOR associated protein, eak-7 homolog			1	0.79	3	3	16
G1SXX9	A0A0C4DGH3	NUMBL	NUMB like, endocytic adaptor protein	Numb-like protein	93	2	0.79	2	2	4
	P17612	PRKACA		cAMP-dependent protein kinase catalytic subunit alpha		4	0.79	6	2	27
G1SR77	P23634	ATP2B4	Calcium-transporting ATPase	Plasma membrane calcium-transporting ATPase 4	87	2	0.79	9	8	10
G1SVV6	Q02750	MAP2K1	Dual-specificity mitogen-activated protein kinase kinase 1	Dual specificity mitogen-activated protein kinase kinase 1	94	2	0.79	5	11	21
G1TRX7	P23743	DGKA	Diacylglycerol kinase	Diacylglycerol kinase alpha	93	2	0.79	4	5	9
B7NZJ1		CPNE1	Copine I, isoform 8 (Predicted)			1	0.79	3	2	11
G1TQD3	Q01968	OCRL	Rho-GAP domain-containing protein	Inositol polyphosphate 5-phosphatase OCRL	95	2	0.79	3	3	5

G1TJX7	P50552	VASP	Vasodilator stimulated phosphoprotein	Vasodilator-stimulated phosphoprotein	87	2	0.79	2	5	6
	K7ER00	FARSA		Phenylalanine--tRNA ligase alpha subunit		4	0.79	3	2	6
G1SN17		PPP4R1	WRNPLPNID domain-containing protein			1	0.79	4	3	8
G1TNY9		TRADD	Death domain-containing protein			1	0.79	2	3	10
G1TEJ4	Q86VW0	SESTD1	SEC14 and spectrin domain containing 1	SEC14 domain and spectrin repeat-containing protein 1	95	2	0.79	2	4	8
G1TEU5	O75131	CPNE3	Uncharacterized protein	Copine-3	96	3	0.79	3	2	7
G1SIG2	P46108	CRK	Uncharacterized protein	Adapter molecule crk	99	3	0.79	7	3	37
G1SS79	P29966	MARCKS	Uncharacterized protein	Myristoylated alanine-rich C-kinase substrate	87	3	0.79	4	12	25
G1SH25	P40616	ARL1	Uncharacterized protein	ADP-ribosylation factor-like protein 1	99	3	0.79	5	15	38
G1TE96	O43813	LANCL1	Uncharacterized protein	Glutathione S-transferase LANCL1	95	3	0.79	4	8	18
G1T4X8	P49721	PSMB2	Proteasome subunit beta	Proteasome subunit beta type-2	99	2	0.79	8	39	60
G1SZI5	P62269	RPS18	Uncharacterized protein	40S ribosomal protein S18	100	3	0.79	9	19	44
G1SZU0		URB2	Urb2 domain-containing protein			1	0.79	2	2	2
G1SNI2	Q12841	FSTL1	Kazal-like domain-containing protein	Follistatin-related protein 1	94	2	0.78	8	21	32
G1TI53	A0A0A0MRM8	MYO6	Uncharacterized protein	Unconventional myosin-VI	94	3	0.78	7	10	8
G1SYA5	X6RA14	ESD	S-formylglutathione hydrolase	S-formylglutathione hydrolase	88	2	0.78	8	14	45
P19943	P05387	RPLP2	60S acidic ribosomal protein P2 (Fragment)	60S acidic ribosomal protein P2	100	2	0.78	8	21	64
G1T2G6	Q9P265	DIP2B	DMAP-interaction domain-containing protein	Disco-interacting protein 2 homolog B	99	2	0.78	2	2	2
G1SHF1	Q9UHV9	PFDN2	Uncharacterized protein	Prefoldin subunit 2	99	3	0.78	4	8	36
G1T670		SORT1	Proteasome subunit alpha type			1	0.78	10	19	48
G1SQG5	P40925	MDH1	Malate dehydrogenase	Malate dehydrogenase, cytoplasmic	97	2	0.78	12	11	57
G1TTU6	E7ERH2	SKP1	Uncharacterized protein	S-phase kinase-associated protein 1 (Fragment)	100	3	0.78	6	55	56
G1SPZ2	Q13107	USP4	Ubiquitin carboxyl-terminal hydrolase	Ubiquitin carboxyl-terminal hydrolase 4	89	2	0.78	3	5	7
G1SCP0	O00203	AP3B1	AP-3 complex subunit beta	AP-3 complex subunit beta-1	88	2	0.78	13	17	16
G1U446	Q14677	CLINT1	ENTH domain-containing protein	Clathrin interactor 1	97	2	0.78	9	14	17
G1T1D9	Q92905	COPS5	MPN domain-containing protein	COP9 signalosome complex subunit 5	100	2	0.78	7	10	31
G1TUH9	Q14195	DPYSL3	Amidohydro-rel domain-containing protein	Dihydropyrimidinase-related protein 3	98	2	0.78	17	22	38
G1SM91		FAH	Fumarylacetoacetase			1	0.78	2	2	7
G1TAF8	P31948	STIP1	Uncharacterized protein	Stress-induced-phosphoprotein 1	96	3	0.78	26	44	52
G1SSP0		STRN	WD_REPEATS_REGION domain-containing protein			1	0.78	3	4	11
U3KP45		SYNE1	Spectrin repeat containing nuclear envelope protein 1			1	0.78	4	5	1
G1SWE5		WDR61	WD repeat domain 61			1	0.78	2	2	12
G1T1R9	Q96A65	EXOC4	Sec8_exocyst domain-containing protein	Exocyst complex component 4	95	2	0.78	11	13	19
G1SH26	F6TLX2	GLOD4	Glyoxalase domain containing 4	Glyoxalase domain-containing protein 4	81	2	0.78	7	11	23
G1TA40	H0YEP5	SMPD1	Sphingomyelin phosphodiesterase	Sphingomyelin phosphodiesterase (Fragment)	78	2	0.78	5	7	14
G1SK52	Q5T6H7	XPNPEP1	Uncharacterized protein	Xaa-Pro aminopeptidase 1	97	3	0.78	10	10	25
	Q8WUW1	BRK1		Protein BRICK1		4	0.78	2	3	23
Q09YN6	C9JKI3	CAV1	Caveolin-1	Caveolin (Fragment)	96	2	0.78	3	4	21
G1T2L1	A0A024RA52	PSMA2	Proteasome subunit alpha type	Proteasome subunit alpha type	100	2	0.78	11	34	59
G1TLL6	Q9UNH6	SNX7	PX domain-containing protein	Sorting nexin-7	96	2	0.78	4	3	15
G1T6S9	P53367	ARFIP1	AH domain-containing protein	Arfaptin-1	98	2	0.78	4	6	15
G1SNL7	Q9H1I8	ASCC2	Activating signal cointegrator 1 complex subunit 2	Activating signal cointegrator 1 complex subunit 2	86	2	0.78	3	4	6
G1SS77	Q15477	SKIV2L	Uncharacterized protein	Helicase SKI2W	95	3	0.78	7	9	7
G1TSL5	C9J6N9	UFD1	Uncharacterized protein	Ubiquitin recognition factor in ER-associated degradation protein 1 (Fragment)	100	3	0.78	3	3	13
G1U1M3	Q12904	AIMP1	tRNA-binding domain-containing protein	Aminoacyl tRNA synthase complex-interacting multifunctional protein 1	91	2	0.78	8	15	36
G1SJ23	P07384	CAPN1	Calpain-1 catalytic subunit	Calpain-1 catalytic subunit	90	2	0.78	10	11	25
P63150	P63151	PPP2R2A	Serine/threonine-protein phosphatase 2A 55 kDa regulatory subunit B alpha isoform	Serine/threonine-protein phosphatase 2A 55 kDa regulatory subunit B alpha isoform	100	2	0.78	4	7	15
G1SN22	Q9Y5K6	CD2AP	Uncharacterized protein	CD2-associated protein	91	3	0.78	2	2	5
G1SZW0	Q7L576	CYFIP1	Cytoplasmic FMR1-interacting protein	Cytoplasmic FMR1-interacting protein 1	98	2	0.78	18	28	17
G1SCE1	Q9H4G4	GLIPR2	SCP domain-containing protein	Golgi-associated plant pathogenesis-related protein 1	97	2	0.78	4	5	41
G1SS33	P36543	ATP6V1E1	Uncharacterized protein	V-type proton ATPase subunit E 1	99	3	0.77	11	12	58
	P09497-2	CLTB		Isoform Non-brain of Clathrin light chain B		4	0.77	5	10	21
	A0A096LP07	GPS1		COP9 signalosome complex subunit 1		4	0.77	4	3	10
G1T0G0		P3H2	Fe2OG dioxygenase domain-containing protein			1	0.77	2	2	4
G1T7F5	A0A1C7CYY0	ADD2	Aldolase_II domain-containing protein	Beta-adducin (Fragment)	93	2	0.77	2	2	8
	M0R261	PGLS		6-phosphogluconolactonase (Fragment)		4	0.77	2	4	13
G1SWD9	B4DKY1	CARS	Cysteinyl-tRNA synthetase	Cysteine--tRNA ligase, cytoplasmic	68	2	0.77	12	22	20
G1SMG5	P15170	GSPT1	Tr-type G domain-containing protein	Eukaryotic peptide chain release factor GTP-binding subunit ERF3A	99	2	0.77	11	16	25
G1SWC9	H3BMU1	IST1	Uncharacterized protein	IST1 homolog (Fragment)	100	3	0.77	5	7	21

G1T281		SNTA1	Alpha-1-syntrophin			1	0.77	2	6	8
G1TBW9	Q96FJ2	DYNLL2	Dynein light chain	Dynein light chain 2, cytoplasmic	100	2	0.77	2	8	33
G1SWR0	H3BP20	HEXA	Beta-hexosaminidase	Beta-hexosaminidase	85	2	0.77	12	15	36
G1SFH8		PDCL3	Phosducin domain-containing protein			1	0.77	2	3	11
G1T060	A0A2R8YD50	HSD17B4	Uncharacterized protein	Peroxisomal multifunctional enzyme type 2	89	3	0.77	9	13	21
G1SH86		TBCD	Tubulin folding cofactor D			1	0.77	13	16	17
G1SK00		USP5	Ubiquitin carboxyl-terminal hydrolase			1	0.77	21	37	38
G1SI22		AKAP12	A-kinase anchoring protein 12			1	0.76	11	11	17
G1TA48	Q9H223	EHD4	Uncharacterized protein	EH domain-containing protein 4	97	3	0.76	14	16	37
G1SZE0	Q96QK1	VPS35	Vacuolar protein sorting-associated protein 35	Vacuolar protein sorting-associated protein 35	100	2	0.76	22	46	37
Q09YN4	P47755	CAPZA2	F-actin-capping protein subunit alpha-2	F-actin-capping protein subunit alpha-2	98	2	0.76	8	10	50
	O95747	OXSR1		Serine/threonine-protein kinase OSR1		4	0.76	4	5	12
G1TBH6	Q9P0S9	TMEM14C	Uncharacterized protein	Transmembrane protein 14C	92	3	0.76	3	3	66
G1U826	O75348	ATP6V1G1	V-type proton ATPase subunit G	V-type proton ATPase subunit G 1	97	2	0.76	3	7	39
G1TM86	A2AB27	GNL1	G protein nucleolar 1 (putative)	Guanine nucleotide-binding protein-like 1 (Fragment)	98	2	0.76	2	3	5
G1SHU8	D3YTB1	RPL32	Uncharacterized protein	60S ribosomal protein L32 (Fragment)	88	3	0.76	4	10	29
G1SVA1	F2Z2X4	XPO4	CRM1_C domain-containing protein	Exportin-4	99	2	0.76	5	5	10
	I3L0A0	TMEM189-UBE2V1		HCG2044781		4	0.76	6	2	23
G1U9I8	P04264	KRT1	IF rod domain-containing protein	Keratin, type II cytoskeletal 1	86	2	0.76	20	35	39
G1SZK4	Q13442	PDAP1	PDGFA associated protein 1	28 kDa heat- and acid-stable phosphoprotein	96	2	0.76	2	4	16
G1SZ14	P25788	PSMA3	Proteasome endopeptidase complex	Proteasome subunit alpha type-3	99	2	0.76	8	19	26
G1TS73	H0YBP1	PTK2	Protein tyrosine kinase 2	Focal adhesion kinase 1 (Fragment)	89	2	0.76	4	5	6
G1SP51	P62277	RPS13	Ribosomal_S13_N domain-containing protein	40S ribosomal protein S13	100	2	0.76	8	17	46
	A0A494C0A9	CBFB		Core-binding factor subunit beta		4	0.76	4	5	31
G1TNL6	P50479	PDLIM4	Uncharacterized protein	PDZ and LIM domain protein 4	86	3	0.76	7	10	33
	O14818	PSMA7		Proteasome subunit alpha type-7		4	0.76	9	18	44
G1SYX4	P50281	MMP14	Matrix metalloproteinase-14	Matrix metalloproteinase-14	95	2	0.76	5	7	11
G1TGF1	Q15185	PTGES3	Prostaglandin E synthase 3	Prostaglandin E synthase 3	100	2	0.76	5	8	52
G1SKD5	Q92783	STAM	Uncharacterized protein	Signal transducing adapter molecule 1	96	3	0.76	5	10	14
G1SEN5	Q8IZ07	ANKRD13A	Ankyrin repeat domain 13A	Ankyrin repeat domain-containing protein 13A	94	2	0.75	2	2	5
	J3KR44	OTUB1		Ubiquitin thioesterase		4	0.75	5	8	34
	P60468	SEC61B		Protein transport protein Sec61 subunit beta		4	0.75	3	5	52
G1SN11	Q01082	SPTBN1	Spectrin beta chain	Spectrin beta chain, non-erythrocytic 1	99	2	0.75	75	13	44
G1SWN4	Q9H832	UBE2Z	UBIQUITIN_CONJUGAT_2 domain-containing protein	Ubiquitin-conjugating enzyme E2 Z	96	2	0.75	3	3	11
G1SXT1	O43865	AHCYL1	AdoHcyase_NAD domain-containing protein	S-adenosylhomocysteine hydrolase-like protein 1	100	2	0.75	3	6	7
G1SMK8	O95816	BAG2	BAG domain-containing protein	BAG family molecular chaperone regulator 2	98	2	0.75	6	9	29
G1T8J0	P02461	COL3A1	Uncharacterized protein	Collagen alpha-1(III) chain	92	3	0.75	17	31	15
G1T706	O00442	RTCA	Uncharacterized protein	RNA 3~-terminal phosphate cyclase	96	3	0.75	5	6	19
G1T3P1	K7EKP8	ACOT7	Uncharacterized protein	Cytosolic acyl coenzyme A thioester hydrolase (Fragment)	98	3	0.75	5	5	23
G1T4Z0	Q96PY5	FMNL2	Formin like 2	Formin-like protein 2	97	2	0.75	7	4	8
G1SCF4	G8JLD5	DNM1L	Uncharacterized protein	Dynamin-1-like protein	93	3	0.75	13	25	26
G1TDU0	P33176	KIF5B	Kinesin-like protein	Kinesin-1 heavy chain	99	2	0.75	28	7	41
G1SX42	Q9Y3P9	RABGAP1	Uncharacterized protein	Rab GTPase-activating protein 1	97	3	0.75	2	2	3
G1TA82	Q12768	WASHC5	Uncharacterized protein	WASH complex subunit 5	97	3	0.75	6	10	9
G1SDU6	P26639	TARS	AA_TRNA_LIGASE_II domain-containing protein	Threonine--tRNA ligase, cytoplasmic	96	2	0.75	17	25	28
G1SE87		ARSE	Arylsulfatase E			1	0.75	3	3	7
G1SE61	O75369	FLNB	Filamin-B	Filamin-B	97	2	0.75	106	510	61
G1SSL8		HEATR5A	HEAT repeat containing 5A			1	0.75	4	3	3
G1SI71		PAIP1	MIF4G domain-containing protein			1	0.75	2	3	6
G1SDG2	P48739	PITPNB	Phosphatidylinositol transfer protein beta	Phosphatidylinositol transfer protein beta isoform	97	2	0.75	4	4	14
G1SVH8	Q13636	RAB31	Uncharacterized protein	Ras-related protein Rab-31	95	3	0.75	6	7	36
G1TBG2	Q96KP1	EXOC2	Exocyst complex component 2	Exocyst complex component 2	94	2	0.74	4	5	6
G1SCT1	A0A499FJL1	PREP	Uncharacterized protein	Prolyl endopeptidase	96	3	0.74	19	34	41
G1SPL3	G3V583	FAM177A1	Uncharacterized protein	Protein FAM177A1 (Fragment)	93	3	0.74	2	2	12
	A0A0A0MT60	FKBP15		Peptidylprolyl isomerase (Fragment)		4	0.74	3	3	3
G1T9D6	C9J5C3	PDCD10	Uncharacterized protein	Programmed cell death protein 10 (Fragment)	99	3	0.74	7	7	48
G1SQN6	J3KN59	BNIP2	CRAL-TRIO domain-containing protein	BCL2/adenovirus E1B 19 kDa protein-interacting protein 2	94	2	0.74	2	3	11
G1ST51		CLIC2	Chloride intracellular channel protein			1	0.74	4	3	23
G1TA78	P23381	WARS	Tryptophan--tRNA ligase, cytoplasmic	Tryptophan--tRNA ligase, cytoplasmic	89	2	0.74	11	23	31

G1TAD1	Q7RTS9	DYM	Uncharacterized protein	Dymeclin	97	3	0.74	4	7	13
G1T6Q9	O15400	STX7	t-SNARE coiled-coil homology domain-containing protein	Syntaxin-7	95	2	0.74	7	16	48
G1TZP0	P61981	YWHAG	14_3_3 domain-containing protein	14-3-3 protein gamma	99	2	0.74	13	50	70
G1TEH2	P21283	ATP6V1C1	V-type proton ATPase subunit C	V-type proton ATPase subunit C 1	99	2	0.74	7	10	20
G1SV22	P30086	PEBP1	Phosphatidylethanolamine-binding protein 1	Phosphatidylethanolamine-binding protein 1	89	2	0.74	5	7	37
G1TDQ5	A0A2R8Y891	PFKM	ATP-dependent 6-phosphofructokinase	ATP-dependent 6-phosphofructokinase	97	2	0.74	5	7	10
G1SQJ2		FOCAD	DUF3730 domain-containing protein			1	0.73	8	8	7
G1SXY5		STX2	Syntaxin 2			1	0.73	3	3	21
G1T6S4	F8W9J4	DST	Dystonin	Dystonin	62	2	0.73	13	6	4
G1TB49		APOA1BP	NAD(P)H-hydrate epimerase			1	0.73	2	4	15
G1TPH5	Q9Y6D5	ARFGEF2	ADP ribosylation factor guanine nucleotide exchange factor 2	Brefeldin A-inhibited guanine nucleotide-exchange protein 2	96	2	0.73	9	10	8
G1T5P0	Q8NDI1	EHBP1	Uncharacterized protein	EH domain-binding protein 1	94	3	0.73	2	3	3
G1SPN9	P36405	ARL3	Uncharacterized protein	ADP-ribosylation factor-like protein 3	99	3	0.73	2	3	9
	A0A2U3U034	ARSB		Arylsulfatase B		4	0.73	4	4	12
	A8MZF9	DRG2		Developmentally-regulated GTP-binding protein 2		4	0.73	3	6	14
G1TPW2		MRI1	Methylthioribose-1-phosphate isomerase			1	0.73	4	6	22
G1TYU5		QTRT1	Queuine tRNA-ribosyltransferase			1	0.73	2	3	12
G1SNM5	A0A2R8Y566	RELCH	LisH domain-containing protein	RAB11-binding protein RELCH	96	2	0.73	3	3	5
G1TMM0	A0A087WW43	ITIH3	Inter-alpha-trypsin inhibitor heavy chain H3	Inter-alpha-trypsin inhibitor heavy chain H3	91	2	0.73	4	11	5
G1TD16	P48426	PIP4K2A	PIPK domain-containing protein	Phosphatidylinositol 5-phosphate 4-kinase type-2 alpha	100	2	0.73	2	2	5
G1SST9	P54727	RAD23B	Uncharacterized protein	UV excision repair protein RAD23 homolog B	94	3	0.73	12	15	42
P40826	P54578	USP14	Ubiquitin carboxyl-terminal hydrolase 14	Ubiquitin carboxyl-terminal hydrolase 14	97	2	0.73	10	14	29
G1SI94		CRLF3	Cytokine receptor like factor 3			1	0.73	2	2	14
G1T3V0	A0A024R4E5	HDLBP	Uncharacterized protein	High density lipoprotein binding protein (Vigilin), isoform CRA_a	97	3	0.73	45	106	47
G1T7H6	Q6UX71	PLXDC2	PSI domain-containing protein	Plexin domain-containing protein 2	94	2	0.73	4	9	9
G1T7B1	Q9H3P7	ACBD3	Uncharacterized protein	Golgi resident protein GCP60	96	3	0.72	7	6	21
G1SP54		LTA4H	Leukotriene A(4) hydrolase			1	0.72	4	3	10
G1T519	P25789	PSMA4	Proteasome subunit alpha type	Proteasome subunit alpha type-4	100	2	0.72	10	20	58
G1SMS3	P61160	ACTR2	Actin-related protein 2	Actin-related protein 2	100	2	0.72	13	108	48
B7NZD2	Q5VWC4	PSMD4	Proteasome 26S subunit, non-ATPase, 4 (Predicted)	26S proteasome non-ATPase regulatory subunit 4	99	2	0.72	8	15	27
G1TGA8	O75396	SEC22B	Uncharacterized protein	Vesicle-trafficking protein SEC22b	98	3	0.72	10	29	48
G1SZT8	P55735	SEC13	WD_REPEATS_REGION domain-containing protein	Protein SEC13 homolog	95	2	0.72	8	16	38
G1TQR0	P12814-3	ACTN1	Uncharacterized protein	Isoform 3 of Alpha-actinin-1	97	3	0.72	59	117	79
G1T188	Q9UIJ7	AK3	GTP:AMP phosphotransferase AK3, mitochondrial	GTP:AMP phosphotransferase AK3, mitochondrial	93	2	0.72	11	20	57
G1SV81	Q9BTE1	DCTN5	Uncharacterized protein	Dynactin subunit 5	100	3	0.72	2	2	9
	Q8TBX8	PIP4K2C		Phosphatidylinositol 5-phosphate 4-kinase type-2 gamma		4	0.72	4	3	18
G1STY4		PLBD2	Phospholipase B-like			1	0.72	5	13	16
G1T089		THUMPD3	THUMP domain containing 3			1	0.72	3	2	9
G1T0Z6	Q9Y4X5	ARIH1	RBR-type E3 ubiquitin transferase	E3 ubiquitin-protein ligase ARIH1	98	2	0.72	3	2	10
G1TC10	P61081	UBE2M	UBIQUITIN_CONJUGAT_2 domain-containing protein	NEDD8-conjugating enzyme Ubc12	100	2	0.72	7	7	51
P0CL18		EIF2D	Eukaryotic translation initiation factor 2D			1	0.72	2	2	8
	P68402	PAFAH1B2		Platelet-activating factor acetylhydrolase IB subunit beta		4	0.72	4	10	37
G1TS93	Q15435	PPP1R7	LRRcap domain-containing protein	Protein phosphatase 1 regulatory subunit 7	95	2	0.72	10	9	46
	O43707	ACTN4		Alpha-actinin-4		4	0.71	52	73	64
G1SN14	Q86VP6	CAND1	TIP120 domain-containing protein	Cullin-associated NEDD8-dissociated protein 1	100	2	0.71	32	47	36
G1SPZ7		GPX1	Glutathione peroxidase			1	0.71	5	5	51
G1T085		SVIL	HP domain-containing protein			1	0.71	4	6	4
G1SK48	K7ES02	BLMH	Bleomycin hydrolase	Bleomycin hydrolase (Fragment)	92	2	0.71	8	10	26
U3KNB6	P48444	ARCN1	Coatomer subunit delta	Coatomer subunit delta	97	2	0.71	18	34	40
G1SCR0	H0YFI1	LAMTOR1	Uncharacterized protein	Ragulator complex protein LAMTOR1 (Fragment)	100	3	0.71	2	3	47
G1U2R2	E9PLK3	NPEPPS	Aminopeptidase	Aminopeptidase	98	2	0.71	16	25	27
G1SVH0	P23526	AHCY	AdoHcyase_NAD domain-containing protein	Adenosylhomocysteinase	97	2	0.71	15	41	36
G1T8P4	P05386	RPLP1	Uncharacterized protein	60S acidic ribosomal protein P1	97	3	0.71	4	8	72
G1SV13	P22314	UBA1	Ubiquitin-like modifier-activating enzyme 1	Ubiquitin-like modifier-activating enzyme 1	97	2	0.71	30	69	48
G1SP48	O60701	UGDH	UDP-glucose 6-dehydrogenase	UDP-glucose 6-dehydrogenase	96	2	0.71	17	4	51
G1SJ77	Q9Y696	CLIC4	Chloride intracellular channel protein	Chloride intracellular channel protein 4	100	2	0.71	11	37	58
	Q6WCQ1	MPRIP		Myosin phosphatase Rho-interacting protein		4	0.71	16	2	20
G1TB17	A0A1B0GWF8	ADSL	Adenylosuccinate lyase	Adenylosuccinate lyase (Fragment)	95	2	0.71	4	2	13
G1TB45	Q86W92	PPFIBP1	Uncharacterized protein	Liprin-beta-1	87	3	0.71	22	39	33

G1SUT8	O95486	SEC24A	Uncharacterized protein	Protein transport protein Sec24A	93	3	0.71	5	7	7
G1T7T6	O60763	USO1	Uncharacterized protein	General vesicular transport factor p115	95	3	0.71	22	38	35
G1T4N4	A0A0B4J210	LARP1	HTH La-type RNA-binding domain-containing protein	La-related protein 1 (Fragment)	93	2	0.71	5	8	8
G1SET0	P35606	COPB2	Coatomer subunit beta~	Coatomer subunit beta~	98	2	0.71	24	55	38
G1SEM0	Q96AC1	FERMT2	PH domain-containing protein	Fermitin family homolog 2	100	2	0.71	22	38	47
G1THW3	A0A3B3IUC0	ITM2B	BRICHOS domain-containing protein	Integral membrane protein 2B	96	2	0.71	5	8	31
G1TJC8		PEX5	TPR_REGION domain-containing protein			1	0.71	2	2	5
	P40429	RPL13A		60S ribosomal protein L13a		4	0.70	9	13	37
G1TP25	Q5VU77	UBAP2L	Ubiquitin associated protein 2 like	Ubiquitin-associated protein 2-like (Fragment)	95	2	0.70	3	4	4
G1SID3	Q709C8	VPS13C	Vacuolar protein sorting 13 homolog C	Vacuolar protein sorting-associated protein 13C	88	2	0.70	10	8	4
G1SVY8	P12277	CKB	Creatine kinase B-type	Creatine kinase B-type	97	2	0.70	19	212	70
G1SHZ2	Q86X83	COMMD2	COMM domain-containing protein	COMM domain-containing protein 2	97	2	0.70	2	4	17
Q28717	F6WIT2	PTPA	Serine/threonine-protein phosphatase 2A activator	Serine/threonine-protein phosphatase 2A activator	97	2	0.70	8	12	41
G1SNY5	H0YK42	SNX1	PX domain-containing protein	Sorting nexin-1	96	2	0.70	9	13	20
P51662	P04083	ANXA1	Annexin A1	Annexin A1	91	2	0.70	20	20	61
G1U2Q7		COL8A1	Collagen alpha-1(VIII) chain			1	0.70	3	4	5
G1TAP1	Q15181	PPA1	Uncharacterized protein	Inorganic pyrophosphatase	96	3	0.70	8	10	48
G1SJJ2	Q9NZ32	ACTR10	Uncharacterized protein	Actin-related protein 10	96	3	0.70	10	23	40
G1SMP6	A0A1B0GVV3	RILPL1	Rab interacting lysosomal protein like 1	RILP-like protein 1	93	2	0.70	4	6	16
G1TC70	O14974	PPP1R12A	Protein phosphatase 1 regulatory subunit	Protein phosphatase 1 regulatory subunit 12A	94	2	0.70	13	29	16
	J3KN75	TBC1D8B		TBC1 domain family member 8B		4	0.70	2	2	3
G1TEM7	D6RA82	ANXA3	Annexin	Annexin	93	2	0.70	13	20	51
	Q6NZI2	CAVIN1		Caveolae-associated protein 1		4	0.70	7	12	19
G1SSZ1	Q01658	DR1	CBFD_NFYB_HMF domain-containing protein	Protein Dr1	100	2	0.70	2	3	21
G1TA50	E7EQ61	UBA5	ThiF domain-containing protein	Ubiquitin-like modifier-activating enzyme 5	93	2	0.70	6	8	27
Q28740		BSG	Basigin			1	0.69	2	4	10
G1SLZ8	Q06124	PTPN11	Tyrosine-protein phosphatase non-receptor type	Tyrosine-protein phosphatase non-receptor type 11	98	2	0.69	6	4	15
G1T057	D6RCE2	TTC37	Uncharacterized protein	Tetratricopeptide repeat protein 37 (Fragment)	83	3	0.69	7	12	6
G1TBW1		TXNDC17	DUF953 domain-containing protein			1	0.69	3	8	31
G1U9R6	P02751	FN1	Fibronectin	Fibronectin	70	2	0.69	69	148	46
G1SZP0	O60784	TOM1	Uncharacterized protein	Target of Myb protein 1	88	3	0.69	11	24	44
G1SDA4	P53618	COPB1	Coatomer subunit beta	Coatomer subunit beta	99	2	0.69	37	187	54
G1SQ23	P42025	ACTR1B	Uncharacterized protein	Beta-centractin	99	3	0.69	7	3	25
G1T2R3		EFL1	Tr-type G domain-containing protein			1	0.69	3	2	6
G1T5Z7	B3KR49	MAPK3	Mitogen-activated protein kinase	Mitogen-activated protein kinase 3	97	2	0.69	6	2	37
G1SYV9	Q9Y490	TLN1	Uncharacterized protein	Talin-1	99	3	0.69	108	34	61
G1SFZ8	Q9UMX0	UBQLN1	Ubiquilin 1	Ubiquilin-1	86	2	0.69	8	9	26
G1TEW4	Q93008	USP9X	USP domain-containing protein	Probable ubiquitin carboxyl-terminal hydrolase FAF-X	99	2	0.69	35	54	21
G1SVI9	Q9P0L0	VAPA	MSP domain-containing protein	Vesicle-associated membrane protein-associated protein A	84	2	0.69	9	18	40
G1T0Z8	Q99471	PFDN5	Uncharacterized protein	Prefoldin subunit 5	99	3	0.69	4	5	44
G1SKT2	E9PGF6	PHLDB2	PH domain-containing protein	Pleckstrin homology-like domain family B member 2	65	2	0.69	8	11	8
G1SQL0	P62191	PSMC1	AAA domain-containing protein	26S proteasome regulatory subunit 4	100	2	0.69	17	29	43
G1TME7		CSTB	Cystatin B			1	0.69	3	4	36
G1SGK1		GIGYF2	GRB10 interacting GYF protein 2			1	0.69	3	2	5
G1TI71	A0A087WYS1	UGP2	UTP--glucose-1-phosphate uridylyltransferase	UTP--glucose-1-phosphate uridylyltransferase	99	2	0.69	24	57	62
G1TVW5	Q96IJ6	GMPPA	NTP_transferase domain-containing protein	Mannose-1-phosphate guanyltransferase alpha	96	2	0.69	7	8	30
G1SGX3	P22059	OSBP	Oxysterol-binding protein	Oxysterol-binding protein 1	97	2	0.69	8	8	12
G1SRQ2	A0A087WTB8	UCHL3	Ubiquitin carboxyl-terminal hydrolase	Ubiquitin carboxyl-terminal hydrolase	98	2	0.69	3	2	21
G1T346	Q13813-3	SPTAN1	Uncharacterized protein	Isoform 3 of Spectrin alpha chain, non-erythrocytic 1	97	3	0.69	108	30	55
G1TUD6	P43686	PSMC4	Proteasome 26S subunit, ATPase 4	26S proteasome regulatory subunit 6B	100	2	0.68	12	49	34
G1T6T5	E9PPQ5	CHORDC1	Uncharacterized protein	Cysteine and histidine-rich domain-containing protein 1	94	3	0.68	2	2	9
	P63241	EIF5A		Eukaryotic translation initiation factor 5A-1		4	0.68	9	209	66
G1T078	O43294	TGFB1I1	Uncharacterized protein	Transforming growth factor beta-1-induced transcript 1 protein	95	3	0.68	4	5	11
G1SVZ8		C9orf64	Queuosine salvage protein			1	0.68	2	2	7
G1SHI2		MYD88	Myeloid differentiation primary response protein MyD88			1	0.68	2	2	9
G1SL68	P35579	MYH9	Uncharacterized protein	Myosin-9	96	3	0.68	119	272	57
G1SNE1	Q6DKJ4	NXN	Thioredoxin domain-containing protein	Nucleoredoxin	98	2	0.68	8	24	30
G1SLQ4	B4DXW1	ACTR3	Uncharacterized protein	Actin-related protein 3	100	3	0.68	17	43	71
G1TS42		AGL	Glycogen debranching enzyme			1	0.68	3	2	4

G1SPV8	O94829	IPO13	Importin N-terminal domain-containing protein	Importin-13	100	2	0.68	2	2	3
P15541		ANPEP	Aminopeptidase N			1	0.68	11	14	19
G1TK63	P22413	ENPP1	Uncharacterized protein	Ectonucleotide pyrophosphatase/phosphodiesterase family member 1	89	3	0.68	18	26	30
G1TER0	Q9NV70	EXOC1	Sec3-PIP2_bind domain-containing protein	Exocyst complex component 1	98	2	0.68	6	9	14
G1TPN2	K7EQA9	CDC37	Cell division cycle 37	Hsp90 co-chaperone Cdc37 (Fragment)	57	2	0.68	5	2	17
G1T090	G3V180	DPP3	Dipeptidyl peptidase 3	Dipeptidyl peptidase 3	94	2	0.68	9	16	24
U3KMD1	A0A0A0MRI2	SNX6	Vps5 domain-containing protein	Sorting nexin	99	2	0.68	11	113	28
G1SRI2	Q6P996	PDXDC1	Uncharacterized protein	Pyridoxal-dependent decarboxylase domain-containing protein 1	89	3	0.68	17	29	35
G1SMU8	Q9NQP4	PFDN4	Prefoldin subunit 4	Prefoldin subunit 4	99	2	0.68	2	3	20
G1SNP8	C9JJP5	TFG	PB1 domain-containing protein	Protein TFG (Fragment)	97	2	0.68	7	16	23
G1SMK4		UCKL1	Uridine-cytidine kinase 1 like 1			1	0.68	2	3	6
G1T652	P09972	ALDOC	Fructose-bisphosphate aldolase	Fructose-bisphosphate aldolase C	99	2	0.68	9	8	30
G1TED6	P08758	ANXA5	Annexin	Annexin A5	93	2	0.68	19	47	65
G1T1G5	Q9H4A6	GOLPH3	Uncharacterized protein	Golgi phosphoprotein 3	99	3	0.68	5	4	32
G1T198	Q8TDX7	NEK7	Protein kinase domain-containing protein	Serine/threonine-protein kinase Nek7	98	2	0.68	3	4	15
	O14617	AP3D1		AP-3 complex subunit delta-1		4	0.67	8	11	9
G1SFP0	P28838	LAP3	CYTOSOL_AP domain-containing protein	Cytosol aminopeptidase	92	2	0.67	17	29	52
G1SNH7	P09417	QDPR	Quinoid dihydropteridine reductase	Dihydropteridine reductase	92	2	0.67	4	5	23
G1SH88	Q8N8S7	ENAH	ENAH, actin regulator	Protein enabled homolog	99	2	0.67	10	2	28
G1T2N2		LTN1	RING-type domain-containing protein			1	0.67	4	3	4
G1SI13		SDC2	Syndecan			1	0.67	3	4	11
G1U9C1	P50570	DNM2	Dynamin 2	Dynamin-2	96	2	0.67	24	7	36
U3KMU7	A0A024R571	EHD1	EH domain containing 1	EH domain-containing protein 1	99	2	0.67	15	15	35
G1U5B3	C9JIZ6	PSAP	Prosaposin	Prosaposin	78	2	0.67	13	47	40
G1SDP2	Q96AY3	FKBP10	FK506-binding protein	Peptidyl-prolyl cis-trans isomerase FKBP10	93	2	0.67	17	23	40
G1SCP8	P26038	MSN	FERM domain-containing protein	Moesin	94	2	0.67	33	22	59
G1SR28	Q15102	PAFAH1B3	SGNH_hydro domain-containing protein	Platelet-activating factor acetylhydrolase IB subunit gamma	97	2	0.67	5	6	31
	V9GYD0	ARL2-SNX15		ARL2-SNX15 readthrough (NMD candidate)		4	0.67	3	5	32
G1TGK3	B4DP31	PRPSAP1	Pribosyltran_N domain-containing protein	Phosphoribosyl pyrophosphate synthase-associated protein 1	99	2	0.67	3	3	13
G1TWQ3		SIRT2	NAD-dependent protein deacetylase			1	0.67	5	5	19
G1T9X4	O95980	RECK	Reversion inducing cysteine rich protein with kazal motifs	Reversion-inducing cysteine-rich protein with Kazal motifs	95	2	0.67	3	4	3
G1SYL5	Q7Z4N8	P4HA3	Fe2OG dioxygenase domain-containing protein	Prolyl 4-hydroxylase subunit alpha-3	92	2	0.67	15	38	43
G1TIW9		FBXO6	F-box protein 6			1	0.67	3	3	11
G1T0J3	E9PDF6	MYO1B	Uncharacterized protein	Unconventional myosin-Ib	96	3	0.67	17	27	22
G1T8H3	P51570	GALK1	Uncharacterized protein	Galactokinase	92	3	0.66	10	11	42
G1SVE3		PRUNE2	CRAL-TRIO domain-containing protein			1	0.66	4	4	2
G1SGJ4		SRR	PALP domain-containing protein			1	0.66	2	3	5
G1SLF1		ADH5	S-(hydroxymethyl)glutathione dehydrogenase			1	0.66	4	3	12
P80912		HINT1	Histidine triad nucleotide-binding protein 1			1	0.66	2	2	28
G1TK30		CSAD	Cysteine sulfinic acid decarboxylase			1	0.66	4	9	18
G1T7D9		FNTB	Protein farnesyltransferase subunit beta			1	0.66	3	2	6
G1SW57	P21589	NT5E	Uncharacterized protein	5~-nucleotidase	89	3	0.66	10	11	21
G1TL06	P39023	RPL3	Uncharacterized protein	60S ribosomal protein L3	98	3	0.66	17	187	44
G1SHX1	P68036	UBE2L3	Ubiquitin conjugating enzyme E2 L3	Ubiquitin-conjugating enzyme E2 L3	96	2	0.66	6	11	57
G1SDZ0		CTSA	Carboxypeptidase			1	0.66	5	6	11
G1SZ63	P49189	ALDH9A1	Aldedh domain-containing protein	4-trimethylaminobutyraldehyde dehydrogenase	94	2	0.66	8	10	29
G1T7I4	P53621	COPA	Coatomer subunit alpha	Coatomer subunit alpha	99	2	0.66	48	96	52
G1TN89	P98160	HSPG2	Heparan sulfate proteoglycan 2	Basement membrane-specific heparan sulfate proteoglycan core protein	90	2	0.66	30	50	10
G1SX70	Q9NRW1	RAB6B	Uncharacterized protein	Ras-related protein Rab-6B	100	3	0.66	7	2	45
G1SMF4	Q9UHP3	USP25	USP domain-containing protein	Ubiquitin carboxyl-terminal hydrolase 25	94	2	0.66	4	4	6
G1SE41	Q9NWM8	FKBP14	Peptidylprolyl isomerase	Peptidyl-prolyl cis-trans isomerase FKBP14	94	2	0.66	4	8	23
G1TWB9		TBC1D5	Rab-GAP TBC domain-containing protein			1	0.66	2	3	4
G1SY85	Q9Y2T2	AP3M1	MHD domain-containing protein	AP-3 complex subunit mu-1	99	2	0.66	9	11	42
G1SN76	O94804	STK10	Serine/threonine kinase 10	Serine/threonine-protein kinase 10	90	2	0.66	2	4	5
G1SX37	Q9Y281	CFL2	Cofilin 2	Cofilin-2	91	2	0.66	8	11	55
	J3QQX2	ARHGDIA		Rho GDP-dissociation inhibitor 1		4	0.66	5	7	18
G1T634	Q9Y5X1	SNX9	Sorting nexin	Sorting nexin-9	89	2	0.66	7	7	23
O77708	D6R938	CAMK2D	Calcium/calmodulin-dependent protein kinase type II subunit delta	Calcium/calmodulin-dependent protein kinase (CaM kinase) II delta, isoform CRA_e	92	2	0.65	12	13	33
G1T9L2	Q86VN1	VPS36	GLUE N-terminal domain-containing protein	Vacuolar protein-sorting-associated protein 36	97	2	0.65	4	4	18

G1U7S8	F8W6C2	SPATS2L	Uncharacterized protein	SPATS2-like protein (Fragment)	100	3	0.65	4	4	9
G1U9U1	P50995	ANXA11	Annexin	Annexin A11	92	2	0.65	7	9	17
G1TUC2	P62633	CNBP	Uncharacterized protein	Cellular nucleic acid-binding protein	100	3	0.65	2	4	13
G1TCY4	Q9Y5X3	SNX5	Sorting nexin	Sorting nexin-5	98	2	0.65	4	6	12
G1T450		RO60	TROVE domain-containing protein			1	0.65	3	6	7
G1SS70	P61247	RPS3A	40S ribosomal protein S3a	40S ribosomal protein S3a	100	2	0.65	15	86	55
G1SCF0	P13807	GYS1	Glycogen [starch] synthase	Glycogen [starch] synthase, muscle	96	2	0.65	3	4	6
G1SDF2	A0A0A0MRJ6	PCMT1	Protein-L-isoaspartate O-methyltransferase	Protein-L-isoaspartate O-methyltransferase	96	2	0.65	5	7	21
G1T8D4	D6REX3	SEC31A	WD_REPEATS_REGION domain-containing protein	Protein transport protein Sec31A	91	2	0.65	29	60	35
G1T168	P46783	RPS10	S10_plectin domain-containing protein	40S ribosomal protein S10	100	2	0.65	10	16	40
	Q16181	SEPTIN7		Septin-7		4	0.65	19	50	57
G1T4F9	E9PR44	CRYAB	Alpha-crystallin B chain	Alpha-crystallin B chain (Fragment)	98	2	0.65	6	13	44
G1SNM1	P41250	GARS	Uncharacterized protein	Glycine--tRNA ligase	95	3	0.65	22	36	39
G1SQ80	Q8TDZ2	MICAL1	Uncharacterized protein	[F-actin]-monooxygenase MICAL1	83	3	0.65	4	4	8
G1SW44	H7C286	NAGK	BcrAD_BadFG domain-containing protein	N-acetyl-D-glucosamine kinase	97	2	0.65	6	8	27
G1SSW2	B4E0Y9	STK26	Protein kinase domain-containing protein	Serine/threonine-protein kinase 26	95	2	0.65	5	3	19
G1T8R3	P46939	UTRN	Uncharacterized protein	Utrophin	91	3	0.65	53	61	24
G1TM35	Q9Y3D6	FIS1	Fission, mitochondrial 1	Mitochondrial fission 1 protein	86	2	0.64	4	4	15
G1SZJ5	O15212	PFDN6	Uncharacterized protein	Prefoldin subunit 6	100	3	0.64	5	7	38
G1SWW4		SYNPO	Synaptopodin			1	0.64	3	6	6
G1SXE6	Q9Y6G9	DYNC1LI1	Uncharacterized protein	Cytoplasmic dynein 1 light intermediate chain 1	94	3	0.64	9	7	26
G1U7Q6	H7C1D4	TSN	Uncharacterized protein	Translin (Fragment)	99	3	0.64	3	47	22
G1SLH7	P42566	EPS15	Uncharacterized protein	Epidermal growth factor receptor substrate 15	93	3	0.64	4	4	6
G1SJZ4	J3KQ32	OLA1	Obg-like ATPase 1	Obg-like ATPase 1	100	2	0.64	9	17	34
P13642	P49591	SARS	Serine--tRNA ligase, cytoplasmic	Serine--tRNA ligase, cytoplasmic	97	2	0.64	14	23	32
G1SD27	B7ZC38	SH3GLB2	SH3 domain containing GRB2 like, endophilin B2	Endophilin-B2	92	2	0.64	2	2	6
G1SUV2		CAMLG	Calcium signal-modulating cyclophilin ligand			1	0.64	2	6	16
G1STV0		CAST	Calpastatin			1	0.64	12	24	21
G1T5B6	P12107	COL11A1	Fibrillar collagen NC1 domain-containing protein	Collagen alpha-1(XI) chain	98	2	0.64	29	53	24
G1TYW1		TPD52L2	TPD52 like 2			1	0.64	3	5	13
G1T2F8	H0YJH8	ATP6V1D	V-type proton ATPase subunit D	V-type proton ATPase subunit D (Fragment)	99	2	0.64	3	13	23
G1SUK5	O43583	DENR	Density-regulated protein	Density-regulated protein	97	2	0.64	2	4	9
G1U7Y3	E7END4	LOXL3	Uncharacterized protein	Lysyl oxidase homolog 3	87	3	0.64	3	3	5
	O15143	ARPC1B		Actin-related protein 2/3 complex subunit 1B		4	0.64	3	6	14
G1SP34	O15144	ARPC2	Arp2/3 complex 34 kDa subunit	Actin-related protein 2/3 complex subunit 2	100	2	0.64	15	29	54
G1SPB8	Q99733	NAP1L4	Uncharacterized protein	Nucleosome assembly protein 1-like 4	93	3	0.64	9	17	40
G1TS18		BSDC1	BSD domain containing 1			1	0.64	3	4	13
	K7EPT8	GFAP		Glial fibrillary acidic protein (Fragment)		4	0.63	3	213	15
G1T0Y9	Q13561	DCTN2	Uncharacterized protein	Dynactin subunit 2	97	3	0.63	10	23	43
G1TTJ1	Q6UWP2	DHRS11	Uncharacterized protein	Dehydrogenase/reductase SDR family member 11	92	3	0.63	4	5	21
G1SL62	P07355	ANXA2	Annexin	Annexin A2	98	2	0.63	26	58	71
Q9TTC6	P62937	PPIA	Peptidyl-prolyl cis-trans isomerase A	Peptidyl-prolyl cis-trans isomerase A	96	2	0.63	16	21	87
G1SHA4		TEP1	Telomerase associated protein 1			1	0.63	5	7	3
G1SQ22	Q9Y678	COPG1	Coatomer subunit gamma	Coatomer subunit gamma-1	99	2	0.63	35	150	58
G1TEA8	P12268	IMPDH2	Inosine-5~-monophosphate dehydrogenase	Inosine-5~-monophosphate dehydrogenase 2	99	2	0.63	9	19	29
G1T8R1		RAP1GAP2	Platelet-activating factor acetylhydrolase IB subunit alpha			1	0.63	8	8	24
G1SPM5	P61163	ACTR1A	Uncharacterized protein	Alpha-centractin	100	3	0.63	11	15	40
G1TAB2		GM2A	ML domain-containing protein			1	0.63	4	7	22
G1U949	Q15691	MAPRE1	Uncharacterized protein	Microtubule-associated protein RP/EB family member 1	99	3	0.63	8	21	43
G1SMY7	C9JIF9	APEH	Acylamino-acid-releasing enzyme	Acylamino-acid-releasing enzyme	93	2	0.63	7	9	18
P06813	A0A075B7C0	CAPNS1	Calpain small subunit 1	Calpain small subunit 1 (Fragment)	96	2	0.63	10	17	69
A0A494BI13		NAAA	N-acylethanolamine-hydrolyzing acid amidase			1	0.63	2	2	8
G1T7T8	P47712	PLA2G4A	Phospholipase A2	Cytosolic phospholipase A2	94	2	0.63	27	122	54
	A0A087X0W8	RELA		Transcription factor p65		4	0.63	2	2	7
G1SX32	O00743	PPP6C	Serine/threonine-protein phosphatase	Serine/threonine-protein phosphatase 6 catalytic subunit	100	2	0.63	2	2	16
	P62854	RPS26		40S ribosomal protein S26		4	0.63	2	11	21
G1SM05	Q9NR31	SAR1A	Uncharacterized protein	GTP-binding protein SAR1a	99	3	0.63	6	8	41
G1SIT9	P31946	YWHAB	14_3_3 domain-containing protein	14-3-3 protein beta/alpha	100	2	0.63	16	51	76
G1T545		BTBD9	Lactoylglutathione lyase			1	0.62	4	6	33

G1U9R8	P06396-2	GSN	Uncharacterized protein	Isoform 2 of Gelsolin	95	3	0.62	19	40	41
G1U5A6	A0A286YFF8	MON2	Uncharacterized protein	Protein MON2 homolog	98	3	0.62	6	7	6
G1TJC3	Q9BUF5	TUBB6	Tubulin beta chain	Tubulin beta-6 chain	97	2	0.62	17	21	57
G1TP81	Q9Y6W5	WASF2	WH2 domain-containing protein	Wiskott-Aldrich syndrome protein family member 2	94	2	0.62	4	6	10
G1SY00	P42858	HTT	Uncharacterized protein	Huntingtin	91	3	0.62	4	3	3
G1TR42		RNPEP	Leuk-A4-hydro_C domain-containing protein			1	0.62	5	4	10
G1TD38	H3BVG8	VPS35L	Uncharacterized protein	VPS35 endosomal protein sorting factor-like	93	3	0.62	6	10	4
G1SWW6	F5H459	AP3S1	Clat_adaptor_s domain-containing protein	AP complex subunit sigma	95	2	0.62	2	3	14
G1SKV7	Q96KP4	CNDP2	M20_dimer domain-containing protein	Cytosolic non-specific dipeptidase	90	2	0.62	12	16	38
G1SQF2	O43237	DYNC1LI2	Uncharacterized protein	Cytoplasmic dynein 1 light intermediate chain 2	98	3	0.62	9	13	37
P48738	Q00169	PITPNA	Phosphatidylinositol transfer protein alpha isoform	Phosphatidylinositol transfer protein alpha isoform	99	2	0.62	6	7	24
G1SY70	F5H365	SEC23A	Protein transport protein SEC23	Protein transport protein SEC23	96	2	0.62	22	38	47
G1T5Y1	P28482	MAPK1	Mitogen-activated protein kinase	Mitogen-activated protein kinase 1	98	2	0.62	11	9	44
G1TN33		SEPT8	Septin-type G domain-containing protein			1	0.62	8	6	24
G1T7D1		HIST1H1C	H15 domain-containing protein			1	0.62	10	23	33
G1SER8	P07737	PFN1	Profilin	Profilin-1	94	2	0.62	7	130	75
G1SEK2		PPM1F	PPM-type phosphatase domain-containing protein			1	0.62	2	2	11
G1T5Q4	Q9H446	RWDD1	RWD domain-containing protein	RWD domain-containing protein 1	91	2	0.62	4	5	27
G1T107	P61088	UBE2N	UBIQUITIN_CONJUGAT_2 domain-containing protein	Ubiquitin-conjugating enzyme E2 N	100	2	0.62	5	9	59
G1SEX8	Q9P299	COPZ2	Clat_adaptor_s domain-containing protein	Coatomer subunit zeta-2	84	2	0.61	4	6	23
G1U6H4	O95865	DDAH2	Uncharacterized protein	N(G),N(G)-dimethylarginine dimethylaminohydrolase 2	97	3	0.61	9	10	56
G1SN09	Q9Y5P6	GMPPB	NTP_transferase domain-containing protein	Mannose-1-phosphate guanyltransferase beta	99	2	0.61	4	3	22
G1SIT6	Q9BPX5	ARPC5L	Actin-related protein 2/3 complex subunit 5	Actin-related protein 2/3 complex subunit 5-like protein	98	2	0.61	7	11	63
G1SX73	E5RGS4	PFDN1	Uncharacterized protein	Prefoldin subunit 1	88	3	0.61	4	7	30
P30801		S100A6	Protein S100-A6			1	0.61	7	17	56
G1TAM3		TBCB	CAP-Gly domain-containing protein			1	0.61	2	2	11
Q9XS70	Q9BR76	CORO1B	Coronin-1B	Coronin-1B	93	2	0.61	9	26	27
G1SCI0	Q14204	DYNC1H1	Dynein cytoplasmic 1 heavy chain 1	Cytoplasmic dynein 1 heavy chain 1	98	2	0.61	121	73	52
G1TVQ3		DYNLRB1	Dynein light chain roadblock			1	0.61	3	4	51
G1TM81	A0A0B4J2C3	TPT1	Translationally-controlled tumor protein	Translationally-controlled tumor protein	98	2	0.61	5	67	34
G1SDL3	P54802	NAGLU	Uncharacterized protein	Alpha-N-acetylglucosaminidase	88	3	0.61	15	18	29
G1SG55	O43491	EPB41L2	FERM domain-containing protein	Band 4.1-like protein 2	90	2	0.61	38	89	43
G1SLX0	G3V4P8	GMFB	Glia maturation factor	Glia maturation factor beta (Fragment)	99	2	0.61	3	7	30
G1SHI0	H0Y4D4	ACAA1	Acetyl-CoA acyltransferase 1	3-ketoacyl-CoA thiolase, peroxisomal (Fragment)	62	2	0.61	8	7	34
G1U0I7	H3BSK9	ATXN2L	Ataxin 2 like	Ataxin-2-like protein (Fragment)	99	2	0.61	5	4	25
G1TKY3	P17655	CAPN2	Calpain-2 catalytic subunit	Calpain-2 catalytic subunit	94	2	0.61	22	36	49
	Q7Z406	MYH14		Myosin-14		4	0.61	14	62	8
B7NZQ6	P31150	GDI1	Rab GDP dissociation inhibitor	Rab GDP dissociation inhibitor alpha	99	2	0.61	16	20	48
G1TA72		HS1BP3	PX domain-containing protein			1	0.61	2	2	6
G1T967	A0A0U1RRB6	EXOC6B	Exocyst complex component	Exocyst complex component	99	2	0.61	7	8	15
G1T473	F8VQE1	LIMA1	LIM zinc-binding domain-containing protein	LIM domain and actin-binding protein 1	85	2	0.61	22	36	38
G1T7A8	Q9Y646	CPQ	Peptidase_M28 domain-containing protein	Carboxypeptidase Q	87	2	0.60	2	2	4
G1T7Z0	P52209	PGD	6-phosphogluconate dehydrogenase, decarboxylating	6-phosphogluconate dehydrogenase, decarboxylating	93	2	0.60	14	16	31
G1SPL1	A0A286YF22	PHGDH	D-3-phosphoglycerate dehydrogenase	D-3-phosphoglycerate dehydrogenase	93	2	0.60	26	99	61
G1TBQ6	O95292	VAPB	VAMP associated protein B and C	Vesicle-associated membrane protein-associated protein B/C	90	2	0.60	3	3	17
	Q63ZY3	KANK2		KN motif and ankyrin repeat domain-containing protein 2		4	0.60	9	5	13
G1SXQ0	A0A0A0MTN3	GSTM3	Glutathione S-transferase	Glutathione S-transferase	90	2	0.60	10	22	37
G1TPZ1		LGALS1	Galectin			1	0.60	9	29	75
G1SE28	C9JNW5	RPL24	TRASH domain-containing protein	60S ribosomal protein L24	100	2	0.60	6	9	34
G1SF08	C9K025	RPL35A	Uncharacterized protein	60S ribosomal protein L35a (Fragment)	99	3	0.60	3	2	23
G1U2E3	Q9H444	CHMP4B	Uncharacterized protein	Charged multivesicular body protein 4b	100	3	0.60	7	8	40
G1SMX4	A0A0G2JH68	DIAPH1	Uncharacterized protein	Protein diaphanous homolog 1	90	3	0.60	15	22	22
G1T3R5		ERO1A	Endoplasmic reticulum oxidoreductase 1 alpha			1	0.59	15	30	34
G1TAK5		WNK4	WNK lysine deficient protein kinase 4			1	0.59	3	3	2
G1SKL7	Q99536	VAT1	Vesicle amine transport 1	Synaptic vesicle membrane protein VAT-1 homolog	78	2	0.59	9	16	35
G1T1V9	P54652	HSPA2	Uncharacterized protein	Heat shock-related 70 kDa protein 2	98	3	0.59	26	38	58
G1T3D9	P62241	RPS8	40S ribosomal protein S8	40S ribosomal protein S8	94	2	0.59	6	23	37
P68105	P68104	EEF1A1	Elongation factor 1-alpha 1	Elongation factor 1-alpha 1	100	2	0.59	22	768	65
G1TMW2		TOLLIP	Toll interacting protein			1	0.59	4	4	25

	Q9NP61	ARFGAP3		ADP-ribosylation factor GTPase-activating protein 3		4	0.59	2	2	4
G1SUZ7		ARSA	Sulfatase domain-containing protein			1	0.59	4	3	18
G1SGX4	P62249	RPS16	Uncharacterized protein	40S ribosomal protein S16	100	3	0.59	7	12	37
G1T9W8	Q8TC07	TBC1D15	Rab-GAP TBC domain-containing protein	TBC1 domain family member 15	92	2	0.59	2	2	3
G1U0Q2	K7EJ78	RPS15	Uncharacterized protein	40S ribosomal protein S15	99	3	0.59	2	14	28
G1SIL8	A0A087WWM4	GMPR2	GMP reductase	GMP reductase	90	2	0.59	3	2	13
G1SVB5	J3QRN6	MYO1D	Uncharacterized protein	Unconventional myosin-Id	95	3	0.59	15	16	20
G1SI83	Q99584	S100A13	S_100 domain-containing protein	Protein S100-A13	91	2	0.59	3	5	27
B7NZG7	O60493	SNX3	Sorting nexin 3 (Predicted)	Sorting nexin-3	100	2	0.59	2	4	17
G1TCD4	E5RGF9	FAM114A2	Uncharacterized protein	Protein FAM114A2 (Fragment)	80	3	0.59	5	2	18
G1T9V6	Q9UPU5	USP24	Ubiquitin specific peptidase 24	Ubiquitin carboxyl-terminal hydrolase 24	98	2	0.59	7	7	5
G1SHS7	O75083	WDR1	WD_REPEATS_REGION domain-containing protein	WD repeat-containing protein 1	94	2	0.59	21	48	51
G1SW24	P49588	AARS	AA_TRNA_LIGASE_II_ALA domain-containing protein	Alanine--tRNA ligase, cytoplasmic	93	2	0.58	33	25	47
G1T9N3	B5MBZ0	EML4	Uncharacterized protein	Echinoderm microtubule-associated protein-like 4	92	3	0.58	4	7	5
G1T3V2	P04792	HSPB1	SHSP domain-containing protein	Heat shock protein beta-1	91	2	0.58	8	21	52
G1SXK6	Q9NR12	PDLIM7	PDZ and LIM domain 7	PDZ and LIM domain protein 7	92	2	0.58	8	24	26
G1SZ12	E7EPB3	RPL14	Ribosomal_L14e domain-containing protein	60S ribosomal protein L14	96	2	0.58	6	12	27
G1T7R2	P62258	YWHAE	14_3_3 domain-containing protein	14-3-3 protein epsilon	100	2	0.58	18	195	78
G1TX63		CDC42BPA	Non-specific serine/threonine protein kinase			1	0.58	4	2	4
G1SM64	Q5T985	ITIH2	Uncharacterized protein	Inter-alpha-trypsin inhibitor heavy chain H2	85	3	0.58	5	4	8
G1ST05	Q15120	PDK3	Protein-serine/threonine kinase	[Pyruvate dehydrogenase (acetyl-transferring)] kinase isozyme 3, mitochondrial	99	2	0.58	6	9	26
G1SXR6	O60282	KIF5C	Kinesin-like protein	Kinesin heavy chain isoform 5C	99	2	0.58	20	13	29
G1SL41		GUSB	Beta-glucuronidase			1	0.58	11	20	30
G1T004	H0Y987	PGM3	Phosphoacetylglucosamine mutase	Phosphoacetylglucosamine mutase	90	2	0.58	8	19	27
G1U0Q7		SEPT2	Septin-type G domain-containing protein			1	0.58	13	61	54
G1TEG8	Q8WUM4	PDCD6IP	BRO1 domain-containing protein	Programmed cell death 6-interacting protein	94	2	0.58	24	87	36
	A0A2R8Y3S6	SNX27		Sorting nexin-27 (Fragment)		4	0.58	2	3	20
G1TKE3	X6RJP6	TAGLN2	Transgelin	Transgelin-2 (Fragment)	77	2	0.58	6	11	40
A7X8X3		HPRT	Hypoxanthine phosphoribosyltransferase			1	0.58	6	9	31
G1SF47		SEPT11	Septin-type G domain-containing protein			1	0.58	12	14	34
G1SDY5	P63104	YWHAZ	14_3_3 domain-containing protein	14-3-3 protein zeta/delta	100	2	0.58	15	132	65
G1U974	A0A1C7CYX9	DPYSL2	Amidohydro-rel domain-containing protein	Dihydropyrimidinase-related protein 2	98	2	0.57	19	38	51
G1SVW5	P36578	RPL4	Ribos_L4_asso_C domain-containing protein	60S ribosomal protein L4	96	2	0.57	14	46	36
G1SQ03	Q8WWI5	SLC44A1	Solute carrier family 44 member 1	Choline transporter-like protein 1	93	2	0.57	3	4	7
G1T3I9	P20073	ANXA7	Annexin	Annexin A7	93	2	0.57	10	13	20
G1TBS1	Q99497	PARK7	DJ-1_PfpI domain-containing protein	Protein/nucleic acid deglycase DJ-1	96	2	0.57	11	21	72
	A0A087WY85	UBE2D3		Ubiquitin-conjugating enzyme E2 D3		4	0.57	2	3	20
Q6TYA7		GJA1	Gap junction alpha-1 protein			1	0.57	2	4	6
G1SN21	P00491	PNP	Purine nucleoside phosphorylase	Purine nucleoside phosphorylase	90	2	0.56	13	28	62
G1TBZ5	E9PCX2	AKR1B1	Aldo-keto reductase family 1 member B1	Aldo-keto reductase family 1 member B1	85	2	0.56	6	14	29
	P06753-5	TPM3		Isoform 5 of Tropomyosin alpha-3 chain		4	0.56	21	22	62
G1U411	F8WBG8	DBNL	Uncharacterized protein	Drebrin-like protein	95	3	0.56	9	19	31
G1T6L5	O75368	SH3BGRL	Uncharacterized protein	SH3 domain-binding glutamic acid-rich-like protein	98	3	0.56	5	10	31
G1TN08	P37235	HPCAL1	Uncharacterized protein	Hippocalcin-like protein 1	99	3	0.56	6	5	34
G1SY84	Q13409-2	DYNC1I2	WD_REPEATS_REGION domain-containing protein	Isoform 2B of Cytoplasmic dynein 1 intermediate chain 2	92	2	0.56	9	2	29
G1TAC4		GLRX	Glutaredoxin-1			1	0.56	3	9	38
G1T432	Q01518	CAP1	Adenylyl cyclase-associated protein	Adenylyl cyclase-associated protein 1	96	2	0.56	24	166	65
G1U670	A0A087WW40	SH3GLB1	Uncharacterized protein	Endophilin-B1	95	3	0.56	2	3	6
G1SNT1	O75822	EIF3J	Eukaryotic translation initiation factor 3 subunit J	Eukaryotic translation initiation factor 3 subunit J	92	2	0.55	11	17	48
G1SW40	E7EMB1	SWAP70	PH domain-containing protein	Switch-associated protein 70	77	2	0.55	7	21	16
G1T1F0	A0A2R8Y811	RPS14	Uncharacterized protein	40S ribosomal protein S14 (Fragment)	100	3	0.55	5	10	37
G1SHR7	Q9P0J7	KCMF1	C2H2-type domain-containing protein	E3 ubiquitin-protein ligase KCMF1	99	2	0.55	2	3	9
G1SHB9	P40121	CAPG	Uncharacterized protein	Macrophage-capping protein	94	3	0.55	9	20	44
G1TV76	Q9Y4D1	DAAM1	Uncharacterized protein	Disheveled-associated activator of morphogenesis 1	97	3	0.55	4	4	6
G1T6D1	P62829	RPL23	Uncharacterized protein	60S ribosomal protein L23	100	3	0.55	6	14	57
G1T705	P00387	CYB5R3	NADH-cytochrome b5 reductase	NADH-cytochrome b5 reductase 3	90	2	0.55	12	48	57
G1SR49		SEPT10	Septin-type G domain-containing protein			1	0.55	6	9	17
G1TCW5	A0A494C165	PEPD	Peptidase D	Xaa-Pro dipeptidase (Fragment)	79	2	0.54	7	11	22
G1T5E6	O60749	SNX2	PX domain-containing protein	Sorting nexin-2	98	2	0.54	11	10	28

G1T5W7	Q14376	GALE	NAD(P)-bd_dom domain-containing protein	UDP-glucose 4-epimerase	95	2	0.54	7	13	27
G1SR27	P62993	GRB2	Uncharacterized protein	Growth factor receptor-bound protein 2	100	3	0.54	3	4	22
G1TNJ2	Q15942	ZYX	Uncharacterized protein	Zyxin	87	3	0.54	6	19	15
G1SFX7	P62330	ARF6	Uncharacterized protein	ADP-ribosylation factor 6	100	3	0.54	5	8	33
	M0QXF7	MYDGF		Myeloid-derived growth factor (Fragment)		4	0.54	2	2	19
G1T823	H3BT58	COTL1	Coactosin like F-actin binding protein 1	Coactosin-like protein	96	2	0.54	3	9	27
G1SZX4	Q14571	ITPR2	Uncharacterized protein	Inositol 1,4,5-trisphosphate receptor type 2	97	3	0.54	13	11	9
	H0Y507	SH3PXD2A		SH3 and PX domain-containing protein 2A (Fragment)		4	0.54	2	2	3
G1SCP7	P46940	IQGAP1	Uncharacterized protein	Ras GTPase-activating-like protein IQGAP1	97	3	0.54	75	216	60
G1TU12	Q5H907	MAGED2	MAGE domain-containing protein	Melanoma antigen family D, 2, isoform CRA_d	79	2	0.54	5	3	10
P62943	P62942	FKBP1A	Peptidyl-prolyl cis-trans isomerase FKBP1A	Peptidyl-prolyl cis-trans isomerase FKBP1A	100	2	0.53	2	6	25
G1SJ56	P18206	VCL	Uncharacterized protein	Vinculin	99	3	0.53	54	163	59
P53787		EEF1D	Elongation factor 1-delta			1	0.53	4	15	24
G1U0Y0	A0A2Q3DQE3	CAMK2G	Protein kinase domain-containing protein	Calcium/calmodulin-dependent protein kinase (CaM kinase) II gamma, isoform CRA_d	91	2	0.53	7	2	23
G1U8T9	Q96CX2	KCTD12	Potassium channel tetramerization domain containing 12	BTB/POZ domain-containing protein KCTD12	97	2	0.53	3	3	17
G1U8P2	J3KTE4	RPL19	Ribosomal protein L19	Ribosomal protein L19	98	2	0.53	7	15	30
	H7BZL4	GPC1		Glypican-1 (Fragment)		4	0.53	5	6	29
G1SWK3	P49902	NT5C2	Uncharacterized protein	Cytosolic purine 5~-nucleotidase	100	3	0.53	8	13	21
G1SQ02	A0A0A0MSI0	PRDX1	Thioredoxin domain-containing protein	Peroxiredoxin-1 (Fragment)	98	2	0.53	10	17	57
G1SKQ9		SEPT6	Septin-type G domain-containing protein			1	0.53	8	3	22
G1TPC5		TPD52	Tumor protein D52			1	0.53	3	6	19
G1SMT2		FAM129B	Family with sequence similarity 129 member B			1	0.52	17	35	36
G1SPD1		MTPN	ANK_REP_REGION domain-containing protein			1	0.52	2	5	32
G1SYE0		ABHD14B	AB hydrolase-1 domain-containing protein			1	0.52	4	7	21
G1SIB6	M0R192	BLVRB	Biliverdin reductase B	Flavin reductase (NADPH)	73	2	0.52	7	32	51
G1TFI4	Q96K17	BTF3L4	Transcription factor BTF3	Transcription factor BTF3 homolog 4	100	2	0.52	3	3	39
G1SE57	P18085	ARF4	Uncharacterized protein	ADP-ribosylation factor 4	95	3	0.52	10	17	76
G1SG54	A0A0U1RQT1	ACAP2	Arf-GAP with coiled-coil, ANK repeat and PH domain-containing protein 2	Arf-GAP with coiled-coil, ANK repeat and PH domain-containing protein 2 (Fragment)	99	2	0.52	4	3	11
G1SZJ7	A0A2R8YEC9	MANBA	Uncharacterized protein	Beta-mannosidase	75	3	0.52	7	8	11
P35748	P35749	MYH11	Myosin-11	Myosin-11	97	2	0.51	42	37	29
G1T3J0		SLC26A7	Anion exchange transporter			1	0.51	2	2	7
	Q08209-2	PPP3CA		Isoform 2 of Serine/threonine-protein phosphatase 2B catalytic subunit alpha isoform		4	0.51	9	15	27
G1T277	F8VR50	ARPC3	Actin-related protein 2/3 complex subunit 3	Actin-related protein 2/3 complex subunit 3 (Fragment)	100	2	0.51	3	8	17
G1SP27	Q16204	CCDC6	Uncharacterized protein	Coiled-coil domain-containing protein 6	93	3	0.51	14	28	31
G1TN29	Q86UU1	PHLDB1	Pleckstrin homology like domain family B member 1	Pleckstrin homology-like domain family B member 1	89	2	0.51	8	3	6
G1SN43	F8W930	IGF2BP2	Uncharacterized protein	Insulin-like growth factor 2 mRNA-binding protein 2	98	3	0.51	7	6	15
	P39019	RPS19		40S ribosomal protein S19		4	0.51	11	34	57
G1T345		UBR1	E3 ubiquitin-protein ligase			1	0.51	2	2	2
G1TIB4	P62857	RPS28	Ribosomal protein S28	40S ribosomal protein S28	100	2	0.51	3	8	46
G1TKH3		SOD1	Superoxide dismutase [Cu-Zn]			1	0.51	3	108	35
G1SN67		SERPINB1	Serpin family B member 1			1	0.50	2	2	6
G1U5Q7		ARPC4	Actin-related protein 2/3 complex subunit 4			1	0.50	7	35	46
G1SGL0	Q92629	SGCD	Uncharacterized protein	Delta-sarcoglycan	98	3	0.50	3	5	14
G1SRR2	E9PMP7	LMO7	Uncharacterized protein	LIM domain only protein 7 (Fragment)	70	3	0.50	30	40	24
P00883	P04075	ALDOA	Fructose-bisphosphate aldolase A	Fructose-bisphosphate aldolase A	98	2	0.50	24	112	76
G1SQ10	Q9NQT8	KIF13B	Uncharacterized protein	Kinesin-like protein KIF13B	89	3	0.50	13	4	9
G1SDH3	E9PIG4	PRCP	Uncharacterized protein	Lysosomal Pro-X carboxypeptidase (Fragment)	89	3	0.50	8	13	30
G1SIB9	P11216	PYGB	Alpha-1,4 glucan phosphorylase	Glycogen phosphorylase, brain form	95	2	0.50	31	23	45
G1SNE8	P31949	S100A11	Protein S100	Protein S100-A11	87	2	0.50	5	37	65
	Q9UHD8	SEPTIN9		Septin-9		4	0.49	10	7	21
G1SIT5	F2Z388	RPL35	Uncharacterized protein	60S ribosomal protein L35	93	3	0.49	3	5	25
G1T8B5	Q9H1E3	NUCKS1	Nuclear casein kinase and cyclin dependent kinase substrate 1	Nuclear ubiquitous casein and cyclin-dependent kinase substrate 1	94	2	0.49	2	3	9
G1U5M4	J3QQS9	SLC16A3	Solute carrier family 16 member 3	Monocarboxylate transporter 4 (Fragment)	92	2	0.49	3	4	6
G1TV31	O15511	ARPC5	Actin-related protein 2/3 complex subunit 5	Actin-related protein 2/3 complex subunit 5	95	2	0.49	8	40	54
G1T3D7		NANS	AFP-like domain-containing protein			1	0.49	4	6	14
G1U9R0	A8MX94	GSTP1	Uncharacterized protein	Glutathione S-transferase P	71	3	0.49	10	84	49
	A0A3B3ISV4	VKORC1L1		Vitamin K epoxide reductase complex subunit 1-like protein 1		4	0.48	2	3	9
G1SZD6	P27348	YWHAQ	14-3-3 protein theta	14-3-3 protein theta	99	2	0.48	16	118	68
G1SXU2	Q96HC4	PDLIM5	Uncharacterized protein	PDZ and LIM domain protein 5	88	3	0.48	10	29	22

G1T013	P48163	ME1	Malic enzyme	NADP-dependent malic enzyme	95	2	0.48	6	16	22
G1SY88	P55263	ADK	PfkB domain-containing protein	Adenosine kinase	98	2	0.47	8	11	38
	B4DDF4	CNN2		Calponin		4	0.46	5	5	18
	P32119	PRDX2		Peroxiredoxin-2		4	0.46	5	10	31
G1SQR6	Q15417	CNN3	Calponin	Calponin-3	98	2	0.46	15	521	55
	Q16658	FSCN1		Fascin		4	0.46	10	9	23
G1SNT8	P08133	ANXA6	Annexin	Annexin A6	96	2	0.46	33	62	48
G1SNY0	E9PJD9	RPL27A	Ribosomal_L18e/L15P domain-containing protein	60S ribosomal protein L27a	98	2	0.46	3	5	22
G1T8C8	A0A087X0R6	SNX12	Sorting nexin 12	Sorting nexin-12	100	2	0.46	2	3	13
	H0Y7A7	CALM2		Calmodulin-2 (Fragment)		4	0.46	7	55	49
G1U723		PGER5	3alpha/17beta/20alpha-hydroxysteroid dehydrogenase			1	0.45	5	6	20
	P32418	SLC8A1		Sodium/calcium exchanger 1		4	0.45	2	2	4
	P68371	TUBB4B		Tubulin beta-4B chain		4	0.45	22	21	71
G1SKS8	E7EVA0	MAP4	Microtubule-associated protein	Microtubule-associated protein	85	2	0.45	25	49	30
	P51178	PLCD1		1-phosphatidylinositol 4,5-bisphosphate phosphodiesterase delta-1		4	0.45	2	3	3
G1TRM4	P62280	RPS11	Ribosomal_S17_N domain-containing protein	40S ribosomal protein S11	100	2	0.45	9	20	55
G1T8Z0	P30041	PRDX6	Thioredoxin domain-containing protein	Peroxiredoxin-6	95	2	0.45	15	32	64
G1SPF5	P18669	PGAM1	Uncharacterized protein	Phosphoglycerate mutase 1	100	3	0.44	13	88	67
G1SWS9	P08670	VIM	IF rod domain-containing protein	Vimentin	97	2	0.44	50	###	88
	H0Y5B4	RPL36A		60S ribosomal protein L36a		4	0.44	3	6	16
G1SUI9	E7EX44	CALD1	Uncharacterized protein	Caldesmon	84	3	0.43	24	103	39
G1SPQ9	O94875	SORBS2	Uncharacterized protein	Sorbin and SH3 domain-containing protein 2	90	3	0.43	17	23	19
G1T4P7	Q9Y4G6	TLN2	Talin 2	Talin-2	98	2	0.43	19	9	11
G1TA83	P09525	ANXA4	Annexin	Annexin A4	94	2	0.43	13	24	51
G1T2Q8	Q96M27	PRRC1	NTPase_I-T domain-containing protein	Protein PRRC1	90	2	0.43	7	13	22
G1T315	A0A2R8Y2R1	SGCE	CADG domain-containing protein	Epsilon-sarcoglycan (Fragment)	88	2	0.43	2	5	7
G1SW82	H0Y9Y3	SYNPO2	PDZ domain-containing protein	Synaptopodin-2 (Fragment)	80	2	0.43	3	4	4
G1SD83		ITGA6	Integrin_alpha2 domain-containing protein			1	0.42	2	6	2
Q8WN94		DBI	Acyl-CoA-binding protein			1	0.42	3	5	39
Q6SQH4	P60903	S100a10	Protein S100-A10	Protein S100-A10	100	2	0.41	4	11	35
G1SUY3	F8VQR7	CSRP2	Uncharacterized protein	Cysteine and glycine-rich protein 2	100	3	0.40	6	15	38
	P33241	LSP1		Lymphocyte-specific protein 1		4	0.40	3	5	9
G1T2C4	Q01995	TAGLN	Transgelin	Transgelin	99	2	0.40	11	166	58
G1TYA7	P07195	LDHB	L-lactate dehydrogenase	L-lactate dehydrogenase B chain	100	2	0.40	17	170	58
	P22392-2	NME2		Isoform 3 of Nucleoside diphosphate kinase B		4	0.40	7	24	35
G1SHL8	P42224	STAT1	Signal transducer and activator of transcription	Signal transducer and activator of transcription 1-alpha/beta	95	2	0.40	13	17	27
G1TRY5	P13797	PLS3	Uncharacterized protein	Plastin-3	100	3	0.39	37	135	77
G1TE78	Q15121	PEA15	DED domain-containing protein	Astrocytic phosphoprotein PEA-15	100	2	0.38	5	3	43
G1TKE7		IQGAP2	IQ motif containing GTPase activating protein 2			1	0.38	4	2	3
G1U7U3		NME1	Nucleoside diphosphate kinase			1	0.38	7	8	57
G1SPY1	E9PGM4	GBE1	Aamy domain-containing protein	1,4-alpha-glucan-branching enzyme	93	2	0.38	21	35	42
G1TMV1	P60981	DSTN	ADF-H domain-containing protein	Destrin	100	2	0.38	9	69	58
G1SNP7	E9PGC8	MAP1A	Uncharacterized protein	Microtubule-associated protein 1A	79	3	0.38	6	3	4
G1SYN4		PTX3	LamGL domain-containing protein			1	0.38	11	20	29
G1TDB3	P62851	RPS25	Uncharacterized protein	40S ribosomal protein S25	100	3	0.37	3	5	16
G1SZ47	P62266	RPS23	Uncharacterized protein	40S ribosomal protein S23	100	3	0.37	6	103	48
P11974-2		PKM	Isoform M2 of Pyruvate kinase PKM			1	0.36	38	442	81
G1U7J6		PRKCA	Protein kinase C			1	0.35	2	2	6
G1U634	P08473	MME	Neprilysin	Neprilysin	94	2	0.34	41	6	61
	Q9Y2V2	CARHSP1		Calcium-regulated heat-stable protein 1		4	0.34	2	2	18
G1TYY5	Q14847	LASP1	LIM and SH3 domain protein 1	LIM and SH3 domain protein 1	94	2	0.34	6	9	23
G1TBJ4	Q92597	NDRG1	Uncharacterized protein	Protein NDRG1	96	3	0.34	3	3	17
P00939	P60174	TPI1	Triosephosphate isomerase	Triosephosphate isomerase	93	2	0.34	15	56	72
G1T2K1	O95340	PAPSS2	Uncharacterized protein	Bifunctional 3~-phosphoadenosine 5~-phosphosulfate synthase 2	94	3	0.34	5	8	14
G1SH05	P07437	TUBB	Tubulin beta chain	Tubulin beta chain	100	2	0.33	23	119	71
G1T6W4		ANXA8	Annexin			1	0.32	18	66	66
G1T0H8	Q01813	PFKP	ATP-dependent 6-phosphofructokinase	ATP-dependent 6-phosphofructokinase, platelet type	93	2	0.31	22	30	42
G1SFG6	A0A0A0MTS2	GPI	Glucose-6-phosphate isomerase	Glucose-6-phosphate isomerase (Fragment)	93	2	0.31	22	122	56
G1T7Z6	P00558	PGK1	Phosphoglycerate kinase	Phosphoglycerate kinase 1	99	2	0.30	26	82	72

G1T387	Q14247	CTTN	Cortactin	Src substrate cortactin	88	2	0.30	5	2	13
G1TBU2		KIAA1217	KIAA1217			1	0.29	2	2	2
G1SYD2		AK4	Adenylate kinase 4, mitochondrial			1	0.29	6	8	41
G1SZ91		FABP5	Lipocln_cytosolic_FA-bd_dom domain-containing protein			1	0.29	4	7	37
G1SMS2	Q8WX93	PALLD	Uncharacterized protein	Palladin	87	3	0.28	20	41	20
G1SW77	Q8NC51	SERBP1	HABP4_PAI-RBP1 domain-containing protein	Plasminogen activator inhibitor 1 RNA-binding protein	99	2	0.28	7	15	19
	Q09666	AHNAK		Neuroblast differentiation-associated protein AHNAK		4	0.28	33	48	6
G1T5T8	O00151	PDLIM1	Uncharacterized protein	PDZ and LIM domain protein 1	92	3	0.28	14	29	60
	P17858	PFKL		ATP-dependent 6-phosphofructokinase, liver type		4	0.25	8	11	12
G1SYJ4	P06733	ENO1	Uncharacterized protein	Alpha-enolase	96	3	0.23	24	136	72
G1TAJ3	P00338	LDHA	L-lactate dehydrogenase	L-lactate dehydrogenase A chain	94	2	0.18	21	208	66
G1SY87		CD200	Ig-like domain-containing protein			1	0.14	2	4	7
G1SZ00	P21291	CSRP1	Uncharacterized protein	Cysteine and glycine-rich protein 1	98	3	0.14	9	2	48

A. Percent sequence Identity (rounded to the nearest integer) between the indicated rabbit and human proteins from blastp analysis of the rabbit and human UniProt databases.
B. Database Identification Categories: Category 1, Characterized in the rabbit database only; Category 2, Characterized in both the rabbit and human databases; Category 3, Uncharacterized in the rabbit database but characterized in the human database; Category 4, Characterized in the human database only.
C. Number of unique peptides quantified.
D. Number of peptide intensities summed for protein quantitation.

Supplemental Table S5
Myofibroblasts Derived from Rabbit Cornea & Bone Marrow
Average Relative Protein Abundance, n = 4 animals

Total Proteins Quantified = 2420; Median Protein iTRAQ Ratio =1.087; Mean Protein iTRAQ Ratio = 1.078; Standard Deviation (SD) = 0.366
Yellow = 1SD from the mean; Brown = 2SD from the mean; Green = $p \leq 0.05$

Rabbit Accession UniProt	Human Accession UniProt	Gene Symbol	Rabbit Protein Description	Human Protein Description	Database Identification category[A]	% identity[B]	Sample Frequency	Linear Ratio Cornea/Bone marrow	SEM[C]	Moderated Adjusted p value[D]	Differentially Expressed Proteins
	A0A2R8Y7G9	H3.Y		Histone domain-containing protein	4		4	7.579	0.414	2.8E-04	X
	P02765	AHSG		Alpha-2-HS-glycoprotein	4		4	4.891	0.126	3.1E-04	X
G1SIW5	Q92820	GGH	Folate gamma-glutamyl hydrolase	Gamma-glutamyl hydrolase	2	82	4	4.783	0.141	3.5E-04	X
O19105		SLC1A5	Neutral amino acid transporter B(0)		1		4	3.416	0.081	1.7E-04	X
G1TEP2		FAM210B	DUF1279 domain-containing protein		1		4	3.204	0.243	8.1E-04	X
G1U4P8	Q9BXN1	ASPN	LRRNT domain-containing protein	Asporin	2	88	4	3.051	0.180	3.5E-04	X
G1TUE1		ATP1B1	Sodium/potassium-transporting ATPase subunit beta		1		4	2.975	0.086	1.7E-04	X
G1SDA2	P29762	CRABP1	FABP domain-containing protein	Cellular retinoic acid-binding protein 1	2	99	4	2.887	0.079	1.7E-04	X
G1TW43	J3QSU6	TNC	Uncharacterized protein	Tenascin	3	74	4	2.735	0.074	1.7E-04	X
G1ST69	P20700	LMNB1	Uncharacterized protein	Lamin-B1	3	98	4	2.733	0.063	1.7E-04	X
G1T380	Q02388	COL7A1	Uncharacterized protein	Collagen alpha-1(VII) chain	3	87	4	2.733	0.246	1.6E-03	X
G1SJF4	A0A0A0MR51	FADS1	Fatty acid desaturase 1	Acyl-CoA (8-3)-desaturase	2	93	4	2.660	0.172	4.7E-04	X
G1T3Y8	P10809	HSPD1	Uncharacterized protein	60 kDa heat shock protein, mitochondrial	3	99	4	2.657	0.098	1.7E-04	X
G1SJW7	J3QLE5	SNRPN	Small nuclear ribonucleoprotein-associated protein	Small nuclear ribonucleoprotein-associated protein N (Fragment)	2	100	4	2.573	0.046	1.7E-04	X
G1SK42	P21980	TGM2	TGc domain-containing protein	Protein-glutamine gamma-glutamyltransferase 2	2	87	4	2.554	0.130	2.8E-04	X
	A0A2R8Y7C0	HBA2		Hemoglobin subunit alpha (Fragment)	4		4	2.549	0.228	1.6E-03	X
G1TAK1		COA3	Coiled-coil_56 domain-containing protein		1		4	2.535	0.131	2.8E-04	X
G1SEF5	Q8NAV1	PRPF38A	PRP38_assoc domain-containing protein	Pre-mRNA-splicing factor 38A	2	100	4	2.518	0.138	3.3E-04	X
	P16104	H2AFX		Histone H2AX	4		4	2.467	0.196	1.1E-03	X
G1SNX5	B4DY09	ILF2	Interleukin enhancer binding factor 2	Interleukin enhancer-binding factor 2	2	100	4	2.454	0.034	1.7E-04	X
G1T2X0	Q99541	PLIN2	Perilipin	Perilipin-2	2	88	4	2.448	0.245	2.6E-03	X
G1SI79	P51991	HNRNPA3	Uncharacterized protein	Heterogeneous nuclear ribonucleoprotein A3	3	100	4	2.443	0.076	1.7E-04	X
G1T017		SLC1A4	Solute carrier family 1 member 4		1		4	2.418	0.031	1.7E-04	X
G1SFC6	A0A087WV29	NAT10	RNA cytidine acetyltransferase	RNA cytidine acetyltransferase	2	96	4	2.406	0.103	2.2E-04	X
G1TD26	D6RAA6	TMEM33	Uncharacterized protein	Transmembrane protein 33 (Fragment)	3	99	4	2.403	0.071	1.7E-04	X
G1SUP9	P46087	NOP2	SAM_MT_RSMB_NOP domain-containing protein	Probable 28S rRNA (cytosine(4447)-C(5))-methyltransferase	2	77	4	2.396	0.090	1.9E-04	X
G1SDL7		ARG2	Arginase		1		4	2.387	0.245	3.0E-03	X
G1TX53		NDUFA8	NADH dehydrogenase [ubiquinone] 1 alpha subcomplex subunit 8		1		4	2.370	0.186	1.1E-03	X
P00389	P16435	POR	NADPH--cytochrome P450 reductase	NADPH--cytochrome P450 reductase	2	92	4	2.365	0.107	2.5E-04	X
G1TLW3	J3KTA4	DDX5	Uncharacterized protein	Probable ATP-dependent RNA helicase DDX5	3	96	4	2.351	0.040	1.7E-04	X
G1SDW8	H0Y2P0	CD44	Link domain-containing protein	CD44 antigen (Fragment)	2	91	4	2.339	0.147	5.2E-04	X
G1T4M2	O60264	SMARCA5	Uncharacterized protein	SWI/SNF-related matrix-associated actin-dependent regulator of chromatin subfamily A member 5	3	100	4	2.321	0.104	2.5E-04	X
G1T2K5	I3L1L3	MYBBP1A	Uncharacterized protein	Myb-binding protein 1A (Fragment)	3	69	4	2.318	0.085	1.9E-04	X
G1SQ07		DTYMK	Thymidylate_kin domain-containing protein		1		4	2.283	0.037	1.7E-04	X
G1SI26	Q4VC31	CCDC58	Uncharacterized protein	Coiled-coil domain-containing protein 58	3	95	4	2.277	0.065	1.7E-04	X
G1TX84	Q9Y5J1	UTP18	WD_REPEATS_REGION domain-containing protein	U3 small nucleolar RNA-associated protein 18 homolog	2	89	4	2.269	0.052	1.7E-04	X
G1SMB3	Q9NV31	IMP3	S4 RNA-binding domain-containing protein	U3 small nucleolar ribonucleoprotein protein IMP3	2	99	4	2.260	0.058	1.7E-04	X
G1SE74	Q7KZ85	SUPT6H	Transcription elongation factor spt6	Transcription elongation factor SPT6	2	99	4	2.247	0.106	2.8E-04	X
G1T3Y0	P98082	DAB2	PID domain-containing protein	Disabled homolog 2	2	90	4	2.233	0.113	3.3E-04	X
G1SIJ6	Q13308	PTK7	Protein tyrosine kinase 7 (inactive)	Inactive tyrosine-protein kinase 7	2	93	4	2.232	0.068	1.7E-04	X
G1TD41	G8JLB6	HNRNPH1	Uncharacterized protein	Heterogeneous nuclear ribonucleoprotein H	3	98	4	2.226	0.033	1.7E-04	X
G1T6T0	J3KNJ3	NAALAD2	Uncharacterized protein	N-acetylated-alpha-linked acidic dipeptidase 2	3	89	4	2.215	0.074	1.9E-04	X
G1T5I0		SLC7A1	Solute carrier family 7 member 1		1		4	2.200	0.053	1.7E-04	X
A0A140TAV7	J3KPF3	SLC3A2	4F2 cell-surface antigen heavy chain	4F2 cell-surface antigen heavy chain	2	81	4	2.200	0.062	1.7E-04	X
G1SIJ7	Q9NR30	DDX21	Uncharacterized protein	Nucleolar RNA helicase 2	3	89	4	2.192	0.099	2.8E-04	X
G1SNS1	Q14692	BMS1	Bms1-type G domain-containing protein	Ribosome biogenesis protein BMS1 homolog	2	88	4	2.190	0.071	1.9E-04	X
	H0Y6E7	RBMX		RNA-binding motif protein, X chromosome (Fragment)	4		4	2.173	0.050	1.7E-04	X
G1T1P3	Q9H845	ACAD9	Uncharacterized protein	Acyl-CoA dehydrogenase family member 9, mitochondrial	3	88	4	2.164	0.048	1.7E-04	X
G1T594	G0XQ39	STIM1	Stromal interaction molecule 1	STIM1L	2	98	4	2.152	0.175	1.5E-03	X
G1SL97	Q15397	PUM3	PUM-HD domain-containing protein	Pumilio homolog 3	2	91	4	2.151	0.109	3.6E-04	X
G1SZ44	B8ZZL8	HSPE1	Uncharacterized protein	10 kDa heat shock protein, mitochondrial	3	100	4	2.131	0.224	3.9E-03	X
G1TB57		MRPS35	MRP-S28 domain-containing protein		1		4	2.122	0.077	2.3E-04	X
G1TL80	Q9Y3B4	SF3B6	RRM domain-containing protein	Splicing factor 3B subunit 6	2	100	4	2.120	0.090	2.8E-04	X
G1TKC9	Q15582	TGFBI	Transforming growth factor-beta-induced protein ig-h3	Transforming growth factor-beta-induced protein ig-h3	2	93	4	2.120	0.181	1.8E-03	X
G1SG11		COX4I1	Cytochrome c oxidase subunit 4 isoform 1, mitochondrial		1		4	2.112	0.098	3.3E-04	X
G1SGP1	P31930	UQCRC1	Uncharacterized protein	Cytochrome b-c1 complex subunit 1, mitochondrial	3	93	4	2.111	0.142	8.1E-04	X
G1SLK6	J3KMX2	SMARCD2	SWIB domain-containing protein	SWI/SNF-related matrix-associated actin-dependent regulator of chromatin subfamily D member 2	2	98	4	2.106	0.023	1.7E-04	X
G1TG89	P62244	RPS15A	Uncharacterized protein	40S ribosomal protein S15a	3	100	4	2.104	0.207	3.1E-03	X
G1SXN0		PES1	Pescadillo homolog		1		4	2.103	0.140	8.0E-04	X
G1TV19	H3BPE7	FUS	FUS RNA binding protein	RNA-binding protein FUS	2	80	4	2.102	0.085	2.6E-04	X
G1T8M9	P42704	LRPPRC	PPR_long domain-containing protein	Leucine-rich PPR motif-containing protein, mitochondrial	2	81	4	2.101	0.014	1.7E-04	X
G1TMU1	Q13151	HNRNPA0	Uncharacterized protein	Heterogeneous nuclear ribonucleoprotein A0	3	78	4	2.096	0.093	3.1E-04	X
G1STC6		WDR3	WD_REPEATS_REGION domain-containing protein		1		4	2.095	0.067	2.0E-04	X

G1T4L3	Q01780	EXOSC10	HRDC domain-containing protein	Exosome component 10	2	89	4	2.088	0.023	1.7E-04	X
G1TZB9	Q13595	TRA2A	RRM domain-containing protein	Transformer-2 protein homolog alpha	2	100	4	2.084	0.038	1.7E-04	X
G1TAR0		RPF2	Brix domain-containing protein		1		4	2.079	0.065	2.0E-04	X
G1SSJ7	P35232	PHB	PHB domain-containing protein	Prohibitin	2	100	4	2.076	0.038	1.7E-04	X
G1SPY7	O14807	MRAS	Uncharacterized protein	Ras-related protein M-Ras	3	100	4	2.071	0.049	1.7E-04	X
G1T9U7	Q8TD30	GPT2	Glutamic--pyruvic transaminase 2	Alanine aminotransferase 2	2	97	4	2.068	0.039	1.7E-04	X
G1TID3		ITPRIP	Inositol 1,4,5-trisphosphate receptor interacting protein		1		4	2.067	0.071	2.3E-04	X
G1TE69	J3KTL2	SRSF1	Uncharacterized protein	Serine/arginine-rich-splicing factor 1	3	100	4	2.067	0.040	1.7E-04	X
G1SZE8		UTP20	DRIM domain-containing protein		1		4	2.066	0.079	2.5E-04	X
G1T9F6		NDUFA6	NADH:ubiquinone oxidoreductase subunit A6		1		4	2.062	0.118	5.4E-04	X
G1TXA3	P82921	MRPS21	Uncharacterized protein	28S ribosomal protein S21, mitochondrial	3	93	4	2.058	0.083	2.8E-04	X
G1SN06	O60488	ACSL4	AMP-binding domain-containing protein	Long-chain-fatty-acid--CoA ligase 4	2	98	4	2.057	0.043	1.7E-04	X
P98049		MT-CO2	Cytochrome c oxidase subunit 2		1		4	2.056	0.202	3.3E-03	X
	I3L3B0	C1QBP		Complement component 1 Q subcomponent-binding protein, mitochondrial	4		4	2.055	0.106	4.1E-04	X
P35953	P98155	VLDLR	Very low-density lipoprotein receptor	Very low-density lipoprotein receptor	2	97	4	2.046	0.077	2.6E-04	X
G1SF56		MICAL2	Microtubule associated monooxygenase, calponin and LIM domain containing 2		1		4	2.043	0.128	7.2E-04	X
G1TI40	P62316	SNRPD2	Small nuclear ribonucleoprotein Sm D2	Small nuclear ribonucleoprotein Sm D2	2	100	4	2.043	0.037	1.7E-04	X
G1SXR7		SLIRP	RRM domain-containing protein		1		4	2.039	0.034	1.7E-04	X
	Q9Y2Z2	MTO1		Protein MTO1 homolog, mitochondrial	4		4	2.037	0.072	2.5E-04	X
G1TPN3	D6R9P3	HNRNPAB	Uncharacterized protein	Heterogeneous nuclear ribonucleoprotein A/B	3	96	4	2.032	0.087	3.1E-04	X
G1TZQ6		NDUFA10	NADH dehydrogenase [ubiquinone] 1 alpha subcomplex subunit 10, mitochondrial		1		4	2.029	0.159	1.5E-03	X
G1T0I5	Q5JRX3	PITRM1	M16C_associated domain-containing protein	Presequence protease, mitochondrial	2	89	4	2.024	0.093	3.5E-04	X
G1TRL8	J3QLI9	SNRPD1	Small nuclear ribonucleoprotein Sm D1	Small nuclear ribonucleoprotein Sm D1	2	100	4	2.023	0.020	1.7E-04	X
G1SXW0	P43304	GPD2	Glycerol-3-phosphate dehydrogenase	Glycerol-3-phosphate dehydrogenase, mitochondrial	2	96	4	2.020	0.090	3.3E-04	X
U3KMH9	P40926	MDH2	Malate dehydrogenase	Malate dehydrogenase, mitochondrial	2	94	4	2.018	0.093	3.5E-04	X
G1SE36	R4GMU1	H6PD	GDH/6PGL endoplasmic bifunctional protein	GDH/6PGL endoplasmic bifunctional protein	2	85	4	2.013	0.030	1.7E-04	X
G1SKI8	Q6DKI1	RPL7L1	Uncharacterized protein	60S ribosomal protein L7-like 1	3	85	4	2.004	0.057	2.1E-04	X
G1TBV4	Q14566	MCM6	DNA helicase	DNA replication licensing factor MCM6	2	97	3	1.998	0.050	4.1E-04	X
G1SUY2	P05091	ALDH2	Aldedh domain-containing protein	Aldehyde dehydrogenase, mitochondrial	2	91	4	1.998	0.114	6.0E-04	X
G1SIM3	O95831	AIFM1	Uncharacterized protein	Apoptosis-inducing factor 1, mitochondrial	3	96	4	1.996	0.058	2.2E-04	X
G1SLK4	Q8WVX9	FAR1	Fatty acyl-CoA reductase	Fatty acyl-CoA reductase 1	2	93	4	1.996	0.121	7.0E-04	X
	Q10570	CPSF1		Cleavage and polyadenylation specificity factor subunit 1	4		4	1.994	0.119	6.8E-04	X
G1SIF2	Q16822	PCK2	Uncharacterized protein	Phosphoenolpyruvate carboxykinase [GTP], mitochondrial	3	95	4	1.988	0.033	1.7E-04	X
G1TA04	Q9UMS4	PRPF19	Uncharacterized protein	Pre-mRNA-processing factor 19	3	96	4	1.986	0.024	1.7E-04	X
G1SJ30		WDR43	WD_REPEATS_REGION domain-containing protein		1		4	1.986	0.122	7.6E-04	X
	H0Y2W2	ATAD3A		ATPase family AAA domain-containing protein 3A (Fragment)	4		4	1.985	0.026	1.7E-04	X
G1T1B6	Q16832	DDR2	Uncharacterized protein	Discoidin domain-containing receptor 2	3	97	4	1.981	0.151	1.5E-03	X
G1SZ76	P05455	SSB	Lupus La protein homolog	Lupus La protein	2	92	4	1.980	0.057	2.2E-04	X
	P38919	EIF4A3		Eukaryotic initiation factor 4A-III	4		4	1.979	0.025	1.7E-04	X
G1SD44	Q13505	MTX1	Uncharacterized protein	Metaxin-1	3	84	4	1.976	0.040	1.9E-04	X
G1TVK1		NOTCH2	Notch 2		1		4	1.972	0.252	1.0E-02	X
G1T7H4	Q8TDN6	BRIX1	Brix domain-containing protein	Ribosome biogenesis protein BRX1 homolog	2	93	4	1.969	0.023	1.7E-04	X
G1TWL0	P22626	HNRNPA2B1	Uncharacterized protein	Heterogeneous nuclear ribonucleoproteins A2/B1	3	99	4	1.967	0.031	1.7E-04	X
G1U5U0	Q9Y2X3	NOP58	Nop domain-containing protein	Nucleolar protein 58	2	92	4	1.962	0.066	2.5E-04	X
G1SUQ9	P11387	TOP1	DNA topoisomerase I	DNA topoisomerase 1	2	97	4	1.957	0.021	1.7E-04	X
G1TU85		FADS3	Cytochrome b5 heme-binding domain-containing protein		1		4	1.956	0.080	3.3E-04	X
G1TNV7	O75600	GCAT	Glycine C-acetyltransferase	2-amino-3-ketobutyrate coenzyme A ligase, mitochondrial	2	94	4	1.953	0.044	2.0E-04	X
G1SLR8	Q9NZI8	IGF2BP1	Uncharacterized protein	Insulin-like growth factor 2 mRNA-binding protein 1	3	99	4	1.944	0.067	2.8E-04	X
G1U018		IGF2R	Insulin like growth factor 2 receptor		1		4	1.941	0.040	1.9E-04	X
G1SL60	Q15393	SF3B3	CPSF_A domain-containing protein	Splicing factor 3B subunit 3	2	98	4	1.933	0.035	1.9E-04	X
G1SR13	Q9UJZ1	STOML2	PHB domain-containing protein	Stomatin-like protein 2, mitochondrial	2	96	4	1.932	0.053	2.3E-04	X
P12345	P00505	GOT2	Aspartate aminotransferase, mitochondrial	Aspartate aminotransferase, mitochondrial	2	94	4	1.928	0.076	3.3E-04	X
G1TKJ4	P61601	NCALD	Uncharacterized protein	Neurocalcin-delta	3	100	4	1.925	0.034	1.9E-04	X
G1SCY8	Q9UKD2	MRTO4	Ribosome assembly factor mrt4	mRNA turnover protein 4 homolog	2	96	4	1.923	0.122	9.3E-04	X
G1SXC8		NXF1	Nuclear RNA export factor 1		1		4	1.920	0.034	1.9E-04	X
	Q13247	SRSF6		Serine/arginine-rich splicing factor 6	4		4	1.919	0.042	2.0E-04	X
G1SMY1	A0A1W2PQ51	DDX17	Uncharacterized protein	Probable ATP-dependent RNA helicase DDX17	3	99	4	1.918	0.027	1.9E-04	X
G1TDQ3	J3QT28	BUB3	WD_REPEATS_REGION domain-containing protein	Mitotic checkpoint protein BUB3 (Fragment)	2	99	4	1.917	0.043	2.1E-04	X
G1TQJ5		DEK	SAP domain-containing protein		1		4	1.915	0.041	2.0E-04	X
G1SF95	H0Y8G5	HNRNPD	Heterogeneous nuclear ribonucleoprotein D	Heterogeneous nuclear ribonucleoprotein D0 (Fragment)	2	99	4	1.912	0.061	2.7E-04	X
G1SEF1		NDUFC2	NADH dehydrogenase [ubiquinone] 1 subunit C2		1		4	1.905	0.190	4.3E-03	X
G1SKM2	P35555	FBN1	Uncharacterized protein	Fibrillin-1	3	97	4	1.905	0.111	7.5E-04	X
G1SL16	A0A0B4J1Z1	SRSF7	Uncharacterized protein	Serine/arginine-rich-splicing factor 7	3	100	4	1.904	0.031	1.9E-04	X
	P02458	COL2A1		Collagen alpha-1(II) chain	4		4	1.900	0.285	2.1E-02	X
G1THR4		GADD45GIP1	GADD45G interacting protein 1		1		3	1.898	0.095	1.1E-03	X
G1TC03	Q13263	TRIM28	Tripartite motif containing 28	Transcription intermediary factor 1-beta	2	96	4	1.896	0.063	2.8E-04	X
G1SI76		MRPL43	L51_S25_CI-B8 domain-containing protein		1		4	1.896	0.043	2.2E-04	X
G1ST81	Q9P035	HACD3	Very-long-chain (3R)-3-hydroxyacyl-CoA dehydratase	Very-long-chain (3R)-3-hydroxyacyl-CoA dehydratase 3	2	96	4	1.895	0.055	2.5E-04	X
	A0A087X2B1	RBFOX1		RNA binding protein fox-1 homolog	4		4	1.891	0.162	2.6E-03	X

G1SJY0		MRPS5	S5 DRBM domain-containing protein		1		4	1.890	0.066	3.0E-04	X
G1T1L7	A0A087WWS1	THOC1	Death domain-containing protein	THO complex subunit 1	2	98	4	1.888	0.128	1.2E-03	X
G1SPY4		ASRGL1	Asparaginase like 1		1		4	1.886	0.084	4.0E-04	X
G1SXI9		COX6B1	Cytochrome c oxidase subunit		1		4	1.885	0.138	1.6E-03	X
G1SZK8	P41223	BUD31	Uncharacterized protein	Protein BUD31 homolog	3	100	4	1.881	0.050	2.5E-04	X
G1U2E6	J3KPX7	PHB2	PHB domain-containing protein	Prohibitin-2	2	99	4	1.880	0.045	2.3E-04	X
	Q9NTI5-2	PDS5B		Isoform 2 of Sister chromatid cohesion protein PDS5 homolog B	4		4	1.879	0.051	2.5E-04	X
G1SYC5	Q9NQZ2	UTP3	Sas10 domain-containing protein	Something about silencing protein 10	2	82	4	1.879	0.088	4.7E-04	X
	Q9BXP5	SRRT		Serrate RNA effector molecule homolog	4		4	1.878	0.036	2.1E-04	X
	P31040	SDHA		Succinate dehydrogenase [ubiquinone] flavoprotein subunit, mitochondrial	4		4	1.872	0.069	3.3E-04	X
G1T8P3	P08621	SNRNP70	Small nuclear ribonucleoprotein U1 subunit 70	U1 small nuclear ribonucleoprotein 70 kDa	2	92	4	1.871	0.036	2.1E-04	X
G1TB02	E7EPS8	PTPRM	Uncharacterized protein	Receptor-type tyrosine-protein phosphatase mu	3	98	4	1.871	0.078	3.7E-04	X
G1TEN4	Q9HD33	MRPL47	Uncharacterized protein	39S ribosomal protein L47, mitochondrial	3	79	4	1.869	0.045	2.4E-04	X
G1SM50	Q15427	SF3B4	Uncharacterized protein	Splicing factor 3B subunit 4	3	100	4	1.869	0.050	2.5E-04	X
G1SUA4	J3KS05	CBX1	Uncharacterized protein	Chromobox protein homolog 1 (Fragment)	3	98	4	1.868	0.025	1.9E-04	X
G1SRF7	P38646	HSPA9	Uncharacterized protein	Stress-70 protein, mitochondrial	3	99	4	1.861	0.049	2.5E-04	X
G1SWD1	M0QXL5	FBL	Fibrillarin	rRNA 2~-O-methyltransferase fibrillarin (Fragment)	2	97	4	1.855	0.118	1.1E-03	X
G1SPW1	Q29RF7	PDS5A	Uncharacterized protein	Sister chromatid cohesion protein PDS5 homolog A	3	99	4	1.853	0.057	2.8E-04	X
G1T359	P28331	NDUFS1	Uncharacterized protein	NADH-ubiquinone oxidoreductase 75 kDa subunit, mitochondrial	3	98	4	1.850	0.081	4.4E-04	X
G1TES6	Q99714	HSD17B10	Uncharacterized protein	3-hydroxyacyl-CoA dehydrogenase type-2	3	92	4	1.849	0.054	2.8E-04	X
G1TZN7		COX5A	Cytochrome c oxidase subunit 5A		1		4	1.848	0.040	2.3E-04	X
G1TM60		NDUFA9	Epimerase domain-containing protein		1		4	1.844	0.094	6.2E-04	X
G1TBS4	P20020	ATP2B1	Calcium-transporting ATPase	Plasma membrane calcium-transporting ATPase 1	2	97	4	1.842	0.112	1.0E-03	X
G1SVK5	P26447	S100A4	Protein S100	Protein S100-A4	2	98	4	1.842	0.291	2.7E-02	X
G1T8H6	K7EK07	H3F3B	Histone H3	Histone H3 (Fragment)	2	98	4	1.840	0.099	7.3E-04	X
G1T2A9	Q14315	FLNC	Uncharacterized protein	Filamin-C	3	90	4	1.840	0.045	2.5E-04	X
	K7EKE6	LONP1		Lon protease homolog, mitochondrial	4		4	1.834	0.062	3.3E-04	X
G1TTM6	Q99797	MIPEP	Peptidase_M3 domain-containing protein	Mitochondrial intermediate peptidase	2	93	4	1.831	0.072	3.7E-04	X
G1SGC2	P0C0S5	H2AFZ	Histone H2A	Histone H2A.Z	2	98	4	1.830	0.093	6.3E-04	X
G1SHL0		SBNO1	Strawberry notch homolog 1		1		4	1.823	0.030	2.2E-04	X
G1T0U4	Q9UIG0	BAZ1B	Bromodomain adjacent to zinc finger domain 1B	Tyrosine-protein kinase BAZ1B	2	94	4	1.822	0.086	5.6E-04	X
G1SIW1	O43143	DHX15	Uncharacterized protein	Pre-mRNA-splicing factor ATP-dependent RNA helicase DHX15	3	99	4	1.818	0.026	2.2E-04	X
G1SFE6	P08579	SNRPB2	Uncharacterized protein	U2 small nuclear ribonucleoprotein B~~	3	96	4	1.817	0.033	2.3E-04	X
G1SPF1	Q5JTH9	RRP12	NUC173 domain-containing protein	RRP12-like protein	2	92	4	1.817	0.065	3.6E-04	X
G1SP02	A8MT40	PDPR	Uncharacterized protein	Pyruvate dehydrogenase phosphatase regulatory subunit, mitochondrial	3	95	4	1.812	0.066	3.7E-04	X
G1ST15	Q13825	AUH	AU RNA binding methylglutaconyl-CoA hydratase	Methylglutaconyl-CoA hydratase, mitochondrial	2	95	4	1.811	0.102	8.5E-04	X
G1SE30		EPS8	SH3 domain-containing protein		1		4	1.811	0.152	2.8E-03	X
G1THH7		SUN2	SUN domain-containing protein		1		4	1.811	0.067	3.7E-04	X
G1TSS7	P05106	ITGB3	Integrin beta	Integrin beta-3	2	95	4	1.810	0.150	2.7E-03	X
G1SSH0	A0A087WUB9	CTNNBL1	DUF1716 domain-containing protein	Beta-catenin-like protein 1	2	96	4	1.806	0.049	2.8E-04	X
G1SS51		MRPS25	L51_S25_CI-B8 domain-containing protein		1		4	1.802	0.063	3.6E-04	X
G1TMU2	P52597	HNRNPF	Uncharacterized protein	Heterogeneous nuclear ribonucleoprotein F	3	99	4	1.801	0.021	2.2E-04	X
	O96008	TOMM40		Mitochondrial import receptor subunit TOM40 homolog	4		4	1.799	0.056	3.3E-04	X
	Q9Y4W6	AFG3L2		AFG3-like protein 2	4		4	1.796	0.065	3.7E-04	X
G1TE50	Q9Y221	NIP7	60S ribosome subunit biogenesis protein NIP7 homolog	60S ribosome subunit biogenesis protein NIP7 homolog	2	97	4	1.796	0.019	2.2E-04	X
G1U862	Q01650	SLC7A5	Large neutral amino acids transporter small subunit 1	Large neutral amino acids transporter small subunit 1	2	94	4	1.792	0.045	2.8E-04	X
G1TD99	A0A1B0GUX9	PCCA	Uncharacterized protein	Propionyl-CoA carboxylase alpha chain, mitochondrial (Fragment)	3	96	4	1.792	0.069	4.0E-04	X
G1U1X6		NT5C3A	5~-nucleotidase		1		4	1.792	0.031	2.5E-04	X
G1SNF2	P13995	MTHFD2	Uncharacterized protein	Bifunctional methylenetetrahydrofolate dehydrogenase/cyclohydrolase, mitochondrial	3	93	4	1.791	0.124	1.6E-03	X
G1SFQ3		MRPL2	Ribosomal_L2_C domain-containing protein		1		4	1.791	0.063	3.7E-04	X
G1TBR6	Q69YN4	VIRMA	VIR_N domain-containing protein	Protein virilizer homolog	2	98	4	1.790	0.046	2.8E-04	X
G1TYH7	Q9HDC9	APMAP	Adipocyte plasma membrane associated protein	Adipocyte plasma membrane-associated protein	2	94	4	1.782	0.095	8.1E-04	X
G1SWA6	A0A2R8Y4T4	SMARCE1	HMG box domain-containing protein	SWI/SNF-related matrix-associated actin-dependent regulator of chromatin subfamily E member 1	2	95	4	1.781	0.076	5.1E-04	X
G1SIP2	A0A3B3ITJ4	HNRNPL	Uncharacterized protein	Heterogeneous nuclear ribonucleoprotein L (Fragment)	3	93	4	1.780	0.057	3.5E-04	X
G1TCA0	Q96KR1	ZFR	DZF domain-containing protein	Zinc finger RNA-binding protein	2	99	4	1.779	0.134	2.1E-03	X
G1T120	S4R369	MRPL37	Uncharacterized protein	39S ribosomal protein L37, mitochondrial	3	84	4	1.777	0.044	2.8E-04	X
G1STI3	Q07666	KHDRBS1	KH domain-containing protein	KH domain-containing, RNA-binding, signal transduction-associated protein 1	2	99	4	1.777	0.021	2.3E-04	X
G1TRS0		DKC1	PUA domain-containing protein		1		4	1.773	0.101	1.0E-03	X
G1T890	Q8NE86	MCU	MCU domain-containing protein	Calcium uniporter protein, mitochondrial	2	98	4	1.771	0.049	3.2E-04	X
	Q08945	SSRP1		FACT complex subunit SSRP1	4		4	1.769	0.097	9.3E-04	X
G1SUR4		ZADH2	PKS_ER domain-containing protein		1		3	1.769	0.025	7.3E-04	X
G1SKQ8	Q13601	KRR1	KRR1 small subunit processome component	KRR1 small subunit processome component homolog	2	95	4	1.767	0.060	3.7E-04	X
G1SMM7	P62318	SNRPD3	Small nuclear ribonucleoprotein Sm D3	Small nuclear ribonucleoprotein Sm D3	2	100	4	1.765	0.027	2.5E-04	X
G1TX03	A0A0J9YW13	RBM8A	RNA-binding protein 8A	RNA-binding protein 8A (Fragment)	2	100	4	1.762	0.032	2.7E-04	X
	P26368	U2AF2		Splicing factor U2AF 65 kDa subunit	4		4	1.762	0.068	4.5E-04	X
G1SQR1	Q93096	PTP4A1	TYR_PHOSPHATASE_2 domain-containing protein	Protein tyrosine phosphatase type IVA 1	2	100	4	1.761	0.136	2.5E-03	X
G1SUP4	J3QRS9	ZNF207	Uncharacterized protein	BUB3-interacting and GLEBS motif-containing protein ZNF207	3	100	4	1.760	0.034	2.8E-04	X
G1SY30	O75643	SNRNP200	Uncharacterized protein	U5 small nuclear ribonucleoprotein 200 kDa helicase	3	100	4	1.759	0.024	2.5E-04	X
G1T116	Q96TA2	YME1L1	AAA domain-containing protein	ATP-dependent zinc metalloprotease YME1L1	2	89	4	1.758	0.015	2.4E-04	X

G1TJW3	Q15233	NONO	Uncharacterized protein	Non-POU domain-containing octamer-binding protein	3	99	4	1.758	0.077	5.8E-04	X
U3KPI1		MPHOSPH10	U3 small nucleolar ribonucleoprotein protein MPP10		1		3	1.758	0.121	2.7E-03	X
G1T6I6	Q12906	ILF3	Interleukin enhancer binding factor 3	Interleukin enhancer-binding factor 3	2	96	4	1.755	0.053	3.5E-04	X
G1T5I9	F8WJN3	CPSF6	RRM domain-containing protein	Cleavage and polyadenylation-specificity factor subunit 6	2	99	4	1.754	0.067	4.5E-04	X
G1TGK9	Q08211	DHX9	Uncharacterized protein	ATP-dependent RNA helicase A	3	93	4	1.754	0.020	2.5E-04	X
	Q9NTZ6	RBM12		RNA-binding protein 12	4		4	1.754	0.056	3.7E-04	X
G1SDP7	H0Y8P4	UTP15	UTP15, small subunit processome component	U3 small nucleolar RNA-associated protein 15 homolog (Fragment)	2	92	4	1.753	0.048	3.3E-04	X
G1TC48	A0A0A0MRA5	HNRNPUL1	Uncharacterized protein	Heterogeneous nuclear ribonucleoprotein U-like protein 1	3	95	4	1.752	0.034	2.8E-04	X
G1SNV4	Q12874	SF3A3	Matrin-type domain-containing protein	Splicing factor 3A subunit 3	2	100	4	1.752	0.025	2.5E-04	X
G1T3N1	Q9NVP1	DDX18	RNA helicase	ATP-dependent RNA helicase DDX18	2	87	4	1.749	0.055	3.7E-04	X
G1SJ32	A0A0A0MTB8	WDR36	WD_REPEATS_REGION domain-containing protein	WD repeat-containing protein 36	2	94	4	1.744	0.043	3.2E-04	X
G1SU13	Q5VW52	GPAM	Glycerol-3-phosphate acyltransferase 1, mitochondrial	Glycerol-3-phosphate acyltransferase 1, mitochondrial	2	94	4	1.742	0.059	4.0E-04	X
G1U450		XYLB	Xylulokinase		1		4	1.739	0.208	1.2E-02	X
G1SKK1		DUT	dUTPase domain-containing protein		1		4	1.738	0.111	1.5E-03	X
	F6RGN5	SLC25A10		Mitochondrial dicarboxylate carrier	4		4	1.737	0.087	8.2E-04	X
G1TIR7	O95777	LSM8	U6 snRNA-associated Sm-like protein LSm8	U6 snRNA-associated Sm-like protein LSm8	2	99	4	1.736	0.032	2.8E-04	X
G1T7B5	Q9H0S4	DDX47	Uncharacterized protein	Probable ATP-dependent RNA helicase DDX47	3	97	4	1.734	0.073	5.9E-04	X
G1SDX3	E9PCY5	TOP2B	DNA topoisomerase 2	DNA topoisomerase 2 (Fragment)	2	99	4	1.733	0.032	2.8E-04	X
G1SSF2		ENG	Endoglin		1		4	1.730	0.037	3.1E-04	X
G1SW10	O94776	MTA2	Uncharacterized protein	Metastasis-associated protein MTA2	3	99	4	1.729	0.056	3.9E-04	X
O79428		MT-ND2	NADH-ubiquinone oxidoreductase chain 2		1		3	1.728	0.096	2.0E-03	X
G1SIQ9	Q9GZL7	WDR12	Ribosome biogenesis protein WDR12	Ribosome biogenesis protein WDR12	2	97	4	1.726	0.065	5.0E-04	X
G1U5M7	A0A3B3IUA2	SNU13	Ribonucleoprotein	Ribonucleoprotein	2	100	4	1.723	0.098	1.2E-03	X
G1SR36	C9JG87	MRPL39	Uncharacterized protein	39S ribosomal protein L39, mitochondrial (Fragment)	3	86	4	1.723	0.052	3.7E-04	X
G1TAH7	P29401	TKT	TRANSKETOLASE_1 domain-containing protein	Transketolase	2	94	4	1.721	0.135	2.9E-03	X
	A0A0C4DFX9	NELFA		Negative elongation factor A	4		4	1.718	0.074	6.5E-04	X
G1SYC1	P83111	LACTB	Uncharacterized protein	Serine beta-lactamase-like protein LACTB, mitochondrial	3	89	4	1.714	0.112	1.7E-03	X
G1SGH2		MRPL15	Ribosomal_L18e/L15P domain-containing protein		1		4	1.713	0.097	1.2E-03	X
G1SMI2	Q9Y305	ACOT9	Acyl-CoA thioesterase 9	Acyl-coenzyme A thioesterase 9, mitochondrial	2	83	4	1.713	0.050	3.7E-04	X
G1T3X2	H7BZW6	SAP18	Histone deacetylase complex subunit SAP18	Histone deacetylase complex subunit SAP18 (Fragment)	2	98	4	1.712	0.033	3.1E-04	X
	Q9P0J1	PDP1		[Pyruvate dehydrogenase [acetyl-transferring]]-phosphatase 1, mitochondrial	4		4	1.712	0.055	4.2E-04	X
G1SWF3		DHODH	Dihydroorotate dehydrogenase (quinone), mitochondrial		1		4	1.711	0.056	4.3E-04	X
G1T011	Q9Y5B9	SUPT16H	Uncharacterized protein	FACT complex subunit SPT16	3	100	4	1.710	0.022	2.8E-04	X
G1SLV3	P42285	MTREX	Uncharacterized protein	Exosome RNA helicase MTR4	3	99	4	1.710	0.025	2.8E-04	X
G1TNT7		DDX27	DEAD-box helicase 27		1		4	1.706	0.170	6.7E-03	X
G1U5D4	F5GY32	PUS1	tRNA pseudouridine synthase	tRNA pseudouridine synthase A (Fragment)	2	78	3	1.706	0.099	2.4E-03	X
	P35268	RPL22		60S ribosomal protein L22	4		3	1.703	0.154	5.9E-03	X
G1T2U6	A0A0C4DG89	DDX46	Uncharacterized protein	Probable ATP-dependent RNA helicase DDX46	3	99	4	1.703	0.037	3.3E-04	X
G1SFH4		MRPL24	KOW domain-containing protein		1		4	1.703	0.057	4.5E-04	X
G1TEI2		FDX1	2Fe-2S ferredoxin-type domain-containing protein		1		4	1.702	0.090	1.1E-03	X
	P43897	TSFM		Elongation factor Ts, mitochondrial	4		4	1.702	0.043	3.7E-04	X
G1T5H0	A0A3B3IU24	HTRA1	PDZ domain-containing protein	Serine protease HTRA1	2	94	4	1.694	0.127	2.7E-03	X
G1SR61	H7BXY3	DHX30	Uncharacterized protein	ATP-dependent RNA helicase DHX30	3	98	4	1.694	0.037	3.5E-04	X
G1SMI7	V9GYL9	DAP3	Uncharacterized protein	28S ribosomal protein S29, mitochondrial (Fragment)	3	86	4	1.692	0.048	4.0E-04	X
G1T701		DBT	Dihydrolipoamide acetyltransferase component of pyruvate dehydrogenase complex		1		4	1.691	0.092	1.2E-03	X
G1U9B4	P62995	TRA2B	RRM domain-containing protein	Transformer-2 protein homolog beta	2	100	4	1.691	0.053	4.4E-04	X
G1SQ11	P54886	ALDH18A1	Delta-1-pyrroline-5-carboxylate synthase	Delta-1-pyrroline-5-carboxylate synthase	2	97	4	1.691	0.039	3.6E-04	X
G1SRF1	J3KT10	NUP85	Nuclear pore complex protein Nup85	Nuclear pore complex protein Nup85	2	84	4	1.691	0.018	2.8E-04	X
G1SSK8	B4DJV2	CS	Citrate synthase	Citrate synthase	2	96	4	1.690	0.080	8.5E-04	X
G1SEM4		THADA	DUF2428 domain-containing protein		1		3	1.689	0.079	1.9E-03	X
	Q15428	SF3A2		Splicing factor 3A subunit 2	4		4	1.688	0.066	6.1E-04	X
G1T6L0	Q5T3Q7	HEATR1	BP28CT domain-containing protein	HEAT repeat-containing protein 1	2	93	4	1.686	0.068	6.4E-04	X
G1SCR7	E9PF10	NUP155	Uncharacterized protein	Nuclear pore complex protein Nup155	3	90	4	1.685	0.053	4.5E-04	X
G1SGY8	Q3ZCQ8	TIMM50	Mitochondrial import inner membrane translocase subunit TIM50	Mitochondrial import inner membrane translocase subunit TIM50	2	96	4	1.685	0.032	3.3E-04	X
	Q8WX92	NELFB		Negative elongation factor B	4		4	1.684	0.040	3.7E-04	X
G1T8F7	A0A494C128	NOP56	Nop domain-containing protein	Nucleolar protein 56	2	98	4	1.679	0.077	8.5E-04	X
G1SI37	D6RF87	ACSF2	Uncharacterized protein	Acyl-CoA synthetase family member 2, mitochondrial (Fragment)	3	82	4	1.677	0.135	3.6E-03	X
G1TAE2	Q15717	ELAVL1	ELAV-like protein	ELAV-like protein 1	2	99	4	1.677	0.040	3.7E-04	X
G1TH59	Q14498	RBM39	Uncharacterized protein	RNA-binding protein 39	3	91	4	1.672	0.029	3.4E-04	X
G1SQB1	P22033	MMUT	B12-binding domain-containing protein	Methylmalonyl-CoA mutase, mitochondrial	2	96	4	1.670	0.139	4.1E-03	X
G1SRH7	P84103	SRSF3	RRM domain-containing protein	Serine/arginine-rich splicing factor 3	2	100	4	1.669	0.043	4.0E-04	X
G1TCU1	I3L2K5	ZC3H7A	Uncharacterized protein	Zinc finger CCCH domain-containing protein 7A (Fragment)	3	95	4	1.669	0.045	4.1E-04	X
G1SZA1	B4DHE8	MSI2	Uncharacterized protein	RNA-binding protein Musashi homolog 2	3	94	4	1.668	0.071	7.7E-04	X
G1SKW4		KIDINS220	Kinase D interacting substrate 220		1		4	1.667	0.126	3.0E-03	X
G1TDQ1	Q9H0C8	ILKAP	PPM-type phosphatase domain-containing protein	Integrin-linked kinase-associated serine/threonine phosphatase 2C	2	94	4	1.665	0.093	1.4E-03	X
G1T5H2	Q9H0D6	XRN2	5~-3~ exoribonuclease	5~-3~ exoribonuclease 2	2	98	4	1.665	0.044	4.1E-04	X
G1T5A2	P08648	ITGA5	Integrin_alpha2 domain-containing protein	Integrin alpha-5	2	91	4	1.665	0.095	1.4E-03	X
G1SWU9		FAM20B	Fam20C domain-containing protein		1		4	1.663	0.098	1.6E-03	X
G1SHV1	Q9Y333	LSM2	U6 snRNA-associated Sm-like protein LSm2	U6 snRNA-associated Sm-like protein LSm2	2	100	4	1.663	0.054	5.3E-04	X

	K7EIE8	MBD3		Methyl-CpG binding domain protein 3, isoform CRA_b	4		4	1.662	0.046	4.4E-04	X
G1TS78	A0A075B6F6	HM13	Uncharacterized protein	Minor histocompatibility antigen H13 (Fragment)	3	84	4	1.662	0.125	3.0E-03	X
G1SQ52	P52788	SMS	PABS domain-containing protein	Spermine synthase	2	99	4	1.659	0.190	1.3E-02	X
G1SME4	Q9BWF3	RBM4	RNA-binding protein 4	RNA-binding protein 4	2	99	4	1.659	0.049	4.7E-04	X
G1T2Y5	P12270	TPR	TPR_MLP1_2 domain-containing protein	Nucleoprotein TPR	2	97	4	1.659	0.039	3.9E-04	X
G1SP32	Q6NVY1	HIBCH	3-hydroxyisobutyryl-CoA hydrolase, mitochondrial	3-hydroxyisobutyryl-CoA hydrolase, mitochondrial	2	84	4	1.658	0.115	2.5E-03	X
G1SJI6		MRPS23	MRP-S23 domain-containing protein		1		4	1.656	0.008	3.1E-04	X
G1SFH9	A0A2R8Y543	CTNNB1	Uncharacterized protein	Catenin beta-1	3	100	4	1.655	0.060	6.3E-04	X
	O95202	LETM1		Mitochondrial proton/calcium exchanger protein	4		4	1.653	0.078	1.0E-03	X
G1T696	Q09161	NCBP1	MIF4G domain-containing protein	Nuclear cap-binding protein subunit 1	2	99	4	1.652	0.033	3.7E-04	X
G1TDH4	P30048	PRDX3	Thioredoxin domain-containing protein	Thioredoxin-dependent peroxide reductase, mitochondrial	2	86	4	1.652	0.033	3.7E-04	X
G1T5L3	Q9H9J2	MRPL44	Uncharacterized protein	39S ribosomal protein L44, mitochondrial	3	90	4	1.652	0.045	4.5E-04	X
G1TVG7	O75533	SF3B1	SF3b1 domain-containing protein	Splicing factor 3B subunit 1	2	99	4	1.650	0.020	3.4E-04	X
G1SD24	A0A494BZU6	PARN	R3H domain-containing protein	Poly(A)-specific ribonuclease PARN (Fragment)	2	96	4	1.649	0.046	4.7E-04	X
G1SQU6	O75489	NDUFS3	Complex1_30kDa domain-containing protein	NADH dehydrogenase [ubiquinone] iron-sulfur protein 3, mitochondrial	2	91	4	1.649	0.089	1.4E-03	X
	Q8N201	INTS1		Integrator complex subunit 1	4		4	1.646	0.031	3.7E-04	X
G1T5J9	O75306	NDUFS2	Complex1_49kDa domain-containing protein	NADH dehydrogenase [ubiquinone] iron-sulfur protein 2, mitochondrial	2	94	4	1.644	0.136	4.4E-03	X
G1SLP3	H3BMM9	RNPS1	RNA binding protein with serine rich domain 1	RNA-binding protein with serine-rich domain 1 (Fragment)	2	99	4	1.642	0.079	1.1E-03	X
G1SUS6	D6RD69	SAR1B	Uncharacterized protein	GTP-binding protein SAR1b (Fragment)	3	98	4	1.641	0.139	4.8E-03	X
G1SQ54	A0A087WT44	HMOX2	Heme oxygenase	Heme oxygenase 2	2	89	4	1.640	0.071	9.0E-04	X
Q28888		DCN	Decorin		1		4	1.638	0.183	1.2E-02	X
G1TX78	Q15388	TOMM20	Uncharacterized protein	Mitochondrial import receptor subunit TOM20 homolog	3	100	4	1.637	0.078	1.1E-03	X
G1SRY1	Q8TCS8	PNPT1	S1 motif domain-containing protein	Polyribonucleotide nucleotidyltransferase 1, mitochondrial	2	94	4	1.636	0.058	6.6E-04	X
G1T958		EBP	EXPERA domain-containing protein		1		4	1.635	0.080	1.2E-03	X
	A0A0U1RQF0	FASN		Fatty acid synthase	4		4	1.635	0.074	1.0E-03	X
G1STP6	P35221	CTNNA1	Catenin alpha-1	Catenin alpha-1	2	99	4	1.635	0.054	6.2E-04	X
G1SQB6	Q9NRX1	PNO1	KH domain-containing protein	RNA-binding protein PNO1	2	96	4	1.633	0.133	4.3E-03	X
	Q969V3	NCLN		Nicalin	4		4	1.633	0.143	5.5E-03	X
G1SD25	Q5SRE5	NUP188	Nucleoporin 188	Nucleoporin NUP188 homolog	2	94	4	1.631	0.092	1.6E-03	X
G1SCK0	Q6P2Q9	PRPF8	MPN domain-containing protein	Pre-mRNA-processing-splicing factor 8	2	100	4	1.629	0.044	5.1E-04	X
	A0A087WTP3	KHSRP		Far upstream element-binding protein 2	4		4	1.628	0.062	7.7E-04	X
G1T7V5	E9PEX6	DLD	Dihydrolipoyl dehydrogenase	Dihydrolipoyl dehydrogenase	2	91	4	1.628	0.082	1.3E-03	X
G1SRN1		LAS1L	LAS1 like, ribosome biogenesis factor		1		4	1.628	0.103	2.1E-03	X
G1TTN9	Q9P258	RCC2	Regulator of chromosome condensation 2	Protein RCC2	2	99	4	1.627	0.100	2.0E-03	X
G1T5H5	Q15050	RRS1	Ribosome biogenesis regulatory protein	Ribosome biogenesis regulatory protein homolog	2	92	4	1.626	0.105	2.3E-03	X
	A0A087WVZ9	POLR2E		DNA-directed RNA polymerases I, II, and III subunit RPABC1	4		4	1.625	0.102	2.1E-03	X
G1TA05	A2AE48	TRIM26	Uncharacterized protein	Tripartite motif-containing protein 26 (Fragment)	3	92	4	1.625	0.094	1.7E-03	X
G1T6T8	O94906	PRPF6	Uncharacterized protein	Pre-mRNA-processing factor 6	3	96	4	1.624	0.083	1.4E-03	X
G1STZ4	P11413	G6PD	Glucose-6-phosphate 1-dehydrogenase	Glucose-6-phosphate 1-dehydrogenase	2	94	4	1.624	0.094	1.7E-03	X
G1T9T5	P36957	DLST	Lipoyl-binding domain-containing protein	Dihydrolipoyllysine-residue succinyltransferase component of 2-oxoglutarate dehydrogenase complex, mitochondrial	2	91	4	1.623	0.070	9.6E-04	X
G1SX50		RBM19	RNA binding motif protein 19		1		4	1.622	0.063	8.1E-04	X
G1THY5	Q9Y3C6	PPIL1	Peptidyl-prolyl cis-trans isomerase	Peptidyl-prolyl cis-trans isomerase-like 1	2	100	3	1.621	0.014	1.4E-03	X
G1U6N8	Q14980	NUMA1	Nuclear mitotic apparatus protein 1	Nuclear mitotic apparatus protein 1	2	90	4	1.621	0.069	9.6E-04	X
G1SST6		ARMC10	Arm_2 domain-containing protein		1		4	1.619	0.054	6.6E-04	X
G1SF97		MRPL46	MRP-L46 domain-containing protein		1		4	1.616	0.050	6.2E-04	X
G1STS0	Q9Y2S7	POLDIP2	ApaG domain-containing protein	Polymerase delta-interacting protein 2	2	98	4	1.616	0.026	4.0E-04	X
G1U7I9	A0A0G2JJZ9	DDX39B	Uncharacterized protein	Spliceosome RNA helicase DDX39B (Fragment)	3	95	4	1.607	0.052	6.8E-04	X
G1SGL3		SRSF11	RRM domain-containing protein		1		4	1.605	0.044	5.9E-04	X
G1SLM0	A0A087WTT1	PABPC1	Polyadenylate-binding protein	Polyadenylate-binding protein	2	99	4	1.603	0.079	1.4E-03	X
G1SRX2	Q15029	EFTUD2	Tr-type G domain-containing protein	116 kDa U5 small nuclear ribonucleoprotein component	2	100	4	1.603	0.036	5.0E-04	X
	A0A3B3IRT8	SSR1		Translocon-associated protein subunit alpha	4		4	1.603	0.087	1.7E-03	X
G1T2N4	E7ET15	U2SURP	Uncharacterized protein	U2 snRNP-associated SURP motif-containing protein	3	99	4	1.603	0.039	5.3E-04	X
G1SS37	A0A494C1M4	ALDH1L2	10-formyltetrahydrofolate dehydrogenase	10-formyltetrahydrofolate dehydrogenase	2	96	4	1.602	0.100	2.3E-03	X
	Q9Y5L4	TIMM13		Mitochondrial import inner membrane translocase subunit Tim13	4		4	1.600	0.104	2.6E-03	X
G1SU17		NSDHL	3Beta_HSD domain-containing protein		1		4	1.600	0.075	1.3E-03	X
G1SJG9	Q6P4Q7	CNNM4	Uncharacterized protein	Metal transporter CNNM4	3	94	4	1.599	0.119	3.8E-03	X
	P08574	CYC1		Cytochrome c1, heme protein, mitochondrial	4		4	1.599	0.090	1.8E-03	X
	A0A0C4DGG8	CCAR1		Cell division cycle and apoptosis regulator protein 1 (Fragment)	4		4	1.596	0.091	1.9E-03	X
G1TKC4	Q15046	KARS	AA_TRNA_LIGASE_II domain-containing protein	Lysine--tRNA ligase	2	88	4	1.595	0.101	2.5E-03	X
G1SFD8	Q9BUQ8	DDX23	Uncharacterized protein	Probable ATP-dependent RNA helicase DDX23	3	99	4	1.595	0.043	6.1E-04	X
G1SKZ3	P12004	PCNA	Proliferating cell nuclear antigen	Proliferating cell nuclear antigen	2	99	3	1.593	0.076	2.9E-03	X
G1TAV2		SMCHD1	SMC hinge domain-containing protein		1		4	1.593	0.042	6.0E-04	X
G1T6E6		NOC3L	Nucleolar complex protein 3 homolog		1		4	1.593	0.149	8.0E-03	X
G1SQF9	Q8WXF1	PSPC1	Uncharacterized protein	Paraspeckle component 1	3	98	4	1.593	0.051	7.3E-04	X
G1SIP9	H0Y9G6	MRPL3	Uncharacterized protein	39S ribosomal protein L3, mitochondrial (Fragment)	3	89	4	1.593	0.008	3.9E-04	X
G1T5A6	Q5T4D3	TMTC4	Transmembrane and tetratricopeptide repeat containing 4	Protein O-mannosyl-transferase TMTC4	2	92	4	1.592	0.055	8.0E-04	X
B7NZN9	P51571	SSR4	Signal sequence receptor, delta (Predicted)	Translocon-associated protein subunit delta	2	97	4	1.591	0.063	9.9E-04	X
G1SMH6	Q9P0M6	H2AFY2	Core histone macro-H2A	Core histone macro-H2A.2	2	99	4	1.591	0.098	2.3E-03	X
U3KMD4	Q8IYU8	MICU2	Uncharacterized protein	Calcium uptake protein 2, mitochondrial	3	83	4	1.591	0.175	1.4E-02	X

G1SY50	E7EPT4	NDUFV2	Uncharacterized protein	NADH dehydrogenase [ubiquinone] flavoprotein 2, mitochondrial	3	98	4	1.589	0.083	1.6E-03	X
G1SVB6	P00367	GLUD1	Glutamate dehydrogenase	Glutamate dehydrogenase 1, mitochondrial	2	98	4	1.588	0.101	2.6E-03	X
G1SI29	P49411	TUFM	Elongation factor Tu	Elongation factor Tu, mitochondrial	2	93	4	1.587	0.048	7.0E-04	X
G1SQ45	A0A0C4DG98	THOC2	Uncharacterized protein	THO complex subunit 2	3	98	4	1.587	0.033	5.3E-04	X
G1T8E0	A3KMH1	VWA8	VWFA domain-containing protein	von Willebrand factor A domain-containing protein 8	2	92	4	1.585	0.077	1.4E-03	X
	B1ANM7	FAF1		FAS-associated factor 1	4		4	1.585	0.182	1.6E-02	X
G1T6M1	Q96DA6	DNAJC19	J domain-containing protein	Mitochondrial import inner membrane translocase subunit TIM14	2	100	4	1.585	0.061	9.6E-04	X
	Q16394	EXT1		Exostosin-1	4		4	1.583	0.119	4.2E-03	X
G1U2T2	H0YIB4	SRSF9	Uncharacterized protein	Serine/arginine-rich-splicing factor 9 (Fragment)	3	65	4	1.581	0.030	5.3E-04	X
G1SHL9	Q15006	EMC2	TPR_REGION domain-containing protein	ER membrane protein complex subunit 2	2	99	4	1.580	0.070	1.3E-03	X
G1T970	P30038	ALDH4A1	Multifunctional fusion protein	Delta-1-pyrroline-5-carboxylate dehydrogenase, mitochondrial	2	91	4	1.579	0.104	2.9E-03	X
	A0A024R4M0	RPS9		40S ribosomal protein S9	4		4	1.578	0.115	3.9E-03	X
G1U2E5	P08243	ASNS	Asparagine synthetase [glutamine-hydrolyzing]	Asparagine synthetase [glutamine-hydrolyzing]	2	87	4	1.578	0.049	7.7E-04	X
G1U0A4	P21810	BGN	Biglycan	Biglycan	2	94	4	1.577	0.228	3.6E-02	X
G1SYB9		ITGA3	Integrin subunit alpha 3		1		4	1.576	0.068	1.2E-03	X
G1T3R1	Q86SX6	GLRX5	Glutaredoxin 5	Glutaredoxin-related protein 5, mitochondrial	2	84	4	1.576	0.033	5.7E-04	X
G1SDL0	Q96DX4	RSPRY1	Uncharacterized protein	RING finger and SPRY domain-containing protein 1	3	93	4	1.576	0.034	5.8E-04	X
	Q01105-2	SET		Isoform 2 of Protein SET	4		4	1.575	0.089	2.0E-03	X
G1T617	P49756	RBM25	Uncharacterized protein	RNA-binding protein 25	3	99	4	1.573	0.116	4.1E-03	X
G1SUF4	O15269	SPTLC1	Aminotran_1_2 domain-containing protein	Serine palmitoyltransferase 1	2	95	4	1.573	0.027	5.3E-04	X
G1T134		ABHD11	Abhydrolase domain containing 11		1		4	1.570	0.098	2.7E-03	X
	Q12931	TRAP1		Heat shock protein 75 kDa, mitochondrial	4		4	1.569	0.048	7.9E-04	
G1SGY1	Q86TB9	PATL1	PAT1 domain-containing protein	Protein PAT1 homolog 1	2	97	4	1.568	0.026	5.3E-04	
U3KM30	Q969X5	ERGIC1	Uncharacterized protein	Endoplasmic reticulum-Golgi intermediate compartment protein 1	3	99	4	1.566	0.032	6.0E-04	
G1TE34	P06756	ITGAV	Integrin_alpha2 domain-containing protein	Integrin alpha-V	2	95	4	1.566	0.090	2.2E-03	
G1U636	G3V153	CAPRIN1	Uncharacterized protein	Caprin-1	3	98	4	1.566	0.048	8.0E-04	
B7NZG9	Q8N3U4	STAG2	Stromal antigen 2 isoform a (Predicted)	Cohesin subunit SA-2	2	97	4	1.564	0.077	1.6E-03	
G1T657	A0A087WUK2	HNRNPDL	Heterogeneous nuclear ribonucleoprotein D like	Heterogeneous nuclear ribonucleoprotein D-like	2	92	4	1.564	0.032	6.0E-04	
G1SQA8	P06576	ATP5F1B	ATP synthase subunit beta	ATP synthase subunit beta, mitochondrial	2	97	4	1.562	0.083	1.9E-03	
G1SWW7	B4DJK0	SRSF5	Uncharacterized protein	Serine/arginine-rich-splicing factor 5	3	100	4	1.562	0.076	1.6E-03	
G1SW61	Q2TAY7	SMU1	Uncharacterized protein	WD40 repeat-containing protein SMU1	3	100	4	1.561	0.150	9.8E-03	
G1TQ57	H3BP71	RNF40	E3 ubiquitin protein ligase	E3 ubiquitin protein ligase	2	92	3	1.558	0.048	2.5E-03	
	O60518	RANBP6		Ran-binding protein 6	4		4	1.556	0.144	8.8E-03	
G1TVN1	I3L4X2	ABCC1	ATP binding cassette subfamily C member 1	Multidrug resistance-associated protein 1 (Fragment)	2	91	4	1.556	0.121	5.2E-03	
G1SV12	P16219	ACADS	Uncharacterized protein	Short-chain specific acyl-CoA dehydrogenase, mitochondrial	3	92	4	1.554	0.072	1.5E-03	
G1T1R4		NR3C1	Glucocorticoid receptor		1		4	1.552	0.065	1.3E-03	
G1SP24	E7EQB9	POLR1C	RPOLD domain-containing protein	DNA-directed RNA polymerases I and III subunit RPAC1	2	84	4	1.552	0.088	2.3E-03	
G1T9V1	P11177	PDHB	Pyruvate dehydrogenase E1 component subunit beta	Pyruvate dehydrogenase E1 component subunit beta, mitochondrial	2	97	4	1.549	0.087	2.3E-03	
G1SV60	J3KS45	TMCO1	Calcium load-activated calcium channel	Calcium load-activated calcium channel (Fragment)	2	95	4	1.546	0.080	2.0E-03	
G1TFE0	H0YHA7	RPL18	Ribosomal_L18e/L15P domain-containing protein	60S ribosomal protein L18 (Fragment)	2	90	4	1.546	0.042	8.1E-04	
G1STX9		COQ8B	Coenzyme Q8B		1		4	1.544	0.022	6.0E-04	
	Q12873	CHD3		Chromodomain-helicase-DNA-binding protein 3	4		4	1.543	0.056	1.1E-03	
G1TI97	Q13769	THOC5	Uncharacterized protein	THO complex subunit 5 homolog	3	97	4	1.543	0.119	5.4E-03	
G1SV32	P18124	RPL7	Uncharacterized protein	60S ribosomal protein L7	3	98	4	1.542	0.073	1.7E-03	
G1T3S2	P54753	EPHB3	Uncharacterized protein	Ephrin type-B receptor 3	3	98	4	1.540	0.056	1.2E-03	
G1SGB5	P23246	SFPQ	Splicing factor proline and glutamine rich	Splicing factor, proline- and glutamine-rich	2	100	4	1.539	0.025	6.3E-04	
G1SHE2		GGCX	HTTM domain-containing protein		1		4	1.534	0.078	2.0E-03	
G1TUX2	A2A274	ACO2	Aconitate hydratase, mitochondrial	Aconitate hydratase, mitochondrial	2	94	4	1.533	0.071	1.7E-03	
G1TEZ1	G3V198	NUP160	Uncharacterized protein	Nuclear pore complex protein Nup160 (Fragment)	3	93	4	1.529	0.021	6.4E-04	
G1TIT1	O75477	ERLIN1	PHB domain-containing protein	Erlin-1	2	98	4	1.528	0.069	1.7E-03	
G1TDX2	A0A3B3ISY9	AGK	Acylglycerol kinase	Acylglycerol kinase, mitochondrial	2	94	4	1.526	0.037	8.2E-04	
G1T0N4	P06748	NPM1	Uncharacterized protein	Nucleophosmin	3	89	4	1.525	0.052	1.2E-03	
G1T301	A0A0A0MRN4	ZNF326	Uncharacterized protein	DBIRD complex subunit ZNF326	3	96	4	1.524	0.053	1.2E-03	
G1SRZ0		TOR2A	Torsin family 2 member A		1		4	1.523	0.059	1.4E-03	
G1SL42	Q9BPW8	NIPSNAP1	NIPSNAP domain-containing protein	Protein NipSnap homolog 1	2	95	4	1.522	0.058	1.4E-03	
G1TTB5	A0A0D9SEM4	SRSF4	Serine and arginine rich splicing factor 4	Serine/arginine-rich-splicing factor 4 (Fragment)	2	75	4	1.521	0.074	2.0E-03	
G1U194	J3KPP4	LUC7L3	Uncharacterized protein	Cisplatin resistance-associated overexpressed protein, isoform CRA_b	3	98	4	1.521	0.045	1.0E-03	
G1SDJ7	B1ANR0	PABPC4	Polyadenylate-binding protein	Polyadenylate-binding protein	2	92	4	1.518	0.058	1.4E-03	
G1TMZ2		MRPS7	Ribosomal_S7 domain-containing protein		1		4	1.518	0.021	7.0E-04	
G1SGI8	O95881	TXNDC12	Thioredoxin domain-containing protein	Thioredoxin domain-containing protein 12	2	95	4	1.518	0.074	2.0E-03	
	P49750-1	YLPM1		Isoform 1 of YLP motif-containing protein 1	4		4	1.518	0.028	7.7E-04	
G1TSL1	A0A087WZN1	IDH3B	Isocitrate dehydrogenase [NAD] subunit, mitochondrial	Isocitrate dehydrogenase [NAD] subunit, mitochondrial	2	95	4	1.517	0.072	1.9E-03	
G1TAB7	A0A2R8Y3X5	OPA1	Dynamin-type G domain-containing protein	Dynamin-like 120 kDa protein, mitochondrial	2	94	4	1.517	0.038	9.1E-04	
G1STH0	Q15459	SF3A1	Uncharacterized protein	Splicing factor 3A subunit 1	3	98	4	1.514	0.038	9.3E-04	
P27124	Q02790	FKBP4	Peptidyl-prolyl cis-trans isomerase FKBP4	Peptidyl-prolyl cis-trans isomerase FKBP4	2	91	4	1.514	0.072	2.0E-03	
G1SES8	G5E9V5	MRPS22	Uncharacterized protein	28S ribosomal protein S22, mitochondrial	3	84	4	1.514	0.063	1.6E-03	
	Q13045	FLII		Protein flightless-1 homolog	4		4	1.513	0.084	2.7E-03	
G1SNR2	Q969S9	GFM2	Ribosome-releasing factor 2, mitochondrial	Ribosome-releasing factor 2, mitochondrial	2	88	4	1.512	0.105	4.8E-03	

G1SN37	O43172	PRPF4	WD_REPEATS_REGION domain-containing protein	U4/U6 small nuclear ribonucleoprotein Prp4	2	99	3	1.512	0.242	5.3E-02
	B7WP74	CWC22		Pre-mRNA-splicing factor CWC22 homolog (Fragment)	4		4	1.511	0.081	2.5E-03
G1TBS2	Q9Y265	RUVBL1	RuvB-like helicase	RuvB-like 1	2	100	4	1.511	0.066	1.8E-03
G1SKD9	P53597	SUCLG1	Succinate--CoA ligase [ADP/GDP-forming] subunit alpha, mitochondrial	Succinate--CoA ligase [ADP/GDP-forming] subunit alpha, mitochondrial	2	96	4	1.510	0.080	2.5E-03
U3KPG6		ICAM1	Intercellular adhesion molecule 1		1		4	1.508	0.147	1.3E-02
G1U1H3	P61018	RAB4B	Uncharacterized protein	Ras-related protein Rab-4B	3	100	4	1.508	0.061	1.6E-03
G1T5G8	Q9HC07	TMEM165	GDT1 family protein	Transmembrane protein 165	2	91	4	1.503	0.060	1.6E-03
	A0A0J9YVP6	PUF60		Poly(U)-binding-splicing factor PUF60 (Fragment)	4		4	1.502	0.026	8.2E-04
G1T4Z1	Q07954	LRP1	Uncharacterized protein	Prolow-density lipoprotein receptor-related protein 1	3	98	4	1.502	0.084	2.9E-03
Q8HZQ5	E7EQR4	EZR	Ezrin	Ezrin	2	94	4	1.501	0.116	6.6E-03
G1SGG2	V9GYM8	ARHGEF2	Uncharacterized protein	Rho guanine nucleotide exchange factor 2	3	95	4	1.501	0.083	2.9E-03
G1SVH1	A0A0U1RRK1	MICU1	Uncharacterized protein	Calcium uptake protein 1, mitochondrial	3	94	4	1.499	0.051	1.4E-03
	G3V0I5	NDUFV1		NADH dehydrogenase [ubiquinone] flavoprotein 1, mitochondrial	4		4	1.499	0.129	9.4E-03
	U3KQK1	LSM4		U6 snRNA-associated Sm-like protein LSm4	4		4	1.499	0.082	2.9E-03
G1SK29		MTIF2	Tr-type G domain-containing protein		1		4	1.498	0.157	1.7E-02
G1TG28	P80723	BASP1	Brain abundant membrane attached signal protein 1	Brain acid soluble protein 1	2	61	4	1.498	0.107	5.5E-03
G1TUB8	Q5VVC8	RPL11	Uncharacterized protein	60S ribosomal protein L11	3	100	4	1.498	0.106	5.4E-03
G1SG80	F8VZG5	AK2	Adenylate kinase 2, mitochondrial	Adenylate kinase 2, mitochondrial	2	92	4	1.497	0.052	1.4E-03
G1T5M0	P04181	OAT	Uncharacterized protein	Ornithine aminotransferase, mitochondrial	3	91	4	1.496	0.091	3.7E-03
G1T8H1	O95573	ACSL3	AMP-binding domain-containing protein	Long-chain-fatty-acid--CoA ligase 3	2	96	4	1.494	0.229	5.7E-02
G1TBL0	E7ETZ4	BZW2	Basic leucine zipper and W2 domains 2	Basic leucine zipper and W2 domain-containing protein 2 (Fragment)	2	100	4	1.494	0.016	7.9E-04
G1SDV5		NOL9	CLP1_P domain-containing protein		1		4	1.493	0.088	3.5E-03
	P0DN76	U2AF1L5		Splicing factor U2AF 35 kDa subunit-like protein	4		4	1.493	0.033	1.0E-03
	P62304	SNRPE		Small nuclear ribonucleoprotein E	4		4	1.490	0.056	1.6E-03
G1TUK6	O14656	TOR1A	Torsin family 1 member A	Torsin-1A	2	90	4	1.488	0.069	2.2E-03
A0A140TAV6		HBB2	Globin A1		1		4	1.488	0.203	4.1E-02
G1STF9	Q13347	EIF3I	Eukaryotic translation initiation factor 3 subunit I	Eukaryotic translation initiation factor 3 subunit I	2	100	4	1.487	0.059	1.8E-03
G1SGY0	O00267	SUPT5H	Transcription elongation factor SPT5	Transcription elongation factor SPT5	2	98	4	1.487	0.019	8.5E-04
G1TCH9	A0A2R8Y473	ABCB7	Uncharacterized protein	ATP-binding cassette sub-family B member 7, mitochondrial	3	95	4	1.486	0.047	1.4E-03
G1TDK8	F8VXC8	SMARCC2	Uncharacterized protein	SWI/SNF complex subunit SMARCC2	3	93	4	1.484	0.047	1.4E-03
G1SM77	P36542	ATP5F1C	ATP synthase subunit gamma	ATP synthase subunit gamma, mitochondrial	2	94	4	1.484	0.057	1.7E-03
G1STJ3		PPIE	Peptidyl-prolyl cis-trans isomerase E		1		4	1.483	0.146	1.5E-02
G1SEC9	Q9HAV0	GNB4	WD_REPEATS_REGION domain-containing protein	Guanine nucleotide-binding protein subunit beta-4	2	99	4	1.483	0.042	1.3E-03
G1SHJ3		KDELC1	CAP10 domain-containing protein		1		4	1.480	0.108	6.4E-03
G1SQZ4	Q9Y230	RUVBL2	RuvB-like helicase	RuvB-like 2	2	99	4	1.480	0.069	2.4E-03
	Q96PK6-5	RBM14		Isoform 5 of RNA-binding protein 14	4		4	1.480	0.083	3.4E-03
G1SYT7	G3V0E4	PMPCB	Uncharacterized protein	Mitochondrial-processing peptidase subunit beta	3	94	4	1.479	0.008	8.2E-04
G1SKZ8	P62906	RPL10A	Ribosomal protein	60S ribosomal protein L10a	2	100	4	1.479	0.075	2.8E-03
G1SVD5	P55265	ADAR	Uncharacterized protein	Double-stranded RNA-specific adenosine deaminase	3	80	4	1.479	0.045	1.4E-03
G1TTK6	E7ETT1	PCCB	Uncharacterized protein	Propionyl-CoA carboxylase beta chain, mitochondrial	3	86	4	1.478	0.099	5.2E-03
G1SMP5	Q5T160	RARS2	DALR_1 domain-containing protein	Probable arginine--tRNA ligase, mitochondrial	2	93	4	1.477	0.089	4.1E-03
	Q9NRZ7	AGPAT3		1-acyl-sn-glycerol-3-phosphate acyltransferase gamma	4		4	1.477	0.051	1.6E-03
G1SWK4	B4E1G1	DERL1	Derlin	Derlin	2	99	4	1.474	0.060	2.0E-03
G1SCT6	E9PB90	HK2	Uncharacterized protein	Hexokinase-2	3	96	4	1.472	0.061	2.1E-03
G1T9E3	E9PLP8	CSTF3	Suf domain-containing protein	Cleavage stimulation factor subunit 3	2	100	4	1.472	0.049	1.6E-03
G1SD54	Q99720	SIGMAR1	Uncharacterized protein	Sigma non-opioid intracellular receptor 1	3	97	4	1.472	0.057	1.9E-03
G1T8B3	Q8IZL8	PELP1	Uncharacterized protein	Proline-, glutamic acid- and leucine-rich protein 1	3	91	4	1.469	0.088	4.2E-03
G1SEX6	Q9H2U1	DHX36	Uncharacterized protein	ATP-dependent DNA/RNA helicase DHX36	3	94	4	1.469	0.081	3.4E-03
G1SRS0	D6RBS5	ELMOD2	ELMO domain-containing protein	ELMO domain-containing protein 2 (Fragment)	2	93	4	1.469	0.036	1.3E-03
G1T069	P57740	NUP107	Nuclear pore complex protein	Nuclear pore complex protein Nup107	2	94	4	1.469	0.078	3.2E-03
G1SGG6	P48449	LSS	Terpene cyclase/mutase family member	Lanosterol synthase	2	89	4	1.469	0.036	1.3E-03
G1U0Y6		ECI1	Enoyl-CoA delta isomerase 1		1		4	1.468	0.091	4.6E-03
G1SML5	Q9H9B4	SFXN1	Sidoreflexin	Sideroflexin-1	2	94	4	1.467	0.041	1.4E-03
	O75937	DNAJC8		DnaJ homolog subfamily C member 8	4		4	1.466	0.040	1.4E-03
G1SLI0	Q96A33	CCDC47	Uncharacterized protein	Coiled-coil domain-containing protein 47	3	98	4	1.465	0.036	1.3E-03
G1SGA5	Q9HCJ6	VAT1L	PKS_ER domain-containing protein	Synaptic vesicle membrane protein VAT-1 homolog-like	2	96	4	1.463	0.075	3.1E-03
Q28618	P67809	YBX1	Nuclease-sensitive element-binding protein 1	Nuclease-sensitive element-binding protein 1	2	99	4	1.463	0.082	3.8E-03
	P37198	NUP62		Nuclear pore glycoprotein p62	4		4	1.462	0.075	3.2E-03
G1SSM6	Q8WUM0	NUP133	Nucleoporin_C domain-containing protein	Nuclear pore complex protein Nup133	2	91	4	1.462	0.058	2.1E-03
G1T813	P08559	PDHA1	Pyruvate dehydrogenase E1 component subunit alpha	Pyruvate dehydrogenase E1 component subunit alpha, somatic form, mitochondrial	2	99	4	1.462	0.088	4.5E-03
G1TG15		FAM210A	DUF1279 domain-containing protein		1		4	1.460	0.099	6.1E-03
	A0A087WUC6	SPCS2		Signal peptidase complex subunit 2	4		4	1.460	0.048	1.7E-03
G1SQP9	E7EMS6	COMT	Catechol-O-methyltransferase	Catechol O-methyltransferase (Fragment)	2	78	4	1.459	0.052	1.9E-03
G1SZZ1	Q9H0V1	TMEM168	Transmembrane protein 168	Transmembrane protein 168	2	96	4	1.459	0.091	4.9E-03
G1TRH3	Q9UBR2	CTSZ	Pept_C1 domain-containing protein	Cathepsin Z	2	86	4	1.458	0.122	1.1E-02
G1TCX6	P08240	SRPRA	SRP54 domain-containing protein	Signal recognition particle receptor subunit alpha	2	99	4	1.455	0.003	1.0E-03
G1SFI7	P49915	GMPS	Uncharacterized protein	GMP synthase [glutamine-hydrolyzing]	3	99	4	1.454	0.072	3.1E-03
G1SSB5	C9JLU1	POLR2H	DNA-directed RNA polymerases I, II, and III subunit RPABC3	DNA-directed RNA polymerases I, II, and III subunit RPABC3 (Fragment)	2	100	4	1.453	0.031	1.3E-03
G1SFG0	A0A087WVP1	FAT1	Uncharacterized protein	Protocadherin Fat 1	3	92	4	1.453	0.081	4.0E-03

G1SLJ8	Q8IY81	FTSJ3	pre-rRNA processing protein FTSJ3	pre-rRNA 2~-O-ribose RNA methyltransferase FTSJ3	2	85	4	1.453	0.155	2.3E-02
G1U864		TRRAP	Transformation/transcription domain associated protein		1		4	1.451	0.111	8.8E-03
	G3V1C3	API5		Apoptosis inhibitor 5	4		4	1.450	0.034	1.4E-03
G1T524	P05141	SLC25A5	Uncharacterized protein	ADP/ATP translocase 2	3	98	4	1.450	0.054	2.1E-03
G1T1L4		GRWD1	WD_REPEATS_REGION domain-containing protein		1		4	1.449	0.076	3.7E-03
G1T2G3	Q5VTR2	RNF20	E3 ubiquitin protein ligase	E3 ubiquitin-protein ligase BRE1A	2	98	4	1.449	0.063	2.6E-03
G1SH10	H3BVG0	NUP93	Nuclear pore complex protein Nup93	Nuclear pore complex protein Nup93	2	99	4	1.449	0.026	1.3E-03
G1SQA4	Q6UWP7	LCLAT1	PlsC domain-containing protein	Lysocardiolipin acyltransferase 1	2	89	4	1.448	0.083	4.4E-03
G1SLC2	O00303	EIF3F	Eukaryotic translation initiation factor 3 subunit F	Eukaryotic translation initiation factor 3 subunit F	2	99	4	1.447	0.103	7.4E-03
G1T647		GCLM	Glutamate-cysteine ligase modifier subunit		1		4	1.445	0.045	1.8E-03
G1TEI0		PARP1	Poly [ADP-ribose] polymerase		1		4	1.445	0.040	1.6E-03
G1SU33		NSUN2	NOP2/Sun RNA methyltransferase family member 2		1		4	1.443	0.053	2.2E-03
G1T920	P49903	SEPHS1	Uncharacterized protein	Selenide, water dikinase 1	3	100	4	1.442	0.078	4.1E-03
G1TEN1	H7C5S0	ACTL6A	Uncharacterized protein	Actin-like protein 6A (Fragment)	3	99	4	1.441	0.102	7.5E-03
G1TWS0	B3KY94	CDIPT	CDP-diacylglycerol--inositol 3-phosphatidyltransferase	CDP-diacylglycerol--inositol 3-phosphatidyltransferase	2	75	4	1.440	0.038	1.6E-03
G1SDN4	O60506-3	SYNCRIP	Uncharacterized protein	Isoform 3 of Heterogeneous nuclear ribonucleoprotein Q	3	100	4	1.438	0.045	1.9E-03
	B4DQT1	MAEA		Macrophage erythroblast attacher	4		4	1.438	0.042	1.8E-03
G1SDD0	P30084	ECHS1	Uncharacterized protein	Enoyl-CoA hydratase, mitochondrial	3	86	4	1.438	0.087	5.4E-03
G1TXS5	Q9UGP8	SEC63	J domain-containing protein	Translocation protein SEC63 homolog	2	93	4	1.438	0.051	2.2E-03
G1T0V4	F5H013	SNRPG	Small nuclear ribonucleoprotein G	Small nuclear ribonucleoprotein G	2	98	4	1.438	0.065	3.0E-03
P42675	E9PCB6	NLN	Neurolysin, mitochondrial	Neurolysin, mitochondrial	2	94	4	1.438	0.026	1.4E-03
G1TTY7	M0R1A7	RPL18A	60S ribosomal protein L18a	60S ribosomal protein L18a	2	90	4	1.437	0.116	1.1E-02
G1TTL1	A0A0B4J1W3	NAA15	Uncharacterized protein	N-alpha-acetyltransferase 15, NatA auxiliary subunit	3	99	4	1.436	0.045	1.9E-03
G1T7L0		CTSC	Pept_C1 domain-containing protein		1		4	1.431	0.148	2.4E-02
G1SHG0	P62899	RPL31	Uncharacterized protein	60S ribosomal protein L31	3	100	4	1.429	0.090	6.2E-03
G1THL2		FTL	Ferritin		1		4	1.427	0.071	3.8E-03
G1SD98	Q13523	PRPF4B	Pre-mRNA processing factor 4B	Serine/threonine-protein kinase PRP4 homolog	2	98	4	1.426	0.037	1.8E-03
G1SSL0	P30040	ERP29	Endoplasmic reticulum resident protein 29	Endoplasmic reticulum resident protein 29	2	93	4	1.425	0.063	3.2E-03
G1SRV1	K7ELV2	SEH1L	Uncharacterized protein	Nucleoporin SEH1 (Fragment)	3	92	4	1.425	0.044	2.1E-03
G1TD91	O43809	NUDT21	Nudix hydrolase domain-containing protein	Cleavage and polyadenylation specificity factor subunit 5	2	100	4	1.424	0.023	1.5E-03
G1SQG1	H0YK61	EMC4	ER membrane protein complex subunit 4	ER membrane protein complex subunit 4	2	97	4	1.424	0.035	1.8E-03
G1SKP2	H0Y8C6	IPO5	Importin N-terminal domain-containing protein	Importin-5 (Fragment)	2	99	4	1.424	0.087	6.1E-03
G1T237	F8VVM2	SLC25A3	Uncharacterized protein	Phosphate carrier protein, mitochondrial	3	86	4	1.423	0.050	2.4E-03
G1U1Q1		THBS2	Thrombospondin 2		1		4	1.423	0.142	2.2E-02
G1U6X4	F8VQZ7	METAP2	Methionine aminopeptidase 2	Methionine aminopeptidase 2	2	94	4	1.422	0.099	8.4E-03
G1TB39	P29353	SHC1	Uncharacterized protein	SHC-transforming protein 1	3	97	4	1.420	0.144	2.4E-02
P30947	P08238	HSP90AB1	Heat shock protein HSP 90-beta	Heat shock protein HSP 90-beta	2	99	4	1.418	0.112	1.2E-02
G1SJW8	H3BND8	USP7	Uncharacterized protein	Ubiquitin carboxyl-terminal hydrolase (Fragment)	3	99	4	1.418	0.095	7.8E-03
G1SPK4	P05388	RPLP0	60S acidic ribosomal protein P0	60S acidic ribosomal protein P0	2	98	3	1.417	0.023	5.3E-03
G1SXR1		PRELP	LRRNT domain-containing protein		1		4	1.416	0.235	9.5E-02
G1SPH7	Q9P2I0	CPSF2	Cleavage and polyadenylation specificity factor subunit 2	Cleavage and polyadenylation specificity factor subunit 2	2	99	4	1.416	0.106	1.0E-02
U3KPB2	P61009	SPCS3	Signal peptidase complex subunit 3	Signal peptidase complex subunit 3	2	100	4	1.416	0.026	1.7E-03
G1STL1	Q8WUA2	PPIL4	Peptidyl-prolyl cis-trans isomerase	Peptidyl-prolyl cis-trans isomerase-like 4	2	97	4	1.416	0.029	1.7E-03
G1TTA5	H0Y8C3	MTCH1	Uncharacterized protein	Mitochondrial carrier homolog 1 (Fragment)	3	93	4	1.415	0.079	5.3E-03
G1TME5	Q9H857	NT5DC2	Uncharacterized protein	5~-nucleotidase domain-containing protein 2	3	86	4	1.413	0.081	5.7E-03
G1T2G5	Q5NDL2	EOGT	Uncharacterized protein	EGF domain-specific O-linked N-acetylglucosamine transferase	3	91	4	1.411	0.053	2.9E-03
G1SCF6		YARS2	Tyrosine--tRNA ligase		1		4	1.410	0.039	2.2E-03
G1TCT4	O95155	UBE4B	U-box domain-containing protein	Ubiquitin conjugation factor E4 B	2	95	4	1.410	0.063	3.7E-03
G1SG07	Q8IWT6	LRRC8A	Leucine rich repeat containing 8 VRAC subunit A	Volume-regulated anion channel subunit LRRC8A	2	98	4	1.409	0.047	2.6E-03
G1TIT4		PTTG1IP	PTTG1 interacting protein		1		4	1.409	0.115	1.4E-02
G1SEE0	Q09028	RBBP4	RB binding protein 4, chromatin remodeling factor	Histone-binding protein RBBP4	2	100	4	1.409	0.052	2.9E-03
G1SIB2	A0A087WVM4	MTHFD1L	Uncharacterized protein	Monofunctional C1-tetrahydrofolate synthase, mitochondrial	3	92	4	1.409	0.021	1.7E-03
G1TGI6	A0A0C4DG17	RPSA	40S ribosomal protein SA	40S ribosomal protein SA	2	98	4	1.408	0.097	9.0E-03
G1TSP3	Q9NZ01	TECR	Uncharacterized protein	Very-long-chain enoyl-CoA reductase	3	98	4	1.406	0.040	2.3E-03
G1SZ23	E9PCG9	BDH1	Uncharacterized protein	D-beta-hydroxybutyrate dehydrogenase, mitochondrial	3	87	4	1.405	0.062	3.8E-03
G1U7L4	P11021	HSPA5	Heat shock protein family A (Hsp70) member 5	Endoplasmic reticulum chaperone BiP	2	99	4	1.405	0.054	3.1E-03
	A0A0A6YYJ8	LUC7L2		Putative RNA-binding protein Luc7-like 2	4		4	1.403	0.054	3.2E-03
G1U4G9	O00299	CLIC1	Chloride intracellular channel protein	Chloride intracellular channel protein 1	2	98	4	1.402	0.092	8.3E-03
G1SDH8	Q5T9L3	WLS	Uncharacterized protein	Protein wntless homolog	3	98	4	1.402	0.082	6.5E-03
G1SPR9	P04844	RPN2	Dolichyl-diphosphooligosaccharide--protein glycosyltransferase subunit 2	Dolichyl-diphosphooligosaccharide--protein glycosyltransferase subunit 2	2	93	4	1.401	0.041	2.5E-03
G1SZR8	P51858	HDGF	Heparin binding growth factor	Hepatoma-derived growth factor	2	96	4	1.401	0.108	1.2E-02
G1SHK6	Q5SSJ5	HP1BP3	Uncharacterized protein	Heterochromatin protein 1-binding protein 3	3	93	4	1.400	0.020	1.8E-03
G1SUC8	P60228	EIF3E	Eukaryotic translation initiation factor 3 subunit E	Eukaryotic translation initiation factor 3 subunit E	2	100	4	1.399	0.033	2.2E-03
G1T511	A0A3F2YNY6	PRPF40A	Pre-mRNA processing factor 40 homolog A	Pre-mRNA-processing factor 40 homolog A	2	83	4	1.399	0.065	4.4E-03
G1STU7	Q5QNZ2	ATP5PB	Uncharacterized protein	ATP synthase F(0) complex subunit B1, mitochondrial	3	84	4	1.398	0.066	4.4E-03
G1SMR7	P30050	RPL12	Uncharacterized protein	60S ribosomal protein L12	3	100	4	1.398	0.046	2.8E-03
G1SM52	Q96AG4	LRRC59	Uncharacterized protein	Leucine-rich repeat-containing protein 59	3	97	4	1.397	0.070	5.0E-03
G1SED9	B0QY89	EIF3L	Eukaryotic translation initiation factor 3 subunit L	Eukaryotic translation initiation factor 3 subunit L	2	97	4	1.397	0.075	5.8E-03
G1SGJ5	Q92600	CNOT9	Uncharacterized protein	CCR4-NOT transcription complex subunit 9	3	97	4	1.396	0.044	2.8E-03

G1TBH1	A0A1W2PPT5	POLR2B	DNA-directed RNA polymerase subunit beta	DNA-directed RNA polymerase subunit beta	2	100	4	1.396	0.054	3.4E-03
	Q03252	LMNB2		Lamin-B2	4		4	1.395	0.065	4.5E-03
G1SXF1		NFU1	Nfu_N domain-containing protein		1		4	1.395	0.052	3.2E-03
G1SVQ8	F8WF48	SEC62	Uncharacterized protein	Translocation protein SEC62	3	100	4	1.394	0.023	1.9E-03
G1SEC8	Q8IWA4	MFN1	Dynamin-type G domain-containing protein	Mitofusin-1	2	92	4	1.394	0.043	2.7E-03
G1SKT4	P25705	ATP5F1A	ATP synthase subunit alpha	ATP synthase subunit alpha, mitochondrial	2	98	4	1.394	0.059	3.9E-03
A0A0A0MQQ6	D6RBW1	EIF4E	Eukaryotic translation initiation factor 4E	Eukaryotic translation initiation factor 4E	2	98	4	1.394	0.069	5.0E-03
G1T3A6	A0A1W2PQH3	ME2	Malic enzyme	Malic enzyme	2	92	4	1.393	0.129	2.2E-02
G1U448	E9PEB5	FUBP1	Uncharacterized protein	Far upstream element-binding protein 1	3	93	4	1.393	0.065	4.5E-03
G1TST9	A0A2R8Y212	CHD4	Uncharacterized protein	Chromodomain-helicase-DNA-binding protein 4	3	97	4	1.392	0.030	2.2E-03
G1SYD6	P02545	LMNA	Uncharacterized protein	Prelamin-A/C	3	98	4	1.392	0.051	3.3E-03
G1TJV3	Q9H7Z7	PTGES2	Prostaglandin E synthase 2	Prostaglandin E synthase 2	2	79	4	1.391	0.082	7.1E-03
G1SSC9	O95373	IPO7	Importin N-terminal domain-containing protein	Importin-7	2	100	4	1.391	0.072	5.6E-03
G1T0Q6	Q9UQE7	SMC3	Structural maintenance of chromosomes protein	Structural maintenance of chromosomes protein 3	2	100	4	1.390	0.155	3.7E-02
G1SRW4	Q9Y6C2	EMILIN1	Elastin microfibril interfacer 1	EMILIN-1	2	87	4	1.390	0.109	1.4E-02
P67828	P48729	CSNK1A1	Casein kinase I isoform alpha	Casein kinase I isoform alpha	2	100	4	1.390	0.071	5.4E-03
G1U276	A0A2R8Y6Y7	SUCLA2	Succinate--CoA ligase [ADP-forming] subunit beta, mitochondrial	Succinate--CoA ligase [ADP-forming] subunit beta, mitochondrial	2	91	4	1.390	0.064	4.6E-03
G1SH80	O14874	BCKDK	Protein-serine/threonine kinase	[3-methyl-2-oxobutanoate dehydrogenase [lipoamide]] kinase, mitochondrial	2	97	4	1.387	0.046	3.1E-03
G1T0W7	M0R1B0	EMC8	ER membrane protein complex subunit 8	ER membrane protein complex subunit 8 (Fragment)	2	98	4	1.386	0.045	3.1E-03
G1T1G2	A0A1B0GWA2	AGPS	Alkylglycerone-phosphate synthase	Alkylglycerone-phosphate synthase (Fragment)	2	96	4	1.385	0.084	7.9E-03
G1SEW1	R4GMQ1	KDM1A	Lysine-specific histone demethylase	Lysine-specific histone demethylase	2	96	4	1.385	0.013	1.9E-03
	Q5R3B4	MPC2		Mitochondrial pyruvate carrier (Fragment)	4		4	1.385	0.110	1.5E-02
U3KM71		ATP5MG	ATP synthase subunit		1		4	1.384	0.067	5.2E-03
G1T925		GPX8	Glutathione peroxidase		1		4	1.384	0.065	5.0E-03
	K7EK33	DAZAP1		DAZ-associated protein 1	4		4	1.383	0.061	4.5E-03
G1SZZ2	P49458	SRP9	Signal recognition particle 9 kDa protein	Signal recognition particle 9 kDa protein	2	92	4	1.383	0.039	2.8E-03
G1U3I5		ECH1	Enoyl-CoA hydratase 1		1		4	1.383	0.091	9.9E-03
G1SJQ6	O00519	FAAH	Amidase domain-containing protein	Fatty-acid amide hydrolase 1	2	91	4	1.382	0.117	1.8E-02
G1TEE3	G3V5Z3	PPP4R3A	SMK-1 domain-containing protein	Serine/threonine-protein phosphatase 4 regulatory subunit 3A	2	100	4	1.382	0.039	2.9E-03
	P19623	SRM		Spermidine synthase	4		4	1.380	0.087	9.1E-03
G1TMM7	H0YN26	ANP32A	LRRcap domain-containing protein	Acidic leucine-rich nuclear phosphoprotein 32 family member A	2	93	4	1.380	0.100	1.2E-02
G1SW97	A0A0A0MT83	IVD	Uncharacterized protein	Isovaleryl-CoA dehydrogenase isoform 1	3	95	4	1.380	0.063	5.0E-03
	P62424	RPL7A		60S ribosomal protein L7a	4		4	1.379	0.079	7.6E-03
G1U383	A0A087WYN9	DHX29	ATP-dependent RNA helicase DHX29	ATP-dependent RNA helicase DHX29	2	95	4	1.378	0.023	2.3E-03
G1T9N2	O75947	ATP5PD	ATP synthase subunit d, mitochondrial	ATP synthase subunit d, mitochondrial	2	91	4	1.377	0.063	5.1E-03
G1TDJ9	F6Y5H0	RBMS1	RNA binding motif single stranded interacting protein 1	RNA-binding motif, single-stranded-interacting protein 1	2	97	4	1.376	0.091	1.0E-02
G1SFU0	P51149	RAB7A	Ras-related protein Rab-7a	Ras-related protein Rab-7a	2	100	4	1.375	0.031	2.7E-03
Q9GKX2		DHRS4	Dehydrogenase/reductase SDR family member 4 (Fragment)		1		4	1.375	0.048	3.6E-03
G1T9M7	E9PK47	PYGL	Alpha-1,4 glucan phosphorylase	Alpha-1,4 glucan phosphorylase	2	94	4	1.375	0.104	1.4E-02
G1SG61		TPST2	Protein-tyrosine sulfotransferase		1		4	1.373	0.073	6.9E-03
G1SLD7	Q9NXF1	TEX10	Ipi1_N domain-containing protein	Testis-expressed protein 10	2	94	4	1.373	0.050	3.9E-03
G1SE65	A0A0C4DG95	PXK	Uncharacterized protein	PX domain-containing protein kinase-like protein	3	94	3	1.372	0.064	1.2E-02
G1T7J5		NCSTN	Ncstrn_small domain-containing protein		1		4	1.371	0.139	3.2E-02
G1SF32	O94826	TOMM70	TPR_REGION domain-containing protein	Mitochondrial import receptor subunit TOM70	2	97	4	1.368	0.081	8.8E-03
G1TCE9		HSDL2	SCP2 domain-containing protein		1		4	1.367	0.048	4.0E-03
G1SHI9	A0A0D9SFS3	OGDH	Transket_pyr domain-containing protein	2-oxoglutarate dehydrogenase, mitochondrial	2	95	4	1.367	0.079	8.6E-03
G1T7W7	P46977	STT3A	Uncharacterized protein	Dolichyl-diphosphooligosaccharide--protein glycosyltransferase subunit STT3A	3	100	4	1.367	0.041	3.4E-03
G1SDD3		KCTD10	BTB domain-containing protein		1		4	1.365	0.086	1.0E-02
G1TE08	H7C3P6	NUP98	Peptidase S59 domain-containing protein	Nuclear pore complex protein Nup98-Nup96 (Fragment)	2	90	4	1.364	0.084	9.8E-03
G1TCU4	H3BUU9	CDH11	Uncharacterized protein	Cadherin-11	3	99	4	1.364	0.066	6.2E-03
G1T095		TMEM147	Transmembrane protein 147		1		4	1.364	0.067	6.4E-03
G1T7T2	Q15424	SAFB	Scaffold attachment factor B	Scaffold attachment factor B1	2	86	4	1.364	0.065	6.1E-03
G1T994	D6RGG3	COL12A1	Collagen alpha-1(XII) chain	Collagen alpha-1(XII) chain	2	95	4	1.361	0.109	1.9E-02
G1T855	C9J5X1	IGF1R	Tyrosine-protein kinase receptor	Tyrosine-protein kinase receptor	2	93	4	1.359	0.016	2.6E-03
G1TZI2		PGAM5	PGAM family member 5, mitochondrial serine/threonine protein phosphatase		1		4	1.359	0.065	6.5E-03
G1STG2	Q9Y5M8	SRPRB	SRP receptor subunit beta	Signal recognition particle receptor subunit beta	2	94	4	1.358	0.063	6.3E-03
G1SLI8	A0A0A0MSJ0	DDX42	Uncharacterized protein	ATP-dependent RNA helicase DDX42	3	96	4	1.357	0.017	2.7E-03
G1SRD2	P28330	ACADL	Uncharacterized protein	Long-chain specific acyl-CoA dehydrogenase, mitochondrial	3	82	4	1.356	0.107	1.9E-02
G1SLW8	Q7L2H7	EIF3M	Eukaryotic translation initiation factor 3 subunit M	Eukaryotic translation initiation factor 3 subunit M	2	100	4	1.356	0.067	7.1E-03
G1T194	Q9ULX6	AKAP8L	A-kinase anchoring protein 8 like	A-kinase anchor protein 8-like	2	86	4	1.356	0.114	2.2E-02
G1T2J0	P14923	JUP	Uncharacterized protein	Junction plakoglobin	3	99	4	1.356	0.052	5.0E-03
G1U150	M0QXU7	TIMM44	Mitochondrial import inner membrane translocase subunit TIM44	Mitochondrial import inner membrane translocase subunit TIM44 (Fragment)	2	83	4	1.354	0.034	3.4E-03
G1TEN9	Q8TB61	SLC35B2	Uncharacterized protein	Adenosine 3~-phospho 5~-phosphosulfate transporter 1	3	89	4	1.354	0.086	1.2E-02
G1T7J9	B1AV70	YIPF6	Protein YIPF	Protein YIPF (Fragment)	2	75	4	1.353	0.062	6.4E-03
G1SW36	H0YDT8	EMC7	DUF2012 domain-containing protein	ER membrane protein complex subunit 7 (Fragment)	2	79	4	1.353	0.078	9.5E-03
G1TPZ3	Q96GK7	FAHD2A	FAA_hydrolase domain-containing protein	Fumarylacetoacetate hydrolase domain-containing protein 2A	2	90	4	1.353	0.020	2.9E-03
G1STF8		TBRG4	RAP domain-containing protein		1		4	1.353	0.077	9.4E-03
G1SXZ9	Q86WA6	BPHL	Biphenyl hydrolase like	Valacyclovir hydrolase	2	89	4	1.352	0.068	7.6E-03
G1SSL5	Q14CX7	NAA25	TPR_REGION domain-containing protein	N-alpha-acetyltransferase 25, NatB auxiliary subunit	2	96	4	1.352	0.038	3.9E-03

O18757	Q6NUK1	SLC25A24	Calcium-binding mitochondrial carrier protein SCaMC-1	Calcium-binding mitochondrial carrier protein SCaMC-1	2	95	4	1.351	0.031	3.4E-03
G1T9S4	H0YDD4	DLAT	Acetyltransferase component of pyruvate dehydrogenase complex	Acetyltransferase component of pyruvate dehydrogenase complex (Fragment)	2	90	4	1.351	0.061	6.5E-03
G1SLJ9	Q9P0I2	EMC3	ER membrane protein complex subunit 3	ER membrane protein complex subunit 3	2	99	4	1.351	0.085	1.2E-02
G1T6L7	F8WAR4	CHCHD3	MICOS complex subunit	MICOS complex subunit	2	78	4	1.348	0.091	1.4E-02
G1SR19	P20594	NPR2	Guanylate cyclase	Atrial natriuretic peptide receptor 2	2	95	4	1.347	0.074	9.2E-03
G1SCW7	Q9UG63	ABCF2	Uncharacterized protein	ATP-binding cassette sub-family F member 2	3	99	4	1.347	0.064	7.2E-03
G1SUU2	A0A087WXS7	ASNA1	ATPase ASNA1	ATPase ASNA1	2	93	4	1.347	0.034	3.8E-03
Q9TT15	P21796	VDAC1	Voltage-dependent anion-selective channel protein 1	Voltage-dependent anion-selective channel protein 1	2	100	4	1.345	0.019	3.1E-03
G1T6J2		APOO	MICOS complex subunit		1		4	1.345	0.071	8.9E-03
	A0A024RBG1	NUDT4B		Diphosphoinositol polyphosphate phosphohydrolase NUDT4B	4		4	1.345	0.350	2.9E-01
G1SMY6	O14980	XPO1	Importin N-terminal domain-containing protein	Exportin-1	2	99	4	1.344	0.087	1.3E-02
G1T2I5	F8W914	RTN4	Reticulon	Reticulon	2	98	4	1.344	0.127	3.2E-02
G1SFR8	P25398	RPS12	40S ribosomal protein S12	40S ribosomal protein S12	2	100	4	1.344	0.158	5.7E-02
	B5MDE0	RFT1		Protein RFT1 homolog	4		4	1.343	0.046	5.0E-03
G1SVW9		VEZT	Vezatin domain-containing protein		1		3	1.342	0.141	4.7E-02
G1TVY5	C9JZR2	CTNND1	Uncharacterized protein	Catenin delta-1	3	97	4	1.340	0.018	3.2E-03
G1T3U3	A0A0G2JRV3	SMARCB1	SWI/SNF related, matrix associated, actin dependent regulator of chromatin, subfamily b, member 1	SWI/SNF-related matrix-associated actin-dependent regulator of chromatin subfamily B member 1	2	85	4	1.340	0.133	3.8E-02
G1T4D2		ACOX1	Acyl-coenzyme A oxidase		1		4	1.339	0.079	1.1E-02
G1TT27	E9PKZ0	RPL8	Ribosomal_L2_C domain-containing protein	60S ribosomal protein L8 (Fragment)	2	100	4	1.339	0.051	5.8E-03
	P18583-2	SON		Isoform A of Protein SON	4		4	1.339	0.042	4.9E-03
G1SN16	P27695	APEX1	DNA-(apurinic or apyrimidinic site) lyase	DNA-(apurinic or apyrimidinic site) lyase	2	95	4	1.338	0.058	6.9E-03
G1T0M2		TRMT10C	SAM-dependent MTase TRM10-type domain-containing protein		1		4	1.337	0.072	9.8E-03
G1SF08	C9K025	RPL35A	Uncharacterized protein	60S ribosomal protein L35a (Fragment)	3	99	4	1.336	0.273	2.0E-01
G1TEG1	O95470	SGPL1	Uncharacterized protein	Sphingosine-1-phosphate lyase 1	3	90	4	1.336	0.068	9.1E-03
G1SPN3	Q14573	ITPR3	Inositol 1,4,5-trisphosphate receptor type 3	Inositol 1,4,5-trisphosphate receptor type 3	2	95	4	1.335	0.158	6.1E-02
G1SUQ4		DNTTIP2	Fcf2 domain-containing protein		1		4	1.335	0.111	2.6E-02
	Q58FF6	HSP90AB4P		Putative heat shock protein HSP 90-beta 4	4		4	1.332	0.089	1.6E-02
G1SNE9	A0A0G2JQ41	ABR	Uncharacterized protein	Active breakpoint cluster region-related protein (Fragment)	3	100	4	1.331	0.043	5.4E-03
G1T7D1		HIST1H1C	H15 domain-containing protein		1		4	1.330	0.286	2.2E-01
G1SWA0		RIPOR1	RHO family interacting cell polarization regulator 1		1		4	1.330	0.144	5.1E-02
G1TWD8		SELENOO	Selenoprotein O		1		3	1.329	0.140	5.2E-02
	Q96PU8	QKI		Protein quaking	4		4	1.327	0.050	6.6E-03
G1SCW9		CHPF2	Hexosyltransferase		1		4	1.326	0.050	6.6E-03
G1STH4	Q14344	GNA13	Uncharacterized protein	Guanine nucleotide-binding protein subunit alpha-13	3	86	4	1.326	0.036	5.0E-03
G1SZF7	P48735	IDH2	Isocitrate dehydrogenase [NADP]	Isocitrate dehydrogenase [NADP], mitochondrial	2	96	4	1.326	0.191	1.0E-01
G1SUD2	Q6UW02	CYP20A1	Uncharacterized protein	Cytochrome P450 20A1	3	88	4	1.326	0.050	6.6E-03
G1TNM3	P23396	RPS3	KH type-2 domain-containing protein	40S ribosomal protein S3	2	100	4	1.326	0.136	4.6E-02
G1SMZ5	Q14152	EIF3A	Eukaryotic translation initiation factor 3 subunit A	Eukaryotic translation initiation factor 3 subunit A	2	93	4	1.325	0.045	6.1E-03
P62139	P62136	PPP1CA	Serine/threonine-protein phosphatase PP1-alpha catalytic subunit	Serine/threonine-protein phosphatase PP1-alpha catalytic subunit	2	100	4	1.325	0.100	2.2E-02
G1T4A5	P02452	COL1A1	Collagen alpha-1(I) chain	Collagen alpha-1(I) chain	2	91	4	1.323	0.168	7.9E-02
	Q9NYU2	UGGT1		UDP-glucose:glycoprotein glucosyltransferase 1	4		4	1.323	0.064	9.5E-03
G1SJ66	C9JME2	FARP1	Uncharacterized protein	FERM, ARHGEF and pleckstrin domain-containing protein 1	3	90	4	1.322	0.034	5.0E-03
	E9PB61	ALYREF		THO complex subunit 4	4		4	1.320	0.071	1.2E-02
G1T3L2	K7ERF1	EIF3K	Eukaryotic translation initiation factor 3 subunit K	Eukaryotic translation initiation factor 3 subunit K	2	87	4	1.320	0.063	9.6E-03
G1TQD4		RALY	RRM domain-containing protein		1		4	1.320	0.089	1.8E-02
G1T419	Q92621	NUP205	Uncharacterized protein	Nuclear pore complex protein Nup205	3	96	4	1.320	0.060	9.1E-03
G1T1T8	O96005	CLPTM1	CLPTM1, transmembrane protein	Cleft lip and palate transmembrane protein 1	2	97	4	1.320	0.040	5.9E-03
G1SQH0	P61254	RPL26	KOW domain-containing protein	60S ribosomal protein L26	2	100	4	1.320	0.084	1.6E-02
G1T923	A0A1B0GTB0	ATP6AP2	Uncharacterized protein	Renin receptor (Fragment)	3	84	4	1.319	0.097	2.2E-02
G1SE12	P13804	ETFA	Electron transfer flavoprotein subunit alpha	Electron transfer flavoprotein subunit alpha, mitochondrial	2	95	4	1.319	0.056	8.3E-03
O46373	P12235	SLC25A4	ADP/ATP translocase 1	ADP/ATP translocase 1	2	96	4	1.318	0.058	8.8E-03
G1TTN7	O00139	KIF2A	Kinesin-like protein	Kinesin-like protein KIF2A	2	94	4	1.318	0.065	1.0E-02
	Q9Y2Q5	LAMTOR2		Ragulator complex protein LAMTOR2	4		4	1.318	0.129	4.3E-02
G1T5X6		HMGCL	Pyruvate carboxyltransferase domain-containing protein		1		4	1.318	0.155	6.8E-02
G1U0U5	E9PDE8	HSPA4L	Uncharacterized protein	Heat shock 70 kDa protein 4L	3	91	4	1.318	0.107	2.8E-02
G1TA69	Q8TBA6	GOLGA5	Uncharacterized protein	Golgin subfamily A member 5	3	83	4	1.317	0.041	6.1E-03
G1SDK8	Q13564	NAE1	NEDD8-activating enzyme E1 regulatory subunit	NEDD8-activating enzyme E1 regulatory subunit	2	96	4	1.316	0.191	1.1E-01
G1SML9	P31689	DNAJA1	Uncharacterized protein	DnaJ homolog subfamily A member 1	3	100	4	1.316	0.062	1.0E-02
G1SXK8	F8WE74	SLC25A17	Uncharacterized protein	Peroxisomal membrane protein PMP34	3	96	4	1.314	0.058	9.3E-03
G1TBW7	P50148	GNAQ	Uncharacterized protein	Guanine nucleotide-binding protein G(q) subunit alpha	3	99	4	1.312	0.051	8.1E-03
G1SIV7	O43776	NARS	AA_TRNA_LIGASE_II domain-containing protein	Asparagine--tRNA ligase, cytoplasmic	2	91	4	1.312	0.102	2.7E-02
G1SU75	O95140	MFN2	Mitofusin 2	Mitofusin-2	2	92	4	1.311	0.072	1.3E-02
G1T4K8	P32189	GK	Uncharacterized protein	Glycerol kinase	3	97	4	1.311	0.111	3.2E-02
P62497	P62495	ETF1	Eukaryotic peptide chain release factor subunit 1	Eukaryotic peptide chain release factor subunit 1	2	100	4	1.310	0.066	1.2E-02
G1T5V3	P61106	RAB14	Uncharacterized protein	Ras-related protein Rab-14	3	100	4	1.310	0.043	7.0E-03
G1T720	F5GXX5	DAD1	Dolichyl-diphosphooligosaccharide--protein glycosyltransferase subunit DAD1	Dolichyl-diphosphooligosaccharide--protein glycosyltransferase subunit DAD1	2	75	4	1.309	0.034	5.9E-03
G1SCY7	Q8N766	EMC1	EMC1_C domain-containing protein	ER membrane protein complex subunit 1	2	96	4	1.309	0.060	1.0E-02
G1SK61	Q9UIW2	PLXNA1	Plexin A1	Plexin-A1	2	91	4	1.309	0.029	5.5E-03
G1T4Z2	P53396	ACLY	ATP-citrate synthase	ATP-citrate synthase	2	98	4	1.308	0.044	7.3E-03

G1U971		EIF3C	Eukaryotic translation initiation factor 3 subunit C		1		4	1.306	0.034	6.1E-03
G1T673	Q99653	CHP1	Calcineurin like EF-hand protein 1	Calcineurin B homologous protein 1	2	98	4	1.306	0.046	7.9E-03
G1SIJ2	P24752	ACAT1	Uncharacterized protein	Acetyl-CoA acetyltransferase, mitochondrial	3	92	4	1.305	0.105	3.0E-02
G1T5C5	E5RHW4	ERLIN2	PHB domain-containing protein	Erlin-2 (Fragment)	2	98	4	1.304	0.051	8.9E-03
G1TEI1	P20645	M6PR	Uncharacterized protein	Cation-dependent mannose-6-phosphate receptor	3	95	4	1.304	0.050	8.8E-03
G1SVP9	G3V4T2	PABPN1	RRM domain-containing protein	Polyadenylate-binding protein 2	2	99	4	1.303	0.033	6.3E-03
G1T888	Q9P2R3	ANKFY1	Ankyrin repeat and FYVE domain containing 1	Rabankyrin-5	2	96	4	1.302	0.065	1.3E-02
	Q9H0B6	KLC2		Kinesin light chain 2	4		4	1.302	0.070	1.4E-02
G1SZI5	P62269	RPS18	Uncharacterized protein	40S ribosomal protein S18	3	100	4	1.302	0.152	7.5E-02
G1SRB1	Q16134	ETFDH	4Fe-4S ferredoxin-type domain-containing protein	Electron transfer flavoprotein-ubiquinone oxidoreductase, mitochondrial	2	94	4	1.301	0.043	7.9E-03
P67873	Q5SRQ6	CSNK2B	Casein kinase II subunit beta	Casein kinase II subunit beta	2	100	4	1.300	0.043	8.0E-03
	J3KN01	AFDN		Afadin	4		4	1.300	0.116	4.1E-02
G1TS38	H0YF06	CCDC90B	Uncharacterized protein	Coiled-coil domain-containing protein 90B, mitochondrial (Fragment)	3	94	4	1.300	0.043	8.0E-03
G1SPN1		NUDCD2	CS domain-containing protein		1		4	1.299	0.147	7.1E-02
G1U800	Q8NC56	LEMD2	MSC domain-containing protein	LEM domain-containing protein 2	2	79	4	1.298	0.143	6.8E-02
G1T159	P62070	RRAS2	Uncharacterized protein	Ras-related protein R-Ras2	3	97	4	1.298	0.081	1.9E-02
G1T619		MCUB	MCU domain-containing protein		1		4	1.297	0.027	6.2E-03
G1SYL3	E9PFH4	TNPO3	Xpo1 domain-containing protein	Transportin-3	2	96	4	1.297	0.044	8.5E-03
G1TWU8	O43264	ZW10	Uncharacterized protein	Centromere/kinetochore protein zw10 homolog	3	89	4	1.297	0.024	6.0E-03
G1SFV7	Q16531	DDB1	Damage specific DNA binding protein 1	DNA damage-binding protein 1	2	100	4	1.296	0.062	1.2E-02
G1SHU8	D3YTB1	RPL32	Uncharacterized protein	60S ribosomal protein L32 (Fragment)	3	88	4	1.295	0.175	1.1E-01
G1TJS2	Q8IV08	PLD3	Phospholipase D family member 3	Phospholipase D3	2	89	4	1.295	0.194	1.3E-01
G1U6X6	P55795	HNRNPH2	Uncharacterized protein	Heterogeneous nuclear ribonucleoprotein H2	3	100	4	1.295	0.062	1.3E-02
G1TAA4	O75844	ZMPSTE24	CAAX prenyl protease	CAAX prenyl protease 1 homolog	2	96	4	1.294	0.053	1.1E-02
G1T8H5		POLR2C	RPOLD domain-containing protein		1		4	1.292	0.072	1.7E-02
G1T157	Q9Y376	CAB39	Uncharacterized protein	Calcium-binding protein 39	3	99	4	1.292	0.153	8.3E-02
G1T3M3	Q9UL25	RAB21	Uncharacterized protein	Ras-related protein Rab-21	3	97	4	1.290	0.055	1.2E-02
G1TS36	B8ZZG1	MPP6	Uncharacterized protein	Membrane protein, palmitoylated 6 (MAGUK p55 subfamily member 6), isoform CRA_a	3	96	4	1.290	0.097	3.0E-02
G1SHH0	Q14139	UBE4A	Ubiquitination factor E4A	Ubiquitin conjugation factor E4 A	2	98	4	1.290	0.112	4.1E-02
G1U3Q0	Q93050	ATP6V0A1	V-type proton ATPase subunit a	V-type proton ATPase 116 kDa subunit a isoform 1	2	96	4	1.290	0.109	3.9E-02
G1SEI8		NUP214	Nup214_FG domain-containing protein		1		4	1.289	0.057	1.2E-02
G1TBC0	A0A499FI31	SART3	Uncharacterized protein	Squamous cell carcinoma antigen recognized by T-cells 3	3	89	4	1.289	0.049	1.0E-02
G1SI85	A0A0A0MS41	SFXN3	Uncharacterized protein	Sidoreflexin	3	94	4	1.287	0.068	1.6E-02
	A0A0U1RRM4	PTBP1		Polypyrimidine tract-binding protein 1	4		4	1.287	0.040	9.0E-03
G1SFC1	H0Y6I0	GOLGA4	Golgin A4	Golgin subfamily A member 4 (Fragment)	2	77	4	1.287	0.073	1.8E-02
G1SJB4	P63244	RACK1	WD_REPEATS_REGION domain-containing protein	Receptor of activated protein C kinase 1	2	100	4	1.286	0.096	3.1E-02
G1TA59	P50213	IDH3A	Isocitrate dehydrogenase [NAD] subunit, mitochondrial	Isocitrate dehydrogenase [NAD] subunit alpha, mitochondrial	2	98	4	1.286	0.065	1.5E-02
G1SKF7	Q02878	RPL6	60S ribosomal protein L6	60S ribosomal protein L6	2	89	4	1.285	0.030	7.6E-03
G1TFZ7	O15355	PPM1G	PPM-type phosphatase domain-containing protein	Protein phosphatase 1G	2	97	4	1.285	0.087	2.6E-02
G1TIP5	C9JCC6	DRAP1	CBFD_NFYB_HMF domain-containing protein	Dr1-associated corepressor	2	87	4	1.284	0.089	2.8E-02
G1TVW1	O60568	PLOD3	Procollagen-lysine,2-oxoglutarate 5-dioxygenase 3	Multifunctional procollagen lysine hydroxylase and glycosyltransferase LH3	2	94	4	1.282	0.084	2.5E-02
G1SLQ3	P15559	NQO1	Flavodoxin_2 domain-containing protein	NAD(P)H dehydrogenase [quinone] 1	2	89	4	1.281	0.060	1.5E-02
G1T5R3		TCF25	Transcription factor 25		1		4	1.281	0.081	2.4E-02
G1TH33	P38117	ETFB	Electron transfer flavoprotein subunit beta	Electron transfer flavoprotein subunit beta	2	95	4	1.281	0.087	2.8E-02
G1TDI0	Q9UQ80	PA2G4	Peptidase_M24 domain-containing protein	Proliferation-associated protein 2G4	2	98	4	1.280	0.051	1.2E-02
G1TAL6	Q10471	GALNT2	Polypeptide N-acetylgalactosaminyltransferase	Polypeptide N-acetylgalactosaminyltransferase 2	2	99	4	1.279	0.103	3.9E-02
G1T9P2		CARKD	ATP-dependent (S)-NAD(P)H-hydrate dehydratase		1		4	1.279	0.008	6.6E-03
G1SGR9	A0A494C1K3	GTF2I	General transcription factor IIi	General transcription factor II-I	2	97	4	1.278	0.033	8.8E-03
G1SIA3	Q8N3C0	ASCC3	Uncharacterized protein	Activating signal cointegrator 1 complex subunit 3	3	94	4	1.277	0.034	9.2E-03
G1SFG7	P48681	NES	Nestin	Nestin	2	60	4	1.276	0.219	1.9E-01
G1TBN5		TOR1AIP2	Torsin 1A interacting protein 2		1		4	1.276	0.073	2.1E-02
G1SEW3	P49792	RANBP2	RAN binding protein 2	E3 SUMO-protein ligase RanBP2	2	84	4	1.275	0.078	2.4E-02
G1U2J5	Q8NE01	CNNM3	Uncharacterized protein	Metal transporter CNNM3	3	88	4	1.275	0.044	1.1E-02
G1SZM2	A0A0A0MRG2	APP	Amyloid-beta A4 protein	Amyloid-beta precursor protein	2	99	4	1.275	0.016	7.4E-03
G1U797	P51114	FXR1	Uncharacterized protein	Fragile X mental retardation syndrome-related protein 1	3	91	4	1.275	0.047	1.2E-02
	P52815	MRPL12		39S ribosomal protein L12, mitochondrial	4		4	1.274	0.029	8.8E-03
G1U0B5	Q9Y570	PPME1	Protein phosphatase methylesterase 1	Protein phosphatase methylesterase 1	2	96	4	1.273	0.063	1.8E-02
Q9N0Z6	P05023	ATP1A1	Sodium/potassium-transporting ATPase subunit alpha-1	Sodium/potassium-transporting ATPase subunit alpha-1	2	98	4	1.272	0.025	8.5E-03
G1TLE4	P62879	GNB2	WD_REPEATS_REGION domain-containing protein	Guanine nucleotide-binding protein G(I)/G(S)/G(T) subunit beta-2	2	100	4	1.271	0.032	9.6E-03
G1STD4	Q9BSR8	YIPF4	Protein YIPF	Protein YIPF4	2	99	4	1.271	0.051	1.4E-02
G1TCT3	F8VPD4	CAD	Carbamoyl-phosphate synthetase 2, aspartate transcarbamylase, and dihydroorotase	CAD protein	2	94	4	1.271	0.055	1.5E-02
	H0Y5K5	ERGIC3		Endoplasmic reticulum-Golgi intermediate compartment protein 3 (Fragment)	4		4	1.270	0.071	2.2E-02
G1TUU9		TRIP12	Thyroid hormone receptor interactor 12		1		4	1.270	0.044	1.2E-02
G1TJR5	Q9NZL4	HSPBP1	HSPA (Hsp70) binding protein 1	Hsp70-binding protein 1	2	96	4	1.269	0.103	4.4E-02
G1SLT8	P31942	HNRNPH3	Uncharacterized protein	Heterogeneous nuclear ribonucleoprotein H3	3	100	4	1.269	0.044	1.2E-02
	F8VV64	TNS2		Tensin-2	4		4	1.268	0.162	1.2E-01
G1SU97	H0YD97	PDHX	Dihydrolipoamide acetyltransferase component of pyruvate dehydrogenase complex	Pyruvate dehydrogenase protein X component, mitochondrial (Fragment)	2	89	4	1.267	0.073	2.4E-02
G1SVU0	C9JAZ1	MTX2	Uncharacterized protein	Metaxin-2 (Fragment)	3	89	4	1.267	0.034	1.0E-02
G1SEQ2		PBDC1	Polysacc_synt_4 domain-containing protein		1		4	1.267	0.097	4.0E-02

G1THT8	J3QT54	CPSF7	Cleavage and polyadenylation specific factor 7	Cleavage and polyadenylation-specificity factor subunit 7 (Fragment)	2	95	4	1.266	0.087	3.3E-02
G1T8D7	Q8IUX7	AEBP1	F5/8 type C domain-containing protein	Adipocyte enhancer-binding protein 1	2	76	4	1.265	0.146	9.6E-02
G1TA37	O75531	BANF1	Uncharacterized protein	Barrier-to-autointegration factor	3	100	4	1.265	0.076	2.6E-02
G1TN86	C9IZG4	CUTA	Uncharacterized protein	Protein CutA	3	93	4	1.264	0.162	1.2E-01
G1SG63	P55011	SLC12A2	Uncharacterized protein	Solute carrier family 12 member 2	3	97	4	1.263	0.055	1.7E-02
	P56385	ATP5ME		ATP synthase subunit e, mitochondrial	4		4	1.262	0.045	1.4E-02
G1SYI3	A0A087X1B2	USP39	Uncharacterized protein	U4/U6.U5 tri-snRNP-associated protein 2	3	99	4	1.262	0.044	1.4E-02
G1T2V6	Q99747	NAPG	Uncharacterized protein	Gamma-soluble NSF attachment protein	3	98	4	1.259	0.056	1.8E-02
G1T0H0	Q9NX46	ADPRHL2	ADP-ribosylhydrolase like 2	ADP-ribose glycohydrolase ARH3	2	95	4	1.259	0.132	8.3E-02
G1SVY6	Q9BTX1	NDC1	Uncharacterized protein	Nucleoporin NDC1	3	88	4	1.259	0.046	1.5E-02
G1SIE6	P29279	CCN2	Cellular communication network factor 2	CCN family member 2	2	90	4	1.258	0.189	1.7E-01
G1TVT0	P19338	NCL	Nucleolin	Nucleolin	2	83	4	1.258	0.064	2.2E-02
	H0YEN5	RPS2		40S ribosomal protein S2 (Fragment)	4		4	1.258	0.164	1.3E-01
G1T1Y3	A0A384DVU0	PNPLA6	Patatin like phospholipase domain containing 6	Neuropathy target esterase	2	98	4	1.257	0.092	4.0E-02
G1SZ03	P55884	EIF3B	Eukaryotic translation initiation factor 3 subunit B	Eukaryotic translation initiation factor 3 subunit B	2	98	4	1.257	0.035	1.2E-02
G1SQ57	Q8N6T3	ARFGAP1	Arf-GAP domain-containing protein	ADP-ribosylation factor GTPase-activating protein 1	2	78	4	1.256	0.041	1.4E-02
U3KM96	P61224	RAP1B	Uncharacterized protein	Ras-related protein Rap-1b	3	100	4	1.255	0.019	1.0E-02
G1U998		NOL6	Nucleolar protein 6		1		4	1.254	0.162	1.3E-01
G1T0L9	P04843	RPN1	Dolichyl-diphosphooligosaccharide--protein glycosyltransferase subunit 1	Dolichyl-diphosphooligosaccharide--protein glycosyltransferase subunit 1	2	97	4	1.254	0.037	1.4E-02
	Q5T8U5	SURF4		Surfeit 4	4		4	1.254	0.083	3.5E-02
G1T2F2	P23284	PPIB	Peptidyl-prolyl cis-trans isomerase	Peptidyl-prolyl cis-trans isomerase B	2	94	4	1.253	0.065	2.4E-02
G1SF78	Q96RL7	VPS13A	Vacuolar protein sorting 13 homolog A	Vacuolar protein sorting-associated protein 13A	2	89	4	1.253	0.044	1.5E-02
	Q9NRP0	OSTC		Oligosaccharyltransferase complex subunit OSTC	4		4	1.252	0.102	5.2E-02
G1T568	Q92973	TNPO1	Transportin 1	Transportin-1	2	93	4	1.252	0.058	2.1E-02
G1T9J3	A2RRP1	NBAS	Uncharacterized protein	Neuroblastoma-amplified sequence	3	90	4	1.251	0.027	1.2E-02
G1U3G0	Q9C0C9	UBE2O	Ubiquitin conjugating enzyme E2 O	(E3-independent) E2 ubiquitin-conjugating enzyme	2	96	4	1.251	0.115	6.8E-02
G1U3V0	Q9BX68	HINT2	HIT domain-containing protein	Histidine triad nucleotide-binding protein 2, mitochondrial	2	94	4	1.251	0.080	3.4E-02
G1SNY3	Q68E01	INTS3	Uncharacterized protein	Integrator complex subunit 3	3	99	4	1.250	0.044	1.6E-02
G1T0U8	Q9UHG3	PCYOX1	Prenylcys_lyase domain-containing protein	Prenylcysteine oxidase 1	2	86	4	1.249	0.041	1.5E-02
G1SX71		AGPAT5	1-acylglycerol-3-phosphate O-acyltransferase 5		1		3	1.249	0.072	4.8E-02
G1T361	Q96I99	SUCLG2	Succinate--CoA ligase [GDP-forming] subunit beta, mitochondrial	Succinate--CoA ligase [GDP-forming] subunit beta, mitochondrial	2	97	4	1.249	0.105	5.9E-02
G1SQV5	O15371	EIF3D	Eukaryotic translation initiation factor 3 subunit D	Eukaryotic translation initiation factor 3 subunit D	2	99	4	1.248	0.033	1.4E-02
G1TIM0	G3V155	TMX2	Thioredoxin domain-containing protein	Thioredoxin domain containing 14, isoform CRA_a	2	94	4	1.248	0.052	2.0E-02
G1SZG3	Q8NEW0	SLC30A7	Uncharacterized protein	Zinc transporter 7	3	97	4	1.248	0.059	2.3E-02
G1T866	Q96CS3	FAF2	UBX domain-containing protein	FAS-associated factor 2	2	99	4	1.247	0.019	1.2E-02
G1SR63		PREB	WD_REPEATS_REGION domain-containing protein		1		4	1.246	0.059	2.4E-02
G1TDA9	O94766	B3GAT3	Galactosylgalactosylxylosylprotein 3-beta-glucuronosyltransferase	Galactosylgalactosylxylosylprotein 3-beta-glucuronosyltransferase 3	2	96	4	1.246	0.062	2.5E-02
B7NZM4	Q9Y295	DRG1	Developmentally regulated GTP binding protein 1 (Predicted)	Developmentally-regulated GTP-binding protein 1	2	100	4	1.246	0.038	1.5E-02
G1T2V2	D6RF62	PAICS	AIRC domain-containing protein	Multifunctional protein ADE2	2	93	4	1.246	0.078	3.5E-02
G1U4R5	A0A0C4DGS1	DDOST	Dolichyl-diphosphooligosaccharide--protein glycosyltransferase 48 kDa subunit	Dolichyl-diphosphooligosaccharide--protein glycosyltransferase 48 kDa subunit	2	94	4	1.246	0.027	1.3E-02
G1TAM8	P61289	PSME3	Uncharacterized protein	Proteasome activator complex subunit 3	3	95	4	1.244	0.050	2.0E-02
	Q9NZN4	EHD2		EH domain-containing protein 2	4		4	1.244	0.145	1.2E-01
G1TIS2		TIMM17B	Mitochondrial import inner membrane translocase subunit TIM17		1		4	1.244	0.064	2.7E-02
G1TUD2	Q9UBI6	GNG12	Guanine nucleotide-binding protein subunit gamma	Guanine nucleotide-binding protein G(I)/G(S)/G(O) subunit gamma-12	2	100	4	1.242	0.086	4.4E-02
G1T093	Q9NZJ4-2	SACS	Uncharacterized protein	Isoform 2 of Sacsin	3	96	4	1.241	0.062	2.7E-02
P20647	P16615	ATP2A2	Sarcoplasmic/endoplasmic reticulum calcium ATPase 2	Sarcoplasmic/endoplasmic reticulum calcium ATPase 2	2	98	4	1.241	0.052	2.2E-02
G1TKL0	M0QZG7	SNRPA	Small nuclear ribonucleoprotein polypeptide A	U1 small nuclear ribonucleoprotein A (Fragment)	2	60	4	1.241	0.176	1.7E-01
G1SK67	Q13547	HDAC1	Histone deacetylase	Histone deacetylase 1	2	99	4	1.240	0.043	1.9E-02
O77622		CCT6	T-complex protein 1 subunit zeta		1		4	1.240	0.093	5.2E-02
G1U7X2	Q15637	SF1	CCHC-type domain-containing protein	Splicing factor 1	2	99	4	1.240	0.023	1.4E-02
G1SPT2	P26196	DDX6	Uncharacterized protein	Probable ATP-dependent RNA helicase DDX6	3	99	4	1.238	0.068	3.2E-02
G1T7I3	Q13085	ACACA	Uncharacterized protein	Acetyl-CoA carboxylase 1	3	98	4	1.238	0.056	2.5E-02
G1SLE1		HARS2	AA_TRNA_LIGASE_II domain-containing protein		1		4	1.237	0.037	1.8E-02
G1SNM8	Q8N5M9	JAGN1	Uncharacterized protein	Protein jagunal homolog 1	3	96	4	1.236	0.051	2.3E-02
G1T8C2	F8W7U8	MRE11	Double-strand break repair protein	Double-strand break repair protein	2	93	4	1.236	0.106	6.9E-02
	A6XGL3	PRSS1		Protease serine 1	4		4	1.235	0.023	1.5E-02
G1TQ31	A0A2R8YD58	CSNK2A1	Casein kinase II subunit alpha	Casein kinase II subunit alpha	2	97	4	1.234	0.061	2.9E-02
G1SGX6		SDR39U1	DUF1731 domain-containing protein		1		4	1.234	0.114	8.1E-02
G1T6E9	Q8N5K1	CISD2	ZnF_CDGSH domain-containing protein	CDGSH iron-sulfur domain-containing protein 2	2	99	4	1.234	0.163	1.6E-01
G1SZD2	P53701	HCCS	Cytochrome c heme lyase	Cytochrome c-type heme lyase	2	83	4	1.233	0.043	2.1E-02
G1TBU9	A0A0B4J2A4	ACAA2	Uncharacterized protein	3-ketoacyl-CoA thiolase, mitochondrial	3	89	4	1.233	0.084	4.7E-02
G1SPB6	E7EPM6	ACSL1	AMP-binding domain-containing protein	Long-chain-fatty-acid--CoA ligase 1	2	80	4	1.233	0.038	1.9E-02
G1ST95	B3KS98	EIF3H	Eukaryotic translation initiation factor 3 subunit H	Eukaryotic translation initiation factor 3 subunit H	2	98	4	1.232	0.035	1.8E-02
G1SLS8	Q9NX62	IMPAD1	Uncharacterized protein	Inositol monophosphatase 3	3	96	4	1.232	0.036	1.9E-02
G1SSX2	Q7Z6Z7	HUWE1	HECT, UBA and WWE domain containing 1, E3 ubiquitin protein ligase	E3 ubiquitin-protein ligase HUWE1	2	96	4	1.231	0.072	3.8E-02
G1SRY7	K7ESP4	DCAKD	Uncharacterized protein	Dephospho-CoA kinase domain-containing protein (Fragment)	3	92	4	1.231	0.025	1.6E-02
G1SK22	P62979	RPS27A	Ubiquitin-like domain-containing protein	Ubiquitin-40S ribosomal protein S27a	2	100	4	1.230	0.075	4.1E-02
G1SSR8	O76031	CLPX	Uncharacterized protein	ATP-dependent Clp protease ATP-binding subunit clpX-like, mitochondrial	3	97	4	1.230	0.039	2.0E-02
G1U535	Q9HBH5	RDH14	Uncharacterized protein	Retinol dehydrogenase 14	3	91	4	1.229	0.097	6.4E-02

G1SP22	Q99943	AGPAT1	1-acyl-sn-glycerol-3-phosphate acyltransferase	1-acyl-sn-glycerol-3-phosphate acyltransferase alpha	2	98	4	1.229	0.190	2.1E-01
G1SMC8	Q14997	PSME4	Uncharacterized protein	Proteasome activator complex subunit 4	3	98	4	1.229	0.052	2.6E-02
	Q8NBJ5	COLGALT1		Procollagen galactosyltransferase 1	4		4	1.229	0.064	3.3E-02
G1TV43	O60218	AKR1B10	Aldo_ket_red domain-containing protein	Aldo-keto reductase family 1 member B10	2	86	4	1.228	0.197	2.2E-01
G1T2G4	P05198	EIF2S1	Eukaryotic translation initiation factor 2 subunit 1	Eukaryotic translation initiation factor 2 subunit 1	2	99	4	1.228	0.054	2.7E-02
G1SRE8		XRCC5	Ku domain-containing protein		1		4	1.228	0.050	2.5E-02
	O43752	STX6		Syntaxin-6	4		4	1.228	0.111	8.3E-02
G1TYV6	Q9HD20	ATP13A1	Cation-transporting ATPase	Manganese-transporting ATPase 13A1	2	95	4	1.227	0.062	3.2E-02
G1SZW5	Q8TD16	BICD2	Uncharacterized protein	Protein bicaudal D homolog 2	3	95	4	1.227	0.099	6.8E-02
G1TFM5	M0R0F0	RPS5	Ribosomal_S7 domain-containing protein	40S ribosomal protein S5 (Fragment)	2	100	4	1.226	0.098	6.9E-02
	A0A087WX29	TARDBP		TAR DNA-binding protein 43 (Fragment)	4		4	1.225	0.063	3.4E-02
G1SRZ8	A5YKK6	CNOT1	Uncharacterized protein	CCR4-NOT transcription complex subunit 1	3	100	4	1.224	0.026	1.8E-02
	E9PAV3-2	NACA		Isoform skNAC-2 of Nascent polypeptide-associated complex subunit alpha, muscle-specific form	4		4	1.224	0.036	2.1E-02
G1SH66		RBM3	RRM domain-containing protein		1		4	1.224	0.122	1.0E-01
G1T336	A0A0D9SF53	DDX3X	Uncharacterized protein	ATP-dependent RNA helicase DDX3X	3	99	4	1.223	0.030	2.0E-02
	P62701	RPS4X		40S ribosomal protein S4, X isoform	4		4	1.223	0.084	5.4E-02
G1SSL2	F8VXJ7	CNPY2	Saposin B-type domain-containing protein	Protein canopy homolog 2 (Fragment)	2	99	4	1.222	0.128	1.1E-01
G1SQL1	Q9HAU5	UPF2	Uncharacterized protein	Regulator of nonsense transcripts 2	3	97	4	1.221	0.034	2.2E-02
G1SWI3	P45880	VDAC2	Voltage-dependent anion-selective channel protein 2	Voltage-dependent anion-selective channel protein 2	2	99	4	1.221	0.061	3.5E-02
G1T9W3	K4DI93	CUL4B	CULLIN_2 domain-containing protein	Cullin 4B, isoform CRA_e	2	100	4	1.220	0.049	2.8E-02
G1TCB7	Q5LJA9	UCHL5	Ubiquitin carboxyl-terminal hydrolase	Ubiquitin carboxyl-terminal hydrolase (Fragment)	2	91	4	1.219	0.061	3.6E-02
G1U9S7	P17987	TCP1	Uncharacterized protein	T-complex protein 1 subunit alpha	3	97	4	1.219	0.052	3.1E-02
G1SCN8	P49368	CCT3	T-complex protein 1 subunit gamma	T-complex protein 1 subunit gamma	2	98	4	1.218	0.074	4.8E-02
G1SHF3		NIT1	CN hydrolase domain-containing protein		1		4	1.218	0.066	4.1E-02
G1TBC1	P14625	HSP90B1	Endoplasmin	Endoplasmin	2	94	4	1.217	0.107	8.8E-02
	O94973	AP2A2		AP-2 complex subunit alpha-2	4		4	1.217	0.122	1.1E-01
	A6NG10	WBP2		WW domain-binding protein 2	4		4	1.216	0.142	1.5E-01
G1SYD3	P54577	YARS	Tyrosine--tRNA ligase	Tyrosine--tRNA ligase, cytoplasmic	2	96	4	1.216	0.067	4.3E-02
G1T5Y2	Q9NUQ9	FAM49B	Uncharacterized protein	Protein FAM49B	3	100	4	1.216	0.049	3.0E-02
G1SFK3		RPA1	Replication protein A subunit		1		3	1.216	0.054	6.1E-02
G1STR6	Q96FN9	DTD2	Uncharacterized protein	D-aminoacyl-tRNA deacylase 2	3	93	4	1.215	0.119	1.1E-01
G1U724	Q9UBE0	SAE1	SUMO1 activating enzyme subunit 1	SUMO-activating enzyme subunit 1	2	88	4	1.214	0.061	3.9E-02
G1U9U0	P50991	CCT4	T-complex protein 1 subunit delta	T-complex protein 1 subunit delta	2	99	4	1.214	0.063	4.1E-02
G1SM24		MDN1	Midasin		1		3	1.213	0.094	9.5E-02
G1T3H5		EIF2B3	NTP_transferase domain-containing protein		1		4	1.213	0.062	4.1E-02
G1SVD7		SYNJ2BP-COX16	PDZ domain-containing protein		1		4	1.213	0.068	4.6E-02
G1TBW2	E7EQB8	IDH3G	Isocitrate dehydrogenase [NAD] subunit, mitochondrial	Isocitrate dehydrogenase [NAD] subunit, mitochondrial	2	94	4	1.213	0.064	4.2E-02
G1U3S3		KIAA2013	KIAA2013		1		4	1.212	0.036	2.6E-02
G1U7M0	Q96AJ9	VTI1A	t-SNARE coiled-coil homology domain-containing protein	Vesicle transport through interaction with t-SNAREs homolog 1A	2	94	4	1.212	0.122	1.2E-01
G1U0M5	H7C1W1	PXDN	Peroxidasin	Peroxidasin homolog (Fragment)	2	93	4	1.212	0.038	2.7E-02
	A0A0A0MTU3	TMEM259		Membralin	4		3	1.211	0.079	8.3E-02
G1T297	Q9UBV2	SEL1L	Fibronectin type-II domain-containing protein	Protein sel-1 homolog 1	2	97	4	1.211	0.026	2.3E-02
G1SEK1	P62140	PPP1CB	Serine/threonine-protein phosphatase	Serine/threonine-protein phosphatase PP1-beta catalytic subunit	2	100	4	1.210	0.135	1.4E-01
G1TY34		ITGA4	Integrin_alpha2 domain-containing protein		1		4	1.210	0.093	7.6E-02
G1SF82	P62072	TIMM10	zf-Tim10_DDP domain-containing protein	Mitochondrial import inner membrane translocase subunit Tim10	2	100	4	1.210	0.135	1.4E-01
G1TNU3		STX16	t-SNARE coiled-coil homology domain-containing protein		1		4	1.209	0.066	4.7E-02
G1SRI8	P19367	HK1	Uncharacterized protein	Hexokinase-1	3	96	4	1.209	0.071	5.2E-02
G1SD91	E9PFD2	UMPS	OMPdecase domain-containing protein	Uridine 5~-monophosphate synthase	2	92	3	1.209	0.101	1.1E-01
G1SDN3	K7EM18	EIF1	SUI1 domain-containing protein	Eukaryotic translation initiation factor 1	2	100	4	1.209	0.065	4.6E-02
G1U6B2		ALAD	Delta-aminolevulinic acid dehydratase		1		4	1.209	0.182	2.3E-01
G1SQU5	Q9UP95	SLC12A4	Solute carrier family 12 member 4	Solute carrier family 12 member 4	2	97	4	1.207	0.041	3.0E-02
G1T3H3	Q8IXI2	RHOT1	Mitochondrial Rho GTPase	Mitochondrial Rho GTPase 1	2	99	4	1.207	0.032	2.6E-02
G1TRG8	P04899	GNAI2	Uncharacterized protein	Guanine nucleotide-binding protein G(i) subunit alpha-2	3	98	4	1.207	0.037	2.8E-02
G1T2K6	Q8NBU5	ATAD1	AAA domain-containing protein	ATPase family AAA domain-containing protein 1	2	100	4	1.207	0.129	1.4E-01
G1U115	Q15008	PSMD6	PCI domain-containing protein	26S proteasome non-ATPase regulatory subunit 6	2	99	4	1.206	0.042	3.1E-02
G1T1U7	A0A494C1T2	MTHFD1	Uncharacterized protein	C-1-tetrahydrofolate synthase, cytoplasmic (Fragment)	3	90	4	1.206	0.077	6.0E-02
G1SYI2	P62873	GNB1	WD_REPEATS_REGION domain-containing protein	Guanine nucleotide-binding protein G(I)/G(S)/G(T) subunit beta-1	2	100	4	1.206	0.051	3.7E-02
G1STQ6	Q9Y617	PSAT1	Phosphoserine aminotransferase	Phosphoserine aminotransferase	2	94	4	1.206	0.167	2.1E-01
G1TWK1	E9PGZ4	SACM1L	SAC domain-containing protein	Phosphatidylinositide phosphatase SAC1	2	98	4	1.206	0.032	2.7E-02
	P30044	PRDX5		Peroxiredoxin-5, mitochondrial	4		4	1.205	0.040	3.1E-02
G1TKQ8	A0A0D9SG77	UBE3A	Ubiquitin-protein ligase E3A	Ubiquitin-protein ligase E3A	2	97	4	1.204	0.036	3.0E-02
G1SNI4	O94760	DDAH1	Uncharacterized protein	N(G),N(G)-dimethylarginine dimethylaminohydrolase 1	3	96	4	1.204	0.053	4.0E-02
G1SJN5		MAN2A1	Alpha-mannosidase		1		4	1.203	0.082	7.0E-02
U3KM62		PTGS2	Prostaglandin G/H synthase 2		1		4	1.203	0.058	4.4E-02
G1U4Y5		FCGRT	Fc fragment of IgG receptor and transporter		1		4	1.201	0.117	1.2E-01
G1T520	Q99615	DNAJC7	Uncharacterized protein	DnaJ homolog subfamily C member 7	3	98	4	1.201	0.146	1.8E-01
G1SYE7	H0Y8R1	GRSF1	Uncharacterized protein	G-rich sequence factor 1 (Fragment)	3	96	4	1.201	0.088	7.9E-02
G1TEK3	Q8IWJ2	GCC2	GRIP domain-containing protein	GRIP and coiled-coil domain-containing protein 2	2	86	4	1.200	0.080	7.0E-02
G1SSX5	P56192	MARS	Uncharacterized protein	Methionine--tRNA ligase, cytoplasmic	3	93	4	1.199	0.070	5.9E-02

G1TE39	Q9NYU1	UGGT2	Uncharacterized protein	UDP-glucose:glycoprotein glucosyltransferase 2	3	84	4	1.199	0.059	4.8E-02
G1SWN7	Q4G0N4	NADK2	NAD kinase 2, mitochondrial	NAD kinase 2, mitochondrial	2	92	4	1.198	0.069	5.9E-02
	Q8NHH9-2	ATL2		Isoform 2 of Atlastin-2	4		4	1.197	0.097	9.6E-02
G1T9R8	Q16222	UAP1	Uncharacterized protein	UDP-N-acetylhexosamine pyrophosphorylase	3	96	4	1.195	0.072	6.5E-02
	P13639	EEF2		Elongation factor 2	4		4	1.195	0.077	7.1E-02
G1TCY7		EIF2B4	Translation initiation factor eIF-2B subunit delta		1		4	1.195	0.056	4.9E-02
G1SY19	O15118	NPC1	SSD domain-containing protein	NPC intracellular cholesterol transporter 1	2	91	4	1.195	0.175	2.4E-01
G1T9L6		TOR1AIP1	Torsin 1A interacting protein 1		1		4	1.194	0.092	9.3E-02
G1SI95	O00291	HIP1	Uncharacterized protein	Huntingtin-interacting protein 1	3	91	4	1.193	0.047	4.2E-02
G1TLK9	P49748	ACADVL	Uncharacterized protein	Very long-chain specific acyl-CoA dehydrogenase, mitochondrial	3	87	4	1.193	0.055	4.9E-02
G1SRA8	P41091	EIF2S3	Eukaryotic translation initiation factor 2 subunit 3	Eukaryotic translation initiation factor 2 subunit 3	2	92	4	1.193	0.071	6.7E-02
G1SZI0		SPG7	SPG7, paraplegin matrix AAA peptidase subunit		1		4	1.191	0.017	3.0E-02
G1SCE7	P55084	HADHB	Uncharacterized protein	Trifunctional enzyme subunit beta, mitochondrial	3	94	4	1.191	0.068	6.4E-02
G1U9T1	Q99832	CCT7	T-complex protein 1 subunit eta	T-complex protein 1 subunit eta	2	97	4	1.191	0.066	6.2E-02
G1TMD8		PPP5C	Serine/threonine-protein phosphatase		1		4	1.191	0.054	5.0E-02
	H3BQQ9	UBE2I		SUMO-conjugating enzyme UBC9 (Fragment)	4		4	1.191	0.072	7.0E-02
G1SP97	P51884	LUM	Lumican	Lumican	2	91	4	1.190	0.278	4.4E-01
G1SFS8	Q7KZF4	SND1	Staphylococcal nuclease domain-containing protein	Staphylococcal nuclease domain-containing protein 1	2	97	4	1.189	0.033	3.7E-02
G1SE56	F6T1Q0	PDE12	Endo/exonuclease/phosphatase domain-containing protein	2~,5~-phosphodiesterase 12	2	90	4	1.188	0.040	4.1E-02
G1SZ66	I3L295	MPDU1	Uncharacterized protein	Mannose-P-dolichol utilization defect 1 isoform 2	3	89	4	1.188	0.028	3.5E-02
	P29992	GNA11		Guanine nucleotide-binding protein subunit alpha-11	4		4	1.188	0.022	3.3E-02
G1SNZ3	P55060	CSE1L	Chromosome segregation 1 like	Exportin-2	2	99	4	1.186	0.078	8.2E-02
G1TAI0		KPNA2	Importin subunit alpha		1		4	1.185	0.081	8.7E-02
	Q92900	UPF1		Regulator of nonsense transcripts 1	4		4	1.185	0.033	3.9E-02
G1SWU1	P55809	OXCT1	Succinyl-CoA:3-ketoacid-coenzyme A transferase	Succinyl-CoA:3-ketoacid coenzyme A transferase 1, mitochondrial	2	94	4	1.185	0.138	1.9E-01
G1SKF1	P07996	THBS1	Uncharacterized protein	Thrombospondin-1	3	97	4	1.184	0.151	2.2E-01
G1T7I8		RIC8A	RIC8 guanine nucleotide exchange factor A		1		4	1.184	0.077	8.3E-02
G1SLM1	Q9HB71	CACYBP	Uncharacterized protein	Calcyclin-binding protein	3	92	4	1.182	0.103	1.3E-01
G1SR03	P55072	VCP	Uncharacterized protein	Transitional endoplasmic reticulum ATPase	3	100	4	1.182	0.042	4.7E-02
G1T2N8	O43592	XPOT	Exportin-T	Exportin-T	2	99	4	1.182	0.068	7.3E-02
G1SZ37	P31937	HIBADH	3-hydroxyisobutyrate dehydrogenase	3-hydroxyisobutyrate dehydrogenase, mitochondrial	2	96	4	1.182	0.085	9.9E-02
G1STE3	A0A0A0MQX8	MBNL1	Uncharacterized protein	Muscleblind-like protein 1	3	100	4	1.182	0.039	4.6E-02
G1SGM2	O14744	PRMT5	Protein arginine N-methyltransferase 5	Protein arginine N-methyltransferase 5	2	98	4	1.181	0.062	6.8E-02
P29694	P26641	EEF1G	Elongation factor 1-gamma	Elongation factor 1-gamma	2	98	4	1.181	0.087	1.0E-01
G1T840	Q92598	HSPH1	Uncharacterized protein	Heat shock protein 105 kDa	3	96	4	1.181	0.031	4.1E-02
	G8JLG1	SMC1A		Structural maintenance of chromosomes protein	4		4	1.181	0.076	8.5E-02
G1SW89	Q9C0E8	LNPK	zinc_ribbon_10 domain-containing protein	Endoplasmic reticulum junction formation protein lunapark	2	89	4	1.181	0.055	6.0E-02
G1U4C2	Q76M96	CCDC80	Uncharacterized protein	Coiled-coil domain-containing protein 80	3	86	4	1.181	0.137	2.0E-01
G1TRL5	A0A087WUT6	EIF5B	Tr-type G domain-containing protein	Eukaryotic translation initiation factor 5B	2	95	4	1.181	0.048	5.3E-02
G1SV05	P34932	HSPA4	Uncharacterized protein	Heat shock 70 kDa protein 4	3	97	4	1.180	0.054	6.0E-02
G1T0F6	Q9UHB9	SRP68	Signal recognition particle subunit SRP68	Signal recognition particle subunit SRP68	2	95	4	1.180	0.036	4.6E-02
G1TWP4	A0A140T936	VARS	GST C-terminal domain-containing protein	Valine--tRNA ligase (Fragment)	2	92	4	1.180	0.049	5.6E-02
G1U1E6		GCSH	Glycine cleavage system H protein		1		3	1.179	0.099	1.5E-01
G1TAW7		EIF2A	Eukaryotic translation initiation factor 2A		1		4	1.178	0.106	1.4E-01
G1T798	Q12846	STX4	t-SNARE coiled-coil homology domain-containing protein	Syntaxin-4	2	96	4	1.178	0.062	7.1E-02
G1SHK7	A0A0C4DGV4	LAMTOR5	Uncharacterized protein	Hepatitis B virus x interacting protein	3	100	4	1.177	0.020	3.9E-02
G1TFB5	Q9NP72	RAB18	Uncharacterized protein	Ras-related protein Rab-18	3	98	4	1.177	0.043	5.2E-02
G1SQY8	Q9Y266	NUDC	CS domain-containing protein	Nuclear migration protein nudC	2	97	4	1.175	0.088	1.1E-01
G1TNW8	Q9Y512	SAMM50	SAMM50 sorting and assembly machinery component	Sorting and assembly machinery component 50 homolog	2	96	4	1.175	0.067	8.1E-02
G1SPG2		TRIP11	GRIP domain-containing protein		1		4	1.174	0.057	7.0E-02
G1SXX5	A0A2R8Y4F5	HADHA	Uncharacterized protein	Trifunctional enzyme subunit alpha, mitochondrial	3	86	4	1.174	0.052	6.5E-02
G1TBX7	P11233	RALA	Uncharacterized protein	Ras-related protein Ral-A	3	100	4	1.174	0.055	6.8E-02
G1SQ93	Q9UKV8	AGO2	Protein argonaute-2	Protein argonaute-2	2	98	4	1.173	0.032	4.8E-02
	P62166	NCS1		Neuronal calcium sensor 1	4		4	1.173	0.135	2.1E-01
G1SKE2	E7ETC0	TIAL1	TIA1 cytotoxic granule associated RNA binding protein like 1	Nucleolysin TIAR	2	98	4	1.173	0.055	6.9E-02
G1SPB2		RNMT	mRNA cap guanine-N7 methyltransferase		1		4	1.172	0.059	7.5E-02
G1SGQ0	Q9UBS4	DNAJB11	J domain-containing protein	DnaJ homolog subfamily B member 11	2	98	4	1.171	0.072	9.3E-02
G1SVT4	P51665	PSMD7	MPN domain-containing protein	26S proteasome non-ATPase regulatory subunit 7	2	99	4	1.171	0.096	1.3E-01
G1SME1		NOP14	NOP14 nucleolar protein		1		4	1.171	0.092	1.3E-01
G1SJH8	K7EP90	RBM42	RRM domain-containing protein	RNA-binding protein 42	2	97	4	1.171	0.065	8.3E-02
G1SVJ8	Q96Q05	TRAPPC9	Uncharacterized protein	Trafficking protein particle complex subunit 9	3	87	4	1.171	0.102	1.5E-01
G1T108	Q9NRG9	AAAS	WD_REPEATS_REGION domain-containing protein	Aladin	2	94	4	1.170	0.115	1.7E-01
G1TWC3		TMX1	Thioredoxin domain-containing protein		1		4	1.169	0.062	8.2E-02
G1T1C4		HSD17B7	Hydroxysteroid 17-beta dehydrogenase 7		1		4	1.167	0.047	6.8E-02
G1SJU1	O75051	PLXNA2	Sema domain-containing protein	Plexin-A2	2	98	4	1.166	0.062	8.6E-02
G1T6P0	Q9H7D0	DOCK5	Dedicator of cytokinesis 5	Dedicator of cytokinesis protein 5	2	96	4	1.165	0.110	1.7E-01
U3KN22	Q9Y2Q3	GSTK1	Glutathione S-transferase kappa	Glutathione S-transferase kappa 1	2	79	4	1.165	0.083	1.2E-01
G1SI54		ILVBL	IlvB acetolactate synthase like		1		4	1.164	0.020	5.1E-02
G1TBR5	B4DR61	SEC61A1	Plug_translocon domain-containing protein	Protein transport protein Sec61 subunit alpha isoform 1	2	100	4	1.164	0.047	7.1E-02

G1T103	F8VQQ3	C12orf10	Uncharacterized protein	UPF0160 protein MYG1, mitochondrial	3	77	4	1.164	0.049	7.4E-02
G1SJK0		APOOL	MICOS complex subunit		1		4	1.164	0.029	5.6E-02
G1SES2		NAA10	N-acetyltransferase domain-containing protein		1		4	1.163	0.091	1.4E-01
Q01971	P61019	RAB2A	Ras-related protein Rab-2A	Ras-related protein Rab-2A	2	100	4	1.163	0.017	5.1E-02
G1SY93	P63000	RAC1	Rac family small GTPase 1	Ras-related C3 botulinum toxin substrate 1	2	90	4	1.163	0.081	1.2E-01
G1T810	A0A0A0MTN0	CUL2	CULLIN_2 domain-containing protein	Cullin-2	2	99	4	1.163	0.022	5.3E-02
G1ST38	Q9Y673	ALG5	ALG5, dolichyl-phosphate beta-glucosyltransferase	Dolichyl-phosphate beta-glucosyltransferase	2	93	4	1.162	0.075	1.1E-01
G1SRP2	Q5SWX8	ODR4	Uncharacterized protein	Protein odr-4 homolog	3	89	4	1.162	0.056	8.4E-02
G1T5N5	C9IZ01	GFM1	Elongation factor G, mitochondrial	Elongation factor G, mitochondrial	2	93	4	1.162	0.070	1.1E-01
O46638	Q00688	FKBP3	Peptidyl-prolyl cis-trans isomerase FKBP3	Peptidyl-prolyl cis-trans isomerase FKBP3	2	96	4	1.161	0.123	2.1E-01
G1SZ59	P60842	EIF4A1	Eukaryotic initiation factor 4A-I	Eukaryotic initiation factor 4A-I	2	100	4	1.160	0.052	8.1E-02
	O43290	SART1		U4/U6.U5 tri-snRNP-associated protein 1	4		4	1.160	0.065	1.0E-01
P00169	P00167	CYB5A	Cytochrome b5	Cytochrome b5	2	90	4	1.160	0.066	1.0E-01
G1SVV2	Q9NZM1	MYOF	Uncharacterized protein	Myoferlin	3	94	4	1.160	0.031	6.1E-02
G1TQ79		CHCHD6	MICOS complex subunit		1		4	1.160	0.076	1.2E-01
G1T146	E7ESY4	MTA1	Metastasis associated 1	Metastasis-associated protein MTA1	2	88	4	1.160	0.168	3.1E-01
G1T8Y0		COASY	CTP_transf_like domain-containing protein		1		4	1.160	0.069	1.1E-01
G1SIB1	P29083	GTF2E1	HTH TFE/IIEalpha-type domain-containing protein	General transcription factor IIE subunit 1	2	94	4	1.160	0.073	1.1E-01
G1SJE4	Q92556	ELMO1	ELMO domain-containing protein	Engulfment and cell motility protein 1	2	97	4	1.159	0.036	6.6E-02
G1TZC9	A0A087WXU3	ESYT2	Extended synaptotagmin 2	Extended synaptotagmin-2	2	92	4	1.159	0.017	5.5E-02
G1SKF5		ERBIN	Erbb2 interacting protein		1		4	1.158	0.050	8.2E-02
	A0A0A0MRA3	TTN		Titin	4		4	1.157	0.029	6.3E-02
G1T7Y5	H0Y368	DPM1	Dolichol-phosphate mannosyltransferase subunit 1	Dolichol-phosphate mannosyltransferase subunit 1 (Fragment)	2	83	4	1.157	0.055	9.0E-02
G1SUP8	P67812	SEC11A	Signal peptidase complex catalytic subunit SEC11	Signal peptidase complex catalytic subunit SEC11A	2	100	4	1.157	0.032	6.6E-02
P19943	P05387	RPLP2	60S acidic ribosomal protein P2 (Fragment)	60S acidic ribosomal protein P2	2	100	4	1.157	0.111	1.9E-01
P43236	P43235	CTSK	Cathepsin K	Cathepsin K	2	94	4	1.156	0.072	1.2E-01
G1SM82	E9PGC0	RASA1	Uncharacterized protein	Ras GTPase-activating protein 1	3	96	4	1.156	0.052	8.7E-02
G1T312		ACAD8	Acyl-CoA dehydrogenase family member 8		1		4	1.156	0.071	1.2E-01
G1SRP7	O00505	KPNA3	Importin subunit alpha	Importin subunit alpha-4	2	99	4	1.156	0.129	2.3E-01
G1T3S4		MECR	PKS_ER domain-containing protein		1		4	1.155	0.091	1.5E-01
	P30533	LRPAP1		Alpha-2-macroglobulin receptor-associated protein	4		4	1.154	0.114	2.1E-01
G1T242		IKBIP	IKBKB interacting protein		1		4	1.152	0.042	8.2E-02
G1TBL1	C9JPE1	SLC25A20	Uncharacterized protein	Mitochondrial carnitine/acylcarnitine carrier protein	3	92	4	1.152	0.113	2.1E-01
G1T6G1		MMAB	Corrinoid adenosyltransferase		1		4	1.152	0.100	1.8E-01
G1SYR5		EIF2B2	Translation initiation factor eIF-2B subunit beta		1		4	1.152	0.114	2.1E-01
G1TMS5	P48643	CCT5	Uncharacterized protein	T-complex protein 1 subunit epsilon	3	99	4	1.152	0.073	1.3E-01
G1TCZ8	P30153	PPP2R1A	Protein phosphatase 2 scaffold subunit Aalpha	Serine/threonine-protein phosphatase 2A 65 kDa regulatory subunit A alpha isoform	2	90	4	1.151	0.174	3.5E-01
G1TQA4		EPM2AIP1	EPM2A interacting protein 1		1		4	1.149	0.040	8.5E-02
G1TFL3	J3KQE5	RAN	GTP-binding nuclear protein Ran	GTP-binding nuclear protein Ran (Fragment)	2	96	4	1.149	0.083	1.5E-01
B6V9S9	P78371	CCT2	Chaperonin-containing T-complex polypeptide beta subunit	T-complex protein 1 subunit beta	2	99	4	1.148	0.062	1.2E-01
G1SEF8	M0R0Y2	NAPA	Uncharacterized protein	Alpha-soluble NSF attachment protein	3	84	4	1.148	0.024	7.3E-02
G1SP21	H0Y9A1	YIPF3	Uncharacterized protein	Protein YIPF3 (Fragment)	3	72	4	1.147	0.041	8.7E-02
G1SGL4	Q9Y276	BCS1L	Uncharacterized protein	Mitochondrial chaperone BCS1	3	96	4	1.147	0.081	1.5E-01
G1SGM3	A0A0J9YXF2	PON2	Uncharacterized protein	Paraoxonase 2, isoform CRA_a	3	93	4	1.147	0.044	9.3E-02
G1TKG2		ISOC2	Isochorismatase domain containing 2		1		4	1.147	0.034	8.2E-02
G1TP30	H0Y4Q3	RANGAP1	RanGAP1_C domain-containing protein	Ran GTPase-activating protein 1 (Fragment)	2	80	4	1.145	0.086	1.7E-01
G1SQW0	E9PIE4	MTCH2	Uncharacterized protein	Mitochondrial carrier homolog 2 (Fragment)	3	92	4	1.145	0.129	2.6E-01
G1ST56	Q96P70	IPO9	Importin N-terminal domain-containing protein	Importin-9	2	99	4	1.145	0.035	8.5E-02
G1T550	P51148	RAB5C	Uncharacterized protein	Ras-related protein Rab-5C	3	91	4	1.144	0.060	1.2E-01
	Q96HP0	DOCK6		Dedicator of cytokinesis protein 6	4		4	1.143	0.099	2.0E-01
G1SQT0	P60510	PPP4C	Serine/threonine-protein phosphatase	Serine/threonine-protein phosphatase 4 catalytic subunit	2	100	4	1.143	0.036	9.0E-02
G1SXJ6		CUL4A	CULLIN_2 domain-containing protein		1		4	1.142	0.046	1.0E-01
G1SLF5	H0YG54	REXO2	Exonuclease domain-containing protein	Oligoribonuclease, mitochondrial	2	98	4	1.142	0.166	3.6E-01
G1T573	H0Y3P2	EIF4G2	Eukaryotic translation initiation factor 4 gamma 2	Eukaryotic translation initiation factor 4 gamma 2	2	95	4	1.142	0.066	1.3E-01
G1TDN6		KRT5	IF rod domain-containing protein		1		4	1.141	0.238	5.0E-01
G1TM48	Q5JRA6	MIA3	SH3 domain-containing protein	Transport and Golgi organization protein 1 homolog	2	71	4	1.141	0.107	2.2E-01
	Q9UNK0	STX8		Syntaxin-8	4		4	1.140	0.047	1.1E-01
G1SU30	A0A024RCR6	BAG6	Ubiquitin-like domain-containing protein	BAG6	2	91	4	1.140	0.053	1.2E-01
G1SUR8	B7ZBJ4	CAB39L	Uncharacterized protein	Calcium-binding protein 39-like	3	98	4	1.140	0.116	2.5E-01
G1SQ38	E5RHK8	DNM3	Uncharacterized protein	Dynamin-3	3	97	4	1.139	0.163	3.6E-01
	E7EVJ3	NDST1		Bifunctional heparan sulfate N-deacetylase/N-sulfotransferase 1	4		4	1.139	0.109	2.3E-01
G1T7Q3	F5H157	RAB35	Uncharacterized protein	Ras-related protein Rab-35 (Fragment)	3	91	4	1.139	0.049	1.1E-01
B7NZS4	O75175	CNOT3	CCR4-NOT transcription complex, subunit 3 (Predicted)	CCR4-NOT transcription complex subunit 3	2	97	4	1.139	0.071	1.5E-01
G1SQK0	A0A087X1A5	STAU1	Uncharacterized protein	Double-stranded RNA-binding protein Staufen homolog 1	3	89	4	1.138	0.093	2.0E-01
G1T601		CLYBL	HpcH_HpaI domain-containing protein		1		4	1.138	0.093	2.0E-01
G1T2C3	O75534	CSDE1	Uncharacterized protein	Cold shock domain-containing protein E1	3	99	4	1.138	0.017	8.3E-02
G1SLA2	Q92604	LPGAT1	Lysophosphatidylglycerol acyltransferase 1	Acyl-CoA:lysophosphatidylglycerol acyltransferase 1	2	94	4	1.138	0.106	2.3E-01
G1SMK9	Q9ULC3	RAB23	Uncharacterized protein	Ras-related protein Rab-23	3	97	4	1.137	0.112	2.5E-01
G1TA10		TMEM109	Transmembrane protein 109		1		4	1.137	0.133	3.0E-01

G1T193		UGT3A2	UDP-glucuronosyltransferase		1		4	1.136	0.031	9.7E-02
G1SMW3	Q92615	LARP4B	La ribonucleoprotein domain family member 4B	La-related protein 4B	2	88	4	1.136	0.058	1.3E-01
G1SDT9	P49754	VPS41	Vacuolar protein sorting-associated protein 41 homolog	Vacuolar protein sorting-associated protein 41 homolog	2	98	4	1.135	0.069	1.6E-01
	Q15366-3	PCBP2		Isoform 3 of Poly(rC)-binding protein 2	4		4	1.135	0.166	3.8E-01
G1T3Q2	Q96HY6	DDRGK1	Uncharacterized protein	DDRGK domain-containing protein 1	3	90	4	1.134	0.053	1.3E-01
G1T364	Q8NDH3	NPEPL1	Aminopeptidase like 1	Probable aminopeptidase NPEPL1	2	90	4	1.134	0.146	3.4E-01
G1TH06	F6WQW2	RANBP1	RAN binding protein 1	Ran-specific GTPase-activating protein	2	91	4	1.133	0.082	1.9E-01
G1SKK5		PEX6	Peroxisomal biogenesis factor 6		1		4	1.133	0.029	1.0E-01
G1SVM1	Q14764	MVP	Uncharacterized protein	Major vault protein	3	91	4	1.133	0.052	1.3E-01
G1SV51	Q08378	GOLGA3	Golgin A3	Golgin subfamily A member 3	2	85	4	1.132	0.038	1.1E-01
G1SQR7	P61970	NUTF2	NTF2 domain-containing protein	Nuclear transport factor 2	2	100	4	1.132	0.176	4.1E-01
G1SEV7	Q9NX55	HYPK	Uncharacterized protein	Huntingtin-interacting protein K	3	98	4	1.132	0.050	1.3E-01
G1TKE0	Q9P2X0	DPM3	Dolichol-phosphate mannosyltransferase subunit 3	Dolichol-phosphate mannosyltransferase subunit 3	2	95	4	1.132	0.085	2.0E-01
U3KMP1	P61026	RAB10	Uncharacterized protein	Ras-related protein Rab-10	3	100	4	1.131	0.100	2.3E-01
G1T9F3	Q14974	KPNB1	Importin N-terminal domain-containing protein	Importin subunit beta-1	2	99	4	1.130	0.051	1.4E-01
G1TBK0		ANKMY2	Ankyrin repeat and MYND domain containing 2		1		4	1.129	0.041	1.2E-01
P30946	P07900	HSP90AA1	Heat shock protein HSP 90-alpha	Heat shock protein HSP 90-alpha	2	94	4	1.129	0.073	1.8E-01
G1SG42	Q52LJ0	FAM98B	Uncharacterized protein	Protein FAM98B	3	94	4	1.129	0.044	1.3E-01
G1TAN9	P21399	ACO1	Cytoplasmic aconitate hydratase	Cytoplasmic aconitate hydratase	2	93	4	1.128	0.103	2.5E-01
G1SG68	O00629	KPNA4	Importin subunit alpha	Importin subunit alpha-3	2	99	4	1.128	0.012	1.0E-01
G1SJ37	F8W9S7	GAPVD1	Uncharacterized protein	GTPase-activating protein and VPS9 domain-containing protein 1	3	95	4	1.127	0.032	1.2E-01
G1SMI6	Q13162	PRDX4	Thioredoxin domain-containing protein	Peroxiredoxin-4	2	96	4	1.127	0.043	1.3E-01
G1SCS8		ANO6	Anoctamin		1		4	1.127	0.065	1.7E-01
G1T3L5	O43719	HTATSF1	Uncharacterized protein	HIV Tat-specific factor 1	3	82	4	1.126	0.091	2.3E-01
G1TX74	Q6IBS0	TWF2	Uncharacterized protein	Twinfilin-2	3	95	4	1.126	0.039	1.3E-01
G1SNZ8		GORASP1	GRASP55_65 domain-containing protein		1		4	1.126	0.085	2.2E-01
G1T501		CCAR2	Cell cycle and apoptosis regulator 2		1		4	1.125	0.076	2.0E-01
G1SXB8	Q10567	AP1B1	AP complex subunit beta	AP-1 complex subunit beta-1	2	98	4	1.125	0.044	1.4E-01
G1T9V2	A0A2R8Y852	CUX1	Cut like homeobox 1	Homeobox protein cut-like	2	87	4	1.125	0.103	2.6E-01
G1SYS5	A0A1B0GW77	ALDH7A1	Aldedh domain-containing protein	Alpha-aminoadipic semialdehyde dehydrogenase	2	93	4	1.124	0.034	1.3E-01
G1SZF9	O75976	CPD	Uncharacterized protein	Carboxypeptidase D	3	91	4	1.123	0.197	4.8E-01
G1SYR9	B9A067	IMMT	MICOS complex subunit MIC60	MICOS complex subunit MIC60	2	89	4	1.123	0.078	2.1E-01
G1T006	Q9UMX5	NENF	Neudesin neurotrophic factor	Neudesin	2	98	4	1.122	0.144	3.7E-01
G1SDA8	P25786	PSMA1	Proteasome endopeptidase complex	Proteasome subunit alpha type-1	2	100	4	1.122	0.035	1.3E-01
G1U0T4		HSD17B14	Hydroxysteroid 17-beta dehydrogenase 14		1		4	1.122	0.093	2.5E-01
G1SU24	O60503	ADCY9	Adenylate cyclase 9	Adenylate cyclase type 9	2	92	3	1.122	0.138	3.6E-01
G1T6M2		DHRS7B	Dehydrogenase/reductase 7B		1		4	1.122	0.008	1.1E-01
G1TMQ8	H0Y9V7	ATP2C1	Calcium-transporting ATPase	Calcium-transporting ATPase type 2C member 1 (Fragment)	2	99	4	1.121	0.114	3.0E-01
G1SLD5	A0A087X054	HYOU1	Hypoxia up-regulated 1	Hypoxia up-regulated protein 1	2	87	4	1.121	0.026	1.2E-01
G1SYL8	I3L0M9	ELOB	Elongin B	Elongin-B (Fragment)	2	82	4	1.121	0.087	2.3E-01
Q9TT13	Q9Y277	VDAC3	Voltage-dependent anion-selective channel protein 3	Voltage-dependent anion-selective channel protein 3	2	98	4	1.121	0.037	1.4E-01
G1T7L7		KDELC2	CAP10 domain-containing protein		1		4	1.121	0.073	2.0E-01
G1SPJ5	Q92616	GCN1	TOG domain-containing protein	eIF-2-alpha kinase activator GCN1	2	96	4	1.121	0.086	2.3E-01
G1U5Z2		TK2	dNK domain-containing protein		1		4	1.120	0.034	1.4E-01
G1U8V2	Q96DZ1	ERLEC1	Uncharacterized protein	Endoplasmic reticulum lectin 1	3	98	4	1.120	0.128	3.5E-01
G1SIJ8	Q8TEX9	IPO4	Importin N-terminal domain-containing protein	Importin-4	2	89	4	1.119	0.054	1.7E-01
	A0A0G2JL54	C4B_2		Complement C4-B	4		4	1.119	0.225	5.4E-01
G1SWR1	O43818	RRP9	WD_REPEATS_REGION domain-containing protein	U3 small nucleolar RNA-interacting protein 2	2	94	4	1.119	0.029	1.3E-01
G1SPG6	A6NEM5	PIGK	GPI-anchor transamidase	GPI-anchor transamidase	2	85	4	1.119	0.048	1.6E-01
Q28611		UGT1	UDP-glucuronosyltransferase 1-6		1		4	1.118	0.050	1.7E-01
G1TA11	P54136	RARS	Uncharacterized protein	Arginine--tRNA ligase, cytoplasmic	3	92	4	1.117	0.047	1.6E-01
G1T643	A0A1W2PNX8	UNC45A	Unc-45 myosin chaperone A	Protein unc-45 homolog A	2	94	4	1.117	0.033	1.4E-01
G1TKY7		OGFOD3	2-oxoglutarate and iron dependent oxygenase domain containing 3		1		4	1.117	0.061	1.9E-01
G1TQR2	P24534	EEF1B2	Uncharacterized protein	Elongation factor 1-beta	3	98	4	1.116	0.087	2.5E-01
	M0QYZ2	AP2S1		AP complex subunit sigma	4		4	1.116	0.081	2.4E-01
G1TD36	Q6PML9	SLC30A9	Uncharacterized protein	Zinc transporter 9	3	96	4	1.116	0.136	3.8E-01
P62493	P62491	RAB11A	Ras-related protein Rab-11A	Ras-related protein Rab-11A	2	100	4	1.116	0.029	1.4E-01
G1SJX1	Q16537	PPP2R5E	Serine/threonine-protein phosphatase 2A 56 kDa regulatory subunit	Serine/threonine-protein phosphatase 2A 56 kDa regulatory subunit epsilon isoform	2	100	4	1.115	0.048	1.7E-01
G1TPM1	Q96SB3	PPP1R9B	PDZ domain-containing protein	Neurabin-2	2	99	4	1.115	0.040	1.6E-01
G1T2R2	A0A3B3ITU8	ITPR1	Uncharacterized protein	Inositol 1,4,5-trisphosphate receptor type 1	3	97	3	1.114	0.097	3.1E-01
	Q9H0U4	RAB1B		Ras-related protein Rab-1B	4		4	1.114	0.051	1.8E-01
G1TY83		NLRX1	NLR family member X1		1		4	1.113	0.070	2.2E-01
G1SER3	Q02978	SLC25A11	Uncharacterized protein	Mitochondrial 2-oxoglutarate/malate carrier protein	3	97	4	1.113	0.057	2.0E-01
G1T7P8	Q9UIV1	CNOT7	Uncharacterized protein	CCR4-NOT transcription complex subunit 7	3	100	4	1.112	0.086	2.6E-01
G1U222	A0A384DVK7	ARHGEF10L	Rho guanine nucleotide exchange factor 10 like	Rho guanine nucleotide exchange factor 10-like protein (Fragment)	2	93	4	1.112	0.046	1.8E-01
G1T329	J3QQY1	CDK5RAP3	Uncharacterized protein	CDK5 regulatory subunit-associated protein 3 (Fragment)	3	88	4	1.112	0.083	2.6E-01
G1TW04	Q16643	DBN1	Drebrin 1	Drebrin	2	63	4	1.111	0.051	1.9E-01
	Q9NWU2	GID8		Glucose-induced degradation protein 8 homolog	4		4	1.111	0.090	2.8E-01
G1TEB0	Q9UKX5	ITGA11	VWFA domain-containing protein	Integrin alpha-11	2	91	4	1.110	0.094	2.9E-01

G1TRV7		QPCTL	Glutaminyl-peptide cyclotransferase like		1		4	1.110	0.051	2.0E-01
G1TGY9		VPS11	VPS11, CORVET/HOPS core subunit		1		4	1.109	0.040	1.8E-01
G1U013	H7C3P9	COPS3	PCI domain-containing protein	COP9 signalosome complex subunit 3	2	92	4	1.109	0.046	1.9E-01
G1SGS7	O94874	UFL1	Uncharacterized protein	E3 UFM1-protein ligase 1	3	94	4	1.109	0.046	1.9E-01
G1SNP4	A0A3B3ITZ9	THRAP3	Uncharacterized protein	Thyroid hormone receptor-associated protein 3	3	95	4	1.109	0.059	2.1E-01
G1T7I0	Q96PU5	NEDD4L	E3 ubiquitin-protein ligase	E3 ubiquitin-protein ligase NEDD4-like	2	96	4	1.109	0.100	3.1E-01
G1SJV2	Q7L523	RRAGA	Uncharacterized protein	Ras-related GTP-binding protein A	3	100	4	1.109	0.124	3.8E-01
G1SF00	Q9BSJ2	TUBGCP2	Gamma-tubulin complex component	Gamma-tubulin complex component 2	2	90	4	1.109	0.047	1.9E-01
G1SN52	E7ESC6	XPO7	Exportin 7	Exportin-7	2	99	4	1.109	0.042	1.8E-01
G1T0E5		SNAP23	Synaptosomal-associated protein		1		4	1.107	0.131	4.0E-01
U3KNL7	Q06210	GFPT1	Uncharacterized protein	Glutamine--fructose-6-phosphate aminotransferase [isomerizing] 1	3	95	4	1.107	0.079	2.7E-01
	O15031	PLXNB2		Plexin-B2	4		4	1.107	0.050	2.0E-01
G1SHZ8	P50990	CCT8	Uncharacterized protein	T-complex protein 1 subunit theta	3	97	4	1.107	0.051	2.1E-01
G1T279	Q9UHW5	GPN3	GPN-loop GTPase 3	GPN-loop GTPase 3	2	96	4	1.106	0.036	1.8E-01
G1T933	Q05707	COL14A1	Uncharacterized protein	Collagen alpha-1(XIV) chain	3	94	4	1.106	0.174	5.0E-01
G1SLK2	P62195	PSMC5	AAA domain-containing protein	26S proteasome regulatory subunit 8	2	100	4	1.106	0.053	2.1E-01
G1SSA2	Q13200	PSMD2	26S proteasome non-ATPase regulatory subunit 2	26S proteasome non-ATPase regulatory subunit 2	2	99	4	1.106	0.074	2.6E-01
G1TC33	P35580	MYH10	Uncharacterized protein	Myosin-10	3	99	4	1.105	0.065	2.4E-01
G1TE27	X6R9L0	DNAJC3	Uncharacterized protein	DnaJ homolog subfamily C member 3	3	88	4	1.105	0.070	2.5E-01
G1SS85	Q9UBQ7	GRHPR	Uncharacterized protein	Glyoxylate reductase/hydroxypyruvate reductase	3	90	4	1.105	0.049	2.1E-01
G1SLF8	J3KN16	ECPAS	Vac14_Fab1_bd domain-containing protein	Proteasome adapter and scaffold protein ECM29	2	97	4	1.105	0.035	1.8E-01
	K7ER00	FARSA		Phenylalanine--tRNA ligase alpha subunit	4		4	1.105	0.107	3.5E-01
G1T239	F5GYQ1	ATP6V0D1	V-type proton ATPase subunit	V-type proton ATPase subunit	2	90	4	1.104	0.027	1.7E-01
G1SMM4		DKK3	Dickkopf_N domain-containing protein		1		4	1.104	0.102	3.4E-01
G1SK33	P05556	ITGB1	Integrin beta	Integrin beta-1	2	94	4	1.104	0.055	2.2E-01
G1T3N8	Q13155	AIMP2	Uncharacterized protein	Aminoacyl tRNA synthase complex-interacting multifunctional protein 2	3	87	4	1.103	0.044	2.1E-01
G1SUL3		TMF1	TMF_TATA_bd domain-containing protein		1		4	1.103	0.078	2.8E-01
G1T6W7	P04040	CAT	Catalase	Catalase	2	91	4	1.103	0.092	3.2E-01
G1SE46	O95159	ZFPL1	Zinc finger protein like 1	Zinc finger protein-like 1	2	92	4	1.103	0.042	2.0E-01
G1SG37	G5E9T8	GOSR1	Golgi SNAP receptor complex member 1	Golgi SNAP receptor complex member 1 (Fragment)	2	99	4	1.102	0.053	2.3E-01
G1SJ43	Q9H3S7	PTPN23	Uncharacterized protein	Tyrosine-protein phosphatase non-receptor type 23	3	91	4	1.102	0.028	1.8E-01
G1T2F5	Q86X52	CHSY1	Hexosyltransferase	Chondroitin sulfate synthase 1	2	93	4	1.101	0.017	1.8E-01
G1SIE8	A0A3B3ISG5	IDE	Uncharacterized protein	Insulin-degrading enzyme	3	96	4	1.101	0.051	2.2E-01
B7NZM8	Q04917	YWHAH	Tyrosine 3-monooxygenase/tryptophan 5-monooxygenase activation protein, eta polypeptide (Predicted)	14-3-3 protein eta	2	99	4	1.100	0.113	3.8E-01
G1SZI6	Q86X10	RALGAPB	Rap-GAP domain-containing protein	Ral GTPase-activating protein subunit beta	2	97	4	1.100	0.049	2.3E-01
G1SFH6	Q9UBT2	UBA2	Uncharacterized protein	SUMO-activating enzyme subunit 2	3	97	4	1.100	0.087	3.2E-01
G1SFN5	E9PFR3	PPP2R5D	Serine/threonine-protein phosphatase 2A 56 kDa regulatory subunit	Serine/threonine-protein phosphatase 2A 56 kDa regulatory subunit	2	98	4	1.099	0.064	2.6E-01
G1T3M5	O43324	EEF1E1	GST C-terminal domain-containing protein	Eukaryotic translation elongation factor 1 epsilon-1	2	95	4	1.097	0.054	2.5E-01
G1T4J2	F5H228	TRIO	Uncharacterized protein	Triple functional domain protein	3	99	4	1.097	0.022	1.9E-01
G1SIH3	A0A2R8Y5A6	ATXN2	Uncharacterized protein	Ataxin-2	3	94	4	1.097	0.080	3.1E-01
G1SFV1	A0A499FI48	PDIA4	Protein disulfide-isomerase	Protein disulfide-isomerase	2	91	4	1.097	0.078	3.1E-01
G1TCS8	P62820	RAB1A	Uncharacterized protein	Ras-related protein Rab-1A	3	100	4	1.096	0.031	2.1E-01
G1TE13	P84095	RHOG	Uncharacterized protein	Rho-related GTP-binding protein RhoG	3	99	4	1.095	0.030	2.1E-01
	P40429	RPL13A		60S ribosomal protein L13a	4		4	1.095	0.125	4.4E-01
G1SEV2	P30101	PDIA3	Protein disulfide-isomerase	Protein disulfide-isomerase A3	2	96	4	1.095	0.108	4.0E-01
G1TA15	Q9P2J5	LARS	Uncharacterized protein	Leucine--tRNA ligase, cytoplasmic	3	95	4	1.094	0.045	2.4E-01
G1TCE2	H7C0R7	CYB5R1	NADH-cytochrome b5 reductase	NADH-cytochrome b5 reductase 1 (Fragment)	2	77	4	1.094	0.096	3.7E-01
G1SWY6	Q15075	EEA1	Early endosome antigen 1	Early endosome antigen 1	2	87	4	1.094	0.035	2.2E-01
	Q13363	CTBP1		C-terminal-binding protein 1	4		4	1.094	0.098	3.7E-01
G1SP36	Q9HAV4	XPO5	Importin N-terminal domain-containing protein	Exportin-5	2	95	4	1.093	0.119	4.3E-01
G1T3V3	Q6R327	RICTOR	Uncharacterized protein	Rapamycin-insensitive companion of mTOR	3	98	4	1.093	0.090	3.6E-01
G1TUY5		OCIAD1	OCIA domain-containing protein		1		4	1.093	0.058	2.7E-01
G1SKK0	P22102	GART	Trifunctional purine biosynthetic protein adenosine-3	Trifunctional purine biosynthetic protein adenosine-3	2	89	4	1.093	0.078	3.3E-01
G1U2W0	B4E321	OS9	OS9, endoplasmic reticulum lectin	Protein OS-9	2	79	4	1.093	0.063	2.9E-01
G1SN95	A0A087WSV8	NUCB2	Nucleobindin 2	Nucleobindin 2, isoform CRA_b	2	93	4	1.092	0.083	3.4E-01
G1TQR9		ZSWIM8	SWIM-type domain-containing protein		1		4	1.092	0.073	3.2E-01
G1T2M9	P09486	SPARC	SPARC	SPARC	2	94	4	1.091	0.166	5.4E-01
G1T0H9	O60645	EXOC3	Exocyst complex component 3	Exocyst complex component 3	2	95	4	1.091	0.035	2.4E-01
P27115		MGAT1	Alpha-1,3-mannosyl-glycoprotein 2-beta-N-acetylglucosaminyltransferase		1		4	1.091	0.091	3.7E-01
B7NZS0		MYADM	Myeloid-associated differentiation marker (Predicted)		1		4	1.091	0.031	2.3E-01
G1SNQ9	A0A0A0MTJ9	NCEH1	Uncharacterized protein	Neutral cholesterol ester hydrolase 1	3	88	4	1.091	0.038	2.4E-01
G1SZ93	Q9NSE4	IARS2	Uncharacterized protein	Isoleucine--tRNA ligase, mitochondrial	3	90	4	1.090	0.140	4.9E-01
G1SM15	A0A087WY71	AP2M1	MHD domain-containing protein	AP-2 complex subunit mu	2	100	4	1.090	0.037	2.4E-01
G1SIP6		CISD1	ZnF_CDGSH domain-containing protein		1		4	1.090	0.108	4.2E-01
G1SH42	Q5VSL9	STRIP1	Uncharacterized protein	Striatin-interacting protein 1	3	98	4	1.089	0.050	2.7E-01
G1TD24	Q9NRY4	ARHGAP35	Rho GTPase activating protein 35	Rho GTPase-activating protein 35	2	98	4	1.089	0.044	2.6E-01
G1SYC9	H0YNE9	RAB8B	Uncharacterized protein	Ras-related protein Rab-8B (Fragment)	3	99	4	1.089	0.050	2.7E-01
G1TCK9	A0A0A0MSX9	IARS	Uncharacterized protein	Isoleucine--tRNA ligase, cytoplasmic	3	94	4	1.089	0.057	2.9E-01
G1TE76	Q15056	EIF4H	Eukaryotic translation initiation factor 4H	Eukaryotic translation initiation factor 4H	2	92	4	1.088	0.030	2.4E-01

G1U684	A0A087X1W8	CADM1	Uncharacterized protein	Cell adhesion molecule 1	3	95	4	1.088	0.058	3.0E-01
G1SWD9	B4DKY1	CARS	Cysteinyl-tRNA synthetase	Cysteine--tRNA ligase, cytoplasmic	2	68	4	1.087	0.112	4.5E-01
G1T8P4	P05386	RPLP1	Uncharacterized protein	60S acidic ribosomal protein P1	3	97	4	1.087	0.137	5.0E-01
G1STW7	Q9NSD9	FARSB	B5 domain-containing protein	Phenylalanine--tRNA ligase beta subunit	2	95	4	1.086	0.063	3.2E-01
O19048	Q15365	PCBP1	Poly(rC)-binding protein 1	Poly(rC)-binding protein 1	2	100	4	1.086	0.062	3.2E-01
G1SQ90	A0A2R8Y5H3	COL4A3BP	Collagen type IV alpha 3 binding protein	Collagen type IV alpha-3-binding protein (Fragment)	2	96	4	1.086	0.025	2.5E-01
G1STS3	Q96T76	MMS19	Uncharacterized protein	MMS19 nucleotide excision repair protein homolog	3	93	4	1.086	0.016	2.4E-01
G1SFE9	H0YJG7	AHSA1	Aha1_N domain-containing protein	Activator of 90 kDa heat shock protein ATPase homolog 1 (Fragment)	2	95	4	1.085	0.053	3.0E-01
G1SCX4	Q15052	ARHGEF6	Uncharacterized protein	Rho guanine nucleotide exchange factor 6	3	94	3	1.084	0.267	6.9E-01
G1SN68	A0A1B0GVU9	QARS	Uncharacterized protein	Glutamine--tRNA ligase (Fragment)	3	92	4	1.084	0.051	3.0E-01
G1T4H3	Q15084	PDIA6	Uncharacterized protein	Protein disulfide-isomerase A6	3	93	4	1.084	0.094	4.2E-01
G1SQT2	Q6P2E9	EDC4	WD_REPEATS_REGION domain-containing protein	Enhancer of mRNA-decapping protein 4	2	96	4	1.082	0.022	2.6E-01
G1SJ20	A0A1W2PNV3	GOSR2	Uncharacterized protein	Golgi SNAP receptor complex member 2 (Fragment)	3	92	4	1.082	0.078	3.8E-01
G1TLQ8	R4GNH3	PSMC3	AAA domain-containing protein	26S proteasome regulatory subunit 6A	2	100	4	1.082	0.051	3.1E-01
G1U484		LLGL1	LLGL scribble cell polarity complex component 1		1		4	1.081	0.089	4.2E-01
G1SR15		CD109	CD109 molecule		1		4	1.081	0.044	3.0E-01
	Q9NUY8	TBC1D23		TBC1 domain family member 23	4		4	1.080	0.034	2.9E-01
G1T9V4	G3V5Z7	PSMA6	Proteasome subunit alpha type	Proteasome subunit alpha type	2	97	4	1.080	0.069	3.7E-01
G1SM62	O43252	PAPSS1	Uncharacterized protein	Bifunctional 3~-phosphoadenosine 5~-phosphosulfate synthase 1	3	99	4	1.079	0.075	3.9E-01
G1SMZ9		ICMT	Protein-S-isoprenylcysteine O-methyltransferase		1		4	1.079	0.059	3.5E-01
G1SFG8	A0A096LNH6	DOCK1	Uncharacterized protein	Dedicator of cytokinesis protein 1	3	95	4	1.078	0.043	3.2E-01
G1SCT9	A0A087WU53	MAGT1	Uncharacterized protein	Magnesium transporter protein 1	3	97	4	1.078	0.110	4.9E-01
G1T3D9	P62241	RPS8	40S ribosomal protein S8	40S ribosomal protein S8	2	94	4	1.078	0.181	6.3E-01
G1SUN1	O75915	ARL6IP5	PRA1 family protein	PRA1 family protein 3	2	96	4	1.077	0.058	3.6E-01
G1T235	P28072	PSMB6	Proteasome subunit beta	Proteasome subunit beta type-6	2	97	4	1.076	0.075	4.1E-01
G1TJX7	P50552	VASP	Vasodilator stimulated phosphoprotein	Vasodilator-stimulated phosphoprotein	2	87	4	1.075	0.096	4.7E-01
G1SL07	Q9NUP9	LIN7C	Protein lin-7 homolog	Protein lin-7 homolog C	2	99	4	1.075	0.066	3.9E-01
G1SYF9	Q9Y613	FHOD1	Uncharacterized protein	FH1/FH2 domain-containing protein 1	3	87	4	1.075	0.021	3.0E-01
G1SSV4	A0A087WT80	PLCB1	1-phosphatidylinositol 4,5-bisphosphate phosphodiesterase	1-phosphatidylinositol 4,5-bisphosphate phosphodiesterase	2	97	4	1.074	0.063	3.9E-01
	E9PM12	TCIRG1		V-type proton ATPase subunit a (Fragment)	4		4	1.074	0.099	4.8E-01
G1T3D1	Q14789	GOLGB1	Uncharacterized protein	Golgin subfamily B member 1	3	85	4	1.074	0.039	3.4E-01
G1SM04	Q13188	STK3	Uncharacterized protein	Serine/threonine-protein kinase 3	3	99	4	1.073	0.048	3.6E-01
G1TVG8	O15173	PGRMC2	Cytochrome b5 heme-binding domain-containing protein	Membrane-associated progesterone receptor component 2	2	97	4	1.073	0.082	4.4E-01
G1SDM6	O75165	DNAJC13	J domain-containing protein	DnaJ homolog subfamily C member 13	2	98	4	1.073	0.037	3.4E-01
	A0A087X1G7	SELENOF		Selenoprotein F	4		4	1.073	0.050	3.7E-01
G1SY96	Q9UJS0	SLC25A13	Uncharacterized protein	Calcium-binding mitochondrial carrier protein Aralar2	3	97	4	1.072	0.052	3.7E-01
G1T7U4	Q9Y4E8	USP15	Ubiquitin carboxyl-terminal hydrolase	Ubiquitin carboxyl-terminal hydrolase 15	2	99	4	1.072	0.061	4.0E-01
G1SMZ8	Q96JJ7	TMX3	Thioredoxin domain-containing protein	Protein disulfide-isomerase TMX3	2	92	4	1.072	0.043	3.6E-01
G1SNN0		CSGALNACT2	Hexosyltransferase		1		3	1.071	0.108	5.3E-01
G1SHZ2	Q86X83	COMMD2	COMM domain-containing protein	COMM domain-containing protein 2	2	97	4	1.071	0.167	6.4E-01
G1TX94	A0A0R4J2E8	MATR3	Uncharacterized protein	Matrin-3	3	99	4	1.071	0.042	3.6E-01
G1SL46		PSMD9	PDZ domain-containing protein		1		4	1.069	0.063	4.2E-01
	A6NM71	WDR45		PRA1 family protein	4		4	1.069	0.090	4.9E-01
G1SIW8	Q08257	CRYZ	PKS_ER domain-containing protein	Quinone oxidoreductase	2	87	4	1.068	0.041	3.8E-01
G1SXI8		PCID2	PCI domain containing 2		1		4	1.068	0.036	3.7E-01
	P54725	RAD23A		UV excision repair protein RAD23 homolog A	4		4	1.067	0.084	4.8E-01
G1SH30	Q8WVY7	UBLCP1	Uncharacterized protein	Ubiquitin-like domain-containing CTD phosphatase 1	3	100	4	1.067	0.055	4.2E-01
G1SRJ6		AFAP1	Actin filament associated protein 1		1		4	1.066	0.093	5.2E-01
G1SGN0	Q15042	RAB3GAP1	Uncharacterized protein	Rab3 GTPase-activating protein catalytic subunit	3	94	4	1.065	0.031	3.7E-01
G1TE61	O60684	KPNA6	Importin subunit alpha	Importin subunit alpha-7	2	99	4	1.064	0.036	3.9E-01
G1SM91		FAH	Fumarylacetoacetase		1		4	1.064	0.121	5.9E-01
G1SSV0	Q9H1E5	TMX4	Thioredoxin domain-containing protein	Thioredoxin-related transmembrane protein 4	2	84	4	1.064	0.085	5.1E-01
G1U4H9	O14907	TAX1BP3	Tax1-binding protein 3	Tax1-binding protein 3	2	100	4	1.063	0.056	4.4E-01
G1SZ15	A8K878	MANF	Mesencephalic astrocyte derived neurotrophic factor	Mesencephalic astrocyte-derived neurotrophic factor	2	97	4	1.063	0.096	5.4E-01
G1SHS4	A0A0C4DGX4	CUL1	CULLIN_2 domain-containing protein	Cullin-1	2	97	4	1.062	0.050	4.3E-01
G1T579	Q6NUQ1	RINT1	Uncharacterized protein	RAD50-interacting protein 1	3	92	4	1.061	0.053	4.5E-01
G1TNH0	O14939	PLD2	Phospholipase D2	Phospholipase D2	2	89	4	1.060	0.086	5.3E-01
G1SGE5	A0A087WZR9	PYCR2	Pyrroline-5-carboxylate reductase	Pyrroline-5-carboxylate reductase	2	94	4	1.060	0.072	5.0E-01
G1SZA3	Q9H2M9	RAB3GAP2	Uncharacterized protein	Rab3 GTPase-activating protein non-catalytic subunit	3	94	4	1.059	0.048	4.5E-01
G1T534	E9PDM8	SEC24D	SEC24 homolog D, COPII coat complex component	Protein transport protein Sec24D	2	92	4	1.059	0.048	4.5E-01
G1T0T5	F5GYF7	COPS7A	PCI domain-containing protein	COP9 signalosome complex subunit 7a (Fragment)	2	100	4	1.059	0.032	4.2E-01
P0CL18		EIF2D	Eukaryotic translation initiation factor 2D		1		4	1.059	0.118	6.1E-01
G1T7R4	P18084	ITGB5	Integrin beta	Integrin beta-5	2	93	4	1.059	0.090	5.5E-01
G1SES9	P31939	ATIC	MGS domain-containing protein	Bifunctional purine biosynthesis protein PURH	2	94	4	1.059	0.028	4.2E-01
G1SMB5		VPS37C	VPS37 C-terminal domain-containing protein		1		4	1.058	0.050	4.7E-01
G1SFE0	O00487	PSMD14	MPN domain-containing protein	26S proteasome non-ATPase regulatory subunit 14	2	100	4	1.057	0.041	4.5E-01
G1T9V7		SRP19	Signal recognition particle 19		1		4	1.057	0.032	4.4E-01
G1SV40	P83436	COG7	Uncharacterized protein	Conserved oligomeric Golgi complex subunit 7	3	93	4	1.057	0.080	5.4E-01
G1T5H7		TMEM132B	Transmembrane protein 132B		1		4	1.057	0.039	4.5E-01

G1SFC4		LMF2	Lipase maturation factor		1		4	1.056	0.063	5.1E-01
G1SW65		ATP5MC2	ATP-synt_C domain-containing protein		1		4	1.056	0.084	5.6E-01
G1SVA3	O00231	PSMD11	PCI domain-containing protein	26S proteasome non-ATPase regulatory subunit 11	2	100	4	1.056	0.045	4.7E-01
G1SPD2	Q16401	PSMD5	Uncharacterized protein	26S proteasome non-ATPase regulatory subunit 5	3	92	4	1.056	0.061	5.1E-01
	P37837	TALDO1		Transaldolase	4		4	1.055	0.064	5.2E-01
G1T645	Q96S52	PIGS	Uncharacterized protein	GPI transamidase component PIG-S	3	85	4	1.055	0.052	4.9E-01
G1T4N5	P42345	MTOR	Serine/threonine-protein kinase mTOR	Serine/threonine-protein kinase mTOR	2	99	4	1.055	0.032	4.5E-01
G1SIS5	O15498	YKT6	Uncharacterized protein	Synaptobrevin homolog YKT6	3	96	4	1.055	0.064	5.2E-01
G1SVV6	Q02750	MAP2K1	Dual-specificity mitogen-activated protein kinase kinase 1	Dual specificity mitogen-activated protein kinase kinase 1	2	94	4	1.055	0.069	5.3E-01
G1SJL0		TIMM29	Translocase of inner mitochondrial membrane 29		1		4	1.054	0.060	5.2E-01
G1TEM4	Q9UEW8	STK39	Protein kinase domain-containing protein	STE20/SPS1-related proline-alanine-rich protein kinase	2	95	4	1.054	0.047	4.9E-01
G1T7T0	Q9Y2G5	POFUT2	Protein O-fucosyltransferase 2	GDP-fucose protein O-fucosyltransferase 2	2	84	4	1.053	0.015	4.5E-01
G1TUP1	O75340	PDCD6	Programmed cell death 6	Programmed cell death protein 6	2	99	4	1.053	0.096	6.1E-01
G1SJC7	Q9UN86	G3BP2	Uncharacterized protein	Ras GTPase-activating protein-binding protein 2	3	100	4	1.052	0.107	6.3E-01
P47823		EIF2B5	Translation initiation factor eIF-2B subunit epsilon		1		4	1.052	0.089	6.0E-01
G1T2G6	Q9P265	DIP2B	DMAP-interaction domain-containing protein	Disco-interacting protein 2 homolog B	2	99	4	1.052	0.092	6.1E-01
G1U8J5		ATP5PF	ATP synthase-coupling factor 6, mitochondrial		1		4	1.052	0.054	5.2E-01
G1SZR6	Q13618	CUL3	CULLIN_2 domain-containing protein	Cullin-3	2	100	4	1.051	0.034	4.9E-01
G1T6N8		ALG12	Mannosyltransferase		1		4	1.051	0.022	4.7E-01
G1SJK7		CYP27A1	Sterol 26-hydroxylase, mitochondrial		1		4	1.051	0.226	7.9E-01
G1TPC8	P08962	CD63	Tetraspanin	CD63 antigen	2	78	4	1.051	0.043	5.1E-01
G1SJU2	O60306	AQR	RNA helicase aquarius	RNA helicase aquarius	2	96	4	1.051	0.052	5.3E-01
G1SE01	A0A0A0MSI8	EXOC5	Exocyst complex component 5	Exocyst complex component 5	2	98	4	1.050	0.034	4.9E-01
G1SYY0	Q96SK2	TMEM209	Uncharacterized protein	Transmembrane protein 209	3	96	4	1.050	0.114	6.6E-01
Q95MN6		PLP2	Proteolipid protein 2		1		4	1.050	0.033	5.0E-01
G1SCY3		UBR4	UBR-type domain-containing protein		1		4	1.050	0.051	5.3E-01
G1U6H0	P27824	CANX	Uncharacterized protein	Calnexin	3	95	4	1.049	0.062	5.6E-01
G1SGV5	Q8NE71	ABCF1	Uncharacterized protein	ATP-binding cassette sub-family F member 1	3	92	4	1.049	0.135	7.0E-01
G1T0S0	Q8NBN3	TMEM87A	Uncharacterized protein	Transmembrane protein 87A	3	96	4	1.049	0.102	6.5E-01
G1SHH1		PPOX	Protoporphyrinogen oxidase		1		4	1.048	0.095	6.3E-01
G1SFF5	O15270	SPTLC2	Aminotran_1_2 domain-containing protein	Serine palmitoyltransferase 2	2	98	4	1.048	0.146	7.2E-01
G1SSV2	Q9UL01	DSE	DUF4962 domain-containing protein	Dermatan-sulfate epimerase	2	96	4	1.048	0.044	5.4E-01
P15253	P27797	CALR	Calreticulin	Calreticulin	2	96	4	1.047	0.090	6.3E-01
G1SF36	Q13423	NNT	Uncharacterized protein	NAD(P) transhydrogenase, mitochondrial	3	97	4	1.047	0.085	6.3E-01
G1TVU4	Q96D15	RCN3	Reticulocalbin 3	Reticulocalbin-3	2	78	4	1.046	0.105	6.7E-01
G1T3Z6	P31323	PRKAR2B	Uncharacterized protein	cAMP-dependent protein kinase type II-beta regulatory subunit	3	97	4	1.046	0.046	5.5E-01
G1U354	R4GMR5	PSMD8	PCI domain-containing protein	26S proteasome non-ATPase regulatory subunit 8	2	97	4	1.046	0.065	5.9E-01
G1T2Z5	A0A087WTA8	COL1A2	Collagen alpha-2(I) chain	Collagen alpha-2(I) chain	2	94	4	1.046	0.135	7.2E-01
G1SVF2	A0A087WW66	PSMD1	26S proteasome non-ATPase regulatory subunit 1	26S proteasome non-ATPase regulatory subunit 1	2	99	4	1.046	0.042	5.5E-01
G1SJB9	Q14257	RCN2	Reticulocalbin 2	Reticulocalbin-2	2	90	4	1.045	0.127	7.1E-01
G1SH95	P30622	CLIP1	CAP-Gly domain containing linker protein 1	CAP-Gly domain-containing linker protein 1	2	85	4	1.045	0.056	5.8E-01
G1TP15	O43242	PSMD3	PCI domain-containing protein	26S proteasome non-ATPase regulatory subunit 3	2	98	4	1.045	0.039	5.5E-01
	Q9UL15	BAG5		BAG family molecular chaperone regulator 5	4		4	1.045	0.076	6.3E-01
G1TB98	Q15293	RCN1	Uncharacterized protein	Reticulocalbin-1	3	85	4	1.045	0.080	6.3E-01
G1TGH1		D2HGDH	D-2-hydroxyglutarate dehydrogenase		1		4	1.044	0.103	6.8E-01
A0A140TAW0	O43852	CALU	Calumenin	Calumenin	2	99	4	1.044	0.112	7.0E-01
G1SG72	P61221	ABCE1	Uncharacterized protein	ATP-binding cassette sub-family E member 1	3	100	4	1.043	0.041	5.7E-01
G1SUX1		TIMP3	Metalloproteinase inhibitor 3		1		4	1.042	0.090	6.7E-01
G1SL80		UROD	Uroporphyrinogen decarboxylase		1		4	1.042	0.054	6.1E-01
G1SHS8	A0A087WY55	VTA1	Uncharacterized protein	Chromosome 6 open reading frame 55, isoform CRA_b	3	87	4	1.041	0.106	7.0E-01
G1T1D9	Q92905	COPS5	MPN domain-containing protein	COP9 signalosome complex subunit 5	2	100	4	1.041	0.121	7.3E-01
G1TA41	Q7L7X3	TAOK1	Protein kinase domain-containing protein	Serine/threonine-protein kinase TAO1	2	100	4	1.041	0.028	5.7E-01
G1T3S1	A0A087X2I1	PSMC6	AAA domain-containing protein	26S proteasome regulatory subunit 10B	2	100	4	1.041	0.053	6.1E-01
G1T3E6	A0A024R442	DNPEP	Uncharacterized protein	Aspartyl aminopeptidase	3	91	4	1.040	0.050	6.1E-01
G1SRJ7	P08253	MMP2	72 kDa type IV collagenase	72 kDa type IV collagenase	2	95	4	1.039	0.079	6.7E-01
P41982	P04179	SOD2	Superoxide dismutase [Mn], mitochondrial (Fragment)	Superoxide dismutase [Mn], mitochondrial	2	92	4	1.039	0.125	7.5E-01
G1SG41		TBL2	WD_REPEATS_REGION domain-containing protein		1		4	1.037	0.055	6.5E-01
G1TAF8	P31948	STIP1	Uncharacterized protein	Stress-induced-phosphoprotein 1	3	96	4	1.037	0.080	7.0E-01
G1U2R1	Q99805	TM9SF2	Transmembrane 9 superfamily member	Transmembrane 9 superfamily member 2	2	93	4	1.037	0.049	6.4E-01
G1SYA5	X6RA14	ESD	S-formylglutathione hydrolase	S-formylglutathione hydrolase	2	88	4	1.036	0.084	7.1E-01
G1SSN9	Q96S59	RANBP9	Uncharacterized protein	Ran-binding protein 9	3	97	4	1.036	0.074	6.9E-01
G1TJ79		KYAT1	Aminotran_1_2 domain-containing protein		1		4	1.036	0.048	6.5E-01
G1SXB6	B4DVA9	POGLUT1	CAP10 domain-containing protein	Protein O-glucosyltransferase 1	2	96	4	1.035	0.082	7.1E-01
G1SQJ2		FOCAD	DUF3730 domain-containing protein		1		4	1.035	0.137	7.9E-01
G1SXL9		XPNPEP3	AMP_N domain-containing protein		1		4	1.035	0.064	6.8E-01
G1T593	F8VVA7	COPZ1	Clat_adaptor_s domain-containing protein	Coatomer subunit zeta-1	2	88	4	1.035	0.043	6.5E-01
G1SRL3	P30419	NMT1	Glycylpeptide N-tetradecanoyltransferase	Glycylpeptide N-tetradecanoyltransferase 1	2	98	4	1.034	0.020	6.3E-01
P80912		HINT1	Histidine triad nucleotide-binding protein 1		1		4	1.033	0.163	8.2E-01
G1TDF6	O75955	FLOT1	PHB domain-containing protein	Flotillin-1	2	99	4	1.033	0.040	6.6E-01

G1TGF1	Q15185	PTGES3	Prostaglandin E synthase 3	Prostaglandin E synthase 3	2	100	4	1.033	0.079	7.2E-01
G1T2T9	P20339	RAB5A	Uncharacterized protein	Ras-related protein Rab-5A	3	99	4	1.033	0.112	7.7E-01
G1T8T0		FRMD6	FERM domain-containing protein		1		3	1.032	0.178	8.3E-01
G1T2W1	O76094	SRP72	Signal recognition particle subunit SRP72	Signal recognition particle subunit SRP72	2	98	4	1.031	0.049	6.9E-01
G1T9M9	P11142	HSPA8	Uncharacterized protein	Heat shock cognate 71 kDa protein	3	100	4	1.031	0.032	6.7E-01
G1TTS1		FUNDC2	FUN14 domain containing 2		1		4	1.031	0.079	7.4E-01
G1SWE5		WDR61	WD repeat domain 61		1		4	1.030	0.082	7.5E-01
A0A0A0MQP7	P62736	ACTA2	Actin, aortic smooth muscle	Actin, aortic smooth muscle	2	100	4	1.030	0.057	7.1E-01
G1SUK0	O75940	SMNDC1	Tudor domain-containing protein	Survival of motor neuron-related-splicing factor 30	2	99	3	1.030	0.148	8.3E-01
G1U886	A0A2R8YGH5	AP1S1	AP complex subunit sigma	AP complex subunit sigma	2	100	4	1.030	0.161	8.4E-01
G1TCM0	J3KQ34	COPS7B	PCI domain-containing protein	COP9 signalosome complex subunit 7b	2	97	4	1.029	0.067	7.4E-01
G1SI20	O75874	IDH1	Isocitrate dehydrogenase [NADP]	Isocitrate dehydrogenase [NADP] cytoplasmic	2	97	4	1.028	0.063	7.4E-01
G1SDU6	P26639	TARS	AA_TRNA_LIGASE_II domain-containing protein	Threonine--tRNA ligase, cytoplasmic	2	96	4	1.028	0.089	7.8E-01
G1U207	Q86Y82	STX12	t-SNARE coiled-coil homology domain-containing protein	Syntaxin-12	2	96	4	1.027	0.035	7.2E-01
G1SJX5	A0A087WTF3	ANK3	Ankyrin 3	Ankyrin-3 (Fragment)	2	93	4	1.026	0.117	8.2E-01
G1TYK8	Q13492	PICALM	Phosphatidylinositol binding clathrin assembly protein	Phosphatidylinositol-binding clathrin assembly protein	2	97	4	1.026	0.022	7.1E-01
G1SML4	P28288	ABCD3	Uncharacterized protein	ATP-binding cassette sub-family D member 3	3	96	4	1.026	0.078	7.8E-01
P41035	P20042	EIF2S2	Eukaryotic translation initiation factor 2 subunit 2	Eukaryotic translation initiation factor 2 subunit 2	2	98	4	1.026	0.055	7.5E-01
G1SWS6	A0A3B3IRN5	FMOD	Fibromodulin	Fibromodulin	2	92	4	1.026	0.114	8.2E-01
G1T6B3	Q9Y3F4	STRAP	WD_REPEATS_REGION domain-containing protein	Serine-threonine kinase receptor-associated protein	2	98	4	1.026	0.020	7.1E-01
G1SW11		CERCAM	Cerebral endothelial cell adhesion molecule		1		4	1.026	0.157	8.6E-01
G1SUM3	Q8TBC4	UBA3	E2_bind domain-containing protein	NEDD8-activating enzyme E1 catalytic subunit	2	99	4	1.025	0.062	7.6E-01
	K7ELL7	PRKCSH		Glucosidase 2 subunit beta	4		4	1.025	0.046	7.5E-01
G1THI9	Q9UKB1	FBXW11	Uncharacterized protein	F-box/WD repeat-containing protein 11	3	96	4	1.025	0.047	7.5E-01
G1TEY6	O00178	GTPBP1	Tr-type G domain-containing protein	GTP-binding protein 1	2	94	4	1.024	0.031	7.4E-01
	I3L0A0	`MEM189-UBE2V1		HCG2044781	4		4	1.024	0.074	7.9E-01
G1SF45		SEC24B	SEC24 homolog B, COPII coat complex component		1		4	1.023	0.024	7.4E-01
G1SWN4	Q9H832	UBE2Z	UBIQUITIN_CONJUGAT_2 domain-containing protein	Ubiquitin-conjugating enzyme E2 Z	2	96	4	1.023	0.098	8.3E-01
G1SCE6	Q9Y223	GNE	Epimerase_2 domain-containing protein	Bifunctional UDP-N-acetylglucosamine 2-epimerase/N-acetylmannosamine kinase	2	100	4	1.022	0.064	8.0E-01
P63169	F8VRV5	DYNLL1	Dynein light chain 1, cytoplasmic	Dynein light chain	2	100	4	1.021	0.058	8.0E-01
G1T284	Q8TCJ2	STT3B	Uncharacterized protein	Dolichyl-diphosphooligosaccharide--protein glycosyltransferase subunit STT3B	3	100	4	1.021	0.111	8.5E-01
G1T4X8	P49721	PSMB2	Proteasome subunit beta	Proteasome subunit beta type-2	2	99	4	1.021	0.075	8.2E-01
G1SJR4	Q15363	TMED2	Transmembrane p24 trafficking protein 2	Transmembrane emp24 domain-containing protein 2	2	99	4	1.020	0.043	7.9E-01
G1TPY7		SUMF2	FGE-sulfatase domain-containing protein		1		4	1.020	0.076	8.3E-01
G1TCY1	Q16513	PKN2	Uncharacterized protein	Serine/threonine-protein kinase N2	3	97	4	1.019	0.027	7.9E-01
G1SKG9	A0A087X2H1	HECTD1	Uncharacterized protein	E3 ubiquitin-protein ligase HECTD1	3	99	4	1.019	0.038	8.0E-01
G1T276		ALDH3A2	Aldehyde dehydrogenase		1		4	1.018	0.082	8.5E-01
G1U2B5		PDXK	Phos_pyr_kin domain-containing protein		1		4	1.017	0.119	8.8E-01
G1T6D4	O00232	PSMD12	PCI domain-containing protein	26S proteasome non-ATPase regulatory subunit 12	2	99	4	1.017	0.047	8.3E-01
G1SZP4	I6L894	ANK2	Ankyrin 2	Ankyrin-2	2	88	4	1.017	0.083	8.6E-01
G1TE47		COG8	Conserved oligomeric Golgi complex subunit 8		1		4	1.016	0.028	8.2E-01
G1SDD2	B7Z2Y2	COG2	Uncharacterized protein	Conserved oligomeric Golgi complex subunit 2	3	91	4	1.015	0.071	8.7E-01
G1SYV0	P35998	PSMC2	AAA domain-containing protein	26S proteasome regulatory subunit 7	2	100	4	1.015	0.042	8.5E-01
G1T7Q2		LOXL2	Lysyl oxidase like 2		1		4	1.014	0.073	8.7E-01
G1T4H6		MTMR6	Myotubularin phosphatase domain-containing protein		1		4	1.014	0.060	8.7E-01
G1SRQ2	A0A087WTB8	UCHL3	Ubiquitin carboxyl-terminal hydrolase	Ubiquitin carboxyl-terminal hydrolase	2	98	4	1.014	0.105	9.0E-01
G1TC19		CPT2	Carnitine palmitoyltransferase 2		1		4	1.014	0.078	8.8E-01
B7NZJ1		CPNE1	Copine I, isoform 8 (Predicted)		1		4	1.013	0.107	9.0E-01
	F5H039	GPHN		Molybdopterin molybdenumtransferase	4		4	1.013	0.048	8.7E-01
G1TIA2		RIOX1	JmjC domain-containing protein		1		4	1.012	0.061	8.8E-01
G1SSN2		SIRT5	NAD-dependent protein deacylase sirtuin-5, mitochondrial		1		4	1.012	0.125	9.2E-01
G1T782	Q9Y6N5	SQOR	Pyr_redox_2 domain-containing protein	Sulfide:quinone oxidoreductase, mitochondrial	2	93	4	1.011	0.117	9.2E-01
G1TB17	A0A1B0GWF8	ADSL	Adenylosuccinate lyase	Adenylosuccinate lyase (Fragment)	2	95	4	1.011	0.157	9.4E-01
G1SIP1	Q96LJ7	DHRS1	Uncharacterized protein	Dehydrogenase/reductase SDR family member 1	3	87	4	1.011	0.033	8.9E-01
G1SD02	E7EM64	COPS6	COP9 signalosome subunit 6	COP9 signalosome complex subunit 6	2	97	4	1.010	0.051	9.0E-01
G1TK72	F8W8H5	RAB24	Uncharacterized protein	Ras-related protein Rab-24	3	98	3	1.010	0.029	9.1E-01
G1SZ72	P51116	FXR2	Uncharacterized protein	Fragile X mental retardation syndrome-related protein 2	3	98	4	1.010	0.052	9.0E-01
G1T2R3		EFL1	Tr-type G domain-containing protein		1		4	1.009	0.097	9.3E-01
G1TET2	P13796	LCP1	Uncharacterized protein	Plastin-2	3	98	4	1.009	0.212	9.6E-01
G1SJQ2	Q92896	GLG1	Uncharacterized protein	Golgi apparatus protein 1	3	97	4	1.009	0.114	9.4E-01
G1TW66		DMAC2	Distal membrane arm assembly complex 2		1		4	1.008	0.147	9.5E-01
G1SFC5	Q86VS8	HOOK3	Calponin-homology (CH) domain-containing protein	Protein Hook homolog 3	2	99	4	1.008	0.041	9.2E-01
G1SQM7	P53999	SUB1	PC4 domain-containing protein	Activated RNA polymerase II transcriptional coactivator p15	2	97	4	1.007	0.060	9.3E-01
G1SLU5	Q9UKZ1	CNOT11	CCR4-NOT transcription complex subunit 11	CCR4-NOT transcription complex subunit 11	2	79	4	1.007	0.034	9.2E-01
	M0R261	PGLS		6-phosphogluconolactonase (Fragment)	4		4	1.006	0.119	9.6E-01
G1SZH8	Q8N1B4	VPS52	Uncharacterized protein	Vacuolar protein sorting-associated protein 52 homolog	3	99	4	1.006	0.054	9.4E-01
G1SI71		PAIP1	MIF4G domain-containing protein		1		4	1.006	0.114	9.6E-01
G1SCU8		OTUD6B	OTU domain-containing protein		1		4	1.006	0.025	9.4E-01
G1T6E8	F5H442	TSG101	Uncharacterized protein	Tumor susceptibility gene 101 protein	3	99	4	1.005	0.046	9.5E-01

G1TWU1		TOR1B	Torsin		1		4	1.005	0.117	9.7E-01
G1T4Q8	J3KNQ4	PARVA	Uncharacterized protein	Alpha-parvin	3	91	4	1.005	0.061	9.6E-01
G1T4M1		LAMC1	Laminin subunit gamma 1		1		4	1.004	0.106	9.7E-01
G1TZQ5	G5EA31	SEC24C	Uncharacterized protein	Protein transport protein Sec24C	3	93	4	1.004	0.035	9.6E-01
G1SPA6	Q32P28	P3H1	Fe2OG dioxygenase domain-containing protein	Prolyl 3-hydroxylase 1	2	91	4	1.004	0.050	9.6E-01
	Q9Y224	RTRAF		RNA transcription, translation and transport factor protein	4		4	1.004	0.024	9.6E-01
G1SPI7	Q92538	GBF1	SEC7 domain-containing protein	Golgi-specific brefeldin A-resistance guanine nucleotide exchange factor 1	2	96	4	1.003	0.028	9.7E-01
G1T373	B1ALA9	PRPS1	Pribosyltran_N domain-containing protein	Ribose-phosphate pyrophosphokinase 1	2	88	4	1.003	0.057	9.7E-01
G1TM00	O75436	VPS26A	VPS26, retromer complex component A	Vacuolar protein sorting-associated protein 26A	2	99	4	1.002	0.044	9.8E-01
G1SFQ7		TUBG1	Tubulin gamma chain		1		4	1.002	0.033	9.8E-01
G1SF26	P53675	CLTCL1	Clathrin heavy chain	Clathrin heavy chain 2	2	91	4	1.002	0.106	9.9E-01
G1SE49	Q9UID3	VPS51	Uncharacterized protein	Vacuolar protein sorting-associated protein 51 homolog	3	96	4	1.002	0.137	9.9E-01
G1TWK7	Q14696	MESD	Uncharacterized protein	LRP chaperone MESD	3	90	4	1.001	0.059	9.9E-01
G1TYL5		DNPH1	2~-deoxynucleoside 5~-phosphate N-hydrolase 1		1		4	1.001	0.091	9.9E-01
U3KMI4	P62834	RAP1A	Uncharacterized protein	Ras-related protein Rap-1A	3	100	4	1.001	0.060	9.9E-01
G1SDN9	Q9P2B2	PTGFRN	Uncharacterized protein	Prostaglandin F2 receptor negative regulator	3	91	4	1.000	0.149	1.0E+00
G1SCY4	P52907	CAPZA1	F-actin-capping protein subunit alpha	F-actin-capping protein subunit alpha-1	2	96	4	1.000	0.031	1.0E+00
G1SMX7	O43731	KDELR3	ER lumen protein-retaining receptor	ER lumen protein-retaining receptor 3	2	98	4	1.000	0.143	1.0E+00
G1TRA4	P01111	NRAS	Uncharacterized protein	GTPase NRas	3	100	3	0.998	0.054	9.9E-01
G1SRB6	Q08752	PPID	Peptidylprolyl isomerase D	Peptidyl-prolyl cis-trans isomerase D	2	93	4	0.998	0.056	9.8E-01
G1SEN5	Q8IZ07	ANKRD13A	Ankyrin repeat domain 13A	Ankyrin repeat domain-containing protein 13A	2	94	4	0.998	0.067	9.8E-01
G1TDN4	P10644	PRKAR1A	Uncharacterized protein	cAMP-dependent protein kinase type I-alpha regulatory subunit	3	99	4	0.997	0.090	9.8E-01
G1SZQ7	Q7L1Q6	BZW1	W2 domain-containing protein	Basic leucine zipper and W2 domain-containing protein 1	2	100	4	0.997	0.025	9.7E-01
G1T6N3		NPC2	ML domain-containing protein		1		4	0.997	0.346	9.9E-01
G1SP51	P62277	RPS13	Ribosomal_S13_N domain-containing protein	40S ribosomal protein S13	2	100	4	0.996	0.107	9.7E-01
G1T887		GPR107	G protein-coupled receptor 107		1		4	0.996	0.071	9.6E-01
G1T0R4	Q96JC1	VPS39	CNH domain-containing protein	Vam6/Vps39-like protein	2	97	4	0.994	0.067	9.5E-01
G1TL06	P39023	RPL3	Uncharacterized protein	60S ribosomal protein L3	3	98	4	0.994	0.118	9.6E-01
G1SQD1	E7EUU4	EIF4G1	Eukaryotic translation initiation factor 4 gamma 1	Eukaryotic translation initiation factor 4 gamma 1	2	94	4	0.994	0.028	9.3E-01
G1T725	Q8TB40	ABHD4	AB hydrolase-1 domain-containing protein	(Lyso)-N-acylphosphatidylethanolamine lipase	2	97	4	0.992	0.029	9.2E-01
G1TDC3	Q14697	GANAB	Gal_mutarotas_2 domain-containing protein	Neutral alpha-glucosidase AB	2	91	4	0.992	0.061	9.2E-01
G1T0H3	P40763	STAT3	Signal transducer and activator of transcription	Signal transducer and activator of transcription 3	2	100	4	0.991	0.059	9.2E-01
G1T3A2	Q9Y3A6	TMED5	GOLD domain-containing protein	Transmembrane emp24 domain-containing protein 5	2	97	4	0.990	0.056	9.0E-01
G1T0B0	Q9H3H3	C11orf68	Uncharacterized protein	UPF0696 protein C11orf68	3	93	4	0.990	0.024	8.9E-01
G1TM22		UGT1A1	UDP-glucuronosyltransferase		1		4	0.990	0.055	9.0E-01
G1T0N5	Q00577	PURA	Purine rich element binding protein A	Transcriptional activator protein Pur-alpha	2	91	4	0.990	0.053	9.0E-01
G1SJF1	A0A499FIZ0	WDR26	Uncharacterized protein	WD repeat-containing protein 26	3	99	4	0.990	0.043	8.9E-01
G1T846	P14868	DARS	AA_TRNA_LIGASE_II domain-containing protein	Aspartate--tRNA ligase, cytoplasmic	2	98	4	0.988	0.033	8.7E-01
G1SJN4	Q9BT78	COPS4	PCI domain-containing protein	COP9 signalosome complex subunit 4	2	100	4	0.988	0.048	8.8E-01
G1U7C7	Q5GLZ8	HERC4	HECT domain-containing protein	Probable E3 ubiquitin-protein ligase HERC4	2	96	4	0.988	0.038	8.7E-01
G1T2Z8	B4DUC8	MTAP	S-methyl-5~-thioadenosine phosphorylase	S-methyl-5~-thioadenosine phosphorylase	2	99	4	0.987	0.051	8.7E-01
G1TD98		GSR	Glutathione reductase		1		4	0.987	0.053	8.7E-01
G1SHV9	P49720	PSMB3	Proteasome subunit beta	Proteasome subunit beta type-3	2	99	4	0.987	0.064	8.8E-01
G1SUP1	A0A0D9SEY1	MAP4K4	Mitogen-activated protein kinase kinase kinase kinase 4	Mitogen-activated protein kinase kinase kinase kinase 4	2	91	4	0.987	0.073	8.8E-01
G1T0K1	Q9BTV4	TMEM43	Uncharacterized protein	Transmembrane protein 43	3	92	4	0.987	0.030	8.5E-01
G1SCQ0		EDEM3	alpha-1,2-Mannosidase		1		4	0.986	0.125	9.1E-01
G1T7H0	A0A1W2PPS1	HNRNPU	Uncharacterized protein	Heterogeneous nuclear ribonucleoprotein U	3	96	4	0.986	0.041	8.5E-01
G1T275	Q8WU90	ZC3H15	Uncharacterized protein	Zinc finger CCCH domain-containing protein 15	3	98	4	0.985	0.103	8.9E-01
G1SVW7	G3V1U5	GOLT1B	Uncharacterized protein	Golgi transport 1 homolog B (S. cerevisiae), isoform CRA_c	3	100	4	0.985	0.063	8.6E-01
G1TDJ3	Q9BSJ8	ESYT1	Uncharacterized protein	Extended synaptotagmin-1	3	89	4	0.984	0.034	8.3E-01
G1TTU6	E7ERH2	SKP1	Uncharacterized protein	S-phase kinase-associated protein 1 (Fragment)	3	100	4	0.984	0.049	8.4E-01
G1SS79	P29966	MARCKS	Uncharacterized protein	Myristoylated alanine-rich C-kinase substrate	3	87	4	0.984	0.090	8.7E-01
G1SGX4	P62249	RPS16	Uncharacterized protein	40S ribosomal protein S16	3	100	4	0.982	0.152	9.0E-01
G1SFH8		PDCL3	Phosducin domain-containing protein		1		4	0.982	0.070	8.4E-01
G1SS70	P61247	RPS3A	40S ribosomal protein S3a	40S ribosomal protein S3a	2	100	4	0.982	0.111	8.7E-01
G1SKM5	Q8TAT6	NPLOC4	NPL4 homolog, ubiquitin recognition factor	Nuclear protein localization protein 4 homolog	2	92	4	0.982	0.035	8.0E-01
G1T168	P46783	RPS10	S10_plectin domain-containing protein	40S ribosomal protein S10	2	100	4	0.981	0.119	8.7E-01
G1SRA9	A0A0A0MTH3	ILK	Uncharacterized protein	Integrin-linked protein kinase	3	93	4	0.981	0.055	8.1E-01
G1U522	P13861	PRKAR2A	Uncharacterized protein	cAMP-dependent protein kinase type II-alpha regulatory subunit	3	90	4	0.981	0.023	7.8E-01
G1U3X5	P46821	MAP1B	Uncharacterized protein	Microtubule-associated protein 1B	3	88	4	0.980	0.154	8.9E-01
G1TZ26		GUK1	Guanylate kinase 1		1		4	0.980	0.113	8.6E-01
	O95747	OXSR1		Serine/threonine-protein kinase OSR1	4		4	0.980	0.068	8.2E-01
G1T748	Q5VZU9	TPP2	Uncharacterized protein	Tripeptidyl-peptidase 2	3	97	4	0.979	0.029	7.7E-01
G1T9H0	Q8NCA5	FAM98A	Uncharacterized protein	Protein FAM98A	3	92	4	0.979	0.056	7.9E-01
G1SZ19	Q9Y3L5	RAP2C	Uncharacterized protein	Ras-related protein Rap-2c	3	100	4	0.979	0.075	8.1E-01
U3KM64		CLTA	Clathrin light chain		1		4	0.977	0.035	7.5E-01
	O43765	SGTA		Small glutamine-rich tetratricopeptide repeat-containing protein alpha	4		4	0.976	0.063	7.8E-01
G1TXN1		NIT2	CN hydrolase domain-containing protein		1		4	0.976	0.077	7.9E-01
G1T2I4	P07814	EPRS	Glutamyl-prolyl-tRNA synthetase	Bifunctional glutamate/proline--tRNA ligase	2	89	4	0.975	0.030	7.2E-01

	Q9UNE7	STUB1		E3 ubiquitin-protein ligase CHIP	4		4	0.975	0.035	7.3E-01
G1SH86		TBCD	Tubulin folding cofactor D		1		4	0.975	0.076	7.8E-01
G1U8C4	H0YKK6	PSME1	Uncharacterized protein	Proteasome activator complex subunit 1	3	98	4	0.975	0.142	8.4E-01
G1U3S6	O15258	RER1	Protein RER1	Protein RER1	2	95	4	0.974	0.053	7.4E-01
G1SIX1	Q9H2G2	SLK	Uncharacterized protein	STE20-like serine/threonine-protein kinase	3	88	4	0.973	0.103	8.0E-01
G1SZW0	Q7L576	CYFIP1	Cytoplasmic FMR1-interacting protein	Cytoplasmic FMR1-interacting protein 1	2	98	4	0.973	0.071	7.5E-01
G1SI94		CRLF3	Cytokine receptor like factor 3		1		4	0.972	0.097	7.8E-01
	P68402	PAFAH1B2		Platelet-activating factor acetylhydrolase IB subunit beta	4		4	0.971	0.099	7.8E-01
G1T4Q9	P28074	PSMB5	Proteasome subunit beta	Proteasome subunit beta type-5	2	98	4	0.971	0.007	6.7E-01
G1T659	Q9UHY7	ENOPH1	Enolase-phosphatase E1	Enolase-phosphatase E1	2	97	4	0.971	0.193	8.6E-01
Q9GLC3		ATP1B3	Sodium/potassium-transporting ATPase subunit beta-3		1		4	0.971	0.099	7.7E-01
G1SYB4	P60953	CDC42	Uncharacterized protein	Cell division control protein 42 homolog	3	100	4	0.970	0.071	7.3E-01
	P55010	EIF5		Eukaryotic translation initiation factor 5	4		4	0.970	0.064	7.1E-01
G1SG59	Q7L9L4	MOB1B	Uncharacterized protein	MOB kinase activator 1B	3	100	4	0.969	0.037	6.7E-01
G1TCP2	A0A087X1E4	ARFIP2	AH domain-containing protein	Arfaptin-2	2	91	4	0.969	0.054	6.9E-01
G1T6P5	Q9NVJ2	ARL8B	ADP ribosylation factor like GTPase 8B	ADP-ribosylation factor-like protein 8B	2	100	4	0.969	0.039	6.7E-01
	P20908	COL5A1		Collagen alpha-1(V) chain	4		4	0.968	0.152	8.1E-01
G1SU71	P20618	PSMB1	Proteasome subunit beta	Proteasome subunit beta type-1	2	94	4	0.968	0.042	6.6E-01
G1SNL4	Q9Y385	UBE2J1	UBIQUITIN_CONJUGAT_2 domain-containing protein	Ubiquitin-conjugating enzyme E2 J1	2	94	4	0.968	0.079	7.2E-01
	A0A096LP07	GPS1		COP9 signalosome complex subunit 1	4		4	0.967	0.071	7.0E-01
G1SIL2	Q9UNW1	MINPP1	Multiple inositol-polyphosphate phosphatase 1	Multiple inositol polyphosphate phosphatase 1	2	87	4	0.966	0.131	7.8E-01
G1TEU8		TBCC	C-CAP/cofactor C-like domain-containing protein		1		4	0.966	0.059	6.7E-01
P40144		ADCY5	Adenylate cyclase type 5		1		4	0.966	0.187	8.3E-01
G1SDC6	Q9NXC5	MIOS	zinc_ribbon_16 domain-containing protein	GATOR complex protein MIOS	2	98	4	0.966	0.055	6.6E-01
G1T918	P28070	PSMB4	Proteasome subunit beta	Proteasome subunit beta type-4	2	95	4	0.966	0.061	6.7E-01
G1SEU9	Q9UHA4	LAMTOR3	Uncharacterized protein	Ragulator complex protein LAMTOR3	3	98	4	0.965	0.017	6.1E-01
G1U1M3	Q12904	AIMP1	tRNA-binding domain-containing protein	Aminoacyl tRNA synthase complex-interacting multifunctional protein 1	2	91	4	0.965	0.056	6.5E-01
G1SMG1		KANK1	KN motif and ankyrin repeat domains 1		1		4	0.964	0.029	6.1E-01
G1SL02	P63010	AP2B1	AP complex subunit beta	AP-2 complex subunit beta	2	98	4	0.964	0.037	6.2E-01
G1T4P8	O76003	GLRX3	Glutaredoxin 3	Glutaredoxin-3	2	93	4	0.964	0.034	6.1E-01
G1SZ14	P25788	PSMA3	Proteasome endopeptidase complex	Proteasome subunit alpha type-3	2	99	4	0.964	0.065	6.6E-01
	A0A0A0MSZ1	MARK3		Non-specific serine/threonine protein kinase	4		4	0.963	0.033	6.0E-01
G1SCQ1		AKR7L	Aldo_ket_red domain-containing protein		1		4	0.962	0.101	7.1E-01
G1SJG0	P28300	LOX	Uncharacterized protein	Protein-lysine 6-oxidase	3	88	4	0.961	0.064	6.4E-01
G1SM51	Q9UNM6	PSMD13	Proteasome 26S subunit, non-ATPase 13	26S proteasome non-ATPase regulatory subunit 13	2	92	4	0.961	0.028	5.7E-01
G1U6Y3		MEAK7	MTOR associated protein, eak-7 homolog		1		4	0.961	0.090	6.8E-01
G1SJ41	Q9Y6Y8	SEC23IP	DDHD domain-containing protein	SEC23-interacting protein	2	89	4	0.960	0.024	5.6E-01
G1SCI5	F5H6E2	MYO1C	Uncharacterized protein	Unconventional myosin-Ic	3	92	4	0.960	0.042	5.9E-01
B7NZD2	Q5VWC4	PSMD4	Proteasome 26S subunit, non-ATPase, 4 (Predicted)	26S proteasome non-ATPase regulatory subunit 4	2	99	4	0.960	0.073	6.4E-01
G1T5I3	A0A0C4DFM1	TM9SF4	Transmembrane 9 superfamily member	Transmembrane 9 superfamily member	2	100	4	0.959	0.089	6.7E-01
G1SQF7	Q14554	PDIA5	Uncharacterized protein	Protein disulfide-isomerase A5	3	92	4	0.959	0.073	6.3E-01
G1TSV3	Q8ND76	CCNY	Cyclin Y	Cyclin-Y	2	99	4	0.958	0.068	6.1E-01
G1T964		TMED1	GOLD domain-containing protein		1		4	0.957	0.077	6.3E-01
G1U101	J3KPF0	HECTD4	HECT domain-containing protein	Probable E3 ubiquitin-protein ligase HECTD4	2	97	4	0.957	0.080	6.3E-01
G1SJ61	Q9Y608	LRRFIP2	Uncharacterized protein	Leucine-rich repeat flightless-interacting protein 2	3	91	4	0.957	0.073	6.1E-01
G1SII9	Q5QJ74	TBCEL	Ubiquitin-like domain-containing protein	Tubulin-specific chaperone cofactor E-like protein	2	99	4	0.956	0.037	5.4E-01
G1TN13	A8CTZ0	ITSN1	Intersectin 1	Intersectin 1 short form variant 13	2	88	4	0.956	0.095	6.5E-01
G1U9D3	Q9UK41	VPS28	Vacuolar protein sorting-associated protein 28 homolog	Vacuolar protein sorting-associated protein 28 homolog	2	93	4	0.956	0.090	6.4E-01
	P50454	SERPINH1		Serpin H1	4		4	0.956	0.148	7.3E-01
G1SMG5	P15170	GSPT1	Tr-type G domain-containing protein	Eukaryotic peptide chain release factor GTP-binding subunit ERF3A	2	99	4	0.956	0.056	5.7E-01
G1SPL1	A0A286YF22	PHGDH	D-3-phosphoglycerate dehydrogenase	D-3-phosphoglycerate dehydrogenase	2	93	4	0.955	0.187	7.8E-01
G1T365	Q9H269	VPS16	Vacuolar protein sorting-associated protein 16 homolog	Vacuolar protein sorting-associated protein 16 homolog	2	98	4	0.955	0.029	5.1E-01
G1TJY2		CHID1	Chitinase domain containing 1		1		4	0.954	0.106	6.6E-01
G1TR92		SYDE1	Synapse defective Rho GTPase homolog 1		1		4	0.954	0.103	6.5E-01
G1SGD9		ATG7	Ubiquitin-like modifier-activating enzyme ATG7		1		4	0.953	0.037	5.0E-01
G1SZI7		CROT	Carn_acyltransf domain-containing protein		1		4	0.953	0.148	7.2E-01
G1SZN0	Q9NZB2	FAM120A	Uncharacterized protein	Constitutive coactivator of PPAR-gamma-like protein 1	3	94	4	0.952	0.050	5.3E-01
Q28647		PPP2R5B	Serine/threonine-protein phosphatase 2A 56 kDa regulatory subunit beta isoform		1		4	0.952	0.068	5.6E-01
	A0A0A0MRE1	EXOC7		Exocyst complex component 7 (Fragment)	4		4	0.952	0.015	4.7E-01
G1TXS2	A0A0D9SFK2	MYO18A	Uncharacterized protein	Unconventional myosin-XVIIIa	3	94	4	0.951	0.027	4.7E-01
G1U7C5	Q9P2E9	RRBP1	Uncharacterized protein	Ribosome-binding protein 1	3	83	4	0.951	0.066	5.4E-01
G1SUV0	Q9NW15	ANO10	Anoctamin	Anoctamin-10	2	94	4	0.950	0.077	5.7E-01
G1TBX9	Q9H8M7	MINDY3	DUF4205 domain-containing protein	Ubiquitin carboxyl-terminal hydrolase MINDY-3	2	99	4	0.950	0.209	7.7E-01
G1TB71	Q14008	CKAP5	Uncharacterized protein	Cytoskeleton-associated protein 5	3	97	4	0.950	0.045	5.0E-01
	Q9Y6I3	EPN1		Epsin-1	4		4	0.950	0.024	4.6E-01
G1U460	Q02252	ALDH6A1	Aldedh domain-containing protein	Methylmalonate-semialdehyde dehydrogenase [acylating], mitochondrial	2	95	4	0.950	0.104	6.2E-01
G1SP89	J3QLD9	FLOT2	PHB domain-containing protein	Flotillin-2	2	97	4	0.950	0.043	4.9E-01
G1SVA1	F2Z2X4	XPO4	CRM1_C domain-containing protein	Exportin-4	2	99	4	0.949	0.050	5.0E-01
G1SM01		AKAP9	A-kinase anchor protein 9		1		4	0.949	0.061	5.2E-01

G1T8E2	A0A3B3ITW1	GSK3B	Protein kinase domain-containing protein	Glycogen synthase kinase-3 beta	2	87	4	0.949	0.046	4.9E-01
G1SET5		CEMIP2	G8 domain-containing protein		1		4	0.949	0.038	4.8E-01
G1T5J8	Q9UJW0	DCTN4	Uncharacterized protein	Dynactin subunit 4	3	97	4	0.948	0.048	4.9E-01
G1TRI7	A0A0C4DG51	PNPLA8	Calcium-independent phospholipase A2-gamma	Calcium-independent phospholipase A2-gamma (Fragment)	2	90	4	0.948	0.160	7.0E-01
G1T4S5	Q8NFW8	CMAS	Uncharacterized protein	N-acylneuraminate cytidylyltransferase	3	96	4	0.948	0.051	4.9E-01
G1T044	A0A3B3IUC4	GLA	Alpha-galactosidase	Alpha-galactosidase	2	75	4	0.947	0.044	4.7E-01
G1T578	E9PHY0	ACP2	Uncharacterized protein	Lysosomal acid phosphatase	3	94	4	0.947	0.100	6.0E-01
G1TE37		ATP6AP1	ATPase H+ transporting accessory protein 1		1		4	0.947	0.105	6.0E-01
G1THP8	P46734	MAP2K3	Protein kinase domain-containing protein	Dual specificity mitogen-activated protein kinase kinase 3	2	97	4	0.946	0.062	5.0E-01
	P60468	SEC61B		Protein transport protein Sec61 subunit beta	4		4	0.946	0.130	6.5E-01
G1T8X7	Q96JH7	VCPIP1	OTU domain-containing protein	Deubiquitinating protein VCIP135	2	96	4	0.945	0.053	4.7E-01
G1SM65		GALNT16	Polypeptide N-acetylgalactosaminyltransferase		1		4	0.945	0.031	4.3E-01
G1SRB7	A0A2R8Y7U1	TPP1	Peptidase S53 domain-containing protein	Tripeptidyl-peptidase 1 (Fragment)	2	93	4	0.945	0.034	4.3E-01
G1SLD6	B3KWE1	HARS	Uncharacterized protein	Histidine--tRNA ligase, cytoplasmic	3	97	4	0.944	0.059	4.8E-01
G1SXG8	F8VU90	FKBP11	Peptidylprolyl isomerase	Peptidylprolyl isomerase	2	95	4	0.942	0.064	4.8E-01
G1TPB1		CRAT	Carnitine O-acetyltransferase		1		4	0.942	0.035	4.1E-01
G1T7F5	A0A1C7CYY0	ADD2	Aldolase_II domain-containing protein	Beta-adducin (Fragment)	2	93	4	0.941	0.042	4.2E-01
G1SL98	Q53EP0	FNDC3B	Uncharacterized protein	Fibronectin type III domain-containing protein 3B	3	97	4	0.941	0.028	3.9E-01
	V9GYD0	ARL2-SNX15		ARL2-SNX15 readthrough (NMD candidate)	4		4	0.941	0.087	5.2E-01
G1SZ34	Q9Y5S2	CDC42BPB	CDC42 binding protein kinase beta	Serine/threonine-protein kinase MRCK beta	2	92	4	0.940	0.081	5.0E-01
G1T302		THYN1	EVE domain-containing protein		1		4	0.940	0.016	3.7E-01
G1T466	A0AVT1	UBA6	UBA_e1_C domain-containing protein	Ubiquitin-like modifier-activating enzyme 6	2	93	4	0.940	0.026	3.8E-01
G1SUT8	O95486	SEC24A	Uncharacterized protein	Protein transport protein Sec24A	3	93	4	0.940	0.106	5.5E-01
G1SWM8	A0A0U1RQQ9	SCYL2	Protein kinase domain-containing protein	SCY1-like protein 2	2	94	4	0.939	0.028	3.8E-01
G1SH09	Q5VIR6	VPS53	VPS53, GARP complex subunit	Vacuolar protein sorting-associated protein 53 homolog	2	95	4	0.939	0.032	3.8E-01
G1T519	P25789	PSMA4	Proteasome subunit alpha type	Proteasome subunit alpha type-4	2	100	4	0.939	0.107	5.5E-01
G1T369	C9J8R4	DCUN1D1	DCN1-like protein	DCN1-like protein (Fragment)	2	100	4	0.938	0.036	3.8E-01
G1T974	A0A3B3IRK6	MOGS	Mannosyl-oligosaccharide glucosidase	Mannosyl-oligosaccharide glucosidase (Fragment)	2	86	4	0.938	0.078	4.8E-01
P40826	P54578	USP14	Ubiquitin carboxyl-terminal hydrolase 14	Ubiquitin carboxyl-terminal hydrolase 14	2	97	4	0.937	0.058	4.2E-01
G1T0H7	A0A087WWM0	TRAPPC3	Trafficking protein particle complex subunit	Trafficking protein particle complex subunit	2	94	4	0.937	0.103	5.3E-01
G1TJA8		FNBP1	Formin binding protein 1		1		4	0.937	0.068	4.4E-01
G1U3B8	K7ER96	TXNL1	PITH domain-containing protein	Thioredoxin-like protein 1 (Fragment)	2	100	4	0.936	0.034	3.6E-01
G1T332		GOT1	Aspartate aminotransferase		1		4	0.936	0.048	3.9E-01
G1SCZ9	Q92575	UBXN4	UBX domain-containing protein	UBX domain-containing protein 4	2	94	4	0.936	0.083	4.7E-01
G1T7N4	P41743	PRKCI	Protein kinase C	Protein kinase C iota type	2	99	4	0.936	0.051	3.9E-01
G1T147	Q8IZ52	CHPF	Hexosyltransferase	Chondroitin sulfate synthase 2	2	95	4	0.935	0.061	4.1E-01
G1U1E5	O60826	CCDC22	Coiled-coil domain containing 22	Coiled-coil domain-containing protein 22	2	75	4	0.935	0.026	3.4E-01
G1TUD6	P43686	PSMC4	Proteasome 26S subunit, ATPase 4	26S proteasome regulatory subunit 6B	2	100	4	0.932	0.074	4.2E-01
G1TBL6	A0A087WVQ6	CLTC	Clathrin heavy chain	Clathrin heavy chain	2	99	4	0.931	0.028	3.2E-01
G1T089		THUMPD3	THUMP domain containing 3		1		4	0.931	0.059	3.8E-01
G1TI39	P35241	RDX	FERM domain-containing protein	Radixin	2	99	4	0.931	0.090	4.6E-01
G1TI71	A0A087WYS1	UGP2	UTP--glucose-1-phosphate uridylyltransferase	UTP--glucose-1-phosphate uridylyltransferase	2	99	4	0.931	0.138	5.7E-01
G1T8P7	A0A2R8YFH5	SEC23B	Protein transport protein SEC23	Protein transport protein SEC23	2	95	4	0.930	0.026	3.1E-01
G1TBC9		TMEM119	Transmembrane protein 119		1		4	0.930	0.188	6.5E-01
G1SXX9	A0A0C4DGH3	NUMBL	NUMB like, endocytic adaptor protein	Numb-like protein	2	93	3	0.930	0.122	5.4E-01
G1SQU0	P15586	GNS	N-acetylglucosamine-6-sulfatase	N-acetylglucosamine-6-sulfatase	2	95	4	0.930	0.069	4.0E-01
	P39019	RPS19		40S ribosomal protein S19	4		4	0.930	0.190	6.5E-01
G1T358	Q96AG3	SLC25A46	Uncharacterized protein	Solute carrier family 25 member 46	3	94	4	0.930	0.074	4.1E-01
G1TA82	Q12768	WASHC5	Uncharacterized protein	WASH complex subunit 5	3	97	4	0.930	0.048	3.4E-01
G1SLZ8	Q06124	PTPN11	Tyrosine-protein phosphatase non-receptor type	Tyrosine-protein phosphatase non-receptor type 11	2	98	4	0.929	0.072	4.0E-01
G1T0B4	Q9Y3B3	TMED7	GOLD domain-containing protein	Transmembrane emp24 domain-containing protein 7	2	92	4	0.929	0.027	3.0E-01
G1STX3	Q8WVM8	SCFD1	Uncharacterized protein	Sec1 family domain-containing protein 1	3	96	4	0.929	0.060	3.6E-01
	A0A087X0W8	RELA		Transcription factor p65	4		4	0.928	0.109	4.9E-01
G1SL52	A0A0A0MS45	COG4	Cog4 domain-containing protein	Conserved oligomeric Golgi complex subunit 4	2	94	4	0.928	0.063	3.7E-01
G1SPZ2	Q13107	USP4	Ubiquitin carboxyl-terminal hydrolase	Ubiquitin carboxyl-terminal hydrolase 4	2	89	4	0.927	0.078	4.0E-01
G1SQ96	Q5H9R7	PPP6R3	Uncharacterized protein	Serine/threonine-protein phosphatase 6 regulatory subunit 3	3	92	4	0.926	0.021	2.7E-01
G1U0Q2	K7EJ78	RPS15	Uncharacterized protein	40S ribosomal protein S15	3	99	4	0.926	0.136	5.3E-01
	P10301	RRAS		Ras-related protein R-Ras	4		4	0.926	0.016	2.7E-01
	A0A0J9YXC7	LIMS4		LIM and senescent cell antigen-like-containing domain protein	4		3	0.926	0.029	3.7E-01
G1TA78	P23381	WARS	Tryptophan--tRNA ligase, cytoplasmic	Tryptophan--tRNA ligase, cytoplasmic	2	89	4	0.925	0.052	3.2E-01
G1SWN1	Q96H20	SNF8	Vacuolar-sorting protein SNF8	Vacuolar-sorting protein SNF8	2	100	4	0.925	0.029	2.8E-01
G1SNM5	A0A2R8Y566	RELCH	LisH domain-containing protein	RAB11-binding protein RELCH	2	96	4	0.925	0.055	3.3E-01
G1SN85	A0A0D9SEN1	FAP	Uncharacterized protein	Prolyl endopeptidase FAP	3	95	4	0.925	0.123	5.0E-01
G1SEM0	Q96AC1	FERMT2	PH domain-containing protein	Fermitin family homolog 2	2	100	4	0.925	0.081	3.9E-01
G1TED6	P08758	ANXA5	Annexin	Annexin A5	2	93	4	0.924	0.096	4.3E-01
G1SMM5	O60884	DNAJA2	Uncharacterized protein	DnaJ homolog subfamily A member 2	3	100	4	0.922	0.033	2.6E-01
G1SGR0	Q8N3P4	VPS8	Uncharacterized protein	Vacuolar protein sorting-associated protein 8 homolog	3	93	4	0.922	0.051	3.0E-01
G1SHI2		MYD88	Myeloid differentiation primary response protein MyD88		1		4	0.919	0.109	4.3E-01
G1SWK5	A0A1W2PNP0	PIGT	Uncharacterized protein	GPI transamidase component PIG-T (Fragment)	3	86	4	0.919	0.027	2.4E-01

G1T845	I3L0N3	NSF	Uncharacterized protein	Vesicle-fusing ATPase	3	99	4	0.919	0.016	2.2E-01
G1T4T7	Q9Y2A7	NCKAP1	Uncharacterized protein	Nck-associated protein 1	3	100	4	0.918	0.046	2.6E-01
G1TT64	Q68EM7	ARHGAP17	Uncharacterized protein	Rho GTPase-activating protein 17	3	91	4	0.917	0.025	2.2E-01
G1TE96	O43813	LANCL1	Uncharacterized protein	Glutathione S-transferase LANCL1	3	95	4	0.917	0.027	2.3E-01
G1T0Z6	Q9Y4X5	ARIH1	RBR-type E3 ubiquitin transferase	E3 ubiquitin-protein ligase ARIH1	2	98	4	0.917	0.063	3.0E-01
G1SQN6	J3KN59	BNIP2	CRAL-TRIO domain-containing protein	BCL2/adenovirus E1B 19 kDa protein-interacting protein 2	2	94	4	0.916	0.049	2.6E-01
G1TZV3	E7ENJ6	AP1M1	Adaptor related protein complex 1 subunit mu 1	AP-1 complex subunit mu-1	2	75	4	0.916	0.028	2.2E-01
G1SSV1	Q99627	COPS8	PCI domain-containing protein	COP9 signalosome complex subunit 8	2	99	4	0.915	0.076	3.2E-01
G1TF67		CORO1A	Coronin		1		3	0.914	0.330	7.1E-01
G1T4N4	A0A0B4J210	LARP1	HTH La-type RNA-binding domain-containing protein	La-related protein 1 (Fragment)	2	93	4	0.914	0.068	2.9E-01
G1T3Z2	Q9UNZ2	NSFL1C	Uncharacterized protein	NSFL1 cofactor p47	3	97	4	0.913	0.028	2.1E-01
G1SXI1		TTC27	TPR_REGION domain-containing protein		1		4	0.913	0.086	3.4E-01
	P63241	EIF5A		Eukaryotic translation initiation factor 5A-1	4		4	0.913	0.068	2.9E-01
G1SK52	Q5T6H7	XPNPEP1	Uncharacterized protein	Xaa-Pro aminopeptidase 1	3	97	4	0.912	0.043	2.3E-01
G1TDB3	P62851	RPS25	Uncharacterized protein	40S ribosomal protein S25	3	100	4	0.912	0.296	7.0E-01
G1SZE0	Q96QK1	VPS35	Vacuolar protein sorting-associated protein 35	Vacuolar protein sorting-associated protein 35	2	100	4	0.912	0.043	2.3E-01
G1T1D7	B1ALD9	POSTN	Uncharacterized protein	Periostin	3	90	4	0.912	0.054	2.5E-01
G1SSL8		HEATR5A	HEAT repeat containing 5A		1		4	0.911	0.108	3.9E-01
G1SM31	Q9P2G1	ANKIB1	RBR-type E3 ubiquitin transferase	Ankyrin repeat and IBR domain-containing protein 1	2	93	3	0.911	0.052	3.1E-01
G1T9L2	Q86VN1	VPS36	GLUE N-terminal domain-containing protein	Vacuolar protein-sorting-associated protein 36	2	97	4	0.911	0.092	3.4E-01
	Q9UBF2	COPG2		Coatomer subunit gamma-2	4		4	0.911	0.049	2.3E-01
G1SXH7	P12931	SRC	Tyrosine-protein kinase	Proto-oncogene tyrosine-protein kinase Src	2	96	4	0.910	0.007	1.8E-01
G1SMP3	A0A2R8Y6F8	CASK	Uncharacterized protein	Peripheral plasma membrane protein CASK	3	97	4	0.910	0.012	1.8E-01
G1U8P2	J3KTE4	RPL19	Ribosomal protein L19	Ribosomal protein L19	2	98	4	0.910	0.174	5.3E-01
G1SHM2		CLPTM1L	CLPTM1 like		1		4	0.910	0.110	3.8E-01
G1T0Z8	Q99471	PFDN5	Uncharacterized protein	Prefoldin subunit 5	3	99	4	0.910	0.084	3.1E-01
G1SDN8	Q92791	P3H4	Uncharacterized protein	Endoplasmic reticulum protein SC65	3	88	4	0.910	0.107	3.7E-01
G1T6S6		ATP6V1F	V-type proton ATPase subunit F		1		4	0.909	0.030	2.0E-01
G1TD47	Q8N3E9	PLCD3	Phosphoinositide phospholipase C	1-phosphatidylinositol 4,5-bisphosphate phosphodiesterase delta-3	2	88	4	0.909	0.032	2.0E-01
G1SIA6	Q5T2E6	ARMH3	DUF1741 domain-containing protein	Armadillo-like helical domain-containing protein 3	2	99	4	0.909	0.035	2.0E-01
G1T3P1	K7EKP8	ACOT7	Uncharacterized protein	Cytosolic acyl coenzyme A thioester hydrolase (Fragment)	3	98	4	0.907	0.040	2.0E-01
G1TE64	O95758	PTBP3	Uncharacterized protein	Polypyrimidine tract-binding protein 3	3	97	4	0.907	0.037	1.9E-01
G1SK48	K7ES02	BLMH	Bleomycin hydrolase	Bleomycin hydrolase (Fragment)	2	92	4	0.907	0.058	2.3E-01
G1SP83	Q9Y2D0	CA5B	Carbonic anhydrase 5B	Carbonic anhydrase 5B, mitochondrial	2	93	4	0.907	0.106	3.6E-01
G1U8F0	O95782	AP2A1	AP-2 complex subunit alpha	AP-2 complex subunit alpha-1	2	98	4	0.906	0.026	1.8E-01
G1SKT3	Q9NUJ1	ABHD10	AB hydrolase-1 domain-containing protein	Mycophenolic acid acyl-glucuronide esterase, mitochondrial	2	88	4	0.906	0.085	3.0E-01
G1THV8	Q9BT09	CNPY3	DUF3456 domain-containing protein	Protein canopy homolog 3	2	92	4	0.906	0.077	2.8E-01
	P35527	KRT9		Keratin, type I cytoskeletal 9	4		4	0.906	0.128	4.1E-01
G1SQG5	P40925	MDH1	Malate dehydrogenase	Malate dehydrogenase, cytoplasmic	2	97	4	0.906	0.081	2.9E-01
G1T670		SORT1	Proteasome subunit alpha type		1		4	0.906	0.030	1.8E-01
G1SU80	O60462	NRP2	Neuropilin	Neuropilin-2	2	95	4	0.905	0.074	2.6E-01
G1TII2	O95197	RTN3	Reticulon	Reticulon-3	2	72	4	0.905	0.069	2.5E-01
G1TQD3	Q01968	OCRL	Rho-GAP domain-containing protein	Inositol polyphosphate 5-phosphatase OCRL	2	95	4	0.905	0.034	1.8E-01
P30801		S100A6	Protein S100-A6		1		4	0.905	0.181	5.2E-01
G1SK00		USP5	Ubiquitin carboxyl-terminal hydrolase		1		4	0.904	0.036	1.8E-01
P06813	A0A075B7C0	CAPNS1	Calpain small subunit 1	Calpain small subunit 1 (Fragment)	2	96	4	0.903	0.112	3.5E-01
G1SEH1		PLSCR3	Phospholipid scramblase		1		4	0.903	0.075	2.5E-01
G1TBU8	P52306	RAP1GDS1	Uncharacterized protein	Rap1 GTPase-GDP dissociation stimulator 1	3	97	4	0.902	0.068	2.3E-01
G1SL38	F8VS81	TWF1	Twinfilin actin binding protein 1	Twinfilin-1 (Fragment)	2	96	4	0.902	0.024	1.6E-01
G1T4K5	A0A1B0GTW1	TJP2	Uncharacterized protein	Tight junction protein ZO-2	3	87	4	0.902	0.074	2.5E-01
G1TC10	P61081	UBE2M	UBIQUITIN_CONJUGAT_2 domain-containing protein	NEDD8-conjugating enzyme Ubc12	2	100	4	0.901	0.049	1.9E-01
G1SLS3	O43747	AP1G1	AP-1 complex subunit gamma	AP-1 complex subunit gamma-1	2	100	4	0.901	0.028	1.6E-01
G1U0B3	P53007	SLC25A1	Solute carrier family 25 member 1	Tricarboxylate transport protein, mitochondrial	2	86	4	0.900	0.082	2.6E-01
G1T2N2		LTN1	RING-type domain-containing protein		1		4	0.900	0.095	2.9E-01
G1SNY0	E9PJD9	RPL27A	Ribosomal_L18e/L15P domain-containing protein	60S ribosomal protein L27a	2	98	4	0.900	0.249	6.1E-01
G1SWC9	H3BMU1	IST1	Uncharacterized protein	IST1 homolog (Fragment)	3	100	4	0.900	0.046	1.8E-01
P63150	P63151	PPP2R2A	Serine/threonine-protein phosphatase 2A 55 kDa regulatory subunit B alpha isoform	Serine/threonine-protein phosphatase 2A 55 kDa regulatory subunit B alpha isoform	2	100	4	0.900	0.054	2.0E-01
G1SLC0	Q9BS26	ERP44	Thioredoxin domain-containing protein	Endoplasmic reticulum resident protein 44	2	97	4	0.900	0.072	2.3E-01
G1T5R0		TUBGCP3	Gamma-tubulin complex component		1		4	0.899	0.089	2.7E-01
G1SST7	Q96SL4	GPX7	Glutathione peroxidase	Glutathione peroxidase 7	2	92	4	0.899	0.073	2.3E-01
G1SST9	P54727	RAD23B	Uncharacterized protein	UV excision repair protein RAD23 homolog B	3	94	4	0.898	0.044	1.7E-01
G1SKS9	A0A087WSW9	TXNRD1	Glutaredoxin domain-containing protein	Thioredoxin reductase 1, cytoplasmic	2	94	4	0.898	0.024	1.4E-01
G1SVW5	P36578	RPL4	Ribos_L4_asso_C domain-containing protein	60S ribosomal protein L4	2	96	4	0.898	0.143	4.0E-01
G1U9R6	P02751	FN1	Fibronectin	Fibronectin	2	70	4	0.898	0.209	5.3E-01
G1SDG2	P48739	PITPNB	Phosphatidylinositol transfer protein beta	Phosphatidylinositol transfer protein beta isoform	2	97	4	0.898	0.035	1.5E-01
G1T3S7	O60476	MAN1A2	alpha-1,2-Mannosidase	Mannosyl-oligosaccharide 1,2-alpha-mannosidase IB	2	95	4	0.896	0.156	4.3E-01
G1T726	A0A0A0MSE2	HADH	Uncharacterized protein	Hydroxyacyl-coenzyme A dehydrogenase, mitochondrial	3	92	4	0.896	0.084	2.5E-01
G1SZR7	Q86UP2	KTN1	Uncharacterized protein	Kinectin	3	90	4	0.896	0.091	2.6E-01
G1SJ77	Q9Y696	CLIC4	Chloride intracellular channel protein	Chloride intracellular channel protein 4	2	100	4	0.896	0.082	2.4E-01

G1SN17		PPP4R1	WRNPLPNID domain-containing protein		1		4	0.896	0.029	1.4E-01
G1T8S0	Q9C0D5	TANC1	Uncharacterized protein	Protein TANC1	3	85	4	0.895	0.072	2.1E-01
G1SP48	O60701	UGDH	UDP-glucose 6-dehydrogenase	UDP-glucose 6-dehydrogenase	2	96	4	0.894	0.063	1.9E-01
G1STX4	P61011	SRP54	Signal recognition particle 54 kDa protein	Signal recognition particle 54 kDa protein	2	99	4	0.894	0.064	1.9E-01
G1SCP0	O00203	AP3B1	AP-3 complex subunit beta	AP-3 complex subunit beta-1	2	88	4	0.894	0.036	1.4E-01
G1SCF0	P13807	GYS1	Glycogen [starch] synthase	Glycogen [starch] synthase, muscle	2	96	4	0.894	0.110	3.0E-01
G1SHF1	Q9UHV9	PFDN2	Uncharacterized protein	Prefoldin subunit 2	3	99	4	0.894	0.031	1.3E-01
	A0A494C1N0	FKBP2		Peptidylprolyl isomerase	4		4	0.893	0.105	2.8E-01
G1SV81	Q9BTE1	DCTN5	Uncharacterized protein	Dynactin subunit 5	3	100	4	0.892	0.126	3.4E-01
G1SWY3	A0A1B0GUZ7	EFR3A	Uncharacterized protein	Protein EFR3 homolog A	3	98	4	0.892	0.088	2.4E-01
G1STP3	O75718	CRTAP	Uncharacterized protein	Cartilage-associated protein	3	94	4	0.892	0.095	2.5E-01
U3KNY1	Q12797	ASPH	Aspartate beta-hydroxylase	Aspartyl/asparaginyl beta-hydroxylase	2	96	4	0.892	0.138	3.6E-01
G1SWK8	Q5TBG5	PSMB7	Proteasome subunit beta	Proteasome subunit beta (Fragment)	2	84	4	0.892	0.026	1.2E-01
G1SZJ5	O15212	PFDN6	Uncharacterized protein	Prefoldin subunit 6	3	100	4	0.891	0.111	2.9E-01
G1STU4	E9PIE3	CAVIN3	Uncharacterized protein	Caveolae-associated protein 3	3	78	4	0.891	0.097	2.6E-01
G1TB18	Q07960	ARHGAP1	Uncharacterized protein	Rho GTPase-activating protein 1	3	95	4	0.890	0.036	1.3E-01
G1SCF4	G8JLD5	DNM1L	Uncharacterized protein	Dynamin-1-like protein	3	93	4	0.890	0.051	1.5E-01
G1TSL5	C9J6N9	UFD1	Uncharacterized protein	Ubiquitin recognition factor in ER-associated degradation protein 1 (Fragment)	3	100	4	0.890	0.032	1.2E-01
	O14818	PSMA7		Proteasome subunit alpha type-7	4		4	0.890	0.062	1.7E-01
G1SR93	B8ZZA2	FAM126A	Uncharacterized protein	Hyccin	3	98	4	0.888	0.046	1.4E-01
G1SL53	Q66K14	TBC1D9B	Uncharacterized protein	TBC1 domain family member 9B	3	89	4	0.888	0.023	1.1E-01
G1TDI6		BLOC1S6	Biogenesis of lysosome-related organelles complex 1 subunit 6		1		3	0.887	0.015	1.8E-01
G1T4Q7		ATP11C	Phospholipid-transporting ATPase		1		4	0.887	0.173	4.2E-01
G1SNS3	Q6P4E1	CASC4	Uncharacterized protein	Protein CASC4	3	89	4	0.887	0.067	1.7E-01
G1U5L3	P49257	LMAN1	L-type lectin-like domain-containing protein	Protein ERGIC-53	2	91	4	0.887	0.068	1.7E-01
G1TIB4	P62857	RPS28	Ribosomal protein S28	40S ribosomal protein S28	2	100	4	0.886	0.199	4.7E-01
G1SPL8		NGB	Neuroglobin		1		4	0.886	0.059	1.5E-01
G1SUK4		MPI	Mannose-6-phosphate isomerase		1		4	0.885	0.209	4.8E-01
G1SEN8		SCCPDH	Sacchrp_dh_NADP domain-containing protein		1		4	0.884	0.168	4.0E-01
G1T7Y7	B1AK87	CAPZB	F-actin-capping protein subunit beta	F-actin-capping protein subunit beta	2	100	4	0.884	0.027	1.0E-01
G1SSZ1	Q01658	DR1	CBFD_NFYB_HMF domain-containing protein	Protein Dr1	2	100	4	0.884	0.051	1.3E-01
Q29502	Q13177	PAK2	Serine/threonine-protein kinase PAK 2	Serine/threonine-protein kinase PAK 2	2	98	4	0.883	0.027	1.0E-01
G1SDW3		CDS2	Phosphatidate cytidylyltransferase		1		4	0.883	0.091	2.1E-01
G1U3F3		NEXN	Ig-like domain-containing protein		1		4	0.883	0.147	3.4E-01
G1SH85	A0A2R8YF87	VPS33A	Uncharacterized protein	Vacuolar protein sorting-associated protein 33A	3	91	4	0.882	0.027	9.9E-02
	J3KR44	OTUB1		Ubiquitin thioesterase	4		4	0.881	0.048	1.2E-01
G1SH25	P40616	ARL1	Uncharacterized protein	ADP-ribosylation factor-like protein 1	3	99	4	0.881	0.040	1.1E-01
G1TM86	A2AB27	GNL1	G protein nucleolar 1 (putative)	Guanine nucleotide-binding protein-like 1 (Fragment)	2	98	4	0.881	0.030	9.9E-02
G1SXT1	O43865	AHCYL1	AdoHcyase_NAD domain-containing protein	S-adenosylhomocysteine hydrolase-like protein 1	2	100	4	0.881	0.065	1.5E-01
G1SCL6	A0A494C1J1	SPECC1L	Calponin-homology (CH) domain-containing protein	Cytospin-A	2	90	4	0.881	0.040	1.1E-01
G1SZU0		URB2	Urb2 domain-containing protein		1		4	0.881	0.033	1.0E-01
G1SHA4		TEP1	Telomerase associated protein 1		1		4	0.880	0.135	3.1E-01
G1T8H8	P08183	ABCB1	Uncharacterized protein	ATP-dependent translocase ABCB1	3	88	4	0.880	0.056	1.3E-01
G1SIB0	Q12907	LMAN2	L-type lectin-like domain-containing protein	Vesicular integral-membrane protein VIP36	2	98	4	0.880	0.047	1.2E-01
	A8MZF9	DRG2		Developmentally-regulated GTP-binding protein 2	4		4	0.880	0.057	1.3E-01
G1SDA4	P53618	COPB1	Coatomer subunit beta	Coatomer subunit beta	2	99	4	0.879	0.076	1.7E-01
G1SVG6	P21359	NF1	Uncharacterized protein	Neurofibromin	3	99	4	0.878	0.048	1.1E-01
G1SJ23	P07384	CAPN1	Calpain-1 catalytic subunit	Calpain-1 catalytic subunit	2	90	4	0.878	0.045	1.1E-01
G1T4Z0	Q96PY5	FMNL2	Formin like 2	Formin-like protein 2	2	97	4	0.877	0.026	8.4E-02
G1T0G0		P3H2	Fe2OG dioxygenase domain-containing protein		1		4	0.876	0.076	1.6E-01
G1TBG2	Q96KP1	EXOC2	Exocyst complex component 2	Exocyst complex component 2	2	94	4	0.876	0.048	1.1E-01
G1T860	M0R0P8	MYO9B	Myosin IXB	Unconventional myosin-IXb	2	83	4	0.876	0.022	8.1E-02
G1T706	O00442	RTCA	Uncharacterized protein	RNA 3~-terminal phosphate cyclase	3	96	4	0.876	0.030	8.5E-02
G1T198	Q8TDX7	NEK7	Protein kinase domain-containing protein	Serine/threonine-protein kinase Nek7	2	98	4	0.875	0.087	1.8E-01
G1TA48	Q9H223	EHD4	Uncharacterized protein	EH domain-containing protein 4	3	97	4	0.875	0.036	9.0E-02
G1SGJ4		SRR	PALP domain-containing protein		1		4	0.875	0.109	2.2E-01
G1SDL9	Q9UEU0	VTI1B	t-SNARE coiled-coil homology domain-containing protein	Vesicle transport through interaction with t-SNAREs homolog 1B	2	93	4	0.874	0.080	1.6E-01
G1T087	Q93034	CUL5	Cullin-5	Cullin-5	2	100	4	0.874	0.025	7.9E-02
Q28717	F6WIT2	PTPA	Serine/threonine-protein phosphatase 2A activator	Serine/threonine-protein phosphatase 2A activator	2	97	4	0.874	0.063	1.3E-01
G1SRT1	Q9UKG1	APPL1	Adaptor protein, phosphotyrosine interacting with PH domain and leucine zipper 1	DCC-interacting protein 13-alpha	2	98	4	0.874	0.036	8.8E-02
G1T1F0	A0A2R8Y811	RPS14	Uncharacterized protein	40S ribosomal protein S14 (Fragment)	3	100	4	0.874	0.145	3.0E-01
G1SH26	F6TLX2	GLOD4	Glyoxalase domain containing 4	Glyoxalase domain-containing protein 4	2	81	4	0.872	0.068	1.3E-01
G1SQL0	P62191	PSMC1	AAA domain-containing protein	26S proteasome regulatory subunit 4	2	100	4	0.872	0.050	1.0E-01
G1SPV0	P61086	UBE2K	Uncharacterized protein	Ubiquitin-conjugating enzyme E2 K	3	100	4	0.872	0.135	2.7E-01
G1SS73	Q92499	DDX1	Uncharacterized protein	ATP-dependent RNA helicase DDX1	3	98	4	0.872	0.007	6.6E-02
G1SVH0	P23526	AHCY	AdoHcyase_NAD domain-containing protein	Adenosylhomocysteinase	2	97	4	0.872	0.070	1.3E-01
G1T196	Q13033	STRN3	WD_REPEATS_REGION domain-containing protein	Striatin-3	2	92	4	0.871	0.090	1.7E-01
G1SUY5	E7EVZ5	PCYOX1L	Prenylcys_lyase domain-containing protein	Prenylcysteine oxidase-like	2	95	4	0.871	0.100	1.9E-01
G1SNM1	P41250	GARS	Uncharacterized protein	Glycine--tRNA ligase	3	95	4	0.870	0.080	1.5E-01

G1TPN2	K7EQA9	CDC37	Cell division cycle 37	Hsp90 co-chaperone Cdc37 (Fragment)	2	57	4	0.870	0.082	1.5E-01
	A0A494C0A9	CBFB		Core-binding factor subunit beta	4		4	0.869	0.062	1.1E-01
G1STY8	G3V5E4	GNPNAT1	Glucosamine 6-phosphate N-acetyltransferase	Glucosamine 6-phosphate N-acetyltransferase	2	100	4	0.869	0.055	1.0E-01
G1TRS4	O95302	FKBP9	Peptidylprolyl isomerase	Peptidyl-prolyl cis-trans isomerase FKBP9	2	97	4	0.868	0.122	2.3E-01
G1SNI2	Q12841	FSTL1	Kazal-like domain-containing protein	Follistatin-related protein 1	2	94	4	0.868	0.086	1.5E-01
G1SD09	A0A0J9YYJ0	CNPY4	Canopy FGF signaling regulator 4	Protein canopy homolog 4 (Fragment)	2	97	4	0.868	0.101	1.8E-01
G1SIG2	P46108	CRK	Uncharacterized protein	Adapter molecule crk	3	99	4	0.868	0.032	7.2E-02
G1SFU4	Q9UIQ6	LNPEP	Uncharacterized protein	Leucyl-cystinyl aminopeptidase	3	90	4	0.868	0.039	7.8E-02
G1U7Q6	H7C1D4	TSN	Uncharacterized protein	Translin (Fragment)	3	99	4	0.868	0.084	1.5E-01
G1SIN4	X6RLX0	ERC1	FIP-RBD domain-containing protein	ELKS/Rab6-interacting/CAST family member 1	2	98	4	0.867	0.063	1.1E-01
G1TKY3	P17655	CAPN2	Calpain-2 catalytic subunit	Calpain-2 catalytic subunit	2	94	4	0.867	0.107	1.9E-01
	P09497-2	CLTB		Isoform Non-brain of Clathrin light chain B	4		4	0.866	0.074	1.2E-01
G1ST51		CLIC2	Chloride intracellular channel protein		1		4	0.866	0.047	8.3E-02
G1T4H0	Q9NYL9	TMOD3	Uncharacterized protein	Tropomodulin-3	3	93	4	0.866	0.044	7.9E-02
G1T1T4		ADPGK	ADP dependent glucokinase		1		4	0.866	0.031	6.8E-02
G1SV13	P22314	UBA1	Ubiquitin-like modifier-activating enzyme 1	Ubiquitin-like modifier-activating enzyme 1	2	97	4	0.865	0.037	7.2E-02
G1SMA1	P49755	TMED10	Transmembrane emp24 domain-containing protein 10	Transmembrane emp24 domain-containing protein 10	2	95	4	0.865	0.079	1.3E-01
G1T2L1	A0A024RA52	PSMA2	Proteasome subunit alpha type	Proteasome subunit alpha type	2	100	4	0.865	0.012	5.6E-02
G1SDR2	P24844	MYL9	Uncharacterized protein	Myosin regulatory light polypeptide 9	3	99	4	0.865	0.101	1.7E-01
G1SGY2	H0YC15	PTPN12	Tyrosine-protein phosphatase non-receptor type 12	Tyrosine-protein phosphatase non-receptor type 12 (Fragment)	2	90	4	0.864	0.025	6.0E-02
P68105	P68104	EEF1A1	Elongation factor 1-alpha 1	Elongation factor 1-alpha 1	2	100	4	0.864	0.107	1.8E-01
G1SPV8	O94829	IPO13	Importin N-terminal domain-containing protein	Importin-13	2	100	4	0.863	0.055	8.8E-02
G1TIZ5	Q8NBS9	TXNDC5	Uncharacterized protein	Thioredoxin domain-containing protein 5	3	87	4	0.863	0.064	1.0E-01
	P02533	KRT14		Keratin, type I cytoskeletal 14	4		4	0.863	0.175	3.3E-01
G1SXL6	O94915	FRYL	Uncharacterized protein	Protein furry homolog-like	3	97	4	0.863	0.080	1.3E-01
G1TRZ2		LAMP1	Lysosomal associated membrane protein 1		1		4	0.862	0.120	2.1E-01
U3KP45		SYNE1	Spectrin repeat containing nuclear envelope protein 1		1		4	0.862	0.051	8.0E-02
G1T1R9	Q96A65	EXOC4	Sec8_exocyst domain-containing protein	Exocyst complex component 4	2	95	4	0.861	0.014	5.2E-02
G1SV22	P30086	PEBP1	Phosphatidylethanolamine-binding protein 1	Phosphatidylethanolamine-binding protein 1	2	89	4	0.861	0.034	6.3E-02
G1TER0	Q9NV70	EXOC1	Sec3-PIP2_bind domain-containing protein	Exocyst complex component 1	2	98	4	0.861	0.062	9.4E-02
G1TBH6	Q9P0S9	TMEM14C	Uncharacterized protein	Transmembrane protein 14C	3	92	4	0.861	0.156	2.8E-01
G1T2I7	Q9HCJ1	ANKH	Uncharacterized protein	Progressive ankylosis protein homolog	3	99	4	0.860	0.114	1.9E-01
G1SIA1	A0A087WWA3	KIF1B	Uncharacterized protein	Kinesin-like protein KIF1B	3	96	4	0.859	0.044	6.8E-02
G1TYU5		QTRT1	Queuine tRNA-ribosyltransferase		1		4	0.859	0.039	6.4E-02
G1T5H8	E7EX17	EIF4B	RRM domain-containing protein	Eukaryotic translation initiation factor 4B	2	93	4	0.859	0.047	7.0E-02
G1TEJ4	Q86VW0	SESTD1	SEC14 and spectrin domain containing 1	SEC14 domain and spectrin repeat-containing protein 1	2	95	3	0.858	0.006	9.7E-02
U3KN73	Q9UBQ0	VPS29	Vacuolar protein sorting-associated protein 29	Vacuolar protein sorting-associated protein 29	2	100	4	0.858	0.056	7.9E-02
G1T8U2		GMDS	GDP-mannose 4,6-dehydratase		1		3	0.858	0.048	1.1E-01
G1T416	Q9UP83	COG5	Uncharacterized protein	Conserved oligomeric Golgi complex subunit 5	3	91	4	0.857	0.041	6.2E-02
G1SKT1	Q7Z7H5	TMED4	GOLD domain-containing protein	Transmembrane emp24 domain-containing protein 4	2	94	4	0.854	0.067	8.7E-02
G1SPB8	Q99733	NAP1L4	Uncharacterized protein	Nucleosome assembly protein 1-like 4	3	93	4	0.854	0.076	1.0E-01
G1TZ40	Q5M775	SPECC1	Calponin-homology (CH) domain-containing protein	Cytospin-B	2	88	4	0.854	0.122	1.9E-01
	Q8WXF7-2	ATL1		Isoform 2 of Atlastin-1	4		4	0.854	0.105	1.5E-01
G1T9Y4	E5RIU9	CHMP7	Uncharacterized protein	Charged multivesicular body protein 7 (Fragment)	3	69	4	0.853	0.007	4.0E-02
	Q9UQ13	SHOC2		Leucine-rich repeat protein SHOC-2	4		3	0.852	0.104	1.7E-01
P13642	P49591	SARS	Serine--tRNA ligase, cytoplasmic	Serine--tRNA ligase, cytoplasmic	2	97	4	0.852	0.090	1.2E-01
	Q8WUW1	BRK1		Protein BRICK1	4		4	0.851	0.084	1.1E-01
G1TRH5	Q9ULV4	CORO1C	Coronin	Coronin-1C	2	97	4	0.850	0.073	8.7E-02
G1SDU5	H3BPE1	MACF1	Uncharacterized protein	Microtubule-actin cross-linking factor 1, isoforms 1/2/3/5	3	88	4	0.850	0.065	7.6E-02
G1U9C1	P50570	DNM2	Dynamin 2	Dynamin-2	2	96	4	0.850	0.067	7.9E-02
G1SGX3	P22059	OSBP	Oxysterol-binding protein	Oxysterol-binding protein 1	2	97	4	0.849	0.070	8.2E-02
G1TJ80	Q9Y3Q3	TMED3	Transmembrane p24 trafficking protein 3	Transmembrane emp24 domain-containing protein 3	2	91	4	0.849	0.088	1.1E-01
G1SZR4	H7BZ14	PPIL3	Peptidyl-prolyl cis-trans isomerase	Peptidyl-prolyl cis-trans isomerase (Fragment)	2	100	4	0.849	0.073	8.4E-02
G1STQ7		TMEM97	Transmembrane protein 97		1		4	0.849	0.059	6.8E-02
G1TAP1	Q15181	PPA1	Uncharacterized protein	Inorganic pyrophosphatase	3	96	4	0.848	0.149	2.3E-01
	M0QXF7	MYDGF		Myeloid-derived growth factor (Fragment)	4		4	0.848	0.149	2.2E-01
G1TJC8		PEX5	TPR_REGION domain-containing protein		1		4	0.848	0.043	5.1E-02
G1SJ72		CSPG4	Chondroitin sulfate proteoglycan 4		1		4	0.848	0.147	2.2E-01
G1SJS2	Q14232	EIF2B1	Uncharacterized protein	Translation initiation factor eIF-2B subunit alpha	3	96	4	0.847	0.082	9.6E-02
G1TBA4	Q86UY8	NT5DC3	Uncharacterized protein	5~-nucleotidase domain-containing protein 3	3	97	4	0.846	0.068	7.4E-02
G1SW24	P49588	AARS	AA_TRNA_LIGASE_II_ALA domain-containing protein	Alanine--tRNA ligase, cytoplasmic	2	93	4	0.845	0.123	1.7E-01
G1TQV4	F6SKB8	NECAP2	DUF1681 domain-containing protein	Adaptin ear-binding coat-associated protein 2	2	95	4	0.845	0.117	1.5E-01
G1SYN5	J3QRU4	VAMP2	V-SNARE coiled-coil homology domain-containing protein	Vesicle-associated membrane protein 2	2	99	4	0.845	0.042	4.6E-02
G1SMK4		UCKL1	Uridine-cytidine kinase 1 like 1		1		4	0.844	0.072	7.6E-02
	Q14318	FKBP8		Peptidyl-prolyl cis-trans isomerase FKBP8	4		4	0.843	0.073	7.6E-02
G1SDU1	Q96A49	SYAP1	BSD domain-containing protein	Synapse-associated protein 1	2	89	4	0.843	0.033	3.8E-02
G1TF32	F5H6I7	ATL3	Atlastin GTPase 3	Atlastin-3	2	95	4	0.842	0.023	3.3E-02
	J3KN75	TBC1D8B		TBC1 domain family member 8B	4		4	0.842	0.097	1.1E-01
G1SZT8	P55735	SEC13	WD_REPEATS_REGION domain-containing protein	Protein SEC13 homolog	2	95	4	0.842	0.032	3.7E-02

G1SNL7	Q9H1I8	ASCC2	Activating signal cointegrator 1 complex subunit 2	Activating signal cointegrator 1 complex subunit 2	2	86	4	0.841	0.069	6.9E-02
G1SZM0	Q9BRT3	MIEN1	Uncharacterized protein	Migration and invasion enhancer 1	3	95	4	0.840	0.027	3.3E-02
G1SR53	P04066	FUCA1	Alpha-L-fucosidase	Tissue alpha-L-fucosidase	2	83	4	0.840	0.079	7.8E-02
A7X8X3		HPRT	Hypoxanthine phosphoribosyltransferase		1		4	0.840	0.115	1.3E-01
G1T090	G3V180	DPP3	Dipeptidyl peptidase 3	Dipeptidyl peptidase 3	2	94	4	0.840	0.053	4.9E-02
G1SIP7	O14976	GAK	Cyclin G associated kinase	Cyclin-G-associated kinase	2	77	4	0.840	0.093	9.9E-02
G1T3V0	A0A024R4E5	HDLBP	Uncharacterized protein	High density lipoprotein binding protein (Vigilin), isoform CRA_a	3	97	4	0.839	0.024	3.2E-02
G1SZW8	M0R165	EPS15L1	Epidermal growth factor receptor pathway substrate 15 like 1	Epidermal growth factor receptor substrate 15-like 1	2	88	4	0.839	0.052	4.8E-02
G1SJJ2	Q9NZ32	ACTR10	Uncharacterized protein	Actin-related protein 10	3	96	4	0.839	0.088	8.9E-02
G1SEJ4	P21281	ATP6V1B2	Vacuolar proton pump subunit B	V-type proton ATPase subunit B, brain isoform	2	99	4	0.839	0.066	6.1E-02
G1SRF5	Q9UPN7	PPP6R1	Protein phosphatase 6 regulatory subunit 1	Serine/threonine-protein phosphatase 6 regulatory subunit 1	2	84	4	0.839	0.084	8.2E-02
G1T9P1		PEAK1	Pseudopodium enriched atypical kinase 1		1		4	0.838	0.132	1.6E-01
G1TT75	O00264	PGRMC1	Cytochrome b5 heme-binding domain-containing protein	Membrane-associated progesterone receptor component 1	2	93	4	0.838	0.106	1.2E-01
G1SUF5	Q8TF66	LRRC15	LRRCT domain-containing protein	Leucine-rich repeat-containing protein 15	2	90	4	0.837	0.156	2.1E-01
G1T057	D6RCE2	TTC37	Uncharacterized protein	Tetratricopeptide repeat protein 37 (Fragment)	3	83	4	0.837	0.070	6.3E-02
G1T182	Q86UE4	MTDH	Metadherin	Protein LYRIC	2	88	4	0.837	0.107	1.1E-01
G1TLD3	Q02818	NUCB1	Nucleobindin 1	Nucleobindin-1	2	87	4	0.836	0.111	1.2E-01
G1TR97	A0A0A0MSA7	EIF4G3	Uncharacterized protein	Eukaryotic translation initiation factor 4 gamma 3	3	89	4	0.836	0.121	1.4E-01
G1SVZ8		C9orf64	Queuosine salvage protein		1		3	0.836	0.060	8.3E-02
G1TEA8	P12268	IMPDH2	Inosine-5~-monophosphate dehydrogenase	Inosine-5~-monophosphate dehydrogenase 2	2	99	4	0.835	0.078	7.0E-02
G1TBC4	G3V126	ATP6V1H	V-type proton ATPase subunit H	V-type proton ATPase subunit H	2	99	4	0.834	0.071	6.0E-02
	O14908	GIPC1		PDZ domain-containing protein GIPC1	4		4	0.834	0.071	6.0E-02
	J3QQX2	ARHGDIA		Rho GDP-dissociation inhibitor 1	4		4	0.833	0.136	1.6E-01
G1T9W8	Q8TC07	TBC1D15	Rab-GAP TBC domain-containing protein	TBC1 domain family member 15	2	92	4	0.833	0.132	1.5E-01
G1TIW9		FBXO6	F-box protein 6		1		4	0.833	0.059	4.7E-02
G1SJH1	H7BXE3	SLTM	RRM domain-containing protein	SAFB-like transcription modulator (Fragment)	2	92	4	0.833	0.072	5.9E-02
G1SQ12	Q8TDJ6	DMXL2	WD_REPEATS_REGION domain-containing protein	DmX-like protein 2	2	93	4	0.832	0.030	2.8E-02
G1T7X6	G3XAI2	LAMB1	Uncharacterized protein	Laminin subunit beta-1	3	91	4	0.831	0.227	3.3E-01
G1T3U1	Q9H8Y8	GORASP2	GRASP55_65 domain-containing protein	Golgi reassembly-stacking protein 2	2	91	4	0.830	0.052	3.8E-02
G1T085		SVIL	HP domain-containing protein		1		4	0.830	0.141	1.6E-01
G1TN89	P98160	HSPG2	Heparan sulfate proteoglycan 2	Basement membrane-specific heparan sulfate proteoglycan core protein	2	90	4	0.829	0.118	1.2E-01
G1T8K2	A0A0U1RR22	PACSIN2	Uncharacterized protein	Protein kinase C and casein kinase substrate in neurons protein 2 (Fragment)	3	93	4	0.829	0.132	1.4E-01
G1SQ70	P01023	A2M	Uncharacterized protein	Alpha-2-macroglobulin	3	78	4	0.829	0.161	2.0E-01
G1T7D0		SRRM2	Serine/arginine repetitive matrix 2		1		3	0.827	0.109	1.2E-01
G1SS77	Q15477	SKIV2L	Uncharacterized protein	Helicase SKI2W	3	95	4	0.827	0.039	2.8E-02
G1TPW2		MRI1	Methylthioribose-1-phosphate isomerase		1		4	0.827	0.016	2.1E-02
U3KMU7	A0A024R571	EHD1	EH domain containing 1	EH domain-containing protein 1	2	99	4	0.826	0.053	3.5E-02
G1U2R2	E9PLK3	NPEPPS	Aminopeptidase	Aminopeptidase	2	98	4	0.826	0.062	4.2E-02
G1SX73	E5RGS4	PFDN1	Uncharacterized protein	Prefoldin subunit 1	3	88	4	0.826	0.090	7.1E-02
G1TS73	H0YBP1	PTK2	Protein tyrosine kinase 2	Focal adhesion kinase 1 (Fragment)	2	89	4	0.826	0.045	3.0E-02
G1TDU0	P33176	KIF5B	Kinesin-like protein	Kinesin-1 heavy chain	2	99	4	0.825	0.070	4.9E-02
G1TDQ5	A0A2R8Y891	PFKM	ATP-dependent 6-phosphofructokinase	ATP-dependent 6-phosphofructokinase	2	97	4	0.825	0.051	3.3E-02
G1SEK2		PPM1F	PPM-type phosphatase domain-containing protein		1		4	0.824	0.186	2.3E-01
G1SCE1	Q9H4G4	GLIPR2	SCP domain-containing protein	Golgi-associated plant pathogenesis-related protein 1	2	97	4	0.824	0.069	4.7E-02
G1SXY5		STX2	Syntaxin 2		1		4	0.824	0.044	2.8E-02
G1T6C0	P61020	RAB5B	Uncharacterized protein	Ras-related protein Rab-5B	3	100	4	0.823	0.072	4.8E-02
G1SYK3	P45985	MAP2K4	Protein kinase domain-containing protein	Dual specificity mitogen-activated protein kinase kinase 4	2	99	3	0.823	0.030	5.1E-02
G1T6S9	P53367	ARFIP1	AH domain-containing protein	Arfaptin-1	2	98	4	0.822	0.049	3.0E-02
	H0Y507	SH3PXD2A		SH3 and PX domain-containing protein 2A (Fragment)	4		4	0.822	0.136	1.4E-01
G1TB45	Q86W92	PPFIBP1	Uncharacterized protein	Liprin-beta-1	3	87	4	0.822	0.032	2.3E-02
G1TM81	A0A0B4J2C3	TPT1	Translationally-controlled tumor protein	Translationally-controlled tumor protein	2	98	4	0.822	0.093	7.0E-02
G1U9U1	P50995	ANXA11	Annexin	Annexin A11	2	92	4	0.821	0.067	4.2E-02
G1SHK8	O94925	GLS	ANK_REP_REGION domain-containing protein	Glutaminase kidney isoform, mitochondrial	2	97	4	0.821	0.094	7.0E-02
G1SVY8	P12277	CKB	Creatine kinase B-type	Creatine kinase B-type	2	97	4	0.821	0.168	1.9E-01
G1SET0	P35606	COPB2	Coatomer subunit beta~	Coatomer subunit beta~	2	98	4	0.821	0.054	3.2E-02
G1SLF1		ADH5	S-(hydroxymethyl)glutathione dehydrogenase		1		4	0.820	0.049	2.8E-02
G1TEU5	O75131	CPNE3	Uncharacterized protein	Copine-3	3	96	4	0.819	0.036	2.3E-02
G1SVH8	Q13636	RAB31	Uncharacterized protein	Ras-related protein Rab-31	3	95	4	0.819	0.067	4.0E-02
G1T295		EPHX1	Epoxide hydrolase		1		4	0.819	0.039	2.3E-02
	P62854	RPS26		40S ribosomal protein S26	4		4	0.819	0.085	5.7E-02
G1TGK3	B4DP31	PRPSAP1	Pribosyltran_N domain-containing protein	Phosphoribosyl pyrophosphate synthase-associated protein 1	2	99	4	0.819	0.045	2.6E-02
G1T450		RO60	TROVE domain-containing protein		1		4	0.819	0.057	3.2E-02
G1SX32	O00743	PPP6C	Serine/threonine-protein phosphatase	Serine/threonine-protein phosphatase 6 catalytic subunit	2	100	4	0.818	0.107	8.2E-02
G1TB49		APOA1BP	NAD(P)H-hydrate epimerase		1		4	0.817	0.017	1.6E-02
G1SDQ5	Q96JG6	VPS50	Uncharacterized protein	Syndetin	3	98	4	0.816	0.062	3.4E-02
G1TKE3	X6RJP6	TAGLN2	Transgelin	Transgelin-2 (Fragment)	2	77	4	0.816	0.186	2.1E-01
	A0A0A0MT60	FKBP15		Peptidylprolyl isomerase (Fragment)	4		4	0.816	0.059	3.2E-02
G1SLL1	A0A087X0K9	TJP1	Uncharacterized protein	Tight junction protein ZO-1	3	82	4	0.815	0.100	7.0E-02
G1SN05	Q14240	EIF4A2	Uncharacterized protein	Eukaryotic initiation factor 4A-II	3	100	4	0.815	0.069	3.8E-02

G1TXW6		GPNMB	PKD domain-containing protein		1		4	0.814	0.156	1.5E-01
	P17612	PRKACA		cAMP-dependent protein kinase catalytic subunit alpha	4		4	0.813	0.023	1.6E-02
G1SJZ4	J3KQ32	OLA1	Obg-like ATPase 1	Obg-like ATPase 1	2	100	4	0.813	0.066	3.4E-02
	O14964	HGS		Hepatocyte growth factor-regulated tyrosine kinase substrate	4		4	0.813	0.103	7.0E-02
G1TBT4	Q9HB90	RRAGC	Uncharacterized protein	Ras-related GTP-binding protein C	3	98	4	0.812	0.075	4.0E-02
G1SQ23	P42025	ACTR1B	Uncharacterized protein	Beta-centractin	3	99	4	0.812	0.051	2.5E-02
U3KNB6	P48444	ARCN1	Coatomer subunit delta	Coatomer subunit delta	2	97	4	0.812	0.025	1.6E-02
G1T9D6	C9J5C3	PDCD10	Uncharacterized protein	Programmed cell death protein 10 (Fragment)	3	99	4	0.811	0.019	1.4E-02
G1T7B1	Q9H3P7	ACBD3	Uncharacterized protein	Golgi resident protein GCP60	3	96	4	0.811	0.046	2.2E-02
G1T4C9	Q8IVL6	P3H3	Prolyl 3-hydroxylase 3	Prolyl 3-hydroxylase 3	2	88	4	0.809	0.071	3.5E-02
G1STD5		SUCO	SUN domain containing ossification factor		1		4	0.809	0.224	2.6E-01
G1SL62	P07355	ANXA2	Annexin	Annexin A2	2	98	4	0.809	0.113	7.7E-02
G1SS33	P36543	ATP6V1E1	Uncharacterized protein	V-type proton ATPase subunit E 1	3	99	4	0.808	0.053	2.3E-02
G1SMP6	A0A1B0GVV3	RILPL1	Rab interacting lysosomal protein like 1	RILP-like protein 1	2	93	4	0.808	0.099	6.0E-02
G1T8X3		NEU1	Sialidase domain-containing protein		1		4	0.808	0.105	6.7E-02
G1SYV9	Q9Y490	TLN1	Uncharacterized protein	Talin-1	3	99	4	0.807	0.045	2.0E-02
G1SE51	A0A087X2D8	SPAG9	Sperm associated antigen 9	C-Jun-amino-terminal kinase-interacting protein 4	2	93	4	0.806	0.089	4.8E-02
G1SN14	Q86VP6	CAND1	TIP120 domain-containing protein	Cullin-associated NEDD8-dissociated protein 1	2	100	4	0.806	0.023	1.3E-02
G1SX42	Q9Y3P9	RABGAP1	Uncharacterized protein	Rab GTPase-activating protein 1	3	97	4	0.806	0.040	1.7E-02
Q28740		BSG	Basigin		1		4	0.804	0.116	7.6E-02
G1TUH9	Q14195	DPYSL3	Amidohydro-rel domain-containing protein	Dihydropyrimidinase-related protein 3	2	98	4	0.804	0.078	3.6E-02
Q28685	Q14118	DAG1	Dystroglycan	Dystroglycan	2	94	4	0.803	0.087	4.3E-02
G1T7I4	P53621	COPA	Coatomer subunit alpha	Coatomer subunit alpha	2	99	4	0.803	0.061	2.5E-02
G1U5B3	C9JIZ6	PSAP	Prosaposin	Prosaposin	2	78	4	0.803	0.170	1.5E-01
G1SD49	X6RCK5	DCTN3	Uncharacterized protein	Dynactin subunit 3 (Fragment)	3	82	4	0.802	0.119	7.6E-02
G1TD16	P48426	PIP4K2A	PIPK domain-containing protein	Phosphatidylinositol 5-phosphate 4-kinase type-2 alpha	2	100	4	0.802	0.058	2.3E-02
G1SN22	Q9Y5K6	CD2AP	Uncharacterized protein	CD2-associated protein	3	91	4	0.802	0.072	3.0E-02
G1SZK4	Q13442	PDAP1	PDGFA associated protein 1	28 kDa heat- and acid-stable phosphoprotein	2	96	4	0.801	0.058	2.3E-02
G1TI53	A0A0A0MRM8	MYO6	Uncharacterized protein	Unconventional myosin-VI	3	94	4	0.801	0.072	3.0E-02
G1TPZ1		LGALS1	Galectin		1		4	0.800	0.105	5.7E-02
	Q9UJY5	GGA1		ADP-ribosylation factor-binding protein GGA1	4		4	0.800	0.077	3.2E-02
G1TNY9		TRADD	Death domain-containing protein		1		4	0.799	0.038	1.4E-02
G1SQ80	Q8TDZ2	MICAL1	Uncharacterized protein	[F-actin]-monooxygenase MICAL1	3	83	4	0.798	0.063	2.3E-02
G1T7T6	O60763	USO1	Uncharacterized protein	General vesicular transport factor p115	3	95	4	0.798	0.051	1.8E-02
G1T8L2	P05997	COL5A2	Uncharacterized protein	Collagen alpha-2(V) chain	3	96	4	0.798	0.116	6.8E-02
G1SLQ4	B4DXW1	ACTR3	Uncharacterized protein	Actin-related protein 3	3	100	4	0.796	0.037	1.3E-02
G1SCW0	A8MT72	RTN1	Reticulon	Reticulon	2	98	4	0.795	0.062	2.1E-02
G1SID3	Q709C8	VPS13C	Vacuolar protein sorting 13 homolog C	Vacuolar protein sorting-associated protein 13C	2	88	4	0.795	0.044	1.4E-02
G1STJ8	G3V394	MYO5A	Uncharacterized protein	Unconventional myosin-Va	3	96	4	0.794	0.094	4.2E-02
G1SPB4		HYAL1	Hyaluronidase		1		4	0.794	0.134	8.4E-02
G1SMF4	Q9UHP3	USP25	USP domain-containing protein	Ubiquitin carboxyl-terminal hydrolase 25	2	94	4	0.793	0.054	1.7E-02
G1TS93	Q15435	PPP1R7	LRRcap domain-containing protein	Protein phosphatase 1 regulatory subunit 7	2	95	4	0.793	0.045	1.4E-02
G1SMS3	P61160	ACTR2	Actin-related protein 2	Actin-related protein 2	2	100	4	0.793	0.014	8.9E-03
G1T1X2	A0A087WZF1	LPP	Uncharacterized protein	Lipoma-preferred partner	3	89	4	0.792	0.079	2.9E-02
	Q8TBX8	PIP4K2C		Phosphatidylinositol 5-phosphate 4-kinase type-2 gamma	4		4	0.792	0.044	1.4E-02
G1T8D4	D6REX3	SEC31A	WD_REPEATS_REGION domain-containing protein	Protein transport protein Sec31A	2	91	4	0.792	0.054	1.7E-02
	A0A1B0GV23	CTSD		Cathepsin D	4		4	0.792	0.246	2.5E-01
G1SW44	H7C286	NAGK	BcrAD_BadFG domain-containing protein	N-acetyl-D-glucosamine kinase	2	97	4	0.790	0.065	2.0E-02
	O15143	ARPC1B		Actin-related protein 2/3 complex subunit 1B	4		4	0.790	0.063	1.9E-02
G1U9I8	P04264	KRT1	IF rod domain-containing protein	Keratin, type II cytoskeletal 1	2	86	4	0.789	0.083	3.0E-02
G1SNE8	P31949	S100A11	Protein S100	Protein S100-A11	2	87	4	0.788	0.154	1.0E-01
G1TBJ8		RB1CC1	RB1 inducible coiled-coil 1		1		4	0.788	0.170	1.3E-01
G1SHX1	P68036	UBE2L3	Ubiquitin conjugating enzyme E2 L3	Ubiquitin-conjugating enzyme E2 L3	2	96	4	0.788	0.045	1.2E-02
G1T5Z7	B3KR49	MAPK3	Mitogen-activated protein kinase	Mitogen-activated protein kinase 3	2	97	4	0.788	0.034	1.0E-02
G1SIT5	F2Z388	RPL35	Uncharacterized protein	60S ribosomal protein L35	3	93	4	0.787	0.160	1.1E-01
G1SR28	Q15102	PAFAH1B3	SGNH_hydro domain-containing protein	Platelet-activating factor acetylhydrolase IB subunit gamma	2	97	4	0.785	0.080	2.5E-02
G1TEW4	Q93008	USP9X	USP domain-containing protein	Probable ubiquitin carboxyl-terminal hydrolase FAF-X	2	99	4	0.785	0.023	8.2E-03
G1TWB9		TBC1D5	Rab-GAP TBC domain-containing protein		1		4	0.785	0.056	1.5E-02
G1TDH8		CLCN5	Chloride channel protein		1		4	0.784	0.083	2.7E-02
G1TMP7	A0A0D9SG72	STXBP1	Syntaxin binding protein 1	Syntaxin-binding protein 1	2	99	4	0.784	0.037	9.9E-03
G1T9Q5		SLC44A2	Solute carrier family 44 member 2		1		4	0.784	0.128	6.5E-02
P51662	P04083	ANXA1	Annexin A1	Annexin A1	2	91	4	0.782	0.055	1.4E-02
	Q63ZY3	KANK2		KN motif and ankyrin repeat domain-containing protein 2	4		4	0.780	0.079	2.2E-02
G1SNP9	P45954	ACADSB	Acyl-CoA dehydrogenase short/branched chain	Short/branched chain specific acyl-CoA dehydrogenase, mitochondrial	2	88	4	0.780	0.094	3.1E-02
G1SV03	Q14165	MLEC	Malectin domain-containing protein	Malectin	2	95	4	0.780	0.103	3.8E-02
G1SCP8	P26038	MSN	FERM domain-containing protein	Moesin	2	94	4	0.779	0.082	2.4E-02
Q09YN4	P47755	CAPZA2	F-actin-capping protein subunit alpha-2	F-actin-capping protein subunit alpha-2	2	98	4	0.779	0.046	1.0E-02
G1TAD1	Q7RTS9	DYM	Uncharacterized protein	Dymeclin	3	97	4	0.779	0.095	3.1E-02
	A0A2U3U034	ARSB		Arylsulfatase B	4		4	0.778	0.042	9.4E-03

G1TQR0	P12814-3	ACTN1	Uncharacterized protein	Isoform 3 of Alpha-actinin-1	3	97	4	0.778	0.064	1.5E-02
G1T156	Q9HB40	SCPEP1	Carboxypeptidase	Retinoid-inducible serine carboxypeptidase	2	86	4	0.778	0.172	1.1E-01
G1SCT1	A0A499FJL1	PREP	Uncharacterized protein	Prolyl endopeptidase	3	96	4	0.778	0.065	1.5E-02
G1TP81	Q9Y6W5	WASF2	WH2 domain-containing protein	Wiskott-Aldrich syndrome protein family member 2	2	94	4	0.777	0.062	1.4E-02
G1SE28	C9JNW5	RPL24	TRASH domain-containing protein	60S ribosomal protein L24	2	100	4	0.777	0.087	2.5E-02
G1SZ12	E7EPB3	RPL14	Ribosomal_L14e domain-containing protein	60S ribosomal protein L14	2	96	4	0.777	0.108	3.9E-02
	O14617	AP3D1		AP-3 complex subunit delta-1	4		4	0.776	0.030	7.3E-03
B7NZQ3		RA_m006_jsm824E	Deoxyribonuclease		1		4	0.776	0.083	2.2E-02
G1SM05	Q9NR31	SAR1A	Uncharacterized protein	GTP-binding protein SAR1a	3	99	4	0.775	0.064	1.4E-02
G1SWL6	Q9P0K7	RAI14	ANK_REP_REGION domain-containing protein	Ankycorbin	2	91	4	0.775	0.059	1.3E-02
G1TNL6	P50479	PDLIM4	Uncharacterized protein	PDZ and LIM domain protein 4	3	86	4	0.774	0.144	7.2E-02
G1TI02	Q9BRP8	PYM1	PYM homolog 1, exon junction complex associated factor	Partner of Y14 and mago	2	91	4	0.773	0.141	6.8E-02
	P56377	AP1S2		AP-1 complex subunit sigma-2	4		4	0.773	0.177	1.1E-01
G1SFZ8	Q9UMX0	UBQLN1	Ubiquilin 1	Ubiquilin-1	2	86	4	0.771	0.022	5.9E-03
G1TCY4	Q9Y5X3	SNX5	Sorting nexin	Sorting nexin-5	2	98	4	0.771	0.062	1.3E-02
G1ST64		LMOD1	Leiomodin 1		1		4	0.771	0.210	1.5E-01
G1TEM7	D6RA82	ANXA3	Annexin	Annexin	2	93	4	0.771	0.049	9.4E-03
G1SMU8	Q9NQP4	PFDN4	Prefoldin subunit 4	Prefoldin subunit 4	2	99	3	0.771	0.054	2.3E-02
G1TBW1		TXNDC17	DUF953 domain-containing protein		1		4	0.770	0.076	1.7E-02
G1SPR5	P16278	GLB1	Glyco_hydro_35 domain-containing protein	Beta-galactosidase	2	82	4	0.770	0.113	3.9E-02
G1SWD8	P42356	PI4KA	Uncharacterized protein	Phosphatidylinositol 4-kinase alpha	3	98	4	0.769	0.051	9.4E-03
G1T9V6	Q9UPU5	USP24	Ubiquitin specific peptidase 24	Ubiquitin carboxyl-terminal hydrolase 24	2	98	4	0.768	0.103	3.0E-02
	Q15149	PLEC		Plectin	4		4	0.768	0.084	2.0E-02
G1SZ63	P49189	ALDH9A1	Aldedh domain-containing protein	4-trimethylaminobutyraldehyde dehydrogenase	2	94	4	0.767	0.099	2.8E-02
G1SUU7	Q9Y3I0	RTCB	tRNA-splicing ligase RtcB homolog	tRNA-splicing ligase RtcB homolog	2	100	4	0.766	0.130	5.0E-02
G1SFX7	P62330	ARF6	Uncharacterized protein	ADP-ribosylation factor 6	3	100	4	0.766	0.105	3.0E-02
G1TZ31	Q9Y680	FKBP7	Peptidylprolyl isomerase	Peptidyl-prolyl cis-trans isomerase FKBP7	2	77	4	0.766	0.074	1.5E-02
G1T107	P61088	UBE2N	UBIQUITIN_CONJUGAT_2 domain-containing protein	Ubiquitin-conjugating enzyme E2 N	2	100	4	0.765	0.057	9.8E-03
G1T078	O43294	TGFB1I1	Uncharacterized protein	Transforming growth factor beta-1-induced transcript 1 protein	3	95	4	0.765	0.018	4.9E-03
G1TS42		AGL	Glycogen debranching enzyme		1		4	0.763	0.023	4.9E-03
G1SI22		AKAP12	A-kinase anchoring protein 12		1		4	0.763	0.287	2.5E-01
G1T652	P09972	ALDOC	Fructose-bisphosphate aldolase	Fructose-bisphosphate aldolase C	2	99	4	0.760	0.082	1.6E-02
G1SL68	P35579	MYH9	Uncharacterized protein	Myosin-9	3	96	4	0.760	0.028	5.0E-03
G1SKY8	Q96A57	TMEM230	Uncharacterized protein	Transmembrane protein 230	3	98	3	0.760	0.032	1.5E-02
G1SPN9	P36405	ARL3	Uncharacterized protein	ADP-ribosylation factor-like protein 3	3	99	4	0.759	0.064	1.0E-02
G1SQ22	Q9Y678	COPG1	Coatomer subunit gamma	Coatomer subunit gamma-1	2	99	4	0.759	0.065	1.0E-02
G1T2I6	P45877	PPIC	Peptidyl-prolyl cis-trans isomerase	Peptidyl-prolyl cis-trans isomerase C	2	92	4	0.759	0.114	3.2E-02
G1T5Q4	Q9H446	RWDD1	RWD domain-containing protein	RWD domain-containing protein 1	2	91	4	0.758	0.053	7.7E-03
G1SKV7	Q96KP4	CNDP2	M20_dimer domain-containing protein	Cytosolic non-specific dipeptidase	2	90	4	0.757	0.075	1.3E-02
G1SRI2	Q6P996	PDXDC1	Uncharacterized protein	Pyridoxal-dependent decarboxylase domain-containing protein 1	3	89	4	0.757	0.091	1.9E-02
G1U304		GALNS	Galactosamine (N-acetyl)-6-sulfatase		1		4	0.757	0.116	3.3E-02
G1SIT9	P31946	YWHAB	14_3_3 domain-containing protein	14-3-3 protein beta/alpha	2	100	4	0.757	0.044	6.2E-03
G1T967	A0A0U1RRB6	EXOC6B	Exocyst complex component	Exocyst complex component	2	99	4	0.757	0.068	1.1E-02
G1SGK1		GIGYF2	GRB10 interacting GYF protein 2		1		4	0.756	0.063	9.2E-03
G1SFP0	P28838	LAP3	CYTOSOL_AP domain-containing protein	Cytosol aminopeptidase	2	92	4	0.755	0.018	3.9E-03
G1SL85	A0A1B0GTM3	ASAH1	Uncharacterized protein	Acid ceramidase	3	81	4	0.755	0.196	1.1E-01
G1U949	Q15691	MAPRE1	Uncharacterized protein	Microtubule-associated protein RP/EB family member 1	3	99	4	0.755	0.043	5.9E-03
G1U446	Q14677	CLINT1	ENTH domain-containing protein	Clathrin interactor 1	2	97	4	0.754	0.047	6.2E-03
G1T2F8	H0YJH8	ATP6V1D	V-type proton ATPase subunit D	V-type proton ATPase subunit D (Fragment)	2	99	4	0.754	0.039	5.2E-03
Q9TTC6	P62937	PPIA	Peptidyl-prolyl cis-trans isomerase A	Peptidyl-prolyl cis-trans isomerase A	2	96	4	0.753	0.111	2.8E-02
G1SDT0	Q562R1	ACTBL2	Uncharacterized protein	Beta-actin-like protein 2	3	97	4	0.753	0.054	7.0E-03
Q9XS70	Q9BR76	CORO1B	Coronin-1B	Coronin-1B	2	93	4	0.753	0.050	6.4E-03
G1T6T5	E9PPQ5	CHORDC1	Uncharacterized protein	Cysteine and histidine-rich domain-containing protein 1	3	94	4	0.752	0.038	4.9E-03
G1SE61	O75369	FLNB	Filamin-B	Filamin-B	2	97	4	0.751	0.034	4.6E-03
G1SNY5	H0YK42	SNX1	PX domain-containing protein	Sorting nexin-1	2	96	4	0.751	0.029	4.2E-03
G1TBW9	Q96FJ2	DYNLL2	Dynein light chain	Dynein light chain 2, cytoplasmic	2	100	4	0.751	0.077	1.2E-02
G1TYW1		TPD52L2	TPD52 like 2		1		4	0.751	0.031	4.2E-03
G1SD27	B7ZC38	SH3GLB2	SH3 domain containing GRB2 like, endophilin B2	Endophilin-B2	2	92	4	0.750	0.046	5.7E-03
G1SHI0	H0Y4D4	ACAA1	Acetyl-CoA acyltransferase 1	3-ketoacyl-CoA thiolase, peroxisomal (Fragment)	2	62	4	0.750	0.057	7.3E-03
G1T7H6	Q6UX71	PLXDC2	PSI domain-containing protein	Plexin domain-containing protein 2	2	94	4	0.750	0.032	4.3E-03
G1T8H3	P51570	GALK1	Uncharacterized protein	Galactokinase	3	92	4	0.750	0.030	4.1E-03
G1U5A6	A0A286YFF8	MON2	Uncharacterized protein	Protein MON2 homolog	3	98	4	0.750	0.051	6.2E-03
	P35908	KRT2		Keratin, type II cytoskeletal 2 epidermal	4		4	0.750	0.159	6.4E-02
G1TZP0	P61981	YWHAG	14_3_3 domain-containing protein	14-3-3 protein gamma	2	99	4	0.749	0.030	4.1E-03
G1TA50	E7EQ61	UBA5	ThiF domain-containing protein	Ubiquitin-like modifier-activating enzyme 5	2	93	4	0.749	0.064	8.4E-03
G1SQ30		POMGNT2	Fibronectin type-III domain-containing protein		1		3	0.748	0.145	5.2E-02
G1SIB6	M0R192	BLVRB	Biliverdin reductase B	Flavin reductase (NADPH)	2	73	4	0.748	0.121	3.0E-02
G1SNE1	Q6DKJ4	NXN	Thioredoxin domain-containing protein	Nucleoredoxin	2	98	4	0.748	0.040	4.8E-03
G1TBY1		CTSB	Pept_C1 domain-containing protein		1		4	0.746	0.079	1.1E-02

G1SDY5	P63104	YWHAZ	14_3_3 domain-containing protein	14-3-3 protein zeta/delta	2	100	4	0.745	0.080	1.1E-02	
G1TD38	H3BVG8	VPS35L	Uncharacterized protein	VPS35 endosomal protein sorting factor-like	3	93	4	0.744	0.039	4.3E-03	
G1SQK1		SERPINB6	SERPIN domain-containing protein		1		4	0.744	0.144	4.5E-02	
G1SKD5	Q92783	STAM	Uncharacterized protein	Signal transducing adapter molecule 1	3	96	4	0.744	0.051	5.5E-03	
G1TYN0	Q9BVK6	TMED9	Transmembrane p24 trafficking protein 9	Transmembrane emp24 domain-containing protein 9	2	80	4	0.744	0.111	2.3E-02	
G1TIZ2	Q02809	PLOD1	Procollagen-lysine,2-oxoglutarate 5-dioxygenase 1	Procollagen-lysine,2-oxoglutarate 5-dioxygenase 1	2	93	4	0.743	0.094	1.5E-02	
G1TAK5		WNK4	WNK lysine deficient protein kinase 4		1		4	0.743	0.064	7.4E-03	
G1SQ02	A0A0A0MSI0	PRDX1	Thioredoxin domain-containing protein	Peroxiredoxin-1 (Fragment)	2	98	4	0.743	0.112	2.3E-02	
G1T7R2	P62258	YWHAE	14_3_3 domain-containing protein	14-3-3 protein epsilon	2	100	4	0.743	0.072	8.9E-03	
	P21333	FLNA		Filamin-A	4		4	0.742	0.077	1.0E-02	
G1SIC4	I3L294	ABHD12	Abhydrolase domain containing 12	Lysophosphatidylserine lipase ABHD12 (Fragment)	2	99	4	0.742	0.130	3.3E-02	
G1SPZ7		GPX1	Glutathione peroxidase		1		4	0.742	0.042	4.4E-03	
G1TJC3	Q9BUF5	TUBB6	Tubulin beta chain	Tubulin beta-6 chain	2	97	4	0.742	0.042	4.3E-03	
G1U2Q7		COL8A1	Collagen alpha-1(VIII) chain		1		4	0.741	0.083	1.1E-02	
G1TA40	H0YEP5	SMPD1	Sphingomyelin phosphodiesterase	Sphingomyelin phosphodiesterase (Fragment)	2	78	4	0.740	0.265	1.8E-01	
G1SZ91		FABP5	Lipocln_cytosolic_FA-bd_dom domain-containing protein		1		4	0.736	0.309	2.2E-01	
G1TMI5		RABAC1	PRA1 family protein		1		4	0.718	0.208	8.1E-02	
G1U4V6		CACNA2D1	Voltage-dependent calcium channel subunit alpha-2/delta-1		1		3	0.714	0.247	1.0E-01	
G1SSL3	P29373	CRABP2	FABP domain-containing protein	Cellular retinoic acid-binding protein 2	2	90	4	0.711	0.243	1.1E-01	
G1T7A8	Q9Y646	CPQ	Peptidase_M28 domain-containing protein	Carboxypeptidase Q	2	87	4	0.689	0.234	7.6E-02	
G1U1H1	Q00535	CDK5	Protein kinase domain-containing protein	Cyclin-dependent-like kinase 5	2	100	4	0.660	0.261	7.4E-02	
P15541		ANPEP	Aminopeptidase N		1		4	0.599	0.360	9.6E-02	
G1U826	O75348	ATP6V1G1	V-type proton ATPase subunit G	V-type proton ATPase subunit G 1	2	97	4	0.739	0.080	1.0E-02	X
G1SJ87	Q9H2D6	TRIOBP	TRIO and F-actin binding protein	TRIO and F-actin-binding protein	2	73	4	0.738	0.110	2.0E-02	X
A0A494BI13		NAAA	N-acylethanolamine-hydrolyzing acid amidase		1		4	0.738	0.153	4.8E-02	X
G1T8R1		RAP1GAP2	Platelet-activating factor acetylhydrolase IB subunit alpha		1		4	0.737	0.031	3.2E-03	X
G1TTJ1	Q6UWP2	DHRS11	Uncharacterized protein	Dehydrogenase/reductase SDR family member 11	3	92	4	0.737	0.085	1.1E-02	X
G1SR77	P23634	ATP2B4	Calcium-transporting ATPase	Plasma membrane calcium-transporting ATPase 4	2	87	4	0.734	0.080	9.0E-03	X
G1TEH2	P21283	ATP6V1C1	V-type proton ATPase subunit C	V-type proton ATPase subunit C 1	2	99	4	0.734	0.032	3.0E-03	X
G1TRX7	P23743	DGKA	Diacylglycerol kinase	Diacylglycerol kinase alpha	2	93	4	0.732	0.064	5.9E-03	X
G1SRL4		NAGA	Alpha-galactosidase		1		4	0.731	0.158	4.7E-02	X
G1TRM4	P62280	RPS11	Ribosomal_S17_N domain-containing protein	40S ribosomal protein S11	2	100	4	0.731	0.159	4.7E-02	X
G1TWQ3		SIRT2	NAD-dependent protein deacetylase		1		4	0.731	0.020	2.4E-03	X
G1SXQ0	A0A0A0MTN3	GSTM3	Glutathione S-transferase	Glutathione S-transferase	2	90	4	0.729	0.061	5.2E-03	X
G1SYX4	P50281	MMP14	Matrix metalloproteinase-14	Matrix metalloproteinase-14	2	95	4	0.729	0.135	3.0E-02	X
G1TM35	Q9Y3D6	FIS1	Fission, mitochondrial 1	Mitochondrial fission 1 protein	2	86	4	0.729	0.054	4.4E-03	X
G1T6Q9	O15400	STX7	t-SNARE coiled-coil homology domain-containing protein	Syntaxin-7	2	95	4	0.729	0.038	3.1E-03	X
G1SPM5	P61163	ACTR1A	Uncharacterized protein	Alpha-centractin	3	100	4	0.729	0.055	4.5E-03	X
G1SQ03	Q8WWI5	SLC44A1	Solute carrier family 44 member 1	Choline transporter-like protein 1	2	93	4	0.729	0.132	2.8E-02	X
U3KMD1	A0A0A0MRI2	SNX6	Vps5 domain-containing protein	Sorting nexin	2	99	4	0.727	0.037	2.9E-03	X
G1T5Y1	P28482	MAPK1	Mitogen-activated protein kinase	Mitogen-activated protein kinase 1	2	98	4	0.727	0.060	4.9E-03	X
G1T634	Q9Y5X1	SNX9	Sorting nexin	Sorting nexin-9	2	89	4	0.726	0.074	6.7E-03	X
G1SYM3		CD9	Tetraspanin		1		4	0.726	0.142	3.2E-02	X
G1TQJ4	Q01433	AMPD2	AMP deaminase	AMP deaminase 2	2	97	4	0.726	0.143	3.3E-02	X
G1TR42		RNPEP	Leuk-A4-hydro_C domain-containing protein		1		4	0.725	0.048	3.5E-03	X
G1SWK3	P49902	NT5C2	Uncharacterized protein	Cytosolic purine 5~-nucleotidase	3	100	4	0.723	0.104	1.3E-02	X
G1TVW5	Q96IJ6	GMPPA	NTP_transferase domain-containing protein	Mannose-1-phosphate guanyltransferase alpha	2	96	4	0.722	0.033	2.5E-03	X
G1SN09	Q9Y5P6	GMPPB	NTP_transferase domain-containing protein	Mannose-1-phosphate guanyltransferase beta	2	99	4	0.722	0.048	3.3E-03	X
G1TBZ5	E9PCX2	AKR1B1	Aldo-keto reductase family 1 member B1	Aldo-keto reductase family 1 member B1	2	85	4	0.721	0.120	1.9E-02	X
G1SCR0	H0YFI1	LAMTOR1	Uncharacterized protein	Ragulator complex protein LAMTOR1 (Fragment)	3	100	4	0.721	0.093	9.8E-03	X
	Q96BM9	ARL8A		ADP-ribosylation factor-like protein 8A	4		4	0.720	0.090	9.1E-03	X
G1SKL7	Q99536	VAT1	Vesicle amine transport 1	Synaptic vesicle membrane protein VAT-1 homolog	2	78	4	0.720	0.061	4.4E-03	X
	Q6WCQ1	MPRIP		Myosin phosphatase Rho-interacting protein	4		4	0.720	0.117	1.7E-02	X
G1SY70	F5H365	SEC23A	Protein transport protein SEC23	Protein transport protein SEC23	2	96	4	0.720	0.042	2.8E-03	X
G1SP34	O15144	ARPC2	Arp2/3 complex 34 kDa subunit	Actin-related protein 2/3 complex subunit 2	2	100	4	0.719	0.022	2.0E-03	X
G1SZ18	A0A0C4DFT3	DLG1	Uncharacterized protein	Disks large homolog 1	3	93	4	0.718	0.106	1.3E-02	X
G1SHS7	O75083	WDR1	WD_REPEATS_REGION domain-containing protein	WD repeat-containing protein 1	2	94	4	0.718	0.053	3.4E-03	X
G1U0I7	H3BSK9	ATXN2L	Ataxin 2 like	Ataxin-2-like protein (Fragment)	2	99	4	0.716	0.102	1.1E-02	X
G1SXU7	F8WA11	CLASP1	Cytoplasmic linker associated protein 1	CLIP-associating protein 1	2	93	3	0.716	0.019	6.2E-03	X
G1SIL8	A0A087WWM4	GMPR2	GMP reductase	GMP reductase	2	90	4	0.715	0.088	7.6E-03	X
G1TPH5	Q9Y6D5	ARFGEF2	ADP ribosylation factor guanine nucleotide exchange factor 2	Brefeldin A-inhibited guanine nucleotide-exchange protein 2	2	96	4	0.715	0.057	3.5E-03	X
G1T6D1	P62829	RPL23	Uncharacterized protein	60S ribosomal protein L23	3	100	4	0.712	0.088	7.4E-03	X
G1SER8	P07737	PFN1	Profilin	Profilin-1	2	94	4	0.710	0.035	2.1E-03	X
G1SY85	Q9Y2T2	AP3M1	MHD domain-containing protein	AP-3 complex subunit mu-1	2	99	4	0.710	0.010	1.5E-03	X
G1TCD4	E5RGF9	FAM114A2	Uncharacterized protein	Protein FAM114A2 (Fragment)	3	80	4	0.707	0.056	3.0E-03	X
G1SMX4	A0A0G2JH68	DIAPH1	Uncharacterized protein	Protein diaphanous homolog 1	3	90	4	0.706	0.097	8.4E-03	X
	O43707	ACTN4		Alpha-actinin-4	4		4	0.706	0.042	2.2E-03	X
	A0A087WY85	UBE2D3		Ubiquitin-conjugating enzyme E2 D3	4		4	0.705	0.077	4.9E-03	X

	Q16181	SEPTIN7		Septin-7	4		4	0.705	0.008	1.4E-03	X
G1SWW6	F5H459	AP3S1	Clat_adaptor_s domain-containing protein	AP complex subunit sigma	2	95	4	0.704	0.041	2.1E-03	X
G1TCW5	A0A494C165	PEPD	Peptidase D	Xaa-Pro dipeptidase (Fragment)	2	79	4	0.703	0.087	6.1E-03	X
G1SSP0		STRN	WD_REPEATS_REGION domain-containing protein		1		4	0.703	0.063	3.2E-03	X
G1TCM9	Q9H299	SH3BGRL3	SH3 domain-binding glutamic acid-rich-like protein	SH3 domain-binding glutamic acid-rich-like protein 3	2	100	3	0.702	0.174	3.9E-02	X
G1T1V9	P54652	HSPA2	Uncharacterized protein	Heat shock-related 70 kDa protein 2	3	98	4	0.702	0.095	7.3E-03	X
G1SDF2	A0A0A0MRJ6	PCMT1	Protein-L-isoaspartate O-methyltransferase	Protein-L-isoaspartate O-methyltransferase	2	96	4	0.702	0.021	1.4E-03	X
G1SNP8	C9JJP5	TFG	PB1 domain-containing protein	Protein TFG (Fragment)	2	97	4	0.701	0.024	1.5E-03	X
G1SI13		SDC2	Syndecan		1		4	0.700	0.048	2.2E-03	X
G1T6S4	F8W9J4	DST	Dystonin	Dystonin	2	62	4	0.700	0.112	1.1E-02	X
G1SCI0	Q14204	DYNC1H1	Dynein cytoplasmic 1 heavy chain 1	Cytoplasmic dynein 1 heavy chain 1	2	98	4	0.699	0.052	2.4E-03	X
	H0Y5B4	RPL36A		60S ribosomal protein L36a	4		4	0.699	0.171	3.6E-02	X
G1T3R5		ERO1A	Endoplasmic reticulum oxidoreductase 1 alpha		1		4	0.699	0.151	2.5E-02	X
G1SNC4	Q5T9B7	AK1	Adenylate kinase isoenzyme 1	Adenylate kinase isoenzyme 1	2	94	4	0.698	0.131	1.6E-02	X
G1TUC2	P62633	CNBP	Uncharacterized protein	Cellular nucleic acid-binding protein	3	100	3	0.698	0.066	7.3E-03	X
G1U670	A0A087WW40	SH3GLB1	Uncharacterized protein	Endophilin-B1	3	95	4	0.697	0.063	3.0E-03	X
G1SMY7	C9JIF9	APEH	Acylamino-acid-releasing enzyme	Acylamino-acid-releasing enzyme	2	93	4	0.696	0.038	1.7E-03	X
G1SN11	Q01082	SPTBN1	Spectrin beta chain	Spectrin beta chain, non-erythrocytic 1	2	99	4	0.696	0.070	3.4E-03	X
G1SL41		GUSB	Beta-glucuronidase		1		4	0.694	0.136	1.7E-02	X
G1T0Y9	Q13561	DCTN2	Uncharacterized protein	Dynactin subunit 2	3	97	4	0.693	0.066	2.9E-03	X
G1TEG8	Q8WUM4	PDCD6IP	BRO1 domain-containing protein	Programmed cell death 6-interacting protein	2	94	4	0.693	0.049	2.0E-03	X
G1T1G5	Q9H4A6	GOLPH3	Uncharacterized protein	Golgi phosphoprotein 3	3	99	4	0.693	0.038	1.6E-03	X
G1TGA8	O75396	SEC22B	Uncharacterized protein	Vesicle-trafficking protein SEC22b	3	98	4	0.692	0.069	3.1E-03	X
G1SN76	O94804	STK10	Serine/threonine kinase 10	Serine/threonine-protein kinase 10	2	90	4	0.692	0.105	8.0E-03	X
G1SD48	A0A0G2JLB3	GBA	Glucosylceramidase	Glucosylceramidase	2	90	4	0.690	0.094	5.8E-03	X
G1SHN4	Q96JB2	COG3	Uncharacterized protein	Conserved oligomeric Golgi complex subunit 3	3	96	4	0.690	0.125	1.2E-02	X
G1TAM3		TBCB	CAP-Gly domain-containing protein		1		4	0.689	0.070	3.1E-03	X
G1SY00	P42858	HTT	Uncharacterized protein	Huntingtin	3	91	4	0.689	0.072	3.2E-03	X
G1SWR0	H3BP20	HEXA	Beta-hexosaminidase	Beta-hexosaminidase	2	85	4	0.689	0.087	4.8E-03	X
G1T5W7	Q14376	GALE	NAD(P)-bd_dom domain-containing protein	UDP-glucose 4-epimerase	2	95	4	0.688	0.099	6.2E-03	X
G1SR27	P62993	GRB2	Uncharacterized protein	Growth factor receptor-bound protein 2	3	100	4	0.687	0.081	4.0E-03	X
G1T060	A0A2R8YD50	HSD17B4	Uncharacterized protein	Peroxisomal multifunctional enzyme type 2	3	89	4	0.685	0.099	6.1E-03	X
G1T281		SNTA1	Alpha-1-syntrophin		1		4	0.684	0.072	2.9E-03	X
G1SW40	E7EMB1	SWAP70	PH domain-containing protein	Switch-associated protein 70	2	77	4	0.683	0.121	9.9E-03	X
G1T7Z0	P52209	PGD	6-phosphogluconate dehydrogenase, decarboxylating	6-phosphogluconate dehydrogenase, decarboxylating	2	93	4	0.683	0.023	1.1E-03	X
G1U7S8	F8W6C2	SPATS2L	Uncharacterized protein	SPATS2-like protein (Fragment)	3	100	4	0.682	0.038	1.4E-03	X
G1SFH5	O00469-2	PLOD2	Fe2OG dioxygenase domain-containing protein		2	90	4	0.682	0.107	7.0E-03	X
G1T545		BTBD9	Lactoylglutathione lyase		1		4	0.679	0.091	4.5E-03	X
G1SUK5	O43583	DENR	Density-regulated protein	Density-regulated protein	2	97	4	0.679	0.031	1.1E-03	X
O77708	D6R938	CAMK2D	Calcium/calmodulin-dependent protein kinase type II subunit delta	Calcium/calmodulin-dependent protein kinase (CaM kinase) II delta, isoform CRA e	2	92	4	0.679	0.051	1.6E-03	X
G1TER4	P33897	ABCD1	Uncharacterized protein	ATP-binding cassette sub-family D member 1	3	94	4	0.678	0.177	3.0E-02	X
G1SMT2		FAM129B	Family with sequence similarity 129 member B		1		4	0.678	0.077	3.0E-03	X
G1SVI9	Q9P0L0	VAPA	MSP domain-containing protein	Vesicle-associated membrane protein-associated protein A	2	84	4	0.677	0.070	2.5E-03	X
Q09YN6	C9JKI3	CAV1	Caveolin-1	Caveolin (Fragment)	2	96	4	0.676	0.082	3.3E-03	X
G1SNH7	P09417	QDPR	Quinoid dihydropteridine reductase	Dihydropteridine reductase	2	92	4	0.676	0.046	1.4E-03	X
G1T3U5	Q9UDY4	DNAJB4	J domain-containing protein	DnaJ homolog subfamily B member 4	2	96	4	0.675	0.170	2.5E-02	X
G1T432	Q01518	CAP1	Adenylyl cyclase-associated protein	Adenylyl cyclase-associated protein 1	2	96	4	0.674	0.050	1.5E-03	X
G1TMW2		TOLLIP	Toll interacting protein		1		4	0.674	0.025	9.6E-04	X
	A0A2R8Y3S6	SNX27		Sorting nexin-27 (Fragment)	4		4	0.674	0.077	2.8E-03	X
G1T7D9		FNTB	Protein farnesyltransferase subunit beta		1		4	0.673	0.057	1.7E-03	X
G1TAB2		GM2A	ML domain-containing protein		1		4	0.673	0.011	8.0E-04	X
G1T188	Q9UIJ7	AK3	GTP:AMP phosphotransferase AK3, mitochondrial	GTP:AMP phosphotransferase AK3, mitochondrial	2	93	4	0.672	0.146	1.5E-02	X
G1SH88	Q8N8S7	ENAH	ENAH, actin regulator	Protein enabled homolog	2	99	4	0.672	0.039	1.2E-03	X
G1T512		CNP	2~,3~-cyclic nucleotide 3~ phosphodiesterase		1		4	0.671	0.116	7.4E-03	X
G1U9R8	P06396-2	GSN	Uncharacterized protein	Isoform 2 of Gelsolin	3	95	4	0.671	0.047	1.3E-03	X
G1TPC5		TPD52	Tumor protein D52		1		4	0.671	0.083	3.1E-03	X
G1T8R3	P46939	UTRN	Uncharacterized protein	Utrophin	3	91	4	0.670	0.018	8.1E-04	X
G1U0Q7		SEPT2	Septin-type G domain-containing protein		1		4	0.670	0.049	1.4E-03	X
G1TC70	O14974	PPP1R12A	Protein phosphatase 1 regulatory subunit	Protein phosphatase 1 regulatory subunit 12A	2	94	4	0.669	0.049	1.4E-03	X
P21195	P07237	P4HB	Protein disulfide-isomerase	Protein disulfide-isomerase	2	91	4	0.669	0.157	1.8E-02	X
G1U0Y0	A0A2Q3DQE3	CAMK2G	Protein kinase domain-containing protein	Calcium/calmodulin-dependent protein kinase (CaM kinase) II gamma, isoform CRA d	2	91	4	0.668	0.083	3.0E-03	X
G1SZP0	O60784	TOM1	Uncharacterized protein	Target of Myb protein 1	3	88	4	0.667	0.061	1.7E-03	X
G1SR49		SEPT10	Septin-type G domain-containing protein		1		4	0.665	0.056	1.5E-03	X
G1SY84	Q13409-2	DYNC1I2	WD_REPEATS_REGION domain-containing protein	Isoform 2B of Cytoplasmic dynein 1 intermediate chain 2	2	92	4	0.663	0.053	1.4E-03	X
G1SE87		ARSE	Arylsulfatase E		1		4	0.663	0.108	5.2E-03	X
P48738	Q00169	PITPNA	Phosphatidylinositol transfer protein alpha isoform	Phosphatidylinositol transfer protein alpha isoform	2	99	4	0.663	0.083	2.8E-03	X
G1TME7		CSTB	Cystatin B		1		4	0.663	0.077	2.4E-03	X
	K7EPT8	GFAP		Glial fibrillary acidic protein (Fragment)	4		4	0.662	0.170	2.0E-02	X
	Q08209-2	PPP3CA		Isoform 2 of Serine/threonine-protein phosphatase 2B catalytic subunit alpha isoform	4		4	0.662	0.082	2.7E-03	X

G1T9X4	O95980	RECK	Reversion inducing cysteine rich protein with kazal motifs	Reversion-inducing cysteine-rich protein with Kazal motifs	2	95	4	0.660	0.050	1.2E-03	X
B7NZQ6	P31150	GDI1	Rab GDP dissociation inhibitor	Rab GDP dissociation inhibitor alpha	2	99	4	0.660	0.026	7.5E-04	X
G1SW57	P21589	NT5E	Uncharacterized protein	5~-nucleotidase	3	89	4	0.659	0.148	1.2E-02	X
G1SIT6	Q9BPX5	ARPC5L	Actin-related protein 2/3 complex subunit 5	Actin-related protein 2/3 complex subunit 5-like protein	2	98	4	0.659	0.017	6.6E-04	X
	Q7Z406	MYH14		Myosin-14	4		4	0.658	0.025	7.3E-04	X
G1THW3	A0A3B3IUC0	ITM2B	BRICHOS domain-containing protein	Integral membrane protein 2B	2	96	4	0.658	0.083	2.6E-03	X
G1TBS1	Q99497	PARK7	DJ-1_PfpI domain-containing protein	Protein/nucleic acid deglycase DJ-1	2	96	4	0.656	0.103	4.1E-03	X
G1TLL6	Q9UNH6	SNX7	PX domain-containing protein	Sorting nexin-7	2	96	4	0.655	0.094	3.2E-03	X
G1TVQ3		DYNLRB1	Dynein light chain roadblock		1		4	0.654	0.034	7.8E-04	X
G1T1V0	P13645	KRT10	IF rod domain-containing protein	Keratin, type I cytoskeletal 10	2	95	4	0.653	0.189	2.5E-02	X
G1U2E3	Q9H444	CHMP4B	Uncharacterized protein	Charged multivesicular body protein 4b	3	100	4	0.652	0.054	1.2E-03	X
	A0A3B3ISV4	VKORC1L1		Vitamin K epoxide reductase complex subunit 1-like protein 1	4		4	0.652	0.114	5.1E-03	X
G1T3I9	P20073	ANXA7	Annexin	Annexin A7	2	93	4	0.650	0.038	7.9E-04	X
G1TU12	Q5H907	MAGED2	MAGE domain-containing protein	Melanoma antigen family D, 2, isoform CRA_d	2	79	4	0.649	0.057	1.2E-03	X
	H0Y7A7	CALM2		Calmodulin-2 (Fragment)	4		4	0.648	0.137	8.2E-03	X
G1TX63		CDC42BPA	Non-specific serine/threonine protein kinase		1		4	0.646	0.031	6.6E-04	X
G1SLX0	G3V4P8	GMFB	Glia maturation factor	Glia maturation factor beta (Fragment)	2	99	4	0.646	0.050	9.6E-04	X
P00883	P04075	ALDOA	Fructose-bisphosphate aldolase A	Fructose-bisphosphate aldolase A	2	98	4	0.646	0.132	7.1E-03	X
G1TAC4		GLRX	Glutaredoxin-1		1		4	0.645	0.075	1.7E-03	X
G1SZH6	Q9BZF1	OSBPL8	Oxysterol-binding protein	Oxysterol-binding protein-related protein 8	2	99	4	0.643	0.175	1.7E-02	X
G1T345		UBR1	E3 ubiquitin-protein ligase		1		4	0.642	0.068	1.4E-03	X
G1SXE6	Q9Y6G9	DYNC1LI1	Uncharacterized protein	Cytoplasmic dynein 1 light intermediate chain 1	3	94	4	0.641	0.033	6.3E-04	X
G1ST05	Q15120	PDK3	Protein-serine/threonine kinase	[Pyruvate dehydrogenase (acetyl-transferring)] kinase isozyme 3, mitochondrial	2	99	4	0.640	0.133	6.6E-03	X
G1SCP7	P46940	IQGAP1	Uncharacterized protein	Ras GTPase-activating-like protein IQGAP1	3	97	4	0.640	0.047	8.0E-04	X
G1SDZ0		CTSA	Carboxypeptidase		1		4	0.639	0.100	2.9E-03	X
G1SE41	Q9NWM8	FKBP14	Peptidylprolyl isomerase	Peptidyl-prolyl cis-trans isomerase FKBP14	2	94	4	0.638	0.071	1.4E-03	X
G1SJ56	P18206	VCL	Uncharacterized protein	Vinculin	3	99	4	0.636	0.068	1.3E-03	X
G1SE57	P18085	ARF4	Uncharacterized protein	ADP-ribosylation factor 4	3	95	4	0.635	0.079	1.6E-03	X
G1SP54		LTA4H	Leukotriene A(4) hydrolase		1		4	0.634	0.125	5.0E-03	X
G1SM64	Q5T985	ITIH2	Uncharacterized protein	Inter-alpha-trypsin inhibitor heavy chain H2	3	85	4	0.633	0.171	1.3E-02	X
G1TV76	Q9Y4D1	DAAM1	Uncharacterized protein	Disheveled-associated activator of morphogenesis 1	3	97	4	0.632	0.086	1.8E-03	X
G1T346	Q13813-3	SPTAN1	Uncharacterized protein	Isoform 3 of Spectrin alpha chain, non-erythrocytic 1	3	97	4	0.631	0.108	3.1E-03	X
P53787		EEF1D	Elongation factor 1-delta		1		4	0.629	0.046	6.6E-04	X
G1U723		PGER5	3alpha/17beta/20alpha-hydroxysteroid dehydrogenase		1		4	0.629	0.122	4.3E-03	X
G1SXK6	Q9NR12	PDLIM7	PDZ and LIM domain 7	PDZ and LIM domain protein 7	2	92	4	0.628	0.052	7.5E-04	X
G1SPL3	G3V583	FAM177A1	Uncharacterized protein	Protein FAM177A1 (Fragment)	3	93	4	0.628	0.142	6.7E-03	X
G1SEX8	Q9P299	COPZ2	Clat_adaptor_s domain-containing protein	Coatomer subunit zeta-2	2	84	4	0.626	0.035	5.1E-04	X
G1T3J0		SLC26A7	Anion exchange transporter		1		4	0.626	0.132	5.2E-03	X
G1U974	A0A1C7CYX9	DPYSL2	Amidohydro-rel domain-containing protein	Dihydropyrimidinase-related protein 2	2	98	4	0.625	0.017	4.0E-04	X
G1T004	H0Y987	PGM3	Phosphoacetylglucosamine mutase	Phosphoacetylglucosamine mutase	2	90	4	0.625	0.069	1.1E-03	X
G1SDL3	P54802	NAGLU	Uncharacterized protein	Alpha-N-acetylglucosaminidase	3	88	4	0.625	0.114	3.3E-03	X
	Q9UHD8	SEPTIN9		Septin-9	4		4	0.625	0.078	1.4E-03	X
G1TS18		BSDC1	BSD domain containing 1		1		3	0.621	0.072	2.5E-03	X
	Q6NZI2	CAVIN1		Caveolae-associated protein 1	4		4	0.620	0.073	1.1E-03	X
B7NZG7	O60493	SNX3	Sorting nexin 3 (Predicted)	Sorting nexin-3	2	100	3	0.619	0.012	1.4E-03	X
G1TER3	A0A2R8Y5M6	BCAP31	Uncharacterized protein	B-cell receptor-associated protein 31	3	81	4	0.619	0.116	3.2E-03	X
G1T277	F8VR50	ARPC3	Actin-related protein 2/3 complex subunit 3	Actin-related protein 2/3 complex subunit 3 (Fragment)	2	100	4	0.619	0.052	6.4E-04	X
G1SZD6	P27348	YWHAQ	14-3-3 protein theta	14-3-3 protein theta	2	99	4	0.618	0.101	2.2E-03	X
G1T5E6	O60749	SNX2	PX domain-containing protein	Sorting nexin-2	2	98	4	0.617	0.035	4.5E-04	X
G1TMM0	A0A087WW43	ITIH3	Inter-alpha-trypsin inhibitor heavy chain H3	Inter-alpha-trypsin inhibitor heavy chain H3	2	91	4	0.616	0.119	3.2E-03	X
G1T4N8	Q8TF42	UBASH3B	Uncharacterized protein	Ubiquitin-associated and SH3 domain-containing protein B	3	98	4	0.616	0.226	2.8E-02	X
G1T7T8	P47712	PLA2G4A	Phospholipase A2	Cytosolic phospholipase A2	2	94	4	0.616	0.033	4.2E-04	X
G1T4P7	Q9Y4G6	TLN2	Talin 2	Talin-2	2	98	4	0.615	0.112	2.7E-03	X
G1TFI4	Q96K17	BTF3L4	Transcription factor BTF3	Transcription factor BTF3 homolog 4	2	100	4	0.611	0.094	1.6E-03	X
G1SN21	P00491	PNP	Purine nucleoside phosphorylase	Purine nucleoside phosphorylase	2	90	4	0.611	0.013	3.4E-04	X
G1TK63	P22413	ENPP1	Uncharacterized protein	Ectonucleotide pyrophosphatase/phosphodiesterase family member 1	3	89	4	0.609	0.076	1.0E-03	X
G1SMK8	O95816	BAG2	BAG domain-containing protein	BAG family molecular chaperone regulator 2	2	98	4	0.609	0.099	1.7E-03	X
P58776	A7XZE4	TPM2	Tropomyosin beta chain	Beta tropomyosin isoform	2	94	4	0.606	0.194	1.4E-02	X
G1SEF9	P17661	DES	IF rod domain-containing protein	Desmin	2	99	3	0.606	0.280	4.1E-02	X
G1U8T9	Q96CX2	KCTD12	Potassium channel tetramerization domain containing 12	BTB/POZ domain-containing protein KCTD12	2	97	4	0.606	0.045	4.5E-04	X
G1TN33		SEPT8	Septin-type G domain-containing protein		1		4	0.605	0.034	3.7E-04	X
G1T3V2	P04792	HSPB1	SHSP domain-containing protein	Heat shock protein beta-1	2	91	4	0.604	0.161	7.2E-03	X
G1SGL0	Q92629	SGCD	Uncharacterized protein	Delta-sarcoglycan	3	98	4	0.603	0.053	5.3E-04	X
G1TP25	Q5VU77	UBAP2L	Ubiquitin associated protein 2 like	Ubiquitin-associated protein 2-like (Fragment)	2	95	4	0.603	0.160	7.0E-03	X
G1U7Y3	E7END4	LOXL3	Uncharacterized protein	Lysyl oxidase homolog 3	3	87	4	0.602	0.109	2.1E-03	X
G1SX37	Q9Y281	CFL2	Cofilin 2	Cofilin-2	2	91	4	0.602	0.059	6.0E-04	X
G1SEA7	Q13131	PRKAA1	Non-specific serine/threonine protein kinase	5~-AMP-activated protein kinase catalytic subunit alpha-1	2	97	3	0.598	0.050	1.4E-03	X
G1TKH3		SOD1	Superoxide dismutase [Cu-Zn]		1		4	0.597	0.119	2.5E-03	X
G1U411	F8WBG8	DBNL	Uncharacterized protein	Drebrin-like protein	3	95	4	0.597	0.044	4.0E-04	X

G1T3D7		NANS	AFP-like domain-containing protein		1		4	0.597	0.060	5.6E-04	X
G1T9N3	B5MBZ0	EML4	Uncharacterized protein	Echinoderm microtubule-associated protein-like 4	3	92	4	0.596	0.105	1.7E-03	X
G1SHR7	Q9P0J7	KCMF1	C2H2-type domain-containing protein	E3 ubiquitin-protein ligase KCMF1	2	99	4	0.594	0.042	3.7E-04	X
G1STY4		PLBD2	Phospholipase B-like		1		4	0.593	0.179	9.0E-03	X
G1SKT2	E9PGF6	PHLDB2	PH domain-containing protein	Pleckstrin homology-like domain family B member 2	2	65	4	0.593	0.095	1.3E-03	X
G1U5M4	J3QQS9	SLC16A3	Solute carrier family 16 member 3	Monocarboxylate transporter 4 (Fragment)	2	92	4	0.593	0.259	3.2E-02	X
G1SLH7	P42566	EPS15	Uncharacterized protein	Epidermal growth factor receptor substrate 15	3	93	4	0.592	0.068	6.4E-04	X
G1SUV2		CAMLG	Calcium signal-modulating cyclophilin ligand		1		4	0.591	0.081	8.7E-04	X
G1SI83	Q99584	S100A13	S_100 domain-containing protein	Protein S100-A13	2	91	4	0.591	0.058	4.9E-04	X
G1SNT1	O75822	EIF3J	Eukaryotic translation initiation factor 3 subunit J	Eukaryotic translation initiation factor 3 subunit J	2	92	4	0.584	0.043	3.5E-04	X
G1T0J3	E9PDF6	MYO1B	Uncharacterized protein	Unconventional myosin-Ib	3	96	4	0.577	0.094	1.0E-03	X
G1SF47		SEPT11	Septin-type G domain-containing protein		1		4	0.577	0.042	3.3E-04	X
G1T823	H3BT58	COTL1	Coactosin like F-actin binding protein 1	Coactosin-like protein	2	96	4	0.576	0.057	3.9E-04	X
Q6SQH4	P60903	S100a10	Protein S100-A10	Protein S100-A10	2	100	4	0.576	0.138	2.9E-03	X
G1SX70	Q9NRW1	RAB6B	Uncharacterized protein	Ras-related protein Rab-6B	3	100	4	0.575	0.171	5.9E-03	X
G1U5Q7		ARPC4	Actin-related protein 2/3 complex subunit 4		1		4	0.573	0.060	4.0E-04	X
G1SQF2	O43237	DYNC1LI2	Uncharacterized protein	Cytoplasmic dynein 1 light intermediate chain 2	3	98	4	0.572	0.089	8.1E-04	X
G1SZX4	Q14571	ITPR2	Uncharacterized protein	Inositol 1,4,5-trisphosphate receptor type 2	3	97	4	0.572	0.119	1.7E-03	X
G1SVB5	J3QRN6	MYO1D	Uncharacterized protein	Unconventional myosin-Id	3	95	4	0.571	0.055	3.7E-04	X
	P32119	PRDX2		Peroxiredoxin-2	4		4	0.571	0.078	6.1E-04	X
	P06753-5	TPM3		Isoform 5 of Tropomyosin alpha-3 chain	4		4	0.570	0.085	7.2E-04	X
G1TYA7	P07195	LDHB	L-lactate dehydrogenase	L-lactate dehydrogenase B chain	2	100	4	0.569	0.106	1.2E-03	X
G1U9R0	A8MX94	GSTP1	Uncharacterized protein	Glutathione S-transferase P	3	71	4	0.568	0.048	3.3E-04	X
Q6TYA7		GJA1	Gap junction alpha-1 protein		1		4	0.565	0.040	2.8E-04	X
G1SG54	A0A0U1RQT1	ACAP2	Arf-GAP with coiled-coil, ANK repeat and PH domain-containing protein 2	Arf-GAP with coiled-coil, ANK repeat and PH domain-containing protein 2 (Fragment)	2	99	4	0.564	0.056	3.5E-04	X
G1SG55	O43491	EPB41L2	FERM domain-containing protein	Band 4.1-like protein 2	2	90	4	0.564	0.060	3.7E-04	X
G1TV31	O15511	ARPC5	Actin-related protein 2/3 complex subunit 5	Actin-related protein 2/3 complex subunit 5	2	95	4	0.564	0.060	3.7E-04	X
G1SWW4		SYNPO	Synaptopodin		1		4	0.563	0.110	1.3E-03	X
G1SXR6	O60282	KIF5C	Kinesin-like protein	Kinesin heavy chain isoform 5C	2	99	4	0.563	0.043	2.8E-04	X
G1SDH3	E9PIG4	PRCP	Uncharacterized protein	Lysosomal Pro-X carboxypeptidase (Fragment)	3	89	4	0.563	0.203	9.1E-03	X
G1T8C8	A0A087X0R6	SNX12	Sorting nexin 12	Sorting nexin-12	2	100	4	0.561	0.098	9.0E-04	X
G1U6H4	O95865	DDAH2	Uncharacterized protein	N(G),N(G)-dimethylarginine dimethylaminohydrolase 2	3	97	4	0.559	0.121	1.5E-03	X
	P22392-2	NME2		Isoform 3 of Nucleoside diphosphate kinase B	4		4	0.558	0.103	9.7E-04	X
	Q9NP61	ARFGAP3		ADP-ribosylation factor GTPase-activating protein 3	4		4	0.558	0.050	3.1E-04	X
P11974-2		PKM	Isoform M2 of Pyruvate kinase PKM		1		4	0.556	0.182	5.6E-03	X
G1SD83		ITGA6	Integrin_alpha2 domain-containing protein		1		4	0.556	0.187	6.2E-03	X
G1SDP2	Q96AY3	FKBP10	FK506-binding protein	Peptidyl-prolyl cis-trans isomerase FKBP10	2	93	4	0.555	0.094	7.3E-04	X
	P68371	TUBB4B		Tubulin beta-4B chain	4		4	0.555	0.062	3.6E-04	X
G1SPF5	P18669	PGAM1	Uncharacterized protein	Phosphoglycerate mutase 1	3	100	4	0.554	0.087	6.1E-04	X
G1SSW2	B4E0Y9	STK26	Protein kinase domain-containing protein	Serine/threonine-protein kinase 26	2	95	4	0.554	0.142	2.3E-03	X
G1T473	F8VQE1	LIMA1	LIM zinc-binding domain-containing protein	LIM domain and actin-binding protein 1	2	85	4	0.552	0.059	3.3E-04	X
G1SN43	F8W930	IGF2BP2	Uncharacterized protein	Insulin-like growth factor 2 mRNA-binding protein 2	3	98	4	0.548	0.057	3.1E-04	X
G1SPD1		MTPN	ANK_REP_REGION domain-containing protein		1		4	0.547	0.057	3.1E-04	X
G1T5P0	Q8NDI1	EHBP1	Uncharacterized protein	EH domain-binding protein 1	3	94	4	0.546	0.169	3.8E-03	X
G1SZ47	P62266	RPS23	Uncharacterized protein	40S ribosomal protein S23	3	100	4	0.543	0.126	1.4E-03	X
G1SP27	Q16204	CCDC6	Uncharacterized protein	Coiled-coil domain-containing protein 6	3	93	4	0.542	0.041	2.4E-04	X
G1SQ10	Q9NQT8	KIF13B	Uncharacterized protein	Kinesin-like protein KIF13B	3	89	4	0.539	0.066	3.3E-04	X
G1SYL5	Q7Z4N8	P4HA3	Fe2OG dioxygenase domain-containing protein	Prolyl 4-hydroxylase subunit alpha-3	2	92	4	0.538	0.113	9.6E-04	X
G1TKE7		IQGAP2	IQ motif containing GTPase activating protein 2		1		4	0.538	0.119	1.1E-03	X
G1T705	P00387	CYB5R3	NADH-cytochrome b5 reductase	NADH-cytochrome b5 reductase 3	2	90	4	0.533	0.034	2.0E-04	X
P62943	P62942	FKBP1A	Peptidyl-prolyl cis-trans isomerase FKBP1A	Peptidyl-prolyl cis-trans isomerase FKBP1A	2	100	4	0.533	0.145	1.9E-03	X
	Q16658	FSCN1		Fascin	4		4	0.532	0.049	2.5E-04	X
G1SIB9	P11216	PYGB	Alpha-1,4 glucan phosphorylase	Glycogen phosphorylase, brain form	2	95	4	0.531	0.019	1.9E-04	X
G1T8Z0	P30041	PRDX6	Thioredoxin domain-containing protein	Peroxiredoxin-6	2	95	4	0.529	0.078	3.7E-04	X
G1SNT8	P08133	ANXA6	Annexin	Annexin A6	2	96	4	0.528	0.037	2.0E-04	X
G1T8B5	Q9H1E3	NUCKS1	Nuclear casein kinase and cyclin dependent kinase substrate 1	Nuclear ubiquitous casein and cyclin-dependent kinase substrate 1	2	94	4	0.527	0.079	3.7E-04	X
	P51178	PLCD1		1-phosphatidylinositol 4,5-bisphosphate phosphodiesterase delta-1	4		4	0.526	0.079	3.7E-04	X
G1SQR6	Q15417	CNN3	Calponin	Calponin-3	2	98	4	0.526	0.131	1.3E-03	X
G1SHB9	P40121	CAPG	Uncharacterized protein	Macrophage-capping protein	3	94	4	0.526	0.033	1.9E-04	X
Q8WN94		DBI	Acyl-CoA-binding protein		1		4	0.525	0.081	3.7E-04	X
	P51911	CNN1		Calponin-1	4		4	0.521	0.274	1.7E-02	X
G1TN08	P37235	HPCAL1	Uncharacterized protein	Hippocalcin-like protein 1	3	99	4	0.518	0.052	2.2E-04	X
G1T6L5	O75368	SH3BGRL	Uncharacterized protein	SH3 domain-binding glutamic acid-rich-like protein	3	98	4	0.516	0.065	2.7E-04	X
G1STV0		CAST	Calpastatin		1		4	0.514	0.104	5.6E-04	X
G1SUZ7		ARSA	Sulfatase domain-containing protein		1		4	0.510	0.078	3.2E-04	X
G1TNJ2	Q15942	ZYX	Uncharacterized protein	Zyxin	3	87	4	0.509	0.183	3.1E-03	X
G1SRD9	A0A1C7CYX8	FAM107B	Uncharacterized protein	Protein FAM107B (Fragment)	3	96	4	0.508	0.253	1.0E-02	X
G1SY88	P55263	ADK	PfkB domain-containing protein	Adenosine kinase	2	98	4	0.503	0.020	1.7E-04	X
G1SXU2	Q96HC4	PDLIM5	Uncharacterized protein	PDZ and LIM domain protein 5	3	88	4	0.501	0.070	2.5E-04	X

G1TK30		CSAD	Cysteine sulfinic acid decarboxylase		1		4	0.497	0.170	2.0E-03	X
G1TBJ4	Q92597	NDRG1	Uncharacterized protein	Protein NDRG1	3	96	4	0.497	0.206	4.1E-03	X
G1TMV1	P60981	DSTN	ADF-H domain-containing protein	Destrin	2	100	4	0.495	0.095	3.7E-04	X
G1SYE0		ABHD14B	AB hydrolase-1 domain-containing protein		1		4	0.492	0.054	1.9E-04	X
G1U7U3		NME1	Nucleoside diphosphate kinase		1		4	0.490	0.089	3.2E-04	X
G1SWS9	P08670	VIM	IF rod domain-containing protein	Vimentin	2	97	4	0.488	0.101	3.7E-04	X
	P33241	LSP1		Lymphocyte-specific protein 1	4		4	0.483	0.070	2.2E-04	X
G1T013	P48163	ME1	Malic enzyme	NADP-dependent malic enzyme	2	95	4	0.472	0.132	6.4E-04	X
G1SH05	P07437	TUBB	Tubulin beta chain	Tubulin beta chain	2	100	4	0.471	0.122	4.9E-04	X
G1TBQ6	O95292	VAPB	VAMP associated protein B and C	Vesicle-associated membrane protein-associated protein B/C	2	90	4	0.468	0.106	3.5E-04	X
P00939	P60174	TPI1	Triosephosphate isomerase	Triosephosphate isomerase	2	93	4	0.468	0.192	2.2E-03	X
G1T2Q8	Q96M27	PRRC1	NTPase_I-T domain-containing protein	Protein PRRC1	2	90	4	0.463	0.033	1.7E-04	X
G1SYN4		PTX3	LamGL domain-containing protein		1		4	0.463	0.256	6.2E-03	X
G1T2C4	Q01995	TAGLN	Transgelin	Transgelin	2	99	4	0.462	0.149	8.2E-04	X
	H7BZL4	GPC1		Glypican-1 (Fragment)	4		4	0.460	0.142	6.9E-04	X
G1SNP7	E9PGC8	MAP1A	Uncharacterized protein	Microtubule-associated protein 1A	3	79	4	0.458	0.075	2.0E-04	X
	B4DDF4	CNN2		Calponin	4		4	0.456	0.038	1.7E-04	X
G1T6W4		ANXA8	Annexin		1		4	0.455	0.254	5.4E-03	X
G1SZJ7	A0A2R8YEC9	MANBA	Uncharacterized protein	Beta-mannosidase	3	75	4	0.455	0.065	1.7E-04	X
G1T0H8	Q01813	PFKP	ATP-dependent 6-phosphofructokinase	ATP-dependent 6-phosphofructokinase, platelet type	2	93	4	0.452	0.129	4.5E-04	X
G1SHL8	P42224	STAT1	Signal transducer and activator of transcription	Signal transducer and activator of transcription 1-alpha/beta	2	95	4	0.451	0.056	1.7E-04	X
G1SVE3		PRUNE2	CRAL-TRIO domain-containing protein		1		4	0.450	0.243	4.3E-03	X
G1T2K1	O95340	PAPSS2	Uncharacterized protein	Bifunctional 3~-phosphoadenosine 5~-phosphosulfate synthase 2	3	94	4	0.450	0.130	4.5E-04	X
G1TRY5	P13797	PLS3	Uncharacterized protein	Plastin-3	3	100	4	0.434	0.035	1.7E-04	X
G1TA83	P09525	ANXA4	Annexin	Annexin A4	2	94	4	0.434	0.072	1.7E-04	X
G1U7J6		PRKCA	Protein kinase C		1		4	0.430	0.059	1.7E-04	X
P35748	P35749	MYH11	Myosin-11	Myosin-11	2	97	4	0.428	0.331	1.1E-02	X
G1TE78	Q15121	PEA15	DED domain-containing protein	Astrocytic phosphoprotein PEA-15	2	100	4	0.423	0.094	2.0E-04	X
G1SFG6	A0A0A0MTS2	GPI	Glucose-6-phosphate isomerase	Glucose-6-phosphate isomerase (Fragment)	2	93	4	0.421	0.158	6.1E-04	X
G1SUY3	F8VQR7	CSRP2	Uncharacterized protein	Cysteine and glycine-rich protein 2	3	100	4	0.418	0.092	1.9E-04	X
G1SPY1	E9PGM4	GBE1	Aamy domain-containing protein	1,4-alpha-glucan-branching enzyme	2	93	4	0.416	0.111	2.5E-04	X
G1SRR2	E9PMP7	LMO7	Uncharacterized protein	LIM domain only protein 7 (Fragment)	3	70	4	0.412	0.095	1.9E-04	X
G1T8J0	P02461	COL3A1	Uncharacterized protein	Collagen alpha-1(III) chain	3	92	4	0.406	0.270	3.8E-03	X
G1SPQ9	O94875	SORBS2	Uncharacterized protein	Sorbin and SH3 domain-containing protein 2	3	90	4	0.406	0.087	1.7E-04	X
G1T7Z6	P00558	PGK1	Phosphoglycerate kinase	Phosphoglycerate kinase 1	2	99	4	0.401	0.158	4.7E-04	X
G1TN29	Q86UU1	PHLDB1	Pleckstrin homology like domain family B member 1	Pleckstrin homology-like domain family B member 1	2	89	4	0.394	0.100	1.9E-04	X
	P32418	SLC8A1		Sodium/calcium exchanger 1	4		4	0.387	0.117	2.2E-04	X
G1SW82	H0Y9Y3	SYNPO2	PDZ domain-containing protein	Synaptopodin-2 (Fragment)	2	80	4	0.383	0.158	3.9E-04	X
G1T5B6	P12107	COL11A1	Fibrillar collagen NC1 domain-containing protein	Collagen alpha-1(XI) chain	2	98	4	0.380	0.214	1.1E-03	X
	Q9Y2V2	CARHSP1		Calcium-regulated heat-stable protein 1	4		4	0.373	0.084	1.7E-04	X
G1SKQ9		SEPT6	Septin-type G domain-containing protein		1		4	0.369	0.155	3.4E-04	X
G1TA72		HS1BP3	PX domain-containing protein		1		4	0.369	0.240	1.5E-03	X
G1U634	P08473	MME	Neprilysin	Neprilysin	2	94	4	0.365	0.242	1.5E-03	X
G1T4F9	E9PR44	CRYAB	Alpha-crystallin B chain	Alpha-crystallin B chain (Fragment)	2	98	4	0.365	0.390	1.0E-02	X
G1SYJ4	P06733	ENO1	Uncharacterized protein	Alpha-enolase	3	96	4	0.363	0.217	9.6E-04	X
	P17858	PFKL		ATP-dependent 6-phosphofructokinase, liver type	4		4	0.362	0.236	1.3E-03	X
G1SYD2		AK4	Adenylate kinase 4, mitochondrial		1		4	0.359	0.206	7.3E-04	X
G1T315	A0A2R8Y2R1	SGCE	CADG domain-containing protein	Epsilon-sarcoglycan (Fragment)	2	88	4	0.357	0.101	1.7E-04	X
G1SUI9	E7EX44	CALD1	Uncharacterized protein	Caldesmon	3	84	4	0.346	0.105	1.7E-04	X
G1TYY5	Q14847	LASP1	LIM and SH3 domain protein 1	LIM and SH3 domain protein 1	2	94	4	0.344	0.052	1.7E-04	X
G1TAJ3	P00338	LDHA	L-lactate dehydrogenase	L-lactate dehydrogenase A chain	2	94	4	0.341	0.279	1.9E-03	X
G1SKS8	E7EVA0	MAP4	Microtubule-associated protein	Microtubule-associated protein	2	85	4	0.341	0.172	3.4E-04	X
G1TBU2		KIAA1217	KIAA1217		1		4	0.340	0.130	1.9E-04	X
G1T387	Q14247	CTTN	Cortactin	Src substrate cortactin	2	88	4	0.338	0.069	1.7E-04	X
G1SW77	Q8NC51	SERBP1	HABP4_PAI-RBP1 domain-containing protein	Plasminogen activator inhibitor 1 RNA-binding protein	2	99	4	0.328	0.112	1.7E-04	X
G1SZ00	P21291	CSRP1	Uncharacterized protein	Cysteine and glycine-rich protein 1	3	98	4	0.325	0.579	3.2E-02	X
G1T5T8	O00151	PDLIM1	Uncharacterized protein	PDZ and LIM domain protein 1	3	92	4	0.302	0.067	1.7E-04	X
	Q09666	AHNAK		Neuroblast differentiation-associated protein AHNAK	4		4	0.285	0.075	1.7E-04	X
G1SY87		CD200	Ig-like domain-containing protein		1		4	0.263	0.263	5.9E-04	X
G1SMS2	Q8WX93	PALLD	Uncharacterized protein	Palladin	3	87	4	0.226	0.201	2.0E-04	X

Proteins quantified in ≤ 2 rabbits

G1TKV4		HIST2H3D	Histone H3		1		2	3.424
G1SLF6	P25205	MCM3	DNA helicase	DNA replication licensing factor MCM3	2	97	1	2.650
G1T9I6	A8MZB2	NAA20	N-acetyltransferase domain-containing protein	N-acetyltransferase 5 (ARD1 homolog, S. cerevisiae), isoform CRA a	2	92	1	2.552
G1SW67	O75367	H2AFY	Core histone macro-H2A	Core histone macro-H2A.1	2	91	2	2.451
G1TDA7	Q99459	CDC5L	Uncharacterized protein	Cell division cycle 5-like protein	3	99	1	2.213
G1T3P4	Q9NRL2	BAZ1A	Bromodomain adjacent to zinc finger domain 1A	Bromodomain adjacent to zinc finger domain protein 1A	2	89	2	2.125
P14519	P34897	SHMT2	Serine hydroxymethyltransferase, mitochondrial	Serine hydroxymethyltransferase, mitochondrial	2	95	1	2.049
G1SUG5		DHX37	DEAH-box helicase 37		1		1	1.927

G1SRE6	A0A2R8Y420	EPB41	FERM domain-containing protein	Protein 4.1	2	95	1	1.877
G1TC42		AXL	AXL receptor tyrosine kinase		1		1	1.862
G1TAU6		SERPINE1	SERPIN domain-containing protein		1		2	1.845
G1SYP7		POLR1A	DNA-directed RNA polymerase subunit		1		1	1.838
G1T5A0	P23921	RRM1	Ribonucleoside-diphosphate reductase	Ribonucleoside-diphosphate reductase large subunit	2	98	2	1.782
G1SDE1		MTG1	Mitochondrial GTPase 1		1		1	1.725
G1TGX2		ZCCHC8	Zinc finger CCHC-type containing 8		1		2	1.603
G1T2J6	O75746	SLC25A12	Solute carrier family 25 member 12	Calcium-binding mitochondrial carrier protein Aralar1	2	95	2	1.559
G1SIC7	Q01432	AMPD3	AMP deaminase	AMP deaminase 3	2	96	2	1.518
	P31153	MAT2A		S-adenosylmethionine synthase isoform type-2	4		2	1.483
G1T7P9	Q96DI7	SNRNP40	WD_REPEATS_REGION domain-containing protein	U5 small nuclear ribonucleoprotein 40 kDa protein	2	99	2	1.480
G1SP33		SYMPK	Symplekin		1		2	1.460
G1TCC8	O75934	BCAS2	Uncharacterized protein	Pre-mRNA-splicing factor SPF27	3	100	1	1.451
G1T8H7	A0A2R8YCL1	GRB10	Growth factor receptor bound protein 10	Growth factor receptor-bound protein 10	2	90	2	1.430
G1T5A5		RTN4IP1	PKS_ER domain-containing protein		1		2	1.429
G1U516	E9PHV4	POLR2D	RPOL4c domain-containing protein	DNA-directed RNA polymerase II subunit RPB4	2	76	2	1.411
G1SPJ7		UTP4	WD_REPEATS_REGION domain-containing protein		1		2	1.344
G1SPX2	Q6KC79	NIPBL	Nipped-B protein	Nipped-B-like protein	2	98	2	1.327
G1SS22		XRCC6	Ku domain-containing protein		1		2	1.325
G1SRD1		EXD2	3~-5~ exonuclease domain-containing protein		1		2	1.324
G1SV75	H3BNK3	NDUFAB1	Acyl carrier protein	Acyl carrier protein (Fragment)	2	63	2	1.294
G1T897	B4DP72	COQ5	2-methoxy-6-polyprenyl-1,4-benzoquinol methylase, mitochondrial	2-methoxy-6-polyprenyl-1,4-benzoquinol methylase, mitochondrial	2	77	2	1.260
G1SU02	A0A1W2PP11	PARL	Rhomboid domain-containing protein	Presenilins-associated rhomboid-like protein, mitochondrial	2	94	2	1.246
G1SL03	Q9BY32	ITPA	Inosine triphosphate pyrophosphatase	Inosine triphosphate pyrophosphatase	2	92	2	1.223
G1T1X4	O43395	PRPF3	PWI domain-containing protein	U4/U6 small nuclear ribonucleoprotein Prp3	2	100	2	1.180
G1SX03	Q8TBQ9	TMEM167A	Protein kish	Protein kish-A	2	96	1	1.148
G1SSF0	A3KFL2	EXOSC2	Uncharacterized protein	Exosome complex component RRP4	3	92	2	1.144
	W4VSQ9	TRIP10		Cdc42-interacting protein 4	4		2	1.143
G1SPV2	J3QRU1	YES1	Tyrosine-protein kinase	Tyrosine-protein kinase	2	97	1	1.120
G1SYC3		FBXL8	F-box domain-containing protein		1		2	1.102
G1SUJ1	P24390	KDELR1	ER lumen protein-retaining receptor	ER lumen protein-retaining receptor 1	2	96	2	1.099
G1T719		TSR1	Bms1-type G domain-containing protein		1		2	1.092
G1SI98	O60502	OGA	Uncharacterized protein	Protein O-GlcNAcase	3	99	2	1.087
G1SSS4		CLCN6	Chloride transport protein 6		1		2	1.078
G1SH78		VKORC1	VKc domain-containing protein		1		1	1.078
G1SYH5		NPTN	Neuroplastin		1		1	1.076
G1SKJ8		TCOF1	LisH domain-containing protein		1		2	1.074
	Q8N122	RPTOR		Regulatory-associated protein of mTOR	4		2	1.046
G1SIY9		ALDH5A1	Succinate-semialdehyde dehydrogenase		1		1	1.043
G1SFQ6	Q9NSK0	KLC4	Uncharacterized protein	Kinesin light chain 4	3	97	2	1.036
G1T5U1		GPN1	GPN-loop GTPase		1		2	1.033
G1T3K1		DESI2	DUF862 domain-containing protein		1		2	1.016
G1T2Y2		MAVS	CARD_2 domain-containing protein		1		1	1.008
G1TSK4		AARS2	AA_TRNA_LIGASE_II_ALA domain-containing protein		1		2	1.007
G1SMQ8	Q9P253	VPS18	Pep3_Vps18 domain-containing protein	Vacuolar protein sorting-associated protein 18 homolog	2	97	1	0.997
G1SXS3	Q13573	SNW1	SKIP_SNW domain-containing protein	SNW domain-containing protein 1	2	100	2	0.984
P35566		MARCKSL1	MARCKS-related protein		1		2	0.960
G1SMM1		USP47	USP domain-containing protein		1		2	0.934
G1SXN1	Q9BXB4	OSBPL11	Oxysterol-binding protein	Oxysterol-binding protein-related protein 11	2	93	2	0.933
G1TUU3	Q5JXR6	ZNFX1	Uncharacterized protein	NFX1-type zinc finger-containing protein 1	3	90	2	0.931
G1T850	B4E0K5	MAPK14	Mitogen-activated protein kinase	Mitogen-activated protein kinase	2	100	1	0.928
G1SGB3		ELP1	Elongator complex protein 1		1		2	0.923
G1TXZ1		RIC1	RIC1 domain-containing protein		1		1	0.912
G1SMI4	Q15276	RABEP1	Uncharacterized protein	Rab GTPase-binding effector protein 1	3	96	2	0.899
G1U2M9		SAMD9	Sterile alpha motif domain containing 9		1		1	0.895
G1SKS0	Q92747	ARPC1A	Actin-related protein 2/3 complex subunit	Actin-related protein 2/3 complex subunit 1A	2	100	1	0.873
G1TCS1		MTMR9	Myotubularin phosphatase domain-containing protein		1		2	0.868
G1SMT7		NHLRC2	Thioredoxin domain-containing protein		1		2	0.867
	M0QXB5	ETHE1		Persulfide dioxygenase ETHE1, mitochondrial	4		2	0.867
	Q8N3D4	EHBP1L1		EH domain-binding protein 1-like protein 1	4		1	0.856
	P23142	FBLN1		Fibulin-1	4		2	0.846
G1T9A1	Q96J02	ITCH	E3 ubiquitin-protein ligase	E3 ubiquitin-protein ligase Itchy homolog	2	93	1	0.834
G1SWM7	Q5W0J6	ECHDC3	Uncharacterized protein	Enoyl-CoA hydratase domain-containing protein 3, mitochondrial (Fragment)	3	90	2	0.833
	Q92696	RABGGTA		Geranylgeranyl transferase type-2 subunit alpha	4		2	0.782
G1TBN2	Q96II5	ARAF	Uncharacterized protein	ARAF protein	3	95	2	0.780
G1SNQ2	Q8WVF1	OSCP1	Uncharacterized protein	Protein OSCP1	3	87	2	0.761
	Q8NBZ7	UXS1		UDP-glucuronic acid decarboxylase 1	4		2	0.743
G1SWU8	A0A0C4DGW6	C5orf51	Uncharacterized protein	UPF0600 protein C5orf51	3	96	2	0.704
G1T4G0	Q96N67	DOCK7	Uncharacterized protein	Dedicator of cytokinesis protein 7	3	97	1	0.700

	P25098	GRK2		Beta-adrenergic receptor kinase 1	4		2	0.696
G1TCW2		OSTF1	Osteoclast stimulating factor 1		1		1	0.693
G1SH63		GSS	Glutathione synthetase		1		2	0.685
G1SKE0	Q15555	MAPRE2	Uncharacterized protein	Microtubule-associated protein RP/EB family member 2	3	98	1	0.684
G1TF20		PLEKHO2	PH domain-containing protein		1		1	0.658
	P78536	ADAM17		Disintegrin and metalloproteinase domain-containing protein 17	4		1	0.649
G1TEA5	P36959	GMPR	GMP reductase	GMP reductase 1	2	97	2	0.648
G1U4A0		FRMD8	FERM domain containing 8		1		2	0.647
	E9PS68	PC		Pyruvate carboxylase, mitochondrial	4		2	0.589
G1T1M9		HBS1L	Tr-type G domain-containing protein		1		1	0.587
G1T9W9		ATG3	Autophagy-related protein 3		1		2	0.577
G1TNB4		PARP3	Poly [ADP-ribose] polymerase		1		2	0.550
G1SN67		SERPINB1	Serpin family B member 1		1		1	0.521
G1SFW9		ACIN1	SAP domain-containing protein		1		1	0.421

A. Database Identification Categories: Category 1, Characterized in the rabbit database only; Category 2, Characterized in both the rabbit and human databases; Category 3, Uncharacterized in the rabbit database but characterized in the human database; Category 4, Characterized in the human database only.
B. Percent sequence Identity between rabbit and human proteins to the nearest integer (from *blastp* analysis of the rabbit and human UniProt databases).
C. SEM, Standard Error of the Mean.
D. Moderated t-test p-values adjusted for multiple testing (calculated for paired samples using the LImma software package in R).
